# N6-methyladenosine-modified SENP1, identified by IGF2BP3, is a novel molecular marker in acute myeloid leukemia and aggravates progression by activating AKT signal via de-SUMOylating HDAC2

**DOI:** 10.1186/s12943-024-02013-y

**Published:** 2024-05-31

**Authors:** Diguang Wen, Hang Xiao, Yueyi Gao, Hanqing Zeng, Jianchuan Deng

**Affiliations:** Department of Hematology, Second Affiliated Hospital of Chongqing Medical University, Chongqing, 400010 China

**Keywords:** Acute myeloid leukemia, SUMO, SENP1, m6A

## Abstract

**Background:**

Elevated evidence suggests that the SENPs family plays an important role in tumor progression. However, the role of SENPs in AML remains unclear.

**Methods:**

We evaluated the expression pattern of SENP1 based on RNA sequencing data obtained from OHSU, TCGA, TARGET, and MILE datasets. Clinical samples were used to verify the expression of SENP1 in the AML cells. Lentiviral vectors shRNA and sgRNA were used to intervene in SENP1 expression in AML cells, and the effects of SENP1 on AML proliferation and anti-apoptosis were detected using in vitro and in vivo models. Chip-qPCR, MERIP-qPCR, CO-IP, RNA pulldown, and dual-luciferase reporter gene assays were used to explore the regulatory mechanisms of SNEP1 in AML.

**Results:**

SENP1 was significantly upregulated in high-risk AML patients and closely related to poor prognosis. The AKT/mTOR signaling pathway is a key downstream pathway that mediates SENP1's regulation of AML proliferation and anti-apoptosis. Mechanistically, the CO-IP assay revealed binding between SENP1 and HDAC2. SUMO and Chip-qPCR assays suggested that SENP1 can desumoylate HDAC2, which enhances EGFR transcription and activates the AKT pathway. In addition, we found that IGF2BP3 expression was upregulated in high-risk AML patients and was positively correlated with SENP1 expression. MERIP-qPCR and RIP-qPCR showed that IGF2BP3 binds SENP1 3-UTR in an m6A manner, enhances SENP1 expression, and promotes AKT pathway conduction.

**Conclusions:**

Our findings reveal a distinct mechanism of SENP1-mediated HDAC2-AKT activation and establish the critical role of the IGF2BP3/SENP1signaling axis in AML development.

**Supplementary Information:**

The online version contains supplementary material available at 10.1186/s12943-024-02013-y.

## Introduction

Acute myeloid leukemia (AML) is one of the most common cancers [1]. AML is caused by gene mutations, chromatin abnormalities, and protein conformational changes that shift the growth and differentiation of hematopoietic stem cells [2]. In recent decades, a number of post-translational modifications of proteins (such as ubiquitination or small ubiquitin-like modifiers) have been reported to play a significant role in AML [3]. Previously, we reported some genetic mechanisms of AML, such as m6A modification and alternative splicing [4]. Further exploration of protein post-translational modifications is required to identify the pathological process of AML.

The small ubiquitin-like modifier (SUMO) is a common form of post-translational modification in many proteins. It has been found that sumo modification is involved in almost all malignant behaviors of tumors, such as tumor proliferation, metastasis, immune microenvironment [5]. The SENPs (SUMO/Sentrin Specific Peptidase) protein family is a class of key enzymes that are mainly responsible for the desumo modification of proteins in cells. Increasing evidence has revealed a correlation between SENPs and tumor development [6]. SENP1 desumoylated HK2 enhanced glycolysis in thyroid cancer, which promoted thyroid cancer proliferation and metastasis [7]. SENP3 transmits ROS information and activates STING to regulate the antitumor immunity of DC cells [8]. SENP7 enhances EMT in breast cancer and promotes its metastasis [9]. Despite this, there is still a lack of systematic analysis and research on the relationship between the SENPs family and AML development. Additionally, m6A plays a significant role in the activity of the body and in the development of AML. Research has shown that m6A modifications regulate the expression and function of many genes in cells. However, there is still a lack of reports on the correlation between SENP1 and m6A [4].

In this study, we found that SENP1 was significantly overexpressed in high-risk AML patients. SENP1 mediated HDAC2-AKT activity was verified both in vitro and in vivo. In addition, we found that the above signaling pathway in AML is driven by IGF2BP3 in an m6A dependent manner.

## Methods

### Online data download and processing

Oregon Health and Science University (OHSU) AML dataset: RNA sequencing data (RPKM) and clinical data of patients were acquired from the cBioPortal data portal (https://www.cbioportal.org/) [10]. TARGET dataset for AML: We acquired RNA sequencing data (FPKM), clinical data, and patient survival information from TATGET (https://ocg.cancer.gov/). The leukemia MILE dataset was obtained from the Gene Expression Omnibus (GEO) database (GEO ID: GSE13159). All the above RNA-seq data are converted into TPM value using R “LIMMA” package for subsequent analysis [11].

### Patients

This study collected bone marrow and peripheral blood samples were collected from 10 patients diagnosed with acute myeloid leukemia at the Second Affiliated Hospital of Chongqing Medical University. This study was approved by the Ethics Committee of Chongqing Medical University. The AML patient risk classification was based on the 2022 European Leukemia Net (ELN) guidelines [12]. The human bone marrow and peripheral blood mononuclear cell isolation kits were purchased from Solarbio (Beijing, China). For the isolation of mononuclear cells, we refer to the manufacturer's instructions. Protein and RNA were extracted from the bone marrow and peripheral blood mononuclear cells and detected by western blotting and RT-PCR, respectively. Patient details can be found in Supplemental Material, Table S1. All the antibody information is provided in Supplementary Table S2. Table S3 lists all the primers.

### Differential gene analysis and prognosis analysis

Differential SENPs genes were identified between the high- and low-risk groups using the R "LIMMA" package. The cutoff value was P less than 0.05, when comparing the adverse with the intermediate and favorable prognosis groups. Survival prognosis analysis was performed by dividing the patients into two groups based on the expression of the SENPs family proteins. The R "surveyor" software package was used to find the most appropriate separation plot the survival curve, calculate the HR value, and the cutoff value *P* < 0.05 [13].

## GO annotation, GSEA and GSVA analysis

First, based on SENP1 expression, we divided AML into two groups. Differential expression genes were identified by the "LIMMA" package. Co-expressed genes were identified using SPERMAN correlation analysis. Differentially expressed genes (DEGs) were used for functional analyses of GO, GSEA, and GSVA. “Enrichplot” and “GOPLOT” packages are available for GO and GSEA analysis. “GSVA” packages are used for GSVA analysis [14].

### Cell transduction, plasmid construction and lentivirus production

The human cell lines HEK-293T (cat. CL-0005), HL-60 (cat. CL-0110) and THP-1 (cat. CL-0233) and NB4 (cat. CL-0676) was purchased from Pricella Life Science&Technology Co.,Ltd. KG-1 (cat. CCL-246) were purchased from American Type Culture Collection (ATCC, USA). Complete medium containing serum was purchased from Pricella Life Science&Technology Co.,Ltd. Lentiviral vectors that knocked down and overexpressed SENP1, HDAC2, or IGF2BP3 were purchased from GeneChem (Shanghai, China). The negative control vector was used. The plasmid with the lentiviral vector knocking down IGF2BP1/2 was purchased from GeneChem (Shanghai, China). pCMV-VSV-G and pCAG-dR8.9 were used to package lentiviruses. Purinomycin was used to screen for cells with stable transduction.

shSENP1#1 CAAGAAGTGCAGCTTATAATT; shSENP1#2 GCAGTGAAACGTTGGACAATT; shSENP1#3 AACTACATCTTCGTGTACCTC**;** shIGF2BP3#1 GCACATTTAATTCCTGGATTA; shIGF2BP3#2 GCAGGAATTGACGCTGTAT; shIGF2BP1#1 ATGTAAAGCTTGTTCATGGTG; shIGF2BP1#2 CCGGCTCCAAAGTTCGTATGGTTATCTCGAGATAACCATACGAACTTTGGAGTTTTTTG; shIGF2BP2#1 TGATTTTCCCATGCAATTCCA; shIGF2BP2#2 ACCGGTGGCAGATGAGACCAAACTATTCAAGAGATAGTTTGGTCTCATCTGCCTTTTTTGAATTC. cDNA3.1. Full-length HDAC2 and HDAC2 fragments were obtained by polymerase chain reaction (PCR) using specific primers and verified by sequencing. Flag, HIS, and EGFP vectors were used to construct expression plasmids. The construction of SENP1 enzyme-inactivated mutants (R630L and K631M) was based on previous studies [15].

### Cas9/CRISPR gene knockout in AML cells

The design of the small cas9 guide RNA (sgRNA) sequence was based on the website tool of Zhang's laboratory (http://crispr.mit.edu/). HL-60 or KG-1 cells were cultured in a 100 mm petri dish, and a single lentivirus with sgRNA-Cas9 plasmid was transfected. Two days after transfection, the cells were transferred into a 100 mm petri dish at a ratio of 1:4 and treated with 2 μg/ml purinomycin after 3 days. Flow cytometry was used to sort single cells for culturing. Positive clones were selected by RT-PCR and Western blotting. Cells transfected with sgRNA were selected using purinamycin, and a pool of resistant cells was used as a knockout control. sgSENP1#1 GTTATCAGGCAGTGAAACGT; sgSENP1#2, ATTAAAAACGGCTGGTTATC.

### Quantitative RT–PCR

Total RNA was extracted from cells using TRIzol reagent according to the manufacturer’s instructions. Subsequently, the PrimeScript TM RT Master Mix Kit (TaKaRa, Dalian, China) was used to reverse-transcribe RNA into cDNA. SYBR (ABclonal Technology Co.Ltd. China ) was used for the real-time PCR analysis. Standardized expression under control ACTIN.

### Cell proliferation and apoptosis assay

Cell counting kit - 8 (APExBio, USA) was used. The protocol recommended by the manufacturer was used to determine the proliferation rate at 0, 24, 36, 48, and 72 h post-infection. The Annexin V-FITC/PI apoptosis detection kit (China Elabscience Biotechnology Co., Ltd.) was used to confirm the level of apoptosis using flow cytometry (FCM) analysis.

## Immunoblot assay

A total protein extraction kit (Beyotime, China) was used to extract total protein from cultured cells. Proteins were separated using SDS-PAGE, which was then transferred to a PVDF membrane (0.45 um hole, Millipore). The bands were blocked with 5% skimmed milk powder for 1 h. The membranes were then incubated with primary antibodies overnight. TBST buffer washed the bands 3 times for 5 min each. Horseradish peroxidase (HRP)-coupled anti-mouse or rabbit secondary antibodies were used for ECL detection.

### EDU Cell Proliferation assay

The cells from each group were cultured in six-well plates. The cells were allowed to grow to 70% confluence, EDU was added at a 10M concentration, and incubated for 4 h. The cells were then fixed in paraformaldehyde. 0.3% Triton X-100–permeable cells. Finally, the EDU-positive cells were detected using flow cytometry. FlowJo_V10 was used for flow analysis.

### Tumor xenograft model and tail vein injection mouse model

All animal experimentation methods employed in this study were approved by the Animal Ethics Committee of the Chongqing Medical University. 4 weeks old male BALB/c Nude Mice (18–22 g) were purchased from Vital River (Beijing, China) and fed in an specific-pathogen-free (SPF) grade animal house. Nude mice were subcutaneously injected with a 1:1 mixture of Matrigel (BD356234; Corning, USA) and HL-60 cell suspension (1 × 10^7^ cells). The mice were sacrificed, and the subcutaneous tumor was completely removed when the tumor grew to a certain size. Tumor volume was calculated as follows: (longest diameter) × (shortest diameter) 2 × (π/6).

### Cell cycle

The cells were fixed overnight in 75% ethanol at 37 °C. On day two, the fixed solution was washed with PBS and 500μL of cell cycle detection working solution (RNase:PI= 1:9) was added. Flow cytometry was used to detect cell cycle distribution.

### Immunohistochemistry and immunofluorescence staining in AML

An immunohistochemistry kit was purchased from Boster Biological Technology Co.,Ltd. Hematoxylin staining solution was purchased from Solarbio. Immunohistochemistry was performed as previously described [4]. Briefly, mouse subcutaneous tumor tissue was embedded in paraffin and made into paraffin sectioned. The IRS immunohistochemical score was used as the immunohistochemistry score, and the staining depth of the entire tumor tissue was evaluated under low magnification. IRS=SI (Positive Intensity) × PP (positive cell ratio), SI can be divided into four levels, with no positive staining at level 0, weak positive in light yellow at level 1, medium positive in brown at level 2, and strong positive in brown at level 3. PP can be divided into five levels, with 0% to 5% for Level 0, 6% to 25% for Level 1, 26% to 50% for Level 2, 51% to 75% for Level 3, and>75% for Level 4. Immunofluorescence co-staining was performed as previously described [16]. Briefly, co-staining was performed using multiple immunofluorescence kits (Servicebio, China). The corresponding secondary antibodies were CY3-TSA and FITC-TSA (G1222, Servicebio). DAPI was used to visualize the nuclei.

### mRNA life-time experiment

AML cells were treated with actinomycin, total RNA was extracted at 0, 3, and 6 h, and the abundance of SENP1 mRNA was detected.

### RNA pull down

An RNA probe with biotin-labeled m6A and guanine bases was synthesized. The RNA probe was co-incubated with streptomycin affinity magnetic beads, and the complex was co-incubated with the protein supernatant. The incubated magnetic beads were washed five times with IP washing buffer for 5 times, an elution time of 2 min. Protein samples were denatured with SDS buffer and analyzed by western blotting.

### HDAC2 activity assay

First, the nuclear extract was prepared using EpiQuickTM Nuclear Extraction Kit I (Epigentek, USA) following the manufacturer’s instructions. Nuclear extracts can be immediately used to detect HDAC activity. HDAC2 activity of each sample was analyzed using the EpiQuickTM HDAC2 Activity Assay Kit (Epigentek, USA). This detection kit contains seven different components, from HB1 to HB7. HB1 was diluted to the working concentration in distilled water. The protein concentration to 2μg/μL using HB2. For the control, 5 μL HB2. Add 150 μL HB3 incubate at 37 °C for 40 min. Each well was extracted, washed with diluted HB1 and HB4, and incubated at room temperature for 60 min. Each well was cleaned, diluted HB5 was added, and incubated at room temperature for 30 min. Each well was cleaned, and 100 μL HB6 was added and incubated at room temperature for 5 min. Then, 50 μL HB7 was added to each well, and the absorbance was read at 450 nm on a microplate reader.

### Tunel staining

Under the action of deoxyribonucleotide terminal transferase (TdT), the derivatives formed by deoxyribonucleotide and biotin (FITC) are labeled at the 3 'end of the DNA to detect apoptotic cells.

### Co-immunoprecipitation (CO-IP), RIP and Chromatin Immunoprecipitation (Chip)

Cells (1 × 107) were used for CO-IP, and cell lysates (RIPA) were used to lyse total cells. Incubate the antibody with A/G magnetic beads at room temperature for 1.5 hours, washed with PBS three times, and co-incubated the cell lysate and antibody complex at 4 ℃ overnight. On the second day, after three times of PBS cleaning, SDS denatured and extracted the proteins. The SUMO probe references Shangguan X ^7^. Briefly, plasmids (flag-HDAC2, UBE2, His-SUMO1, and EGFP-SENP1) were transfected into HEK-293T cells, and the formation of the SUMO1-HDAC2 complex was detected using CO-IP. Chip-qPCR was performed as previous literature [17, 18]. The RIP assay was performed according to the instructions of the RIP Kit (Guangzhou, China). Briefly, magnetic beads were mixed with anti-M6A /IGF2BP3/IgG antibodies before adding cell lysate. The bound complexes were thoroughly cleaned, eluded, purified, and analyzed by RT-PCR. The enrichment of the precipitated RNA was normalized relative to the input control. RT-PCR products were subjected to agarose gel electrophoresis to visualize the results.

### RNA Fluorescence in situ hybridization (FISH)

The RNA fish assay procedure has been described in our previous studies [19]. Briefly, the cells were placed on a polylysine-coated cover slide, fixed with formaldehyde, and permeabilized. Cells were incubated overnight with a specific SENP1 RNA probe working solution. They were washed with the washing solution three times, IGF2BP3 protein was labeled by immunofluorescence, and colocalization was detected using laser confocal detection.

### Dual luciferase assay

The HEK-293T cells were inoculated into 24-well plates and incubated until they reached 70% confluence. Then 0.75μg pGL3-basic-SENP1 3-UTR wt or pGL3-basic-SENP1 3-UTR mut luciferase reporter and 0.25 μg IGF2BP3 expression plasmid or empty vector were used. The luciferase activity was measured after 48h of incubation. The activity of the pGL3-basic-SENP1 3-UTR luciferase reporter gene was normalized to that of the pRL-TK luciferase reporter gene, and compared between IGF2BP3 overexpressed cells and cells transfected with empty vectors.

### Statistical analysis

To compare non-normalized quantitative data between the two groups that followed a normal distribution, we used the Student’s t-test. The statistical test of two groups of non-normalized quantitative data that do not conform to a normal distribution is adopted by a non-parametric test. Single sample t and Wilcoxon tests were used to compare the normalized quantitative data between the two groups. Chi-square test or Fisher’s exact test was used to compare categorical variables. The Kaplan–Meier method was used for the survival analysis. One-way analysis of variance was used for pairwise comparisons between multiple groups. Statistical significance was set at *P* < 0.05, and all statistical tests were bilateral unless otherwise specified. * *P*<0.05; ** *P*<0.01; *** *P*<0.001; ns, not significant. All statistical data were analyzed using R software version 4.4.10, and PRISM 8.0.

## Results

### High-throughput library screening identifies the expression patterns, prognostic significance, and clinical correlation of SENP1

**Fig. 1:**
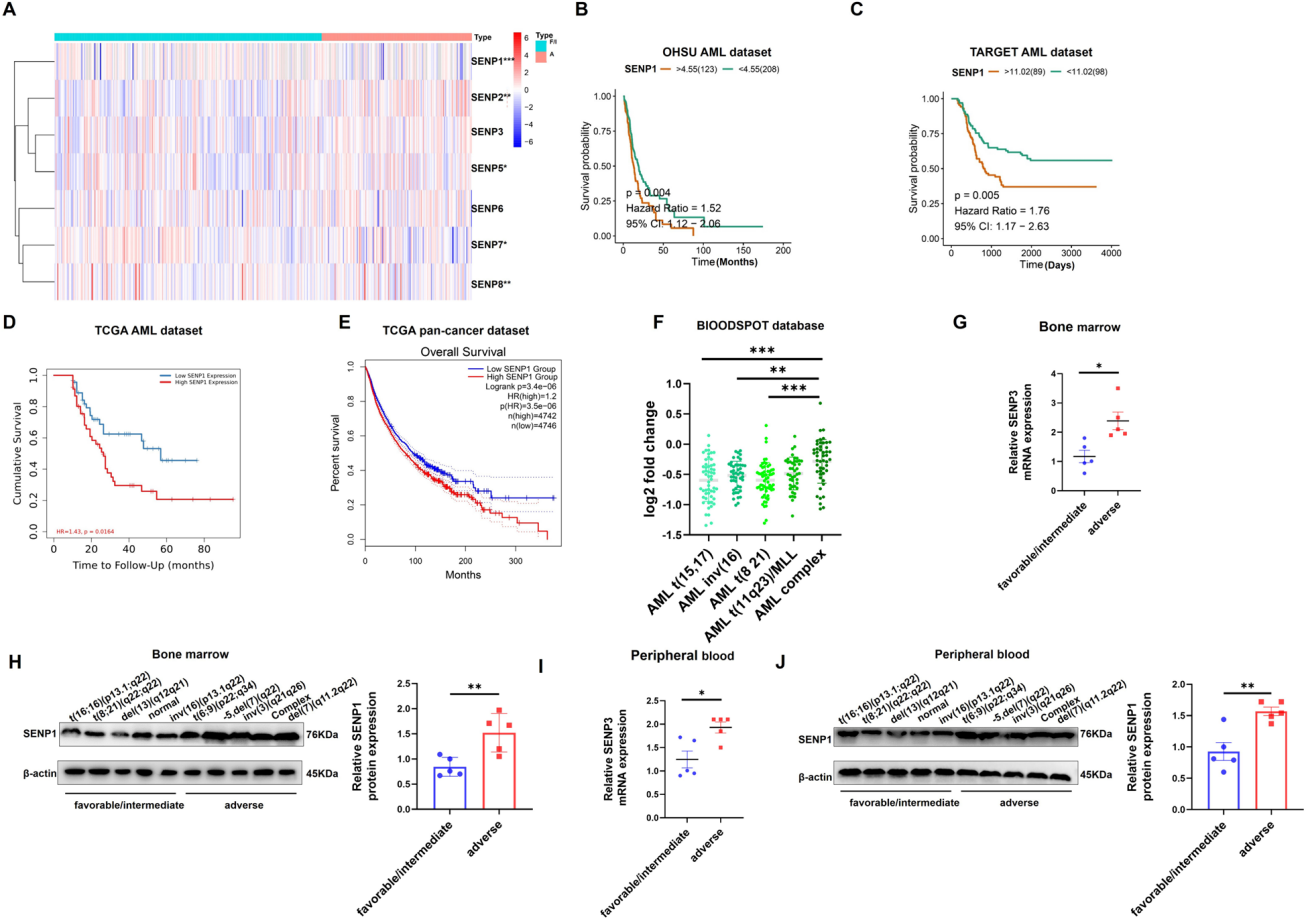
Identify SENP1 as a key candidate gene for AML. **A** Differential gene analysis of SENPs protein families in adverse prognosis group and favorable/intermediate prognosis group using OHSU AML dataset (risk stratification based on ELN 2017, because the latest classification of the dataset is only 2017). **B** Survival analysis of SENPs family proteins in AML using OHSU AML dataset. (Only SENP1 results are shown, and the rest SENPs results are in the supplementary materials.) (**C**) Survival analysis of SENPs family proteins in AML using TARGET AML dataset. (Only SENP1 results are shown, and the rest SENPs results are in the supplementary materials.) (**D**) Survival analysis of SENP1 in AML using TCGA AML dataset. **E** Survival analysis of SENP1 in pan cancer using TCGA pan cancer data. **F** Clinical correlation analysis of SENP1 and AML karyotype using BLOODSPOT database. **G** Mononuclear cells RNA from the bone marrow of AML patients were extracted to detect the expression of SENP1 mRNA expression in AML patients with different risk levels. **H** Mononuclear cells protein from the bone marrow of AML patients were extracted to detect the expression of SENP1 protein expression in AML patients with different risk levels. **I** Mononuclear cells RNA from the peripheral blood of AML patients were extracted to detect the expression of SENP1 mRNA expression in AML patients with different risk levels. **J** Mononuclear cells protein from the peripheral blood of AML patients were extracted to detect the expression of SENP1 protein expression in AML patients with different risk levels

To comprehensively investigate the role of SENPs (SENP1-3 and SENP5-8) family proteins in AML, we analyzed the expression pattern and survival significance by investigating publicly available OHSU-AML datasets (Table S4). We found that SENP1 and SENP2 were highly expressed in the high-risk AML group and closely related to poor prognosis (Fig. 1A, B, and S1A ). We explored the TARGET dataset for prognostic analysis to avoid errors in single-center sample datasets. We found that both SENP1 and SENP2 were strongly associated with poor clinical outcomes (Fig. 1C and S1B). SENP1, however, is more clinically relevant. Therefore, we focus on SENP1's role in AML in this study. In the TCGA-AML dataset, SENP1 was associated with poor prognosis (Fig. 1D). Pan cancer analysis showed that tumor patients with SENP1 high expression had a shorter survival period (HR=1.2, *P*<0.001) than those with low SENP1 (Fig. 1E). These results suggest that SENP1 is a key molecule in AML pathology.

Subsequently, we analyzed the correlation between SENP1 expression and clinical features of patients with AML. We found that SENP1 expression was the highest in AML patients with P53 mutation (Figure.S2A). SENP1 expression did not significantly correlate with patient age (Figure.S2B). TP53 mutations have been identified by the 2022 ELN guidelines as markers of poor AML prognosis. Exploring the MILE dataset, a large dataset of AML showed no significant difference in the expression of SENP1 in bone marrow mononuclear cells of normal and AML patients (Figure.S2C); however, we found that SENP1 mRNA expression was significantly upregulated in AML patients with adverse prognosis (Figure.S2D). In addition, exploring the BLOODSPOT database, we found that the expression level of SENP1 in AML with adverse prognosis karyotypes, such as complex karyotypes, was significantly higher than that in AML with favorable prognosis karyotypes, such as t(15,17) (Fig. 1F) [20]. Furthermore, we found that the expression of SENP1 mRNA and protein was significantly higher in AML patients with poor prognosis than in AML patients with favorable prognosis (Fig. 1G-J). Clinical correlation analysis found that patients with high SENP1 expression had lower BM BLASTS (65.96 vs 53.26, *P*=0.0002), more M1 type (1 vs. 7, *P*=0.0015), and fewer M4 type (22 vs. 3, *P*=0.0026) (Table 1). Univariate and multivariate COX regression analyses suggested that SENP1 was an independent risk factor for AML (hazard ratio [HR] =1.03, *P*=0.03) (Table 2). These data support an important role for SENP1 in AML development.

**Table 1:** Correlation analysis between SENP1 and clinical characteristics of AML patients based on OHSU database

**Characteristic**	**Total**	**low-SENP1(*n*=225)**	**high-SENP1(*n*=224)**	***P*value**
Age/years, median (range)		56.65(8-86)	57.59(2-87)	0.5822
Age group/n (%)				0.3878
<60 years	263	136(60.4%)	127(56.7%)	
≥60 years	185	88(39.6%)	97(43.3%)	
WBC/×10 9 /L, median (range)		38.05	35.91	0.6832
PM blasts/%, median (range)		48.45	53.26	0.1732
BM blasts/%, median (range)		65.96	53.26	0.0002
FAB subtype/n (%)				
M0	6	3	3	0.4633
M1	8	1	7	0.0015
M2	10	7	3	0.6663
M3	10	9	1	0.0665
M4	25	22	3	0.0026
M5	31	20	11	0.9229
M6	0	0	0	ns
M7	2	0	2	0.0571
Cytogenetics/n				
FLT3_ITD_CONSENSUS_CALL		62(27.6%)	43(19.2%)	0.0364
NPM1_CONSENSUS_CALL		61(27.4%)	46(25.8%)	0.0912
CEBPA_MUTATION		14(13.9%)	14(15.4%)	0.7652
BRAF_MUTATION		1(5.9%)	3(12.0%)	0.5074
DNMT3A_MUTATION		34(39.5%)	25(34.2%)	0.4915
IDH1_MUTATION		15(16.7%)	16(15.0%)	0.7422
KRAS_MUTATION		8(9.8%)	6(10.2%)	0.9355
NRAS_MUTATION		27(37.5%)	18(20.7%)	0.0192
TP53_MUTATION		5(6.7%)	22(31.4%)	0.0001
INDUCTION_RESPONSE/n				0.4424
Complete Response		108(61.3%)	95(65.6%)	
Refractory		68(38.7%)	50(34.4%)	
Risk_Cyto/n (%)				
Favorable		77(34.2%)	53(23.7%)	0.0136
Intermediate		87(38.7%)	70(31.3%)	0.0994
Adverse		61(27.1%)	101(45.1%)	<0.0001
PLATELET/×10^9/L, median (range)		63.79	68.34	0.6289
LDH/***U/L,***median (range)		644.9	937.2	0.1794

**Table 2:** Univariate and multivariate analysis of the relationship between SENP1 expression and overall survival in AML patients

**Variable**	**Univariate analysis**			**Multivariate analysis**		
	**HR**	**95% CI**	***P* value**	**HR**	**95% CI**	***P* value**
WBC	1.00	1.00-1.00	0.51			
PB_BASOPHILS	1.19	0.96-1.48	0.11			
BM_BLAST	1.00	0.99-1.00	0.36			
ELN_2017	1.47	0.93-2.32	0.10			
PLATELET_COUNT	1.00	1.00-1.00	0.77			
SEX	0.85	0.55-1.33	0.48			
SENP1	1.03	1.00-1.06	0.03	1.03	1.00-1.06	0.03

Multivariate COX both direction inclusion variables

Subsequent pan-cancer analysis showed that SENP1 was most highly expressed in AML among all TCGA tumors (Figure.S2E). Differential gene analysis revealed that SENP1 is differentially expressed in multiple malignancies, such as BRCA and BLCA (Figure.S2F), and is closely associated with poor prognosis in malignant tumors, such as LIHC and KIRP (Figure.S2G). These data support SENP1's potential role in promoting malignancy progression.

### Knockdown of SENP1 significantly inhibits AML progression in vitro and in vivo

**Fig. 2:**
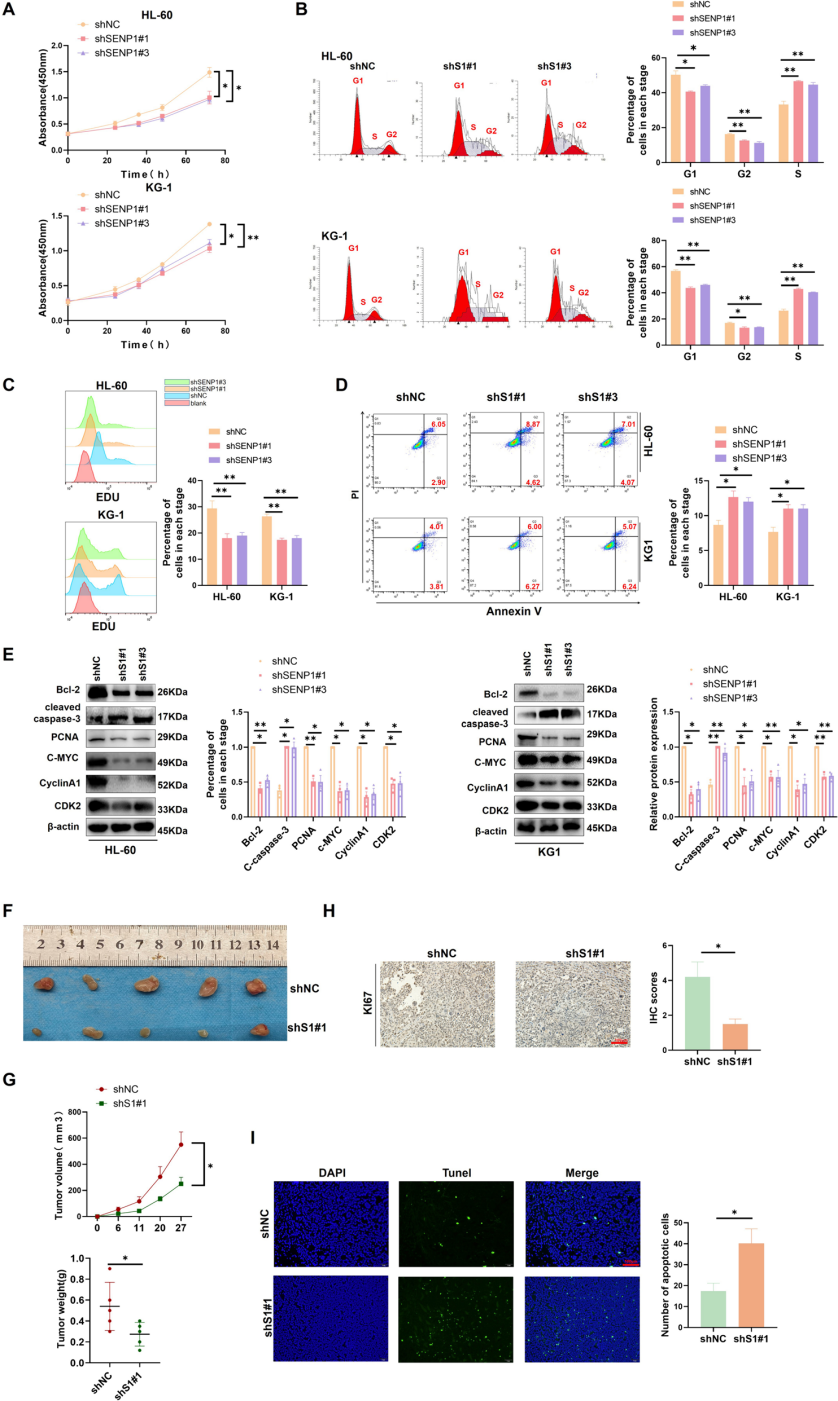
Knockdown of SENP1 inhibits AML cell proliferation and resistance to apoptosis. **A** AML proliferation ability was detected using CCK-8 assay at different time points (0, 24, 36, 48, and 72 hours) in HL-60 and KG-1 cells after shSENP1 and shNC transduction. **B** The effect of silencing SENP1 on the cell cycle of AML was detected by cell cycle assay. **C** EDU probe was used to detect the effect of silencing SENP1 on AML proliferation rate. **D** Flow cytometry (representative images are shown) was used to confirm that SENP1 knockdown induced apoptosis. **E** The levels of cell proliferation (PCNA, C-MYC, PCNA, CYCLINA1 and CDK2) and apoptosis (cleaved caspase-3 and Bcl-2) related proteins were detected by Western blot after SENP1 silencing. **F** At 27 days, stripped subcutaneous tumors were observed in two different groups. **G** Use a vernier caliper to measure the growth curve of shSENP1 # 1 and shNC group xenografts every 6 days to draw the tumor size (width 2 × length × π/6) (Left). Subcutaneous tumors were stripped and weighed (Right). **H** Representative image of KI67 immunohistochemical staining in tumors resected from xenotransplantation model mice. **I** Representative images of Tunel staining in tumors resected from xenotransplantation model mice

Next, we verified SENP1's potential role in AML using in vitro and in vivo experiments. We measured SENP1 expression in four classical AML cell lines. We found that SENP1 was highly expressed in HL-60 and KG-1, and low in THP-1 and NB4 (Figure.S3A). This may be because KG-1 cells have complex karyotypes and HL-60 cells have P53 mutations. We used three independent lentivirus sequences to interfere with SENP1 expression in AML cells (HL-60 and KG-1) and found that the first and third sequences were effective in interfering with SENP1 expression (Figure.S3B). Using the CCK8 cell proliferation assay, we found that AML cell proliferation was significantly reduced after SENP1 silencing (Fig. 2A). The cell cycle assay showed that AML cells were blocked in the S1 phase after silencing of SENP1 (Fig. 2B). The EDU probe showed that the AML cell proliferation rate decreased after SENP1 silencing (Fig. 2C). The apoptosis rate were markedly increased after SENP1 silencing (Fig. 2D). Western blotting also showed that after silencing SENP1, the expression of anti-apoptosis protein (Bcl-2), proliferation protein (PCNA, C-MYC), and S phase checkpoint (CyclinA1 and CDK2) in AML cells was downregulated, and pro-apoptosis markers (Cleaved Caspase-3) were elevated (Fig. 2E). Next, we used two independent sgRNA sequences to knock out SENP1 in HL-60 and KG-1(Figure.S4A and S4B). CCK8 assay showed that the proliferation rate of AML cells decreased significantly after SENP1 knockout (Figure.S4C). The EDU probe showed that the AML cell proliferation rate decreased after SENP1 knockout (Figure.S4D). The apoptosis rate were markedly increased after SENP1 knockout (Figure.S4E). Cell cycle analysis showed that after SENP1 knockout, AML cells were blocked in S1 phase (Figure.S4F).

Subcutaneous tumorigenesis experiments in nude mice showed that SENP1 promoted AML cell proliferation in vivo (Fig. 2F, 2G, and S5A). Immunohistochemical results showed that the subcutaneous tumor proliferation marker KI67 decreased after SENP1 was silenced (Fig. 2H). Immunofluorescence showed that after SENP1 silencing, the abundance of proliferating PCNA protein in the subcutaneous tumors decreased (Figure.S5B). Immunofluorescence also showed that after SENP1 silencing, the expression of anti-apoptotic markers (Bcl-2) in subcutaneous tumors decreased (Figure.S5B). TUNEL staining revealed an increase in apoptosis in the silent SENP1 group (Fig. 2I). Western blotting also showed that after silencing of SENP1, PCNA expression was downregulated (Figure.S5C).

### Overexpression of SENP1 promoted the proliferation and anti-apoptosis of AML

**Fig. 3:**
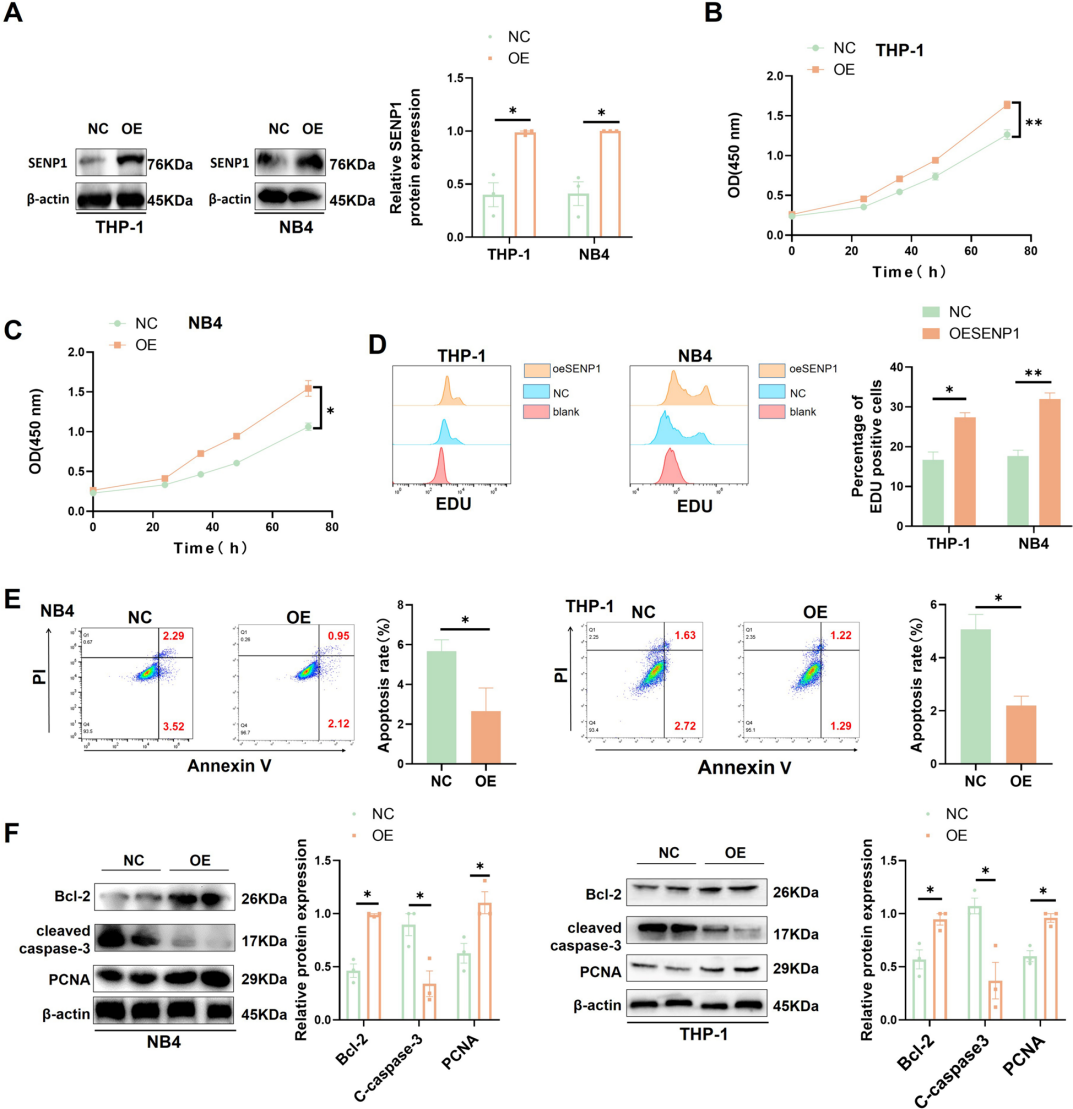
Overexpression of SENP1 promoted the proliferation and anti-apoptosis of AML. **A** Western blot was used to detect the efficiency of SENP1 overexpression. **B** The effect of overexpression of SENP1 on the proliferation of THP-1 was detected by the CCK8 assay. **C** The effect of overexpression of SENP1 on the proliferation of NB-4 was detected by the CCK8 assay. **D** EDU probes were used to detect the effects of overexpression of SENP1 on the proliferation rate of AML. **E** Apoptosis flow was used to detect the effect of overexpression of SENP1 on AML cells apoptosis. **F** The influence of overexpression of SENP1 on AML cells proliferating protein (PCNA) and apoptotic-related (cleaved caspase-3 and Bcl-2) protein was detected by western blot

Next, we overexpressed SENP1 in THP-1 and NB4 cells (Fig. 3A). CCK8 assay showed that overexpression of SENP1 promoted AML cell proliferation (Fig. 3B and C). EDU showed an increased the proliferation rate of AML cells after increasing SENP1 expression (Fig. 3D). The apoptosis rate of AML cells was markedly reduced by increasing SENP1 expression (Fig. 3E). Western blotting showed that the expression of anti-apoptotic proteins (BCL-2) and proliferative proteins (PCNA) was upregulated, and the expression of pro-apoptotic protein (cleaved caspase-3) was downregulated after overexpression of SENP1 (Fig. 3F).

We aimed to determine whether SENP1 regulates AML progression in a sumo enzyme-dependent manner. We overexpressed two SENP1 enzyme-inactivated mutants (R630L and K631M) in AML cells (Figure.S3A). CCK8, EDU, and cell cycle assays showed that the SENP1 mutant did not affect AML cell growth (Figure.S6B-E).

### SENP1 promotes AML progression through AKT signaling pathway

Next, we investigated the mechanism by which SENP1 regulates AML progression. To predict the downstream signaling pathways of SENP1, we conducted differential gene analysis based on SENP1 expression and identified 4746 DEGs between high-SENP1 and low-SENP1 expression groups. GO annotation was performed on 4746 DEGs (Figure.S7A and Table.S5). GO annotation showed that these genes were related to biological processes, such as mitotic division, nuclear division, and regulation of cell proliferation (Figure.S7B). GSEA and GSVA were performed on 4746 DEGs (Figure.S7C and S7D, Tables S6 and S7). The intersection of the GSEA and GSVA results was used to avoid errors caused by a single analysis method. These identified common signaling pathways, such as the adherent junction, cell cycle, and AKT/mTOR signaling pathways (Figure.S7E). Abnormal activation of the AKT/mTOR signaling pathway has been identified as a key feature of AML progression [21].

**Fig. 4:**
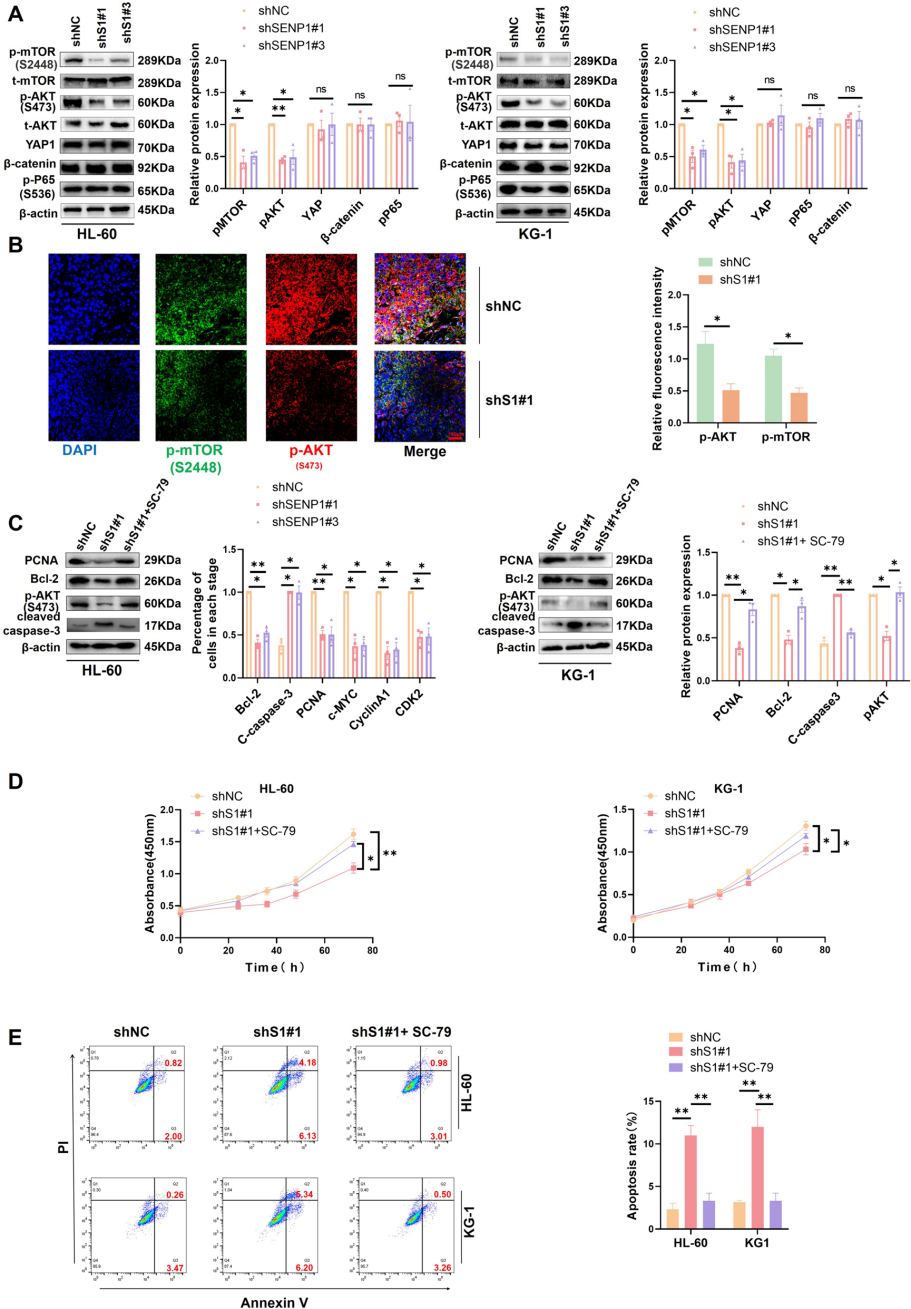
SENP1 promotes AML progression through AKT signal. **A** The changes in AKT signal, YAP1, β-catenin and p-P65 protein expression levels were detected by Western blot after SENP1 was silenced. **B** Immunofluorescence double labeling shows silencing of SENP1, impairing AKT and mTOR phosphorylation signals. **C** Comparison of proliferation and apoptosis related markers detected by Western Blot in shNC, shSENP1 and AKT activator groups. After using AKT phosphorylation activator, the expression of PCNA, cleaved caspase-3 and Bcl-2 returned. **D** CCK8 assay showed that activating AKT can restore the effect of silencing SENP1 on AML proliferation. **E** Apoptosis probes showed that activating AKT can restore the apoptotic effect of silencing SENP1 on AML

We then examined the changes in the expression of key molecules (p-AKT, p-mTOR, YAP1, p-P65, and β-catenin), which AML common signaling pathways in AML, after SENP1 knockdown. We found that AKT/mTOR signaling was significantly downregulated after SENP1 silencing (Fig. 4A). Next, we used the previous subcutaneous tumor tissue sections for double-fluorescence labeling. We found that silencing SENP1 significantly weakened AKT and mTOR phosphorylation (Fig. 4B). Addition of the AKT activator SC-79 restored the effect of SENP1 silencing on the expression of AML cell proliferation and apoptosis markers (Fig. 4C). CCK8 and apoptosis flow probes showed that SC-79 reversed the effect of SENP1 interference on AML cell proliferation and apoptosis (Fig. 4D and E).

### SENP1 regulates HDAC2 activity in a sumo-dependent manner

**Fig. 5:**
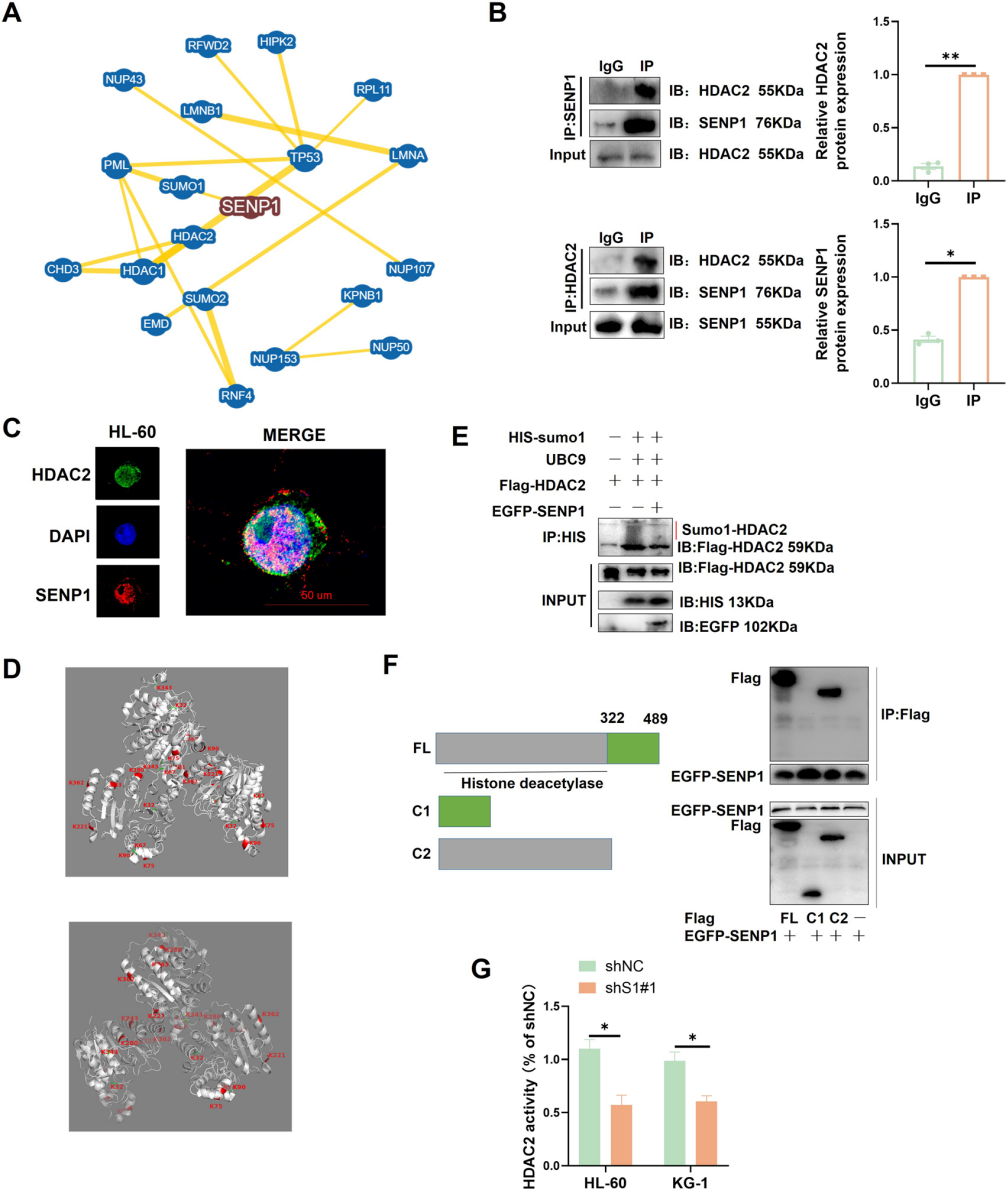
HDAC2 is the downstream sumo target of SENP1. **A** Conduct PPI analysis based on the bioGRID database to identify HDAC2 as a potential downstream target for SENP1. **B** Forward and reverse CO-IP identified HDAC2 and SENP1 interactions. **C** Dual immunofluorescence assay showed that SENP1 and HDAC2 were partially co-located in the nucleus. **D** The HDAC2 sumo sites reported or predicted in previous literature were identified based on the GPS SUMO database, the protein structure was downloaded from the PDB database, and Pymol software was used for protein structure visualization. **E** SUMO probe found that SENP1 can de-sumo modify HDAC2. **F** CO-IP identifies the specific domain of HDAC2 to which SENP1 binds. **G** HDAC2 enzyme activity probe was used to detect the effect of silencing SENP1 on HDAC2 activity

To further investigate the mechanism by which SENP1 activates AKT signaling, we excavated the BioGRID database and conducted protein-protein interaction network analysis (PPI analysis) [22]. Interestingly, we observed potential interactions between HDAC2 and SENP1 (Fig. 5A). HDAC2 was also located in the central region of the network, suggesting that it may play a crucial role in SENP1 function. Studies have reported that sumo1 modification of HDAC2 may inhibit HDAC2 function [23]. HDAC2 plays a key role in AKT activation by transcriptionally activating EGFR expression [17]. Importantly, HDAC2 has also been proven to be a critical regulatory molecule in AML development [18]. The CO-IP assay revealed that SENP1 was combined with HDAC2 (Fig. 5B). Fluorescence colocalization showed that SENP1 and HDAC2 were partially colocalized in the AML nucleus (Fig. 5C). We identified a large number of sumo modification sites in HDAC2 using the GPS SUMO database (Fig. 5D). The SUMO probe showed that after overexpression of SENP1, HDAC2 sumo modification was suppressed (Fig. 5E). HDAC2 has both deacetylase and non-deacetylase protein domains, and SENP1 mainly binds to the HDAC2 deacetylase protein domain (Fig. 5F). These data support the regulation of HDAC2 deacetylase activity by SENP1 in a sumo enzyme–dependent manner. We found that silencing SENP1 did not affect HDAC2 expression but significantly reduced HDAC2 activity (Figure.S8A and 5G).

### HDAC2 mediates SENP1 to regulate AKT-mTOR signaling in AML

**Fig. 6:**
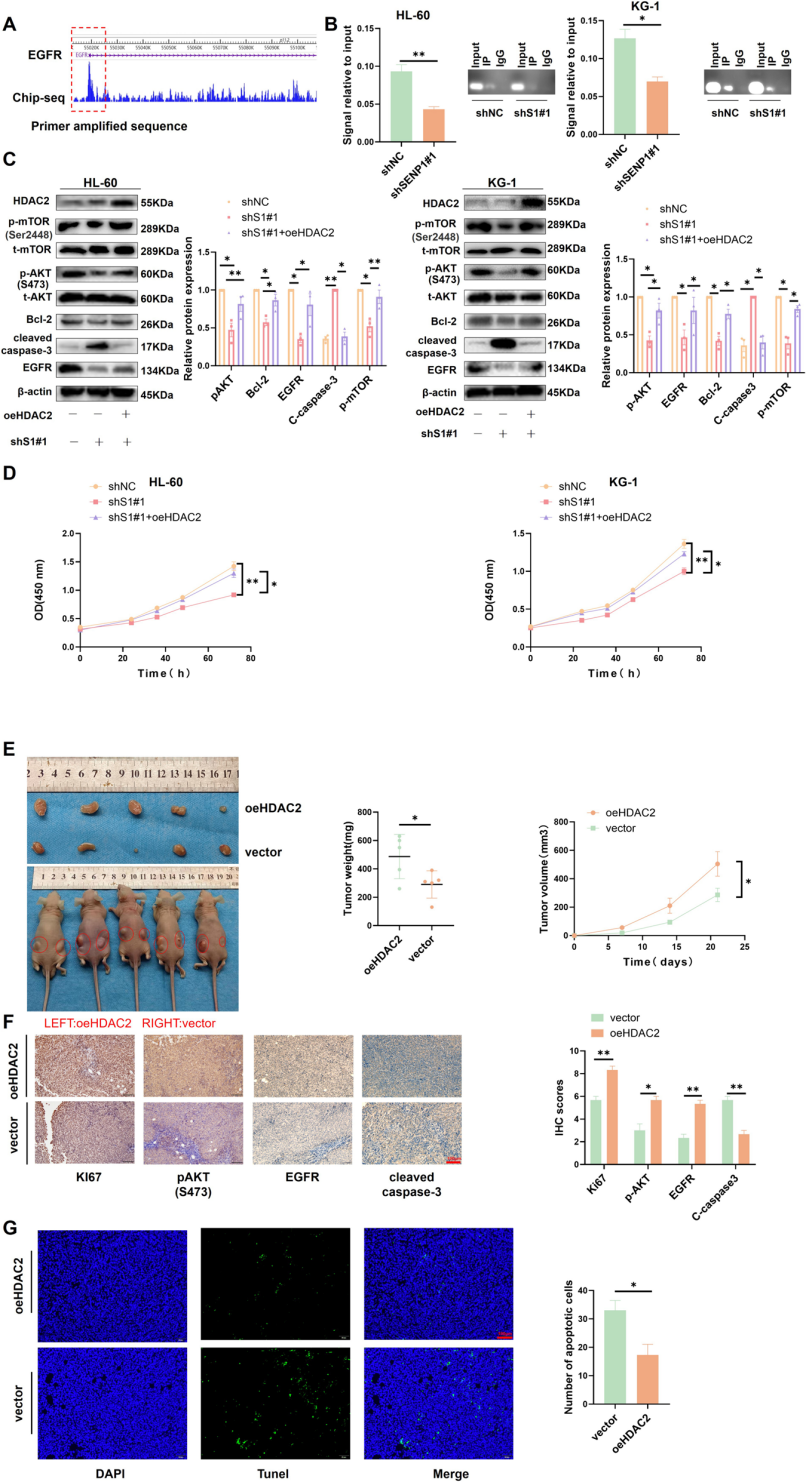
HDAC2 mediates SENP1 regulation of AKT signaling. **A** Exploring the CitromeDB database (CistromeDB: 93), HDAC2 has PEAK enrichment in the EGFR promoter. **B** After silencing SENP1, the ability of HDAC2 to bind to the EGFR promoter decreases. PCR products were used for gel electrophoresis for data visualization. **C** Overexpression of HDAC2 can restore the effect of silencing SENP1 on AKT phosphorylation signaling, EGFR, cleaved caspase-3 and Bcl-2. **D** Overexpression of HDAC2 can reverse the effect of silencing SENP1 on the proliferation of AML cells. **E** Overexpression of HDAC2 promotes AML (HL-60) growth in vivo. **F** Immunohistochemistry suggests overexpression of HDAC2, which can increase the expression of KI67, EGFR and pAKT (S473), and decrease cleaved caspase-3 expression. **G** Tunel staining showed that overexpression of HDAC2 reduced the apoptosis of AML cells in mice

By exploring the Cistrome DB database, we found that HDAC2 has peak enrichment in the EGFR promoter based on a previous ChIP-seq dataset (Fig. 6A) [24]. After silencing of SENP1, the ability of HDAC2 to bind to the EGFR promoter decreased (Fig. 6B). Overexpression of HDAC2 partially restored the effect of silencing SENP1 on EGFR, AKT phosphorylation signaling, and anti-apoptosis-related markers in AML cells (Fig. 6C and S9A). CCK8 experiments showed that overexpression of HDAC2 partially restored the silencing effect of SENP1 on AML cell proliferation (Fig. 6D). In the subcutaneous tumor experiment in nude mice, overexpression of HDAC2 promoted AML growth in vivo (Fig. 6E). Immunohistochemistry suggested overexpression of HADC2, enhancing KI67, EGFR, and p-AKT, and reducing apoptosis proteins (cleaved caspase-3) (Fig. 6F). TUNEL staining showed that HDAC2 overexpression reduced apoptosis (Fig. 6G). These results suggest that SENP1 activates the AKT pathway and promotes AML progression, at least in part dependent on HDAC2.

### SENP1, which is regulated by IGF2BP3 mediated m6A, is highly expressed in high-risk groups of AML

**Fig. 7:**
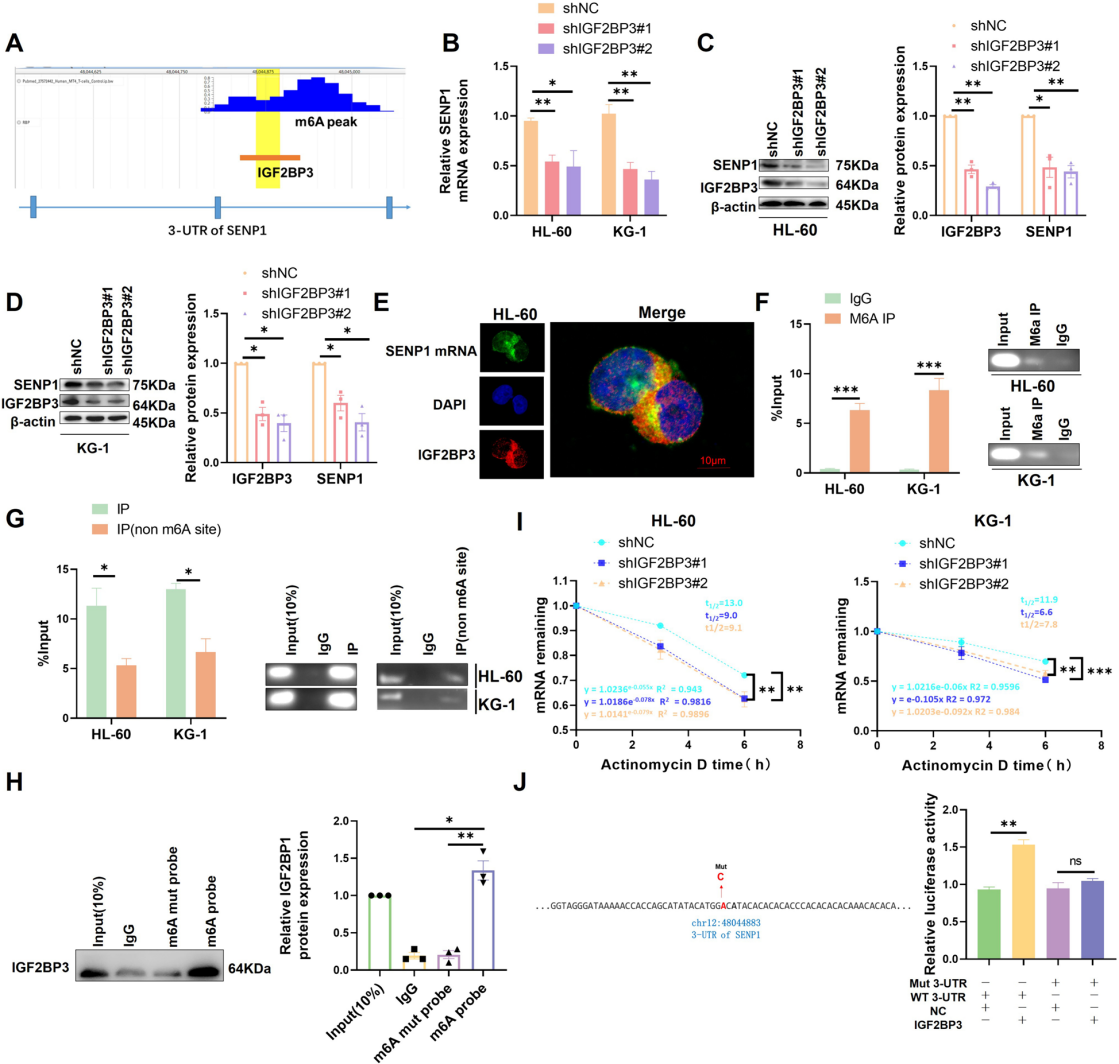
IGF2BP3 drives SENP1 expression in an m6A dependent manner. **A** Analyzing the RMVar database, it was found that IGF2BP3 binds to the SENP1 3-UTR region and there is a m6A site near the peak. **B** The effect of silencing IGF2BP3 on SENP1 mRNA expression was detected by RT-PCR. **C** The effect of silencing IGF2BP3 on the expression of SENP1 protein in HL-60 was detected by western blot. **D** The effect of silencing IGF2BP3 on the expression of SENP1 protein in KG-1 was detected by western blot. **E** The co-localization of SENP1 mRNA and IGF2BP3 protein was detected by FISH combined with immunofluorescence. **F** MeRIP-qPCR showed that there was m6A modification in the 3-UTR region of SENP1. PCR products were used for gel electrophoresis for data visualization. **G** RIP-PCR showed IGF2BP3 binding in the SENP1 3UTR region in a m6A manner. PCR products were used for gel electrophoresis for data visualization. **H** RNA pulldown assay detected that IGF2BP3 could bind SENP1 mRNA in an m6A-dependent manner. **I** mRNA attenuation experiment showed that silencing IGF2BP3 significantly promoted the degradation of SENP1 mRNA. **J** After mutating the IGF2BP3 binding m6A site identified by the aforementioned RMVar database, double luciferase reporter gene experiment showed that IGF2BP3 could not regulate mutant SENP1 3-UTR

Next, we explored the upstream regulatory mechanism of SENP1 in high-risk AML patients. In our previous study, we found that m6A is abnormally modified in the high-risk group of AML and systematically analyzed that the m6A reading protein IGF2BP3 is highly expressed in high-risk groups of AML and promotes AML development [4]. Therefore, we speculated that m6A may participate in SENP1 regulation in AML. By exploring the RM2Target database, we found that 14 RNA m6A regulators could potentially regulate SENP1 [25]. In order to screen the core factors, we conducted differential gene analysis between high-risk and low-risk AML groups and found that only YTHDF1, IGF2BP3, and FTO were significantly different (Figure.S10A). Survival analysis based on the TARGET and TCGA databases revealed that only IGF2BP3 was positively associated with poor prognosis in AML and pan-cancer patients (Figure.S10B-D). Correlation analysis revealed a positive correlation between IGF2BP3 and SENP1 expression (Figure.S10E and S10F). These findings suggest that IGF2BP3 is a key regulator of SENP1 expression. To determine the binding mechanism between IGF2BP3 and SENP1, we searched the RMVar database. The results showed that IGF2BP3 has the potential to bind to the SENP1 mRNA 3-UTR region (Fig. 7A) [26]. We found that SENP1 protein and mRNA levels decreased significantly after silencing IGF2BP3 (Fig. 7B-D). It is known that the IGF2BP1/2/3 protein family can bind m6A and contribute to the regulation of mRNA stability, so what role does IGF2BP1/2 play when it comes to SENP1. The results showed that silencing IGF2BP1/2 did not affect the regulation of SENP1 expression (Figure.S10G and S10H). These results suggested that SENP1 is specifically regulated by IGF2BP3.

Immunofluorescence combined with FISH revealed a large amount of colocalization of SENP1 mRNA and IGF2BP3 protein in AML cells (Fig. 7E). RIP experiments confirmed that IGF2BP3 and m6A could bind to the 3-UTR region of SENP1 mRNA (Fig. 7F and G). RNA PULL DOWN experiments also showed that IGF2BP3 binds to SENP1 mRNA in an m6A-dependent manner (Fig. 7H). mRNA half-life experiments showed that after silencing IGF2BP3, SENP1 mRNA decay accelerated (Fig. 7I). Mutation at the m6A site predicted by the aforementioned database and a double luciferase assay revealed that IGF2BP3 does not regulate mut-SENP1 (Fig. 7J).

### SENP1 mediates IGF2BP3's ability to regulate AKT/mTOR pathway activity and AML proliferation and anti-apoptosis

**Fig. 8:**
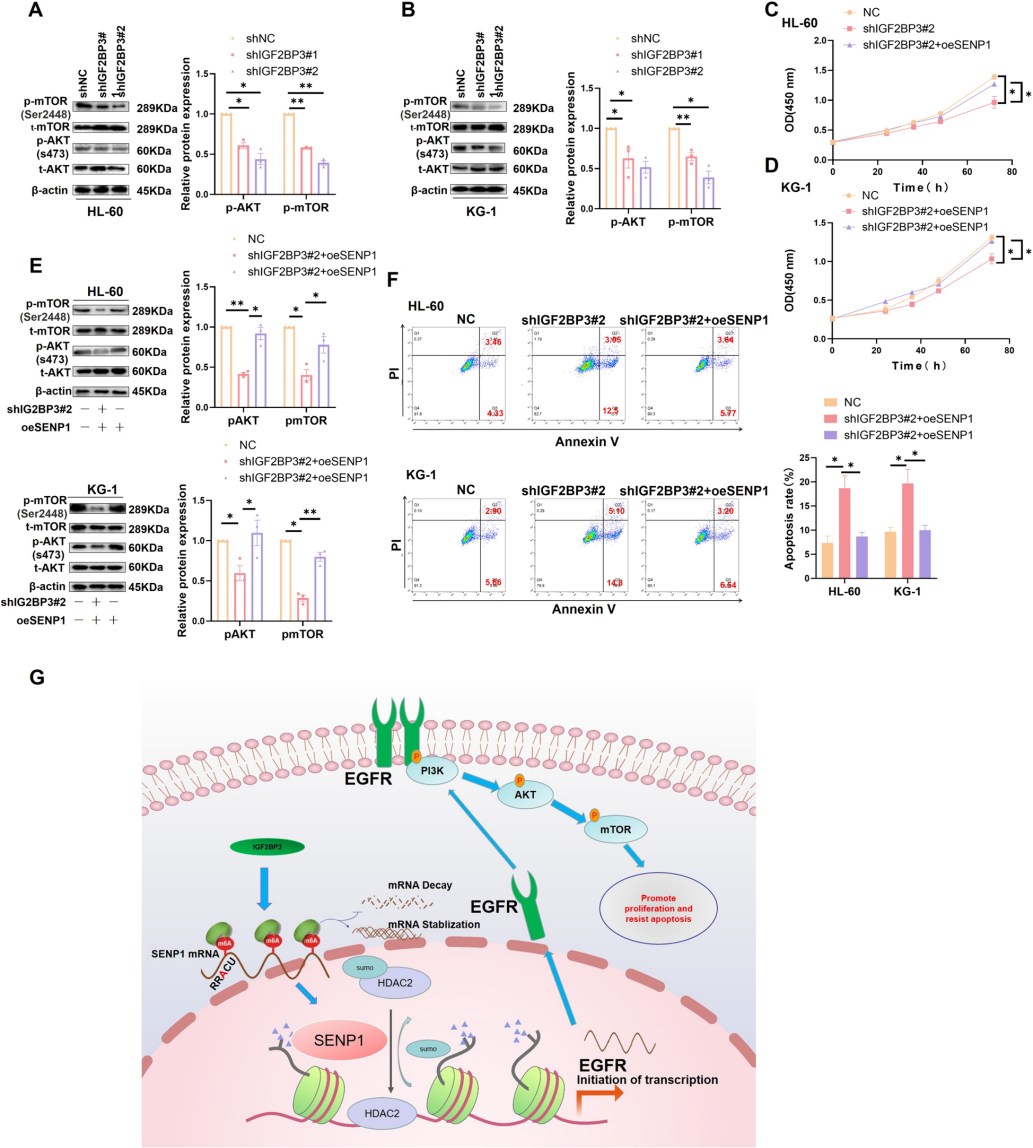
SENP1 mediates IGF2BP3's ability to regulate AKT/mTOR pathway activity and AML proliferation and anti-apoptosis. **A** After silencing IGF2BP3, EGFR and SENP1 expression were decreased, and AKT/mTOR pathway activity was down-regulated in HL-60. **B** After silencing IGF2BP3, SENP1 expression was decreased, and AKT/mTOR pathway activity was down-regulated in KG-1. **C** CCK8 showed that Over-expression of SENP1 could recover the effects of silencing IGF2BP3 on the proliferation of HL-60. (D) CCK8 showed that Over-expression of SENP1 could recovery the effects of silencing IGF2BP3 on the proliferation of KG-1. **E** Western blot assays showed that over-expression of SENP1 could reverse the effect of silencing IGF2BP3 on AKT pathway activity in AML cells. **F** Apoptosis flow cytometry showed that over-expression of SENP1 could reverse the effect of silencing IGF2BP3 on apoptosis of AML cells. **G** Research mechanism diagram

Next, we explored the role of SENP1 in IGF2BP3 regulated AML progression and signaling pathways. By re-analyzing our previous RNA-seq data of IGF2BP3 silencing in HL-60 [4], we found that IGF2BP3 function was significantly enriched in the PI3K/AKT/mTOR signaling pathway (Figure.S11A). Finally, we found that after silencing IGF2BP3, the expression of SENP1 and the activity of the AKT/mTOR pathway were significantly reduced (Fig. 8A and B). Functional recovery experiments showed that overexpression of SENP1 could reverse the effect of silencing IGF2BP3 on the proliferation, anti-apoptosis, and AKT pathway activities of AML cells (Fig. 8C-F).

## Discussion

Although great progress has been made in chemotherapy and radiotherapy, because of the lack of sensitive and specific diagnostic biomarkers and common drug resistance, the overall prognosis of AML patients has not improved significantly [27]. Therefore, it is necessary to define an effective evaluation marker to accurately predict tumor progression and survival as well as the treatment response of patients with AML. In this study, we found that AML was a suitable candidate for anti-SENP1. In vitro and in vivo experiments were performed to verify SENP1's specific role in AML.

At present, AML with an adverse prognosis is the main challenge for clinical AML treatment [27]. Although exploring the largest online AML dataset, including normal samples, we found that the differential expression of SENP1 was not significant in AML and healthy bone marrow. A large number of studies have found that many non-differential genes play an important role in the development of AML. For example, Katharina et al. found that RPS6KA1 is not differentially expressed in AML and normal human bone marrow, but it is one of the essential genes in AML [28]. Our analysis found that SENP1 was highly expressed in the AML with adverse effects group and was positively correlated with shortened patient survival time. SENP1 high expression group was also accompanied by more P53 mutations. According to the latest ELN guidelines, the P53 mutation is a marker of poor prognosis in AML. We further mined the BLOODSPOT database and found that karyotypes with poor prognosis had higher SENP1 expression. Moreover, in clinical correlation analysis, we found that SENP1 was more highly expressed in M1 type AML and less expressed in M4 type AML, and studies showed that M1 type AML had a poor prognosis [29]. Together, these data support the pivotal role of SENP1 in the malignant progression of AML.

Abnormal transmission of signaling pathways is one of the basic pathological mechanisms of AML. Recent studies have mapped the genome in large AML cohorts and found mutations in more than 60% of AML patients that confer abnormal "carcinogenic" activation, such as the PI3K/AKT signaling pathway, particularly in high-risk groups of AML patients [30]. Using the joint analysis of GSVA, GSEA, and PPI, we found that SENP1 is closely related to the AKT signaling pathway. The AKT signaling pathway can activate mTOR, which plays an important role in survival, death fate, drug resistance and stemness [30]. Energy metabolic reprogramming is a central feature of tumors. Recent studies have found that the AKT signaling pathway is closely related to tumor metabolism, and AKT can promote tumor cell glycolysis by activating MYC. Studies have also found that AML proliferation in vivo depends on glycolysis for energy supply [31]. Interestingly, previous studies have also reported that SENP1 enhances tumor cell glycolysis [7]. Next, we detected AML-related signaling pathway changes after interference with SENP1 expression. These results indicated that AKT/mTOR signaling is one of the most altered signaling pathways after silencing SENP1 expression.

SENP1 plays a role by influences the modification status of proteins and regulates downstream protein functional status or protein abundance. However, the downstream targets of SENP1 in AML remains unclear [32]. By exploring the bioGRID database and combining it with STRING analysis, we found that HDAC2 might be an important downstream target of SENP1. HDAC2 has been reported to have tumor-promoting properties in various malignancies, such as AML. HDAC2 has also been shown to transcriptionally activate EGFR and enhance AKT signaling. Previous studies have confirmed that sumo modifies HDAC2, which disturbs its transcriptional activity [17, 23]. Studies have confirmed the presence of more than 10 proven sumo sites for HDAC2 [33]. In this study, through sumo assays, we found that SENP1 affected HDAC2 sumo modifications. We found that SENP1 binds to the HDAC2 deacetylase protein domain. The HDAC2 active probe and Chip-qPCR probe showed that SENP1 increased HDAC2 transcriptional activity on EGFR. Therefore, we suggest that HDAC2 is a key mediator of SENP1 in the regulation of AKT signalling. A large number of studies have supported that m6A is abnormally modified in various malignant tumors, such as leukemia, and is involved in various signaling pathways, including AKT [34].

m6A is a novel RNA modification that has attracted considerable attention in recent years [35]. m6A has been reported to play a key role in tumor proliferation, death programming, microenvironment shaping, stemness maintenance, etc.. m6A may be a potential tumor treatment strategy due to its high plasticity [36]. For example, ALKBH5 has been reported to be involved in liver cancer development and immune microenvironment shaping, and IGF2BP3 promotes AML progression and stemness maintenance [19, 37]. In addition, m6A, as a post-transcriptional modification, has also been reported to interact with other modifications, such as histone methylation modification and ubiquitination, to promote tumor progression, and understanding its internal regulatory mechanism can further help us understand the regulatory network of tumors, such as AML [38]. The relationship between m6A and sumo modification and its potential role in AML require in-depth understanding. Consistent with previous reports that m6A mainly exists in 3-UTR and 5-UTR regions, we also found that SENP1 has m6A modification in 3-UTR. m6A modification in the 3-UTR mainly affects mRNA stability. IGF2BP3 promotes the stability of target mRNA by binding to m6A sites. We found that IGF2BP3 can promote the expression SENP1 via m6A modification. Furthermore, we analyzed our previous RNA-seq data from intervening IGF2BP3 in AML and found that the function of IGF2BP3 is enriched in the AKT/mTOR pathway [4]. Overexpression of SENP1 reversed the effect of silencing IGF2BP3 on the AKT pathway. Together, these results support our proposal of a novel mechanism by which IGF2BP3 drives SENP1 to activate AKT in an m6A dependent manner to promote AML. We also provided a new perspective on the relationship between m6A and sumo modifications.

This study also had some limitations. As there are more than 20 predicted and verified sumo sites on HDAC2, we did not look for a specific HDAC2 sumo site regulated by SENP1. However, we verified through various experiments that SENP1 binds to the HDAC2 deacetylase protein domain, supporting SENP1's influence on HDAC2 enzyme activity rather than protein expression. In addition, the effects of SENP1 on AML energy metabolism and the tumor microenvironment need to be further studied.

In conclusion, we have provided a broad perspective on the role of SENP1 in AML progression. Next, we explored the direct regulatory mechanisms of SENP1 upstream and downstream using in vivo and in vitro AML models, and proposed that SENP1 is driven by IGF2BP3 in m6A mode and activates AKT signaling via HDAC2 to promote AML progression (Fig. 8G).

### Supplementary Information

Supplementary Material 1.

## Data Availability

The original contributions presented in the study are included in the article/Supplementary Material . Further inquiries can be directed to the corresponding authors.

## Abbreviations

AML: Acute myeloid leukemia
SUMO: Small ubiquitin-like modifier
GSEA: Gene set enrichment analysis
GSVA: Gene set variation analysis
ELN: European Leukemia Net guidelines
FISH: Fluorescence in situ hybridization
DEGs: Differentially expressed genes
OHSU: Oregon Health and Science University
