# Supplementary material for: N6-methyladenosine-modified SENP1, identified by IGF2BP3, is a novel molecular marker in acute myeloid leukemia and aggravates progression by activating AKT signal via de-SUMOylating HDAC2
Source: Mol Cancer. 2024 May 31;23:116. doi: 10.1186/s12943-024-02013-y (PMC11141000; doi:10.1186/s12943-024-02013-y)
Supplement: Supplementary file 1 — Supplementary Material 1. [file 12943_2024_2013_MOESM1_ESM.doc]

supply material

Figure legend

**FigureS1.** Expression and prognosis of SENPs in AML

(A) Correlation between SENPs family proteins and prognosis of AML, based on OHSU AML dataset. (B) Correlation between SENPs family proteins and prognosis of AML, based on TARGET AML dataset.

**FigureS2.** Clinical characterstics and prognosis values of SENP1 in AML and pancarcinoma

(A) Clinical correlation analysis of SENP1 and AML classical mutations in AML using OHSU AML dataset. (B) Clinical correlation analysis of SENP1 and patient age in AML using OHSU AML dataset. (C) Exploring the MILE dataset showed no significant difference in the expression of SENP1 in bone marrow mononuclear cell of normal and AML patients. (D) Exploring the MILE dataset showed that SENP1 expression were significantly up-regulated in AML patients with adverse prognosis. (E) Pan cancer analysis SENP1 expression profile based on the TCGA pan cancer datasets. (F) Analyze the difference of SENP1 expression in tumor and normal tissues, based on TCGA pan cancer datasets.(G) Survival analysis was based on the TCGA pan-cancer datasets.

**FigureS3.** SENP1 expression was silenced in AML cell lines

SENP1 protein expression was detected in AML cell lines. (B) Using western blot to verify SENP1 silence efficiency based on three independent sequences of lentivirus.

**FigureS4.** Knockout of SENP1 inhibits AML cell proliferation and resistance to apoptosis

(A) Western blot was used to detect the knockout efficiency of SENP1 by sgRNA. (B) RT-PCR was used to detect the knockout efficiency of SENP1 by sgRNA. (C) CCK8 assay was used to detect the in vitro proliferation of AML cells after SENP1 knockout. (D) EDU probe was used to detect the in vitro proliferation of AML cells after SENP1 knockout. (E) Apoptosisprobe was used to detect the apoptosis rates of AML cells after SENP1 knockout. (F) The effect of SENP1 knockout on AML cell cycle was examined by cell cycle assay.

**FigureS5.**  (A) Subcutaneous tumor in nude mice after injection of HL-60 cells. (B) Fluorescence double labeling showed that the expression of Bcl-2 and PCNA decreased after silencing SENP1. (C) Western blot showed that after silencing SENP1, PCNA expression was downregulated.

**FigureS6.** Effect of SENP3 mutant on AML

(A) Western blotting detected transfection and overexpression efficiency of SENP1 mutants (R630L and K631M with flag label). (B) The effect of SENP1 mutant on the proliferation of THP-1 cells was detected by CCK8 assay. (C) The effect of SENP1 mutant on the proliferation of NB-4 cells was detected by CCK8 assay. (D) EDU probe was used to detect the effect of SENP1 mutant on the proliferation rate of AML cells. (E) Cell cycle probe was used to detect the effect of SENP1 mutants on cell cycle distribution in AML.

**FigureS7.** Biological Process, Signal Pathway and Molecular Function Analysis of SENP1

(A) Identification of DEGs in high and low SENP1 expression groups by differential gene analysis using OHSU AML dataset. (B) GO analysis of the aforementioned DEGs. (C) GSVA analysis of the aforementioned DEGs. (D) GSEA analysis of the aforementioned DEGs. (E) Take the intersection of the aforementioned GSEA and GSVA results. Note: mTOR and AKT pathways are specifically listed because they are overlap.

**FigureS8.** (A) Western blot showed that silencing SENP1 did not affect HDAC2 expression in AML

**FigureS9.** (A) RT-qPCR showed that overexpression of HDAC2 could reverse the effect of SENP1 silencing on EGFR mRNA

**FigureS10.** IGF2BP3 drives SENP1 expression in an m6A dependent manner

The expression of the m6A gene in AML patients with good prognosis, moderate poor prognosis and poor prognosis was compared. (F/I favorable/intermediate, A adverse.) (B) Analyzing the prognostic correlation of FTO, YTHDF1, and IGF2BP3 based on the TCGA database. (C) Analyzing the correlation between IGF2BP3 and the prognosis of pan cancer patients based on the TCGA database. (D) Analyzing the correlation between IGF2BP3 and the prognosis of AML patients based on the TARGET database. (E) Analyzing the correlation between IGF2BP3 and SENP1 mRNA expression based on the OHSU dataset. (F) Analyzing the correlation between IGF2BP3 and SENP1 mRNA expression based on the TCGA database.

**FigureS11.** IGF2BP1 and IGF2BP2 do not regulate SENP1 expression

(A) The effect of silencing IGF2BP1 on the expression of SENP1 protein was detected by western blot. (B) The effect of silencing IGF2BP2 on the expression of SENP1 protein was detected by western blot.

**FigureS12.** SENP1 mediates IGF2BP3's ability to regulate AKT/mTOR pathway activity

(A) Analysis of previous RNA-seq data of IGF2BP3 silenced in HL-60 revealed that the function of IGF2BP3 is enriched in the PI3K/AKT/mTOR pathway.

TableS1

Patient information

| Sample_ID | Age | Sex | Diagnosis | Cytogenetic | RISK | Category | Extract |
| --- | --- | --- | --- | --- | --- | --- | --- |
| 1 | 55 | male | Acute Myeloid  Leukaemia | inv(16)(p13.1q22) | favorable | M4 | RNA/protein |
| 2 | 78 | Female | Acute Myeloid Leukaemia | t(8;21)(q22;q22) | favorable | M2 | RNA/protein |
| 3 | 56 | male | Acute Myeloid Leukaemia | t(16;16)(p13.1;q22) | favorable | M4 | RNA/protein |
| 4 | 65 | male | Acute Myeloid Leukaemia | del(13)(q12q21) | intermediate | M1 | RNA/protein |
| 5 | 34 | Female | Acute Myeloid Leukaemia | normal | intermediate | M5 | RNA/protein |
| 6 | 64 | male | Acute Myeloid Leukaemia | Complex | adverse | M4 | RNA/protein |
| 7 | 26 | male | Acute Myeloid Leukaemia | t(6;9)(p22;q34) | adverse | M4 | RNA/protein |
| 8 | 61 | male | Acute Myeloid Leukaemia | inv(3)(q21q26) | adverse | M1 | RNA/protein |
| 9 | 27 | Female | Acute Myeloid Leukaemia | -5,del(7)(q22) | adverse | M0 | RNA/protein |
| 10 | 48 | Female | Acute Myeloid Leukaemia | del(7)(q11.2q22) | adverse | M5 | RNA/protein |

Table S2

Antibody information

| Antibody | Producer | Application |
| --- | --- | --- |
| SENP1 | 25349-1-AP, Proteintech | WB,IF,IP |
| PCNA | 10205-2-AP, Proteintech | WB,IF |
| AKT | 60203-2-Ig, Proteintech | WB |
| p-AKT | 9271,CST | WB,IHC,IF |
| mTOR | 66888-1-Ig, Proteintech | WB |
| p-mTOR | 5536, CST | WB,IHC,IF |
| β-actin | 81115-1-RR, Proteintech | WB |
| YAP1 | 13584-1-AP,Proteintech | WB |
| β-catenin | 51067-2-AP,Proteintech | WB |
| p-P65 | 3033,CST | WB |
| Bcl2 | 12789-1-AP,Proteintech | WB,IF |
| cleaved  caspase-3 | ab32042,ABCAM | WB,IHC |
| c-MYC | 10828-1-AP,Proteintech | WB |
| CyclinA1 | 13295-1-AP,Proteintech | WB |
| CDK2 | 10122-1-AP,Proteintech | WB |
| KI67 | 27309-1-AP,Proteintech | IHC |
| Flag | 14793，CST | IP |
| HDAC2 | 12922-3-AP,Proteintech | WB,IP,CHIP |
| HIS | 12698,CST | WB,IP |
| M6A | HA601049,HUABIO | RIP |
| EGFR | 66455-1-Ig,Proteintech | WB,IHC |
| Flag | HA601080,HUABIO | WB IP |
| IGF2BP1 | 22803-1-AP,Proteintech | WB |
| IGF2BP2 | 11601-1-AP,Proteintech | WB |
| IGF2BP3 | 14642-1-AP,Proteintech | WB,RIP |
| Mouse Anti-goat IgG for IP (HRP) | ab157532,Abcam | WB |

Table S3

PCR primer

| Gene | Forward primer (5’-3’) | Reverse primer (5’-3’) |
| --- | --- | --- |
| SENP1 | TTGGCCAGAGTGCAAATGG | TCGGCTGTTTCTTGATTTTTGTAA |
| SENP1 3-UTR (contain m6A site) | AAGTAGGAAAGGGCATGGGC | GGGCCCCAAAGTCCAGTTTA |
| SENP1 3-UTR (non contain m6A site) | CGGGTGGAGTTTGGACTGAT | TCAGAGGGTGGGGAGAGATG |
| EGFR promoter | AGCTAGACGTCCGGGCA | GGGGCTAGCTCGGGACT |
| IGF2BP3 | CTTCCTGGTGAAGACTGGCT | TATCCAGCACCTCCCACTGTAA |

TableS4

SENPs protein family expression profile in OHSU dataset

| gene | conMean | treatMean | logFC | pValue |
| --- | --- | --- | --- | --- |
| SENP8 | 0.753894602 | 0.036769641 | -0.717124961 | 1.14E-05 |
| SENP7 | 5.483478128 | 5.605953476 | 0.122475348 | 0.003050616 |
| SENP6 | 6.137983504 | 6.204281383 | 0.066297879 | 0.485957635 |
| SENP5 | 5.060730974 | 5.168931131 | 0.108200157 | 0.019263466 |
| SENP3 | 4.549947105 | 4.47829894 | -0.071648166 | 0.313357972 |
| SENP2 | 4.940763681 | 5.112850035 | 0.172086353 | 3.62E-06 |
| SENP1 | 4.383884227 | 4.610332868 | 0.226448641 | 2.90E-06 |

TableS5

Differentially expressed genes in high and low SENP1 groups

| logFC | AveExpr | t | P.Value | adj.P.Val |
| --- | --- | --- | --- | --- |
| SENP1 | 0.652214625 | 4.489287913 | 28.39890437 | 1.63E-102 |
| HMGXB4 | 0.767686606 | 4.796347268 | 16.69935498 | 4.25E-49 |
| ADNP2 | 1.220340839 | 5.619598649 | 15.51943856 | 7.73E-44 |
| HAUS2 | 0.497229373 | 5.19096378 | 14.93094074 | 2.95E-41 |
| BARD1 | 0.879895857 | 3.628757315 | 14.65115327 | 4.85E-40 |
| USO1 | -0.661447491 | 6.429738405 | -14.26489772 | 2.25E-38 |
| PPP1R21 | -0.632654235 | 5.330659613 | -14.17370456 | 5.53E-38 |
| ZNF780B | -1.245501795 | 2.35994477 | -14.12082872 | 9.30E-38 |
| CARD8 | -0.885643957 | 5.645806619 | -14.10517948 | 1.09E-37 |
| CDCA4 | 0.866220722 | 5.94575154 | 14.07900487 | 1.40E-37 |
| SETD1A | 0.56686674 | 5.829733624 | 14.00299797 | 2.96E-37 |
| RP11-379H18.1 | -1.108866253 | 3.195061332 | -13.95960735 | 4.53E-37 |
| CENPL | 0.700556497 | 2.942840107 | 13.95893249 | 4.56E-37 |
| HERC4 | -0.626701446 | 4.438815846 | -13.88241993 | 9.65E-37 |
| CTDSPL2 | 0.386084156 | 5.432626621 | 13.8713611 | 1.08E-36 |
| MED26 | 1.025078898 | 3.410019147 | 13.84519374 | 1.39E-36 |
| IKBKB | -0.584659706 | 5.552883355 | -13.73446768 | 4.09E-36 |
| MASTL | 0.766955694 | 4.839446345 | 13.51517323 | 3.43E-35 |
| SLC25A20 | -0.912300779 | 6.134757849 | -13.42911103 | 7.89E-35 |
| TFCP2 | -0.68248039 | 6.086440276 | -13.4151518 | 9.02E-35 |
| INTS8 | -0.53697144 | 5.720420365 | -13.41005517 | 9.48E-35 |
| SPG11 | -0.524354294 | 5.798064774 | -13.37516467 | 1.33E-34 |
| MON2 | -0.345971916 | 5.025395255 | -13.34471877 | 1.78E-34 |
| STAT6 | -0.747122816 | 7.87602868 | -13.26296059 | 3.90E-34 |
| RER1 | -0.448748261 | 6.053154229 | -13.26223594 | 3.93E-34 |
| SMPD4 | 0.498063663 | 5.498010454 | 13.23033818 | 5.34E-34 |
| TROVE2 | 0.434257042 | 5.491517793 | 13.22575294 | 5.58E-34 |
| DBF4P1 | 0.85425428 | 3.585774039 | 13.1995532 | 7.17E-34 |
| KRCC1 | -1.115355771 | 5.680381355 | -13.15169387 | 1.13E-33 |
| RLIM | 0.693578712 | 5.781509482 | 13.12711656 | 1.43E-33 |
| HSF2 | 0.879012693 | 5.159642006 | 13.10341274 | 1.80E-33 |
| LMAN2L | -0.937093633 | 4.211500669 | -13.09686869 | 1.91E-33 |
| FASTKD1 | -0.805153029 | 4.846115883 | -13.08064409 | 2.23E-33 |
| SNIP1 | 1.032095518 | 4.058003679 | 13.05531579 | 2.84E-33 |
| MAPKAPK2 | 0.71346027 | 7.849839246 | 13.04122222 | 3.25E-33 |
| ZSWIM7 | -0.569529637 | 4.909555345 | -12.96110505 | 6.97E-33 |
| UBA3 | -0.514322108 | 6.43231137 | -12.95125987 | 7.65E-33 |
| NDUFAF1 | -0.894634251 | 4.45998713 | -12.94785903 | 7.90E-33 |
| N4BP2L2 | -0.474797398 | 6.211792986 | -12.91758057 | 1.05E-32 |
| RFWD3 | 0.587207094 | 5.70879072 | 12.86706097 | 1.70E-32 |
| PDCD6IP | -0.52652249 | 6.736256743 | -12.8456296 | 2.08E-32 |
| GMEB2 | 0.696384386 | 4.886240915 | 12.8024407 | 3.13E-32 |
| C10orf12 | 0.682304686 | 4.738253278 | 12.76185244 | 4.60E-32 |
| UFD1L | 0.617135572 | 5.13492524 | 12.74569584 | 5.35E-32 |
| TIMMDC1 | -0.763233905 | 5.874674239 | -12.72827959 | 6.31E-32 |
| ZNF594 | -1.658794664 | 1.769694771 | -12.72029176 | 6.80E-32 |
| ZNF234 | -1.033090683 | 2.705701351 | -12.71640412 | 7.06E-32 |
| KRT8P12 | -1.029093435 | 3.714035557 | -12.68587798 | 9.41E-32 |
| DBF4 | 0.846520875 | 4.486089892 | 12.68217837 | 9.74E-32 |
| PPM1A | 0.559898325 | 5.847892472 | 12.67452327 | 1.05E-31 |
| USP30 | 0.744145739 | 4.414405605 | 12.66810662 | 1.11E-31 |
| CCDC61 | -0.799587092 | 3.932059324 | -12.63414309 | 1.53E-31 |
| DYNLL1 | -1.161370632 | 6.264456834 | -12.63082556 | 1.58E-31 |
| RP11-1398P2.1 | -1.316181036 | 3.333068482 | -12.5955844 | 2.20E-31 |
| C17orf53 | 1.093907066 | 3.254525092 | 12.58995793 | 2.32E-31 |
| CDK12 | 0.401040603 | 5.940001611 | 12.56686031 | 2.88E-31 |
| HIAT1 | 0.626036332 | 6.930599655 | 12.54604403 | 3.49E-31 |
| NUP153 | 0.51867995 | 6.559731278 | 12.54239499 | 3.62E-31 |
| ATP10D | -1.007932548 | 4.029089601 | -12.53664435 | 3.82E-31 |
| GTF2B | 0.541178624 | 6.535942662 | 12.53433518 | 3.90E-31 |
| GUCD1 | 0.457864128 | 6.879125792 | 12.52947105 | 4.08E-31 |
| UBE3B | -0.493010095 | 4.455813456 | -12.52575141 | 4.23E-31 |
| CRY2 | 0.777112828 | 4.614488513 | 12.51335391 | 4.74E-31 |
| SNAPC3 | -0.778509679 | 4.123040763 | -12.50119492 | 5.32E-31 |
| GOPC | -0.625879923 | 5.53138735 | -12.48565161 | 6.15E-31 |
| ZFP91 | 0.535703073 | 6.999267108 | 12.45843237 | 7.92E-31 |
| KB-1460A1.5 | -1.490724196 | 1.484834735 | -12.45772214 | 7.98E-31 |
| DCUN1D1 | 0.451915711 | 5.200182005 | 12.44957037 | 8.61E-31 |
| VPS13C | -0.588655702 | 6.523962443 | -12.43262867 | 1.01E-30 |
| TTC17 | -0.533836924 | 6.058058553 | -12.42275855 | 1.11E-30 |
| PDS5B | 0.406604002 | 5.374339288 | 12.41910182 | 1.14E-30 |
| NIT1 | -0.517857613 | 4.985766583 | -12.41891062 | 1.15E-30 |
| TBCK | -0.849216197 | 3.449677573 | -12.39295641 | 1.46E-30 |
| RP5-1091N2.9 | -1.099908172 | 3.695123633 | -12.38127749 | 1.63E-30 |
| RNASEL | -1.124275012 | 3.79512939 | -12.3801578 | 1.64E-30 |
| MRPL53 | -0.980680427 | 2.058634352 | -12.36930855 | 1.82E-30 |
| GOLT1B | 0.674369811 | 5.754971682 | 12.36814549 | 1.84E-30 |
| ZKSCAN1 | -0.917231343 | 5.787434215 | -12.36750792 | 1.85E-30 |
| SLC35B3 | -0.614918077 | 4.365099625 | -12.34612772 | 2.25E-30 |
| KRR1 | 0.653975646 | 6.259019191 | 12.3125401 | 3.08E-30 |
| PLK4 | 0.890461042 | 4.508458062 | 12.30562013 | 3.28E-30 |
| CTB-113P19.5 | -1.214038913 | 1.650857774 | -12.29899959 | 3.49E-30 |
| TMEM251 | -0.785278788 | 3.674738366 | -12.29669212 | 3.57E-30 |
| TIA1 | -0.728005485 | 6.600256666 | -12.26984139 | 4.57E-30 |
| CCNK | 0.484295561 | 5.870210874 | 12.26966971 | 4.58E-30 |
| ARID2 | 0.530534635 | 5.247933101 | 12.2536938 | 5.31E-30 |
| NMNAT1 | -1.107233095 | 2.695640909 | -12.25225794 | 5.38E-30 |
| AP1G1 | 0.630213045 | 5.692946198 | 12.24804358 | 5.60E-30 |
| ZNF687 | 0.503575936 | 5.723257143 | 12.24458676 | 5.78E-30 |
| MED9 | 0.706489135 | 4.281302575 | 12.24107357 | 5.97E-30 |
| C11orf54 | -0.664641251 | 4.193859702 | -12.2140016 | 7.67E-30 |
| WHAMM | 0.995394336 | 5.646725082 | 12.20445719 | 8.37E-30 |
| CENPCP1 | 0.832066601 | 3.595707955 | 12.20201598 | 8.57E-30 |
| PRIMPOL | -0.837620595 | 4.480726593 | -12.19665647 | 9.00E-30 |
| ZKSCAN3 | -1.147856873 | 2.874369677 | -12.18506459 | 1.00E-29 |
| TRMT13 | -0.77396443 | 4.683757217 | -12.17777688 | 1.07E-29 |
| SNX15 | -1.285898514 | -0.456737473 | -12.17362264 | 1.11E-29 |
| STX7 | -0.690932741 | 5.117125382 | -12.1582236 | 1.28E-29 |
| RPRD1B | 0.769604578 | 5.20160469 | 12.13640245 | 1.57E-29 |
| GDI2 | -0.555776514 | 8.741839731 | -12.10606516 | 2.07E-29 |
| MTIF3 | -0.756000278 | 6.726847601 | -12.09128025 | 2.38E-29 |
| BICD2 | -0.506854108 | 6.359430194 | -12.05453669 | 3.33E-29 |
| HSD17B4 | -0.901084358 | 5.859264478 | -12.04585805 | 3.61E-29 |
| MED6 | 0.607120506 | 3.923635129 | 12.03842582 | 3.86E-29 |
| MAD2L2 | 0.588640483 | 5.212638297 | 12.03086983 | 4.14E-29 |
| ZNF367 | 0.99883227 | 4.46841518 | 12.02540691 | 4.35E-29 |
| TCAIM | -0.674279827 | 3.98061424 | -12.02381465 | 4.42E-29 |
| DHFRL1 | -1.064304127 | 2.045524763 | -12.02161726 | 4.51E-29 |
| TMEM260 | -0.653844883 | 4.014997456 | -11.99694467 | 5.65E-29 |
| ZNF781 | -1.716197371 | -0.769581807 | -11.9927731 | 5.87E-29 |
| GALNT7 | -0.800486816 | 5.457631577 | -11.98939152 | 6.05E-29 |
| GNPAT | -0.455002907 | 6.132063747 | -11.97918868 | 6.64E-29 |
| NUP155 | 0.473821079 | 4.374351717 | 11.96491129 | 7.57E-29 |
| CNIH2 | 1.245356909 | 1.479565311 | 11.94561496 | 9.03E-29 |
| PARP4 | -0.689227973 | 7.087804394 | -11.93397575 | 1.00E-28 |
| FCF1P2 | -1.145385633 | 4.239171087 | -11.93219733 | 1.02E-28 |
| PROSC | -0.673201504 | 6.052117657 | -11.9311123 | 1.03E-28 |
| AP001372.2 | -1.402963791 | 1.897964607 | -11.92733261 | 1.07E-28 |
| TMEM43 | -0.634324625 | 7.093783945 | -11.92167698 | 1.12E-28 |
| HAUS8 | 0.688774544 | 2.551257753 | 11.90342619 | 1.33E-28 |
| SMARCAL1 | -0.643027655 | 4.681989085 | -11.88429892 | 1.58E-28 |
| GLT8D1 | -0.645252579 | 5.151448768 | -11.88089449 | 1.63E-28 |
| CCDC142 | -0.866313127 | 2.400097173 | -11.87929213 | 1.65E-28 |
| RP5-961K14.1 | 0.632839617 | 3.728874111 | 11.85193783 | 2.12E-28 |
| DCP1A | 0.73811179 | 5.457404242 | 11.84977093 | 2.17E-28 |
| RPE | -0.807538184 | 5.510077015 | -11.84895091 | 2.18E-28 |
| CENPC | 0.668875195 | 5.344963616 | 11.84773722 | 2.21E-28 |
| MRPS14 | -0.680615219 | 4.825271305 | -11.84694732 | 2.22E-28 |
| RP11-395A13.2 | -0.681458095 | 4.049617316 | -11.84516436 | 2.26E-28 |
| KIAA0319L | -0.506853405 | 5.410345644 | -11.82045768 | 2.83E-28 |
| LIN54 | 0.45045906 | 4.188523262 | 11.81047813 | 3.09E-28 |
| CLP1 | 0.82356833 | 4.491663923 | 11.80726744 | 3.19E-28 |
| ZNF407 | 0.466748039 | 3.446305231 | 11.80315863 | 3.31E-28 |
| PLEKHM2 | 0.56152545 | 7.182229569 | 11.80041209 | 3.39E-28 |
| RNF146 | -0.636158862 | 5.066192571 | -11.78195586 | 4.01E-28 |
| APOM | 0.859372747 | 2.850640033 | 11.76930792 | 4.50E-28 |
| FMO4 | -0.925994889 | 2.590336533 | -11.76094577 | 4.85E-28 |
| FIG4 | -0.683434376 | 5.299348948 | -11.75766531 | 5.00E-28 |
| GIN1 | -1.241266392 | 1.490842096 | -11.74620075 | 5.55E-28 |
| RIC8B | -0.71939724 | 2.900046406 | -11.74557794 | 5.58E-28 |
| SUV39H1 | 0.548361291 | 4.729597421 | 11.74391448 | 5.66E-28 |
| IDH1 | -1.150571236 | 5.612003917 | -11.74140928 | 5.79E-28 |
| EXOSC9 | 0.423142554 | 5.37035099 | 11.70510561 | 8.04E-28 |
| ATG4C | -0.878501302 | 4.74536683 | -11.7005023 | 8.39E-28 |
| MKKS | -0.77793773 | 4.229960673 | -11.69748087 | 8.62E-28 |
| THAP8 | -1.135760428 | 2.883193469 | -11.69495975 | 8.82E-28 |
| CCDC19 | 1.66691471 | 2.117081636 | 11.69054468 | 9.18E-28 |
| DYNC1I2P1 | -0.729853698 | 5.734850044 | -11.66452132 | 1.16E-27 |
| PCMTD1 | -0.525728416 | 5.981866669 | -11.65943063 | 1.22E-27 |
| ZNF559 | -0.780589494 | 3.054028777 | -11.65708922 | 1.24E-27 |
| SMUG1 | -0.691887683 | 3.091609808 | -11.65633885 | 1.25E-27 |
| RP11-5C23.1 | -1.117113235 | 3.332141221 | -11.64845352 | 1.34E-27 |
| RNF170 | -0.732507968 | 3.582986827 | -11.64669153 | 1.36E-27 |
| FPGT | -0.690048376 | 3.108255032 | -11.62812585 | 1.61E-27 |
| YIPF4 | -0.676126211 | 3.851905237 | -11.62042213 | 1.73E-27 |
| PRNCR1 | 0.41197877 | 4.686933373 | 11.61896404 | 1.75E-27 |
| KAT7 | 0.531929694 | 6.31566881 | 11.60088619 | 2.06E-27 |
| CTB-36H16.2 | -1.3365086 | 2.271309667 | -11.60001247 | 2.08E-27 |
| DUSP11 | 0.439707631 | 4.640992745 | 11.59919772 | 2.09E-27 |
| CTD-2085J24.4 | -1.458411365 | 1.947918553 | -11.58878673 | 2.30E-27 |
| EMD | 0.519856834 | 7.369453575 | 11.58751189 | 2.32E-27 |
| RP11-798K23.5 | -1.633533933 | 2.858710772 | -11.57302199 | 2.65E-27 |
| RELB | 1.272114368 | 5.325988983 | 11.57167712 | 2.68E-27 |
| RP5-1099D15.1 | 0.675572276 | 3.616866094 | 11.57042815 | 2.71E-27 |
| RAB10 | -0.884230684 | 7.105174973 | -11.56974193 | 2.73E-27 |
| KIAA2018 | 0.575187234 | 5.429086857 | 11.54841408 | 3.30E-27 |
| PMS1 | -0.680187586 | 4.574890454 | -11.54817927 | 3.31E-27 |
| TTC1 | -0.65616236 | 6.433121237 | -11.53661003 | 3.67E-27 |
| ZNF780A | -1.134361527 | 1.373407209 | -11.52758005 | 3.98E-27 |
| ACAD10 | -0.491413043 | 4.333077311 | -11.51431389 | 4.48E-27 |
| ZMYM1 | -0.650182821 | 3.869443376 | -11.50687316 | 4.79E-27 |
| FAM173B | -0.779103192 | 3.66897013 | -11.50595755 | 4.83E-27 |
| DUSP4 | 2.304476949 | 0.914956876 | 11.49891979 | 5.15E-27 |
| TLK2 | 0.414237579 | 4.488383479 | 11.48609141 | 5.77E-27 |
| SLC35A5 | -0.695554508 | 4.628989689 | -11.4728874 | 6.50E-27 |
| PKMP3 | -1.433472615 | 1.16161 | -11.45361255 | 7.72E-27 |
| DUSP8 | 2.423669126 | 1.450772825 | 11.45172598 | 7.85E-27 |
| ME2 | -0.556810972 | 6.975772331 | -11.44221983 | 8.55E-27 |
| COQ5 | -0.574790207 | 4.792654394 | -11.43474733 | 9.14E-27 |
| SYAP1 | 0.763779737 | 5.334769749 | 11.4289521 | 9.63E-27 |
| ZNF2 | -1.437421689 | 1.112043211 | -11.42083073 | 1.03E-26 |
| IFFO1 | -0.629165797 | 5.242578207 | -11.40875229 | 1.15E-26 |
| C6orf136 | 0.472583461 | 4.571234699 | 11.40788051 | 1.16E-26 |
| WDR92 | -1.073531883 | 1.619873512 | -11.40559078 | 1.19E-26 |
| PLEKHA8P1 | -0.799130413 | 3.803434808 | -11.39890502 | 1.26E-26 |
| ZNF248 | -0.973569694 | 2.877602241 | -11.39848733 | 1.26E-26 |
| CTNNAL1 | 1.237647496 | 4.272829307 | 11.39727378 | 1.28E-26 |
| USP42 | 0.651222286 | 4.325526513 | 11.39694935 | 1.28E-26 |
| PSMD11 | 0.408551478 | 5.502503607 | 11.39278428 | 1.33E-26 |
| TRAPPC12 | -0.494202255 | 4.687562158 | -11.39105644 | 1.35E-26 |
| SCRN3 | -0.54814143 | 3.989120542 | -11.39068269 | 1.35E-26 |
| ZNF271 | -0.393960011 | 4.655696879 | -11.38846824 | 1.38E-26 |
| FAM208A | -0.539883369 | 6.046940338 | -11.38475735 | 1.43E-26 |
| LARS | -0.829301902 | 6.378289388 | -11.38338128 | 1.45E-26 |
| ANKIB1 | -0.402472595 | 5.018025503 | -11.38087352 | 1.48E-26 |
| RLF | 0.793295916 | 5.668430317 | 11.36158087 | 1.76E-26 |
| SLC35C1 | -0.697307158 | 3.90126555 | -11.34973563 | 1.95E-26 |
| BAG4 | 0.736464202 | 5.126657998 | 11.3471751 | 2.00E-26 |
| MIER3 | 0.664484523 | 4.863150457 | 11.32807272 | 2.36E-26 |
| ZBTB43 | 1.051822467 | 5.205475377 | 11.32493216 | 2.43E-26 |
| RP11-97C16.1 | -0.974608886 | 2.865127471 | -11.32091936 | 2.52E-26 |
| WBP11 | 0.514304741 | 7.662094274 | 11.32036427 | 2.53E-26 |
| FAM83D | 1.26172619 | 3.028239836 | 11.31824836 | 2.58E-26 |
| RP11-644F5.11 | -1.112820455 | 3.697862803 | -11.29007077 | 3.31E-26 |
| MSANTD3 | 0.876696377 | 4.430686583 | 11.28886915 | 3.35E-26 |
| WBP1L | -0.881107244 | 5.930361418 | -11.25130536 | 4.67E-26 |
| AP1AR | -0.625308044 | 4.365983216 | -11.24360073 | 5.00E-26 |
| AC002056.3 | 0.480483607 | 4.920606579 | 11.23758035 | 5.28E-26 |
| TSPAN31 | -0.632010445 | 3.73299471 | -11.22875046 | 5.70E-26 |
| DYNC1I2 | -0.613902419 | 5.145574128 | -11.226398 | 5.82E-26 |
| MFSD3 | -0.98831186 | 4.483432645 | -11.22234821 | 6.04E-26 |
| MIER1 | 0.531984666 | 6.274013138 | 11.21696334 | 6.33E-26 |
| RP11-121A8.1 | -1.488571831 | 2.174137937 | -11.21535519 | 6.42E-26 |
| PAPD7 | 0.707244934 | 5.257764661 | 11.214791 | 6.45E-26 |
| ING4 | -0.715316739 | 5.617325778 | -11.21437727 | 6.48E-26 |
| KCNE3 | -1.332033558 | 5.112490288 | -11.19844573 | 7.46E-26 |
| CTD-2132N18.4 | -1.41071558 | 1.098034889 | -11.19772254 | 7.50E-26 |
| TEP1 | -0.536927282 | 5.33281615 | -11.18489413 | 8.40E-26 |
| SOS1 | -0.633096544 | 5.767413091 | -11.18381581 | 8.48E-26 |
| UBTD2 | -0.95704166 | 4.396497345 | -11.17829409 | 8.91E-26 |
| ZNF638 | -0.53795965 | 5.76274284 | -11.17555098 | 9.13E-26 |
| TTC37 | -0.507812076 | 5.770878393 | -11.16935544 | 9.64E-26 |
| KATNBL1 | -0.576351021 | 3.629216734 | -11.15944199 | 1.05E-25 |
| INTS3 | -0.764886722 | 5.870981767 | -11.1540123 | 1.10E-25 |
| IFITM10 | 1.795289549 | 1.646736735 | 11.14196295 | 1.23E-25 |
| COIL | 0.489334163 | 5.425586225 | 11.14150812 | 1.23E-25 |
| GTPBP1 | 0.629590596 | 5.564523211 | 11.14088569 | 1.24E-25 |
| SH3GL1 | 0.458832068 | 6.183564106 | 11.13954572 | 1.25E-25 |
| LYRM7 | -0.661460244 | 4.259124494 | -11.12863023 | 1.38E-25 |
| BBS10 | -0.885663756 | 3.455641792 | -11.12822561 | 1.38E-25 |
| AHCTF1 | 0.589714583 | 5.031987768 | 11.12557699 | 1.42E-25 |
| SMURF1 | 0.873190421 | 4.99509699 | 11.12543397 | 1.42E-25 |
| OSER1-AS1 | -1.043714154 | 2.822697301 | -11.12402241 | 1.44E-25 |
| RPL23AP82 | -0.849364781 | 2.239121198 | -11.12095338 | 1.48E-25 |
| TMEM184C | -0.636301294 | 4.805638764 | -11.11914143 | 1.50E-25 |
| CHAF1B | 0.851187505 | 3.605002461 | 11.11051323 | 1.62E-25 |
| TRMT10B | -0.503247041 | 4.282797361 | -11.10318403 | 1.73E-25 |
| CHTF8 | -0.573692362 | 5.843684995 | -11.10145568 | 1.75E-25 |
| C17orf62 | -0.780685776 | 5.570969858 | -11.09492009 | 1.86E-25 |
| TRIM27 | -0.627178874 | 5.090533115 | -11.07127432 | 2.28E-25 |
| PGBD2 | -1.158509121 | 2.91742041 | -11.06986827 | 2.31E-25 |
| N6AMT1 | -1.273125308 | 1.433075922 | -11.06386937 | 2.44E-25 |
| RBM15 | 0.679834565 | 5.735366231 | 11.05632295 | 2.60E-25 |
| PHYKPL | -0.551437268 | 5.397547676 | -11.0510824 | 2.73E-25 |
| RP11-46C24.7 | -0.939710145 | 3.073555562 | -11.03868278 | 3.04E-25 |
| RBL2 | -0.5427667 | 6.679421236 | -11.03807137 | 3.06E-25 |
| YOD1 | 1.115432241 | 5.569657097 | 11.03281827 | 3.20E-25 |
| CXorf24 | -0.920125766 | 2.083290611 | -11.02543703 | 3.41E-25 |
| AUNIP | 1.095018364 | 2.986992548 | 11.02537284 | 3.42E-25 |
| CTB-75G16.3 | -1.476402483 | 0.413479955 | -11.0205411 | 3.56E-25 |
| NEK9 | -0.620747099 | 6.173630796 | -11.01680029 | 3.68E-25 |
| TXNDC15 | -0.560759541 | 4.164554192 | -11.01454224 | 3.76E-25 |
| RP1-136B1.1 | -1.313397734 | 0.055496018 | -11.01344379 | 3.79E-25 |
| FBXO5 | 0.929030606 | 4.122903327 | 11.00643527 | 4.03E-25 |
| CTC-487M23.5 | -1.194390622 | 1.519655485 | -11.00269821 | 4.17E-25 |
| CXorf56 | 0.354874587 | 4.933313106 | 10.99718761 | 4.37E-25 |
| THUMPD3 | 0.35390942 | 5.003287452 | 10.98998972 | 4.66E-25 |
| ALMS1-IT1 | 1.242071022 | 2.145926535 | 10.98119588 | 5.03E-25 |
| CREBL2 | -0.768082726 | 4.963151099 | -10.97730229 | 5.20E-25 |
| NDUFAF5 | 0.865580048 | 3.448740986 | 10.97600477 | 5.26E-25 |
| BCL7B | 0.533957317 | 6.531935198 | 10.97241221 | 5.43E-25 |
| PAK2 | -0.475939224 | 7.508379241 | -10.9655906 | 5.76E-25 |
| NCAPH2 | 0.526810528 | 5.78287798 | 10.95810383 | 6.15E-25 |
| RP11-252A24.7 | -1.049936735 | 4.029605668 | -10.94202541 | 7.08E-25 |
| RP5-886K2.3 | -0.844641667 | 3.297063826 | -10.94038809 | 7.18E-25 |
| MAPK1IP1L | 0.432385094 | 6.681713488 | 10.92736826 | 8.04E-25 |
| FZR1 | 0.492889043 | 5.361649359 | 10.91329623 | 9.09E-25 |
| TNFAIP8L2 | -1.537681486 | 5.154341992 | -10.90854913 | 9.47E-25 |
| ACOX3 | -0.419152539 | 3.735281013 | -10.90616047 | 9.67E-25 |
| PIP4K2B | -0.618288047 | 6.406199443 | -10.89661529 | 1.05E-24 |
| RBM4B | -0.645345605 | 3.877213286 | -10.89479978 | 1.07E-24 |
| TMEM79 | -0.910326408 | 2.981408609 | -10.89362958 | 1.08E-24 |
| MTHFSD | -0.554298681 | 3.668982272 | -10.89274567 | 1.09E-24 |
| IKBIP | -0.858500504 | 3.775212172 | -10.89117118 | 1.10E-24 |
| GGPS1 | -0.514296677 | 4.693542532 | -10.8888091 | 1.12E-24 |
| C14orf159 | -0.646391614 | 5.738251487 | -10.88264987 | 1.19E-24 |
| PREPL | -0.929869423 | 4.022023227 | -10.88031486 | 1.21E-24 |
| TIMM17B | 0.351408643 | 5.300141728 | 10.87106596 | 1.31E-24 |
| SIN3A | 0.363167266 | 5.931893544 | 10.8697764 | 1.33E-24 |
| KCMF1 | 0.39234731 | 5.443691151 | 10.86364322 | 1.40E-24 |
| SAR1A | 0.488384133 | 6.012650695 | 10.85973543 | 1.45E-24 |
| AC142528.1 | -1.425957922 | 0.236226438 | -10.84968102 | 1.58E-24 |
| ORAI3 | -0.699724557 | 4.420394561 | -10.84881764 | 1.59E-24 |
| LA16c-313D11.12 | -1.323261562 | 0.250229436 | -10.84750652 | 1.61E-24 |
| RAE1 | 0.476223326 | 4.848724467 | 10.84694827 | 1.62E-24 |
| AASDH | -0.437269393 | 4.02907499 | -10.84632727 | 1.63E-24 |
| SNX1 | -0.381187619 | 5.831551665 | -10.8396987 | 1.72E-24 |
| TRADD | -0.6534419 | 4.738566858 | -10.8383006 | 1.74E-24 |
| RP11-74E22.3 | -1.673330506 | 1.149085949 | -10.83165775 | 1.85E-24 |
| RP5-1000K24.2 | 1.036773944 | 1.331775527 | 10.83113178 | 1.86E-24 |
| VPS41 | -0.57125291 | 4.885961667 | -10.82967574 | 1.88E-24 |
| PSME4 | 0.517520564 | 5.814902116 | 10.82916827 | 1.89E-24 |
| RNF20 | -0.463608248 | 5.671695777 | -10.8249059 | 1.96E-24 |
| PIGF | -0.570226828 | 2.95742366 | -10.82378929 | 1.98E-24 |
| SP3 | 0.366088971 | 6.426055428 | 10.81248486 | 2.18E-24 |
| VPS45 | -0.620427789 | 4.327274908 | -10.81099001 | 2.21E-24 |
| CHD1 | 0.639770922 | 6.60328652 | 10.81070994 | 2.22E-24 |
| SAP130 | 0.344420524 | 5.547002017 | 10.80568782 | 2.31E-24 |
| ZNRD1-AS1 | -0.824694595 | 2.15990761 | -10.79447 | 2.55E-24 |
| GLCE | -0.913488026 | 3.396191794 | -10.79431257 | 2.55E-24 |
| KIAA1033 | -0.46595251 | 6.591338226 | -10.7942213 | 2.55E-24 |
| AC012065.5 | -1.213041629 | 0.093672728 | -10.79280634 | 2.59E-24 |
| AC100830.4 | -1.214154777 | 1.362923356 | -10.78817226 | 2.69E-24 |
| BAIAP2-AS1 | -1.067265727 | 4.936870191 | -10.7846999 | 2.77E-24 |
| CKMT2-AS1 | -0.994718257 | 1.985148797 | -10.77778567 | 2.95E-24 |
| C9orf69 | -0.777261004 | 5.529538405 | -10.76758812 | 3.22E-24 |
| METTL7A | -1.014998744 | 6.640400108 | -10.76579392 | 3.27E-24 |
| MTFR2 | 0.860190802 | 2.959889997 | 10.74774017 | 3.82E-24 |
| UGDH | -0.972006808 | 3.570362033 | -10.73639309 | 4.21E-24 |
| JRKL | -0.915824466 | 3.741602496 | -10.73350419 | 4.32E-24 |
| CTC-462L7.1 | -1.274560365 | 0.080412253 | -10.7304007 | 4.43E-24 |
| RP1-68D18.3 | 1.761845371 | -0.954902704 | 10.73011294 | 4.44E-24 |
| CTNS | -0.594880299 | 4.383694832 | -10.72425826 | 4.68E-24 |
| TNFRSF12A | 1.533707849 | 3.097894737 | 10.72423741 | 4.68E-24 |
| RP11-644N4.1 | -1.451583901 | 0.566268837 | -10.71098334 | 5.24E-24 |
| TCTE1 | 1.621417391 | -0.123347843 | 10.70932214 | 5.32E-24 |
| RP11-372K14.2 | 1.707746105 | 3.997474292 | 10.70878975 | 5.34E-24 |
| PAQR8 | -1.753109122 | 3.169040928 | -10.70863894 | 5.35E-24 |
| RAD50 | -0.60041117 | 5.228731656 | -10.70695281 | 5.43E-24 |
| VTI1A | 0.424250631 | 4.856146507 | 10.70695033 | 5.43E-24 |
| PRC1 | 0.958110028 | 4.890387446 | 10.69751775 | 5.89E-24 |
| YY1AP1 | 0.347538764 | 6.564162543 | 10.69378933 | 6.08E-24 |
| KIAA1009 | -0.64050301 | 3.751037532 | -10.68245148 | 6.70E-24 |
| STX1A | 1.992099343 | 1.395265181 | 10.67241104 | 7.30E-24 |
| STK36 | -0.690680013 | 3.805298625 | -10.66181624 | 8.00E-24 |
| OMA1 | -0.731880184 | 4.390052396 | -10.66102642 | 8.06E-24 |
| COG8 | -0.530999662 | 3.24771842 | -10.66025112 | 8.11E-24 |
| EAPP | 0.463239189 | 6.87538033 | 10.64880218 | 8.95E-24 |
| ATAD5 | 0.719164937 | 4.267435256 | 10.64800321 | 9.01E-24 |
| TMOD2 | -1.157941695 | 3.19718927 | -10.64639108 | 9.13E-24 |
| RP11-10L12.4 | -0.844435753 | 1.538326202 | -10.64593003 | 9.17E-24 |
| KIF22 | 0.48308864 | 6.136724355 | 10.64512841 | 9.23E-24 |
| PDE12 | 0.440353504 | 4.365570545 | 10.64005373 | 9.64E-24 |
| RNASEH1 | 0.458200755 | 4.534925197 | 10.63741869 | 9.86E-24 |
| CEP19 | -1.235112145 | 3.383862397 | -10.6337241 | 1.02E-23 |
| IGIP | -1.333697564 | 1.20845824 | -10.63328151 | 1.02E-23 |
| TRIM13 | 0.701728857 | 3.950888548 | 10.63032593 | 1.05E-23 |
| ATG4D | 0.627506607 | 5.790427942 | 10.62910639 | 1.06E-23 |
| ATXN1L | 0.686405113 | 5.935210717 | 10.62573472 | 1.09E-23 |
| AL161915.1 | -0.8495476 | 2.939938075 | -10.62448554 | 1.10E-23 |
| EXOC1 | -0.411341442 | 5.37879371 | -10.62303355 | 1.12E-23 |
| GSG2 | 1.028712501 | 3.060947649 | 10.6209431 | 1.14E-23 |
| HOMEZ | -0.911713228 | 3.315693764 | -10.61770288 | 1.17E-23 |
| UHRF1BP1 | 0.51495617 | 5.135383389 | 10.61343228 | 1.21E-23 |
| SYCE2 | 0.995527603 | 2.860284562 | 10.6075155 | 1.27E-23 |
| EIF1AD | 0.573433923 | 5.290427685 | 10.60249438 | 1.33E-23 |
| MZT2A | 0.522132111 | 4.603865842 | 10.59659424 | 1.40E-23 |
| APAF1 | -0.732710068 | 6.012499428 | -10.59468444 | 1.42E-23 |
| ZNF818P | -1.121319341 | 3.327313792 | -10.5910017 | 1.47E-23 |
| STIL | 0.872600831 | 3.195200146 | 10.58842685 | 1.50E-23 |
| FUT5 | 1.575148963 | -2.58012438 | 10.58629062 | 1.53E-23 |
| DDX20 | 0.440075555 | 4.50333852 | 10.58514816 | 1.54E-23 |
| SLC25A11 | -0.474790163 | 6.210026162 | -10.58419471 | 1.56E-23 |
| ZNF283 | -0.72316719 | 1.899655864 | -10.57830851 | 1.64E-23 |
| RAD52 | -0.659792864 | 3.857492328 | -10.57778238 | 1.64E-23 |
| C3orf38 | 0.522823516 | 5.845105672 | 10.57719933 | 1.65E-23 |
| DNASE1 | -1.020490726 | 3.661066583 | -10.57270166 | 1.72E-23 |
| POM121C | 0.422033462 | 5.445690428 | 10.56707398 | 1.80E-23 |
| ZNF546 | -0.825121575 | 1.426127845 | -10.56649891 | 1.81E-23 |
| WTAP | 0.524912725 | 6.452151725 | 10.56398759 | 1.85E-23 |
| ZKSCAN2 | 0.686541647 | 2.920694708 | 10.5634133 | 1.86E-23 |
| RP11-5C23.2 | -1.170296534 | 1.289532643 | -10.55787449 | 1.95E-23 |
| PHF13 | 0.956779929 | 4.161256332 | 10.55615242 | 1.98E-23 |
| PRKAR1A | -0.703959431 | 7.088721239 | -10.55594992 | 1.98E-23 |
| RP11-229P13.20 | -1.506906541 | 4.542901805 | -10.55509523 | 2.00E-23 |
| GLIPR1 | -1.198526482 | 7.479676046 | -10.55360395 | 2.02E-23 |
| PPP3CB | -0.409142414 | 5.757716255 | -10.54739917 | 2.13E-23 |
| EDEM3 | -0.722030275 | 5.504800039 | -10.54345819 | 2.20E-23 |
| WDR62 | 1.004822711 | 2.799098687 | 10.54267877 | 2.22E-23 |
| SLC35A1 | -0.741131967 | 4.552650641 | -10.54245975 | 2.22E-23 |
| LONP2 | -0.315746843 | 5.505524789 | -10.54135709 | 2.24E-23 |
| PARG | -0.509094565 | 3.645451645 | -10.54005084 | 2.27E-23 |
| GATAD2A | 0.336711891 | 6.566718143 | 10.53607164 | 2.35E-23 |
| CWC15 | 0.346448213 | 6.159955163 | 10.53242533 | 2.42E-23 |
| SENP2 | 0.359392803 | 5.054677128 | 10.53139943 | 2.44E-23 |
| CTB-191K22.6 | -1.300449156 | -0.452976297 | -10.52863383 | 2.50E-23 |
| ISY1 | 0.399096859 | 4.217371528 | 10.5221645 | 2.64E-23 |
| SAP30BP | 0.438221946 | 5.228219665 | 10.52087623 | 2.67E-23 |
| ZNF691 | -0.731386938 | 3.523243884 | -10.51695443 | 2.76E-23 |
| ZNF692 | -0.747264764 | 4.983146129 | -10.51507872 | 2.81E-23 |
| ELK1 | 0.403912448 | 5.97740706 | 10.50692597 | 3.01E-23 |
| TP53BP2 | 0.53844891 | 6.377485001 | 10.50652445 | 3.02E-23 |
| FAM161B | -0.685451625 | 2.272139465 | -10.50470917 | 3.07E-23 |
| ETNK1 | -0.512623841 | 4.876141314 | -10.50167008 | 3.15E-23 |
| ZFP62 | -0.646773914 | 4.686750359 | -10.49019695 | 3.47E-23 |
| CTD-2089O24.1 | 0.680168363 | 3.388407153 | 10.48539445 | 3.62E-23 |
| FLI1 | -0.767658495 | 6.829528966 | -10.47792573 | 3.85E-23 |
| AC007228.11 | -1.495380719 | 0.254855545 | -10.4751952 | 3.94E-23 |
| CDK17 | 0.620293993 | 5.100826795 | 10.47499993 | 3.95E-23 |
| BPNT1 | -0.815783343 | 4.168027653 | -10.47332157 | 4.01E-23 |
| NUPL2 | -0.497027476 | 4.809976781 | -10.47000407 | 4.12E-23 |
| THUMPD2 | 0.575966219 | 4.598222662 | 10.46999519 | 4.12E-23 |
| RP11-585P4.5 | 0.930742798 | 2.875133466 | 10.46687319 | 4.23E-23 |
| MPHOSPH8 | -0.49208643 | 5.884304361 | -10.46531537 | 4.29E-23 |
| PPP1R11 | -0.402260144 | 6.970525799 | -10.46492238 | 4.30E-23 |
| C18orf21 | 0.487455697 | 5.223378988 | 10.46295029 | 4.38E-23 |
| TMX3 | -0.394333981 | 5.43467177 | -10.45489074 | 4.69E-23 |
| UBALD2 | 0.701935685 | 8.440705597 | 10.43877243 | 5.37E-23 |
| STAM2 | -0.441567049 | 4.6378811 | -10.43780374 | 5.42E-23 |
| CTD-2366F13.1 | -1.519460117 | -0.12533449 | -10.4374681 | 5.43E-23 |
| DNAJC3-AS1 | -0.984124898 | 2.272141364 | -10.4346364 | 5.56E-23 |
| COA3 | -0.875307913 | 4.48448394 | -10.43264856 | 5.66E-23 |
| RBM6 | -0.584856686 | 6.668105712 | -10.43144767 | 5.72E-23 |
| FAM118B | -0.837079053 | 3.484001087 | -10.4277366 | 5.90E-23 |
| OIP5-AS1 | -0.634699952 | 6.385410309 | -10.41216618 | 6.73E-23 |
| NFKBIB | 0.558352718 | 5.728145928 | 10.40338549 | 7.25E-23 |
| PSMD7 | 0.311517989 | 7.394940351 | 10.39951723 | 7.49E-23 |
| MAD2L1BP | 0.673275805 | 5.187343727 | 10.39375415 | 7.87E-23 |
| GGCX | -0.477839143 | 3.77018161 | -10.39370144 | 7.87E-23 |
| ALG6 | -0.473431819 | 3.740401249 | -10.39054589 | 8.08E-23 |
| CRTC1 | -0.484992096 | 4.193009883 | -10.38901809 | 8.19E-23 |
| GABPB1 | 0.606688906 | 5.376919102 | 10.3841682 | 8.53E-23 |
| RP4-758J18.2 | -0.613890852 | 4.689242094 | -10.37940036 | 8.88E-23 |
| VKORC1L1 | -0.778560344 | 4.43780715 | -10.37069695 | 9.56E-23 |
| CDK16 | 0.475863254 | 5.845773252 | 10.36946003 | 9.66E-23 |
| TRIM14 | -0.742481433 | 6.082886536 | -10.36640108 | 9.91E-23 |
| FAM76B | 0.513199428 | 5.520139247 | 10.36615999 | 9.93E-23 |
| DOCK8 | -0.529538843 | 7.015769537 | -10.36531153 | 1.00E-22 |
| ARHGAP8 | 1.815630449 | -2.733213559 | 10.3602716 | 1.04E-22 |
| C16orf52 | -0.575312975 | 2.710332417 | -10.35660475 | 1.08E-22 |
| NME6 | -0.645626426 | 3.362206173 | -10.35043312 | 1.13E-22 |
| NUDT9 | -0.500248021 | 4.650004996 | -10.34993563 | 1.14E-22 |
| ZNF74 | -0.884591951 | 3.289116376 | -10.34992536 | 1.14E-22 |
| RP11-160O5.1 | 1.312467496 | -0.321404521 | 10.34884107 | 1.15E-22 |
| SNAPIN | -0.597291102 | 5.703215129 | -10.34699852 | 1.17E-22 |
| RP4-714D9.5 | -1.220124928 | -0.507470771 | -10.3441044 | 1.20E-22 |
| MBIP | 0.559677353 | 4.883130108 | 10.33724514 | 1.27E-22 |
| HIVEP1 | 0.608195961 | 4.585875119 | 10.33508396 | 1.29E-22 |
| DCTN6 | 0.551819442 | 5.207667554 | 10.32760428 | 1.38E-22 |
| GTF2H1 | -0.607306938 | 4.823440942 | -10.32752347 | 1.38E-22 |
| RNF103 | 0.808908997 | 4.807608805 | 10.32652247 | 1.39E-22 |
| KB-431C1.5 | -1.257714568 | 0.217178151 | -10.32608326 | 1.39E-22 |
| KDM3A | 0.603320737 | 5.680691485 | 10.32401669 | 1.42E-22 |
| CSRP2BP | -0.70837835 | 3.866758318 | -10.31907874 | 1.48E-22 |
| EIF2AK3 | 0.629700119 | 4.724145961 | 10.30970548 | 1.60E-22 |
| RP5-1112D6.4 | -1.012867725 | 2.735938238 | -10.30837037 | 1.62E-22 |
| SPAG9 | 0.479380022 | 5.470283839 | 10.30683255 | 1.64E-22 |
| PHKB | -0.461378715 | 5.605527714 | -10.30319334 | 1.69E-22 |
| TRAF6 | 0.673571982 | 5.234363895 | 10.30190398 | 1.71E-22 |
| CENPN | 0.754285831 | 3.629274599 | 10.30137221 | 1.72E-22 |
| DNAJC19P9 | -0.691047498 | 4.856328891 | -10.2961569 | 1.79E-22 |
| C10orf118 | 0.575510637 | 4.976381055 | 10.29381736 | 1.83E-22 |
| C1GALT1C1 | -0.753677978 | 5.013923046 | -10.28808799 | 1.92E-22 |
| XXbac-B444P24.13 | -0.985884459 | 0.786145257 | -10.28752112 | 1.93E-22 |
| DHX8 | 0.330944379 | 5.355872242 | 10.28463842 | 1.98E-22 |
| PATL1 | 0.462332976 | 6.989880126 | 10.28187237 | 2.02E-22 |
| WDR5B | -0.74859568 | 3.469081842 | -10.28179988 | 2.02E-22 |
| TMEM106A | -0.750927957 | 5.192509818 | -10.28146196 | 2.03E-22 |
| SRRM1 | 0.316265865 | 6.789761399 | 10.27891394 | 2.07E-22 |
| DDX17 | -0.651017088 | 8.989818776 | -10.27810872 | 2.09E-22 |
| GNA13 | 0.685298366 | 7.558204998 | 10.27293412 | 2.18E-22 |
| HIF1A | 1.078723853 | 8.107143467 | 10.27199253 | 2.20E-22 |
| IPO13 | -0.636766169 | 4.59489073 | -10.26901646 | 2.25E-22 |
| ZNF397 | -0.601305093 | 3.498962093 | -10.26515219 | 2.33E-22 |
| PSMD12 | 0.481232685 | 5.479510957 | 10.26213206 | 2.39E-22 |
| TIFA | 0.724446452 | 4.078622117 | 10.26136385 | 2.40E-22 |
| RP4-813F11.4 | -1.168088435 | 0.233203188 | -10.25507212 | 2.53E-22 |
| RIMBP3 | -1.8364117 | -1.05974286 | -10.25312172 | 2.57E-22 |
| TRIP11 | -0.550013884 | 4.540948246 | -10.25128122 | 2.61E-22 |
| HNRNPLP1 | 0.597757635 | 2.811542306 | 10.24944004 | 2.65E-22 |
| RQCD1 | 0.307564608 | 6.007122238 | 10.2478784 | 2.69E-22 |
| LARP4B | -0.505403253 | 5.694032648 | -10.24656271 | 2.72E-22 |
| UBAP1 | 0.546620115 | 7.31027266 | 10.24550383 | 2.74E-22 |
| C4orf46 | 0.521020055 | 5.010006644 | 10.24226587 | 2.82E-22 |
| ZNF485 | -1.06528673 | 1.789715693 | -10.24218808 | 2.82E-22 |
| BRAP | 0.351856869 | 5.537741153 | 10.24100138 | 2.85E-22 |
| TMCC1-AS1 | -1.39737721 | 2.730338562 | -10.24017529 | 2.87E-22 |
| SETDB2 | -0.629150743 | 5.181609241 | -10.22606325 | 3.23E-22 |
| ECHDC1 | -0.658238383 | 4.771300846 | -10.22388729 | 3.29E-22 |
| C2orf43 | -0.731678535 | 3.985887537 | -10.22347024 | 3.30E-22 |
| AC093838.4 | 0.651469997 | 5.121361307 | 10.22251421 | 3.33E-22 |
| USP39 | 0.267808195 | 6.34814073 | 10.2212918 | 3.36E-22 |
| USP38 | 0.447829652 | 5.190389793 | 10.21631545 | 3.50E-22 |
| AGO2 | 0.692784238 | 5.356249248 | 10.21362141 | 3.58E-22 |
| ARL11 | -0.867854521 | 4.431274665 | -10.21275633 | 3.61E-22 |
| TRAF3IP3 | -0.795157025 | 5.995047551 | -10.21029017 | 3.69E-22 |
| COX15 | -0.540714733 | 5.128342496 | -10.20968757 | 3.70E-22 |
| TBXAS1 | -0.803863069 | 6.035387521 | -10.20445694 | 3.87E-22 |
| RP11-35G9.3 | -0.808388798 | 1.756076635 | -10.19990354 | 4.02E-22 |
| C21orf49 | -1.22288367 | 0.593102213 | -10.19520414 | 4.18E-22 |
| SRSF1 | -0.633539037 | 7.767405238 | -10.19413041 | 4.22E-22 |
| NBR1 | -0.514708512 | 5.94776829 | -10.19346864 | 4.24E-22 |
| AL031663.2 | -1.21103747 | 0.194170291 | -10.19316833 | 4.25E-22 |
| RP11-266L9.4 | -1.103096283 | 1.517585444 | -10.19214011 | 4.29E-22 |
| CTD-2083E4.5 | 0.64842617 | 3.180736026 | 10.18974049 | 4.38E-22 |
| SPOP | -0.468782493 | 5.174859906 | -10.18862661 | 4.42E-22 |
| AC002310.12 | -1.019788183 | 2.941726607 | -10.18005011 | 4.75E-22 |
| KCTD9 | 0.619987015 | 4.924028519 | 10.17758231 | 4.85E-22 |
| SPAG4 | 1.041610004 | 1.97757571 | 10.17711522 | 4.86E-22 |
| SUMO3 | 0.499595255 | 8.039274531 | 10.17693575 | 4.87E-22 |
| SPDEF | 1.34770342 | -1.738223556 | 10.17012684 | 5.16E-22 |
| GID4 | -0.542607557 | 4.025339106 | -10.16820288 | 5.24E-22 |
| RP11-686D22.4 | -1.438814148 | 1.520332026 | -10.16710468 | 5.29E-22 |
| GEMIN7 | -0.484855828 | 5.023762914 | -10.16149516 | 5.54E-22 |
| CHURC1 | -0.649025166 | 5.348487563 | -10.15978473 | 5.62E-22 |
| PSMC1 | 0.560973675 | 4.277534821 | 10.15673633 | 5.77E-22 |
| SEC14L2 | 1.765891532 | -0.604455752 | 10.15365977 | 5.92E-22 |
| YEATS4 | 0.432129308 | 6.090557295 | 10.15304194 | 5.95E-22 |
| ZNF267 | 0.616526508 | 4.93892565 | 10.15227526 | 5.99E-22 |
| KCNK7 | 1.254434209 | 2.219229765 | 10.1380265 | 6.74E-22 |
| FBXO22 | -0.425183513 | 3.93496556 | -10.13675769 | 6.81E-22 |
| CTD-2035E11.5 | -1.049468708 | 2.573623396 | -10.13030203 | 7.19E-22 |
| TXNDC11 | 0.486170357 | 6.270062955 | 10.12716553 | 7.38E-22 |
| DBT | -0.491791439 | 2.836965028 | -10.12711229 | 7.38E-22 |
| CASZ1 | 0.825233133 | 2.86351303 | 10.12195297 | 7.71E-22 |
| CDK11B | 0.433628347 | 5.982864561 | 10.12040777 | 7.81E-22 |
| ZNF800 | 0.648588954 | 5.569306491 | 10.10597085 | 8.80E-22 |
| POGLUT1 | -0.534911437 | 3.811804358 | -10.10361238 | 8.98E-22 |
| XAB2 | 0.353976981 | 6.085501525 | 10.100159 | 9.24E-22 |
| MITD1 | -0.544420192 | 4.414990676 | -10.09980007 | 9.27E-22 |
| FAM105B | 0.567676438 | 4.933699193 | 10.09790105 | 9.41E-22 |
| HCG18 | -0.651987627 | 3.711107866 | -10.09049168 | 1.00E-21 |
| FRS2 | 0.412594685 | 4.506965754 | 10.08951077 | 1.01E-21 |
| LINC00638 | -1.506357158 | 1.060999275 | -10.08639501 | 1.04E-21 |
| PIGM | -0.629309614 | 3.957030832 | -10.08592926 | 1.04E-21 |
| IKBKE | -0.591454542 | 5.290841642 | -10.08403932 | 1.06E-21 |
| RP11-523H24.3 | -1.150193238 | -0.237394228 | -10.07989095 | 1.09E-21 |
| NDUFV2 | 1.35029637 | 4.492876785 | 10.07768447 | 1.11E-21 |
| QRICH1 | 0.315687744 | 6.732803567 | 10.07343626 | 1.15E-21 |
| BRCC3 | -0.395017147 | 5.21619143 | -10.07187528 | 1.17E-21 |
| RP11-78O7.2 | -1.267951491 | 2.70560054 | -10.0700491 | 1.19E-21 |
| MED11 | -0.669076211 | 4.228752976 | -10.06920566 | 1.19E-21 |
| SUPV3L1 | 0.525050304 | 4.968851652 | 10.0688903 | 1.20E-21 |
| RP11-24B19.4 | -1.094197892 | 1.051807016 | -10.06782738 | 1.21E-21 |
| RP11-395L14.17 | 0.444070567 | 5.17686095 | 10.06584516 | 1.23E-21 |
| NLGN3 | 1.130399605 | 2.041806655 | 10.06494747 | 1.24E-21 |
| VPS36 | -0.471500796 | 6.14961894 | -10.06387961 | 1.25E-21 |
| ATP6V0E1 | -0.702841566 | 7.857944362 | -10.05717857 | 1.32E-21 |
| ABRACL | -0.574763843 | 7.126580841 | -10.05595876 | 1.33E-21 |
| FAM209B | 1.092680411 | 2.145999345 | 10.05136008 | 1.39E-21 |
| REXO1 | 0.394056874 | 6.162856357 | 10.0464075 | 1.44E-21 |
| LRRC32 | 2.133865246 | -0.172987159 | 10.04576803 | 1.45E-21 |
| GALK2 | -0.533819843 | 3.726855728 | -10.03991267 | 1.52E-21 |
| CCDC12 | 0.362459824 | 4.713540398 | 10.03852496 | 1.54E-21 |
| RANBP9 | 0.366600072 | 7.110479813 | 10.03586125 | 1.58E-21 |
| ARRDC1 | -0.429044576 | 6.497147296 | -10.0336951 | 1.60E-21 |
| METTL14 | 0.339641793 | 4.253139514 | 10.03148996 | 1.63E-21 |
| C19orf45 | 2.000814332 | 0.043045318 | 10.03147497 | 1.63E-21 |
| C5orf15 | -0.521659079 | 6.689661633 | -10.02545071 | 1.72E-21 |
| BTK | -0.665048308 | 7.301905421 | -10.0185889 | 1.82E-21 |
| SIRT5 | -0.654295028 | 3.799372184 | -10.01812106 | 1.82E-21 |
| CHRM4 | 1.31552466 | 1.538989442 | 10.0176723 | 1.83E-21 |
| IRF3 | -0.444448899 | 6.277743558 | -10.0173432 | 1.84E-21 |
| PRKRIRP7 | 0.557482121 | 3.968325925 | 10.01721484 | 1.84E-21 |
| PCNXL4 | -0.493875332 | 4.932691085 | -10.01549984 | 1.86E-21 |
| CCDC53 | -0.498921546 | 5.429481862 | -10.01241281 | 1.91E-21 |
| SNRK | 0.490614975 | 6.439211465 | 10.01136263 | 1.93E-21 |
| TSC22D2 | 0.585223631 | 4.669547479 | 10.00718085 | 2.00E-21 |
| KIAA0100 | -0.413709867 | 7.613505595 | -10.00368217 | 2.06E-21 |
| C1D | 0.565316587 | 4.142621325 | 9.998317962 | 2.15E-21 |
| STK38 | -0.683951126 | 7.095979871 | -9.995784099 | 2.20E-21 |
| VN1R108P | -1.463214226 | 0.856108549 | -9.995474548 | 2.20E-21 |
| MAPK6 | 0.606498898 | 6.270864775 | 9.991493465 | 2.27E-21 |
| PREP | -0.384413568 | 5.742914635 | -9.987848621 | 2.34E-21 |
| ELP3 | -0.546730515 | 4.84661385 | -9.979338558 | 2.51E-21 |
| AC003665.1 | -1.288011183 | 0.642943593 | -9.978867056 | 2.52E-21 |
| IL34 | 1.328394268 | -1.909955192 | 9.968269343 | 2.76E-21 |
| GEMIN6 | -0.959921293 | 2.237634685 | -9.967490081 | 2.77E-21 |
| GBA2 | -0.406639298 | 5.761077232 | -9.967007392 | 2.78E-21 |
| CEBPA-AS1 | -1.458568856 | -0.007625529 | -9.963533134 | 2.86E-21 |
| ZNF317 | 0.454891778 | 6.29895758 | 9.962242269 | 2.90E-21 |
| SERINC3 | -0.406206383 | 7.550666216 | -9.961457706 | 2.91E-21 |
| TRGV8 | -1.427203902 | 1.940043051 | -9.960104812 | 2.95E-21 |
| DGCR2 | -0.489514824 | 5.740853358 | -9.958261647 | 2.99E-21 |
| RP11-474D14.2 | -1.3033866 | 0.564112197 | -9.957161549 | 3.02E-21 |
| TMEM42 | -0.566899565 | 3.918498495 | -9.956202176 | 3.04E-21 |
| C5orf54 | -1.287784435 | 0.764476926 | -9.948768323 | 3.24E-21 |
| PTTG1 | 0.772854583 | 5.113202575 | 9.944093097 | 3.36E-21 |
| TTC9C | -0.708145841 | 4.655581429 | -9.941763411 | 3.43E-21 |
| ZNF451 | 0.53544387 | 5.41580652 | 9.941202754 | 3.44E-21 |
| REL | 0.632712281 | 5.279783718 | 9.940872313 | 3.45E-21 |
| CTC-459F4.3 | -0.977430244 | 1.751077605 | -9.940772044 | 3.46E-21 |
| ZNF404 | -1.758119057 | 1.158321076 | -9.940294037 | 3.47E-21 |
| STYXL1 | -0.602905674 | 5.578198479 | -9.938442943 | 3.52E-21 |
| RP11-259N19.1 | -1.506607216 | 2.528789778 | -9.935499138 | 3.61E-21 |
| ORC1 | 0.98497691 | 4.729539255 | 9.934999386 | 3.62E-21 |
| DIS3 | 0.313612379 | 5.576124673 | 9.929782506 | 3.78E-21 |
| EDC3 | -0.558449587 | 4.678888912 | -9.928044223 | 3.84E-21 |
| C9orf156 | 0.522926245 | 4.234274032 | 9.928005152 | 3.84E-21 |
| LZTS3 | 1.444280637 | 1.26295885 | 9.927725374 | 3.85E-21 |
| NUDT18 | -0.571230647 | 4.763140472 | -9.926136794 | 3.90E-21 |
| METTL6 | -0.556331909 | 2.828005316 | -9.921269208 | 4.06E-21 |
| ZNF181 | -0.807776263 | 2.721073331 | -9.90465596 | 4.65E-21 |
| MOB4 | 0.463285722 | 4.948581981 | 9.902844135 | 4.72E-21 |
| RPEL1 | -0.916812136 | 1.345989083 | -9.901574315 | 4.77E-21 |
| TAPSAR1 | -0.829733616 | 5.950210683 | -9.900639724 | 4.81E-21 |
| COPZ1 | -0.308461041 | 6.567432632 | -9.899460624 | 4.85E-21 |
| NUDCD1 | -0.718140402 | 4.165731408 | -9.893965911 | 5.08E-21 |
| HCG4P3 | -1.005336945 | 1.638256162 | -9.893369074 | 5.10E-21 |
| RP11-53O19.3 | -1.204676697 | 1.435923702 | -9.882649126 | 5.57E-21 |
| TRMT1L | -0.725245028 | 4.025773354 | -9.873207514 | 6.02E-21 |
| COPS2 | 0.493228757 | 6.604759702 | 9.873079217 | 6.03E-21 |
| E2F2 | 1.226141227 | 3.8931538 | 9.872912608 | 6.04E-21 |
| RP11-283I3.6 | -0.973161547 | 2.697406963 | -9.871815523 | 6.09E-21 |
| CRELD1 | -0.705047851 | 4.724914552 | -9.867925769 | 6.29E-21 |
| YAF2 | 0.480327304 | 3.906153599 | 9.864245425 | 6.48E-21 |
| NIPAL3 | -0.589053009 | 4.268324108 | -9.862972517 | 6.55E-21 |
| LANCL2 | 0.542681891 | 4.118091028 | 9.862848969 | 6.56E-21 |
| RNF138 | 0.457738293 | 6.883001777 | 9.86270963 | 6.56E-21 |
| RP11-32D17.4 | 1.217313079 | -0.21375317 | 9.861536838 | 6.63E-21 |
| RP11-226L15.5 | -0.907535906 | 3.237122787 | -9.860467209 | 6.69E-21 |
| PARS2 | -1.38674581 | 1.43052634 | -9.85876265 | 6.78E-21 |
| GPRC5A | 2.218505092 | -3.083990515 | 9.857815182 | 6.83E-21 |
| JOSD1 | 0.59954576 | 7.087214435 | 9.852337189 | 7.15E-21 |
| TMOD3 | -0.49928599 | 6.297895545 | -9.852194045 | 7.15E-21 |
| UGP2 | -0.582440614 | 6.197123296 | -9.850206048 | 7.27E-21 |
| HPS3 | -0.524701043 | 5.053961569 | -9.849751412 | 7.30E-21 |
| CHM | -0.565420984 | 3.471403011 | -9.849140783 | 7.34E-21 |
| CTD-2349P21.9 | -0.925043364 | 3.503074459 | -9.847754478 | 7.42E-21 |
| BAP1 | 0.271392206 | 6.626626693 | 9.844509043 | 7.62E-21 |
| AHCYL2 | -0.554576714 | 4.069329065 | -9.842857226 | 7.72E-21 |
| RNPS1 | 0.381527419 | 6.485057473 | 9.842376993 | 7.75E-21 |
| SLC38A10 | -0.756910779 | 6.600729988 | -9.841051133 | 7.84E-21 |
| RP11-536C5.7 | 1.052196559 | 1.028972925 | 9.840251858 | 7.89E-21 |
| C1orf123 | -0.417135062 | 5.068547091 | -9.831864986 | 8.45E-21 |
| ZNF41 | -0.582414268 | 3.583140285 | -9.826718712 | 8.81E-21 |
| RICTOR | 0.55441653 | 5.773303173 | 9.825775483 | 8.88E-21 |
| ZNF252P | -0.662873363 | 3.216386347 | -9.822294511 | 9.14E-21 |
| ATG16L2 | -0.626733453 | 7.193060301 | -9.819600232 | 9.34E-21 |
| SMN1 | 0.53022722 | 4.282188703 | 9.816741708 | 9.56E-21 |
| PCYOX1 | -0.716108937 | 4.503936113 | -9.81406042 | 9.77E-21 |
| SLC27A4 | -0.733704378 | 4.453036061 | -9.813715745 | 9.80E-21 |
| MRPS31 | -0.499978259 | 5.131720279 | -9.805257585 | 1.05E-20 |
| SEC22B | -0.384859668 | 6.654151045 | -9.798195468 | 1.11E-20 |
| TUFT1 | 1.35110386 | 1.171019092 | 9.797539048 | 1.12E-20 |
| CBLL1 | 0.439661426 | 5.550689195 | 9.796812399 | 1.13E-20 |
| HEXDC | -0.61372043 | 5.005416229 | -9.79192111 | 1.17E-20 |
| THNSL1 | -1.206167369 | 1.892098127 | -9.790094948 | 1.19E-20 |
| CHCHD4 | -0.815556664 | 4.32979008 | -9.790092632 | 1.19E-20 |
| SEC23IP | -0.470982133 | 5.329123208 | -9.782118379 | 1.27E-20 |
| RP5-1050D4.5 | -0.956075523 | 2.568591291 | -9.78056185 | 1.28E-20 |
| RP4-798A17.5 | -1.105063008 | 0.591978243 | -9.777963172 | 1.31E-20 |
| C16orf87 | 0.762423728 | 4.048640617 | 9.776715926 | 1.33E-20 |
| RP11-574K11.28 | -1.002589659 | -0.15641342 | -9.774801901 | 1.35E-20 |
| PREB | -0.646769263 | 5.678801481 | -9.773532749 | 1.36E-20 |
| TYW5 | 0.474762607 | 3.32002658 | 9.768757327 | 1.41E-20 |
| RAD51AP1 | 1.024799721 | 4.09747178 | 9.767184974 | 1.43E-20 |
| RP11-469M7.1 | 1.28584017 | 4.005044619 | 9.765378824 | 1.45E-20 |
| SNX17 | -0.359743093 | 6.966208865 | -9.765212672 | 1.46E-20 |
| RP11-51J9.6 | -1.062680928 | 0.085913541 | -9.764908818 | 1.46E-20 |
| TMEM69 | -0.495460167 | 5.444408106 | -9.764056144 | 1.47E-20 |
| PAXIP1-AS2 | -0.710583036 | 3.402168045 | -9.761374696 | 1.50E-20 |
| SMEK2 | -0.347809011 | 6.316740906 | -9.759418773 | 1.53E-20 |
| MINA | -0.734555184 | 3.89270738 | -9.758161171 | 1.54E-20 |
| CKLF-CMTM1 | -1.231653208 | 0.714655867 | -9.756614033 | 1.56E-20 |
| MTPAP | 0.450532671 | 4.131544912 | 9.751668399 | 1.63E-20 |
| LRRC8D | -0.542736037 | 5.702696111 | -9.747717734 | 1.68E-20 |
| ZMYM4 | -0.418944753 | 6.087387925 | -9.745499402 | 1.71E-20 |
| TCEANC2 | -0.557105721 | 4.012009794 | -9.74460568 | 1.72E-20 |
| PLEKHA3 | 0.429664401 | 3.412614326 | 9.737467804 | 1.82E-20 |
| ZNF696 | -0.791875038 | 2.291133731 | -9.731529289 | 1.91E-20 |
| NIFK-AS1 | -0.652204648 | 3.618047633 | -9.725724216 | 2.01E-20 |
| CWF19L1 | 0.422054986 | 5.508088558 | 9.72510876 | 2.02E-20 |
| SOS1-IT1 | -1.012818544 | 2.635234999 | -9.724788549 | 2.02E-20 |
| GMPR2 | -0.451789638 | 6.36180704 | -9.71957157 | 2.11E-20 |
| ARPC5L | 0.711861446 | 5.808029686 | 9.708655652 | 2.31E-20 |
| FAM209A | 0.914276886 | 2.949131378 | 9.704623413 | 2.38E-20 |
| SREK1 | 0.484843795 | 6.200259213 | 9.703947684 | 2.40E-20 |
| KAT6A | 0.449051726 | 6.26777656 | 9.703014728 | 2.41E-20 |
| CCDC9 | 0.559243129 | 5.090181286 | 9.701432037 | 2.44E-20 |
| CTD-2528L19.6 | -0.976338207 | 2.39760893 | -9.699561357 | 2.48E-20 |
| COQ10B | 0.70954326 | 5.376321232 | 9.69374234 | 2.60E-20 |
| RP11-57H14.4 | -0.589266694 | 2.427745861 | -9.689447534 | 2.69E-20 |
| RP11-872D17.8 | 1.342920264 | -1.104055755 | 9.689206862 | 2.70E-20 |
| TMEM165 | -0.4125993 | 5.471524045 | -9.684758881 | 2.80E-20 |
| RP11-884K10.7 | -0.690338292 | 2.588909387 | -9.683464845 | 2.83E-20 |
| RNF38 | 0.392859402 | 6.551402928 | 9.68182911 | 2.87E-20 |
| NCDN | -0.609978762 | 4.332019679 | -9.680824189 | 2.89E-20 |
| LRRC57 | 0.493560145 | 5.398521165 | 9.68068896 | 2.89E-20 |
| EIF2B4 | -0.277684204 | 4.785373786 | -9.679140354 | 2.93E-20 |
| RP4-761J14.10 | -0.75966013 | 2.198153478 | -9.677275362 | 2.97E-20 |
| RP11-635N19.1 | -1.027914463 | 1.40398283 | -9.676353182 | 3.00E-20 |
| RP11-649A18.7 | 0.738300919 | 2.587762937 | 9.675057233 | 3.03E-20 |
| MYBL2 | 1.315959979 | 5.913116456 | 9.673862769 | 3.06E-20 |
| HDHD2 | -0.549042478 | 5.143218 | -9.6729804 | 3.08E-20 |
| DIABLO | 0.666126525 | 3.05618583 | 9.672007632 | 3.10E-20 |
| RP11-85F14.5 | -0.715470236 | 2.75606341 | -9.670560485 | 3.14E-20 |
| RTF1 | 0.330607141 | 6.529758509 | 9.66664929 | 3.24E-20 |
| BRCA2 | 0.787024961 | 3.90265194 | 9.662912877 | 3.34E-20 |
| POM121 | 0.37537303 | 5.689617725 | 9.662520336 | 3.35E-20 |
| DDX19B | 0.466056858 | 4.388230968 | 9.659724552 | 3.43E-20 |
| TMEM218 | -0.694122021 | 3.666237538 | -9.658422184 | 3.46E-20 |
| PDE4D | 1.003152788 | 4.744271131 | 9.655164948 | 3.56E-20 |
| SENP8 | -1.196523531 | 0.31516152 | -9.65497616 | 3.56E-20 |
| CHD2 | 0.470196211 | 6.831348883 | 9.651884413 | 3.65E-20 |
| HIBCH | -0.611126659 | 2.773526754 | -9.646724029 | 3.81E-20 |
| PCYOX1L | -0.659067084 | 4.798581628 | -9.642678461 | 3.93E-20 |
| STAT2 | -0.57184441 | 6.698983791 | -9.642613686 | 3.94E-20 |
| COA4 | -0.510893466 | 5.383593647 | -9.642230252 | 3.95E-20 |
| CTD-2196E14.4 | -0.779607051 | 3.208757059 | -9.635872401 | 4.16E-20 |
| RSPH3 | -0.722614842 | 3.208988036 | -9.634907609 | 4.19E-20 |
| AP001258.4 | -0.860725348 | 1.960111686 | -9.634723297 | 4.19E-20 |
| ARL5B | 0.822149636 | 5.587398658 | 9.634685201 | 4.20E-20 |
| RP4-614O4.12 | -1.102288994 | 1.126104502 | -9.633137074 | 4.25E-20 |
| POLE3 | 0.427963195 | 7.826462521 | 9.63184208 | 4.29E-20 |
| POLH | -0.641689308 | 4.485675748 | -9.631136371 | 4.32E-20 |
| DUSP28 | 0.515374567 | 3.432480061 | 9.629144842 | 4.39E-20 |
| AC008746.12 | -1.071378024 | 1.366904807 | -9.627940216 | 4.43E-20 |
| BCL3 | 1.049117862 | 6.158521025 | 9.625438962 | 4.52E-20 |
| ZNF616 | -0.9905 | 2.619714466 | -9.622302392 | 4.64E-20 |
| LARP1 | -0.520309294 | 7.265846032 | -9.622155792 | 4.64E-20 |
| ZNF137P | -0.819497167 | 3.192063832 | -9.621499118 | 4.67E-20 |
| METTL13 | -0.813922449 | 4.84910607 | -9.621193724 | 4.68E-20 |
| PER2 | 0.557250478 | 4.387712022 | 9.620690754 | 4.70E-20 |
| CTD-2006H14.2 | -0.999113272 | 2.7683641 | -9.617292946 | 4.83E-20 |
| RBM38 | 1.056894956 | 7.215259649 | 9.613773005 | 4.97E-20 |
| COG6 | -0.551106456 | 3.434613513 | -9.612723984 | 5.01E-20 |
| FAM103A1 | 0.608207543 | 3.840314141 | 9.60812406 | 5.20E-20 |
| DTWD1 | -0.444821039 | 4.196613025 | -9.602218902 | 5.45E-20 |
| HJURP | 1.392206694 | 3.569928492 | 9.600779635 | 5.52E-20 |
| RP11-793H13.11 | -0.892872877 | 1.606993974 | -9.600721845 | 5.52E-20 |
| ZFYVE20 | 0.471025685 | 4.181101072 | 9.600030525 | 5.55E-20 |
| CCNE2 | 0.9472685 | 3.147856754 | 9.59877237 | 5.61E-20 |
| RMND1 | -0.466275429 | 4.973570877 | -9.598119764 | 5.64E-20 |
| CTA-223H9.9 | -1.306144664 | -0.388400817 | -9.597369164 | 5.67E-20 |
| ATG2A | 0.730181674 | 5.298370365 | 9.594996103 | 5.78E-20 |
| GMNN | 0.670839611 | 4.678646607 | 9.594934093 | 5.78E-20 |
| DESI2 | 0.386612763 | 5.118413718 | 9.593639634 | 5.84E-20 |
| CACUL1 | 0.448073364 | 5.476115521 | 9.592923199 | 5.88E-20 |
| APPBP2 | -0.358590502 | 4.275962038 | -9.592228695 | 5.91E-20 |
| UTP6 | -0.5339467 | 5.226941243 | -9.589990593 | 6.02E-20 |
| PYCARD | -0.780790243 | 7.201415072 | -9.589308762 | 6.05E-20 |
| C1R | 1.100237451 | 1.635527801 | 9.585432092 | 6.24E-20 |
| DDN | 1.227406137 | 0.215254571 | 9.576338666 | 6.71E-20 |
| SEC22C | 0.383024079 | 5.028104873 | 9.57254052 | 6.92E-20 |
| PPP4R2 | 0.4344664 | 6.185694154 | 9.572510201 | 6.92E-20 |
| NR4A3 | 2.5603805 | 2.244772376 | 9.572488062 | 6.93E-20 |
| TRAPPC3 | -0.257007894 | 5.692919926 | -9.569285646 | 7.11E-20 |
| RP11-840I19.5 | 1.324990618 | 0.749208454 | 9.564749013 | 7.37E-20 |
| EP300 | 0.380866477 | 7.095749131 | 9.564139622 | 7.41E-20 |
| ZBTB21 | 0.871528194 | 4.651706225 | 9.563894762 | 7.42E-20 |
| MAT2B | -0.615218561 | 5.82960487 | -9.553337653 | 8.08E-20 |
| DPY30 | -0.425392183 | 4.502408426 | -9.551918013 | 8.17E-20 |
| NSF | -0.610652003 | 4.840954164 | -9.551565305 | 8.19E-20 |
| C2orf48 | 1.337173522 | 1.869566195 | 9.548292755 | 8.41E-20 |
| TIGD7 | -0.987827094 | 2.583831244 | -9.547770051 | 8.45E-20 |
| GOLGA4 | 0.365199383 | 5.263446323 | 9.5460525 | 8.57E-20 |
| CTA-963H5.5 | -0.834812911 | 3.0871641 | -9.543556288 | 8.74E-20 |
| AHCTF1P1 | 0.513052578 | 4.216138375 | 9.540908785 | 8.93E-20 |
| CTB-25B13.12 | -0.701995346 | 3.933897522 | -9.539857492 | 9.00E-20 |
| TMEM150A | -0.590714284 | 3.818915632 | -9.537161134 | 9.20E-20 |
| PPP3CA | -0.410717203 | 6.372915604 | -9.535438965 | 9.33E-20 |
| CEP104 | -0.459675397 | 4.709062149 | -9.535267387 | 9.34E-20 |
| ZNF639 | 0.603923469 | 5.971687243 | 9.535246662 | 9.34E-20 |
| RPS6KC1 | -0.436442891 | 5.160186161 | -9.533684515 | 9.46E-20 |
| B3GNT5 | 0.948740251 | 5.140082678 | 9.533506493 | 9.47E-20 |
| GLE1 | -0.391159988 | 6.279226377 | -9.530173588 | 9.73E-20 |
| RFC1 | 0.32839754 | 5.579191722 | 9.528289253 | 9.88E-20 |
| RP4-800J21.3 | 1.342561442 | 0.477854613 | 9.528106314 | 9.89E-20 |
| ADIPOR1 | 0.547276243 | 7.839993439 | 9.524486631 | 1.02E-19 |
| CBY1 | -0.653154299 | 3.584133647 | -9.519634257 | 1.06E-19 |
| LYPLAL1 | -0.406477997 | 4.334719844 | -9.513913466 | 1.11E-19 |
| ECD | 0.361384113 | 5.571977894 | 9.512003523 | 1.13E-19 |
| RSF1-IT2 | 1.077587057 | 1.536684533 | 9.511004745 | 1.13E-19 |
| COQ9 | -0.471731581 | 4.795268343 | -9.510269584 | 1.14E-19 |
| RP11-297D21.4 | -0.754852903 | 2.245883871 | -9.505028314 | 1.19E-19 |
| SETDB1 | -0.352846769 | 5.768411202 | -9.502955891 | 1.21E-19 |
| ARF6 | 0.43351993 | 8.983509958 | 9.500229073 | 1.24E-19 |
| TUBD1 | -0.632934489 | 2.685039727 | -9.492744973 | 1.31E-19 |
| MPC2 | 0.523772669 | 6.574221494 | 9.488019409 | 1.36E-19 |
| C19orf44 | -0.899411432 | 1.92817283 | -9.487357578 | 1.37E-19 |
| RP11-96K19.2 | 1.469432647 | 1.297744301 | 9.486154703 | 1.38E-19 |
| SPRTN | 0.520874404 | 3.560698394 | 9.478163928 | 1.48E-19 |
| ARSD | -0.825622632 | 5.60364556 | -9.477030154 | 1.49E-19 |
| C1orf122 | 0.43645197 | 4.451974382 | 9.47702087 | 1.49E-19 |
| LIG4 | -0.589755485 | 4.357357236 | -9.47648371 | 1.50E-19 |
| C11orf73 | -0.484522145 | 4.397935212 | -9.473633128 | 1.53E-19 |
| SPIDR | -0.442321874 | 5.480728402 | -9.472844125 | 1.54E-19 |
| ZNF136 | 0.668736543 | 3.920273787 | 9.470230407 | 1.57E-19 |
| MPV17 | -0.371178388 | 5.399229416 | -9.465130279 | 1.64E-19 |
| AC013283.1 | -1.101183124 | 1.49129947 | -9.465051751 | 1.64E-19 |
| GDI2P2 | -0.47364996 | 4.299599151 | -9.464453673 | 1.65E-19 |
| POU2F1 | 0.461147725 | 4.468654129 | 9.463459468 | 1.66E-19 |
| ROCK2 | -0.45163147 | 5.050766202 | -9.462291946 | 1.67E-19 |
| COX10-DT | -0.618089539 | 2.125768417 | -9.460776216 | 1.70E-19 |
| SELPLG | -1.059228443 | 7.955444313 | -9.450174777 | 1.84E-19 |
| RABGEF1 | 1.016885836 | 1.605299333 | 9.440698635 | 1.99E-19 |
| MANBA | -0.457042671 | 6.056876819 | -9.439050243 | 2.02E-19 |
| NDRG3 | -0.494671148 | 5.904620448 | -9.438717731 | 2.02E-19 |
| PRMT2 | -0.437824091 | 6.123218205 | -9.438011406 | 2.03E-19 |
| RP1-64K7.4 | 0.700377721 | 3.476658682 | 9.437112501 | 2.05E-19 |
| ANAPC15 | 0.47642619 | 5.715364084 | 9.435997211 | 2.07E-19 |
| OSBP | 0.32207055 | 6.182042002 | 9.431111356 | 2.15E-19 |
| TMEM44-AS1 | -0.633839708 | 3.225121371 | -9.424182789 | 2.27E-19 |
| ZNF226 | -0.457428705 | 3.021052398 | -9.423493056 | 2.28E-19 |
| BCL10 | 0.590360966 | 5.041426001 | 9.422863709 | 2.29E-19 |
| AC098820.3 | -1.057268908 | 0.131300979 | -9.418117016 | 2.38E-19 |
| ZNF350 | 0.523939204 | 4.430868079 | 9.417345521 | 2.40E-19 |
| MTMR6 | 0.591810581 | 5.233377102 | 9.412672145 | 2.49E-19 |
| ZSWIM3 | -0.889586518 | 2.105546734 | -9.408270104 | 2.58E-19 |
| SLC30A5 | -0.392918877 | 4.988738176 | -9.406113636 | 2.62E-19 |
| DNMBP | 0.749126843 | 3.871151192 | 9.405432746 | 2.63E-19 |
| NRAS | 0.408307949 | 7.356707925 | 9.404776842 | 2.65E-19 |
| LZTR1 | -0.407954201 | 5.227204828 | -9.402746629 | 2.69E-19 |
| TRAPPC11 | -0.321804297 | 5.827086719 | -9.402548484 | 2.70E-19 |
| NUP54 | 0.380702565 | 5.552844769 | 9.402364108 | 2.70E-19 |
| ZNF821 | 0.984041903 | 2.680865215 | 9.40085646 | 2.73E-19 |
| SKIL | 0.972905214 | 4.456079161 | 9.399070453 | 2.77E-19 |
| IFT27 | -0.569814761 | 3.281244677 | -9.396469753 | 2.83E-19 |
| ZNF326 | 0.504901911 | 5.632075006 | 9.395044468 | 2.86E-19 |
| NCKAP1L | -0.437453065 | 7.96113332 | -9.394787465 | 2.87E-19 |
| BTG3 | 1.226214999 | 4.967132117 | 9.39331651 | 2.90E-19 |
| ARID3B | 0.764333217 | 4.56036437 | 9.393088842 | 2.91E-19 |
| PTPN1 | 0.441442318 | 7.224224833 | 9.392527318 | 2.92E-19 |
| SLC35E2B | -0.482561152 | 5.510735168 | -9.389892169 | 2.98E-19 |
| RP11-170N16.3 | -1.236040437 | -0.069863148 | -9.381645489 | 3.18E-19 |
| ACOT13 | -0.63548136 | 3.810743581 | -9.376915781 | 3.30E-19 |
| MUL1 | 0.408768617 | 5.663123721 | 9.376887307 | 3.30E-19 |
| FBXL3 | 0.527573887 | 6.042671931 | 9.372528404 | 3.42E-19 |
| RP11-27I1.4 | -0.887908736 | 1.805910695 | -9.371009034 | 3.46E-19 |
| RP11-274B21.10 | -0.956594638 | 2.301265964 | -9.370803294 | 3.47E-19 |
| SEC31A | -0.316781413 | 6.698120562 | -9.370567866 | 3.47E-19 |
| SMG1 | -0.343439765 | 7.095470569 | -9.365233596 | 3.62E-19 |
| KIDINS220 | -0.346575631 | 6.115268312 | -9.359676031 | 3.79E-19 |
| PARP2 | 0.43480529 | 4.758909293 | 9.35894227 | 3.81E-19 |
| YWHAB | -0.490390038 | 8.653670468 | -9.358493525 | 3.82E-19 |
| RP5-983L19.2 | 1.498658085 | -2.78625548 | 9.357417046 | 3.86E-19 |
| CSTF3 | -0.508464927 | 4.693468682 | -9.357310518 | 3.86E-19 |
| BIN3 | 0.475165847 | 4.729121213 | 9.356204932 | 3.89E-19 |
| LINC00847 | -0.637300126 | 4.640109337 | -9.355041959 | 3.93E-19 |
| DTD2 | -0.554848264 | 3.673059169 | -9.352798481 | 4.00E-19 |
| AC093627.10 | -1.182043784 | 2.949729103 | -9.351316441 | 4.05E-19 |
| RP11-383C5.4 | -0.946886097 | -0.220680216 | -9.351129764 | 4.05E-19 |
| RBM48 | 0.489896143 | 4.276267839 | 9.350050036 | 4.09E-19 |
| GVQW1 | -1.268736719 | -1.265550963 | -9.348555099 | 4.14E-19 |
| SIGLEC15 | -1.448383029 | 1.389542047 | -9.348526616 | 4.14E-19 |
| SUFU | -0.582319435 | 3.976958062 | -9.347400985 | 4.17E-19 |
| CEPT1 | -0.4009353 | 4.462887344 | -9.346015756 | 4.22E-19 |
| TMEM173 | -0.71170031 | 7.98536704 | -9.345787153 | 4.23E-19 |
| PSMG3-AS1 | -0.902908641 | 2.116546953 | -9.345028445 | 4.25E-19 |
| C4orf21 | 0.605951452 | 3.464489169 | 9.34359325 | 4.30E-19 |
| ZDHHC16 | -0.618502148 | 5.123812584 | -9.343262249 | 4.31E-19 |
| PTEN | -0.396565441 | 6.584141705 | -9.343143279 | 4.32E-19 |
| GCOM2 | 0.481586954 | 3.688616823 | 9.340373753 | 4.41E-19 |
| IPO8 | -0.392093178 | 5.312181705 | -9.337352063 | 4.52E-19 |
| ZNF518A | -0.433206513 | 5.005695586 | -9.336323895 | 4.56E-19 |
| CKAP2L | 1.233354963 | 3.093262967 | 9.334227982 | 4.63E-19 |
| TLE3 | 0.692250045 | 6.183320764 | 9.33419819 | 4.63E-19 |
| RP11-37E23.5 | -1.187541237 | 2.71410093 | -9.327640992 | 4.88E-19 |
| ACADM | -0.62324717 | 5.732551915 | -9.3250599 | 4.98E-19 |
| SENP5 | 0.397400017 | 5.11742531 | 9.324680256 | 5.00E-19 |
| UEVLD | -0.674074305 | 3.544144933 | -9.319874457 | 5.19E-19 |
| HSPA13 | 0.537093013 | 5.744576156 | 9.318344433 | 5.25E-19 |
| ZBTB17 | 0.390536909 | 5.757024428 | 9.316511927 | 5.33E-19 |
| ZDHHC6 | -0.335102761 | 6.409031375 | -9.314233607 | 5.43E-19 |
| BTN3A2 | -0.853822819 | 5.396865259 | -9.313300971 | 5.47E-19 |
| RP11-266J6.2 | -1.005029933 | 0.407514002 | -9.308202532 | 5.69E-19 |
| ZNF292 | 0.445806233 | 5.746680538 | 9.308112111 | 5.70E-19 |
| METTL25B | -0.451872661 | 4.619641231 | -9.307160359 | 5.74E-19 |
| NCOA4 | -0.536556896 | 9.574414899 | -9.30512263 | 5.83E-19 |
| ZNF628 | 0.491392337 | 4.480449704 | 9.302878799 | 5.94E-19 |
| SCAF8 | 0.328190232 | 6.57532466 | 9.302824852 | 5.94E-19 |
| KBTBD4 | 0.422069656 | 4.957693789 | 9.300239849 | 6.06E-19 |
| HIN1L | 0.451334284 | 3.870709871 | 9.2980719 | 6.17E-19 |
| FAM183B | 1.603630173 | 0.709106088 | 9.29302878 | 6.42E-19 |
| ELL | 0.64229105 | 4.877679346 | 9.289594217 | 6.59E-19 |
| WRN | 0.511854092 | 4.414550307 | 9.289421817 | 6.60E-19 |
| B4GALT1 | 0.628562662 | 7.128037323 | 9.285425489 | 6.81E-19 |
| AP006621.6 | -1.245950216 | 1.577810202 | -9.284933384 | 6.84E-19 |
| ZNF275 | 0.513659438 | 3.758515196 | 9.281102573 | 7.05E-19 |
| MIR940 | -0.99812689 | 1.231475237 | -9.275138973 | 7.39E-19 |
| BBS7 | -0.64834102 | 3.028864025 | -9.274086887 | 7.45E-19 |
| UBA1 | -0.386938076 | 7.877843996 | -9.273925649 | 7.46E-19 |
| NDUFB8 | 0.486806698 | 3.1866117 | 9.271933943 | 7.58E-19 |
| ATR | -0.371244618 | 4.2171925 | -9.27128457 | 7.62E-19 |
| NCBP2 | -0.347032222 | 6.029822244 | -9.268255029 | 7.80E-19 |
| HRSP12 | -0.77577636 | 3.594517047 | -9.258322524 | 8.43E-19 |
| ABT1 | 0.350108009 | 6.049016117 | 9.257711308 | 8.47E-19 |
| RP11-473D24.1 | 1.266844148 | -0.435157389 | 9.25592328 | 8.59E-19 |
| NR1H2 | 0.374521055 | 6.700705877 | 9.255313088 | 8.64E-19 |
| SYNRG | -0.469815791 | 4.7631713 | -9.249169134 | 9.06E-19 |
| ZNF785 | -0.655656268 | 3.917540178 | -9.249124354 | 9.07E-19 |
| CRYZ | -0.79797352 | 3.651589799 | -9.248786615 | 9.09E-19 |
| TMEM183A | 0.487728627 | 6.248880194 | 9.243887748 | 9.45E-19 |
| ITPRIP | 1.036010927 | 6.73767482 | 9.243723914 | 9.46E-19 |
| RP11-111M22.4 | -1.239406471 | 1.548861619 | -9.243026699 | 9.51E-19 |
| DYNLL1-AS1 | -0.864215425 | 2.491885955 | -9.241552867 | 9.62E-19 |
| TLK2P1 | 0.417103159 | 5.655298204 | 9.241073237 | 9.66E-19 |
| MZT1 | 0.516958617 | 5.989852361 | 9.240126313 | 9.73E-19 |
| AC005306.3 | -0.766614316 | 3.428441708 | -9.239384851 | 9.79E-19 |
| USP36 | 0.645390743 | 5.508325267 | 9.23897542 | 9.82E-19 |
| UGDH-AS1 | -0.843074518 | 1.063279006 | -9.236941535 | 9.98E-19 |
| TMA16 | -0.542873041 | 4.18970537 | -9.236181928 | 1.00E-18 |
| ARSI | 1.695695461 | -2.965898089 | 9.230993851 | 1.05E-18 |
| PDIK1L | 0.558504053 | 4.193756664 | 9.230213086 | 1.05E-18 |
| PAFAH1B2 | 0.437506932 | 5.819981194 | 9.229420727 | 1.06E-18 |
| TRGV7 | -1.396876151 | 2.335094537 | -9.228991282 | 1.06E-18 |
| L3HYPDH | -0.689736893 | 3.345691106 | -9.227632665 | 1.07E-18 |
| RP11-216B9.6 | -0.904469451 | 0.456573367 | -9.225505848 | 1.09E-18 |
| EIF4E | 0.424598234 | 2.936243849 | 9.225136812 | 1.09E-18 |
| TMEM119 | 1.410178434 | 0.499962399 | 9.221211399 | 1.13E-18 |
| RP5-882C2.2 | 0.769446305 | 3.8061902 | 9.219746007 | 1.14E-18 |
| RGS14 | -0.697090691 | 6.327925702 | -9.219328196 | 1.15E-18 |
| UPB1 | 1.474715864 | 0.615749232 | 9.219046133 | 1.15E-18 |
| MTCH1 | 0.457269344 | 7.379708217 | 9.214595657 | 1.19E-18 |
| MON1A | -0.732464186 | 2.60525976 | -9.212339775 | 1.21E-18 |
| TAOK1 | -0.378487789 | 6.029292994 | -9.211248651 | 1.22E-18 |
| GFPT1 | 0.378524672 | 4.469862384 | 9.2104294 | 1.23E-18 |
| MLYCD | -0.53928978 | 2.442863097 | -9.209752908 | 1.24E-18 |
| NAF1 | 0.377716098 | 3.951684813 | 9.208435666 | 1.25E-18 |
| EPT1 | 0.439347769 | 4.565218601 | 9.199822169 | 1.34E-18 |
| CRYM-AS1 | -1.174257935 | -0.353664077 | -9.199358192 | 1.34E-18 |
| TMEM87B | -0.392443633 | 5.360169988 | -9.198310381 | 1.35E-18 |
| C14orf142 | -0.597288707 | 4.782518485 | -9.196770722 | 1.37E-18 |
| CCIN | 1.413193818 | 0.587526088 | 9.196738066 | 1.37E-18 |
| PMVK | -0.57919372 | 6.086320063 | -9.194679629 | 1.39E-18 |
| RP4-769N13.6 | -0.66406747 | 2.300152466 | -9.194558985 | 1.39E-18 |
| MMD | 0.745371842 | 4.133386515 | 9.192929659 | 1.41E-18 |
| HNRNPL | 0.388607211 | 6.791073912 | 9.191736372 | 1.42E-18 |
| CYCSP34 | -0.919627742 | 2.087767987 | -9.189050511 | 1.45E-18 |
| ATF4P4 | 0.746474991 | 2.828980969 | 9.18846416 | 1.46E-18 |
| RP5-867C24.1 | 1.080212926 | 1.546603381 | 9.188194375 | 1.46E-18 |
| TMEM131 | -0.332338699 | 5.698394016 | -9.185734838 | 1.49E-18 |
| ZNF763 | -0.68074382 | 0.901051168 | -9.185317496 | 1.50E-18 |
| N4BP2L1 | -0.514719406 | 4.932997879 | -9.182963854 | 1.52E-18 |
| TMEM41A | 0.502243991 | 3.410395558 | 9.175318165 | 1.62E-18 |
| SHCBP1 | 1.015626566 | 3.555719529 | 9.175279527 | 1.62E-18 |
| FAM133B | 0.491748036 | 3.660714309 | 9.173396856 | 1.64E-18 |
| NDC80 | 0.849164501 | 3.538636562 | 9.173147487 | 1.65E-18 |
| PPTC7 | 0.562531424 | 6.14060101 | 9.172708459 | 1.65E-18 |
| DHX16 | -0.306209816 | 5.635764054 | -9.169936434 | 1.69E-18 |
| SKA1 | 1.121937957 | 3.141923454 | 9.16864979 | 1.70E-18 |
| PAPOLA | -0.274477774 | 7.084698267 | -9.166962052 | 1.73E-18 |
| SPAG5 | 0.797511219 | 4.612354768 | 9.166084166 | 1.74E-18 |
| TMEM187 | -0.762442904 | 2.860759074 | -9.165741792 | 1.74E-18 |
| ADRA2B | 1.637218648 | -1.718772983 | 9.165658512 | 1.74E-18 |
| RP3-329A5.8 | -1.132526134 | -0.438415499 | -9.164309368 | 1.76E-18 |
| CCDC117 | 0.349558058 | 5.730848891 | 9.162734766 | 1.78E-18 |
| DHX35 | 0.328265792 | 3.990957274 | 9.161831467 | 1.80E-18 |
| ZNF805 | 0.81131809 | 4.117376427 | 9.161190894 | 1.81E-18 |
| ST6GAL1 | -0.709049998 | 5.295059244 | -9.161036953 | 1.81E-18 |
| CHEK1 | 0.758604248 | 3.911291061 | 9.160848737 | 1.81E-18 |
| ZNF623 | -0.490790361 | 4.426076314 | -9.160057535 | 1.82E-18 |
| ORC6 | 0.736853836 | 3.623096743 | 9.1596804 | 1.83E-18 |
| ARPP19 | 0.324216078 | 6.67342677 | 9.159156101 | 1.84E-18 |
| WASL | 0.540131041 | 3.798090261 | 9.153486891 | 1.92E-18 |
| APBB1IP | -0.586372594 | 7.317446259 | -9.149467799 | 1.98E-18 |
| LINC01134 | -1.185834866 | -0.436906009 | -9.146904226 | 2.02E-18 |
| RP11-283G6.4 | -1.166182424 | 0.885627407 | -9.146413032 | 2.03E-18 |
| RP11-16N11.2 | -1.106463303 | 0.539963734 | -9.144607942 | 2.06E-18 |
| RP11-692C23.1 | -0.90458438 | 1.72679007 | -9.140290862 | 2.13E-18 |
| MCM9 | 0.346423612 | 4.082353172 | 9.139669428 | 2.14E-18 |
| PSMD6 | 0.34638669 | 5.508731983 | 9.129981128 | 2.30E-18 |
| SDCCAG3 | -0.402506679 | 5.58984279 | -9.128059415 | 2.34E-18 |
| TTC14 | -0.469085634 | 5.6629255 | -9.127131644 | 2.36E-18 |
| SDAD1 | -0.501501588 | 5.349884482 | -9.125487198 | 2.39E-18 |
| DUSP3 | -0.616399726 | 6.433165137 | -9.125464719 | 2.39E-18 |
| MRPL24 | -0.664905726 | 5.929058046 | -9.123300465 | 2.43E-18 |
| RNU6-32P | 0.978526217 | 1.2772395 | 9.12134148 | 2.47E-18 |
| AC018755.1 | -1.194460106 | -0.454735228 | -9.114929406 | 2.59E-18 |
| CCNT1 | 0.57492656 | 5.696883111 | 9.113703905 | 2.62E-18 |
| MIR644A | 1.032552043 | 1.632809702 | 9.113539563 | 2.62E-18 |
| TRNT1 | -0.434319327 | 3.98712708 | -9.113005577 | 2.63E-18 |
| C17orf80 | -0.650740544 | 4.274032503 | -9.112006389 | 2.65E-18 |
| RP11-299H21.1 | -1.032692591 | 0.082555096 | -9.109659883 | 2.70E-18 |
| CCM2 | -0.490676946 | 4.916776174 | -9.109259182 | 2.71E-18 |
| PDK1 | 0.681691782 | 4.368755088 | 9.107702146 | 2.74E-18 |
| LOH12CR1 | 0.474383873 | 4.002878024 | 9.107362458 | 2.75E-18 |
| VPS33A | -0.482207677 | 2.815009113 | -9.107228388 | 2.75E-18 |
| BCL2L13 | -0.399859467 | 5.582166813 | -9.107034935 | 2.76E-18 |
| IBTK | -0.343851241 | 5.306647374 | -9.104458127 | 2.81E-18 |
| CDR2 | 0.637155574 | 4.325046534 | 9.102264298 | 2.86E-18 |
| NAPSA | -1.087935027 | -0.102621621 | -9.09937983 | 2.93E-18 |
| SLC16A14 | 1.468792452 | 0.328484294 | 9.099232055 | 2.93E-18 |
| NRIP3 | 1.362148136 | 3.616771715 | 9.097841226 | 2.96E-18 |
| CSF1 | 1.597301095 | 3.77837229 | 9.096696111 | 2.99E-18 |
| AHCYL1 | -0.456411714 | 6.684977644 | -9.094946141 | 3.03E-18 |
| SAP30L | -0.503098972 | 4.433508669 | -9.09323826 | 3.07E-18 |
| SMOX | 1.200731788 | 4.183437248 | 9.092707051 | 3.08E-18 |
| BCS1L | -0.569404559 | 4.715754014 | -9.092434412 | 3.09E-18 |
| TMEM102 | -0.739767154 | 4.566082356 | -9.091685048 | 3.11E-18 |
| OSGEPL1 | -0.57205144 | 2.513402772 | -9.089368097 | 3.16E-18 |
| FAM63A | -0.667669502 | 5.577085851 | -9.089285705 | 3.16E-18 |
| FUT3 | 1.409255274 | -2.530383172 | 9.088204357 | 3.19E-18 |
| RP11-458D21.6 | -1.223357222 | -0.423343848 | -9.087781487 | 3.20E-18 |
| DDX58 | -0.682012523 | 4.358489049 | -9.086638286 | 3.23E-18 |
| PRKAB1 | 0.438213033 | 4.892471842 | 9.084686454 | 3.28E-18 |
| FBXO3 | 0.498436062 | 3.928322883 | 9.083948929 | 3.30E-18 |
| PDHB | -0.43608758 | 5.221849506 | -9.082995646 | 3.32E-18 |
| RP5-1112D6.8 | -0.975331507 | -0.256628724 | -9.082876216 | 3.33E-18 |
| CTD-2083E4.4 | 0.95597123 | 0.478871514 | 9.082640391 | 3.33E-18 |
| SIAH1 | 0.807601494 | 4.189876621 | 9.080017761 | 3.40E-18 |
| THOC2 | -0.335928804 | 5.909889905 | -9.079680267 | 3.41E-18 |
| LINC00094 | -0.669559004 | 2.743289248 | -9.078841778 | 3.43E-18 |
| TACC1 | 0.463559702 | 6.47257233 | 9.075319938 | 3.53E-18 |
| RBM34 | 0.929072869 | 2.357226359 | 9.075225914 | 3.53E-18 |
| EPS15 | -0.301221255 | 6.583114014 | -9.074154435 | 3.56E-18 |
| RP5-837I24.1 | 0.757287738 | 3.109751808 | 9.067615702 | 3.74E-18 |
| INO80D | 0.435290891 | 5.363922311 | 9.065449892 | 3.81E-18 |
| CTC-325H20.2 | -0.94546991 | 0.641709616 | -9.065075056 | 3.82E-18 |
| HSPA8 | -0.681625661 | 9.873076706 | -9.058547399 | 4.02E-18 |
| DDA1 | 0.286555147 | 5.038834442 | 9.058226035 | 4.03E-18 |
| TMEM55B | 0.443330327 | 6.028498689 | 9.057025203 | 4.06E-18 |
| RP11-529H20.6 | -0.755319702 | 2.557461472 | -9.056761145 | 4.07E-18 |
| KCTD21 | -0.886498522 | 2.533579538 | -9.054274238 | 4.15E-18 |
| ESPL1 | 1.109978658 | 2.74165775 | 9.053095016 | 4.19E-18 |
| ADORA2A | 1.288868378 | 0.481053263 | 9.05018183 | 4.29E-18 |
| RP5-1057J7.6 | -1.45083327 | 0.195046187 | -9.047604959 | 4.37E-18 |
| ARID5A | 0.796752586 | 6.451235566 | 9.046008529 | 4.43E-18 |
| RIPK2 | 0.840702296 | 6.66687307 | 9.044360725 | 4.48E-18 |
| VAMP8 | -0.672668758 | 9.108203425 | -9.043664728 | 4.51E-18 |
| BCDIN3D-AS1 | -1.015695764 | -1.523019915 | -9.041822872 | 4.57E-18 |
| PSKH1 | -0.449684792 | 4.789579896 | -9.038003333 | 4.71E-18 |
| LRRC27 | -0.831516415 | 1.785654285 | -9.036872439 | 4.75E-18 |
| ADAM1A | -0.784433421 | 3.374875215 | -9.035778439 | 4.79E-18 |
| PYGO2 | -0.516239608 | 5.160366049 | -9.03131718 | 4.96E-18 |
| CDCA5 | 1.094651761 | 4.548613902 | 9.030626115 | 4.99E-18 |
| SUPT20H | -0.427790357 | 5.303302688 | -9.027892483 | 5.09E-18 |
| RP11-227D13.2 | -0.884451931 | 0.842048236 | -9.027015356 | 5.13E-18 |
| SRSF10 | 0.738724544 | 1.856966828 | 9.025223523 | 5.20E-18 |
| MFSD1 | -0.589458275 | 6.831942461 | -9.025013546 | 5.21E-18 |
| VPS72 | -0.564189978 | 4.952260728 | -9.024295203 | 5.24E-18 |
| C18orf25 | 0.505355318 | 5.674436554 | 9.024263931 | 5.24E-18 |
| AC016716.1 | 0.604999137 | 2.103603375 | 9.023589346 | 5.27E-18 |
| AC006042.8 | 1.37321815 | 2.541247459 | 9.019664805 | 5.43E-18 |
| RP11-4O1.2 | -0.721958212 | 3.67304634 | -9.019448119 | 5.44E-18 |
| RP3-368A4.5 | -0.746734727 | 4.55506058 | -9.016717352 | 5.55E-18 |
| GTPBP4 | 0.42815723 | 5.401832359 | 9.016319767 | 5.57E-18 |
| NFE2 | -1.069303747 | 7.91360035 | -9.015835453 | 5.59E-18 |
| SLC27A1 | -0.720653723 | 3.621979627 | -9.015627094 | 5.60E-18 |
| HSDL2 | -0.540680544 | 5.666071409 | -9.014338635 | 5.66E-18 |
| RP11-778D9.12 | -1.113873551 | -0.449879511 | -9.011256124 | 5.79E-18 |
| DCAF7 | -0.778846855 | 6.453227421 | -9.008842299 | 5.90E-18 |
| E2F1 | 0.890664623 | 5.353577651 | 9.007075926 | 5.98E-18 |
| HMGA1P1 | 0.717274679 | 2.736058226 | 9.005883765 | 6.04E-18 |
| AC074286.1 | -0.881581179 | 0.973558439 | -9.004418125 | 6.11E-18 |
| CHAF1A | 0.521600175 | 5.582562707 | 9.003034972 | 6.17E-18 |
| DHRS3 | 1.523765577 | 3.22071099 | 9.001428713 | 6.25E-18 |
| CDK11A | 0.752115048 | 3.034282282 | 9.000717534 | 6.28E-18 |
| PSD4 | -0.445421648 | 5.981446233 | -9.000676607 | 6.29E-18 |
| GNE | 0.388375979 | 5.107506179 | 9.000382712 | 6.30E-18 |
| RBM17 | 0.260576369 | 6.593182538 | 8.999837173 | 6.33E-18 |
| ZNF408 | 0.494786595 | 4.438545501 | 8.998428917 | 6.40E-18 |
| RNMT | 0.465422919 | 5.230046382 | 8.99791925 | 6.42E-18 |
| DAXX | -0.430264369 | 6.196363237 | -8.996998425 | 6.47E-18 |
| RPP25L | -0.639625387 | 4.741928242 | -8.991402287 | 6.75E-18 |
| ACTR2 | -0.491161509 | 9.569776087 | -8.990920571 | 6.78E-18 |
| TRIM11 | 0.442910741 | 4.612306759 | 8.989247149 | 6.87E-18 |
| PMF1 | 0.413174756 | 5.061080255 | 8.986759402 | 7.00E-18 |
| RP11-555M1.3 | -0.976164315 | 0.087891047 | -8.98615982 | 7.03E-18 |
| PRG2 | 1.461599006 | 1.330048662 | 8.983518263 | 7.18E-18 |
| CORO7 | 0.532788236 | 3.962597478 | 8.982565567 | 7.23E-18 |
| PKMYT1 | 1.171120053 | 3.393638974 | 8.982117496 | 7.25E-18 |
| WSB1 | -0.503555538 | 7.029811462 | -8.981701422 | 7.28E-18 |
| SMYD4 | -0.458557104 | 3.596767789 | -8.98150514 | 7.29E-18 |
| CDIPT | -0.301474445 | 6.10213692 | -8.980663014 | 7.34E-18 |
| GTPBP10 | -0.541627934 | 3.206888958 | -8.980224155 | 7.36E-18 |
| HADHB | -0.303241998 | 6.794519694 | -8.979803399 | 7.39E-18 |
| TMEM223 | -0.605602395 | 3.654151512 | -8.978832737 | 7.44E-18 |
| HOOK2 | -0.466087187 | 3.713589425 | -8.977249694 | 7.53E-18 |
| RIOK3 | 0.655381237 | 6.969496744 | 8.976313137 | 7.59E-18 |
| CTD-2044J15.2 | -1.294227745 | -0.001491963 | -8.975102859 | 7.66E-18 |
| EAF1 | 0.727723509 | 6.255995727 | 8.973038541 | 7.78E-18 |
| PHF12 | 0.414447616 | 6.030494785 | 8.972851208 | 7.79E-18 |
| RP4-621B10.8 | -1.140709233 | -1.120619973 | -8.968056908 | 8.08E-18 |
| DNAJB14 | 0.345733032 | 4.869235878 | 8.962085311 | 8.47E-18 |
| PGM2 | -0.467963407 | 5.880208902 | -8.960794929 | 8.55E-18 |
| PPP1R2P3 | 0.654901693 | 4.211967762 | 8.960523738 | 8.57E-18 |
| MRS2 | -0.473720852 | 4.708244821 | -8.959589172 | 8.63E-18 |
| KMO | -1.269741916 | 0.406920013 | -8.959478945 | 8.64E-18 |
| NAA16 | 0.39009696 | 5.239199239 | 8.95931776 | 8.65E-18 |
| NUFIP1P | 0.493504783 | 2.55615959 | 8.959177394 | 8.66E-18 |
| MRM1 | -0.950751923 | 3.910858283 | -8.95905637 | 8.67E-18 |
| XYLT2 | -0.517594155 | 3.996494969 | -8.958965848 | 8.67E-18 |
| CCL2 | 2.440729305 | 0.894317014 | 8.958393257 | 8.71E-18 |
| GAS5-AS1 | -0.705181174 | 1.54817055 | -8.956369248 | 8.85E-18 |
| CES2 | -0.40515633 | 5.04795272 | -8.951207244 | 9.20E-18 |
| SLBP | 0.391479266 | 7.477165129 | 8.951089497 | 9.21E-18 |
| ZRANB1 | 0.375107881 | 5.821796481 | 8.950708912 | 9.24E-18 |
| B3GALT6 | -0.601409395 | 5.019009073 | -8.94964695 | 9.32E-18 |
| IKZF5 | 0.548941658 | 5.797740814 | 8.944709127 | 9.68E-18 |
| TRIM69 | -0.813546226 | 3.055136171 | -8.944560489 | 9.69E-18 |
| ETV3 | 0.625014079 | 4.947261365 | 8.942626718 | 9.83E-18 |
| EFCAB14 | -0.37631123 | 6.496297077 | -8.937113475 | 1.03E-17 |
| FAM114A2 | -0.53390028 | 3.86098334 | -8.931714512 | 1.07E-17 |
| CCDC90B | -0.329237546 | 4.298519987 | -8.930992252 | 1.08E-17 |
| UTP15 | 0.46865067 | 3.992786294 | 8.930868277 | 1.08E-17 |
| ZNF777 | 0.406713736 | 4.988170821 | 8.930561184 | 1.08E-17 |
| DGCR14 | 0.389674012 | 4.133993649 | 8.926643319 | 1.11E-17 |
| VPRBP | 0.317943417 | 5.327844538 | 8.922782657 | 1.15E-17 |
| CENPBD1P | -0.817259503 | 3.801678485 | -8.922403186 | 1.15E-17 |
| SNX9 | 1.247909576 | 4.52996264 | 8.921931734 | 1.15E-17 |
| FBXO7 | 0.41612935 | 6.774982039 | 8.920053874 | 1.17E-17 |
| PIK3C3 | -0.320132703 | 4.641260062 | -8.919367818 | 1.18E-17 |
| AL022341.3 | -1.008612632 | 1.302606075 | -8.91803795 | 1.19E-17 |
| PIGN | -0.552554401 | 4.129090112 | -8.917066872 | 1.20E-17 |
| SNTA1 | 0.937485269 | 2.112518044 | 8.914671655 | 1.22E-17 |
| NDUFA8 | -0.65625304 | 5.550351308 | -8.914179557 | 1.22E-17 |
| RP11-340F14.5 | -1.186448433 | 3.829307104 | -8.914128468 | 1.22E-17 |
| TM9SF4 | -0.360205114 | 6.372579503 | -8.913309619 | 1.23E-17 |
| DNAL4 | -0.460876653 | 4.055080735 | -8.912043624 | 1.24E-17 |
| RP3-467N11.2 | 0.575969403 | 2.209985023 | 8.91191254 | 1.24E-17 |
| CTD-2245F17.3 | -0.95056743 | 3.354703623 | -8.911371427 | 1.25E-17 |
| LRRC69 | -0.95595941 | 0.440807629 | -8.911150586 | 1.25E-17 |
| SLC7A5 | 1.021650267 | 6.643585821 | 8.910835703 | 1.26E-17 |
| RP5-1112D6.7 | -1.038765988 | -0.046894996 | -8.909441641 | 1.27E-17 |
| RP11-569A11.1 | -0.956294975 | -0.27228494 | -8.90849096 | 1.28E-17 |
| RGPD5 | 0.896935742 | 0.523170527 | 8.907854166 | 1.28E-17 |
| UQCRFS1 | 0.309392786 | 7.870463128 | 8.907830551 | 1.28E-17 |
| FAM115C | 1.102328082 | 0.983536171 | 8.905600029 | 1.31E-17 |
| TRIM39 | 0.46279868 | 4.223912304 | 8.904251497 | 1.32E-17 |
| M6PR | -0.437607015 | 7.619547962 | -8.89853446 | 1.38E-17 |
| ZBTB11-AS1 | -0.454783958 | 2.92870799 | -8.894182882 | 1.43E-17 |
| HMCES | 0.424668845 | 5.832516965 | 8.893608633 | 1.43E-17 |
| FBXW4P1 | 0.989604674 | 3.414590789 | 8.89157893 | 1.45E-17 |
| TADA1 | 0.453774945 | 4.794685237 | 8.889179318 | 1.48E-17 |
| ZUFSP | 0.383528891 | 5.017256786 | 8.88039241 | 1.58E-17 |
| CDC16 | -0.400823023 | 6.164629345 | -8.879828809 | 1.59E-17 |
| AC007292.3 | 0.847968034 | 0.04682164 | 8.878428119 | 1.61E-17 |
| SUPT5H | 0.234297958 | 6.574974825 | 8.876684523 | 1.63E-17 |
| FAM149B1 | -0.417946655 | 3.707161088 | -8.876257275 | 1.64E-17 |
| RP11-612B6.2 | -0.820015535 | 4.090166948 | -8.875240667 | 1.65E-17 |
| COPE | 0.213011252 | 7.046049562 | 8.871635286 | 1.69E-17 |
| LINC00205 | -0.685364092 | 3.496280265 | -8.871453226 | 1.70E-17 |
| ARMC10 | -0.464856212 | 4.32620186 | -8.868074337 | 1.74E-17 |
| GOLPH3L | -0.639692321 | 4.895136388 | -8.86324832 | 1.81E-17 |
| CEP63 | -0.404832062 | 4.725182052 | -8.860906812 | 1.84E-17 |
| PLRG1 | -0.350115526 | 5.02566588 | -8.859933931 | 1.85E-17 |
| E2F7 | 1.223862193 | 1.608458926 | 8.859370538 | 1.86E-17 |
| HEMK1 | -0.513031175 | 4.088309435 | -8.858461302 | 1.87E-17 |
| AFMID | -0.640217517 | 3.859330909 | -8.857434005 | 1.89E-17 |
| RP11-122G18.5 | -0.627011237 | 3.403346005 | -8.855385868 | 1.92E-17 |
| ARL6IP5 | -0.603855548 | 8.330575455 | -8.855354743 | 1.92E-17 |
| COG4 | -0.546413646 | 5.2584915 | -8.853198158 | 1.95E-17 |
| ARMC10P1 | -0.658987011 | 3.791448541 | -8.852478225 | 1.96E-17 |
| SPN | -0.925602677 | 8.526526458 | -8.850902494 | 1.99E-17 |
| NAA60 | 0.647338409 | 2.468364205 | 8.85087818 | 1.99E-17 |
| RETSAT | -0.449781988 | 4.992303951 | -8.849360245 | 2.01E-17 |
| RP1-39G22.7 | -0.65941902 | 3.913561289 | -8.848630382 | 2.02E-17 |
| RP11-347C12.10 | -0.929420747 | 2.322816065 | -8.84854132 | 2.02E-17 |
| ZNF284 | -0.78303217 | 0.844896066 | -8.84770208 | 2.03E-17 |
| RP11-477N3.1 | -0.931299436 | 1.418990514 | -8.844853753 | 2.08E-17 |
| N6AMT2 | -0.807298329 | 3.270583371 | -8.844387775 | 2.09E-17 |
| CORO1B | -0.6263931 | 6.665770767 | -8.844304179 | 2.09E-17 |
| POP5 | -0.500518803 | 4.946070622 | -8.844176529 | 2.09E-17 |
| LINC01011 | -0.982272483 | 1.735869934 | -8.844068744 | 2.09E-17 |
| CWC25 | 0.552311973 | 6.135608839 | 8.84356268 | 2.10E-17 |
| BBS2 | -0.467316443 | 4.040619872 | -8.842123203 | 2.12E-17 |
| POLI | -0.416640483 | 3.801180397 | -8.839860024 | 2.16E-17 |
| ZCCHC8 | 0.435756344 | 4.85825106 | 8.836638877 | 2.21E-17 |
| PIK3R1 | 0.607923762 | 7.059885588 | 8.835919081 | 2.23E-17 |
| BET1 | -0.575167293 | 3.128754173 | -8.833038073 | 2.28E-17 |
| FBXO18 | -0.34897615 | 5.7429472 | -8.831420247 | 2.30E-17 |
| TP53 | -0.774670478 | 6.130753136 | -8.830062148 | 2.33E-17 |
| ZNF573 | -0.768028912 | 1.514820116 | -8.82969613 | 2.33E-17 |
| RP11-686D22.5 | -1.205677888 | -0.441719852 | -8.825512416 | 2.41E-17 |
| NAA30 | 0.44823905 | 4.353202512 | 8.825473176 | 2.41E-17 |
| MEN1 | -0.48548527 | 5.455766331 | -8.824978381 | 2.42E-17 |
| CRABP2 | 0.997159372 | 1.466358927 | 8.819409309 | 2.52E-17 |
| ARRB1 | -0.501671261 | 6.390734717 | -8.819100415 | 2.53E-17 |
| MCPH1 | 0.322433452 | 5.104652561 | 8.818925324 | 2.53E-17 |
| ZNF432 | 0.577882241 | 3.435244954 | 8.817028256 | 2.57E-17 |
| GVINP1 | -1.134728539 | 3.605387975 | -8.815275958 | 2.61E-17 |
| CTD-2517M22.17 | -0.952835084 | 0.937333614 | -8.813733042 | 2.64E-17 |
| LIMD1-AS1 | -0.97052309 | 0.119941475 | -8.810548379 | 2.70E-17 |
| C21orf59 | -0.528313807 | 3.332376319 | -8.807806989 | 2.76E-17 |
| INTS12 | 0.300869753 | 4.872581734 | 8.800934783 | 2.91E-17 |
| KLHL28 | 0.458858953 | 4.17577984 | 8.800503606 | 2.92E-17 |
| MUS81 | -0.382433462 | 4.961550728 | -8.798800914 | 2.95E-17 |
| C9orf139 | -1.226355735 | 3.83963417 | -8.798225174 | 2.97E-17 |
| RPUSD2 | 0.51172588 | 4.353848282 | 8.796712456 | 3.00E-17 |
| DNAJC13 | -0.527100155 | 5.971892122 | -8.790465979 | 3.15E-17 |
| LEMD3 | 0.369846522 | 5.826986958 | 8.790180627 | 3.15E-17 |
| CBR4 | -0.42469689 | 4.222830102 | -8.789573024 | 3.17E-17 |
| ZBTB5 | 0.598392894 | 5.212325135 | 8.788016523 | 3.21E-17 |
| RP11-680H20.1 | -0.473192114 | 9.0398726 | -8.786891205 | 3.23E-17 |
| ZNF514 | -0.637755572 | 3.80075756 | -8.785924669 | 3.26E-17 |
| ICAM1 | 1.265050055 | 5.929089754 | 8.785884266 | 3.26E-17 |
| ZNF607 | -0.887183764 | 1.906386462 | -8.785467577 | 3.27E-17 |
| SLC52A2 | -0.447276259 | 5.536892012 | -8.778403154 | 3.45E-17 |
| VPS35 | -0.429770843 | 5.847802297 | -8.776382632 | 3.50E-17 |
| HIPK3 | -0.423207036 | 7.092853789 | -8.772504911 | 3.61E-17 |
| REM2 | 1.305023795 | 2.324124361 | 8.772226818 | 3.61E-17 |
| RP1-281H8.3 | 0.509488143 | 3.802681042 | 8.77165431 | 3.63E-17 |
| C19orf47 | 0.2831992 | 3.671501686 | 8.770847 | 3.65E-17 |
| RFT1 | -0.391728405 | 4.218779561 | -8.770815338 | 3.65E-17 |
| ABHD16A | 0.490095955 | 3.140203231 | 8.770619957 | 3.66E-17 |
| ASTL | 1.43114259 | 0.169338873 | 8.770247987 | 3.67E-17 |
| RHOT1P1 | -0.486855247 | 4.075434691 | -8.767251605 | 3.75E-17 |
| RP11-231G3.1 | -0.884182738 | 1.572601245 | -8.765283841 | 3.81E-17 |
| RASD2 | 1.707924309 | -1.672260147 | 8.762894847 | 3.88E-17 |
| RASSF7 | -0.558349327 | 4.896201301 | -8.762532764 | 3.89E-17 |
| CDK19 | -0.393695671 | 5.37491495 | -8.760265204 | 3.96E-17 |
| SFSWAP | 0.244061815 | 5.371539156 | 8.757957285 | 4.03E-17 |
| ZNF786 | -0.836112883 | 2.801998329 | -8.755409081 | 4.11E-17 |
| AL356585.2 | 0.383555404 | 5.015154757 | 8.753992661 | 4.15E-17 |
| BUD31 | 0.376764956 | 6.786434967 | 8.753269544 | 4.17E-17 |
| FEM1C | 0.815584784 | 5.784370841 | 8.750702446 | 4.26E-17 |
| CCNE1 | 0.840312588 | 4.238697118 | 8.749749308 | 4.29E-17 |
| RNF11 | 0.563947171 | 7.186127127 | 8.748112085 | 4.34E-17 |
| RP11-400F19.6 | -0.543051205 | 4.559581891 | -8.747949179 | 4.34E-17 |
| C6orf57 | -0.482769104 | 3.690831117 | -8.745652379 | 4.42E-17 |
| ANTXR2 | -0.536555675 | 6.048095182 | -8.745424848 | 4.43E-17 |
| MAT2A | -0.646117453 | 8.391403052 | -8.744006898 | 4.48E-17 |
| ZNF189 | -0.576279813 | 4.531509754 | -8.739117773 | 4.65E-17 |
| SLC7A11 | 1.430006066 | 0.833373137 | 8.738104257 | 4.68E-17 |
| SNORD117 | 0.784378785 | 3.758357397 | 8.737197987 | 4.71E-17 |
| KIF18A | 0.748351381 | 2.816370863 | 8.734646905 | 4.81E-17 |
| SLC46A3 | -0.656344144 | 4.410535335 | -8.732672292 | 4.88E-17 |
| MTFR1L | -0.274902903 | 4.956835727 | -8.732490051 | 4.88E-17 |
| LMTK2 | 0.754162006 | 4.46393379 | 8.731286431 | 4.93E-17 |
| DONSON | 0.432568759 | 4.129040791 | 8.730296893 | 4.97E-17 |
| LZIC | -0.303793764 | 5.522184471 | -8.727922734 | 5.06E-17 |
| SRP14-DT | -0.758550159 | 2.638809494 | -8.726718629 | 5.10E-17 |
| CTC-303L1.1 | -0.899979857 | -0.309872787 | -8.725865576 | 5.14E-17 |
| DUSP18 | -0.561375784 | 3.720689175 | -8.723349103 | 5.23E-17 |
| AP003774.4 | -1.261524486 | 1.888054702 | -8.721934022 | 5.29E-17 |
| EZH2 | 0.533558233 | 5.604359782 | 8.719012074 | 5.41E-17 |
| JARID2 | 0.533383025 | 6.980159621 | 8.713928588 | 5.62E-17 |
| SART1 | 0.235074186 | 6.939815465 | 8.713559975 | 5.64E-17 |
| FAM219B | -0.485456059 | 5.883710825 | -8.712472638 | 5.68E-17 |
| NLN | -0.776498343 | 3.644768216 | -8.711237898 | 5.74E-17 |
| TOR1AIP2 | 0.457267844 | 4.903892043 | 8.711180006 | 5.74E-17 |
| ZNF699 | 0.670947946 | 2.714578882 | 8.709855365 | 5.80E-17 |
| ADCK4 | -0.328953702 | 4.398837599 | -8.703023138 | 6.10E-17 |
| FTH1P16 | 0.751998055 | 3.265255169 | 8.702359489 | 6.13E-17 |
| FAM91A1 | 0.480031677 | 5.912964476 | 8.700522838 | 6.22E-17 |
| WBP11P1 | 0.69775269 | 1.134655546 | 8.700397874 | 6.22E-17 |
| RP11-15A1.3 | -1.261823577 | 0.276612203 | -8.698145911 | 6.33E-17 |
| CSF1R | -1.473707209 | 6.021455847 | -8.695227656 | 6.47E-17 |
| CBFA2T2 | -0.286441686 | 4.406068895 | -8.693129685 | 6.58E-17 |
| MYPOP | -0.441259188 | 4.28387365 | -8.692399688 | 6.61E-17 |
| C1orf116 | 1.358015097 | -1.546295364 | 8.692032908 | 6.63E-17 |
| RP1-249I4.2 | -0.978307565 | -0.165816466 | -8.691929537 | 6.64E-17 |
| ACTL10 | -1.128361336 | 0.426028919 | -8.69180654 | 6.64E-17 |
| RAD9B | -0.727567173 | 1.504043289 | -8.691673772 | 6.65E-17 |
| PEX6 | -0.568417616 | 5.416850174 | -8.688925252 | 6.79E-17 |
| RBM7 | 0.510531594 | 4.28074185 | 8.688156086 | 6.83E-17 |
| CTB-55O6.12 | -0.530394221 | 3.628975078 | -8.685919026 | 6.94E-17 |
| RGPD8 | 0.761582756 | 2.207320225 | 8.683055044 | 7.09E-17 |
| WBP1LP2 | -1.188440508 | -0.278530054 | -8.682896446 | 7.10E-17 |
| RXRB | -0.313036939 | 5.924910964 | -8.681841562 | 7.16E-17 |
| TEX14 | 1.307191696 | -0.360646295 | 8.680552892 | 7.23E-17 |
| ZNF791 | 0.508747782 | 4.713640832 | 8.680311901 | 7.24E-17 |
| KATNBL1P6 | -0.560855231 | 2.865525966 | -8.679108579 | 7.31E-17 |
| SHB | 0.988522921 | 2.297616351 | 8.677960371 | 7.37E-17 |
| AC097662.2 | -0.915692614 | -1.016321766 | -8.677089339 | 7.42E-17 |
| SPTLC2 | -0.653947186 | 6.776477581 | -8.676064307 | 7.48E-17 |
| DUSP19 | -1.108079925 | 0.517085836 | -8.675149797 | 7.53E-17 |
| RCSD1 | -0.656062673 | 7.597941064 | -8.673300761 | 7.64E-17 |
| BCLAF1 | 0.434042253 | 8.012015348 | 8.671897525 | 7.72E-17 |
| CABP7 | 1.097953941 | -2.253202086 | 8.67093939 | 7.77E-17 |
| SNORD71 | 1.070062705 | 0.829320651 | 8.666859634 | 8.01E-17 |
| SLC16A13 | -0.975813724 | 2.119141808 | -8.665045294 | 8.12E-17 |
| ERMN | 1.452537498 | 2.206601318 | 8.661305221 | 8.36E-17 |
| KRAS | 0.415467448 | 6.246161533 | 8.660144678 | 8.43E-17 |
| RNU6-574P | 1.221934649 | 1.268644143 | 8.656039768 | 8.69E-17 |
| RFC3 | 0.536849978 | 4.5254428 | 8.655016082 | 8.76E-17 |
| UQCC1 | -0.452874636 | 4.048291419 | -8.650161146 | 9.09E-17 |
| FBXO8 | 0.455417802 | 3.794978404 | 8.650031135 | 9.10E-17 |
| ARMC7 | -0.524350675 | 4.414685581 | -8.64921911 | 9.15E-17 |
| SLC15A2 | -0.852021938 | 4.317120592 | -8.649122971 | 9.16E-17 |
| ENTPD3-AS1 | -0.687110903 | 0.839956283 | -8.6469548 | 9.31E-17 |
| COPB2 | -0.563872952 | 5.82483371 | -8.645586374 | 9.40E-17 |
| N4BP3 | 1.485210947 | 0.454818422 | 8.645083876 | 9.44E-17 |
| VMP1 | -0.615932009 | 6.809366458 | -8.643198286 | 9.58E-17 |
| C14orf119 | -0.728993418 | 4.691594373 | -8.643099781 | 9.58E-17 |
| ANKDD1A | 1.01695673 | 2.247898981 | 8.643081672 | 9.58E-17 |
| ATG16L1 | 0.448128183 | 4.905050359 | 8.642822428 | 9.60E-17 |
| GMEB1 | 0.287009005 | 4.670070521 | 8.641893854 | 9.67E-17 |
| PDE6D | -0.392366146 | 5.146481125 | -8.64156364 | 9.69E-17 |
| CD99L2 | -0.454289133 | 5.426497312 | -8.635255499 | 1.02E-16 |
| DCP1B | -0.545143418 | 4.154934035 | -8.63480847 | 1.02E-16 |
| TRAPPC1 | -0.379268781 | 7.571871773 | -8.634362018 | 1.02E-16 |
| RP5-940J5.6 | -1.053919429 | 0.26375729 | -8.631529823 | 1.05E-16 |
| PARP10 | -0.499915688 | 6.295304221 | -8.631326043 | 1.05E-16 |
| SNAI3-AS1 | -0.743475568 | 0.935416225 | -8.631228821 | 1.05E-16 |
| OSGIN2 | 0.487449216 | 5.573686777 | 8.630537965 | 1.05E-16 |
| FAM206A | -0.313395621 | 4.85273216 | -8.629244299 | 1.06E-16 |
| DET1 | -0.466603254 | 3.478069733 | -8.629170844 | 1.06E-16 |
| HBS1L | 0.400071185 | 5.093867518 | 8.62906622 | 1.06E-16 |
| DHTKD1 | -0.570268258 | 5.125530176 | -8.624338986 | 1.10E-16 |
| PPIL4 | 0.3500344 | 6.331618832 | 8.615055567 | 1.18E-16 |
| CREM | 0.643045479 | 3.231731413 | 8.613509335 | 1.20E-16 |
| BHLHE40 | 1.095380799 | 7.720528595 | 8.610852431 | 1.22E-16 |
| RP11-1109F11.3 | -1.179763436 | -1.258535373 | -8.608730864 | 1.24E-16 |
| LACTB2 | -0.890733566 | 4.168079713 | -8.605384529 | 1.27E-16 |
| CTD-2623N2.3 | -0.702882435 | 2.913671163 | -8.597703744 | 1.35E-16 |
| PLAA | 0.324125404 | 5.746209806 | 8.596069158 | 1.36E-16 |
| NMT1 | -0.506683421 | 5.968070099 | -8.595811153 | 1.37E-16 |
| TTK | 1.047317963 | 3.077200083 | 8.594863694 | 1.38E-16 |
| ELMSAN1 | 0.577298585 | 5.865654934 | 8.593179538 | 1.39E-16 |
| PTPLB | -0.394100617 | 5.194966241 | -8.590774604 | 1.42E-16 |
| OPRL1 | -0.754137235 | 3.77230294 | -8.590231022 | 1.42E-16 |
| CARD6 | -0.768653828 | 4.161780624 | -8.589280611 | 1.43E-16 |
| TMLHE | -0.385080654 | 4.208606856 | -8.587466781 | 1.45E-16 |
| JMJD1C | 0.474097383 | 6.72207994 | 8.587332815 | 1.46E-16 |
| CX3CR1 | -2.238769247 | 3.74462183 | -8.586994109 | 1.46E-16 |
| DDX12P | 0.571448166 | 4.291858281 | 8.586561698 | 1.46E-16 |
| RHEB | 0.421152112 | 5.103339449 | 8.583689998 | 1.50E-16 |
| CREBBP | 0.346793722 | 6.635473488 | 8.583481486 | 1.50E-16 |
| RP11-33O4.1 | -0.642170284 | 2.13012448 | -8.58257447 | 1.51E-16 |
| GORASP2 | 0.253924297 | 6.357851484 | 8.580546819 | 1.53E-16 |
| HNRNPLP2 | 0.305498991 | 7.507240086 | 8.580489181 | 1.53E-16 |
| SGTA | 0.286122856 | 6.52604356 | 8.579737872 | 1.54E-16 |
| NFKBID | 0.835186465 | 5.352157538 | 8.579507508 | 1.54E-16 |
| LPCAT4 | -0.505489851 | 5.081135078 | -8.577472715 | 1.57E-16 |
| CNTRL | -0.460721714 | 6.525524593 | -8.574927416 | 1.60E-16 |
| FNDC7 | 1.056754137 | -2.33517718 | 8.57357691 | 1.61E-16 |
| ZBED5-AS1 | -0.565181876 | 3.578502155 | -8.573228253 | 1.62E-16 |
| ATF6B | -0.345037046 | 7.87555086 | -8.572905684 | 1.62E-16 |
| ZNF410 | 0.740758812 | 0.931372508 | 8.571678528 | 1.64E-16 |
| RP3-437C15.1 | 0.370691881 | 6.490934104 | 8.570398177 | 1.65E-16 |
| C6orf229 | -1.027698296 | -1.031435305 | -8.570294992 | 1.65E-16 |
| NIPAL4 | 1.417982854 | -1.223726498 | 8.56675466 | 1.70E-16 |
| PSMB2 | 0.244613944 | 7.595247537 | 8.565906942 | 1.71E-16 |
| LY6G5B | 0.649517158 | 3.457661402 | 8.565894554 | 1.71E-16 |
| SMNDC1 | 0.272876441 | 5.620349456 | 8.558311289 | 1.81E-16 |
| ZNF592 | 0.265415192 | 6.037094474 | 8.555241695 | 1.85E-16 |
| IQGAP2 | -0.565994377 | 7.044995755 | -8.555012427 | 1.85E-16 |
| SYF2 | 0.421189113 | 7.01851043 | 8.55440257 | 1.86E-16 |
| ZNF146 | -0.578907069 | 6.434572388 | -8.54953642 | 1.93E-16 |
| RNF19A | 0.465071042 | 6.211731033 | 8.549389685 | 1.93E-16 |
| ATG14 | 0.575344006 | 5.499971903 | 8.548242105 | 1.95E-16 |
| RP11-97O12.6 | -0.847456416 | 1.232436759 | -8.545431059 | 1.99E-16 |
| ZNF850 | 0.802364909 | 2.39610858 | 8.544564416 | 2.00E-16 |
| INO80 | 0.247948322 | 5.685409074 | 8.536196444 | 2.13E-16 |
| ZSCAN20 | -0.756455196 | 0.961555657 | -8.532197428 | 2.20E-16 |
| CIC | 0.315336954 | 6.687225578 | 8.531588821 | 2.21E-16 |
| AGAP5 | -0.990928187 | -1.986881665 | -8.530892955 | 2.22E-16 |
| PSPN | -0.713390411 | 1.241190265 | -8.53030644 | 2.23E-16 |
| LRR1 | 0.325768834 | 4.588254881 | 8.529066897 | 2.25E-16 |
| TRAPPC6B | -0.422959262 | 4.963018915 | -8.526624268 | 2.29E-16 |
| PRMT10 | 0.437710562 | 4.123896474 | 8.521155958 | 2.38E-16 |
| DDX24 | 0.44873104 | 7.244408175 | 8.518853984 | 2.43E-16 |
| HEATR3 | -0.57247722 | 4.731717179 | -8.518097744 | 2.44E-16 |
| FAM216A | -0.726574997 | 4.386239317 | -8.517741976 | 2.45E-16 |
| DPH3 | -0.447608766 | 4.781779654 | -8.515090378 | 2.49E-16 |
| PKNOX1 | -0.324423062 | 3.670080823 | -8.514074051 | 2.51E-16 |
| USP33 | -0.239698293 | 5.676081751 | -8.512963194 | 2.53E-16 |
| PFKFB3 | 0.982341997 | 6.317184668 | 8.510947109 | 2.57E-16 |
| ZBTB48 | -0.277237647 | 5.088259474 | -8.50990129 | 2.59E-16 |
| ARSK | -0.636779956 | 1.931046367 | -8.506637558 | 2.66E-16 |
| TAS2R5 | -0.989687937 | 1.790791304 | -8.505859109 | 2.67E-16 |
| C1orf86 | 0.33045943 | 3.480655105 | 8.503052155 | 2.73E-16 |
| TFIP11 | 0.367369246 | 5.349214923 | 8.501258824 | 2.76E-16 |
| RBMXL1 | 0.341835203 | 6.005672309 | 8.500879899 | 2.77E-16 |
| BRAF | 0.390088567 | 5.250147408 | 8.50044488 | 2.78E-16 |
| MRPS22 | 0.284650484 | 4.474497886 | 8.497781388 | 2.84E-16 |
| ACYP1 | -0.606610225 | 4.210505981 | -8.496971362 | 2.85E-16 |
| RBCK1 | -0.300249897 | 6.634896484 | -8.495763696 | 2.88E-16 |
| PTGR2 | -0.864684757 | 1.056084608 | -8.493504784 | 2.93E-16 |
| CCDC82 | 0.271703262 | 5.386766984 | 8.493083941 | 2.94E-16 |
| RP11-817O13.8 | -0.641561469 | 1.961353752 | -8.493000529 | 2.94E-16 |
| YWHAEP1 | -0.674145337 | 3.424062809 | -8.492774211 | 2.94E-16 |
| SLC25A19 | -0.724023689 | 4.060374229 | -8.491762847 | 2.97E-16 |
| FAM111A | -0.551089474 | 6.190482028 | -8.490727784 | 2.99E-16 |
| SNX19 | -0.347878825 | 5.685133852 | -8.489985938 | 3.01E-16 |
| NECAP1 | 0.622537184 | 6.042412746 | 8.487986186 | 3.05E-16 |
| POMZP3 | 0.652902451 | 4.352056999 | 8.487900395 | 3.05E-16 |
| DAD1 | -0.589875682 | 8.556019451 | -8.486311006 | 3.09E-16 |
| FOXM1 | 1.147609808 | 4.586740768 | 8.482728882 | 3.17E-16 |
| NDUFB5 | -0.45998589 | 5.120356169 | -8.482052927 | 3.19E-16 |
| GNL1 | 0.462403879 | 5.156791136 | 8.479936918 | 3.24E-16 |
| SNW1 | 0.251228167 | 7.785754934 | 8.478952025 | 3.26E-16 |
| RP11-613F22.8 | 1.016032943 | 0.913320397 | 8.4776072 | 3.29E-16 |
| ABCF2 | -0.456644124 | 5.291608266 | -8.477541836 | 3.30E-16 |
| TRIM5 | -0.488113233 | 4.4239646 | -8.476353648 | 3.33E-16 |
| TM9SF2 | -0.370164868 | 7.77251751 | -8.476034792 | 3.33E-16 |
| LINC00998 | -0.645821095 | 4.99882459 | -8.475997877 | 3.33E-16 |
| CTD-2501E16.2 | -0.992646913 | -0.426884147 | -8.473998265 | 3.38E-16 |
| ZNF133 | -0.559706559 | 3.05822107 | -8.473288636 | 3.40E-16 |
| RP11-61L19.3 | 0.746443904 | 3.018079443 | 8.472579819 | 3.42E-16 |
| JAGN1 | -0.387627006 | 6.008463645 | -8.470689063 | 3.47E-16 |
| LCOR | 0.428702262 | 5.363735259 | 8.470577522 | 3.47E-16 |
| DDX46 | -0.337952142 | 6.157555405 | -8.468497673 | 3.52E-16 |
| NDUFA13 | 0.621359477 | 1.557881186 | 8.465849036 | 3.59E-16 |
| FOXO3 | 0.536710944 | 5.814718444 | 8.460888865 | 3.73E-16 |
| RBM44 | 0.926504588 | 1.248062414 | 8.460295564 | 3.75E-16 |
| PROCA1 | -0.615596821 | 2.934812977 | -8.45978784 | 3.76E-16 |
| WDR6 | -0.498930616 | 7.094845836 | -8.454417346 | 3.91E-16 |
| SLC2A1 | 0.93763628 | 5.683870854 | 8.453288171 | 3.94E-16 |
| RP11-576N17.5 | -0.996538397 | 0.808748809 | -8.45303388 | 3.95E-16 |
| CTD-2196E14.9 | -0.555160716 | 3.177069814 | -8.451461279 | 4.00E-16 |
| RSF1 | 0.236710249 | 4.933712809 | 8.448250066 | 4.09E-16 |
| HIC2 | 0.501476166 | 3.792839304 | 8.447805764 | 4.11E-16 |
| GOLGA3 | -0.294022413 | 5.464539574 | -8.445752292 | 4.17E-16 |
| SPG20 | -0.505570987 | 6.120803467 | -8.443408044 | 4.24E-16 |
| SCAF4 | 0.323810357 | 6.357711307 | 8.441846681 | 4.29E-16 |
| DNAJC25 | 0.607492577 | 3.983862787 | 8.440827898 | 4.33E-16 |
| PAIP1 | 0.384568024 | 5.314642032 | 8.438740452 | 4.39E-16 |
| HELLS | 0.737883487 | 3.475588286 | 8.436892828 | 4.45E-16 |
| HTATSF1P2 | -1.060474327 | 3.988932897 | -8.436778123 | 4.46E-16 |
| HSPA14 | 0.349684981 | 5.320085642 | 8.435617467 | 4.49E-16 |
| WEE1 | 0.623771419 | 3.796284485 | 8.43549728 | 4.50E-16 |
| ATF4 | 0.402691189 | 9.807254128 | 8.434797228 | 4.52E-16 |
| MGST2 | -0.647455169 | 5.24893594 | -8.434428332 | 4.53E-16 |
| SLC39A1 | -0.410833397 | 6.338578686 | -8.434197526 | 4.54E-16 |
| RP1-193H18.2 | -0.987731053 | 1.599734255 | -8.429363248 | 4.71E-16 |
| ZNF512 | -0.507567901 | 5.04844008 | -8.426774968 | 4.80E-16 |
| CD3EAP | -1.10677572 | 1.775731171 | -8.426618221 | 4.80E-16 |
| REP15 | -1.177686073 | -0.834857508 | -8.426128571 | 4.82E-16 |
| GALK1 | -0.535234247 | 4.667454408 | -8.424313607 | 4.89E-16 |
| LINC00909 | -0.560361171 | 3.453954775 | -8.423490939 | 4.92E-16 |
| IVD | -0.464115474 | 5.083724929 | -8.421996672 | 4.97E-16 |
| RPP40 | -0.876160745 | 3.388054885 | -8.417802198 | 5.13E-16 |
| GRSF1 | -0.30591847 | 5.603602888 | -8.417127846 | 5.15E-16 |
| DNAJC9 | 0.450263971 | 5.699832462 | 8.416961152 | 5.16E-16 |
| FAM160B1 | 0.602766972 | 5.867084239 | 8.410819929 | 5.40E-16 |
| RP11-504P24.2 | -0.635234542 | 2.857258723 | -8.408365226 | 5.50E-16 |
| RP11-358L4.1 | -0.975015818 | 0.035557634 | -8.408332705 | 5.50E-16 |
| TAS2R4 | -1.177617883 | 0.446797313 | -8.407816628 | 5.52E-16 |
| EIF2B1 | -0.213257135 | 6.157558235 | -8.406275402 | 5.58E-16 |
| PIK3R6 | -0.873792261 | 5.079472828 | -8.405630663 | 5.61E-16 |
| NARS | -0.314648656 | 6.783177349 | -8.405250135 | 5.62E-16 |
| RP11-133N21.7 | -1.070908755 | -0.117192823 | -8.404068161 | 5.67E-16 |
| ZNF598 | 0.307896448 | 6.133319036 | 8.400036644 | 5.84E-16 |
| SCAP | -0.369369794 | 6.092782335 | -8.399986833 | 5.85E-16 |
| RELA | 0.34775524 | 6.663660967 | 8.395147776 | 6.06E-16 |
| VIPAS39 | -0.331233016 | 5.211879996 | -8.394869176 | 6.07E-16 |
| ZNF285 | -1.361407605 | 0.005864018 | -8.393683422 | 6.12E-16 |
| SECISBP2L | 0.359062338 | 5.644301519 | 8.392185375 | 6.19E-16 |
| RP11-365O16.6 | -0.884772361 | -1.186240399 | -8.390962531 | 6.25E-16 |
| AC125232.1 | 0.503376623 | 5.121158924 | 8.389935513 | 6.29E-16 |
| MYL5 | 0.639758967 | 3.073259792 | 8.387220649 | 6.42E-16 |
| RP11-468O2.1 | -0.960857419 | -1.400159994 | -8.387051155 | 6.43E-16 |
| AC018867.1 | 1.105552049 | 3.061462873 | 8.386975114 | 6.43E-16 |
| DDX23 | -0.456628685 | 6.227003134 | -8.386168728 | 6.47E-16 |
| SPTY2D1 | 0.58451787 | 5.74880962 | 8.384338695 | 6.56E-16 |
| RP11-637A17.2 | -0.771162637 | 0.463074169 | -8.383951168 | 6.58E-16 |
| RP11-363E6.4 | -1.091214607 | -0.45512324 | -8.38352346 | 6.60E-16 |
| NET1 | 0.889288803 | 6.115820746 | 8.38238388 | 6.65E-16 |
| SLC29A3 | -0.640847962 | 4.13717211 | -8.382099586 | 6.67E-16 |
| MED14 | 0.250159266 | 5.099587988 | 8.380817423 | 6.73E-16 |
| ATP2A1 | 0.698108538 | 0.952200183 | 8.37814513 | 6.87E-16 |
| C17orf59 | -0.513419736 | 4.917994808 | -8.377219527 | 6.91E-16 |
| RIMBP3B | -1.309566537 | -3.498756136 | -8.375499429 | 7.00E-16 |
| RSBN1L | -0.380797674 | 4.474702762 | -8.37497246 | 7.03E-16 |
| RP11-7O11.3 | -1.081940127 | 1.642235633 | -8.371410748 | 7.21E-16 |
| DOT1L | 0.348912921 | 5.587477112 | 8.369352632 | 7.32E-16 |
| KIF2C | 1.107191099 | 3.933337978 | 8.368111389 | 7.39E-16 |
| UBE2FP1 | 0.938651515 | 4.491441495 | 8.367941863 | 7.40E-16 |
| MIB1 | -0.456874564 | 5.528133186 | -8.366367574 | 7.49E-16 |
| AQP8 | 1.045805583 | -1.54383687 | 8.365890544 | 7.51E-16 |
| PCP2 | -0.769881737 | 2.941110944 | -8.365780056 | 7.52E-16 |
| RNF126 | 0.294861196 | 6.126109664 | 8.36364218 | 7.64E-16 |
| MOSPD2 | -0.841312567 | 4.151327188 | -8.363479076 | 7.65E-16 |
| SMIM13 | 0.47483933 | 3.415093149 | 8.362549113 | 7.70E-16 |
| ZNFX1 | 0.514724663 | 5.486865536 | 8.360390017 | 7.82E-16 |
| TAPT1-AS1 | -0.702766853 | 2.918341535 | -8.360081645 | 7.84E-16 |
| CNOT1 | -0.362813341 | 7.012253476 | -8.359048307 | 7.90E-16 |
| PPIF | 0.566739262 | 7.323673817 | 8.358669628 | 7.92E-16 |
| NAP1L4P1 | 0.80272748 | 3.411865461 | 8.358380907 | 7.94E-16 |
| ANKRD9 | 1.034278251 | 3.933064844 | 8.353433172 | 8.23E-16 |
| AC084219.4 | -1.127114017 | -1.022850897 | -8.352170765 | 8.31E-16 |
| CTC-471F3.6 | -0.900925823 | 0.32226698 | -8.352064867 | 8.31E-16 |
| SAMD9L | -0.737202128 | 5.75753897 | -8.351031545 | 8.38E-16 |
| OGDH | -0.561708833 | 6.605865224 | -8.350255611 | 8.43E-16 |
| IRF2 | -0.751216079 | 5.895030797 | -8.347317219 | 8.61E-16 |
| WDR27 | -0.618948255 | 3.572691079 | -8.34600673 | 8.69E-16 |
| CPNE1 | -0.413279579 | 7.068058077 | -8.345425205 | 8.73E-16 |
| ARID4A | 0.352534991 | 5.69415509 | 8.344624557 | 8.78E-16 |
| NUFIP1 | 0.435084734 | 3.654801178 | 8.344094055 | 8.81E-16 |
| RHOT1P3 | -0.486144022 | 5.052980734 | -8.340161383 | 9.07E-16 |
| MAPK8IP1 | 1.038895911 | 0.512370583 | 8.339613324 | 9.11E-16 |
| MPP5 | 0.485058467 | 3.035313255 | 8.339060029 | 9.15E-16 |
| DISP1 | -0.774812839 | 1.148245742 | -8.336603845 | 9.31E-16 |
| LRCH3 | -0.295285618 | 4.094240574 | -8.335035082 | 9.42E-16 |
| RPS19BP1 | 0.306023278 | 5.343722629 | 8.33388903 | 9.50E-16 |
| LINC00861 | -1.60706126 | 1.396454154 | -8.330755331 | 9.72E-16 |
| GID8 | 0.294457533 | 7.360696905 | 8.330076344 | 9.77E-16 |
| TRIM52-AS1 | 0.621215812 | 4.898258954 | 8.327740918 | 9.94E-16 |
| RAP2C | 0.404842265 | 6.490493242 | 8.327260655 | 9.97E-16 |
| TATDN2 | 0.357841004 | 4.821340988 | 8.32654267 | 1.00E-15 |
| RNF4 | 0.320788055 | 5.656699512 | 8.325633163 | 1.01E-15 |
| NGDN | 0.417101216 | 5.050842287 | 8.324735487 | 1.02E-15 |
| ETF1P2 | 0.764307795 | 2.630053259 | 8.324479127 | 1.02E-15 |
| NNT-AS1 | -0.767197691 | 2.104089042 | -8.32320164 | 1.03E-15 |
| PQLC3 | -0.55798607 | 5.928638214 | -8.318932931 | 1.06E-15 |
| STK19 | 0.377224601 | 4.383924823 | 8.318693155 | 1.06E-15 |
| AC025171.1 | -0.630681341 | 4.095999501 | -8.318630771 | 1.06E-15 |
| RP11-767N6.2 | 0.725939817 | 3.562635362 | 8.317754755 | 1.07E-15 |
| RIMOC1 | -0.512183925 | 4.648144043 | -8.313064129 | 1.11E-15 |
| RP11-348P10.2 | 0.885997341 | 3.213412324 | 8.31245348 | 1.11E-15 |
| GPR156 | 1.043028145 | -3.089098215 | 8.311382685 | 1.12E-15 |
| C12orf29 | 0.514257205 | 3.646403753 | 8.311259606 | 1.12E-15 |
| CLCF1 | 1.119954531 | 3.335753704 | 8.30974188 | 1.13E-15 |
| ACLY | -0.409380803 | 7.252752158 | -8.309718783 | 1.13E-15 |
| C11orf96 | 1.463655784 | 1.528867714 | 8.308710531 | 1.14E-15 |
| ZBTB2 | 0.418526887 | 5.503215118 | 8.30799366 | 1.15E-15 |
| PPID | -0.349685847 | 6.289054044 | -8.307908372 | 1.15E-15 |
| BAG3 | 1.502277221 | 3.53586257 | 8.302952671 | 1.19E-15 |
| PLEKHM3 | 0.51983144 | 3.945840639 | 8.302337963 | 1.20E-15 |
| TAF12 | -0.402099725 | 5.912189836 | -8.29878795 | 1.23E-15 |
| SETD8 | 0.347821797 | 5.409099721 | 8.294024254 | 1.27E-15 |
| ZNF426 | 0.484704069 | 4.020680646 | 8.293402543 | 1.28E-15 |
| NFKB2 | 0.861272327 | 5.672109439 | 8.292798957 | 1.28E-15 |
| CTD-3247F14.2 | 1.847146845 | -1.18229002 | 8.292678091 | 1.28E-15 |
| AC133528.2 | -0.481831409 | 4.626463843 | -8.290287265 | 1.31E-15 |
| TMEM186 | -0.57524425 | 2.942999167 | -8.28800362 | 1.33E-15 |
| CCSAP | 0.525737011 | 4.701180404 | 8.287001062 | 1.34E-15 |
| CYBRD1 | -0.701425666 | 5.668825621 | -8.285852821 | 1.35E-15 |
| DNAJC10 | -0.4927459 | 5.709657017 | -8.284244173 | 1.37E-15 |
| ITPA | -0.407354014 | 5.75830192 | -8.282375169 | 1.38E-15 |
| IFI27L1 | 0.429294181 | 3.664334092 | 8.281715431 | 1.39E-15 |
| PTPLAD2 | -0.469067019 | 6.06295338 | -8.279861958 | 1.41E-15 |
| BNIP1 | 0.609697338 | 4.401760669 | 8.275230379 | 1.46E-15 |
| RP11-165F24.2 | -0.913273446 | -0.566257823 | -8.274415115 | 1.47E-15 |
| PIGA | 0.740261038 | 5.264269163 | 8.274201569 | 1.47E-15 |
| MIR4477A | -1.097577939 | 3.5274009 | -8.273277877 | 1.48E-15 |
| XPC | -0.368771036 | 5.691387037 | -8.271268732 | 1.50E-15 |
| RNF26 | 0.339602833 | 5.995200323 | 8.270395564 | 1.51E-15 |
| INTS7 | -0.525616397 | 4.39312425 | -8.270013374 | 1.51E-15 |
| MCTS1 | -0.301654879 | 5.010026918 | -8.267276965 | 1.54E-15 |
| MMACHC | -0.923155427 | 2.266419089 | -8.263742242 | 1.59E-15 |
| JADE2 | -0.532497593 | 5.140136959 | -8.263434713 | 1.59E-15 |
| RP5-935K16.1 | -0.527730355 | 4.341696025 | -8.262223012 | 1.60E-15 |
| SLC46A1 | -1.181038487 | 1.144699641 | -8.26079606 | 1.62E-15 |
| TRAF6P1 | 0.723431578 | 0.591998765 | 8.259691516 | 1.63E-15 |
| GAPT | -1.111901046 | 6.379488297 | -8.258814217 | 1.64E-15 |
| NAPEPLD | -0.794696446 | 1.557780761 | -8.257459932 | 1.66E-15 |
| RNFT1 | -0.345481205 | 4.960297164 | -8.255960714 | 1.68E-15 |
| C22orf39 | -0.259907885 | 4.602762729 | -8.255595112 | 1.68E-15 |
| ZNF583 | -0.671362976 | 0.919122764 | -8.251895591 | 1.73E-15 |
| NUP98 | 0.322862219 | 6.478171642 | 8.247544636 | 1.78E-15 |
| RP11-15A1.7 | -1.12618562 | -1.110107443 | -8.245889261 | 1.81E-15 |
| C1orf63 | -0.493433047 | 6.846203043 | -8.244540598 | 1.82E-15 |
| BOLA1 | -0.741857401 | 3.554188388 | -8.243957895 | 1.83E-15 |
| RP11-73E17.2 | 0.540397828 | 4.856902538 | 8.24301682 | 1.84E-15 |
| NR1D2 | 0.728338924 | 4.951929637 | 8.241568783 | 1.86E-15 |
| COL1A1 | 1.610714373 | -1.724499149 | 8.238478328 | 1.90E-15 |
| TTLL11 | -0.564265021 | 2.031015038 | -8.237890487 | 1.91E-15 |
| PRKCI | -0.330596505 | 4.874437172 | -8.236889314 | 1.93E-15 |
| TFB1M | -0.441208451 | 3.520706719 | -8.236099939 | 1.94E-15 |
| ARMCX5 | 0.424282148 | 3.644743039 | 8.235889916 | 1.94E-15 |
| RP11-230C9.2 | -0.890399379 | 0.625765184 | -8.235578981 | 1.95E-15 |
| ATP2B4 | -0.656051463 | 6.837115268 | -8.235545018 | 1.95E-15 |
| RP11-318A15.2 | -0.951481386 | -0.569257409 | -8.235145777 | 1.95E-15 |
| RP11-4K3__A.3 | -0.886160568 | -0.209935544 | -8.233455574 | 1.98E-15 |
| AC006978.6 | 0.541531837 | 6.001531092 | 8.23167368 | 2.00E-15 |
| METTL22 | 0.355464186 | 3.933379011 | 8.230403934 | 2.02E-15 |
| IKBKAP | -0.297006549 | 5.202144455 | -8.230150436 | 2.02E-15 |
| SLC38A9 | -0.43443817 | 2.793858237 | -8.230077394 | 2.02E-15 |
| EXOSC5 | -0.68186413 | 5.162988674 | -8.227326415 | 2.07E-15 |
| PBRM1 | -0.375241177 | 6.239030863 | -8.227307978 | 2.07E-15 |
| ZNF585B | -0.572474324 | 3.010020229 | -8.227130285 | 2.07E-15 |
| DIP2A | -0.38026222 | 5.413803096 | -8.226386537 | 2.08E-15 |
| FBXO30 | 0.467128553 | 3.843000608 | 8.224941761 | 2.10E-15 |
| DHX9P1 | 0.344768824 | 3.954684155 | 8.223381059 | 2.13E-15 |
| AC006116.24 | -0.955493234 | -0.393795941 | -8.222359185 | 2.14E-15 |
| HEATR5A | -0.606334294 | 1.99393635 | -8.221876909 | 2.15E-15 |
| TMEM205 | -0.44646212 | 6.346148587 | -8.219795367 | 2.18E-15 |
| RPRD1A | -0.29508037 | 4.844886897 | -8.21872638 | 2.20E-15 |
| NEU4 | 2.089637835 | 2.012683471 | 8.21777009 | 2.21E-15 |
| KRBA2 | -0.966818317 | 1.277008373 | -8.215489166 | 2.25E-15 |
| MAP4K3 | 0.605063068 | 3.956355647 | 8.215251588 | 2.25E-15 |
| RP11-498D10.6 | -0.559210119 | 2.418917331 | -8.213887693 | 2.28E-15 |
| RHOF | 0.66223022 | 5.077981928 | 8.213541515 | 2.28E-15 |
| AC132872.1 | 1.337965471 | 1.466927353 | 8.212097563 | 2.31E-15 |
| NBPF1 | -0.577908266 | 3.973250187 | -8.210248137 | 2.34E-15 |
| SNRPF | 0.375374678 | 5.641413637 | 8.208948493 | 2.36E-15 |
| MFSD6L | -1.069694974 | 0.303708746 | -8.208382761 | 2.37E-15 |
| LINC00339 | -0.496975665 | 4.841434583 | -8.206636073 | 2.40E-15 |
| RP11-686D22.8 | -1.062130486 | 1.922291355 | -8.206499896 | 2.40E-15 |
| AP1G2 | -0.453585216 | 6.628409147 | -8.206037887 | 2.41E-15 |
| RP11-466F5.8 | -0.936385877 | -0.126714559 | -8.20597202 | 2.41E-15 |
| HAS1 | 1.752493458 | -1.785193038 | 8.205256574 | 2.42E-15 |
| CNOT6 | -0.321677104 | 5.518086335 | -8.203812973 | 2.45E-15 |
| CTD-3195I5.1 | -0.422339761 | 5.301091844 | -8.203448933 | 2.46E-15 |
| RP11-690D19.3 | 0.623859646 | 3.116445998 | 8.202673725 | 2.47E-15 |
| KLHL15 | 0.79241903 | 5.763258811 | 8.202013838 | 2.48E-15 |
| ATF7 | -0.359194608 | 5.641278394 | -8.20062155 | 2.51E-15 |
| ITGB1BP2 | 0.608421444 | 1.993206049 | 8.200183242 | 2.51E-15 |
| SDCCAG8 | -0.44767771 | 5.219641019 | -8.198599909 | 2.54E-15 |
| RP1-125I3.2 | -0.87473068 | 0.914839138 | -8.196815342 | 2.58E-15 |
| MCAM | 1.63942238 | -0.786926009 | 8.196209808 | 2.59E-15 |
| CD53 | -0.629146804 | 9.099739527 | -8.195731753 | 2.60E-15 |
| TAF5L | 0.301491101 | 5.047314761 | 8.195218979 | 2.61E-15 |
| PUS10 | -0.461103309 | 2.47813397 | -8.19456307 | 2.62E-15 |
| ARFIP1 | -0.658316137 | 4.06888505 | -8.192736499 | 2.65E-15 |
| IRAK2 | 1.181420774 | 3.150618998 | 8.192118492 | 2.67E-15 |
| TCTN3 | -0.468494216 | 5.274988516 | -8.189668732 | 2.71E-15 |
| DPEP2 | -0.603379599 | 5.59726287 | -8.189441743 | 2.72E-15 |
| NARG2 | -0.348941149 | 4.518829655 | -8.189113398 | 2.72E-15 |
| XBP1P1 | -0.916561063 | -0.688059177 | -8.188818319 | 2.73E-15 |
| ZNF644 | 0.338114681 | 5.296598674 | 8.187936901 | 2.75E-15 |
| NOL9 | 0.290042641 | 4.598318686 | 8.187341373 | 2.76E-15 |
| UBXN6 | 0.326953788 | 6.089804553 | 8.184027286 | 2.83E-15 |
| HEXA-AS1 | -0.862742305 | -0.209398799 | -8.181675083 | 2.88E-15 |
| GEM | 1.478843469 | -0.698525016 | 8.18128857 | 2.88E-15 |
| RP11-307L14.1 | -0.862139494 | -0.164200433 | -8.180132964 | 2.91E-15 |
| RELL1 | 0.753945144 | 5.491377139 | 8.179103231 | 2.93E-15 |
| CIR1 | 0.470105749 | 6.117314138 | 8.177993188 | 2.95E-15 |
| ARHGEF6 | -0.567267894 | 7.833998812 | -8.176010628 | 3.00E-15 |
| RP11-305E6.4 | -0.642156113 | 0.953618846 | -8.173840874 | 3.04E-15 |
| RP4-740C4.6 | -0.940301636 | 1.190508126 | -8.173705468 | 3.05E-15 |
| ZNF787 | 0.300625892 | 6.041458572 | 8.173654439 | 3.05E-15 |
| PTBP2 | 0.446055851 | 4.51359213 | 8.172940199 | 3.06E-15 |
| NARF-IT1 | 0.736387568 | 4.328273125 | 8.161494598 | 3.33E-15 |
| NEK2 | 1.12026469 | 2.637671544 | 8.160261991 | 3.36E-15 |
| DDX39A | 0.373243488 | 6.937874228 | 8.158610983 | 3.40E-15 |
| LACC1 | -0.580290402 | 3.519463545 | -8.157836338 | 3.42E-15 |
| UBE2C | 1.141553006 | 4.708397088 | 8.156958928 | 3.44E-15 |
| TBCE | -0.350208234 | 5.172572837 | -8.155760093 | 3.47E-15 |
| CDCA8 | 0.955197186 | 4.399514187 | 8.154619836 | 3.50E-15 |
| ARMC6 | -0.455271973 | 5.215127739 | -8.149820389 | 3.62E-15 |
| AP001053.11 | -1.937050535 | 1.227694895 | -8.149519299 | 3.63E-15 |
| ZKSCAN7 | -0.981903282 | 1.871248773 | -8.149347336 | 3.63E-15 |
| CDKN2D | 0.82195301 | 7.587450447 | 8.148583768 | 3.65E-15 |
| TBX19 | -0.528127455 | 2.398856188 | -8.148379611 | 3.66E-15 |
| SDC4 | 1.380687541 | 4.028950916 | 8.145161248 | 3.74E-15 |
| TCEB1 | 0.376124769 | 4.515429499 | 8.144805818 | 3.75E-15 |
| TFG | 0.309813826 | 6.130778101 | 8.143343586 | 3.79E-15 |
| PEX11A | -0.804599127 | 1.752719456 | -8.141158197 | 3.85E-15 |
| RP11-496H1.1 | 0.738931858 | 0.588593829 | 8.140837084 | 3.86E-15 |
| ADAM10 | -0.476897527 | 6.386195851 | -8.140681189 | 3.86E-15 |
| CTA-212D2.2 | -0.996641071 | -0.707622461 | -8.135421604 | 4.01E-15 |
| NCAPH | 1.035545193 | 3.987001902 | 8.132823213 | 4.09E-15 |
| GMFB | 0.418320439 | 5.435404719 | 8.131710007 | 4.12E-15 |
| RP11-84C13.2 | -0.955146522 | -0.124386655 | -8.131053899 | 4.14E-15 |
| RP11-574K11.29 | -0.67642797 | 2.097829757 | -8.130332744 | 4.16E-15 |
| RFFL | 0.396795048 | 4.558398823 | 8.130096216 | 4.17E-15 |
| KLF3 | 0.561925854 | 6.711418205 | 8.129703651 | 4.18E-15 |
| RP11-122K13.12 | 0.639699516 | 4.571145872 | 8.127856932 | 4.24E-15 |
| RP11-1020A11.1 | -0.779210276 | 1.423409284 | -8.126218561 | 4.29E-15 |
| PRKRIR | 0.335836118 | 5.954652646 | 8.12503502 | 4.33E-15 |
| PDE2A | 1.495047051 | -0.243290247 | 8.12236149 | 4.41E-15 |
| SDE2 | 0.787968993 | 5.786345536 | 8.121623788 | 4.43E-15 |
| KB-1836B5.1 | -0.97344661 | -0.101402948 | -8.116166885 | 4.61E-15 |
| C2orf47 | 0.371756774 | 4.441511458 | 8.115368519 | 4.64E-15 |
| SIRT4 | -0.877615942 | 0.025041605 | -8.115085159 | 4.65E-15 |
| CYB5B | -0.533640958 | 4.880325405 | -8.113200058 | 4.71E-15 |
| VPS37D | 1.150046506 | -1.401169532 | 8.110881664 | 4.79E-15 |
| ALDH6A1 | -0.568708545 | 3.800395593 | -8.110611494 | 4.80E-15 |
| RP11-428J1.5 | -0.820823357 | 2.461853824 | -8.110369934 | 4.81E-15 |
| RP11-529E10.6 | -0.753504026 | 1.307091545 | -8.109327812 | 4.84E-15 |
| AC002467.7 | -0.810687763 | 2.03605482 | -8.108519265 | 4.87E-15 |
| KRBOX5 | -0.407649501 | 2.821814496 | -8.108367417 | 4.88E-15 |
| RP11-267M23.4 | -0.848534962 | 0.154279478 | -8.107872459 | 4.89E-15 |
| RALBP1 | -0.384863872 | 6.570460226 | -8.107734519 | 4.90E-15 |
| C8orf33 | 0.379852199 | 5.675266675 | 8.107722978 | 4.90E-15 |
| KRBOX4 | -0.37234391 | 3.551354828 | -8.1061729 | 4.95E-15 |
| NR1H3 | -0.808849294 | 4.169944263 | -8.105386256 | 4.98E-15 |
| MCMDC2 | -1.090813761 | -0.908284187 | -8.101717778 | 5.11E-15 |
| ABHD13 | 0.403854733 | 5.66252204 | 8.101439161 | 5.12E-15 |
| CTC-241F20.3 | -0.662821215 | 4.734310748 | -8.10010253 | 5.17E-15 |
| KBTBD2 | 0.384198 | 5.956989052 | 8.098805218 | 5.22E-15 |
| SIRPB2 | -1.339702232 | 4.895767134 | -8.097186028 | 5.28E-15 |
| BIRC5 | 1.145301685 | 3.907739552 | 8.096494393 | 5.31E-15 |
| TTLL1 | -0.624293262 | 4.410509226 | -8.096052527 | 5.33E-15 |
| TMEM177 | -0.840121863 | 2.11384393 | -8.095917967 | 5.33E-15 |
| HEATR5B | -0.446698721 | 5.998576087 | -8.094626766 | 5.38E-15 |
| TAB1 | -0.322295807 | 5.12833887 | -8.089040913 | 5.60E-15 |
| RP11-451M19.3 | 0.978420461 | -0.544263179 | 8.087429304 | 5.67E-15 |
| ST7-AS1 | 1.509745458 | 1.604049141 | 8.087365497 | 5.67E-15 |
| C16orf72 | 0.475634915 | 5.680212248 | 8.08729717 | 5.67E-15 |
| VPS18 | 0.361557608 | 5.58041793 | 8.086904348 | 5.69E-15 |
| FAF2 | 0.307544979 | 5.44527459 | 8.083976197 | 5.81E-15 |
| RPAP3 | -0.306589467 | 5.472181516 | -8.081466136 | 5.91E-15 |
| USPL1 | 0.445297643 | 5.673674532 | 8.08100671 | 5.93E-15 |
| C11orf71 | -0.462317038 | 4.454973895 | -8.080368621 | 5.96E-15 |
| MIR4742 | 1.009785156 | 1.56793301 | 8.08027082 | 5.96E-15 |
| RSBN1 | 0.343646628 | 5.347798868 | 8.079828006 | 5.98E-15 |
| PLEKHF2 | 0.542186397 | 6.694613267 | 8.077656677 | 6.08E-15 |
| B3GNT7 | 1.486167141 | 2.613246753 | 8.075903851 | 6.15E-15 |
| RP11-468E2.4 | 0.88308855 | 1.476690317 | 8.074765027 | 6.20E-15 |
| SOGA1 | -0.615064854 | 4.519553312 | -8.073891062 | 6.24E-15 |
| MAGEF1 | -0.801470261 | 5.397158568 | -8.072802555 | 6.29E-15 |
| MMS22L | 0.493690621 | 3.527659144 | 8.072558684 | 6.30E-15 |
| IQCH-AS1 | -0.715510857 | 2.330199796 | -8.072127152 | 6.32E-15 |
| PDDC1 | -0.428839924 | 4.968060622 | -8.072092325 | 6.32E-15 |
| PIM3 | 0.895936343 | 8.088689907 | 8.072002801 | 6.33E-15 |
| DFFA | -0.417861847 | 5.135697741 | -8.071951861 | 6.33E-15 |
| EXTL2 | -0.7170714 | 3.201656208 | -8.070764279 | 6.38E-15 |
| IFNAR1 | -0.489478513 | 5.548390522 | -8.070345697 | 6.40E-15 |
| RGS13 | 1.425192214 | -1.921066464 | 8.069622631 | 6.44E-15 |
| APOL1 | -0.600371413 | 4.660107826 | -8.068258345 | 6.50E-15 |
| KDM4A | -0.445076839 | 5.785745978 | -8.067295627 | 6.54E-15 |
| HSPE1P26 | 1.076170912 | -0.30951319 | 8.067039934 | 6.56E-15 |
| RTTN | 0.377359493 | 3.005030938 | 8.067038853 | 6.56E-15 |
| RP11-446E9.1 | -0.360907301 | 5.991989444 | -8.065074885 | 6.65E-15 |
| KANSL2 | 0.576143034 | 5.90638544 | 8.06429674 | 6.69E-15 |
| BTN3A3 | -0.669700031 | 4.581465444 | -8.063881466 | 6.71E-15 |
| HDDC3 | -0.446071825 | 5.138688057 | -8.062873171 | 6.76E-15 |
| RPL23AP7 | -0.513163307 | 3.806878061 | -8.062812581 | 6.76E-15 |
| GUK1 | 0.369533276 | 6.732910984 | 8.062239757 | 6.79E-15 |
| GIMAP2 | -0.743660403 | 5.425036304 | -8.061457994 | 6.82E-15 |
| RP11-686D22.3 | -1.098355581 | 0.64377964 | -8.061048499 | 6.84E-15 |
| RP11-154H23.3 | -0.982119851 | 1.440487882 | -8.059201733 | 6.93E-15 |
| LIAS | -0.317124708 | 4.179890097 | -8.054740247 | 7.16E-15 |
| RP11-355B11.2 | -0.668326324 | 2.455119705 | -8.05382214 | 7.21E-15 |
| ADCK1 | -0.52318554 | 2.681490178 | -8.048995658 | 7.46E-15 |
| RAB30 | 1.031062709 | 1.063029398 | 8.04799844 | 7.51E-15 |
| SFN | 1.168627703 | 0.322290625 | 8.047775783 | 7.53E-15 |
| ANGPTL4 | 1.780417468 | -1.410610374 | 8.047691167 | 7.53E-15 |
| ATXN2 | -0.468721373 | 4.391551251 | -8.045522533 | 7.65E-15 |
| RP11-977G19.5 | -0.4007796 | 5.607203634 | -8.044788341 | 7.69E-15 |
| FAM168B | 0.274755553 | 7.24549492 | 8.043232961 | 7.77E-15 |
| CDK10 | -0.39822846 | 4.890476364 | -8.042613458 | 7.81E-15 |
| LEAP2 | -0.836878741 | 0.291144973 | -8.042107948 | 7.84E-15 |
| C4orf29 | -0.260759294 | 4.20454773 | -8.04184412 | 7.85E-15 |
| TEFM | -0.414315691 | 2.828088585 | -8.041621567 | 7.86E-15 |
| CTD-2026D20.2 | -0.915431502 | 0.560053432 | -8.040828784 | 7.91E-15 |
| AC016773.1 | 0.655314795 | 2.767327839 | 8.040727239 | 7.91E-15 |
| FFAR1 | 1.259182722 | -0.587931585 | 8.040617155 | 7.92E-15 |
| DPAGT1 | -0.560660888 | 4.525279696 | -8.040201786 | 7.94E-15 |
| USP49 | 0.398765483 | 3.495163627 | 8.040114774 | 7.95E-15 |
| RBM12B | -0.494720952 | 4.381742088 | -8.039456971 | 7.99E-15 |
| RP11-119B16.2 | -0.646208912 | 2.792208789 | -8.03513408 | 8.24E-15 |
| RP11-541N10.3 | -0.672501571 | 3.633281015 | -8.032811772 | 8.37E-15 |
| PXDC1 | 1.346481887 | 0.841797904 | 8.031598749 | 8.45E-15 |
| KIAA1107 | -0.710789086 | 2.22426724 | -8.029757088 | 8.56E-15 |
| KIAA1199 | 1.851554025 | -3.160922329 | 8.028618638 | 8.63E-15 |
| FEM1B | 0.294507003 | 6.126311351 | 8.027914904 | 8.67E-15 |
| C19orf82 | -0.672685089 | 2.179355428 | -8.027679381 | 8.69E-15 |
| GRM2 | 1.117699606 | -0.130913446 | 8.026859735 | 8.74E-15 |
| KCNRG | 0.877862489 | 0.186132949 | 8.026672141 | 8.75E-15 |
| RP11-738E22.3 | -0.764736255 | 0.23239899 | -8.026334258 | 8.77E-15 |
| AC022558.1 | -1.024893583 | -0.107181011 | -8.025607479 | 8.81E-15 |
| SNORD11 | 0.758986898 | 3.159051042 | 8.02395977 | 8.92E-15 |
| PPAP2B | 1.533671567 | 0.869129171 | 8.022547178 | 9.01E-15 |
| CTC-534A2.2 | 0.677899459 | 4.06721583 | 8.021990171 | 9.05E-15 |
| AC079210.1 | -0.977984744 | -0.968953221 | -8.021939789 | 9.05E-15 |
| SLC35A4 | -0.348881975 | 6.062249639 | -8.020469016 | 9.14E-15 |
| C15orf48 | 1.589692588 | 0.107207145 | 8.018886955 | 9.25E-15 |
| GAL3ST2 | 1.614211929 | 0.734072938 | 8.013814519 | 9.59E-15 |
| VEZF1P1 | 0.461929484 | 2.750297956 | 8.004462818 | 1.02E-14 |
| HUS1B | 0.773477466 | 0.850178555 | 8.002821376 | 1.04E-14 |
| ZKSCAN4 | -0.724019725 | 3.689040896 | -8.001232774 | 1.05E-14 |
| 7-Mar | -0.367911708 | 6.864012625 | -8.000770625 | 1.05E-14 |
| UBA6-DT | -0.53431107 | 2.731964285 | -8.000623421 | 1.05E-14 |
| SRF | 0.251587662 | 6.571665743 | 8.000231219 | 1.06E-14 |
| FBXO33 | 0.635533865 | 5.600338763 | 7.998164254 | 1.07E-14 |
| OSER1 | 0.535522613 | 7.278230888 | 7.996460109 | 1.08E-14 |
| SANBR | -0.625396555 | 2.578177888 | -7.994005895 | 1.10E-14 |
| RP11-258C19.7 | -0.700569805 | 3.878195128 | -7.992894182 | 1.11E-14 |
| GLTSCR1 | 0.37420439 | 4.079860293 | 7.991493231 | 1.12E-14 |
| UBE2V1 | 0.591038697 | 2.981416399 | 7.987950198 | 1.15E-14 |
| RINL | -0.545452036 | 3.91516452 | -7.987770706 | 1.15E-14 |
| RP11-773D16.1 | 0.40013592 | 4.847106731 | 7.987399289 | 1.16E-14 |
| TRIM4 | -0.404013238 | 4.789166634 | -7.987200047 | 1.16E-14 |
| RP5-1180C10.2 | -0.583028759 | 3.336297941 | -7.985476614 | 1.17E-14 |
| NEK8 | -0.513511366 | 3.019322554 | -7.982086506 | 1.20E-14 |
| ATRX | -0.308601496 | 6.045436479 | -7.978955573 | 1.23E-14 |
| OCEL1 | -0.548892294 | 4.247319657 | -7.978833234 | 1.23E-14 |
| GTF2I | -0.376905966 | 5.626629163 | -7.977113517 | 1.24E-14 |
| RP11-389C8.3 | -0.795498488 | 1.577441453 | -7.975155911 | 1.26E-14 |
| Z69666.2 | 1.226759682 | -1.103991351 | 7.973752533 | 1.27E-14 |
| CRTC2 | 0.407308316 | 6.90585014 | 7.972970712 | 1.28E-14 |
| CARHSP1 | 0.549958699 | 4.654967227 | 7.96976878 | 1.31E-14 |
| ZCWPW2 | -0.686298987 | 0.218265503 | -7.968651265 | 1.32E-14 |
| CRTC3 | 0.486274204 | 4.854869652 | 7.967471448 | 1.33E-14 |
| PAN2 | -0.337526855 | 5.543194836 | -7.965256812 | 1.35E-14 |
| SMU1 | 0.188185653 | 5.396840235 | 7.964687265 | 1.36E-14 |
| NOSTRIN | 1.031929161 | -3.079669973 | 7.964206323 | 1.36E-14 |
| ORMDL2 | -0.447875584 | 4.47698182 | -7.963197543 | 1.37E-14 |
| RALB | -0.464505793 | 7.384883711 | -7.96231794 | 1.38E-14 |
| MFSD8 | -0.375734278 | 3.467125793 | -7.961387198 | 1.39E-14 |
| RGL2 | -0.421041019 | 6.540008755 | -7.960524902 | 1.40E-14 |
| FOXO3B | 0.611333867 | 2.891739206 | 7.960154142 | 1.40E-14 |
| BRD7 | 0.24086299 | 5.068070754 | 7.959549324 | 1.41E-14 |
| LRRC42 | 0.318527548 | 4.582320081 | 7.958664092 | 1.42E-14 |
| ANKS3 | -0.424902891 | 2.477661117 | -7.956201144 | 1.44E-14 |
| TRIM66 | -0.546374001 | 4.01111094 | -7.955707501 | 1.45E-14 |
| RP11-613F22.6 | 0.870975457 | 1.905962032 | 7.955495654 | 1.45E-14 |
| FLII | -0.293781594 | 7.526742118 | -7.95411985 | 1.46E-14 |
| KBTBD8 | 1.002767258 | 3.678901297 | 7.953859533 | 1.47E-14 |
| LINC00324 | -0.941449651 | 2.055071245 | -7.951372602 | 1.49E-14 |
| ARL6 | -0.586638324 | 1.502259518 | -7.950237613 | 1.51E-14 |
| NUF2 | 0.892491153 | 3.297991372 | 7.948557892 | 1.52E-14 |
| KDM6A | 0.419660307 | 5.601872831 | 7.945430654 | 1.56E-14 |
| RP11-216N14.7 | 0.79070721 | 1.447743983 | 7.945064174 | 1.56E-14 |
| RP11-1012A1.7 | 0.487098981 | 3.405200628 | 7.942683834 | 1.59E-14 |
| TOB1-AS1 | -0.778185329 | 0.674600769 | -7.941421023 | 1.60E-14 |
| PI4K2A | 1.059286469 | 3.420114203 | 7.941317033 | 1.60E-14 |
| GAPVD1 | 0.264696113 | 5.155256419 | 7.939323759 | 1.63E-14 |
| NAMPTL | 1.240222911 | 7.372543635 | 7.935366561 | 1.67E-14 |
| METTL3 | -0.410490954 | 5.628289756 | -7.933728187 | 1.69E-14 |
| SPATA41 | -1.004615244 | 0.0815745 | -7.933415727 | 1.70E-14 |
| RPS6KA3 | -0.421104492 | 6.666940454 | -7.931447589 | 1.72E-14 |
| RP11-760H22.2 | -1.194695598 | 2.472687067 | -7.931245722 | 1.72E-14 |
| AC104651.1 | -0.98947685 | -0.380263118 | -7.929427401 | 1.74E-14 |
| STXBP3 | -0.300904499 | 5.840611678 | -7.92829875 | 1.76E-14 |
| CTD-2006C1.12 | -0.716060802 | 2.212418929 | -7.924029434 | 1.81E-14 |
| SELK | 0.539430824 | 5.539455888 | 7.923513573 | 1.82E-14 |
| DSTYK | 0.37209229 | 4.933078867 | 7.917996153 | 1.89E-14 |
| POLR2J4 | 0.823669716 | 1.668462657 | 7.917631703 | 1.90E-14 |
| RP11-197P3.5 | 0.777492582 | 2.131133737 | 7.916153561 | 1.92E-14 |
| AREL1 | 0.356627778 | 5.405234923 | 7.916023175 | 1.92E-14 |
| SAMD8 | 0.620698376 | 4.823648513 | 7.91578879 | 1.92E-14 |
| DPYD | -0.814825085 | 5.128717853 | -7.915233006 | 1.93E-14 |
| RP4-657D16.3 | 0.889968565 | 2.742111003 | 7.912848341 | 1.96E-14 |
| LMBRD2 | -0.404564434 | 3.883762461 | -7.91272337 | 1.96E-14 |
| C9orf114 | -0.327253501 | 5.105708186 | -7.909364179 | 2.01E-14 |
| KIF14 | 0.973044107 | 2.556811688 | 7.909231155 | 2.01E-14 |
| IRAK4 | -0.295984341 | 5.516018588 | -7.9088963 | 2.02E-14 |
| PSMA4 | -0.264199006 | 6.932478942 | -7.907682468 | 2.03E-14 |
| IQCC | 0.525054357 | 2.597898796 | 7.906926703 | 2.04E-14 |
| RP11-24B19.3 | -0.587055764 | 4.102835577 | -7.906142792 | 2.06E-14 |
| GEMIN5 | -0.583138886 | 3.834588904 | -7.903421439 | 2.10E-14 |
| RP11-1079K10.2 | -0.879345582 | 0.736693546 | -7.901009254 | 2.13E-14 |
| ERVK3-1 | 0.599934418 | 5.101414859 | 7.899634297 | 2.15E-14 |
| ILKAP | 0.432845118 | 5.09484129 | 7.899155688 | 2.16E-14 |
| CFL2 | 0.700118726 | 4.469859195 | 7.898550636 | 2.17E-14 |
| TMEM230 | -0.36372544 | 7.888549958 | -7.898357698 | 2.17E-14 |
| TPCN2 | -0.399604641 | 4.65600884 | -7.898084388 | 2.18E-14 |
| RAB3D | -0.622266668 | 6.360952023 | -7.895954883 | 2.21E-14 |
| SEC24B | 0.310190888 | 5.902837694 | 7.894938262 | 2.23E-14 |
| AC009948.5 | -0.574637612 | 4.635146487 | -7.894227779 | 2.24E-14 |
| EFNA4 | -0.657907589 | 2.76092693 | -7.888783649 | 2.32E-14 |
| DISC1 | -0.599898952 | 3.307636625 | -7.888261005 | 2.33E-14 |
| ZBTB39 | 0.515010003 | 4.459757788 | 7.888208965 | 2.33E-14 |
| KHSRPP1 | 0.360880641 | 3.479341731 | 7.886619072 | 2.36E-14 |
| SLC25A46 | -0.315769188 | 5.662642972 | -7.885536397 | 2.38E-14 |
| PRPF40A | 0.181128032 | 6.752245065 | 7.884991433 | 2.39E-14 |
| RP11-707M3.3 | -0.992190877 | 1.802211051 | -7.884786921 | 2.39E-14 |
| ARL8A | 0.45523612 | 7.427054431 | 7.884328675 | 2.40E-14 |
| GRHL1 | 1.152696825 | -1.617826555 | 7.882636538 | 2.43E-14 |
| MRPS15 | 0.298431119 | 5.808586861 | 7.881873667 | 2.44E-14 |
| RP11-452F19.3 | 0.495093379 | 3.635441176 | 7.880654051 | 2.46E-14 |
| USP47 | 0.229513145 | 5.472904124 | 7.878832845 | 2.49E-14 |
| CTC-444N24.8 | 0.504483952 | 3.412418794 | 7.877859943 | 2.51E-14 |
| EEA1 | -0.522741477 | 4.749050105 | -7.876181468 | 2.54E-14 |
| DNAJC28 | -0.78089575 | 1.268680895 | -7.876153299 | 2.54E-14 |
| AHSA1 | -0.463147597 | 6.830638532 | -7.874506221 | 2.57E-14 |
| SRBD1 | -0.451781435 | 4.984243462 | -7.871455015 | 2.63E-14 |
| FAM178A | -0.390326979 | 4.693549846 | -7.870351814 | 2.65E-14 |
| PAPSS1 | -0.47839309 | 6.274035121 | -7.868750921 | 2.68E-14 |
| PDCD6 | -0.232658038 | 4.772681606 | -7.868336343 | 2.68E-14 |
| SNRPA1 | 0.65209678 | 5.231176981 | 7.86784884 | 2.69E-14 |
| CEP89 | -0.479218083 | 3.566538219 | -7.86726098 | 2.70E-14 |
| SEMA3F | 1.51385445 | -0.27340969 | 7.866684388 | 2.71E-14 |
| FBRS | 0.366998699 | 7.178194983 | 7.865006325 | 2.75E-14 |
| HBP1 | 0.512452426 | 5.546529957 | 7.863198091 | 2.78E-14 |
| PCMTD2 | -0.378477927 | 5.723607543 | -7.862066107 | 2.80E-14 |
| RP11-722E23.2 | -1.025182148 | 0.986603367 | -7.86052658 | 2.83E-14 |
| APRG1 | 0.842591473 | 1.091715385 | 7.860113305 | 2.84E-14 |
| PIEZO1 | -0.500001058 | 7.488027824 | -7.85868449 | 2.87E-14 |
| TMEM167B | 0.40067038 | 6.303826098 | 7.854281126 | 2.96E-14 |
| SNAP23 | -0.333351651 | 6.129909706 | -7.852496521 | 3.00E-14 |
| GLTSCR1L | -0.331825557 | 5.480410041 | -7.852385426 | 3.00E-14 |
| STT3A | -0.605649109 | 5.774245329 | -7.850457744 | 3.04E-14 |
| LIN7C | 0.345704148 | 5.569403507 | 7.849575554 | 3.06E-14 |
| CDK7 | 0.25870131 | 5.068163252 | 7.84919404 | 3.07E-14 |
| AC005822.1 | -0.43006348 | 6.474080612 | -7.849097112 | 3.07E-14 |
| SLC25A17 | -0.398401401 | 3.242656584 | -7.845977858 | 3.14E-14 |
| RBM14 | -0.433689637 | 6.394140351 | -7.844709688 | 3.17E-14 |
| NRD1 | -0.332123045 | 7.270881916 | -7.843047171 | 3.20E-14 |
| TMEM80 | -0.398576493 | 3.937572878 | -7.842081136 | 3.23E-14 |
| RMI1 | -0.679080399 | 3.841254152 | -7.842059769 | 3.23E-14 |
| CHMP4B | 0.371792773 | 8.796315902 | 7.839312622 | 3.29E-14 |
| RP11-104G3.2 | -1.010089104 | -0.544341663 | -7.838205951 | 3.32E-14 |
| RP11-519G16.3 | 1.452301188 | -2.66453338 | 7.837609604 | 3.33E-14 |
| RHOT1 | -0.312814498 | 5.515551054 | -7.8369743 | 3.34E-14 |
| AC006026.10 | -0.420036606 | 2.883536013 | -7.835516145 | 3.38E-14 |
| DNAJA3 | -0.482012204 | 4.300281628 | -7.835197027 | 3.39E-14 |
| GPR55 | -1.042893553 | 1.083140413 | -7.834321266 | 3.41E-14 |
| TMEM150B | -1.29579195 | 2.721976155 | -7.833478151 | 3.43E-14 |
| TMEM41B | 0.449831016 | 4.366983341 | 7.832906991 | 3.44E-14 |
| CTD-2199O4.6 | -0.893170308 | 0.693346614 | -7.830904183 | 3.49E-14 |
| FAM220A | -0.435119765 | 4.510923857 | -7.827936711 | 3.56E-14 |
| RP11-298I3.1 | -0.959740586 | -0.261634997 | -7.827603962 | 3.57E-14 |
| CASP1 | -0.826556289 | 6.064049461 | -7.827470419 | 3.57E-14 |
| SCML2P2 | -1.127006196 | 2.160048632 | -7.826987676 | 3.59E-14 |
| MIR553 | 0.781277889 | 1.871893876 | 7.825632381 | 3.62E-14 |
| ZNF790 | -0.612368055 | 1.714642842 | -7.825279608 | 3.63E-14 |
| FTSJ3 | -0.341356359 | 5.435748691 | -7.824963142 | 3.64E-14 |
| TYSND1 | -0.487110463 | 3.696384896 | -7.823929371 | 3.66E-14 |
| DNAJB5 | 1.185400604 | -0.021794793 | 7.823029665 | 3.69E-14 |
| SNRPB | 0.362818054 | 7.354021461 | 7.820677609 | 3.75E-14 |
| CIDECP | 0.467832301 | 4.147664081 | 7.820636762 | 3.75E-14 |
| RP11-115C21.2 | -0.554024869 | 2.015720934 | -7.820337622 | 3.76E-14 |
| C10orf76 | -0.218483709 | 5.31825334 | -7.819434794 | 3.78E-14 |
| POLR3A | -0.356063842 | 4.155074027 | -7.81926785 | 3.79E-14 |
| NISCH | -0.268098123 | 6.114187185 | -7.817268019 | 3.84E-14 |
| GMPPA | -0.316613365 | 4.308754759 | -7.816369464 | 3.86E-14 |
| RP11-1020A11.2 | -1.14418372 | -1.038584657 | -7.815305048 | 3.89E-14 |
| RAB21 | 0.339137763 | 5.850621345 | 7.813354319 | 3.94E-14 |
| PPM1D | 0.352013251 | 5.503875268 | 7.812211084 | 3.98E-14 |
| ZNF35 | 0.557712873 | 3.719397419 | 7.811480174 | 4.00E-14 |
| UCN | -0.647674667 | 3.527260286 | -7.80994023 | 4.04E-14 |
| ING3 | 0.457160445 | 5.098360508 | 7.808329897 | 4.09E-14 |
| BRF2 | 0.379791546 | 4.363118971 | 7.808104005 | 4.09E-14 |
| NUDT4 | 0.490532419 | 5.249730537 | 7.807676648 | 4.10E-14 |
| POP1 | -0.584252009 | 2.183602694 | -7.803919943 | 4.21E-14 |
| CXorf40B | 0.408810662 | 5.354545994 | 7.803338471 | 4.23E-14 |
| NAGK | -0.504978451 | 5.257224645 | -7.798380829 | 4.38E-14 |
| POLR3H | -0.458670449 | 4.215532771 | -7.798008328 | 4.39E-14 |
| TAB3 | 0.285974852 | 5.269500425 | 7.797030191 | 4.42E-14 |
| LRRC63 | -1.061685506 | 1.831275211 | -7.795586059 | 4.47E-14 |
| GTF2E2 | -0.299076665 | 5.093631138 | -7.79417317 | 4.51E-14 |
| RP11-169K17.4 | 0.912567335 | -0.614852984 | 7.793390708 | 4.54E-14 |
| RP3-355L5.5 | -0.872351681 | 0.139296087 | -7.791997609 | 4.58E-14 |
| RP5-1024G6.2 | -0.775919348 | 0.093915527 | -7.791935001 | 4.58E-14 |
| CTD-2095E4.1 | 0.519888573 | 2.419360301 | 7.791578624 | 4.59E-14 |
| PHLPP1 | 0.524517099 | 4.292114089 | 7.791528292 | 4.59E-14 |
| AFF4 | 0.41626766 | 6.085368329 | 7.79131114 | 4.60E-14 |
| TOM1L2 | -0.332334756 | 4.199251689 | -7.790209593 | 4.64E-14 |
| ZNF609 | -0.572015526 | 6.075327352 | -7.788609027 | 4.69E-14 |
| AC007229.3 | -0.630382144 | 2.135411481 | -7.788026432 | 4.71E-14 |
| RNF168 | 0.542686498 | 5.809161685 | 7.786097555 | 4.77E-14 |
| RP11-737O24.5 | -0.900247326 | 2.780052443 | -7.785816732 | 4.78E-14 |
| SMAD7 | 1.121604902 | 3.418089845 | 7.785728422 | 4.78E-14 |
| SKP1P1 | -0.333258238 | 5.643643471 | -7.785409605 | 4.79E-14 |
| SPHK1 | 0.947647258 | 2.552037511 | 7.784645116 | 4.82E-14 |
| AP001469.7 | -0.944660581 | 0.778269547 | -7.784606605 | 4.82E-14 |
| GTF2H5 | -0.378151443 | 2.787808619 | -7.784096537 | 4.84E-14 |
| HNRNPUL1 | 0.25075109 | 8.722605815 | 7.781654027 | 4.92E-14 |
| SETD2 | 0.194301802 | 6.647483382 | 7.7814812 | 4.93E-14 |
| GFER | 0.249132937 | 4.90847328 | 7.778116017 | 5.05E-14 |
| HN1L | -0.643688151 | 5.191923388 | -7.77682857 | 5.09E-14 |
| LINC00910 | -0.661843919 | 1.207105535 | -7.773458465 | 5.21E-14 |
| ZNF304 | 0.501380668 | 3.880129391 | 7.769469414 | 5.36E-14 |
| ZNF281 | 0.559874756 | 6.024003711 | 7.768732529 | 5.39E-14 |
| UBXN2A | 0.363528741 | 4.304049471 | 7.76872184 | 5.39E-14 |
| TIMP2 | -0.593849456 | 7.036687104 | -7.766658816 | 5.46E-14 |
| AFG3L2 | 0.32599243 | 5.637498669 | 7.766654991 | 5.46E-14 |
| RP11-130L8.1 | -0.821713693 | 1.520887946 | -7.76300978 | 5.60E-14 |
| CYSLTR1 | -0.731537943 | 4.221063605 | -7.762792135 | 5.61E-14 |
| DPP9 | 0.206027726 | 5.37334274 | 7.76245241 | 5.63E-14 |
| HIC1 | 1.21163664 | 2.756228014 | 7.760372204 | 5.71E-14 |
| ATP1A1OS | -0.677404951 | 1.232795321 | -7.758740693 | 5.77E-14 |
| TWF2 | -0.356539661 | 5.535388294 | -7.755804617 | 5.89E-14 |
| SSBP3-AS1 | 0.984161331 | -0.158726077 | 7.754460241 | 5.95E-14 |
| SF1 | 0.324822069 | 8.705351559 | 7.751068269 | 6.09E-14 |
| POLR2M | 0.283761917 | 3.657535554 | 7.750727548 | 6.10E-14 |
| CTC-503J8.6 | -0.391198507 | 6.573762585 | -7.750135865 | 6.13E-14 |
| RP11-206F17.2 | -0.292151321 | 5.895388674 | -7.750095666 | 6.13E-14 |
| CALCOCO1 | -0.372488881 | 6.230443531 | -7.744055093 | 6.39E-14 |
| ETF1 | 0.477442633 | 7.351367012 | 7.743757479 | 6.41E-14 |
| HSD17B7 | -0.509685157 | 2.709672245 | -7.74275574 | 6.45E-14 |
| ASPM | 1.053656518 | 3.199838536 | 7.742050817 | 6.48E-14 |
| CCNC | 0.273833294 | 5.095641154 | 7.739537357 | 6.60E-14 |
| KIAA0247 | 0.512670902 | 6.580317744 | 7.738127944 | 6.66E-14 |
| AZIN1 | 0.344763211 | 7.179939052 | 7.737595727 | 6.69E-14 |
| SCARF2 | 0.791856806 | 1.529301863 | 7.735293742 | 6.80E-14 |
| AC007365.3 | -0.98222729 | 0.566297336 | -7.731294045 | 6.99E-14 |
| STRAP | 0.373139904 | 8.007275465 | 7.729795351 | 7.06E-14 |
| PHLDA1 | 1.576696477 | 3.168280175 | 7.727843427 | 7.16E-14 |
| IL11 | 1.230183343 | -1.741733435 | 7.723557899 | 7.37E-14 |
| NBL1 | 1.427993055 | 0.07048868 | 7.722665131 | 7.42E-14 |
| LSM12 | 0.348276844 | 3.669136876 | 7.722444149 | 7.43E-14 |
| PPP1R2 | 0.565997942 | 6.28273181 | 7.719319051 | 7.59E-14 |
| MCEE | -0.350584821 | 4.22839969 | -7.719001671 | 7.61E-14 |
| C16orf62 | -0.337962745 | 4.729348032 | -7.717850367 | 7.67E-14 |
| ZNF572 | -0.852778609 | 1.029314725 | -7.714903229 | 7.83E-14 |
| CTC-308K20.4 | 0.860195911 | 0.738974654 | 7.71275305 | 7.94E-14 |
| PDS5A | -0.327934574 | 6.648603905 | -7.712725561 | 7.95E-14 |
| GCNT1 | -0.82220307 | 5.124486562 | -7.712330549 | 7.97E-14 |
| RP5-1086K13.1 | -0.89684114 | 2.364129298 | -7.712305527 | 7.97E-14 |
| CNTF | 0.736238044 | 2.118270264 | 7.712172931 | 7.98E-14 |
| NAP1L5 | 0.684207826 | 4.132632923 | 7.710783856 | 8.05E-14 |
| TAS2R6 | -0.972509936 | -0.010933765 | -7.709682817 | 8.12E-14 |
| FITM2 | -0.521120181 | 1.803236361 | -7.708178406 | 8.20E-14 |
| CHMP2B | 0.406977337 | 6.291714954 | 7.707468098 | 8.24E-14 |
| LRIF1 | -0.704448684 | 4.215642389 | -7.705361273 | 8.36E-14 |
| PCID2 | -0.302734193 | 4.821794006 | -7.70495548 | 8.39E-14 |
| RIMBP3C | -1.193956705 | -3.922707774 | -7.703839681 | 8.45E-14 |
| SLC27A3 | -0.618649487 | 4.611042573 | -7.703117448 | 8.49E-14 |
| RP11-179B2.2 | -0.88915237 | -0.940009922 | -7.702842192 | 8.51E-14 |
| RHBDD2 | 0.54955468 | 6.135423218 | 7.702708112 | 8.52E-14 |
| MCCC2 | -0.419867326 | 5.088968923 | -7.702202286 | 8.55E-14 |
| RNASEK | 0.597545995 | 4.358252248 | 7.701856763 | 8.57E-14 |
| SPC24 | 1.012422916 | 3.017351652 | 7.701178789 | 8.61E-14 |
| DAZAP2 | 0.371601505 | 8.860365446 | 7.698379436 | 8.78E-14 |
| PAPD5 | 0.508676467 | 4.392238845 | 7.698302056 | 8.78E-14 |
| GPATCH3 | 0.29313449 | 5.282506381 | 7.69800142 | 8.80E-14 |
| RP13-131K19.6 | 0.646079687 | 2.592327298 | 7.697876915 | 8.81E-14 |
| SLC24A1 | -0.611987982 | 3.248463135 | -7.697712585 | 8.82E-14 |
| DCST1 | 1.082029035 | -2.608425823 | 7.697503961 | 8.83E-14 |
| SDC2 | 1.949980167 | -0.169962627 | 7.697181757 | 8.85E-14 |
| SIAH2 | 0.433328948 | 6.659340967 | 7.696425954 | 8.89E-14 |
| ZNF280D | -0.336704455 | 4.690813982 | -7.694974082 | 8.98E-14 |
| RP11-158K1.3 | -0.663917043 | 1.37222188 | -7.694795878 | 9.00E-14 |
| ARV1 | -0.69099356 | 4.458833762 | -7.691024009 | 9.23E-14 |
| GJB6 | 1.939214252 | 0.73768515 | 7.69079007 | 9.25E-14 |
| ENDOU | 1.303380209 | -0.904941091 | 7.687987119 | 9.43E-14 |
| HSCB | -0.363601545 | 4.328591223 | -7.687064717 | 9.49E-14 |
| AC073052.1 | 0.98550808 | 0.921019223 | 7.686144822 | 9.55E-14 |
| CDCA2 | 1.033363654 | 2.404643217 | 7.685937351 | 9.56E-14 |
| ARF1 | 0.253480346 | 9.384582728 | 7.684086908 | 9.69E-14 |
| NCBP2-AS1 | 0.85572885 | 2.23321582 | 7.682873625 | 9.77E-14 |
| CHRAC1 | 0.293730569 | 6.289787172 | 7.68281754 | 9.77E-14 |
| LHX4 | 1.139673157 | -3.424079965 | 7.682642136 | 9.78E-14 |
| DOCK2 | -0.307779833 | 7.371996137 | -7.682267518 | 9.81E-14 |
| RP11-403I13.5 | -0.919100263 | -0.286739757 | -7.682076211 | 9.82E-14 |
| SERTAD2 | 0.568837025 | 7.082281002 | 7.678030301 | 1.01E-13 |
| PLK1 | 0.961812323 | 3.763845058 | 7.677955303 | 1.01E-13 |
| RP11-707G14.7 | -0.839039053 | 0.248871886 | -7.677002504 | 1.02E-13 |
| UBE2V2 | -0.448003031 | 4.947334492 | -7.6768847 | 1.02E-13 |
| PRICKLE3 | -0.484565025 | 3.760214783 | -7.676128027 | 1.02E-13 |
| CTD-2310F14.1 | -0.860113456 | -0.422148819 | -7.675528983 | 1.03E-13 |
| RP11-108L7.4 | -0.943840117 | 0.155413733 | -7.67415466 | 1.04E-13 |
| ZNF212 | 0.480083768 | 4.329654493 | 7.672553748 | 1.05E-13 |
| RSRC2 | 0.417612173 | 6.78940009 | 7.670965939 | 1.06E-13 |
| TTC31 | -0.243344428 | 5.287837727 | -7.663833224 | 1.11E-13 |
| CAMTA1 | 0.372669769 | 3.24215089 | 7.661236546 | 1.13E-13 |
| SLC41A1 | 1.065805898 | 3.092596753 | 7.66108508 | 1.14E-13 |
| RP4-647C14.2 | -1.348438233 | 0.45763811 | -7.661024477 | 1.14E-13 |
| CDC42EP4 | 0.922724065 | 3.513181741 | 7.660443444 | 1.14E-13 |
| KIAA1919 | -0.421390026 | 3.112813847 | -7.660287265 | 1.14E-13 |
| CDK1 | 1.085301118 | 3.468005941 | 7.659492858 | 1.15E-13 |
| FOXK1 | 0.346326056 | 4.955649476 | 7.65875996 | 1.15E-13 |
| PTER | -0.400081403 | 4.203190663 | -7.657290713 | 1.17E-13 |
| RBFADN | -1.02867593 | -0.927361216 | -7.656486491 | 1.17E-13 |
| RP11-355F16.1 | -0.89555533 | -2.364031956 | -7.655686002 | 1.18E-13 |
| CASC5 | 0.815656733 | 3.271756022 | 7.655291912 | 1.18E-13 |
| C9orf64 | -0.943013424 | 2.862210003 | -7.655171151 | 1.18E-13 |
| DUSP22 | -0.412308284 | 5.474008621 | -7.654734302 | 1.19E-13 |
| LMLN | -0.745259175 | 2.019794681 | -7.653455253 | 1.20E-13 |
| CD83 | 1.26302139 | 6.545659572 | 7.653031357 | 1.20E-13 |
| AGGF1P2 | -1.042994895 | -0.474938453 | -7.651834719 | 1.21E-13 |
| SMIM11 | -0.383872049 | 3.412121726 | -7.651653151 | 1.21E-13 |
| MESDC2 | -0.42970738 | 5.012105243 | -7.650637041 | 1.22E-13 |
| C19orf25 | -0.376639081 | 3.596801422 | -7.650265321 | 1.22E-13 |
| ASCL2 | -1.016312244 | 3.359846383 | -7.647616982 | 1.25E-13 |
| FAM86A | -0.62335978 | 2.657657008 | -7.647532201 | 1.25E-13 |
| RP11-66B24.4 | -0.987716571 | 0.373554367 | -7.647309045 | 1.25E-13 |
| SPAG5-AS1 | 0.750816624 | 0.675091052 | 7.645840024 | 1.26E-13 |
| PHF5A | 0.235626445 | 6.862477622 | 7.645571065 | 1.26E-13 |
| ZNF575 | -0.505928345 | 1.603178693 | -7.64404515 | 1.28E-13 |
| POLR3F | 0.270828557 | 4.614228312 | 7.643884318 | 1.28E-13 |
| LONRF1 | 0.498377905 | 4.489197899 | 7.643561657 | 1.28E-13 |
| TRMT12 | -0.392910681 | 4.668140433 | -7.642836274 | 1.29E-13 |
| NUDT16 | -0.553038285 | 5.406801863 | -7.641725733 | 1.30E-13 |
| EPRS | -0.367372708 | 7.03962546 | -7.640919414 | 1.30E-13 |
| C9orf78 | 0.285125322 | 6.840300053 | 7.640346144 | 1.31E-13 |
| RAB5C | 0.292776205 | 6.968023556 | 7.639008577 | 1.32E-13 |
| NINJ1 | 1.079370237 | 6.730415551 | 7.638763531 | 1.32E-13 |
| MYO9A | -0.324125217 | 4.442481808 | -7.636788827 | 1.34E-13 |
| TSR2 | 0.217687609 | 6.66595163 | 7.635356644 | 1.36E-13 |
| ZNF322 | -0.724161921 | 2.124744417 | -7.633166114 | 1.38E-13 |
| HIBADH | -0.44875769 | 5.474543817 | -7.629930953 | 1.41E-13 |
| RP13-131K19.7 | -0.803835033 | 0.947265845 | -7.624861899 | 1.46E-13 |
| RSAD1 | -0.389666698 | 5.246405986 | -7.624696837 | 1.46E-13 |
| DNAJC27-AS1 | -0.787655307 | -1.008354979 | -7.62441538 | 1.46E-13 |
| DMRTC2 | 1.221767904 | -2.998783513 | 7.62414491 | 1.46E-13 |
| EMC3-AS1 | 0.51098865 | 3.563354025 | 7.624014843 | 1.46E-13 |
| SCYL3 | -0.367683416 | 3.781071295 | -7.623878122 | 1.47E-13 |
| SIVA1 | 0.434880756 | 5.252549794 | 7.623621449 | 1.47E-13 |
| ZNF484 | 0.440490035 | 3.708364988 | 7.622970442 | 1.48E-13 |
| RAB8B | 0.548780181 | 6.379796752 | 7.621166831 | 1.49E-13 |
| FAR2 | -0.655294888 | 5.004195059 | -7.620402676 | 1.50E-13 |
| RP11-63P12.6 | -1.188141671 | 1.049231887 | -7.620164788 | 1.50E-13 |
| NOC3L | -0.466597076 | 3.557373181 | -7.619936749 | 1.51E-13 |
| SUPT6H | 0.266261897 | 6.820094774 | 7.618343198 | 1.52E-13 |
| RP11-219E7.1 | -1.499616452 | 1.379558537 | -7.617335494 | 1.53E-13 |
| RBM8A | -0.26665493 | 6.396082941 | -7.616636874 | 1.54E-13 |
| RP11-384B12.3 | 0.307606655 | 3.238720231 | 7.616608668 | 1.54E-13 |
| C2orf69 | 0.501936927 | 4.887131148 | 7.616555587 | 1.54E-13 |
| CBX1P1 | -0.769215748 | 1.774084004 | -7.616409631 | 1.54E-13 |
| USP40 | -0.559280207 | 3.741923969 | -7.616195676 | 1.55E-13 |
| RPUSD3 | -0.537890038 | 4.073378908 | -7.616077275 | 1.55E-13 |
| ZNF845 | 0.47048707 | 3.705345025 | 7.611539236 | 1.60E-13 |
| PSMA6 | 0.442399255 | 3.801624936 | 7.610122418 | 1.61E-13 |
| ZNF585A | -0.463038471 | 1.6783556 | -7.609237919 | 1.62E-13 |
| MIR3128 | 0.884393802 | 1.333377864 | 7.607456315 | 1.64E-13 |
| ABCC5 | -0.559025669 | 4.40803646 | -7.606187866 | 1.66E-13 |
| CTD-3214H19.6 | -0.679790422 | 2.787628998 | -7.606020346 | 1.66E-13 |
| TBX15 | 1.074538852 | -2.912602179 | 7.604324177 | 1.68E-13 |
| RGPD6 | 0.799093142 | -0.388828921 | 7.601541355 | 1.71E-13 |
| EDEM2 | -0.285429196 | 6.019035758 | -7.600577135 | 1.72E-13 |
| RP11-244F12.1 | 0.574441036 | 2.225232359 | 7.60054264 | 1.72E-13 |
| PNPLA8 | 0.547809087 | 5.35350346 | 7.600416429 | 1.72E-13 |
| CUL5 | 0.201495313 | 5.444354339 | 7.598813083 | 1.74E-13 |
| TOR1A | -0.378730866 | 6.308615488 | -7.59781546 | 1.75E-13 |
| C1GALT1 | 0.336916832 | 5.117461037 | 7.595520384 | 1.78E-13 |
| ISCA1 | 0.525235962 | 5.562315507 | 7.593790413 | 1.80E-13 |
| ALG8 | -0.456662137 | 4.704590876 | -7.591382565 | 1.83E-13 |
| WBSCR22 | 0.393017873 | 4.925453521 | 7.590748679 | 1.84E-13 |
| RP11-459F6.3 | -0.922432335 | -0.492774917 | -7.590722722 | 1.84E-13 |
| PBX4 | 1.001754644 | 2.046956396 | 7.588799804 | 1.86E-13 |
| CHMP4A | 0.479808135 | 2.960783965 | 7.587839735 | 1.88E-13 |
| RPS20P22 | 1.355578078 | -0.237045594 | 7.5873044 | 1.88E-13 |
| RP11-356M20.1 | -1.657312609 | 0.423317128 | -7.587022334 | 1.89E-13 |
| PHACTR1 | 0.649656991 | 3.944102935 | 7.585596479 | 1.91E-13 |
| ORMDL1 | -0.314374867 | 6.684671366 | -7.584960689 | 1.91E-13 |
| CTB-89H12.4 | 0.632360691 | 4.173276297 | 7.584891355 | 1.92E-13 |
| AC083799.1 | 0.474648806 | 6.065893285 | 7.58411862 | 1.93E-13 |
| AC006116.27 | -0.891418325 | -0.60008844 | -7.58377387 | 1.93E-13 |
| CTD-2647E9.1 | 0.820567782 | -0.666253535 | 7.582998512 | 1.94E-13 |
| CTD-2270L9.4 | -0.726222041 | 1.617310416 | -7.582735382 | 1.94E-13 |
| TADA2B | 0.700212567 | 4.725834792 | 7.579928768 | 1.98E-13 |
| RP5-1096D14.3 | -0.959768072 | 0.438667094 | -7.578831225 | 2.00E-13 |
| CCDC97 | -0.324926384 | 6.249233501 | -7.578663093 | 2.00E-13 |
| RP11-421L21.3 | -0.690627181 | 2.791149302 | -7.577631864 | 2.01E-13 |
| SEZ6 | 1.1114933 | -3.385260599 | 7.577321274 | 2.02E-13 |
| WAC | 0.205665241 | 6.951957005 | 7.577079315 | 2.02E-13 |
| POLG | -0.251002802 | 6.300184134 | -7.576348819 | 2.03E-13 |
| BX322557.10 | -0.600301284 | 4.082324561 | -7.575208072 | 2.05E-13 |
| PPP1R16B | 1.253151543 | 4.89419214 | 7.575110945 | 2.05E-13 |
| ZNF524 | -0.344299639 | 5.334601189 | -7.574940215 | 2.05E-13 |
| RNF138P1 | 0.48632363 | 3.439375741 | 7.574614707 | 2.05E-13 |
| TFAP4 | -0.60175093 | 2.628265236 | -7.574311155 | 2.06E-13 |
| APEH | -0.402958194 | 6.282966412 | -7.573115745 | 2.08E-13 |
| UTP14C | -0.534021586 | 3.642817008 | -7.572739945 | 2.08E-13 |
| FLT3 | -1.224188388 | 7.789078842 | -7.571585252 | 2.10E-13 |
| OSTF1 | -0.560059273 | 7.712044107 | -7.571380075 | 2.10E-13 |
| TRIM52 | -0.308586196 | 5.112754713 | -7.571180836 | 2.10E-13 |
| CPPED1 | -0.692856238 | 6.731354234 | -7.570785008 | 2.11E-13 |
| ENSA | 0.230851294 | 6.850425584 | 7.566311165 | 2.17E-13 |
| PYGB | -0.369149548 | 5.945684904 | -7.566305899 | 2.17E-13 |
| KCNQ4 | 0.807365391 | -0.710377221 | 7.564931785 | 2.20E-13 |
| PRRT3-AS1 | -0.996100147 | 0.878057035 | -7.564812361 | 2.20E-13 |
| NDUFA9 | 0.357392495 | 2.334791612 | 7.564384791 | 2.20E-13 |
| SCAMP1 | -0.35383995 | 4.830487901 | -7.564010745 | 2.21E-13 |
| DTWD2 | -0.57579526 | 2.647382926 | -7.561398424 | 2.25E-13 |
| LRRC40 | 0.248992944 | 5.509249631 | 7.55836736 | 2.30E-13 |
| SPDYA | 0.834558254 | 1.122255489 | 7.557558553 | 2.31E-13 |
| GIMAP8 | -1.480150251 | 3.265106833 | -7.556698221 | 2.32E-13 |
| RP11-686D22.7 | -0.981871741 | 3.053335109 | -7.555612772 | 2.34E-13 |
| PRDM10 | 0.282641087 | 3.770654713 | 7.554307087 | 2.36E-13 |
| RP11-111K18.2 | -0.80982079 | -1.080293432 | -7.553144653 | 2.38E-13 |
| ZDBF2 | 1.156289433 | -0.282384245 | 7.5528419 | 2.38E-13 |
| STK16 | 0.352124402 | 4.954297019 | 7.551498115 | 2.41E-13 |
| AHSA2 | -0.449448067 | 6.326449212 | -7.550890462 | 2.42E-13 |
| ZNF470 | -0.724926584 | 1.202043103 | -7.550445174 | 2.42E-13 |
| BAG5 | 0.311862146 | 6.005095284 | 7.548798144 | 2.45E-13 |
| TMEM229B | -0.785674989 | 2.721166434 | -7.54741987 | 2.47E-13 |
| C6orf211 | -0.487142937 | 4.603329553 | -7.547203516 | 2.48E-13 |
| PFN2 | 1.092741571 | -0.000972235 | 7.546611943 | 2.49E-13 |
| WDR20 | 0.292658871 | 3.844291657 | 7.546074657 | 2.50E-13 |
| OASL | 1.379494372 | 3.654067233 | 7.545560635 | 2.51E-13 |
| WDTC1 | -0.301789339 | 6.520086626 | -7.544926116 | 2.52E-13 |
| ZNF511 | 0.308638924 | 4.973438492 | 7.544683943 | 2.52E-13 |
| RP3-412A9.16 | -0.812368478 | 0.775164424 | -7.542572334 | 2.56E-13 |
| PPP1R7 | -0.243140564 | 5.702740643 | -7.542043211 | 2.57E-13 |
| PTPN2P1 | -0.363229196 | 3.670474507 | -7.540232058 | 2.60E-13 |
| DACT3 | 1.341870417 | -1.68023425 | 7.540213443 | 2.60E-13 |
| VRK1 | 0.382223095 | 5.754854926 | 7.539939851 | 2.60E-13 |
| E2F8 | 1.074512785 | 2.854972294 | 7.539627451 | 2.61E-13 |
| STX6 | -0.381979444 | 4.639638532 | -7.538285612 | 2.63E-13 |
| HMGA1 | 0.565935903 | 9.44051227 | 7.536730778 | 2.66E-13 |
| BROX | 0.263174353 | 5.872107857 | 7.53518838 | 2.69E-13 |
| GIMAP6 | -1.115053702 | 4.809720022 | -7.533299732 | 2.72E-13 |
| GLB1 | -0.405886326 | 6.323604492 | -7.532314885 | 2.74E-13 |
| RP11-398C13.6 | -0.792067128 | 1.381693077 | -7.529752786 | 2.79E-13 |
| BCAT2 | -0.468865545 | 3.466262168 | -7.529337817 | 2.80E-13 |
| STK17A | 0.595653972 | 5.063822793 | 7.528126279 | 2.82E-13 |
| VCPIP1 | 0.432566883 | 5.6542005 | 7.526382521 | 2.85E-13 |
| SLC45A3 | -1.127111411 | 5.100711382 | -7.524244779 | 2.90E-13 |
| ICOSLG | 1.018487072 | 3.66174689 | 7.521490602 | 2.95E-13 |
| SNORA54 | 0.820399191 | 1.002793466 | 7.517504848 | 3.03E-13 |
| ZNF236 | 0.22456818 | 4.136639991 | 7.516333327 | 3.06E-13 |
| FAM186B | 1.032942768 | -2.529503971 | 7.515587767 | 3.07E-13 |
| ZNF430 | 0.325223868 | 4.218852532 | 7.515039545 | 3.08E-13 |
| SPTSSA | -0.495602192 | 5.360862739 | -7.51346819 | 3.12E-13 |
| GCM1 | 1.100003807 | -2.157665405 | 7.51290439 | 3.13E-13 |
| RAD23B | 0.308013027 | 7.415651033 | 7.512779641 | 3.13E-13 |
| ENOX2 | -0.452189898 | 4.030916975 | -7.50999341 | 3.19E-13 |
| RP11-245J9.5 | -0.713197222 | 0.127986366 | -7.509762405 | 3.20E-13 |
| KIF20B | 0.453869993 | 4.810659052 | 7.507816878 | 3.24E-13 |
| WDR3 | -0.522475466 | 3.539798584 | -7.507715209 | 3.24E-13 |
| POMGNT1 | -0.411382572 | 4.600250311 | -7.50683691 | 3.26E-13 |
| RP11-218F10.3 | -0.952507485 | 1.573354573 | -7.506678326 | 3.26E-13 |
| RP11-18B3.2 | 0.737654774 | -0.269830727 | 7.506146304 | 3.28E-13 |
| UCK1 | 0.220472604 | 5.414524614 | 7.506064533 | 3.28E-13 |
| DUSP12 | 0.318304148 | 5.883516253 | 7.50549136 | 3.29E-13 |
| CTD-2286N8.2 | 0.793378738 | 3.09732902 | 7.505264232 | 3.30E-13 |
| ZSCAN25 | 0.362518236 | 4.316479851 | 7.504787297 | 3.31E-13 |
| CBX1 | -0.57543227 | 5.904429157 | -7.504073643 | 3.32E-13 |
| NONOP2 | -0.684218958 | 1.738652818 | -7.503668151 | 3.33E-13 |
| CELSR3 | 0.957815797 | 1.012705018 | 7.497610846 | 3.47E-13 |
| RP11-384K6.2 | 0.55061236 | 4.064352115 | 7.491840209 | 3.61E-13 |
| RP11-46F15.2 | -0.942316408 | 0.154619731 | -7.491120353 | 3.63E-13 |
| DEDD2 | 0.511630376 | 7.068861727 | 7.489322421 | 3.67E-13 |
| CTD-2302E22.5 | -0.766076685 | 0.636477307 | -7.488500575 | 3.69E-13 |
| DCTN4 | 0.456577065 | 6.131212635 | 7.48746781 | 3.72E-13 |
| EIF3J-AS1 | -0.417318178 | 3.746176995 | -7.485954274 | 3.76E-13 |
| RAB9B | -1.023137244 | 0.856384614 | -7.484904219 | 3.78E-13 |
| RECQL5 | -0.392645549 | 3.786411382 | -7.484429754 | 3.80E-13 |
| ATP13A3 | 0.443101305 | 5.779103253 | 7.48429428 | 3.80E-13 |
| LINC00846 | -0.968852263 | -1.253887227 | -7.4828681 | 3.84E-13 |
| RP11-295P9.3 | -0.475467739 | 2.644850755 | -7.476794141 | 4.00E-13 |
| AC004257.3 | -0.667939867 | 3.319703131 | -7.475645638 | 4.03E-13 |
| RP11-143J12.2 | 0.584207149 | 2.894121486 | 7.475001256 | 4.05E-13 |
| PPIH | 0.275311382 | 5.602709835 | 7.472794961 | 4.11E-13 |
| UBN1 | 0.27879862 | 6.243576242 | 7.472645842 | 4.11E-13 |
| CLNS1A | -0.386652046 | 6.676811048 | -7.472545128 | 4.11E-13 |
| ILK | -0.376419867 | 6.955407433 | -7.472493362 | 4.11E-13 |
| USP12 | 0.337701747 | 6.277695976 | 7.472266359 | 4.12E-13 |
| MKLN1 | 0.273657037 | 5.040588391 | 7.471817927 | 4.13E-13 |
| MRPL44 | 0.294641603 | 6.543337438 | 7.471268679 | 4.15E-13 |
| SVIP | 0.666652062 | 5.081103308 | 7.467051567 | 4.27E-13 |
| LEPRE1 | -0.370317463 | 5.271573342 | -7.465704488 | 4.31E-13 |
| KIAA0101 | 0.891885006 | 4.448830639 | 7.464504187 | 4.34E-13 |
| CYLD | 0.420178318 | 6.133922409 | 7.463300172 | 4.38E-13 |
| KCTD10 | 0.338582694 | 4.632857814 | 7.462881494 | 4.39E-13 |
| PPP1R3B | 0.831919064 | 4.062692546 | 7.462161547 | 4.41E-13 |
| PXMP4 | -0.56253856 | 3.627407439 | -7.462020035 | 4.42E-13 |
| JPX | -0.355867084 | 4.297501047 | -7.460539938 | 4.46E-13 |
| RAB11FIP2 | 0.322780034 | 5.190037178 | 7.459977401 | 4.48E-13 |
| EHMT2 | -0.38692179 | 5.778913727 | -7.459093426 | 4.50E-13 |
| RP11-655C2.3 | -0.90119564 | -0.838735064 | -7.45894666 | 4.51E-13 |
| TLR5 | -1.638222779 | 1.649779828 | -7.457755536 | 4.55E-13 |
| DFFB | -0.463594523 | 3.762561012 | -7.457722863 | 4.55E-13 |
| SNORD58 | 0.825199234 | 0.757942522 | 7.45629795 | 4.59E-13 |
| CCDC94 | 0.350726872 | 6.210431966 | 7.456167147 | 4.59E-13 |
| EXOC2 | -0.260073788 | 4.897667332 | -7.455925276 | 4.60E-13 |
| CTD-2619J13.14 | -0.544296839 | 2.827294896 | -7.455335331 | 4.62E-13 |
| EHHADH | -0.957377032 | 1.971449285 | -7.455288619 | 4.62E-13 |
| ZNF324B | -0.472028336 | 2.149949642 | -7.455181922 | 4.63E-13 |
| RP11-425I13.3 | -0.796209206 | 1.086065447 | -7.45269493 | 4.70E-13 |
| PBXIP1 | -0.58302773 | 7.692117112 | -7.452612149 | 4.71E-13 |
| FURIN | 0.450745762 | 7.860485214 | 7.451601851 | 4.74E-13 |
| INSIG2 | -0.474133962 | 3.748688699 | -7.450483081 | 4.77E-13 |
| FBXL19 | -0.387364711 | 5.583356597 | -7.449388551 | 4.81E-13 |
| AP000355.2 | 1.118506384 | 0.107291606 | 7.448214044 | 4.85E-13 |
| PCNP | 0.265742366 | 7.799823461 | 7.447126998 | 4.88E-13 |
| CLEC12A | -1.101246927 | 6.959832649 | -7.446720194 | 4.90E-13 |
| CTD-2583A14.11 | -0.428737338 | 4.229991814 | -7.446225778 | 4.91E-13 |
| CTD-2514K5.2 | -1.229354499 | -0.194770103 | -7.445987222 | 4.92E-13 |
| CTD-2006C1.6 | 0.840355055 | -0.691953387 | 7.44591752 | 4.92E-13 |
| MIR29A | 0.973612982 | 0.736348537 | 7.444441587 | 4.97E-13 |
| TALDO1 | -0.41492009 | 9.17543176 | -7.443964348 | 4.99E-13 |
| R3HCC1L | 0.301928562 | 5.096403545 | 7.443542525 | 5.00E-13 |
| ARHGEF7 | 0.407082591 | 5.203243173 | 7.442858836 | 5.03E-13 |
| CTA-407F11.8 | 1.007236163 | -0.278724556 | 7.442703254 | 5.03E-13 |
| RP11-809N8.4 | -0.905151116 | 3.351223347 | -7.442518578 | 5.04E-13 |
| SBDSP1 | 0.563225151 | 6.561405819 | 7.44045111 | 5.11E-13 |
| RP11-173M1.8 | -0.45124443 | 2.961151629 | -7.44023253 | 5.12E-13 |
| 15-Sep | -0.291168601 | 7.781830694 | -7.438955606 | 5.16E-13 |
| WDR78 | -0.94892709 | -0.54994148 | -7.438218272 | 5.19E-13 |
| CENPH | 0.538287276 | 4.979017824 | 7.436250658 | 5.26E-13 |
| ENY2 | 0.298210965 | 5.930317974 | 7.434947342 | 5.30E-13 |
| TAS2R10 | -0.983657307 | -0.256765966 | -7.43348417 | 5.35E-13 |
| SMC3 | 0.245587155 | 7.042512531 | 7.432609181 | 5.39E-13 |
| CBX1P2 | -0.781812035 | 1.059377516 | -7.432198281 | 5.40E-13 |
| FCF1 | -0.257511524 | 5.993426037 | -7.43205098 | 5.41E-13 |
| FBXL8 | -0.455796154 | 2.360064288 | -7.431881634 | 5.41E-13 |
| AC013474.4 | 1.16975748 | 1.909808093 | 7.427330216 | 5.58E-13 |
| RP11-529K1.2 | -0.661681711 | 0.78672538 | -7.427074655 | 5.59E-13 |
| DMXL1 | -0.250235048 | 5.148851742 | -7.42689735 | 5.60E-13 |
| AVIL | -0.85201714 | 0.86590154 | -7.42573253 | 5.64E-13 |
| CMTR2 | -0.532580318 | 3.917038036 | -7.425264932 | 5.66E-13 |
| RP11-363E6.3 | -0.960499401 | 0.319744636 | -7.42496699 | 5.67E-13 |
| FBXL12 | 0.304354737 | 5.386440245 | 7.424214057 | 5.70E-13 |
| SESN2 | 0.788337216 | 5.007014524 | 7.424052066 | 5.71E-13 |
| SUCO | 0.448095835 | 5.867358372 | 7.423784872 | 5.72E-13 |
| AVPI1 | 0.773344552 | 2.970172032 | 7.423348726 | 5.73E-13 |
| STARD5 | 0.597752641 | 1.701377072 | 7.422805712 | 5.75E-13 |
| RP11-18H7.1 | -0.627567921 | 0.938315937 | -7.422579638 | 5.76E-13 |
| EXO1 | 0.956215147 | 3.334074282 | 7.419824148 | 5.87E-13 |
| SNHG11 | 0.40388187 | 3.312313252 | 7.418853424 | 5.91E-13 |
| TRGV6 | -1.249579892 | 0.088203426 | -7.418712471 | 5.91E-13 |
| PSMC3IP | 0.524738282 | 3.713797601 | 7.415504349 | 6.04E-13 |
| RP11-405L18.2 | 0.650006715 | 0.866381862 | 7.415404965 | 6.05E-13 |
| FAM134C | -0.455702591 | 5.256265459 | -7.41464895 | 6.08E-13 |
| RP11-573G6.6 | -0.932465135 | -0.55329836 | -7.414459862 | 6.09E-13 |
| RP11-32B5.1 | -0.391628638 | 5.741876496 | -7.414062022 | 6.10E-13 |
| RP11-11C20.3 | -0.845456944 | -0.106763509 | -7.413456889 | 6.13E-13 |
| USP12PY | 0.502887817 | 1.912492012 | 7.411783522 | 6.20E-13 |
| CHAMP1 | 0.328307192 | 5.552702954 | 7.410796724 | 6.24E-13 |
| NARS2 | -0.47623826 | 4.256872581 | -7.409464034 | 6.29E-13 |
| P2RX7 | -0.870242 | 4.04937069 | -7.407151114 | 6.39E-13 |
| ATF4P3 | 0.507763 | 3.89135279 | 7.4049225 | 6.49E-13 |
| MYNN | 0.258140577 | 4.571560646 | 7.403940057 | 6.53E-13 |
| SAMSN1 | 0.79026214 | 6.570257402 | 7.403518409 | 6.55E-13 |
| CHKB-AS1 | -0.633477085 | 1.644365783 | -7.403121141 | 6.57E-13 |
| RP11-15J10.1 | -0.554752322 | 2.258565505 | -7.401922067 | 6.62E-13 |
| CCDC22 | -0.287189215 | 5.90311346 | -7.400626869 | 6.68E-13 |
| NUSAP1 | 0.756440834 | 6.150367008 | 7.399208929 | 6.74E-13 |
| KLLN | -0.786282673 | 0.653296518 | -7.398076819 | 6.79E-13 |
| UFSP1 | -0.873607825 | 1.031710185 | -7.395562913 | 6.91E-13 |
| CYB561D2 | -0.389150397 | 4.439189395 | -7.394810849 | 6.94E-13 |
| TRIM60P18 | -0.789447519 | 1.645713359 | -7.392653487 | 7.05E-13 |
| CCT5 | -0.442480255 | 6.927450447 | -7.391966554 | 7.08E-13 |
| LAIR1 | -0.650230104 | 7.561339912 | -7.390399896 | 7.15E-13 |
| LINC00176 | 0.699714951 | -0.204357948 | 7.388824536 | 7.23E-13 |
| RP5-874C20.3 | -0.375071168 | 4.641607918 | -7.388659838 | 7.24E-13 |
| HERC2P2 | -0.731832046 | 5.659760934 | -7.386535406 | 7.34E-13 |
| STK35 | 0.268059736 | 4.580086598 | 7.385446181 | 7.39E-13 |
| SASS6 | 0.351915262 | 3.958621135 | 7.385210408 | 7.41E-13 |
| FAM120A | -0.326097387 | 7.265693534 | -7.384676442 | 7.43E-13 |
| RNU4ATAC18P | -0.801503073 | 1.142651173 | -7.384006945 | 7.47E-13 |
| FARSA | -0.703015316 | 6.897117768 | -7.383676707 | 7.48E-13 |
| ALG13 | -0.31420529 | 5.175140159 | -7.381061124 | 7.62E-13 |
| COG2 | -0.397108112 | 3.591520142 | -7.379544934 | 7.69E-13 |
| CNOT6LP1 | 0.442462823 | 4.70105597 | 7.37882765 | 7.73E-13 |
| CETN3 | -0.611550348 | 3.781520186 | -7.377691721 | 7.79E-13 |
| CYP27B1 | 0.880230947 | 0.957916037 | 7.376370734 | 7.86E-13 |
| RPP30 | 0.251057166 | 4.090208514 | 7.375924241 | 7.88E-13 |
| RP11-8L8.2 | -0.959420974 | 0.850505779 | -7.375317168 | 7.91E-13 |
| FYB | -0.75334842 | 7.424464777 | -7.375231098 | 7.92E-13 |
| KLF7-IT1 | 0.920454864 | 3.630944788 | 7.373856437 | 7.99E-13 |
| CLSPN | 0.831949784 | 3.868566087 | 7.373600558 | 8.01E-13 |
| WDR11 | -0.259511702 | 5.210705953 | -7.370909949 | 8.15E-13 |
| HMGCL | -0.466797864 | 3.487518643 | -7.367415143 | 8.34E-13 |
| JRK | -0.479028512 | 3.42564492 | -7.36741165 | 8.34E-13 |
| PHF3 | 0.253355873 | 6.497059 | 7.364579458 | 8.50E-13 |
| DLST | 0.296180878 | 6.630111065 | 7.360386467 | 8.75E-13 |
| NECAP2 | -0.356946172 | 5.721520884 | -7.359609204 | 8.79E-13 |
| C20ORF135 | -0.66759943 | 3.647134153 | -7.359440471 | 8.80E-13 |
| MIR146A | -1.406258337 | 0.976738132 | -7.357548548 | 8.91E-13 |
| BCL7C | 0.265112029 | 4.70097961 | 7.356644925 | 8.97E-13 |
| RP11-263K4.1 | -0.902977399 | -0.808826528 | -7.355804573 | 9.02E-13 |
| DRAM2 | -0.329323053 | 6.450991405 | -7.35531037 | 9.05E-13 |
| TOB2 | -0.40867611 | 5.898933904 | -7.354361573 | 9.11E-13 |
| MMS19 | -0.245563363 | 5.572939606 | -7.353961384 | 9.13E-13 |
| FAHD1 | -0.453140705 | 3.881565399 | -7.353388762 | 9.17E-13 |
| TRGVA | -0.944304865 | -0.556320042 | -7.352364494 | 9.23E-13 |
| RP11-881M11.2 | -0.818503442 | -0.897509362 | -7.351419691 | 9.29E-13 |
| CEP95 | 0.40532942 | 5.508687785 | 7.350130878 | 9.37E-13 |
| BZW1 | 0.307188684 | 7.029110661 | 7.350040568 | 9.37E-13 |
| CHST14 | -0.514198573 | 5.25999699 | -7.349784479 | 9.39E-13 |
| DALRD3 | -0.33545922 | 4.54167944 | -7.349630166 | 9.40E-13 |
| GPALPP1 | -0.328268034 | 4.967510884 | -7.349088978 | 9.43E-13 |
| RBM43 | 0.386447068 | 3.610811755 | 7.347931766 | 9.51E-13 |
| MTG2 | -0.333458147 | 3.812999993 | -7.347634939 | 9.52E-13 |
| LAMB3 | 1.536351216 | 0.863315162 | 7.347077439 | 9.56E-13 |
| CENPP | 0.463513515 | 2.656916863 | 7.345360086 | 9.67E-13 |
| MTFMT | -0.31204354 | 4.058280699 | -7.345157101 | 9.68E-13 |
| TIGD5 | -0.471095836 | 4.22286933 | -7.345083173 | 9.69E-13 |
| RPL7AP64 | -1.737826978 | 0.413388939 | -7.345073178 | 9.69E-13 |
| TMTC3 | 0.26960166 | 3.211764313 | 7.34403853 | 9.76E-13 |
| IST1 | 0.361875025 | 6.910528966 | 7.343132074 | 9.82E-13 |
| RUNX3 | 0.888950592 | 6.587771332 | 7.342111332 | 9.88E-13 |
| LINC-PINT | 0.94994839 | 4.220861908 | 7.341069448 | 9.95E-13 |
| RINT1 | 0.366109007 | 4.62294416 | 7.340802346 | 9.97E-13 |
| AC074117.10 | -0.54504024 | 3.033109883 | -7.340407155 | 1.00E-12 |
| ZNF551 | 0.607300428 | 3.996620079 | 7.340197506 | 1.00E-12 |
| CTD-2555A7.3 | -0.782894923 | 0.140356749 | -7.340075508 | 1.00E-12 |
| UNC45A | -0.386751485 | 5.202454091 | -7.339936387 | 1.00E-12 |
| ADSS | -0.371351059 | 6.446442342 | -7.339768377 | 1.00E-12 |
| TMSB10 | -0.399444562 | 11.48394834 | -7.338900757 | 1.01E-12 |
| SLC25A26 | 0.442238843 | 3.57353383 | 7.338802381 | 1.01E-12 |
| MYCBP2 | -0.327756355 | 6.753507059 | -7.338231946 | 1.01E-12 |
| FASTKD5 | 0.574849331 | 6.295639562 | 7.337783947 | 1.02E-12 |
| PRRC2C | 0.230602439 | 7.573391369 | 7.337015885 | 1.02E-12 |
| PIGH | 0.50604901 | 4.202606784 | 7.336280693 | 1.03E-12 |
| RP11-285G1.14 | -0.814968739 | -0.133049182 | -7.335940585 | 1.03E-12 |
| ABCC10 | -0.304034081 | 3.895744673 | -7.33519337 | 1.03E-12 |
| MKS1 | -0.512410318 | 3.389491823 | -7.334683197 | 1.04E-12 |
| CYP21A1P | 0.905733892 | -0.683751925 | 7.329901635 | 1.07E-12 |
| TGFBR3L | 0.897715625 | 0.140922948 | 7.328816438 | 1.08E-12 |
| MORC2-AS1 | -1.366210904 | 0.121245626 | -7.325442852 | 1.10E-12 |
| ZC3H12A | 0.919337736 | 5.933487413 | 7.324026877 | 1.11E-12 |
| WDR45B | 0.407102434 | 7.044421117 | 7.322877039 | 1.12E-12 |
| RP11-339B21.15 | -0.674917888 | 1.961229823 | -7.320747286 | 1.14E-12 |
| FEZ2 | -0.291957443 | 4.475654891 | -7.320507209 | 1.14E-12 |
| ZXDB | 0.633690646 | 4.065699472 | 7.319973659 | 1.15E-12 |
| AKR7L | 0.83845069 | 0.331697522 | 7.319093638 | 1.15E-12 |
| BIN2 | -0.571281682 | 8.434292566 | -7.318943461 | 1.15E-12 |
| BUB1B | 0.822020351 | 3.825269405 | 7.318739407 | 1.15E-12 |
| AC009120.3 | -0.652912224 | 3.153659045 | -7.317971738 | 1.16E-12 |
| NFKBIE | 0.586134291 | 5.589682523 | 7.317254396 | 1.17E-12 |
| SLC25A12 | -0.304244074 | 4.090092009 | -7.316755278 | 1.17E-12 |
| CTD-3224K15.3 | -1.001564245 | 0.245449227 | -7.315807506 | 1.18E-12 |
| LINC00957 | -1.122034531 | -0.133930392 | -7.314252712 | 1.19E-12 |
| CTC-479C5.12 | 0.619329108 | 2.464664506 | 7.312784161 | 1.20E-12 |
| C1orf220 | -0.993003733 | -1.414786903 | -7.312417471 | 1.20E-12 |
| AL357673.1 | -0.564830921 | 3.157551983 | -7.312282763 | 1.21E-12 |
| CASP8AP2 | -0.497870686 | 4.220065065 | -7.311489552 | 1.21E-12 |
| SLC19A2 | 0.639493397 | 3.326989276 | 7.309565751 | 1.23E-12 |
| RP11-205K6.1 | 0.923549722 | -1.044688892 | 7.309196889 | 1.23E-12 |
| FBXW4 | 0.333669557 | 4.763149715 | 7.307615283 | 1.24E-12 |
| UBE4B | -0.266289595 | 5.815542118 | -7.307563317 | 1.24E-12 |
| RP11-395B7.7 | -0.524329309 | 5.746338141 | -7.307455699 | 1.24E-12 |
| AC116366.6 | 1.333605922 | 2.967416666 | 7.307341846 | 1.25E-12 |
| RP11-473I1.10 | 0.717053465 | 6.723853506 | 7.307302817 | 1.25E-12 |
| CCDC71L | 0.570791006 | 3.947655647 | 7.307012036 | 1.25E-12 |
| PPCS | -0.248445488 | 6.368307013 | -7.304325426 | 1.27E-12 |
| AREGB | 2.040469938 | 1.810235258 | 7.300652098 | 1.30E-12 |
| AC006538.1 | -1.08765397 | 0.94392538 | -7.298244871 | 1.32E-12 |
| AC078899.1 | -0.45082452 | 6.788296013 | -7.297940973 | 1.33E-12 |
| NEURL4 | -0.317837202 | 4.412766853 | -7.296796515 | 1.34E-12 |
| RN7SL272P | -1.006571393 | -0.818193378 | -7.296633729 | 1.34E-12 |
| NPEPPS | -0.209257448 | 5.456029489 | -7.296412793 | 1.34E-12 |
| LRRC17 | -1.135523484 | -0.287124912 | -7.295731197 | 1.35E-12 |
| ANAPC7 | -0.303130444 | 5.156488519 | -7.29535996 | 1.35E-12 |
| H2AFV | 0.366413393 | 7.074704472 | 7.294118691 | 1.36E-12 |
| CATSPERD | 1.091231589 | -1.984354814 | 7.294069846 | 1.36E-12 |
| TPTE2P5 | -0.615099943 | 1.520016837 | -7.290409336 | 1.39E-12 |
| SLC25A43 | -0.494192755 | 4.400650008 | -7.289307154 | 1.40E-12 |
| CLK3 | 0.336903047 | 5.752633787 | 7.288462923 | 1.41E-12 |
| BBS9 | -0.532325848 | 2.83786382 | -7.287746345 | 1.42E-12 |
| HIST1H2AK | -1.125097886 | 2.188466787 | -7.287535992 | 1.42E-12 |
| RP11-305L7.7 | -0.841551136 | -2.655703354 | -7.284268451 | 1.45E-12 |
| FERMT2 | 1.10670229 | -2.305471977 | 7.283869148 | 1.46E-12 |
| HBZ | 1.304145899 | -1.012406733 | 7.28384573 | 1.46E-12 |
| RP11-1018J11.1 | -0.318086746 | 5.510383604 | -7.28382616 | 1.46E-12 |
| FBXO48 | -0.534257745 | 1.320164579 | -7.279584278 | 1.50E-12 |
| NDUFB3 | -0.47429266 | 5.92496555 | -7.278725269 | 1.51E-12 |
| PARVG | -0.431990248 | 7.397257376 | -7.277719359 | 1.52E-12 |
| AK9 | -0.433723003 | 2.220814932 | -7.277402761 | 1.52E-12 |
| RABL2B | -0.350949248 | 4.332138596 | -7.275433415 | 1.54E-12 |
| ZBTB49 | 0.389044985 | 3.960936364 | 7.275373844 | 1.54E-12 |
| RP4-791M13.3 | -0.869955715 | -0.010776665 | -7.274655779 | 1.55E-12 |
| SEC14L1 | 0.469541722 | 5.843817774 | 7.273324744 | 1.56E-12 |
| ANKFY1 | -0.350541524 | 4.986845219 | -7.272037858 | 1.58E-12 |
| RSPH4A | 0.954839316 | 0.572823715 | 7.271912537 | 1.58E-12 |
| CREB1 | -0.249842515 | 5.620828393 | -7.271606401 | 1.58E-12 |
| RP11-727A23.11 | -0.789892869 | 0.706999936 | -7.271450614 | 1.58E-12 |
| RP11-384K6.6 | 0.500050003 | 4.376617733 | 7.270764727 | 1.59E-12 |
| GDPGP1 | -0.493311844 | 1.613483177 | -7.269732314 | 1.60E-12 |
| LYL1 | -0.500784163 | 6.692450233 | -7.269238866 | 1.60E-12 |
| IL6R | -0.673808127 | 6.87441882 | -7.268608978 | 1.61E-12 |
| G2E3 | 0.271168646 | 3.670364664 | 7.268190728 | 1.62E-12 |
| CTD-2007H13.3 | -0.730865754 | 1.031822898 | -7.267645535 | 1.62E-12 |
| CATSPER3 | -0.726463841 | -0.040973093 | -7.262947032 | 1.67E-12 |
| RND1 | 0.984808117 | -0.164741129 | 7.262598767 | 1.68E-12 |
| NMI | -0.287706093 | 6.410475528 | -7.262333166 | 1.68E-12 |
| ZNF343 | -0.337724145 | 3.007821621 | -7.261991172 | 1.68E-12 |
| RP11-169K17.3 | 0.864320334 | -0.436101 | 7.260896923 | 1.70E-12 |
| RNU6-1016P | 0.597032895 | 3.929729286 | 7.260822423 | 1.70E-12 |
| NOL8 | -0.288537954 | 5.18680379 | -7.260806704 | 1.70E-12 |
| ZNF891 | -0.542957527 | 1.896227543 | -7.258687848 | 1.72E-12 |
| OAS1 | -1.088224 | 4.333335435 | -7.25619085 | 1.75E-12 |
| CTU1 | -0.471119148 | 3.332422406 | -7.254711124 | 1.77E-12 |
| PAFAH1B1 | -0.28978871 | 6.1825364 | -7.254311103 | 1.77E-12 |
| MUM1 | -0.530745087 | 4.048166168 | -7.253146838 | 1.78E-12 |
| THBD | 1.316993322 | 3.827508215 | 7.25157693 | 1.80E-12 |
| FAM65B | -0.765000117 | 6.426693094 | -7.2515356 | 1.80E-12 |
| DHODH | -0.562565343 | 2.936361631 | -7.25054638 | 1.82E-12 |
| EBAG9P1 | -0.850306933 | -0.237287277 | -7.249572321 | 1.83E-12 |
| MLH3 | -0.455248144 | 3.236551521 | -7.248919479 | 1.84E-12 |
| PSMA1 | 0.402583966 | 4.796156858 | 7.248672966 | 1.84E-12 |
| MTSS1L | 0.793987773 | 4.651431109 | 7.248083591 | 1.85E-12 |
| GIMAP1 | -0.963541523 | 3.814322847 | -7.246486587 | 1.87E-12 |
| MRPS26 | -0.475731237 | 6.020105918 | -7.24502757 | 1.88E-12 |
| NUDT7 | -1.016038978 | 3.077906238 | -7.244784256 | 1.89E-12 |
| AC006111.1 | -0.814608977 | -0.879781625 | -7.243765697 | 1.90E-12 |
| RP11-432J22.2 | -0.644717984 | 2.540597769 | -7.242000011 | 1.92E-12 |
| LINC00152 | 0.639310906 | 4.221839597 | 7.237603738 | 1.98E-12 |
| U2AF1L4 | 0.505163894 | 4.443193648 | 7.236809498 | 1.99E-12 |
| LRSAM1 | -0.254921065 | 4.809712035 | -7.23674073 | 1.99E-12 |
| RP3-408N23.4 | -0.860218915 | 0.02439353 | -7.236665934 | 1.99E-12 |
| CKAP2 | 0.358119384 | 6.390229235 | 7.236600933 | 1.99E-12 |
| FRG1 | 0.223627161 | 6.594019595 | 7.236592653 | 1.99E-12 |
| GABARAPL1 | 0.828787233 | 6.139705075 | 7.235867056 | 2.00E-12 |
| MED13 | 0.192434981 | 6.021315043 | 7.233313934 | 2.03E-12 |
| HELQ | -0.244928042 | 4.341848297 | -7.233026695 | 2.04E-12 |
| TSEN2 | -0.489085167 | 2.842308686 | -7.232426554 | 2.05E-12 |
| FAM20B | -0.374940612 | 5.876426814 | -7.232342156 | 2.05E-12 |
| CCL7 | 1.592161471 | -1.428453284 | 7.232154054 | 2.05E-12 |
| EIF4G3 | -0.330098722 | 6.384282989 | -7.231068113 | 2.07E-12 |
| C5orf63 | -0.810675844 | 1.677113377 | -7.230502521 | 2.07E-12 |
| KIAA0586 | -0.292602674 | 4.343387075 | -7.229749235 | 2.08E-12 |
| PLGLB1 | -0.912875026 | 0.378444641 | -7.229611944 | 2.09E-12 |
| HSPA8P14 | -0.870024749 | -1.993579558 | -7.229591905 | 2.09E-12 |
| NTPCR | -0.507494498 | 2.851777114 | -7.228046107 | 2.11E-12 |
| BTN2A3P | -0.580899511 | 1.978440245 | -7.227758244 | 2.11E-12 |
| RP11-175O19.4 | 0.365420819 | 4.661612238 | 7.226867684 | 2.12E-12 |
| CMTM1 | -0.886073451 | 2.792432537 | -7.226801948 | 2.12E-12 |
| CPEB4 | 0.658475223 | 5.320411804 | 7.226800648 | 2.12E-12 |
| CCNB2 | 0.985552145 | 5.155015075 | 7.226475523 | 2.13E-12 |
| DCAF16 | 0.442436416 | 5.761702952 | 7.226465983 | 2.13E-12 |
| CTD-2619J13.16 | -0.831025135 | 0.998762404 | -7.222813206 | 2.18E-12 |
| RP3-404F18.5 | -0.897670664 | -0.370154463 | -7.222129404 | 2.19E-12 |
| ARFGEF2 | 0.303355219 | 5.694116089 | 7.222038066 | 2.19E-12 |
| CCDC132 | -0.379197654 | 3.420973797 | -7.220214162 | 2.22E-12 |
| RASAL1 | 1.513649609 | -2.573322532 | 7.216822792 | 2.27E-12 |
| C4orf27 | -0.485489115 | 5.268643296 | -7.215511309 | 2.29E-12 |
| CLUAP1 | -0.514155337 | 2.256262607 | -7.215017663 | 2.30E-12 |
| RPS6KL1 | 1.523859503 | 0.085990362 | 7.213151717 | 2.32E-12 |
| RP11-792A8.4 | -0.401840066 | 4.400054341 | -7.212169189 | 2.34E-12 |
| DCAF15 | 0.224987043 | 6.311041867 | 7.210686639 | 2.36E-12 |
| HAUS6 | 0.289106111 | 4.480236519 | 7.210497478 | 2.37E-12 |
| FOSL2 | 0.776111893 | 8.200841317 | 7.209566463 | 2.38E-12 |
| GART | -0.484534714 | 5.343536309 | -7.209155674 | 2.39E-12 |
| AC093627.9 | -0.583836756 | 1.576615183 | -7.209035961 | 2.39E-12 |
| AC103801.2 | -0.822148057 | 0.278160714 | -7.208762625 | 2.39E-12 |
| PHF14 | -0.403939193 | 4.930871335 | -7.20590284 | 2.44E-12 |
| ESYT1 | -0.489205713 | 7.537179785 | -7.20577599 | 2.44E-12 |
| TBC1D9B | -0.320351073 | 6.4834353 | -7.205733717 | 2.44E-12 |
| PRRT3 | 0.662989215 | 1.755290412 | 7.205481911 | 2.44E-12 |
| LINC00622 | -0.946960984 | -1.369356292 | -7.204890294 | 2.45E-12 |
| ATP13A1 | -0.235679165 | 5.724970743 | -7.20254571 | 2.49E-12 |
| ZFP2 | -0.973228411 | 0.523034282 | -7.200975297 | 2.52E-12 |
| STRN4 | 0.19767201 | 6.131862205 | 7.200638085 | 2.52E-12 |
| TMEM161B-DT | -0.570051452 | 2.921680907 | -7.199973131 | 2.53E-12 |
| RP11-629B11.4 | -0.602748417 | 1.138290767 | -7.199631092 | 2.54E-12 |
| PIK3CA | 0.317649172 | 5.138711058 | 7.199033809 | 2.55E-12 |
| RNU6-6P | 0.59630367 | 2.082784184 | 7.198725165 | 2.56E-12 |
| HNRNPH1P1 | -0.867339647 | 1.018202494 | -7.197482435 | 2.58E-12 |
| RNF6 | 0.248006455 | 6.050075354 | 7.195578303 | 2.61E-12 |
| UBL7 | -0.284851725 | 6.490691604 | -7.194642099 | 2.63E-12 |
| ZFP1 | 0.406814549 | 3.707725014 | 7.193015635 | 2.65E-12 |
| SPHK2 | -0.326225326 | 3.037610375 | -7.19294095 | 2.65E-12 |
| LAMTOR2 | -0.425540569 | 6.469409762 | -7.192288873 | 2.67E-12 |
| ANAPC16 | -0.329024898 | 6.299793785 | -7.191780898 | 2.68E-12 |
| RP11-844P9.3 | -0.429655287 | 3.073073606 | -7.191572975 | 2.68E-12 |
| DUSP23 | -0.43226596 | 5.974480794 | -7.19154036 | 2.68E-12 |
| RNF115 | 0.239848342 | 5.109503017 | 7.191405167 | 2.68E-12 |
| CCDC80 | 1.353849562 | -2.616476542 | 7.191114363 | 2.69E-12 |
| FAM86C2P | -0.818564273 | 0.893985502 | -7.190094935 | 2.70E-12 |
| SEC14L1P1 | 0.541115572 | 2.725343351 | 7.189551865 | 2.71E-12 |
| COPS8 | 0.271492344 | 4.976731219 | 7.188095222 | 2.74E-12 |
| SPAG8 | -0.619405316 | 2.070925858 | -7.187892221 | 2.74E-12 |
| ANAPC10 | 0.348908795 | 3.376064214 | 7.187420823 | 2.75E-12 |
| IRF5 | -0.600178791 | 6.265291201 | -7.186020046 | 2.78E-12 |
| MRPS34 | -0.486789964 | 7.022729048 | -7.18581996 | 2.78E-12 |
| PRICKLE1 | -1.415294634 | 1.943542468 | -7.18400693 | 2.82E-12 |
| RAB2B | 0.324415136 | 5.397423234 | 7.181695457 | 2.86E-12 |
| ZNF33B | -0.527590281 | 4.526238133 | -7.181671905 | 2.86E-12 |
| ACTA2 | -0.812656607 | 4.421668578 | -7.181471584 | 2.86E-12 |
| ZNF668 | 0.390542308 | 4.166494069 | 7.180821273 | 2.87E-12 |
| CTB-129O4.1 | -0.777377609 | 1.842603909 | -7.180408729 | 2.88E-12 |
| YTHDF3 | 0.372035992 | 6.848379845 | 7.179323768 | 2.90E-12 |
| PRPF40B | -0.625659642 | 1.185801175 | -7.179011165 | 2.91E-12 |
| UBAC2 | -0.267877567 | 5.819972139 | -7.178625259 | 2.92E-12 |
| GATAD1 | -0.309640654 | 5.584133943 | -7.177261564 | 2.94E-12 |
| LINC01128 | 0.459260855 | 2.468187981 | 7.176268907 | 2.96E-12 |
| DARS2 | -0.590606552 | 4.602329059 | -7.175950171 | 2.97E-12 |
| PTGES3P1 | -0.448895897 | 8.156382827 | -7.175259981 | 2.98E-12 |
| RP11-339B21.14 | -0.571888635 | 2.952067311 | -7.173542152 | 3.02E-12 |
| RP11-102L12.2 | 0.851873518 | 1.047355071 | 7.173030946 | 3.03E-12 |
| HMGB2 | 0.563764561 | 9.093621418 | 7.171319462 | 3.06E-12 |
| REXO4 | -0.385585689 | 5.930447766 | -7.168672453 | 3.11E-12 |
| VPS4A | 0.225572623 | 6.013042594 | 7.168060133 | 3.13E-12 |
| AKAP8 | 0.285109697 | 6.050289979 | 7.167972368 | 3.13E-12 |
| RP11-158H5.7 | 0.612099632 | 1.846447229 | 7.167956769 | 3.13E-12 |
| TRIQK | -0.610163142 | 4.58739673 | -7.165298999 | 3.18E-12 |
| ATXN7L1 | -0.392747137 | 4.366758755 | -7.165079727 | 3.19E-12 |
| SUMO1 | 0.239715418 | 7.032286913 | 7.16370132 | 3.22E-12 |
| MAMLD1 | 1.114381159 | -0.187476562 | 7.163359575 | 3.22E-12 |
| SPRY1 | 1.435000943 | 4.05358749 | 7.163037147 | 3.23E-12 |
| RP13-444K19.1 | -0.48651009 | 3.779521387 | -7.16008427 | 3.29E-12 |
| RP11-37B2.1 | 0.636249508 | 3.297297861 | 7.156818611 | 3.36E-12 |
| TRIM65 | -0.36795061 | 5.066470583 | -7.15627984 | 3.38E-12 |
| ABHD15 | -0.571089874 | 5.01753054 | -7.154545941 | 3.42E-12 |
| HSPA8P11 | -0.792269197 | -0.564676983 | -7.151799294 | 3.48E-12 |
| DCTN3 | 0.27442222 | 5.360132336 | 7.151232019 | 3.49E-12 |
| RNF167 | -0.268864161 | 7.361092988 | -7.150546368 | 3.51E-12 |
| ABAT | -0.501812619 | 2.764854709 | -7.149723204 | 3.52E-12 |
| RP11-504P24.8 | -0.447288772 | 3.916320068 | -7.149476852 | 3.53E-12 |
| XPO1 | -0.409466715 | 6.895594146 | -7.149394283 | 3.53E-12 |
| DEDD | 0.236844879 | 6.560808434 | 7.147915051 | 3.57E-12 |
| SAP30L-AS1 | -0.825179532 | -0.093048009 | -7.147522297 | 3.58E-12 |
| PLCB2 | -0.562547437 | 7.742430529 | -7.146561432 | 3.60E-12 |
| ALG9 | 0.384035984 | 1.926974984 | 7.146057013 | 3.61E-12 |
| RP3-324O17.4 | -0.46311781 | 3.13232382 | -7.14447758 | 3.65E-12 |
| DENR | -0.302546012 | 6.796407085 | -7.143927695 | 3.66E-12 |
| PHKG1 | 0.712940366 | 1.675104831 | 7.14352498 | 3.67E-12 |
| NSUN5P1 | -0.541125554 | 4.325728544 | -7.142584978 | 3.69E-12 |
| CTD-2287O16.5 | -0.708377473 | 1.710882685 | -7.140350587 | 3.75E-12 |
| HSPA8P8 | -0.69835395 | 1.937988234 | -7.13978819 | 3.76E-12 |
| MLEC | -0.552213669 | 6.911853199 | -7.139648294 | 3.76E-12 |
| PCNPP1 | 0.419299852 | 2.591478942 | 7.139203616 | 3.78E-12 |
| ZNF468 | 0.364478084 | 3.840499251 | 7.138413013 | 3.80E-12 |
| IARS2 | -0.327396235 | 6.432530998 | -7.138320237 | 3.80E-12 |
| GTSE1 | 0.931839282 | 2.802054781 | 7.1374681 | 3.82E-12 |
| RP11-139K1.2 | 1.10018248 | 0.652363556 | 7.136658603 | 3.84E-12 |
| NICN1 | -0.401445039 | 4.88899673 | -7.136296207 | 3.85E-12 |
| RN7SL650P | 0.660057281 | 2.24985324 | 7.136228482 | 3.85E-12 |
| CIAO2AP2 | -0.510708761 | 3.159346929 | -7.135500064 | 3.87E-12 |
| RP4-592A1.2 | -0.704791335 | 7.748264822 | -7.13466326 | 3.89E-12 |
| SDAD1P1 | -0.535547635 | 2.149212491 | -7.134397263 | 3.90E-12 |
| AC069363.1 | 1.007575588 | -0.287056278 | 7.134396868 | 3.90E-12 |
| PPIG | 0.271205602 | 6.136631497 | 7.13369084 | 3.91E-12 |
| CALM2P2 | -0.559356445 | 7.096991183 | -7.131144081 | 3.98E-12 |
| GOSR2 | -0.255125696 | 4.000304602 | -7.130564303 | 3.99E-12 |
| RP11-342K6.2 | -0.760160047 | 1.347227652 | -7.129918732 | 4.01E-12 |
| RP11-422P24.11 | -0.604002302 | 1.866850299 | -7.129488507 | 4.02E-12 |
| S100PBP | -0.362242702 | 4.83168738 | -7.129142264 | 4.03E-12 |
| LUZP1 | 0.618191708 | 5.641430639 | 7.12615743 | 4.11E-12 |
| AARS | -0.575682306 | 5.805481679 | -7.125799351 | 4.12E-12 |
| REC8 | 0.773765017 | 4.855871332 | 7.125695042 | 4.12E-12 |
| CXXC11 | 1.365235477 | -0.095124234 | 7.123463985 | 4.18E-12 |
| PDE4B | 1.058138508 | 4.834261128 | 7.123203939 | 4.19E-12 |
| RP11-218C14.8 | -1.04398832 | -0.697017037 | -7.122629221 | 4.21E-12 |
| KMT2E | 0.345234991 | 7.074060484 | 7.12221712 | 4.22E-12 |
| AKAP10 | -0.306891162 | 5.396042631 | -7.120642347 | 4.26E-12 |
| CTBP2 | -0.440737854 | 5.072681047 | -7.12064133 | 4.26E-12 |
| EIF4H | 0.330924027 | 8.704801864 | 7.119859081 | 4.28E-12 |
| CERS2 | -0.288051972 | 7.459769938 | -7.118155879 | 4.33E-12 |
| IWS1 | -0.320737199 | 6.209363427 | -7.117321244 | 4.36E-12 |
| ANAPC4 | -0.236210627 | 4.767240755 | -7.117238153 | 4.36E-12 |
| DENND2C | 1.337949383 | -0.997469232 | 7.116635004 | 4.37E-12 |
| RP11-262H14.1 | -0.826227152 | 5.456601478 | -7.115968938 | 4.39E-12 |
| RRP9 | -0.443597768 | 5.277870326 | -7.113620458 | 4.46E-12 |
| TSR3 | -0.260779537 | 6.607369026 | -7.112723797 | 4.49E-12 |
| RPS20P33 | -0.722408391 | 1.076287451 | -7.111328616 | 4.53E-12 |
| ATAD3B | 0.406149211 | 4.300766612 | 7.106183318 | 4.68E-12 |
| NAMPT | 1.070088449 | 6.625293344 | 7.106083708 | 4.69E-12 |
| SNX14 | -0.220626782 | 5.592040075 | -7.105619877 | 4.70E-12 |
| RP11-31F15.2 | -0.793302388 | -2.007948734 | -7.104803659 | 4.73E-12 |
| BRD1 | 0.316536206 | 6.106476272 | 7.104771559 | 4.73E-12 |
| ZNF674 | -0.444883417 | 1.457000495 | -7.103681418 | 4.76E-12 |
| SEC61A2 | 0.626553218 | 4.183246242 | 7.103441774 | 4.77E-12 |
| MEI1 | 0.564344499 | 3.027409509 | 7.103332086 | 4.77E-12 |
| RAB4B | -0.401478458 | 3.647876206 | -7.101230989 | 4.84E-12 |
| ABHD12 | 0.293366877 | 5.267387874 | 7.100121839 | 4.87E-12 |
| C4orf3 | -0.323371004 | 7.568131068 | -7.099909418 | 4.88E-12 |
| C7orf13 | -1.256563443 | 0.195052509 | -7.099310487 | 4.90E-12 |
| NSMAF | -0.332947533 | 6.223224084 | -7.097161259 | 4.97E-12 |
| PRELID2P1 | -1.06681749 | -0.586799883 | -7.096016755 | 5.00E-12 |
| RAB30-AS1 | -0.462282356 | 2.788301717 | -7.093884488 | 5.07E-12 |
| RREB1 | 0.346520434 | 6.151715199 | 7.092019504 | 5.14E-12 |
| CCDC121 | -0.744588521 | 1.757056895 | -7.091305632 | 5.16E-12 |
| MAP3K12 | -0.635861502 | 3.422532917 | -7.090905551 | 5.17E-12 |
| LINC00886 | -1.198327381 | -0.443402915 | -7.087954849 | 5.27E-12 |
| MAPK8 | 0.300229811 | 4.531925056 | 7.087363581 | 5.29E-12 |
| JMJD6 | 0.527911919 | 5.662295504 | 7.086925935 | 5.31E-12 |
| SLC25A34 | 0.630614909 | 2.025438276 | 7.084166216 | 5.40E-12 |
| LRRIQ3 | -0.996377136 | -1.358937282 | -7.083833735 | 5.42E-12 |
| ZNF252P-AS1 | -0.879058787 | -1.288338402 | -7.083605108 | 5.42E-12 |
| SERBP1 | -0.355830553 | 7.375760064 | -7.083494531 | 5.43E-12 |
| MGAT2 | -0.457508572 | 6.419675533 | -7.083264705 | 5.44E-12 |
| ZDHHC8 | -0.35840143 | 5.326398153 | -7.083210087 | 5.44E-12 |
| TMEM254 | -0.337270457 | 4.701717468 | -7.082899805 | 5.45E-12 |
| DTL | 0.792476394 | 4.321539399 | 7.081123832 | 5.51E-12 |
| SLC25A51 | 0.331455801 | 3.712290857 | 7.079527765 | 5.57E-12 |
| CLK2 | -0.316067132 | 6.236260528 | -7.078257113 | 5.62E-12 |
| RAD1 | 0.201423591 | 3.803406917 | 7.078119646 | 5.62E-12 |
| GPR162 | -1.046742161 | 3.907321416 | -7.077726577 | 5.64E-12 |
| SIAH3 | 0.960449311 | -3.349520161 | 7.077399571 | 5.65E-12 |
| AC114730.3 | 1.657959426 | -0.284312327 | 7.077260951 | 5.65E-12 |
| RP11-888D10.4 | 0.582572601 | 2.879047557 | 7.076664876 | 5.67E-12 |
| RP11-52A20.2 | 0.878325445 | 2.047114936 | 7.075244603 | 5.73E-12 |
| RBBP9 | -0.38295963 | 3.886607536 | -7.074448244 | 5.76E-12 |
| GALT | -0.316125682 | 4.330755096 | -7.074248584 | 5.76E-12 |
| PRDM1 | 0.980571315 | 3.686093127 | 7.074231049 | 5.76E-12 |
| RP4-671G15.2 | -0.813663962 | -0.645963965 | -7.073853596 | 5.78E-12 |
| RP11-455J20.3 | -0.839594888 | -0.061704931 | -7.073811308 | 5.78E-12 |
| LGMN | 1.23905366 | 1.573300209 | 7.073443198 | 5.79E-12 |
| EIF4A2 | -0.306708335 | 8.512874194 | -7.072623765 | 5.83E-12 |
| YWHAE | -0.415592631 | 8.455504757 | -7.071675472 | 5.86E-12 |
| NFKB1 | 0.446967554 | 6.22119979 | 7.070306128 | 5.91E-12 |
| USP16 | 0.365336845 | 5.719877034 | 7.070048196 | 5.92E-12 |
| BTBD8 | -0.728486139 | 0.330925545 | -7.06899131 | 5.96E-12 |
| ZNF3 | 0.354999748 | 4.659958107 | 7.068159218 | 6.00E-12 |
| BAIAP2L1 | 0.970790987 | -1.86718218 | 7.068065939 | 6.00E-12 |
| HSPA8P5 | -0.479107902 | 4.034983135 | -7.067974569 | 6.00E-12 |
| COMT | -0.428818823 | 6.40730156 | -7.067839377 | 6.01E-12 |
| TRAIP | 0.516955211 | 2.954877909 | 7.067748028 | 6.01E-12 |
| RP11-305M3.2 | -0.474827499 | 6.078240663 | -7.067647189 | 6.02E-12 |
| C10orf25 | -0.934006774 | 2.428596613 | -7.066128495 | 6.08E-12 |
| C21orf33 | -0.379190008 | 6.682877189 | -7.065394209 | 6.10E-12 |
| RNU6-638P | 0.821522132 | 2.584389043 | 7.065149643 | 6.11E-12 |
| RP11-546K22.3 | -0.851193522 | -0.348050959 | -7.062468809 | 6.22E-12 |
| CCDC30 | -0.65689319 | 0.773515422 | -7.062395547 | 6.22E-12 |
| OSTM1 | -0.550066711 | 4.585413365 | -7.061407821 | 6.26E-12 |
| LINC00869 | -0.901525319 | 2.56945528 | -7.060645876 | 6.30E-12 |
| TMEM214 | -0.29551797 | 5.830948057 | -7.057707503 | 6.42E-12 |
| PHF1 | 0.507535142 | 6.817779905 | 7.057425419 | 6.43E-12 |
| THAP4 | 0.247527211 | 5.925479382 | 7.056429716 | 6.47E-12 |
| MRPL38 | 0.534046997 | 2.283665633 | 7.055943541 | 6.49E-12 |
| ZNF382 | -0.757334075 | 1.483440443 | -7.054883425 | 6.54E-12 |
| DNAJC27 | 0.444311381 | 3.328094217 | 7.054714778 | 6.54E-12 |
| NDUFS8 | 0.243636054 | 5.16795335 | 7.053245935 | 6.60E-12 |
| RRM2 | 1.056174433 | 5.144489281 | 7.053128487 | 6.61E-12 |
| CTSC | -0.486164887 | 6.324575666 | -7.052838108 | 6.62E-12 |
| RP11-613M10.6 | -0.586179004 | 3.495392814 | -7.052834232 | 6.62E-12 |
| EDA2R | -1.455507132 | 1.04446075 | -7.052748366 | 6.63E-12 |
| SH3YL1 | -0.401942454 | 3.028625804 | -7.051697683 | 6.67E-12 |
| DECR1 | -0.338359059 | 5.883977044 | -7.050448353 | 6.73E-12 |
| SARNP | 0.56831174 | 0.973457121 | 7.050339456 | 6.73E-12 |
| VOPP1 | 0.634338656 | 4.107818413 | 7.048722638 | 6.80E-12 |
| RP11-661A12.14 | -0.606057946 | 2.090177302 | -7.048404942 | 6.82E-12 |
| FKTN | -0.358713689 | 3.319310576 | -7.048309664 | 6.82E-12 |
| FGF9 | 1.338819937 | -1.951191981 | 7.047792961 | 6.84E-12 |
| OSTC | -0.343059368 | 7.107000431 | -7.047477728 | 6.86E-12 |
| USP14 | 0.294876211 | 5.7447924 | 7.047473123 | 6.86E-12 |
| ATL2 | 0.287358915 | 4.991335622 | 7.046536767 | 6.90E-12 |
| AGGF1 | -0.309794368 | 5.357491268 | -7.046535927 | 6.90E-12 |
| ARL1 | -0.25074671 | 5.1016218 | -7.046113622 | 6.92E-12 |
| YKT6 | 0.29501102 | 6.584523172 | 7.045873892 | 6.93E-12 |
| DHX15 | 0.225677221 | 6.935482761 | 7.045864213 | 6.93E-12 |
| NSMCE1 | -0.298565186 | 5.109558288 | -7.04544935 | 6.95E-12 |
| CRNKL1 | 0.220670686 | 5.260867541 | 7.045368746 | 6.95E-12 |
| CDC25A | 0.835485048 | 3.557422006 | 7.043942736 | 7.01E-12 |
| SLC4A2 | -0.335028348 | 5.586673935 | -7.043819361 | 7.02E-12 |
| KPNA3 | 0.25247814 | 6.096060126 | 7.043106958 | 7.05E-12 |
| ANXA4 | -0.526971671 | 4.665072625 | -7.04272437 | 7.07E-12 |
| PPP2R1B | 0.329041312 | 4.100776456 | 7.04249604 | 7.08E-12 |
| CBX4 | 0.572185593 | 7.055888602 | 7.042222114 | 7.09E-12 |
| MXD3 | 0.50890197 | 4.086453008 | 7.042060156 | 7.10E-12 |
| C6orf165 | -0.671600323 | 0.297009346 | -7.041758957 | 7.11E-12 |
| MAVS | -0.371389549 | 6.192208263 | -7.040835148 | 7.16E-12 |
| RP9 | 0.364115142 | 4.911578183 | 7.040550938 | 7.17E-12 |
| TAF1A | -0.450044388 | 2.538422039 | -7.040173699 | 7.19E-12 |
| RP11-452K12.3 | -0.807521702 | 0.773563098 | -7.036422629 | 7.36E-12 |
| CD33 | -0.830360835 | 5.980880007 | -7.036024011 | 7.38E-12 |
| AHNAK | -0.793121951 | 9.363975817 | -7.034595381 | 7.45E-12 |
| STRIP2 | 1.021085928 | 0.947012381 | 7.034577325 | 7.45E-12 |
| MZT2B | 0.29816028 | 7.38761425 | 7.034497504 | 7.46E-12 |
| BRIP1 | 0.681568473 | 2.900389338 | 7.034475985 | 7.46E-12 |
| USP1 | 0.289884709 | 6.442747452 | 7.033219869 | 7.52E-12 |
| RP11-421M1.8 | -0.862769772 | -0.401831042 | -7.033183141 | 7.52E-12 |
| MEF2D | 0.497536903 | 7.451463425 | 7.032883362 | 7.53E-12 |
| RP11-565F19.2 | -0.544133062 | 1.60272098 | -7.032343986 | 7.56E-12 |
| PTCD2 | -0.368765608 | 2.53937502 | -7.030285124 | 7.66E-12 |
| IQGAP3 | 1.101726211 | 3.412307815 | 7.029820796 | 7.69E-12 |
| KB-1507C5.3 | -0.921446977 | -0.593741745 | -7.029046147 | 7.72E-12 |
| TMEM199 | 0.289639637 | 3.826092406 | 7.028987698 | 7.73E-12 |
| ESCO1 | 0.293114048 | 5.30217337 | 7.027979009 | 7.78E-12 |
| SNAI3 | -0.680888898 | 4.085102058 | -7.026963954 | 7.83E-12 |
| RP3-388E23.2 | -1.113897721 | -0.451823084 | -7.026391291 | 7.86E-12 |
| SUB1 | 0.31406491 | 6.762687967 | 7.023238317 | 8.02E-12 |
| ITPR1-AS1 | -0.868775737 | 1.292557368 | -7.02297377 | 8.03E-12 |
| ZBTB40 | -0.25118292 | 5.215398477 | -7.022572254 | 8.05E-12 |
| MED27 | 0.21048711 | 4.561673257 | 7.022396597 | 8.06E-12 |
| ACBD7 | 0.87424443 | -0.078495441 | 7.021966509 | 8.08E-12 |
| RP13-516M14.4 | 0.811179862 | 0.505482349 | 7.020371755 | 8.17E-12 |
| HIST1H2BD | -0.845562403 | 6.120969235 | -7.020045811 | 8.19E-12 |
| RP11-10O17.3 | -0.726526212 | 0.345975169 | -7.020006488 | 8.19E-12 |
| RP11-216P16.2 | -0.698540702 | 0.454627596 | -7.017315023 | 8.33E-12 |
| UBR1 | 0.25019338 | 4.542636375 | 7.01730071 | 8.33E-12 |
| RPAP2 | -0.374776314 | 4.066649785 | -7.017230866 | 8.34E-12 |
| DYNLL1P1 | -0.869315768 | -0.138294199 | -7.016873469 | 8.35E-12 |
| YTHDC1 | 0.266675919 | 6.633588643 | 7.015159928 | 8.45E-12 |
| RP5-914P20.5 | -0.520252816 | 1.380656331 | -7.013969886 | 8.51E-12 |
| RECQL | -0.328677254 | 5.786728887 | -7.013782718 | 8.52E-12 |
| RP11-1319K7.1 | -0.636468222 | 2.936684141 | -7.013674383 | 8.53E-12 |
| LTBR | -0.452338723 | 5.854989527 | -7.012840464 | 8.58E-12 |
| FKBP15 | -0.568861533 | 5.025330686 | -7.012672267 | 8.58E-12 |
| POM121B | 0.694170319 | 2.470049167 | 7.012053157 | 8.62E-12 |
| MAPK7 | 0.657963722 | 4.213521135 | 7.011678605 | 8.64E-12 |
| RP11-362F19.3 | -1.15981021 | -0.74864397 | -7.009693892 | 8.75E-12 |
| PDXDC2P | 0.528450171 | 2.157198811 | 7.009006384 | 8.79E-12 |
| EPDR1 | -1.025961101 | 4.790546962 | -7.008145774 | 8.84E-12 |
| LENG9 | -0.654190917 | 2.683632233 | -7.005616482 | 8.98E-12 |
| CFAP210 | 0.92087572 | 0.990830401 | 7.004864518 | 9.03E-12 |
| LPXN | -0.696002026 | 6.386813132 | -7.003990056 | 9.08E-12 |
| SPATA13 | 0.496268084 | 4.806114707 | 7.003317412 | 9.12E-12 |
| NCAPG | 0.934999963 | 3.776000615 | 7.001842363 | 9.20E-12 |
| URAD | -1.176191795 | 0.716818227 | -7.001792131 | 9.21E-12 |
| RP11-166N6.2 | -0.987224919 | -0.330600127 | -7.001692476 | 9.21E-12 |
| CCDC77 | -0.375223591 | 3.706341701 | -7.0007703 | 9.27E-12 |
| ACOT2 | 0.473712044 | 3.897403426 | 6.999969708 | 9.32E-12 |
| ATAD2 | 0.47946367 | 5.35909623 | 6.99912635 | 9.37E-12 |
| SDCBP2 | 0.637570455 | 1.383105209 | 6.998868851 | 9.38E-12 |
| NUDT5 | -0.430973494 | 5.662044799 | -6.998195495 | 9.42E-12 |
| SYCE1L | 0.536241164 | 2.221631727 | 6.99654655 | 9.52E-12 |
| ELF2 | 0.324192155 | 5.46430705 | 6.996317866 | 9.54E-12 |
| RP11-416A17.6 | -0.784589482 | 1.022468261 | -6.996255694 | 9.54E-12 |
| RP11-157J24.2 | -1.20277486 | 0.657199545 | -6.99305396 | 9.74E-12 |
| RCBTB2 | -0.717042524 | 4.705766003 | -6.992852573 | 9.75E-12 |
| ROM1 | 0.473008852 | 1.662395229 | 6.991655011 | 9.83E-12 |
| HMGN4 | -0.347607044 | 7.527000191 | -6.990388938 | 9.91E-12 |
| IDI1 | 0.415608811 | 6.655215187 | 6.990360547 | 9.91E-12 |
| RP11-1017G21.5 | -0.583316655 | 2.275907242 | -6.988383669 | 1.00E-11 |
| ZNF331 | 0.774893502 | 3.968088858 | 6.985554459 | 1.02E-11 |
| VPS16 | -0.209085019 | 5.806459702 | -6.985343933 | 1.02E-11 |
| EFCAB6 | -1.069838769 | -2.582760782 | -6.985307875 | 1.02E-11 |
| PCBP2 | 0.346259283 | 6.873363145 | 6.985252778 | 1.02E-11 |
| STAG3L4 | 0.420522896 | 4.571974531 | 6.984877156 | 1.03E-11 |
| CTD-2342J14.6 | -0.705436382 | 1.60605259 | -6.982696959 | 1.04E-11 |
| DOCK11 | -0.314986661 | 7.091168564 | -6.982693332 | 1.04E-11 |
| EML4 | 0.56669233 | 5.309340184 | 6.982126343 | 1.04E-11 |
| CDK2AP2 | -0.507510608 | 6.203562804 | -6.981372388 | 1.05E-11 |
| CGGBP1 | -0.205481283 | 7.333971864 | -6.980560129 | 1.06E-11 |
| NDUFV3 | -0.295826752 | 4.90185975 | -6.980416513 | 1.06E-11 |
| SNX27 | -0.484044281 | 5.145097818 | -6.980402217 | 1.06E-11 |
| IRGQ | 0.696289908 | 4.437183514 | 6.980036345 | 1.06E-11 |
| RP11-15E18.1 | -0.849222318 | -0.224603281 | -6.97962313 | 1.06E-11 |
| PLGLB2 | -1.015513619 | -0.619719078 | -6.9794475 | 1.06E-11 |
| RP11-1143G9.2 | -0.7486879 | -0.842032211 | -6.979009989 | 1.07E-11 |
| RP11-111M22.2 | -0.840710141 | 0.489494823 | -6.978991771 | 1.07E-11 |
| METTL25 | -0.297757687 | 2.499739117 | -6.978780814 | 1.07E-11 |
| ANKRD16 | -0.486632525 | 3.101889656 | -6.978333271 | 1.07E-11 |
| WDR52 | -0.591238838 | 2.85412301 | -6.978194172 | 1.07E-11 |
| VPS33B | 0.205993373 | 4.773344836 | 6.97812687 | 1.07E-11 |
| AC138655.4 | -0.846188211 | -1.016889077 | -6.975765932 | 1.09E-11 |
| GLIPR1L2 | -0.898604242 | -1.596419704 | -6.974906861 | 1.09E-11 |
| AC027612.1 | 0.396333323 | 2.279162097 | 6.974813193 | 1.10E-11 |
| INPP4A | -0.359894531 | 4.854151428 | -6.973997807 | 1.10E-11 |
| NUDT6 | -0.647979046 | 1.32252306 | -6.97379011 | 1.10E-11 |
| EIF4A1P10 | -0.348739686 | 8.73022002 | -6.972583635 | 1.11E-11 |
| C19orf73 | -0.689815028 | 0.92568284 | -6.971901336 | 1.12E-11 |
| MTRF1L | 0.394973471 | 3.440673987 | 6.970982064 | 1.12E-11 |
| RP11-59D5__B.2 | -1.093845296 | 0.550169498 | -6.970626411 | 1.13E-11 |
| TLK1P1 | 0.398617724 | 1.554954642 | 6.970082887 | 1.13E-11 |
| CTC-251D13.1 | -0.831305199 | 0.075768168 | -6.969691971 | 1.13E-11 |
| ADORA2A-AS1 | 1.265485039 | -1.860256062 | 6.969246055 | 1.14E-11 |
| RP11-313P22.1 | -0.785074411 | -0.019879814 | -6.968499312 | 1.14E-11 |
| RP11-171I2.5 | -0.943437334 | 2.424338143 | -6.967304408 | 1.15E-11 |
| PINK1 | -0.398743494 | 5.101979726 | -6.962911639 | 1.18E-11 |
| RP11-144O23.8 | -0.883646822 | -0.840078901 | -6.962779296 | 1.18E-11 |
| SLC9A8 | 0.425891554 | 5.021784149 | 6.962469367 | 1.19E-11 |
| ZFP64 | -0.441021122 | 3.051514393 | -6.961734384 | 1.19E-11 |
| LSM14A | 0.191575043 | 7.90723591 | 6.959414465 | 1.21E-11 |
| IBA57 | 0.451387831 | 2.910988651 | 6.959159339 | 1.21E-11 |
| RNF152 | 1.263649492 | -2.400996704 | 6.958839461 | 1.21E-11 |
| NT5C2 | -0.267043256 | 7.033051449 | -6.957582328 | 1.22E-11 |
| THBS1 | 1.912978082 | 4.024242291 | 6.955584943 | 1.24E-11 |
| ZNF761 | -0.320973686 | 4.030762202 | -6.955426261 | 1.24E-11 |
| CIAO1 | -0.193159771 | 6.832117506 | -6.953288653 | 1.26E-11 |
| TM7SF3 | -0.652451753 | 7.312244647 | -6.952025087 | 1.27E-11 |
| QRSL1 | -0.273744213 | 3.971778082 | -6.951011624 | 1.28E-11 |
| SFT2D2 | 0.36498228 | 6.034806908 | 6.950436314 | 1.28E-11 |
| DNAJB4 | 0.486681608 | 4.157538784 | 6.948588313 | 1.30E-11 |
| SULT1B1 | -1.093579068 | 3.330342326 | -6.94851196 | 1.30E-11 |
| BRD9 | 0.245540898 | 4.594633523 | 6.946863437 | 1.31E-11 |
| RP5-867C24.4 | 0.775756163 | 0.615283117 | 6.945795987 | 1.32E-11 |
| EXOC4 | -0.336771251 | 5.290950948 | -6.945546594 | 1.32E-11 |
| CDT1 | 0.710504532 | 5.64043188 | 6.94438213 | 1.33E-11 |
| RP11-137L10.6 | -0.401910655 | 3.868356695 | -6.941115651 | 1.36E-11 |
| RP11-512M8.3 | 0.764387572 | 0.003292908 | 6.940958992 | 1.36E-11 |
| AIP | -0.295268013 | 6.968814927 | -6.940017001 | 1.37E-11 |
| ZMPSTE24 | -0.329132674 | 5.585697162 | -6.939422214 | 1.37E-11 |
| MED16 | -0.326262555 | 5.813165344 | -6.938385509 | 1.38E-11 |
| SDHAF1 | -0.406499672 | 4.765892797 | -6.938309824 | 1.38E-11 |
| DHRS4 | -0.335814856 | 5.097257731 | -6.937390108 | 1.39E-11 |
| MCM3AP-AS1 | -0.762930673 | 1.059276045 | -6.936768842 | 1.40E-11 |
| EXD2 | -0.548281252 | 3.573295357 | -6.936064322 | 1.40E-11 |
| CTBP1 | -0.221041428 | 6.261913585 | -6.935856361 | 1.41E-11 |
| INHBA | 1.546393745 | 1.608678748 | 6.934586955 | 1.42E-11 |
| AP006621.1 | -0.700777902 | 2.897986098 | -6.934541716 | 1.42E-11 |
| RP11-298I3.4 | -0.921330975 | -0.196838961 | -6.933540165 | 1.43E-11 |
| RALGAPA1 | 0.305213696 | 2.929714271 | 6.932036472 | 1.44E-11 |
| RP11-440L14.1 | -0.488171641 | 3.675801988 | -6.931927646 | 1.44E-11 |
| HINFP | -0.316289167 | 3.310315967 | -6.931869572 | 1.44E-11 |
| PPP2CB | -0.312337913 | 4.673188233 | -6.931846438 | 1.44E-11 |
| ARID4B | 0.264613372 | 5.508641735 | 6.930316279 | 1.46E-11 |
| MRPL37P1 | 0.991550588 | -0.187781672 | 6.929605532 | 1.46E-11 |
| IFT172 | -0.455696413 | 3.977476012 | -6.928707203 | 1.47E-11 |
| TMEM168 | -0.32585981 | 3.738457102 | -6.928486375 | 1.47E-11 |
| CCDC174 | 0.265604095 | 5.05694869 | 6.928169009 | 1.48E-11 |
| TBX6 | -0.725940959 | 1.333214864 | -6.927126586 | 1.49E-11 |
| IGSF8 | -0.417855818 | 5.231024472 | -6.925368846 | 1.50E-11 |
| RP11-1391J7.1 | 0.829510649 | 0.977441362 | 6.924029251 | 1.52E-11 |
| FAM169A | 1.203789306 | 1.300403804 | 6.923862926 | 1.52E-11 |
| DDX6 | 0.264904865 | 6.816419111 | 6.922463609 | 1.53E-11 |
| AC003682.16 | -0.788217215 | -0.265151596 | -6.92206668 | 1.54E-11 |
| ZNF14 | 0.814345435 | 3.167762757 | 6.921666676 | 1.54E-11 |
| SPATA2 | 0.546225459 | 4.727102691 | 6.920887045 | 1.55E-11 |
| MRI1 | -0.392441124 | 5.687012115 | -6.920511738 | 1.55E-11 |
| CTD-2561J22.2 | -0.742531033 | 2.885432038 | -6.919361878 | 1.56E-11 |
| TMEM192 | -0.297449526 | 4.332668063 | -6.918368282 | 1.57E-11 |
| RP11-701P16.1 | 1.368190873 | 2.887195792 | 6.917961637 | 1.58E-11 |
| CAPN1 | -0.295832604 | 7.022085015 | -6.917169503 | 1.58E-11 |
| RP11-412D9.4 | -0.856365551 | -0.122808006 | -6.916686825 | 1.59E-11 |
| SPTY2D1-AS1 | -0.784473529 | -0.625668121 | -6.912033688 | 1.64E-11 |
| C19orf12 | -0.341417541 | 4.01906633 | -6.911961451 | 1.64E-11 |
| MLF2 | 0.299656088 | 7.493525523 | 6.910019743 | 1.66E-11 |
| RN7SL333P | -0.773845508 | 0.466574998 | -6.909713783 | 1.66E-11 |
| CXCL16 | 1.484110678 | 4.840513999 | 6.909304664 | 1.67E-11 |
| C6orf3 | -0.713347347 | 2.212163717 | -6.908938272 | 1.67E-11 |
| FBXO11 | 0.22028155 | 6.393100376 | 6.907670301 | 1.68E-11 |
| SLC35B4 | -0.440172456 | 4.049827095 | -6.907134242 | 1.69E-11 |
| FLAD1 | -0.23822867 | 5.048896864 | -6.906563412 | 1.69E-11 |
| RP1-168P16.2 | 0.895283812 | 1.2786752 | 6.905118936 | 1.71E-11 |
| MFN2 | 0.243649043 | 6.777594374 | 6.905088201 | 1.71E-11 |
| FAM126B | 0.313412641 | 4.536280912 | 6.903873361 | 1.72E-11 |
| ERRFI1 | 1.157854135 | 0.153247686 | 6.903322409 | 1.73E-11 |
| AL590560.1 | 0.73808437 | 3.269015838 | 6.902712404 | 1.74E-11 |
| WDR77 | -0.254251072 | 4.729153681 | -6.901691667 | 1.75E-11 |
| RP5-901A4.1 | -0.83155624 | -0.680297127 | -6.901597202 | 1.75E-11 |
| CSNK2B | 0.2843773 | 5.874396154 | 6.901372369 | 1.75E-11 |
| ELMOD3 | -0.280619578 | 3.713201765 | -6.901319382 | 1.75E-11 |
| SF3B2 | 0.176027542 | 7.195129792 | 6.898662643 | 1.78E-11 |
| PSMG2 | 0.308198061 | 5.9899195 | 6.898448866 | 1.78E-11 |
| TMEM14B | 0.300217047 | 4.995547724 | 6.896667217 | 1.80E-11 |
| CTC-241N9.1 | -0.733082351 | 1.83372945 | -6.896419628 | 1.81E-11 |
| AREG | 1.759146022 | 2.51082849 | 6.895877782 | 1.81E-11 |
| NMD3 | 0.309449009 | 5.950895389 | 6.895527752 | 1.82E-11 |
| PASK | 0.406299574 | 3.753799805 | 6.89370959 | 1.84E-11 |
| ADCY7 | -0.512435796 | 6.676604912 | -6.892678197 | 1.85E-11 |
| ATM | -0.44774913 | 5.526432786 | -6.890868154 | 1.87E-11 |
| RBM11 | 1.156591778 | -0.114486221 | 6.889499494 | 1.89E-11 |
| ZFP41 | -0.435175625 | 1.885892108 | -6.889496416 | 1.89E-11 |
| RP11-379F4.4 | -0.709532342 | -1.7320484 | -6.889094728 | 1.89E-11 |
| ZFP14 | -0.449873808 | 2.578173898 | -6.888371167 | 1.90E-11 |
| RIT1 | 0.537893906 | 6.726178927 | 6.886793609 | 1.92E-11 |
| THAP11 | -0.302806814 | 6.216424797 | -6.884401069 | 1.95E-11 |
| RP11-22B23.1 | 0.463344457 | 4.000319126 | 6.884258751 | 1.95E-11 |
| GNPDA2 | 0.374156376 | 3.519578897 | 6.882901388 | 1.97E-11 |
| FKBP3 | -0.313462409 | 6.614615298 | -6.8825062 | 1.97E-11 |
| WIPI2 | 0.239547316 | 5.619843027 | 6.880826734 | 2.00E-11 |
| RN7SL749P | 0.973614724 | -0.236037403 | 6.880021452 | 2.01E-11 |
| CGRRF1 | 0.452730607 | 3.147929473 | 6.880018389 | 2.01E-11 |
| RUSC1-AS1 | -0.626792825 | 1.346951691 | -6.879871113 | 2.01E-11 |
| FAM199X | 0.270517177 | 5.542601853 | 6.878081096 | 2.03E-11 |
| TAPT1 | 0.343089652 | 5.123529784 | 6.876848567 | 2.05E-11 |
| ANKRD63 | -1.053403842 | 0.825743466 | -6.876259071 | 2.05E-11 |
| SLFN12 | -0.808768692 | 3.233942763 | -6.876257001 | 2.05E-11 |
| RP1-111B22.3 | -0.752031781 | -0.352268703 | -6.876024955 | 2.06E-11 |
| COG5 | -0.312396863 | 4.652301672 | -6.875155233 | 2.07E-11 |
| SLC30A6 | -0.301810927 | 4.328030118 | -6.874767269 | 2.07E-11 |
| WDR76 | 0.547577308 | 4.290213931 | 6.874599324 | 2.08E-11 |
| SPAG1 | 0.70615228 | 3.062134639 | 6.873986546 | 2.08E-11 |
| RNU6ATAC18P | 0.938799144 | -0.228256978 | 6.873826536 | 2.09E-11 |
| WDR46 | -0.272511306 | 6.071141366 | -6.872150091 | 2.11E-11 |
| ZNF324 | 0.290002828 | 3.574410662 | 6.872080916 | 2.11E-11 |
| MKRN9P | 0.529641894 | 2.2844923 | 6.870275681 | 2.13E-11 |
| RPS6KA1 | -0.363056969 | 6.931659476 | -6.869439977 | 2.15E-11 |
| PKD1P6 | 0.383743955 | 4.166854199 | 6.869024198 | 2.15E-11 |
| ZNF766 | 0.376682263 | 4.997171885 | 6.86881605 | 2.15E-11 |
| NTAN1 | 0.448637901 | 5.205422959 | 6.867169396 | 2.18E-11 |
| SLC35B1 | 0.322302558 | 4.704041796 | 6.866173702 | 2.19E-11 |
| SLC39A11 | -0.592550285 | 4.501885762 | -6.86512367 | 2.21E-11 |
| KRT8P33 | 0.459950948 | 2.665680779 | 6.865022505 | 2.21E-11 |
| AURKB | 0.894025604 | 4.176433237 | 6.863638578 | 2.23E-11 |
| METTL10 | 0.323605656 | 3.210133501 | 6.863572113 | 2.23E-11 |
| RP11-554I8.1 | -0.925495622 | 1.177262576 | -6.863004088 | 2.24E-11 |
| SSFA2 | 0.374776537 | 6.650548752 | 6.861162549 | 2.26E-11 |
| ETNK2 | 1.040181669 | -0.648254576 | 6.860364624 | 2.27E-11 |
| GTF3C4 | 0.331822799 | 4.688328332 | 6.858840823 | 2.30E-11 |
| CD302 | -0.606056876 | 7.390303797 | -6.858363164 | 2.30E-11 |
| SEPT7P2 | 0.403041625 | 4.254941621 | 6.857614741 | 2.31E-11 |
| CUL2 | 0.203157107 | 4.958649902 | 6.857307828 | 2.32E-11 |
| ZNF740 | -0.319826983 | 5.612683595 | -6.855444716 | 2.34E-11 |
| ERCC6L | 0.824392764 | 1.633828113 | 6.854143206 | 2.36E-11 |
| LYPD3 | 0.846367352 | 0.569401134 | 6.853177617 | 2.38E-11 |
| AL109806.1 | 0.628529099 | 0.976439502 | 6.852919682 | 2.38E-11 |
| ELL2P1 | 0.811785909 | 2.971332072 | 6.852381412 | 2.39E-11 |
| DHX36 | 0.271662548 | 4.835417258 | 6.852021217 | 2.40E-11 |
| FBXL4 | -0.210111891 | 4.269369504 | -6.851919392 | 2.40E-11 |
| PEAK1 | 0.437336989 | 4.518425638 | 6.850404246 | 2.42E-11 |
| CMC2 | 0.315672441 | 4.49884135 | 6.850388772 | 2.42E-11 |
| RP11-16F15.2 | 0.437764713 | 2.462895057 | 6.848941825 | 2.44E-11 |
| RAF1 | 0.299770939 | 6.533181965 | 6.848470691 | 2.45E-11 |
| ZNF491 | -0.608037203 | 1.3658056 | -6.848321417 | 2.45E-11 |
| RP5-894D12.3 | -1.037527762 | -1.276727947 | -6.846696164 | 2.48E-11 |
| RP11-409C19.2 | 0.40449012 | 2.795466179 | 6.845348178 | 2.50E-11 |
| ZC3H15 | 0.284948524 | 7.395033419 | 6.845300806 | 2.50E-11 |
| PARN | -0.222300708 | 5.899095275 | -6.845289184 | 2.50E-11 |
| ARL10 | -0.836965838 | 1.893312908 | -6.844389565 | 2.51E-11 |
| ZSWIM6 | -0.505842956 | 5.052574332 | -6.843216708 | 2.53E-11 |
| PSMD9 | 0.423139705 | 2.296553686 | 6.842791768 | 2.54E-11 |
| USP6NL | 0.400423073 | 4.012831242 | 6.84210179 | 2.55E-11 |
| RWDD1 | 0.297710173 | 4.867683673 | 6.841169186 | 2.57E-11 |
| SEMA3B | 0.851713462 | -0.133640142 | 6.840222796 | 2.58E-11 |
| FAM200A | -0.393768028 | 2.963961021 | -6.839346846 | 2.60E-11 |
| SOCS6 | 0.624619201 | 4.11251058 | 6.838563845 | 2.61E-11 |
| ZNF837 | -0.589935252 | 2.126967064 | -6.837309826 | 2.63E-11 |
| XXbac-B444P24.14 | -0.831290197 | -0.688454635 | -6.83622478 | 2.65E-11 |
| RTN3P1 | -0.51719549 | 7.215775322 | -6.83599533 | 2.65E-11 |
| GATC | -0.307423861 | 5.434229711 | -6.834967467 | 2.67E-11 |
| HM13 | -0.279526364 | 6.632593827 | -6.834911816 | 2.67E-11 |
| AC127904.2 | 0.743810876 | 3.094634561 | 6.834073009 | 2.68E-11 |
| DHRS7B | -0.455477541 | 2.868231801 | -6.834024838 | 2.69E-11 |
| RNA5SP283 | -0.879930996 | 2.888250784 | -6.832077162 | 2.72E-11 |
| ACBD4 | -0.392341711 | 3.77798972 | -6.831595654 | 2.73E-11 |
| NCOR1 | -0.292130617 | 6.584559838 | -6.831206658 | 2.73E-11 |
| RP11-182I10.3 | -0.830051346 | -1.369557602 | -6.830595123 | 2.74E-11 |
| RP11-196G11.2 | -0.647373141 | 2.227175173 | -6.827829883 | 2.79E-11 |
| CDC6 | 0.792251059 | 3.99300226 | 6.827466845 | 2.80E-11 |
| TCP10L | -0.821954273 | -0.318935728 | -6.827226081 | 2.80E-11 |
| RP11-242D8.3 | -0.694315351 | 2.007485036 | -6.826974327 | 2.81E-11 |
| CCDC134 | 0.35274874 | 4.494979841 | 6.826845925 | 2.81E-11 |
| MAD2L1 | 0.583083984 | 3.525539277 | 6.825896173 | 2.83E-11 |
| ST6GALNAC4P1 | -0.741811678 | 0.011504191 | -6.825636909 | 2.83E-11 |
| HECA | 0.523666464 | 6.502153369 | 6.824878932 | 2.85E-11 |
| RP11-134K13.4 | -0.65252046 | 1.207089611 | -6.824449724 | 2.85E-11 |
| TSPAN10 | -0.949833541 | -1.755550999 | -6.824289367 | 2.86E-11 |
| ZNF81 | -0.329099855 | 3.337089721 | -6.824252066 | 2.86E-11 |
| LEO1 | -0.481497745 | 5.166713681 | -6.823890484 | 2.86E-11 |
| MATN1-AS1 | 0.60133721 | 1.72518556 | 6.822723 | 2.88E-11 |
| SLC16A4 | -0.684637403 | -1.347429328 | -6.822065931 | 2.90E-11 |
| ZFHX2 | -0.897605991 | -0.720597787 | -6.821249675 | 2.91E-11 |
| BZW1P1 | 0.597153893 | 0.693927824 | 6.819798979 | 2.94E-11 |
| TOPBP1 | 0.224169138 | 6.525942509 | 6.819194503 | 2.95E-11 |
| PNPO | -0.595673296 | 3.829466928 | -6.818852462 | 2.96E-11 |
| ANP32BP1 | 0.335036476 | 4.85049736 | 6.814600841 | 3.04E-11 |
| RP11-495P10.1 | -0.825589547 | -1.918262451 | -6.814277882 | 3.04E-11 |
| GALNT2 | -0.35046568 | 6.210362278 | -6.814127438 | 3.04E-11 |
| PARP6 | 0.326633364 | 5.80004176 | 6.813180817 | 3.06E-11 |
| DRAXIN | -0.738371808 | -0.733454648 | -6.812859832 | 3.07E-11 |
| NDEL1 | 0.390672261 | 5.564581291 | 6.812665601 | 3.07E-11 |
| TRAPPC8 | -0.19918895 | 5.331533617 | -6.812269929 | 3.08E-11 |
| SEPSECS | 0.330084557 | 3.971800919 | 6.810334993 | 3.12E-11 |
| ANKRD17 | -0.312212171 | 6.093299513 | -6.810311276 | 3.12E-11 |
| RUFY1 | -0.403327935 | 5.871030033 | -6.810036496 | 3.12E-11 |
| NR1I2 | -1.065921678 | 1.058090002 | -6.808344634 | 3.16E-11 |
| FNTAL1 | -0.461183519 | 1.432911956 | -6.808076345 | 3.16E-11 |
| ABCA1 | 0.905584594 | 3.709990674 | 6.807904534 | 3.17E-11 |
| NF2 | 0.216847664 | 4.428085727 | 6.806829311 | 3.19E-11 |
| TCAP | 0.557443982 | 2.001437334 | 6.806785127 | 3.19E-11 |
| ULK4 | 0.470348754 | 2.50811673 | 6.806669053 | 3.19E-11 |
| REST | 0.315885615 | 6.13263079 | 6.806603174 | 3.19E-11 |
| TRIM33 | 0.210349468 | 5.847745929 | 6.802095067 | 3.28E-11 |
| SYNGR3 | 0.964111238 | -1.201163956 | 6.799986926 | 3.33E-11 |
| FXYD7 | 1.015358655 | -0.491716606 | 6.798491033 | 3.36E-11 |
| SNORD62B | 0.528833047 | 3.796252078 | 6.798325589 | 3.36E-11 |
| RP11-166O4.5 | -0.71813082 | 1.136746656 | -6.797617479 | 3.38E-11 |
| RNU2-22P | -0.796396571 | -0.267790356 | -6.797103433 | 3.39E-11 |
| RP11-750H9.5 | -0.669335314 | 7.220979767 | -6.797060782 | 3.39E-11 |
| PLOD1 | -0.563067858 | 5.475210679 | -6.796551575 | 3.40E-11 |
| ZBTB12 | -0.832909449 | 3.882156764 | -6.796544643 | 3.40E-11 |
| RP11-553K23.2 | -0.619701546 | 0.793760287 | -6.796338293 | 3.41E-11 |
| SLC33A1 | -0.239234003 | 4.056295554 | -6.796093182 | 3.41E-11 |
| SZRD1 | 0.189384019 | 7.5744147 | 6.794734766 | 3.44E-11 |
| RP5-1061H20.4 | -0.937541833 | 0.342472298 | -6.794224556 | 3.45E-11 |
| PLCXD2 | 0.827691622 | 1.670104597 | 6.794145127 | 3.45E-11 |
| RP11-356B19.11 | -0.858279493 | -0.150439664 | -6.793663573 | 3.46E-11 |
| TBK1 | 0.320669261 | 5.496847471 | 6.788314854 | 3.58E-11 |
| ZBTB20 | 0.622854366 | 1.975785617 | 6.787745569 | 3.60E-11 |
| RP11-80A15.1 | -0.927335056 | -0.603452243 | -6.786301197 | 3.63E-11 |
| AGGF1P1 | -0.711768217 | -0.301843547 | -6.784371843 | 3.67E-11 |
| RNF14 | -0.380944958 | 4.316496751 | -6.783151912 | 3.70E-11 |
| MELK | 0.885995128 | 3.179377545 | 6.782676723 | 3.71E-11 |
| ANKRD44-IT1 | 0.85676739 | 2.82341347 | 6.781918153 | 3.73E-11 |
| PTGES | 1.413689692 | 0.020005552 | 6.780614479 | 3.76E-11 |
| PACRGL | -0.307206066 | 2.928964576 | -6.779816294 | 3.78E-11 |
| OTUD4 | 0.258642678 | 6.037883841 | 6.779774427 | 3.78E-11 |
| ZNF746 | 0.352073435 | 5.073319564 | 6.778324904 | 3.81E-11 |
| LAMP3 | 0.879171815 | -0.58697565 | 6.778307678 | 3.81E-11 |
| H1FX-AS1 | 0.688777746 | 2.556636419 | 6.77710454 | 3.84E-11 |
| VPS37B | 0.409601176 | 6.078381425 | 6.777043862 | 3.85E-11 |
| RPL34-AS1 | -0.766264384 | -1.598461384 | -6.776912864 | 3.85E-11 |
| CCDC154 | -0.574434843 | 2.932398465 | -6.776781939 | 3.85E-11 |
| MAPKAPK5-AS1 | 0.347896056 | 4.211025639 | 6.776420147 | 3.86E-11 |
| UTP3 | -0.419033668 | 6.541615513 | -6.775767356 | 3.88E-11 |
| NDST2 | 0.538413917 | 1.80154565 | 6.77535549 | 3.89E-11 |
| TMEM189 | 0.324906782 | 4.883789681 | 6.773889319 | 3.92E-11 |
| PRR14L | -0.276781654 | 5.5539916 | -6.773245874 | 3.94E-11 |
| MVB12A | -0.295975285 | 3.926308188 | -6.773221894 | 3.94E-11 |
| C1orf186 | -1.428009007 | 5.155134537 | -6.770352207 | 4.01E-11 |
| ZSCAN5A | -0.447917824 | 1.887097785 | -6.768683189 | 4.05E-11 |
| RNF19B | 0.556363604 | 6.91258203 | 6.767615778 | 4.08E-11 |
| PUM2 | -0.242402534 | 6.970886352 | -6.766851805 | 4.10E-11 |
| KIAA2013 | -0.292156317 | 6.165217704 | -6.766713783 | 4.10E-11 |
| SPEN | 0.224341442 | 7.004900298 | 6.76613053 | 4.12E-11 |
| DDX28 | 0.344431997 | 5.372831167 | 6.765013889 | 4.15E-11 |
| RBM27 | 0.229221163 | 5.819392636 | 6.764941816 | 4.15E-11 |
| BCL9 | 0.785396454 | 3.555023856 | 6.763961245 | 4.17E-11 |
| CTD-2547E10.3 | -0.792898021 | -0.228694175 | -6.762453614 | 4.21E-11 |
| SLC2A3 | 0.997964785 | 7.786922485 | 6.762041297 | 4.22E-11 |
| ERCC4 | -0.37772085 | 2.552711794 | -6.760241656 | 4.27E-11 |
| STAG3L5P-PVRIG2P-PILRB | 0.67617555 | -0.778036405 | 6.757266843 | 4.35E-11 |
| ZSCAN30 | -0.435100999 | 3.931486428 | -6.757198107 | 4.35E-11 |
| FAM132A | 0.798495633 | 1.23850791 | 6.756423884 | 4.38E-11 |
| FMNL1 | 0.378257792 | 8.37870492 | 6.755461976 | 4.40E-11 |
| DISP2 | -0.889028435 | -0.561161543 | -6.753954728 | 4.44E-11 |
| TAS2R3 | -0.941770403 | -0.589016407 | -6.752813137 | 4.48E-11 |
| AC139712.2 | -0.743576694 | -1.388317724 | -6.752465291 | 4.49E-11 |
| PDZD11 | -0.313444092 | 4.559097475 | -6.751263663 | 4.52E-11 |
| OPN3 | -0.607652838 | 4.764857491 | -6.751137033 | 4.52E-11 |
| RP11-285J16.1 | -0.920643594 | -0.306072418 | -6.750877371 | 4.53E-11 |
| UPRT | 0.200269163 | 4.727640906 | 6.750300869 | 4.55E-11 |
| LINC00959 | -0.611066395 | 0.905061544 | -6.749121838 | 4.58E-11 |
| ZNF75D | -0.269893363 | 4.047059528 | -6.749009297 | 4.58E-11 |
| TMCO6 | -0.305776888 | 3.856973991 | -6.748333855 | 4.60E-11 |
| NIF3L1 | -0.411412947 | 4.845951262 | -6.747613246 | 4.62E-11 |
| SP6 | 0.972490213 | -1.998037955 | 6.746869216 | 4.65E-11 |
| ZNF767 | -0.462925153 | 4.772488052 | -6.746839963 | 4.65E-11 |
| PITPNA-AS1 | -0.340759061 | 4.030665982 | -6.74672725 | 4.65E-11 |
| RP11-715J22.2 | -0.817991065 | -2.898141305 | -6.744066765 | 4.73E-11 |
| RP11-394O4.3 | -0.755434665 | -0.017521106 | -6.740967434 | 4.82E-11 |
| RP1-121G13.3 | 0.393589919 | 2.777732431 | 6.74031787 | 4.84E-11 |
| MATN1 | 1.061266782 | -0.898880642 | 6.739816305 | 4.86E-11 |
| FKBP7 | -0.641563847 | 2.885942636 | -6.739326616 | 4.87E-11 |
| C1orf216 | -0.474391049 | 4.728985746 | -6.738498019 | 4.90E-11 |
| CHD7 | 0.872495217 | 4.246234952 | 6.737320469 | 4.93E-11 |
| YTHDF1 | 0.219897183 | 6.614599929 | 6.737252226 | 4.93E-11 |
| BOD1 | -0.362310037 | 4.881938648 | -6.737063111 | 4.94E-11 |
| RP11-442N24__B.1 | -0.754833171 | -0.715675273 | -6.736017896 | 4.97E-11 |
| MSANTD2 | -0.486041418 | 2.340575369 | -6.735955133 | 4.97E-11 |
| APOBEC3D | -0.626019056 | 3.892222843 | -6.73522043 | 5.00E-11 |
| SFMBT2 | 0.678658719 | 4.329286199 | 6.734625837 | 5.02E-11 |
| SLC25A32 | 0.293797895 | 5.1955453 | 6.734086028 | 5.03E-11 |
| AIM2 | -1.130474263 | 2.0013444 | -6.733059901 | 5.06E-11 |
| VAV1 | -0.313848374 | 6.796270594 | -6.732502294 | 5.08E-11 |
| XIRP1 | 1.374907791 | -3.244854736 | 6.732452303 | 5.08E-11 |
| SDAD1P2 | -0.496071858 | 1.186309727 | -6.731564697 | 5.11E-11 |
| COLGALT1 | -0.417467778 | 7.126289274 | -6.730665409 | 5.14E-11 |
| BX936347.1 | -0.594906712 | 3.516370114 | -6.730521508 | 5.15E-11 |
| RP1-257A7.5 | -0.615631895 | 2.084555967 | -6.72990644 | 5.17E-11 |
| CD180 | -1.406893421 | 4.405302907 | -6.729291673 | 5.19E-11 |
| PTS | 0.517440553 | 4.057387648 | 6.728488232 | 5.21E-11 |
| RP1-102E24.8 | -0.735887339 | 2.167558594 | -6.727578201 | 5.24E-11 |
| YIPF1 | -0.242315146 | 5.501072857 | -6.726259427 | 5.28E-11 |
| LINC00907 | -0.863119266 | -2.810848713 | -6.72433348 | 5.35E-11 |
| PCBD2 | -0.345405754 | 2.958531807 | -6.722559711 | 5.41E-11 |
| RP11-656D10.3 | -0.633050148 | 1.585149504 | -6.722442098 | 5.41E-11 |
| RP11-121L10.3 | 0.486151088 | 3.500801034 | 6.721733858 | 5.44E-11 |
| TNFSF13 | -0.533090412 | 4.529698009 | -6.721425974 | 5.45E-11 |
| SNX2 | -0.311680751 | 6.664853911 | -6.720880039 | 5.46E-11 |
| RP11-682N22.1 | -0.756834851 | -0.758455948 | -6.72083451 | 5.47E-11 |
| RP3-402G11.25 | 0.778102312 | 1.349370964 | 6.718980708 | 5.53E-11 |
| SMEK1 | 0.186916598 | 7.0565017 | 6.718447063 | 5.55E-11 |
| GET4 | 0.458697337 | 2.855124304 | 6.717979627 | 5.56E-11 |
| LACE1 | -0.448326888 | 1.560692736 | -6.717493079 | 5.58E-11 |
| RP11-177C12.1 | -0.495826197 | 3.078555569 | -6.717404923 | 5.58E-11 |
| RP11-295G24.5 | -0.772918495 | 0.115595886 | -6.716992133 | 5.60E-11 |
| MRPL3 | -0.328365923 | 6.699772805 | -6.71533154 | 5.66E-11 |
| RELT | 0.523253315 | 6.304565997 | 6.70975438 | 5.86E-11 |
| ATF1 | -0.376683458 | 5.388168811 | -6.708150569 | 5.92E-11 |
| GPN3 | -0.427075515 | 4.755989753 | -6.707454286 | 5.94E-11 |
| PMS2CL | 0.35560199 | 4.034151001 | 6.705499273 | 6.01E-11 |
| DIAPH3 | 0.953495839 | 2.047989711 | 6.704602706 | 6.05E-11 |
| KLF9 | 1.077474947 | 5.32753377 | 6.704479627 | 6.05E-11 |
| RP11-378A13.1 | -0.497375169 | 3.579856279 | -6.702312027 | 6.14E-11 |
| MAMDC2-AS1 | -1.0463831 | -3.080800811 | -6.70171021 | 6.16E-11 |
| MIR623 | 0.817721901 | 0.843716538 | 6.70110981 | 6.18E-11 |
| CTD-2342N23.3 | -0.908347075 | -0.844663015 | -6.699363332 | 6.25E-11 |
| RNF41 | -0.220974821 | 6.027668372 | -6.698986586 | 6.26E-11 |
| TPT1P10 | -0.678118336 | 0.63628472 | -6.694882732 | 6.43E-11 |
| TMEM158 | 1.114935885 | 0.889604765 | 6.693882274 | 6.47E-11 |
| AC135178.1 | -0.80862501 | 3.535182618 | -6.693824043 | 6.47E-11 |
| KCNC4-AS1 | -0.818184357 | -0.532471506 | -6.693574962 | 6.48E-11 |
| U91328.19 | 0.509422461 | 3.662241884 | 6.692816206 | 6.51E-11 |
| ZC3H13 | -0.407314189 | 5.560083747 | -6.692339053 | 6.53E-11 |
| CYP4V2 | -0.727131256 | 3.05589346 | -6.691441233 | 6.56E-11 |
| UBE2CP2 | -0.800342899 | -0.200561063 | -6.691302437 | 6.57E-11 |
| DYRK1A | 0.251314407 | 6.509446599 | 6.690882846 | 6.59E-11 |
| ZNF202 | -0.358393285 | 3.73152843 | -6.689149542 | 6.66E-11 |
| PIN4P1 | -0.519640357 | 3.547105316 | -6.68787118 | 6.71E-11 |
| TAF10 | 0.246438254 | 5.917352954 | 6.687834824 | 6.71E-11 |
| CDC40 | 0.281786746 | 5.115284432 | 6.686984756 | 6.75E-11 |
| RP11-473I1.9 | 0.605390738 | 6.957385584 | 6.686835093 | 6.76E-11 |
| ZNF689 | 0.296296179 | 4.525906774 | 6.686527034 | 6.77E-11 |
| YEATS2 | -0.271747947 | 5.501186785 | -6.68499189 | 6.83E-11 |
| RANBP2 | 0.359679083 | 6.593197201 | 6.684476323 | 6.85E-11 |
| SLC25A25 | 0.446622741 | 3.915946111 | 6.684221164 | 6.87E-11 |
| ASH1L | 0.240179514 | 5.616871827 | 6.683715867 | 6.89E-11 |
| FUNDC2P2 | 0.379707799 | 2.428978659 | 6.683685231 | 6.89E-11 |
| CCDC17 | 0.530708867 | 2.786335382 | 6.682715471 | 6.93E-11 |
| RRN3 | 0.428799198 | 5.860342108 | 6.680735374 | 7.02E-11 |
| GRASP | 1.376796868 | 4.668277856 | 6.678186079 | 7.13E-11 |
| PHTF1 | 0.406642234 | 4.935267359 | 6.676881439 | 7.19E-11 |
| RWDD2B | -0.585313376 | 3.537058945 | -6.675309579 | 7.26E-11 |
| MIR4746 | 0.746819623 | 1.109490166 | 6.6748261 | 7.28E-11 |
| MTIF2 | -0.230802307 | 5.870468043 | -6.674428992 | 7.30E-11 |
| USP8P1 | 0.485387708 | 1.367369012 | 6.674385496 | 7.30E-11 |
| PEX16 | 0.262849888 | 5.056727719 | 6.674134876 | 7.31E-11 |
| IFT46 | -0.365529314 | 3.64080634 | -6.674033423 | 7.31E-11 |
| KBTBD3 | -0.433647016 | 1.063263105 | -6.673381228 | 7.34E-11 |
| APOL2 | -0.437228887 | 5.19293939 | -6.672657252 | 7.38E-11 |
| GIT2 | -0.21809963 | 6.093628772 | -6.671209712 | 7.44E-11 |
| MIR3909 | 0.809270376 | 0.478931404 | 6.670371225 | 7.48E-11 |
| RP11-192H23.6 | 0.757157334 | -0.260049487 | 6.669588036 | 7.52E-11 |
| BAD | 0.232635107 | 5.39962233 | 6.666365834 | 7.67E-11 |
| GABARAPL3 | 0.783240429 | -1.456580658 | 6.665973585 | 7.69E-11 |
| CTD-3199J23.6 | -0.716371251 | 0.808865006 | -6.665258793 | 7.72E-11 |
| PPP4C | 0.203870542 | 6.953261125 | 6.664721223 | 7.75E-11 |
| SH3BGRL | -0.305967745 | 9.100607901 | -6.663150284 | 7.82E-11 |
| RP11-373N22.3 | -0.795500166 | -0.695840505 | -6.662931222 | 7.84E-11 |
| DCAF17 | -0.271634299 | 4.426835328 | -6.662720302 | 7.85E-11 |
| ENDOG | -0.44964435 | 4.900406606 | -6.66231835 | 7.87E-11 |
| ELP4 | -0.336949364 | 4.557199151 | -6.662151284 | 7.87E-11 |
| MRPL22 | 0.330070411 | 4.171820439 | 6.661179559 | 7.92E-11 |
| ABHD3 | -0.398652429 | 4.704387186 | -6.660946287 | 7.93E-11 |
| RP11-204L24.2 | -0.76297772 | 0.401423621 | -6.660414812 | 7.96E-11 |
| TMPPE | 0.431962603 | 3.742454208 | 6.659425784 | 8.01E-11 |
| UTP14A | -0.266509408 | 4.997264556 | -6.658996672 | 8.03E-11 |
| LINC01114 | -1.44465092 | -1.520496147 | -6.657305007 | 8.11E-11 |
| WDR90 | 0.49148192 | 3.778848673 | 6.657255965 | 8.12E-11 |
| FAM177A1 | 0.343985631 | 3.817917421 | 6.65713235 | 8.12E-11 |
| RANP4 | -0.789002044 | -0.167454579 | -6.65631134 | 8.16E-11 |
| ZDHHC18 | 0.563879314 | 5.7413772 | 6.654403949 | 8.26E-11 |
| SLC2A14 | 1.044579968 | 1.719638961 | 6.653579681 | 8.30E-11 |
| TBC1D15 | 0.299897608 | 5.533997171 | 6.653495391 | 8.31E-11 |
| POLR2H | -0.319234038 | 5.05104094 | -6.652864451 | 8.34E-11 |
| TMEM203 | -0.323837483 | 5.694189353 | -6.65265205 | 8.35E-11 |
| ZNF843 | -0.839023176 | -0.760624659 | -6.651034848 | 8.43E-11 |
| TBC1D5 | -0.342947154 | 6.021761817 | -6.648507889 | 8.57E-11 |
| RP11-339B21.13 | -0.689877442 | 0.294574483 | -6.646888368 | 8.65E-11 |
| RP11-61N20.3 | 0.435822384 | 4.937990964 | 6.645674563 | 8.72E-11 |
| PDCD2 | -0.233323968 | 5.661790118 | -6.6447098 | 8.77E-11 |
| RBM42 | 0.169045019 | 6.773846739 | 6.643999748 | 8.81E-11 |
| FNIP1 | 0.423868013 | 5.429266848 | 6.64394371 | 8.81E-11 |
| FAM160A1 | 1.256596116 | -1.953349875 | 6.643918535 | 8.81E-11 |
| GAB3 | -0.380777094 | 6.062034463 | -6.643651393 | 8.83E-11 |
| BRD8 | -0.387189422 | 5.057331683 | -6.643564373 | 8.83E-11 |
| EOGT | 0.343122601 | 4.417644635 | 6.64317916 | 8.85E-11 |
| NBR2 | -0.411420589 | 3.845673292 | -6.642027439 | 8.92E-11 |
| CARD9 | -0.850679359 | 4.902909051 | -6.639889278 | 9.04E-11 |
| BACH1 | 0.457264084 | 6.382026069 | 6.637755733 | 9.16E-11 |
| AL136419.6 | 0.440890085 | 1.4483295 | 6.637642151 | 9.16E-11 |
| RP11-693J15.5 | -1.228523334 | -3.941369056 | -6.636765766 | 9.21E-11 |
| RP11-448A19.1 | -0.547738338 | 1.590243788 | -6.635854816 | 9.26E-11 |
| AP1S1 | -0.506195278 | 5.307496019 | -6.633499655 | 9.40E-11 |
| TXLNB | -0.999386267 | 1.692205106 | -6.63300468 | 9.43E-11 |
| ING2 | 0.323299653 | 6.129676808 | 6.631974474 | 9.49E-11 |
| TSSC4 | 0.253224903 | 5.592129707 | 6.63146099 | 9.52E-11 |
| DARS | -0.270247909 | 6.629908896 | -6.631214243 | 9.53E-11 |
| RABEP1 | -0.270694266 | 5.687520168 | -6.630883351 | 9.55E-11 |
| MKI67 | 1.045959298 | 5.375142434 | 6.629496234 | 9.64E-11 |
| EIF2S1 | -0.340961548 | 5.657103715 | -6.628748887 | 9.68E-11 |
| FOXD4 | -0.831206502 | -1.353486849 | -6.62800696 | 9.72E-11 |
| TADA2A | -0.326717609 | 4.036283044 | -6.627820549 | 9.74E-11 |
| FAM104A | 0.234352055 | 6.394816715 | 6.627122971 | 9.78E-11 |
| PRMT5 | -0.368029199 | 6.233513874 | -6.627024172 | 9.78E-11 |
| PLA2G12A | -0.400769368 | 3.737248454 | -6.626837041 | 9.79E-11 |
| RPS6KB2 | 0.209791214 | 5.107263063 | 6.625938161 | 9.85E-11 |
| LSM3 | 0.234812615 | 5.583515509 | 6.625268848 | 9.89E-11 |
| ABHD17C | 0.961869538 | 3.069842198 | 6.625185707 | 9.89E-11 |
| CYP2T2P | -0.970538894 | 0.918512302 | -6.623514496 | 1.00E-10 |
| AC022431.1 | -0.287619332 | 10.78980297 | -6.623021374 | 1.00E-10 |
| TOP2B | -0.391224372 | 8.39756923 | -6.622860215 | 1.00E-10 |
| CLTB | 0.263022639 | 6.417098619 | 6.621292625 | 1.01E-10 |
| SIRT1 | 0.280734756 | 5.320151044 | 6.620547824 | 1.02E-10 |
| AKR7A3 | 0.692909109 | 1.266643159 | 6.619351891 | 1.03E-10 |
| ZNF570 | 0.404897832 | 2.717732324 | 6.618043855 | 1.03E-10 |
| PIGV | -0.39208032 | 4.020313529 | -6.617659055 | 1.04E-10 |
| RPS4XP16 | -0.453434371 | 3.2070687 | -6.615987912 | 1.05E-10 |
| SLC25A33 | 0.538856158 | 3.449151908 | 6.615857576 | 1.05E-10 |
| SRSF6 | -0.359794422 | 8.777493714 | -6.615005916 | 1.05E-10 |
| RP11-517B11.7 | -0.443931023 | 1.712269984 | -6.614899891 | 1.05E-10 |
| PDCD11 | -0.392604711 | 5.41243688 | -6.613505195 | 1.06E-10 |
| DDR2 | 0.784374603 | -1.065997869 | 6.612606485 | 1.07E-10 |
| ZBTB10 | 0.737759045 | 5.269406575 | 6.612034283 | 1.07E-10 |
| NOP56 | -0.395752822 | 6.771230634 | -6.611769072 | 1.07E-10 |
| SEC16B | -0.915577424 | -3.951460374 | -6.611721379 | 1.08E-10 |
| TTI1 | -0.33315396 | 4.807099589 | -6.610715768 | 1.08E-10 |
| MZF1 | -0.343349782 | 4.817059581 | -6.610216934 | 1.09E-10 |
| DUS1L | 0.199190456 | 6.025784899 | 6.609873671 | 1.09E-10 |
| NCAPG2 | 0.526884804 | 4.636341496 | 6.608946137 | 1.09E-10 |
| C16orf98 | -0.546062971 | 3.094923202 | -6.608619917 | 1.10E-10 |
| SMAD3 | 0.537608779 | 5.304555958 | 6.607608644 | 1.10E-10 |
| RP3-368A4.6 | -0.651143394 | 3.804402733 | -6.606828211 | 1.11E-10 |
| EXOSC3P1 | -0.938574918 | -0.131022861 | -6.606802088 | 1.11E-10 |
| TMEM179B | -0.26614303 | 7.056992975 | -6.605718574 | 1.12E-10 |
| LAMTOR5 | 0.243881421 | 6.953975899 | 6.603773269 | 1.13E-10 |
| GZF1 | 0.482199128 | 5.102201698 | 6.601955035 | 1.14E-10 |
| JADE1 | 0.305984919 | 5.173009863 | 6.601499366 | 1.14E-10 |
| U2AF1 | 0.40327865 | 6.600042488 | 6.601301153 | 1.15E-10 |
| SMC5-AS1 | -0.782195069 | 0.489628958 | -6.600043909 | 1.16E-10 |
| MED12 | -0.341388748 | 6.295043223 | -6.599587861 | 1.16E-10 |
| METTL20 | -0.484655172 | 0.123747023 | -6.599072877 | 1.16E-10 |
| MKNK2 | 0.375742198 | 7.291274851 | 6.598949002 | 1.16E-10 |
| JKAMP | -0.246928697 | 5.308248876 | -6.597180021 | 1.18E-10 |
| FOXJ3 | 0.235632874 | 6.247575113 | 6.595874602 | 1.19E-10 |
| CROCCP3 | -0.618571564 | 1.77033855 | -6.595576967 | 1.19E-10 |
| RBM33 | 0.236666351 | 6.435143537 | 6.595349713 | 1.19E-10 |
| MAFF | 1.220418836 | 5.445689592 | 6.594866632 | 1.19E-10 |
| PRR7 | 0.663870278 | 5.074152597 | 6.594580931 | 1.19E-10 |
| PRDX3 | -0.457461796 | 6.219351455 | -6.59414478 | 1.20E-10 |
| CCDC84 | 0.397336785 | 4.790909985 | 6.59347601 | 1.20E-10 |
| NKRF | 0.2772213 | 4.672442192 | 6.591990755 | 1.21E-10 |
| EFTUD1 | -0.289023367 | 4.816228829 | -6.591862951 | 1.21E-10 |
| RABL2A | -0.371162836 | 3.679908591 | -6.591167311 | 1.22E-10 |
| CDC73 | 0.205168797 | 5.529164029 | 6.59112057 | 1.22E-10 |
| RP11-430B1.2 | 0.898288477 | 0.359846968 | 6.590247396 | 1.23E-10 |
| CTD-3234P18.2 | -0.914277927 | 0.364200791 | -6.590199829 | 1.23E-10 |
| AL353898.2 | 0.735530109 | 1.693330769 | 6.587305686 | 1.25E-10 |
| DHX33 | -0.311599465 | 4.640154631 | -6.582739401 | 1.28E-10 |
| EFCAB14P1 | -0.464522254 | 3.135156034 | -6.582614619 | 1.29E-10 |
| HEY1 | 1.242730909 | -1.562525851 | 6.58254695 | 1.29E-10 |
| BRPF1 | 0.298066058 | 6.11035492 | 6.582297182 | 1.29E-10 |
| LINC01132 | -0.831015937 | -0.714411559 | -6.581883342 | 1.29E-10 |
| CTDSP2 | -0.310782428 | 7.725248355 | -6.581641708 | 1.29E-10 |
| RRS1 | -0.398712287 | 5.267949917 | -6.578531906 | 1.32E-10 |
| RAD54L2 | 0.241964165 | 4.764459421 | 6.577888178 | 1.32E-10 |
| FES | -0.491789537 | 7.472391609 | -6.577732411 | 1.32E-10 |
| CREBRF | 0.481882386 | 4.698661258 | 6.577396223 | 1.33E-10 |
| COA6 | -0.335632064 | 4.874547045 | -6.577276212 | 1.33E-10 |
| PPP2R5D | -0.226898703 | 6.296765438 | -6.574999883 | 1.35E-10 |
| RP11-760D2.7 | -0.681972991 | 1.502824477 | -6.573658813 | 1.36E-10 |
| PELI1 | 1.025610998 | 6.451285044 | 6.573639554 | 1.36E-10 |
| RASGEF1B | 0.900498428 | 3.439232035 | 6.572471729 | 1.37E-10 |
| SRRT | 0.24942133 | 7.397662746 | 6.571882003 | 1.37E-10 |
| NPHP3-AS1 | 1.05784736 | -2.283807382 | 6.571545249 | 1.38E-10 |
| FGD3 | -0.389539155 | 6.281423403 | -6.571066815 | 1.38E-10 |
| EPC1 | 0.248493591 | 5.49428577 | 6.571046968 | 1.38E-10 |
| IRAK1 | 0.307801966 | 7.736360895 | 6.570897125 | 1.38E-10 |
| TBPL1 | 0.36871205 | 3.842394691 | 6.570403785 | 1.39E-10 |
| GPATCH8 | 0.202957334 | 5.942755255 | 6.570329009 | 1.39E-10 |
| RP11-114N19.3 | -0.674247711 | 0.761655533 | -6.5695752 | 1.39E-10 |
| RRP8 | 0.223715852 | 4.363856744 | 6.569342368 | 1.39E-10 |
| PRKRIRP2 | 0.649667392 | 1.203378546 | 6.56928432 | 1.40E-10 |
| AP3M2 | 0.390147857 | 3.580240743 | 6.568493767 | 1.40E-10 |
| CCNA2 | 0.866463945 | 5.071125172 | 6.568093802 | 1.41E-10 |
| STT3B | -0.346433777 | 8.18156559 | -6.56699769 | 1.42E-10 |
| CTRL | 0.788693672 | -1.491944483 | 6.566792381 | 1.42E-10 |
| RP3-354N19.3 | 0.95941093 | 1.105440357 | 6.566575851 | 1.42E-10 |
| ACSL5 | -0.365698759 | 6.152266004 | -6.564834951 | 1.43E-10 |
| NCR3LG1 | 0.846077082 | 2.159987956 | 6.564633887 | 1.44E-10 |
| SCARB2 | -0.357168466 | 6.271082331 | -6.564348249 | 1.44E-10 |
| CSRNP2 | -0.340706926 | 4.256261311 | -6.562948943 | 1.45E-10 |
| ANKRD46 | -0.428080911 | 2.202909897 | -6.562504781 | 1.45E-10 |
| SETD1B | 0.302814854 | 5.847600227 | 6.56209091 | 1.46E-10 |
| AC132872.2 | 1.067220894 | 1.580393713 | 6.561374082 | 1.46E-10 |
| SPEF2 | -0.622276525 | 1.680042732 | -6.560575948 | 1.47E-10 |
| XRN1 | 0.233736341 | 5.508288392 | 6.560274979 | 1.47E-10 |
| GGNBP2 | 0.291799328 | 6.018504539 | 6.559838456 | 1.48E-10 |
| IGKV1D-22 | -1.280864041 | -0.643474389 | -6.559216746 | 1.48E-10 |
| NAA38 | 0.295611147 | 4.920684892 | 6.558040067 | 1.49E-10 |
| PQLC1 | 0.360366811 | 5.485756632 | 6.557383973 | 1.50E-10 |
| TOR1B | -0.344851244 | 5.727949059 | -6.55637209 | 1.51E-10 |
| RP11-354E23.5 | 0.91180502 | 0.573031373 | 6.555250427 | 1.52E-10 |
| ARHGEF19 | -0.403492933 | 3.105202305 | -6.55446065 | 1.53E-10 |
| SMIM14 | -0.9008977 | 3.252781303 | -6.554393547 | 1.53E-10 |
| SPCS2P4 | -0.398453163 | 6.425331189 | -6.553691836 | 1.54E-10 |
| RP11-6N17.4 | 0.482890779 | 1.152360126 | 6.553629075 | 1.54E-10 |
| UGCG | 0.876847227 | 5.330519489 | 6.553625753 | 1.54E-10 |
| C8orf76 | 0.488823492 | 1.791131082 | 6.553125843 | 1.54E-10 |
| ZNF613 | -0.556096902 | 2.527879911 | -6.552816582 | 1.54E-10 |
| KLHL24 | 0.439249142 | 5.421084538 | 6.552295456 | 1.55E-10 |
| RYBP | 0.358995824 | 5.751514772 | 6.552246453 | 1.55E-10 |
| PCGF5 | 0.461072844 | 5.246551083 | 6.551930065 | 1.55E-10 |
| ATP6AP1L | -0.661968867 | 0.789127846 | -6.550556959 | 1.56E-10 |
| RP11-1057B6.1 | -0.70457594 | 0.653850799 | -6.550504944 | 1.57E-10 |
| NTHL1 | -0.542934551 | 4.124119991 | -6.550052135 | 1.57E-10 |
| GDPD3 | -0.549202851 | 3.116616354 | -6.549838298 | 1.57E-10 |
| RP11-1094M14.11 | -0.340262661 | 3.889385707 | -6.549539818 | 1.57E-10 |
| METTL4 | -0.299088459 | 3.29030485 | -6.547218961 | 1.60E-10 |
| TRIM56 | -0.416806488 | 5.219923629 | -6.542978311 | 1.64E-10 |
| IFT80 | -0.500480049 | 3.410677795 | -6.542700069 | 1.64E-10 |
| CANX | -0.318141854 | 9.483872295 | -6.542246448 | 1.65E-10 |
| UBQLN1 | 0.217635099 | 7.159162865 | 6.542165847 | 1.65E-10 |
| RITA1 | -0.36827082 | 4.888974905 | -6.541007344 | 1.66E-10 |
| EZR | 0.487202646 | 7.842410312 | 6.540940461 | 1.66E-10 |
| RP11-295M18.6 | -0.868879723 | -0.319015395 | -6.539426665 | 1.68E-10 |
| RP11-87F15.2 | -0.475399489 | 1.883966624 | -6.537858854 | 1.69E-10 |
| CALCOCO2 | -0.438631148 | 6.854637868 | -6.537161801 | 1.70E-10 |
| SESN1 | 0.592980693 | 5.606894135 | 6.537117056 | 1.70E-10 |
| C1orf52 | 0.284912952 | 5.16918545 | 6.537033953 | 1.70E-10 |
| RP11-554J4.1 | -0.430463923 | 3.106703434 | -6.535967358 | 1.71E-10 |
| GAPDHP38 | 0.412508655 | 3.635031443 | 6.535054081 | 1.72E-10 |
| OTUD3 | 0.311803272 | 4.16535533 | 6.534352334 | 1.73E-10 |
| RAB1A | 0.331030951 | 7.535582776 | 6.532950202 | 1.74E-10 |
| PTPRC | -0.450252258 | 8.67544272 | -6.532800549 | 1.74E-10 |
| RP11-56B16.2 | -0.671519339 | -0.557538165 | -6.532588507 | 1.75E-10 |
| KRIT1 | -0.243154017 | 4.648475716 | -6.530482338 | 1.77E-10 |
| TWF1 | 0.512679303 | 5.525356439 | 6.530317749 | 1.77E-10 |
| CLEC16A | -0.219026333 | 4.573523323 | -6.528819047 | 1.79E-10 |
| GTF2H2C | -0.357757767 | 2.64807092 | -6.527566993 | 1.80E-10 |
| LATS2 | 0.477896902 | 5.983338358 | 6.526252742 | 1.82E-10 |
| BIRC3 | 0.668135175 | 4.788880461 | 6.52606564 | 1.82E-10 |
| TEX264 | -0.242945054 | 4.71303221 | -6.52421536 | 1.84E-10 |
| ATXN7 | 0.293805148 | 5.705564551 | 6.523491081 | 1.85E-10 |
| NUDT13 | -0.467427111 | 2.509116368 | -6.523420687 | 1.85E-10 |
| PIGUP1 | -0.661274825 | 2.655175511 | -6.521832151 | 1.86E-10 |
| SSNA1 | -0.24081941 | 7.12606559 | -6.520857884 | 1.88E-10 |
| GDF15 | 1.099857873 | 0.342170275 | 6.517684235 | 1.91E-10 |
| DR1 | 0.197917419 | 5.980326898 | 6.517492194 | 1.91E-10 |
| RAB40C | -0.297008214 | 5.047739526 | -6.514479147 | 1.95E-10 |
| LINC00663 | -0.702715321 | -0.169230228 | -6.513728401 | 1.96E-10 |
| STK24 | 0.437748483 | 6.551716416 | 6.51309101 | 1.97E-10 |
| ARIH1 | 0.21003563 | 6.05289213 | 6.507878616 | 2.03E-10 |
| NEK4 | -0.326190817 | 4.930267964 | -6.507768491 | 2.03E-10 |
| RP11-141B14.1 | -0.633315836 | 0.384693408 | -6.507768223 | 2.03E-10 |
| MED10 | 0.251671948 | 6.079171096 | 6.50756152 | 2.03E-10 |
| RP11-1415C14.4 | -0.622258137 | 2.351416 | -6.506430039 | 2.05E-10 |
| PDGFA | 1.179850165 | -0.203148528 | 6.505770155 | 2.06E-10 |
| INO80E | -0.292384947 | 5.250136264 | -6.505363386 | 2.06E-10 |
| IRAK3 | -0.485964416 | 6.896607076 | -6.504348638 | 2.07E-10 |
| RP11-59K5.1 | -0.434907606 | 5.044233649 | -6.50398656 | 2.08E-10 |
| AP000783.1 | 0.949385542 | 2.535453973 | 6.503464565 | 2.09E-10 |
| ZNF665 | -0.596194308 | 1.191810576 | -6.502153554 | 2.10E-10 |
| ZNF30 | -0.735534125 | 1.284243608 | -6.500694881 | 2.12E-10 |
| SF3A2 | 0.247446203 | 6.804793224 | 6.499583094 | 2.14E-10 |
| TMEM182 | -0.574803253 | 1.544596717 | -6.498695923 | 2.15E-10 |
| MRPL18 | -0.446624178 | 6.926310349 | -6.49847333 | 2.15E-10 |
| ZNF589 | -0.362329974 | 4.456154438 | -6.498233424 | 2.15E-10 |
| CENPU | 0.573892003 | 5.941069301 | 6.497411725 | 2.16E-10 |
| RP11-297N6.4 | -0.648774245 | 0.770499455 | -6.496605305 | 2.17E-10 |
| GUSBP5 | 0.816130644 | 0.707048017 | 6.496419767 | 2.18E-10 |
| ERI2 | -0.605490704 | 3.168110597 | -6.494054436 | 2.21E-10 |
| AGO3 | -0.205945052 | 5.086073109 | -6.493926712 | 2.21E-10 |
| RP11-511H9.3 | 0.794302144 | 3.077693829 | 6.492270515 | 2.23E-10 |
| XAF1 | -0.730501703 | 5.827721847 | -6.488370299 | 2.29E-10 |
| AK2 | -0.525134278 | 7.20800573 | -6.487788955 | 2.29E-10 |
| NMD3P1 | 0.485899034 | 1.05702971 | 6.487139743 | 2.30E-10 |
| RAB28 | -0.222914639 | 4.950428766 | -6.487072438 | 2.30E-10 |
| MRPL21 | 0.249911022 | 4.904102402 | 6.485241309 | 2.33E-10 |
| TGS1 | 0.255415413 | 5.107545761 | 6.48520812 | 2.33E-10 |
| CTB-50L17.7 | 0.564574174 | 2.363027565 | 6.484897816 | 2.33E-10 |
| MGA | -0.346269881 | 5.391072926 | -6.483163929 | 2.36E-10 |
| NELFA | 0.202258123 | 4.435553311 | 6.482544393 | 2.37E-10 |
| C7orf55-LUC7L2 | 0.344889531 | 5.435352091 | 6.481666052 | 2.38E-10 |
| RP11-134E15.2 | 0.231390544 | 4.920099532 | 6.481585641 | 2.38E-10 |
| RP11-333E13.2 | -0.320219249 | 8.654841727 | -6.480401064 | 2.40E-10 |
| IMPA2 | -0.443520504 | 5.895846002 | -6.479222165 | 2.42E-10 |
| METTL15 | -0.338737617 | 2.498781103 | -6.477961331 | 2.43E-10 |
| NRF1 | 0.214330921 | 4.716394068 | 6.477113045 | 2.45E-10 |
| UPP1 | 0.969152959 | 5.287171279 | 6.476959254 | 2.45E-10 |
| AKAP8L | 0.261041416 | 6.722086313 | 6.476868202 | 2.45E-10 |
| PRKACB | -0.490489455 | 6.390596511 | -6.474634699 | 2.48E-10 |
| VPS4B | -0.234829908 | 6.212295451 | -6.473638573 | 2.50E-10 |
| ARNT | -0.264115437 | 6.167934588 | -6.472964034 | 2.51E-10 |
| ARFIP2 | 0.241033262 | 5.691103168 | 6.472037327 | 2.52E-10 |
| MMP24-AS1 | 0.380258151 | 5.434966761 | 6.471938337 | 2.53E-10 |
| RCL1 | 0.439042767 | 4.370187059 | 6.470512593 | 2.55E-10 |
| MBLAC2 | -0.485310592 | 3.232590593 | -6.470239644 | 2.55E-10 |
| USP8 | 0.238047076 | 5.586389267 | 6.470146263 | 2.55E-10 |
| CCT3 | -0.376683548 | 7.479769764 | -6.469836865 | 2.56E-10 |
| DAZAP2P1 | 0.476583155 | 3.39798202 | 6.469741889 | 2.56E-10 |
| NDUFB9 | 0.356214699 | 7.511902323 | 6.469215047 | 2.57E-10 |
| LYG1 | -0.573512557 | 2.097476518 | -6.468676506 | 2.58E-10 |
| CLASRP | 0.236347765 | 6.446578338 | 6.468148092 | 2.58E-10 |
| HSPA4 | -0.309445557 | 6.626209977 | -6.467719805 | 2.59E-10 |
| NRP2 | 1.297610225 | -1.173452075 | 6.467356703 | 2.60E-10 |
| NOP9 | -0.269319678 | 5.646048375 | -6.466870325 | 2.60E-10 |
| ACBD3 | 0.217565382 | 6.269598504 | 6.466342497 | 2.61E-10 |
| LNPEP | -0.319855727 | 5.483178166 | -6.465883346 | 2.62E-10 |
| CSNK2A2 | 0.231030396 | 6.183165082 | 6.465416239 | 2.63E-10 |
| FER | -0.50509205 | 3.076767144 | -6.464948987 | 2.63E-10 |
| FAM109A | -0.563012343 | 3.523874987 | -6.464664816 | 2.64E-10 |
| DNAJC11 | -0.298183851 | 5.373614503 | -6.46433754 | 2.64E-10 |
| DGKZP1 | -0.298109276 | 7.993278872 | -6.464006581 | 2.65E-10 |
| FAM86C1 | -0.55939118 | 2.248460702 | -6.461745454 | 2.69E-10 |
| DENND6A | 0.281232167 | 5.552001032 | 6.461220952 | 2.69E-10 |
| RAB18 | 0.315979613 | 6.021739304 | 6.4609582 | 2.70E-10 |
| EDN1 | 0.958900184 | 0.601864271 | 6.460621039 | 2.70E-10 |
| RP11-689B22.2 | -0.678828614 | 0.567703462 | -6.459171325 | 2.73E-10 |
| GRK5-IT1 | 1.001034508 | 0.242737294 | 6.458734985 | 2.74E-10 |
| ASB7 | 0.253569203 | 4.69221049 | 6.457846974 | 2.75E-10 |
| KIAA0753 | -0.263139755 | 3.990474724 | -6.457478571 | 2.76E-10 |
| ZNF335 | 0.24703172 | 5.174893159 | 6.4567753 | 2.77E-10 |
| FCRL3 | -1.09955677 | 1.326886061 | -6.456333925 | 2.78E-10 |
| KLHL20 | 0.222250194 | 4.413598232 | 6.456227663 | 2.78E-10 |
| BBS12 | -0.692146858 | 1.083464912 | -6.455952016 | 2.78E-10 |
| TUBGCP5 | 0.311451777 | 4.095900015 | 6.453840745 | 2.82E-10 |
| CHST7 | 0.755561956 | 3.295267176 | 6.451922671 | 2.85E-10 |
| PRPS1 | 0.286895748 | 6.61757631 | 6.451749215 | 2.85E-10 |
| C17orf70 | -0.260160001 | 5.279799495 | -6.450081645 | 2.88E-10 |
| RP3-399L15.3 | -0.642649904 | -1.019796088 | -6.44940174 | 2.89E-10 |
| FAM115D | 0.952680493 | -2.216736628 | 6.448419654 | 2.91E-10 |
| ANKS1A | -0.381145153 | 4.769974439 | -6.448249593 | 2.91E-10 |
| ITGA4 | -0.504864052 | 7.709267988 | -6.448064344 | 2.92E-10 |
| CUL4B | -0.200884335 | 5.92099868 | -6.444587569 | 2.98E-10 |
| CDC45 | 0.780571568 | 4.541776894 | 6.443666581 | 3.00E-10 |
| C9orf89 | 0.472126585 | 6.653154617 | 6.443240435 | 3.00E-10 |
| FUT7 | -0.738565947 | 5.168801986 | -6.442548522 | 3.02E-10 |
| CDKN3 | 0.762110863 | 3.727432667 | 6.439483697 | 3.07E-10 |
| EIF1B | 0.380109725 | 7.70975522 | 6.439071577 | 3.08E-10 |
| ZNF205-AS1 | -0.600343406 | 1.408578753 | -6.436546815 | 3.13E-10 |
| MED28P1 | 0.725029952 | -0.472989453 | 6.436472006 | 3.13E-10 |
| ZFAND2A | 0.496151902 | 5.808732593 | 6.43610769 | 3.14E-10 |
| ZNF630 | -0.509999124 | 1.464627556 | -6.435134307 | 3.15E-10 |
| DTX4 | 1.009773447 | 3.089640038 | 6.434367912 | 3.17E-10 |
| KIAA1429 | 0.193016777 | 5.096064598 | 6.4334812 | 3.19E-10 |
| GFM2 | -0.223383202 | 4.875947327 | -6.433194744 | 3.19E-10 |
| NAA25 | 0.273051057 | 4.340846078 | 6.431531927 | 3.22E-10 |
| ZNF483 | 0.975505381 | -1.607304338 | 6.430773263 | 3.24E-10 |
| WIBG | -0.38335545 | 5.21886675 | -6.430429742 | 3.24E-10 |
| HAUS3 | 0.363644543 | 4.844003169 | 6.430429536 | 3.24E-10 |
| CSK | -0.324510677 | 7.418912581 | -6.429910248 | 3.26E-10 |
| DDX31 | -0.440402645 | 3.484006385 | -6.429902436 | 3.26E-10 |
| POFUT2 | 0.316163948 | 4.51226494 | 6.42955617 | 3.26E-10 |
| CHMP5 | -0.264919226 | 6.093659682 | -6.429390187 | 3.27E-10 |
| GAS2L3 | 1.032022001 | 1.342936553 | 6.42910377 | 3.27E-10 |
| ZNF530 | 0.398118235 | 2.105397209 | 6.428843473 | 3.28E-10 |
| BCL6B | 1.511558188 | -1.248345788 | 6.427036984 | 3.31E-10 |
| SCYL1 | 0.186708803 | 6.091474353 | 6.424873924 | 3.36E-10 |
| ZBTB32 | 0.950882775 | -0.511575492 | 6.424641247 | 3.36E-10 |
| AF146191.4 | -0.850156541 | -0.13587675 | -6.423926313 | 3.37E-10 |
| CUL4A | -0.196473751 | 6.549026754 | -6.42322222 | 3.39E-10 |
| ANAPC13 | -0.256144865 | 5.880105668 | -6.423207639 | 3.39E-10 |
| PMM1 | 0.72182529 | 2.716647243 | 6.421251224 | 3.43E-10 |
| RAPGEF1 | 0.379388046 | 6.826385626 | 6.419732249 | 3.46E-10 |
| CCDC102B | -0.647375637 | 0.63849564 | -6.419546216 | 3.46E-10 |
| NSUN3 | 0.245784843 | 4.033291932 | 6.418855191 | 3.48E-10 |
| CTC-365E16.1 | -0.446058851 | 3.092525836 | -6.4183704 | 3.49E-10 |
| SLC26A2 | -0.476889726 | 3.901611757 | -6.417961273 | 3.50E-10 |
| MED23 | 0.239762052 | 5.23279697 | 6.417805571 | 3.50E-10 |
| LRRC70 | 0.71833608 | 1.688769135 | 6.417423789 | 3.51E-10 |
| CTA-797E19.1 | 0.807018089 | 0.611564102 | 6.416621962 | 3.53E-10 |
| PITHD1 | 0.252197584 | 6.978504871 | 6.414163682 | 3.58E-10 |
| RP3-449M8.6 | -0.794887917 | -0.786950621 | -6.414148766 | 3.58E-10 |
| LOXL1 | 1.359070664 | 0.399813751 | 6.413986267 | 3.58E-10 |
| RAB6A | -0.23664957 | 6.693663454 | -6.41393955 | 3.58E-10 |
| SNX20 | -0.441663601 | 5.656444335 | -6.412805932 | 3.61E-10 |
| SLC25A16 | 0.261786481 | 3.537202573 | 6.412614043 | 3.61E-10 |
| SURF2 | -0.250359622 | 5.067841395 | -6.41254457 | 3.61E-10 |
| CTC-455F18.3 | 0.936118909 | -1.244464939 | 6.409918763 | 3.67E-10 |
| FOSL1 | 0.901568538 | 5.197591608 | 6.409360636 | 3.68E-10 |
| ECH1 | -0.272627143 | 6.634539893 | -6.40843623 | 3.70E-10 |
| AC007551.3 | -0.505211672 | 2.015052971 | -6.408352904 | 3.71E-10 |
| CTA-250D10.23 | 0.927065483 | 1.626087844 | 6.407565595 | 3.72E-10 |
| ALG3 | -0.305296819 | 5.213690554 | -6.406177101 | 3.75E-10 |
| SLC38A6 | -0.368004861 | 2.185430278 | -6.405416501 | 3.77E-10 |
| SNORD104 | -0.438981397 | 4.959811269 | -6.401397284 | 3.86E-10 |
| ADRB2 | -0.908248609 | 5.237932631 | -6.400379472 | 3.89E-10 |
| SFXN1 | 0.496654935 | 4.17776962 | 6.400369621 | 3.89E-10 |
| RPS6KA4 | -0.402853632 | 6.426090737 | -6.400156387 | 3.89E-10 |
| ZNF221 | -0.689228962 | 1.695728525 | -6.39976966 | 3.90E-10 |
| MB21D1 | 0.463905142 | 4.524665253 | 6.399755395 | 3.90E-10 |
| TBRG1 | -0.235466343 | 5.688867392 | -6.399464378 | 3.91E-10 |
| STARD10 | 0.641038095 | 2.840848457 | 6.399115441 | 3.92E-10 |
| ALYREF | 0.27780847 | 7.659669374 | 6.397538111 | 3.95E-10 |
| KDM2A | 0.209873013 | 7.196844021 | 6.395236773 | 4.01E-10 |
| TMX1 | -0.33840248 | 6.486674237 | -6.39404647 | 4.04E-10 |
| SUZ12 | 0.206550906 | 6.382435862 | 6.393114372 | 4.06E-10 |
| ITGA10 | -0.725829071 | -0.409424635 | -6.3919118 | 4.09E-10 |
| HECTD3 | -0.222848415 | 5.601186728 | -6.391675139 | 4.10E-10 |
| MAP2K7 | 0.185638017 | 5.57266115 | 6.391240481 | 4.11E-10 |
| HVCN1 | -0.871593226 | 4.755743518 | -6.390380335 | 4.13E-10 |
| NEK11 | -0.783637863 | 1.305519478 | -6.38835272 | 4.18E-10 |
| RB1CC1 | 0.308798404 | 6.074327683 | 6.388175686 | 4.18E-10 |
| RP11-328C8.4 | -0.757607006 | -1.937881721 | -6.388008382 | 4.19E-10 |
| RAB9A | 0.278729165 | 5.80864164 | 6.38591555 | 4.24E-10 |
| RPL21P11 | 0.459844179 | 1.788619182 | 6.38522749 | 4.26E-10 |
| RP13-1032I1.7 | 0.339837039 | 4.090292301 | 6.383410043 | 4.30E-10 |
| RP11-1415C14.3 | -0.502841837 | 3.657149161 | -6.38340811 | 4.30E-10 |
| ZRANB2-DT | -0.797412739 | -1.965650067 | -6.383255145 | 4.31E-10 |
| WDR93 | -0.956518528 | -1.8447257 | -6.382052369 | 4.34E-10 |
| FANCD2 | 0.425572134 | 4.271104849 | 6.38161605 | 4.35E-10 |
| RP11-267N12.3 | -0.527297407 | 0.486059573 | -6.381321845 | 4.36E-10 |
| RALGAPA1P | 0.282364616 | 4.010795162 | 6.380291477 | 4.39E-10 |
| TMEM5 | -0.320692934 | 3.671815206 | -6.379892874 | 4.40E-10 |
| EBLN2 | 0.448476213 | 3.813079267 | 6.379326114 | 4.41E-10 |
| ZNF846 | -0.315480859 | 3.58013881 | -6.378918628 | 4.42E-10 |
| SRP72P2 | 0.362552701 | 2.370510415 | 6.378643 | 4.43E-10 |
| TMEM107 | -0.456717941 | 3.461335596 | -6.377703493 | 4.45E-10 |
| VPS53 | -0.258689891 | 4.699950911 | -6.3771484 | 4.47E-10 |
| TNIP2 | 0.289979729 | 5.998469608 | 6.377120609 | 4.47E-10 |
| TWF1P1 | 0.465260419 | 5.548800876 | 6.376726339 | 4.48E-10 |
| RP11-382A20.7 | -0.83491781 | -0.047406261 | -6.376051583 | 4.50E-10 |
| RP11-820I16.4 | 0.639605919 | 2.032530582 | 6.375343575 | 4.52E-10 |
| AC114730.5 | 1.200919308 | -0.754790649 | 6.374904296 | 4.53E-10 |
| ZNF302 | -0.387679003 | 4.271055584 | -6.374411191 | 4.54E-10 |
| SRD5A3 | -0.421613724 | 4.299262379 | -6.374043159 | 4.55E-10 |
| PRKD2 | 0.363074573 | 5.842193528 | 6.373318841 | 4.57E-10 |
| DNAJB9 | 0.563992101 | 4.928433625 | 6.372566423 | 4.59E-10 |
| AIMP2 | -0.397910535 | 5.53068041 | -6.372314565 | 4.60E-10 |
| KPNA6 | 0.190706572 | 5.873547797 | 6.372150575 | 4.60E-10 |
| DISC1-IT1 | -0.777421584 | -0.609825114 | -6.371495859 | 4.62E-10 |
| KIAA0196 | -0.252960625 | 6.130176514 | -6.37116801 | 4.63E-10 |
| FNBP1 | -0.358224761 | 7.897866338 | -6.37114532 | 4.63E-10 |
| RP13-580F15.2 | -0.94340571 | 1.596664048 | -6.371058677 | 4.63E-10 |
| CTC-448F2.6 | -0.560518682 | 1.53129374 | -6.369361124 | 4.68E-10 |
| USP27X-AS1 | -0.633834865 | 0.279377376 | -6.3692525 | 4.69E-10 |
| RNASEH1-DT | -0.426381691 | 3.830582159 | -6.368901368 | 4.70E-10 |
| LTBP2 | 0.80060392 | -0.548220451 | 6.368486261 | 4.71E-10 |
| CALML4 | -0.555310337 | 3.551338557 | -6.367669928 | 4.73E-10 |
| RP11-110J1.2 | 0.213787143 | 4.356571566 | 6.36753144 | 4.73E-10 |
| AE000661.37 | -1.490910463 | 3.511815036 | -6.367223476 | 4.74E-10 |
| C9orf41 | -0.278314774 | 4.435429152 | -6.366779591 | 4.75E-10 |
| BTF3L4P2 | -0.248890867 | 5.592430557 | -6.366700124 | 4.76E-10 |
| SNF8 | 0.239190391 | 4.896018015 | 6.365684189 | 4.79E-10 |
| RAP1GDS1 | -0.271475448 | 5.284279323 | -6.364987203 | 4.81E-10 |
| LRTM2 | 0.9135109 | -3.364035693 | 6.364943522 | 4.81E-10 |
| TSC1 | -0.201234972 | 5.025321324 | -6.363481599 | 4.85E-10 |
| MAMSTR | -0.657009617 | -0.134429207 | -6.363279994 | 4.86E-10 |
| DNAJC16 | -0.290551685 | 3.860651353 | -6.360102502 | 4.95E-10 |
| FTH1P23 | 0.662237387 | 3.743475148 | 6.359240051 | 4.97E-10 |
| SPRED2 | 0.710625659 | 3.757037403 | 6.358185251 | 5.01E-10 |
| CCDC91 | -0.276810915 | 4.674975528 | -6.357371032 | 5.03E-10 |
| MUC1 | 0.644140046 | 4.112958763 | 6.357336322 | 5.03E-10 |
| ZNF134 | 0.523426594 | 5.314512256 | 6.355607475 | 5.08E-10 |
| PACSIN2 | -0.290662013 | 6.481308617 | -6.354865753 | 5.11E-10 |
| RMND5A | 0.235243109 | 6.251091392 | 6.354686528 | 5.11E-10 |
| BDNF-AS | -0.693811622 | 0.330076585 | -6.354104989 | 5.13E-10 |
| RP11-134G8.7 | -0.63986801 | 2.045090012 | -6.353374753 | 5.15E-10 |
| RGS17P1 | 0.642726077 | 3.800456993 | 6.353329732 | 5.15E-10 |
| CCT7 | -0.373014236 | 7.801038505 | -6.352702368 | 5.17E-10 |
| ARHGEF33 | -0.779980341 | -2.61055159 | -6.35048961 | 5.24E-10 |
| COX18 | -0.215326511 | 4.563752233 | -6.350072673 | 5.25E-10 |
| GFOD1 | 0.656661657 | 3.915135477 | 6.348974509 | 5.29E-10 |
| TOR4A | -0.493296217 | 5.221502379 | -6.34805486 | 5.32E-10 |
| AAMP | -0.196447224 | 6.512247226 | -6.34613095 | 5.38E-10 |
| CDO1 | 0.850693693 | -1.554753062 | 6.34477789 | 5.42E-10 |
| TBCCD1 | -0.221558829 | 4.396280572 | -6.344357695 | 5.44E-10 |
| RP11-490K7.4 | 0.505259407 | 2.129307225 | 6.343106114 | 5.48E-10 |
| SLC18A1 | -0.871837643 | -2.821484053 | -6.341501704 | 5.53E-10 |
| PDSS2 | -0.30537131 | 3.934023424 | -6.340679119 | 5.56E-10 |
| RP11-1094M14.7 | -0.424190561 | 4.13702823 | -6.33986693 | 5.58E-10 |
| RP11-214O1.2 | 0.860718067 | 2.07564254 | 6.339408597 | 5.60E-10 |
| RGS3 | -0.570068074 | 3.457685333 | -6.33882746 | 5.62E-10 |
| GYPC | 0.937762745 | 7.041321553 | 6.33771266 | 5.66E-10 |
| RP11-47I22.3 | 0.810972035 | -0.410307633 | 6.337653371 | 5.66E-10 |
| DTNB | 0.38679119 | 2.720926898 | 6.336800627 | 5.69E-10 |
| RBBP6 | 0.284409021 | 6.52119154 | 6.336393336 | 5.70E-10 |
| HMGB3P22 | -0.755288378 | 0.394344563 | -6.335917529 | 5.72E-10 |
| MLF1 | 1.118291748 | 0.505798339 | 6.335730007 | 5.72E-10 |
| RP4-564F22.5 | -0.775525293 | -0.258704648 | -6.334513203 | 5.76E-10 |
| CCS | -0.404692908 | 6.062958933 | -6.333268291 | 5.81E-10 |
| MKRN1 | 0.323079448 | 6.940442036 | 6.331971773 | 5.85E-10 |
| NR2C2 | 0.275442872 | 4.761152988 | 6.331691479 | 5.86E-10 |
| RP11-342D11.3 | -0.816805575 | -1.19420789 | -6.331307597 | 5.88E-10 |
| ZNF138 | -0.354378097 | 4.307385578 | -6.331216723 | 5.88E-10 |
| RP11-341G23.4 | -0.962797442 | 0.832815609 | -6.330196851 | 5.91E-10 |
| RP11-532M24.1 | 0.726419852 | -0.034114199 | 6.330096085 | 5.92E-10 |
| USP48 | -0.282211944 | 6.103977799 | -6.329256096 | 5.95E-10 |
| TRIM59 | 0.453583095 | 2.195002088 | 6.328895802 | 5.96E-10 |
| CENPM | 0.783382571 | 3.482025056 | 6.327679959 | 6.00E-10 |
| NCSTN | -0.262131514 | 7.674503449 | -6.327213126 | 6.02E-10 |
| GPR4 | 0.818937075 | -2.600444587 | 6.326986304 | 6.03E-10 |
| RP11-438L19.1 | -0.577609872 | 1.058047121 | -6.326067751 | 6.06E-10 |
| HCG25 | -0.568735724 | 1.002766453 | -6.326020756 | 6.06E-10 |
| CAT | -0.643635826 | 9.550969479 | -6.325752393 | 6.07E-10 |
| MYO10 | 1.188735053 | -0.757764085 | 6.32556207 | 6.08E-10 |
| STAC3 | -0.593487786 | 5.670634608 | -6.324612878 | 6.11E-10 |
| ITGB1P1 | -0.460680419 | 8.09867031 | -6.324362739 | 6.12E-10 |
| LRRC8A | 0.419743265 | 5.529354994 | 6.322778739 | 6.18E-10 |
| TNFRSF8 | -0.990352758 | 1.096088227 | -6.319989993 | 6.28E-10 |
| IPO7 | -0.328275321 | 6.590433262 | -6.31928518 | 6.31E-10 |
| MFI2 | 0.74008479 | 2.082202335 | 6.318293821 | 6.35E-10 |
| RP11-533E19.3 | 0.76083496 | 0.507102191 | 6.317985406 | 6.36E-10 |
| CTD-2017C7.2 | -0.683368116 | 1.304146111 | -6.317368439 | 6.38E-10 |
| LIF | 1.250383316 | 1.069478706 | 6.315932691 | 6.44E-10 |
| KPNA2 | 0.425926521 | 6.826110249 | 6.315029953 | 6.47E-10 |
| ACOX1 | -0.270507548 | 4.805593127 | -6.31430365 | 6.50E-10 |
| AKAP11 | -0.259582579 | 5.320432131 | -6.31182521 | 6.60E-10 |
| ANKRD53 | 0.775260416 | -0.87624038 | 6.311428588 | 6.61E-10 |
| PYCR2 | -0.280736799 | 5.671837137 | -6.310521863 | 6.65E-10 |
| NUPL1 | 0.288781715 | 5.367947816 | 6.310275 | 6.66E-10 |
| HSD17B7P2 | -0.742005423 | 3.072683555 | -6.309615144 | 6.68E-10 |
| RP11-566E18.3 | -0.511518341 | 3.02966355 | -6.309030368 | 6.71E-10 |
| CTC-228N24.3 | -0.388203553 | 3.142227258 | -6.308885987 | 6.71E-10 |
| CTB-78O21.1 | -0.889938641 | -0.76212662 | -6.308537719 | 6.73E-10 |
| FUNDC2 | 0.278849609 | 4.194923712 | 6.308265655 | 6.74E-10 |
| CRY1 | 0.644452512 | 4.028554184 | 6.307966561 | 6.75E-10 |
| LIG1 | 0.400154532 | 5.399104625 | 6.307559964 | 6.77E-10 |
| RP11-46A10.5 | -0.643193069 | 0.106605952 | -6.305477292 | 6.85E-10 |
| RBBP7 | 0.231615402 | 6.342930579 | 6.304867532 | 6.88E-10 |
| RABIF | -0.275688952 | 5.36024982 | -6.304845148 | 6.88E-10 |
| C6orf47 | -0.270069533 | 5.521991338 | -6.304589697 | 6.89E-10 |
| C2orf76 | -0.323776155 | 3.83564567 | -6.304134463 | 6.91E-10 |
| DPH2 | -0.35907467 | 5.166841391 | -6.303341533 | 6.94E-10 |
| SLC9A7P1 | -1.163321747 | 1.843627354 | -6.300638925 | 7.05E-10 |
| RPGRIP1L | -0.620373731 | 0.875414284 | -6.300594694 | 7.05E-10 |
| POFUT1 | -0.294303986 | 5.637453722 | -6.300392025 | 7.06E-10 |
| FBXL18 | 0.333940177 | 2.467580225 | 6.300023826 | 7.08E-10 |
| SPG20OS | -0.790501647 | 1.740238699 | -6.299058152 | 7.12E-10 |
| KCTD2 | 0.228303123 | 5.353996702 | 6.298935676 | 7.12E-10 |
| KIF23 | 0.762704654 | 3.662855204 | 6.298216104 | 7.15E-10 |
| CTD-3138B18.5 | -0.431471331 | 1.334537774 | -6.297697785 | 7.17E-10 |
| RP11-505K9.1 | 0.480527162 | 1.697757457 | 6.297407781 | 7.19E-10 |
| LSM10 | -0.304861936 | 5.786742321 | -6.295781648 | 7.26E-10 |
| TMEM57 | 0.342369981 | 5.851553146 | 6.291803156 | 7.43E-10 |
| NEDD1 | 0.209051111 | 5.127645852 | 6.291054596 | 7.46E-10 |
| CTD-2192J16.20 | -0.565690656 | -0.747237492 | -6.291046347 | 7.46E-10 |
| TRABD | 0.364308626 | 7.383967672 | 6.290801255 | 7.47E-10 |
| RNU6-322P | -0.75134795 | 1.767473447 | -6.290180244 | 7.50E-10 |
| ZNF175 | -0.550720197 | 3.427093926 | -6.289647314 | 7.52E-10 |
| RP11-649G15.2 | -0.616013206 | 0.128010423 | -6.288977847 | 7.55E-10 |
| C11orf21 | -0.575259077 | 6.860324578 | -6.288465326 | 7.58E-10 |
| B4GALT4 | 0.329404516 | 3.568614839 | 6.288251482 | 7.59E-10 |
| WIPF1 | -0.351815188 | 7.167653437 | -6.28789791 | 7.60E-10 |
| TGIF2P1 | -0.701750396 | -0.336712059 | -6.287710591 | 7.61E-10 |
| RAB5A | 0.234082409 | 6.599023726 | 6.287134821 | 7.64E-10 |
| OTUB1 | 0.165825619 | 5.995630444 | 6.286369779 | 7.67E-10 |
| AP003068.18 | -0.846520588 | 0.49739399 | -6.284534247 | 7.76E-10 |
| ITPR2 | -0.526789818 | 5.729419097 | -6.283246724 | 7.82E-10 |
| EPHA4 | 0.991845558 | 0.139686199 | 6.282932797 | 7.83E-10 |
| FAM78A | -0.355760459 | 6.546014854 | -6.282780852 | 7.84E-10 |
| HSPA8P1 | -0.488387787 | 2.275199148 | -6.282215085 | 7.86E-10 |
| SLK | 0.23905824 | 6.355328423 | 6.279207732 | 8.00E-10 |
| CISD1 | -0.403693616 | 3.072306372 | -6.278523261 | 8.04E-10 |
| CTC-459F4.6 | -0.62551887 | 1.284730311 | -6.278114712 | 8.06E-10 |
| MAPK6PS4 | 0.678492618 | -0.769149511 | 6.277140264 | 8.10E-10 |
| SLC35C2 | -0.264344389 | 6.190724671 | -6.276802023 | 8.12E-10 |
| BRI3BP | -0.652912706 | 5.040807319 | -6.27619671 | 8.15E-10 |
| MRPL35 | -0.350186474 | 4.396816565 | -6.275957019 | 8.16E-10 |
| ELOVL1 | -0.264559543 | 6.665752592 | -6.27554127 | 8.18E-10 |
| MAPK1 | -0.275755891 | 6.338012366 | -6.275340607 | 8.19E-10 |
| AOC4P | 0.778080025 | -2.883006735 | 6.274866579 | 8.21E-10 |
| ZNF736 | 0.433639898 | 4.273054805 | 6.274340047 | 8.24E-10 |
| RP11-888D10.3 | -0.886290205 | -0.35706921 | -6.273536474 | 8.28E-10 |
| DNASE1L2 | -0.728117704 | 0.585199323 | -6.272724331 | 8.32E-10 |
| PARP4P2 | -0.570671474 | 0.360594737 | -6.272195149 | 8.34E-10 |
| ODC1 | 0.518612669 | 7.844414896 | 6.272007041 | 8.35E-10 |
| MIR3916 | -0.354500454 | 7.543610666 | -6.271840201 | 8.36E-10 |
| CPSF2 | -0.419360703 | 5.425081552 | -6.271743386 | 8.37E-10 |
| PET100 | 0.392907635 | 3.90682189 | 6.271135553 | 8.40E-10 |
| CALHM2 | -0.805255984 | 4.54199981 | -6.271020041 | 8.40E-10 |
| BCAR1 | 1.136335688 | -3.803458635 | 6.270456381 | 8.43E-10 |
| DNAJC2 | 0.348487023 | 5.15045994 | 6.269963404 | 8.45E-10 |
| SUSD1 | -0.334018992 | 6.185714279 | -6.269396303 | 8.48E-10 |
| AC007560.1 | -0.400043567 | 3.761645291 | -6.268208784 | 8.54E-10 |
| CABLES2 | 0.281640359 | 4.087429753 | 6.267885171 | 8.56E-10 |
| SRSF7 | -0.314844064 | 7.744074802 | -6.267749016 | 8.57E-10 |
| SPTSSB | -0.861388877 | -2.723808272 | -6.266425056 | 8.63E-10 |
| ZFP3 | -0.715804266 | 2.940689727 | -6.264376964 | 8.74E-10 |
| A1BG | -0.773543964 | -1.535157126 | -6.264194779 | 8.75E-10 |
| LL22NC03-N27C7.1 | -0.569917933 | 3.525466054 | -6.263958243 | 8.76E-10 |
| UIMC1 | 0.314928549 | 5.739305117 | 6.263742349 | 8.77E-10 |
| ST20 | -0.509408691 | 3.270776497 | -6.262834935 | 8.82E-10 |
| CENPO | 0.377490279 | 3.876268075 | 6.258973899 | 9.02E-10 |
| LINC00662 | -0.351265207 | 2.293995936 | -6.258920717 | 9.02E-10 |
| CAD | -0.520571413 | 5.440549308 | -6.25763748 | 9.09E-10 |
| TMCO1 | -0.297825059 | 6.629022255 | -6.25733947 | 9.11E-10 |
| MED20 | -0.380284289 | 4.47198517 | -6.257240743 | 9.11E-10 |
| HECTD1 | 0.196210671 | 6.236548187 | 6.256579163 | 9.15E-10 |
| ZBTB42 | -0.506740206 | 2.707431719 | -6.255544953 | 9.20E-10 |
| RTN3 | -0.404240757 | 7.430740963 | -6.254880742 | 9.24E-10 |
| ZNF429 | -0.544261872 | 2.891795007 | -6.25394832 | 9.29E-10 |
| RP11-378J18.3 | -0.632758867 | -0.129978706 | -6.253037204 | 9.34E-10 |
| SMIM20 | -0.282839654 | 4.988418267 | -6.252087166 | 9.39E-10 |
| OPA1 | 0.221920923 | 6.030419927 | 6.251240978 | 9.44E-10 |
| SLC38A7 | -0.319720176 | 3.057942326 | -6.250551576 | 9.48E-10 |
| GOLGA5 | -0.228541268 | 5.737672802 | -6.250371958 | 9.49E-10 |
| CTD-2124B8.2 | -0.600837021 | 1.0666183 | -6.249872135 | 9.52E-10 |
| ABHD17A | 0.313357742 | 5.417703117 | 6.248429851 | 9.60E-10 |
| KCTD11 | -0.558872666 | 2.863416384 | -6.248371745 | 9.60E-10 |
| KRT10-AS1 | -0.769266768 | 0.579832093 | -6.24792989 | 9.63E-10 |
| SHQ1 | -0.352936463 | 3.720700271 | -6.247898217 | 9.63E-10 |
| TECRP1 | -0.38264376 | 6.721902465 | -6.246992673 | 9.68E-10 |
| CEP41 | -0.647327517 | 2.648261039 | -6.246709256 | 9.70E-10 |
| AKR1A1 | -0.347444267 | 6.397160057 | -6.246538938 | 9.71E-10 |
| FAM222B | 0.312478562 | 4.136132576 | 6.245623298 | 9.76E-10 |
| CSF3 | 1.123146162 | -2.17827634 | 6.245067213 | 9.79E-10 |
| RP11-493E12.2 | -0.693472283 | -0.48919332 | -6.243650688 | 9.87E-10 |
| MEX3C | 0.430278263 | 5.876684604 | 6.243279551 | 9.89E-10 |
| STK3 | -0.392884814 | 2.91134345 | -6.243244161 | 9.90E-10 |
| CENPA | 0.832270647 | 2.511179736 | 6.241753378 | 9.98E-10 |
| FAM133DP | 0.30054023 | 5.053866145 | 6.2376077 | 1.02E-09 |
| RP11-1277A3.3 | -0.739201466 | -0.478506954 | -6.237514509 | 1.02E-09 |
| RNF130 | -0.312068479 | 6.708671446 | -6.237461299 | 1.02E-09 |
| RP4-704D21.2 | -0.749590063 | -0.667135753 | -6.23550609 | 1.04E-09 |
| ARG2 | 0.908257436 | 1.566579967 | 6.235491759 | 1.04E-09 |
| OGT | -0.41970587 | 8.255027122 | -6.234827639 | 1.04E-09 |
| AC092835.2 | -0.59843759 | 1.464103732 | -6.234146406 | 1.04E-09 |
| PPP6C | 0.181239604 | 6.656957482 | 6.233610952 | 1.05E-09 |
| ZFPL1 | 0.204203547 | 5.628113479 | 6.233587616 | 1.05E-09 |
| SYNJ1 | 0.396405149 | 4.61860973 | 6.233433467 | 1.05E-09 |
| ALG12 | -0.183904793 | 4.184798332 | -6.233218525 | 1.05E-09 |
| TUG1 | -0.229674056 | 7.686999379 | -6.231906529 | 1.06E-09 |
| SSPN | -0.958443403 | -0.98555704 | -6.230930251 | 1.06E-09 |
| RNU1-60P | -0.88177112 | 4.822291853 | -6.228684008 | 1.08E-09 |
| CCBL2 | -0.255423718 | 5.622255714 | -6.227761596 | 1.08E-09 |
| MIR568 | 0.605941169 | 3.33263922 | 6.226775408 | 1.09E-09 |
| CNIH1 | -0.27698112 | 7.257005661 | -6.22512386 | 1.10E-09 |
| FLJ27365 | -0.858925522 | 1.266247503 | -6.225069895 | 1.10E-09 |
| RPAP1 | -0.29398444 | 4.10882071 | -6.224879959 | 1.10E-09 |
| TMEM237 | 0.466858735 | 2.160100805 | 6.224645154 | 1.10E-09 |
| TNFAIP3 | 0.927539342 | 7.759398773 | 6.224044179 | 1.11E-09 |
| ZNF420 | -0.440153796 | 2.219731576 | -6.223557977 | 1.11E-09 |
| OVGP1 | 0.676727604 | 2.33093302 | 6.222408708 | 1.12E-09 |
| CBX3P9 | -0.287146306 | 6.572580483 | -6.222332602 | 1.12E-09 |
| C1orf51 | 0.712266592 | -0.673986322 | 6.222019813 | 1.12E-09 |
| PAN3-AS1 | -0.656189909 | 4.402996326 | -6.22118481 | 1.13E-09 |
| ECE2 | -0.66353616 | 0.744213136 | -6.21819534 | 1.15E-09 |
| ZNF132 | -0.740892221 | 1.174181374 | -6.217891574 | 1.15E-09 |
| PSMC6 | 0.300422853 | 4.770742575 | 6.217618171 | 1.15E-09 |
| METTL12 | -0.529170949 | 0.678845988 | -6.216216852 | 1.16E-09 |
| NCKAP1 | 0.939073057 | -1.293642217 | 6.216165146 | 1.16E-09 |
| C3orf58 | 0.495669265 | 5.063037554 | 6.216043274 | 1.16E-09 |
| PRPSAP1 | -0.317547893 | 4.371106616 | -6.213890202 | 1.18E-09 |
| AC092574.2 | 0.711736514 | 0.884236391 | 6.213430397 | 1.18E-09 |
| STAM | 0.426945028 | 6.345637095 | 6.213230586 | 1.18E-09 |
| ZMYM3 | -0.462617269 | 5.700683429 | -6.212921188 | 1.18E-09 |
| LRRC47 | -0.196006095 | 6.132568523 | -6.212744589 | 1.18E-09 |
| UBE2M | 0.169002666 | 7.094276029 | 6.211860272 | 1.19E-09 |
| DLGAP4 | -0.334467864 | 4.963613195 | -6.208094482 | 1.22E-09 |
| RP11-394I13.1 | -0.749716101 | -2.283890367 | -6.20726753 | 1.22E-09 |
| SMG1P1 | 0.393162524 | 3.329078923 | 6.206838769 | 1.23E-09 |
| AGPAT1 | -0.318306465 | 6.355143028 | -6.206499903 | 1.23E-09 |
| TRMT2A | -0.207186395 | 5.872518556 | -6.20571863 | 1.23E-09 |
| HADHA | -0.250061572 | 8.424037343 | -6.205661051 | 1.23E-09 |
| AC007038.7 | -0.579848801 | 2.102723494 | -6.205443999 | 1.24E-09 |
| KB-1440D3.13 | -0.818519567 | 0.84486924 | -6.204110119 | 1.25E-09 |
| CCL28 | 0.825022152 | 2.888169941 | 6.203885508 | 1.25E-09 |
| C21orf128 | -1.240323773 | 0.14290353 | -6.203182695 | 1.25E-09 |
| LMAN2 | -0.277979083 | 8.066165141 | -6.20298387 | 1.25E-09 |
| ATP5EP2 | -0.30774984 | 7.252025715 | -6.202178963 | 1.26E-09 |
| C3orf33 | 0.434672596 | 2.550524587 | 6.20215853 | 1.26E-09 |
| ADPRH | -0.644389345 | 3.855817941 | -6.201874491 | 1.26E-09 |
| MOGS | -0.242756819 | 6.575877989 | -6.200927717 | 1.27E-09 |
| ARRDC5 | -0.577913495 | 3.310405116 | -6.200781794 | 1.27E-09 |
| RP11-642P15.1 | -0.56829929 | -1.056171254 | -6.200418402 | 1.27E-09 |
| FOXA1 | 0.946315779 | -3.320795608 | 6.200183938 | 1.27E-09 |
| NELFE | 0.155487619 | 6.722293373 | 6.199658261 | 1.28E-09 |
| COPS3 | 0.252260703 | 6.961312108 | 6.197926573 | 1.29E-09 |
| RP11-981G7.1 | -0.714283575 | -2.265019139 | -6.196878035 | 1.30E-09 |
| PPP1R11P1 | -0.561768363 | 1.000245867 | -6.19656426 | 1.30E-09 |
| RNF10 | 0.284959614 | 7.55974951 | 6.195939986 | 1.31E-09 |
| ATRN | -0.293426346 | 5.24496166 | -6.195179523 | 1.31E-09 |
| EXOSC3 | -0.31582294 | 4.0673753 | -6.195095963 | 1.31E-09 |
| PIGU | -0.444477856 | 4.387681186 | -6.19393538 | 1.32E-09 |
| EMC9 | 0.31696905 | 3.900214369 | 6.193698164 | 1.32E-09 |
| FTH1 | 0.427766992 | 11.31675709 | 6.19339285 | 1.33E-09 |
| INTS6 | -0.285900662 | 4.757553792 | -6.191794218 | 1.34E-09 |
| RP11-242D8.1 | -0.508940906 | 4.052672226 | -6.19111678 | 1.34E-09 |
| ENKD1 | -0.35280407 | 4.405275997 | -6.190252858 | 1.35E-09 |
| B3GNT1 | -0.610615997 | 2.954563854 | -6.189692724 | 1.35E-09 |
| TNFSF4 | -0.970715671 | 2.953514346 | -6.188229181 | 1.37E-09 |
| XXyac-YX65C7_A.2 | -0.898707814 | 0.731739611 | -6.188188153 | 1.37E-09 |
| USP7 | 0.179805283 | 7.179535909 | 6.188103277 | 1.37E-09 |
| HMGN3-AS1 | -0.617144597 | 1.16248651 | -6.188013732 | 1.37E-09 |
| DENND4A | 0.309021764 | 5.587038628 | 6.187634389 | 1.37E-09 |
| ACTR8 | -0.247969638 | 4.905371869 | -6.187430635 | 1.37E-09 |
| TMEM60 | -0.477341458 | 4.536498293 | -6.186910192 | 1.38E-09 |
| TIMM8B | -0.3586132 | 5.526679093 | -6.186535595 | 1.38E-09 |
| HIP1R | 0.68415989 | 3.692745321 | 6.186174721 | 1.38E-09 |
| SQSTM1 | 0.371933587 | 6.810215552 | 6.185645378 | 1.39E-09 |
| RP11-305E17.6 | -0.578390707 | 2.64101551 | -6.184213681 | 1.40E-09 |
| TNFRSF13C | 0.868370306 | 2.272879815 | 6.182188177 | 1.42E-09 |
| PRKRA | 0.275072652 | 5.696034766 | 6.180923499 | 1.43E-09 |
| CTD-3187F8.15 | -1.00317768 | -0.205027547 | -6.180290201 | 1.43E-09 |
| AURKC | 0.5596112 | 1.233661197 | 6.176926636 | 1.46E-09 |
| EXOSC7 | 0.252189953 | 3.936587576 | 6.176636967 | 1.46E-09 |
| GAPDH | 0.208725972 | 11.64904948 | 6.176526552 | 1.46E-09 |
| TNKS1BP1 | 0.486630773 | 3.524123236 | 6.17645446 | 1.46E-09 |
| SLC7A9 | -0.749338817 | -1.668770015 | -6.175458272 | 1.47E-09 |
| YY1 | 0.180717016 | 6.962381939 | 6.175049847 | 1.48E-09 |
| C14orf183 | -0.683199215 | -0.770657995 | -6.174514741 | 1.48E-09 |
| SETX | -0.20807308 | 6.587660322 | -6.172058378 | 1.50E-09 |
| AC007880.1 | -0.952811624 | -0.384780861 | -6.171436113 | 1.51E-09 |
| RGPD3 | 0.613383601 | 0.17860784 | 6.171195802 | 1.51E-09 |
| CYB5RL | -0.400088905 | 2.179896684 | -6.170900442 | 1.51E-09 |
| PTP4A2 | 0.271141336 | 8.455157621 | 6.169869523 | 1.52E-09 |
| POLE2 | 0.545380964 | 3.524350874 | 6.169490066 | 1.52E-09 |
| ACAP3 | -0.257988779 | 5.583680413 | -6.16839308 | 1.53E-09 |
| ZNF684 | 0.301035172 | 2.847998192 | 6.166302993 | 1.55E-09 |
| RNU6-583P | 0.671884894 | 0.046713281 | 6.166208106 | 1.55E-09 |
| RP11-483P21.2 | -0.917518865 | -0.117278495 | -6.166004583 | 1.56E-09 |
| RP4-548D19.3 | -0.588207253 | 2.76494072 | -6.165629182 | 1.56E-09 |
| ZNF552 | 0.343285055 | 3.675668567 | 6.165152469 | 1.56E-09 |
| YBX2 | 0.979050098 | -2.952570299 | 6.164630346 | 1.57E-09 |
| RP11-254B13.3 | -0.364224743 | 4.362421928 | -6.164186948 | 1.57E-09 |
| RP11-255N24.4 | -0.340526047 | 4.11675621 | -6.162885322 | 1.58E-09 |
| KANSL3 | 0.23747194 | 5.861003361 | 6.160919458 | 1.60E-09 |
| CAMKK2 | -0.360184446 | 6.314855854 | -6.159156617 | 1.62E-09 |
| TRMT10A | -0.377793962 | 2.329505521 | -6.159021601 | 1.62E-09 |
| RP11-251G23.5 | -0.482687551 | 1.060602167 | -6.157424091 | 1.64E-09 |
| NARG2P2 | -0.536245579 | -0.530954645 | -6.157394204 | 1.64E-09 |
| RPF1 | 0.220678321 | 7.0779653 | 6.15735873 | 1.64E-09 |
| YWHAG | -0.465690194 | 7.601154503 | -6.154694519 | 1.66E-09 |
| CDC42SE2 | 0.348221378 | 7.251464846 | 6.154263164 | 1.67E-09 |
| RP11-38J22.6 | -1.418906518 | 4.460354179 | -6.152686712 | 1.68E-09 |
| IFNAR2 | -0.356929394 | 5.551938464 | -6.152391511 | 1.68E-09 |
| BCAR3 | 0.914074048 | -0.461125115 | 6.151978062 | 1.69E-09 |
| UBA2 | 0.198835959 | 7.252907388 | 6.150476938 | 1.70E-09 |
| AC010336.1 | -0.777890782 | -2.518903358 | -6.150283375 | 1.71E-09 |
| SUMO2P1 | -0.429656432 | 8.768736229 | -6.149214679 | 1.72E-09 |
| HS1BP3 | -0.387787768 | 3.663633293 | -6.1481199 | 1.73E-09 |
| DGKK | -1.028844585 | -3.562643519 | -6.147579109 | 1.73E-09 |
| ZWINT | 0.689886806 | 5.469508815 | 6.147490234 | 1.73E-09 |
| ZNF28 | -0.493338055 | 3.125802611 | -6.147025011 | 1.74E-09 |
| PDCL3P4 | -0.321159521 | 4.547859626 | -6.146773596 | 1.74E-09 |
| ASB8 | 0.235252135 | 5.190923165 | 6.146117735 | 1.75E-09 |
| CHCHD1 | -0.274006194 | 4.891698421 | -6.145831867 | 1.75E-09 |
| ASH2L | -0.302104741 | 5.859473793 | -6.14335018 | 1.78E-09 |
| VILL | -0.303745996 | 4.128397359 | -6.143291735 | 1.78E-09 |
| GLYCTK | 0.394104977 | 3.199988578 | 6.143020193 | 1.78E-09 |
| ZDHHC23 | -0.656449902 | 3.060231331 | -6.142677971 | 1.78E-09 |
| ABHD6 | -0.63045687 | 2.026883874 | -6.142523205 | 1.78E-09 |
| C19orf55 | 0.241125801 | 3.695164388 | 6.141357093 | 1.80E-09 |
| DDHD1 | 0.337142974 | 3.625114058 | 6.140450096 | 1.81E-09 |
| PRKCG | 0.826824668 | -1.569003043 | 6.140223611 | 1.81E-09 |
| AL137127.1 | 0.614187391 | 1.221485023 | 6.139741393 | 1.81E-09 |
| MICB | 0.32502197 | 5.300822566 | 6.137415853 | 1.84E-09 |
| SMARCA2 | -0.3425162 | 7.286565909 | -6.136696509 | 1.85E-09 |
| CTD-2313J17.5 | -0.677445438 | 0.49181057 | -6.136440768 | 1.85E-09 |
| WDR41 | -0.296331929 | 4.98402877 | -6.135421876 | 1.86E-09 |
| CTD-2541M15.1 | -0.749075198 | 0.200877559 | -6.135243079 | 1.86E-09 |
| AC013394.2 | 0.552567893 | 6.894467459 | 6.134698746 | 1.87E-09 |
| SPSB2 | -0.39991662 | 3.347374354 | -6.134116033 | 1.87E-09 |
| MED15 | 0.195922188 | 5.9133023 | 6.134040608 | 1.87E-09 |
| FTH1P3 | 0.564323921 | 3.419356218 | 6.133126511 | 1.88E-09 |
| RP11-14I17.3 | -0.705783565 | -0.836770151 | -6.133069811 | 1.88E-09 |
| BOLA3 | 0.401583272 | 3.116532179 | 6.132089957 | 1.90E-09 |
| EIF2B3 | -0.362041283 | 4.025089609 | -6.132035442 | 1.90E-09 |
| SLFN11 | -0.554122209 | 5.25469167 | -6.131086388 | 1.91E-09 |
| RP11-498C9.12 | -0.947603322 | 0.124627571 | -6.130820305 | 1.91E-09 |
| SLC24A4 | -1.111817924 | 2.310487905 | -6.130708976 | 1.91E-09 |
| ZNF131 | 0.215937364 | 5.326607926 | 6.129887029 | 1.92E-09 |
| VCPKMT | 0.375825872 | 5.105608717 | 6.128886769 | 1.93E-09 |
| SAMD15 | -0.623817973 | 0.948403424 | -6.128631019 | 1.93E-09 |
| C2orf44 | -0.439044726 | 2.731077768 | -6.12808893 | 1.94E-09 |
| RABGAP1 | -0.282117675 | 5.0625034 | -6.126930489 | 1.95E-09 |
| FAM83F | 1.357569233 | -2.346666415 | 6.125412465 | 1.97E-09 |
| GANAB | -0.354059373 | 8.830058275 | -6.124703371 | 1.98E-09 |
| RP13-753N3.1 | -0.703462705 | -0.264346267 | -6.124185373 | 1.98E-09 |
| ELL2 | 0.764862629 | 4.625418296 | 6.121882928 | 2.01E-09 |
| ZNF860 | -0.82601145 | -2.136889966 | -6.12177578 | 2.01E-09 |
| NIPAL2 | -0.568696159 | 4.913627398 | -6.121628184 | 2.01E-09 |
| SAMHD1 | -1.085497331 | 7.276093455 | -6.121413523 | 2.02E-09 |
| MIR378I | 0.863768128 | 1.190336344 | 6.121120069 | 2.02E-09 |
| ZNF274 | 0.367778809 | 4.517347391 | 6.120468467 | 2.03E-09 |
| CTD-2132N18.2 | -0.644041442 | 1.89270248 | -6.119672885 | 2.04E-09 |
| APOE | 1.149274819 | 2.503448285 | 6.119141769 | 2.04E-09 |
| PABPN1L | 0.827691133 | 0.70150613 | 6.118899939 | 2.05E-09 |
| CSRNP1 | 0.807620253 | 8.186543051 | 6.118734829 | 2.05E-09 |
| RP11-175P19.2 | -0.686511323 | 0.016013977 | -6.118451478 | 2.05E-09 |
| C2orf68 | -0.215661 | 5.893368355 | -6.118174008 | 2.05E-09 |
| PRMT7 | -0.315428788 | 4.22546434 | -6.117732856 | 2.06E-09 |
| NSMF | 0.471770876 | 5.251251016 | 6.117311946 | 2.06E-09 |
| CAPNS2 | -0.668579589 | -0.041458109 | -6.116248894 | 2.08E-09 |
| VTA1 | -0.311617883 | 4.813032649 | -6.115815523 | 2.08E-09 |
| YBX1P6 | 0.494122669 | 2.931646061 | 6.114018857 | 2.10E-09 |
| LINC00327 | -0.924160622 | -1.242844504 | -6.114012616 | 2.10E-09 |
| RTN4IP1 | -0.347559029 | 2.559977298 | -6.11378011 | 2.11E-09 |
| RFX1 | -0.245395435 | 4.122174141 | -6.113118166 | 2.12E-09 |
| RP5-1024G6.8 | 0.472948096 | 3.854515216 | 6.112540133 | 2.12E-09 |
| TAF2 | 0.173307696 | 4.980697197 | 6.112350658 | 2.12E-09 |
| ZNF724P | 0.438674429 | 2.963580994 | 6.111948283 | 2.13E-09 |
| CKS1B | 0.466878341 | 3.293356215 | 6.111819531 | 2.13E-09 |
| CXorf65 | 0.686229297 | 3.993933378 | 6.110971923 | 2.14E-09 |
| MAP3K8 | 0.657232127 | 6.511499759 | 6.110527069 | 2.15E-09 |
| ANKRD49 | 0.317913137 | 5.582841425 | 6.109978079 | 2.15E-09 |
| PPP1R1B | 0.950397207 | -3.080251315 | 6.109858725 | 2.16E-09 |
| PROSER2 | 0.797236716 | 3.318560149 | 6.108852433 | 2.17E-09 |
| SEMA6A | 1.406658767 | -2.130968907 | 6.108708359 | 2.17E-09 |
| HNRNPUP1 | -0.298068493 | 6.964006221 | -6.106920999 | 2.19E-09 |
| DFNB31 | 0.890240249 | 2.013005182 | 6.106518955 | 2.20E-09 |
| RP11-156E8.1 | 0.462246638 | 2.335417586 | 6.106209551 | 2.20E-09 |
| USP28 | -0.388985597 | 3.924725479 | -6.104880357 | 2.22E-09 |
| MIEF1 | 0.227103055 | 5.126602956 | 6.103559977 | 2.24E-09 |
| RP11-73K9.2 | 0.596509066 | 1.54488937 | 6.103481868 | 2.24E-09 |
| TMEM232 | -0.863412146 | -2.501665364 | -6.102756705 | 2.25E-09 |
| NELFB | -0.209976523 | 6.672766835 | -6.10229572 | 2.25E-09 |
| APOBEC3B | 0.784122097 | 4.402664045 | 6.102101483 | 2.25E-09 |
| IPO7P1 | -0.373663704 | 4.235723167 | -6.101735601 | 2.26E-09 |
| AC016292.3 | -0.351068777 | 5.220174118 | -6.100803597 | 2.27E-09 |
| CUEDC2 | -0.233719032 | 6.921598103 | -6.100505639 | 2.28E-09 |
| EAF2 | -0.485207418 | 6.730206662 | -6.10018789 | 2.28E-09 |
| DIMT1 | -0.2558348 | 4.85747127 | -6.094303824 | 2.36E-09 |
| SHC1 | 0.200450532 | 6.333910212 | 6.094272975 | 2.36E-09 |
| SWSAP1 | -0.31495482 | 3.338443841 | -6.09347615 | 2.37E-09 |
| LRP5L | 0.575431976 | 3.757537207 | 6.093269782 | 2.37E-09 |
| FOXO1 | 0.825863236 | 4.127459004 | 6.092973341 | 2.38E-09 |
| TMEM19 | -0.406339061 | 3.555014504 | -6.092676081 | 2.38E-09 |
| RP4-781K5.2 | 0.837453893 | 1.548607967 | 6.091539157 | 2.40E-09 |
| CLHC1 | -0.56476087 | 2.271762783 | -6.09024012 | 2.41E-09 |
| BEND3 | 0.483166126 | 1.819019162 | 6.08912589 | 2.43E-09 |
| MSRB2 | -0.395116636 | 4.647794097 | -6.08834063 | 2.44E-09 |
| RP11-282O18.3 | -0.526598019 | 2.406188528 | -6.088325578 | 2.44E-09 |
| CCR10 | 0.672443458 | -0.142122189 | 6.087486071 | 2.45E-09 |
| RP5-902P8.12 | 0.608932263 | 0.112266753 | 6.087208444 | 2.46E-09 |
| AF064858.7 | -0.880424802 | -1.883649994 | -6.086529815 | 2.47E-09 |
| ZNF563 | -0.633927842 | 0.592118688 | -6.086121563 | 2.47E-09 |
| RAB40A | -0.780529754 | -1.54548533 | -6.086008174 | 2.47E-09 |
| SMARCC2 | -0.248677987 | 6.599511567 | -6.085661824 | 2.48E-09 |
| SYK | -0.393744898 | 8.128447041 | -6.084790764 | 2.49E-09 |
| FNTAP2 | -0.600330758 | 0.44331231 | -6.084253955 | 2.50E-09 |
| ZBTB24 | 0.249523435 | 4.862072671 | 6.08424228 | 2.50E-09 |
| CYB5D2 | -0.343125568 | 4.314297371 | -6.083973776 | 2.50E-09 |
| FPGT-TNNI3K | -0.761477274 | -1.299751942 | -6.083901813 | 2.50E-09 |
| C1S | 0.779051511 | 0.263550179 | 6.082877803 | 2.52E-09 |
| ZFC3H1 | -0.268187071 | 6.088243039 | -6.082413747 | 2.53E-09 |
| BCAS3 | -0.313065094 | 3.176999535 | -6.08014885 | 2.56E-09 |
| CNN3 | 1.169155731 | 0.65995457 | 6.079913207 | 2.56E-09 |
| ZNF394 | 0.498686585 | 6.490801271 | 6.079773049 | 2.56E-09 |
| SEMA6B | 1.222211268 | 1.762134588 | 6.079643906 | 2.57E-09 |
| CTD-2306M10.1 | -1.189183813 | 1.832656603 | -6.078953666 | 2.58E-09 |
| HIGD2A | -0.311393893 | 8.012447408 | -6.078036769 | 2.59E-09 |
| SCN1B | 0.797305334 | 1.066816436 | 6.077214238 | 2.60E-09 |
| PANK1 | -0.723824383 | 1.47669389 | -6.076523568 | 2.61E-09 |
| SRP9P1 | -0.314813059 | 6.817880124 | -6.076500875 | 2.61E-09 |
| SLC7A5P1 | 0.578511064 | 4.174821851 | 6.076426185 | 2.61E-09 |
| KIR3DX1 | 1.132792013 | -0.271585979 | 6.073648316 | 2.66E-09 |
| RP11-266L9.3 | -0.848368063 | -1.191793538 | -6.07321729 | 2.66E-09 |
| DNTTIP2 | 0.311589988 | 6.345448728 | 6.073032186 | 2.67E-09 |
| DENND2D | -0.474216094 | 4.46620477 | -6.07278883 | 2.67E-09 |
| KCNG1 | -1.082499349 | -3.093472904 | -6.072012453 | 2.68E-09 |
| RPL7L1 | -0.319759675 | 5.661971067 | -6.071657135 | 2.69E-09 |
| TBCAP1 | -0.386669554 | 4.84093984 | -6.070783795 | 2.70E-09 |
| ZC3H12A-DT | -0.490834806 | 2.828755377 | -6.069271674 | 2.72E-09 |
| MED30 | 0.332178838 | 5.712563832 | 6.069011192 | 2.73E-09 |
| LOH12CR2 | -0.500671209 | 0.526480628 | -6.068598289 | 2.74E-09 |
| ZNF773 | 0.435989877 | 2.08116017 | 6.068585606 | 2.74E-09 |
| EARS2 | -0.388167054 | 3.445256531 | -6.067389954 | 2.75E-09 |
| PLEKHG1 | 0.947601496 | 0.191762922 | 6.065858487 | 2.78E-09 |
| ISCA1P1 | 0.40978653 | 4.54361251 | 6.064581584 | 2.80E-09 |
| LINC00304 | 0.900664493 | -0.983679548 | 6.063604103 | 2.82E-09 |
| CTB-58E17.1 | 0.51394332 | 2.709115788 | 6.062142244 | 2.84E-09 |
| RP11-732A19.1 | -0.751819009 | -0.611891754 | -6.059829748 | 2.88E-09 |
| KIAA0930 | -0.560298764 | 6.154497788 | -6.059756489 | 2.88E-09 |
| AC007566.10 | -0.447900976 | 3.398275808 | -6.058409834 | 2.90E-09 |
| RP11-162G10.5 | -0.61970191 | 0.266791114 | -6.056899875 | 2.93E-09 |
| MAFG-AS1 | -0.744809404 | 0.390914459 | -6.05674261 | 2.93E-09 |
| RP6-218J18.2 | 0.307971978 | 6.526229094 | 6.055178938 | 2.95E-09 |
| ABHD10 | -0.298865077 | 5.493064302 | -6.054894373 | 2.96E-09 |
| AP001257.1 | -1.17111791 | 0.70006191 | -6.054293739 | 2.97E-09 |
| TARS2 | -0.220933723 | 4.894591252 | -6.052537597 | 3.00E-09 |
| USP9X | 0.219481995 | 5.949156876 | 6.051868067 | 3.01E-09 |
| FGFR1OP | -0.378395336 | 2.526996057 | -6.05118355 | 3.02E-09 |
| ZSCAN31 | -0.72726224 | 1.281946929 | -6.050553708 | 3.03E-09 |
| HNRNPH2 | 0.238205216 | 8.082115383 | 6.049485128 | 3.05E-09 |
| C10orf88 | -0.291018177 | 2.95538929 | -6.04811106 | 3.08E-09 |
| TINCR | 0.727691361 | -0.436665886 | 6.047227342 | 3.09E-09 |
| CDK5RAP3 | -0.267464911 | 6.750311922 | -6.04689891 | 3.10E-09 |
| RNF216P1 | 0.309780343 | 3.488103856 | 6.046858004 | 3.10E-09 |
| RP11-513D5.5 | -0.576970682 | 0.655837077 | -6.046480688 | 3.11E-09 |
| RUVBL1 | -0.490967299 | 4.447196878 | -6.044650459 | 3.14E-09 |
| CTRC | 0.776120576 | -1.814619711 | 6.043790647 | 3.15E-09 |
| RP11-465B22.3 | 1.293736051 | 1.120791649 | 6.042351686 | 3.18E-09 |
| HMGA1P8 | 0.451748702 | 2.515910104 | 6.042086435 | 3.19E-09 |
| RP11-352G9.1 | -0.783490249 | -0.716716181 | -6.041697308 | 3.19E-09 |
| GLS | -0.282292587 | 5.876572498 | -6.041320158 | 3.20E-09 |
| NIN | -0.256484752 | 6.835859524 | -6.040367502 | 3.22E-09 |
| PWWP2A | -0.222785501 | 4.651434008 | -6.039427599 | 3.23E-09 |
| MTRF1 | -0.243223525 | 4.228010369 | -6.038962931 | 3.24E-09 |
| RPA2P3 | -0.598774869 | 0.912328275 | -6.038949068 | 3.24E-09 |
| CTD-2369P2.2 | 0.69397756 | 1.563407011 | 6.038823998 | 3.25E-09 |
| TNFRSF1A | -0.424880325 | 7.143199058 | -6.038323916 | 3.25E-09 |
| TPP1 | -0.387689358 | 9.276004696 | -6.038203705 | 3.26E-09 |
| MAP2K4 | -0.328920924 | 4.233639487 | -6.037657901 | 3.27E-09 |
| AC024704.2 | -0.703969626 | -0.125656576 | -6.036173941 | 3.30E-09 |
| RGCC | 0.844809801 | 7.571257255 | 6.036059747 | 3.30E-09 |
| AGBL5 | 0.335302507 | 4.873524788 | 6.034987741 | 3.32E-09 |
| RASSF5 | 0.433346702 | 7.047779471 | 6.0346761 | 3.32E-09 |
| NRDE2 | 0.245849721 | 3.584307187 | 6.033951208 | 3.34E-09 |
| AC108448.2 | -0.753400054 | 1.650155759 | -6.033600521 | 3.34E-09 |
| LGMNP1 | 0.822066928 | -1.189987539 | 6.032072154 | 3.37E-09 |
| MIR4772 | 1.041130641 | 0.647097673 | 6.029412674 | 3.43E-09 |
| FJX1 | 0.852584208 | -2.262749292 | 6.029170607 | 3.43E-09 |
| ANLN | 0.856715575 | 3.825756373 | 6.027372792 | 3.47E-09 |
| CDC5L | 0.184969706 | 6.012001466 | 6.026769692 | 3.48E-09 |
| GCDH | -0.299193676 | 4.5735964 | -6.026681922 | 3.48E-09 |
| MSH2 | -0.459298339 | 5.096374315 | -6.026459367 | 3.48E-09 |
| PPARD | 0.678717314 | 3.620620295 | 6.026062729 | 3.49E-09 |
| AKAP1 | -0.372557588 | 4.669322043 | -6.026047859 | 3.49E-09 |
| MT-ND3 | -0.219067453 | 12.08392739 | -6.025405491 | 3.50E-09 |
| CHIC2 | 0.226557118 | 6.563665525 | 6.025082789 | 3.51E-09 |
| OSGEPL1-AS1 | -0.49837875 | 0.997228299 | -6.025076258 | 3.51E-09 |
| ARPC5 | -0.474344784 | 7.113343025 | -6.023949287 | 3.53E-09 |
| RP11-64D22.2 | -1.131674201 | -1.658974838 | -6.023687397 | 3.54E-09 |
| GCHFR | 0.736291938 | 3.604683238 | 6.022826267 | 3.56E-09 |
| RGS19 | -0.479012132 | 7.659400442 | -6.021239788 | 3.59E-09 |
| RP1-168L15.5 | -0.625944768 | -0.965607429 | -6.021027258 | 3.59E-09 |
| C12orf79 | 0.839826661 | 2.403260377 | 6.020819263 | 3.60E-09 |
| UAP1 | 0.475198423 | 5.199687243 | 6.020238556 | 3.61E-09 |
| HMOX2 | -0.308567357 | 4.890989739 | -6.019505213 | 3.63E-09 |
| RHBDL1 | 0.639015637 | 1.936066776 | 6.018915543 | 3.64E-09 |
| CALM2 | -0.337527163 | 6.697619596 | -6.018315999 | 3.65E-09 |
| CYB561D1 | -0.317672285 | 4.141250442 | -6.017351664 | 3.67E-09 |
| TMEM62 | 0.274193891 | 3.990592773 | 6.016894911 | 3.68E-09 |
| PLXNB2 | -0.522893583 | 7.667695081 | -6.015825364 | 3.70E-09 |
| CNDP2 | -0.394669711 | 5.544473371 | -6.015036332 | 3.72E-09 |
| ACAA1 | -0.263151377 | 5.765792809 | -6.014459281 | 3.73E-09 |
| RHBDD1 | -0.254121934 | 4.256168545 | -6.014200869 | 3.74E-09 |
| SBNO1 | 0.175202775 | 5.820042221 | 6.014041032 | 3.74E-09 |
| VRK3 | -0.198570549 | 4.365396561 | -6.01392514 | 3.74E-09 |
| AL359091.2 | -0.65440674 | -1.39553017 | -6.012946377 | 3.76E-09 |
| PPIP5K2 | -0.273806806 | 5.807627587 | -6.01138176 | 3.80E-09 |
| TMEM248 | -0.202741304 | 6.574533316 | -6.010025979 | 3.83E-09 |
| CCNT2 | 0.258041244 | 5.515111621 | 6.009803393 | 3.83E-09 |
| RP4-682C21.2 | 0.271018331 | 3.981522203 | 6.007231 | 3.89E-09 |
| RBM26 | -0.192238093 | 6.135948576 | -6.006621356 | 3.90E-09 |
| IGKV1-22 | -1.661092404 | 0.571440205 | -6.00570923 | 3.92E-09 |
| ERF | 0.338880626 | 6.141597308 | 6.003989969 | 3.96E-09 |
| UBE4A | -0.24347795 | 6.043817375 | -6.003511151 | 3.97E-09 |
| DEXI | -0.352540344 | 4.747290256 | -6.003058984 | 3.98E-09 |
| MIDN | 0.531063149 | 7.594706692 | 6.002986438 | 3.98E-09 |
| RP11-76E17.4 | -0.994724877 | 1.234168667 | -6.002278441 | 4.00E-09 |
| GPX3 | 1.094820141 | 1.666017989 | 6.001977991 | 4.01E-09 |
| CTA-217C2.1 | -0.436963503 | 4.15576336 | -6.001487622 | 4.02E-09 |
| FAM96B | -0.204742061 | 6.771987655 | -6.000710709 | 4.04E-09 |
| HMGB2P1 | 0.576611331 | 3.716283173 | 5.999575937 | 4.06E-09 |
| RP11-546B8.3 | 0.52000435 | 0.666260024 | 5.996951358 | 4.12E-09 |
| GBGT1 | -0.53885535 | 3.757078116 | -5.996807916 | 4.13E-09 |
| CSNK1D | 0.288411157 | 6.08609262 | 5.996453778 | 4.14E-09 |
| GSKIP | 0.27734587 | 3.735793791 | 5.995598397 | 4.16E-09 |
| GPR84 | 1.072796768 | 3.155572439 | 5.995492804 | 4.16E-09 |
| TRIM44 | -0.301743462 | 6.750408321 | -5.995227608 | 4.16E-09 |
| RP11-173B14.4 | -0.630634801 | 0.349626978 | -5.994864382 | 4.17E-09 |
| RBM3 | 0.361025717 | 8.886058924 | 5.993741896 | 4.20E-09 |
| PPP1R10 | -0.506505685 | 5.36788946 | -5.993684618 | 4.20E-09 |
| METTL5 | -0.22530677 | 5.024013891 | -5.991604271 | 4.25E-09 |
| ANKZF1 | -0.305109871 | 5.257131001 | -5.990657825 | 4.27E-09 |
| OBFC1 | -0.340054465 | 6.560259773 | -5.990242167 | 4.28E-09 |
| TBC1D17 | -0.283150968 | 4.918332164 | -5.988345259 | 4.33E-09 |
| USP37 | 0.220542992 | 4.052532774 | 5.988152339 | 4.34E-09 |
| C8orf82 | -0.329787887 | 5.022480746 | -5.987391058 | 4.35E-09 |
| COG1 | 0.216804736 | 5.029336979 | 5.987329509 | 4.36E-09 |
| ATG9A | 0.373459838 | 4.871813759 | 5.986913002 | 4.37E-09 |
| DIP2A-IT1 | 0.584348515 | 3.19376348 | 5.986875729 | 4.37E-09 |
| AC012487.2 | -0.644789388 | 0.725348997 | -5.985403569 | 4.40E-09 |
| BNIPL | -0.636177858 | -0.77357748 | -5.984088806 | 4.44E-09 |
| RNA5SP450 | 0.771852516 | 1.164457298 | 5.983815652 | 4.44E-09 |
| DYNC1LI2 | 0.299681712 | 5.85694595 | 5.983757369 | 4.45E-09 |
| HHLA3 | -0.529637932 | 3.137746391 | -5.983397047 | 4.45E-09 |
| GSDMB | -0.462635088 | 3.226442239 | -5.98277794 | 4.47E-09 |
| PIP4K2A | -0.32110417 | 7.191703691 | -5.982371499 | 4.48E-09 |
| ATP5H | -0.249244786 | 5.9468056 | -5.982260569 | 4.48E-09 |
| ZMAT2 | -0.25519853 | 7.664586652 | -5.982224791 | 4.48E-09 |
| LRRC39 | 0.603722297 | 1.724020161 | 5.981912098 | 4.49E-09 |
| RPP25 | -0.844404738 | 1.959172163 | -5.981161954 | 4.51E-09 |
| POT1 | -0.282452005 | 4.458302405 | -5.980208087 | 4.54E-09 |
| TAS2R31 | -0.657251757 | 0.679672621 | -5.979911127 | 4.54E-09 |
| LSR | 0.850816184 | 1.990102809 | 5.979458089 | 4.56E-09 |
| TFRC | 0.60954509 | 7.085534393 | 5.978927894 | 4.57E-09 |
| RP11-337C18.8 | -0.408002596 | 1.547093875 | -5.97716341 | 4.62E-09 |
| CMTM7 | -0.366737313 | 6.06422588 | -5.976191149 | 4.64E-09 |
| UCP2 | -0.442422783 | 7.839894685 | -5.976039576 | 4.64E-09 |
| MYB | -0.665822088 | 7.937329371 | -5.973061324 | 4.72E-09 |
| SRSF11 | 0.244273588 | 7.40679262 | 5.972893064 | 4.73E-09 |
| MIR421 | -0.776279666 | 1.582378448 | -5.972173725 | 4.75E-09 |
| CTB-113I20.2 | -0.65600929 | 0.290906634 | -5.971459256 | 4.77E-09 |
| HMGN1P8 | 0.554147211 | 1.753029591 | 5.970986083 | 4.78E-09 |
| RP5-1065J22.8 | -0.475565828 | 0.631953302 | -5.970386766 | 4.80E-09 |
| ZNF669 | 0.394083979 | 4.543671665 | 5.969166099 | 4.83E-09 |
| RNF214 | -0.250002877 | 5.14616792 | -5.967976316 | 4.86E-09 |
| RP11-1029J19.4 | 0.884394403 | -1.0404346 | 5.96633519 | 4.91E-09 |
| COX11 | -0.299792264 | 4.162989947 | -5.96629755 | 4.91E-09 |
| TTF1 | 0.198923785 | 5.27127911 | 5.965643095 | 4.93E-09 |
| HIPK1 | 0.275316068 | 7.332684036 | 5.964896977 | 4.95E-09 |
| AKAP5 | 0.635634798 | -0.461163806 | 5.963499604 | 4.99E-09 |
| ASXL2 | -0.283219583 | 5.012679411 | -5.962530242 | 5.02E-09 |
| SPRED3 | 0.709263252 | -0.653533774 | 5.96167887 | 5.04E-09 |
| VDAC3 | -0.266877781 | 6.793964799 | -5.961547269 | 5.04E-09 |
| R3HDM4 | 0.364811693 | 7.831957092 | 5.959802236 | 5.09E-09 |
| CUL7 | -0.281713313 | 4.870182299 | -5.959006157 | 5.12E-09 |
| OCIAD1 | -0.158688945 | 6.551685415 | -5.958105074 | 5.14E-09 |
| RAPGEF2 | 0.470445991 | 5.201603981 | 5.957289027 | 5.17E-09 |
| SMARCD1 | 0.262839566 | 6.23540578 | 5.956787235 | 5.18E-09 |
| TINF2 | -0.165250274 | 6.983333177 | -5.953749521 | 5.27E-09 |
| RP11-159N11.4 | -0.831781038 | 0.526445588 | -5.953599316 | 5.28E-09 |
| PER1 | 0.771826184 | 7.006181315 | 5.953522087 | 5.28E-09 |
| CDPF1 | -0.337084979 | 3.570856002 | -5.953139065 | 5.29E-09 |
| DZIP3 | -0.399639238 | 3.303726423 | -5.952199943 | 5.32E-09 |
| AC009473.1 | -0.604359099 | -0.283441379 | -5.951805659 | 5.33E-09 |
| POMGNT2 | 0.511463108 | 3.308452008 | 5.950814157 | 5.36E-09 |
| C3orf18 | -0.521530822 | 2.158047505 | -5.949422152 | 5.40E-09 |
| MAN2B2 | -0.271143301 | 5.91783077 | -5.947864391 | 5.45E-09 |
| RN7SL328P | 0.741053343 | 0.800011435 | 5.947785176 | 5.45E-09 |
| AAAS | -0.240920901 | 5.519900986 | -5.947416945 | 5.46E-09 |
| IKZF4 | 0.297325398 | 3.302259737 | 5.947348263 | 5.47E-09 |
| YAE1D1 | -0.530713976 | 2.573742352 | -5.946961656 | 5.48E-09 |
| EXOC7 | -0.181770453 | 6.258850758 | -5.945832711 | 5.51E-09 |
| ADHFE1 | -0.461091762 | 3.718699001 | -5.945094223 | 5.54E-09 |
| MRPL51 | -0.358960253 | 6.822611294 | -5.945055652 | 5.54E-09 |
| RNASEH1P2 | 0.359770651 | 2.388858872 | 5.944686015 | 5.55E-09 |
| LRRC37B | 0.316275133 | 4.988108041 | 5.94344752 | 5.59E-09 |
| TROAP | 0.891242529 | 3.175553829 | 5.941405257 | 5.65E-09 |
| ZCCHC2 | 0.403820862 | 4.892164691 | 5.940456604 | 5.68E-09 |
| AC009403.2 | -0.360978866 | 3.579109411 | -5.939829513 | 5.70E-09 |
| RP11-46A10.4 | -0.536790461 | 1.303224759 | -5.93976317 | 5.71E-09 |
| BNIP2 | 0.241899218 | 6.876051318 | 5.939717609 | 5.71E-09 |
| RP11-1275H24.3 | -0.834362798 | 3.332407074 | -5.939011757 | 5.73E-09 |
| DDX3YP3 | 0.433855384 | 2.570245394 | 5.938332401 | 5.75E-09 |
| RP11-441O15.3 | -0.736445941 | 0.278882677 | -5.938057673 | 5.76E-09 |
| CLEC2B | 0.805837432 | 6.930576042 | 5.937124194 | 5.79E-09 |
| POC5 | -0.26603302 | 4.244354872 | -5.936942927 | 5.80E-09 |
| ZNF582 | -0.407893002 | 1.894069207 | -5.936798595 | 5.80E-09 |
| AKIP1 | -0.305963324 | 4.135987973 | -5.936454644 | 5.81E-09 |
| ZNF446 | 0.254394862 | 3.58145638 | 5.936390048 | 5.82E-09 |
| A1BG-AS1 | -0.560198952 | 3.171265036 | -5.935940514 | 5.83E-09 |
| BANF1P3 | -0.650923595 | 6.079269411 | -5.935410714 | 5.85E-09 |
| RORA | 0.940498182 | 2.374447532 | 5.934711547 | 5.87E-09 |
| RCN1P2 | -0.526027667 | 5.899673251 | -5.933063509 | 5.93E-09 |
| FLJ00418 | 0.799985861 | -3.003554 | 5.932945351 | 5.93E-09 |
| IGKV2-23 | -1.73493871 | 0.924442022 | -5.932757814 | 5.94E-09 |
| DUSP5 | 0.924827749 | 5.429547449 | 5.932455777 | 5.95E-09 |
| FBXO32 | 0.638181433 | 2.36320952 | 5.931798357 | 5.97E-09 |
| RP11-326I11.3 | -0.347722981 | 3.158459876 | -5.93121148 | 5.99E-09 |
| RP11-410L14.1 | -0.748205708 | -0.108406394 | -5.931100704 | 5.99E-09 |
| ZNF92 | 0.480889191 | 5.484663553 | 5.930017166 | 6.03E-09 |
| GS1-114I9.1 | -0.625897209 | 0.499000658 | -5.92831489 | 6.09E-09 |
| CTD-2313J17.1 | -0.603249965 | 0.999885701 | -5.927670007 | 6.11E-09 |
| ZFYVE19 | -0.269928601 | 4.644686287 | -5.927491424 | 6.12E-09 |
| PHF5CP | 0.484021513 | 1.047750012 | 5.927395511 | 6.12E-09 |
| ARL6IP1 | 0.265742337 | 7.019864955 | 5.926325326 | 6.16E-09 |
| MPLKIP | -0.358133403 | 3.228589286 | -5.926164804 | 6.16E-09 |
| RP1-261D10.1 | -0.566640291 | 0.552231512 | -5.926051343 | 6.17E-09 |
| RP11-344B5.2 | -1.093191205 | 1.392147147 | -5.925947439 | 6.17E-09 |
| TRIM68 | -0.386563037 | 3.680495429 | -5.925617004 | 6.18E-09 |
| NNT | -0.296208986 | 6.168999817 | -5.925255747 | 6.19E-09 |
| NME3 | -0.475241915 | 5.745511714 | -5.923286779 | 6.26E-09 |
| LETM2 | 0.467436529 | 2.224753044 | 5.922783215 | 6.28E-09 |
| MAP3K9 | 0.733653378 | -0.227123914 | 5.921124276 | 6.34E-09 |
| TRIOBP | 0.292588436 | 3.117478446 | 5.92036647 | 6.37E-09 |
| RP11-362K14.5 | -0.779651221 | -1.106184752 | -5.919611209 | 6.39E-09 |
| ZNF425 | -0.607658688 | 0.948822375 | -5.917696545 | 6.46E-09 |
| MESDC1 | 0.409094816 | 5.677413524 | 5.917525554 | 6.47E-09 |
| COASY | -0.18606166 | 5.994972515 | -5.917120553 | 6.48E-09 |
| TMEM129 | -0.196726626 | 5.326123099 | -5.916878037 | 6.49E-09 |
| CENPE | 0.703263509 | 3.26527054 | 5.916845105 | 6.49E-09 |
| WDR12 | -0.410137247 | 4.065934676 | -5.915895421 | 6.53E-09 |
| ANKRD33B | 0.832346028 | 2.871422016 | 5.915193229 | 6.55E-09 |
| RB1 | -0.397520345 | 6.309341885 | -5.91479942 | 6.57E-09 |
| IL6STP1 | 0.631163117 | 2.345724176 | 5.914665771 | 6.57E-09 |
| GEN1 | 0.328852239 | 3.977470608 | 5.914263907 | 6.59E-09 |
| RASA2 | 0.34001056 | 5.636014657 | 5.913581186 | 6.61E-09 |
| RNF139 | 0.416166311 | 7.589718442 | 5.912669166 | 6.65E-09 |
| GLTPD1 | -0.313864783 | 4.856779644 | -5.9113197 | 6.70E-09 |
| SCAND1 | 0.192244551 | 6.86238561 | 5.910143442 | 6.74E-09 |
| SRP72P1 | 0.295639907 | 3.35253045 | 5.909678807 | 6.76E-09 |
| DBR1 | 0.259007958 | 4.438699969 | 5.908301914 | 6.81E-09 |
| TAF13 | 0.423202949 | 5.3951265 | 5.906922313 | 6.87E-09 |
| ABCF3 | 0.210477215 | 5.488172708 | 5.906526541 | 6.88E-09 |
| TMBIM6 | -0.251673205 | 8.883282226 | -5.906450981 | 6.89E-09 |
| NDUFS2 | -0.188787023 | 6.434670797 | -5.906164885 | 6.90E-09 |
| APLF | -0.43836332 | 2.329206976 | -5.906038615 | 6.90E-09 |
| MXI1 | 0.390420504 | 5.676827369 | 5.905673068 | 6.92E-09 |
| DHDH | 0.667570747 | -0.994721336 | 5.904719961 | 6.95E-09 |
| APH1B | -0.299407799 | 4.395264283 | -5.903236532 | 7.01E-09 |
| ZNF354A | -0.335993644 | 4.530380425 | -5.902789151 | 7.03E-09 |
| UBXN7 | 0.214757741 | 5.726111505 | 5.901030304 | 7.10E-09 |
| RFX5 | -0.251488671 | 6.583142461 | -5.900843956 | 7.11E-09 |
| EIF2S2P4 | -0.26370673 | 7.340677596 | -5.899650214 | 7.15E-09 |
| TMEM70 | 0.411294944 | 4.449546202 | 5.89746699 | 7.24E-09 |
| DYRK2 | 0.35827136 | 4.992582755 | 5.897139303 | 7.26E-09 |
| Z83851.4 | -0.624818431 | 0.848979661 | -5.896601621 | 7.28E-09 |
| PKN2 | 0.243964103 | 5.772838958 | 5.896493966 | 7.28E-09 |
| TAF4B | 0.411107438 | 3.708822664 | 5.896401037 | 7.29E-09 |
| RNU6-36P | 0.498661632 | 1.949364698 | 5.896071173 | 7.30E-09 |
| PNRC2 | 0.616346082 | 3.49165682 | 5.895601409 | 7.32E-09 |
| RP13-941N14.1 | -0.604910929 | 1.786494543 | -5.89529309 | 7.33E-09 |
| ZNF354B | -0.304230079 | 4.087286001 | -5.894187208 | 7.38E-09 |
| RP1-197O17.2 | -0.52097369 | 0.472363287 | -5.893757129 | 7.40E-09 |
| RP1-93H18.1 | -0.639824809 | -1.161276424 | -5.893439965 | 7.41E-09 |
| TOE1 | 0.299412856 | 5.332600405 | 5.892433627 | 7.45E-09 |
| AC093673.5 | -0.40918633 | 6.069939634 | -5.891455351 | 7.49E-09 |
| RP3-402G11.26 | 0.504802657 | 3.612716538 | 5.890898265 | 7.52E-09 |
| ACSS2 | -0.380786853 | 5.217558769 | -5.889361922 | 7.58E-09 |
| RP11-164H13.1 | -0.838668628 | -2.763615487 | -5.889038616 | 7.59E-09 |
| NEDD4L | 0.902828139 | 0.786877116 | 5.888997133 | 7.60E-09 |
| IRF9 | 0.511871169 | 3.855892419 | 5.886784897 | 7.69E-09 |
| ORC2 | -0.254356188 | 4.64476596 | -5.886186021 | 7.72E-09 |
| FTH1P5 | 0.828196009 | 5.280776448 | 5.885771725 | 7.73E-09 |
| NECAP1P1 | 0.608321594 | 0.120092508 | 5.885321745 | 7.75E-09 |
| EED | 0.250958998 | 3.29261159 | 5.88471551 | 7.78E-09 |
| CD27-AS1 | -0.382050794 | 4.093046749 | -5.883210078 | 7.85E-09 |
| CTD-2235H24.2 | -0.489905318 | 1.689734307 | -5.883167391 | 7.85E-09 |
| ARAF | 0.154577052 | 6.966074039 | 5.881558811 | 7.92E-09 |
| RP11-190A12.8 | -0.921073507 | 0.402858703 | -5.881427404 | 7.93E-09 |
| CTD-3222D19.7 | 0.574550695 | 0.30462462 | 5.88127807 | 7.93E-09 |
| SERTAD3 | 0.472960911 | 4.878823542 | 5.881200373 | 7.94E-09 |
| IQGAP1 | -0.556136565 | 7.771901881 | -5.879397861 | 8.02E-09 |
| SIGIRR | -0.29657433 | 6.448128448 | -5.879265116 | 8.02E-09 |
| SLC23A3 | -0.611456594 | -0.852893683 | -5.878861037 | 8.04E-09 |
| CTD-2530H12.2 | 0.877220514 | -0.778863041 | 5.878816835 | 8.04E-09 |
| AC005154.7 | -0.51409133 | 3.145519589 | -5.878764145 | 8.05E-09 |
| TRPV2 | -0.423252978 | 6.057749932 | -5.878121508 | 8.07E-09 |
| MIR181A2HG | -0.822357686 | 0.717555636 | -5.877727931 | 8.09E-09 |
| FBXO36 | 0.542020211 | 1.161984524 | 5.877219953 | 8.12E-09 |
| FFAR4 | -1.146775414 | -2.177127728 | -5.876572498 | 8.14E-09 |
| EPAS1 | 0.963495947 | 1.641118603 | 5.875836777 | 8.18E-09 |
| TICAM1 | 0.433532392 | 5.683257199 | 5.875386251 | 8.20E-09 |
| CBL | -0.32951522 | 6.208988538 | -5.874834786 | 8.22E-09 |
| TYMS | 0.693546157 | 6.935325482 | 5.874211222 | 8.25E-09 |
| WDR26 | 0.278892126 | 7.000664164 | 5.873328799 | 8.29E-09 |
| CTD-2619J13.19 | -0.724132652 | -1.722032767 | -5.872987663 | 8.31E-09 |
| HNRNPUL2 | -0.184269648 | 6.028307734 | -5.871157336 | 8.40E-09 |
| PIP5K1C | -0.287732158 | 5.203242948 | -5.870969978 | 8.40E-09 |
| CCL4L2 | 1.136103908 | -0.836322396 | 5.870669487 | 8.42E-09 |
| BTBD9 | 0.297015327 | 3.533649514 | 5.870528584 | 8.43E-09 |
| LUC7L3 | -0.367052831 | 6.969072484 | -5.870527636 | 8.43E-09 |
| RP11-79N23.1 | -0.8666552 | 0.363171835 | -5.870044992 | 8.45E-09 |
| CTA-268H5.12 | 0.61137285 | 1.248382319 | 5.869975583 | 8.45E-09 |
| RP11-166O4.6 | -0.578737679 | 1.770613134 | -5.869787029 | 8.46E-09 |
| RP11-151A6.4 | 0.723980066 | -0.650373101 | 5.869280979 | 8.48E-09 |
| PIF1 | 0.781536431 | 0.69215264 | 5.867070174 | 8.59E-09 |
| EVI2A | -0.658996867 | 6.637258016 | -5.866132198 | 8.64E-09 |
| PLAGL2 | 0.32461473 | 5.786497726 | 5.866124787 | 8.64E-09 |
| CXCL5 | 1.386440045 | -0.568416669 | 5.864923542 | 8.69E-09 |
| IFFO2 | 0.525581542 | 4.75761878 | 5.86437513 | 8.72E-09 |
| RP11-545I5.3 | -0.463757333 | 0.801566753 | -5.863642593 | 8.76E-09 |
| ECHS1 | -0.321132361 | 6.730173203 | -5.862961856 | 8.79E-09 |
| MBD5 | 0.259732433 | 3.113696965 | 5.861881969 | 8.84E-09 |
| SLC25A40 | -0.427209846 | 5.383245756 | -5.86011976 | 8.93E-09 |
| ACTL6A | -0.380710542 | 5.012110982 | -5.859703051 | 8.95E-09 |
| TRAPPC10 | 0.193940145 | 6.074709397 | 5.858709649 | 9.00E-09 |
| RP11-793H13.10 | -0.523181365 | -0.202891543 | -5.85804413 | 9.04E-09 |
| RP3-473L9.4 | -0.82107645 | -0.886307313 | -5.854570357 | 9.21E-09 |
| URB1 | -0.449080636 | 4.010085648 | -5.853842737 | 9.25E-09 |
| RP3-388M5.9 | -0.668215994 | -1.476164193 | -5.852817793 | 9.30E-09 |
| ANKRD36B | 0.408921412 | 3.240933944 | 5.852426009 | 9.32E-09 |
| CIRBP-AS1 | -0.665312397 | -0.062081162 | -5.850067747 | 9.45E-09 |
| TMEM126B | -0.21957184 | 5.241046139 | -5.850042443 | 9.45E-09 |
| NHSL2 | -0.975536118 | 3.507454652 | -5.849796838 | 9.46E-09 |
| ZNF256 | 0.788692267 | 4.613914142 | 5.849022282 | 9.50E-09 |
| EIF5AL1 | 0.271189155 | 6.14329188 | 5.84854832 | 9.53E-09 |
| ZNF414 | 0.254501542 | 5.686411323 | 5.84828701 | 9.54E-09 |
| RP11-653J6.1 | -0.711209233 | -0.52736129 | -5.847182416 | 9.60E-09 |
| KAT5 | 0.183414204 | 6.178017439 | 5.847095211 | 9.61E-09 |
| MAP7D1 | 0.21534896 | 7.226619404 | 5.846429328 | 9.64E-09 |
| RP11-651P23.4 | 0.18115889 | 6.87862235 | 5.845291556 | 9.70E-09 |
| ZNF140 | -0.349998847 | 2.347181378 | -5.84460582 | 9.74E-09 |
| PLIN2 | 0.734878564 | 6.318356227 | 5.843882434 | 9.78E-09 |
| IFI16 | -0.499856863 | 7.019045944 | -5.842935263 | 9.83E-09 |
| GATA3 | 0.974679973 | 2.817148491 | 5.842634202 | 9.85E-09 |
| HAUS6P1 | 0.309912588 | 3.71632799 | 5.842228559 | 9.87E-09 |
| RP11-333E1.1 | -0.469019621 | 1.750722123 | -5.841812072 | 9.89E-09 |
| PSMB5 | -0.358003275 | 5.775820987 | -5.84144373 | 9.91E-09 |
| RP11-464F9.22 | -0.749863275 | 0.545594271 | -5.841006282 | 9.94E-09 |
| AC010642.1 | 0.613716085 | 0.685882848 | 5.840961247 | 9.94E-09 |
| TNFRSF9 | 0.851864733 | 0.80908377 | 5.838231107 | 1.01E-08 |
| AC009120.6 | -0.368807418 | 3.650186748 | -5.837907068 | 1.01E-08 |
| CTA-331P3.1 | -0.658275832 | 3.718714843 | -5.837742163 | 1.01E-08 |
| TERF1 | 0.200130563 | 3.607099954 | 5.837579188 | 1.01E-08 |
| RNF5P1 | -0.441750599 | 5.231372196 | -5.836681197 | 1.02E-08 |
| RP11-7F17.5 | 0.814140233 | -1.57240556 | 5.834492113 | 1.03E-08 |
| RP11-296E7.1 | 0.8003921 | 1.641964888 | 5.833893979 | 1.03E-08 |
| AC005682.5 | 0.570531234 | 2.785454331 | 5.833462469 | 1.04E-08 |
| GPD1L | -0.352782896 | 4.372074462 | -5.832607207 | 1.04E-08 |
| PSPH | -0.492186301 | 3.816232551 | -5.831811157 | 1.05E-08 |
| MORN3 | 0.643974481 | 0.949224918 | 5.831111862 | 1.05E-08 |
| B3GNT9 | -0.497194179 | 1.647933915 | -5.829508531 | 1.06E-08 |
| AC009945.4 | -0.690027946 | 0.849115523 | -5.829062285 | 1.06E-08 |
| CTD-2587H19.3 | -0.634565181 | 0.143232502 | -5.826686307 | 1.08E-08 |
| RP11-156K13.3 | -0.929073482 | -0.947113882 | -5.824526046 | 1.09E-08 |
| GJA1 | 1.692933143 | -0.917236603 | 5.823511315 | 1.10E-08 |
| SGSM3 | -0.239663563 | 5.929458105 | -5.823266456 | 1.10E-08 |
| ACAD9 | -0.25489058 | 5.104852105 | -5.821954859 | 1.11E-08 |
| RP11-358B23.1 | -0.6829563 | -0.143269255 | -5.821366344 | 1.11E-08 |
| RP11-314N13.3 | -0.578173459 | 1.432726801 | -5.821248497 | 1.11E-08 |
| PAPLN | -0.981917647 | 0.243061968 | -5.821149168 | 1.11E-08 |
| RP1-118J21.25 | -0.817284031 | -0.57962974 | -5.819866311 | 1.12E-08 |
| HIRA | 0.209444913 | 4.081166351 | 5.819456213 | 1.12E-08 |
| SPATA5L1 | 0.304776162 | 3.85957494 | 5.818076099 | 1.13E-08 |
| HNRNPA2B1 | -0.249013646 | 9.665349271 | -5.817959847 | 1.13E-08 |
| MGP | 0.962446536 | -0.871882639 | 5.817811127 | 1.13E-08 |
| TUBA3FP | -0.712414475 | -1.74254807 | -5.817683644 | 1.13E-08 |
| CDH24 | 0.651707637 | 2.297489925 | 5.816373226 | 1.14E-08 |
| SLC25A36 | 0.346612921 | 6.684847267 | 5.815972474 | 1.14E-08 |
| RAPGEF6 | 0.280574692 | 2.654693825 | 5.81564864 | 1.14E-08 |
| TRIM23 | 0.326928653 | 2.833556489 | 5.814922849 | 1.15E-08 |
| ACTR6 | -0.237508235 | 3.892270024 | -5.814385159 | 1.15E-08 |
| RP11-534L20.5 | -0.721376722 | 0.88797631 | -5.814017003 | 1.16E-08 |
| RP4-655J12.4 | -0.731526671 | -0.871972352 | -5.813385374 | 1.16E-08 |
| WASH7P | 0.241597006 | 4.834819594 | 5.813242627 | 1.16E-08 |
| PGAP1 | 0.659385172 | 1.617771806 | 5.812926498 | 1.16E-08 |
| AC079780.3 | -0.665229523 | 5.33918874 | -5.812675432 | 1.16E-08 |
| PLA2G12AP1 | -0.466474343 | 2.379371149 | -5.812616012 | 1.16E-08 |
| RP11-163N6.2 | -0.595506935 | -1.364821807 | -5.812549625 | 1.16E-08 |
| MLX | -0.257256543 | 5.90937459 | -5.812527376 | 1.16E-08 |
| MET | 1.245314362 | -2.35134422 | 5.812435946 | 1.17E-08 |
| LINC01150 | -1.047939116 | 4.069228444 | -5.812381025 | 1.17E-08 |
| TTI2 | 0.273968224 | 3.584310849 | 5.810440802 | 1.18E-08 |
| MICALL1 | 0.247394027 | 4.96556341 | 5.809548967 | 1.18E-08 |
| AC024937.6 | 0.581840163 | -0.606899134 | 5.809266915 | 1.19E-08 |
| PAGR1 | -0.329637189 | 2.923037433 | -5.808883747 | 1.19E-08 |
| ABCB7 | -0.252186875 | 5.759251409 | -5.80802457 | 1.19E-08 |
| NOXRED1 | -0.53341178 | 0.785595397 | -5.807902503 | 1.19E-08 |
| MEGF8 | -0.357399577 | 4.331800703 | -5.80732059 | 1.20E-08 |
| API5 | -0.221256511 | 6.440143048 | -5.807041417 | 1.20E-08 |
| ZNF322P1 | -0.702564471 | 0.021274318 | -5.80534758 | 1.21E-08 |
| CTD-2262B20.1 | 0.942025448 | 2.012262051 | 5.805042362 | 1.21E-08 |
| RP11-74C1.2 | 0.466079743 | 1.260925777 | 5.804753457 | 1.22E-08 |
| TEX12 | 0.567369497 | 0.684496992 | 5.802979661 | 1.23E-08 |
| ANO6 | -0.313507634 | 6.040359343 | -5.802325476 | 1.23E-08 |
| HMGB1P3 | -0.610373958 | 0.499851622 | -5.802111773 | 1.23E-08 |
| PFN1P1 | -0.399611511 | 7.826821631 | -5.801647581 | 1.24E-08 |
| RRAD | 1.053736 | 1.144043111 | 5.800843167 | 1.24E-08 |
| STAU1 | 0.181008674 | 6.899083785 | 5.800790172 | 1.24E-08 |
| FCF1P6 | -0.497522125 | 1.592570179 | -5.799137832 | 1.25E-08 |
| KNOP1 | -0.479861769 | 3.169564368 | -5.798748262 | 1.26E-08 |
| MSRB1 | -0.711068389 | 6.56955213 | -5.797860449 | 1.26E-08 |
| IL16 | -0.523563611 | 5.554534999 | -5.797735394 | 1.26E-08 |
| RP11-394I13.2 | -0.658576632 | 0.33399999 | -5.797377324 | 1.27E-08 |
| NSUN5P2 | -0.459670906 | 4.651794716 | -5.796960718 | 1.27E-08 |
| ACSF3 | -0.244505163 | 4.382958384 | -5.796012378 | 1.28E-08 |
| BBC3 | 0.593264237 | 3.729776698 | 5.794670395 | 1.29E-08 |
| FANCI | 0.464762656 | 4.84169874 | 5.794470034 | 1.29E-08 |
| CTD-2619J13.3 | -0.745672084 | 0.566084712 | -5.793510499 | 1.29E-08 |
| HSPA8P9 | -0.642589223 | 0.250338447 | -5.792287372 | 1.30E-08 |
| RTEL1-TNFRSF6B | 0.43926528 | 0.604733017 | 5.79225193 | 1.30E-08 |
| RP11-613D13.4 | -0.608776637 | -0.967980159 | -5.79096342 | 1.31E-08 |
| CCNJ | 0.379253071 | 5.133385239 | 5.78960626 | 1.32E-08 |
| PDRG1 | 0.346327492 | 5.346973589 | 5.789536969 | 1.32E-08 |
| RP5-1021I20.4 | 0.71389218 | -1.96223531 | 5.78807114 | 1.33E-08 |
| SH2B3 | 0.445713451 | 7.0111495 | 5.787399227 | 1.34E-08 |
| HGSNAT | -0.279364106 | 5.927397941 | -5.787105182 | 1.34E-08 |
| UQCRFS1P1 | 0.265220924 | 6.001928502 | 5.785057247 | 1.36E-08 |
| CCDC24 | -0.696979535 | 2.104384818 | -5.784615132 | 1.36E-08 |
| LINC00202-1 | -0.864281923 | -1.611943196 | -5.783300809 | 1.37E-08 |
| DHRS2 | 0.912315338 | -3.934277157 | 5.783213843 | 1.37E-08 |
| FAM185A | -0.338008519 | 2.949732249 | -5.782586597 | 1.38E-08 |
| ST6GALNAC6 | 0.399681967 | 4.778234759 | 5.780273777 | 1.39E-08 |
| VEZF1 | 0.195024974 | 6.59515989 | 5.780209775 | 1.39E-08 |
| EIF2S3 | -0.269182749 | 8.165261325 | -5.780138739 | 1.39E-08 |
| COX4I1 | 0.293221505 | 7.885533642 | 5.779228141 | 1.40E-08 |
| AP006621.5 | -0.416903198 | 3.4304648 | -5.778191901 | 1.41E-08 |
| LINC01071 | -1.20596529 | 0.954523373 | -5.776766644 | 1.42E-08 |
| NCOA2 | 0.319828586 | 5.641994082 | 5.775898477 | 1.43E-08 |
| RP11-883A18.3 | -1.220331341 | -0.669226967 | -5.775864041 | 1.43E-08 |
| TRIM22 | -0.416839681 | 7.003629464 | -5.775785842 | 1.43E-08 |
| RP11-363E7.4 | -0.534581458 | 0.839610431 | -5.775287324 | 1.43E-08 |
| SCRIB | -0.287267536 | 5.712848685 | -5.774868325 | 1.44E-08 |
| TIPRL | -0.193159677 | 6.871534472 | -5.774648056 | 1.44E-08 |
| AC008984.2 | 1.198503024 | 2.004679828 | 5.773699328 | 1.44E-08 |
| KIF4A | 0.847312834 | 2.827543755 | 5.771463279 | 1.46E-08 |
| PHKA1P1 | 0.620271599 | 1.775222137 | 5.771379722 | 1.46E-08 |
| VEGFA | 0.853334261 | 3.941882706 | 5.769962541 | 1.47E-08 |
| VN1R83P | -0.462149746 | 2.530118584 | -5.76993675 | 1.47E-08 |
| AE000658.25 | -0.743682112 | -0.07873308 | -5.769921969 | 1.47E-08 |
| RP11-110I1.12 | -0.573122658 | 2.513899745 | -5.768784928 | 1.48E-08 |
| RP11-932O9.10 | 0.478912675 | 3.138058079 | 5.767197863 | 1.50E-08 |
| MAP3K11 | -0.238391453 | 5.742681641 | -5.765000708 | 1.52E-08 |
| PIN4 | -0.297389016 | 3.324537102 | -5.764903182 | 1.52E-08 |
| RP11-242D8.2 | -0.57896752 | 1.534711718 | -5.764175256 | 1.52E-08 |
| FAM229A | 0.445849704 | 5.033473432 | 5.763782631 | 1.53E-08 |
| MPI | -0.333146843 | 4.269940323 | -5.763753086 | 1.53E-08 |
| SNORA71C | 0.529873616 | 2.427452093 | 5.763120065 | 1.53E-08 |
| RGS16 | 0.987969194 | 0.42568868 | 5.760087663 | 1.56E-08 |
| ZNF169 | -0.329461305 | 2.546916067 | -5.759644155 | 1.56E-08 |
| DPY19L4 | -0.307323075 | 4.252175669 | -5.75958068 | 1.56E-08 |
| ZNF776 | 0.371333405 | 4.929806534 | 5.758703907 | 1.57E-08 |
| EYS | -0.84773555 | -3.783753661 | -5.758344915 | 1.57E-08 |
| MLLT1 | -0.335046204 | 6.484738046 | -5.758115125 | 1.57E-08 |
| RFC5 | 0.299973968 | 5.166901389 | 5.756304564 | 1.59E-08 |
| SSX2IP | 0.412025235 | 3.805293715 | 5.756039361 | 1.59E-08 |
| SSR3 | -0.203317505 | 6.625312432 | -5.755652768 | 1.60E-08 |
| DCAF12 | -0.536542671 | 5.318148371 | -5.755027961 | 1.60E-08 |
| RP5-991G20.4 | -0.568741538 | 2.84854127 | -5.754682196 | 1.60E-08 |
| KY | -0.993903491 | -2.189043661 | -5.753992281 | 1.61E-08 |
| MSH3 | -0.213860094 | 4.626326705 | -5.753420864 | 1.62E-08 |
| FAM203A | -0.620588198 | 0.888078938 | -5.751952622 | 1.63E-08 |
| ZNF117 | -0.434182607 | 4.875904937 | -5.750993822 | 1.64E-08 |
| SLC6A8 | 1.109723672 | 1.309604357 | 5.750136726 | 1.64E-08 |
| RP11-26J3.3 | 0.38575584 | 3.083801651 | 5.750086325 | 1.65E-08 |
| RP1-101K10.6 | 0.440269454 | 2.320917171 | 5.749992098 | 1.65E-08 |
| DNAJB12 | 0.202949607 | 5.618308568 | 5.748201856 | 1.66E-08 |
| MAP10 | -0.744764298 | 2.440005184 | -5.747101105 | 1.67E-08 |
| HNRNPCP2 | -0.256228238 | 9.396287539 | -5.747061667 | 1.67E-08 |
| CSTF3-AS1 | -0.598599168 | 0.892154123 | -5.746949416 | 1.67E-08 |
| ENGASE | -0.346895867 | 5.792988507 | -5.746495683 | 1.68E-08 |
| C3orf52 | 0.824453303 | -2.450957332 | 5.746387141 | 1.68E-08 |
| ZADH2 | -0.247344327 | 5.250069842 | -5.746206853 | 1.68E-08 |
| LINC00968 | -1.046813488 | 0.312023201 | -5.745912243 | 1.68E-08 |
| GORAB | 0.315439931 | 3.976455045 | 5.743762831 | 1.70E-08 |
| WAS | -0.296073304 | 8.132110668 | -5.743737964 | 1.70E-08 |
| RP11-754B17.1 | 0.712942546 | -0.186585573 | 5.742554546 | 1.72E-08 |
| USP2 | 1.054764814 | 0.077827978 | 5.742445711 | 1.72E-08 |
| C1orf174 | 0.229761776 | 5.022990996 | 5.74227246 | 1.72E-08 |
| FGD2 | -1.184778926 | 3.421322915 | -5.742008965 | 1.72E-08 |
| IL15RA | 0.503451862 | 3.509476713 | 5.741862887 | 1.72E-08 |
| MT-TT | 0.577429576 | 5.78033798 | 5.741646222 | 1.72E-08 |
| ZNF557 | 0.28003032 | 3.485074916 | 5.740465075 | 1.73E-08 |
| ARHGEF40 | -0.545705491 | 5.599123671 | -5.740180941 | 1.74E-08 |
| TNNC2 | 0.627139819 | 0.710645184 | 5.739930662 | 1.74E-08 |
| RAB33A | 0.735632757 | 3.251776174 | 5.739107726 | 1.75E-08 |
| RP11-556H2.3 | -0.707962364 | -0.397070018 | -5.73891386 | 1.75E-08 |
| TTLL12 | -0.460830493 | 5.870013721 | -5.73865187 | 1.75E-08 |
| CTC-436P18.1 | -0.626588313 | -0.68530789 | -5.73855751 | 1.75E-08 |
| CEP57L1P1 | -0.487731384 | 1.548512877 | -5.73814356 | 1.76E-08 |
| UMPS | -0.377120199 | 4.130360127 | -5.737556521 | 1.76E-08 |
| PPP5D1P | -0.526301949 | -0.879288099 | -5.736715241 | 1.77E-08 |
| TTC27 | -0.507215579 | 5.219062264 | -5.736116204 | 1.78E-08 |
| DEPDC1B | 0.626226382 | 2.58144876 | 5.736011495 | 1.78E-08 |
| TMEM71 | -0.519290366 | 6.051461408 | -5.735105018 | 1.79E-08 |
| RP4-798P15.3 | -1.005583673 | -2.392567228 | -5.734058473 | 1.80E-08 |
| MBTPS1 | -0.203148134 | 6.186561438 | -5.733707533 | 1.80E-08 |
| AC093495.4 | -0.649747996 | 0.024642206 | -5.733359743 | 1.80E-08 |
| RBM10 | -0.204714128 | 6.673505049 | -5.732199164 | 1.82E-08 |
| RP4-657D16.6 | 0.691970969 | 0.344047341 | 5.731745423 | 1.82E-08 |
| FAM227A | -1.019693978 | -1.203830992 | -5.7307688 | 1.83E-08 |
| ICOS | 0.972704922 | 2.079287684 | 5.730511596 | 1.83E-08 |
| KEAP1 | -0.305336349 | 5.69344098 | -5.730231496 | 1.84E-08 |
| RNU6-9 | 0.477634536 | 2.269517649 | 5.728334575 | 1.85E-08 |
| RP11-1029J19.5 | 0.786158941 | -1.011299858 | 5.726646496 | 1.87E-08 |
| AC004383.3 | -0.678078079 | -0.039441094 | -5.726441261 | 1.87E-08 |
| IL6ST | 0.606701487 | 5.360128391 | 5.725413963 | 1.88E-08 |
| CCR2 | -1.486522168 | 2.696787728 | -5.723942085 | 1.90E-08 |
| ZNF782 | -0.291134025 | 2.632403883 | -5.723834742 | 1.90E-08 |
| TRAPPC4 | 0.241044129 | 4.082430495 | 5.723405384 | 1.91E-08 |
| FAM86JP | -0.655976096 | 0.465885468 | -5.723256099 | 1.91E-08 |
| CTA-445C9.14 | -0.470081094 | 2.153248333 | -5.722282698 | 1.92E-08 |
| RPL34P22 | 0.738838272 | 1.102505879 | 5.721459246 | 1.93E-08 |
| STX11 | 0.784397237 | 7.2231553 | 5.721328983 | 1.93E-08 |
| GRPEL1 | 0.379277822 | 5.239789102 | 5.721269326 | 1.93E-08 |
| CERS6 | -0.338288878 | 6.765039681 | -5.720720891 | 1.93E-08 |
| SNORD53 | 0.667413082 | 0.924016061 | 5.720401853 | 1.94E-08 |
| ABCF1 | 0.198336741 | 6.553430435 | 5.720313508 | 1.94E-08 |
| AP2B1 | -0.273254178 | 7.177744691 | -5.719123343 | 1.95E-08 |
| AC073415.2 | -0.63471994 | 0.685171015 | -5.718396621 | 1.96E-08 |
| OARD1 | 0.209562371 | 4.838067608 | 5.718068471 | 1.96E-08 |
| RP11-764K9.1 | -0.717940336 | 1.54440229 | -5.717453112 | 1.97E-08 |
| AC004381.7 | -0.618191576 | 0.36915238 | -5.717266508 | 1.97E-08 |
| ZNF599 | -0.484404219 | 0.756554781 | -5.716249082 | 1.98E-08 |
| S1PR4 | -0.553176725 | 7.020933482 | -5.716039176 | 1.98E-08 |
| RP11-488C13.5 | -0.308477285 | 4.307218328 | -5.715576592 | 1.99E-08 |
| ITFG1 | -0.259518482 | 4.749559559 | -5.715046735 | 1.99E-08 |
| RP1-93H18.7 | -0.756689015 | 0.160720477 | -5.714128509 | 2.00E-08 |
| NUDT3 | -0.232905304 | 6.806977665 | -5.7129278 | 2.02E-08 |
| CHORDC2P | -0.632730607 | -0.638577233 | -5.712389933 | 2.02E-08 |
| ABCF2P1 | -0.437268022 | 2.03003291 | -5.710324937 | 2.05E-08 |
| DNAH17 | 1.011354438 | -2.911243787 | 5.710149251 | 2.05E-08 |
| ACADSB | -0.321922887 | 4.075193155 | -5.709662277 | 2.05E-08 |
| AC003090.1 | -0.871082949 | -2.503390872 | -5.709279735 | 2.06E-08 |
| PPP2R5C | 0.259906254 | 5.683945113 | 5.708821901 | 2.06E-08 |
| RP11-142L4.3 | -0.325331728 | 5.30763816 | -5.708622899 | 2.07E-08 |
| C21orf2 | 0.32062577 | 3.776453947 | 5.708167034 | 2.07E-08 |
| ZNF836 | -0.370197465 | 1.985292848 | -5.707795432 | 2.08E-08 |
| LRFN4 | -0.393531122 | 5.841579373 | -5.707388404 | 2.08E-08 |
| MTMR10 | 0.254921052 | 5.362906115 | 5.707241527 | 2.08E-08 |
| ZNF440 | 0.335188523 | 3.123912214 | 5.707005664 | 2.08E-08 |
| CREB3L4 | -0.466636645 | 4.690132089 | -5.706779144 | 2.09E-08 |
| MRPS36P1 | -0.331608616 | 4.451933583 | -5.706765298 | 2.09E-08 |
| COMMD10 | -0.265679716 | 4.705072999 | -5.706615216 | 2.09E-08 |
| FNIP2 | 0.502204598 | 3.595161905 | 5.706324107 | 2.09E-08 |
| PTGES3 | -0.263180903 | 8.180515922 | -5.705369887 | 2.10E-08 |
| PHLDB2 | 0.727461566 | -1.224542286 | 5.705311531 | 2.10E-08 |
| SRPRB | -0.310873207 | 4.917267813 | -5.704308695 | 2.12E-08 |
| PTTG1IP | -0.326377093 | 8.476648746 | -5.704155323 | 2.12E-08 |
| AC011043.1 | -0.829733631 | 3.831867602 | -5.704071426 | 2.12E-08 |
| RP11-767N6.7 | -0.569320037 | -0.040931721 | -5.703628135 | 2.12E-08 |
| ZNF341 | -0.275222065 | 2.629432977 | -5.703394268 | 2.13E-08 |
| MRPS18B | -0.301086706 | 6.041274254 | -5.702938137 | 2.13E-08 |
| SPTBN4 | 1.040425302 | -3.339082427 | 5.700997891 | 2.15E-08 |
| XKR3 | -0.888982392 | -1.177016299 | -5.700290045 | 2.16E-08 |
| JMJD4 | -0.290878973 | 3.454172905 | -5.700054249 | 2.17E-08 |
| TACO1 | 0.206938626 | 4.761171166 | 5.696875029 | 2.20E-08 |
| SP2 | 0.231601288 | 6.087910407 | 5.695359811 | 2.22E-08 |
| SNHG9 | 0.314788629 | 4.560394485 | 5.695231244 | 2.22E-08 |
| PSMC2 | 0.17983532 | 6.590686636 | 5.694876641 | 2.23E-08 |
| HIST1H4K | -0.75815272 | 0.931029426 | -5.694136821 | 2.24E-08 |
| ACVR2B-AS1 | 0.791215904 | -1.665892434 | 5.693986919 | 2.24E-08 |
| AC108463.2 | -0.747011211 | 1.625834686 | -5.693887987 | 2.24E-08 |
| ZNF222 | 0.516468701 | 3.38806372 | 5.692260663 | 2.26E-08 |
| RP11-458N5.1 | -0.538104718 | 2.313965613 | -5.691958495 | 2.26E-08 |
| RP11-452L6.1 | -0.616566406 | 4.244031488 | -5.691862455 | 2.26E-08 |
| SLC10A3 | 0.267979569 | 6.181422853 | 5.689502288 | 2.29E-08 |
| OPTN | 0.792977432 | 4.488264232 | 5.689338217 | 2.30E-08 |
| RP11-799M12.2 | -0.697851277 | 0.211127419 | -5.689320329 | 2.30E-08 |
| KIAA1731 | 0.222573325 | 4.295491732 | 5.688011284 | 2.31E-08 |
| IER5 | 0.567959835 | 7.725493537 | 5.687937974 | 2.31E-08 |
| BCL2L11 | 0.706715054 | 4.560153857 | 5.687264045 | 2.32E-08 |
| RP11-762E8.1 | -0.864847593 | -1.44155172 | -5.686842844 | 2.33E-08 |
| RP11-989F5.3 | -0.768994676 | 0.32368908 | -5.686328866 | 2.33E-08 |
| RP11-10O17.1 | -0.943700877 | -1.657561576 | -5.685626271 | 2.34E-08 |
| RAB32 | -0.482201402 | 7.454021326 | -5.685286698 | 2.35E-08 |
| SGOL1 | 0.624284958 | 2.649027118 | 5.684180694 | 2.36E-08 |
| RP11-332H14.1 | -0.704064633 | 0.84635659 | -5.684166698 | 2.36E-08 |
| FOXJ2 | 0.224206138 | 5.612408166 | 5.683976374 | 2.36E-08 |
| SEC24A | 0.291255709 | 4.901236679 | 5.683740986 | 2.37E-08 |
| AC007256.5 | 0.292762552 | 2.979902532 | 5.682845438 | 2.38E-08 |
| RNU6-1157P | 0.571673759 | 0.272258749 | 5.682684856 | 2.38E-08 |
| FZD6 | 1.07473323 | 2.476739281 | 5.682164882 | 2.39E-08 |
| TGFBR1 | 0.368516396 | 5.44490037 | 5.682037622 | 2.39E-08 |
| TMPRSS5 | 0.608656125 | 0.407689486 | 5.682027794 | 2.39E-08 |
| ATP2B1 | 0.522730379 | 6.102923599 | 5.681511215 | 2.40E-08 |
| MIR3136 | 0.695161641 | 0.958947664 | 5.681443436 | 2.40E-08 |
| CTBP2P8 | -0.40901789 | 2.044173019 | -5.681234382 | 2.40E-08 |
| DMPK | 0.717139297 | 1.709557338 | 5.68040309 | 2.41E-08 |
| TBC1D24 | -0.385294893 | 3.182848407 | -5.680160729 | 2.41E-08 |
| MRPL34 | -0.262571325 | 5.742829189 | -5.679115817 | 2.43E-08 |
| RP11-16E23.4 | 0.333950836 | 3.632213454 | 5.678235312 | 2.44E-08 |
| RP11-443P15.2 | -0.867306345 | 0.709931939 | -5.677815094 | 2.45E-08 |
| RP11-1186N24.5 | 0.626351394 | -0.182686086 | 5.677797749 | 2.45E-08 |
| PIK3AP1 | -0.429312227 | 7.477895998 | -5.677781888 | 2.45E-08 |
| HMGN2P6 | 0.49682828 | 3.638088202 | 5.677684759 | 2.45E-08 |
| SETD3 | -0.219991705 | 6.125374081 | -5.67732133 | 2.45E-08 |
| ARHGAP30 | -0.364327788 | 8.186831139 | -5.677059978 | 2.46E-08 |
| MLLT3 | 0.908120321 | 3.050037785 | 5.676120821 | 2.47E-08 |
| ANKRD32 | 0.287468033 | 2.982334814 | 5.675544053 | 2.48E-08 |
| KLHL26 | 0.364920032 | 2.898763198 | 5.675413707 | 2.48E-08 |
| RP11-171I2.2 | -0.748268526 | 2.80532317 | -5.673628577 | 2.50E-08 |
| RP11-421N8.1 | -0.285142169 | 7.07581992 | -5.673503337 | 2.50E-08 |
| RP3-322L4.2 | -0.753509681 | -0.352205276 | -5.673423084 | 2.50E-08 |
| PIN1 | 0.1525066 | 5.288459508 | 5.672296887 | 2.52E-08 |
| SKOR1 | 0.554587957 | 0.172810067 | 5.672255739 | 2.52E-08 |
| SNORA34 | 0.604640435 | 2.243134405 | 5.671283869 | 2.53E-08 |
| AC004840.9 | -0.802133709 | 4.069225783 | -5.670406729 | 2.55E-08 |
| NR2F1 | 0.872428067 | -3.611431348 | 5.669919093 | 2.55E-08 |
| DND1P1 | -0.793005878 | 1.546925334 | -5.66915649 | 2.56E-08 |
| ZC3H4 | -0.321387972 | 5.208352765 | -5.668044903 | 2.58E-08 |
| PISD | -0.240923203 | 5.881203735 | -5.667515202 | 2.59E-08 |
| BRAFP1 | 0.568903489 | -0.2898639 | 5.667492326 | 2.59E-08 |
| MNS1 | 0.671498255 | 2.676299876 | 5.666306919 | 2.60E-08 |
| AC105760.2 | -0.472317083 | 0.176046216 | -5.66559995 | 2.61E-08 |
| RP11-631M6.2 | 0.368727097 | 3.893959401 | 5.665486561 | 2.62E-08 |
| TFPI2 | 0.98144202 | -2.746954389 | 5.665106961 | 2.62E-08 |
| RP11-30P6.1 | 0.274535403 | 2.844161064 | 5.663574386 | 2.64E-08 |
| CNP | -0.263371762 | 5.217352345 | -5.663422006 | 2.64E-08 |
| PXT1 | 0.650208668 | -1.313644573 | 5.66256758 | 2.66E-08 |
| TBC1D23 | 0.223387952 | 5.197526833 | 5.662356341 | 2.66E-08 |
| Z95704.4 | -1.066260347 | -0.015225205 | -5.659779437 | 2.70E-08 |
| MAP3K7 | -0.158799646 | 5.590037343 | -5.659331387 | 2.70E-08 |
| C1orf134 | -0.51297961 | 2.771650876 | -5.659121155 | 2.71E-08 |
| CRLS1 | -0.264806671 | 4.717874489 | -5.658412141 | 2.72E-08 |
| RP11-474L23.3 | -0.645889004 | 0.198307065 | -5.658134561 | 2.72E-08 |
| BRIX1 | 0.245157477 | 4.785584813 | 5.658043269 | 2.72E-08 |
| GIMAP4 | -1.164708622 | 4.390237992 | -5.657450365 | 2.73E-08 |
| S1PR2 | 0.644676523 | 1.306584548 | 5.657072186 | 2.74E-08 |
| AC007278.2 | 1.198415909 | 1.993119513 | 5.656826728 | 2.74E-08 |
| MED21 | 0.302006442 | 5.394016703 | 5.656464638 | 2.75E-08 |
| RP11-755B10.2 | 0.69200541 | -0.561470978 | 5.655567145 | 2.76E-08 |
| HIST1H4D | -0.744881791 | 0.274884311 | -5.65403125 | 2.78E-08 |
| USF2 | 0.177294629 | 7.288664865 | 5.653973557 | 2.78E-08 |
| CFDP1 | -0.218923992 | 5.259057936 | -5.653454837 | 2.79E-08 |
| TSKU | 1.049175786 | -1.688034648 | 5.653213947 | 2.80E-08 |
| AC005534.8 | 0.646995538 | 1.187414253 | 5.653001938 | 2.80E-08 |
| FAM173A | -0.348146264 | 4.728935716 | -5.652958222 | 2.80E-08 |
| C19orf35 | -1.059273132 | 1.51887269 | -5.652826166 | 2.80E-08 |
| RNU7-181P | 0.775902508 | 1.566287097 | 5.652306959 | 2.81E-08 |
| ERI3-IT1 | -0.663809859 | -0.112775758 | -5.65209978 | 2.81E-08 |
| AL713999.1 | 0.612814312 | 3.457426674 | 5.652069938 | 2.81E-08 |
| PTTG3P | 0.565133369 | 1.644105419 | 5.651488345 | 2.82E-08 |
| C16orf86 | -0.377236549 | 3.735889868 | -5.650254225 | 2.84E-08 |
| AC019221.4 | 0.659759387 | -0.440539398 | 5.650128261 | 2.84E-08 |
| CPSF3 | -0.224789173 | 6.257614469 | -5.649835254 | 2.85E-08 |
| RP4-614O4.11 | -0.460799997 | 2.292510304 | -5.64978273 | 2.85E-08 |
| CDKL3 | -0.550751974 | -0.661033042 | -5.649502827 | 2.85E-08 |
| OST4 | -0.219901992 | 8.683935256 | -5.649287965 | 2.86E-08 |
| TSPYL2 | 0.744824213 | 6.126195391 | 5.648932389 | 2.86E-08 |
| PCNXL3 | 0.173594815 | 5.628773372 | 5.646073364 | 2.91E-08 |
| SCNN1A | 0.598647209 | 0.119475046 | 5.645850941 | 2.91E-08 |
| CTB-118N6.3 | -0.451191463 | 1.791389241 | -5.645489888 | 2.92E-08 |
| RP11-598D12.4 | 0.821416115 | -2.395980106 | 5.644946092 | 2.92E-08 |
| AC026271.5 | 0.21077152 | 6.597840757 | 5.644677618 | 2.93E-08 |
| CTNNB1 | 0.306961069 | 7.400547157 | 5.644674866 | 2.93E-08 |
| RP11-361H10.3 | -0.641912747 | -0.304883482 | -5.644651792 | 2.93E-08 |
| CCDC150 | 0.605882962 | 0.087184424 | 5.644059271 | 2.94E-08 |
| YPEL4 | 1.016503882 | 0.866384915 | 5.643878064 | 2.94E-08 |
| ZNF320 | -0.441555249 | 3.419421502 | -5.643134782 | 2.95E-08 |
| ZMYM2 | 0.226733541 | 6.098753316 | 5.642966185 | 2.96E-08 |
| RP11-774O3.3 | -0.471917937 | 2.012630974 | -5.642963194 | 2.96E-08 |
| B3GNT8 | -0.723515283 | 4.978412067 | -5.642663826 | 2.96E-08 |
| RP11-571L19.8 | 0.463073982 | 1.757998238 | 5.64250202 | 2.96E-08 |
| PTGES3P3 | -0.339326559 | 5.321391778 | -5.642271994 | 2.97E-08 |
| SARS | -0.222111333 | 6.492085614 | -5.641455672 | 2.98E-08 |
| CRKL | 0.189770294 | 6.902231329 | 5.641318593 | 2.98E-08 |
| COL15A1 | 0.912419082 | -2.867831536 | 5.640975729 | 2.99E-08 |
| TMEM63A | -0.318951975 | 5.460638977 | -5.640126424 | 3.00E-08 |
| YBX1 | 0.211476662 | 10.43345011 | 5.639437358 | 3.01E-08 |
| HNRNPU-AS1 | -0.516641808 | 5.239275744 | -5.63942206 | 3.01E-08 |
| CHRNA10 | -0.516527125 | 0.467311082 | -5.638976552 | 3.02E-08 |
| CENPV | 0.584216287 | 4.101255562 | 5.638969512 | 3.02E-08 |
| RP11-65E22.2 | -0.736123789 | -0.568417072 | -5.638555483 | 3.03E-08 |
| UBE2H | 0.356864322 | 5.774349843 | 5.637834239 | 3.04E-08 |
| ATF6 | -0.224494134 | 6.00629882 | -5.637743062 | 3.04E-08 |
| RP11-366L5.1 | -0.555846952 | 1.676966901 | -5.637213017 | 3.05E-08 |
| RNF44 | -0.221078901 | 6.999658997 | -5.636389206 | 3.06E-08 |
| SNUPN | -0.230389179 | 3.914170674 | -5.636177703 | 3.07E-08 |
| SRSF4 | 0.219060391 | 7.368533105 | 5.634731587 | 3.09E-08 |
| AC022182.3 | -0.838316828 | 2.310163862 | -5.634680878 | 3.09E-08 |
| PYROXD2 | -0.387578389 | 3.573632653 | -5.634612276 | 3.09E-08 |
| WDR43 | 0.255987989 | 6.348826818 | 5.633851399 | 3.11E-08 |
| ZFYVE16 | -0.350877766 | 4.221826918 | -5.633560864 | 3.11E-08 |
| RGS18 | -0.690826078 | 6.97134336 | -5.632784538 | 3.12E-08 |
| AKAP12 | 1.153300654 | -0.520128513 | 5.632572841 | 3.13E-08 |
| ZNF610 | -0.777743073 | 1.426246153 | -5.631734275 | 3.14E-08 |
| AKIRIN2 | 0.302660575 | 8.136857447 | 5.631508196 | 3.15E-08 |
| RP11-196G18.24 | -0.527081917 | 4.989346966 | -5.630157087 | 3.17E-08 |
| CHST13 | -0.786819184 | 4.636564713 | -5.628932887 | 3.19E-08 |
| NUP43 | -0.311351028 | 4.419629204 | -5.628148589 | 3.20E-08 |
| NDUFA6 | 0.198655024 | 6.775070285 | 5.628127088 | 3.20E-08 |
| CTD-2353F22.1 | -0.597611247 | 4.028097875 | -5.62734016 | 3.22E-08 |
| RASSF4 | -0.915637355 | 4.116603368 | -5.627036181 | 3.22E-08 |
| CCR4 | 0.884633515 | 1.620775229 | 5.626814569 | 3.23E-08 |
| RP1-239B22.5 | 0.74059562 | 1.019263014 | 5.626304326 | 3.24E-08 |
| CPB2-AS1 | -0.695455377 | -1.910683462 | -5.625639354 | 3.25E-08 |
| ACP1 | 0.222025937 | 6.599053808 | 5.625334201 | 3.25E-08 |
| GADD45A | 0.614790945 | 6.919031459 | 5.625211992 | 3.25E-08 |
| RP11-762H8.2 | -0.498670589 | 1.609214734 | -5.624780996 | 3.26E-08 |
| CCDC23 | 0.201657017 | 5.956055207 | 5.624228408 | 3.27E-08 |
| METRNL | 0.720885791 | 5.843593261 | 5.623875943 | 3.28E-08 |
| APH1A | 0.212528343 | 7.52045388 | 5.623687546 | 3.28E-08 |
| AP000266.7 | -0.618645604 | -0.604100857 | -5.622322682 | 3.31E-08 |
| FBXO28 | 0.185995991 | 4.752218345 | 5.621461481 | 3.32E-08 |
| MCOLN2 | 1.072874203 | 1.110481929 | 5.621195712 | 3.33E-08 |
| RP11-546J1.1 | -0.739375247 | -0.480678327 | -5.621176379 | 3.33E-08 |
| AC007278.3 | 1.211701255 | 3.239911221 | 5.61893047 | 3.37E-08 |
| LGALSL | 0.969710992 | 2.137532195 | 5.618444582 | 3.38E-08 |
| AC003104.1 | 0.594241966 | 2.688188957 | 5.618378206 | 3.38E-08 |
| TNXB | 0.842994555 | 0.734903979 | 5.617755077 | 3.39E-08 |
| CIT | 0.727409731 | 2.979594779 | 5.616840185 | 3.41E-08 |
| SPTLC1 | -0.191694775 | 6.000451809 | -5.616388564 | 3.41E-08 |
| KDELR1 | -0.222981367 | 7.383848116 | -5.615302167 | 3.43E-08 |
| RP3-428L16.2 | 0.52525879 | 0.770280556 | 5.614564366 | 3.45E-08 |
| IQCG | -0.397176458 | 2.629342431 | -5.613865165 | 3.46E-08 |
| CTD-2619J13.23 | -0.720280411 | 0.336570524 | -5.613076792 | 3.48E-08 |
| TRAV12-2 | -0.88577456 | 0.267728285 | -5.6114442 | 3.51E-08 |
| EID3 | 0.633188328 | 2.978799328 | 5.611150038 | 3.51E-08 |
| ARRDC2 | 0.344675805 | 6.401600395 | 5.610725292 | 3.52E-08 |
| OS9 | -0.239374649 | 8.445140823 | -5.610158373 | 3.53E-08 |
| NFIL3 | 0.727054783 | 8.044250204 | 5.609580842 | 3.54E-08 |
| KIAA1407 | -0.378428357 | 3.04371379 | -5.609524475 | 3.54E-08 |
| RP11-164P12.4 | 0.746341437 | -0.081477837 | 5.608238287 | 3.57E-08 |
| PSMD12P | 0.445706451 | 0.548596576 | 5.608089024 | 3.57E-08 |
| SELO | 0.308898309 | 6.540565126 | 5.607635276 | 3.58E-08 |
| GJB2 | 0.976502779 | -1.987803477 | 5.607557762 | 3.58E-08 |
| DEK | 0.208905748 | 7.76657367 | 5.605088103 | 3.63E-08 |
| RP11-432J24.3 | -0.803125777 | 0.916047014 | -5.604004085 | 3.65E-08 |
| SFMBT1 | 0.654612116 | 2.811876134 | 5.602747286 | 3.67E-08 |
| GM2A | -0.456253768 | 6.309286075 | -5.602407073 | 3.68E-08 |
| KMT2B | 0.427787465 | 0.607624048 | 5.602147194 | 3.69E-08 |
| POLM | -0.27679636 | 4.812980154 | -5.60123851 | 3.70E-08 |
| RP11-332H14.2 | 0.481488911 | 3.284398569 | 5.600453575 | 3.72E-08 |
| GAPDHP40 | 0.406406414 | 3.276759921 | 5.600406633 | 3.72E-08 |
| RP3-431A14.4 | -0.684996592 | 0.758292375 | -5.600254226 | 3.72E-08 |
| XPNPEP1 | 0.218196302 | 6.051081097 | 5.599536533 | 3.74E-08 |
| CTD-3203P2.1 | -0.583852241 | 0.345330432 | -5.599457092 | 3.74E-08 |
| NEMF | 0.155811228 | 5.473973678 | 5.598584683 | 3.76E-08 |
| SLC30A1 | 0.511695139 | 5.251720214 | 5.598369379 | 3.76E-08 |
| SLC7A6 | -0.375308841 | 5.486892067 | -5.597989895 | 3.77E-08 |
| RP11-3J10.4 | -0.342141372 | 5.560789431 | -5.596823225 | 3.79E-08 |
| SAPCD1 | 0.486747264 | 3.504053583 | 5.596654267 | 3.80E-08 |
| MYL12A | -0.327931791 | 8.597096975 | -5.596465436 | 3.80E-08 |
| RP11-346E8.1 | -0.582682034 | -0.574356293 | -5.594400311 | 3.84E-08 |
| RP11-389C8.2 | -1.093615072 | -0.530808586 | -5.593813322 | 3.86E-08 |
| BTBD2 | -0.252688884 | 6.41872835 | -5.590508437 | 3.93E-08 |
| UBA7 | -0.413250652 | 7.598931935 | -5.590090682 | 3.93E-08 |
| YIPF3 | -0.160591747 | 6.88797284 | -5.589623005 | 3.94E-08 |
| GNB1L | -0.444080386 | 0.813045788 | -5.589388125 | 3.95E-08 |
| RHOQP1 | 0.277808434 | 3.331395095 | 5.589218715 | 3.95E-08 |
| RP11-83N9.5 | -0.619468975 | 0.066387565 | -5.588474849 | 3.97E-08 |
| RNF114 | -0.2082259 | 7.710701501 | -5.58779909 | 3.98E-08 |
| PDZD8 | 0.605256944 | 6.965082965 | 5.587115362 | 4.00E-08 |
| EFNB2 | 0.965562196 | -0.210158908 | 5.585990185 | 4.02E-08 |
| TK1 | 0.732647087 | 5.481813412 | 5.585468566 | 4.03E-08 |
| RP11-415F23.3 | -0.610895906 | 1.78507606 | -5.584956279 | 4.04E-08 |
| NBEAL1 | -0.292395864 | 2.148903409 | -5.584567888 | 4.05E-08 |
| WDFY4 | -0.405902765 | 5.61859322 | -5.584556222 | 4.05E-08 |
| ACHE | 1.292344938 | -0.359842226 | 5.584121643 | 4.06E-08 |
| RP11-24C3.2 | 0.585496081 | -0.777781933 | 5.582916745 | 4.09E-08 |
| ZKSCAN8 | 0.322282439 | 5.581902556 | 5.582062416 | 4.11E-08 |
| DMWD | 0.638315729 | 2.118413687 | 5.581609837 | 4.12E-08 |
| RP11-159D12.2 | 0.514010351 | 4.144659748 | 5.58131603 | 4.12E-08 |
| HP1BP3 | -0.231441887 | 7.341485091 | -5.580969724 | 4.13E-08 |
| IRF2BP2 | 0.360803776 | 9.443046576 | 5.580327986 | 4.15E-08 |
| LEMD2 | 0.187424752 | 5.280983948 | 5.579790847 | 4.16E-08 |
| MTX2 | -0.259928377 | 5.166193127 | -5.579508978 | 4.16E-08 |
| ZNF706 | 0.271842112 | 5.658348165 | 5.578720467 | 4.18E-08 |
| MRP63 | -0.298978846 | 6.2045368 | -5.57790135 | 4.20E-08 |
| PRKXP1 | -0.667103692 | 2.382922206 | -5.577537185 | 4.21E-08 |
| CTD-2033A16.3 | -0.665950538 | -0.43010315 | -5.577186031 | 4.22E-08 |
| CTD-2256P15.2 | -0.389042249 | 1.889138125 | -5.576701961 | 4.23E-08 |
| RP11-50D9.1 | 0.330747178 | 4.452378626 | 5.576626956 | 4.23E-08 |
| RP11-162D16.2 | -0.687803202 | 0.488988382 | -5.575888377 | 4.25E-08 |
| CDC25C | 0.77441673 | 1.347451446 | 5.573892399 | 4.29E-08 |
| CARF | -0.333007129 | 3.525173449 | -5.572385241 | 4.33E-08 |
| TOPORS | 0.385563822 | 6.073488432 | 5.571846379 | 4.34E-08 |
| IL8 | 1.194183694 | 9.658598017 | 5.570982601 | 4.36E-08 |
| ALDH1B1 | -0.60257823 | 3.700878352 | -5.570399158 | 4.37E-08 |
| ANAPC5 | -0.269033878 | 6.094819801 | -5.569784788 | 4.39E-08 |
| ALDH18A1 | -0.46322297 | 6.394586492 | -5.569691426 | 4.39E-08 |
| PWWP2B | -0.381693251 | 5.398669813 | -5.569460322 | 4.40E-08 |
| INHBB | 0.773087734 | -1.827639117 | 5.569228524 | 4.40E-08 |
| PRKAB2 | 0.335858713 | 5.079551633 | 5.568945816 | 4.41E-08 |
| PSMD1 | -0.221858282 | 6.108144877 | -5.568924434 | 4.41E-08 |
| EMR2 | -0.482446104 | 6.320482743 | -5.568791378 | 4.41E-08 |
| IDE | -0.2574445 | 4.779591966 | -5.568491538 | 4.42E-08 |
| RP11-484D2.3 | -0.665714837 | 1.50323655 | -5.567409591 | 4.44E-08 |
| XPNPEP3 | 0.22377687 | 3.30251124 | 5.567071862 | 4.45E-08 |
| HMGB1P24 | 0.513729098 | 0.820137025 | 5.566573167 | 4.46E-08 |
| RP11-552F3.4 | 0.571212898 | 1.267389839 | 5.566393085 | 4.47E-08 |
| RP11-44N11.1 | 0.771914641 | 0.157984381 | 5.565908186 | 4.48E-08 |
| RP11-973H7.3 | -0.469484424 | 1.500655035 | -5.565669829 | 4.49E-08 |
| TXNDC2 | 0.821393194 | -1.98138644 | 5.565555407 | 4.49E-08 |
| AC087294.2 | -0.516803827 | 0.547694895 | -5.564365532 | 4.52E-08 |
| LLPH | 0.206919225 | 5.661670134 | 5.563111225 | 4.55E-08 |
| RP11-48B3.4 | 0.60588488 | 2.00895761 | 5.562206515 | 4.57E-08 |
| TRGC2 | -0.961466393 | 6.849735186 | -5.562087762 | 4.57E-08 |
| PLOD2 | 1.348017897 | -1.987755671 | 5.561573608 | 4.59E-08 |
| UQCR10 | -0.295243979 | 7.096737601 | -5.56109169 | 4.60E-08 |
| PIERCE2 | -0.695142233 | 1.240902884 | -5.559760266 | 4.63E-08 |
| KPNB1 | -0.24758159 | 7.541420078 | -5.559343188 | 4.64E-08 |
| BSDC1 | 0.267961066 | 5.810302856 | 5.558869919 | 4.65E-08 |
| LRP8 | 0.34651665 | 3.892955094 | 5.5586492 | 4.66E-08 |
| FTH1P12 | 0.610837189 | 4.463029261 | 5.558383848 | 4.66E-08 |
| HSPE1P18 | -1.027988611 | 1.276000916 | -5.557439485 | 4.69E-08 |
| CTD-2012J19.1 | 0.344805695 | 3.434370094 | 5.556500109 | 4.71E-08 |
| THEM6 | -0.357152974 | 4.143486478 | -5.556301653 | 4.72E-08 |
| AGL | -0.270552959 | 4.139073427 | -5.556277922 | 4.72E-08 |
| TATDN1P1 | -0.281472822 | 5.133508329 | -5.555796758 | 4.73E-08 |
| RP11-1017G21.6 | -0.659372451 | -0.597926234 | -5.555665121 | 4.73E-08 |
| AIF1 | -0.451495229 | 8.893427975 | -5.555560584 | 4.74E-08 |
| FAM57B | 0.770860085 | -1.627182403 | 5.555093285 | 4.75E-08 |
| XPO4 | 0.202717143 | 4.828385537 | 5.554080896 | 4.77E-08 |
| DENND4B | -0.171849136 | 6.841593713 | -5.553913889 | 4.78E-08 |
| RP11-803B1.8 | -0.532828727 | 0.339938152 | -5.553306082 | 4.79E-08 |
| LINC00899 | -0.940604037 | 2.783667766 | -5.551878222 | 4.83E-08 |
| LRRC41 | -0.232051734 | 5.606924509 | -5.551767024 | 4.83E-08 |
| SPATA9 | -0.95002476 | -1.005233502 | -5.551611502 | 4.84E-08 |
| TPX2 | 0.629929012 | 5.703607216 | 5.551328956 | 4.84E-08 |
| RP11-467H10.1 | 0.359111347 | 2.762745516 | 5.550917838 | 4.86E-08 |
| HMGB3 | 0.536361053 | 5.130877521 | 5.549533951 | 4.89E-08 |
| NHLRC4 | -0.622403985 | 2.251904777 | -5.548289761 | 4.92E-08 |
| RP11-255C15.3 | -0.581754992 | -0.846979071 | -5.547510421 | 4.94E-08 |
| PGAM1P7 | 0.719577325 | 1.051878837 | 5.547363487 | 4.95E-08 |
| RP11-205M20.7 | 0.512147141 | 3.07736608 | 5.547287051 | 4.95E-08 |
| C11orf68 | -0.320101071 | 5.686397963 | -5.547106495 | 4.96E-08 |
| LIMK2 | 0.468047968 | 5.122796981 | 5.546899283 | 4.96E-08 |
| PHIP | 0.183597767 | 6.576055493 | 5.54645236 | 4.97E-08 |
| ATP6V1D | -0.280981859 | 4.332362592 | -5.545413331 | 5.00E-08 |
| HSD17B1P1 | -0.668066417 | 0.490034383 | -5.544928672 | 5.01E-08 |
| GSDMD | -0.25899192 | 6.535873945 | -5.54415458 | 5.03E-08 |
| AKIRIN1 | 0.27240081 | 7.877426806 | 5.54347224 | 5.05E-08 |
| SERPINI2 | -1.132789684 | 2.032016448 | -5.543298415 | 5.06E-08 |
| STYX | -0.19711502 | 5.60202594 | -5.541886284 | 5.10E-08 |
| RFPL3S | 0.540936405 | 1.447153702 | 5.541669725 | 5.10E-08 |
| ANKLE2 | 0.221487851 | 5.933504142 | 5.541515887 | 5.11E-08 |
| CCDC51 | -0.39130273 | 3.293488515 | -5.541442454 | 5.11E-08 |
| PYROXD1 | 0.334092501 | 3.827126574 | 5.540871216 | 5.12E-08 |
| RP11-362F19.1 | -1.165635625 | -1.586922242 | -5.539668449 | 5.16E-08 |
| RBPJ | 0.314142857 | 5.987007972 | 5.539393467 | 5.16E-08 |
| CTD-2547E10.2 | 0.366889784 | 3.517348321 | 5.538870361 | 5.18E-08 |
| BCKDK | -0.260791024 | 5.690156128 | -5.538736781 | 5.18E-08 |
| FAM172BP | 0.651495932 | -2.379634911 | 5.538406079 | 5.19E-08 |
| AC009961.3 | 0.345917512 | 1.641667327 | 5.537674083 | 5.21E-08 |
| SUGP1 | -0.190042913 | 4.953131218 | -5.536336182 | 5.25E-08 |
| RP11-159F24.5 | -0.601520467 | 0.140664447 | -5.535975049 | 5.26E-08 |
| AMFR | 0.252248595 | 5.97658814 | 5.534738001 | 5.29E-08 |
| PUS1 | -0.266511998 | 4.871408537 | -5.533299737 | 5.33E-08 |
| UBXN2B | -0.366239583 | 5.711475047 | -5.532043521 | 5.37E-08 |
| ATRIP | -0.249877542 | 3.727747135 | -5.531637345 | 5.38E-08 |
| RP5-1103G7.4 | -0.491506363 | 3.504351038 | -5.530911072 | 5.40E-08 |
| MED13L | 0.282554288 | 7.656062597 | 5.530266585 | 5.42E-08 |
| CTD-3148I10.15 | -0.57638461 | -1.32455218 | -5.529709688 | 5.44E-08 |
| PPIAP22 | -0.324439047 | 10.63215011 | -5.529706485 | 5.44E-08 |
| G6PC3 | -0.283058432 | 4.859466491 | -5.529016117 | 5.46E-08 |
| RFK | 0.281687909 | 4.867215462 | 5.528953231 | 5.46E-08 |
| RP11-848P1.9 | 0.541523718 | 1.958336562 | 5.528918838 | 5.46E-08 |
| TMEM11 | 0.278111512 | 5.275871243 | 5.528557328 | 5.47E-08 |
| NUDCD3 | -0.244079463 | 4.793060821 | -5.528229853 | 5.48E-08 |
| AMBRA1 | 0.201260809 | 4.64464226 | 5.527774955 | 5.49E-08 |
| RP5-1180E21.5 | -0.54993116 | 3.423283797 | -5.527490009 | 5.50E-08 |
| GNPTAB | -0.33598882 | 6.201862531 | -5.526297813 | 5.54E-08 |
| RP11-55K13.1 | -0.673738314 | -1.984248258 | -5.525841364 | 5.55E-08 |
| ASXL1 | 0.186867317 | 6.037654036 | 5.525647545 | 5.56E-08 |
| AC009506.1 | -0.470223084 | 5.890352127 | -5.52401052 | 5.61E-08 |
| PLK3 | 0.595070845 | 5.753507986 | 5.523772538 | 5.61E-08 |
| RN7SL121P | -0.812198977 | -0.794280672 | -5.523281047 | 5.63E-08 |
| ALG11 | 0.255121802 | 2.863036593 | 5.522622449 | 5.65E-08 |
| KIAA1524 | 0.387022377 | 3.740073586 | 5.521818842 | 5.67E-08 |
| RP11-396C23.4 | -0.556474586 | 1.436643989 | -5.520730718 | 5.70E-08 |
| KIFC1 | 0.766136453 | 4.319259585 | 5.520702248 | 5.71E-08 |
| PHC2 | 0.332539907 | 6.749938847 | 5.520263283 | 5.72E-08 |
| DGKE | 0.418937202 | 3.662751538 | 5.520164758 | 5.72E-08 |
| DKK3 | 0.847978073 | -2.661062691 | 5.520130622 | 5.72E-08 |
| POLL | -0.212406743 | 4.196256149 | -5.520106067 | 5.72E-08 |
| AC012358.7 | -0.680472614 | -0.239550249 | -5.519693796 | 5.74E-08 |
| AP1M1 | -0.227918192 | 6.453218239 | -5.519144817 | 5.75E-08 |
| RP11-524L6.3 | -0.623321969 | -0.341322354 | -5.518917871 | 5.76E-08 |
| FLYWCH2 | -0.26624084 | 4.779040146 | -5.518194205 | 5.78E-08 |
| MCM3AP | -0.195891704 | 6.272847716 | -5.516984278 | 5.82E-08 |
| RCE1 | 0.205323129 | 5.072508565 | 5.516909827 | 5.82E-08 |
| CSTPP1 | -0.372750339 | 3.55700613 | -5.516907713 | 5.82E-08 |
| AC055811.5 | -0.582387817 | -0.515511337 | -5.516871203 | 5.82E-08 |
| MIR5194 | 0.726421895 | 1.520103956 | 5.516693622 | 5.83E-08 |
| BLOC1S2 | 0.257410375 | 6.358814818 | 5.516146287 | 5.85E-08 |
| KANK1 | -0.639831226 | 2.711729835 | -5.516105195 | 5.85E-08 |
| NREP | -0.602032123 | 5.853168744 | -5.515652068 | 5.86E-08 |
| OPA3 | -0.302510602 | 3.388976045 | -5.515587178 | 5.86E-08 |
| PFKFB2 | 0.829478044 | 2.978866115 | 5.515194961 | 5.88E-08 |
| RP11-320N21.2 | -0.717427449 | -1.049789143 | -5.51431149 | 5.90E-08 |
| RP11-38P22.2 | 0.599883879 | 4.236819503 | 5.513863346 | 5.92E-08 |
| PMM2 | -0.305949159 | 3.18636426 | -5.513730965 | 5.92E-08 |
| SNRNP27 | 0.182325985 | 6.107739984 | 5.513443872 | 5.93E-08 |
| BPHL | -0.386583796 | 1.976106287 | -5.513212144 | 5.94E-08 |
| DIDO1 | -0.230614084 | 5.682754481 | -5.512594175 | 5.96E-08 |
| P2RY8 | -0.474399884 | 7.530984785 | -5.512418544 | 5.96E-08 |
| AL672183.2 | -0.607780384 | -0.14065075 | -5.511981873 | 5.98E-08 |
| C5orf45 | -0.338603373 | 3.296582179 | -5.511964743 | 5.98E-08 |
| NARF | 0.270952138 | 5.274149251 | 5.511719751 | 5.99E-08 |
| PPP1R15B | 0.366165798 | 7.640992769 | 5.511331193 | 6.00E-08 |
| PDCL | 0.267778534 | 5.283132243 | 5.509949204 | 6.04E-08 |
| MRPL15 | -0.344594635 | 6.177373594 | -5.50880261 | 6.08E-08 |
| C6orf195 | -0.660900467 | -1.563458463 | -5.507005257 | 6.14E-08 |
| LEKR1 | -0.663472103 | -1.352146285 | -5.506808674 | 6.14E-08 |
| RP11-1275H24.1 | -0.805927763 | 1.160351724 | -5.504443575 | 6.22E-08 |
| MIR22HG | 0.902626973 | 5.893466317 | 5.502971376 | 6.27E-08 |
| RP11-603K19.1 | -0.629555127 | -0.630714242 | -5.501898546 | 6.31E-08 |
| C16orf58 | -0.311344802 | 6.611745442 | -5.501797634 | 6.31E-08 |
| ICMT | -0.436684199 | 5.439340296 | -5.501507924 | 6.32E-08 |
| SNORD125 | 0.461238818 | 1.613174184 | 5.500437789 | 6.36E-08 |
| RP11-399K21.10 | -0.719608316 | -0.440630945 | -5.500268306 | 6.36E-08 |
| TMEM222 | 0.17458698 | 4.949844517 | 5.499619426 | 6.38E-08 |
| SPNS1 | 0.445494146 | 1.262037207 | 5.499251052 | 6.40E-08 |
| RN7SKP127 | 0.616594379 | 0.728508211 | 5.499067173 | 6.40E-08 |
| FAM45B | -0.447853291 | 5.993495618 | -5.498559017 | 6.42E-08 |
| LGI4 | 0.716791762 | -1.121210704 | 5.49827488 | 6.43E-08 |
| AC007249.3 | -0.641211438 | -0.540830105 | -5.496795593 | 6.48E-08 |
| TP53INP2 | 0.847697891 | 5.790820801 | 5.496722343 | 6.48E-08 |
| HHIPL2 | -0.582758358 | -1.593409586 | -5.496648141 | 6.48E-08 |
| HMGB1P10 | 0.278807313 | 3.776992212 | 5.496584281 | 6.49E-08 |
| TMCC3 | 0.947473798 | 2.393525766 | 5.496089495 | 6.50E-08 |
| RP11-166B2.3 | 0.591052672 | 0.79656654 | 5.495632899 | 6.52E-08 |
| PEAR1 | 1.18905403 | 1.951973102 | 5.494087433 | 6.57E-08 |
| RP11-209M4.1 | 0.28073706 | 2.236873546 | 5.493284736 | 6.60E-08 |
| AC009951.1 | 0.896351105 | 1.084868709 | 5.492220659 | 6.64E-08 |
| C14orf79 | -0.62148223 | 1.433137124 | -5.491001596 | 6.68E-08 |
| MIR223 | -0.74396293 | 5.597031513 | -5.490843961 | 6.69E-08 |
| POLR3GL | -0.214143984 | 6.283346983 | -5.490763873 | 6.69E-08 |
| CTD-2514C3.1 | -0.822614325 | 0.246521681 | -5.490590321 | 6.70E-08 |
| ZMYND11 | -0.306159677 | 6.087122309 | -5.489610267 | 6.73E-08 |
| TPBGL | 0.713047146 | -2.243323457 | 5.489390246 | 6.74E-08 |
| RP11-390K5.6 | 0.390959913 | 3.938210186 | 5.487659819 | 6.80E-08 |
| ZBP1 | 0.829369447 | 2.307603168 | 5.48750082 | 6.81E-08 |
| FAM120B | -0.203041566 | 5.18727596 | -5.486776374 | 6.83E-08 |
| WHAMMP3 | 0.801809412 | 2.430190626 | 5.486626121 | 6.84E-08 |
| RP11-250B2.5 | -0.739114293 | -0.339379756 | -5.485896836 | 6.87E-08 |
| C22orf46 | -0.243467341 | 3.3454629 | -5.485495884 | 6.88E-08 |
| AC007272.3 | -0.662903305 | -0.083221951 | -5.484865357 | 6.90E-08 |
| ATP5F1P5 | -0.696794315 | 0.341541861 | -5.484568142 | 6.91E-08 |
| MTHFR | 0.265871107 | 4.664361927 | 5.483794835 | 6.94E-08 |
| ZNF737 | -0.637885557 | 3.475243336 | -5.4836164 | 6.95E-08 |
| MAP2K3 | 0.359581959 | 6.990535789 | 5.483511511 | 6.95E-08 |
| RP3-414A15.2 | -0.625731317 | 0.634449152 | -5.482784311 | 6.98E-08 |
| PAIP1P1 | 0.585117467 | 1.669964572 | 5.481217347 | 7.04E-08 |
| XRCC5 | -0.212286067 | 8.220391142 | -5.480943856 | 7.05E-08 |
| TRAV29DV5 | -0.782361426 | -0.299749914 | -5.480892685 | 7.05E-08 |
| CASP4 | -0.279835156 | 6.233072438 | -5.479967994 | 7.08E-08 |
| LINC00694 | -0.613830103 | 3.966640083 | -5.479520919 | 7.10E-08 |
| RP11-480I12.4 | -0.776635368 | -2.346431023 | -5.478601663 | 7.14E-08 |
| RP11-1275H24.2 | -0.695841505 | 1.462112722 | -5.477453909 | 7.18E-08 |
| DDIT4 | 0.888764256 | 7.76183614 | 5.477128548 | 7.19E-08 |
| ASH1L-AS1 | 0.426826319 | 3.233186987 | 5.475672097 | 7.25E-08 |
| ZNF280C | 0.24184871 | 3.227966709 | 5.475470077 | 7.26E-08 |
| RP11-410L14.2 | -0.402911273 | 2.922691197 | -5.475192801 | 7.27E-08 |
| SLFNL1 | -0.695638026 | -1.528937036 | -5.474421861 | 7.30E-08 |
| CTC-479C5.10 | 0.421613677 | 1.560847229 | 5.474388033 | 7.30E-08 |
| SLC12A9 | -0.460416495 | 4.824719814 | -5.474235476 | 7.30E-08 |
| RPS17L | 0.356030263 | 4.282771038 | 5.473774831 | 7.32E-08 |
| LYST | -0.602414476 | 6.732437867 | -5.473085454 | 7.35E-08 |
| SNHG17 | 0.364663324 | 5.047012316 | 5.473008931 | 7.35E-08 |
| RP11-566K11.7 | 0.584156684 | 1.200016244 | 5.472751125 | 7.36E-08 |
| RP11-535K18.2 | 0.471343166 | 3.011574461 | 5.472518179 | 7.37E-08 |
| MAPRE1 | 0.231891408 | 8.781350968 | 5.472376174 | 7.38E-08 |
| MORF4L1 | 0.234384545 | 5.981414084 | 5.4717948 | 7.40E-08 |
| TMEM39A | 0.277362838 | 5.046269241 | 5.471620707 | 7.41E-08 |
| SMN2 | 0.49919068 | 3.571717917 | 5.471398459 | 7.41E-08 |
| MTAP | -0.382370875 | 3.582804273 | -5.46928051 | 7.50E-08 |
| C14orf2 | 0.195201535 | 6.38436252 | 5.468933956 | 7.51E-08 |
| CTA-14H9.5 | -0.95446053 | 2.067228464 | -5.468917019 | 7.51E-08 |
| RP11-630C16.2 | -0.735258286 | -0.860312539 | -5.467796923 | 7.56E-08 |
| RP11-415F23.4 | -0.581160929 | 1.653069026 | -5.467580819 | 7.57E-08 |
| U62631.5 | -0.654544376 | -0.714100433 | -5.467124702 | 7.58E-08 |
| RP3-514P16.1 | -0.299762759 | 3.014971691 | -5.466403129 | 7.61E-08 |
| UBE2K | -0.162793171 | 6.438421218 | -5.465678224 | 7.64E-08 |
| RP11-290L1.4 | -0.35382845 | 2.695433628 | -5.46526941 | 7.66E-08 |
| EMC3 | 0.218527602 | 5.592208905 | 5.464823731 | 7.68E-08 |
| PSMB1 | 0.182116611 | 7.516035002 | 5.463935597 | 7.71E-08 |
| NKPD1 | 0.611522852 | -0.970204686 | 5.463150042 | 7.74E-08 |
| FAM86B1 | -0.72955923 | -0.889136621 | -5.462726802 | 7.76E-08 |
| GSPT1 | -0.263707876 | 6.48065644 | -5.462388701 | 7.78E-08 |
| FAM73A | 0.165110973 | 3.4994653 | 5.461592417 | 7.81E-08 |
| RP11-37N22.1 | -0.798411089 | -1.022750868 | -5.460930042 | 7.84E-08 |
| C10orf2 | -0.487014804 | 4.075276017 | -5.460749007 | 7.84E-08 |
| ARHGAP31-AS1 | -0.635778238 | 1.295365878 | -5.458887117 | 7.92E-08 |
| RSG1 | -0.376946874 | 3.009288189 | -5.458065857 | 7.96E-08 |
| RP11-245P10.4 | -0.467914246 | 1.180739567 | -5.457428222 | 7.98E-08 |
| TAPBPL | -0.313655874 | 5.572837269 | -5.457328821 | 7.99E-08 |
| FABP4 | 1.21048144 | -0.973231069 | 5.457069559 | 8.00E-08 |
| METTL23 | -0.208673589 | 4.668823068 | -5.455151921 | 8.08E-08 |
| APOBEC3F | -0.348432512 | 3.761164348 | -5.454865057 | 8.09E-08 |
| TRAV21 | -0.808907385 | 0.038471851 | -5.454617713 | 8.10E-08 |
| RP11-266L9.5 | -0.704567253 | 2.642268371 | -5.454508421 | 8.11E-08 |
| MPZL1 | -0.560276545 | 4.890313822 | -5.453628863 | 8.14E-08 |
| PDLIM4 | 1.019399126 | -2.651156287 | 5.452869853 | 8.18E-08 |
| FDFT1 | -0.267197396 | 6.71880537 | -5.45286946 | 8.18E-08 |
| RALA | -0.389279696 | 5.970211634 | -5.452239745 | 8.20E-08 |
| RP11-1212A22.1 | 0.335771604 | 2.593689763 | 5.451616449 | 8.23E-08 |
| KB-1410C5.5 | -0.627924258 | 1.062917613 | -5.451575394 | 8.23E-08 |
| MOV10L1 | 0.716609592 | -3.681284608 | 5.45109612 | 8.25E-08 |
| SKAP2 | -0.717187846 | 6.270155519 | -5.451073144 | 8.25E-08 |
| RP11-403I13.4 | -0.721257358 | -1.028399089 | -5.450807496 | 8.27E-08 |
| RP13-270P17.3 | -0.339923094 | 3.853205905 | -5.449915778 | 8.30E-08 |
| TSPAN6 | 0.897427675 | -1.30596416 | 5.449822182 | 8.31E-08 |
| CTD-3203P2.2 | -0.611438496 | -1.107574461 | -5.449519516 | 8.32E-08 |
| MFSD2A | 0.794537863 | 2.758631885 | 5.449429659 | 8.33E-08 |
| ZNF366 | -1.08573172 | -1.068394024 | -5.449322323 | 8.33E-08 |
| RP11-156N15.1 | 0.333514568 | 3.38657268 | 5.448697018 | 8.36E-08 |
| SLC35F5 | -0.192230723 | 4.296282634 | -5.44859159 | 8.36E-08 |
| SCARNA9 | -0.506450261 | 6.313677776 | -5.448199561 | 8.38E-08 |
| BABAM1 | 0.209206061 | 4.278478306 | 5.447465692 | 8.41E-08 |
| CNKSR2 | 0.922332092 | 0.540832435 | 5.446430963 | 8.46E-08 |
| MLKL | -0.397206216 | 5.182471698 | -5.446418026 | 8.46E-08 |
| SLC16A10 | 0.805813312 | -1.236760271 | 5.445490613 | 8.50E-08 |
| FMR1 | 0.251617398 | 5.587948748 | 5.445120731 | 8.52E-08 |
| CDKN2AIP | 0.258625194 | 5.74595227 | 5.444736473 | 8.53E-08 |
| HDHD1 | -0.512918234 | 3.281653981 | -5.44441446 | 8.55E-08 |
| SORD | -0.425140443 | 4.06430352 | -5.443556816 | 8.59E-08 |
| RP11-363L24.2 | 0.485685649 | 0.589252183 | 5.442968248 | 8.61E-08 |
| FAM3C2 | -0.399408218 | 5.777508307 | -5.442489571 | 8.64E-08 |
| SEC23B | -0.330167334 | 6.198553592 | -5.441136547 | 8.70E-08 |
| AOC3 | 0.720832325 | 0.368622043 | 5.440796195 | 8.71E-08 |
| CTD-2006C1.2 | 0.417770394 | 3.386946823 | 5.440729344 | 8.72E-08 |
| MAP4 | -0.289376068 | 6.074866892 | -5.440654555 | 8.72E-08 |
| RP11-365O10.1 | -0.454874477 | 1.642403294 | -5.440520144 | 8.73E-08 |
| CTD-2192J16.11 | -0.593072163 | 0.239946762 | -5.43984371 | 8.76E-08 |
| FAM86EP | -0.578685553 | 0.401034938 | -5.438471807 | 8.82E-08 |
| CTC-366B18.4 | -0.48378741 | 1.754924133 | -5.437353057 | 8.87E-08 |
| ANKMY1 | -0.258248997 | 3.755583017 | -5.437004671 | 8.89E-08 |
| RP11-330O11.3 | -0.63189506 | 0.748389374 | -5.436743226 | 8.90E-08 |
| GPR135 | -0.544413293 | 1.663775874 | -5.436416299 | 8.92E-08 |
| RTN4RL1 | -0.91661063 | -1.613245726 | -5.436380879 | 8.92E-08 |
| LTC4S | -0.666392182 | -0.245429417 | -5.436317615 | 8.92E-08 |
| NAE1 | 0.23437048 | 5.388328309 | 5.436075358 | 8.93E-08 |
| CCDC170 | -1.153610219 | 0.00495632 | -5.435922134 | 8.94E-08 |
| XCL1 | 0.858600178 | 0.658665136 | 5.435819515 | 8.95E-08 |
| HPS5 | -0.256637055 | 4.14915296 | -5.434742027 | 9.00E-08 |
| CDC27 | 0.230741031 | 5.488481091 | 5.433981239 | 9.03E-08 |
| FTH1P11 | 0.637877906 | 6.515200583 | 5.4336448 | 9.05E-08 |
| GAD1 | 1.187541029 | -2.369063007 | 5.433052456 | 9.08E-08 |
| PLCG2 | -0.290465758 | 5.494568899 | -5.432875947 | 9.08E-08 |
| RP11-434H6.7 | -0.572567239 | 0.331547967 | -5.432752764 | 9.09E-08 |
| HSD17B10 | -0.224365586 | 6.816097057 | -5.432494343 | 9.10E-08 |
| POLR2C | 0.157915827 | 5.878566875 | 5.431597667 | 9.15E-08 |
| SLC17A5 | 0.401019475 | 3.956791852 | 5.431566881 | 9.15E-08 |
| TSEN15P1 | -0.576895709 | 0.011788185 | -5.431234711 | 9.16E-08 |
| RP11-139H15.1 | -0.315492125 | 4.306716216 | -5.430729047 | 9.19E-08 |
| SPC25 | 0.821696687 | 2.925871169 | 5.430175845 | 9.21E-08 |
| FAM149B1P1 | -0.504483486 | -0.245207916 | -5.430062534 | 9.22E-08 |
| CTD-3131K8.2 | -0.47686082 | 1.896686532 | -5.429497231 | 9.25E-08 |
| AC093616.4 | 0.309144802 | 4.109243684 | 5.429328856 | 9.26E-08 |
| CCBL1 | -0.338924458 | 2.556927894 | -5.429070892 | 9.27E-08 |
| PRDM4 | 0.214385411 | 5.656932367 | 5.428948572 | 9.27E-08 |
| SEMA7A | 0.707829708 | 3.751643063 | 5.42865763 | 9.29E-08 |
| RP11-589F5.4 | -0.559081996 | 2.334337871 | -5.428481822 | 9.30E-08 |
| WNT1 | 0.755241653 | -0.94950888 | 5.428188064 | 9.31E-08 |
| SFT2D3 | -0.260712248 | 4.269621425 | -5.428004368 | 9.32E-08 |
| AC092620.3 | 0.740303216 | 1.463636944 | 5.427976094 | 9.32E-08 |
| UNC79 | -0.772892789 | -2.061194516 | -5.426729052 | 9.38E-08 |
| TCTA | -0.308535449 | 6.062632931 | -5.426641007 | 9.39E-08 |
| 5-Mar | 0.210095555 | 5.689145063 | 5.426439712 | 9.40E-08 |
| RP11-894P9.1 | 0.583604001 | -0.604753262 | 5.424669086 | 9.49E-08 |
| C6orf120 | -0.216384027 | 5.053034785 | -5.424119865 | 9.51E-08 |
| IGF2BP3 | 0.842885896 | 1.661081813 | 5.423950804 | 9.52E-08 |
| NXT1 | 0.317699427 | 6.505391191 | 5.422698672 | 9.58E-08 |
| TOPORS-AS1 | 0.287218997 | 3.534957277 | 5.422676566 | 9.59E-08 |
| H1FX | 0.391329543 | 10.92596828 | 5.422589076 | 9.59E-08 |
| VRK2 | 0.149931554 | 5.439628456 | 5.421989568 | 9.62E-08 |
| CDK13 | -0.169795119 | 5.717190111 | -5.421474902 | 9.65E-08 |
| AKAP3 | -0.656596302 | -1.255346861 | -5.420349426 | 9.70E-08 |
| TNFSF10 | -0.757593444 | 5.032838855 | -5.419333192 | 9.76E-08 |
| PSMA2 | 0.368145907 | 2.431934891 | 5.419029675 | 9.77E-08 |
| APBB3 | -0.320382878 | 4.734919652 | -5.418885491 | 9.78E-08 |
| RBM45 | -0.210456321 | 3.223115256 | -5.418850038 | 9.78E-08 |
| GAL3ST1 | -0.660225818 | -2.352385987 | -5.418437794 | 9.80E-08 |
| SUGT1 | -0.201095972 | 4.618754002 | -5.418281665 | 9.81E-08 |
| RNFT1P3 | -0.617443224 | -0.768067804 | -5.41813475 | 9.82E-08 |
| RP11-756P10.3 | 0.20849923 | 4.674357533 | 5.417693427 | 9.84E-08 |
| RP11-304L19.11 | -0.641235296 | -0.855095679 | -5.417063611 | 9.87E-08 |
| RP11-305K5.1 | 0.553070623 | 2.393045683 | 5.414679161 | 1.00E-07 |
| RP1-199J3.7 | -0.627988756 | -0.514230288 | -5.413976958 | 1.00E-07 |
| MYH7B | -0.541149467 | -1.054854676 | -5.412394284 | 1.01E-07 |
| RNU6-1010P | 0.733437783 | 0.170867829 | 5.412279819 | 1.01E-07 |
| IL6 | 1.243055518 | -0.404955298 | 5.412129498 | 1.01E-07 |
| DSN1 | 0.288277912 | 4.878254394 | 5.411340305 | 1.02E-07 |
| RP11-728K20.1 | -0.643471729 | 0.056325848 | -5.410082644 | 1.02E-07 |
| TOMM5 | 0.288114399 | 3.374600211 | 5.407732895 | 1.04E-07 |
| IP6K2 | 0.331340333 | 5.917853033 | 5.407454557 | 1.04E-07 |
| LYSMD3 | 0.269611781 | 5.168000149 | 5.406675684 | 1.04E-07 |
| AC068134.10 | -0.509814887 | 0.722217546 | -5.405550813 | 1.05E-07 |
| AC104809.3 | -1.104321709 | -2.253552885 | -5.405362808 | 1.05E-07 |
| LIMD1 | -0.291291696 | 4.889077108 | -5.405159443 | 1.05E-07 |
| PRTFDC1 | 1.05325649 | 0.682702527 | 5.403928974 | 1.06E-07 |
| RP11-407N17.6 | 0.384172041 | 1.493743334 | 5.403614024 | 1.06E-07 |
| PNO1 | 0.221808305 | 4.656238863 | 5.402397542 | 1.07E-07 |
| LRRC8B | 0.416878474 | 3.850297511 | 5.401410508 | 1.07E-07 |
| CNPY4 | -0.314888123 | 4.137392309 | -5.401212816 | 1.07E-07 |
| INPP5D | -0.251258267 | 7.809696437 | -5.400596373 | 1.08E-07 |
| ASPH | -0.440374587 | 5.073915497 | -5.399446876 | 1.08E-07 |
| PSMD5 | 0.23947708 | 5.187332127 | 5.399331312 | 1.08E-07 |
| PPCDC | 0.325127448 | 4.129911397 | 5.398963639 | 1.09E-07 |
| KLHL8 | -0.351438299 | 4.014505448 | -5.398404543 | 1.09E-07 |
| CENPJ | -0.450237666 | 5.332923089 | -5.396983443 | 1.10E-07 |
| RP11-727F15.11 | -0.575245716 | 0.243297791 | -5.396636982 | 1.10E-07 |
| AC104653.1 | -0.572846879 | 0.385788227 | -5.396597361 | 1.10E-07 |
| MIR3192 | 0.563446012 | 0.809420037 | 5.396193717 | 1.10E-07 |
| ACOT7 | 0.443134881 | 3.755657101 | 5.394754352 | 1.11E-07 |
| HMGA1P3 | 0.427285253 | 1.891428521 | 5.394701017 | 1.11E-07 |
| GNS | -0.516157682 | 6.918452856 | -5.394586909 | 1.11E-07 |
| TINAGL1 | 1.162381914 | -2.130892542 | 5.394272272 | 1.11E-07 |
| HIST1H4E | -0.853425626 | 3.394058075 | -5.393907042 | 1.11E-07 |
| RP11-426C22.5 | -0.547611206 | 0.69917229 | -5.393012756 | 1.12E-07 |
| FAIM | -0.489198255 | 2.966181864 | -5.391927816 | 1.13E-07 |
| MRPL3P1 | -0.268522022 | 4.316323025 | -5.391675225 | 1.13E-07 |
| NUDT11 | 1.029847897 | -0.793810562 | 5.389371506 | 1.14E-07 |
| AC025335.1 | -0.378115741 | 2.886890244 | -5.389041974 | 1.14E-07 |
| ZNF774 | -0.658369781 | -1.330359523 | -5.388869001 | 1.14E-07 |
| KB-1562D12.3 | 0.404447974 | 2.101819349 | 5.388615602 | 1.15E-07 |
| PAPPA | 1.050572483 | -5.36863152 | 5.387421008 | 1.15E-07 |
| PSTK | 0.423342944 | 1.909207145 | 5.387287202 | 1.15E-07 |
| SNORD112 | 0.623231781 | 0.354613532 | 5.386734142 | 1.16E-07 |
| TCHP | -0.220751291 | 4.412550908 | -5.385024896 | 1.17E-07 |
| LINC00471 | -0.630426064 | 0.7555807 | -5.384930834 | 1.17E-07 |
| RP11-467D6.1 | -0.425058635 | 1.530160429 | -5.383442138 | 1.18E-07 |
| NUTM2HP | 0.637773916 | -1.054263234 | 5.3829823 | 1.18E-07 |
| MTMR3 | 0.326016211 | 6.212243622 | 5.382899962 | 1.18E-07 |
| BCKDHB | -0.33843397 | 4.116738238 | -5.381039434 | 1.19E-07 |
| PAQR3 | 0.318938198 | 3.388776362 | 5.380132424 | 1.20E-07 |
| GNA12 | 0.450390938 | 6.050971906 | 5.37996976 | 1.20E-07 |
| RP11-195C7.1 | -0.519157276 | 0.404190695 | -5.379570212 | 1.20E-07 |
| BZRAP1 | 0.723415049 | 5.197693148 | 5.377770391 | 1.21E-07 |
| ZSCAN22 | -0.389693476 | 1.714058959 | -5.377055071 | 1.22E-07 |
| CHMP4BP1 | 0.34315085 | 4.864184499 | 5.376274246 | 1.22E-07 |
| CYTIP | 0.612359872 | 6.806162837 | 5.375661348 | 1.23E-07 |
| PSMD4 | -0.160122094 | 6.73244393 | -5.374969849 | 1.23E-07 |
| RP11-383B4.4 | -0.541231397 | 0.316925621 | -5.372766405 | 1.24E-07 |
| MBTD1 | -0.238026909 | 5.561483886 | -5.371810454 | 1.25E-07 |
| RP11-252I13.1 | 0.365334297 | 1.175259785 | 5.371758572 | 1.25E-07 |
| ETF1P3 | 0.522712147 | 0.040800359 | 5.371334139 | 1.25E-07 |
| HMGB3P6 | 0.547755086 | 4.125692627 | 5.370696458 | 1.26E-07 |
| RP11-81K2.1 | -0.572981541 | 0.712614139 | -5.370197183 | 1.26E-07 |
| IMP3 | -0.30826324 | 5.753242325 | -5.369762549 | 1.26E-07 |
| TFPT | -0.198644966 | 5.7147623 | -5.369607537 | 1.27E-07 |
| DOC2A | -0.912141788 | 0.873668456 | -5.369565804 | 1.27E-07 |
| RP11-181E10.3 | -0.728595758 | -0.393980833 | -5.369326874 | 1.27E-07 |
| C9orf85 | -0.240377094 | 3.243901049 | -5.368621448 | 1.27E-07 |
| RP11-61L23.2 | 0.70830182 | -1.846391134 | 5.368449296 | 1.27E-07 |
| RP11-214K3.22 | -0.678520819 | 0.121961568 | -5.367735098 | 1.28E-07 |
| SNRPG | 0.218757738 | 4.485781153 | 5.367721196 | 1.28E-07 |
| TCEB3 | 0.239758932 | 5.851922988 | 5.367057158 | 1.28E-07 |
| COPA | -0.18845752 | 7.213649872 | -5.366790605 | 1.28E-07 |
| CTC-236F12.4 | -0.704659602 | -0.872574254 | -5.366499256 | 1.29E-07 |
| ZFAND6 | 0.237096863 | 5.97784658 | 5.365912668 | 1.29E-07 |
| TCEA1 | 0.207424545 | 5.737921204 | 5.365308571 | 1.29E-07 |
| RP11-319G6.1 | -0.456575327 | 1.303333518 | -5.364985471 | 1.30E-07 |
| LSS | -0.357086116 | 4.650033396 | -5.36494007 | 1.30E-07 |
| SRXN1 | 0.579990508 | 0.459879586 | 5.364263554 | 1.30E-07 |
| CCDC59 | 0.351465451 | 5.974373067 | 5.364098444 | 1.30E-07 |
| RP11-64C12.6 | -0.87171863 | -1.42910338 | -5.363809359 | 1.30E-07 |
| FAM192A | -0.152755308 | 6.675839346 | -5.3636373 | 1.31E-07 |
| ZNF529 | 0.418475607 | 3.580542869 | 5.363081752 | 1.31E-07 |
| ANKHD1 | 0.431485448 | 1.140524839 | 5.362904712 | 1.31E-07 |
| RP11-150L8.4 | 0.308763911 | 1.60372379 | 5.362135815 | 1.32E-07 |
| TXLNA | -0.220502598 | 6.284327001 | -5.362068422 | 1.32E-07 |
| ANP32B | 0.240335248 | 9.618701584 | 5.360477073 | 1.33E-07 |
| GPR82 | -0.94145057 | -1.705146475 | -5.360265272 | 1.33E-07 |
| CTC-378H22.1 | -0.784785317 | 2.071194341 | -5.359800526 | 1.33E-07 |
| FMR1-IT1 | 0.514825946 | 1.995830061 | 5.359284247 | 1.34E-07 |
| IQSEC2 | -0.674001786 | 2.900225593 | -5.359154812 | 1.34E-07 |
| LAMA4 | 1.159826575 | -5.026223774 | 5.358722975 | 1.34E-07 |
| YES1 | 0.822098749 | 3.65777234 | 5.35838227 | 1.34E-07 |
| TPD52L2 | 0.254836854 | 7.056941358 | 5.357908246 | 1.34E-07 |
| CDK2 | 0.307927724 | 5.676019401 | 5.357442311 | 1.35E-07 |
| NHS | -0.959033682 | -0.801949329 | -5.357433875 | 1.35E-07 |
| DERL1 | 0.185709377 | 6.004721075 | 5.357429374 | 1.35E-07 |
| CDC42BPG | 0.468592186 | 2.203060251 | 5.35706168 | 1.35E-07 |
| CACYBPP2 | -0.278908721 | 6.397160166 | -5.356711501 | 1.35E-07 |
| MRPL41 | -0.378969189 | 6.829680248 | -5.356650296 | 1.35E-07 |
| RP5-991G20.2 | -0.651385257 | -0.395049514 | -5.356574826 | 1.35E-07 |
| STAB1 | -1.091012105 | 6.269524307 | -5.356449358 | 1.36E-07 |
| COMMD8 | -0.341580268 | 5.715129732 | -5.356227696 | 1.36E-07 |
| UBE2D3 | 0.184685861 | 7.691436351 | 5.356208477 | 1.36E-07 |
| STX10 | -0.237145408 | 7.250231725 | -5.355327829 | 1.36E-07 |
| CTLA4 | 0.880782615 | 1.385448016 | 5.355152149 | 1.36E-07 |
| CCDC7 | -0.335281701 | 1.539450222 | -5.353545023 | 1.38E-07 |
| RP11-260M2.1 | -0.446633404 | 2.151098478 | -5.353125606 | 1.38E-07 |
| CRHR1-IT1 | -0.480927835 | 1.726707548 | -5.352081786 | 1.39E-07 |
| MID1IP1-AS1 | -0.579376591 | 0.279779681 | -5.351440004 | 1.39E-07 |
| KMT2C | 0.248525027 | 6.712805924 | 5.351388964 | 1.39E-07 |
| RP5-1021I20.5 | -0.582703051 | -2.531514587 | -5.35079421 | 1.40E-07 |
| WASH4P | 0.254035617 | 1.939326341 | 5.350640178 | 1.40E-07 |
| CRK | -0.283341436 | 5.109715289 | -5.350581338 | 1.40E-07 |
| CTC-312O10.2 | -0.666380612 | -0.461416428 | -5.350191134 | 1.40E-07 |
| MIR590 | 0.503556064 | 3.810850003 | 5.350061314 | 1.40E-07 |
| NFE2L1 | -0.252360752 | 7.025342053 | -5.349541505 | 1.40E-07 |
| TTC4P1 | -0.554534861 | -0.321771145 | -5.349168112 | 1.41E-07 |
| TGFBR3 | 0.811034882 | 1.753235357 | 5.348436848 | 1.41E-07 |
| FBLIM1 | 0.810308114 | -3.327930537 | 5.348113025 | 1.42E-07 |
| RP4-607J23.2 | 0.585146039 | 3.234670346 | 5.346797855 | 1.42E-07 |
| VN1R84P | -0.80670178 | -0.688604677 | -5.346023668 | 1.43E-07 |
| MR1 | -0.275607265 | 4.892209889 | -5.345955809 | 1.43E-07 |
| ARMC1 | -0.210285881 | 5.322936367 | -5.345920203 | 1.43E-07 |
| FTH1P10 | 0.525988716 | 7.066318557 | 5.344712408 | 1.44E-07 |
| KIRREL3 | -0.953251875 | -3.433746328 | -5.344442265 | 1.44E-07 |
| ZSCAN23 | -1.035155532 | -1.247628333 | -5.344187561 | 1.44E-07 |
| SBDS | 0.489128905 | 8.135787687 | 5.3440544 | 1.45E-07 |
| MRPS31P5 | 0.564764487 | 1.856284278 | 5.343904791 | 1.45E-07 |
| PDE1B | 0.526505553 | 3.490323226 | 5.343788468 | 1.45E-07 |
| AC016747.3 | -0.666006923 | 2.143756198 | -5.343185148 | 1.45E-07 |
| KLHL11 | 0.541036318 | 4.792985457 | 5.342723675 | 1.46E-07 |
| AP001412.1 | -0.61037311 | 0.40071765 | -5.342411503 | 1.46E-07 |
| TREML3P | 0.917156043 | 1.891956012 | 5.342010283 | 1.46E-07 |
| TRPM7 | -0.144955901 | 6.142282557 | -5.341596614 | 1.46E-07 |
| MIR5690 | 0.791016993 | 1.788976161 | 5.340362878 | 1.47E-07 |
| ICT1 | -0.332556761 | 5.621625932 | -5.340313038 | 1.47E-07 |
| RP11-84D1.1 | 0.786441576 | 0.876368625 | 5.339537606 | 1.48E-07 |
| RP11-380G5.2 | 0.536835367 | 3.458788074 | 5.338847705 | 1.48E-07 |
| SCARNA5 | 0.555556427 | 0.595665684 | 5.337780311 | 1.49E-07 |
| EGLN2 | 0.293864284 | 3.601278415 | 5.337361793 | 1.50E-07 |
| ADCY4 | 0.598162949 | 2.624856598 | 5.336359894 | 1.50E-07 |
| NCS1 | 1.14591573 | 0.325733692 | 5.336327574 | 1.50E-07 |
| NCKAP5L | -0.30029176 | 4.386992146 | -5.335484893 | 1.51E-07 |
| RP11-398K22.12 | -0.562362017 | 0.291993716 | -5.33470114 | 1.52E-07 |
| MON1B | -0.21425919 | 5.843298437 | -5.334019814 | 1.52E-07 |
| RP11-98D18.3 | -0.657698663 | -0.230864834 | -5.333998332 | 1.52E-07 |
| YDJC | -0.254165316 | 6.063654146 | -5.333858141 | 1.52E-07 |
| TLK1 | 0.169026521 | 5.790608451 | 5.333337078 | 1.53E-07 |
| KLF7 | 0.394135058 | 5.723621475 | 5.332217273 | 1.54E-07 |
| SSH1 | 0.238416076 | 5.639340245 | 5.331929225 | 1.54E-07 |
| ACCS | -0.521475202 | 5.094154265 | -5.331880061 | 1.54E-07 |
| LRRN3 | -0.868860755 | -1.907765277 | -5.331659201 | 1.54E-07 |
| RP11-894J14.5 | -0.469274622 | 0.571132578 | -5.331443542 | 1.54E-07 |
| MRPS9 | -0.239599904 | 5.41511691 | -5.331265489 | 1.54E-07 |
| MTCH2 | -0.212687177 | 6.2328927 | -5.331189614 | 1.54E-07 |
| COA5 | -0.216051757 | 5.318476579 | -5.330934479 | 1.55E-07 |
| BAZ2A | 0.210530551 | 7.343456964 | 5.33076479 | 1.55E-07 |
| CD244 | -0.55645949 | 6.447666343 | -5.330578774 | 1.55E-07 |
| STOM | 0.545590081 | 7.984489118 | 5.329729434 | 1.56E-07 |
| RP11-97O12.7 | -0.533167502 | 0.570270621 | -5.329335233 | 1.56E-07 |
| RP11-968O1.5 | -0.746525681 | 0.833660487 | -5.328890153 | 1.56E-07 |
| CTD-2231E14.8 | -1.049575432 | 1.467420731 | -5.328799189 | 1.56E-07 |
| AFG3L2P1 | 0.3563927 | 1.466420286 | 5.32870763 | 1.56E-07 |
| STPG1 | -0.482673551 | 1.389911296 | -5.328622637 | 1.57E-07 |
| FARP2 | 0.360971701 | 3.198449588 | 5.327362545 | 1.58E-07 |
| FASTK | -0.215585494 | 5.502525221 | -5.327215343 | 1.58E-07 |
| EMILIN2 | -0.587099097 | 6.906361459 | -5.327005824 | 1.58E-07 |
| RPS10 | 0.3176725 | 3.600176133 | 5.326886017 | 1.58E-07 |
| RP11-526I2.5 | -0.645378951 | 2.291637131 | -5.326052036 | 1.59E-07 |
| DECR2 | 0.342000615 | 3.393907608 | 5.325305661 | 1.59E-07 |
| GLI4 | -0.214433184 | 4.273625641 | -5.325124805 | 1.59E-07 |
| CXorf21 | -0.608702084 | 6.188931741 | -5.324509627 | 1.60E-07 |
| ILF3 | 0.249080666 | 7.822804961 | 5.324313378 | 1.60E-07 |
| AC091133.1 | -0.405461226 | 1.851008002 | -5.323763493 | 1.61E-07 |
| CASP1P2 | -0.672349288 | -1.291641822 | -5.32369032 | 1.61E-07 |
| ARGLU1 | -0.256743825 | 8.010016594 | -5.323638169 | 1.61E-07 |
| RP13-582O9.7 | -0.639627332 | 0.65133463 | -5.323275595 | 1.61E-07 |
| ZCCHC11 | 0.252070893 | 5.077497597 | 5.322609727 | 1.62E-07 |
| RP5-890E16.2 | -0.605658009 | -0.904431544 | -5.321931117 | 1.62E-07 |
| MXRA7 | 0.715940668 | 2.786039748 | 5.32117486 | 1.63E-07 |
| C12orf61 | 0.476728817 | 1.390413964 | 5.321027551 | 1.63E-07 |
| STAT5B | -0.254282844 | 7.233295737 | -5.321023289 | 1.63E-07 |
| ABI1 | 0.153418477 | 7.323646285 | 5.319018732 | 1.65E-07 |
| CCT6B | 0.531418326 | 1.836225903 | 5.317621253 | 1.66E-07 |
| FMOD | 0.985561901 | -2.064649961 | 5.316998938 | 1.66E-07 |
| ZMYND12 | -0.611113767 | 0.959797358 | -5.316894839 | 1.66E-07 |
| NDUFB10 | -0.21497312 | 7.581294934 | -5.316494683 | 1.67E-07 |
| RP11-315O6.1 | -0.514892576 | 1.107500901 | -5.315177229 | 1.68E-07 |
| FAM122C | 0.248804924 | 2.958681416 | 5.314750852 | 1.68E-07 |
| CSGALNACT2 | 0.386096389 | 7.649719541 | 5.314253699 | 1.69E-07 |
| ZNF641 | -0.370118129 | 4.739653671 | -5.314138991 | 1.69E-07 |
| TTL | -0.238969132 | 4.50751195 | -5.313111386 | 1.70E-07 |
| AC104634.3 | -0.75381274 | 1.175518629 | -5.312757923 | 1.70E-07 |
| COX7B | 0.202576411 | 6.52776094 | 5.311791119 | 1.71E-07 |
| SIGLECL1 | -0.746991037 | -1.728348876 | -5.311673024 | 1.71E-07 |
| AC114730.2 | 0.956471272 | 2.579258485 | 5.310766957 | 1.72E-07 |
| SLAMF6 | -0.739586928 | 3.93636261 | -5.310611706 | 1.72E-07 |
| ALG1 | -0.356124732 | 3.354720398 | -5.310093367 | 1.72E-07 |
| THADA | -0.225589619 | 4.013549077 | -5.310009479 | 1.72E-07 |
| SCARB1 | -0.466015475 | 4.681764438 | -5.309293645 | 1.73E-07 |
| NDST1 | -0.360374325 | 5.324877384 | -5.308973887 | 1.73E-07 |
| KB-1027C11.4 | -0.368146165 | 3.741863549 | -5.307658382 | 1.74E-07 |
| MNDA | -1.068060532 | 8.479549083 | -5.306870174 | 1.75E-07 |
| ING1 | 0.276534452 | 5.284956829 | 5.305820178 | 1.76E-07 |
| JDP2 | 0.58156333 | 5.159393305 | 5.304823984 | 1.77E-07 |
| PROSER1 | 0.30403183 | 6.120425207 | 5.304735994 | 1.77E-07 |
| EIF1 | 0.344489168 | 10.15840605 | 5.304409225 | 1.77E-07 |
| PSMA5 | -0.213141795 | 5.298484144 | -5.303295986 | 1.78E-07 |
| OSTCP2 | -0.487508941 | 0.099261664 | -5.302248738 | 1.79E-07 |
| RP3-417G15.1 | -0.240863117 | 10.45266753 | -5.301950005 | 1.80E-07 |
| NFYC | -0.189244281 | 5.373533131 | -5.300887252 | 1.81E-07 |
| AP003774.6 | -1.174411637 | 0.342267079 | -5.299613761 | 1.82E-07 |
| ZNF155 | 0.321154893 | 3.314728751 | 5.299465833 | 1.82E-07 |
| HID1 | 0.727725802 | -1.339793401 | 5.298777029 | 1.83E-07 |
| RP11-359K18.4 | 0.64316492 | 0.839935333 | 5.298344735 | 1.83E-07 |
| HIST2H3D | -0.819849083 | 1.489043227 | -5.298182984 | 1.83E-07 |
| CTC-428G20.6 | -0.548208777 | 1.187997905 | -5.298055761 | 1.83E-07 |
| CABP4 | 0.888793769 | 0.048753562 | 5.29801252 | 1.83E-07 |
| CTD-2554C21.1 | 0.27356114 | 4.917771855 | 5.29687112 | 1.84E-07 |
| WNT6 | -0.93321607 | -0.306428541 | -5.29657989 | 1.85E-07 |
| RP11-171I2.4 | -0.769982426 | 3.029559483 | -5.296042774 | 1.85E-07 |
| RP13-638C3.2 | 0.511572708 | 2.093787076 | 5.295640206 | 1.86E-07 |
| SMARCD2 | -0.198229511 | 7.252482218 | -5.295494677 | 1.86E-07 |
| RP11-271K11.5 | -0.861113227 | 0.758162695 | -5.295422688 | 1.86E-07 |
| DDX11L8 | -0.832222574 | 0.409742609 | -5.295390448 | 1.86E-07 |
| RP11-484D2.4 | 0.466771578 | 2.039256015 | 5.29530494 | 1.86E-07 |
| MUT | -0.190930704 | 4.678341142 | -5.295128362 | 1.86E-07 |
| SMG6 | 0.169819764 | 4.553161577 | 5.29512685 | 1.86E-07 |
| ZNF862 | -0.365901106 | 3.801499904 | -5.294943386 | 1.86E-07 |
| SMARCA5 | -0.216237086 | 6.624402336 | -5.29472332 | 1.87E-07 |
| FBXL14 | 0.290083649 | 5.094229482 | 5.294485116 | 1.87E-07 |
| UBQLN4 | -0.265810836 | 5.561675801 | -5.294314857 | 1.87E-07 |
| MND1 | 0.662408483 | 3.214592261 | 5.293898155 | 1.87E-07 |
| PRDX1 | -0.487020634 | 8.155052138 | -5.293589994 | 1.88E-07 |
| BHLHE40-AS1 | -0.53576631 | -0.212331982 | -5.293386565 | 1.88E-07 |
| KDM5A | 0.169497784 | 5.882606252 | 5.292991855 | 1.88E-07 |
| ST7 | 0.744020259 | 3.289165427 | 5.292656417 | 1.89E-07 |
| LCMT2 | -0.447984559 | 3.082984096 | -5.292514901 | 1.89E-07 |
| STRADA | 0.303076713 | 3.243723126 | 5.291449876 | 1.90E-07 |
| EOLA2-DT | -0.390086429 | 2.754718617 | -5.291000488 | 1.90E-07 |
| RP1-102E24.6 | 0.562982181 | 0.989663565 | 5.290462921 | 1.91E-07 |
| SLX4IP | -0.321431265 | 3.553510629 | -5.289175917 | 1.92E-07 |
| CLTA | -0.201677938 | 7.24404647 | -5.289058553 | 1.92E-07 |
| GLRX5 | 0.307869297 | 6.168430162 | 5.288886843 | 1.92E-07 |
| RP5-1157M23.2 | -0.407829287 | 1.487685586 | -5.288137723 | 1.93E-07 |
| RP11-65I12.1 | -1.01829113 | -0.316676221 | -5.287556714 | 1.94E-07 |
| DSCR3 | -0.181476353 | 5.52384324 | -5.284801454 | 1.96E-07 |
| RBX1 | 0.260133602 | 4.086031442 | 5.284561877 | 1.97E-07 |
| GINM1 | 0.231027408 | 6.217447038 | 5.284080605 | 1.97E-07 |
| RP13-401N8.1 | -0.555710592 | -0.199875899 | -5.283673571 | 1.97E-07 |
| NPM1P37 | 0.421650454 | 1.444884294 | 5.283386271 | 1.98E-07 |
| RPH3AL | -0.600458982 | 1.058534982 | -5.283350275 | 1.98E-07 |
| ZNF225 | -0.393723002 | 1.657053835 | -5.282524404 | 1.99E-07 |
| MBP | -0.303557757 | 5.589354742 | -5.282445292 | 1.99E-07 |
| PHF23 | -0.374026879 | 5.132606729 | -5.281581788 | 2.00E-07 |
| LIME1 | -0.436605775 | 1.667012913 | -5.27918977 | 2.02E-07 |
| PPAPDC2 | -0.463414384 | 3.353440401 | -5.27887469 | 2.02E-07 |
| BEX5 | 0.862266222 | 0.122256898 | 5.276544788 | 2.05E-07 |
| CASP5 | -1.01446394 | 0.735376901 | -5.275968861 | 2.05E-07 |
| C9orf16 | 0.171127096 | 6.520615819 | 5.275734922 | 2.06E-07 |
| PDE7B | -0.864160263 | 0.394439275 | -5.275444222 | 2.06E-07 |
| C2orf81 | -0.538892566 | 1.210795557 | -5.272125625 | 2.09E-07 |
| ACIN1 | -0.173498958 | 7.391065749 | -5.271798168 | 2.10E-07 |
| LGALS8-AS1 | -0.573154472 | -1.047502794 | -5.271148288 | 2.11E-07 |
| RP11-424I19.2 | -0.659558539 | -0.582416271 | -5.269836616 | 2.12E-07 |
| BZW1P2 | 0.232961465 | 8.475347004 | 5.269787584 | 2.12E-07 |
| PUS3 | -0.256789144 | 4.029532 | -5.268248845 | 2.14E-07 |
| KLF5 | 0.877467483 | 3.160982104 | 5.267440297 | 2.15E-07 |
| RP1-159A19.4 | -0.738003293 | 1.260737851 | -5.267024701 | 2.15E-07 |
| ITGAD | 0.656801629 | -0.005461751 | 5.266527317 | 2.16E-07 |
| FARS2 | -0.275146403 | 4.521973405 | -5.265780092 | 2.16E-07 |
| MAGOH | 0.162490654 | 7.101220028 | 5.265624729 | 2.17E-07 |
| MT-ND6 | -0.606321675 | 9.13050013 | -5.265211684 | 2.17E-07 |
| TMEM38B | 0.434713976 | 3.965423793 | 5.264214116 | 2.18E-07 |
| HDLBP | -0.226552529 | 7.175960122 | -5.263031128 | 2.20E-07 |
| P2RY1 | 0.596483265 | 3.91153165 | 5.261721285 | 2.21E-07 |
| WASH6P | 0.39470816 | 1.31079327 | 5.26151353 | 2.21E-07 |
| FAM13B | -0.212982313 | 5.919680254 | -5.260419201 | 2.22E-07 |
| RP11-178G16.5 | -0.536139081 | 0.113623695 | -5.260161363 | 2.23E-07 |
| RAD23BP1 | 0.257496824 | 2.789421193 | 5.259917171 | 2.23E-07 |
| PXMP2 | 0.407283574 | 3.337923335 | 5.257356397 | 2.26E-07 |
| BMS1P4 | 0.350315376 | 1.867363588 | 5.256955165 | 2.26E-07 |
| RP11-214K3.21 | -0.66698928 | -0.23623807 | -5.256198299 | 2.27E-07 |
| MTFP1 | 0.483537633 | 1.775108666 | 5.254813783 | 2.29E-07 |
| AL021068.1 | -0.570903277 | 0.680549893 | -5.254341172 | 2.29E-07 |
| INTS9 | -0.221546686 | 4.071356528 | -5.253929593 | 2.30E-07 |
| RNA5SP33 | 0.655126302 | 0.731594637 | 5.253802829 | 2.30E-07 |
| RP11-342K6.1 | -0.371355447 | 3.490026658 | -5.253758988 | 2.30E-07 |
| RP11-173A16.2 | 0.567591324 | -0.059403321 | 5.252914776 | 2.31E-07 |
| SATB2 | -0.966759331 | -0.660542631 | -5.252909059 | 2.31E-07 |
| PLEKHH2 | 0.835073598 | -3.104206146 | 5.252481924 | 2.32E-07 |
| CASP2 | 0.33769964 | 6.510583458 | 5.251632299 | 2.33E-07 |
| PCDH12 | -1.017479354 | -1.061468224 | -5.251237971 | 2.33E-07 |
| RP11-588K22.2 | -1.126344413 | 4.038356419 | -5.250122982 | 2.34E-07 |
| AC005339.2 | -0.451397308 | 0.540291584 | -5.249015 | 2.36E-07 |
| TRIP4 | -0.163147971 | 5.221874937 | -5.248044742 | 2.37E-07 |
| VDAC2 | 0.234127788 | 5.881184119 | 5.248027419 | 2.37E-07 |
| RP11-1379J22.2 | 0.647564188 | 0.667942258 | 5.247643739 | 2.37E-07 |
| RP11-33B1.2 | -0.518943638 | 2.083297941 | -5.247638088 | 2.37E-07 |
| CRYZL1 | -0.210867015 | 4.036655889 | -5.246896068 | 2.38E-07 |
| STRIP1 | 0.157091232 | 5.134199563 | 5.24676195 | 2.39E-07 |
| PPP6R2 | -0.151964875 | 6.150408827 | -5.246726365 | 2.39E-07 |
| RP11-1277A3.2 | -0.510781794 | 4.192239009 | -5.246355161 | 2.39E-07 |
| PYCRL | -0.479111091 | 2.346121671 | -5.245616471 | 2.40E-07 |
| SMCR8 | 0.308686442 | 6.372550978 | 5.245459784 | 2.40E-07 |
| SPIN2B | -0.328595798 | 3.547855465 | -5.244371472 | 2.41E-07 |
| TSC2 | -0.151065387 | 5.583649041 | -5.244236866 | 2.42E-07 |
| RP4-641G12.4 | 0.382974105 | 1.163485972 | 5.244002217 | 2.42E-07 |
| TSTD2 | 0.226517923 | 4.540488446 | 5.24304457 | 2.43E-07 |
| ATXN7L3 | 0.15108401 | 6.650391643 | 5.242542394 | 2.44E-07 |
| RP11-152N13.5 | -0.395767632 | 2.492649287 | -5.242268814 | 2.44E-07 |
| SLC35E1 | 0.232931421 | 5.251568593 | 5.241560546 | 2.45E-07 |
| RP11-140I16.3 | -0.608208857 | -0.256282526 | -5.23953314 | 2.48E-07 |
| HAPLN3 | 0.647977958 | 2.588302688 | 5.239068242 | 2.48E-07 |
| MYO18A | -0.360527634 | 4.172729368 | -5.238263707 | 2.49E-07 |
| CTC-529P8.1 | -0.985157565 | -2.565001092 | -5.238014701 | 2.49E-07 |
| RP11-863P13.4 | -0.822180853 | -2.51446758 | -5.236944095 | 2.51E-07 |
| ZNF738 | 0.381111852 | 4.224806658 | 5.236907242 | 2.51E-07 |
| CD99P1 | 0.434467251 | 4.020266623 | 5.236788823 | 2.51E-07 |
| RP11-266L9.6 | -0.672028642 | -0.052022159 | -5.236463256 | 2.51E-07 |
| STX18 | 0.212496198 | 4.366152094 | 5.236415238 | 2.52E-07 |
| RP11-435F17.3 | -0.18228714 | 6.043232624 | -5.235498191 | 2.53E-07 |
| C6orf226 | 0.298600293 | 4.552302187 | 5.234388813 | 2.54E-07 |
| RP11-213G2.2 | -0.628593762 | -1.312087446 | -5.233598936 | 2.55E-07 |
| TMEM194B | -0.315643243 | 3.292495013 | -5.233578067 | 2.55E-07 |
| AUTS2 | 0.61861219 | 3.14402398 | 5.232553801 | 2.57E-07 |
| PDCD1 | 0.756813209 | 0.873636251 | 5.232219199 | 2.57E-07 |
| RP11-383C5.5 | -0.502632385 | 1.01263908 | -5.23191598 | 2.57E-07 |
| FANCG | 0.280252695 | 4.944670117 | 5.231730236 | 2.58E-07 |
| RPL21P119 | 0.234568193 | 4.148298275 | 5.23141019 | 2.58E-07 |
| RAG1 | -0.703899081 | -0.533672559 | -5.229993099 | 2.60E-07 |
| TMEM9B-AS1 | -0.453356481 | 3.524134393 | -5.229297 | 2.61E-07 |
| SLC46A2 | -1.304035188 | -0.162137394 | -5.228752745 | 2.62E-07 |
| RP11-1094M14.4 | -0.757883244 | 0.48311454 | -5.22553874 | 2.66E-07 |
| ENTPD6 | -0.175236218 | 5.208016041 | -5.22431364 | 2.68E-07 |
| CNPY3 | -0.260075951 | 9.182400915 | -5.224253851 | 2.68E-07 |
| PTPN9 | -0.257320285 | 5.765387869 | -5.22398259 | 2.68E-07 |
| RP11-324O2.6 | -0.576314228 | 0.024963035 | -5.22321689 | 2.69E-07 |
| RP1-122P22.2 | -0.897585526 | 0.299201296 | -5.220470339 | 2.73E-07 |
| GAL | 0.86838403 | -0.79302993 | 5.219832609 | 2.74E-07 |
| MEOX1 | -0.657239596 | -2.104852107 | -5.217851543 | 2.76E-07 |
| NAA50 | 0.222886155 | 6.661462828 | 5.217780716 | 2.77E-07 |
| ZNF32 | -0.28101165 | 5.68365197 | -5.217334011 | 2.77E-07 |
| INSIG1 | 0.344603906 | 6.577222108 | 5.216868963 | 2.78E-07 |
| SRCRB4D | -0.609660615 | -0.160004978 | -5.216508328 | 2.78E-07 |
| GCSAM | -0.730868884 | 1.783673581 | -5.216241988 | 2.79E-07 |
| MILR1 | -0.567818923 | 4.786752115 | -5.216228515 | 2.79E-07 |
| ADCK5 | -0.277011331 | 3.782087179 | -5.214275305 | 2.82E-07 |
| FLOT2 | -0.314932018 | 8.394370748 | -5.21311654 | 2.83E-07 |
| ZNF18 | 0.282083552 | 4.511729992 | 5.213090181 | 2.83E-07 |
| RP11-417L14.1 | 0.370708221 | 1.859116728 | 5.21271411 | 2.84E-07 |
| TNFRSF10A | 0.427013552 | 4.619937572 | 5.210553715 | 2.87E-07 |
| RP11-316E14.6 | -0.563351401 | 0.418837352 | -5.209760186 | 2.88E-07 |
| AC079807.2 | -0.391822401 | 2.566699122 | -5.209733793 | 2.88E-07 |
| SCML1 | 0.859191753 | 1.424644017 | 5.209647891 | 2.88E-07 |
| RPS2P1 | 0.622837114 | 0.06412267 | 5.209291514 | 2.89E-07 |
| TSG101 | 0.185593963 | 5.861272851 | 5.208263614 | 2.90E-07 |
| SERP1 | -0.148839352 | 7.42107097 | -5.208261281 | 2.90E-07 |
| AC116366.5 | -0.755875759 | 2.029764275 | -5.208018998 | 2.91E-07 |
| NPHP1 | -0.832586232 | -2.661463833 | -5.208006082 | 2.91E-07 |
| RP1-13D10.2 | -0.592536326 | 0.214999106 | -5.207894798 | 2.91E-07 |
| CHMP6 | -0.269181527 | 5.600111961 | -5.207658614 | 2.91E-07 |
| DSTNP2 | -0.327216223 | 3.408233226 | -5.207033482 | 2.92E-07 |
| RP11-666A8.8 | -0.568322169 | 0.07364113 | -5.206364571 | 2.93E-07 |
| SNX29P1 | -0.508566864 | 0.482188656 | -5.206322867 | 2.93E-07 |
| C15orf52 | -0.547510573 | 2.489203902 | -5.205904901 | 2.94E-07 |
| RP13-516M14.2 | 0.67225764 | -0.055914702 | 5.20521542 | 2.95E-07 |
| RAPGEFL1 | 0.428413153 | 2.432389162 | 5.204155023 | 2.96E-07 |
| ZNF771 | -0.46360796 | 0.34076246 | -5.203449597 | 2.97E-07 |
| PTP4A1 | 0.334282216 | 7.90479655 | 5.202865199 | 2.98E-07 |
| CCL20 | 0.93921456 | -0.603268808 | 5.202727078 | 2.99E-07 |
| SEPT12 | 0.694830506 | -1.730041462 | 5.202641689 | 2.99E-07 |
| GALNT3 | 0.58953033 | 4.027501189 | 5.20176555 | 3.00E-07 |
| JAG2 | 0.634265047 | -0.247339215 | 5.201013358 | 3.01E-07 |
| RP3-508I15.19 | 0.447702912 | 2.399678168 | 5.199850786 | 3.03E-07 |
| HSPD1P1 | -0.366119702 | 5.697280862 | -5.199304941 | 3.04E-07 |
| AC015849.14 | 0.335298336 | 2.699829668 | 5.197964717 | 3.06E-07 |
| C3orf17 | 0.213447513 | 4.951976433 | 5.197699098 | 3.06E-07 |
| ACR | 0.995758292 | -0.651387383 | 5.197137468 | 3.07E-07 |
| RABGGTB | 0.255220309 | 6.189511006 | 5.196064814 | 3.09E-07 |
| RP11-219B17.1 | -0.536571671 | 1.288976603 | -5.19511929 | 3.10E-07 |
| PRDM2 | 0.339111558 | 6.160453859 | 5.194945058 | 3.11E-07 |
| RP11-277I20.3 | -0.478966843 | 2.131164764 | -5.194926475 | 3.11E-07 |
| IGHV5-78 | -0.62207449 | -1.062589279 | -5.194660688 | 3.11E-07 |
| RAB11FIP4 | 0.608487845 | 3.071014787 | 5.194075971 | 3.12E-07 |
| NKX3-1 | 0.921313539 | 1.216577857 | 5.194043505 | 3.12E-07 |
| RP11-597D13.9 | -1.159876585 | 0.486512486 | -5.192848164 | 3.14E-07 |
| SAC3D1 | -0.462908423 | 4.157689229 | -5.191680767 | 3.16E-07 |
| TRANK1 | -0.46008782 | 4.693278028 | -5.191225194 | 3.17E-07 |
| C6orf62 | -0.185111382 | 8.243855888 | -5.189422304 | 3.19E-07 |
| RP11-10K16.1 | -0.44404673 | 2.946851765 | -5.187715499 | 3.22E-07 |
| TNFSF13B | -0.595740055 | 6.671780144 | -5.186689664 | 3.24E-07 |
| SMCO4 | -0.727530405 | 3.894442653 | -5.186135295 | 3.25E-07 |
| VPS29 | -0.215779538 | 7.002978546 | -5.18436835 | 3.28E-07 |
| PUS7L | -0.193161511 | 4.139762437 | -5.184217978 | 3.28E-07 |
| LIN7B | -0.318659192 | 2.028839442 | -5.183425467 | 3.29E-07 |
| APBB2 | 0.98354976 | -1.17586852 | 5.182274353 | 3.31E-07 |
| RPL7AP60 | 0.276228639 | 2.607001959 | 5.182060861 | 3.32E-07 |
| STAU2 | -0.285438392 | 4.192155815 | -5.181986432 | 3.32E-07 |
| RP1-197B17.3 | -0.500644275 | 0.352764032 | -5.181525927 | 3.32E-07 |
| DERL2 | 0.245926412 | 4.516633993 | 5.180858294 | 3.34E-07 |
| TRIM35 | -0.243163534 | 4.829923165 | -5.180710848 | 3.34E-07 |
| P2RX1 | -0.393021309 | 7.32108604 | -5.177983771 | 3.38E-07 |
| FAM57A | 0.765501469 | 1.735330472 | 5.17690421 | 3.40E-07 |
| SMG7 | 0.12863088 | 6.645021258 | 5.176135373 | 3.42E-07 |
| ICK | -0.342337467 | 3.960933297 | -5.175857822 | 3.42E-07 |
| CDK5 | -0.329964616 | 3.883983296 | -5.175227372 | 3.43E-07 |
| TXNDC17 | -0.251445287 | 3.830700947 | -5.174038713 | 3.45E-07 |
| HMGN2 | 0.322719619 | 7.574962824 | 5.173964092 | 3.45E-07 |
| SDCBP2-AS1 | -0.43141634 | 1.554205144 | -5.173726583 | 3.46E-07 |
| RP11-538D16.2 | -0.927172593 | -2.116970542 | -5.172457877 | 3.48E-07 |
| BOD1L1 | 0.210890004 | 5.833727097 | 5.172328466 | 3.48E-07 |
| COX10 | -0.190692044 | 4.788457772 | -5.170874008 | 3.51E-07 |
| PRNP | 0.429001136 | 7.647508001 | 5.170468489 | 3.52E-07 |
| MTHFD2L | 0.408295471 | 3.349169992 | 5.16911039 | 3.54E-07 |
| RP6-74O6.6 | -0.694716644 | 0.007607456 | -5.168895492 | 3.54E-07 |
| SELM | 0.689032316 | 0.495496544 | 5.168667033 | 3.55E-07 |
| PLEKHG2 | 0.433513935 | 4.836441188 | 5.168481429 | 3.55E-07 |
| SND1 | -0.262809999 | 7.369725036 | -5.167212568 | 3.57E-07 |
| CACFD1 | -0.455132655 | 2.012738493 | -5.166718838 | 3.58E-07 |
| RP11-262H14.5 | -0.683419103 | 0.130677569 | -5.166709039 | 3.58E-07 |
| EEF1D | 0.182194745 | 7.305503779 | 5.164029845 | 3.63E-07 |
| CANT1 | 0.235965626 | 5.512526688 | 5.163649521 | 3.64E-07 |
| TLN1 | -0.292724807 | 8.963769817 | -5.162543796 | 3.66E-07 |
| CHCHD5 | 0.227335263 | 3.804397947 | 5.161812111 | 3.67E-07 |
| LPCAT2 | -0.551065173 | 5.900131124 | -5.161647225 | 3.68E-07 |
| RP11-552D4.1 | -0.913102051 | -0.857839596 | -5.161326794 | 3.68E-07 |
| LRRC45 | -0.373103235 | 4.654864771 | -5.158783787 | 3.73E-07 |
| FAM225B | -0.879020923 | -3.096583231 | -5.158396109 | 3.74E-07 |
| MRPS21 | -0.194727296 | 7.001027382 | -5.158292746 | 3.74E-07 |
| BRD4 | 0.176860022 | 6.065881304 | 5.1578965 | 3.75E-07 |
| VCAM1 | 1.310497164 | -1.555560314 | 5.157637178 | 3.75E-07 |
| RNPS1P1 | 0.168700119 | 6.820392647 | 5.157358877 | 3.76E-07 |
| CHPT1 | 0.382166747 | 4.957415207 | 5.157054995 | 3.76E-07 |
| AC016586.1 | 0.595149469 | 1.794324896 | 5.156585827 | 3.77E-07 |
| C1orf50 | 0.18319053 | 3.870264222 | 5.156521778 | 3.77E-07 |
| EPG5 | 0.208233971 | 4.813503799 | 5.154442668 | 3.81E-07 |
| LINC00667 | -0.326337371 | 4.455421085 | -5.154418946 | 3.81E-07 |
| ZNF12 | 0.288154226 | 4.652201915 | 5.153638862 | 3.83E-07 |
| RTN4 | -0.196769098 | 7.682319275 | -5.152092018 | 3.86E-07 |
| DERA | -0.261775902 | 5.490611785 | -5.152004842 | 3.86E-07 |
| ARNTL | 0.620388123 | 3.560393234 | 5.150646931 | 3.89E-07 |
| GHDC | -0.24468086 | 5.148346655 | -5.150551908 | 3.89E-07 |
| KLHDC1 | -0.358615511 | 3.050362607 | -5.149806582 | 3.90E-07 |
| TRAV27 | -0.636774918 | -0.902975728 | -5.149458534 | 3.91E-07 |
| POU6F1 | 0.537039859 | 2.139262019 | 5.149274098 | 3.91E-07 |
| DROSHA | -0.271369012 | 5.480532341 | -5.149157608 | 3.91E-07 |
| SRP14 | 0.144757749 | 8.938488577 | 5.14906619 | 3.92E-07 |
| RP11-582J16.5 | 0.400499068 | 2.993234164 | 5.148589711 | 3.93E-07 |
| CD5 | 0.796508137 | 3.840517307 | 5.147802223 | 3.94E-07 |
| ZNF462 | 1.22405582 | -1.96927448 | 5.147750539 | 3.94E-07 |
| ZSCAN21 | -0.304624229 | 4.127697641 | -5.147187768 | 3.95E-07 |
| RNF182 | 1.072350545 | -2.225815935 | 5.146686483 | 3.96E-07 |
| LYPLA2P1 | -0.260311135 | 4.888161144 | -5.146547479 | 3.97E-07 |
| RP11-460C6.1 | -0.500092422 | -1.127336107 | -5.145993385 | 3.98E-07 |
| TRBV7-5 | -1.034751978 | -0.364048397 | -5.145765291 | 3.98E-07 |
| RSL24D1 | 0.299898062 | 7.655616917 | 5.145351287 | 3.99E-07 |
| PSMC3 | 0.178091988 | 6.977315069 | 5.144953288 | 4.00E-07 |
| USP35 | -0.299476259 | 3.33420294 | -5.144541554 | 4.01E-07 |
| NRIP1 | 0.643439409 | 6.284128654 | 5.144011367 | 4.02E-07 |
| AF001548.6 | 0.593922128 | 0.771252842 | 5.143174013 | 4.03E-07 |
| RP11-271C24.3 | 0.499634741 | 1.717284189 | 5.142670265 | 4.04E-07 |
| MAGEH1 | -0.409061554 | 4.249067176 | -5.142443909 | 4.05E-07 |
| PAXIP1 | 0.229112561 | 3.928142574 | 5.142210254 | 4.05E-07 |
| TRAK2 | -0.322526191 | 5.48065252 | -5.141673141 | 4.06E-07 |
| TLDC1 | -0.453886902 | 1.865533191 | -5.14158027 | 4.07E-07 |
| RP11-117D22.2 | -0.948058448 | 0.113749256 | -5.141428499 | 4.07E-07 |
| AP3S1 | 0.281300137 | 6.323165071 | 5.140775753 | 4.08E-07 |
| CCDC157 | 0.328763727 | 1.331884632 | 5.140570793 | 4.09E-07 |
| NDUFS4 | -0.21971021 | 6.346863627 | -5.140259435 | 4.09E-07 |
| AC092620.2 | 0.695627123 | 0.426242519 | 5.139643331 | 4.11E-07 |
| CBX6 | 0.370438985 | 5.154752294 | 5.139459635 | 4.11E-07 |
| CCT5P2 | -0.504238097 | 0.684971883 | -5.139030433 | 4.12E-07 |
| FTH1P22 | -0.715709813 | -0.319329052 | -5.138939951 | 4.12E-07 |
| RP11-108M9.6 | -0.6604147 | -0.488236529 | -5.138872577 | 4.12E-07 |
| MRPL54 | 0.177690347 | 6.313569332 | 5.138470688 | 4.13E-07 |
| SUOX | -0.299904536 | 4.269752241 | -5.138229446 | 4.14E-07 |
| YIF1A | -0.236121548 | 5.108489098 | -5.1380394 | 4.14E-07 |
| KATNA1 | 0.142709049 | 5.576986776 | 5.137958629 | 4.14E-07 |
| HERC5 | 0.533970332 | 4.153405278 | 5.137797983 | 4.14E-07 |
| KRT86 | 0.691066067 | -2.090231843 | 5.136866424 | 4.16E-07 |
| NUP62CL | 0.840215847 | -0.888839619 | 5.136016843 | 4.18E-07 |
| RNU1-1 | -0.827793787 | 0.080534691 | -5.135878455 | 4.18E-07 |
| ZNF232 | -0.441121139 | 2.573573767 | -5.135564171 | 4.19E-07 |
| RP11-460I13.6 | -0.588029939 | 0.048328387 | -5.135082886 | 4.20E-07 |
| CTD-2619J13.17 | -0.560490716 | 1.425752948 | -5.1342729 | 4.22E-07 |
| RP13-383K5.4 | -0.258124392 | 5.426777417 | -5.134157568 | 4.22E-07 |
| THAP10 | -0.883798907 | -1.344666101 | -5.134033683 | 4.22E-07 |
| FRAS1 | 0.968407247 | -4.775960967 | 5.13373449 | 4.23E-07 |
| KLHL5 | 0.305001988 | 4.84981111 | 5.133388294 | 4.24E-07 |
| C2CD3 | 0.154263059 | 4.368678974 | 5.133083506 | 4.24E-07 |
| RP11-474G23.3 | -0.529291346 | 1.266337 | -5.131495204 | 4.28E-07 |
| CDS2 | 0.248265515 | 5.771719313 | 5.131425495 | 4.28E-07 |
| CYFIP1 | -0.453607695 | 5.092089948 | -5.130677998 | 4.30E-07 |
| C3orf20 | 0.554941281 | -0.246561369 | 5.129591628 | 4.32E-07 |
| HIST1H2BB | -0.921105797 | 0.149754308 | -5.129448447 | 4.32E-07 |
| PPP3CC | 0.320934208 | 5.541390398 | 5.128870498 | 4.33E-07 |
| ZNF653 | -0.294087346 | 2.146173494 | -5.128827797 | 4.34E-07 |
| RP11-819M15.1 | 0.304634157 | 3.68550291 | 5.128693784 | 4.34E-07 |
| CEP57L1 | -0.34239706 | 3.0429273 | -5.128289169 | 4.35E-07 |
| OGG1 | -0.386451605 | 3.26890306 | -5.127131836 | 4.37E-07 |
| HMGB1 | 0.247997375 | 7.883187675 | 5.127059718 | 4.37E-07 |
| CCND1 | 1.013043308 | 0.49397877 | 5.126423315 | 4.39E-07 |
| TMEM101 | -0.232768666 | 4.830872275 | -5.125289429 | 4.41E-07 |
| GBAP1 | -0.361532851 | 3.214229178 | -5.124623288 | 4.43E-07 |
| GOLPH3 | -0.286727128 | 7.693194973 | -5.123334969 | 4.46E-07 |
| PRUNE | -0.300998249 | 4.658238731 | -5.12204766 | 4.49E-07 |
| CTA-390C10.10 | -0.55024018 | 1.232253288 | -5.121937918 | 4.49E-07 |
| COPS7B | -0.154777957 | 4.908250451 | -5.121779891 | 4.49E-07 |
| PHF2 | 0.186389614 | 5.934544499 | 5.121600851 | 4.50E-07 |
| RP11-75L1.1 | 0.673970797 | 0.478330586 | 5.121439968 | 4.50E-07 |
| ISCU | 0.273223916 | 6.224731218 | 5.120797157 | 4.51E-07 |
| RP11-734J24.1 | 0.287427012 | 3.145957376 | 5.11945171 | 4.54E-07 |
| RBM4 | 0.351296209 | 3.509859891 | 5.119084761 | 4.55E-07 |
| RP11-561O4.1 | 0.291657247 | 2.162880262 | 5.118879905 | 4.56E-07 |
| ZXDA | 0.517555909 | 2.760727188 | 5.118554444 | 4.56E-07 |
| RARS | -0.201257676 | 5.681676797 | -5.118502174 | 4.57E-07 |
| C9orf40 | 0.441527553 | 3.124312435 | 5.11783816 | 4.58E-07 |
| RP4-576H24.2 | -0.977273327 | 0.26040315 | -5.117486239 | 4.59E-07 |
| FAM84B | 0.72173935 | 2.665279599 | 5.117388384 | 4.59E-07 |
| FMNL2 | -0.701524058 | 1.23245334 | -5.117237598 | 4.60E-07 |
| ARMCX3 | -0.263203716 | 5.405072587 | -5.11705425 | 4.60E-07 |
| RPL21P134 | 0.300627242 | 2.438406119 | 5.117041847 | 4.60E-07 |
| FDX1 | 0.30445626 | 4.687546713 | 5.116311916 | 4.62E-07 |
| EIF4A2P4 | -0.259450182 | 3.049507177 | -5.115330209 | 4.64E-07 |
| PCDHB2 | 1.118339514 | -2.339884229 | 5.114807144 | 4.65E-07 |
| TRIM26 | 0.221607376 | 6.135393381 | 5.114162221 | 4.67E-07 |
| C18orf8 | 0.252979242 | 6.175255909 | 5.113944718 | 4.67E-07 |
| C15orf39 | 0.411073577 | 7.31068768 | 5.113682913 | 4.68E-07 |
| HNRNPA1P52 | 0.513375021 | 0.399287215 | 5.11353742 | 4.68E-07 |
| GUSB | 0.285574501 | 7.237992805 | 5.113407048 | 4.68E-07 |
| WDFY1 | 0.289161709 | 6.47756113 | 5.112146047 | 4.71E-07 |
| CHST12 | -0.588907671 | 6.053586802 | -5.110847092 | 4.74E-07 |
| AK2P2 | -0.728708208 | 0.814395605 | -5.110538879 | 4.75E-07 |
| PDIA4 | -0.384712351 | 7.330357916 | -5.109858316 | 4.77E-07 |
| TBC1D13 | -0.255983406 | 5.845519757 | -5.109051369 | 4.79E-07 |
| DENND1B | 0.268366006 | 4.173697154 | 5.108051164 | 4.81E-07 |
| HIST3H2A | 1.112993166 | 2.468068104 | 5.107974477 | 4.81E-07 |
| CELA1 | -0.681428562 | -0.111072901 | -5.107561571 | 4.82E-07 |
| SLC25A22 | -0.229765732 | 4.071260038 | -5.107206712 | 4.83E-07 |
| CDCA3 | 0.646323838 | 2.336292613 | 5.106178447 | 4.86E-07 |
| RPS7P3 | 0.434748101 | 1.73987324 | 5.102543309 | 4.95E-07 |
| F13A1 | -1.094418096 | 6.375003936 | -5.102513042 | 4.95E-07 |
| MB21D2 | 0.839362833 | -0.431575303 | 5.101978453 | 4.96E-07 |
| WASF2 | -0.206124094 | 8.460145045 | -5.101175501 | 4.98E-07 |
| CTC-559E9.6 | -0.416272173 | 2.645339996 | -5.100771019 | 4.99E-07 |
| MYOM1 | -0.489005524 | 2.031151802 | -5.100765726 | 4.99E-07 |
| FTH1P20 | 0.509091891 | 7.523390076 | 5.100215664 | 5.00E-07 |
| RP11-90E9.1 | 0.269389977 | 4.013379245 | 5.10001601 | 5.01E-07 |
| RP11-797A18.6 | 0.54187086 | 4.424001224 | 5.099939234 | 5.01E-07 |
| ZBTB44 | -0.23260935 | 5.993260252 | -5.099427292 | 5.02E-07 |
| LINS | -0.230902673 | 3.668304636 | -5.098746336 | 5.04E-07 |
| SIN3B | -0.204225721 | 4.819295645 | -5.098104696 | 5.06E-07 |
| RBM39 | 0.256380358 | 8.017383456 | 5.098085951 | 5.06E-07 |
| GAPDHP72 | 0.466905667 | 3.154091455 | 5.097577623 | 5.07E-07 |
| TAS2R19 | -0.552675657 | -0.384662598 | -5.096678454 | 5.09E-07 |
| ARCN1 | -0.266764552 | 7.11914725 | -5.096394717 | 5.10E-07 |
| RBBP4P2 | -0.257033281 | 5.50869342 | -5.095417755 | 5.12E-07 |
| FLT1 | 0.939677355 | -1.850518148 | 5.095021598 | 5.13E-07 |
| ZIK1 | 0.599323704 | 2.602094387 | 5.094869297 | 5.14E-07 |
| MANBAL | -0.245511699 | 4.641392438 | -5.094606695 | 5.15E-07 |
| PMPCA | 0.192502681 | 5.55975364 | 5.094184853 | 5.16E-07 |
| CDK5RAP1 | 0.17068894 | 5.004412844 | 5.093621544 | 5.17E-07 |
| HMGA1P2 | 0.390664379 | 5.563020808 | 5.092409112 | 5.20E-07 |
| FGL2 | -1.10181891 | 6.583576855 | -5.092211705 | 5.21E-07 |
| ESD | -0.216137318 | 6.583047296 | -5.091944069 | 5.21E-07 |
| RP11-214K3.23 | -0.667882585 | -0.120427099 | -5.091649836 | 5.22E-07 |
| KIAA1244 | 1.118247983 | -3.043105586 | 5.091129705 | 5.24E-07 |
| CFD | -0.900247358 | 9.368813106 | -5.091055651 | 5.24E-07 |
| HHAT | -0.550973733 | 2.607640843 | -5.090898443 | 5.24E-07 |
| GPR160 | 0.699242318 | 4.707977816 | 5.090031473 | 5.26E-07 |
| SPTAN1 | -0.243716782 | 6.568718083 | -5.089417416 | 5.28E-07 |
| CTD-2541M15.3 | -0.699724108 | -0.891052509 | -5.088984142 | 5.29E-07 |
| TAS2R14 | -0.459182057 | 1.673310444 | -5.088660763 | 5.30E-07 |
| U91328.2 | -0.760173597 | -0.841437254 | -5.087157921 | 5.34E-07 |
| ASF1B | 0.617194271 | 4.773110489 | 5.086572039 | 5.36E-07 |
| ID4 | 0.84940538 | -2.670555286 | 5.086525461 | 5.36E-07 |
| MRPS6 | -0.28241338 | 4.281795945 | -5.08624031 | 5.36E-07 |
| NAPA | 0.209551219 | 6.329911482 | 5.086127743 | 5.37E-07 |
| TRAF3IP2 | 0.383871498 | 4.451594829 | 5.086002622 | 5.37E-07 |
| FCRL2 | -0.925258066 | -0.135397737 | -5.085945883 | 5.37E-07 |
| RP11-20I23.6 | -0.557780844 | -0.14475582 | -5.08590548 | 5.37E-07 |
| BTN3A1 | -0.392186815 | 5.618349317 | -5.083848612 | 5.43E-07 |
| CEP250 | -0.266651981 | 4.944043794 | -5.080894222 | 5.51E-07 |
| CALY | 0.644022049 | -1.845338712 | 5.080404412 | 5.52E-07 |
| AKT1S1 | 0.158539254 | 4.551122209 | 5.079829912 | 5.54E-07 |
| RP11-973N13.2 | 0.34047496 | 2.432894737 | 5.079451963 | 5.55E-07 |
| PTPRN | 0.81003801 | -4.364524193 | 5.07906424 | 5.56E-07 |
| AMACR | -0.434939627 | 0.342584481 | -5.076583475 | 5.63E-07 |
| WASH5P | 0.415775886 | 1.099993827 | 5.076128026 | 5.64E-07 |
| LONRF3 | 0.748707135 | 2.567949543 | 5.075941418 | 5.65E-07 |
| TMEM234 | -0.217929668 | 3.509526581 | -5.075684795 | 5.65E-07 |
| KLHL25 | 0.510415534 | 0.839859877 | 5.07566387 | 5.65E-07 |
| NSDHL | -0.213851234 | 4.986547875 | -5.074098192 | 5.70E-07 |
| FBXL16 | -0.694750897 | 0.244468412 | -5.073538582 | 5.72E-07 |
| RP11-442H21.2 | 0.859512029 | -0.250806695 | 5.07310896 | 5.73E-07 |
| SERPINE2 | 0.977648737 | 0.778705139 | 5.071714147 | 5.77E-07 |
| MIR374B | -0.604797229 | 0.037166145 | -5.071706638 | 5.77E-07 |
| HSPH1 | -0.41604281 | 6.200372881 | -5.071629369 | 5.77E-07 |
| RP11-187C18.3 | -0.737865908 | -0.879269828 | -5.071484662 | 5.77E-07 |
| ASIC4 | 0.810567378 | -2.765791117 | 5.071201252 | 5.78E-07 |
| PRSS42 | -0.647321339 | -2.022521266 | -5.071102243 | 5.78E-07 |
| RHOH | 0.645489952 | 4.418690814 | 5.070602563 | 5.80E-07 |
| SPATA24 | -0.420659181 | 2.248704379 | -5.069519751 | 5.83E-07 |
| MAST4-AS1 | -0.67828243 | 1.186962182 | -5.069286498 | 5.84E-07 |
| GMCL1 | -0.21512498 | 5.112872249 | -5.068517907 | 5.86E-07 |
| BRPF3 | 0.248763422 | 5.350586569 | 5.067069101 | 5.90E-07 |
| ERLIN2 | -0.262989106 | 5.131420688 | -5.067005452 | 5.90E-07 |
| RTCA | 0.166866191 | 5.722857372 | 5.066840746 | 5.91E-07 |
| SREBF1 | 0.332939615 | 5.154872976 | 5.066406752 | 5.92E-07 |
| B9D1 | 0.587088025 | -0.055239447 | 5.066376215 | 5.92E-07 |
| KIAA0195 | -0.204417616 | 5.677234084 | -5.066244669 | 5.93E-07 |
| RP13-228J13.5 | -0.717188747 | -0.642741149 | -5.066205557 | 5.93E-07 |
| KIAA0141 | -0.207132294 | 6.285066301 | -5.066039077 | 5.93E-07 |
| CCT4 | -0.246663761 | 7.87683609 | -5.065842046 | 5.94E-07 |
| AC006001.1 | 0.583980175 | -0.433113822 | 5.065441091 | 5.95E-07 |
| MGME1 | -0.225424861 | 5.94107251 | -5.065090351 | 5.96E-07 |
| SLAMF8 | -0.807686054 | -0.201190694 | -5.063839139 | 6.00E-07 |
| PRMT6 | -0.356378744 | 4.60387912 | -5.063819959 | 6.00E-07 |
| TMEM68 | -0.289590626 | 2.675897435 | -5.063748249 | 6.00E-07 |
| EPB41L4A-AS1 | 0.441893987 | 5.55897274 | 5.062953927 | 6.02E-07 |
| CTTNBP2NL | -0.793640698 | 1.396136245 | -5.06283501 | 6.03E-07 |
| ALG2 | 0.273830763 | 5.2526199 | 5.062712358 | 6.03E-07 |
| SLC15A4 | 0.355535428 | 5.039891206 | 5.062475577 | 6.04E-07 |
| SNRPC | 0.199564381 | 7.582412409 | 5.061625445 | 6.06E-07 |
| ZNF347 | -0.472265838 | 2.326037661 | -5.06074244 | 6.09E-07 |
| IGLON5 | 1.324636789 | -0.73779891 | 5.060640818 | 6.09E-07 |
| AC006026.13 | -0.785038423 | 0.484721275 | -5.060511996 | 6.10E-07 |
| SCIMP | -1.229587845 | 2.87930239 | -5.059783025 | 6.12E-07 |
| RP11-589P10.5 | -0.356737333 | 3.675231803 | -5.059044684 | 6.14E-07 |
| RP11-815M8.1 | -0.829038482 | -1.399058113 | -5.058611397 | 6.15E-07 |
| SLC22A23 | 0.8973703 | -0.217110314 | 5.057730735 | 6.18E-07 |
| HKDC1 | -0.638435076 | -1.781790372 | -5.055606849 | 6.25E-07 |
| ATP8B4 | -0.616211789 | 6.409239017 | -5.055047097 | 6.26E-07 |
| MED4 | 0.13616493 | 6.72989746 | 5.05496523 | 6.27E-07 |
| STARD4 | 0.601392235 | 4.773798696 | 5.054962165 | 6.27E-07 |
| RP11-400F19.18 | 0.584102594 | 2.432014089 | 5.054827074 | 6.27E-07 |
| C12orf60 | -0.525864528 | -1.900140506 | -5.05230987 | 6.35E-07 |
| LILRA1 | -0.904371215 | 4.682879933 | -5.052132335 | 6.36E-07 |
| GTF3C2 | -0.149148145 | 5.622484515 | -5.052118092 | 6.36E-07 |
| CPLX1 | 0.765535445 | -1.712754741 | 5.052117106 | 6.36E-07 |
| GMDS-AS1 | -0.316395256 | 1.985378704 | -5.050257785 | 6.42E-07 |
| DNA2 | 0.329183313 | 3.059140831 | 5.05015854 | 6.42E-07 |
| IFI35 | -0.299615538 | 5.71027385 | -5.04951778 | 6.44E-07 |
| CTSL | 1.146131935 | 4.36390259 | 5.049279163 | 6.45E-07 |
| PARGP1 | -0.268553922 | 2.534550196 | -5.046536203 | 6.53E-07 |
| FBXW7 | 0.271079632 | 4.706287741 | 5.046458193 | 6.54E-07 |
| CTD-2555K7.2 | -0.549547945 | -1.028741147 | -5.045009526 | 6.58E-07 |
| PPP1CB | 0.215962592 | 7.142336499 | 5.044860971 | 6.59E-07 |
| PHC3 | 0.193076698 | 5.862690446 | 5.044683072 | 6.59E-07 |
| FAM215A | -0.686122439 | -0.935108306 | -5.044533285 | 6.60E-07 |
| TOMM40 | 0.240740371 | 4.985046925 | 5.044486465 | 6.60E-07 |
| DOK1 | -0.335569692 | 6.006215544 | -5.044463626 | 6.60E-07 |
| APOBEC3C | -0.309026577 | 7.439280726 | -5.044451438 | 6.60E-07 |
| CXCR3 | 0.702245053 | 2.206478155 | 5.0443872 | 6.60E-07 |
| FMO5 | -0.397079896 | 2.339083964 | -5.042576619 | 6.66E-07 |
| ANKAR | -0.282786187 | 1.989561136 | -5.042298004 | 6.67E-07 |
| NIFK | -0.210550314 | 6.081172509 | -5.042057363 | 6.68E-07 |
| TGFB3 | 0.419771349 | 1.620287302 | 5.041578576 | 6.70E-07 |
| KCND1 | 0.355877886 | 2.38436425 | 5.038744445 | 6.79E-07 |
| RNU1-122P | -0.626794775 | 0.002933266 | -5.038609963 | 6.80E-07 |
| HMGB1P31 | -0.417160718 | 1.771485486 | -5.038351982 | 6.80E-07 |
| RNU12 | -0.82112048 | 2.571675306 | -5.036467829 | 6.87E-07 |
| BRMS1L | 0.316900469 | 2.664401953 | 5.036198365 | 6.88E-07 |
| PCF11 | -0.199499152 | 6.179479029 | -5.035088311 | 6.92E-07 |
| DKFZP667F0711 | 0.553200721 | 1.484246592 | 5.034089017 | 6.95E-07 |
| NSMCE2 | 0.207817974 | 4.611843057 | 5.033797436 | 6.96E-07 |
| NANP | 0.373884664 | 3.260991945 | 5.033000031 | 6.99E-07 |
| TTC33 | -0.354037789 | 3.707431988 | -5.03271057 | 7.00E-07 |
| RP11-389O22.1 | -0.633144552 | -0.460564645 | -5.032046987 | 7.02E-07 |
| RP11-173M1.5 | -0.602703777 | -0.48191495 | -5.031916717 | 7.02E-07 |
| AP006216.11 | 0.437837253 | 2.861972823 | 5.031791289 | 7.03E-07 |
| RP11-156P1.3 | -0.332226812 | 3.587857453 | -5.031705935 | 7.03E-07 |
| ABHD5 | 0.484871728 | 5.055977967 | 5.031514237 | 7.04E-07 |
| PXDNL | -0.559442903 | -1.83470249 | -5.030931394 | 7.06E-07 |
| TUBA4A | 0.745957823 | 5.917446402 | 5.030763883 | 7.06E-07 |
| CECR1 | -0.788664939 | 6.637762409 | -5.03036497 | 7.08E-07 |
| RP11-204M4.2 | -0.684964941 | -1.51949679 | -5.030184209 | 7.08E-07 |
| CD79B | -0.564647502 | 4.940986931 | -5.027417691 | 7.18E-07 |
| UBE2L6 | -0.310842577 | 7.476223221 | -5.026884627 | 7.20E-07 |
| AP000295.10 | 0.554200393 | 0.743049802 | 5.025916006 | 7.24E-07 |
| ARL6IP4 | 0.356663463 | 1.903247793 | 5.025333375 | 7.26E-07 |
| RAB35 | 0.179130794 | 6.24676678 | 5.025201477 | 7.26E-07 |
| RP11-727F15.9 | -0.552804155 | 1.2862833 | -5.025090716 | 7.27E-07 |
| PTPN18 | -0.364545641 | 6.852636536 | -5.024959454 | 7.27E-07 |
| C17orf66 | 0.742060339 | -0.709641783 | 5.023183053 | 7.33E-07 |
| RP11-1277A3.1 | 0.377577129 | 2.761821266 | 5.023094745 | 7.34E-07 |
| USMG5 | -0.244729997 | 7.718490483 | -5.022800779 | 7.35E-07 |
| TOP2A | 0.791494522 | 4.722524763 | 5.021486597 | 7.40E-07 |
| EIF4A2P2 | -0.516535997 | 0.093294668 | -5.021086958 | 7.41E-07 |
| ZNF165 | 0.519833114 | 2.067976507 | 5.019782806 | 7.46E-07 |
| C1RL | -0.359028302 | 5.816513403 | -5.01911612 | 7.48E-07 |
| GTF2H3 | -0.219404482 | 4.994732393 | -5.018526593 | 7.50E-07 |
| AC093818.1 | 0.434127273 | 2.915608124 | 5.018113549 | 7.52E-07 |
| RP11-21A7A.3 | -0.61117534 | -0.213072469 | -5.016468247 | 7.58E-07 |
| CSNK1E | 0.339530806 | 5.853611403 | 5.016445533 | 7.58E-07 |
| RP11-98L4.1 | 0.389488629 | 3.740390248 | 5.014464888 | 7.66E-07 |
| RP1-278E11.3 | -0.258094777 | 8.541832402 | -5.014011754 | 7.67E-07 |
| KLHL6 | -0.303207962 | 5.138720042 | -5.013892301 | 7.68E-07 |
| TRAV4 | -0.706729194 | -0.023706038 | -5.013216302 | 7.70E-07 |
| RP11-142L4.2 | -0.340421516 | 3.43757818 | -5.012706516 | 7.72E-07 |
| CHTF18 | 0.307420208 | 4.832450436 | 5.011903061 | 7.75E-07 |
| NPB | -0.561330855 | -1.357488679 | -5.011706617 | 7.76E-07 |
| AC127496.1 | -0.418297449 | 2.261644864 | -5.011289098 | 7.78E-07 |
| MSC | 0.986343517 | 0.469579045 | 5.010764133 | 7.80E-07 |
| COPS7A | -0.196256582 | 5.774644616 | -5.00953722 | 7.84E-07 |
| SUCLG1 | -0.172092781 | 5.786492114 | -5.008360184 | 7.89E-07 |
| ASMT | 0.5888129 | -1.489875406 | 5.007005499 | 7.94E-07 |
| AMZ2P1 | 0.32001597 | 3.701725189 | 5.006000178 | 7.98E-07 |
| RP11-932O9.7 | -0.435115648 | 1.780327485 | -5.005686226 | 7.99E-07 |
| RN7SL648P | -0.701904487 | 0.121450333 | -5.005562527 | 8.00E-07 |
| TMEM169 | -0.87825401 | -1.468292498 | -5.00515072 | 8.02E-07 |
| STK25 | -0.181133075 | 5.336691149 | -5.004772197 | 8.03E-07 |
| RP5-1024N4.4 | -0.769133923 | -1.025252738 | -5.004247976 | 8.05E-07 |
| MIR3150A | -0.77397859 | -1.304771702 | -5.003446864 | 8.08E-07 |
| AMMECR1 | 0.418903186 | 3.492451638 | 5.003239243 | 8.09E-07 |
| DNLZ | -0.539449483 | -1.653713177 | -5.002113344 | 8.14E-07 |
| CD2AP | 0.296826225 | 5.356650628 | 5.001904616 | 8.14E-07 |
| ATP2C1 | -0.25610864 | 6.167481049 | -5.00033432 | 8.21E-07 |
| RP1-34L19.1 | 0.64541324 | -1.801436207 | 4.999626724 | 8.24E-07 |
| SFXN4 | -0.308426454 | 5.495625511 | -4.998691856 | 8.27E-07 |
| CTD-2410N18.3 | -0.516381676 | -0.006066717 | -4.998376037 | 8.29E-07 |
| RP11-647K16.1 | 0.420410326 | 1.410946884 | 4.99797437 | 8.30E-07 |
| NCOA5 | -0.301467999 | 5.736141121 | -4.997285674 | 8.33E-07 |
| ANO8 | -0.415492122 | 4.089898159 | -4.997241423 | 8.33E-07 |
| ANKRD26P4 | 0.567340877 | 0.193676204 | 4.996724897 | 8.35E-07 |
| GS1-358P8.4 | -0.348990077 | 4.004268484 | -4.996623577 | 8.36E-07 |
| HNRNPCP1 | -0.34379491 | 2.680200433 | -4.996528614 | 8.36E-07 |
| RP13-977J11.2 | -0.686362927 | 1.583936542 | -4.995062757 | 8.42E-07 |
| RTN4R | -0.806984498 | 2.242415668 | -4.994409009 | 8.45E-07 |
| CTC-297N7.1 | -0.530549978 | -0.132216728 | -4.993967966 | 8.47E-07 |
| MYLK-AS1 | -0.483223113 | 1.80639334 | -4.993282235 | 8.50E-07 |
| KAT6B | 0.272993384 | 5.312336198 | 4.992801058 | 8.52E-07 |
| SLC22A16 | -0.563376951 | 4.195122615 | -4.992487815 | 8.53E-07 |
| MAP3K2 | 0.223847098 | 6.206454452 | 4.991479716 | 8.57E-07 |
| RAB7A | -0.222972465 | 9.13113758 | -4.990577868 | 8.61E-07 |
| RP4-620F22.3 | -0.529745564 | -0.652522325 | -4.989015453 | 8.68E-07 |
| AC068538.2 | 0.421387565 | 1.404950246 | 4.988188504 | 8.71E-07 |
| RP11-385J23.1 | 0.407606832 | 1.935788688 | 4.987829143 | 8.73E-07 |
| RP11-192H23.8 | -0.354993516 | 3.205947072 | -4.986769045 | 8.77E-07 |
| ZNF770 | -0.248653303 | 5.517993014 | -4.986342129 | 8.79E-07 |
| NPBWR1 | 0.970952194 | -2.172935132 | 4.986242767 | 8.79E-07 |
| BPTF | -0.194841304 | 6.389304991 | -4.985923936 | 8.81E-07 |
| CD164 | -0.344369753 | 8.096060182 | -4.984776683 | 8.86E-07 |
| AARS2 | -0.233260321 | 4.218520387 | -4.984563399 | 8.87E-07 |
| TSFM | 0.216593906 | 3.857599525 | 4.98340037 | 8.92E-07 |
| VN1R42P | -0.515462153 | 0.427752572 | -4.98332273 | 8.92E-07 |
| RP11-431N15.2 | -0.679775994 | -0.282172384 | -4.981386984 | 9.01E-07 |
| ZNF615 | 0.284637181 | 3.171614905 | 4.981303495 | 9.01E-07 |
| KIAA0232 | 0.236089196 | 5.450282513 | 4.980518282 | 9.04E-07 |
| PKD2 | -0.37047052 | 5.233572971 | -4.9802842 | 9.06E-07 |
| PUS7 | -0.427105431 | 3.667580227 | -4.979611011 | 9.09E-07 |
| TIMP3 | 1.148109623 | 1.602004474 | 4.979502527 | 9.09E-07 |
| WNT11 | 0.731441083 | 0.46189536 | 4.979032576 | 9.11E-07 |
| RP11-932O9.8 | -0.550072562 | -0.539034569 | -4.978490712 | 9.14E-07 |
| ERGIC2 | -0.279281439 | 4.165215544 | -4.97832323 | 9.14E-07 |
| HSPA9 | 0.21565548 | 7.902354591 | 4.978259624 | 9.15E-07 |
| EIF1AXP1 | -0.307707112 | 7.440759624 | -4.977906117 | 9.16E-07 |
| ATP6V1B2 | -0.32100563 | 6.381384971 | -4.977574 | 9.18E-07 |
| RP11-98D18.16 | -0.610351061 | -0.410512417 | -4.977533545 | 9.18E-07 |
| VTI1B | -0.17623567 | 5.863502157 | -4.976539362 | 9.22E-07 |
| LINC01058 | -0.588492608 | -0.425084134 | -4.976003454 | 9.25E-07 |
| MIR628 | 0.574437092 | 1.157462276 | 4.974848776 | 9.30E-07 |
| PSEN1 | -0.242773427 | 5.951008543 | -4.974825319 | 9.30E-07 |
| RP11-390B4.5 | -0.598555852 | -1.035645686 | -4.974676651 | 9.31E-07 |
| RN7SL834P | 0.410297941 | 4.004755093 | 4.974515115 | 9.31E-07 |
| RCBTB1 | -0.29594346 | 5.706213957 | -4.974432721 | 9.32E-07 |
| PLEKHD1 | 0.651476743 | -2.712738513 | 4.974349379 | 9.32E-07 |
| ASAP1 | 0.487555483 | 6.091895712 | 4.97373953 | 9.35E-07 |
| HYI-AS1 | 0.353168954 | 2.150954135 | 4.97314126 | 9.38E-07 |
| FANCF | -0.347800677 | 3.926897451 | -4.97292053 | 9.39E-07 |
| FBXO45 | 0.235969559 | 4.572500706 | 4.972643922 | 9.40E-07 |
| OXLD1 | -0.209359924 | 5.648756989 | -4.97085534 | 9.48E-07 |
| TPBG | -1.166186337 | 0.632149504 | -4.970646 | 9.49E-07 |
| LL0XNC01-116E7.1 | -0.6896094 | 0.393610394 | -4.969979895 | 9.52E-07 |
| MGST1 | -0.859241284 | 5.60929406 | -4.969462874 | 9.55E-07 |
| RP11-958N24.2 | 0.400290104 | 2.622262425 | 4.968872528 | 9.58E-07 |
| LIX1L | -0.24488228 | 5.261362256 | -4.968696575 | 9.58E-07 |
| HS3ST1 | 0.96774782 | -2.027021731 | 4.966458174 | 9.69E-07 |
| MAPKBP1 | -0.374745731 | 4.316736324 | -4.966186276 | 9.70E-07 |
| ARHGAP11A | 0.4367611 | 4.717957882 | 4.965422015 | 9.74E-07 |
| C1orf198 | 0.531615655 | 2.677999372 | 4.964801027 | 9.77E-07 |
| PIGO | -0.17302433 | 4.040715825 | -4.963975423 | 9.81E-07 |
| RP4-595K12.2 | -0.37422991 | 1.386410199 | -4.963582855 | 9.83E-07 |
| GS1-124K5.12 | 0.229317965 | 5.31652911 | 4.962396591 | 9.88E-07 |
| AC012358.8 | -0.533165521 | -0.716095346 | -4.961644402 | 9.92E-07 |
| VAMP4 | -0.190308378 | 5.139906762 | -4.961495484 | 9.93E-07 |
| RP11-744D14.1 | -0.484746529 | 2.442530619 | -4.960164743 | 9.99E-07 |
| PARP1 | -0.364424564 | 7.058945012 | -4.960017322 | 1.00E-06 |
| SLC25A37 | 0.503789361 | 6.388682688 | 4.959711495 | 1.00E-06 |
| NMRAL1 | -0.215689861 | 5.223357374 | -4.959392758 | 1.00E-06 |
| NUAK2 | 0.825043056 | 3.531824445 | 4.959324814 | 1.00E-06 |
| EIF5A2 | 0.682498253 | 1.832358402 | 4.95928763 | 1.00E-06 |
| AOAH-IT1 | -0.659948692 | 0.593950134 | -4.958920466 | 1.01E-06 |
| TPST2 | -0.285402487 | 6.208531912 | -4.958052356 | 1.01E-06 |
| E2F6 | 0.262992648 | 3.326228895 | 4.957964848 | 1.01E-06 |
| USP24 | 0.179407036 | 6.196511272 | 4.957837851 | 1.01E-06 |
| GCC1 | 0.303183198 | 4.980635523 | 4.957624999 | 1.01E-06 |
| SLC35D3 | 0.688113095 | -2.021570683 | 4.957498844 | 1.01E-06 |
| GGA3 | -0.140261734 | 6.176477307 | -4.957352306 | 1.01E-06 |
| FANCB | 0.291818971 | 2.554055618 | 4.957303317 | 1.01E-06 |
| PGBD4 | -0.313551743 | 3.367315715 | -4.957211604 | 1.01E-06 |
| DTX3L | -0.287225293 | 5.94972232 | -4.957129624 | 1.01E-06 |
| CTD-2196E14.6 | 0.465958173 | 2.078051209 | 4.95556165 | 1.02E-06 |
| CMKLR1 | -0.931882931 | 0.965641056 | -4.955475821 | 1.02E-06 |
| FBXL22 | -0.51593015 | -0.893409106 | -4.955169926 | 1.02E-06 |
| C2orf15 | -0.53031963 | -2.288186025 | -4.955164206 | 1.02E-06 |
| SLC9A6 | 0.256439326 | 4.53893686 | 4.954851705 | 1.03E-06 |
| EDRF1 | 0.173616849 | 4.398681242 | 4.954541949 | 1.03E-06 |
| UBALD1 | 0.232354846 | 5.17287713 | 4.952358357 | 1.04E-06 |
| SLC25A24 | -0.323795556 | 5.331677958 | -4.952229272 | 1.04E-06 |
| HTRA4 | 0.975680531 | -0.055064786 | 4.952100966 | 1.04E-06 |
| MIR4519 | 0.281172239 | 3.194251006 | 4.951555614 | 1.04E-06 |
| RP11-289I10.2 | 0.450364707 | 4.927738643 | 4.951207766 | 1.04E-06 |
| NFYA | -0.244357301 | 6.287480964 | -4.951138802 | 1.04E-06 |
| FAM222A | 0.522627834 | 0.69982456 | 4.951091924 | 1.04E-06 |
| UBXN7-AS1 | 0.551691689 | -0.598004107 | 4.95031827 | 1.05E-06 |
| AC005003.1 | -0.523365246 | 1.460516464 | -4.95024567 | 1.05E-06 |
| POLR3D | -0.272735359 | 5.155248952 | -4.949725332 | 1.05E-06 |
| RP11-235E17.4 | -0.51173322 | -0.425377311 | -4.948986949 | 1.06E-06 |
| RP11-467L13.4 | -0.493206751 | 0.495967406 | -4.948393791 | 1.06E-06 |
| RP11-485G4.2 | 0.335467585 | 3.730862608 | 4.947776601 | 1.06E-06 |
| HNRNPA1L2 | 0.277113624 | 6.004772378 | 4.947440198 | 1.06E-06 |
| RIF1 | 0.206734035 | 5.657498234 | 4.947256084 | 1.06E-06 |
| GAA | -0.473801318 | 6.321486671 | -4.947009478 | 1.07E-06 |
| MAPRE1P1 | 0.364814715 | 2.039994426 | 4.946904025 | 1.07E-06 |
| RP11-506M13.3 | 0.406812359 | 1.034494127 | 4.945463428 | 1.07E-06 |
| FLOT1 | 0.475520199 | 7.382332745 | 4.945277718 | 1.07E-06 |
| RP11-1072C15.4 | 0.424160968 | 0.933773751 | 4.944853935 | 1.08E-06 |
| AC011747.7 | 0.575325693 | -1.006868182 | 4.94480068 | 1.08E-06 |
| SLC9A5 | 0.516768244 | -0.252151225 | 4.944737934 | 1.08E-06 |
| UQCRC2 | -0.172588993 | 7.346895733 | -4.944688837 | 1.08E-06 |
| RP11-342L8.2 | -0.631344106 | -0.678048737 | -4.943655332 | 1.08E-06 |
| C16orf74 | -0.626256155 | 3.314972144 | -4.943522846 | 1.08E-06 |
| RP11-715J22.4 | -0.599608491 | -0.652940522 | -4.943118885 | 1.09E-06 |
| POGK | -0.183152149 | 5.521874278 | -4.943034778 | 1.09E-06 |
| RP11-384P7.6 | -0.68866314 | -0.715279301 | -4.942609815 | 1.09E-06 |
| DUS3L | 0.256230931 | 5.582008718 | 4.942490737 | 1.09E-06 |
| CELF2 | -0.18896651 | 7.515230168 | -4.942402268 | 1.09E-06 |
| TUBB2A | 0.798364844 | 3.516540215 | 4.941236052 | 1.10E-06 |
| FLVCR1-AS1 | -0.465260041 | 1.494896877 | -4.941107232 | 1.10E-06 |
| TBC1D25 | 0.215569175 | 4.769922398 | 4.940041627 | 1.10E-06 |
| AC136289.1 | -0.534517094 | -1.251967343 | -4.939053281 | 1.11E-06 |
| TRAPPC2 | 0.215903719 | 3.578991088 | 4.938007538 | 1.11E-06 |
| PKD1L1 | -0.496968962 | -2.632828879 | -4.936891617 | 1.12E-06 |
| RP11-687F6.1 | -0.574117176 | -0.182634468 | -4.936799516 | 1.12E-06 |
| TCL1A | -1.05998694 | -0.333271819 | -4.936123602 | 1.12E-06 |
| GINS4 | 0.429950167 | 2.896242185 | 4.9358173 | 1.12E-06 |
| RP11-1280N14.3 | -0.388475768 | 2.100942874 | -4.935816856 | 1.12E-06 |
| RP11-64K7.1 | 0.426918641 | 0.955305416 | 4.935710202 | 1.13E-06 |
| PPARA | -0.2783813 | 3.463598044 | -4.935287689 | 1.13E-06 |
| ZNF839 | 0.201365815 | 4.148119644 | 4.935172895 | 1.13E-06 |
| RP11-248J18.3 | 0.323486213 | 4.98993098 | 4.934757734 | 1.13E-06 |
| TAS2R15 | -0.557709537 | 0.288963058 | -4.933040906 | 1.14E-06 |
| PIBF1 | -0.212688789 | 4.750073563 | -4.932777962 | 1.14E-06 |
| MIR589 | 0.582503392 | -0.059827812 | 4.931786226 | 1.15E-06 |
| RP1-35C21.1 | -1.134249707 | -0.906238933 | -4.931384967 | 1.15E-06 |
| BRD3 | -0.203500684 | 5.635604085 | -4.931211104 | 1.15E-06 |
| TMEM164 | -0.260796961 | 6.501420294 | -4.92984258 | 1.16E-06 |
| ACACA | -0.272663638 | 4.524476689 | -4.927465934 | 1.17E-06 |
| TIPIN | 0.264115462 | 3.444594887 | 4.927172874 | 1.17E-06 |
| KIAA0947 | 0.150379413 | 5.62023566 | 4.926353075 | 1.18E-06 |
| CTTN | 0.980324058 | 0.732197914 | 4.925842455 | 1.18E-06 |
| CATSPER2 | -0.461686398 | 1.861604903 | -4.925512555 | 1.18E-06 |
| RP11-247I13.3 | 0.480808333 | 0.422406093 | 4.924718735 | 1.19E-06 |
| PLAC8L1 | -0.548064163 | 0.174623679 | -4.924702237 | 1.19E-06 |
| MCF2L-AS1 | -0.633936975 | -0.931276076 | -4.923769935 | 1.19E-06 |
| SCARNA6 | 0.47139123 | 1.291077882 | 4.923567031 | 1.19E-06 |
| ITPRIPL2 | -0.580538496 | 3.552660293 | -4.923370365 | 1.20E-06 |
| FGFR1 | 0.852900872 | 2.700555583 | 4.922795176 | 1.20E-06 |
| STX16-NPEPL1 | 0.461616021 | 2.609469459 | 4.921681641 | 1.20E-06 |
| ALG1L15P | -0.411635569 | 1.985438143 | -4.920039653 | 1.21E-06 |
| FAU | 0.194459151 | 8.956247488 | 4.918640238 | 1.22E-06 |
| RP11-434H6.6 | 0.715198218 | 3.358408995 | 4.918512823 | 1.22E-06 |
| SNORA73B | -0.535812721 | 1.997495174 | -4.916940372 | 1.23E-06 |
| AGFG1 | 0.341541806 | 6.115696457 | 4.916647754 | 1.23E-06 |
| PPP1R8 | 0.180654159 | 6.427564836 | 4.916511678 | 1.24E-06 |
| MIR425 | -0.608868934 | 0.044005247 | -4.916507469 | 1.24E-06 |
| RP11-96K19.5 | 0.40155711 | 0.803804403 | 4.915761613 | 1.24E-06 |
| CASP8 | 0.285678394 | 6.350947148 | 4.9146675 | 1.25E-06 |
| GGH | 0.488365176 | 4.698013404 | 4.914614346 | 1.25E-06 |
| RIPK4 | 0.841071562 | -3.000975061 | 4.914320197 | 1.25E-06 |
| AC016735.2 | 0.781836944 | -1.369824068 | 4.914146861 | 1.25E-06 |
| HYAL2 | -0.436152782 | 2.697927358 | -4.913511331 | 1.25E-06 |
| RP11-353B9.1 | -0.511427235 | 0.743298245 | -4.913475165 | 1.25E-06 |
| C18orf56 | 0.557336431 | 0.811281036 | 4.913240284 | 1.26E-06 |
| AC016405.1 | -0.491328018 | 0.771679548 | -4.91259693 | 1.26E-06 |
| RP11-958N24.1 | 0.260021365 | 3.502841753 | 4.912362807 | 1.26E-06 |
| RABEPK | -0.180043087 | 4.159819992 | -4.910577134 | 1.27E-06 |
| OSBPL5 | -0.408415276 | 5.434728472 | -4.910319645 | 1.27E-06 |
| RP11-986G18.1 | -0.60938027 | -0.457828329 | -4.910175499 | 1.27E-06 |
| CTD-3051D23.1 | -0.747754884 | -1.366881879 | -4.909970264 | 1.28E-06 |
| SUCLA2 | -0.191330945 | 4.74908937 | -4.909915299 | 1.28E-06 |
| DIRC2 | 0.413381082 | 4.412158275 | 4.909850423 | 1.28E-06 |
| ANKRD29 | 0.708989049 | -2.774394251 | 4.908956711 | 1.28E-06 |
| SULT1A4 | -0.441433171 | -1.210466333 | -4.90864564 | 1.28E-06 |
| ERLIN1 | -0.426459979 | 6.780890664 | -4.908060497 | 1.29E-06 |
| RP11-314A20.2 | -0.521088402 | -0.188221366 | -4.907735524 | 1.29E-06 |
| AC083900.1 | 0.512372719 | 1.420731281 | 4.906325058 | 1.30E-06 |
| YWHAEP5 | -0.325022504 | 2.493756761 | -4.906161592 | 1.30E-06 |
| HMGN2P46 | 0.527788451 | -2.051165158 | 4.905729238 | 1.30E-06 |
| HAUS5 | 0.227508046 | 4.970065219 | 4.905638112 | 1.30E-06 |
| FBXO10 | -0.459675689 | 0.910907785 | -4.904711473 | 1.31E-06 |
| NDUFAF4 | -0.284641268 | 3.802603915 | -4.904607789 | 1.31E-06 |
| ANGEL1 | -0.245433801 | 4.733709577 | -4.902662904 | 1.32E-06 |
| AC006116.22 | -0.505353487 | 1.666809957 | -4.90238869 | 1.32E-06 |
| APOBEC3G | -0.263973638 | 4.866607117 | -4.902214549 | 1.32E-06 |
| NGRN | 0.207412931 | 4.962796894 | 4.902035716 | 1.33E-06 |
| PLIN1 | -0.616257137 | -1.646525424 | -4.901857404 | 1.33E-06 |
| SNRNP70 | -0.258948449 | 8.168019323 | -4.899198842 | 1.34E-06 |
| ASIC3 | 0.528006369 | 1.773495181 | 4.898838667 | 1.35E-06 |
| HIST1H4C | -0.697636342 | 1.546625427 | -4.898480109 | 1.35E-06 |
| SPOPL | 0.28458792 | 5.584544538 | 4.898025865 | 1.35E-06 |
| B3GALTL | 0.31924885 | 3.261623874 | 4.897852077 | 1.35E-06 |
| RP11-477D19.2 | -0.476480068 | 1.714451512 | -4.897808425 | 1.35E-06 |
| SCFD2 | -0.260111457 | 4.640106658 | -4.896646639 | 1.36E-06 |
| TMEM144 | -0.588946742 | 1.768309349 | -4.896227315 | 1.36E-06 |
| SLMO2 | 0.168909107 | 6.694135044 | 4.895560194 | 1.37E-06 |
| ZNF500 | -0.227292597 | 3.014846347 | -4.89553123 | 1.37E-06 |
| MIR3198-1 | 0.602852266 | 0.706611254 | 4.895259538 | 1.37E-06 |
| DNAJB1 | 0.323682164 | 7.184661357 | 4.894629486 | 1.37E-06 |
| RP11-134L10.1 | 0.576980079 | 3.460987423 | 4.894620786 | 1.37E-06 |
| PARVB | 0.35755563 | 5.051003466 | 4.893989852 | 1.38E-06 |
| TAS2R20 | -0.528890682 | 0.311879414 | -4.892033693 | 1.39E-06 |
| PHLPP2 | 0.361668512 | 2.227383772 | 4.891734147 | 1.39E-06 |
| CCDC64 | 0.651222267 | 1.707979903 | 4.891729883 | 1.39E-06 |
| RNU6-226P | -0.653365699 | -0.061240705 | -4.89169425 | 1.39E-06 |
| RP1-178F15.4 | -0.408830455 | 2.002433326 | -4.891217449 | 1.40E-06 |
| RP11-497H16.5 | -0.509431336 | 1.828281991 | -4.891143143 | 1.40E-06 |
| C17orf51 | -0.805307278 | -0.949016172 | -4.890682558 | 1.40E-06 |
| HYPK | 0.348543288 | 2.703154917 | 4.890312154 | 1.40E-06 |
| TCEAL1 | -0.294418476 | 4.834618114 | -4.890161739 | 1.40E-06 |
| MS4A14 | -0.985875559 | 3.001553417 | -4.889528594 | 1.41E-06 |
| TTC13 | -0.320737984 | 4.607802796 | -4.889309551 | 1.41E-06 |
| RNA5SP155 | 0.596310943 | 0.565867037 | 4.886237283 | 1.43E-06 |
| G3BP1 | -0.240180158 | 6.240352554 | -4.885954016 | 1.43E-06 |
| RP11-611E13.2 | 0.382853784 | 4.980931459 | 4.885534279 | 1.43E-06 |
| NCOR2 | 0.175231882 | 6.716355798 | 4.88499621 | 1.44E-06 |
| RP11-582J16.4 | -0.66578859 | -3.207384935 | -4.88451526 | 1.44E-06 |
| KDM1B | -0.316382484 | 6.623910934 | -4.884489042 | 1.44E-06 |
| RP11-83A24.2 | -0.29297338 | 1.853991895 | -4.884404468 | 1.44E-06 |
| HIST1H2AH | -0.90991762 | 1.376067977 | -4.884199601 | 1.44E-06 |
| RP11-146F11.1 | 0.331165834 | 1.715388088 | 4.884089488 | 1.45E-06 |
| PLXNA1 | 0.601345696 | 1.902390169 | 4.883730693 | 1.45E-06 |
| CTC-444N24.6 | -0.437118049 | 1.039778862 | -4.883708014 | 1.45E-06 |
| UBE2Q2P1 | 0.476635606 | 3.198601762 | 4.883464707 | 1.45E-06 |
| SPATA25 | -0.500124051 | 2.126530401 | -4.882821047 | 1.45E-06 |
| RECQL4 | 0.490842117 | 4.657646366 | 4.882362476 | 1.46E-06 |
| HAX1 | -0.159897748 | 6.613116821 | -4.882329289 | 1.46E-06 |
| ANKRD13C | 0.174976716 | 4.708180315 | 4.881060305 | 1.47E-06 |
| YPEL5 | 0.405252372 | 8.392948532 | 4.880167019 | 1.47E-06 |
| CTD-2290P7.1 | 0.326467754 | 2.243247383 | 4.879331521 | 1.48E-06 |
| METTL2B | -0.274066335 | 3.147955911 | -4.879305698 | 1.48E-06 |
| METTL21B | -0.355570605 | 2.885594924 | -4.879124658 | 1.48E-06 |
| RPSAP9 | 0.256835983 | 3.738854044 | 4.879120581 | 1.48E-06 |
| CTC-471F3.5 | -0.389639063 | 2.443842745 | -4.878355143 | 1.49E-06 |
| EXT2 | -0.239315352 | 5.480249155 | -4.877422445 | 1.49E-06 |
| RP11-104O19.2 | -0.598404517 | -1.237963304 | -4.877078731 | 1.49E-06 |
| DIS3L | -0.193972795 | 5.357713336 | -4.876925537 | 1.50E-06 |
| NBPF12 | 0.201295467 | 3.193634887 | 4.876139658 | 1.50E-06 |
| GPR108 | -0.183934262 | 6.272649026 | -4.876099861 | 1.50E-06 |
| RNU6-652P | 0.451743837 | 1.40367083 | 4.875556031 | 1.51E-06 |
| RP11-379B18.5 | -0.666717894 | -1.266362303 | -4.875271291 | 1.51E-06 |
| RGPD4 | 0.551014008 | -0.025604856 | 4.874950364 | 1.51E-06 |
| XRCC6P1 | 0.308128283 | 2.208611869 | 4.87406854 | 1.52E-06 |
| CTR9 | -0.242537973 | 6.014630967 | -4.87340255 | 1.52E-06 |
| POLR3E | 0.242877733 | 4.834172492 | 4.873391156 | 1.52E-06 |
| TAF9 | 0.186163274 | 6.464620645 | 4.873325389 | 1.52E-06 |
| RP4-728D4.2 | -0.385971817 | 4.92458289 | -4.873202748 | 1.52E-06 |
| ZC3H7B | -0.211637415 | 5.775173102 | -4.872844087 | 1.53E-06 |
| IFT20 | 0.301935736 | 5.173445086 | 4.87245838 | 1.53E-06 |
| TRAV23DV6 | -0.674460431 | -0.340653676 | -4.87212422 | 1.53E-06 |
| BRF1 | 0.247579082 | 4.239585137 | 4.871826472 | 1.53E-06 |
| ATF7IP2 | 0.439018445 | 4.609166464 | 4.871640595 | 1.53E-06 |
| CTD-3064M3.3 | -0.749997774 | -2.302026144 | -4.870772817 | 1.54E-06 |
| CAPN10 | -0.147704291 | 4.248787296 | -4.869037932 | 1.55E-06 |
| PTRH2 | -0.270560541 | 3.655296325 | -4.867256758 | 1.57E-06 |
| KLHL42 | -0.22282839 | 4.634096772 | -4.866060551 | 1.58E-06 |
| AL161645.2 | -0.737659081 | -0.374435682 | -4.865569528 | 1.58E-06 |
| CXADR | 0.97234548 | -2.438882756 | 4.865329479 | 1.58E-06 |
| PPP1R3E | -0.301521217 | 4.121613698 | -4.86531938 | 1.58E-06 |
| SYNJ2 | 0.518147963 | 3.81825834 | 4.865196502 | 1.58E-06 |
| SEC11C | 0.328017926 | 4.815626306 | 4.865150257 | 1.58E-06 |
| CTD-2201E18.5 | -0.558586799 | 0.535738018 | -4.864606404 | 1.59E-06 |
| MEF2BNB | -0.294032478 | 2.944917518 | -4.864521688 | 1.59E-06 |
| PLAC8 | -0.433094215 | 7.309758568 | -4.864018838 | 1.59E-06 |
| P2RY11 | 0.375236584 | 4.168847915 | 4.862132807 | 1.61E-06 |
| SOWAHC | 0.951504012 | -1.229500481 | 4.861964549 | 1.61E-06 |
| L3MBTL2 | -0.180436009 | 4.587024297 | -4.861942556 | 1.61E-06 |
| RP11-83A24.1 | -0.548305886 | -1.115662393 | -4.861572819 | 1.61E-06 |
| SIGLEC22P | -0.749272249 | 4.152340502 | -4.861276611 | 1.61E-06 |
| CCDC71 | -0.309277099 | 5.3460844 | -4.859336757 | 1.63E-06 |
| RP11-516A11.3 | 0.301277541 | 2.162652016 | 4.859141681 | 1.63E-06 |
| ZFAND3 | 0.209165746 | 6.969679119 | 4.859093749 | 1.63E-06 |
| AC010731.6 | -0.926822648 | 0.474571218 | -4.857822359 | 1.64E-06 |
| TTC21A | -0.393133633 | 1.192131589 | -4.857443912 | 1.64E-06 |
| ARAP2 | 0.512546359 | 4.484948175 | 4.857131937 | 1.64E-06 |
| KHSRP | 0.188147571 | 7.7353914 | 4.857041876 | 1.65E-06 |
| NSUN4 | 0.181250226 | 4.330116821 | 4.856857372 | 1.65E-06 |
| BRMS1 | 0.140413039 | 6.356111678 | 4.856630118 | 1.65E-06 |
| DHRS13 | 0.32674365 | 4.245388198 | 4.854609064 | 1.67E-06 |
| RNU6-101P | 0.616622384 | 0.859348843 | 4.854516615 | 1.67E-06 |
| GTF3C3 | -0.156938923 | 5.229558798 | -4.854457347 | 1.67E-06 |
| POLRMT | -0.17678684 | 4.938192631 | -4.854211395 | 1.67E-06 |
| CEP85L | 0.474163234 | 3.812758932 | 4.852755095 | 1.68E-06 |
| SIK1 | 0.839147031 | 5.713884886 | 4.852188145 | 1.68E-06 |
| SNORA81 | 0.455077272 | 1.405731665 | 4.851811823 | 1.69E-06 |
| RP11-708J19.1 | -0.389485437 | 2.276099524 | -4.851773273 | 1.69E-06 |
| RP11-181C21.4 | -0.288093291 | 5.262409584 | -4.848712445 | 1.71E-06 |
| P2RY12 | -0.915432681 | -0.120260911 | -4.848494451 | 1.71E-06 |
| RP4-742C19.12 | 0.825939616 | 1.131535024 | 4.848016203 | 1.72E-06 |
| AEBP2 | 0.177757935 | 5.133346036 | 4.847229738 | 1.72E-06 |
| TRDMT1 | -0.207769954 | 2.472153562 | -4.846877889 | 1.73E-06 |
| ARFRP1 | -0.222437807 | 3.951306639 | -4.845817883 | 1.74E-06 |
| KCTD21-AS1 | -0.627136407 | 0.057503602 | -4.84573559 | 1.74E-06 |
| RPL36A | 0.360422 | 3.212586878 | 4.845106852 | 1.74E-06 |
| TRGV9 | -0.869762806 | 2.185919237 | -4.844560906 | 1.75E-06 |
| PDK3 | 0.296211078 | 4.735801117 | 4.844107391 | 1.75E-06 |
| ANKRD26 | -0.259982174 | 3.145492266 | -4.84366215 | 1.75E-06 |
| MOAP1 | 0.308375339 | 6.692583988 | 4.843595871 | 1.76E-06 |
| INHBC | 0.627525727 | -0.835240603 | 4.841849218 | 1.77E-06 |
| PPARGC1B | -0.435697267 | 3.111495371 | -4.841065456 | 1.78E-06 |
| DSC1 | -0.679400422 | -2.648955906 | -4.840877862 | 1.78E-06 |
| BMP2 | 1.090300544 | 0.218675861 | 4.84064182 | 1.78E-06 |
| MCCC1 | -0.235996058 | 5.396487011 | -4.839872974 | 1.79E-06 |
| VDAC1P8 | 0.5365868 | 0.677917135 | 4.838331905 | 1.80E-06 |
| AC006116.19 | -0.542897589 | 0.163870758 | -4.837754249 | 1.80E-06 |
| RP11-717D12.1 | 0.572173096 | 0.905285577 | 4.837399765 | 1.81E-06 |
| RP3-477M7.5 | -0.734506796 | 0.600408716 | -4.83627913 | 1.82E-06 |
| HMGN5 | 0.547138118 | 3.68299272 | 4.835983159 | 1.82E-06 |
| ZNF701 | -0.297727806 | 3.292793931 | -4.835830079 | 1.82E-06 |
| TOM1 | 0.378290687 | 5.550298417 | 4.83508473 | 1.83E-06 |
| SPIRE2 | -0.564580808 | -1.486240343 | -4.832558625 | 1.85E-06 |
| KB-1836B5.4 | -0.615412281 | -0.581012834 | -4.832322508 | 1.85E-06 |
| CD109 | 1.00437137 | 3.238701407 | 4.832280109 | 1.85E-06 |
| SLC4A10 | -0.778748977 | -2.416983596 | -4.831213313 | 1.86E-06 |
| SRRM1P3 | 0.417842927 | -0.741934666 | 4.830970527 | 1.86E-06 |
| HIST2H2BC | -0.510508471 | 3.249004533 | -4.830130982 | 1.87E-06 |
| TBC1D22B | 0.204937615 | 5.579795467 | 4.829099641 | 1.88E-06 |
| FTH1P2 | 0.451188347 | 9.582131604 | 4.825869457 | 1.91E-06 |
| AC069278.4 | -0.456785526 | 0.852429622 | -4.825520863 | 1.91E-06 |
| RP11-324I22.3 | 0.577782998 | 0.794090805 | 4.824999632 | 1.92E-06 |
| GPR97 | 0.916102084 | 5.545794458 | 4.824388243 | 1.92E-06 |
| EPSTI1 | 0.787532917 | 3.928681545 | 4.824066047 | 1.93E-06 |
| ELF4 | 0.286597898 | 7.596601574 | 4.823952823 | 1.93E-06 |
| KRT10 | 0.281463678 | 4.723801077 | 4.823923075 | 1.93E-06 |
| CEACAMP3 | 0.784909875 | -1.230877687 | 4.823849695 | 1.93E-06 |
| HNRNPA1P41 | 0.340845627 | 2.727379417 | 4.823215169 | 1.93E-06 |
| ARL13B | -0.26889144 | 2.909915359 | -4.822982548 | 1.94E-06 |
| GLUD1 | -0.216788594 | 7.165234392 | -4.821621652 | 1.95E-06 |
| AGFG2 | -0.413249264 | 2.492432191 | -4.821389022 | 1.95E-06 |
| ATIC | -0.287038438 | 6.285958645 | -4.819230914 | 1.97E-06 |
| GPR34 | -0.992026058 | 1.764166399 | -4.819096703 | 1.97E-06 |
| ZNF648 | -0.742113032 | -3.082218778 | -4.818933647 | 1.97E-06 |
| RPA2 | -0.306768019 | 7.093309603 | -4.818673949 | 1.98E-06 |
| PRCC | 0.139294819 | 6.14017673 | 4.81782801 | 1.99E-06 |
| RP11-446E9.2 | -0.434585708 | 2.346682013 | -4.817174374 | 1.99E-06 |
| NAGA | -0.476785803 | 6.338449817 | -4.816750157 | 2.00E-06 |
| C1orf35 | 0.195810912 | 3.803015645 | 4.816661708 | 2.00E-06 |
| WDR81 | -0.253075286 | 5.437996735 | -4.816390046 | 2.00E-06 |
| SMIM2-AS1 | -0.625480443 | -0.669040858 | -4.816212684 | 2.00E-06 |
| RP13-228J13.1 | -0.528138977 | -1.198352612 | -4.815987883 | 2.00E-06 |
| AF127936.5 | -0.674606321 | -0.563468501 | -4.815805832 | 2.00E-06 |
| TPRG1-AS1 | -1.002544071 | -0.914779084 | -4.814945028 | 2.01E-06 |
| ZNF627 | 0.350671364 | 3.567228748 | 4.814877633 | 2.01E-06 |
| SEC61G | 0.213187537 | 5.257651077 | 4.814859607 | 2.01E-06 |
| RP3-476K8.3 | -0.464415156 | 1.626623372 | -4.814608308 | 2.02E-06 |
| KAZALD1 | 0.738286583 | -0.853397261 | 4.813621898 | 2.03E-06 |
| CHORDC1 | 0.214713449 | 4.355370328 | 4.812781865 | 2.03E-06 |
| HSPA9P1 | 0.27012388 | 3.828665349 | 4.810063224 | 2.06E-06 |
| TRAF4 | 0.570801261 | 4.741435268 | 4.809824036 | 2.06E-06 |
| RNASEH2B | -0.258968059 | 6.911736 | -4.809138436 | 2.07E-06 |
| ARMCX2 | -0.651098123 | 0.423302963 | -4.808331789 | 2.08E-06 |
| CCDC68 | 0.774852181 | -2.3119792 | 4.808298835 | 2.08E-06 |
| TMEM30B | 0.733485571 | -0.34016594 | 4.808051611 | 2.08E-06 |
| BOLA3-DT | -0.542345194 | 1.939776345 | -4.807965162 | 2.08E-06 |
| VHL | 0.165507209 | 6.61646618 | 4.807893576 | 2.08E-06 |
| EXOC8 | 0.318185918 | 5.175274668 | 4.807490454 | 2.09E-06 |
| RP11-532F6.3 | -0.832968645 | -0.08331753 | -4.807480679 | 2.09E-06 |
| CAPRIN1 | -0.251446051 | 7.987156121 | -4.806471109 | 2.10E-06 |
| DDX6P2 | 0.391551461 | 1.00039905 | 4.806156528 | 2.10E-06 |
| ACO1 | -0.287841813 | 3.528270046 | -4.805806312 | 2.10E-06 |
| FAM227B | -0.453065167 | 0.384971354 | -4.805670627 | 2.10E-06 |
| EGR3 | 1.071467742 | 2.716151045 | 4.805342542 | 2.11E-06 |
| RNF150 | 1.209674091 | -2.754582579 | 4.802881074 | 2.13E-06 |
| RP11-473M20.14 | -0.286351522 | 3.188279655 | -4.80249804 | 2.14E-06 |
| RP11-386G11.10 | 0.508480327 | 1.926129333 | 4.802380509 | 2.14E-06 |
| MBD4 | -0.144947593 | 6.481263002 | -4.801802785 | 2.14E-06 |
| NUB1 | -0.191582223 | 6.211151445 | -4.801787046 | 2.14E-06 |
| ACO2 | -0.175843271 | 6.82956448 | -4.801461325 | 2.15E-06 |
| DLL1 | 0.827775879 | 1.158952662 | 4.801460803 | 2.15E-06 |
| TUBGCP4 | -0.289388058 | 3.724979493 | -4.800752185 | 2.15E-06 |
| ORC5 | -0.274474193 | 3.571648864 | -4.800348688 | 2.16E-06 |
| RP11-390P2.4 | -0.566575028 | 2.020822598 | -4.800254562 | 2.16E-06 |
| DDX47 | 0.467109011 | 2.574181153 | 4.798642958 | 2.17E-06 |
| KB-226F1.1 | -0.685606892 | -0.613532126 | -4.798289603 | 2.18E-06 |
| CPEB2 | 0.539705166 | 4.584366347 | 4.798083847 | 2.18E-06 |
| IRF2BPL | 0.359133202 | 5.636374733 | 4.797947243 | 2.18E-06 |
| JAK2 | -0.265861457 | 6.248014743 | -4.796385747 | 2.20E-06 |
| ADCY3 | 0.474263626 | 5.083780302 | 4.795886666 | 2.20E-06 |
| RP11-84C10.2 | -1.029505968 | 5.675869257 | -4.795587205 | 2.21E-06 |
| CEP192 | -0.162011739 | 5.104585525 | -4.795424603 | 2.21E-06 |
| DIAPH1 | -0.26419074 | 8.183406697 | -4.795009914 | 2.21E-06 |
| C17orf107 | 0.764133643 | 0.799102758 | 4.794936358 | 2.21E-06 |
| CARM1 | 0.154440136 | 5.619052497 | 4.794221425 | 2.22E-06 |
| PGBD3 | 0.505855536 | -1.777313969 | 4.793674768 | 2.23E-06 |
| ITCH | 0.15150607 | 6.157266061 | 4.793661696 | 2.23E-06 |
| RP3-462E2.3 | -0.789421015 | 1.037815795 | -4.793285786 | 2.23E-06 |
| GARNL3 | -0.627747173 | -1.261743349 | -4.792789098 | 2.24E-06 |
| ZNF586 | 0.33419694 | 5.21724114 | 4.792303135 | 2.24E-06 |
| RAC2 | -0.327475516 | 8.786168883 | -4.792080799 | 2.24E-06 |
| EBP | 0.190757438 | 5.779908742 | 4.791681399 | 2.25E-06 |
| RP5-1092A3.4 | -0.493863463 | 1.980571898 | -4.790967262 | 2.26E-06 |
| PPAN | 0.338047118 | 2.265258259 | 4.790638291 | 2.26E-06 |
| RP11-187O7.1 | 0.387240597 | 1.714292828 | 4.790563281 | 2.26E-06 |
| APLN | 0.888616148 | -1.027930723 | 4.789812034 | 2.27E-06 |
| RPL12P38 | 0.388110198 | 2.863690199 | 4.787900211 | 2.29E-06 |
| RP11-574K11.31 | 0.44931188 | -0.284266581 | 4.787794338 | 2.29E-06 |
| RP11-641A6.5 | -0.431904284 | -0.504971918 | -4.787617258 | 2.29E-06 |
| AAMDC | -0.261092923 | 3.558348886 | -4.787517491 | 2.29E-06 |
| NUDT2 | -0.212110811 | 5.462070758 | -4.786960872 | 2.30E-06 |
| SPRYD3 | -0.303673978 | 4.901699306 | -4.786584414 | 2.30E-06 |
| RLN1 | -0.548474384 | -1.258914677 | -4.786284333 | 2.31E-06 |
| CTA-363E19.2 | -0.396030251 | 2.578412865 | -4.786160865 | 2.31E-06 |
| TAMM41 | -0.194687776 | 2.852499866 | -4.785802257 | 2.31E-06 |
| ZNF416 | 0.357798741 | 2.972767551 | 4.785588693 | 2.31E-06 |
| FCAR | 1.118129528 | 5.161277323 | 4.785255659 | 2.32E-06 |
| TTYH2 | -0.402904565 | 3.98137402 | -4.785229619 | 2.32E-06 |
| RP11-87H9.2 | -0.320169088 | 3.614168093 | -4.784403551 | 2.33E-06 |
| AC064850.4 | -0.461006046 | -0.348241281 | -4.783924511 | 2.33E-06 |
| ZNF398 | 0.20819351 | 4.395158644 | 4.783482627 | 2.34E-06 |
| GLUD1P2 | -0.725012552 | -1.302996556 | -4.782992692 | 2.34E-06 |
| MAP2K5 | -0.175711805 | 4.692418676 | -4.781711923 | 2.36E-06 |
| RP4-583P15.15 | -0.500994059 | -1.290016562 | -4.781446847 | 2.36E-06 |
| TNFRSF21 | 0.793212214 | 4.059012929 | 4.781252457 | 2.36E-06 |
| MIR4292 | 0.470619309 | 2.507186522 | 4.780645735 | 2.37E-06 |
| RP11-423P10.2 | -0.349571749 | 0.667887784 | -4.779972079 | 2.38E-06 |
| AC022210.2 | 0.259244083 | 3.494023255 | 4.779329176 | 2.38E-06 |
| RP11-129K12.4 | -0.473936408 | 0.596358382 | -4.779237789 | 2.38E-06 |
| IMMT | -0.172919532 | 6.700870464 | -4.778376629 | 2.39E-06 |
| CTSA | -0.405883898 | 7.836785992 | -4.778189981 | 2.40E-06 |
| RP5-1041C10.3 | 0.357139913 | 1.713252248 | 4.777773834 | 2.40E-06 |
| RP11-488L18.1 | -0.558247979 | -0.884195492 | -4.777688716 | 2.40E-06 |
| SCAMP3 | -0.168767509 | 6.4556252 | -4.777280502 | 2.41E-06 |
| CTC-277H1.7 | 0.59299646 | -1.325348011 | 4.777237159 | 2.41E-06 |
| BRK1 | -0.172021812 | 8.567920667 | -4.776866867 | 2.41E-06 |
| LL09NC01-251B2.3 | -0.491339467 | 0.113452691 | -4.776321747 | 2.42E-06 |
| RAD23A | 0.178084402 | 7.725099478 | 4.775265401 | 2.43E-06 |
| MGAT4A | 0.417736979 | 4.20542895 | 4.775169573 | 2.43E-06 |
| MAF1 | 0.166068344 | 8.218379107 | 4.77412772 | 2.44E-06 |
| PLAUR | 0.698500167 | 6.426074279 | 4.773902273 | 2.45E-06 |
| RP11-223C24.1 | -0.603208421 | -1.011855622 | -4.773865367 | 2.45E-06 |
| TVP23C | 0.440282814 | 2.060495966 | 4.772603803 | 2.46E-06 |
| MXRA8 | 0.785178646 | -1.411861564 | 4.771789345 | 2.47E-06 |
| TK2 | -0.326180667 | 3.948094643 | -4.769805602 | 2.49E-06 |
| AC006272.1 | -0.600888653 | 0.088688968 | -4.769361947 | 2.50E-06 |
| RP11-430C7.4 | -0.576331661 | 2.097035521 | -4.769035569 | 2.50E-06 |
| ARHGEF12 | 0.702785722 | 3.274235656 | 4.768766624 | 2.51E-06 |
| DDX56 | -0.186919697 | 5.421750849 | -4.768457075 | 2.51E-06 |
| POU5F1P6 | -0.707318325 | 0.110132211 | -4.768320306 | 2.51E-06 |
| TLR9 | -0.558870714 | -2.649223933 | -4.767736644 | 2.52E-06 |
| CEP290 | -0.243270993 | 4.511315604 | -4.767546072 | 2.52E-06 |
| RP11-1055B8.7 | -0.836193096 | 6.216277738 | -4.766880085 | 2.53E-06 |
| TBC1D7 | 0.277816582 | 2.518800281 | 4.766870043 | 2.53E-06 |
| TRAV1-2 | -0.667196116 | -0.344315265 | -4.766463121 | 2.53E-06 |
| LRCH4 | -0.180390595 | 6.758682227 | -4.765924932 | 2.54E-06 |
| MT-TG | 0.60151651 | 2.520476284 | 4.765790338 | 2.54E-06 |
| RP11-527J8.1 | 0.329967918 | 2.648215909 | 4.764100722 | 2.56E-06 |
| RN7SL800P | 0.495719148 | 1.13590103 | 4.763430782 | 2.57E-06 |
| HNRNPU | -0.198196804 | 8.800713607 | -4.763308625 | 2.57E-06 |
| FAM212A | -0.647005313 | 5.044194074 | -4.763162499 | 2.57E-06 |
| NDUFS3 | 0.193453577 | 5.623162125 | 4.762478939 | 2.58E-06 |
| GRAMD4 | -0.305376614 | 6.016056694 | -4.761103953 | 2.60E-06 |
| RP3-465N24.6 | 0.443858703 | 0.496412774 | 4.76037785 | 2.61E-06 |
| ELP6 | -0.207964438 | 4.3443571 | -4.760198333 | 2.61E-06 |
| AFTPH | -0.204819913 | 6.287156339 | -4.759262405 | 2.62E-06 |
| HSPD1 | -0.330525769 | 7.521602048 | -4.75921882 | 2.62E-06 |
| CCDC146 | -0.34756177 | 2.782685293 | -4.759089128 | 2.62E-06 |
| RP11-890B15.2 | 0.567585787 | -1.289058404 | 4.758378382 | 2.63E-06 |
| ITPR1 | -0.294900117 | 5.443113572 | -4.757320781 | 2.64E-06 |
| THY1 | 0.95026484 | -3.352798013 | 4.757285982 | 2.64E-06 |
| RP11-345P4.9 | 0.260572116 | 4.317862297 | 4.757219724 | 2.65E-06 |
| HAMP | 0.642335403 | -0.914881742 | 4.75706963 | 2.65E-06 |
| AGAP6 | -0.237558049 | 4.665206647 | -4.757055275 | 2.65E-06 |
| ZGPAT | 0.219891112 | 3.989269316 | 4.756319025 | 2.66E-06 |
| C12orf76 | -0.273805843 | 3.419169474 | -4.755853998 | 2.66E-06 |
| GDF11 | -0.66222742 | 4.202646245 | -4.755057924 | 2.67E-06 |
| MTM1 | -0.183734092 | 5.508558329 | -4.753577853 | 2.69E-06 |
| PRAM1 | -0.485175563 | 7.450602361 | -4.753575519 | 2.69E-06 |
| TNK2-AS1 | 0.5342748 | 2.070762681 | 4.753401217 | 2.69E-06 |
| RP11-613G13.1 | -0.516596347 | -0.300272863 | -4.753180885 | 2.70E-06 |
| GPN2 | 0.162531001 | 5.155197376 | 4.753159855 | 2.70E-06 |
| MAP1LC3B | 0.379743593 | 7.181085061 | 4.752863766 | 2.70E-06 |
| ZBTB33 | 0.266688394 | 5.97675449 | 4.752433725 | 2.71E-06 |
| PABPC4 | 0.226452285 | 7.284488431 | 4.752126724 | 2.71E-06 |
| HIST1H2AM | -0.782536609 | 0.363031646 | -4.752093774 | 2.71E-06 |
| SNORD89 | 0.482227908 | 3.749445349 | 4.751543242 | 2.72E-06 |
| RP11-56G10.2 | 0.496023811 | 0.297299461 | 4.751518352 | 2.72E-06 |
| SKIV2L2 | -0.180587827 | 5.654738254 | -4.751485109 | 2.72E-06 |
| AC138744.2 | -0.573922335 | 0.19955448 | -4.750087602 | 2.74E-06 |
| ENPP4 | 0.62010829 | 4.751523879 | 4.749669572 | 2.74E-06 |
| RNF7 | 0.243127881 | 5.663192799 | 4.749606379 | 2.74E-06 |
| TEX22 | -0.537004276 | 1.487925994 | -4.748823409 | 2.75E-06 |
| ZNF112 | -0.878355087 | -0.941382437 | -4.747759446 | 2.77E-06 |
| FAM53C | 0.356958284 | 6.677651086 | 4.747608095 | 2.77E-06 |
| CIR1P2 | 0.477933461 | 0.157384033 | 4.747456035 | 2.77E-06 |
| RP11-417O11.5 | 0.580274686 | 1.277692645 | 4.74672442 | 2.78E-06 |
| CLCN3 | -0.381707098 | 4.287576039 | -4.746043538 | 2.79E-06 |
| ISCA1P6 | 0.433602633 | 1.271835372 | 4.745935212 | 2.79E-06 |
| TRDV1 | -0.715684178 | -0.164325792 | -4.74550933 | 2.80E-06 |
| IL1A | 0.875355936 | -1.804695166 | 4.745373252 | 2.80E-06 |
| USP41 | -0.788422027 | 0.030854387 | -4.745012764 | 2.80E-06 |
| RP11-340I6.6 | 0.191443464 | 4.92023614 | 4.744734395 | 2.81E-06 |
| RAB20 | 0.576474349 | 5.044767017 | 4.743756197 | 2.82E-06 |
| CTD-3094K11.1 | -0.445579935 | 2.268753186 | -4.742776628 | 2.83E-06 |
| KIF15 | 0.655063315 | 3.134002855 | 4.7421889 | 2.84E-06 |
| DNAJA1P3 | -0.365398855 | 2.288739446 | -4.74184145 | 2.84E-06 |
| GNRHR2 | -0.425230202 | -0.378308925 | -4.739964034 | 2.87E-06 |
| RP11-383I23.2 | -0.575922659 | -2.562945943 | -4.7395724 | 2.87E-06 |
| ACTG1P3 | 0.433021181 | 2.379059594 | 4.739125428 | 2.88E-06 |
| CDC20 | 0.737139099 | 4.330661168 | 4.738954287 | 2.88E-06 |
| LIPA | -0.350356142 | 6.781915163 | -4.738806189 | 2.89E-06 |
| GPATCH4 | -0.304824367 | 4.63042499 | -4.737944888 | 2.90E-06 |
| DHRS4L2 | -0.230239305 | 4.504818391 | -4.737104964 | 2.91E-06 |
| BACE1 | -0.542982693 | 4.328560276 | -4.73603763 | 2.92E-06 |
| ZC3H8 | -0.20939981 | 3.83914375 | -4.735193411 | 2.93E-06 |
| RPL21P39 | 0.264281634 | 3.093546355 | 4.735003963 | 2.94E-06 |
| ZMAT3 | -0.422495279 | 4.80232419 | -4.734510171 | 2.94E-06 |
| RP11-435O5.5 | -0.664806658 | -2.039825883 | -4.733831489 | 2.95E-06 |
| RN7SL181P | -0.497075269 | 0.738802085 | -4.733377445 | 2.96E-06 |
| PPM1B | 0.169363689 | 5.557406235 | 4.732744754 | 2.97E-06 |
| THYN1 | -0.20707674 | 6.16247996 | -4.732112273 | 2.98E-06 |
| TRBV8-2 | -0.738428784 | 0.26560258 | -4.73156341 | 2.99E-06 |
| RP11-682B13.2 | 0.472657109 | 0.960748088 | 4.730804056 | 3.00E-06 |
| AC079922.3 | -0.360956661 | 2.329046796 | -4.730469514 | 3.00E-06 |
| HIST1H4B | -0.646339927 | -0.587388249 | -4.730460022 | 3.00E-06 |
| IRF4 | 0.758198133 | 2.42663345 | 4.730085107 | 3.01E-06 |
| SLC6A12 | -0.979273761 | -0.770386539 | -4.72908309 | 3.02E-06 |
| RP11-458D21.1 | -0.619985037 | -0.950837496 | -4.729019648 | 3.02E-06 |
| ZNF695 | 0.645039612 | 0.024189053 | 4.728950345 | 3.02E-06 |
| HMGN2P28 | -0.438915352 | 1.012075411 | -4.728887253 | 3.02E-06 |
| TNC | 1.24162495 | -3.499383589 | 4.728213341 | 3.03E-06 |
| MRPS18C | -0.226794644 | 3.081697551 | -4.72813398 | 3.03E-06 |
| MRFAP1L1 | -0.350128659 | 6.263760392 | -4.726569331 | 3.06E-06 |
| RP11-395I6.3 | -0.472664818 | 2.741983816 | -4.726540176 | 3.06E-06 |
| RP11-875O11.3 | -0.768779059 | 0.513213941 | -4.726335826 | 3.06E-06 |
| SAMD4B | 0.155726399 | 5.570496678 | 4.725576142 | 3.07E-06 |
| UACA | -0.819899106 | 1.083404686 | -4.72494605 | 3.08E-06 |
| RP11-540O11.1 | 0.460065508 | 0.210289768 | 4.72482019 | 3.08E-06 |
| HIST2H2BD | -0.568048034 | 1.93425163 | -4.723779888 | 3.10E-06 |
| ETAA1 | 0.178858424 | 4.7330187 | 4.723541858 | 3.10E-06 |
| PM20D2 | -0.390675039 | 5.835432585 | -4.723531782 | 3.10E-06 |
| PRPF6 | -0.21032493 | 7.620748953 | -4.723342475 | 3.10E-06 |
| RRP12 | 0.389828367 | 5.587188533 | 4.722502517 | 3.11E-06 |
| KDELR2 | -0.240029252 | 5.937975186 | -4.722214217 | 3.12E-06 |
| PABPC1L | -0.391321642 | 5.012060885 | -4.720875026 | 3.14E-06 |
| RRAGA | -0.202943757 | 6.44978686 | -4.719543861 | 3.16E-06 |
| MAPKAPK3 | -0.224190458 | 8.069712779 | -4.718731723 | 3.17E-06 |
| APPL2 | 0.261842671 | 5.088527886 | 4.71764451 | 3.19E-06 |
| PLEKHH3 | 0.643628668 | 1.755448541 | 4.716965855 | 3.20E-06 |
| BMP6 | 0.881583588 | 1.522141533 | 4.715682857 | 3.22E-06 |
| SDCCAG3P2 | -0.277614043 | 2.278158577 | -4.715364862 | 3.22E-06 |
| FBXO31 | 0.270230465 | 3.648836164 | 4.71478119 | 3.23E-06 |
| RP11-692D12.1 | -0.445764307 | 1.065445361 | -4.71452045 | 3.23E-06 |
| BIRC2 | -0.147403528 | 6.454214036 | -4.713990768 | 3.24E-06 |
| ABCA11P | -0.610655696 | -1.285086983 | -4.713692604 | 3.25E-06 |
| RP11-795F19.5 | -0.656720149 | -1.54537759 | -4.712086174 | 3.27E-06 |
| PTPRE | 0.489498196 | 5.997006761 | 4.711305458 | 3.28E-06 |
| LENG8-AS1 | -0.33556167 | 5.084577421 | -4.710812196 | 3.29E-06 |
| DPP8 | -0.140558351 | 5.156449751 | -4.709875221 | 3.30E-06 |
| CARS2 | -0.177377142 | 5.156725309 | -4.709599084 | 3.31E-06 |
| C9orf163 | -0.560832881 | -1.682609411 | -4.709424136 | 3.31E-06 |
| ERCC2 | -0.257454786 | 4.079598706 | -4.707902124 | 3.33E-06 |
| MARS | -0.144765085 | 6.407176672 | -4.706750883 | 3.35E-06 |
| COL6A2 | 0.90121303 | 2.644762789 | 4.706262981 | 3.36E-06 |
| RP11-488P3.1 | 0.62799787 | -1.107536527 | 4.705919991 | 3.37E-06 |
| LNP1 | -0.46537288 | 1.019936998 | -4.705917807 | 3.37E-06 |
| FANCL | -0.34786627 | 4.129878146 | -4.705399594 | 3.37E-06 |
| RBBP4 | -0.198809728 | 6.235459417 | -4.704814022 | 3.38E-06 |
| FDXACB1 | -0.394354168 | 0.777709527 | -4.704094563 | 3.39E-06 |
| CCDC113 | -0.671934779 | -0.315430493 | -4.704038067 | 3.40E-06 |
| TOX2 | 0.7095229 | 0.265955687 | 4.703947235 | 3.40E-06 |
| ULK1 | 0.306853621 | 6.159100499 | 4.70302477 | 3.41E-06 |
| BEST4 | 0.547622619 | 0.08463359 | 4.702935454 | 3.41E-06 |
| PNPLA6 | -0.250954309 | 5.965802346 | -4.702064539 | 3.43E-06 |
| UBE2I | 0.135181863 | 5.899387862 | 4.7015295 | 3.44E-06 |
| CYB5A | -0.357679862 | 2.616434925 | -4.700802676 | 3.45E-06 |
| RNA5SP395 | 0.581854813 | 0.172000695 | 4.700638429 | 3.45E-06 |
| RP11-981P6.1 | -0.551104691 | 0.015854391 | -4.700390382 | 3.45E-06 |
| CTD-3222D19.5 | 0.588731117 | -0.52387109 | 4.699724561 | 3.46E-06 |
| CRYM | 0.714174048 | -2.73702621 | 4.698967058 | 3.48E-06 |
| COPG2 | -0.30669526 | 5.329712884 | -4.698179831 | 3.49E-06 |
| NUP50 | 0.215680503 | 6.14819307 | 4.698007487 | 3.49E-06 |
| NAA20 | -0.193239176 | 5.930775509 | -4.697938703 | 3.49E-06 |
| AC012493.1 | -0.410734608 | 2.500708404 | -4.697649713 | 3.50E-06 |
| PCMT1 | -0.309732194 | 6.455108238 | -4.697301011 | 3.50E-06 |
| FRMD6 | 0.898379388 | -1.781207458 | 4.697042516 | 3.51E-06 |
| ARHGAP1 | -0.188468706 | 6.43267374 | -4.695818508 | 3.53E-06 |
| SYDE2 | 0.843216705 | -1.343953226 | 4.695563382 | 3.53E-06 |
| DKK2 | -1.118583082 | -2.106190853 | -4.693284296 | 3.57E-06 |
| IL1RL2 | 0.678879224 | -2.475403134 | 4.692807862 | 3.58E-06 |
| RP11-157G21.2 | -0.375831265 | 2.809817619 | -4.691485721 | 3.60E-06 |
| EPOR | 0.617496437 | 3.612435523 | 4.690646201 | 3.61E-06 |
| TRAV22 | -0.649534814 | -0.054558777 | -4.690339484 | 3.62E-06 |
| RBBP4P1 | -0.227569816 | 5.352312853 | -4.690108762 | 3.62E-06 |
| RP11-497H16.8 | -0.561536588 | 0.136807113 | -4.689707288 | 3.63E-06 |
| CASP6 | -0.380285266 | 5.495304589 | -4.689475439 | 3.63E-06 |
| RNF169 | 0.187650953 | 5.774280152 | 4.68913476 | 3.64E-06 |
| RP11-885B4.1 | -0.533855057 | -0.136718464 | -4.688880039 | 3.64E-06 |
| RP11-196I18.3 | -0.308550267 | 3.159371934 | -4.68877175 | 3.65E-06 |
| AC007000.12 | -0.510023009 | -0.011279679 | -4.68856836 | 3.65E-06 |
| PET112 | -0.301511175 | 2.819569812 | -4.688542235 | 3.65E-06 |
| RHOA-IT1 | 0.451572699 | 2.516828879 | 4.688352975 | 3.65E-06 |
| ACAP2 | -0.175831934 | 6.266971269 | -4.68699363 | 3.68E-06 |
| SH2D2A | 0.608315553 | 2.974665829 | 4.686249095 | 3.69E-06 |
| XXbac-BPG249D20.9 | -0.598638596 | 0.793313261 | -4.685675377 | 3.70E-06 |
| RP1-30M3.5 | -0.325590887 | 2.97905276 | -4.685673233 | 3.70E-06 |
| ELAVL1 | -0.208209772 | 5.582956349 | -4.685530135 | 3.70E-06 |
| C17orf49 | 0.436636022 | 3.076841705 | 4.685235897 | 3.71E-06 |
| C17orf85 | -0.191470498 | 5.979996921 | -4.685107753 | 3.71E-06 |
| TBCA | -0.22337756 | 6.581496393 | -4.68461716 | 3.72E-06 |
| RWDD4 | 0.202160751 | 3.783482315 | 4.684168985 | 3.73E-06 |
| TIMM44 | 0.172032713 | 4.592965232 | 4.68410521 | 3.73E-06 |
| SLC6A10P | 0.655483177 | -3.941679137 | 4.683986764 | 3.73E-06 |
| LY6G6C | 0.730029343 | 1.493031501 | 4.683880435 | 3.73E-06 |
| DMXL2 | -0.61255677 | 6.081570383 | -4.683701726 | 3.73E-06 |
| MYCBP | -0.312895213 | 3.70039064 | -4.683497108 | 3.74E-06 |
| GOLGA2P5 | 0.419745906 | 2.266635806 | 4.682594724 | 3.75E-06 |
| SRSF5 | -0.198165007 | 9.147290177 | -4.681259184 | 3.78E-06 |
| LRRC46 | -0.427540741 | 0.876796812 | -4.681060306 | 3.78E-06 |
| ZMYND15 | 0.930939072 | 0.946328006 | 4.680763204 | 3.78E-06 |
| NOP14-AS1 | 0.242291624 | 2.882746056 | 4.680158634 | 3.80E-06 |
| RP11-621H8.2 | 0.382334053 | 3.359422622 | 4.679703056 | 3.80E-06 |
| ASF1A | -0.336616455 | 5.203875591 | -4.679500971 | 3.81E-06 |
| RNU6-350P | 0.793287919 | -0.01932504 | 4.679164955 | 3.81E-06 |
| TTC16 | -0.733976549 | -0.471674032 | -4.678904374 | 3.82E-06 |
| RP11-166B2.8 | -0.468393693 | -0.113060444 | -4.678420953 | 3.83E-06 |
| RP11-12M9.4 | -0.46833553 | -0.212756363 | -4.678329115 | 3.83E-06 |
| BTG2-DT | 0.58372956 | -0.544752874 | 4.676937784 | 3.85E-06 |
| TNNT3 | 0.770555728 | 2.956098884 | 4.676852992 | 3.85E-06 |
| VPS52 | -0.134835785 | 6.0192048 | -4.675708674 | 3.87E-06 |
| HCLS1 | -0.30052818 | 8.284275433 | -4.675654045 | 3.88E-06 |
| APOA1 | 0.506986589 | 0.220833026 | 4.675133721 | 3.89E-06 |
| DTX2 | -0.315239239 | 3.452211676 | -4.67490149 | 3.89E-06 |
| ACD | -0.205734352 | 4.79991476 | -4.67437326 | 3.90E-06 |
| CENPT | -0.195171117 | 5.645507748 | -4.673962447 | 3.91E-06 |
| ALS2 | -0.3948451 | 4.467582539 | -4.673015809 | 3.92E-06 |
| RP11-213G2.3 | -0.307550394 | 4.28573793 | -4.672906851 | 3.93E-06 |
| MBTPS2 | -0.242475589 | 2.641985227 | -4.672504388 | 3.93E-06 |
| SLC2A9 | -0.629262621 | 2.399736594 | -4.671980823 | 3.94E-06 |
| APOC1 | 1.134081972 | 1.014117819 | 4.67085129 | 3.96E-06 |
| RP1-266L20.4 | -0.495652275 | 1.23337364 | -4.670474093 | 3.97E-06 |
| TRIP12 | -0.120065756 | 6.87319246 | -4.670180958 | 3.98E-06 |
| AC007000.10 | -0.566164482 | -0.161795188 | -4.669818535 | 3.98E-06 |
| EIF4EBP2 | -0.180468093 | 7.539257008 | -4.669804286 | 3.98E-06 |
| HDAC10 | -0.294280504 | 1.452985842 | -4.669199921 | 3.99E-06 |
| C11orf48 | -0.210747448 | 4.576416417 | -4.668520447 | 4.01E-06 |
| VDAC3P1 | -0.320161717 | 3.217753477 | -4.667231967 | 4.03E-06 |
| RRN3P2 | 0.291096458 | 4.483115094 | 4.667108538 | 4.03E-06 |
| TBC1D3 | -0.523239678 | -2.593001702 | -4.666887299 | 4.04E-06 |
| MYH10 | 0.998511611 | 1.15895727 | 4.666775638 | 4.04E-06 |
| LINC01059 | 0.580677525 | -1.668471038 | 4.666652788 | 4.04E-06 |
| SEMA4B | -0.433492204 | 3.652936365 | -4.665797998 | 4.06E-06 |
| CBR3-AS1 | -0.41941425 | -0.030564337 | -4.665615803 | 4.06E-06 |
| MXD1 | 0.519587697 | 7.163136184 | 4.664553758 | 4.08E-06 |
| MCM5 | 0.306258709 | 6.612503038 | 4.664043536 | 4.09E-06 |
| WDR53 | 0.15828071 | 5.003193145 | 4.663885459 | 4.09E-06 |
| DDIT3 | 0.45754329 | 7.109099054 | 4.662617995 | 4.12E-06 |
| ZNF37A | -0.212459383 | 4.365921495 | -4.661899775 | 4.13E-06 |
| EPB41L4B | 0.640819905 | -4.705688288 | 4.661796627 | 4.13E-06 |
| RP11-74E24.2 | 0.186753743 | 4.536744528 | 4.660965896 | 4.15E-06 |
| DPY19L3 | -0.217413657 | 4.609604224 | -4.660703232 | 4.15E-06 |
| LIN37 | -0.281150253 | 2.1043303 | -4.660412053 | 4.16E-06 |
| RP11-47L3.1 | -0.839089776 | 1.517330915 | -4.659829698 | 4.17E-06 |
| CUTC | -0.166592618 | 6.021609869 | -4.658388372 | 4.20E-06 |
| PFKP | -0.409005069 | 6.545472122 | -4.658021559 | 4.21E-06 |
| IFT81 | -0.458383271 | 1.854704735 | -4.65699577 | 4.23E-06 |
| RP11-1E11.1 | -0.65307822 | -1.14180943 | -4.656965606 | 4.23E-06 |
| FAM198B | -0.987271193 | 3.449381872 | -4.656390954 | 4.24E-06 |
| COA1 | 0.178522716 | 3.736414147 | 4.655911649 | 4.25E-06 |
| LINC00467 | -0.395470641 | 3.229323463 | -4.655737809 | 4.25E-06 |
| YWHAH-AS1 | 0.539583087 | -0.241493932 | 4.655240734 | 4.26E-06 |
| SYN1 | 0.855166749 | 0.373555236 | 4.65475644 | 4.27E-06 |
| CIR1P1 | 0.460103568 | -0.26516532 | 4.654664399 | 4.27E-06 |
| PIERCE1 | 0.449735665 | 0.11765031 | 4.653494668 | 4.30E-06 |
| DOLPP1 | -0.285514076 | 4.700924912 | -4.65335417 | 4.30E-06 |
| MYOZ3 | -0.874723326 | 0.430255827 | -4.653327937 | 4.30E-06 |
| PEF1 | 0.164098768 | 6.248790705 | 4.653309481 | 4.30E-06 |
| ZNF493 | -0.351450575 | 3.115835102 | -4.65246101 | 4.32E-06 |
| FZD5 | 0.516129564 | 2.268870029 | 4.652110858 | 4.32E-06 |
| FAM86B2 | -0.56936136 | -1.760195084 | -4.652046085 | 4.32E-06 |
| PPP1R26 | -0.855496674 | 3.230497634 | -4.651970042 | 4.33E-06 |
| PYGM | 0.435798163 | 1.654254269 | 4.651350992 | 4.34E-06 |
| RAB14 | 0.171437445 | 6.948063268 | 4.651188964 | 4.34E-06 |
| RAP2B | 0.278451835 | 6.387506233 | 4.651038204 | 4.34E-06 |
| SPAST | -0.164788344 | 5.20727541 | -4.650457175 | 4.36E-06 |
| CCDC122 | -0.466980243 | 1.392291343 | -4.649355004 | 4.38E-06 |
| MRPS25 | -0.2363884 | 5.572672572 | -4.64850015 | 4.40E-06 |
| MCM10 | 0.645220889 | 3.118347275 | 4.648390242 | 4.40E-06 |
| 3-Mar | 0.517213938 | 1.845830823 | 4.6474604 | 4.42E-06 |
| FAM96A | -0.220280374 | 6.235677561 | -4.647170607 | 4.42E-06 |
| GATSL2 | 0.599630298 | -0.112946004 | 4.64600109 | 4.45E-06 |
| RP11-54O7.18 | 0.770717738 | -1.062066533 | 4.64587673 | 4.45E-06 |
| SEC61B | 0.190712333 | 7.251665552 | 4.645789726 | 4.45E-06 |
| RNU6-593P | 0.559669103 | 0.311588201 | 4.644964982 | 4.47E-06 |
| RP11-409K20.7 | -0.468642299 | 0.325481131 | -4.644719464 | 4.47E-06 |
| NAIP | -0.585552282 | 2.918243512 | -4.644663146 | 4.47E-06 |
| AC022400.2 | -0.452324337 | 0.841503304 | -4.644575731 | 4.48E-06 |
| KCNN4 | -0.363941549 | 5.636369116 | -4.64449316 | 4.48E-06 |
| ID3 | 0.666312934 | 1.189295169 | 4.643565636 | 4.50E-06 |
| UBE2G1 | 0.17141072 | 6.183378277 | 4.64340232 | 4.50E-06 |
| RP11-126F18.2 | -0.437083351 | 0.492756094 | -4.642814965 | 4.51E-06 |
| ZFYVE1 | 0.199223516 | 4.648824222 | 4.642747833 | 4.51E-06 |
| NADSYN1 | -0.168873373 | 5.781290635 | -4.642519878 | 4.52E-06 |
| MRPS16 | -0.164403836 | 5.948093575 | -4.642181216 | 4.53E-06 |
| MTR | -0.215413266 | 5.196012559 | -4.642166471 | 4.53E-06 |
| RP11-15A1.2 | -0.617732411 | -0.765279735 | -4.642162387 | 4.53E-06 |
| ITGAL | -0.49769308 | 7.289023064 | -4.642024835 | 4.53E-06 |
| GFPT2 | 0.713968986 | -2.544483916 | 4.641578045 | 4.54E-06 |
| CDK2AP2P1 | -0.619761583 | -0.384110748 | -4.640546871 | 4.56E-06 |
| PTBP3 | -0.193070162 | 7.949211048 | -4.640090624 | 4.57E-06 |
| KRT8P39 | 0.436375047 | 0.925368366 | 4.639957936 | 4.57E-06 |
| RP11-379K17.12 | 0.606843225 | 2.131251616 | 4.63943018 | 4.58E-06 |
| THAP5 | -0.256073848 | 3.625903999 | -4.639069394 | 4.59E-06 |
| RMDN3 | 0.194667453 | 4.401605885 | 4.638775292 | 4.60E-06 |
| TMEM81 | -0.306308944 | 2.238702427 | -4.637875436 | 4.62E-06 |
| DNAH6 | -0.850320598 | -1.703191971 | -4.637525406 | 4.62E-06 |
| RP11-57G10.8 | -0.457592815 | -0.660622161 | -4.63744214 | 4.63E-06 |
| RP11-53M11.2 | -0.264581169 | 4.554888404 | -4.636860472 | 4.64E-06 |
| SIPA1 | -0.297471195 | 7.595766861 | -4.636454454 | 4.65E-06 |
| RNU6-384P | 0.476723856 | 0.745504469 | 4.636343102 | 4.65E-06 |
| FGD5 | 1.456504992 | -1.405202002 | 4.636328269 | 4.65E-06 |
| SIPA1L1 | 0.436775441 | 4.624755262 | 4.636127559 | 4.65E-06 |
| RP5-1126H10.2 | 0.443522892 | -0.485518198 | 4.634489975 | 4.69E-06 |
| CYP2D8P | 0.338853256 | 1.740940063 | 4.634088861 | 4.70E-06 |
| SMARCAD1 | -0.190525453 | 4.990790106 | -4.633645543 | 4.71E-06 |
| HIST2H4B | -0.69662839 | -0.325895581 | -4.633363217 | 4.71E-06 |
| FTH1P8 | 0.437872072 | 7.228789137 | 4.631274214 | 4.76E-06 |
| CCRN4L | 0.544710972 | 5.797480264 | 4.631151503 | 4.76E-06 |
| AC005042.2 | 0.536662623 | 0.156999183 | 4.630279951 | 4.78E-06 |
| RP11-597D13.7 | -0.38775955 | 1.198699389 | -4.63003494 | 4.79E-06 |
| RP11-285F7.2 | -0.340108507 | 5.438999274 | -4.628344171 | 4.82E-06 |
| RP11-229P13.25 | 0.415541523 | 0.755055485 | 4.628023206 | 4.83E-06 |
| CCDC149 | -0.77657609 | 2.196823141 | -4.626820388 | 4.86E-06 |
| IGFBP4 | 0.829228963 | 2.175365836 | 4.624821604 | 4.90E-06 |
| CTD-2319I12.4 | -0.454335412 | 1.404499675 | -4.624609577 | 4.91E-06 |
| CPSF4 | 0.177145038 | 4.983139037 | 4.623973958 | 4.92E-06 |
| HMGN2P15 | 0.436615031 | 1.416214961 | 4.623849396 | 4.93E-06 |
| RP11-307C12.12 | -0.364327423 | 2.394760877 | -4.623587604 | 4.93E-06 |
| RDH10 | 0.42858563 | 2.123444885 | 4.623558405 | 4.93E-06 |
| RPL7P22 | -0.351130959 | 3.409521831 | -4.622496884 | 4.96E-06 |
| SMKR1 | -0.671365989 | 0.416184826 | -4.62231144 | 4.96E-06 |
| ZNF445 | -0.272675512 | 4.926320942 | -4.622093165 | 4.97E-06 |
| PBK | 0.826606032 | 0.677910501 | 4.621188978 | 4.99E-06 |
| MRPS33 | -0.227407981 | 5.136250598 | -4.620656876 | 5.00E-06 |
| RNASE6 | -0.949756323 | 6.020966828 | -4.620601307 | 5.00E-06 |
| SH3TC2 | -0.879023743 | 0.353508412 | -4.619595512 | 5.02E-06 |
| UBBP4 | 0.383142134 | 2.045820538 | 4.619059825 | 5.04E-06 |
| RP11-56B16.1 | -0.491186193 | -0.83945534 | -4.619053081 | 5.04E-06 |
| TMEM160 | -0.265080597 | 5.630993541 | -4.618633384 | 5.05E-06 |
| AC091633.3 | 0.445847502 | 0.533077591 | 4.617514657 | 5.07E-06 |
| NVL | -0.144171664 | 4.557121784 | -4.616060731 | 5.11E-06 |
| TRAPPC13 | 0.213214964 | 3.519154181 | 4.615875896 | 5.11E-06 |
| MIR4420 | 0.588620555 | 3.083185469 | 4.614974878 | 5.13E-06 |
| GSTK1 | -0.254792326 | 7.866702495 | -4.614877135 | 5.13E-06 |
| POLR3C | -0.171133294 | 5.909126704 | -4.614575683 | 5.14E-06 |
| COQ4 | -0.211193011 | 4.713033303 | -4.614461743 | 5.14E-06 |
| PIGP | -0.279164342 | 2.350158921 | -4.614171138 | 5.15E-06 |
| SUSD2 | 0.473910045 | -0.626753803 | 4.613482 | 5.17E-06 |
| TOR2A | 0.208738009 | 5.057461071 | 4.612589144 | 5.19E-06 |
| CIB2 | 0.563605452 | 1.729536824 | 4.611909383 | 5.20E-06 |
| CTSO | -0.342659163 | 5.47867145 | -4.611317605 | 5.22E-06 |
| FBN1 | 0.770932988 | 1.949284269 | 4.611166812 | 5.22E-06 |
| AOAH | -0.65645815 | 6.598724997 | -4.610519496 | 5.24E-06 |
| ASB6 | 0.181054375 | 5.870693386 | 4.610352039 | 5.24E-06 |
| SLC47A1 | 0.928435582 | -1.244082497 | 4.609887514 | 5.25E-06 |
| AC073115.6 | -0.589998182 | -1.002031275 | -4.608909488 | 5.28E-06 |
| CCT5P1 | -0.408275425 | 1.373142203 | -4.608763251 | 5.28E-06 |
| CREB3L3 | 0.814247898 | -1.240726162 | 4.607353869 | 5.31E-06 |
| P2RY10 | 0.655383847 | 3.231713926 | 4.606787712 | 5.33E-06 |
| FAM47E | -0.74651903 | -1.744884089 | -4.606511103 | 5.33E-06 |
| RP11-110I1.14 | -0.440380324 | 0.553257039 | -4.606452865 | 5.34E-06 |
| AC011841.2 | -0.44558167 | 2.556143137 | -4.605869365 | 5.35E-06 |
| GK5 | 0.317082844 | 3.940718193 | 4.605335684 | 5.36E-06 |
| NUCB1 | -0.178773792 | 7.747774493 | -4.605218708 | 5.37E-06 |
| RP11-95D17.1 | -0.325417275 | 1.87690072 | -4.604609242 | 5.38E-06 |
| IL18RAP | 0.892468439 | 3.816312723 | 4.603035438 | 5.42E-06 |
| RP11-439L18.1 | 0.645397487 | 1.001930287 | 4.602900023 | 5.42E-06 |
| IFIT5 | 0.421748961 | 4.549578276 | 4.602784701 | 5.43E-06 |
| GPBP1L1 | -0.137916496 | 6.726577856 | -4.602618849 | 5.43E-06 |
| LIMA1 | -0.401144488 | 4.008606649 | -4.602004826 | 5.45E-06 |
| MARVELD1 | -0.602700726 | 5.63381238 | -4.601931939 | 5.45E-06 |
| PPP1R13L | -0.379433658 | 1.422388751 | -4.60178068 | 5.45E-06 |
| RPL23P8 | -0.21704922 | 7.097528377 | -4.60026059 | 5.49E-06 |
| MRPL19 | -0.211050828 | 4.510348913 | -4.599968849 | 5.50E-06 |
| MRPL27 | -0.229209023 | 4.164182746 | -4.599329582 | 5.51E-06 |
| TSNAXIP1 | -0.526142369 | 0.069305489 | -4.599154527 | 5.52E-06 |
| MRPL43 | -0.159660039 | 5.192650217 | -4.599042782 | 5.52E-06 |
| FOXP4 | 0.325193023 | 4.04382396 | 4.598816135 | 5.53E-06 |
| RNU5D-1 | -0.705464257 | 3.060405461 | -4.597512537 | 5.56E-06 |
| G0S2 | 1.316699843 | 6.664077341 | 4.597354597 | 5.56E-06 |
| LRFN3 | -0.735096006 | 0.395078704 | -4.596518955 | 5.58E-06 |
| CDKN1B | 0.325031196 | 7.486450469 | 4.596385208 | 5.59E-06 |
| RP11-885B4.2 | -0.553679663 | 0.248826566 | -4.595859818 | 5.60E-06 |
| AGO4 | -0.236251653 | 6.969810117 | -4.595500585 | 5.61E-06 |
| ZNF714 | 0.35774541 | 3.512546134 | 4.595378003 | 5.61E-06 |
| HNRNPA1P35 | 0.253068575 | 4.480425166 | 4.59531968 | 5.61E-06 |
| CTD-2201E18.3 | -0.275757019 | 3.858855953 | -4.594891294 | 5.63E-06 |
| RP1-228H13.2 | -0.652825793 | -0.990086348 | -4.594881751 | 5.63E-06 |
| AC084117.3 | -0.474009305 | 2.060669413 | -4.593641727 | 5.66E-06 |
| RP3-508I15.14 | 0.425252705 | 2.236540964 | 4.592300519 | 5.69E-06 |
| BANF1 | -0.332261624 | 7.258997852 | -4.591000063 | 5.73E-06 |
| C5 | -0.423304587 | 3.621430467 | -4.590775955 | 5.73E-06 |
| UBE2J2 | -0.139988587 | 5.44004065 | -4.58973018 | 5.76E-06 |
| TFEC | -0.536504554 | 5.67450675 | -4.589600154 | 5.76E-06 |
| MGAT5B | 0.822396164 | -2.590579535 | 4.589384857 | 5.77E-06 |
| DCAF13P3 | 0.384789655 | 0.44026112 | 4.58889341 | 5.78E-06 |
| RP13-890H12.2 | 0.565287537 | 1.281259203 | 4.588801481 | 5.79E-06 |
| ZNF197 | -0.257275733 | 3.814137196 | -4.587863723 | 5.81E-06 |
| TBC1D31 | -0.324174585 | 2.650019822 | -4.587671705 | 5.82E-06 |
| FAM211A | 0.567236298 | 1.943445881 | 4.58748526 | 5.82E-06 |
| MOB1B | 0.303286918 | 5.637937188 | 4.587249097 | 5.83E-06 |
| DCTN1 | -0.160465617 | 6.182490968 | -4.586800425 | 5.84E-06 |
| IQSEC1 | 0.500776757 | 5.635232159 | 4.58665467 | 5.84E-06 |
| ZNF833P | 0.501829446 | -2.533183329 | 4.586572231 | 5.84E-06 |
| NMB | 0.33998982 | 3.65099945 | 4.586440128 | 5.85E-06 |
| FOXN3P1 | 0.342970248 | 1.208782327 | 4.586137026 | 5.86E-06 |
| NRBP1 | 0.160996455 | 6.936840033 | 4.586028748 | 5.86E-06 |
| ARHGAP19 | 0.298952255 | 4.574482618 | 4.585682289 | 5.87E-06 |
| POT1-AS1 | -0.512155431 | -2.220681375 | -4.585617564 | 5.87E-06 |
| PIAS4 | -0.214711161 | 4.688569674 | -4.585347537 | 5.88E-06 |
| ERC1 | 0.156683349 | 4.738956163 | 4.584537362 | 5.90E-06 |
| LINC00482 | -1.127983879 | 1.043892911 | -4.584385948 | 5.90E-06 |
| FKRP | 0.177424529 | 3.967071398 | 4.583380309 | 5.93E-06 |
| RP11-234B24.4 | -0.816799996 | -0.907012085 | -4.582392528 | 5.96E-06 |
| ELAC2 | -0.187540984 | 5.67220395 | -4.581726653 | 5.98E-06 |
| TBC1D10B | -0.16021683 | 5.74689909 | -4.58153143 | 5.98E-06 |
| MARCKS | 0.923634805 | 5.807997019 | 4.581199223 | 5.99E-06 |
| SUPT4H1 | 0.142404448 | 7.807892651 | 4.580891115 | 6.00E-06 |
| KIF3C | -0.356971819 | 3.385612825 | -4.580630477 | 6.01E-06 |
| TNNI2 | 0.714039153 | 4.14716545 | 4.580426185 | 6.01E-06 |
| CCT6A | -0.196767502 | 7.446536409 | -4.579940931 | 6.02E-06 |
| SSBP2 | -0.436090534 | 4.66384743 | -4.579681272 | 6.03E-06 |
| BMS1P7 | -0.663560158 | -0.1237552 | -4.578681481 | 6.06E-06 |
| TDRD3 | -0.176197644 | 4.471349977 | -4.578563205 | 6.06E-06 |
| CXorf57 | 0.669187842 | -0.846383766 | 4.57846859 | 6.06E-06 |
| PPM1K | -0.321031653 | 3.713152946 | -4.577997532 | 6.08E-06 |
| SSBP1 | 0.218403801 | 5.842104105 | 4.577948402 | 6.08E-06 |
| RP11-537I16.2 | 0.520585827 | 0.390888329 | 4.577101343 | 6.10E-06 |
| C8orf88 | 0.996771153 | 2.200151632 | 4.576520403 | 6.12E-06 |
| EMC1 | -0.24509271 | 4.896692143 | -4.576119018 | 6.13E-06 |
| LRRFIP2 | 0.219921361 | 5.491716353 | 4.575696739 | 6.14E-06 |
| RP11-337C18.9 | -0.308422548 | 3.158824379 | -4.575494596 | 6.15E-06 |
| CFAP418 | -0.281023364 | 2.493900323 | -4.575124923 | 6.16E-06 |
| RAD21 | 0.162518594 | 7.59042001 | 4.574954498 | 6.16E-06 |
| UBE2Q2 | 0.236582101 | 6.258343729 | 4.574629385 | 6.17E-06 |
| ZSCAN29 | -0.182137666 | 4.800668534 | -4.574266377 | 6.18E-06 |
| AC007382.1 | -0.456714985 | 0.336508722 | -4.57376156 | 6.20E-06 |
| GS1-115G20.1 | 0.597585355 | -0.63775033 | 4.571877701 | 6.25E-06 |
| RP11-468E2.9 | -0.369572954 | 0.805049728 | -4.570403224 | 6.29E-06 |
| SUMF1 | -0.25929181 | 4.115241083 | -4.570030007 | 6.30E-06 |
| RP11-356J5.5 | 0.341575909 | 1.508164881 | 4.569912573 | 6.31E-06 |
| PSMD5-AS1 | -0.449728785 | 4.519533974 | -4.569687868 | 6.31E-06 |
| PTGFRN | -0.994732792 | 0.93029893 | -4.569473767 | 6.32E-06 |
| MARK2 | -0.204515813 | 6.066852329 | -4.568521666 | 6.35E-06 |
| MSRB1P1 | -0.694674363 | 0.463810374 | -4.568474035 | 6.35E-06 |
| TSLP | -0.982735265 | -1.160336101 | -4.568399226 | 6.35E-06 |
| THOC1 | 0.183965436 | 4.921779492 | 4.567237394 | 6.38E-06 |
| EIF5AP2 | 0.507549495 | 0.164810956 | 4.567112665 | 6.39E-06 |
| WDR48 | 0.216211306 | 5.709032315 | 4.566492816 | 6.41E-06 |
| ANXA9 | -0.454267001 | 0.516168195 | -4.566397977 | 6.41E-06 |
| RP11-589M4.1 | -0.410482509 | 0.866195958 | -4.56602178 | 6.42E-06 |
| RP11-175I17.2 | -0.416003482 | 0.630907357 | -4.565837849 | 6.42E-06 |
| SNTB1 | -0.323950092 | 6.050069978 | -4.564895502 | 6.45E-06 |
| PTRH1 | -0.313455725 | 1.856576637 | -4.563629831 | 6.49E-06 |
| RP11-477J21.6 | -0.83397104 | 0.167695812 | -4.560425584 | 6.58E-06 |
| RN7SL832P | -0.385556083 | 0.616114261 | -4.560186237 | 6.59E-06 |
| DYNC2H1 | -0.576904485 | 0.886979216 | -4.559960139 | 6.60E-06 |
| ZNF26 | 0.437576552 | -0.480205072 | 4.559186045 | 6.62E-06 |
| LTN1 | -0.166738424 | 5.138766663 | -4.55916232 | 6.62E-06 |
| TAF11 | 0.163747339 | 6.98840201 | 4.558690416 | 6.64E-06 |
| CTA-989H11.1 | -0.483965322 | -0.287056935 | -4.5578588 | 6.66E-06 |
| FTH1P7 | 0.457969436 | 7.481679325 | 4.557037528 | 6.69E-06 |
| PLAU | 0.615573641 | 5.194673634 | 4.556909387 | 6.69E-06 |
| SRP9 | -0.189482812 | 7.929007826 | -4.556802706 | 6.69E-06 |
| C17orf97 | -0.650537755 | 0.580229563 | -4.555900473 | 6.72E-06 |
| RP1-111C20.3 | -0.771721884 | -1.084342789 | -4.555114077 | 6.75E-06 |
| PIGX | -0.197293561 | 4.5944476 | -4.55504886 | 6.75E-06 |
| MIAT | 0.641647138 | 2.902707817 | 4.554373824 | 6.77E-06 |
| GATSL1 | 0.538125035 | 0.687350146 | 4.554267777 | 6.77E-06 |
| PCED1A | -0.294380601 | 6.865231406 | -4.554047067 | 6.78E-06 |
| MYBPC2 | 0.587026177 | -1.942163711 | 4.553767722 | 6.79E-06 |
| CD274 | 0.643620371 | 0.5395601 | 4.553669192 | 6.79E-06 |
| SPOCK2 | 0.666820638 | 5.139175431 | 4.553055404 | 6.81E-06 |
| RP11-312B8.1 | -0.453106176 | 0.979587685 | -4.552841246 | 6.82E-06 |
| RP11-162A12.2 | -0.304164511 | 2.175592954 | -4.552259536 | 6.83E-06 |
| U73169.1 | 0.445545816 | 2.690497732 | 4.550667517 | 6.88E-06 |
| RRP15 | 0.211019389 | 3.572126868 | 4.550354199 | 6.89E-06 |
| ZNF71 | -0.262325212 | 3.072068265 | -4.54965457 | 6.92E-06 |
| RP11-439A17.7 | 0.67314768 | 0.181304424 | 4.549107982 | 6.93E-06 |
| COQ2 | -0.222132679 | 5.051548745 | -4.548817472 | 6.94E-06 |
| RP11-350N15.5 | -0.377598581 | 1.581143767 | -4.548683874 | 6.95E-06 |
| RGL4 | 0.825661057 | 3.992285875 | 4.548481972 | 6.95E-06 |
| C8orf46 | -0.556622231 | -0.866998246 | -4.547980306 | 6.97E-06 |
| TRAV10 | -0.556698832 | -0.900003444 | -4.547827114 | 6.97E-06 |
| LRP2BP | 0.539829805 | -2.104777667 | 4.546416883 | 7.02E-06 |
| ANK2 | 0.95849258 | -4.847371818 | 4.546071343 | 7.03E-06 |
| TRBV9 | -0.611070644 | 1.024680416 | -4.54418241 | 7.09E-06 |
| RP11-468E2.5 | -0.373773811 | 2.288440657 | -4.544158387 | 7.09E-06 |
| RP11-981G7.6 | -0.366598219 | 1.930844336 | -4.54398237 | 7.10E-06 |
| HTR3A | 0.511719986 | -3.125323913 | 4.543549413 | 7.11E-06 |
| RP11-326C3.7 | 0.567453569 | 2.765862109 | 4.542918388 | 7.13E-06 |
| AC008746.10 | -0.6568811 | -0.863213265 | -4.542660248 | 7.14E-06 |
| METTL16 | 0.180820259 | 4.627253448 | 4.542176156 | 7.15E-06 |
| CCDC159 | -0.2674235 | 4.71616899 | -4.541070329 | 7.19E-06 |
| EML2 | -0.20837348 | 4.118839131 | -4.540169181 | 7.22E-06 |
| CTD-2587H19.1 | -0.383014525 | 4.734396264 | -4.540052344 | 7.22E-06 |
| RP11-90H3.1 | 0.206687861 | 5.44531288 | 4.539859723 | 7.23E-06 |
| DNAJA4 | 0.576672533 | 4.099677231 | 4.539430173 | 7.24E-06 |
| MRPL11 | 0.194493826 | 5.862315693 | 4.539294121 | 7.25E-06 |
| ECE1 | 0.65695588 | 5.088194979 | 4.5392239 | 7.25E-06 |
| BGN | 0.908924947 | -1.932563248 | 4.538212504 | 7.28E-06 |
| HLA-C | 0.274213931 | 10.92596142 | 4.537913515 | 7.29E-06 |
| NPHP3 | -0.188397264 | 3.81583209 | -4.536457466 | 7.34E-06 |
| CTC-421K24.1 | -0.497862259 | -0.141484047 | -4.535702344 | 7.37E-06 |
| SPIN3 | -0.692972079 | 1.086546829 | -4.534679212 | 7.40E-06 |
| RP11-480D4.6 | -0.64070332 | -1.023726478 | -4.533848222 | 7.43E-06 |
| UBOX5 | 0.205383342 | 3.597662755 | 4.533499873 | 7.44E-06 |
| TNKS2 | 0.151088386 | 6.566783763 | 4.531990727 | 7.49E-06 |
| TMED6 | -0.48470407 | 0.01859934 | -4.531979485 | 7.49E-06 |
| IL13 | 0.725404329 | -1.533951435 | 4.529884917 | 7.56E-06 |
| C2CD5 | -0.188999102 | 5.119716023 | -4.52972414 | 7.57E-06 |
| KLHDC10 | -0.167984546 | 4.971639697 | -4.529384204 | 7.58E-06 |
| NOL3 | -0.465796365 | 2.10103016 | -4.528215044 | 7.62E-06 |
| SPPL2A | 0.264135763 | 5.349633538 | 4.52778704 | 7.64E-06 |
| KB-1732A1.1 | 0.405167305 | 1.378071516 | 4.527341486 | 7.65E-06 |
| GMFG | -0.208533281 | 8.981873182 | -4.527236109 | 7.66E-06 |
| GRPEL2 | 0.276549165 | 3.540519797 | 4.526048104 | 7.70E-06 |
| TTN | 0.542140537 | 3.787996423 | 4.525892447 | 7.70E-06 |
| SOX9 | 0.691332665 | -3.584537927 | 4.525872893 | 7.70E-06 |
| STRN | 0.129160011 | 5.764397575 | 4.524531086 | 7.75E-06 |
| SLC51A | 0.878325306 | -0.637261349 | 4.524324737 | 7.76E-06 |
| IL1RN | 0.926278404 | 4.815283207 | 4.523965726 | 7.77E-06 |
| Z97634.3 | -0.430738039 | 0.410461106 | -4.523298851 | 7.79E-06 |
| EDNRB | 0.970102376 | -1.94418255 | 4.523072926 | 7.80E-06 |
| C1orf127 | -0.741411607 | -0.206199819 | -4.523052092 | 7.80E-06 |
| RP11-474O21.5 | 0.695195328 | -0.239041811 | 4.521149429 | 7.87E-06 |
| AC007386.3 | -0.547569981 | 0.480221341 | -4.520867666 | 7.88E-06 |
| AC007000.11 | -0.475819222 | 1.174811995 | -4.520717049 | 7.88E-06 |
| AC093620.5 | -0.568073464 | -0.283329815 | -4.519090332 | 7.94E-06 |
| RP11-18H21.1 | -0.717818884 | -0.463112087 | -4.519064326 | 7.94E-06 |
| WHSC1 | 0.258219567 | 6.020154027 | 4.518875993 | 7.95E-06 |
| SMARCE1 | 0.161379861 | 4.665385286 | 4.518772125 | 7.95E-06 |
| GSK3B | -0.130553191 | 5.960971739 | -4.517538069 | 8.00E-06 |
| RP11-77K12.5 | -0.442521329 | -0.388628854 | -4.51711721 | 8.01E-06 |
| ARHGAP27 | -0.31171767 | 5.856396647 | -4.5170202 | 8.02E-06 |
| ENPP5 | -0.694610824 | -1.322745346 | -4.516714864 | 8.03E-06 |
| NCF4 | -0.378056661 | 8.362977115 | -4.51635102 | 8.04E-06 |
| ZNF76 | -0.159936308 | 4.83838145 | -4.516137121 | 8.05E-06 |
| CTD-2517M14.5 | -0.555342827 | -0.126159644 | -4.514722804 | 8.10E-06 |
| PPP2CA | 0.214150161 | 6.86588113 | 4.514247958 | 8.12E-06 |
| SLU7 | 0.190163816 | 6.458636561 | 4.513576103 | 8.14E-06 |
| DNAJA1 | -0.207447163 | 8.24422568 | -4.513243894 | 8.15E-06 |
| MRPL1 | -0.224872986 | 5.320059288 | -4.512685889 | 8.18E-06 |
| PEX2 | 0.190435008 | 4.83506168 | 4.511611219 | 8.22E-06 |
| FTSJ2 | -0.270298138 | 5.419848943 | -4.51069242 | 8.25E-06 |
| JMY | 0.53696903 | 4.198992914 | 4.510640796 | 8.25E-06 |
| DDRGK1 | -0.15445628 | 6.681840597 | -4.509802966 | 8.28E-06 |
| RPS10P7 | -0.39498684 | 2.718547746 | -4.509202782 | 8.30E-06 |
| SRRD | 0.168104054 | 4.494258908 | 4.50878331 | 8.32E-06 |
| PNPLA4 | -0.335465291 | 3.27197076 | -4.508651954 | 8.33E-06 |
| RP11-159C21.4 | -0.17795672 | 10.11572146 | -4.507507117 | 8.37E-06 |
| AQR | 0.115834488 | 5.015548065 | 4.507473035 | 8.37E-06 |
| RP11-215G15.4 | -0.561624183 | 1.048081338 | -4.5072851 | 8.38E-06 |
| MCMBP | -0.281424354 | 5.802796793 | -4.507127675 | 8.38E-06 |
| ARHGAP21 | 0.63882894 | 4.262979138 | 4.506898197 | 8.39E-06 |
| GOLGA7 | -0.153126196 | 7.127759107 | -4.506838684 | 8.39E-06 |
| SLC2A3P4 | 0.584460975 | -0.905928634 | 4.506303047 | 8.41E-06 |
| GPRIN3 | 0.676633126 | 3.19841968 | 4.506231724 | 8.42E-06 |
| RP11-422P24.10 | -0.479857841 | 0.323543623 | -4.50619768 | 8.42E-06 |
| SV2B | -1.151801685 | -1.620247304 | -4.506083564 | 8.42E-06 |
| ARPC3P5 | -0.267702537 | 5.43035519 | -4.505856568 | 8.43E-06 |
| ZNF814 | 0.425697816 | 1.740124526 | 4.505686667 | 8.44E-06 |
| ZNF25 | -0.214123876 | 4.52658477 | -4.505341532 | 8.45E-06 |
| ELF1 | 0.250629789 | 8.620422692 | 4.504450482 | 8.48E-06 |
| CYP1B1-AS1 | -0.680446065 | -2.477914023 | -4.504078415 | 8.50E-06 |
| TRIM34 | -0.545655835 | -1.764476421 | -4.50349164 | 8.52E-06 |
| SRGAP3 | -0.804486137 | 0.716437423 | -4.502805879 | 8.55E-06 |
| RIPK3 | -0.310195429 | 4.271793693 | -4.502574219 | 8.56E-06 |
| RP11-129J12.1 | -0.881219547 | -0.851968149 | -4.502494333 | 8.56E-06 |
| SNX33 | -0.473203957 | 3.285469028 | -4.5021476 | 8.57E-06 |
| LL22NC03-2H8.5 | -0.48728868 | 4.479570977 | -4.501275156 | 8.61E-06 |
| ANKEF1 | -0.564348076 | 0.666815611 | -4.501086806 | 8.61E-06 |
| CA5B | -0.375349316 | 4.29514912 | -4.500950381 | 8.62E-06 |
| CARD16 | -0.415234961 | 6.169793636 | -4.499889365 | 8.66E-06 |
| ZFP69 | -0.300904456 | 3.251972995 | -4.499723822 | 8.67E-06 |
| RP4-545K15.5 | 0.489793025 | 3.204015535 | 4.499435554 | 8.68E-06 |
| AC104088.1 | -0.841253948 | -0.781956723 | -4.498428994 | 8.72E-06 |
| AC016831.7 | 0.547504363 | -1.055711428 | 4.498399853 | 8.72E-06 |
| KCTD9P2 | 0.238362561 | 3.451155116 | 4.498381744 | 8.72E-06 |
| ZBTB7A | 0.289466571 | 5.656154701 | 4.498283763 | 8.72E-06 |
| DLX4 | -0.651320393 | 0.369363559 | -4.498099329 | 8.73E-06 |
| FAM106B | -0.543446354 | -0.930170923 | -4.498058143 | 8.73E-06 |
| MT-ND4L | -0.137131497 | 12.486152 | -4.498049209 | 8.73E-06 |
| RPS27L | -0.239803125 | 5.088150495 | -4.497417847 | 8.76E-06 |
| XXbac-B135H6.18 | -0.431341542 | 0.807487668 | -4.497395491 | 8.76E-06 |
| ALG10 | 0.204883698 | 3.286057366 | 4.497358191 | 8.76E-06 |
| C4orf48 | -0.377321848 | 4.513058489 | -4.496196603 | 8.81E-06 |
| RP11-534L20.4 | 0.263544937 | 2.918187577 | 4.495530474 | 8.83E-06 |
| RNF43 | 0.50717999 | -0.066009401 | 4.495528893 | 8.83E-06 |
| RP11-700J17.2 | 0.387878374 | 3.785807226 | 4.495185415 | 8.85E-06 |
| SNRPGP14 | -0.442026343 | 1.194441195 | -4.495125244 | 8.85E-06 |
| ZMYM5 | 0.22390947 | 5.140362569 | 4.494974351 | 8.85E-06 |
| MIPEPP3 | -0.463221344 | 2.105417674 | -4.49398432 | 8.89E-06 |
| AC092155.1 | 0.385520901 | 2.812734649 | 4.493506535 | 8.91E-06 |
| AGPAT6 | 0.218721112 | 5.911052203 | 4.491655995 | 8.99E-06 |
| FAF1 | 0.180450543 | 5.597186312 | 4.49096255 | 9.02E-06 |
| FCF1P7 | -0.434257459 | 0.196418247 | -4.490448225 | 9.04E-06 |
| RP11-440D17.3 | -0.790599484 | -0.791547974 | -4.490183404 | 9.05E-06 |
| SSR4 | 0.188068189 | 6.489170599 | 4.490050495 | 9.05E-06 |
| PPM1F | -0.295237909 | 6.818257438 | -4.48980115 | 9.06E-06 |
| RP3-342P20.2 | 0.309989307 | 3.255286515 | 4.489645258 | 9.07E-06 |
| SGK2 | -0.512972906 | -0.192140827 | -4.489251962 | 9.09E-06 |
| CIAPIN1 | 0.153161458 | 5.232895661 | 4.489085868 | 9.09E-06 |
| SERPINF2 | -0.477264765 | 2.437526543 | -4.4889212 | 9.10E-06 |
| TNFSF11 | 0.629880393 | -2.409712548 | 4.488578262 | 9.11E-06 |
| RP4-814D15.2 | 0.385413963 | 1.06311209 | 4.487450105 | 9.16E-06 |
| C6orf223 | 0.789718027 | -2.221752991 | 4.487428026 | 9.16E-06 |
| LSM14B | -0.228420792 | 6.628122313 | -4.48726701 | 9.17E-06 |
| SMG8 | 0.253927324 | 4.300911688 | 4.486468148 | 9.20E-06 |
| ESCO2 | 0.692684928 | 1.257757182 | 4.485061323 | 9.26E-06 |
| GPR180 | -0.255905404 | 2.936966823 | -4.484668493 | 9.27E-06 |
| POLQ | 0.552225705 | 2.857982406 | 4.484277003 | 9.29E-06 |
| MAPKAPK5 | 0.128896599 | 5.164600769 | 4.483864488 | 9.31E-06 |
| RNA5SP129 | 0.507121093 | -0.101397853 | 4.483693953 | 9.31E-06 |
| PSEN2 | -0.432351183 | 3.059485012 | -4.483043 | 9.34E-06 |
| SNRPEP4 | -0.246917448 | 6.722911723 | -4.482920046 | 9.35E-06 |
| ZDHHC13 | -0.287796034 | 3.29537916 | -4.482671022 | 9.36E-06 |
| CNNM2 | 0.264502382 | 2.697917233 | 4.482463377 | 9.37E-06 |
| SH2B1 | -0.166229314 | 5.391528298 | -4.482414497 | 9.37E-06 |
| MIS18BP1 | -0.229325578 | 6.943258039 | -4.482411378 | 9.37E-06 |
| SMAD2 | -0.144191204 | 5.189038647 | -4.482031707 | 9.38E-06 |
| CD6 | 0.655377084 | 3.642065282 | 4.481460999 | 9.41E-06 |
| RP11-178F10.2 | -0.568929005 | -0.904597372 | -4.481221116 | 9.42E-06 |
| CTD-2023M8.1 | -0.536653268 | 0.944377246 | -4.479837875 | 9.48E-06 |
| SNORA31 | -0.397718935 | 1.735917164 | -4.479762155 | 9.48E-06 |
| POLR2K | 0.17341849 | 6.769182698 | 4.479572428 | 9.49E-06 |
| S100P | 1.062784325 | 5.500316083 | 4.479480329 | 9.49E-06 |
| PPP1R26-AS1 | -0.782731676 | -0.647969669 | -4.478626458 | 9.53E-06 |
| RNF219 | -0.326887898 | 4.307988774 | -4.478593496 | 9.53E-06 |
| TRUB2 | -0.264276479 | 4.991110984 | -4.478312127 | 9.54E-06 |
| RP11-513M16.8 | -0.41714211 | 1.527570411 | -4.477890603 | 9.56E-06 |
| PAXBP1-AS1 | -0.368581666 | 1.536024638 | -4.477837247 | 9.56E-06 |
| RP11-196G18.3 | -0.591918223 | -1.518845495 | -4.476864962 | 9.60E-06 |
| GLYR1 | -0.14906858 | 6.071683888 | -4.476605265 | 9.62E-06 |
| VAPA | 0.188363878 | 5.905289289 | 4.474681797 | 9.70E-06 |
| CCDC147-AS1 | 0.605785832 | 1.097777521 | 4.474212103 | 9.72E-06 |
| GXYLT1 | -0.171891019 | 4.326761842 | -4.474090579 | 9.72E-06 |
| NFS1 | -0.238341714 | 3.12668556 | -4.473195971 | 9.76E-06 |
| RP4-635E18.6 | -0.552111953 | -0.353064434 | -4.472701195 | 9.79E-06 |
| DARC | 0.845874434 | -1.200441854 | 4.472548579 | 9.79E-06 |
| MORN4 | -0.657595557 | 0.320523721 | -4.47169773 | 9.83E-06 |
| PPEF2 | -0.59171891 | -2.404101293 | -4.470445432 | 9.88E-06 |
| TMEM106C | 0.372224917 | 4.988121918 | 4.47023048 | 9.89E-06 |
| FAM204A | 0.155566672 | 4.96466427 | 4.469989699 | 9.90E-06 |
| RP11-353N4.4 | -0.521317359 | 1.13534342 | -4.468783251 | 9.96E-06 |
| DNM1P35 | -0.663805874 | -1.427464169 | -4.468534198 | 9.97E-06 |
| B3GALT4 | -0.360055794 | 3.459768273 | -4.467953885 | 1.00E-05 |
| HSPE1P2 | -0.349635363 | 5.453328156 | -4.467749079 | 1.00E-05 |
| FRRS1 | 0.415848034 | 2.223171529 | 4.46710558 | 1.00E-05 |
| EXOC5 | -0.173877501 | 5.172216313 | -4.465576769 | 1.01E-05 |
| PDLIM7 | 0.357414268 | 5.929771752 | 4.465446671 | 1.01E-05 |
| MSL1 | -0.184025303 | 6.890866576 | -4.465226012 | 1.01E-05 |
| TCOF1 | -0.241676806 | 4.793538593 | -4.464899096 | 1.01E-05 |
| RP5-827C21.4 | 0.311449614 | 4.017264211 | 4.464667413 | 1.01E-05 |
| SDHC | -0.215599278 | 5.785115018 | -4.464452774 | 1.02E-05 |
| PEX13 | 0.177295093 | 4.102004331 | 4.463936662 | 1.02E-05 |
| CTNNA1 | -0.362453944 | 6.114133642 | -4.463888119 | 1.02E-05 |
| SLC5A9 | -0.724179889 | -2.935649232 | -4.463745069 | 1.02E-05 |
| CCDC58P3 | -0.346526188 | 2.381144672 | -4.463525602 | 1.02E-05 |
| HIP1 | -0.455803182 | 5.478701437 | -4.462145239 | 1.03E-05 |
| NEK1 | -0.220162675 | 3.472819536 | -4.462138668 | 1.03E-05 |
| ZNF101 | 0.252546601 | 3.74966956 | 4.461849061 | 1.03E-05 |
| CDC20P1 | 0.682317527 | 1.692619357 | 4.461212133 | 1.03E-05 |
| SPG21 | -0.206975478 | 6.685105814 | -4.460592147 | 1.03E-05 |
| MSRB3 | -0.591679873 | 6.671455836 | -4.460241257 | 1.03E-05 |
| RP11-1096G20.5 | -0.431119214 | -0.014627763 | -4.459922665 | 1.04E-05 |
| CCL4 | 0.747251134 | 3.332492008 | 4.45963086 | 1.04E-05 |
| RP11-9L18.2 | 0.165759025 | 6.313979429 | 4.459630463 | 1.04E-05 |
| HECW2 | -0.599451161 | -1.681707287 | -4.459576405 | 1.04E-05 |
| FNBP1P1 | 0.375657326 | 3.111360485 | 4.458679385 | 1.04E-05 |
| STXBP4 | -0.335717314 | 1.619654789 | -4.458061175 | 1.04E-05 |
| CTBP2P4 | -0.355907936 | 2.711944027 | -4.45750665 | 1.05E-05 |
| RP11-544I20.2 | -0.521675944 | -1.558965082 | -4.457333267 | 1.05E-05 |
| EYA3 | -0.190075872 | 4.523051679 | -4.456943378 | 1.05E-05 |
| CTD-2516F10.2 | -0.631677956 | 0.405257438 | -4.45647901 | 1.05E-05 |
| DYX1C1 | -0.61594433 | -1.342461227 | -4.456439638 | 1.05E-05 |
| RP11-45P15.4 | 0.411611625 | 1.514331733 | 4.455608094 | 1.06E-05 |
| TTC32 | 0.246109373 | 5.164919667 | 4.45556705 | 1.06E-05 |
| BGLAP | -0.505546639 | 0.023586659 | -4.454976279 | 1.06E-05 |
| FXR2 | -0.210923809 | 5.087990602 | -4.454313432 | 1.06E-05 |
| CCDC96 | 0.38367269 | 1.568439966 | 4.45402201 | 1.06E-05 |
| TMEM161A | -0.236449774 | 4.053743557 | -4.452988419 | 1.07E-05 |
| BCL7A | 0.510616681 | 4.221223694 | 4.452794331 | 1.07E-05 |
| RP11-357C3.3 | -0.267687973 | 4.129430773 | -4.451735341 | 1.07E-05 |
| ANP32A | -0.189241688 | 7.348989837 | -4.451600434 | 1.08E-05 |
| PDE7A | -0.345499548 | 6.43779756 | -4.451579983 | 1.08E-05 |
| PIM2 | 0.403453741 | 6.578473842 | 4.451579141 | 1.08E-05 |
| RNF122 | 0.337257217 | 5.127189695 | 4.451465397 | 1.08E-05 |
| FBXO34 | 0.217595275 | 5.607596124 | 4.45119556 | 1.08E-05 |
| AGBL2 | -0.468335668 | 0.560073289 | -4.450174811 | 1.08E-05 |
| ATXN2L | 0.156931164 | 7.809260559 | 4.449547774 | 1.09E-05 |
| RSL24D1P11 | 0.222141776 | 4.076006546 | 4.449403606 | 1.09E-05 |
| CLDND1 | 0.200667339 | 5.532667459 | 4.448783875 | 1.09E-05 |
| FAM188A | 0.205647906 | 4.680265493 | 4.448739272 | 1.09E-05 |
| PURA | -0.414849129 | 3.827150529 | -4.448271871 | 1.09E-05 |
| DDX42 | -0.143503055 | 6.738795901 | -4.448150607 | 1.09E-05 |
| MT-TF | 0.444262511 | 2.194055352 | 4.447895682 | 1.09E-05 |
| COQ7 | -0.182682053 | 4.035197385 | -4.446921246 | 1.10E-05 |
| RP11-307P22.1 | 0.391893175 | 3.09242887 | 4.446591872 | 1.10E-05 |
| EPHB3 | -0.930772287 | -0.139574469 | -4.446491296 | 1.10E-05 |
| JMJD7-PLA2G4B | -0.248480277 | 2.141260655 | -4.445799807 | 1.10E-05 |
| CBR1 | -0.469920382 | 4.988313227 | -4.445586567 | 1.10E-05 |
| RP11-333J10.3 | 0.480695743 | 1.644173137 | 4.445120887 | 1.11E-05 |
| ZFP36L1 | 0.664950846 | 6.151385565 | 4.444234933 | 1.11E-05 |
| PDGFD | -0.81068402 | 2.219937198 | -4.444083477 | 1.11E-05 |
| SLC25A44 | 0.199766996 | 5.011509639 | 4.443638785 | 1.11E-05 |
| CCDC85C | 0.676247468 | 0.811003 | 4.442976894 | 1.12E-05 |
| JSRP1 | 0.538187273 | 1.268638042 | 4.442949712 | 1.12E-05 |
| ERLEC1 | 0.180651222 | 6.01603185 | 4.442450746 | 1.12E-05 |
| CTC-1337H24.1 | 0.71428671 | 0.205420789 | 4.441828212 | 1.12E-05 |
| TEAD3 | 0.814429102 | -0.721321279 | 4.441751343 | 1.12E-05 |
| RP11-361D15.2 | -0.359540753 | 2.872578376 | -4.44076512 | 1.13E-05 |
| DUSP13 | 0.910425148 | -0.905651153 | 4.43974484 | 1.13E-05 |
| TMEM30A | -0.24106838 | 7.220874853 | -4.43957809 | 1.13E-05 |
| SERINC1 | 0.23830808 | 8.020152232 | 4.439428924 | 1.14E-05 |
| EFNA3 | -0.49727537 | 0.060238189 | -4.439386671 | 1.14E-05 |
| VAPB | -0.212675444 | 4.077003324 | -4.439267687 | 1.14E-05 |
| RP11-445F6.2 | -0.779284328 | -0.647881495 | -4.439170787 | 1.14E-05 |
| CNKSR3 | 0.751473265 | 0.77976797 | 4.439063339 | 1.14E-05 |
| ANKRD12 | 0.215910361 | 5.563064976 | 4.438384207 | 1.14E-05 |
| PRKCD | -0.389530659 | 7.376911447 | -4.437745194 | 1.14E-05 |
| THAP9-AS1 | 0.230203829 | 5.978998402 | 4.436824577 | 1.15E-05 |
| GARS | 0.161957581 | 6.728518327 | 4.436779193 | 1.15E-05 |
| C22orf15 | -0.608705593 | 0.126637351 | -4.436016862 | 1.15E-05 |
| AC013264.2 | -0.530640796 | -0.444870037 | -4.434846374 | 1.16E-05 |
| RP4-635E18.7 | -0.52674556 | 0.50235441 | -4.434418057 | 1.16E-05 |
| VPS26AP1 | 0.302841441 | 2.373086671 | 4.434352327 | 1.16E-05 |
| METTL15P1 | -0.321605511 | 2.073741412 | -4.433765102 | 1.16E-05 |
| ZBTB18 | 0.323049833 | 5.901687514 | 4.433504162 | 1.17E-05 |
| DLGAP5 | 0.767834515 | 3.118082548 | 4.433201602 | 1.17E-05 |
| KIF18B | 0.725163881 | 2.530366063 | 4.432943431 | 1.17E-05 |
| TRIM46 | -0.503765864 | 2.125770601 | -4.432323369 | 1.17E-05 |
| SLC35D1 | 0.194993747 | 4.361636025 | 4.432037351 | 1.17E-05 |
| AGAP2 | -0.35087062 | 5.513618173 | -4.431790492 | 1.17E-05 |
| SSSCA1-AS1 | -0.34338056 | 2.264898203 | -4.430188385 | 1.18E-05 |
| ZNF680 | -0.310744709 | 4.253548593 | -4.429758279 | 1.19E-05 |
| WDR47 | 0.176508314 | 4.632312507 | 4.4294133 | 1.19E-05 |
| KCNJ14 | 0.456010885 | 1.064091992 | 4.428447364 | 1.19E-05 |
| HMGN1P37 | 0.296315641 | 3.24392609 | 4.426976209 | 1.20E-05 |
| MED31 | -0.242382231 | 2.848831073 | -4.426169944 | 1.20E-05 |
| SNORA16B | 0.412015841 | 1.471092773 | 4.423990784 | 1.22E-05 |
| GUCY2D | -0.766439688 | -1.204549864 | -4.423739931 | 1.22E-05 |
| ZC3H7A | 0.145174053 | 5.96160777 | 4.423681599 | 1.22E-05 |
| TXNDC9 | -0.176592298 | 4.954815743 | -4.423151891 | 1.22E-05 |
| RASA1 | -0.209176142 | 5.396330308 | -4.422886408 | 1.22E-05 |
| PTOV1-AS1 | -0.347102377 | -0.544737067 | -4.42218985 | 1.23E-05 |
| RP11-815J4.6 | -0.545317845 | -0.905044919 | -4.422138517 | 1.23E-05 |
| IL10RB | -0.323584569 | 6.101167236 | -4.421909356 | 1.23E-05 |
| RPS2P44 | 0.579623454 | -0.189452836 | 4.4215058 | 1.23E-05 |
| DPP3 | -0.214400637 | 5.436426168 | -4.421197261 | 1.23E-05 |
| FAM86B3P | -0.366206693 | 1.958316241 | -4.420860595 | 1.23E-05 |
| ACSS1 | -0.303394616 | 5.605117571 | -4.420716834 | 1.23E-05 |
| LDAF1 | -0.231586776 | 4.679723164 | -4.42055504 | 1.23E-05 |
| DUSP16 | 0.549529939 | 3.065272345 | 4.420447991 | 1.24E-05 |
| MICA | -0.239409965 | 5.063814249 | -4.419867754 | 1.24E-05 |
| RP11-82L2.1 | -0.63638085 | -2.904875737 | -4.419745157 | 1.24E-05 |
| GAB2 | 0.354793178 | 7.156519583 | 4.419480453 | 1.24E-05 |
| ETS2 | 0.446904876 | 7.901597406 | 4.419093694 | 1.24E-05 |
| OSBPL11 | -0.354201201 | 5.384880529 | -4.418807518 | 1.24E-05 |
| AC007773.2 | -0.473959578 | -0.095341831 | -4.418562937 | 1.25E-05 |
| RP11-382A20.2 | 0.683311279 | -0.049045451 | 4.418403243 | 1.25E-05 |
| AC079325.6 | -1.006739668 | 2.334219363 | -4.417777022 | 1.25E-05 |
| RAB23 | -0.722284525 | 0.379782007 | -4.417776843 | 1.25E-05 |
| HMGA1P4 | -0.506484836 | 2.118174183 | -4.417606414 | 1.25E-05 |
| RP11-557J10.3 | -0.39213026 | 0.901131191 | -4.417286923 | 1.25E-05 |
| CNTD2 | 0.565377608 | -0.361885649 | 4.416688126 | 1.26E-05 |
| NAPRT1 | -0.239987327 | 7.01753849 | -4.41648823 | 1.26E-05 |
| CDRT4 | -0.586676436 | -0.48144484 | -4.415864354 | 1.26E-05 |
| SPICE1 | -0.237522199 | 3.479276243 | -4.415162255 | 1.26E-05 |
| CD7 | 0.690930833 | 4.897535595 | 4.415029757 | 1.27E-05 |
| SHISA5 | -0.196650975 | 6.7829093 | -4.413865193 | 1.27E-05 |
| PTGDR2 | -0.770904605 | 1.769486964 | -4.413831463 | 1.27E-05 |
| NAAA | -0.437790958 | 5.207161193 | -4.413174524 | 1.28E-05 |
| NR4A2 | 0.758995032 | 6.211534842 | 4.412511973 | 1.28E-05 |
| RP11-950C14.7 | -0.521087176 | -0.226521456 | -4.412188137 | 1.28E-05 |
| FAM172A | -0.185974419 | 5.06719046 | -4.412114568 | 1.28E-05 |
| RPS2P4 | 0.268677638 | 2.710151873 | 4.411638658 | 1.28E-05 |
| ABCC1 | -0.37189059 | 6.871907003 | -4.411623801 | 1.28E-05 |
| MIB2 | -0.175298171 | 4.717683725 | -4.411367851 | 1.29E-05 |
| MTBP | 0.263144626 | 2.267145068 | 4.410671236 | 1.29E-05 |
| MS4A4E | -0.960987032 | 4.300424883 | -4.410438078 | 1.29E-05 |
| HELZ2 | 0.466229952 | 4.622552118 | 4.410148251 | 1.29E-05 |
| HLA-H | 0.411446915 | 6.892164093 | 4.409473978 | 1.30E-05 |
| ARNT2 | 0.880881694 | -3.419628736 | 4.409466982 | 1.30E-05 |
| MKRN4P | 0.444085021 | 0.082367182 | 4.409301971 | 1.30E-05 |
| RNA18S5 | 0.461434249 | 3.524421468 | 4.408611981 | 1.30E-05 |
| RNF213 | -0.27505074 | 7.705601145 | -4.408516376 | 1.30E-05 |
| FAM219A | -0.190306423 | 4.154668979 | -4.407814187 | 1.31E-05 |
| SNORD59A | 0.430363416 | 3.349280293 | 4.406013701 | 1.32E-05 |
| ZNF264 | 0.24121536 | 4.516081602 | 4.405791678 | 1.32E-05 |
| RP11-641D5.1 | -0.18428825 | 10.46938533 | -4.405724636 | 1.32E-05 |
| RLN2 | -0.51375929 | -1.348566849 | -4.404625744 | 1.32E-05 |
| NENF | 0.232290484 | 5.755444679 | 4.404315562 | 1.33E-05 |
| CTD-2086O20.3 | 0.292787807 | 2.962689132 | 4.402852373 | 1.34E-05 |
| VPS9D1 | 0.307591839 | 5.044684553 | 4.402245814 | 1.34E-05 |
| RP11-168J18.6 | 0.223747803 | 4.900712653 | 4.401717489 | 1.34E-05 |
| SPATS2 | 0.283174605 | 3.828874814 | 4.401699812 | 1.34E-05 |
| CHUK | 0.179984481 | 4.806637685 | 4.401362649 | 1.34E-05 |
| RP5-991G20.1 | -0.46566077 | 2.371105389 | -4.40125536 | 1.34E-05 |
| NECAB1 | 0.787034774 | -3.265303718 | 4.400843208 | 1.35E-05 |
| GORASP1 | -0.142809322 | 5.213716489 | -4.400365344 | 1.35E-05 |
| RP11-318E3.9 | -0.297481776 | 3.144156892 | -4.399149589 | 1.36E-05 |
| ZNF329 | 0.446823216 | 3.910725974 | 4.399005887 | 1.36E-05 |
| RP11-816J6.3 | 0.54530175 | 0.941838727 | 4.398612766 | 1.36E-05 |
| MDM2 | 0.285972013 | 5.756799412 | 4.398308394 | 1.36E-05 |
| AP001347.6 | 0.5562195 | -0.640324851 | 4.397380688 | 1.37E-05 |
| RASGRF1 | -0.853682952 | -2.119441041 | -4.397355442 | 1.37E-05 |
| SPSB1 | 0.629815988 | 1.142681576 | 4.395996738 | 1.38E-05 |
| KLHDC8A | 0.829108739 | -2.627819146 | 4.39588538 | 1.38E-05 |
| TPCN1 | -0.432713359 | 4.482439557 | -4.394967802 | 1.38E-05 |
| TBC1D3H | -0.635096437 | -2.912507425 | -4.394838018 | 1.38E-05 |
| TSEN54 | 0.304810152 | 4.782788575 | 4.394781731 | 1.38E-05 |
| ZBTB34 | 0.224321438 | 5.537099062 | 4.39472675 | 1.38E-05 |
| CPT2 | -0.290076324 | 4.728500628 | -4.394697418 | 1.38E-05 |
| NSL1 | -0.161616285 | 4.702466796 | -4.394622244 | 1.38E-05 |
| LINC00892 | -0.569386948 | -1.328911365 | -4.393951638 | 1.39E-05 |
| DGKQ | -0.229940903 | 5.061476921 | -4.393754671 | 1.39E-05 |
| RP5-991C6.4 | 0.554838003 | 0.026670879 | 4.393578486 | 1.39E-05 |
| ZNF704 | 1.090502661 | 0.477382769 | 4.39352186 | 1.39E-05 |
| LA16c-325D7.2 | -0.530571957 | -0.751239438 | -4.392348378 | 1.40E-05 |
| AEN | 0.302385417 | 4.391813433 | 4.391352095 | 1.40E-05 |
| GGA1 | 0.220740156 | 5.065427869 | 4.390640948 | 1.41E-05 |
| RP11-563N4.1 | 0.426568186 | 0.512810932 | 4.390083059 | 1.41E-05 |
| SCARNA24 | -0.496463608 | 0.806304329 | -4.389942727 | 1.41E-05 |
| ZNF460 | 0.329222619 | 4.440062546 | 4.389533473 | 1.42E-05 |
| RHOG | 0.23175054 | 8.818078445 | 4.389499081 | 1.42E-05 |
| PSPC1 | 0.130938343 | 6.814540302 | 4.389420395 | 1.42E-05 |
| RP11-474I16.8 | -0.484213574 | 0.030025636 | -4.389364246 | 1.42E-05 |
| C19orf52 | 0.185606677 | 4.730641007 | 4.389323892 | 1.42E-05 |
| CCAR2 | 0.13086951 | 6.675607029 | 4.389046225 | 1.42E-05 |
| EIF4E2 | -0.168101137 | 5.549337053 | -4.388829029 | 1.42E-05 |
| PPP4R1L | 0.522756693 | 2.603500403 | 4.388560985 | 1.42E-05 |
| RP11-66D17.5 | 0.466598929 | 1.038640593 | 4.388325723 | 1.42E-05 |
| C5orf28 | -0.176636246 | 3.143275385 | -4.38828998 | 1.42E-05 |
| ZNF77 | -0.257440055 | 3.10145422 | -4.387988069 | 1.43E-05 |
| SIGLEC12 | -1.11191648 | 2.687877124 | -4.38758115 | 1.43E-05 |
| NUAK1 | 0.75540028 | -1.9816021 | 4.386803065 | 1.43E-05 |
| ZNF622 | 0.173632401 | 6.27919338 | 4.386346325 | 1.44E-05 |
| RP11-428G5.5 | -0.730848566 | 0.492911168 | -4.385148952 | 1.44E-05 |
| ERAP1 | -0.272794891 | 6.512223427 | -4.38490803 | 1.45E-05 |
| NAB2 | 0.447089904 | 4.619375115 | 4.384889266 | 1.45E-05 |
| CLDN15 | -0.295790403 | 5.054992854 | -4.384791658 | 1.45E-05 |
| RP11-531F16.3 | 0.483496904 | 0.549090948 | 4.384515201 | 1.45E-05 |
| MTERFD2 | -0.165183764 | 4.388125166 | -4.384435241 | 1.45E-05 |
| PARK7 | -0.155619258 | 7.509245898 | -4.384373596 | 1.45E-05 |
| CNPY2 | 0.193537003 | 3.220067801 | 4.383965057 | 1.45E-05 |
| FNTA | -0.136083625 | 4.499592649 | -4.383811419 | 1.45E-05 |
| OSCP1 | 0.496128991 | 0.672546534 | 4.383615122 | 1.45E-05 |
| PFDN4 | 0.210008206 | 5.004308737 | 4.383593792 | 1.45E-05 |
| WDR70 | -0.153072602 | 4.468982624 | -4.383535128 | 1.45E-05 |
| BEND3P3 | -0.440160522 | 2.364249977 | -4.382118054 | 1.46E-05 |
| BDH2P1 | -0.251288594 | 3.965738488 | -4.381762299 | 1.47E-05 |
| AC005224.2 | -0.418584142 | 0.368025186 | -4.381495137 | 1.47E-05 |
| CYP2D7P | 0.309829935 | 2.196759146 | 4.381411497 | 1.47E-05 |
| GLS2 | -0.496093234 | -1.705693464 | -4.381369417 | 1.47E-05 |
| CTB-129P6.11 | -0.48149713 | 0.258803161 | -4.381073795 | 1.47E-05 |
| WEE2-AS1 | -0.461018529 | 0.594963275 | -4.381059675 | 1.47E-05 |
| SEC24B-AS1 | -0.403915332 | 0.943687859 | -4.381042181 | 1.47E-05 |
| RP11-1084A12.1 | 0.450503626 | 1.026946144 | 4.380518541 | 1.47E-05 |
| FAM117A | -0.441390395 | 6.884515222 | -4.380216565 | 1.48E-05 |
| CTD-2031P19.3 | 0.513851585 | -0.917360868 | 4.37989882 | 1.48E-05 |
| RP11-496N12.6 | -0.629257958 | -0.891594532 | -4.379692495 | 1.48E-05 |
| HEATR2 | -0.285423173 | 3.891645232 | -4.379056626 | 1.48E-05 |
| LIPJ | -0.498766421 | -2.649832976 | -4.378311306 | 1.49E-05 |
| ERMP1 | -0.413296559 | 5.746977764 | -4.377872625 | 1.49E-05 |
| UVRAG | 0.208811016 | 5.383733587 | 4.375844281 | 1.50E-05 |
| FAM83G | 0.493564572 | 2.308908497 | 4.37416099 | 1.52E-05 |
| PAFAH2 | -0.290471534 | 3.840083942 | -4.373703988 | 1.52E-05 |
| MYO9B | 0.14738879 | 7.47964488 | 4.373166964 | 1.52E-05 |
| TRAV9-2 | -0.636321248 | -0.200515863 | -4.373093566 | 1.52E-05 |
| GTF3C1 | -0.185471574 | 5.211010909 | -4.372965681 | 1.52E-05 |
| DNM2 | 0.1113122 | 6.486000516 | 4.372525957 | 1.53E-05 |
| KDR | 0.834714261 | -3.265340741 | 4.372453327 | 1.53E-05 |
| RUNX1 | -0.38081599 | 7.446498767 | -4.371819443 | 1.53E-05 |
| ADAMTS10 | 0.678295309 | 3.040527892 | 4.371663502 | 1.53E-05 |
| FHOD1 | -0.185366632 | 5.767836567 | -4.371150033 | 1.54E-05 |
| ABCD4 | -0.175322267 | 4.584186778 | -4.370087947 | 1.54E-05 |
| PJA1 | -0.258559586 | 4.308055526 | -4.369960097 | 1.54E-05 |
| EEF1A1P3 | 0.510389922 | 1.423528519 | 4.369647115 | 1.55E-05 |
| GAPDHP61 | 0.290846544 | 4.431065583 | 4.368411767 | 1.55E-05 |
| THAP7 | 0.187578061 | 4.666297843 | 4.368357894 | 1.55E-05 |
| BCL2 | -0.393615886 | 5.409779487 | -4.368339909 | 1.55E-05 |
| TRAV14DV4 | -0.651634235 | 0.380863817 | -4.367687642 | 1.56E-05 |
| RSF1-IT1 | 0.525729649 | -0.428980266 | 4.36679252 | 1.57E-05 |
| CEBPA | -0.517540231 | 3.216030601 | -4.365956116 | 1.57E-05 |
| PIH1D1 | -0.145624017 | 5.969590808 | -4.365897257 | 1.57E-05 |
| MIR3679 | 0.506825418 | 0.028450178 | 4.364715381 | 1.58E-05 |
| RN7SL737P | -0.353602886 | 2.639060646 | -4.364189375 | 1.58E-05 |
| RP11-876N24.5 | -0.386761062 | 3.681640025 | -4.363766075 | 1.59E-05 |
| RP4-784A16.5 | -0.59063239 | -0.32712258 | -4.363153769 | 1.59E-05 |
| CCDC40 | -0.756965216 | -1.506861198 | -4.363024636 | 1.59E-05 |
| AC006195.2 | 0.542976051 | -1.095789971 | 4.362791327 | 1.59E-05 |
| VPS8 | -0.199114177 | 4.890399398 | -4.361633694 | 1.60E-05 |
| APRT | -0.190116481 | 7.507351218 | -4.360982252 | 1.61E-05 |
| FAR1-IT1 | 0.557459171 | 2.608552463 | 4.360082932 | 1.61E-05 |
| PPP1R15A | 0.380981214 | 9.80305498 | 4.359968612 | 1.61E-05 |
| AC010226.4 | 0.467986235 | 0.065890993 | 4.359847534 | 1.61E-05 |
| ZGLP1 | -0.416940684 | 0.869660627 | -4.359540439 | 1.62E-05 |
| HGD | 0.588599438 | -0.264425735 | 4.3594708 | 1.62E-05 |
| ASB16-AS1 | -0.183761042 | 4.848035684 | -4.359321151 | 1.62E-05 |
| RP11-645C24.2 | 0.339730213 | 2.165100265 | 4.358791759 | 1.62E-05 |
| RP5-1039K5.17 | -0.469087287 | -0.636404809 | -4.358411354 | 1.62E-05 |
| IPPK | 0.183155127 | 3.19416622 | 4.357695147 | 1.63E-05 |
| NFIB | 0.819017242 | -4.212619566 | 4.357351154 | 1.63E-05 |
| MFI2-AS1 | 0.372396916 | 2.998914075 | 4.357277225 | 1.63E-05 |
| TMEM138 | 0.174420531 | 4.360430209 | 4.355991245 | 1.64E-05 |
| SH3GL1P1 | 0.352891803 | 2.403075616 | 4.355955158 | 1.64E-05 |
| RP11-295D4.1 | -0.363135863 | 0.673165269 | -4.355617029 | 1.64E-05 |
| RP5-1024G6.5 | -0.398040395 | 0.139626652 | -4.354750114 | 1.65E-05 |
| HNRNPA1P16 | -0.317536546 | 2.16188063 | -4.354644219 | 1.65E-05 |
| GPR179 | -0.572974386 | -3.126803842 | -4.353798688 | 1.66E-05 |
| CTD-2139B15.2 | 0.581318584 | -0.935736482 | 4.352707995 | 1.66E-05 |
| GNRH1 | 0.359137987 | 2.276763239 | 4.352472897 | 1.67E-05 |
| ARHGEF2 | -0.218016267 | 7.160788832 | -4.351373623 | 1.67E-05 |
| RP11-286H14.6 | -0.514502177 | -0.693085714 | -4.351221456 | 1.68E-05 |
| ACAD8 | 0.207311989 | 4.17797303 | 4.350908358 | 1.68E-05 |
| BAZ1A | -0.268422071 | 6.809044401 | -4.350853265 | 1.68E-05 |
| PHACTR4 | 0.189938547 | 5.54245357 | 4.350364272 | 1.68E-05 |
| GATSL3 | -0.390551338 | -0.045857923 | -4.349725311 | 1.69E-05 |
| ZNF69 | -0.377635413 | 3.38911744 | -4.34793812 | 1.70E-05 |
| JAZF1-AS1 | -0.654487902 | -2.143246059 | -4.347594607 | 1.70E-05 |
| SORCS1 | -1.084046305 | -2.262906787 | -4.347237644 | 1.71E-05 |
| C11orf74 | -0.423476737 | 4.57032289 | -4.346764661 | 1.71E-05 |
| PARD6B | 0.406806915 | 0.938744516 | 4.346678452 | 1.71E-05 |
| GARIN4 | 0.728197947 | -0.838585652 | 4.346621441 | 1.71E-05 |
| ZBTB7C | -0.77752486 | -3.222648195 | -4.346192918 | 1.71E-05 |
| PDE4A | 0.75357769 | 2.623939063 | 4.344539556 | 1.73E-05 |
| ELOVL6 | 0.487433365 | 2.69357238 | 4.344522304 | 1.73E-05 |
| MORF4L2-AS1 | -0.447385816 | 1.29283637 | -4.344089832 | 1.73E-05 |
| TBC1D19 | -0.316115668 | 1.761482629 | -4.343640856 | 1.73E-05 |
| SNAPC5 | -0.148574208 | 4.274332771 | -4.343339381 | 1.73E-05 |
| HSD17B11 | -0.240895986 | 7.823433086 | -4.342493101 | 1.74E-05 |
| CTD-2008A1.2 | -0.466053049 | 1.584313544 | -4.342483194 | 1.74E-05 |
| SF3B3 | -0.253168767 | 6.2516258 | -4.341361146 | 1.75E-05 |
| HGS | 0.129674644 | 6.003892471 | 4.341311008 | 1.75E-05 |
| CEBPG | 0.201914559 | 5.802333381 | 4.34127508 | 1.75E-05 |
| HELZ | -0.128897354 | 6.072594125 | -4.340748032 | 1.75E-05 |
| SECISBP2 | 0.192277782 | 5.531213639 | 4.340611651 | 1.76E-05 |
| STX16 | -0.172892684 | 7.112622976 | -4.34024298 | 1.76E-05 |
| RIOK1 | 0.161944306 | 5.643566781 | 4.339946733 | 1.76E-05 |
| MBNL1-AS1 | 0.428674181 | 1.199245108 | 4.339283112 | 1.77E-05 |
| SNORD124 | 0.389118237 | 0.984997775 | 4.338898566 | 1.77E-05 |
| CD59 | 0.684613672 | 5.238702119 | 4.337188174 | 1.78E-05 |
| HPCAL1 | -0.243236844 | 5.525112866 | -4.336345809 | 1.79E-05 |
| PXN-AS1 | 0.281357364 | 2.606973046 | 4.336165551 | 1.79E-05 |
| C17orf58 | 0.426482452 | 3.946492317 | 4.335188492 | 1.80E-05 |
| MDH1 | -0.202110816 | 5.54278623 | -4.334363608 | 1.80E-05 |
| AC145212.1 | -0.882353685 | 0.648797559 | -4.334326626 | 1.80E-05 |
| MAN1B1 | -0.146056851 | 5.226884631 | -4.334293551 | 1.80E-05 |
| EHBP1 | -0.228713673 | 3.900829363 | -4.332768133 | 1.82E-05 |
| ZNF383 | 0.180743119 | 3.419900114 | 4.33265101 | 1.82E-05 |
| ZEB1 | 0.566236424 | 3.986430541 | 4.331989469 | 1.82E-05 |
| XIAP | 0.14595693 | 4.494379879 | 4.331237364 | 1.83E-05 |
| RP1-60N8.1 | -0.598770564 | -1.506136381 | -4.331093726 | 1.83E-05 |
| EMC7 | 0.121703124 | 6.856799117 | 4.330737847 | 1.83E-05 |
| RNU6-118P | 0.374762646 | 3.542416866 | 4.330129232 | 1.84E-05 |
| RP11-462G22.1 | -0.700281176 | -1.305499542 | -4.329718305 | 1.84E-05 |
| CTD-2165H16.4 | -0.519380542 | -1.765156896 | -4.328244105 | 1.85E-05 |
| FHL2 | 0.905691193 | -0.236525942 | 4.328227928 | 1.85E-05 |
| RCOR1 | 0.188161984 | 6.606488666 | 4.328171673 | 1.85E-05 |
| HES1 | 0.747795325 | 0.35316663 | 4.32808403 | 1.85E-05 |
| AC005255.3 | -0.35737582 | 4.913000163 | -4.327498311 | 1.86E-05 |
| SLC52A3 | -1.127896641 | -0.490561447 | -4.326407765 | 1.87E-05 |
| CEP72 | 0.247756012 | 2.604349366 | 4.325775186 | 1.87E-05 |
| APCDD1 | -1.002456959 | -0.970616516 | -4.325452987 | 1.88E-05 |
| MDC1 | 0.198181567 | 5.724610595 | 4.325067136 | 1.88E-05 |
| KRT13 | 0.995748721 | -2.199602448 | 4.324343621 | 1.88E-05 |
| MYD88 | -0.309239589 | 7.574647235 | -4.324330405 | 1.88E-05 |
| RN7SL81P | 0.401785608 | 1.25326412 | 4.323241362 | 1.89E-05 |
| RP13-726E6.2 | 0.546487444 | -1.884702736 | 4.323111561 | 1.89E-05 |
| NR4A1 | 0.771064502 | 4.502834324 | 4.321973224 | 1.90E-05 |
| SUV420H2 | 0.231679665 | 3.159758939 | 4.321410619 | 1.91E-05 |
| QRICH2 | -0.383459044 | 1.681879113 | -4.320403003 | 1.92E-05 |
| OCIAD2 | 0.454555794 | 4.633891685 | 4.319507314 | 1.92E-05 |
| ZNF674-AS1 | 0.374721785 | 4.11932279 | 4.317648622 | 1.94E-05 |
| S1PR1 | 0.701309682 | 4.132605651 | 4.317518954 | 1.94E-05 |
| WDR49 | -0.794539276 | 3.288894779 | -4.31743719 | 1.94E-05 |
| LY75 | -0.279586849 | 3.79177304 | -4.316924959 | 1.95E-05 |
| ITGA5 | -0.295650112 | 7.086107665 | -4.316349913 | 1.95E-05 |
| CLMN | -0.609775122 | 2.934641962 | -4.316071211 | 1.95E-05 |
| NR2C1 | 0.181457891 | 4.825793981 | 4.315979434 | 1.95E-05 |
| WDR91 | 0.301964717 | 4.717938287 | 4.315975551 | 1.95E-05 |
| RNU6-925P | -0.470994714 | 1.317194176 | -4.315842723 | 1.96E-05 |
| E4F1 | 0.131323847 | 4.838098378 | 4.315652798 | 1.96E-05 |
| CTC-459F4.1 | -0.449603822 | 1.287004831 | -4.315075623 | 1.96E-05 |
| CTPS2 | -0.252823427 | 4.328405889 | -4.315031579 | 1.96E-05 |
| GRK5 | 0.541303876 | 4.074868901 | 4.31393137 | 1.97E-05 |
| G3BP2 | 0.134227717 | 6.967211481 | 4.313730558 | 1.97E-05 |
| SLCO4A1 | 0.511478196 | 3.275793783 | 4.313467533 | 1.98E-05 |
| BBX | 0.204503634 | 5.087222245 | 4.313229664 | 1.98E-05 |
| PTX4 | -1.003116247 | 0.014329392 | -4.312332311 | 1.99E-05 |
| ATL3 | -0.251880922 | 5.40352327 | -4.311990571 | 1.99E-05 |
| HEXIM2 | -0.36344798 | 2.167863844 | -4.311768448 | 1.99E-05 |
| ST3GAL1 | 0.263787953 | 6.331761589 | 4.31162716 | 1.99E-05 |
| CCDC93 | 0.159739437 | 5.455060828 | 4.311472654 | 1.99E-05 |
| SSTR3 | 0.607617126 | -1.221553908 | 4.309120717 | 2.01E-05 |
| MANF | -0.321132467 | 6.591556626 | -4.308655343 | 2.02E-05 |
| FAM200B | -0.281006247 | 4.186143712 | -4.308438152 | 2.02E-05 |
| ICAM2 | -0.284460377 | 5.495713054 | -4.308128034 | 2.02E-05 |
| RP11-98J23.2 | -0.424578721 | 2.857772897 | -4.307936997 | 2.02E-05 |
| STAT4 | 0.62632183 | 3.291868698 | 4.305953914 | 2.04E-05 |
| SH3BP5 | 0.618403701 | 5.292029727 | 4.305902382 | 2.04E-05 |
| ZBED5 | -0.167856868 | 5.007939145 | -4.305806909 | 2.04E-05 |
| SF3A3P1 | 0.197315976 | 2.627373341 | 4.305517919 | 2.04E-05 |
| ATG13 | 0.147167342 | 6.059812236 | 4.304894088 | 2.05E-05 |
| AP2M1 | -0.150411362 | 7.863004018 | -4.304255193 | 2.06E-05 |
| RP4-785G19.2 | -0.321011271 | 3.797833448 | -4.302761815 | 2.07E-05 |
| ELMOD2 | -0.163332793 | 3.994832212 | -4.302408787 | 2.07E-05 |
| RNF5 | -0.247216774 | 6.335039 | -4.301675169 | 2.08E-05 |
| GINS3 | 0.427434627 | 2.376070759 | 4.301512436 | 2.08E-05 |
| FAM221B | -0.620793456 | -3.062523667 | -4.30107911 | 2.08E-05 |
| SUV420H1 | -0.144878676 | 4.994786728 | -4.300815819 | 2.09E-05 |
| PDE4C | 0.558510556 | -3.650110645 | 4.300756009 | 2.09E-05 |
| XKR7 | -0.628097258 | -2.41438166 | -4.300349013 | 2.09E-05 |
| DHRSX | -0.178203218 | 4.338818641 | -4.300318619 | 2.09E-05 |
| TCL6 | -0.798447752 | -3.147959248 | -4.299796973 | 2.10E-05 |
| CDKN2AIPNLP1 | -0.373301759 | 1.672098147 | -4.299426315 | 2.10E-05 |
| WHAMMP2 | 0.761394942 | 2.685860861 | 4.298405752 | 2.11E-05 |
| ACBD5 | 0.160455106 | 5.077489506 | 4.298140374 | 2.11E-05 |
| OGFOD1P1 | -0.462999748 | -0.22124941 | -4.297635854 | 2.12E-05 |
| RP11-323I15.2 | 0.262848177 | 2.935881199 | 4.297246745 | 2.12E-05 |
| ACSL3 | 0.218010364 | 5.078720758 | 4.297216036 | 2.12E-05 |
| MPHOSPH6 | -0.268510949 | 4.821328186 | -4.295452438 | 2.14E-05 |
| JARID2-AS1 | 0.47679699 | 1.862189584 | 4.295421921 | 2.14E-05 |
| FYTTD1 | 0.149085827 | 6.215228062 | 4.295148239 | 2.14E-05 |
| CBR3 | 0.559010847 | 0.931410591 | 4.295110273 | 2.14E-05 |
| FBXO43 | 0.568525685 | 0.619412253 | 4.294778919 | 2.14E-05 |
| GPX1P1 | -0.336378569 | 6.787044346 | -4.294334081 | 2.15E-05 |
| CCNG2 | 0.297866497 | 5.156701715 | 4.293992101 | 2.15E-05 |
| PELI2 | 0.389805691 | 5.389821055 | 4.29339105 | 2.16E-05 |
| TM2D3 | 0.198492808 | 3.557662319 | 4.293336861 | 2.16E-05 |
| ANKRD36 | 0.276763309 | 3.997187915 | 4.29176984 | 2.17E-05 |
| RP11-512H23.2 | -0.557259646 | -0.472037568 | -4.291300148 | 2.17E-05 |
| PHPT1 | -0.248227336 | 6.35812489 | -4.291190923 | 2.18E-05 |
| GNG12 | 0.725300679 | -2.379813378 | 4.290475827 | 2.18E-05 |
| MSX1 | 0.566444599 | -1.888141127 | 4.290467303 | 2.18E-05 |
| RNU2-59P | -0.632692741 | 2.110011945 | -4.290164983 | 2.19E-05 |
| PLXNB3 | 0.679706611 | -0.564513606 | 4.28967967 | 2.19E-05 |
| TUBBP1 | 0.301677905 | 6.514262648 | 4.289540097 | 2.19E-05 |
| XRCC6P2 | 0.147192726 | 6.056962494 | 4.28880543 | 2.20E-05 |
| RP11-280G9.1 | 0.766365725 | -2.311474166 | 4.287857829 | 2.21E-05 |
| ENPP7P11 | -0.833978905 | 0.702997937 | -4.28779834 | 2.21E-05 |
| RP11-700J17.1 | -0.44567885 | 0.078287277 | -4.28610432 | 2.22E-05 |
| USP12PX | 0.355118094 | 0.970859511 | 4.28532996 | 2.23E-05 |
| PIH1D2 | -0.490976274 | 0.03537734 | -4.285063205 | 2.23E-05 |
| FCRL6 | -0.713205826 | 2.020153955 | -4.283993019 | 2.24E-05 |
| RP11-345K20.2 | -0.277254503 | 2.452497761 | -4.283412339 | 2.25E-05 |
| COX6A1 | 0.16699172 | 5.367984476 | 4.283355652 | 2.25E-05 |
| RP1-140A9.1 | -0.461469022 | 0.849077694 | -4.282820241 | 2.26E-05 |
| FA2H | 0.519140739 | -2.359545449 | 4.281944283 | 2.26E-05 |
| MCF2L2 | 0.517177648 | -1.302642267 | 4.280402021 | 2.28E-05 |
| RNU6ATAC24P | 0.445181335 | 1.99856009 | 4.280018547 | 2.28E-05 |
| HOXB8 | -1.475020043 | -1.166682789 | -4.279878511 | 2.28E-05 |
| FAM203B | -0.549708335 | -2.001700737 | -4.27983155 | 2.29E-05 |
| ZNF605 | -0.254195386 | 3.033989699 | -4.279738965 | 2.29E-05 |
| RP11-710F7.3 | -0.511110007 | -1.781115332 | -4.279089537 | 2.29E-05 |
| RP11-288H12.3 | 0.646193454 | 1.54560564 | 4.279068114 | 2.29E-05 |
| AAR2 | 0.170326416 | 5.536894412 | 4.278732788 | 2.30E-05 |
| BRICD5 | -0.333199699 | 3.058851111 | -4.278554208 | 2.30E-05 |
| VIL1 | 0.587344485 | -2.474914613 | 4.278461276 | 2.30E-05 |
| SUN2 | -0.203517916 | 8.148843289 | -4.278298704 | 2.30E-05 |
| TRAV25 | -0.552526461 | -0.822406292 | -4.277936649 | 2.30E-05 |
| NUTM2A-AS1 | 0.21495073 | 3.731326112 | 4.277659797 | 2.31E-05 |
| TRAPPC6A | -0.253501281 | 6.222873887 | -4.277630148 | 2.31E-05 |
| RP11-182J1.1 | -0.447117324 | 1.169704914 | -4.27721988 | 2.31E-05 |
| PIK3C2A | 0.164242059 | 4.786656204 | 4.275640376 | 2.33E-05 |
| C1orf56 | -0.41717485 | 1.190584012 | -4.275558571 | 2.33E-05 |
| MTCP1 | 0.339797545 | 0.877861045 | 4.27510028 | 2.33E-05 |
| HIST1H2BPS2 | -0.646367452 | -0.300436176 | -4.273032271 | 2.35E-05 |
| EXOC6 | -0.173604559 | 4.909430716 | -4.272899902 | 2.35E-05 |
| EHMT1 | -0.158801971 | 5.406887624 | -4.272794843 | 2.36E-05 |
| RP1-29C18.9 | -0.616240299 | -0.082073108 | -4.272307043 | 2.36E-05 |
| TMEM221 | -0.685108814 | -2.154200899 | -4.272065952 | 2.36E-05 |
| CLN5 | -0.184513853 | 3.886221377 | -4.271964914 | 2.36E-05 |
| HLA-B | 0.262252898 | 11.27730423 | 4.271171181 | 2.37E-05 |
| RP11-411B10.4 | -0.443262515 | 0.416142473 | -4.270777202 | 2.38E-05 |
| TOB1 | 0.427897011 | 7.608648637 | 4.270763963 | 2.38E-05 |
| FBXW2 | -0.124853797 | 5.696591426 | -4.26999507 | 2.38E-05 |
| LRCH1 | 0.189682521 | 4.619327687 | 4.269795777 | 2.39E-05 |
| ENC1 | 0.518528582 | 4.043484383 | 4.269224125 | 2.39E-05 |
| KDM4B | -0.165019077 | 5.305318084 | -4.26808292 | 2.40E-05 |
| SLC13A3 | -0.60453509 | -2.788382395 | -4.267775331 | 2.41E-05 |
| NEK7 | -0.226182879 | 5.824851194 | -4.267592853 | 2.41E-05 |
| RP11-550I24.2 | -0.354520718 | 1.16267264 | -4.267584733 | 2.41E-05 |
| AC007319.1 | 0.596555342 | -1.266870801 | 4.267546871 | 2.41E-05 |
| CTB-95D12.1 | 0.381044775 | -0.382556942 | 4.267476311 | 2.41E-05 |
| RP11-603B24.1 | -0.684147209 | 0.320405809 | -4.267268638 | 2.41E-05 |
| BMPR1A | -0.836926284 | -1.26785966 | -4.26718845 | 2.41E-05 |
| TSSK4 | 0.4686687 | -1.425217512 | 4.266498915 | 2.42E-05 |
| MKNK1 | -0.210076074 | 5.416161646 | -4.266300483 | 2.42E-05 |
| BAG2 | 0.50324376 | 1.761191525 | 4.266175843 | 2.42E-05 |
| TGDS | -0.17436989 | 4.062359029 | -4.265707457 | 2.43E-05 |
| MEGF10 | -0.87483917 | -1.77827794 | -4.264667962 | 2.44E-05 |
| KIF4B | 0.555168224 | -0.973922892 | 4.264438783 | 2.44E-05 |
| DLEC1 | -0.503292985 | -0.15607179 | -4.263427853 | 2.45E-05 |
| AF129075.5 | 0.414343557 | 1.233044287 | 4.263062652 | 2.46E-05 |
| RP11-79P5.9 | -0.440647189 | 0.071084872 | -4.262958041 | 2.46E-05 |
| IL17D | 0.671804501 | -1.20786271 | 4.262714034 | 2.46E-05 |
| ZBTB4 | -0.249832501 | 6.001292332 | -4.262520515 | 2.46E-05 |
| ZNF433 | 0.524192117 | 0.72910283 | 4.262239536 | 2.47E-05 |
| EIF5B | -0.222942003 | 6.151872516 | -4.261739671 | 2.47E-05 |
| RP11-418J17.1 | -0.261523478 | 2.394278734 | -4.261650235 | 2.47E-05 |
| PRSS36 | -0.483905018 | 1.3342563 | -4.261513935 | 2.47E-05 |
| EIF2S3L | -0.206191754 | 7.279753584 | -4.261231458 | 2.48E-05 |
| SIK3-IT1 | 0.460338022 | 2.133065624 | 4.261120016 | 2.48E-05 |
| MIR570 | -0.339603194 | 3.275071063 | -4.260294443 | 2.49E-05 |
| AP3D1 | -0.121329667 | 6.352416036 | -4.259579075 | 2.49E-05 |
| MORF4 | 0.237825859 | 2.946351355 | 4.259483191 | 2.49E-05 |
| CTD-2574D22.2 | -0.394467668 | 0.495119387 | -4.259459654 | 2.50E-05 |
| CTD-3179P9.1 | -1.011901314 | -0.132057278 | -4.258703658 | 2.50E-05 |
| PPIP5K1 | -0.244227142 | 3.380178425 | -4.258417439 | 2.51E-05 |
| DHCR24 | -0.694610385 | 4.34124436 | -4.258273837 | 2.51E-05 |
| RP11-178G16.4 | -0.262915502 | 2.399125193 | -4.258251036 | 2.51E-05 |
| NABP1 | -0.354884399 | 5.041865558 | -4.257823546 | 2.51E-05 |
| MASP2 | 0.526282008 | -3.035242178 | 4.257466673 | 2.52E-05 |
| RP11-79P5.3 | 0.43461088 | 0.241129907 | 4.256879649 | 2.52E-05 |
| AGPAT5 | -0.35353957 | 5.112424764 | -4.256845583 | 2.52E-05 |
| MYOM2 | -0.596929991 | 0.593453136 | -4.256822046 | 2.52E-05 |
| DPYSL2 | -0.71830544 | 4.935131759 | -4.256032243 | 2.53E-05 |
| RP11-333J10.2 | 0.505954964 | 0.113188827 | 4.255834139 | 2.53E-05 |
| LAMP2 | -0.227705249 | 7.704445049 | -4.255621643 | 2.54E-05 |
| AC007386.4 | -0.520853673 | -0.444029781 | -4.255243385 | 2.54E-05 |
| RP11-402D21.2 | -0.450169934 | 1.231061928 | -4.25523562 | 2.54E-05 |
| SEC63 | 0.138812219 | 6.28103122 | 4.253942446 | 2.55E-05 |
| RPS23P8 | -0.274039408 | 10.09615865 | -4.252276866 | 2.57E-05 |
| RN7SL172P | -0.432533476 | 1.358334371 | -4.251853693 | 2.58E-05 |
| USP5 | -0.211417304 | 6.402062857 | -4.251751119 | 2.58E-05 |
| EGR1 | -0.723063538 | 8.141113565 | -4.251714042 | 2.58E-05 |
| KDM5C-IT1 | 0.515275113 | -0.340646584 | 4.251540684 | 2.58E-05 |
| PRPF3 | 0.160092352 | 5.889909037 | 4.249368746 | 2.61E-05 |
| HMGB3P32 | -0.605674481 | 0.391116232 | -4.249101248 | 2.61E-05 |
| KIF11 | 0.512682582 | 4.793424607 | 4.248234578 | 2.62E-05 |
| AC024560.3 | -0.233660895 | 4.057452248 | -4.246952861 | 2.63E-05 |
| PTGFR | -0.987372696 | -2.535896199 | -4.246273054 | 2.64E-05 |
| FLG-AS1 | -0.77229047 | -2.911070728 | -4.246152197 | 2.64E-05 |
| PPP2R2A | 0.158027593 | 4.887661959 | 4.24522517 | 2.65E-05 |
| MSS51 | -0.332877409 | 2.687946769 | -4.245105638 | 2.65E-05 |
| MRPL42P6 | -0.760209473 | -0.448429091 | -4.244989988 | 2.66E-05 |
| EXPH5 | 0.634589056 | -1.453761227 | 4.244602098 | 2.66E-05 |
| AC132192.1 | -0.368618695 | 2.32038277 | -4.244113299 | 2.67E-05 |
| RP11-605F14.2 | 0.310882153 | 0.613610545 | 4.243976074 | 2.67E-05 |
| DHCR7 | 0.440129118 | 4.7618175 | 4.243508212 | 2.67E-05 |
| MANEA | -0.287531936 | 4.136810728 | -4.24331184 | 2.67E-05 |
| RP11-8J9.1 | 0.406807091 | 0.152556311 | 4.243309867 | 2.67E-05 |
| RP11-568N6.1 | -0.410700467 | 3.194107702 | -4.242991502 | 2.68E-05 |
| EIF4HP2 | 0.561951006 | -0.335446875 | 4.242695527 | 2.68E-05 |
| CTC-303L1.2 | -0.449100314 | -0.015805608 | -4.241291933 | 2.70E-05 |
| MYO16 | 0.790887246 | -3.518701816 | 4.240878686 | 2.70E-05 |
| AFAP1 | 0.738370267 | 0.477997156 | 4.240013656 | 2.71E-05 |
| RP11-791G16.4 | 0.214099089 | 3.44435186 | 4.239121722 | 2.72E-05 |
| UGGT1 | -0.212038303 | 6.212813633 | -4.238977608 | 2.72E-05 |
| PRRT1 | 0.484850077 | 0.434977196 | 4.238855725 | 2.73E-05 |
| FAM207BP | -0.268686201 | 4.29875384 | -4.238739562 | 2.73E-05 |
| AC064834.2 | -0.684481118 | -0.382526071 | -4.238720747 | 2.73E-05 |
| MMRN2 | 0.674165853 | -3.39922403 | 4.23844214 | 2.73E-05 |
| TNS1 | 0.572715828 | 2.956154709 | 4.238340626 | 2.73E-05 |
| USP46 | 0.302895988 | 3.133605565 | 4.238280113 | 2.73E-05 |
| RP11-123M21.2 | -0.462302957 | 1.335351763 | -4.237911721 | 2.74E-05 |
| CRCP | 0.142885585 | 5.870779183 | 4.237895209 | 2.74E-05 |
| SPATA7 | -0.292787215 | 2.216526426 | -4.237684361 | 2.74E-05 |
| ADAMTS9 | 0.879256936 | -4.920111843 | 4.237647898 | 2.74E-05 |
| NFAT5 | 0.221171587 | 5.681718247 | 4.237345057 | 2.74E-05 |
| SLC26A4 | -0.499327818 | -2.440887463 | -4.237225642 | 2.74E-05 |
| HIATL1 | -0.33154518 | 6.22141305 | -4.237116122 | 2.75E-05 |
| PRR13 | -0.157708296 | 6.996324948 | -4.236713927 | 2.75E-05 |
| PHB1P8 | -0.451112657 | -0.027697686 | -4.236595071 | 2.75E-05 |
| CXCR5 | 0.686940242 | 0.635343096 | 4.23643784 | 2.75E-05 |
| ZC3H12D | 0.38323714 | 3.016178885 | 4.235784827 | 2.76E-05 |
| YAP1P1 | 0.673781246 | 0.09155142 | 4.235592028 | 2.76E-05 |
| GATA6 | 0.509834007 | -2.926902628 | 4.23456295 | 2.78E-05 |
| GPR171 | -0.643060257 | 1.321198019 | -4.234063226 | 2.78E-05 |
| A2M | 0.986785723 | -0.793713351 | 4.233219235 | 2.79E-05 |
| ACAP1 | -0.261984944 | 6.606373201 | -4.233194122 | 2.79E-05 |
| NICN1-AS1 | -0.478598722 | -0.582806029 | -4.233098371 | 2.79E-05 |
| ACOT1 | 0.472910024 | 1.151012566 | 4.233042037 | 2.79E-05 |
| GALNT1 | -0.195045963 | 6.991215397 | -4.231965443 | 2.81E-05 |
| RBM28 | -0.223386947 | 4.413548902 | -4.231345469 | 2.81E-05 |
| CTD-2308G16.1 | -0.481566386 | -0.5219399 | -4.231053438 | 2.82E-05 |
| AC123768.4 | -0.420908519 | 0.279888515 | -4.230192578 | 2.83E-05 |
| CSNK2A3 | -0.192982536 | 6.915473007 | -4.228970509 | 2.84E-05 |
| RP11-506B6.3 | 0.312860667 | 2.700700346 | 4.228647166 | 2.85E-05 |
| XXbac-BPG248L24.12 | 0.611237958 | 1.827678801 | 4.228128498 | 2.85E-05 |
| CXorf31 | -0.535776914 | -0.106272896 | -4.227681866 | 2.86E-05 |
| SEC63P1 | 0.172080555 | 6.390897459 | 4.227658614 | 2.86E-05 |
| RP3-337H4.6 | 0.299023397 | 2.990313677 | 4.226280716 | 2.88E-05 |
| ESYT2 | -0.272914329 | 7.887019964 | -4.226071744 | 2.88E-05 |
| CCDC41 | -0.242249503 | 3.340462635 | -4.225700211 | 2.88E-05 |
| FAT4 | 0.794550164 | -3.196535594 | 4.225496697 | 2.89E-05 |
| DCAKD | -0.209470591 | 4.981258205 | -4.224602262 | 2.90E-05 |
| MIR3143 | -0.587726893 | -0.39552582 | -4.224124926 | 2.90E-05 |
| ZCRB1 | 0.177440625 | 6.642844044 | 4.2237796 | 2.91E-05 |
| CSNK2A1 | -0.154265751 | 6.640339928 | -4.2236473 | 2.91E-05 |
| FAM46C | 0.680710399 | 5.226358764 | 4.223494335 | 2.91E-05 |
| RGS1 | 0.766290537 | 4.828756918 | 4.223015805 | 2.92E-05 |
| DDC8 | 0.53006313 | 1.415284806 | 4.222787094 | 2.92E-05 |
| TTC5 | -0.227264083 | 3.331464783 | -4.222405284 | 2.92E-05 |
| FAM72B | 0.470833443 | 2.390535898 | 4.221171677 | 2.94E-05 |
| AC011841.1 | -0.791577714 | 2.263958733 | -4.221139708 | 2.94E-05 |
| TPMTP1 | -0.31962244 | 2.211900634 | -4.221040083 | 2.94E-05 |
| C15orf61 | 0.23744915 | 4.350716614 | 4.220444535 | 2.95E-05 |
| AC114730.8 | 0.522364288 | 1.876923369 | 4.220111936 | 2.95E-05 |
| PRPF4B | 0.135927689 | 6.688286974 | 4.219925485 | 2.96E-05 |
| KDM4A-AS1 | -0.506680866 | -0.538796004 | -4.219815829 | 2.96E-05 |
| C18orf54 | -0.339412316 | 2.621838246 | -4.219660013 | 2.96E-05 |
| RP11-10L12.2 | -0.461910164 | -0.68238832 | -4.218738233 | 2.97E-05 |
| KMT2E-AS1 | -0.346005715 | 4.425736712 | -4.218333621 | 2.98E-05 |
| CDK2AP2P2 | -0.570550132 | 0.071466487 | -4.217947698 | 2.98E-05 |
| RP11-350E12.5 | 0.216636736 | 3.039059222 | 4.217460678 | 2.99E-05 |
| DDTL | 0.284665802 | 2.22068307 | 4.216695823 | 3.00E-05 |
| KANK3 | 0.621320599 | -0.514113271 | 4.216534616 | 3.00E-05 |
| IGFALS | 0.490785464 | -0.075303332 | 4.21636363 | 3.00E-05 |
| PERP | 0.728906845 | -0.653599921 | 4.213437153 | 3.04E-05 |
| VASN | 0.735745385 | -0.633607713 | 4.21332073 | 3.04E-05 |
| COX19 | 0.182691617 | 4.43730416 | 4.212613984 | 3.05E-05 |
| CROT | -0.256678412 | 2.400735158 | -4.212445265 | 3.05E-05 |
| MEF2B | -0.352722292 | 0.062030345 | -4.212234958 | 3.05E-05 |
| RP11-391M20.1 | -0.260184084 | 4.096176138 | -4.21187322 | 3.06E-05 |
| OCLM | 0.353563009 | 3.124523715 | 4.211757597 | 3.06E-05 |
| ITGB6 | -0.590643288 | -3.704328659 | -4.211630448 | 3.06E-05 |
| RP11-799B12.4 | -0.489693852 | -0.269319169 | -4.211494891 | 3.06E-05 |
| TAF4 | -0.183770748 | 3.837729671 | -4.21023717 | 3.08E-05 |
| ZNF579 | 0.267862056 | 4.372185786 | 4.209111676 | 3.09E-05 |
| HLF | 0.829253959 | -0.756623376 | 4.20883869 | 3.10E-05 |
| FAM180B | -0.548147521 | -0.095786141 | -4.20758474 | 3.12E-05 |
| KLHDC3 | -0.189034494 | 7.430919142 | -4.207277412 | 3.12E-05 |
| SIGLEC19P | -0.586946675 | -0.680712492 | -4.206818741 | 3.13E-05 |
| CDC27P1 | 0.298732548 | 1.600786784 | 4.206485175 | 3.13E-05 |
| DNAJB6 | 0.286078683 | 5.616218328 | 4.204870373 | 3.15E-05 |
| SMAGP | 0.581161308 | 1.707191742 | 4.20408318 | 3.16E-05 |
| AC138517.4 | -0.401310274 | 1.423189294 | -4.203691262 | 3.17E-05 |
| EIF3A | -0.189279214 | 8.398887611 | -4.203531572 | 3.17E-05 |
| AKNA | -0.249985041 | 6.881018862 | -4.203070516 | 3.18E-05 |
| LINC00672 | 0.417359984 | 2.724166138 | 4.202675991 | 3.18E-05 |
| GANC | -0.219964169 | 4.191089925 | -4.201975048 | 3.19E-05 |
| RP11-443B7.3 | -0.511224256 | 3.008390307 | -4.201410427 | 3.20E-05 |
| HSDL1 | -0.266241014 | 4.273251884 | -4.201388401 | 3.20E-05 |
| BPI | 1.16073171 | 6.079658831 | 4.201356901 | 3.20E-05 |
| AC000068.9 | -0.470970878 | 0.487588223 | -4.200942702 | 3.20E-05 |
| PINK1-AS | 0.208649876 | 4.031932032 | 4.200801209 | 3.21E-05 |
| KLHL31 | -0.590644641 | -1.656080509 | -4.200634057 | 3.21E-05 |
| RP11-363H12.1 | -0.342630468 | 1.727884133 | -4.200310685 | 3.21E-05 |
| HDGFRP3 | 0.902165559 | 3.077005965 | 4.199612932 | 3.22E-05 |
| TTC30B | -0.416985129 | 1.897152916 | -4.199410975 | 3.23E-05 |
| AC105053.4 | -0.562486517 | -0.136637497 | -4.198910752 | 3.23E-05 |
| COL16A1 | 0.730273018 | -3.567525618 | 4.198865022 | 3.23E-05 |
| RHOV | 0.589565075 | -1.490848845 | 4.198332783 | 3.24E-05 |
| RP11-770J1.7 | 0.457829139 | -0.272166011 | 4.198084507 | 3.24E-05 |
| HAL | -0.68742582 | 4.205185451 | -4.197831829 | 3.25E-05 |
| EMC8 | 0.143809945 | 4.476767826 | 4.197144674 | 3.26E-05 |
| SEPT11 | -0.422166612 | 5.78296877 | -4.19697751 | 3.26E-05 |
| ATP6V1G1 | 0.153919559 | 8.582631922 | 4.196827601 | 3.26E-05 |
| AC005154.6 | -0.228495345 | 4.544757212 | -4.196560098 | 3.26E-05 |
| RP11-382J12.1 | 0.515190567 | -2.544014688 | 4.196248416 | 3.27E-05 |
| KCNC4 | -0.364964895 | 1.424167201 | -4.19537999 | 3.28E-05 |
| CASC2 | -0.52259506 | -2.260426732 | -4.195361652 | 3.28E-05 |
| GNMT | 0.32697745 | 3.388054065 | 4.195248747 | 3.28E-05 |
| PGLS | -0.154193589 | 6.752568084 | -4.195020324 | 3.29E-05 |
| HOMER3 | -0.653680104 | 5.294362623 | -4.194414339 | 3.29E-05 |
| ELOVL3 | -0.79581362 | -0.024937111 | -4.194138124 | 3.30E-05 |
| LRIT3 | -0.46099498 | -1.189022359 | -4.193520017 | 3.31E-05 |
| RP11-508M1.7 | -0.859374475 | -0.582132935 | -4.193235592 | 3.31E-05 |
| LINC00680 | -0.280788358 | 3.136529861 | -4.192769088 | 3.32E-05 |
| ZCCHC3 | 0.272679369 | 1.795290095 | 4.192191891 | 3.33E-05 |
| TJP1 | 0.832874373 | -4.677613711 | 4.192110879 | 3.33E-05 |
| SYTL3 | 0.589376199 | 4.479443975 | 4.19152836 | 3.34E-05 |
| MIPEP | -0.292984071 | 3.427585751 | -4.190502922 | 3.35E-05 |
| DLG3 | 0.597514879 | 0.202143252 | 4.190161583 | 3.35E-05 |
| MRPS5 | -0.172717209 | 5.2641351 | -4.190027218 | 3.36E-05 |
| RP1-261G23.7 | 0.515362625 | -0.367447995 | 4.189702428 | 3.36E-05 |
| RASGRP4 | -0.381685395 | 6.711379758 | -4.189648448 | 3.36E-05 |
| DLEU1 | 0.376369727 | 1.609062554 | 4.188512491 | 3.38E-05 |
| GPX1 | -0.300920153 | 10.06810691 | -4.187714309 | 3.39E-05 |
| WNT7B | -1.009489863 | -3.247938949 | -4.187621195 | 3.39E-05 |
| DTHD1 | -0.644532404 | -0.865670569 | -4.187355505 | 3.39E-05 |
| SIRT6 | 0.14774163 | 4.856536441 | 4.187177833 | 3.40E-05 |
| RP11-678B3.2 | 0.241022915 | 2.260531441 | 4.187027243 | 3.40E-05 |
| CIB1 | 0.186495655 | 7.82462664 | 4.186942599 | 3.40E-05 |
| CTD-2547L16.1 | -0.389962569 | -0.626422902 | -4.186524772 | 3.41E-05 |
| FKBP2 | -0.211861243 | 6.584295984 | -4.186485985 | 3.41E-05 |
| BCO2 | -0.493346484 | 0.111966493 | -4.186454751 | 3.41E-05 |
| XXbac-B135H6.15 | -0.574589993 | 3.313381003 | -4.186086443 | 3.41E-05 |
| MPPED1 | -0.741640093 | -3.560083517 | -4.184223615 | 3.44E-05 |
| RALGAPB | -0.107585522 | 5.146040519 | -4.184096593 | 3.44E-05 |
| DKFZP779L1853 | -0.470324866 | -0.847155491 | -4.183204799 | 3.45E-05 |
| PCM1 | -0.176449494 | 6.751742048 | -4.182792495 | 3.46E-05 |
| SIGLEC16 | -0.935769403 | 0.314412299 | -4.182059297 | 3.47E-05 |
| EIF3J | -0.134766227 | 6.596239618 | -4.181652139 | 3.48E-05 |
| UFSP2 | -0.202995578 | 4.437103319 | -4.181638069 | 3.48E-05 |
| RAB12 | 0.25855967 | 5.192539152 | 4.181298283 | 3.48E-05 |
| NIPSNAP3A | -0.210817483 | 6.379202839 | -4.180550045 | 3.49E-05 |
| LONP1 | 0.164283664 | 5.812647523 | 4.179409144 | 3.51E-05 |
| CLN8 | 0.307245852 | 3.998036877 | 4.178769484 | 3.52E-05 |
| C2orf42 | -0.211499567 | 3.500818963 | -4.178535645 | 3.52E-05 |
| B3GALNT1 | -0.554385041 | 3.375854534 | -4.177389557 | 3.54E-05 |
| RP11-758P17.3 | 0.366303984 | 1.660274592 | 4.177162121 | 3.54E-05 |
| RP3-476K8.4 | -0.428315491 | -0.37379547 | -4.176818049 | 3.55E-05 |
| RP11-752L20.1 | -0.472865518 | -0.456045565 | -4.176410779 | 3.56E-05 |
| TDPX2 | -0.477321418 | 0.984173436 | -4.175486089 | 3.57E-05 |
| TSPYL1 | 0.274007399 | 7.304992975 | 4.175361649 | 3.57E-05 |
| AL355490.1 | 0.342766668 | 2.33269833 | 4.175192894 | 3.57E-05 |
| POLB | 0.216705751 | 4.430720366 | 4.174694759 | 3.58E-05 |
| TLR4 | -0.697906094 | 5.606550706 | -4.174444117 | 3.59E-05 |
| NAP1L3 | 0.802108898 | 0.130113396 | 4.17436454 | 3.59E-05 |
| YIPF6 | -0.186387743 | 3.636130883 | -4.174283964 | 3.59E-05 |
| KIAA1279 | -0.23972397 | 4.014380881 | -4.174232034 | 3.59E-05 |
| MEMO1P1 | -0.114191153 | 6.330793985 | -4.17415436 | 3.59E-05 |
| APEX1 | -0.281207266 | 9.325678868 | -4.17375638 | 3.60E-05 |
| BCAS4 | 0.480466618 | 2.082191752 | 4.17353566 | 3.60E-05 |
| RP11-632K20.2 | -0.673318039 | 0.674084184 | -4.17320448 | 3.60E-05 |
| UBE2V1P2 | -0.260881344 | 6.005611244 | -4.172826921 | 3.61E-05 |
| PRR22 | 0.303044648 | 2.100667564 | 4.172757854 | 3.61E-05 |
| RP11-135L13.4 | 0.504723478 | -1.444559872 | 4.172120121 | 3.62E-05 |
| ITGB4 | 0.617661543 | 0.744657088 | 4.171964104 | 3.62E-05 |
| DNAJC7 | -0.158953743 | 5.678378457 | -4.170421231 | 3.65E-05 |
| E2F5 | -0.465541709 | 1.147543931 | -4.170137474 | 3.65E-05 |
| ZNF688 | -0.168579994 | 3.986946552 | -4.16941012 | 3.66E-05 |
| ACTR3 | -0.207950649 | 7.429095251 | -4.168008456 | 3.68E-05 |
| RP13-258O15.1 | -0.288477273 | 6.648922784 | -4.166728257 | 3.70E-05 |
| WRNIP1 | -0.252725394 | 6.588645579 | -4.166344976 | 3.71E-05 |
| LTA | 0.549224731 | 1.551637068 | 4.164995768 | 3.73E-05 |
| SEC22A | -0.192469588 | 2.486591942 | -4.164519317 | 3.74E-05 |
| SUZ12P | -0.236162807 | 4.944667253 | -4.164186777 | 3.74E-05 |
| RP11-295H24.3 | 0.294150962 | 2.856037034 | 4.163360711 | 3.76E-05 |
| DIEXF | -0.244474839 | 3.177219374 | -4.163308793 | 3.76E-05 |
| RP11-1136G11.6 | -0.197656591 | 6.099948361 | -4.163089736 | 3.76E-05 |
| WNT2B | -0.693566442 | -1.940010731 | -4.162956396 | 3.76E-05 |
| SAMD9 | -0.337234892 | 5.579584033 | -4.161400161 | 3.79E-05 |
| DGKG | -0.399248909 | 3.557927908 | -4.161398294 | 3.79E-05 |
| KIAA1456 | -1.207898547 | -1.47979445 | -4.1613239 | 3.79E-05 |
| AC090587.5 | -0.413513663 | -0.05069103 | -4.160994822 | 3.79E-05 |
| RP11-276H19.4 | -0.355414955 | 0.432982044 | -4.160720552 | 3.80E-05 |
| CTD-2611O12.7 | 0.427931167 | 1.249289734 | 4.160327018 | 3.81E-05 |
| TOMM20L | 0.440440175 | 0.638982251 | 4.159957685 | 3.81E-05 |
| ABCG2 | 0.696385222 | -1.049668403 | 4.159746677 | 3.81E-05 |
| PYGL | -0.383369061 | 7.762397743 | -4.159365553 | 3.82E-05 |
| RP11-557L19.1 | -0.48984883 | -0.663050528 | -4.158964076 | 3.83E-05 |
| TIPARP | 0.404949225 | 6.016232881 | 4.158642166 | 3.83E-05 |
| BEX2 | 0.866139612 | 3.57077677 | 4.157986619 | 3.84E-05 |
| UBAC1 | 0.2321853 | 6.135944557 | 4.156646168 | 3.87E-05 |
| C12orf75 | 0.484133463 | 3.99621253 | 4.156496229 | 3.87E-05 |
| LBP | 0.693484438 | -1.650586368 | 4.156430596 | 3.87E-05 |
| TIMM17A | 0.182135872 | 5.568718566 | 4.156125174 | 3.87E-05 |
| HPN | 0.522827748 | -2.580786382 | 4.156093336 | 3.87E-05 |
| C1QTNF1 | 0.75567223 | -3.090007361 | 4.15607796 | 3.87E-05 |
| TRMT5 | -0.312623488 | 2.453261536 | -4.155914985 | 3.88E-05 |
| LASP1 | -0.187968046 | 7.950616468 | -4.155746383 | 3.88E-05 |
| INTS5 | -0.236179103 | 4.591172455 | -4.155518392 | 3.88E-05 |
| PARD3B | 0.936133358 | -3.461011466 | 4.15534135 | 3.89E-05 |
| TMEM261 | -0.191035203 | 4.82602642 | -4.155150945 | 3.89E-05 |
| C1orf159 | 0.171083477 | 3.083612237 | 4.154698326 | 3.90E-05 |
| WASF3 | 0.804798536 | -1.04734948 | 4.154415296 | 3.90E-05 |
| POLR3K | -0.253018153 | 4.398864036 | -4.15424335 | 3.90E-05 |
| UBE2L3 | -0.1447559 | 6.617816969 | -4.154211429 | 3.90E-05 |
| ZC3H11B | 0.139364885 | 4.203526626 | 4.153539898 | 3.92E-05 |
| RP11-308N19.5 | -0.359961891 | 1.049382237 | -4.153277029 | 3.92E-05 |
| GABPA | 0.147633066 | 5.494278598 | 4.152694406 | 3.93E-05 |
| AP000351.10 | -0.602245093 | 1.42045876 | -4.15190165 | 3.94E-05 |
| MORC2 | -0.170444203 | 4.976602764 | -4.151862585 | 3.94E-05 |
| SPACA6P | 0.504794675 | 0.947406665 | 4.15121241 | 3.95E-05 |
| SF3A3P2 | 0.189426806 | 3.216487906 | 4.150780739 | 3.96E-05 |
| RP3-510D11.2 | -0.679953296 | 0.215758118 | -4.150547033 | 3.97E-05 |
| DDX21 | 0.215072764 | 7.909708708 | 4.150004303 | 3.97E-05 |
| MAP1B | 0.816533694 | -4.907874526 | 4.149962404 | 3.98E-05 |
| GIMAP5 | -0.650399661 | 2.76712679 | -4.149137829 | 3.99E-05 |
| MEA1 | -0.190732097 | 6.970108822 | -4.14894521 | 3.99E-05 |
| LRFN1 | -0.35683282 | 3.485729784 | -4.148475507 | 4.00E-05 |
| RP11-345M22.1 | -0.647671931 | -2.041239832 | -4.148232553 | 4.00E-05 |
| NFKBIA | 0.473679989 | 9.932842288 | 4.148152028 | 4.01E-05 |
| RGS9BP | -0.648554755 | -1.032688578 | -4.148076391 | 4.01E-05 |
| PRKCH | 0.489604731 | 4.381716086 | 4.147620873 | 4.01E-05 |
| MBNL3 | -0.249511116 | 5.007222849 | -4.146654694 | 4.03E-05 |
| RRP1B | -0.224847487 | 5.504346649 | -4.146261114 | 4.04E-05 |
| MAFK | 0.290859676 | 5.095689042 | 4.144740026 | 4.06E-05 |
| VPS37C | 0.316724731 | 3.860803935 | 4.143180898 | 4.09E-05 |
| PLIN4 | 0.808024708 | 0.163862721 | 4.142881158 | 4.10E-05 |
| RP11-156E6.1 | 0.407428106 | 5.027956689 | 4.142151424 | 4.11E-05 |
| AC129492.6 | -0.592501814 | -0.192879358 | -4.141821772 | 4.11E-05 |
| RP11-149I2.5 | -0.553598434 | -0.816506529 | -4.141543957 | 4.12E-05 |
| IRS2 | 0.566869868 | 6.266226211 | 4.141357385 | 4.12E-05 |
| ALMS1 | 0.144597862 | 4.479589872 | 4.140801849 | 4.13E-05 |
| ITGB1BP1 | -0.17911374 | 4.835983074 | -4.140755859 | 4.13E-05 |
| NOSIP | -0.152687984 | 5.017829463 | -4.140661329 | 4.13E-05 |
| SLC16A6 | 0.807623646 | 1.988451165 | 4.139824006 | 4.15E-05 |
| ULBP2 | 0.695257136 | -0.295430993 | 4.139480859 | 4.15E-05 |
| SNORA2B | 0.42804762 | 1.797457367 | 4.138990998 | 4.16E-05 |
| MTX3 | 0.614604611 | 2.557260211 | 4.138707737 | 4.17E-05 |
| RN7SL481P | 0.423033432 | 2.908150122 | 4.138031594 | 4.18E-05 |
| ABLIM1 | 0.600920871 | 4.550244836 | 4.137987341 | 4.18E-05 |
| WDR33 | 0.115794803 | 4.937312983 | 4.137979847 | 4.18E-05 |
| RP11-175P13.3 | 0.385392028 | 1.087130083 | 4.137890269 | 4.18E-05 |
| ESPN | 0.81888952 | -0.588326074 | 4.137234343 | 4.19E-05 |
| ATP5C1 | -0.165545525 | 7.956082533 | -4.137089968 | 4.20E-05 |
| ABCA9 | -0.779759572 | -1.208112132 | -4.136251842 | 4.21E-05 |
| ROR1 | 0.582969961 | -3.888218838 | 4.1357204 | 4.22E-05 |
| TIMM21 | -0.2004478 | 3.957390771 | -4.135713278 | 4.22E-05 |
| SGPP1 | 0.691277424 | 3.272251142 | 4.135525804 | 4.22E-05 |
| PXN | 0.428826409 | 6.544433174 | 4.135426706 | 4.23E-05 |
| RP11-225H22.4 | -0.517171303 | -0.760910786 | -4.135426122 | 4.23E-05 |
| GP6 | 0.764378216 | 0.397662589 | 4.135246493 | 4.23E-05 |
| RP11-218M22.1 | -0.2724791 | 4.600813989 | -4.134707495 | 4.24E-05 |
| MNT | 0.179770432 | 4.933599465 | 4.134500225 | 4.24E-05 |
| MISP | 0.640334616 | -2.69105351 | 4.134041531 | 4.25E-05 |
| TAF15 | -0.17775116 | 7.441628922 | -4.134004404 | 4.25E-05 |
| ULK4P2 | 0.467264878 | 1.821579449 | 4.133983923 | 4.25E-05 |
| ASUN | -0.233841511 | 5.673876675 | -4.133671452 | 4.26E-05 |
| LINC00402 | -0.736114809 | -0.425631336 | -4.133470523 | 4.26E-05 |
| RN7SKP97 | -0.509330637 | 0.292828785 | -4.133357488 | 4.26E-05 |
| TRIM62 | 0.275552216 | 3.636165831 | 4.13269527 | 4.27E-05 |
| RP11-982M15.8 | -0.556143037 | -2.936356468 | -4.132317951 | 4.28E-05 |
| RN7SL381P | -0.509369946 | 2.042907587 | -4.132105908 | 4.28E-05 |
| BX649553.2 | 0.538881934 | 0.776169196 | 4.131982603 | 4.29E-05 |
| CCL1 | -1.137775893 | -0.282732657 | -4.131272396 | 4.30E-05 |
| RP11-64K12.10 | -0.528878907 | -1.33246924 | -4.130446659 | 4.31E-05 |
| CTSS | -0.556726192 | 8.753471278 | -4.130405314 | 4.32E-05 |
| RP4-779E11.3 | 0.537860936 | -0.107117961 | 4.130135958 | 4.32E-05 |
| TSC22D4 | -0.187036196 | 6.979266378 | -4.12993017 | 4.32E-05 |
| PSMB10 | -0.213156481 | 6.071827504 | -4.129876184 | 4.33E-05 |
| NEIL1 | 0.595532387 | 1.502906867 | 4.129773136 | 4.33E-05 |
| KIAA1143 | -0.142622275 | 5.134450792 | -4.129345011 | 4.33E-05 |
| FAM175A | -0.233980193 | 4.536357673 | -4.128695229 | 4.35E-05 |
| GPATCH1 | -0.196860717 | 3.465515195 | -4.128581948 | 4.35E-05 |
| ETV1 | 0.733784374 | -3.824443908 | 4.128541598 | 4.35E-05 |
| INTS6P1 | -0.234559408 | 2.600814281 | -4.128093437 | 4.36E-05 |
| ADAM9 | 0.506611787 | 4.883350489 | 4.127763806 | 4.36E-05 |
| WNT3 | 0.54238352 | -2.923847773 | 4.127474649 | 4.37E-05 |
| GPR125 | -0.520883744 | 3.271440171 | -4.127406547 | 4.37E-05 |
| C1orf101 | -0.364823841 | 0.76916942 | -4.127381638 | 4.37E-05 |
| HOOK1 | 0.728533485 | 0.385826174 | 4.127326878 | 4.37E-05 |
| CXCR4 | 0.569487309 | 9.389035691 | 4.127261986 | 4.37E-05 |
| BCAS2P2 | 0.275205286 | 2.898647301 | 4.127174772 | 4.37E-05 |
| CDK8 | -0.175428528 | 4.562322141 | -4.127053454 | 4.38E-05 |
| OSTCP4 | -0.337671626 | 2.186734449 | -4.126348003 | 4.39E-05 |
| ASB1 | -0.216446539 | 4.221006398 | -4.126136395 | 4.39E-05 |
| RP11-711M9.1 | 0.258171825 | 3.566771604 | 4.125762213 | 4.40E-05 |
| C10orf32 | 0.21302576 | 4.862248106 | 4.125575169 | 4.40E-05 |
| TMEM92 | -0.812974096 | -1.095734881 | -4.125344212 | 4.41E-05 |
| SIPA1L2 | 0.729199015 | 2.757351228 | 4.124824054 | 4.42E-05 |
| DHX30 | 0.149427864 | 5.505932465 | 4.124122683 | 4.43E-05 |
| SERPINB2 | 1.116606225 | 3.678836545 | 4.123718261 | 4.44E-05 |
| ANKRD23 | -0.388742131 | 0.813414499 | -4.123618985 | 4.44E-05 |
| RP11-310E22.5 | -0.559824992 | -0.684439551 | -4.123484081 | 4.44E-05 |
| SPPL3 | 0.166541816 | 5.075395862 | 4.123352761 | 4.44E-05 |
| GUSBP1 | -0.228261079 | 2.639769135 | -4.122975898 | 4.45E-05 |
| RMDN1 | -0.176081454 | 4.870389537 | -4.122510576 | 4.46E-05 |
| CNDP1 | 0.570913671 | -3.331757086 | 4.122247021 | 4.47E-05 |
| PPP4R1 | 0.15221717 | 5.975676847 | 4.12223872 | 4.47E-05 |
| FAM85A | -0.509710059 | -0.51473125 | -4.121339544 | 4.48E-05 |
| DEPDC4 | -0.427368045 | -0.272523221 | -4.120972626 | 4.49E-05 |
| CTC-265F19.1 | -0.495539505 | -1.890862333 | -4.120606033 | 4.50E-05 |
| RCVRN | 0.607410782 | -0.281720374 | 4.12058144 | 4.50E-05 |
| ZNF213 | -0.209331778 | 3.618609555 | -4.120507584 | 4.50E-05 |
| F2RL2 | 0.661990122 | -2.918365568 | 4.120293081 | 4.50E-05 |
| DLGAP1-AS2 | 0.502371681 | 2.164975844 | 4.120166323 | 4.50E-05 |
| NFE2L3 | -0.573468517 | 3.206736491 | -4.119662125 | 4.51E-05 |
| HECTD2 | 0.529981808 | 0.331113641 | 4.119350725 | 4.52E-05 |
| ZC2HC1C | -0.588052095 | -1.620808532 | -4.11932402 | 4.52E-05 |
| GS1-124K5.3 | -0.280871342 | 2.839023107 | -4.119025745 | 4.53E-05 |
| RP11-727F15.12 | -0.217706717 | 2.34783584 | -4.118921858 | 4.53E-05 |
| PAPD4 | 0.118612187 | 6.551073874 | 4.118702655 | 4.53E-05 |
| TCTN1 | -0.370462068 | 2.62034457 | -4.118053534 | 4.54E-05 |
| KRI1 | -0.229712371 | 6.104293003 | -4.118007108 | 4.55E-05 |
| DICER1 | -0.16482509 | 5.920358444 | -4.117854904 | 4.55E-05 |
| CNOT6L | 0.189935119 | 5.84654712 | 4.11771769 | 4.55E-05 |
| NUMA1 | -0.176457756 | 7.420873188 | -4.117379213 | 4.56E-05 |
| RP11-573D15.8 | -0.521091674 | -1.92132135 | -4.117143944 | 4.56E-05 |
| ANXA6 | -0.387414338 | 7.500685069 | -4.116544134 | 4.57E-05 |
| SDSL | -0.486356781 | 3.651467067 | -4.116496195 | 4.57E-05 |
| MYSM1 | 0.133056521 | 5.840509075 | 4.116458189 | 4.57E-05 |
| TRAV41 | -0.550222082 | -0.609292175 | -4.116180516 | 4.58E-05 |
| RP1-265C24.8 | 0.487938096 | -1.044499288 | 4.115584391 | 4.59E-05 |
| ATP5A1P2 | -0.22951067 | 4.506593911 | -4.11439119 | 4.61E-05 |
| TRAV8-4 | -0.603443802 | -0.283240865 | -4.114019661 | 4.62E-05 |
| NDUFB7 | -0.217675288 | 7.623187773 | -4.112829838 | 4.64E-05 |
| HDAC6 | -0.173691929 | 5.438765656 | -4.11274124 | 4.65E-05 |
| RAC3 | 0.41133003 | 4.238800272 | 4.112437089 | 4.65E-05 |
| CTB-133G6.1 | -0.335126036 | 5.165118377 | -4.112260268 | 4.66E-05 |
| RP11-618I10.4 | -0.644834966 | -1.475670721 | -4.11193785 | 4.66E-05 |
| A4GALT | 1.007725794 | 0.677491511 | 4.110827946 | 4.68E-05 |
| TIMM8A | -0.188702817 | 3.751784128 | -4.110509551 | 4.69E-05 |
| TRAV26-2 | -0.518666613 | -0.913374936 | -4.110494627 | 4.69E-05 |
| VEGFB | 0.212633636 | 5.634200259 | 4.109756872 | 4.70E-05 |
| FAM204BP | 0.370209764 | 0.514112076 | 4.109520634 | 4.71E-05 |
| GPNMB | 0.878326689 | 0.594992007 | 4.108600676 | 4.73E-05 |
| SOD2 | 0.415951639 | 6.621213209 | 4.107899156 | 4.74E-05 |
| AMICA1 | -0.804932297 | 4.806427037 | -4.107564952 | 4.75E-05 |
| SNAI2 | 0.789060492 | -2.254614183 | 4.106306917 | 4.77E-05 |
| RP11-203F10.6 | 0.271946857 | 2.983303386 | 4.106271122 | 4.77E-05 |
| FAM167A | 0.697184586 | -0.483344029 | 4.105294401 | 4.79E-05 |
| NRARP | 0.616137412 | 2.111010561 | 4.105110206 | 4.80E-05 |
| GAPDHP73 | 0.386422494 | 1.147051751 | 4.10429938 | 4.81E-05 |
| CTD-2081C10.7 | -0.452253247 | -0.164293985 | -4.104075271 | 4.82E-05 |
| XRN2 | -0.1420454 | 7.881335324 | -4.103685069 | 4.83E-05 |
| MBOAT1 | -0.235560997 | 5.444183269 | -4.10325828 | 4.83E-05 |
| RP5-1021I20.6 | 0.425833238 | 0.821500581 | 4.103121689 | 4.84E-05 |
| RP11-348N5.7 | 0.419689248 | 1.995740654 | 4.103112866 | 4.84E-05 |
| TAF9P3 | -0.406743795 | 0.283233663 | -4.102687428 | 4.85E-05 |
| PARL | -0.161261703 | 6.884522024 | -4.102530019 | 4.85E-05 |
| TIMM50 | 0.173356866 | 4.871436935 | 4.102379772 | 4.85E-05 |
| KIRREL | 1.166548049 | -1.568537377 | 4.102376855 | 4.85E-05 |
| HSPB6 | 0.744173792 | -0.453894161 | 4.101826194 | 4.86E-05 |
| APBB1 | 0.618268695 | 1.927213425 | 4.101767306 | 4.86E-05 |
| LINC00963 | 0.313256974 | 4.362859734 | 4.101725873 | 4.87E-05 |
| SNRPN | 0.683242148 | 1.156988078 | 4.101439544 | 4.87E-05 |
| AIFM1 | -0.174255758 | 5.566587465 | -4.100307678 | 4.89E-05 |
| AL645728.1 | 0.308399274 | 1.947018818 | 4.100166975 | 4.90E-05 |
| ACSM3 | -0.498287817 | 4.017683612 | -4.099439776 | 4.91E-05 |
| ZSWIM1 | 0.223587097 | 4.588781846 | 4.099174961 | 4.92E-05 |
| NGFR | 0.702392501 | -0.509347805 | 4.097325224 | 4.96E-05 |
| RP11-1036E20.9 | 0.55747599 | -2.112285938 | 4.097195532 | 4.96E-05 |
| RP11-142C4.4 | -0.699574403 | -0.110317651 | -4.096497986 | 4.97E-05 |
| TIMM13 | -0.227974782 | 6.407348652 | -4.095674801 | 4.99E-05 |
| MT1XP1 | 0.419108077 | 1.295226571 | 4.094871471 | 5.01E-05 |
| C7orf49 | -0.209022267 | 4.733894391 | -4.094661156 | 5.01E-05 |
| MDM1 | -0.202784422 | 4.311561069 | -4.094586595 | 5.01E-05 |
| DAP3P2 | -0.390406876 | 0.688041537 | -4.094215002 | 5.02E-05 |
| CFP | -0.465506031 | 6.879205385 | -4.094052382 | 5.02E-05 |
| IFT140 | -0.315833132 | 3.471162223 | -4.093717998 | 5.03E-05 |
| PTPDC1 | -0.426167792 | 1.698948397 | -4.093612989 | 5.03E-05 |
| PRPS1P2 | 0.294198326 | 0.692115984 | 4.093241856 | 5.04E-05 |
| RP11-138A9.1 | 0.597062563 | -0.631647454 | 4.093062892 | 5.04E-05 |
| RP11-480D4.2 | -0.539079656 | -1.356363542 | -4.092465221 | 5.06E-05 |
| PHF6 | -0.243784544 | 4.456430363 | -4.092103557 | 5.06E-05 |
| METTL7B | -0.964941985 | 2.756524332 | -4.091666269 | 5.07E-05 |
| CHKA | 0.257992355 | 5.562927001 | 4.091647382 | 5.07E-05 |
| ASB13 | -0.30882294 | 4.908610297 | -4.091215549 | 5.08E-05 |
| EIF5A | 0.197123802 | 8.494131958 | 4.090695917 | 5.09E-05 |
| KIAA0895 | 0.601499301 | 0.86268512 | 4.090637427 | 5.09E-05 |
| LY6K | 0.81880563 | -2.352459456 | 4.090471929 | 5.10E-05 |
| ZNF702P | -0.587932538 | 0.730501969 | -4.089695158 | 5.11E-05 |
| LRIG1 | 0.611402802 | 2.040985265 | 4.089377331 | 5.12E-05 |
| RP11-73M18.7 | -0.229075983 | 5.288306146 | -4.088187759 | 5.15E-05 |
| RNU6-757P | 0.450583368 | 0.07503308 | 4.087168683 | 5.17E-05 |
| C1orf204 | -0.467089456 | 0.895604894 | -4.085851584 | 5.20E-05 |
| MYEOV | 0.604464086 | -2.695067607 | 4.084076054 | 5.24E-05 |
| RP11-365O16.1 | -0.2277844 | 6.033257196 | -4.082412498 | 5.27E-05 |
| USP46-AS1 | -0.337818279 | 2.630637167 | -4.082304421 | 5.27E-05 |
| RP11-536C10.12 | -0.274571472 | 2.239839157 | -4.081518729 | 5.29E-05 |
| H2AFZ | 0.247061261 | 7.476598927 | 4.081446619 | 5.29E-05 |
| CCL4L1 | 0.666997369 | 0.520363691 | 4.079730585 | 5.33E-05 |
| ARAP1 | -0.248810115 | 7.021924836 | -4.079670752 | 5.33E-05 |
| CCDC86 | -0.258808686 | 5.483094315 | -4.07890128 | 5.35E-05 |
| SLC25A21 | 0.778978118 | -2.909966204 | 4.078747308 | 5.35E-05 |
| RAD51C | -0.22368977 | 3.919530999 | -4.078382971 | 5.36E-05 |
| PHB1 | -0.226936595 | 5.944101344 | -4.077695019 | 5.38E-05 |
| SIDT1 | 0.43668896 | 2.565205604 | 4.077693987 | 5.38E-05 |
| EIF5 | 0.196579539 | 7.425440145 | 4.077467898 | 5.38E-05 |
| CTSK | 0.348780344 | 4.302583596 | 4.077262001 | 5.39E-05 |
| CCDC136 | -0.650328814 | 1.685626866 | -4.07719696 | 5.39E-05 |
| EMR1 | -0.632284338 | 5.255634326 | -4.076998233 | 5.39E-05 |
| TBRG4 | -0.191166221 | 4.893839152 | -4.076706286 | 5.40E-05 |
| ZNF438 | -0.348720757 | 4.319432418 | -4.07658225 | 5.40E-05 |
| STK39 | -0.20908266 | 4.102743357 | -4.076119476 | 5.41E-05 |
| IPO11 | -0.271428254 | 3.681522821 | -4.076112331 | 5.41E-05 |
| KB-431C1.4 | -0.323621035 | 4.691851889 | -4.076001656 | 5.41E-05 |
| FKBP8 | 0.163236586 | 8.348036315 | 4.075490861 | 5.43E-05 |
| MIR4435-1HG | 0.343648427 | 3.189325397 | 4.075237597 | 5.43E-05 |
| AC026806.2 | -0.56802713 | 2.954607594 | -4.074744666 | 5.44E-05 |
| RP11-214K3.19 | -0.46522936 | 0.402733877 | -4.074576497 | 5.45E-05 |
| IPO7P2 | -0.209650428 | 4.605276223 | -4.07453132 | 5.45E-05 |
| RP5-977B1.7 | -0.441584846 | 1.406388179 | -4.074325043 | 5.45E-05 |
| TANGO2 | -0.201811761 | 4.832891623 | -4.074319997 | 5.45E-05 |
| GAPDHP60 | 0.236216711 | 6.756986911 | 4.074108165 | 5.46E-05 |
| COL11A2 | 0.339885338 | 0.23596837 | 4.074005576 | 5.46E-05 |
| ADPRHL1 | -0.476103167 | 0.599814691 | -4.074001539 | 5.46E-05 |
| HSD17B1 | -0.444183791 | 0.142844571 | -4.073911262 | 5.46E-05 |
| PVRL2 | 0.491749143 | 5.424619008 | 4.073910724 | 5.46E-05 |
| BMF | 0.411437398 | 5.470543223 | 4.073345984 | 5.47E-05 |
| C6orf58 | -0.511362256 | -1.020574932 | -4.073208422 | 5.48E-05 |
| OGFOD1 | -0.214540609 | 4.649216657 | -4.072916429 | 5.48E-05 |
| PKIG | 0.406305438 | 4.821044994 | 4.07273868 | 5.49E-05 |
| TMEM249 | -0.452333599 | -1.783411397 | -4.071974046 | 5.51E-05 |
| CAB39 | -0.175726667 | 6.944352265 | -4.071099164 | 5.53E-05 |
| PPP1CA | -0.161050144 | 8.463539665 | -4.070773029 | 5.53E-05 |
| CXCL3 | 0.911160091 | 3.672976733 | 4.069580502 | 5.56E-05 |
| PPP4R4 | 0.629813051 | -2.947405711 | 4.068955475 | 5.57E-05 |
| AC026202.3 | -0.417149083 | -0.016448937 | -4.0688874 | 5.58E-05 |
| LINC00908 | -0.566884472 | -1.856447572 | -4.068716235 | 5.58E-05 |
| AP000347.2 | 0.281282238 | 2.132133679 | 4.068706639 | 5.58E-05 |
| NXPH4 | 0.501192538 | -1.94207874 | 4.067399996 | 5.61E-05 |
| AC007040.8 | -0.659553628 | -0.828041468 | -4.066775758 | 5.62E-05 |
| GS1-393G12.13 | -0.461357154 | -0.923138969 | -4.06666815 | 5.63E-05 |
| RPIA | 0.200707889 | 6.880950085 | 4.066583203 | 5.63E-05 |
| ZSCAN12 | 0.279209031 | 3.43968322 | 4.066530932 | 5.63E-05 |
| RPA1 | -0.202189553 | 6.392643053 | -4.06614227 | 5.64E-05 |
| ZNF829 | 0.390607389 | 1.396731044 | 4.065853256 | 5.65E-05 |
| TERF1P5 | 0.20370813 | 2.43918504 | 4.065584051 | 5.65E-05 |
| UTP23 | 0.150559017 | 4.488264617 | 4.065577064 | 5.65E-05 |
| KIAA0895L | -0.269043201 | 4.262337302 | -4.065418843 | 5.66E-05 |
| RN7SL145P | -0.419181338 | 1.202833432 | -4.065114281 | 5.66E-05 |
| CBLB | 0.232392956 | 4.032282348 | 4.063995221 | 5.69E-05 |
| MBOAT7 | -0.295287623 | 7.903052898 | -4.063765044 | 5.70E-05 |
| NEK6 | -0.304433777 | 5.961536136 | -4.063393537 | 5.70E-05 |
| ID2 | 0.538617973 | 7.615644967 | 4.063163683 | 5.71E-05 |
| RP11-346D6.6 | -0.795293125 | 0.369185678 | -4.062818754 | 5.72E-05 |
| YARS2 | -0.175892294 | 4.920874764 | -4.062353083 | 5.73E-05 |
| SELL | -0.545889982 | 9.397504041 | -4.061892224 | 5.74E-05 |
| TRIM29 | 0.97487693 | -4.519681807 | 4.061841834 | 5.74E-05 |
| TBC1D22A | -0.13483734 | 5.042519571 | -4.06168399 | 5.74E-05 |
| DLGAP1-AS3 | -0.669734007 | -1.016311362 | -4.061425713 | 5.75E-05 |
| GTF2E1 | -0.285798915 | 3.945822407 | -4.060874858 | 5.76E-05 |
| RP11-91J19.4 | -0.403278409 | 2.047708043 | -4.060766332 | 5.77E-05 |
| TMEM198B | -0.295801434 | 5.70573806 | -4.060749924 | 5.77E-05 |
| RP11-304F15.3 | -0.374018319 | 1.193408036 | -4.060564538 | 5.77E-05 |
| AC009495.3 | -0.48500243 | -0.279718346 | -4.060555296 | 5.77E-05 |
| SPATA6 | -0.738792826 | 1.948331795 | -4.059173633 | 5.80E-05 |
| FAM162A | 0.226073769 | 4.305325549 | 4.058767221 | 5.81E-05 |
| C8orf44 | -0.297001965 | 3.719187172 | -4.058657725 | 5.82E-05 |
| RN7SL559P | -0.452063099 | -0.112981363 | -4.057006615 | 5.86E-05 |
| ATP11A | -0.269474183 | 5.681610446 | -4.05590646 | 5.88E-05 |
| NEU1 | -0.197120209 | 5.516257444 | -4.05549184 | 5.89E-05 |
| RP11-800A3.4 | -0.691589123 | 2.988631581 | -4.055464704 | 5.89E-05 |
| USP10 | -0.158435411 | 6.279730598 | -4.055434667 | 5.89E-05 |
| TMEM2 | 0.427159885 | 4.308349757 | 4.055162794 | 5.90E-05 |
| RP11-930O11.1 | 0.56761297 | 0.137997118 | 4.054975406 | 5.91E-05 |
| FN3K | 0.566440731 | 1.457773891 | 4.054613055 | 5.91E-05 |
| AP000692.9 | 0.380873228 | 1.142325928 | 4.054372957 | 5.92E-05 |
| PCDHB5 | 0.688758628 | -2.910473812 | 4.053615729 | 5.94E-05 |
| CCDC120 | 0.25004568 | 3.038801635 | 4.052812122 | 5.96E-05 |
| H2AFJ | 0.540540335 | 4.839637136 | 4.052762108 | 5.96E-05 |
| UBC | 0.213912437 | 10.31966755 | 4.051901776 | 5.98E-05 |
| CTD-2324F15.2 | -0.522197632 | -1.243536763 | -4.051858504 | 5.98E-05 |
| AC005831.1 | 0.487702928 | -0.536435181 | 4.051785736 | 5.98E-05 |
| MVD | 0.205845672 | 4.41659521 | 4.051463332 | 5.99E-05 |
| LINC00623 | -0.418907412 | -0.956547255 | -4.05139695 | 5.99E-05 |
| QRFP | -0.626388905 | -0.277040438 | -4.050996502 | 6.00E-05 |
| RP11-334J6.6 | 0.399291936 | -0.234830243 | 4.050791304 | 6.01E-05 |
| ARHGDIA | 0.14022366 | 9.522087565 | 4.050235171 | 6.02E-05 |
| LRRIQ4 | -0.500209153 | -1.679822037 | -4.050162149 | 6.02E-05 |
| ANKRD50 | -0.338610467 | 4.594931699 | -4.049799656 | 6.03E-05 |
| FAM107B | 0.451177949 | 7.16949515 | 4.049705657 | 6.04E-05 |
| MSTO2P | 0.281296042 | 1.698807098 | 4.049247452 | 6.05E-05 |
| RP11-293M10.5 | -0.570097109 | 1.084513261 | -4.048603947 | 6.06E-05 |
| BAMBI | 0.87032716 | 2.241974507 | 4.048115215 | 6.08E-05 |
| ZNF70 | -0.350465489 | 3.905263804 | -4.048068761 | 6.08E-05 |
| RP11-214K3.20 | -0.496494108 | 0.869839877 | -4.048044184 | 6.08E-05 |
| PRSS33 | 0.713546265 | -1.442422392 | 4.047941418 | 6.08E-05 |
| FAM171B | 0.921528179 | 0.26759199 | 4.047687094 | 6.09E-05 |
| SFPQ | 0.235528836 | 8.841726778 | 4.04760925 | 6.09E-05 |
| TXNDC5 | 0.28351416 | 2.220040334 | 4.046850517 | 6.11E-05 |
| COL1A2 | 1.081906003 | -3.584920935 | 4.046391988 | 6.12E-05 |
| FRA10AC1 | -0.186064031 | 3.91350721 | -4.045514433 | 6.14E-05 |
| CUBN | -0.42276433 | 1.24721532 | -4.045075729 | 6.15E-05 |
| LY86-AS1 | -0.651531841 | -2.778800539 | -4.045036893 | 6.15E-05 |
| RP11-191L17.1 | -0.429343401 | 0.315514245 | -4.045024308 | 6.15E-05 |
| GOLIM4 | -0.297516632 | 5.682805709 | -4.044819972 | 6.16E-05 |
| OSBPL6 | 0.774687718 | -2.15385824 | 4.044486488 | 6.17E-05 |
| NPEPL1 | 0.263988966 | 3.723646069 | 4.043520994 | 6.19E-05 |
| RPL21P120 | 0.242799836 | 2.053950173 | 4.042377665 | 6.22E-05 |
| ZKSCAN8P1 | -0.392387749 | 1.154206106 | -4.042089827 | 6.23E-05 |
| LAPTM5 | 0.20047375 | 10.99479885 | 4.041627182 | 6.24E-05 |
| MIR4453 | -0.283595223 | 2.51320211 | -4.040293674 | 6.27E-05 |
| ADM | 0.894921087 | 4.498077756 | 4.040266006 | 6.27E-05 |
| RP11-240E2.2 | -0.660602478 | -1.102517513 | -4.038535292 | 6.32E-05 |
| RP3-508I15.21 | 0.340056877 | 3.922818037 | 4.038382657 | 6.32E-05 |
| GIMAP7 | -0.824254612 | 4.33686323 | -4.037929449 | 6.34E-05 |
| AC009237.8 | -0.672386825 | -2.985089153 | -4.037820434 | 6.34E-05 |
| RP4-814D15.1 | -0.370427053 | 1.33271429 | -4.037464696 | 6.35E-05 |
| RANBP1 | 0.222299577 | 5.897579863 | 4.037291029 | 6.35E-05 |
| PLVAP | 0.700066361 | 0.888974761 | 4.037020549 | 6.36E-05 |
| INSL3 | 0.493896154 | 2.426418329 | 4.03692243 | 6.36E-05 |
| PGAM1P11 | 0.440834579 | -0.039686254 | 4.036569114 | 6.37E-05 |
| RP11-146F11.5 | -0.467703284 | 0.114686671 | -4.036488552 | 6.37E-05 |
| SSH3 | -0.220061573 | 5.615591934 | -4.036381519 | 6.38E-05 |
| TXK | -0.494247865 | 4.128882855 | -4.036197524 | 6.38E-05 |
| KDM2B | 0.184795044 | 4.998351009 | 4.036141149 | 6.38E-05 |
| RP11-342M1.6 | 0.441800466 | -0.534161476 | 4.036027298 | 6.39E-05 |
| TRAV1-1 | -0.494403918 | -0.937201192 | -4.035913456 | 6.39E-05 |
| EIF2S2 | -0.144318284 | 6.274809904 | -4.035165941 | 6.41E-05 |
| RPS26P21 | 0.549833905 | 0.48718225 | 4.034723919 | 6.42E-05 |
| RP11-190P13.1 | -0.347057325 | 0.835248831 | -4.03466743 | 6.42E-05 |
| TRAF3 | 0.226791489 | 4.592752497 | 4.034477563 | 6.43E-05 |
| SUDS3P1 | -0.188980634 | 4.126991935 | -4.034095204 | 6.44E-05 |
| KCTD6 | 0.324103773 | 3.485088742 | 4.034053781 | 6.44E-05 |
| CTD-2017D11.1 | -0.349152741 | 2.560820153 | -4.033949715 | 6.44E-05 |
| IL13RA1 | -0.570486955 | 5.518174004 | -4.033709351 | 6.45E-05 |
| AP000255.6 | -0.430821818 | -0.409378129 | -4.033673213 | 6.45E-05 |
| RP11-23J9.4 | -0.474074945 | -2.906407263 | -4.033402012 | 6.45E-05 |
| METAP1 | -0.133535501 | 5.865716102 | -4.032751351 | 6.47E-05 |
| AC012314.8 | -0.39347334 | -0.694378333 | -4.031791213 | 6.50E-05 |
| ACTN4 | -0.214318912 | 8.383843078 | -4.03166467 | 6.50E-05 |
| TAF7L | 0.522850815 | -2.026471613 | 4.031153376 | 6.51E-05 |
| RP11-834C11.11 | -0.498746404 | -2.279902108 | -4.030985609 | 6.52E-05 |
| IGKJ5 | 0.661817586 | 0.920777247 | 4.030479086 | 6.53E-05 |
| RP11-133O22.6 | -0.523955251 | -0.139254523 | -4.030046362 | 6.54E-05 |
| GIT1 | -0.15960307 | 6.181115478 | -4.029909936 | 6.55E-05 |
| EFHC1 | -0.28965674 | 3.438674381 | -4.029488054 | 6.56E-05 |
| TERF2IP | 0.149116874 | 7.384915448 | 4.028907927 | 6.57E-05 |
| PFDN6 | -0.193701896 | 5.016032014 | -4.028669813 | 6.58E-05 |
| JUND | 0.288619487 | 10.59142773 | 4.028640855 | 6.58E-05 |
| SAG | 0.622880466 | -4.163425157 | 4.028475076 | 6.59E-05 |
| NHLH1 | -0.410598301 | -0.464985983 | -4.028260897 | 6.59E-05 |
| ADCY10P1 | -0.286057897 | 2.78853194 | -4.028048479 | 6.60E-05 |
| FTO | -0.451431336 | 3.520096016 | -4.02769553 | 6.61E-05 |
| WDR31 | -0.434951541 | 1.870490849 | -4.027500658 | 6.61E-05 |
| RP11-697N18.4 | -0.467855107 | 0.371392559 | -4.027177978 | 6.62E-05 |
| SNORD119 | 0.362013599 | 1.827436921 | 4.026957012 | 6.63E-05 |
| GPRASP1 | 0.537189097 | 2.119808139 | 4.026610289 | 6.64E-05 |
| MIR17HG | -0.407810607 | 1.339131439 | -4.02618005 | 6.65E-05 |
| RP6-159A1.3 | -0.590614635 | 0.031857306 | -4.026148297 | 6.65E-05 |
| SEPT2 | -0.143709412 | 7.504416309 | -4.026080268 | 6.65E-05 |
| TGFBRAP1 | 0.269278241 | 6.537186881 | 4.024738121 | 6.69E-05 |
| GADD45GIP1 | -0.234983902 | 7.203692094 | -4.024728584 | 6.69E-05 |
| FKBP4 | -0.396515067 | 6.047059969 | -4.024727683 | 6.69E-05 |
| LINC00649 | -0.724489505 | 1.17791685 | -4.024287769 | 6.70E-05 |
| WFIKKN1 | -0.419259669 | -0.230479885 | -4.023627693 | 6.72E-05 |
| ZNF91 | -0.287374539 | 3.605827304 | -4.023387375 | 6.73E-05 |
| HSD17B12 | -0.230240253 | 5.075476981 | -4.023377686 | 6.73E-05 |
| PIK3CD | -0.230665933 | 7.179937175 | -4.023288714 | 6.73E-05 |
| RP13-140E4.1 | -0.448843745 | 2.518809311 | -4.022805271 | 6.74E-05 |
| CHERP | 0.132124925 | 5.461302721 | 4.022500994 | 6.75E-05 |
| KPNA1 | 0.13614234 | 5.31140952 | 4.021808954 | 6.77E-05 |
| ELAC1 | -0.198308421 | 2.837234809 | -4.02143699 | 6.78E-05 |
| SCLY | -0.282557268 | 0.402598838 | -4.020418434 | 6.81E-05 |
| MCM2 | 0.384218838 | 6.470078632 | 4.020309521 | 6.81E-05 |
| RP1-40E16.12 | 0.319144584 | 2.667101053 | 4.020229677 | 6.81E-05 |
| MIR142 | -0.343145699 | 7.421899078 | -4.020081251 | 6.82E-05 |
| IL18 | -0.334559727 | 5.90025192 | -4.019132848 | 6.84E-05 |
| ZNF503-AS2 | -0.582988579 | -1.308064876 | -4.018873988 | 6.85E-05 |
| TFAP2E | -0.246765496 | 3.395626109 | -4.018386665 | 6.86E-05 |
| TGM2 | 0.784268095 | 1.799532082 | 4.018263471 | 6.87E-05 |
| WDR13 | -0.15439168 | 5.885985742 | -4.018067198 | 6.87E-05 |
| C14orf132 | -0.664881715 | -3.201129654 | -4.017582572 | 6.89E-05 |
| ORC3 | -0.142421613 | 4.572667357 | -4.017481869 | 6.89E-05 |
| ARNTL2 | 0.490064011 | 3.082297055 | 4.01724501 | 6.90E-05 |
| RP5-1092A11.5 | -0.630136726 | -0.870228959 | -4.016695457 | 6.91E-05 |
| KLHDC2 | 0.194565305 | 5.352533775 | 4.016206526 | 6.93E-05 |
| NHP2P1 | -0.232036056 | 5.255886444 | -4.016030773 | 6.93E-05 |
| BCL6 | 0.633805546 | 6.319181464 | 4.015764821 | 6.94E-05 |
| TPRG1L | -0.181669002 | 6.319511181 | -4.014848564 | 6.96E-05 |
| THBS4 | 0.901242992 | 1.062139968 | 4.014807901 | 6.97E-05 |
| AC090571.1 | 0.444241711 | 2.270573148 | 4.014790531 | 6.97E-05 |
| RP11-1217F2.1 | 0.388401373 | 3.119234406 | 4.014202788 | 6.98E-05 |
| RNU1-2 | -0.53602788 | -0.421503021 | -4.013998264 | 6.99E-05 |
| SGTB | 0.372260767 | 4.307050431 | 4.013975361 | 6.99E-05 |
| TOLLIP | -0.215321155 | 5.123314507 | -4.013555866 | 7.00E-05 |
| RP11-248B24.4 | -0.416603249 | -0.131158162 | -4.012603936 | 7.03E-05 |
| SH2D3C | -0.364666511 | 5.596613293 | -4.012549903 | 7.03E-05 |
| RP4-785G19.5 | -0.484838368 | -0.556108613 | -4.01120243 | 7.07E-05 |
| RHNO1 | 0.170920231 | 5.421073444 | 4.011050831 | 7.07E-05 |
| MANEAL | -0.650871685 | 2.649228259 | -4.010831526 | 7.08E-05 |
| POLG2 | -0.222634657 | 5.333004607 | -4.010685815 | 7.08E-05 |
| TBC1D8B | -0.969160567 | -1.295931369 | -4.010469569 | 7.09E-05 |
| ZNF879 | -0.295027455 | 1.835777531 | -4.010430463 | 7.09E-05 |
| HIST1H4A | -0.501485749 | -0.320519995 | -4.009966814 | 7.11E-05 |
| DYRK1B | -0.255864875 | 4.646040226 | -4.009262196 | 7.13E-05 |
| TMF1 | 0.134285786 | 6.160960343 | 4.009194034 | 7.13E-05 |
| NDUFA5 | 0.195277609 | 4.414927949 | 4.008936519 | 7.14E-05 |
| OXR1 | -0.166874105 | 5.263674369 | -4.008179086 | 7.16E-05 |
| MCC | 0.494728169 | -2.1001485 | 4.007852985 | 7.17E-05 |
| GIPC2 | 0.648743112 | -2.531340075 | 4.006120462 | 7.22E-05 |
| ITIH1 | -0.670306701 | -2.430312959 | -4.005073188 | 7.25E-05 |
| PTGES2 | -0.14610518 | 5.336493975 | -4.004695562 | 7.26E-05 |
| CRYGS | -0.258648801 | 2.433836727 | -4.004498137 | 7.27E-05 |
| HDGFL2 | -0.16068334 | 5.637974509 | -4.004466223 | 7.27E-05 |
| AC005785.2 | 0.432483119 | -1.040126012 | 4.004451599 | 7.27E-05 |
| RNF126P1 | 0.373476048 | -0.199246806 | 4.004176549 | 7.28E-05 |
| RP4-680D5.9 | 0.496341935 | 0.269438489 | 4.003155691 | 7.31E-05 |
| KLF13 | 0.219823409 | 7.596109164 | 4.002994312 | 7.31E-05 |
| NBPF2P | -0.512698475 | 1.504788208 | -4.002517949 | 7.32E-05 |
| HIST1H2AB | -0.634842896 | 0.003228204 | -4.00215732 | 7.34E-05 |
| FBXL2 | -0.80861638 | -3.100184713 | -4.001999212 | 7.34E-05 |
| FGF10 | -0.931003943 | -2.948388924 | -4.001076509 | 7.37E-05 |
| ANKDD1B | -0.448967972 | -0.424015841 | -4.0001193 | 7.40E-05 |
| POLR3B | -0.233897681 | 2.922235765 | -3.999157818 | 7.43E-05 |
| SIPA1L3 | -0.191514592 | 4.444902753 | -3.999080256 | 7.43E-05 |
| POMK | 0.269363253 | 3.968977292 | 3.998991783 | 7.43E-05 |
| SIRPD | -0.781611132 | 0.755869663 | -3.998843621 | 7.44E-05 |
| MAGI2-AS3 | 1.019655959 | -1.857310571 | 3.998317751 | 7.45E-05 |
| COX6B2 | 0.613455745 | -2.602770165 | 3.99831672 | 7.45E-05 |
| CTGLF8P | -0.527081149 | 0.290561361 | -3.998305718 | 7.45E-05 |
| ELOVL7 | 0.695826961 | 0.9468895 | 3.997939436 | 7.46E-05 |
| MYL4 | 1.029086963 | 1.479165821 | 3.99747261 | 7.48E-05 |
| AP5M1 | -0.141647758 | 5.163509974 | -3.996128789 | 7.52E-05 |
| ATP5SL | -0.146987384 | 5.49086193 | -3.995541548 | 7.54E-05 |
| PPP1R37 | 0.130236984 | 3.678105132 | 3.995095318 | 7.55E-05 |
| OR14L1P | -0.687432529 | -0.689734681 | -3.994777129 | 7.56E-05 |
| RP1-206D15.5 | 0.441459211 | 0.233456654 | 3.994688676 | 7.56E-05 |
| OTUD5 | 0.152312064 | 6.96075723 | 3.994520591 | 7.57E-05 |
| CAP1 | -0.230289514 | 9.32390311 | -3.99420666 | 7.58E-05 |
| ANKRD27 | -0.29324997 | 5.226902615 | -3.993966568 | 7.58E-05 |
| AC010878.3 | 0.328485512 | 1.05965009 | 3.993603313 | 7.60E-05 |
| DOCK4 | 0.593399905 | 1.112477469 | 3.993317041 | 7.60E-05 |
| LIMS1 | -0.242256331 | 6.099731932 | -3.993099359 | 7.61E-05 |
| RP11-439E19.10 | -0.523700688 | 1.949750071 | -3.992846795 | 7.62E-05 |
| PTAR1 | 0.156207208 | 5.932228577 | 3.992684463 | 7.62E-05 |
| GS1-124K5.11 | -0.227407798 | 2.730389173 | -3.992247346 | 7.64E-05 |
| FCGR1C | -0.742808716 | 1.889176528 | -3.992111252 | 7.64E-05 |
| ARPC1B | -0.263975321 | 7.983640531 | -3.991749694 | 7.65E-05 |
| RP4-666F24.3 | -0.535919181 | 1.618740688 | -3.991662115 | 7.66E-05 |
| EIF4G1 | -0.192518042 | 7.942123548 | -3.991624837 | 7.66E-05 |
| MKRN2 | -0.110456992 | 6.166676809 | -3.991601873 | 7.66E-05 |
| ILVBL | 0.178626559 | 4.350326473 | 3.991016885 | 7.68E-05 |
| RP11-244O19.1 | -0.371982008 | 1.668936409 | -3.99052397 | 7.69E-05 |
| IFI27 | 0.771686054 | 0.010615894 | 3.990063719 | 7.71E-05 |
| BTF3P9 | -0.211822596 | 7.451542106 | -3.989269906 | 7.73E-05 |
| REV1 | -0.104132366 | 5.03047583 | -3.989145202 | 7.74E-05 |
| SNRNP25 | 0.241419907 | 4.352980999 | 3.988901311 | 7.74E-05 |
| RPL39P36 | -0.382534341 | 0.302830113 | -3.988090295 | 7.77E-05 |
| TMEM206 | -0.255064249 | 5.181744064 | -3.987922576 | 7.77E-05 |
| RP11-1006G14.1 | -0.443067079 | -1.905117206 | -3.987770851 | 7.78E-05 |
| FBXO21 | 0.282092877 | 3.662127483 | 3.987616977 | 7.78E-05 |
| Z84812.4 | -0.462331034 | -0.776317049 | -3.987489916 | 7.79E-05 |
| RP11-264B17.3 | 0.367017423 | 0.172989295 | 3.987070396 | 7.80E-05 |
| RARRES1 | 0.544808752 | -1.703151512 | 3.987007038 | 7.80E-05 |
| RP11-293A21.1 | 0.423620303 | 0.862481251 | 3.987004959 | 7.80E-05 |
| ABL1 | 0.191235547 | 6.069581347 | 3.986967728 | 7.80E-05 |
| RP3-406P24.1 | -0.158312299 | 4.733046756 | -3.986535424 | 7.82E-05 |
| AC007362.1 | 0.596205803 | -1.445506798 | 3.986505306 | 7.82E-05 |
| ACTR10 | -0.099247022 | 5.554588373 | -3.986491339 | 7.82E-05 |
| SOCS5 | 0.236435102 | 5.037045158 | 3.986421397 | 7.82E-05 |
| AMN | -0.686062934 | 2.369622482 | -3.986355018 | 7.82E-05 |
| ZNF211 | 0.225392422 | 4.919793141 | 3.98577664 | 7.84E-05 |
| CCDC107 | -0.215924394 | 5.097450624 | -3.985663457 | 7.85E-05 |
| CIRBP | 0.225245029 | 7.474192709 | 3.985441997 | 7.85E-05 |
| HMGB1P19 | -0.46433638 | -0.4276542 | -3.985380128 | 7.85E-05 |
| DTX3 | 0.672345607 | 2.161694521 | 3.984649249 | 7.88E-05 |
| CTD-2639E6.9 | 0.522376441 | 1.006207237 | 3.984638169 | 7.88E-05 |
| KIF5B | 0.124302171 | 7.627945445 | 3.984578993 | 7.88E-05 |
| LINC00106 | -0.296966039 | 4.842742983 | -3.983975093 | 7.90E-05 |
| RP11-72I8.1 | -0.475780126 | -1.208673878 | -3.983881787 | 7.90E-05 |
| CTD-2561J22.5 | 0.87052279 | 0.943485818 | 3.98334368 | 7.92E-05 |
| TESK2 | 0.234360514 | 4.61744259 | 3.983286981 | 7.92E-05 |
| AC003075.4 | 0.453369812 | 0.484271233 | 3.983122306 | 7.93E-05 |
| IKZF1 | -0.203617547 | 7.233340469 | -3.981411753 | 7.98E-05 |
| MTERFD1 | -0.230661297 | 4.802391724 | -3.981153755 | 7.99E-05 |
| TPH1 | 0.392857209 | 0.258121381 | 3.981132264 | 7.99E-05 |
| C7orf73 | -0.167391069 | 6.160851232 | -3.981048046 | 7.99E-05 |
| RFC4 | 0.235884998 | 4.973195323 | 3.980148287 | 8.02E-05 |
| C5orf24 | 0.215611376 | 4.875449533 | 3.979992252 | 8.03E-05 |
| JPH1 | -0.942802997 | -2.549264057 | -3.978807946 | 8.07E-05 |
| RP13-476E20.1 | -0.545693988 | 0.589511257 | -3.977979468 | 8.09E-05 |
| RPL34P18 | -0.213767459 | 8.107959192 | -3.97782471 | 8.10E-05 |
| RAP2A | -0.255763042 | 4.781994002 | -3.976763177 | 8.13E-05 |
| FAM86FP | -0.377188151 | 1.356080119 | -3.975231584 | 8.19E-05 |
| TMEM167A | -0.222485228 | 6.624962858 | -3.975045998 | 8.19E-05 |
| KLF10 | 0.460460202 | 8.65857682 | 3.9750163 | 8.19E-05 |
| MT-ATP8 | -0.139032479 | 12.1992612 | -3.974435529 | 8.21E-05 |
| SMARCE1P6 | 0.183857136 | 4.211780042 | 3.974152962 | 8.22E-05 |
| CDIP1 | -0.305466171 | 4.978401516 | -3.973772261 | 8.23E-05 |
| AC017002.2 | 0.531442081 | 0.146204012 | 3.973691521 | 8.24E-05 |
| INTS15 | -0.16656534 | 5.862911919 | -3.97366723 | 8.24E-05 |
| SWI5 | -0.155341746 | 4.013077286 | -3.973193938 | 8.25E-05 |
| FSD1L | -0.354350207 | 2.456713906 | -3.97301632 | 8.26E-05 |
| AOC2 | 0.447841689 | 1.449175149 | 3.972942938 | 8.26E-05 |
| ZFP90 | -0.236129596 | 4.095108159 | -3.97254581 | 8.27E-05 |
| IGHJ2 | 0.606090195 | 0.814586537 | 3.972159133 | 8.29E-05 |
| IGFBP3 | 0.71122854 | -0.11767222 | 3.972031406 | 8.29E-05 |
| ELF3 | 0.48363941 | -0.174901292 | 3.971720971 | 8.30E-05 |
| ZNF10 | 0.334548824 | 2.753081849 | 3.971162041 | 8.32E-05 |
| RP11-206L10.8 | 0.304769183 | 1.477863246 | 3.971152408 | 8.32E-05 |
| RP11-346C20.3 | -0.565822881 | -1.704047908 | -3.970865684 | 8.33E-05 |
| RP11-82O19.2 | -0.52326781 | 0.061735579 | -3.970819216 | 8.33E-05 |
| IGF2 | 0.894997784 | -2.181523876 | 3.970702272 | 8.34E-05 |
| TRIM2 | -0.625091828 | -0.281111482 | -3.970230385 | 8.35E-05 |
| AC139887.4 | -0.430435509 | 0.310389633 | -3.970113505 | 8.36E-05 |
| SNORD108 | 0.618510909 | 2.065403049 | 3.969769242 | 8.37E-05 |
| XRCC3 | 0.259785042 | 3.383636894 | 3.96930044 | 8.38E-05 |
| GAS8 | -0.313957848 | 2.287794191 | -3.969078057 | 8.39E-05 |
| KLHL9 | -0.193435051 | 5.278332451 | -3.969019834 | 8.39E-05 |
| KCNIP2 | -0.306113562 | 1.165088835 | -3.968034465 | 8.43E-05 |
| HSPA8P7 | -0.491284302 | 1.019163768 | -3.968029096 | 8.43E-05 |
| HMOX1 | 0.793628186 | 5.219405329 | 3.967870862 | 8.43E-05 |
| LINC00685 | -0.333024942 | 2.369221627 | -3.96771523 | 8.44E-05 |
| GRIN3A | -0.658274914 | -3.1557637 | -3.967113485 | 8.46E-05 |
| ZBTB22 | -0.164694011 | 5.468768093 | -3.966899937 | 8.47E-05 |
| RP11-798G7.8 | -0.468975801 | -1.998517242 | -3.965899504 | 8.50E-05 |
| RP4-730K3.3 | -0.377755794 | 1.713821656 | -3.965749292 | 8.51E-05 |
| WI2-3658N16.1 | 0.260385277 | 2.981464507 | 3.965638374 | 8.51E-05 |
| HNRNPM | 0.145804216 | 8.038349373 | 3.965034038 | 8.53E-05 |
| CDC42P6 | -0.191687357 | 8.897406074 | -3.964896177 | 8.54E-05 |
| ANKRD36BP2 | 0.608055214 | 0.100941767 | 3.963978359 | 8.57E-05 |
| CXCL6 | 0.636207438 | -0.758382087 | 3.96381951 | 8.57E-05 |
| CTD-2083E4.6 | -0.418581007 | 0.159760954 | -3.963516222 | 8.58E-05 |
| TMEM98 | 0.856070333 | -2.935384422 | 3.962778722 | 8.61E-05 |
| RP11-561O23.8 | -0.531575543 | 1.017812223 | -3.961711065 | 8.65E-05 |
| HARS | -0.157501075 | 4.944250239 | -3.961666945 | 8.65E-05 |
| RP11-207C16.4 | -0.466714185 | 0.041449533 | -3.96165029 | 8.65E-05 |
| RBM41 | -0.14346914 | 3.741710048 | -3.961248214 | 8.66E-05 |
| RP11-91P24.1 | -0.419148977 | 0.579435725 | -3.961206636 | 8.66E-05 |
| DEPDC1 | 0.645484651 | 1.730365575 | 3.960846869 | 8.68E-05 |
| SETD4 | -0.182299589 | 3.630798265 | -3.960165094 | 8.70E-05 |
| KRT8P46 | 0.317938405 | 3.563073835 | 3.960062178 | 8.70E-05 |
| LARP4 | -0.153709254 | 4.58439469 | -3.959979117 | 8.71E-05 |
| CALM2P3 | -0.411136559 | 1.450871319 | -3.958970132 | 8.74E-05 |
| GADD45B | 0.45236362 | 7.684330398 | 3.958837697 | 8.75E-05 |
| SNX8 | 0.262785997 | 3.690719699 | 3.958546607 | 8.76E-05 |
| ATP6V1E2 | -0.218815177 | 2.529007219 | -3.95832498 | 8.77E-05 |
| TNKS | 0.163881729 | 4.699054241 | 3.957942639 | 8.78E-05 |
| MOSPD1 | 0.201073527 | 3.894910105 | 3.957184471 | 8.81E-05 |
| MVK | -0.234189741 | 2.454449026 | -3.95676961 | 8.82E-05 |
| SH3RF3-AS1 | 0.634683862 | -0.72099947 | 3.956697976 | 8.82E-05 |
| TMCO3 | -0.232573378 | 6.973997937 | -3.956357751 | 8.84E-05 |
| PSMD7P1 | 0.254393632 | 1.84361132 | 3.956276267 | 8.84E-05 |
| MIR320E | 0.419228435 | 0.674668123 | 3.95575046 | 8.86E-05 |
| ZNF44 | 0.271170764 | 3.786637097 | 3.95504838 | 8.88E-05 |
| C6orf99 | -0.443527403 | -0.789659089 | -3.954326349 | 8.91E-05 |
| RP11-157J24.1 | -0.451368515 | -0.980370969 | -3.95406978 | 8.92E-05 |
| ZNF587B | 0.279980798 | 3.975051152 | 3.95364191 | 8.93E-05 |
| SPATA17 | -0.645541048 | -2.902788063 | -3.95287473 | 8.96E-05 |
| FAM98B | -0.259396552 | 4.238818502 | -3.952027736 | 8.99E-05 |
| ACTR3B | -0.26235815 | 1.98187296 | -3.951638333 | 9.01E-05 |
| HLA-G | 0.410056224 | 5.094331421 | 3.950269267 | 9.06E-05 |
| RP11-258F1.1 | 0.52682607 | 0.121295406 | 3.949963903 | 9.07E-05 |
| RALGDS | 0.270425762 | 6.203408176 | 3.949820316 | 9.07E-05 |
| OSBP2 | 0.810864925 | 1.627805643 | 3.949661322 | 9.08E-05 |
| BLOC1S3 | 0.215793192 | 4.327575047 | 3.949156905 | 9.10E-05 |
| IL4I1 | 0.710880885 | 1.570327453 | 3.948918076 | 9.11E-05 |
| MIOS | 0.13057319 | 4.662036784 | 3.947700529 | 9.15E-05 |
| WBP4 | 0.129520983 | 5.252486242 | 3.947289234 | 9.17E-05 |
| PARPBP | 0.269404938 | 3.233734601 | 3.94701041 | 9.18E-05 |
| RP1-111C20.4 | -0.275930016 | 1.398398236 | -3.946750534 | 9.19E-05 |
| H3F3C | 0.32711587 | 6.302459548 | 3.946313333 | 9.20E-05 |
| RPS2P32 | 0.343371781 | 1.96632428 | 3.946171555 | 9.21E-05 |
| RP11-23J9.5 | -0.357627351 | -0.252218134 | -3.945190739 | 9.24E-05 |
| DSG2 | 1.302531196 | 0.154316 | 3.944392756 | 9.27E-05 |
| IL27RA | -0.31955709 | 6.050795888 | -3.943773256 | 9.30E-05 |
| SH2D3A | 0.415280161 | 1.532298156 | 3.942899819 | 9.33E-05 |
| RNU6-1005P | 0.416915318 | 0.751440077 | 3.94277513 | 9.33E-05 |
| H6PD | -0.260547653 | 5.67070762 | -3.942498507 | 9.34E-05 |
| USP15 | 0.210869727 | 6.826465293 | 3.942372333 | 9.35E-05 |
| XXbac-B33L19.4 | -0.552437873 | -1.042868043 | -3.942237673 | 9.35E-05 |
| ACTN1-AS1 | 0.491077524 | 0.792744108 | 3.942069557 | 9.36E-05 |
| TRBV6-6 | -0.631052709 | 1.366897794 | -3.94188784 | 9.37E-05 |
| ZNF490 | -0.204858403 | 1.725753716 | -3.941256458 | 9.39E-05 |
| ZNF17 | -0.221376973 | 2.698648622 | -3.941000189 | 9.40E-05 |
| PTK2 | 0.796153036 | 1.574582227 | 3.940950437 | 9.40E-05 |
| SMIM8 | -0.17439279 | 3.129022521 | -3.94077395 | 9.41E-05 |
| CTD-2292M16.8 | -0.394932766 | 0.950958239 | -3.940418818 | 9.42E-05 |
| EIF4EBP1 | -0.236426629 | 6.891849631 | -3.939757319 | 9.45E-05 |
| UHRF2 | 0.157467001 | 5.650378459 | 3.939082845 | 9.47E-05 |
| ANK1 | 0.933331279 | 2.424297764 | 3.938975436 | 9.48E-05 |
| TGFB2 | 0.528028568 | -2.59011129 | 3.938974938 | 9.48E-05 |
| CTD-2248H3.1 | -0.292871158 | 1.653967085 | -3.938685549 | 9.49E-05 |
| RP11-156G14.6 | 0.294265684 | 3.675366826 | 3.938646745 | 9.49E-05 |
| C7orf50 | 0.307017763 | 4.323384991 | 3.938022402 | 9.51E-05 |
| SLC35E2 | -0.266208067 | 3.155344493 | -3.937917103 | 9.52E-05 |
| RPL36AL | -0.136720809 | 9.72599807 | -3.937784517 | 9.52E-05 |
| RTKN | -0.574403089 | 1.748231459 | -3.93767533 | 9.53E-05 |
| ETFB | 0.192046511 | 3.584488584 | 3.936882779 | 9.56E-05 |
| DNAAF2 | -0.262135691 | 4.566143001 | -3.93684405 | 9.56E-05 |
| SLC39A6 | -0.195359841 | 5.804566811 | -3.936431177 | 9.58E-05 |
| FTX | -0.244685907 | 2.0199131 | -3.936396444 | 9.58E-05 |
| AC120194.1 | 0.532856068 | 0.232466717 | 3.936025045 | 9.59E-05 |
| PHTF2 | 0.195943146 | 4.525766076 | 3.934396781 | 9.65E-05 |
| LPAR4 | -0.721644873 | 1.267983868 | -3.933964 | 9.67E-05 |
| CD1E | -0.923120664 | -1.489908116 | -3.933682784 | 9.68E-05 |
| RPS24P8 | -0.185670173 | 6.027599286 | -3.933532644 | 9.69E-05 |
| ZNF890P | -0.46268284 | -0.868051859 | -3.933434571 | 9.69E-05 |
| ZNF507 | 0.256867462 | 4.08177307 | 3.9334116 | 9.69E-05 |
| UNC119 | 0.333803755 | 5.215727898 | 3.933371238 | 9.69E-05 |
| RP11-1055B8.4 | -1.020777885 | 3.02504497 | -3.932763088 | 9.72E-05 |
| EPN2 | 0.632846232 | 0.696546253 | 3.932749595 | 9.72E-05 |
| FAM214A | 0.204888723 | 4.792012776 | 3.932487383 | 9.73E-05 |
| ERICH1 | -0.164466017 | 4.771727875 | -3.932338054 | 9.73E-05 |
| C1orf61 | 0.62933645 | -4.266526865 | 3.932175265 | 9.74E-05 |
| FOXD2 | -0.480764646 | -1.459176528 | -3.931971759 | 9.75E-05 |
| MT-ATP6 | -0.112678226 | 12.95741778 | -3.931684272 | 9.76E-05 |
| HIST1H3D | -0.609708443 | 2.903699095 | -3.931422689 | 9.77E-05 |
| RP11-692M12.4 | 0.46767302 | -0.013809837 | 3.931354425 | 9.77E-05 |
| USP3 | 0.294566715 | 5.736864928 | 3.931262719 | 9.78E-05 |
| XRCC4 | -0.261369494 | 4.338567557 | -3.930794557 | 9.79E-05 |
| PLCB3 | -0.247899287 | 4.98622524 | -3.930474777 | 9.81E-05 |
| HIST1H2BK | -0.35159808 | 8.254829064 | -3.929565681 | 9.84E-05 |
| RND3 | 0.794117219 | -2.64143812 | 3.929347886 | 9.85E-05 |
| PKLR | 1.095312202 | 0.03090622 | 3.9289921 | 9.87E-05 |
| TPRN | -0.220663743 | 3.279977283 | -3.928914611 | 9.87E-05 |
| NOA1 | -0.18816119 | 6.12120205 | -3.928692719 | 9.88E-05 |
| CCDC148 | -0.520559701 | -2.135281019 | -3.928604043 | 9.88E-05 |
| RP11-184I16.3 | -0.485446796 | -0.261643456 | -3.928594973 | 9.88E-05 |
| GAPDHP63 | 0.410376329 | 4.754580481 | 3.928076299 | 9.90E-05 |
| TRIB3 | 0.397547129 | 4.15024121 | 3.926962129 | 9.95E-05 |
| BBIP1 | -0.183818908 | 2.975328398 | -3.926785074 | 9.95E-05 |
| GPR20 | -0.575275162 | -2.079968404 | -3.926596447 | 9.96E-05 |
| TMEM72-AS1 | -0.458719753 | -2.302074801 | -3.926496771 | 9.97E-05 |
| FTH1P4 | 0.51948955 | 0.00802865 | 3.925807886 | 9.99E-05 |
| CDON | -0.556063106 | -2.495444661 | -3.925085228 | 0.000100223 |
| FZD3 | 0.752942212 | -0.203081496 | 3.924957715 | 0.000100274 |
| HLA-S | 0.527483733 | 1.539860248 | 3.924571197 | 0.00010043 |
| ZNF101P2 | -0.417085367 | -0.842802973 | -3.923721099 | 0.000100773 |
| FRAT2 | -0.374848279 | 6.503133344 | -3.923157253 | 0.000101002 |
| RP11-89M16.1 | -0.513926749 | -3.522073952 | -3.92245205 | 0.000101288 |
| KLF8 | -0.519694383 | 0.90311367 | -3.922442762 | 0.000101292 |
| CNEP1R1 | 0.276154588 | 4.169451213 | 3.921454191 | 0.000101694 |
| PTPRO | -0.705431812 | 0.778142899 | -3.921265663 | 0.000101771 |
| RP13-20L14.4 | 0.427474337 | 0.067005348 | 3.921017688 | 0.000101873 |
| HSPA8P3 | 0.443822772 | 0.553484568 | 3.920706373 | 0.000102 |
| GINS2 | 0.427897538 | 3.895928487 | 3.920623431 | 0.000102034 |
| CLDN23 | -0.596428195 | -1.930975743 | -3.920059357 | 0.000102265 |
| PGS1 | 0.195792311 | 5.938625965 | 3.919649753 | 0.000102433 |
| EME1 | 0.38753187 | 2.822691018 | 3.919624119 | 0.000102444 |
| PEX11G | -0.409659168 | 1.082081713 | -3.918879193 | 0.000102751 |
| PHLDA3 | -0.675354982 | 2.099665687 | -3.918807195 | 0.00010278 |
| RP11-215P8.3 | 0.453882 | 0.223556339 | 3.91641851 | 0.000103769 |
| FAM64A | 0.725780373 | 0.28360241 | 3.916392224 | 0.00010378 |
| AC017002.1 | 0.670845909 | 0.227213551 | 3.915763835 | 0.000104042 |
| RP11-420K14.1 | -0.625640508 | -0.194113025 | -3.915664271 | 0.000104084 |
| DMTF1 | -0.154736309 | 5.973451875 | -3.914539259 | 0.000104554 |
| RP11-329B9.4 | -0.566845793 | -0.693306334 | -3.913779226 | 0.000104873 |
| SNRNP40 | 0.149752066 | 5.134592742 | 3.91355235 | 0.000104968 |
| LINC00896 | -0.486766249 | -1.41377203 | -3.912936602 | 0.000105228 |
| GUCY1A3 | -0.866922006 | 4.598204737 | -3.912635685 | 0.000105354 |
| RP11-457M11.5 | -0.495659382 | 1.390241746 | -3.912531525 | 0.000105398 |
| RP11-798M19.3 | -0.445816737 | 2.611031008 | -3.912456937 | 0.00010543 |
| SP110 | -0.257695844 | 5.466922862 | -3.912180814 | 0.000105547 |
| CDX2 | -0.627392667 | -2.499417673 | -3.911978654 | 0.000105632 |
| ANKRD20A1 | 0.541537093 | -2.638258681 | 3.911645774 | 0.000105773 |
| TRGV10 | -0.638491049 | 2.094519388 | -3.911533566 | 0.000105821 |
| RP11-20I23.13 | -0.558995411 | -0.240322404 | -3.911116437 | 0.000105997 |
| EPHX1 | -0.412574152 | 4.354429118 | -3.910874062 | 0.0001061 |
| RP11-399K21.14 | -0.612744735 | -1.410681348 | -3.910854717 | 0.000106109 |
| TRAV24 | -0.506833625 | -0.835087397 | -3.910616741 | 0.00010621 |
| BTAF1 | 0.181187541 | 6.599647283 | 3.910419282 | 0.000106294 |
| IVNS1ABP | 0.377415631 | 6.425368662 | 3.909343703 | 0.000106752 |
| TRAV20 | -0.540357163 | -0.435549733 | -3.908756001 | 0.000107004 |
| MIR3687 | 0.462846564 | 2.440242889 | 3.908402927 | 0.000107155 |
| NOP10 | -0.214225272 | 8.018839306 | -3.90795965 | 0.000107345 |
| RP11-1007O24.2 | -0.39338528 | 0.279527095 | -3.906750079 | 0.000107866 |
| GRK7 | 0.421966036 | -1.677246948 | 3.906686178 | 0.000107894 |
| LIN9 | 0.237379902 | 2.923715638 | 3.906541054 | 0.000107956 |
| COX11P1 | -0.391340605 | -0.363807315 | -3.906300224 | 0.000108061 |
| AC021224.1 | 0.535305827 | 1.140515766 | 3.906092625 | 0.00010815 |
| SNAP47 | -0.205953405 | 3.365488416 | -3.905527108 | 0.000108395 |
| RP1-1J6.2 | 0.469448049 | -0.886510092 | 3.905449071 | 0.000108429 |
| AP001046.5 | 0.616049103 | -0.532341016 | 3.904824537 | 0.0001087 |
| ZMIZ2 | -0.146034612 | 6.025165907 | -3.904355145 | 0.000108904 |
| EZH1 | -0.183207008 | 5.403219756 | -3.904186374 | 0.000108978 |
| RP11-326K13.4 | 0.345470998 | 1.817729926 | 3.904031354 | 0.000109046 |
| ZDHHC3 | 0.173940787 | 5.939170919 | 3.904018331 | 0.000109051 |
| TIGD2 | -0.227261986 | 4.081342269 | -3.903939929 | 0.000109085 |
| PITPNM3 | 0.456821477 | -1.112971771 | 3.903795836 | 0.000109148 |
| KIFC3 | 0.592973215 | 2.396137103 | 3.903492472 | 0.000109281 |
| TRBJ2-2 | 0.501347681 | -0.312949978 | 3.902693838 | 0.00010963 |
| ZNF654 | 0.160854709 | 5.386590958 | 3.902568689 | 0.000109685 |
| FZD2 | -0.800987011 | 2.1411152 | -3.902025808 | 0.000109923 |
| SERBP1P5 | -0.182535971 | 7.385596694 | -3.901275345 | 0.000110253 |
| SLC25A39 | 0.253884673 | 6.730388804 | 3.900706056 | 0.000110505 |
| RP1-244F24.1 | -0.486378959 | 3.716384926 | -3.900675373 | 0.000110518 |
| MT-TK | 0.436948156 | 0.929217549 | 3.900486172 | 0.000110602 |
| RP4-800M22.1 | -0.372622796 | 0.685239005 | -3.90047496 | 0.000110607 |
| TMED9 | -0.16521338 | 7.746317866 | -3.90034615 | 0.000110664 |
| LTF | 1.248993954 | 6.851680641 | 3.899634888 | 0.000110978 |
| PRDX4 | 0.343962743 | 5.162278201 | 3.899337562 | 0.00011111 |
| UBE2F | 0.272057424 | 4.11054622 | 3.899225388 | 0.00011116 |
| CHMP7 | -0.268362479 | 5.113314257 | -3.89882247 | 0.000111339 |
| RP11-15L13.4 | -0.348296545 | 1.016972149 | -3.898571193 | 0.000111451 |
| A2M-AS1 | -0.694884171 | 0.874339981 | -3.897813575 | 0.000111789 |
| CECR5 | -0.326501524 | 4.704153279 | -3.897657903 | 0.000111858 |
| RP11-701H24.7 | 0.635768811 | 4.015276188 | 3.897616347 | 0.000111877 |
| DHX9 | 0.139718604 | 7.970036897 | 3.897566508 | 0.000111899 |
| PTPN23 | -0.096954778 | 5.601363469 | -3.896805785 | 0.000112239 |
| PTRHD1 | 0.183488735 | 5.895567874 | 3.896673517 | 0.000112299 |
| RP11-90B9.2 | 0.365409652 | 1.337907326 | 3.896386487 | 0.000112427 |
| YTHDF2 | 0.180228691 | 6.885392638 | 3.895671201 | 0.000112749 |
| ZNF16 | 0.175415006 | 2.629011629 | 3.895065449 | 0.000113022 |
| UBE2B | 0.205279426 | 6.142856789 | 3.894949484 | 0.000113074 |
| MFSD5 | 0.224826666 | 5.135346242 | 3.894916329 | 0.000113089 |
| HMCN1 | 0.738793663 | -3.659127615 | 3.894353488 | 0.000113343 |
| RP11-644F5.10 | 0.330112798 | 1.654857241 | 3.893219186 | 0.000113857 |
| CD1A | -0.725202898 | -1.149165292 | -3.892150226 | 0.000114344 |
| LINC00977 | -0.624486436 | -0.765113977 | -3.891516189 | 0.000114633 |
| AC003102.3 | 0.575246009 | -2.491538532 | 3.891086434 | 0.00011483 |
| NAA10 | 0.139945185 | 3.897653083 | 3.890417428 | 0.000115137 |
| LRP12 | 0.532588773 | 2.33893767 | 3.890215553 | 0.000115229 |
| NT5DC1 | -0.269618496 | 4.052043514 | -3.890210918 | 0.000115232 |
| KCTD14 | -0.450884794 | -0.762941302 | -3.888932396 | 0.00011582 |
| NQO1 | -0.464526751 | 2.074358291 | -3.888638965 | 0.000115956 |
| C7orf25 | -0.345627633 | -0.59066772 | -3.88844969 | 0.000116043 |
| HNRNPF | -0.221278115 | 8.641269527 | -3.888428662 | 0.000116053 |
| WNT10B | 0.586200094 | 0.989255337 | 3.888152327 | 0.000116181 |
| COX20P1 | -0.188521083 | 4.534118645 | -3.887560138 | 0.000116455 |
| RP1-178F10.3 | 0.217672054 | 6.349245057 | 3.887444761 | 0.000116509 |
| INGX | -0.421821944 | -1.035013322 | -3.887373977 | 0.000116542 |
| RNU6-4P | 0.36074976 | 1.09898446 | 3.887241734 | 0.000116603 |
| BTD | -0.188279099 | 3.524119306 | -3.886963991 | 0.000116732 |
| GOLGA6L5P | 0.413603987 | 2.405679227 | 3.886580624 | 0.000116911 |
| LAYN | 0.668343388 | -0.189187535 | 3.886525456 | 0.000116936 |
| EREG | 1.161644684 | 4.219578102 | 3.886350404 | 0.000117018 |
| S100Z | -0.789357922 | 4.680977571 | -3.885769993 | 0.000117289 |
| BCOR | 0.271585381 | 6.061202489 | 3.885691753 | 0.000117325 |
| VDAC1P1 | -0.216010885 | 5.771782408 | -3.885543787 | 0.000117395 |
| SS18L1 | -0.152694131 | 4.09174776 | -3.885183889 | 0.000117563 |
| ZNF195 | -0.200984468 | 4.484581099 | -3.884883939 | 0.000117703 |
| MRPL2 | 0.138540951 | 4.701114319 | 3.884669276 | 0.000117804 |
| PNKD | -0.265579464 | 6.333769942 | -3.883885708 | 0.000118172 |
| FCGR3A | -0.778603627 | 5.761000885 | -3.88349299 | 0.000118357 |
| SLC35G2 | 0.772533443 | -0.115490649 | 3.882946685 | 0.000118615 |
| RP11-405A12.1 | 0.187378184 | 4.6788128 | 3.882927561 | 0.000118624 |
| ACBD6 | 0.174056341 | 6.073139633 | 3.882722735 | 0.000118721 |
| AC073283.4 | -0.670461426 | -2.560744307 | -3.882691284 | 0.000118735 |
| C12orf39 | 0.710687666 | -1.392982534 | 3.882532116 | 0.000118811 |
| TRBJ2-1 | 0.526932379 | 0.012428998 | 3.88242336 | 0.000118862 |
| RP5-1139B12.3 | -0.547183251 | 0.398971569 | -3.881892732 | 0.000119113 |
| NTRK2 | 0.867603291 | -4.949079332 | 3.881410694 | 0.000119342 |
| SCP2 | -0.188811162 | 5.52628968 | -3.880924512 | 0.000119573 |
| UCK2 | -0.293632761 | 4.818881942 | -3.880494025 | 0.000119778 |
| TLR10 | -0.596593393 | 1.307966604 | -3.879793492 | 0.000120112 |
| RP11-762I7.4 | 0.378090915 | 2.223997938 | 3.879651917 | 0.00012018 |
| RNVU1-13 | -0.786707821 | 2.633740842 | -3.879633395 | 0.000120189 |
| INTS4 | -0.100802714 | 4.574536471 | -3.879394174 | 0.000120303 |
| ERO1LB | 0.23853041 | 3.83024584 | 3.879361829 | 0.000120319 |
| RRAS2 | 0.5884489 | 1.295333402 | 3.879121305 | 0.000120434 |
| EPN3 | 0.546251614 | -3.40138168 | 3.879019935 | 0.000120482 |
| DHRS7 | -0.185284997 | 6.73076201 | -3.878890072 | 0.000120545 |
| MCM6 | 0.286652468 | 6.272910027 | 3.878887207 | 0.000120546 |
| TMEM201 | 0.222931673 | 3.927862544 | 3.878804149 | 0.000120586 |
| C19orf69 | 0.483887657 | -0.637517466 | 3.878220339 | 0.000120866 |
| PTPRA | -0.131066416 | 7.006453028 | -3.877914765 | 0.000121013 |
| ACTR3P2 | -0.384320335 | 1.983822914 | -3.87787034 | 0.000121034 |
| RP11-10C24.3 | -0.36006648 | 1.097020383 | -3.877701307 | 0.000121116 |
| RP11-672L10.6 | -0.48764262 | -0.431694014 | -3.877101545 | 0.000121405 |
| DCSTAMP | 0.664539033 | -1.919585739 | 3.876916622 | 0.000121494 |
| RAB22A | 0.171352339 | 4.327183182 | 3.876716903 | 0.000121591 |
| RNASEH1P1 | 0.271479724 | 1.270302974 | 3.876680293 | 0.000121608 |
| RAD18 | 0.15995901 | 3.925692747 | 3.876639933 | 0.000121628 |
| SLC25A3 | 0.184276496 | 8.42939871 | 3.875813997 | 0.000122028 |
| LSM2 | -0.226096825 | 7.073893374 | -3.875470894 | 0.000122194 |
| LINC00310 | -0.389800791 | -0.848259536 | -3.874919812 | 0.000122462 |
| HDGF | 0.192342306 | 9.056849827 | 3.873776047 | 0.00012302 |
| FANCC | 0.289489401 | 2.200133942 | 3.873768041 | 0.000123024 |
| HLA-A | 0.194846692 | 10.87082477 | 3.873205464 | 0.000123299 |
| NFE2L3P1 | -0.499640042 | -0.302501191 | -3.872933089 | 0.000123433 |
| MED17 | 0.221118145 | 4.161673724 | 3.872700512 | 0.000123547 |
| C5orf42 | -0.245355586 | 3.70026843 | -3.871782507 | 0.000123998 |
| RP11-30L15.6 | -0.385873081 | -0.294634727 | -3.871489521 | 0.000124142 |
| DUSP2 | 0.576900516 | 5.860891872 | 3.871015716 | 0.000124376 |
| GYS1 | -0.23823642 | 6.099054656 | -3.870204494 | 0.000124777 |
| SNORD3D | -0.640402612 | 2.710504204 | -3.869599274 | 0.000125077 |
| RP11-3K24.1 | -0.497629702 | 0.268132808 | -3.869383434 | 0.000125185 |
| MICALCL | 0.540648992 | 2.148860721 | 3.868956313 | 0.000125397 |
| FAM110D | 0.474003628 | -1.803646525 | 3.868744602 | 0.000125502 |
| AL590822.2 | -0.520822104 | -0.718110806 | -3.868724243 | 0.000125512 |
| KREMEN1 | 0.744051579 | 0.98843257 | 3.868447791 | 0.00012565 |
| ABCE1 | -0.199908179 | 6.001306041 | -3.867813018 | 0.000125967 |
| ARHGEF1 | -0.168954189 | 7.699821691 | -3.86762015 | 0.000126064 |
| EPM2A | -0.209789281 | 3.021735848 | -3.867583295 | 0.000126082 |
| CRLF3 | -0.181790972 | 5.672821196 | -3.867166461 | 0.000126291 |
| CYP21A2 | 0.472052459 | -2.096361166 | 3.867139233 | 0.000126304 |
| MYH3 | -0.257519492 | 2.517526078 | -3.866605288 | 0.000126572 |
| MAPK14 | -0.225507203 | 7.048300599 | -3.866567497 | 0.000126591 |
| HMGN3 | -0.308055353 | 8.301551435 | -3.865865286 | 0.000126944 |
| ZMAT1 | -0.324092668 | 4.654003796 | -3.865528088 | 0.000127114 |
| BTN2A1 | -0.179092173 | 5.836439924 | -3.865423079 | 0.000127167 |
| ST6GALNAC1 | 0.752206937 | -0.01039996 | 3.865049054 | 0.000127356 |
| FOPNL | -0.145704349 | 6.490458568 | -3.864848525 | 0.000127457 |
| RP5-1009N12.1 | -0.242888968 | 3.290091254 | -3.864771995 | 0.000127496 |
| TCEAL3 | 0.347913585 | 5.24680398 | 3.864636227 | 0.000127564 |
| C10orf11 | -0.504017287 | 2.474761071 | -3.86437261 | 0.000127698 |
| HENMT1 | -0.417116024 | 5.69132602 | -3.864310942 | 0.000127729 |
| ARMC8 | 0.158188361 | 5.015721828 | 3.863904499 | 0.000127935 |
| FAM46A | 0.479677695 | 7.135306524 | 3.863725054 | 0.000128026 |
| TEKT4P2 | -0.722481265 | 0.411526919 | -3.863691445 | 0.000128043 |
| CLEC4F | -0.731011602 | -1.85641284 | -3.863092949 | 0.000128347 |
| CTB-32O4.2 | -0.402066982 | -0.287959533 | -3.862480388 | 0.000128659 |
| RP11-395B7.4 | -0.605713447 | -0.063492013 | -3.862333694 | 0.000128734 |
| TUBB3 | 0.573952181 | -3.424152774 | 3.86225472 | 0.000128774 |
| GLUD1P3 | 0.21894355 | 4.542009728 | 3.862169739 | 0.000128817 |
| STARD7 | -0.183259212 | 7.292818041 | -3.862151789 | 0.000128827 |
| GPR25 | -0.554961617 | -0.802393798 | -3.862144604 | 0.00012883 |
| RPL8 | 0.135463574 | 11.10402771 | 3.862037434 | 0.000128885 |
| KIF20A | 0.673347356 | 2.911559917 | 3.861880961 | 0.000128965 |
| SH3BGR | -0.352174587 | 1.807913014 | -3.861699277 | 0.000129058 |
| CTD-2033C11.1 | -0.44369986 | -0.309355346 | -3.861311413 | 0.000129256 |
| WWTR1 | 0.594664518 | -2.212559695 | 3.861047355 | 0.000129392 |
| ETV5 | 0.599566848 | 1.542126665 | 3.860965941 | 0.000129433 |
| ZNF816 | -0.280642302 | 2.428668447 | -3.860735926 | 0.000129551 |
| RP5-940J5.9 | 0.55513025 | 3.858125721 | 3.860644384 | 0.000129598 |
| PI16 | -0.595461055 | -0.702629543 | -3.859728176 | 0.00013007 |
| LRRC6 | -0.381738767 | 2.282542576 | -3.859313696 | 0.000130283 |
| PTMAP4 | 0.204515179 | 4.741755852 | 3.858713174 | 0.000130593 |
| FAM111B | 0.623563888 | 1.585517703 | 3.858470791 | 0.000130719 |
| EIF4A1P2 | -0.224987407 | 5.925316755 | -3.858334222 | 0.000130789 |
| SAAL1 | 0.163216405 | 4.564536272 | 3.858149332 | 0.000130885 |
| LDLR | 0.471199881 | 4.692135031 | 3.857836322 | 0.000131048 |
| KHDC1 | 0.468436824 | 0.765408551 | 3.857738602 | 0.000131098 |
| DNAJC19 | -0.136038888 | 3.779036653 | -3.857714213 | 0.000131111 |
| C12orf43 | 0.110083176 | 4.12962527 | 3.85723947 | 0.000131358 |
| RP13-46H24.1 | -0.475031819 | 0.433053084 | -3.856866868 | 0.000131551 |
| HIST1H2AC | -0.414686584 | 7.127270322 | -3.8556828 | 0.000132169 |
| C12orf4 | -0.177629917 | 3.956297594 | -3.855501663 | 0.000132264 |
| CDAN1 | 0.171724114 | 4.833461665 | 3.855492265 | 0.000132269 |
| WT1-AS | -1.130133073 | 0.975825648 | -3.855281064 | 0.00013238 |
| FAM81A | -0.724882687 | -0.866266956 | -3.854996388 | 0.000132529 |
| DLL4 | 0.493753628 | -2.401049096 | 3.854150695 | 0.000132973 |
| MAP6D1 | -0.297411802 | 2.328529036 | -3.85269704 | 0.00013374 |
| MTMR2 | 0.217166532 | 4.824941805 | 3.852679146 | 0.000133749 |
| TST | -0.411405376 | 4.475786777 | -3.85266101 | 0.000133759 |
| TMEM238 | 0.274718049 | 2.669371173 | 3.852523587 | 0.000133831 |
| SULT1C4 | -0.838659374 | 1.266451219 | -3.851381318 | 0.000134437 |
| PIK3IP1 | 0.452072626 | 6.054787827 | 3.850935922 | 0.000134674 |
| VPREB3 | -0.570870806 | 0.292359102 | -3.850255962 | 0.000135037 |
| RP11-563J2.2 | 0.409676118 | 1.108560978 | 3.849890813 | 0.000135232 |
| TPPP3 | -0.952840388 | 3.148051002 | -3.849792078 | 0.000135284 |
| LINC01140 | -0.501507887 | -2.331249135 | -3.848903082 | 0.00013576 |
| ATP8B3 | -0.359757305 | 2.916103991 | -3.84848791 | 0.000135983 |
| SYNPO2 | -0.569235041 | -2.128930064 | -3.84828911 | 0.00013609 |
| AC016700.3 | -0.381492844 | 0.695624258 | -3.847436676 | 0.000136549 |
| RP11-18I14.10 | -0.300222355 | 3.900965227 | -3.84721792 | 0.000136667 |
| FANK1 | -0.578224989 | -0.079734825 | -3.846753928 | 0.000136918 |
| TNS4 | 0.676595215 | -3.005183787 | 3.846526688 | 0.000137041 |
| KAT2A | -0.282203123 | 6.515390679 | -3.8459839 | 0.000137335 |
| RP11-533F5.2 | -0.359023252 | 0.187031654 | -3.845597665 | 0.000137545 |
| UQCC2 | -0.242529197 | 3.247293885 | -3.845114048 | 0.000137807 |
| RP11-627G1.1 | 0.356751827 | 0.528801561 | 3.844376802 | 0.000138209 |
| CTGLF10P | -0.380407065 | 1.011110383 | -3.84388832 | 0.000138476 |
| RP11-75L1.2 | -0.224607503 | 6.24499763 | -3.843452283 | 0.000138714 |
| PTPRCAP | -0.50226773 | 7.629810695 | -3.843421362 | 0.000138731 |
| RBBP8 | 0.209732033 | 5.002606887 | 3.843220171 | 0.000138842 |
| RP11-731C17.2 | -0.310635289 | 2.886707049 | -3.842610707 | 0.000139176 |
| SLC3A1 | 0.450520231 | -2.383169494 | 3.842507971 | 0.000139232 |
| RPL29P14 | 0.403934759 | 1.530466295 | 3.84249536 | 0.000139239 |
| AC004893.11 | -0.22775424 | 2.618014681 | -3.841705769 | 0.000139674 |
| TARDBPP2 | -0.560693093 | -0.446474973 | -3.841405071 | 0.000139839 |
| SPATA18 | -0.779166208 | -2.336975242 | -3.840896274 | 0.00014012 |
| NAPG | 0.173701077 | 5.177077019 | 3.840773911 | 0.000140188 |
| SLC7A6OS | -0.134022346 | 4.964681554 | -3.840746224 | 0.000140203 |
| NDUFA11 | 0.173175185 | 2.25247603 | 3.838592597 | 0.000141399 |
| RP11-403B2.7 | -0.64694935 | 0.924569716 | -3.838534524 | 0.000141431 |
| CBX3P2 | 0.23954578 | 2.620922625 | 3.837822099 | 0.000141829 |
| MN1 | 0.859949885 | 3.393780416 | 3.837470402 | 0.000142026 |
| TRAV38-1 | -0.474034593 | -0.896377163 | -3.837289265 | 0.000142127 |
| CCDC144CP | -0.451779488 | 0.21685805 | -3.837019415 | 0.000142279 |
| MDK | 0.553237367 | 5.088318055 | 3.836871868 | 0.000142361 |
| DBI | -0.278180545 | 7.544271595 | -3.836553484 | 0.00014254 |
| TRAV5 | -0.493074677 | -0.748450011 | -3.83623032 | 0.000142722 |
| CYSLTR2 | -0.610540519 | 3.441260223 | -3.836029642 | 0.000142835 |
| KLHL21 | 0.227809634 | 5.019461089 | 3.835389476 | 0.000143195 |
| PIAS2 | 0.164620576 | 3.895365517 | 3.834512598 | 0.000143691 |
| VAMP2 | 0.260675086 | 7.536412257 | 3.834194706 | 0.000143871 |
| BST2 | -0.396318717 | 8.050073752 | -3.83392273 | 0.000144025 |
| ALCAM | -0.385112359 | 5.852203795 | -3.833457418 | 0.000144289 |
| PLEKHS1 | 0.491749911 | -3.216810038 | 3.833451693 | 0.000144293 |
| DNAJC18 | -0.334889455 | 2.421178892 | -3.833209078 | 0.000144431 |
| BTBD3 | 0.269995838 | 5.037875386 | 3.832846256 | 0.000144637 |
| S100A4 | -0.383025527 | 9.658817399 | -3.831698072 | 0.000145292 |
| H1F0 | 0.766714215 | 8.758636471 | 3.831550736 | 0.000145377 |
| RP11-274B21.1 | 0.266970093 | 6.587256433 | 3.831398167 | 0.000145464 |
| RFXAP | -0.225567885 | 4.455211816 | -3.830573554 | 0.000145937 |
| MYO1D | 0.689077662 | 1.185386278 | 3.829966545 | 0.000146286 |
| MAFG | 0.204843922 | 6.292882679 | 3.829492445 | 0.000146559 |
| PRR5L | -0.571661142 | 3.307298069 | -3.829456967 | 0.000146579 |
| SH3BP5L | -0.267151757 | 4.671425284 | -3.829141308 | 0.000146762 |
| BIN2P2 | -0.422258097 | -0.78814905 | -3.828519036 | 0.000147121 |
| GNG2 | 0.481627086 | 6.012978981 | 3.828115491 | 0.000147355 |
| SEC23A | -0.143828566 | 4.73284788 | -3.827842716 | 0.000147513 |
| CLDN7 | -0.573468799 | 0.493096741 | -3.827625331 | 0.000147639 |
| DHX29 | 0.102556921 | 5.634670848 | 3.827409012 | 0.000147765 |
| PLXNA4 | 0.580399221 | -0.772930069 | 3.827312028 | 0.000147821 |
| AC004797.1 | 0.145018788 | 6.3034697 | 3.8272314 | 0.000147868 |
| RP11-737O24.3 | -0.246166342 | 4.111033502 | -3.827165321 | 0.000147907 |
| NDUFB6 | -0.163589703 | 5.232547141 | -3.826730412 | 0.00014816 |
| GTF3C6 | -0.167870208 | 6.298521212 | -3.82576832 | 0.000148722 |
| NACA3P | -0.148386092 | 7.328528181 | -3.825750025 | 0.000148732 |
| CDC37L1 | 0.17530909 | 3.75829918 | 3.8253054 | 0.000148992 |
| CD320 | 0.356074386 | 3.845408315 | 3.825286793 | 0.000149003 |
| RP11-378G13.2 | 0.410021134 | 1.445792213 | 3.825204914 | 0.000149051 |
| ZNF566 | -0.237483266 | 1.94068262 | -3.824703382 | 0.000149345 |
| MAP2K4P1 | -0.313643629 | 0.735361584 | -3.824458331 | 0.000149489 |
| RP11-16C1.2 | 0.527925041 | 0.908940264 | 3.824420773 | 0.000149511 |
| SLC25A10 | 0.346954585 | 1.239618792 | 3.823954142 | 0.000149786 |
| EWSR1 | 0.13117114 | 7.514692447 | 3.823557356 | 0.00015002 |
| PSMC1P9 | 0.340997016 | 0.60651991 | 3.823501384 | 0.000150053 |
| SYNE3 | 0.281634989 | 4.246640929 | 3.823274525 | 0.000150186 |
| HCK | -0.501781105 | 7.262360851 | -3.82299164 | 0.000150353 |
| CHRDL1 | 1.219831689 | -0.633902273 | 3.822264283 | 0.000150784 |
| RP11-43F13.1 | 0.363947193 | 2.554777291 | 3.822134813 | 0.00015086 |
| RP5-1148A21.3 | -0.293784802 | 0.72949342 | -3.821622237 | 0.000151164 |
| CXCL1 | 0.864289691 | 3.829606685 | 3.821610921 | 0.000151171 |
| RP3-425P12.4 | 0.207366649 | 4.958653317 | 3.821254303 | 0.000151383 |
| HYAL1 | -0.463987767 | -0.972274883 | -3.821150123 | 0.000151445 |
| ZDHHC19 | 0.714414141 | -0.241935275 | 3.821017883 | 0.000151524 |
| TCP1 | 0.178194113 | 7.612279281 | 3.820577892 | 0.000151786 |
| RASSF3 | -0.335524995 | 6.412519469 | -3.820446245 | 0.000151864 |
| WDR61 | -0.170472171 | 4.425444542 | -3.819897522 | 0.000152192 |
| UCHL3 | 0.179428171 | 3.483628288 | 3.819554525 | 0.000152397 |
| AC009299.3 | 0.798437869 | 2.039468231 | 3.81942569 | 0.000152474 |
| MGAT1 | -0.215301331 | 7.24698202 | -3.81939261 | 0.000152494 |
| FABP3 | 0.605175704 | -1.120112672 | 3.81915249 | 0.000152638 |
| PCGF1 | 0.185595731 | 4.345118631 | 3.818973356 | 0.000152745 |
| ACTB | -0.14576767 | 11.99204786 | -3.818808018 | 0.000152844 |
| ELTD1 | 0.594715697 | -2.994804541 | 3.81852728 | 0.000153013 |
| ARMC12 | 0.368000794 | 1.71091919 | 3.818216182 | 0.0001532 |
| AHR | 0.514542732 | 6.726732931 | 3.818172703 | 0.000153226 |
| RP11-250B2.6 | -0.364095613 | 0.244951604 | -3.817458214 | 0.000153656 |
| ZBTB1 | 0.220095319 | 6.334530506 | 3.817257576 | 0.000153777 |
| SYNE4 | -0.353314169 | -0.114880744 | -3.817084723 | 0.000153882 |
| PHF10 | 0.192182764 | 6.3781159 | 3.817038224 | 0.00015391 |
| CTB-61M7.2 | 0.872282562 | 5.231245304 | 3.816993356 | 0.000153937 |
| SLC41A2 | 0.533942989 | -0.312015903 | 3.816696383 | 0.000154116 |
| FOCAD | -0.280431387 | 3.741965952 | -3.816543464 | 0.000154209 |
| FCRLA | -0.786436569 | 0.350792702 | -3.816144686 | 0.00015445 |
| MYEF2 | 0.854272977 | 1.565190603 | 3.815921466 | 0.000154585 |
| RP11-340I6.8 | -0.57804679 | -0.263037655 | -3.815843462 | 0.000154633 |
| RP11-396F22.1 | -0.646455094 | 0.85110608 | -3.814950313 | 0.000155175 |
| LYPLA1P3 | -0.199896843 | 6.337695503 | -3.814825356 | 0.000155252 |
| ATP6V1A | -0.22014631 | 6.248221397 | -3.814775727 | 0.000155282 |
| RP11-213G21.1 | -0.49099707 | -1.349258629 | -3.814439585 | 0.000155487 |
| MEAF6 | -0.111520948 | 6.324390811 | -3.814228579 | 0.000155615 |
| RP11-318M2.2 | 0.67196011 | -0.147872941 | 3.813451221 | 0.00015609 |
| MAMDC4 | -0.296086631 | 3.344366186 | -3.813433766 | 0.000156101 |
| SLTM | -0.114490284 | 6.953952174 | -3.813034086 | 0.000156346 |
| IRS1 | 0.55574291 | -0.261060194 | 3.813032766 | 0.000156347 |
| ACP2 | -0.237422203 | 4.546765261 | -3.812791556 | 0.000156495 |
| ZNF576 | -0.212689684 | 2.567286092 | -3.812787566 | 0.000156497 |
| AC104809.4 | -1.133280591 | 1.169675912 | -3.812340248 | 0.000156772 |
| ARG1 | 1.016209152 | 4.830624047 | 3.811405829 | 0.000157347 |
| CIRH1A | 0.160055526 | 5.110506326 | 3.811087102 | 0.000157544 |
| MAP4K4 | 0.220713207 | 6.667346538 | 3.810495134 | 0.00015791 |
| ITPR3 | 0.480774429 | 3.26330618 | 3.810323077 | 0.000158016 |
| C14orf64 | -0.619967026 | -0.862552619 | -3.809805448 | 0.000158337 |
| RP11-76E17.3 | -0.596616312 | 2.190906343 | -3.809612312 | 0.000158457 |
| RP11-549K20.1 | -0.61504645 | -1.042383057 | -3.80835367 | 0.00015924 |
| RPL21P44 | -0.442000941 | 0.665999047 | -3.80707259 | 0.000160041 |
| HSD11B1L | 0.266511088 | 1.584069926 | 3.805983248 | 0.000160725 |
| CTB-175E5.7 | -0.399788403 | 1.112496873 | -3.805642399 | 0.000160939 |
| CYTH1 | 0.235512567 | 6.787928674 | 3.804951862 | 0.000161375 |
| DNASE2 | -0.374867289 | 5.533797037 | -3.804787661 | 0.000161479 |
| QPCTL | 0.229021904 | 4.421211845 | 3.804609077 | 0.000161591 |
| RP11-87C12.5 | -0.563808573 | 0.183961314 | -3.80460111 | 0.000161597 |
| RP11-49K24.4 | -0.448400555 | 0.305986395 | -3.803726504 | 0.000162151 |
| RP11-90P5.5 | 0.381855439 | 0.376547691 | 3.80353131 | 0.000162274 |
| ALKBH2 | -0.224589101 | 3.932788089 | -3.80308917 | 0.000162555 |
| PHKG2 | -0.147350321 | 4.427139962 | -3.802588655 | 0.000162874 |
| ZNF681 | -0.436102021 | 2.012174664 | -3.802509404 | 0.000162924 |
| CXXC1 | -0.131677851 | 6.47020874 | -3.802093311 | 0.00016319 |
| MT-TW | 0.37661926 | 5.403595991 | 3.801919987 | 0.0001633 |
| HFE | -0.695828689 | -0.795997524 | -3.801827301 | 0.00016336 |
| ZNF254 | 0.246412719 | 4.355309126 | 3.801519385 | 0.000163557 |
| AZI1 | 0.177320414 | 4.717541949 | 3.801038043 | 0.000163865 |
| LMCD1 | 0.573931511 | -0.912558261 | 3.800004232 | 0.000164528 |
| MFSD2B | 0.705230907 | 1.865828774 | 3.798642523 | 0.000165406 |
| RP11-20O24.4 | -0.16662887 | 9.935948361 | -3.798032142 | 0.000165801 |
| RP1-43E13.2 | -0.195677078 | 3.322112429 | -3.797820536 | 0.000165939 |
| CLDND2 | 0.306852181 | 3.046564206 | 3.797493387 | 0.000166151 |
| RP11-495P10.10 | 0.351759594 | -0.894118674 | 3.797455409 | 0.000166176 |
| GS1-124K5.4 | -0.304663271 | 1.937892577 | -3.797282096 | 0.000166288 |
| NPR2 | -0.46155786 | 0.979220177 | -3.797197812 | 0.000166343 |
| SPARCL1 | 0.692800481 | -2.95450376 | 3.795570636 | 0.000167404 |
| TUBB2B | 0.536901589 | -1.269439709 | 3.795112398 | 0.000167703 |
| NBN | 0.177454394 | 6.005024619 | 3.794909231 | 0.000167837 |
| CRIP3 | -0.641644444 | -0.134529732 | -3.794074158 | 0.000168385 |
| CD160 | -0.582069855 | -0.178114119 | -3.79394469 | 0.00016847 |
| CTD-2020K17.1 | -0.284575355 | 4.861921122 | -3.793329202 | 0.000168875 |
| SETP17 | -0.394280636 | 0.114963648 | -3.793291067 | 0.0001689 |
| FSCN3 | 0.438805534 | -0.426588427 | 3.792973695 | 0.00016911 |
| FAM154B | -0.559305334 | -0.951953453 | -3.79277265 | 0.000169242 |
| MRE11A | -0.157828022 | 5.557176664 | -3.792726369 | 0.000169273 |
| ZNF417 | 0.199688734 | 3.990573989 | 3.792216026 | 0.00016961 |
| RP11-141P6.1 | 0.412614934 | 0.290758155 | 3.792185747 | 0.000169631 |
| XRRA1 | -0.393067098 | 2.031327054 | -3.791672994 | 0.00016997 |
| MRPS30 | -0.138997324 | 5.102152235 | -3.791333098 | 0.000170196 |
| RP4-816N1.7 | -0.480299997 | -0.40264075 | -3.790947258 | 0.000170452 |
| MVP | -0.272536565 | 6.308776923 | -3.790716661 | 0.000170606 |
| XPNPEP2 | 0.547689711 | -0.641100876 | 3.790426668 | 0.000170799 |
| VDR | 0.566607431 | 3.37680768 | 3.790111978 | 0.000171009 |
| AC008982.2 | 0.306235023 | 4.426187525 | 3.789762582 | 0.000171242 |
| AC124890.1 | -0.461291157 | -0.396585554 | -3.789531901 | 0.000171396 |
| ZNF827 | 0.617959344 | 2.749391883 | 3.789498361 | 0.000171418 |
| RP11-452D12.1 | -0.481785783 | -1.011571231 | -3.789205719 | 0.000171614 |
| HSPA7 | -0.816233779 | 3.566792021 | -3.789195444 | 0.000171621 |
| SLPI | 0.909772172 | 5.457447986 | 3.789193869 | 0.000171622 |
| TMEM127 | 0.200531929 | 6.701092011 | 3.788586941 | 0.000172029 |
| PPM1L | -0.317640045 | 3.513617924 | -3.788548606 | 0.000172055 |
| HMGN2P4 | 0.248601792 | 4.4144772 | 3.787765059 | 0.000172581 |
| LINC00032 | -0.543105158 | -2.310291637 | -3.786566396 | 0.000173389 |
| EXOG | 0.167663675 | 3.380432056 | 3.786481474 | 0.000173447 |
| ALG1L10P | -0.530570024 | -0.110006549 | -3.786089586 | 0.000173712 |
| KLRAP1 | -0.284233523 | 2.496188546 | -3.785242252 | 0.000174287 |
| SPATA32 | -0.47052 | -0.695518085 | -3.785033272 | 0.000174429 |
| RP5-1071N3.1 | 0.488861425 | -1.044187942 | 3.784309876 | 0.000174921 |
| PGF | 0.63927701 | -2.379281537 | 3.784304288 | 0.000174925 |
| PSME2P2 | -0.225920155 | 5.227233808 | -3.783809924 | 0.000175262 |
| YPEL2 | 0.279152588 | 5.187047574 | 3.783578903 | 0.00017542 |
| ZNF224 | -0.152356004 | 4.406081845 | -3.783005128 | 0.000175812 |
| RP11-403I13.8 | -0.444781103 | 4.172947824 | -3.782815221 | 0.000175942 |
| COL6A1 | 0.771958281 | 2.087084403 | 3.782623113 | 0.000176074 |
| TNRC6B | -0.166039414 | 5.643931745 | -3.782109363 | 0.000176427 |
| HSBP1 | -0.200718026 | 5.196644698 | -3.781515928 | 0.000176835 |
| CCNY | 0.171107571 | 7.053101466 | 3.781453193 | 0.000176878 |
| CTD-3236F5.1 | 0.402592782 | 0.970817156 | 3.781148495 | 0.000177088 |
| SLCO5A1 | 0.978534304 | -0.054993534 | 3.780992952 | 0.000177195 |
| RP11-246A10.1 | -0.525875338 | -0.252198188 | -3.780984045 | 0.000177201 |
| NDRG4 | 0.623240035 | -4.497318792 | 3.780585541 | 0.000177476 |
| SGMS1 | 0.276987552 | 3.995829785 | 3.780505968 | 0.000177531 |
| CYR61 | 0.688903223 | 0.763753309 | 3.780322426 | 0.000177658 |
| HSF4 | -0.296436349 | 2.411978811 | -3.780041737 | 0.000177853 |
| C1orf233 | -0.390238979 | 5.052893768 | -3.779739363 | 0.000178062 |
| CCDC125 | -0.271521841 | 4.688899957 | -3.77913542 | 0.000178481 |
| GPX4 | 0.161861765 | 8.409011137 | 3.779015569 | 0.000178564 |
| RP11-769O8.3 | 0.579906193 | -0.286925963 | 3.778880987 | 0.000178658 |
| IGKV2-24 | -0.805506882 | 3.329728824 | -3.778653349 | 0.000178816 |
| SPECC1 | -0.307825543 | 4.657660725 | -3.778170581 | 0.000179152 |
| YWHAZP6 | 0.234903973 | 3.096769689 | 3.777708878 | 0.000179474 |
| FGF11 | -0.453596575 | -1.024200782 | -3.777674035 | 0.000179499 |
| TUBA1C | 0.219248093 | 7.327610082 | 3.777024488 | 0.000179953 |
| ZNF649 | 0.206803959 | 3.252895034 | 3.77699824 | 0.000179971 |
| PLOD3 | -0.237103357 | 5.501563948 | -3.776865641 | 0.000180064 |
| IARS | -0.236286359 | 6.065259874 | -3.776708188 | 0.000180174 |
| MTHFD1L | 0.298752697 | 4.457654001 | 3.776310268 | 0.000180453 |
| THOC5 | 0.110568818 | 4.759884305 | 3.775755534 | 0.000180843 |
| RP11-552M11.8 | 0.379707395 | 1.81659871 | 3.775533811 | 0.000180999 |
| LINC00211 | -0.640599728 | 1.301665867 | -3.775503345 | 0.000181021 |
| DEPDC5 | 0.151826115 | 3.919753851 | 3.775117384 | 0.000181292 |
| IMPG2 | -0.42723033 | -0.378169831 | -3.77507279 | 0.000181324 |
| KIR2DP1 | 0.535699662 | -1.5349597 | 3.774623128 | 0.000181641 |
| C20orf96 | -0.322215596 | 5.55105866 | -3.774620074 | 0.000181643 |
| TAGLN2P1 | -0.31375747 | 8.228221959 | -3.773886625 | 0.000182162 |
| FAM104B | -0.184970706 | 3.734616736 | -3.773141681 | 0.00018269 |
| SH3D19 | 0.552122456 | -1.367694957 | 3.772998126 | 0.000182792 |
| RP11-600F24.7 | -0.366711357 | 0.668252234 | -3.772687235 | 0.000183013 |
| FLRT2 | 0.59146884 | -4.851617957 | 3.772646771 | 0.000183042 |
| RCN1 | -0.270654015 | 4.994932136 | -3.772604921 | 0.000183072 |
| C7orf55 | -0.339018704 | 0.810392558 | -3.77197176 | 0.000183523 |
| CYHR1 | -0.117269707 | 4.544657791 | -3.770288967 | 0.000184726 |
| ZC3H6 | -0.253455531 | 2.773025794 | -3.770264921 | 0.000184743 |
| KLF11 | 0.560450723 | 5.504730639 | 3.769988315 | 0.000184942 |
| AL049542.1 | -0.456494223 | 0.969485247 | -3.769641652 | 0.000185191 |
| CDKN2C | 0.479807822 | 4.836954112 | 3.769632175 | 0.000185198 |
| MAP3K13 | 0.60434844 | 1.401712461 | 3.769480884 | 0.000185307 |
| LINC00493 | -0.154914381 | 6.402138564 | -3.769329837 | 0.000185416 |
| RP3-426I6.5 | -0.430757699 | 0.548908396 | -3.768953595 | 0.000185687 |
| RTP4 | -0.499875377 | 2.683709424 | -3.768620682 | 0.000185927 |
| RAPSN | 0.49318217 | -0.050987209 | 3.768183943 | 0.000186242 |
| ZNF547 | -0.37188123 | 0.345295917 | -3.7681671 | 0.000186255 |
| C2orf62 | -0.496578807 | -1.914674001 | -3.768121952 | 0.000186287 |
| ARL3 | -0.186402442 | 3.695980153 | -3.768060446 | 0.000186332 |
| COL18A1 | 0.489379302 | 3.443533964 | 3.767989132 | 0.000186383 |
| CTDSP1 | -0.127041553 | 7.049744032 | -3.767872662 | 0.000186468 |
| ICA1L | 0.318937307 | 2.236335284 | 3.767418732 | 0.000186796 |
| RP11-34E5.4 | -0.365534162 | 1.434208656 | -3.766548298 | 0.000187429 |
| DGAT2 | 0.703529674 | 1.928441093 | 3.766298353 | 0.00018761 |
| PTCD1 | 0.217902276 | 1.875957283 | 3.766291821 | 0.000187615 |
| ARHGEF10 | 0.558286262 | 0.792725001 | 3.766287954 | 0.000187618 |
| RP11-755B10.3 | 0.411288581 | -2.018310014 | 3.765468249 | 0.000188216 |
| RHBDD3 | 0.149839379 | 4.326394064 | 3.765106751 | 0.00018848 |
| RP11-734K2.4 | -0.259778471 | 1.785672985 | -3.764212631 | 0.000189135 |
| RP3-375P9.2 | 0.236381925 | 1.77168624 | 3.764086126 | 0.000189228 |
| AP000282.2 | -0.504331934 | -1.12712163 | -3.762792001 | 0.00019018 |
| AL157871.2 | -0.437153793 | -0.162969452 | -3.761068937 | 0.000191454 |
| PIAS1 | 0.150792956 | 6.320673768 | 3.760703218 | 0.000191726 |
| CMTM5 | 0.901408333 | -0.126935513 | 3.760686666 | 0.000191738 |
| NAV2 | 0.600302596 | -1.347327169 | 3.759901312 | 0.000192323 |
| LECT1 | 0.493890956 | -2.081304495 | 3.759872529 | 0.000192344 |
| RNF222 | 0.48951476 | -2.615815673 | 3.758979217 | 0.000193011 |
| RP11-680G24.5 | -0.568986224 | -0.429606099 | -3.758974546 | 0.000193014 |
| NEDD9 | 0.437316668 | 3.750542993 | 3.758302384 | 0.000193518 |
| ZBTB11 | 0.176413528 | 5.469263937 | 3.757548565 | 0.000194084 |
| LL0XNC01-116E7.2 | -0.482908742 | 0.895290993 | -3.757422143 | 0.000194179 |
| MTF1 | 0.259759134 | 5.227061266 | 3.757312092 | 0.000194261 |
| IGBP1 | -0.162266944 | 7.497838405 | -3.756886082 | 0.000194582 |
| FZD7 | 0.53815174 | 1.351577672 | 3.756768679 | 0.000194671 |
| UBXN8 | -0.242441917 | 3.440797007 | -3.756709515 | 0.000194715 |
| CKB | 0.527277391 | 4.155895376 | 3.756382439 | 0.000194962 |
| ZNF492 | 0.579863353 | 0.744158892 | 3.756170138 | 0.000195122 |
| IRF1 | 0.326680518 | 6.473081949 | 3.754766689 | 0.000196185 |
| AF131217.1 | -0.437534526 | -2.506934934 | -3.754189423 | 0.000196624 |
| NDUFB1P1 | -0.462903945 | 0.027713189 | -3.754035376 | 0.000196741 |
| RP11-339B21.11 | 0.211729469 | 2.843413052 | 3.753612866 | 0.000197063 |
| CAND1 | 0.129450336 | 5.208578327 | 3.753398138 | 0.000197227 |
| EEF2K | -0.200106908 | 5.08073533 | -3.753135202 | 0.000197428 |
| NEK5 | -0.64580136 | -0.848424295 | -3.75184051 | 0.000198419 |
| TMEM17 | -0.428925377 | 0.715279444 | -3.751734733 | 0.000198501 |
| IPMK | -0.246980861 | 4.673183599 | -3.751610966 | 0.000198596 |
| OMG | 0.480120805 | -0.073435474 | 3.75151118 | 0.000198672 |
| FKBP5 | -0.373827597 | 6.713356162 | -3.751503384 | 0.000198678 |
| IER3 | 0.747492144 | 6.126284943 | 3.751260779 | 0.000198865 |
| SCAPER | 0.155041389 | 3.115918028 | 3.750552657 | 0.00019941 |
| MAP1A | -0.705659832 | 3.670696316 | -3.750393139 | 0.000199533 |
| RPLP2 | 0.154599926 | 10.06321997 | 3.750266938 | 0.000199631 |
| PRKCB | -0.243583876 | 7.1263381 | -3.75014515 | 0.000199725 |
| KCTD1 | -0.55646584 | 1.13142779 | -3.749690494 | 0.000200076 |
| RP11-454L9.2 | 0.321610981 | 2.63888357 | 3.74918076 | 0.000200471 |
| AKAP7 | 0.436135192 | 3.453041845 | 3.749028608 | 0.000200589 |
| MIR210HG | 0.687186915 | -0.062394499 | 3.747972655 | 0.000201409 |
| KIAA0020 | -0.182236761 | 5.717283282 | -3.747089732 | 0.000202097 |
| FTSJ1 | -0.106717163 | 5.872152837 | -3.746694537 | 0.000202406 |
| VIM-AS1 | -0.280063692 | 4.567733341 | -3.746531998 | 0.000202533 |
| PTPMT1 | 0.16274526 | 4.565652635 | 3.746145404 | 0.000202836 |
| MAMDC2 | -1.123961591 | 3.663971712 | -3.74601992 | 0.000202934 |
| GOLGA5P1 | -0.479275395 | -0.247863365 | -3.745956101 | 0.000202984 |
| SNORD3B-2 | -0.661366689 | 1.79301048 | -3.74586054 | 0.000203059 |
| ZER1 | -0.227779435 | 6.194651995 | -3.745590013 | 0.000203272 |
| TMEM64 | 0.344575198 | 2.953709056 | 3.745199716 | 0.000203578 |
| RP11-96D1.3 | -0.403300148 | 0.262279484 | -3.744964992 | 0.000203763 |
| SEC14L6 | 0.437546592 | -3.366068217 | 3.744210287 | 0.000204358 |
| AC123768.3 | -0.610494339 | -0.330588255 | -3.744112603 | 0.000204435 |
| PI4KA | -0.121480616 | 6.564595755 | -3.743179034 | 0.000205173 |
| RASGRP1 | 0.510980174 | 3.137753137 | 3.743170741 | 0.00020518 |
| TIFAB | -1.027839379 | -0.434555189 | -3.742521952 | 0.000205694 |
| ZNF658 | -0.380870989 | 1.05714102 | -3.742467294 | 0.000205738 |
| GOT2 | -0.186394724 | 5.88681513 | -3.742427893 | 0.000205769 |
| PTGES3P4 | -0.476793806 | -0.275155725 | -3.742354247 | 0.000205827 |
| APLNR | 0.610703359 | -3.070952225 | 3.741841915 | 0.000206235 |
| AC090945.1 | -0.454534841 | 0.131151715 | -3.740787254 | 0.000207076 |
| ABCG1 | 0.525331091 | 3.171685893 | 3.740775428 | 0.000207085 |
| ALG14 | -0.328739333 | -0.01258225 | -3.740592349 | 0.000207232 |
| LBX2-AS1 | -0.463510083 | 2.514111992 | -3.740341136 | 0.000207433 |
| RP11-455F5.3 | -0.415566421 | -0.396154008 | -3.740013012 | 0.000207696 |
| FLVCR1 | 0.183979038 | 3.638293945 | 3.740001905 | 0.000207704 |
| ATG7 | -0.270599436 | 3.977191648 | -3.739867719 | 0.000207812 |
| MAGED2 | -0.219483321 | 6.764102499 | -3.739810724 | 0.000207858 |
| TYK2 | -0.116144905 | 7.163542269 | -3.739050793 | 0.000208468 |
| DNMT1 | 0.21095914 | 6.288159642 | 3.738580344 | 0.000208847 |
| ADCYAP1 | 0.705297157 | -3.257278209 | 3.736696693 | 0.000210369 |
| C1orf162 | -0.432212908 | 7.143309129 | -3.736649231 | 0.000210407 |
| HAUS7 | 0.268484771 | 0.881379025 | 3.736260978 | 0.000210723 |
| AP001468.1 | -0.377923116 | 1.615598866 | -3.735951157 | 0.000210974 |
| KCNJ5 | 0.562162545 | -2.149063664 | 3.735604096 | 0.000211257 |
| RHCE | 0.737673012 | 0.451883633 | 3.735428714 | 0.0002114 |
| RP3-406P24.3 | -0.425845956 | 0.435316973 | -3.735232965 | 0.000211559 |
| HNRNPA1P4 | 0.187727739 | 4.68060311 | 3.734964258 | 0.000211778 |
| SLC26A1 | -0.411703851 | -1.067116911 | -3.734678835 | 0.000212011 |
| METAP2 | 0.141608654 | 6.568417373 | 3.734436568 | 0.000212209 |
| HSBP1L1 | 0.498700448 | 4.147591056 | 3.734392903 | 0.000212245 |
| PSMD8 | 0.106711691 | 7.185073721 | 3.732796308 | 0.000213555 |
| HCG4P5 | 0.512536369 | 1.545156563 | 3.732404652 | 0.000213877 |
| IL10RA | 0.558269217 | 6.254620255 | 3.732318267 | 0.000213948 |
| POLR2D | 0.131541908 | 6.011975209 | 3.732309386 | 0.000213956 |
| RP11-10J21.4 | 0.424704029 | 1.300887065 | 3.731781775 | 0.000214391 |
| HERC2P5 | -0.492485126 | -1.562929608 | -3.731287478 | 0.000214799 |
| LL0XNC01-237H1.2 | -0.246743901 | 2.127035937 | -3.731272667 | 0.000214812 |
| AMZ1 | 0.632497762 | -2.134405119 | 3.731110642 | 0.000214946 |
| TMEM63B | 0.307400583 | 3.948740026 | 3.730789324 | 0.000215212 |
| FAM161A | -0.34346759 | 1.717911777 | -3.730733821 | 0.000215258 |
| SDHDP6 | -0.337126084 | 1.560667911 | -3.730714116 | 0.000215274 |
| TDRKH | -0.433840597 | 1.437582284 | -3.730582658 | 0.000215383 |
| TMEM108 | 0.618076431 | -4.444597356 | 3.730217158 | 0.000215686 |
| RP11-687F6.4 | 0.492186419 | -0.452123247 | 3.730056782 | 0.000215819 |
| ACTR1A | -0.107834276 | 6.765736402 | -3.728753435 | 0.000216905 |
| PWP1 | -0.137180038 | 6.264671435 | -3.72862396 | 0.000217013 |
| DDIT4L | 1.088796751 | -0.268135259 | 3.728121012 | 0.000217433 |
| AP3M1 | -0.149837461 | 5.969742489 | -3.7279766 | 0.000217554 |
| SLC25A1 | -0.19082995 | 6.740684682 | -3.727661433 | 0.000217818 |
| TTLL4 | -0.223411676 | 4.19197683 | -3.727469041 | 0.000217979 |
| CTD-2122P11.1 | 0.335616681 | 1.608700761 | 3.727330895 | 0.000218095 |
| CTD-2313N18.5 | -0.397819601 | -1.824014193 | -3.727056206 | 0.000218326 |
| G6PD | -0.254237515 | 7.255852457 | -3.726764557 | 0.000218571 |
| SCARNA4 | 0.37302171 | 1.617123986 | 3.726723223 | 0.000218606 |
| RP11-54C4.1 | -0.33255062 | 1.337572165 | -3.72606906 | 0.000219156 |
| AC009948.7 | -0.179808436 | 4.743865053 | -3.725411964 | 0.000219711 |
| ATP1B2 | 0.857612314 | -0.410166315 | 3.724453403 | 0.000220522 |
| GTF2H2 | -0.24941673 | 2.296682489 | -3.724364218 | 0.000220598 |
| CYGB | 0.823382735 | -0.787407674 | 3.724308301 | 0.000220645 |
| RP11-1084E5.1 | -0.696078208 | -0.856170825 | -3.723888106 | 0.000221002 |
| HNRNPAB | -0.247662262 | 8.518602964 | -3.723248821 | 0.000221546 |
| AC005740.6 | -0.384420777 | -0.153596554 | -3.723212421 | 0.000221577 |
| FBXO27 | 0.495269556 | -2.811417875 | 3.723201277 | 0.000221586 |
| RP11-35J23.1 | -0.774581398 | -0.742754273 | -3.72294118 | 0.000221808 |
| SCARNA13 | -0.350160197 | 1.60980422 | -3.722798893 | 0.000221929 |
| KHK | -0.373317842 | 1.904209926 | -3.72245689 | 0.000222221 |
| BMP2K | -0.256187447 | 5.371617415 | -3.722186088 | 0.000222452 |
| KIF21B | 0.332242539 | 5.137041945 | 3.721927301 | 0.000222674 |
| MBNL2 | 0.258728186 | 4.762959864 | 3.721561429 | 0.000222987 |
| WASH3P | 0.166516373 | 5.291681566 | 3.72143854 | 0.000223092 |
| GMPR | 0.564325866 | 3.768919878 | 3.721239599 | 0.000223263 |
| CCDC180 | -0.458647508 | -0.308047284 | -3.721213034 | 0.000223286 |
| CCR9 | 0.554348715 | -1.276853666 | 3.720696389 | 0.000223729 |
| CMPK2 | 0.384738127 | 4.414049117 | 3.720529335 | 0.000223873 |
| AC114755.7 | -0.405734405 | -0.279318364 | -3.72048447 | 0.000223912 |
| RP11-869B15.1 | -0.328045947 | 3.07633147 | -3.719729027 | 0.000224562 |
| CCDC81 | -0.363477679 | -0.890591106 | -3.719518522 | 0.000224744 |
| TAS2R13 | -0.46081034 | -1.26476255 | -3.719158353 | 0.000225055 |
| RNY4P7 | 0.419337327 | 0.100306939 | 3.719153116 | 0.000225059 |
| HS3ST2 | 0.528159131 | -3.055458664 | 3.718994277 | 0.000225197 |
| CAND2 | 0.549038353 | -1.056708242 | 3.718895341 | 0.000225282 |
| CTB-26E19.1 | -0.711893308 | -0.984929382 | -3.718826337 | 0.000225342 |
| MAGI2 | -0.552858804 | -1.166248658 | -3.718669295 | 0.000225478 |
| CTC-498J12.3 | -0.408057224 | -0.297070579 | -3.718457539 | 0.000225661 |
| XXbac-BPG252P9.10 | 0.504486132 | 0.591856306 | 3.718233769 | 0.000225855 |
| TP53I11 | 0.532017093 | 4.543604658 | 3.718090969 | 0.000225979 |
| DDX1 | -0.156770732 | 6.451593898 | -3.717881306 | 0.000226161 |
| RP4-756G23.5 | -0.36708066 | 1.085578191 | -3.71735449 | 0.000226619 |
| GOT2P3 | -0.394919775 | -0.610590247 | -3.717205816 | 0.000226748 |
| CTD-2609K8.3 | -0.474459296 | 0.685601203 | -3.717185582 | 0.000226766 |
| EIF2S2P3 | -0.217684496 | 4.067865103 | -3.717183555 | 0.000226768 |
| RP11-567G24.1 | 0.192509037 | 3.163538618 | 3.717142543 | 0.000226803 |
| CAMK2A | 0.699533553 | -3.443647775 | 3.716261738 | 0.000227571 |
| STK11IP | -0.135704157 | 5.115759053 | -3.714950091 | 0.00022872 |
| TLR7 | -0.848813171 | 1.368872818 | -3.71466318 | 0.000228972 |
| GAPDHP70 | 0.383918144 | 1.921967868 | 3.714341163 | 0.000229255 |
| EGFR | 0.84444446 | -5.756882513 | 3.714190838 | 0.000229387 |
| PNISR | -0.151002421 | 7.59255431 | -3.713854991 | 0.000229682 |
| AC092198.1 | 0.664048195 | -1.603342147 | 3.712535031 | 0.000230848 |
| CCDC18 | -0.187762141 | 4.328593503 | -3.712494085 | 0.000230884 |
| RSPRY1 | -0.136138972 | 4.773125441 | -3.712411604 | 0.000230957 |
| SLC38A11 | -0.638814739 | -2.809483424 | -3.712008726 | 0.000231314 |
| RP11-110I1.13 | -0.296122431 | 1.272394141 | -3.711931136 | 0.000231383 |
| HES6 | 0.570064813 | 3.449716145 | 3.71152722 | 0.000231742 |
| RNU11 | -0.524055468 | 2.260112025 | -3.711138069 | 0.000232088 |
| SC5D | 0.197348531 | 4.28785023 | 3.711108903 | 0.000232114 |
| PSMA7 | -0.116254968 | 8.031084697 | -3.710924947 | 0.000232277 |
| MTHFD2P7 | 0.31215051 | 2.132786045 | 3.710243899 | 0.000232885 |
| GK-IT1 | 0.529340432 | -0.377360561 | 3.709282253 | 0.000233744 |
| ZHX1-C8ORF76 | 0.40290431 | -0.032636086 | 3.709136081 | 0.000233875 |
| RAB43 | 0.440149396 | 0.864195946 | 3.708634362 | 0.000234325 |
| BCAS2 | 0.17215804 | 6.667810828 | 3.70858961 | 0.000234365 |
| ADH5 | -0.136615857 | 5.359808315 | -3.708010603 | 0.000234886 |
| EIF4BP5 | 0.330704128 | 0.35913196 | 3.707649103 | 0.000235211 |
| RP11-597A11.4 | -0.329823549 | 0.264320321 | -3.707627697 | 0.000235231 |
| CLUHP3 | 0.519980975 | 2.529009433 | 3.707489675 | 0.000235355 |
| DOPEY1 | -0.158497813 | 4.003598295 | -3.707144154 | 0.000235667 |
| NLRP9P | 0.590879659 | -1.84699122 | 3.707134519 | 0.000235675 |
| ZNF480 | -0.192184393 | 4.314330441 | -3.707131558 | 0.000235678 |
| AFAP1L2 | -0.579654052 | -0.360248395 | -3.706336369 | 0.000236397 |
| TAB2 | 0.212458383 | 7.301367875 | 3.706095823 | 0.000236615 |
| MAD1L1 | -0.165870815 | 4.248018048 | -3.706041095 | 0.000236664 |
| CCDC66 | -0.172333894 | 3.628429686 | -3.705690929 | 0.000236982 |
| RP11-178D12.2 | -0.384396969 | 0.211676617 | -3.705512786 | 0.000237143 |
| MTX1 | 0.179736941 | 4.860068704 | 3.705058333 | 0.000237556 |
| AC009303.3 | 0.346082688 | 0.32865038 | 3.704792088 | 0.000237799 |
| KB-1208A12.3 | 0.574471135 | 0.307703837 | 3.704784607 | 0.000237805 |
| RAD51D | -0.179213072 | 3.647589308 | -3.704716391 | 0.000237867 |
| TCF12 | -0.138028349 | 5.804366589 | -3.704231372 | 0.000238309 |
| ZNF512B | 0.216464035 | 5.269593714 | 3.704054223 | 0.000238471 |
| TXNL1 | 0.132239027 | 5.175548828 | 3.703871995 | 0.000238637 |
| MMGT1 | 0.147304385 | 6.518280671 | 3.70377393 | 0.000238727 |
| HNRNPA1P2 | 0.355054113 | 1.38811373 | 3.703573031 | 0.000238911 |
| AC079354.2 | -0.211269199 | 5.025176919 | -3.70339384 | 0.000239075 |
| CCL5 | 0.500129416 | 7.052560588 | 3.703295619 | 0.000239164 |
| CTD-2647L4.4 | -0.223997018 | 4.421707564 | -3.703284349 | 0.000239175 |
| CHRNA6 | -0.772104254 | -1.836440763 | -3.70295835 | 0.000239473 |
| CTB-193M12.3 | 0.422818303 | 0.591199295 | 3.702673985 | 0.000239734 |
| PPARGC1A | -1.097698127 | -2.726966792 | -3.702673896 | 0.000239734 |
| RP1-309F20.3 | -0.349385149 | 0.718895046 | -3.702526694 | 0.000239869 |
| RP11-524O1.4 | -0.430943747 | -0.684553882 | -3.702011865 | 0.000240342 |
| CTD-2531D15.4 | -0.611442429 | -0.253014376 | -3.70177025 | 0.000240564 |
| CA13 | -0.37286831 | 0.855693622 | -3.701763297 | 0.000240571 |
| HIST2H2AC | -0.462285017 | 4.637766282 | -3.701342697 | 0.000240958 |
| SNHG12 | -0.218283335 | 4.694816524 | -3.701086964 | 0.000241194 |
| RP11-849F2.7 | -0.468777455 | -1.767049222 | -3.700988947 | 0.000241284 |
| ZCCHC12 | 0.692692926 | -1.510266214 | 3.699504301 | 0.000242658 |
| CNOT2 | 0.120553152 | 5.472766972 | 3.699324953 | 0.000242824 |
| CEBPZ | 0.12268935 | 6.368510052 | 3.699208743 | 0.000242932 |
| UBE2N | 0.110352142 | 6.380834691 | 3.698816708 | 0.000243297 |
| UBXN10 | 0.71218257 | 0.242800962 | 3.698618375 | 0.000243481 |
| INA | -0.561177503 | -2.650133152 | -3.698041673 | 0.000244018 |
| SPR | -0.533937919 | 1.434223217 | -3.697777674 | 0.000244265 |
| PPP6R1 | -0.145671946 | 7.810907597 | -3.697477227 | 0.000244545 |
| CHEK2 | -0.265740559 | 3.807403063 | -3.697371022 | 0.000244645 |
| TRMT11 | 0.189092108 | 3.819274439 | 3.697161045 | 0.000244841 |
| CCDC47 | -0.162097718 | 6.26102979 | -3.69690318 | 0.000245082 |
| PCDH17 | 0.558990978 | -4.851885673 | 3.696820912 | 0.000245159 |
| MRPL45P2 | 0.248550252 | 3.464996712 | 3.696809676 | 0.00024517 |
| TERF2 | -0.145698613 | 5.244896469 | -3.696311921 | 0.000245637 |
| CDH1 | 0.876914555 | -0.360931706 | 3.696162054 | 0.000245777 |
| RANBP20P | 0.341485932 | 0.618679525 | 3.695942752 | 0.000245983 |
| GYG2 | -0.739115977 | 0.077064748 | -3.695608911 | 0.000246297 |
| RP11-504P24.3 | -0.408214591 | -0.150651121 | -3.695568409 | 0.000246335 |
| TFDP1 | 0.265798474 | 6.677670905 | 3.695330412 | 0.000246559 |
| MIR3180-1 | 0.433974269 | -0.972171821 | 3.695103076 | 0.000246774 |
| GALNT12 | 0.416800181 | 2.971255393 | 3.694116338 | 0.000247705 |
| QSER1 | -0.179234369 | 4.546227357 | -3.693932974 | 0.000247879 |
| TNFRSF11B | -0.55471982 | -2.792224308 | -3.6938767 | 0.000247932 |
| CDK5R1 | 0.446897261 | 1.522211266 | 3.693430047 | 0.000248355 |
| BATF2 | -0.598228957 | 0.397701995 | -3.69340924 | 0.000248375 |
| TAX1BP3 | -0.215806538 | 2.644952796 | -3.692865516 | 0.000248891 |
| FOXN3-AS1 | -0.324783934 | 1.575894399 | -3.692821589 | 0.000248933 |
| FAM231D | -0.448952846 | 1.819493773 | -3.69227834 | 0.00024945 |
| HOMER2 | 0.520580816 | 1.982527845 | 3.692167469 | 0.000249555 |
| CTHRC1 | 0.699035665 | -1.031938535 | 3.692147119 | 0.000249575 |
| RP11-356N1.2 | -0.561256715 | 1.330947812 | -3.692007622 | 0.000249707 |
| APBA2 | 0.536121197 | 2.18079655 | 3.691996323 | 0.000249718 |
| BIN2P1 | -0.30352465 | 2.620382493 | -3.691806139 | 0.0002499 |
| PEX14 | 0.132816152 | 4.548436259 | 3.691461882 | 0.000250228 |
| RNF181 | -0.121625028 | 6.984818639 | -3.691101392 | 0.000250573 |
| SYT11 | -0.268434861 | 4.867211884 | -3.690835014 | 0.000250827 |
| GAPDHP44 | 0.256342757 | 4.921926745 | 3.690559644 | 0.000251091 |
| MSL3 | -0.173462272 | 5.515580899 | -3.690548359 | 0.000251102 |
| TMEM14A | -0.389744804 | 3.772308442 | -3.690543588 | 0.000251106 |
| PIP5KL1 | 0.398642072 | -0.283283315 | 3.690234706 | 0.000251403 |
| FGFR1OP2 | -0.183910257 | 6.139876799 | -3.690161974 | 0.000251472 |
| ZW10 | -0.18245255 | 4.658767846 | -3.689922145 | 0.000251703 |
| AC106900.6 | 0.424963406 | 0.146238003 | 3.689026949 | 0.000252564 |
| RASA3 | 0.267555868 | 6.593983934 | 3.688657515 | 0.00025292 |
| DEPTOR | -0.519880345 | 5.199332118 | -3.688453681 | 0.000253116 |
| PAQR6 | 0.358299979 | 4.253740338 | 3.688357988 | 0.000253209 |
| DRD4 | 0.418085311 | 1.813431206 | 3.688215234 | 0.000253347 |
| TMEM245 | -0.174784684 | 4.977965307 | -3.688083445 | 0.000253474 |
| RP11-165N19.2 | 0.377877162 | 0.855844003 | 3.686933741 | 0.000254588 |
| RHBDL3 | 0.830106788 | -1.231214685 | 3.686899488 | 0.000254621 |
| RAP2C-AS1 | 0.310672342 | 2.17126252 | 3.686686989 | 0.000254827 |
| HNRNPH3 | 0.143553124 | 7.954768107 | 3.686371752 | 0.000255134 |
| LRRC36 | -0.529075576 | -1.674353591 | -3.685992988 | 0.000255502 |
| FRZB | 0.547052806 | -3.105252129 | 3.684674957 | 0.000256789 |
| UBE3C | -0.152168132 | 5.574274605 | -3.684232223 | 0.000257222 |
| HSPA1A | -0.51020055 | 6.625781303 | -3.683852819 | 0.000257595 |
| HDAC9 | -0.612636251 | 2.633598071 | -3.683534877 | 0.000257907 |
| CCL22 | 0.75913853 | -1.942606914 | 3.682795961 | 0.000258634 |
| XXbac-BPGBPG55C20.1 | -0.434939358 | -0.650954916 | -3.681996127 | 0.000259423 |
| KCNQ5-IT1 | 0.540783802 | 0.448787592 | 3.681495376 | 0.000259918 |
| ODF2 | -0.18122119 | 5.865736506 | -3.681365356 | 0.000260047 |
| ABCA3 | -0.471155816 | 2.014990133 | -3.681247373 | 0.000260163 |
| NEXN-AS1 | 0.491506519 | -2.222605865 | 3.679814475 | 0.000261587 |
| KRT18P4 | -0.370758105 | 0.47978538 | -3.679554882 | 0.000261845 |
| TRGV2 | -0.528641466 | -0.126901719 | -3.679347502 | 0.000262052 |
| TCF4 | 0.54468689 | 4.268295015 | 3.679278152 | 0.000262121 |
| ZNF461 | 0.264634621 | 2.794801015 | 3.678873907 | 0.000262525 |
| VWA7 | 0.459207627 | 1.819613267 | 3.678783833 | 0.000262615 |
| TRGJP2 | -0.734254895 | 4.218821487 | -3.678161894 | 0.000263237 |
| AC016629.8 | -0.336055233 | 1.250718524 | -3.678031736 | 0.000263367 |
| HERC2P8 | -0.489193449 | -2.906740721 | -3.677331582 | 0.00026407 |
| MIP | -0.486257879 | -2.853059723 | -3.676957525 | 0.000264446 |
| LENG1 | -0.196836938 | 4.605701917 | -3.676436 | 0.000264971 |
| ZNF330 | 0.125199688 | 6.079762552 | 3.675654827 | 0.000265759 |
| ATP6V1C1 | -0.22699259 | 5.076865769 | -3.675005349 | 0.000266416 |
| MRPL12 | -0.263731063 | 3.313235837 | -3.673937504 | 0.0002675 |
| RP11-167N4.2 | 0.377478032 | 3.161279226 | 3.673683365 | 0.000267759 |
| ZFYVE21 | -0.229311865 | 2.730646628 | -3.673116802 | 0.000268336 |
| AC007292.6 | -0.343782713 | 1.384142691 | -3.673034547 | 0.00026842 |
| NME4 | 0.27115744 | 6.258093075 | 3.672612502 | 0.000268851 |
| RGS10 | 0.489420664 | 6.683064508 | 3.672462219 | 0.000269004 |
| DBNL | -0.166060082 | 6.37684612 | -3.672242136 | 0.000269229 |
| FXN | 0.206733797 | 4.689018616 | 3.671702715 | 0.000269782 |
| CCZ1 | 0.160767208 | 4.002439255 | 3.670099711 | 0.000271429 |
| FAM103A2P | 0.195788629 | 5.543779884 | 3.668831739 | 0.000272739 |
| MRPS28 | -0.189689128 | 3.890161174 | -3.668814414 | 0.000272757 |
| KLHL2 | 0.300303248 | 5.111732388 | 3.668754836 | 0.000272819 |
| PLSCR4 | 0.922411992 | -0.747052262 | 3.668603791 | 0.000272976 |
| PI4KB | 0.075964599 | 6.577583355 | 3.668511031 | 0.000273072 |
| FAM150B | 0.499034137 | -2.282027892 | 3.668455883 | 0.000273129 |
| CTC-471J1.2 | -0.338561082 | 1.45457361 | -3.667892106 | 0.000273714 |
| MRPL36 | -0.174216435 | 4.987990426 | -3.667729337 | 0.000273883 |
| MRPS27 | -0.193627999 | 5.658285292 | -3.665849218 | 0.000275845 |
| RP11-701P16.5 | 0.561955058 | 0.417554847 | 3.665699729 | 0.000276001 |
| LDLRAP1 | -0.288379048 | 4.586205255 | -3.66540331 | 0.000276312 |
| SDHA | 0.142931039 | 5.772616065 | 3.664709689 | 0.00027704 |
| ZNF106 | -0.170384082 | 6.361844139 | -3.664205746 | 0.00027757 |
| RNU1-103P | 0.319727177 | 1.117387551 | 3.663159757 | 0.000278673 |
| C17orf96 | 0.529619324 | 1.240999102 | 3.662559093 | 0.000279308 |
| ALG1L2 | -0.460772333 | 0.787489917 | -3.661998649 | 0.000279903 |
| SNX16 | 0.185154406 | 3.424198604 | 3.661669085 | 0.000280252 |
| GPR3 | 0.569215179 | 1.396037405 | 3.661659474 | 0.000280263 |
| TRGV5P | -0.629950949 | 2.368237809 | -3.661443875 | 0.000280492 |
| NHLRC1 | -0.481948166 | 1.089785929 | -3.660777607 | 0.000281201 |
| DCTPP1 | -0.24288324 | 5.779247798 | -3.659377044 | 0.000282697 |
| ELFN1 | 0.55003194 | -2.487773228 | 3.65923154 | 0.000282853 |
| WNT5A | 0.556693836 | -4.336696191 | 3.658704524 | 0.000283419 |
| RP11-443J23.1 | -0.377661307 | -1.663683834 | -3.658580662 | 0.000283552 |
| UBAC2-AS1 | -0.304194791 | 1.283959071 | -3.658449026 | 0.000283693 |
| CA4 | 0.916473681 | 1.443565486 | 3.65808378 | 0.000284086 |
| AP000974.1 | -0.441037328 | -2.005926048 | -3.657432275 | 0.000284788 |
| ACTN3 | 0.412970725 | -2.22989138 | 3.657232446 | 0.000285003 |
| PDCL3P5 | -0.1898605 | 3.994795739 | -3.656572139 | 0.000285717 |
| FUT10 | -0.328697887 | 2.542502437 | -3.655747052 | 0.000286611 |
| LUC7L | -0.169163617 | 5.634281164 | -3.655499579 | 0.000286879 |
| RP11-147L13.8 | -0.451301445 | 0.120269562 | -3.65529948 | 0.000287097 |
| TRAC | -0.542392969 | 5.843356134 | -3.654548597 | 0.000287914 |
| ATP5G2P4 | -0.172870119 | 4.960727807 | -3.654221972 | 0.00028827 |
| ASAH2B | 0.417548995 | -2.715933078 | 3.653396111 | 0.000289172 |
| EFCAB12 | 0.423642565 | -0.219649536 | 3.653353726 | 0.000289219 |
| LGALS4 | 0.357385687 | 0.761401102 | 3.653344164 | 0.000289229 |
| RAB11B-AS1 | 0.190663528 | 2.795943302 | 3.653213194 | 0.000289372 |
| SORBS1 | 0.502142565 | 0.619712327 | 3.652645741 | 0.000289994 |
| AC090286.2 | -0.329094925 | -0.012268453 | -3.652011299 | 0.000290691 |
| AC011475.1 | 0.421265474 | 0.570121968 | 3.651643126 | 0.000291096 |
| PRICKLE4 | -0.34124489 | 3.049021975 | -3.650078839 | 0.000292823 |
| FAM72A | 0.428476085 | 1.562060648 | 3.649731621 | 0.000293208 |
| APC2 | 0.490219561 | -2.150723691 | 3.649375547 | 0.000293603 |
| AC139100.2 | -0.443951554 | -0.560239875 | -3.649357876 | 0.000293622 |
| NPIPA8 | 0.245441118 | 1.481724014 | 3.648205955 | 0.000294903 |
| TAOK3 | -0.155317302 | 6.48450642 | -3.64794033 | 0.0002952 |
| WDR18 | -0.197756313 | 5.558934282 | -3.647910393 | 0.000295233 |
| RP11-923I11.4 | -0.424854312 | -1.23162107 | -3.647908758 | 0.000295235 |
| GPR85 | -0.930635049 | -1.813483347 | -3.647313302 | 0.0002959 |
| TRAV26-1 | -0.518892317 | -0.259304404 | -3.647061454 | 0.000296182 |
| RP11-452K12.4 | -0.322998302 | 0.824370741 | -3.645998448 | 0.000297373 |
| TSPAN15 | 0.596849891 | -0.050182167 | 3.645840049 | 0.000297551 |
| CRLF2 | 0.799980733 | 1.885604385 | 3.645479062 | 0.000297957 |
| ZNF395 | 0.360640661 | 5.604946602 | 3.645057924 | 0.000298432 |
| FAM32A | -0.11609751 | 7.600288195 | -3.644829238 | 0.000298689 |
| TCF19 | 0.327360747 | 5.422060533 | 3.643897804 | 0.000299742 |
| IPO9 | -0.136233072 | 5.467455222 | -3.643636891 | 0.000300037 |
| AC013449.1 | -0.213197244 | 5.543094144 | -3.643299955 | 0.000300419 |
| MFNG | -0.216876398 | 7.100076633 | -3.64291292 | 0.000300858 |
| ABCA10 | -0.417119964 | -1.500962408 | -3.642638375 | 0.00030117 |
| SMIM19 | 0.148795041 | 4.680356756 | 3.642094134 | 0.000301789 |
| AC009133.17 | -0.364211733 | 1.526583831 | -3.641537083 | 0.000302425 |
| MIR27A | 0.537089509 | 2.502261939 | 3.641414817 | 0.000302564 |
| EIF2B2 | -0.171970693 | 5.050401654 | -3.641040013 | 0.000302992 |
| CTD-2541M15.4 | -0.228449997 | 2.313534808 | -3.640900126 | 0.000303152 |
| RP11-430G17.3 | -0.475947301 | -1.008147989 | -3.640841607 | 0.000303219 |
| CTBP2P7 | 0.484571242 | -1.131612531 | 3.640731041 | 0.000303346 |
| STS | -0.412511681 | 3.367832561 | -3.638589466 | 0.000305806 |
| SENP6 | -0.137382616 | 6.216640305 | -3.638360592 | 0.00030607 |
| SURF6 | -0.158168571 | 5.780313819 | -3.637975289 | 0.000306515 |
| AC008440.10 | 0.530976102 | -1.022016538 | 3.637514148 | 0.000307048 |
| TMEM50B | -0.150969259 | 5.277266048 | -3.637413948 | 0.000307164 |
| SGIP1 | 0.559195764 | -2.284810239 | 3.63734086 | 0.000307249 |
| DNAAF1 | 0.61082814 | -2.274904695 | 3.636249793 | 0.000308515 |
| RP4-673M15.1 | 0.412900304 | 1.420184861 | 3.636142369 | 0.00030864 |
| METTL21A | -0.20547094 | 3.952825809 | -3.635976105 | 0.000308833 |
| C7 | 0.751219938 | -2.791472015 | 3.635499287 | 0.000309389 |
| RP11-706O15.1 | -0.279721883 | 4.253605488 | -3.635478072 | 0.000309414 |
| RP11-215G15.5 | 0.494493066 | 3.873377054 | 3.63511214 | 0.000309841 |
| WDR34 | 0.314619819 | 5.442121794 | 3.635099299 | 0.000309856 |
| CSPG5 | 0.409549252 | -2.153265229 | 3.635074181 | 0.000309885 |
| KCTD19 | -0.578491808 | -3.363698055 | -3.634543207 | 0.000310505 |
| TMA7 | 0.165310801 | 6.648797035 | 3.634145658 | 0.000310971 |
| AMOTL2 | 0.692551561 | -2.428981132 | 3.633331401 | 0.000311926 |
| SLC30A9 | -0.130049249 | 5.646792591 | -3.632266473 | 0.00031318 |
| U40455.1 | -0.278810473 | 0.574826363 | -3.632254519 | 0.000313194 |
| PHOSPHO2 | -0.292687458 | 2.376232879 | -3.631586245 | 0.000313983 |
| RP11-349G13.1 | 0.31477012 | 1.096469255 | 3.630802522 | 0.00031491 |
| DVL3 | 0.126264884 | 6.339331902 | 3.630763729 | 0.000314956 |
| NUP160 | -0.178857092 | 5.137918228 | -3.630708593 | 0.000315022 |
| WDFY2 | -0.198750209 | 6.131201044 | -3.630641425 | 0.000315101 |
| FAAH2 | 0.450683628 | 0.340733341 | 3.630403779 | 0.000315383 |
| COPS6 | -0.141011805 | 6.471650206 | -3.630271561 | 0.00031554 |
| C10orf55 | 0.454909875 | 0.496937383 | 3.63003285 | 0.000315824 |
| TMCO4 | -0.188958916 | 4.278771113 | -3.629940903 | 0.000315933 |
| PGM2L1 | 0.24512191 | 3.305900972 | 3.628986204 | 0.00031707 |
| DCPS | -0.201746778 | 4.769138064 | -3.628738468 | 0.000317366 |
| RP6-105D16.1 | 0.255446754 | 4.731146906 | 3.628582175 | 0.000317553 |
| RABL6 | 0.118503056 | 5.350671254 | 3.628229741 | 0.000317974 |
| TIMM23 | 0.17222376 | 5.276815835 | 3.628073336 | 0.000318161 |
| ITGB5 | 0.647320624 | 1.609057901 | 3.627961433 | 0.000318295 |
| RP11-115N4.1 | 0.537170298 | -1.594440476 | 3.62727653 | 0.000319116 |
| HSP90B2P | 0.229124122 | 4.211223759 | 3.627129859 | 0.000319292 |
| RP5-854E16.2 | -0.178082028 | 3.344480858 | -3.62587157 | 0.000320807 |
| AL392086.1 | -0.606640435 | -1.335474964 | -3.625849888 | 0.000320833 |
| ZC3H11A | -0.089278866 | 6.658540859 | -3.625707188 | 0.000321005 |
| HIST1H2BF | -0.575460995 | 3.199322078 | -3.625641404 | 0.000321084 |
| 6-Mar | 0.166040836 | 6.505490513 | 3.625455263 | 0.000321309 |
| PTPRG | -0.94234581 | -2.865605522 | -3.625396413 | 0.00032138 |
| TRIM16 | -0.345606949 | 2.091844588 | -3.625334916 | 0.000321455 |
| TGFB1I1 | 0.579486271 | 0.909637704 | 3.624643184 | 0.000322292 |
| TMEM106B | -0.198673907 | 4.254473232 | -3.624229541 | 0.000322793 |
| XXbac-BPG252P9.9 | 0.381183363 | 1.606903886 | 3.623995785 | 0.000323077 |
| ERBB2 | -0.294420143 | 0.874291615 | -3.623971244 | 0.000323107 |
| PSD | 0.353336237 | -0.685069842 | 3.623606785 | 0.000323549 |
| IL12A-AS1 | -1.124232117 | 0.902926611 | -3.623497161 | 0.000323683 |
| RP11-715J22.6 | -0.393161903 | -0.463423041 | -3.62152379 | 0.000326091 |
| RRAGD | 0.329836379 | 5.234375195 | 3.621309005 | 0.000326355 |
| RNPEP | -0.209796933 | 6.886492905 | -3.620765092 | 0.000327022 |
| SF3A1 | 0.100778231 | 7.472053275 | 3.620474397 | 0.000327379 |
| RP11-64J19.1 | 0.417552524 | 0.730834245 | 3.620226321 | 0.000327684 |
| RN7SL138P | -0.627628181 | 1.142445427 | -3.620183228 | 0.000327737 |
| MBOAT2 | 0.566021513 | 3.854683344 | 3.620172242 | 0.000327751 |
| CTD-3088G3.4 | -0.589435497 | 0.121384285 | -3.619406789 | 0.000328694 |
| COL4A2 | 0.78091113 | -2.817685354 | 3.619187133 | 0.000328965 |
| PCIF1 | 0.137288596 | 6.629230196 | 3.618863873 | 0.000329365 |
| SEC61A1 | -0.124490108 | 8.039202781 | -3.618844832 | 0.000329388 |
| RP11-420L9.5 | 0.501195143 | -2.099107956 | 3.618825353 | 0.000329412 |
| EIF5AP4 | 0.201052228 | 3.737952842 | 3.618667827 | 0.000329607 |
| SCD | 0.528650218 | 4.302119147 | 3.618522271 | 0.000329787 |
| RERE | 0.175899182 | 6.013150591 | 3.618159833 | 0.000330236 |
| EIF4BP8 | 0.293776547 | 1.088425245 | 3.617955052 | 0.00033049 |
| TSGA10IP | -0.48648932 | -1.679021292 | -3.61678724 | 0.000331942 |
| OR7M1P | 0.452773085 | -0.714544501 | 3.616729707 | 0.000332013 |
| CASC4 | -0.136916059 | 6.244787516 | -3.616155479 | 0.00033273 |
| DENND5B | 0.45512071 | 2.147140523 | 3.616056258 | 0.000332853 |
| SNORD94 | 0.304763323 | 2.147629726 | 3.615239278 | 0.000333875 |
| HUWE1 | -0.178082765 | 7.323625086 | -3.615190811 | 0.000333936 |
| AC018755.16 | -0.470776185 | -0.706297155 | -3.614460685 | 0.000334851 |
| KNTC1 | 0.238244929 | 4.807201325 | 3.61322201 | 0.00033641 |
| MRPL33 | -0.292633771 | 6.577002305 | -3.61304151 | 0.000336638 |
| IQCK | -0.439249883 | 0.909142557 | -3.611580924 | 0.000338486 |
| ZNF540 | -0.425148831 | -0.125082899 | -3.611483919 | 0.000338609 |
| RP11-216L13.19 | 0.295081065 | 1.388409213 | 3.610751445 | 0.00033954 |
| RAB7L1 | -0.324456465 | 5.111379098 | -3.609901276 | 0.000340623 |
| ZNF141 | -0.241806331 | 4.056247112 | -3.60971744 | 0.000340858 |
| PLCG1 | 0.455676783 | 4.049377761 | 3.609614688 | 0.000340989 |
| PTMA | 0.150661537 | 10.72452391 | 3.609324907 | 0.000341359 |
| STAB2 | 0.721056981 | -3.696792006 | 3.609013783 | 0.000341757 |
| ATXN7L2 | 0.213250498 | 3.161455368 | 3.608601847 | 0.000342285 |
| AC020571.3 | -0.640986257 | 0.475278434 | -3.60753525 | 0.000343655 |
| RPL7P21 | -0.287391612 | 3.036308654 | -3.607263566 | 0.000344004 |
| RP11-592B15.4 | -0.379972807 | 0.121707218 | -3.606579243 | 0.000344887 |
| IFIT2 | 0.568807009 | 5.162611986 | 3.606391369 | 0.00034513 |
| RP11-209D14.4 | -0.399141414 | 0.621189823 | -3.606380456 | 0.000345144 |
| SLC2A4RG | 0.195414869 | 6.750025889 | 3.605990115 | 0.000345648 |
| MS4A7 | -0.598877269 | 4.829431439 | -3.605554894 | 0.000346212 |
| LINC00565 | 0.566401452 | -0.166302056 | 3.605156107 | 0.000346729 |
| ANKRD34A | -0.340862923 | 0.031673092 | -3.604874743 | 0.000347094 |
| MGAT4B | 0.179008832 | 6.401128985 | 3.604668637 | 0.000347362 |
| OCM | -0.400023593 | 0.519040512 | -3.604067863 | 0.000348143 |
| SLC22A3 | 0.594800696 | -3.84399619 | 3.602740399 | 0.000349877 |
| ZNF567 | 0.208890391 | 2.759118363 | 3.602552463 | 0.000350123 |
| AC018766.6 | -0.357007178 | 0.551322521 | -3.601941603 | 0.000350923 |
| RNU4ATAC | -0.514844428 | 2.619107867 | -3.601406536 | 0.000351626 |
| MSH5 | 0.267872221 | 2.49432765 | 3.601358752 | 0.000351689 |
| FAM178B | 0.97100766 | -0.862723694 | 3.601076811 | 0.00035206 |
| DLGAP3 | 0.566690858 | 0.714290385 | 3.600111427 | 0.000353333 |
| IGKJ3 | 0.554259263 | 0.499312233 | 3.599697579 | 0.00035388 |
| DHX40 | 0.117834346 | 5.341613534 | 3.599549894 | 0.000354075 |
| CCDC101 | -0.155367181 | 5.168778249 | -3.599067203 | 0.000354714 |
| SMIM15 | -0.223398546 | 5.786350105 | -3.598966723 | 0.000354847 |
| SNORD83A | -0.278284908 | 2.375979031 | -3.598358537 | 0.000355655 |
| IFIT1 | 0.650256424 | 3.228449059 | 3.598234179 | 0.00035582 |
| RPS15A | 0.214094467 | 5.133523826 | 3.598191405 | 0.000355877 |
| SLC38A5 | 0.335776157 | 4.610388178 | 3.598177894 | 0.000355895 |
| RP11-73M18.8 | -0.191032099 | 5.74634515 | -3.59805218 | 0.000356062 |
| HCST | -0.26701758 | 8.496083167 | -3.597323377 | 0.000357033 |
| LGALS1 | -0.476297576 | 9.147552828 | -3.597050349 | 0.000357397 |
| RP11-284M14.1 | -0.312678098 | 1.606914232 | -3.597021909 | 0.000357435 |
| UHRF1BP1L | 0.222816566 | 5.867670254 | 3.597010468 | 0.00035745 |
| IGKJ4 | 0.601734181 | 0.871045254 | 3.596951633 | 0.000357529 |
| RP11-876N24.1 | -0.465268433 | -0.822413686 | -3.595656373 | 0.000359262 |
| RP4-647J21.1 | 0.296947616 | 5.98377561 | 3.594900864 | 0.000360277 |
| GNL3L | -0.137464187 | 5.33364453 | -3.594839815 | 0.000360359 |
| IL1R2 | 0.963477499 | 2.760729994 | 3.594622711 | 0.000360652 |
| RPL7P9 | -0.130811675 | 11.13930912 | -3.594527199 | 0.00036078 |
| NUDT15 | 0.24524856 | 4.907671403 | 3.593484991 | 0.000362186 |
| RP11-488L18.10 | -0.195179479 | 6.824340027 | -3.592919613 | 0.000362951 |
| R3HCC1 | 0.118057645 | 5.691332115 | 3.592017709 | 0.000364175 |
| C21orf91 | -0.320910309 | 5.086213854 | -3.59184451 | 0.00036441 |
| COL25A1 | -0.572817913 | -2.805292658 | -3.591655103 | 0.000364668 |
| CTC-338M12.5 | -0.280080549 | 2.044621109 | -3.591607066 | 0.000364733 |
| RP11-454L1.2 | 0.42163979 | -0.113351044 | 3.591512748 | 0.000364861 |
| CTD-2616J11.2 | 0.395126613 | 1.146135569 | 3.59149163 | 0.00036489 |
| CAPZB | -0.145234616 | 8.359175605 | -3.590971324 | 0.000365599 |
| RP4-798A10.2 | 0.338813988 | 2.051455898 | 3.590183659 | 0.000366675 |
| DNAH12 | -0.429558347 | -2.293346134 | -3.589789613 | 0.000367214 |
| LRRTM2 | 0.485020572 | -2.109916413 | 3.589763156 | 0.00036725 |
| VMO1 | 0.535925726 | 0.582180615 | 3.589554087 | 0.000367536 |
| ADAM8 | 0.460772237 | 6.939149978 | 3.589358637 | 0.000367804 |
| CYB561A3 | -0.167117195 | 4.622213174 | -3.588827713 | 0.000368533 |
| ABCD2 | -0.576288282 | -0.076638293 | -3.588168787 | 0.00036944 |
| TMEM242 | 0.159697888 | 4.302158764 | 3.588142036 | 0.000369476 |
| PVR | -0.47553703 | 4.160478661 | -3.587988566 | 0.000369688 |
| C1orf177 | -0.453646954 | -1.770251704 | -3.587802997 | 0.000369944 |
| TDP2 | 0.138419209 | 6.015866048 | 3.587021598 | 0.000371022 |
| ZNF250 | -0.195377711 | 1.763595749 | -3.586672302 | 0.000371506 |
| HBEGF | 0.681357201 | 5.150680497 | 3.5860666 | 0.000372345 |
| DGKZ | -0.142972383 | 5.747369085 | -3.586031023 | 0.000372394 |
| HMGN2P21 | 0.383317158 | -0.093921868 | 3.58576599 | 0.000372762 |
| RP11-861A13.4 | 0.530242578 | 4.356447258 | 3.585741259 | 0.000372797 |
| RP11-304L19.3 | -0.400152117 | 0.378175529 | -3.585461411 | 0.000373186 |
| ZFR2 | 0.473321264 | -3.520505897 | 3.585456411 | 0.000373192 |
| CENPF | 0.465947006 | 4.391956082 | 3.585270843 | 0.000373451 |
| RP1-20C7.6 | -0.281154745 | 1.920133098 | -3.585224002 | 0.000373516 |
| HNRNPLL | 0.545861922 | 3.785473711 | 3.583606604 | 0.000375772 |
| CD300A | -0.379973392 | 6.356671527 | -3.58297013 | 0.000376664 |
| RP3-508I15.22 | 0.402766901 | 0.154436521 | 3.582863306 | 0.000376814 |
| PANX2 | 0.550907215 | 3.772308959 | 3.582549085 | 0.000377255 |
| RP11-613H2.2 | 0.331499615 | 1.521377162 | 3.582370273 | 0.000377506 |
| RP11-17M16.2 | -0.368852196 | 0.70425159 | -3.582132883 | 0.00037784 |
| OR2L6P | -0.449009716 | -1.559610232 | -3.581915886 | 0.000378145 |
| AC067945.3 | 0.38358508 | -0.250357787 | 3.581896495 | 0.000378172 |
| PSMG4 | -0.196243213 | 2.432052728 | -3.581066875 | 0.000379342 |
| EMP1 | 0.640312491 | 5.39876603 | 3.580731973 | 0.000379815 |
| DCAF10 | -0.117717204 | 4.536656773 | -3.580571756 | 0.000380041 |
| TXNRD2 | -0.155866264 | 3.462684032 | -3.580408228 | 0.000380273 |
| AC010761.8 | 0.480214759 | 0.550665338 | 3.579986353 | 0.00038087 |
| IDH3G | 0.095322027 | 6.773610543 | 3.579756878 | 0.000381195 |
| ISCA2 | -0.141915523 | 4.36749087 | -3.579331794 | 0.000381799 |
| BAG1 | 0.152154895 | 5.717545215 | 3.578785377 | 0.000382575 |
| RP11-408P14.1 | -0.155592091 | 9.76861836 | -3.578345048 | 0.000383202 |
| FAM120C | 0.17665283 | 2.593837542 | 3.577681218 | 0.00038415 |
| RN7SL809P | 0.305516419 | 1.934314879 | 3.577359207 | 0.00038461 |
| SLC35A2 | 0.196013551 | 4.429649867 | 3.577346662 | 0.000384628 |
| CSPP1 | -0.165442582 | 4.201065029 | -3.577340536 | 0.000384636 |
| ZC3H14 | -0.115070412 | 5.232933749 | -3.577043101 | 0.000385062 |
| HSD17B6 | -0.397523583 | -0.430542656 | -3.576144436 | 0.000386351 |
| COBLL1 | 0.576221345 | 1.994644263 | 3.576064661 | 0.000386465 |
| METAP1D | -0.29435264 | 3.240789027 | -3.576027474 | 0.000386519 |
| RNF34 | -0.14338141 | 4.907238119 | -3.575938364 | 0.000386647 |
| RPS29 | 0.228018372 | 7.838656836 | 3.575821678 | 0.000386814 |
| ZBED10P | 0.323915599 | 4.501421791 | 3.575793098 | 0.000386856 |
| FCER1A | -0.793479611 | 5.451530593 | -3.575682429 | 0.000387015 |
| AC011330.5 | -0.338901332 | 1.634496118 | -3.574202648 | 0.000389149 |
| RP11-305L7.3 | -0.485074 | -0.405650793 | -3.574139849 | 0.00038924 |
| BACH2 | 0.468470897 | 1.928190933 | 3.574085819 | 0.000389318 |
| ERGIC1 | -0.215459317 | 6.404217304 | -3.573734289 | 0.000389826 |
| DDHD2 | 0.195212258 | 4.89611659 | 3.573244285 | 0.000390537 |
| BFSP1 | 0.453840578 | -0.618035945 | 3.573188542 | 0.000390618 |
| HIGD1A | 0.209969539 | 6.54085805 | 3.573136349 | 0.000390693 |
| RP11-434D2.6 | 0.232086484 | 2.139642412 | 3.572715868 | 0.000391304 |
| FUCA1 | -0.346319243 | 5.246302564 | -3.572008482 | 0.000392333 |
| RP11-363N22.2 | 0.434696006 | -0.318277893 | 3.571854904 | 0.000392557 |
| AC068039.4 | -0.417145022 | 0.159494595 | -3.571639947 | 0.000392871 |
| TKTL1 | 0.850965945 | 0.240108121 | 3.571615462 | 0.000392906 |
| ALOX12P2 | -0.91887127 | -0.685936055 | -3.571381564 | 0.000393248 |
| FKBP14 | 0.554374722 | 2.392690738 | 3.571041715 | 0.000393744 |
| CCR7 | 0.607020931 | 3.305633763 | 3.570220081 | 0.000394947 |
| BMS1P20 | -0.33243837 | 0.594959506 | -3.570194698 | 0.000394984 |
| ANKRD36C | 0.267098335 | 3.992527847 | 3.570070909 | 0.000395166 |
| RP11-350J20.4 | 0.387257536 | 0.428345749 | 3.570037268 | 0.000395215 |
| ZCCHC7 | -0.169903276 | 4.864788636 | -3.568844468 | 0.000396969 |
| DNMT3A | -0.242781858 | 5.642438776 | -3.568682704 | 0.000397207 |
| OR2L3 | -0.495382718 | -1.240939806 | -3.568354489 | 0.000397691 |
| CTD-3128G10.6 | -0.432474452 | -0.260019381 | -3.568181113 | 0.000397947 |
| DNAJC24 | -0.158920734 | 2.416751117 | -3.567848675 | 0.000398438 |
| P2RX6 | -0.499493789 | -1.390911648 | -3.567410184 | 0.000399087 |
| MACROD1 | 0.361745444 | 3.614103934 | 3.566825031 | 0.000399954 |
| IQUB | -0.394305578 | -2.603928046 | -3.566787623 | 0.000400009 |
| PRKX | -0.210615985 | 5.861042695 | -3.56669267 | 0.00040015 |
| OR2AK2 | 0.53528202 | -0.668932886 | 3.566304764 | 0.000400726 |
| AC138123.2 | 0.258008602 | 5.27614274 | 3.566104073 | 0.000401025 |
| TUBGCP2 | 0.10621144 | 6.21519316 | 3.565668417 | 0.000401673 |
| MRPL46 | -0.252934759 | 1.358455339 | -3.565647712 | 0.000401704 |
| FCGR2B | -0.707238519 | 1.561019924 | -3.565557516 | 0.000401838 |
| CTD-2184D3.5 | -0.670694307 | -1.154232408 | -3.565505779 | 0.000401915 |
| RBL1 | 0.246467539 | 4.332717047 | 3.56523778 | 0.000402315 |
| MIR3945 | 0.513630099 | 0.125835951 | 3.56522333 | 0.000402337 |
| CTD-2545M3.8 | -0.75546971 | 0.815582081 | -3.564835659 | 0.000402915 |
| IFI30 | 0.721943767 | 2.369624425 | 3.563852313 | 0.000404387 |
| RDX | 0.208296318 | 4.900779471 | 3.562703639 | 0.000406112 |
| C9orf9 | -0.404455172 | 2.659322759 | -3.562528581 | 0.000406375 |
| GBA | -0.241171272 | 6.0414432 | -3.562493685 | 0.000406428 |
| CTC-246B18.8 | -0.40954547 | 1.405630023 | -3.562077721 | 0.000407055 |
| CYB5R3 | 0.128233268 | 6.691541163 | 3.561374181 | 0.000408117 |
| RARG | 0.353479026 | 2.86002834 | 3.560955527 | 0.000408751 |
| MREG | 0.526980511 | 2.276898682 | 3.560854923 | 0.000408903 |
| SRGAP2B | 0.321779093 | 4.513918759 | 3.560673152 | 0.000409178 |
| BX842568.2 | 0.280600777 | 3.816988051 | 3.560114365 | 0.000410026 |
| SLC2A4 | 0.669190457 | -1.427145675 | 3.559969369 | 0.000410246 |
| SPATA12 | -0.478033566 | -2.469212055 | -3.559736312 | 0.0004106 |
| TBCB | -0.115394802 | 6.114327513 | -3.559566943 | 0.000410858 |
| ATXN1 | 0.401598647 | 4.115301336 | 3.559071166 | 0.000411613 |
| GPKOW | -0.139833974 | 5.598282709 | -3.55837751 | 0.000412671 |
| DAPL1 | -0.712688415 | -0.716838414 | -3.558281531 | 0.000412818 |
| RP1-122O8.7 | -0.338671435 | 0.428833362 | -3.557923715 | 0.000413365 |
| AC007879.7 | -0.576587063 | 0.272123307 | -3.557363165 | 0.000414223 |
| RASA3-IT1 | 0.450831306 | -0.435735032 | 3.557057911 | 0.000414692 |
| GDPD1 | -0.360395639 | 2.107685637 | -3.556977048 | 0.000414816 |
| BMPR1B | -0.738350469 | -2.803201676 | -3.556734161 | 0.000415189 |
| FLCN | -0.215697977 | 3.345468258 | -3.556659115 | 0.000415304 |
| LAMTOR3 | 0.160138466 | 5.081808203 | 3.556414939 | 0.00041568 |
| LL21NC02-1C16.2 | -0.618936599 | 1.622474942 | -3.556377462 | 0.000415737 |
| ZNF260 | -0.21067621 | 3.397153767 | -3.556237923 | 0.000415952 |
| TRAV38-2DV8 | -0.489331036 | -0.305570719 | -3.556013937 | 0.000416297 |
| RCOR3 | 0.116541443 | 5.458987399 | 3.555602378 | 0.000416931 |
| RABEP2 | -0.1856971 | 4.76862617 | -3.555595785 | 0.000416941 |
| DDX39B | 0.163329065 | 7.477077264 | 3.555098065 | 0.00041771 |
| FKBP10 | 0.722974567 | -0.55389926 | 3.554843229 | 0.000418104 |
| ANXA2P2 | -0.53362245 | 8.408590338 | -3.554262018 | 0.000419003 |
| RP11-832N8.1 | 0.224993748 | 2.492030988 | 3.552655951 | 0.000421499 |
| TBC1D12 | 0.816545697 | 0.082089253 | 3.552092185 | 0.000422378 |
| FRY | -0.328755062 | 4.944646332 | -3.55192992 | 0.000422632 |
| RP11-120D5.1 | 0.317485803 | -0.423474971 | 3.551849905 | 0.000422757 |
| RP11-301G21.1 | 0.16973697 | 2.695231826 | 3.551823839 | 0.000422797 |
| RNASEH2C | -0.264393031 | 5.576740054 | -3.551755235 | 0.000422905 |
| FNTB | -0.1802463 | 2.811997682 | -3.551034542 | 0.000424033 |
| SLAMF1 | 0.523840731 | 2.050726297 | 3.550938986 | 0.000424182 |
| TOR1AIP1 | 0.163230049 | 5.870151288 | 3.550695479 | 0.000424564 |
| MECR | -0.196707838 | 3.504848872 | -3.550605815 | 0.000424705 |
| MOB3C | -0.195620317 | 3.943109972 | -3.549895237 | 0.000425821 |
| XKRX | -0.41896172 | -2.124565746 | -3.549775522 | 0.00042601 |
| RP11-664D1.1 | -0.499246778 | -0.996190021 | -3.549244575 | 0.000426846 |
| CTD-2201I18.1 | -0.444311475 | -0.670782703 | -3.549027422 | 0.000427189 |
| RP11-582E3.4 | 0.346951655 | 1.314393182 | 3.548977753 | 0.000427267 |
| ZNF568 | -0.277693905 | 1.493732634 | -3.548946548 | 0.000427316 |
| DAZAP1 | 0.119731003 | 6.557499375 | 3.548652486 | 0.000427781 |
| NTRK1 | 0.793533725 | 1.380421546 | 3.548554751 | 0.000427935 |
| TMEM216 | -0.200800636 | 4.563612261 | -3.547872111 | 0.000429015 |
| ZNF501 | -0.448067665 | 1.132741412 | -3.547047554 | 0.000430323 |
| HIST2H4A | -0.504531326 | -0.583808336 | -3.546948745 | 0.00043048 |
| BCL2L12 | 0.171055983 | 4.576282667 | 3.546830584 | 0.000430668 |
| GTF2H4 | -0.346883384 | -0.193960009 | -3.546703355 | 0.000430871 |
| NLRP6 | 0.553088345 | 1.084858613 | 3.545410768 | 0.000432931 |
| RP11-505E24.2 | -0.365032967 | -0.483176757 | -3.545004676 | 0.000433581 |
| TRMU | 0.137182936 | 3.810940418 | 3.544445528 | 0.000434476 |
| RP1-130H16.16 | -0.289426076 | 1.334860142 | -3.544292338 | 0.000434722 |
| TIGD3 | 0.371035688 | 1.636062624 | 3.543996939 | 0.000435196 |
| RP11-121M22.1 | -0.302876148 | -0.084105866 | -3.543360013 | 0.00043622 |
| LTA4H | -0.328012681 | 7.259158959 | -3.543313158 | 0.000436295 |
| SEPHS1P4 | 0.195832407 | 2.050061639 | 3.542949711 | 0.00043688 |
| SPAG6 | 1.052370164 | -0.116451887 | 3.542369095 | 0.000437817 |
| RP11-194N12.2 | 0.440752441 | -0.454755301 | 3.542333874 | 0.000437874 |
| RP11-153M3.1 | -0.229862168 | 4.340903227 | -3.541555322 | 0.000439133 |
| JMJD1C-AS1 | 0.637990831 | 0.464494048 | 3.541141629 | 0.000439803 |
| PRKRIP1 | 0.16914096 | 5.476512727 | 3.541099393 | 0.000439872 |
| RP11-440L14.4 | -0.391718666 | 0.561997103 | -3.540665526 | 0.000440576 |
| TCP1P1 | 0.200472801 | 3.867097215 | 3.540331454 | 0.000441119 |
| TOMM20 | 0.216880319 | 8.170649506 | 3.539468315 | 0.000442524 |
| RP11-1112J20.1 | -0.404710963 | 2.019943684 | -3.538753355 | 0.000443692 |
| ALKBH1 | 0.157608565 | 3.519794961 | 3.538752763 | 0.000443693 |
| COL6A3 | 0.702387689 | -1.445594988 | 3.538019526 | 0.000444893 |
| UBE3D | -0.270633839 | 0.831973137 | -3.537788601 | 0.000445272 |
| AL773604.8 | -0.35485292 | -0.254404262 | -3.53726052 | 0.000446139 |
| STX2 | -0.29063644 | 4.710666401 | -3.536916221 | 0.000446705 |
| SOCS5P4 | -0.303692559 | 0.841369301 | -3.536760751 | 0.000446961 |
| TMEM143 | -0.157563754 | 3.326270455 | -3.53669847 | 0.000447063 |
| EPC2 | 0.119825993 | 5.833258103 | 3.536427732 | 0.000447509 |
| HMGB1P1 | 0.234748966 | 3.58627022 | 3.535854951 | 0.000448454 |
| ZNF143 | -0.177856773 | 4.396704962 | -3.53549053 | 0.000449056 |
| CTB-50L17.16 | -0.373184081 | -0.434316521 | -3.535425728 | 0.000449164 |
| SLC20A2 | -0.14096281 | 4.223465609 | -3.535208219 | 0.000449523 |
| LSM11 | -0.222800103 | 2.436878592 | -3.535047603 | 0.000449789 |
| SEMA4F | -0.386363076 | 0.889330967 | -3.535046715 | 0.000449791 |
| TMOD1 | 0.530488295 | 2.859516524 | 3.534704044 | 0.000450359 |
| BTBD6 | 0.288978282 | 5.264948819 | 3.534594011 | 0.000450541 |
| UBE2V1P1 | 0.411336299 | 1.224290448 | 3.534293112 | 0.00045104 |
| AC010745.4 | -0.667097332 | -0.693560649 | -3.533992128 | 0.00045154 |
| RAD54L | 0.462846916 | 3.189515979 | 3.53396402 | 0.000451587 |
| HMGN2P41 | 0.346973242 | 1.231365172 | 3.533917352 | 0.000451665 |
| LXN | -0.374473896 | 5.45984067 | -3.533775433 | 0.000451901 |
| XPO5 | -0.166479882 | 4.704700559 | -3.533389696 | 0.000452543 |
| SNX18 | 0.334486448 | 5.912983538 | 3.532708725 | 0.000453678 |
| HNRNPRP1 | 0.229928855 | 5.101934652 | 3.532458326 | 0.000454096 |
| AC008443.1 | -0.306616776 | 2.506346881 | -3.531607937 | 0.000455519 |
| RP4-669P10.19 | -0.354840047 | 0.579624693 | -3.531067548 | 0.000456426 |
| TRPC6 | 0.640800313 | -3.672102128 | 3.530880506 | 0.00045674 |
| COX17 | 0.134919884 | 4.929640005 | 3.530567203 | 0.000457266 |
| BEX1 | 1.081518161 | 4.149995638 | 3.530404802 | 0.00045754 |
| RP11-235E17.6 | -0.313638596 | 1.608464661 | -3.530099246 | 0.000458054 |
| TVP23CP1 | 0.543966065 | -0.233383446 | 3.529740849 | 0.000458658 |
| TSGA10 | -0.363992902 | 2.150206366 | -3.529507881 | 0.000459051 |
| FAM3A | 0.131920692 | 4.551313599 | 3.529476448 | 0.000459104 |
| PTPN6 | -0.231127949 | 8.697825738 | -3.528224781 | 0.000461222 |
| PLAG1 | 0.663407529 | 0.817952495 | 3.528135649 | 0.000461373 |
| MAP9 | 0.497798869 | 0.169223579 | 3.527112975 | 0.00046311 |
| RP11-589C21.6 | 0.568006494 | 1.559272222 | 3.52684364 | 0.000463569 |
| PLIN5 | 0.614866874 | 0.456497116 | 3.526788388 | 0.000463663 |
| AC125238.2 | 0.277005001 | 1.609625636 | 3.526744179 | 0.000463738 |
| NAA15 | 0.143505901 | 5.063905789 | 3.52664076 | 0.000463915 |
| RP11-464F9.20 | -0.319218285 | 1.954609397 | -3.52606166 | 0.000464903 |
| RP11-159F24.3 | -0.334962651 | -0.035073594 | -3.525015251 | 0.000466693 |
| MIR4482-1 | -0.365372289 | 0.156620577 | -3.524758505 | 0.000467134 |
| TPGS2 | 0.184832661 | 5.454189795 | 3.524160418 | 0.000468161 |
| RP11-731F5.2 | -0.524176711 | -1.235155578 | -3.523175786 | 0.000469856 |
| SLC9A3 | 0.599429702 | -2.054753629 | 3.521978968 | 0.000471925 |
| MIR4648 | 0.522666708 | 0.222932996 | 3.521655663 | 0.000472486 |
| KIAA1217 | 0.937358847 | -2.22773671 | 3.521538262 | 0.000472689 |
| NIP7 | 0.146760131 | 4.955874672 | 3.521274757 | 0.000473147 |
| INPPL1 | -0.213269692 | 7.102970652 | -3.520342856 | 0.000474767 |
| ZNF444 | -0.20183968 | 3.299329968 | -3.520147384 | 0.000475108 |
| RP11-486O12.2 | -0.318070359 | 1.129341531 | -3.519640834 | 0.000475992 |
| NWD1 | -0.560226181 | -4.554630182 | -3.519428361 | 0.000476363 |
| USP54 | -0.36561487 | 2.711369864 | -3.518597789 | 0.000477816 |
| CEP68 | -0.239049612 | 4.406871375 | -3.518492023 | 0.000478001 |
| ST6GALNAC4 | 0.281571654 | 4.984354204 | 3.518088142 | 0.00047871 |
| CTB-41I6.2 | -0.311506564 | 3.514844291 | -3.517874071 | 0.000479086 |
| FEN1 | 0.324168556 | 5.73889557 | 3.517239271 | 0.000480202 |
| MT-TD | 0.518061006 | 6.288656843 | 3.516716374 | 0.000481124 |
| AC022007.5 | -0.435154075 | -0.601784812 | -3.516640134 | 0.000481258 |
| SCARNA7 | -0.39643828 | 3.348776095 | -3.516506725 | 0.000481494 |
| SLC16A5 | -0.217577972 | 5.527282935 | -3.515751528 | 0.000482828 |
| AGAP1 | 0.58248197 | -0.092686563 | 3.515631036 | 0.000483042 |
| RRM2P3 | 0.391303538 | 2.803628241 | 3.514800241 | 0.000484514 |
| CD5L | 0.9110455 | -0.9630455 | 3.514517556 | 0.000485017 |
| SNORA65 | -0.333443344 | 0.798520306 | -3.514395391 | 0.000485234 |
| AC008984.7 | 0.616499365 | 0.207709545 | 3.514172804 | 0.00048563 |
| GPR176 | 0.484873625 | -2.099334841 | 3.513904823 | 0.000486107 |
| RNU6-998P | 0.420603485 | -0.210346721 | 3.513890437 | 0.000486132 |
| IDNK | -0.204851903 | 4.139549274 | -3.513467564 | 0.000486886 |
| CXorf38 | -0.169148961 | 4.834530109 | -3.513267074 | 0.000487244 |
| DHRS9 | -0.697728372 | 2.927933685 | -3.513022895 | 0.00048768 |
| CCDC151 | -0.426593868 | -1.381393455 | -3.512417651 | 0.000488762 |
| TOX4P1 | 0.177705544 | 2.089482028 | 3.511515248 | 0.00049038 |
| PSMA6P1 | -0.15684747 | 6.174719484 | -3.510069994 | 0.000492981 |
| ANKRD13A | 0.1490952 | 6.652858671 | 3.510030227 | 0.000493053 |
| LA16c-OS12.2 | -0.365636842 | 0.075357308 | -3.509889826 | 0.000493306 |
| RP11-250B2.3 | -0.41088025 | 0.937930894 | -3.50929756 | 0.000494376 |
| UNC13D | -0.187865193 | 6.914277218 | -3.509187343 | 0.000494576 |
| RP11-849F2.8 | -0.242067413 | 4.146460819 | -3.508553983 | 0.000495723 |
| AC005523.2 | 0.431549418 | -2.299259778 | 3.508222045 | 0.000496326 |
| METTL8 | -0.187644768 | 3.359625155 | -3.507822521 | 0.000497052 |
| SLC25A53 | -0.17657115 | 1.679192166 | -3.507811976 | 0.000497071 |
| RP11-350F4.2 | -0.134910629 | 3.973977469 | -3.507603714 | 0.00049745 |
| NOTCH4 | -0.420745944 | -0.078569299 | -3.507119152 | 0.000498332 |
| RPL3P4 | -0.160034406 | 11.40250821 | -3.506669274 | 0.000499153 |
| RNU5F-1 | -0.561180048 | 2.794066131 | -3.506564785 | 0.000499344 |
| ATG12 | -0.127819048 | 4.492888236 | -3.506144275 | 0.000500112 |
| CCDC65 | -0.454521827 | 0.246642713 | -3.50590884 | 0.000500543 |
| ZNF239 | -0.523399198 | 0.821898454 | -3.505642179 | 0.000501031 |
| TET3 | 0.142213511 | 5.989367981 | 3.505527583 | 0.000501241 |
| RP11-346D6.4 | -0.550748848 | -0.578858714 | -3.504179006 | 0.000503718 |
| UBA52 | 0.13470281 | 8.976299633 | 3.503388323 | 0.000505176 |
| C16orf13 | -0.162425933 | 6.363888362 | -3.503334714 | 0.000505275 |
| SMPDL3A | 0.798720317 | 2.041591439 | 3.502989782 | 0.000505912 |
| RN7SL517P | 0.425002804 | 0.831251105 | 3.502058626 | 0.000507636 |
| TAF3 | 0.128663861 | 4.658809901 | 3.501992425 | 0.000507759 |
| SMTN | 0.303111574 | 2.135476791 | 3.501088637 | 0.000509438 |
| SLC11A2 | 0.196965901 | 4.203671462 | 3.500488022 | 0.000510557 |
| SNORD78 | -0.425006828 | 1.063055452 | -3.500365567 | 0.000510785 |
| B3GALT2 | -0.42184676 | -2.815713961 | -3.500334186 | 0.000510844 |
| SNAPC1 | 0.238418325 | 3.338244518 | 3.500290493 | 0.000510925 |
| NDUFA4 | 0.182867461 | 5.858121317 | 3.499881511 | 0.000511689 |
| TTC23 | -0.54753729 | 0.822099553 | -3.49877221 | 0.000513765 |
| ADAMTSL4 | -0.470899511 | 5.124015012 | -3.498698199 | 0.000513904 |
| RP11-946L16.1 | -0.48710424 | 0.655814456 | -3.498556864 | 0.000514169 |
| BRSK2 | 0.545158544 | -3.737913069 | 3.498518602 | 0.000514241 |
| GBP3 | -0.256002133 | 4.176171071 | -3.498312764 | 0.000514628 |
| CCRL2 | 0.395201833 | 2.973104928 | 3.498291615 | 0.000514668 |
| TUSC3 | 0.643364919 | -3.582275707 | 3.498203017 | 0.000514834 |
| THTPA | -0.189674275 | 2.958586586 | -3.498080873 | 0.000515064 |
| BACH1-IT1 | -0.276564642 | 2.2446495 | -3.497766749 | 0.000515654 |
| F3 | 0.717571242 | 2.206316009 | 3.497688653 | 0.000515801 |
| HERC2P7 | -0.505589406 | -0.436859771 | -3.497644803 | 0.000515884 |
| ISG20 | 0.416880981 | 3.511952326 | 3.49721432 | 0.000516695 |
| SIGLEC20P | -0.610640947 | -0.113800882 | -3.496989349 | 0.000517119 |
| NCAPD3 | 0.206883774 | 4.887591075 | 3.496959076 | 0.000517176 |
| F12 | 0.419074879 | 3.056257597 | 3.496229997 | 0.000518554 |
| ANO1 | 0.598890785 | -4.386470265 | 3.496171855 | 0.000518664 |
| KLHL30 | -0.71263276 | -2.928553911 | -3.496168723 | 0.00051867 |
| ACTG1 | -0.15384437 | 11.53613688 | -3.496168491 | 0.00051867 |
| ZKSCAN5 | 0.158983847 | 4.103978218 | 3.496105149 | 0.00051879 |
| EXOC5P1 | -0.180513447 | 3.905815188 | -3.496103689 | 0.000518793 |
| CA11 | 0.325688508 | 2.60113623 | 3.495996231 | 0.000518996 |
| BLOC1S5 | -0.182665436 | 4.084664877 | -3.49501163 | 0.000520864 |
| RP4-665J23.1 | 0.550332067 | -2.693847707 | 3.494950722 | 0.000520979 |
| PLAT | 0.550372199 | -3.781883236 | 3.494505613 | 0.000521826 |
| RP11-480I12.5 | -0.549026305 | 1.988692648 | -3.493927925 | 0.000522926 |
| ITIH5 | 0.673221419 | -6.230848631 | 3.493917361 | 0.000522946 |
| PRKAR2A-AS1 | -0.321960201 | 0.150707938 | -3.493588483 | 0.000523574 |
| TBX1 | -0.73993444 | -0.639751507 | -3.493198106 | 0.000524319 |
| FOS | -0.323925196 | 10.81993158 | -3.493123141 | 0.000524463 |
| GOLGA6L21P | 0.503595685 | -0.211872901 | 3.492408554 | 0.000525831 |
| RNF123 | -0.162652885 | 4.96103664 | -3.491827034 | 0.000526946 |
| CTD-2260A17.2 | -0.242099913 | 2.664342667 | -3.491659559 | 0.000527268 |
| RP11-262H14.4 | -0.353449018 | 1.605074301 | -3.491511987 | 0.000527552 |
| SH3KBP1 | -0.194159779 | 7.377568354 | -3.490901747 | 0.000528726 |
| PCDHB9 | 0.623409907 | -2.629531838 | 3.489819712 | 0.000530814 |
| AXL | 0.799488249 | -0.619582112 | 3.489784672 | 0.000530882 |
| AC090587.2 | -0.309132268 | 0.383849264 | -3.488423051 | 0.000533521 |
| AC006116.17 | -0.396392451 | -0.866928154 | -3.488097902 | 0.000534153 |
| OXER1 | -0.374057497 | 3.779386354 | -3.487866165 | 0.000534604 |
| HSF1 | 0.094394186 | 6.346554208 | 3.487805282 | 0.000534723 |
| SYMPK | -0.120431467 | 5.704598637 | -3.487703599 | 0.000534921 |
| STIP1 | -0.195793394 | 6.725175092 | -3.487421839 | 0.00053547 |
| VBP1 | -0.143747761 | 5.93907149 | -3.487411246 | 0.00053549 |
| RP11-43F13.4 | 0.62774644 | -2.031829552 | 3.487398446 | 0.000535515 |
| VN1R82P | -0.264236583 | 3.22213265 | -3.486823274 | 0.000536638 |
| CD1C | -0.894905246 | 2.189596603 | -3.485363962 | 0.000539496 |
| PLGRKT | -0.156657504 | 4.830635272 | -3.484425252 | 0.000541341 |
| RP11-153M7.5 | -0.485428535 | -0.92427455 | -3.484098527 | 0.000541985 |
| GLULP4 | 0.399424607 | 0.559999754 | 3.482020289 | 0.000546097 |
| SHPRH | -0.118845644 | 4.068057455 | -3.481659594 | 0.000546814 |
| ZNF614 | 0.200527497 | 2.865183768 | 3.481609866 | 0.000546913 |
| SOCS4 | 0.130211728 | 5.069616849 | 3.481400165 | 0.00054733 |
| RP11-572P18.1 | 0.169307199 | 5.468469198 | 3.481234688 | 0.000547659 |
| BLOC1S1 | 0.206457906 | 4.033213875 | 3.481177973 | 0.000547772 |
| RP11-164J13.1 | -0.363782229 | 1.687356534 | -3.48048457 | 0.000549154 |
| SYNM | 0.524237796 | -0.311380982 | 3.480308658 | 0.000549506 |
| NEO1 | 0.583204792 | 1.462914005 | 3.479925715 | 0.000550271 |
| FXR1 | 0.139753423 | 6.596279818 | 3.479832226 | 0.000550458 |
| SMAP2 | -0.294262734 | 8.571597056 | -3.478954529 | 0.000552216 |
| RAB3IL1 | 0.734336978 | 0.494700065 | 3.478678743 | 0.00055277 |
| RP11-799D4.4 | -0.401303638 | 0.380832378 | -3.478600227 | 0.000552928 |
| ZNF296 | 0.276783274 | 4.733634108 | 3.478044992 | 0.000554044 |
| RAB11B | 0.096852237 | 7.328949804 | 3.477745926 | 0.000554646 |
| BCL2A1 | 0.613530676 | 7.292344146 | 3.477375136 | 0.000555394 |
| CUZD1 | -0.541977012 | -0.39513924 | -3.476904973 | 0.000556343 |
| ERAL1 | -0.136649401 | 6.133365098 | -3.476695057 | 0.000556767 |
| GNRH2 | -0.382505197 | -0.839350595 | -3.476603708 | 0.000556952 |
| TANC1 | 0.677582557 | 0.930553946 | 3.476157554 | 0.000557855 |
| IMPDH1 | -0.21272811 | 6.731991163 | -3.476118865 | 0.000557933 |
| ZNF558 | -0.185810396 | 4.158924461 | -3.475750217 | 0.00055868 |
| MAP3K14 | 0.260087822 | 4.49212258 | 3.474869975 | 0.000560468 |
| PIK3R5 | 0.340748212 | 5.915786559 | 3.4748151 | 0.00056058 |
| BRD7P4 | 0.185616662 | 2.011948462 | 3.474751346 | 0.00056071 |
| CPT1A | -0.21327312 | 7.626147516 | -3.472994755 | 0.000564295 |
| CHN2 | -0.460834452 | 1.869781225 | -3.472589481 | 0.000565126 |
| TIGD4 | -0.380455805 | -1.141405443 | -3.472487787 | 0.000565334 |
| WWOX | 0.199987227 | 3.318992001 | 3.471907269 | 0.000566526 |
| EPN1 | 0.136628894 | 6.310667805 | 3.471702872 | 0.000566946 |
| TMTC4 | -0.233228705 | 4.205920641 | -3.47133621 | 0.000567701 |
| PSMB8 | -0.158699344 | 7.678026654 | -3.470760872 | 0.000568886 |
| DSCAML1 | 0.503817825 | -3.854938836 | 3.469812881 | 0.000570845 |
| CDS1 | 0.490900921 | -2.897007178 | 3.469751636 | 0.000570972 |
| GTF2A1 | -0.207066393 | 4.77321128 | -3.469749519 | 0.000570976 |
| ZNF804A | -0.706966179 | 2.445732535 | -3.469470652 | 0.000571554 |
| RP11-875O11.1 | -0.306953279 | 2.085963444 | -3.469135738 | 0.000572248 |
| UBE2E3 | 0.312905674 | 5.584308532 | 3.469126269 | 0.000572268 |
| MED7 | -0.174879169 | 4.0107518 | -3.4691203 | 0.00057228 |
| NUDT4P1 | 0.204198823 | 6.538630561 | 3.46896975 | 0.000572592 |
| RPL21P116 | 0.404707598 | 2.019072881 | 3.468687099 | 0.000573179 |
| RP11-367N14.2 | -0.322790292 | 0.43490392 | -3.468565149 | 0.000573433 |
| AL137059.1 | -0.261785492 | 2.71081123 | -3.468461282 | 0.000573649 |
| KB-1991G8.1 | -0.583815152 | -1.435481188 | -3.468193551 | 0.000574206 |
| HLA-E | 0.203844098 | 10.81959896 | 3.46754617 | 0.000575554 |
| RP11-770J1.3 | -0.453936079 | 0.950041774 | -3.467341849 | 0.00057598 |
| NTMT1 | 0.147924015 | 4.251560567 | 3.467337746 | 0.000575989 |
| RP11-492M23.2 | 0.177555215 | 4.234100888 | 3.467147482 | 0.000576386 |
| ATP2A2 | 0.158250616 | 6.405398587 | 3.466860443 | 0.000576986 |
| DNAJC3 | 0.178557207 | 6.544686647 | 3.466670755 | 0.000577383 |
| ANO7P1 | 0.352955926 | 0.896257007 | 3.466315639 | 0.000578126 |
| EFNA5 | 0.526331067 | -2.625586921 | 3.466174144 | 0.000578422 |
| CARKD | 0.259502436 | 4.03507164 | 3.466010251 | 0.000578766 |
| NOD1 | -0.153165881 | 4.486273666 | -3.465983005 | 0.000578823 |
| PLXDC2 | -0.450709785 | 5.045275754 | -3.465193613 | 0.00058048 |
| LRP6 | 0.847750389 | 0.549285863 | 3.465159612 | 0.000580551 |
| SPOCK1 | -0.712345509 | -1.122357147 | -3.465113577 | 0.000580648 |
| ATP6V0E2-AS1 | -0.452979843 | -1.051954668 | -3.465106623 | 0.000580663 |
| RP11-90P5.7 | 0.340837524 | 0.438516508 | 3.464703352 | 0.000581511 |
| CCL3L1 | 0.714038716 | 1.868953915 | 3.464548306 | 0.000581838 |
| AC005789.11 | 0.409816297 | -1.243440358 | 3.463719151 | 0.000583587 |
| SAE1 | 0.143082014 | 6.432418932 | 3.463616673 | 0.000583804 |
| SKI | 0.232754045 | 6.152272659 | 3.463527192 | 0.000583993 |
| CCL3L3 | 0.711934491 | 0.224404473 | 3.463316368 | 0.000584439 |
| CD46 | -0.141856977 | 6.609360731 | -3.46053689 | 0.000590347 |
| PPAPDC1A | 0.584247059 | -2.361267904 | 3.460208745 | 0.000591048 |
| TYRO3P | 0.360838569 | 0.025012726 | 3.459696743 | 0.000592143 |
| DLEU7-AS1 | -0.592304886 | -0.526113361 | -3.458857018 | 0.000593944 |
| SON | 0.121748716 | 7.937647425 | 3.458495998 | 0.00059472 |
| TRPV1 | -0.388463191 | -2.045809062 | -3.456861917 | 0.000598243 |
| PIGB | 0.18719181 | 3.947784938 | 3.456779468 | 0.000598422 |
| ACVR1 | -0.283940941 | 4.270532718 | -3.456769723 | 0.000598443 |
| AGAP4 | -0.314941181 | 1.870573033 | -3.456275287 | 0.000599513 |
| UXS1 | -0.157092203 | 5.270704096 | -3.456133043 | 0.000599821 |
| RP11-114H7.2 | -0.339293019 | -0.839893387 | -3.455673467 | 0.000600818 |
| DCAF13 | -0.131381146 | 4.773415616 | -3.455112851 | 0.000602036 |
| RP6-91H8.3 | 0.473396747 | -1.604320405 | 3.454581075 | 0.000603194 |
| MRPL10 | 0.132390967 | 6.140252851 | 3.454539643 | 0.000603284 |
| RP11-203M5.6 | -0.578109974 | -0.872436832 | -3.454409238 | 0.000603569 |
| SGK3 | -0.174476485 | 3.87769063 | -3.45436655 | 0.000603662 |
| LINC00630 | 0.203002763 | 1.283896491 | 3.453751105 | 0.000605005 |
| RP11-51F16.5 | 0.376299116 | 1.487192922 | 3.453297286 | 0.000605997 |
| HDAC1 | -0.12016308 | 7.110070786 | -3.453266478 | 0.000606065 |
| RNF24 | -0.207866174 | 6.939727153 | -3.453199051 | 0.000606212 |
| TPR | -0.109893937 | 6.799359512 | -3.453179563 | 0.000606255 |
| RP11-584P21.2 | 0.38647242 | -0.54149897 | 3.453091769 | 0.000606447 |
| GTF2A2 | -0.156447755 | 5.810983992 | -3.45306874 | 0.000606498 |
| RP11-434P11.2 | 0.304234907 | 2.384330057 | 3.452955664 | 0.000606745 |
| MORC4 | 0.473365183 | 0.021224505 | 3.452949121 | 0.00060676 |
| U2AF2 | -0.124377022 | 7.994148008 | -3.452295342 | 0.000608194 |
| RP11-118K6.3 | -0.462616496 | 1.053824262 | -3.452100455 | 0.000608622 |
| HNRNPA1P12 | 0.281724386 | 1.362137592 | 3.449758025 | 0.000613789 |
| CTD-3193O13.10 | -0.205044497 | 3.360491626 | -3.448886287 | 0.000615722 |
| MAP1LC3B2 | 0.251230243 | 6.541947333 | 3.448181753 | 0.000617289 |
| B4GALNT4 | 0.604894987 | -1.215174262 | 3.447490299 | 0.00061883 |
| CATSPER2P1 | 0.28426031 | 0.502017364 | 3.447478294 | 0.000618857 |
| CHD4 | -0.176619456 | 8.168629274 | -3.44729747 | 0.000619261 |
| GJA4 | 0.65012453 | -1.318227192 | 3.44699891 | 0.000619928 |
| RP5-1115A15.1 | -0.396813078 | 1.468235541 | -3.446636885 | 0.000620737 |
| RP3-481A17.1 | -0.361638239 | 0.132401028 | -3.445981436 | 0.000622206 |
| RP11-502I4.3 | -0.265923384 | 1.586867854 | -3.445711707 | 0.000622811 |
| LONRF2 | 0.720191957 | -2.733289708 | 3.444624326 | 0.000625257 |
| ZCCHC17 | -0.12740463 | 6.031613765 | -3.443238199 | 0.000628387 |
| YWHAZP5 | 0.213217631 | 3.081786506 | 3.442786646 | 0.00062941 |
| DDT | 0.145207126 | 5.348285737 | 3.442468837 | 0.000630131 |
| TSHZ3 | -0.672192877 | 1.690036506 | -3.442083067 | 0.000631007 |
| PCYT1A | -0.138032516 | 5.478745661 | -3.441829859 | 0.000631583 |
| KBTBD6 | 0.270536659 | 3.214631062 | 3.44161341 | 0.000632075 |
| HERC2P3 | -0.390217399 | 2.797144771 | -3.441257353 | 0.000632886 |
| TSPAN9 | 0.635732288 | -0.575728959 | 3.439695649 | 0.000636454 |
| CXCR2 | -0.771839842 | 3.662752565 | -3.439347258 | 0.000637253 |
| PAQR9 | 0.72532649 | -2.287454871 | 3.439025959 | 0.00063799 |
| CDC42EP3 | 0.439204834 | 5.721085057 | 3.438853561 | 0.000638386 |
| GTPBP6 | -0.147216887 | 6.491221008 | -3.438323042 | 0.000639606 |
| DHX32 | -0.315287261 | 4.422253582 | -3.438209486 | 0.000639868 |
| RP11-745O10.2 | -0.584022223 | 0.881024769 | -3.437915286 | 0.000640545 |
| FAN1 | -0.196383731 | 4.824954312 | -3.437512309 | 0.000641475 |
| PDHA1 | -0.148438198 | 6.438960789 | -3.437388004 | 0.000641761 |
| GNG4 | 0.678685786 | -2.413931487 | 3.437060501 | 0.000642518 |
| CTD-2240J17.1 | -0.53308366 | -1.125359512 | -3.436405201 | 0.000644034 |
| PCSK1N | 0.484561362 | -0.463609911 | 3.436030555 | 0.000644902 |
| RP11-529H20.3 | 0.242341389 | 4.33677427 | 3.435729043 | 0.000645602 |
| KLHL2P1 | -0.381709357 | 0.354874227 | -3.434851871 | 0.000647641 |
| RSU1 | -0.190624465 | 6.697119091 | -3.434553033 | 0.000648338 |
| RP1-80N2.3 | -0.566005085 | -1.692427052 | -3.434262405 | 0.000649015 |
| HIF1A-AS2 | -0.482555453 | -0.131490662 | -3.433807681 | 0.000650077 |
| TTC30A | -0.35423711 | 0.997603318 | -3.43364116 | 0.000650466 |
| SSB | -0.124727927 | 6.447855612 | -3.43333513 | 0.000651182 |
| MERTK | -0.414564751 | 4.105633304 | -3.433245191 | 0.000651393 |
| MYO19 | 0.151939016 | 4.462168483 | 3.4331236 | 0.000651677 |
| TMEM56 | 0.916189221 | -0.124232321 | 3.433038602 | 0.000651876 |
| PRADC1P1 | -0.350336376 | -0.091905082 | -3.432902747 | 0.000652195 |
| CD8A | 0.536962683 | 3.901942638 | 3.432854591 | 0.000652308 |
| CCT6P1 | 0.206674339 | 4.22997806 | 3.432824323 | 0.000652379 |
| CALCB | 0.421383727 | -2.789715648 | 3.432209961 | 0.00065382 |
| MARCKSL1 | 0.370229619 | 7.524332224 | 3.432067428 | 0.000654155 |
| KTI12 | 0.195872132 | 5.506507826 | 3.431835743 | 0.0006547 |
| A3GALT2 | 0.449871124 | 0.019720788 | 3.431675047 | 0.000655078 |
| PVRL4 | 0.719759315 | -1.127842224 | 3.431534864 | 0.000655408 |
| OR2L5 | -0.444975667 | -1.535672217 | -3.430920187 | 0.000656857 |
| NPM1P27 | -0.199699545 | 10.36802028 | -3.430682351 | 0.000657418 |
| LINC00863 | 0.237205448 | 3.210387645 | 3.430393312 | 0.000658101 |
| IL5RA | -0.702520978 | 1.114557131 | -3.430240139 | 0.000658463 |
| RP11-140K17.3 | -0.166470514 | 2.860809345 | -3.430082597 | 0.000658836 |
| DUSP1 | -0.320792944 | 10.65688702 | -3.429582234 | 0.00066002 |
| RP11-393N4.2 | 0.249303539 | 3.337102379 | 3.429551724 | 0.000660093 |
| SNX25 | 0.547490435 | 1.066694351 | 3.429529568 | 0.000660145 |
| AC144831.1 | 0.512313694 | 1.315209508 | 3.429209581 | 0.000660904 |
| COX20P2 | -0.237154522 | 2.273830009 | -3.428797907 | 0.000661882 |
| AC002511.3 | 0.443907318 | -1.386439761 | 3.428773003 | 0.000661941 |
| RP11-259G18.1 | 0.377448679 | 0.317908602 | 3.428656319 | 0.000662218 |
| GRAMD1B | 0.472504945 | 2.996081857 | 3.428210011 | 0.00066328 |
| CTD-2651B20.3 | -0.42355022 | -0.850831005 | -3.427537934 | 0.000664882 |
| CTGF | 0.92496711 | -0.191282069 | 3.427246312 | 0.000665578 |
| RP11-381E24.1 | 0.268045493 | 2.974095674 | 3.427002116 | 0.000666161 |
| FBLN5 | 0.701112459 | 0.62440806 | 3.426928837 | 0.000666337 |
| SIGLEC6 | -0.66366851 | 1.636291762 | -3.425873971 | 0.000668863 |
| FCGR1BP | -0.53887383 | 3.307018679 | -3.425823366 | 0.000668985 |
| RP11-34P1.2 | -0.199095541 | 7.539857996 | -3.425300977 | 0.00067024 |
| BIN1 | 0.442639155 | 4.066854871 | 3.42525057 | 0.000670361 |
| RP13-131K19.2 | -0.299701279 | 1.153664313 | -3.424538356 | 0.000672075 |
| MPP2 | 0.56439134 | -1.031911287 | 3.423596622 | 0.000674349 |
| SHFM1 | 0.153499148 | 4.703975545 | 3.423274193 | 0.000675129 |
| VPS37A | -0.1355325 | 4.823618059 | -3.422399396 | 0.000677249 |
| ACSL4 | -0.202417911 | 6.18912517 | -3.422355298 | 0.000677356 |
| PIK3CG | 0.238245226 | 5.949232293 | 3.422012258 | 0.00067819 |
| CYP51A1P2 | 0.2499616 | 2.904496316 | 3.421997907 | 0.000678224 |
| COG7 | -0.10392893 | 4.404811147 | -3.421261204 | 0.000680017 |
| CTC-820M8.1 | -0.405647026 | -0.145431927 | -3.421108215 | 0.00068039 |
| ECI2 | -0.196182598 | 4.637567166 | -3.420674069 | 0.00068145 |
| RNU6-48P | 0.328156403 | 0.643556925 | 3.420471321 | 0.000681945 |
| RP11-39C10.1 | 0.375841815 | 2.400711822 | 3.420004219 | 0.000683087 |
| RPS19P3 | 0.233317065 | 1.638954228 | 3.419940751 | 0.000683242 |
| WDR83 | -0.111156033 | 4.144456047 | -3.419380514 | 0.000684615 |
| TCEAL5 | 0.56165185 | -1.046535982 | 3.419239851 | 0.00068496 |
| DISC1FP1 | 0.438587256 | -2.460321223 | 3.418754235 | 0.000686152 |
| TRAV12-3 | -0.486449734 | 0.073689257 | -3.418606493 | 0.000686515 |
| GPATCH2L | 0.127785694 | 5.830917847 | 3.418231769 | 0.000687437 |
| RP11-488C13.1 | -0.213289508 | 8.766219946 | -3.418096175 | 0.000687771 |
| IL1R1 | 0.694232744 | 2.753580678 | 3.417697814 | 0.000688753 |
| STX8 | -0.163660703 | 4.626586526 | -3.417616748 | 0.000688953 |
| DDX49 | 0.097405735 | 6.077833584 | 3.417435114 | 0.000689401 |
| RP11-927P21.1 | -0.524657638 | -1.213053132 | -3.416904549 | 0.000690711 |
| THOP1 | 0.16090664 | 5.038887266 | 3.41620937 | 0.000692432 |
| RNU1-106P | -0.438534206 | 0.437782521 | -3.416168068 | 0.000692535 |
| CDC23 | -0.153892151 | 5.112749513 | -3.416010682 | 0.000692925 |
| SYT17 | -0.727109403 | -1.732460929 | -3.415258266 | 0.000694793 |
| CTA-217C2.2 | -0.340456556 | 1.801866942 | -3.415204347 | 0.000694927 |
| CDC7 | 0.218373298 | 4.630338781 | 3.41381292 | 0.000698395 |
| AC006116.20 | -0.542520231 | -1.28698779 | -3.413775168 | 0.000698489 |
| POLD4 | -0.182064831 | 4.015211101 | -3.413771021 | 0.0006985 |
| LINC00174 | -0.244596566 | 3.223094318 | -3.413090818 | 0.000700201 |
| ATE1-AS1 | -0.525673297 | 1.442760738 | -3.412824656 | 0.000700868 |
| EIF3C | 0.162316758 | 3.681406234 | 3.412762166 | 0.000701025 |
| RBAK | 0.146782058 | 4.33295576 | 3.411489463 | 0.000704222 |
| LYSMD2 | -0.193373468 | 4.984808957 | -3.411311882 | 0.000704669 |
| FAM65C | 0.853232483 | 1.276645349 | 3.410610226 | 0.000706439 |
| AC000068.5 | -0.365824176 | -0.397271448 | -3.410220364 | 0.000707424 |
| TTC21B | 0.096573869 | 3.921800924 | 3.410083654 | 0.00070777 |
| BMPR1APS2 | -0.582684139 | -0.360068286 | -3.409833534 | 0.000708402 |
| RP11-483I13.5 | -0.312605855 | 1.116639681 | -3.408811453 | 0.000710994 |
| TRAV36DV7 | -0.380258292 | -0.273674948 | -3.408673893 | 0.000711344 |
| RPL7P1 | -0.183464867 | 9.817736192 | -3.408644381 | 0.000711419 |
| C1orf131 | -0.203556494 | 3.150974763 | -3.40799365 | 0.000713074 |
| RGL1 | 0.62596454 | 0.950550858 | 3.406987734 | 0.000715641 |
| CORO6 | 0.460933144 | 1.615627302 | 3.406615387 | 0.000716593 |
| AP1S2 | -0.340389271 | 6.32536607 | -3.406601043 | 0.00071663 |
| CLASP1 | 0.097588183 | 5.520112548 | 3.40645378 | 0.000717007 |
| NGLY1 | -0.130434652 | 5.2460537 | -3.406414014 | 0.000717109 |
| SLC25A29 | -0.310014681 | 5.365639843 | -3.405929513 | 0.00071835 |
| MKRN7P | -0.449048467 | -1.892347207 | -3.405892098 | 0.000718446 |
| MT-CO2 | -0.116683492 | 13.58480045 | -3.405234805 | 0.000720134 |
| RAI2 | 0.435124538 | -2.381572759 | 3.404994891 | 0.000720751 |
| AC005014.5 | 0.249769314 | 0.75381308 | 3.404730166 | 0.000721432 |
| RP11-428K3.1 | -0.428214762 | -0.884497884 | -3.404672343 | 0.000721581 |
| TNRC18 | -0.179503634 | 7.271812601 | -3.403806685 | 0.000723814 |
| JOSD2 | -0.242886507 | 4.289789632 | -3.403790033 | 0.000723857 |
| ZNF273 | 0.30000318 | 3.362126907 | 3.403638654 | 0.000724248 |
| TNIK | 0.447956122 | 3.257179718 | 3.403301377 | 0.00072512 |
| TOX4 | 0.108104651 | 6.04399821 | 3.402904808 | 0.000726147 |
| MTMR14 | 0.15755013 | 5.959375988 | 3.402869577 | 0.000726238 |
| AC005336.5 | -0.346706679 | -0.540800195 | -3.402764591 | 0.00072651 |
| DQX1 | -0.398502214 | -1.893890206 | -3.40241414 | 0.000727419 |
| DAB2 | -0.37001669 | 3.824977642 | -3.402333014 | 0.000727629 |
| AC008427.2 | 0.177545977 | 2.598345506 | 3.401950923 | 0.000728622 |
| CTD-2203K17.1 | -0.383851905 | 2.653840012 | -3.401600346 | 0.000729533 |
| LYAR | -0.215601292 | 5.635176754 | -3.401223438 | 0.000730514 |
| CTD-2525I3.5 | -0.503670741 | 0.281810439 | -3.400900183 | 0.000731357 |
| GSE1 | -0.241267062 | 6.544042185 | -3.400885474 | 0.000731395 |
| FBXW8 | -0.243221084 | 4.270200646 | -3.400875646 | 0.000731421 |
| DNAJC19P5 | 0.292043368 | 2.501825497 | 3.40024634 | 0.000733064 |
| OAS2 | -0.501180757 | 4.923215113 | -3.400170722 | 0.000733262 |
| C1DP1 | 0.141157589 | 5.634277506 | 3.400069492 | 0.000733526 |
| SLC16A1 | 0.380252048 | 3.287923834 | 3.399661472 | 0.000734594 |
| ZNF865 | 0.162362726 | 5.948355832 | 3.399547585 | 0.000734892 |
| DOC2B | 0.790274499 | 1.039008117 | 3.399264473 | 0.000735634 |
| PTGDS | -0.727742902 | 2.959543753 | -3.399091069 | 0.000736089 |
| KIF3A | -0.240444079 | 2.646969924 | -3.398931133 | 0.000736509 |
| SLC19A1 | -0.239299978 | 3.855620362 | -3.397960823 | 0.00073906 |
| SMC6 | -0.162541206 | 4.953307255 | -3.397739604 | 0.000739643 |
| PLD1 | -0.317926324 | 4.291271354 | -3.39769144 | 0.00073977 |
| AGO1 | -0.113557514 | 5.839368182 | -3.397027918 | 0.000741521 |
| RP11-347I19.8 | 0.380703928 | -0.251365756 | 3.396958406 | 0.000741704 |
| AP4S1 | -0.202097654 | 2.627811561 | -3.396650614 | 0.000742518 |
| TMEM128 | 0.137299096 | 4.850173556 | 3.39552716 | 0.000745495 |
| MAP2K6 | -0.342678342 | 2.80674583 | -3.395352308 | 0.000745959 |
| TFP1 | -0.370666572 | -0.044666607 | -3.394815136 | 0.000747388 |
| LRRC37A15P | 0.25376745 | 3.950285256 | 3.394779755 | 0.000747482 |
| RNH1 | -0.237890695 | 6.46991575 | -3.394629422 | 0.000747882 |
| TIMD4 | 0.637535719 | 0.686382186 | 3.393958561 | 0.00074967 |
| DDX52 | 0.123103181 | 4.60809991 | 3.393866172 | 0.000749917 |
| CEP170B | 0.503030509 | 0.889169405 | 3.393523547 | 0.000750832 |
| GPR114 | -0.528094319 | 4.61807935 | -3.393305934 | 0.000751414 |
| RP11-298O21.2 | -0.590883262 | -0.908312805 | -3.39308718 | 0.000751999 |
| TRMT44 | -0.146609067 | 3.045806182 | -3.392955155 | 0.000752353 |
| TDRD7 | -0.259096542 | 4.859803866 | -3.391914045 | 0.000755145 |
| NUBPL | -0.186047907 | 1.892699739 | -3.391530242 | 0.000756177 |
| AC005264.2 | 0.403429624 | -1.409816346 | 3.391281177 | 0.000756847 |
| ZBTB3 | -0.211017024 | 2.756044143 | -3.390702484 | 0.000758407 |
| RP11-301G23.1 | -0.338033854 | -0.410192547 | -3.389968724 | 0.000760389 |
| RP11-217B7.2 | 0.43683675 | -0.014244242 | 3.389841633 | 0.000760732 |
| EBF1 | 0.634458005 | -0.80720005 | 3.389510963 | 0.000761628 |
| H3F3B | 0.252699698 | 10.13638029 | 3.388171901 | 0.000765262 |
| RP11-829H16.3 | -0.436099325 | 0.515949527 | -3.387921004 | 0.000765945 |
| AC009133.12 | -0.367087636 | -0.95213667 | -3.387558551 | 0.000766932 |
| PABPC1P3 | -0.14488783 | 7.275209418 | -3.387419445 | 0.000767312 |
| FBXO41 | -0.241038194 | 5.034801443 | -3.387196738 | 0.000767919 |
| RN7SL587P | -0.374656386 | 1.194706129 | -3.387122496 | 0.000768122 |
| ANGPTL2 | 0.565312634 | -3.274912035 | 3.386416375 | 0.000770052 |
| NFX1 | 0.119148551 | 5.648721041 | 3.386234894 | 0.000770549 |
| RP11-353H3.1 | 0.357462948 | 1.130509517 | 3.386045306 | 0.000771068 |
| RNF135 | -0.214245973 | 5.203720738 | -3.385810709 | 0.000771711 |
| SNORD63 | 0.383330613 | 1.087729911 | 3.385600692 | 0.000772287 |
| CEP76 | 0.174169737 | 2.739702337 | 3.385228224 | 0.000773309 |
| SF3A3 | 0.115027363 | 6.843755369 | 3.385202523 | 0.00077338 |
| TBC1D3C | -0.41942753 | -1.403489094 | -3.3851589 | 0.0007735 |
| TGM1 | -0.332104582 | 0.327689164 | -3.385155447 | 0.000773509 |
| TRIT1 | -0.151959155 | 4.73886433 | -3.385060973 | 0.000773769 |
| TMTC2 | -0.576029431 | 3.234088358 | -3.384503852 | 0.000775302 |
| EXOC3L1 | -0.500727003 | -1.574136189 | -3.383773623 | 0.000777315 |
| CRYBG3 | -0.449987729 | 3.260362863 | -3.382879462 | 0.000779786 |
| PPIL6 | -0.412253311 | -0.259071743 | -3.38286509 | 0.000779826 |
| PEX7 | 0.16004697 | 3.466707911 | 3.38254971 | 0.0007807 |
| RP11-37C7.3 | -0.384391649 | -0.655579362 | -3.382317219 | 0.000781344 |
| SCRN2 | -0.194732459 | 5.23642463 | -3.382278568 | 0.000781452 |
| ELMO3 | -0.197508139 | 2.63198375 | -3.381900082 | 0.000782502 |
| LINC00526 | -0.299011187 | 1.942190974 | -3.381366412 | 0.000783986 |
| MAPKAP1 | -0.120513431 | 5.906999106 | -3.381100751 | 0.000784725 |
| RP1-257I20.14 | 0.258917773 | 2.594914962 | 3.381095431 | 0.00078474 |
| SNORD11B | 0.347621503 | 0.236386368 | 3.381093092 | 0.000784747 |
| ADCY2 | -1.111671012 | -2.901694672 | -3.38101017 | 0.000784978 |
| LCA5L | -0.476072605 | -1.715269557 | -3.380970505 | 0.000785088 |
| CTSE | 0.795802328 | -0.264846905 | 3.380913451 | 0.000785247 |
| SLC4A1AP | -0.11504861 | 4.764197686 | -3.380351283 | 0.000786815 |
| SVEP1 | 0.72289819 | -5.233651698 | 3.380326953 | 0.000786883 |
| ARHGAP17 | -0.150773045 | 4.94127687 | -3.380004634 | 0.000787783 |
| PLCD3 | 0.401605805 | 2.413358883 | 3.379746251 | 0.000788506 |
| PLEKHJ1 | -0.137407479 | 5.083017534 | -3.379656965 | 0.000788755 |
| ZNRF1 | -0.339706088 | 4.652341617 | -3.379142917 | 0.000790195 |
| ACVR2A | 0.515149403 | 0.744483846 | 3.378792807 | 0.000791177 |
| TRAF1 | 0.308323466 | 3.997695884 | 3.378472213 | 0.000792077 |
| RP11-25I15.3 | -0.5413649 | -1.68367602 | -3.377765682 | 0.000794064 |
| GP9 | 0.727975534 | 2.078818071 | 3.375641322 | 0.000800066 |
| CTD-2006K23.1 | -0.769375614 | 0.574494725 | -3.373932424 | 0.000804925 |
| ELMO2 | 0.163557228 | 5.84734711 | 3.373015025 | 0.000807545 |
| TDP1 | 0.152991786 | 4.360634489 | 3.372970787 | 0.000807672 |
| MLC1 | -0.517018278 | 6.543967933 | -3.372828593 | 0.000808079 |
| RP11-408O19.5 | -0.423816465 | 0.459470847 | -3.371539523 | 0.000811776 |
| KPNA5 | 0.204432359 | 4.562021131 | 3.371149019 | 0.000812899 |
| TBC1D3B | -0.42477398 | -0.824752757 | -3.370925799 | 0.000813541 |
| RP1-152L7.5 | -0.475882444 | 0.929616613 | -3.370796626 | 0.000813913 |
| TNFAIP1 | -0.170553328 | 4.247259975 | -3.370518948 | 0.000814714 |
| CTA-357J21.1 | -0.413950498 | -0.162046365 | -3.370321584 | 0.000815283 |
| AC093724.2 | 0.180912908 | 4.022495784 | 3.368767624 | 0.000819779 |
| ZNF564 | 0.30768551 | -0.761753562 | 3.368534584 | 0.000820455 |
| NUDT12 | -0.424685159 | 1.970851461 | -3.367370269 | 0.000823841 |
| CPSF1 | -0.173256398 | 7.153158225 | -3.367293411 | 0.000824065 |
| HPCAL4 | 0.508011193 | -0.342593628 | 3.366978961 | 0.000824982 |
| RP11-42O15.2 | -0.38430294 | -0.626179012 | -3.366914454 | 0.00082517 |
| NME7 | -0.238162314 | 2.919426311 | -3.366342788 | 0.00082684 |
| CTD-3224K15.2 | -0.437881305 | 0.112608357 | -3.366044735 | 0.000827712 |
| CTD-2659N19.9 | -0.399614185 | -0.376806217 | -3.365794488 | 0.000828444 |
| HIST1H2AI | -0.539312592 | 2.221243113 | -3.36555431 | 0.000829148 |
| SETBP1 | 0.612506798 | 2.04553827 | 3.364274447 | 0.000832908 |
| RP11-256L6.2 | -0.428545574 | -0.012640345 | -3.364171995 | 0.000833209 |
| TBC1D2 | -0.319174434 | 5.260440651 | -3.363546912 | 0.000835052 |
| MYCT1 | 0.933980045 | 0.287592687 | 3.363257044 | 0.000835907 |
| RNU7-163P | 0.335797682 | 2.759049013 | 3.361994265 | 0.000839645 |
| URI1 | -0.140780539 | 6.486600102 | -3.361705995 | 0.0008405 |
| C1orf43 | -0.126622891 | 7.738049431 | -3.361581442 | 0.00084087 |
| PKP4P1 | -0.328170201 | 0.322730599 | -3.361558918 | 0.000840937 |
| STK11 | -0.143774629 | 5.579349421 | -3.361088348 | 0.000842336 |
| NKAPL | -0.45761284 | -1.76652697 | -3.360737842 | 0.000843379 |
| HBQ1 | 0.670281495 | 1.343011511 | 3.360551302 | 0.000843934 |
| RP11-456P18.2 | 0.319901322 | 1.839362808 | 3.359376246 | 0.000847443 |
| RAB8A | -0.17281379 | 7.116812906 | -3.358894652 | 0.000848885 |
| RP3-327A19.5 | -0.196948985 | 3.768262737 | -3.35838547 | 0.000850411 |
| FLJ45079 | 0.468455193 | -2.775274213 | 3.358303199 | 0.000850658 |
| DMTN | 0.599472021 | 3.489722821 | 3.358164957 | 0.000851073 |
| MYC | -0.383522996 | 7.177430323 | -3.358052376 | 0.000851412 |
| RP11-706O15.7 | -0.505660289 | -1.23940659 | -3.357225833 | 0.000853898 |
| AC004069.2 | -0.231853293 | 4.145471653 | -3.356637621 | 0.000855672 |
| RP6-109B7.4 | -0.376242586 | 0.413251444 | -3.356275767 | 0.000856765 |
| ZSCAN32 | -0.167768929 | 3.979251884 | -3.356043344 | 0.000857468 |
| LPAR2 | 0.263341313 | 6.128966521 | 3.355752485 | 0.000858348 |
| GFRA1 | 0.591690616 | -5.117324011 | 3.35554374 | 0.00085898 |
| GRIP1 | -0.605050316 | 0.03910587 | -3.355354763 | 0.000859552 |
| TREML1 | 0.732110318 | 1.13886051 | 3.354816729 | 0.000861185 |
| LMX1B | -0.777967372 | -3.905034357 | -3.354761467 | 0.000861352 |
| ARL5C | -0.595080101 | -0.975356115 | -3.354718398 | 0.000861483 |
| RP11-463O9.9 | -0.349211426 | -1.091979613 | -3.354638399 | 0.000861726 |
| B3GNT2 | 0.266474645 | 7.209689884 | 3.354441588 | 0.000862324 |
| RP3-477O4.14 | -0.151230482 | 4.354007408 | -3.353853416 | 0.000864114 |
| UBL4A | -0.186952817 | 5.676919477 | -3.353847159 | 0.000864133 |
| TRAV17 | -0.502646321 | 0.03083984 | -3.353777093 | 0.000864347 |
| CILP2 | 0.623726473 | 0.992848015 | 3.353441697 | 0.000865369 |
| RP11-543C4.1 | 0.439656183 | -1.099346539 | 3.352633273 | 0.000867839 |
| RAB19 | 0.573526533 | 0.006003794 | 3.351540792 | 0.000871186 |
| ONECUT3 | -0.706400619 | -5.067471939 | -3.351418004 | 0.000871563 |
| ZBTB6 | 0.268432912 | 3.883270659 | 3.350524494 | 0.00087431 |
| RPL23AP53 | -0.217496886 | 2.364111562 | -3.350294999 | 0.000875017 |
| TRAV19 | -0.483860609 | 0.062077112 | -3.350286446 | 0.000875044 |
| C18orf32 | 0.237379028 | 2.598571817 | 3.350278179 | 0.000875069 |
| UBN2 | -0.171838511 | 4.416045568 | -3.350255587 | 0.000875139 |
| ATMIN | 0.133157821 | 5.763819523 | 3.350025703 | 0.000875847 |
| RP11-318C24.1 | 0.354914786 | 2.051475731 | 3.349399571 | 0.000877781 |
| AC004967.7 | -0.182785995 | 4.625278907 | -3.349000108 | 0.000879016 |
| C16orf70 | 0.206488778 | 4.241072088 | 3.348501771 | 0.00088056 |
| AC105402.4 | -0.476558767 | -2.539336015 | -3.348499696 | 0.000880566 |
| VPS13D | -0.092929435 | 5.374032423 | -3.348441402 | 0.000880747 |
| KANSL1-AS1 | -0.338430129 | 2.937329978 | -3.348176907 | 0.000881568 |
| BTBD11 | 0.433966665 | 3.828458234 | 3.347879752 | 0.00088249 |
| MIR643 | 0.353484771 | 0.200645099 | 3.34775461 | 0.000882879 |
| RP11-312O7.2 | 0.317142398 | 2.659994807 | 3.347646508 | 0.000883215 |
| CA12 | 0.556780927 | -4.159049485 | 3.347541285 | 0.000883542 |
| STIM2 | 0.197829432 | 4.897086201 | 3.347379371 | 0.000884046 |
| PITX1 | 0.678519261 | -2.489632764 | 3.346591067 | 0.000886502 |
| SUPT3H | -0.262504395 | 4.159423139 | -3.346529393 | 0.000886694 |
| AP000344.3 | -0.534078935 | -0.861160821 | -3.3465212 | 0.00088672 |
| ZNF488 | -0.616298527 | -2.079642023 | -3.34617726 | 0.000887793 |
| MIR4523 | -0.371006905 | -0.144815782 | -3.346164747 | 0.000887832 |
| RP11-32B5.7 | -0.554755056 | 1.016188045 | -3.345800699 | 0.00088897 |
| RP11-29H23.5 | 0.328769517 | 0.474542282 | 3.345624498 | 0.000889522 |
| PIGT | 0.188807036 | 8.275486154 | 3.345597288 | 0.000889607 |
| DAPK1 | -0.379803558 | 5.563994025 | -3.345244336 | 0.000890712 |
| VWA2 | 0.500907601 | -3.152232824 | 3.344618272 | 0.000892676 |
| AC018804.1 | 0.330346319 | 0.295630189 | 3.344179643 | 0.000894054 |
| AMIGO2 | 0.451653206 | 1.827825178 | 3.343274205 | 0.000896905 |
| ROCK1P1 | 0.2643771 | 1.622858521 | 3.343191526 | 0.000897166 |
| TPT1P4 | -0.310809491 | 7.091676363 | -3.343145757 | 0.00089731 |
| SOHLH2 | -0.495320745 | -1.69529092 | -3.342620484 | 0.000898969 |
| ANAPC1 | -0.192240445 | 4.322410426 | -3.342128594 | 0.000900524 |
| ORAI1 | -0.247879505 | 7.027024823 | -3.341755973 | 0.000901704 |
| RP11-331N16.1 | 0.380854476 | 1.197549386 | 3.341469669 | 0.000902612 |
| N4BP1 | 0.164481999 | 5.450792052 | 3.341454887 | 0.000902659 |
| LAMTOR1 | -0.137147538 | 7.489803919 | -3.341270348 | 0.000903245 |
| MRPS24 | 0.27567178 | 0.372268349 | 3.340142942 | 0.00090683 |
| EFCAB11 | -0.2672862 | 1.190396786 | -3.339727168 | 0.000908155 |
| RP11-305L7.1 | -0.45769461 | 0.156746743 | -3.338782855 | 0.000911173 |
| CTD-3092A11.1 | 0.215946881 | 4.363634973 | 3.338762104 | 0.000911239 |
| POLR1A | -0.202796205 | 4.661107432 | -3.338679968 | 0.000911502 |
| RHOBTB1 | -0.649683803 | 2.78166727 | -3.338286342 | 0.000912763 |
| IL33 | 0.452747479 | -2.890910488 | 3.338276493 | 0.000912794 |
| PTGDR | -0.496526434 | 0.951017797 | -3.338105567 | 0.000913342 |
| AMZ2 | 0.156332288 | 5.468951579 | 3.338085836 | 0.000913406 |
| HTR1F | 0.92420695 | 2.373507477 | 3.337841983 | 0.000914188 |
| RRAGC | 0.153596053 | 5.132465305 | 3.337653512 | 0.000914793 |
| LGALS9 | -0.25026337 | 6.518172631 | -3.337564946 | 0.000915078 |
| TMEM37 | -0.560672141 | -0.779206307 | -3.337556722 | 0.000915104 |
| AL139377.1 | -0.392546995 | 0.307567156 | -3.33701644 | 0.000916842 |
| TRAV13-1 | -0.507713643 | 0.995106864 | -3.336897302 | 0.000917225 |
| COL7A1 | 0.362437781 | 0.068282481 | 3.336877652 | 0.000917289 |
| RP11-2H3.6 | -0.422267351 | 0.111070526 | -3.336672113 | 0.000917951 |
| LPHN1 | 0.482874311 | 2.707544709 | 3.336015548 | 0.000920069 |
| IL1RL1 | 0.785722728 | 1.713872468 | 3.335949261 | 0.000920283 |
| RP11-248J18.2 | 0.305591418 | 2.094565322 | 3.335271505 | 0.000922474 |
| RNU7-40P | 0.387955378 | 1.068314592 | 3.335121711 | 0.000922959 |
| SH3RF1 | 0.29838497 | 3.939332111 | 3.335072434 | 0.000923119 |
| ADAMTS2 | -1.045485017 | -0.399837301 | -3.335030002 | 0.000923256 |
| MYLK | 0.645878756 | 0.390289643 | 3.334843986 | 0.000923859 |
| NFATC4 | 0.721756583 | -3.228800008 | 3.33473099 | 0.000924225 |
| UBASH3B | 0.344335935 | 5.405092627 | 3.334534468 | 0.000924863 |
| LRRC20 | -0.362122058 | 2.773693189 | -3.334410614 | 0.000925265 |
| TMEM163 | 0.673807846 | -1.405557433 | 3.334335879 | 0.000925507 |
| ZBTB41 | 0.134673335 | 3.952574588 | 3.334137382 | 0.000926152 |
| RP11-4M23.2 | -0.420393683 | -0.331321332 | -3.334088796 | 0.00092631 |
| RP11-266L9.1 | -0.37088352 | -2.223118204 | -3.333479629 | 0.000928291 |
| ZNF682 | 0.461204965 | 1.346413141 | 3.333461607 | 0.00092835 |
| RP11-5L12.1 | 0.435173842 | -0.429401719 | 3.333378348 | 0.000928621 |
| TTC3 | -0.209629684 | 6.585637229 | -3.332778442 | 0.000930577 |
| FAM166B | -0.571517303 | -0.311180203 | -3.332617416 | 0.000931103 |
| ATP5J2-PTCD1 | -0.364958902 | -2.087534387 | -3.33252439 | 0.000931406 |
| UBE2Z | -0.098345806 | 6.490073116 | -3.332049238 | 0.00093296 |
| DMC1 | 0.464711833 | 0.375460645 | 3.331870672 | 0.000933544 |
| PTBP1P | 0.273511803 | 1.549367899 | 3.331687859 | 0.000934142 |
| BDNF | 0.678671973 | -3.679350899 | 3.331343568 | 0.000935271 |
| NOS1AP | 0.606187079 | -1.458572604 | 3.331280236 | 0.000935478 |
| RP11-16E23.3 | 0.42263252 | 0.726875693 | 3.330750029 | 0.000937218 |
| DFNA5 | 0.693482713 | -1.749564147 | 3.330639794 | 0.000937581 |
| POLR3G | -0.28618925 | 1.952121493 | -3.330568241 | 0.000937816 |
| PKD1P5 | 0.315245161 | 2.582381038 | 3.330511325 | 0.000938003 |
| BUB1 | 0.369044686 | 3.791704084 | 3.330084109 | 0.000939408 |
| TPT1-AS1 | -0.210441754 | 3.439408632 | -3.329678416 | 0.000940745 |
| RP3-508I15.18 | 0.3111047 | 0.664920926 | 3.329054814 | 0.000942803 |
| TMIE | -0.490052205 | -0.422610815 | -3.328577453 | 0.000944381 |
| MINOS1 | 0.164504329 | 2.646505236 | 3.328545366 | 0.000944487 |
| PPP1R9B | -0.180635046 | 7.819639301 | -3.328362417 | 0.000945093 |
| RAI14 | 0.720451404 | -1.663163154 | 3.328000557 | 0.000946291 |
| RNF13 | -0.207784755 | 5.934987094 | -3.327393253 | 0.000948306 |
| FDXR | -0.292369669 | 3.281204843 | -3.32713042 | 0.000949179 |
| GPR75 | 0.232451626 | 2.782190529 | 3.326867193 | 0.000950055 |
| ZNF362 | 0.154763717 | 5.308157117 | 3.326605273 | 0.000950927 |
| DNAJC21 | 0.095102388 | 5.338575361 | 3.326365176 | 0.000951726 |
| ARL17A | -0.28204956 | 1.804374554 | -3.325551091 | 0.000954443 |
| MT-TL2 | 0.40156339 | 0.782910918 | 3.325248874 | 0.000955453 |
| KEL | 0.776767243 | 1.787943284 | 3.324239512 | 0.000958834 |
| RP11-638I8.1 | -0.278945001 | 2.287408263 | -3.323151973 | 0.00096249 |
| HIF1AN | -0.108271201 | 5.260263728 | -3.323054789 | 0.000962817 |
| ZCCHC5 | -0.568029642 | -2.219775784 | -3.323040177 | 0.000962866 |
| CNNM3 | -0.15957561 | 4.753314399 | -3.322318806 | 0.000965299 |
| TRAV8-5 | -0.490597086 | 0.51238926 | -3.321198231 | 0.00096909 |
| ZSCAN10 | 0.424600701 | -2.286509159 | 3.320992713 | 0.000969786 |
| RP11-108M9.4 | -0.358796181 | 3.184957626 | -3.32094861 | 0.000969936 |
| ALDH3A2 | -0.354869495 | 4.190582903 | -3.320284035 | 0.000972192 |
| EVA1B | -0.30514435 | 6.00282904 | -3.320141055 | 0.000972678 |
| RP11-506M12.1 | -0.357147047 | -0.219571065 | -3.319853596 | 0.000973656 |
| RN7SL566P | 0.376158643 | -0.024470143 | 3.319729862 | 0.000974077 |
| KIF16B | -0.421420153 | 3.280471553 | -3.319160759 | 0.000976017 |
| PAAF1 | -0.166969393 | 3.867576622 | -3.31915531 | 0.000976036 |
| MEIG1 | -0.396528658 | -1.177776357 | -3.318685122 | 0.000977641 |
| YEATS2-AS1 | -0.347140929 | -1.177462964 | -3.318634848 | 0.000977813 |
| MPP6 | 0.423914449 | 2.389122482 | 3.318418235 | 0.000978553 |
| SCARNA3 | 0.357185712 | 0.482346099 | 3.318085352 | 0.000979692 |
| LINC00996 | -0.61131311 | 1.510512541 | -3.317544055 | 0.000981547 |
| RP11-732A19.8 | 0.278907586 | 0.976790362 | 3.316963582 | 0.000983539 |
| AXIN1 | 0.130752062 | 5.637108604 | 3.316887895 | 0.000983799 |
| ARL4A | 0.338825173 | 5.75428377 | 3.316404845 | 0.00098546 |
| GS1-279B7.2 | 0.329004509 | 0.983251174 | 3.315964336 | 0.000986978 |
| ING5 | 0.182840631 | 4.205255139 | 3.315672701 | 0.000987983 |
| ALDH5A1 | -0.345514369 | 4.529647175 | -3.31540864 | 0.000988895 |
| RP11-77O7.1 | 0.383315539 | 0.446977402 | 3.315391145 | 0.000988955 |
| RP11-473N11.2 | 0.178704806 | 3.532563823 | 3.315381408 | 0.000988989 |
| STPG2 | -0.425083447 | -2.09711216 | -3.315027703 | 0.000990211 |
| RP11-449H11.1 | -0.280471818 | 1.779648218 | -3.314466272 | 0.000992154 |
| RN7SKP16 | 0.386679623 | 1.11337197 | 3.314418974 | 0.000992318 |
| CCDC106 | -0.244528094 | 4.248476846 | -3.314393474 | 0.000992406 |
| CTD-2666L21.1 | -0.371744941 | 0.460193626 | -3.314136793 | 0.000993296 |
| CHFR | 0.167993383 | 5.22244917 | 3.314071579 | 0.000993522 |
| RBM18 | 0.099053293 | 5.1420298 | 3.313714978 | 0.000994759 |
| RPL24P4 | -0.151400829 | 10.01531677 | -3.313703419 | 0.000994799 |
| NACAP3 | -0.341142377 | 0.062105214 | -3.313047106 | 0.000997081 |
| FAM89B | -0.197281258 | 4.468539157 | -3.31240939 | 0.000999302 |
| NSFP1 | -0.392568983 | 1.956535048 | -3.312401945 | 0.000999328 |
| RSL1D1 | -0.197492992 | 7.818226553 | -3.311683755 | 0.001001835 |
| BTC | -0.543803582 | -2.635503267 | -3.311382079 | 0.00100289 |
| RP11-144L1.8 | 0.270281998 | 1.816064254 | 3.311223762 | 0.001003444 |
| SYTL1 | -0.310661563 | 6.168370588 | -3.310593326 | 0.001005653 |
| RPL35 | 0.160412092 | 9.617717939 | 3.309800958 | 0.001008436 |
| HARS2 | -0.147765236 | 5.271485263 | -3.309722198 | 0.001008712 |
| NOLC1 | -0.213543377 | 7.30107825 | -3.309701316 | 0.001008786 |
| TSPAN2 | -0.518879337 | 5.203363494 | -3.309628421 | 0.001009042 |
| KIAA0040 | -0.338003969 | 5.120570212 | -3.309207224 | 0.001010525 |
| SOSTDC1 | 0.677838883 | -2.454184385 | 3.30852057 | 0.001012947 |
| SNHG16 | 0.175374665 | 5.80722535 | 3.3082809 | 0.001013794 |
| BX649553.4 | 0.42714534 | 0.26484597 | 3.308224141 | 0.001013994 |
| ANAPC11 | 0.166706796 | 4.334038734 | 3.308118671 | 0.001014367 |
| FZD4 | 0.493392943 | -2.018234043 | 3.307465834 | 0.001016678 |
| LL21NC02-1C16.1 | -0.370091346 | -0.444828009 | -3.307463444 | 0.001016686 |
| FST | 0.576090184 | -1.899811394 | 3.307079875 | 0.001018046 |
| SLC25A13 | 0.166464831 | 4.683230563 | 3.306748888 | 0.001019221 |
| TP53INP1 | 0.372269908 | 6.455851171 | 3.306240159 | 0.001021029 |
| FAM117B | 0.209715033 | 3.971787234 | 3.306100664 | 0.001021525 |
| PHKA2 | -0.149563647 | 5.439932246 | -3.305297659 | 0.001024387 |
| MRPL37 | 0.132621471 | 6.779969222 | 3.304983715 | 0.001025508 |
| TCEAL8 | -0.177260523 | 6.759177993 | -3.303522742 | 0.001030738 |
| RP11-109A6.3 | 0.37785541 | -0.063278792 | 3.303264903 | 0.001031664 |
| FAM53A | -0.281772181 | 0.263465306 | -3.301885017 | 0.001036631 |
| ADAMTS17 | 0.369796502 | -1.467532867 | 3.30144868 | 0.001038206 |
| RP11-114I8.4 | -0.312419383 | 1.639306431 | -3.300914368 | 0.001040138 |
| AP003419.16 | -0.388501554 | -0.063903706 | -3.300551875 | 0.00104145 |
| ACKR2 | 0.473393779 | -1.44569165 | 3.299657772 | 0.001044695 |
| RCCD1 | -0.244066467 | 3.588095207 | -3.299273302 | 0.001046093 |
| SLC37A1 | 0.258090645 | 5.076516644 | 3.299265 | 0.001046123 |
| AQP1 | 0.710151863 | 1.968059362 | 3.299227472 | 0.001046259 |
| RP11-347C18.3 | -0.35361312 | 1.862182798 | -3.298510874 | 0.00104887 |
| SH3GLB2 | 0.150304355 | 5.03188037 | 3.29813245 | 0.001050251 |
| RPS16P5 | 0.462985228 | 0.825034298 | 3.298047247 | 0.001050562 |
| MSL2 | 0.195457338 | 7.005994656 | 3.297810609 | 0.001051427 |
| GREM2 | 0.51070398 | -2.288131006 | 3.297618608 | 0.001052129 |
| OSBPL10 | -0.54327577 | -1.580663878 | -3.29704185 | 0.00105424 |
| MTG1 | -0.175502205 | 2.571092055 | -3.297005083 | 0.001054375 |
| POLE | 0.250827934 | 5.545369892 | 3.296434016 | 0.00105647 |
| PLA2G15 | -0.215918626 | 4.385210486 | -3.296332227 | 0.001056844 |
| SLC39A10 | -0.215697743 | 4.531633437 | -3.296234297 | 0.001057203 |
| KANSL1L | -0.259716663 | 3.785711253 | -3.296220487 | 0.001057254 |
| MYO1F | -0.268531783 | 7.754370687 | -3.296022822 | 0.001057981 |
| CRISP3 | 1.074013129 | 3.696368105 | 3.295997937 | 0.001058072 |
| RP4-755D9.1 | -0.635523875 | -1.764782077 | -3.29566372 | 0.001059302 |
| ARL17B | -0.354136068 | 0.628926747 | -3.29545604 | 0.001060066 |
| DNAJB11 | 0.126617074 | 6.4521564 | 3.295154843 | 0.001061176 |
| QTRTD1 | 0.121105665 | 4.180162031 | 3.294608983 | 0.00106319 |
| PI4K2B | 0.129025031 | 4.183585562 | 3.293584338 | 0.001066981 |
| GZMA | -0.529224241 | 4.339204023 | -3.293579415 | 0.001066999 |
| FKBP1A | -0.176386773 | 8.991257836 | -3.292446914 | 0.001071204 |
| PDE4DIP | -0.281466867 | 4.724260533 | -3.292370118 | 0.001071489 |
| RP11-367J7.4 | -0.333913833 | -0.194629499 | -3.292283374 | 0.001071812 |
| XX-C283C717.1 | -0.420148235 | 0.161963181 | -3.29157383 | 0.001074455 |
| NUDT19 | 0.212557147 | 4.423942567 | 3.290909019 | 0.001076937 |
| COPS4 | 0.094341396 | 5.359424809 | 3.290889795 | 0.001077009 |
| CTB-11I22.1 | 0.421665016 | -1.016607607 | 3.290211887 | 0.001079546 |
| INTS2 | -0.135598532 | 3.814726346 | -3.290185933 | 0.001079643 |
| UPF2 | -0.145455835 | 6.575399226 | -3.289814555 | 0.001081035 |
| ZNF98 | 0.674855794 | -2.526211284 | 3.288439954 | 0.001086203 |
| STAMBP | -0.100162654 | 4.7014191 | -3.287809856 | 0.001088579 |
| TNRC6C-AS1 | -0.287510448 | 4.481663269 | -3.287804107 | 0.001088601 |
| RNU4-1 | -0.461945181 | -0.080261629 | -3.287622723 | 0.001089286 |
| AC004985.12 | 0.310543046 | 0.374291795 | 3.287505149 | 0.00108973 |
| LAMB1 | 0.660916197 | -3.006008334 | 3.287386788 | 0.001090177 |
| TMEM53 | -0.293774897 | 2.433425322 | -3.287025782 | 0.001091543 |
| APP | 0.880004583 | 5.879843452 | 3.28663925 | 0.001093006 |
| LINC00202-2 | 0.480780024 | -3.155154242 | 3.286573878 | 0.001093254 |
| ZSCAN16 | -0.18744895 | 4.241560381 | -3.285516695 | 0.001097268 |
| GGTA1P | 0.810094085 | 2.112190963 | 3.284635061 | 0.001100626 |
| RP11-521B24.3 | -0.345706443 | 0.833178852 | -3.284372072 | 0.001101629 |
| HS3ST5 | 0.531441255 | -3.258019278 | 3.28394292 | 0.001103268 |
| TRGV5 | -0.613953496 | 1.938602284 | -3.283589677 | 0.001104619 |
| CLEC10A | -0.96454593 | 1.533129852 | -3.283158484 | 0.00110627 |
| RP3-497J21.1 | 0.41931228 | 0.066937097 | 3.282228385 | 0.00110984 |
| HOOK3 | -0.175845901 | 5.100176944 | -3.282124508 | 0.001110239 |
| LCNL1 | -0.567675208 | -1.823947945 | -3.281977611 | 0.001110804 |
| PGRMC1 | 0.153044602 | 6.590516401 | 3.280854997 | 0.00111513 |
| PSENEN | -0.176654013 | 4.146078914 | -3.280742379 | 0.001115565 |
| TMEM180 | -0.232744254 | 3.313894267 | -3.279139493 | 0.001121771 |
| TRAV8-1 | -0.43054832 | -0.415668832 | -3.279021182 | 0.00112223 |
| ABL2 | 0.145074984 | 4.320470617 | 3.278842581 | 0.001122924 |
| LINC00571 | -0.404961375 | -0.841977444 | -3.278201427 | 0.001125418 |
| RP11-133K1.1 | 0.282228659 | 3.121966619 | 3.278073521 | 0.001125916 |
| NOB1 | -0.187416037 | 6.821874918 | -3.277669014 | 0.001127493 |
| PCYT2 | -0.168659154 | 4.11814222 | -3.276740768 | 0.001131118 |
| LINC00570 | 0.547013694 | -1.843278081 | 3.27674049 | 0.001131119 |
| MTRR | 0.12657834 | 5.201328762 | 3.276543078 | 0.001131892 |
| GPR89B | 0.185243994 | 1.73649671 | 3.276357169 | 0.00113262 |
| DPH5 | -0.190610621 | 5.11751299 | -3.275958372 | 0.001134183 |
| FAM211B | 0.406007663 | 2.184624773 | 3.275830669 | 0.001134684 |
| HNRNPA1P50 | 0.287570661 | 1.18025172 | 3.27551773 | 0.001135912 |
| AP001062.7 | 0.255697738 | 1.84787583 | 3.275293788 | 0.001136792 |
| RP11-521I2.3 | -0.331543521 | 0.530844443 | -3.275220439 | 0.00113708 |
| ZNF597 | 0.329460437 | 3.709225775 | 3.275147717 | 0.001137366 |
| ADD1 | -0.094401584 | 7.10318945 | -3.274481859 | 0.001139987 |
| RP11-108P20.1 | -0.306620179 | 2.078187673 | -3.274054142 | 0.001141673 |
| UHMK1 | 0.114735614 | 6.472919658 | 3.273682789 | 0.001143139 |
| RP3-449O17.1 | -0.30684317 | 2.427134276 | -3.273572647 | 0.001143574 |
| FPGS | 0.192007933 | 6.419963168 | 3.273396872 | 0.001144269 |
| PLEKHF1 | 0.330131963 | 2.785719023 | 3.272484103 | 0.001147884 |
| CC2D1A | -0.127068055 | 4.986599282 | -3.272056627 | 0.00114958 |
| CAMSAP1 | 0.130146777 | 4.8567877 | 3.271189239 | 0.001153029 |
| ZNF726 | 0.360163168 | 2.011219594 | 3.271139613 | 0.001153227 |
| CCNF | 0.362824648 | 2.716810425 | 3.270537189 | 0.001155628 |
| PAPOLG | -0.150526421 | 4.099292578 | -3.270017718 | 0.001157703 |
| GPR116 | 0.478209177 | -4.581957093 | 3.269758567 | 0.001158739 |
| ATP6V1H | 0.124686395 | 4.790720676 | 3.268846347 | 0.001162394 |
| RP11-304F15.5 | -0.297172946 | 1.894222069 | -3.268496365 | 0.001163798 |
| PHB1P3 | -0.283942264 | 1.450595415 | -3.26806676 | 0.001165525 |
| PTGER4 | 0.353715292 | 7.272800753 | 3.267964688 | 0.001165936 |
| WBP1 | 0.190060351 | 4.788864643 | 3.267962838 | 0.001165943 |
| ACSBG1 | 0.533255147 | -1.098600015 | 3.267402128 | 0.001168201 |
| MYT1 | 0.626166238 | -2.058810485 | 3.26728645 | 0.001168667 |
| XXbac-B444P24.8 | -0.870767418 | -0.653785485 | -3.266866965 | 0.00117036 |
| GAPDHP67 | 0.357030482 | 0.445692355 | 3.266371256 | 0.001172363 |
| ELOF1 | 0.111188814 | 4.730847588 | 3.266103689 | 0.001173445 |
| LINC00431 | -0.581888289 | -0.39902877 | -3.26579638 | 0.00117469 |
| 1-Mar | -0.670727127 | 3.130313915 | -3.265596067 | 0.001175502 |
| PPIHP1 | 0.20759506 | 4.48970523 | 3.265045407 | 0.001177736 |
| KCNN1 | 0.599259746 | -2.613220887 | 3.264981216 | 0.001177997 |
| SPTB | 0.903989605 | 0.979288439 | 3.264288489 | 0.001180814 |
| GDF10 | 0.595767861 | -1.766124413 | 3.264144539 | 0.0011814 |
| ZFP92 | 0.467395541 | 1.788854988 | 3.263873921 | 0.001182502 |
| PIDD | 0.145041442 | 4.790849762 | 3.263785954 | 0.001182861 |
| MTHFD2 | 0.230037722 | 5.620886272 | 3.263442118 | 0.001184264 |
| BEND6 | -0.788938744 | -0.319904845 | -3.262974669 | 0.001186173 |
| RP11-47A8.5 | 0.295650438 | 2.038138729 | 3.262177413 | 0.001189437 |
| MDFI | -0.949839024 | 2.113605109 | -3.262053409 | 0.001189945 |
| WFDC2 | -0.415685396 | -1.399179935 | -3.261865703 | 0.001190715 |
| MSANTD4 | -0.184280593 | 3.641994195 | -3.261823287 | 0.001190889 |
| SHC4 | -0.401651009 | -2.839481443 | -3.261765214 | 0.001191127 |
| INPP5F | 0.163723726 | 3.947375882 | 3.261501546 | 0.00119221 |
| RPS17 | 0.418009653 | 5.514654943 | 3.260434678 | 0.001196599 |
| BRI3 | 0.277601658 | 6.498175965 | 3.260106496 | 0.001197952 |
| RP1-145M24.1 | 0.216237692 | 1.025983647 | 3.259944519 | 0.001198621 |
| TMEM105 | -0.865348108 | -0.80790195 | -3.259728808 | 0.001199511 |
| LPPR3 | -0.775073386 | 5.782381245 | -3.259362304 | 0.001201026 |
| EIF4BP2 | 0.252528913 | 0.757093236 | 3.259044378 | 0.001202342 |
| TMEM9B | 0.164311067 | 6.020879466 | 3.259020133 | 0.001202442 |
| MYO15B | -0.206000632 | 7.430941514 | -3.258949897 | 0.001202733 |
| ZNF595 | 0.789550856 | 0.65069314 | 3.258564509 | 0.00120433 |
| ZNF471 | -0.531382733 | 1.331130395 | -3.258474415 | 0.001204703 |
| CTD-2528L19.3 | 0.29474657 | 1.213195069 | 3.257609119 | 0.001208297 |
| RP3-400N23.6 | 0.379841706 | 1.616872378 | 3.25754372 | 0.001208569 |
| SUB1P1 | 0.23640189 | 3.392522808 | 3.257310247 | 0.00120954 |
| GRB10 | -0.419069601 | 4.215751024 | -3.256964173 | 0.001210982 |
| C20orf112 | 0.233079602 | 5.354076149 | 3.256893592 | 0.001211276 |
| PEX3 | -0.161208888 | 4.540802519 | -3.256875345 | 0.001211352 |
| GMPS | 0.136937403 | 5.656361718 | 3.2565976 | 0.00121251 |
| AP4M1 | -0.167966731 | 3.485276653 | -3.256403148 | 0.001213322 |
| PCSK7 | 0.144006022 | 5.088783871 | 3.255569551 | 0.001216806 |
| MIR371B | -0.533800542 | 0.518510814 | -3.255413889 | 0.001217458 |
| LYSMD4 | 0.220875648 | 2.469473458 | 3.255335424 | 0.001217787 |
| CHMP1A | 0.099085891 | 6.590525425 | 3.254904108 | 0.001219595 |
| NAT10 | -0.1786477 | 6.105880292 | -3.254467977 | 0.001221425 |
| AC137932.6 | 0.407229522 | -1.909129091 | 3.254176526 | 0.00122265 |
| FICD | 0.203094932 | 1.806907093 | 3.253434173 | 0.001225775 |
| DTNBP1 | -0.126292763 | 5.06390132 | -3.253344431 | 0.001226153 |
| PPFIA1 | 0.149507103 | 4.912101151 | 3.252807801 | 0.001228417 |
| RP11-346I3.4 | -0.320732693 | -1.950660493 | -3.252514329 | 0.001229657 |
| RAB11A | -0.107512184 | 6.581921314 | -3.252452667 | 0.001229918 |
| RP11-889L3.1 | -0.132640297 | 9.998437966 | -3.252021361 | 0.001231742 |
| SAMD1 | -0.178386838 | 6.55813204 | -3.251876514 | 0.001232356 |
| TDRD12 | -0.487850692 | -3.159769947 | -3.251833123 | 0.00123254 |
| AF213884.2 | -0.37128714 | -0.047074303 | -3.251608318 | 0.001233492 |
| MSH4 | -0.378877692 | -0.935015228 | -3.251224246 | 0.001235121 |
| RP11-330M2.4 | 0.295987153 | 2.189697948 | 3.250787143 | 0.001236978 |
| CMTR1 | 0.110495791 | 5.933170255 | 3.250782868 | 0.001236996 |
| TTLL6 | -0.785741148 | -1.108179296 | -3.250685497 | 0.00123741 |
| TTC18 | -0.31740139 | 1.071636821 | -3.250136395 | 0.001239746 |
| ZDHHC1 | -0.359432461 | 2.265441412 | -3.250093821 | 0.001239927 |
| CTC-329D1.2 | -0.187806646 | 3.114940153 | -3.250028291 | 0.001240207 |
| OLFML2A | -0.767284402 | 1.425281999 | -3.250013702 | 0.001240269 |
| RP11-112J1.2 | -0.536710527 | -1.411707911 | -3.249811483 | 0.001241131 |
| RC3H2 | 0.119102023 | 5.266677092 | 3.249794001 | 0.001241205 |
| NMU | 0.646604227 | -0.759722575 | 3.249228599 | 0.001243618 |
| MIR3690 | 0.433403984 | 0.100665493 | 3.24881079 | 0.001245404 |
| RP11-547L9.1 | -0.384498546 | -0.099124387 | -3.248774394 | 0.001245559 |
| TECPR2 | -0.19354939 | 4.0156754 | -3.248619643 | 0.001246221 |
| XXbac-BPG181B23.6 | -0.453464824 | -0.958962931 | -3.24860504 | 0.001246284 |
| ZNF253 | -0.235999286 | 3.887769238 | -3.248252898 | 0.001247792 |
| AGAP8 | -0.303673261 | 0.916477964 | -3.24786516 | 0.001249454 |
| IL2RG | -0.321888671 | 8.132452897 | -3.247751875 | 0.00124994 |
| CAMK2N1 | 0.444609415 | -0.572764953 | 3.247554606 | 0.001250787 |
| APOBR | -0.323869062 | 7.550657198 | -3.247317709 | 0.001251805 |
| NR2F2 | 0.504438931 | -4.168706352 | 3.247279451 | 0.001251969 |
| ANKRD20A11P | 0.600137157 | -1.950678264 | 3.247232606 | 0.00125217 |
| MRPL20 | -0.098358127 | 5.803571044 | -3.24655425 | 0.001255089 |
| SLC16A7 | -0.290619499 | 3.419930548 | -3.246209988 | 0.001256573 |
| MMP15 | -0.644905513 | 1.259392607 | -3.246121404 | 0.001256955 |
| ENPEP | 0.754721272 | -3.641801845 | 3.245832192 | 0.001258203 |
| ALOX12 | 0.746182144 | -0.038874328 | 3.245348131 | 0.001260295 |
| AC006277.2 | -0.295777781 | 1.184209218 | -3.245169759 | 0.001261066 |
| GRTP1 | 0.622186972 | -1.449213686 | 3.244584603 | 0.0012636 |
| GGA2 | 0.237214699 | 6.031724763 | 3.244516473 | 0.001263896 |
| SLC20A1 | 0.167398476 | 6.334909487 | 3.244441984 | 0.001264219 |
| TDRP | 0.436489395 | -3.162208412 | 3.244016474 | 0.001266065 |
| TPST1 | 0.572193838 | 2.18185176 | 3.243951354 | 0.001266348 |
| CSNK1A1P3 | -0.376122284 | 0.402540889 | -3.243148927 | 0.001269838 |
| RP11-488L18.6 | -0.483335335 | 0.924175366 | -3.24224002 | 0.001273802 |
| CTC-378H22.2 | -0.479713186 | 2.642514485 | -3.242223482 | 0.001273874 |
| TPT1P9 | -0.285094407 | 8.218443449 | -3.241852777 | 0.001275494 |
| PBX1 | 0.803171024 | -1.760883496 | 3.241532667 | 0.001276894 |
| SEPT7P9 | -0.31110698 | 0.35223717 | -3.240619467 | 0.001280897 |
| TMEM50A | -0.08977525 | 7.372373153 | -3.240586629 | 0.001281041 |
| RP11-271K11.1 | -0.598354387 | 0.450651829 | -3.240581499 | 0.001281064 |
| ZFPM2 | 0.625022578 | -3.249198228 | 3.23994928 | 0.001283842 |
| AF196972.4 | 0.335597108 | -0.23188135 | 3.239695717 | 0.001284958 |
| RP11-69L16.4 | -0.45186051 | -0.097737337 | -3.239531876 | 0.001285679 |
| HLX | 0.405645486 | 5.524971802 | 3.238939058 | 0.001288293 |
| AGAP10 | -0.397917298 | -1.79154401 | -3.238124181 | 0.001291894 |
| CBX8 | -0.304978044 | 1.881514688 | -3.237916837 | 0.001292812 |
| PHEX | 0.458048387 | -2.118775364 | 3.237622492 | 0.001294115 |
| NDUFAF4P3 | -0.386011603 | -0.991972372 | -3.23761545 | 0.001294147 |
| TEPP | -0.474613063 | -1.969247395 | -3.237558005 | 0.001294401 |
| TNXA | 0.45807949 | -1.71092438 | 3.237351678 | 0.001295316 |
| TMEM219 | -0.128413735 | 6.932966059 | -3.237333016 | 0.001295399 |
| JAG1 | 0.549203942 | 4.558775181 | 3.236942958 | 0.00129713 |
| SMURF2 | 0.147186902 | 4.787082904 | 3.236753807 | 0.00129797 |
| RPL4P2 | 0.285171637 | 1.15132448 | 3.236680009 | 0.001298298 |
| RP3-352A20.1 | -0.276108435 | 0.21162558 | -3.236640017 | 0.001298476 |
| DOPEY2 | -0.186443561 | 5.288277428 | -3.236438919 | 0.00129937 |
| RP5-1139B12.2 | -0.468190109 | -0.440103704 | -3.235857559 | 0.001301959 |
| RP11-1105G2.4 | -0.357532602 | -0.592558267 | -3.235790172 | 0.001302259 |
| TMEM184B | 0.171652341 | 4.759318698 | 3.235760752 | 0.00130239 |
| CEP164 | -0.129961398 | 4.125550671 | -3.235491423 | 0.001303591 |
| CIITA | -0.475471306 | 5.416427047 | -3.235077206 | 0.001305441 |
| MRPL55 | 0.13796024 | 5.679729899 | 3.234988833 | 0.001305836 |
| C9orf169 | 0.36941347 | -0.63184831 | 3.234384791 | 0.001308537 |
| CDKN2AIPNL | -0.150654837 | 5.131105471 | -3.234207618 | 0.001309331 |
| CHD6 | 0.142059593 | 5.2844171 | 3.234089924 | 0.001309858 |
| MTMR1 | 0.150139454 | 4.748054103 | 3.233463201 | 0.00131267 |
| TESC | 0.478386105 | 5.73917347 | 3.231873086 | 0.001319828 |
| LINC00116 | -0.269516906 | 2.809326287 | -3.231514798 | 0.001321446 |
| CTD-2540F13.2 | -0.464326833 | -0.003415211 | -3.231324496 | 0.001322306 |
| KIAA0825 | -0.287153939 | 0.742333419 | -3.23117134 | 0.001322998 |
| VAT1 | -0.317404241 | 7.632566408 | -3.230959964 | 0.001323955 |
| ST3GAL6 | -0.362383749 | 3.737384369 | -3.230549939 | 0.001325812 |
| RP11-197N18.2 | -0.340515019 | -2.690314649 | -3.230490669 | 0.00132608 |
| WSB2 | 0.227323089 | 5.796700539 | 3.23033002 | 0.001326809 |
| RP11-416K24.2 | 0.25788256 | 5.021138078 | 3.229882317 | 0.001328841 |
| SRR | -0.221717963 | 3.203284605 | -3.229644557 | 0.001329921 |
| RP11-385F7.1 | 0.333367308 | 0.750668752 | 3.229294208 | 0.001331514 |
| CST3 | -0.470475991 | 8.231868302 | -3.228962391 | 0.001333025 |
| SPTLC3 | -0.715890032 | 0.307621491 | -3.228931603 | 0.001333165 |
| RPL3P1 | 0.32420577 | 1.477502013 | 3.22815478 | 0.001336708 |
| RP11-53B2.2 | -0.363804297 | 0.718620189 | -3.227538237 | 0.001339526 |
| USP51 | 0.285487506 | 1.501881895 | 3.227516949 | 0.001339624 |
| C1orf109 | -0.185834646 | 3.339311652 | -3.226826628 | 0.001342786 |
| NEU3 | 0.162974061 | 4.389774959 | 3.225701346 | 0.001347955 |
| Z69720.2 | 0.38574342 | -0.104415539 | 3.225404801 | 0.00134932 |
| RABAC1 | -0.151722751 | 6.104144484 | -3.224631108 | 0.001352889 |
| HIST2H2BE | -0.399451438 | 8.28147291 | -3.22440181 | 0.001353948 |
| RP11-734E19.1 | 0.170782305 | 2.70777369 | 3.224269559 | 0.001354559 |
| HINT2 | 0.161884967 | 4.202783861 | 3.223478627 | 0.00135822 |
| TBC1D16 | -0.50754802 | 2.081275026 | -3.223075208 | 0.001360091 |
| RP11-163E9.2 | -0.299406067 | 1.364605587 | -3.223037787 | 0.001360264 |
| RP11-517C16.2 | -0.347845052 | -1.179088553 | -3.222300774 | 0.001363689 |
| PSAT1 | 0.546223468 | 1.817300707 | 3.222169499 | 0.0013643 |
| UCA1 | 0.691393707 | -1.062304414 | 3.222128791 | 0.001364489 |
| RP11-571M6.7 | 0.331396012 | 0.638693603 | 3.222107369 | 0.001364589 |
| RGPD1 | 0.525533082 | -0.703460484 | 3.221839844 | 0.001365835 |
| AGPS | -0.168850571 | 6.03488276 | -3.221686014 | 0.001366551 |
| SP7 | 0.584096354 | -2.624017054 | 3.221362014 | 0.001368062 |
| ACY1 | -0.30892345 | -0.349604786 | -3.221175371 | 0.001368933 |
| PIGHP1 | 0.212100982 | 1.706596486 | 3.220792938 | 0.00137072 |
| RP4-740C4.7 | -0.317514512 | 1.333361134 | -3.220318332 | 0.00137294 |
| NHSL1 | -0.357973877 | 1.390719125 | -3.219606556 | 0.001376275 |
| RNU5E-1 | -0.497814163 | 1.219638319 | -3.219029249 | 0.001378986 |
| SYNC | -0.510315304 | -0.806118271 | -3.218992097 | 0.001379161 |
| TARSL2 | -0.235226868 | 4.523396485 | -3.218785186 | 0.001380133 |
| HPS4 | 0.177698551 | 5.301100535 | 3.218495414 | 0.001381497 |
| RP11-47I22.2 | 0.433971082 | -1.795393047 | 3.218417206 | 0.001381865 |
| MBD1 | 0.086275393 | 6.233486539 | 3.218068605 | 0.001383508 |
| CD68 | 0.471812846 | 0.352727529 | 3.21791409 | 0.001384236 |
| TRDV2 | -0.516797897 | -0.162942294 | -3.217582288 | 0.001385802 |
| NR2F6 | -0.230088674 | 4.257773396 | -3.21755788 | 0.001385917 |
| RP11-473M20.9 | -0.416557118 | 4.774727046 | -3.217138077 | 0.001387901 |
| DUSP27 | -0.914749359 | -0.447810499 | -3.216438885 | 0.00139121 |
| REEP5 | -0.32493104 | 5.519602034 | -3.216412686 | 0.001391335 |
| ADH5P4 | -0.138319357 | 4.296131998 | -3.215698009 | 0.001394725 |
| CEP44 | 0.124818418 | 3.446140851 | 3.215181085 | 0.001397182 |
| NCK1 | 0.129809952 | 5.664523265 | 3.214984748 | 0.001398117 |
| AP5S1 | -0.125947209 | 4.565534326 | -3.214734863 | 0.001399307 |
| LAT2 | -0.310623554 | 7.433691302 | -3.214477064 | 0.001400535 |
| NXPE3 | -0.201007449 | 5.178611788 | -3.213984051 | 0.001402888 |
| C6orf203 | -0.138394403 | 4.309723883 | -3.213925416 | 0.001403168 |
| PYGO1 | 0.622805843 | -3.392839577 | 3.213920048 | 0.001403193 |
| TMEM39B | -0.129079633 | 4.693697018 | -3.213752613 | 0.001403993 |
| IGSF6 | -0.713345551 | 4.444363276 | -3.212403095 | 0.001410456 |
| MFHAS1 | 0.225321567 | 4.575819717 | 3.212250859 | 0.001411187 |
| RP11-803D5.4 | -0.422425286 | -0.858193687 | -3.212155087 | 0.001411647 |
| NRSN2 | 0.429191988 | 2.595683351 | 3.211933807 | 0.00141271 |
| SMYD5 | 0.191242101 | 4.834623616 | 3.211866757 | 0.001413032 |
| NUTM2E | 0.452407865 | -1.750803375 | 3.21175659 | 0.001413562 |
| RPS10L | 0.265082546 | 1.344433523 | 3.211749439 | 0.001413596 |
| PELO | 0.245821088 | 3.588718795 | 3.211444937 | 0.001415061 |
| RP11-439E19.9 | -0.345271053 | 0.587355885 | -3.21118546 | 0.001416311 |
| EIF5AP3 | 0.365981986 | -0.011300287 | 3.210658677 | 0.001418851 |
| ZFP57 | -1.067077772 | -0.738560346 | -3.209974669 | 0.001422155 |
| CEBPB | 0.41551748 | 8.049679534 | 3.209049342 | 0.001426636 |
| CSNK1G2 | 0.116284226 | 7.388874215 | 3.208901745 | 0.001427353 |
| UXT-AS1 | -0.288280663 | 0.149694294 | -3.208482627 | 0.001429388 |
| PPIL1 | -0.280732616 | 5.031718863 | -3.208042117 | 0.001431529 |
| TBC1D10A | 0.222974248 | 2.629817173 | 3.207669959 | 0.001433341 |
| RPP21 | 0.243437772 | 2.283474306 | 3.207339617 | 0.001434951 |
| PMP22 | 0.750573854 | 1.168480395 | 3.207027786 | 0.001436472 |
| COL17A1 | 0.919744926 | 1.339917918 | 3.206949194 | 0.001436856 |
| NACAD | 0.515127204 | -2.119582497 | 3.206604111 | 0.001438542 |
| RNF187 | 0.125676392 | 6.732375615 | 3.206220022 | 0.00144042 |
| CD28 | 0.539886688 | 2.187521245 | 3.205402167 | 0.001444427 |
| THEMIS | -0.506677238 | 1.167364127 | -3.205029923 | 0.001446254 |
| BAZ2B | -0.153225429 | 5.800246672 | -3.204935621 | 0.001446718 |
| LMNB1 | -0.275799113 | 7.307872875 | -3.204706478 | 0.001447844 |
| MAP3K1 | -0.227814988 | 6.369632641 | -3.204695444 | 0.001447898 |
| CPNE9 | 0.297234495 | 0.039539826 | 3.204669785 | 0.001448024 |
| PPEF1 | 0.472484986 | -1.831767946 | 3.204658891 | 0.001448078 |
| RP11-179G5.1 | 0.212522247 | 3.195864172 | 3.20447527 | 0.001448981 |
| C6orf164 | -0.36090105 | 0.7627824 | -3.204146094 | 0.001450602 |
| CTC-338M12.9 | -0.377207091 | -0.598261079 | -3.202523437 | 0.001458614 |
| RP11-51J9.5 | 0.284267952 | 3.966973533 | 3.202269049 | 0.001459874 |
| UBR4 | 0.142047957 | 7.195423916 | 3.202248154 | 0.001459977 |
| RP4-717I23.3 | -0.270250781 | 5.437780172 | -3.201985641 | 0.001461279 |
| EVC2 | 0.688821944 | -1.761385079 | 3.201969431 | 0.001461359 |
| PAWR | 0.799600218 | 0.478834524 | 3.201741352 | 0.001462491 |
| SNORA55 | 0.278426647 | 2.81187021 | 3.201702533 | 0.001462683 |
| RNU4-9P | 0.41191517 | -0.028497735 | 3.201513072 | 0.001463624 |
| ZNF709 | 0.407954526 | -1.559134632 | 3.201493684 | 0.00146372 |
| CTC-786C10.1 | 0.424754631 | -1.276630742 | 3.201096136 | 0.001465696 |
| EDC4 | 0.100596467 | 6.035187288 | 3.201029224 | 0.001466029 |
| LRWD1 | 0.202127478 | 3.820401863 | 3.200805822 | 0.00146714 |
| KIAA0391 | 0.263793089 | 0.058375871 | 3.200182399 | 0.001470246 |
| ARPC2 | 0.123156914 | 8.820268498 | 3.200124258 | 0.001470536 |
| BEND4 | 0.85513049 | 0.82549469 | 3.200035564 | 0.001470978 |
| RP11-493L12.4 | -0.404582568 | -0.396917 | -3.199737792 | 0.001472465 |
| RP4-676J13.2 | 0.363990221 | 0.341510506 | 3.199407996 | 0.001474113 |
| CLDN5 | 0.585891765 | -1.117398986 | 3.199324768 | 0.001474529 |
| CTB-51J22.1 | -0.504465701 | -1.205098927 | -3.198645188 | 0.001477931 |
| TNN | -0.665111948 | -2.583913524 | -3.198485544 | 0.001478731 |
| RACGAP1 | 0.292424042 | 4.526280663 | 3.198415016 | 0.001479084 |
| ITFG2 | -0.128585859 | 4.920779099 | -3.198330505 | 0.001479508 |
| RP11-159G9.5 | 0.186150004 | 3.793404297 | 3.198277267 | 0.001479775 |
| MYO7B | -0.575559232 | 1.845779791 | -3.198110735 | 0.001480611 |
| PHB1P7 | -0.310490022 | 0.144250884 | -3.198073461 | 0.001480798 |
| TIMELESS | 0.279948714 | 5.472260105 | 3.19804753 | 0.001480928 |
| KRTCAP3 | 0.240021406 | 2.592585161 | 3.198022255 | 0.001481055 |
| RP5-1021I20.1 | -0.30228394 | 2.841854975 | -3.197655023 | 0.0014829 |
| SHC2 | 0.419467059 | -3.379597471 | 3.197323274 | 0.001484569 |
| ADD3 | -0.242135606 | 7.212548453 | -3.196973829 | 0.001486328 |
| RPP38 | -0.149029364 | 4.672889116 | -3.196942455 | 0.001486486 |
| TTLL9 | -0.43277086 | -1.581204762 | -3.196905634 | 0.001486671 |
| NLRC4 | -0.623477916 | 3.435024468 | -3.196352917 | 0.001489459 |
| LUCAT1 | 0.651129892 | 0.845592323 | 3.196092955 | 0.001490771 |
| TARDBP | -0.133082298 | 7.433159352 | -3.195945141 | 0.001491518 |
| RP11-925D8.3 | 0.532938487 | -1.027957199 | 3.195583393 | 0.001493347 |
| MPDZ | 0.737234173 | -4.506472557 | 3.195567456 | 0.001493428 |
| FOXN3 | 0.129718163 | 6.613229435 | 3.195078942 | 0.001495902 |
| SAMD14 | 0.524038124 | 0.001879591 | 3.194901652 | 0.0014968 |
| RP5-906A24.2 | -0.469104536 | -0.561380509 | -3.193704879 | 0.001502879 |
| NEK3 | 0.219931091 | 4.344075845 | 3.193474699 | 0.001504051 |
| P4HTM | -0.155342523 | 3.977875202 | -3.193361286 | 0.001504629 |
| MIR3667HG | -0.349665167 | 2.415170092 | -3.193340274 | 0.001504736 |
| HSPE1P13 | 0.389628164 | 0.529413327 | 3.193318611 | 0.001504846 |
| CCNA1 | -0.801006142 | 4.502166695 | -3.193232312 | 0.001505286 |
| C1orf74 | 0.259558233 | 2.123937238 | 3.192155138 | 0.001510786 |
| AC097713.4 | -0.402424733 | -0.81609 | -3.192020632 | 0.001511474 |
| TLCD1 | -0.36218844 | 0.384311225 | -3.191985511 | 0.001511653 |
| PDE6A | -0.500019024 | -4.109338687 | -3.191755659 | 0.00151283 |
| RPL39 | 0.199771975 | 5.154486502 | 3.191501244 | 0.001514133 |
| AC108676.1 | -0.478555302 | -2.679587191 | -3.191200005 | 0.001515677 |
| MIR222 | 0.412871284 | 0.437465691 | 3.190930925 | 0.001517058 |
| CYP4F22 | -0.491849477 | -1.394429138 | -3.19032296 | 0.001520182 |
| RP11-196G18.22 | -0.294895929 | 4.512401176 | -3.190314082 | 0.001520228 |
| HSPE1 | 0.233928528 | 3.958326832 | 3.190185397 | 0.00152089 |
| CNOT7 | -0.092688653 | 6.101806059 | -3.189878175 | 0.001522471 |
| LAMA2 | 0.398357933 | -2.653808348 | 3.189847909 | 0.001522627 |
| RAPH1 | 0.662090288 | -0.376031034 | 3.189674605 | 0.00152352 |
| IGFLR1 | -0.27295341 | 2.788070981 | -3.1883068 | 0.001530585 |
| CACNA2D3 | -0.829425145 | -0.171357205 | -3.187984933 | 0.001532252 |
| ARMC9 | -0.429064805 | -0.200840534 | -3.187478614 | 0.001534877 |
| POGZ | 0.093592866 | 5.899754181 | 3.186396671 | 0.001540501 |
| RP11-282I1.1 | -0.431814603 | -1.413814353 | -3.186387447 | 0.001540549 |
| RP11-713C5.1 | -0.668206756 | -1.654473541 | -3.185924804 | 0.00154296 |
| TENM2 | 0.502772018 | -5.825104373 | 3.185801068 | 0.001543605 |
| MANEA-AS1 | -0.267242877 | 1.560224004 | -3.185668957 | 0.001544294 |
| PKN3 | 0.339320065 | 4.209008419 | 3.185475412 | 0.001545305 |
| RP11-79O8.1 | -0.358983174 | -0.471694095 | -3.185008317 | 0.001547746 |
| OR2W6P | 0.46141656 | -1.028013275 | 3.184970632 | 0.001547943 |
| HNRNPA1P8 | 0.22077382 | 4.528496267 | 3.184686289 | 0.00154943 |
| ANXA1 | -0.312000192 | 9.5318154 | -3.184589651 | 0.001549936 |
| PLGLA | -0.441392992 | -2.358546622 | -3.184589452 | 0.001549937 |
| GMCL1P1 | -0.226728467 | 1.015756074 | -3.184334206 | 0.001551274 |
| PPP5C | -0.161254707 | 4.829596084 | -3.184270339 | 0.001551609 |
| RPUSD4 | -0.124984871 | 4.87987404 | -3.184152887 | 0.001552225 |
| AP3S2 | -0.143362957 | 3.271913341 | -3.183566667 | 0.001555302 |
| RP11-73M7.6 | 0.341082024 | -0.616791103 | 3.183511955 | 0.001555589 |
| PCDHB16 | 0.479301136 | -3.88466267 | 3.182842693 | 0.001559109 |
| PCGF6 | 0.154475386 | 3.653156251 | 3.182499832 | 0.001560915 |
| NBAS | -0.173282318 | 5.017244116 | -3.180961612 | 0.001569042 |
| RPA2P2 | -0.348671533 | -0.386210956 | -3.180505846 | 0.001571457 |
| GOLGA6L17P | 0.372798303 | 0.121054608 | 3.179871025 | 0.001574827 |
| KIAA0430 | -0.107267624 | 6.316058061 | -3.17961586 | 0.001576184 |
| SMIM5 | 0.338538736 | 1.033056171 | 3.179273497 | 0.001578006 |
| FRAT1 | -0.264653657 | 5.787606779 | -3.179003866 | 0.001579442 |
| FANCA | 0.24223619 | 3.909420462 | 3.179002352 | 0.00157945 |
| PSMA3 | 0.104248594 | 5.960380722 | 3.178765473 | 0.001580712 |
| SLC25A35 | -0.206058881 | 3.845605862 | -3.178726999 | 0.001580917 |
| SARS2 | 0.188772267 | 2.160526635 | 3.178317411 | 0.001583103 |
| GPR137 | 0.105190642 | 3.953960748 | 3.178012485 | 0.001584732 |
| EGOT | -0.552470032 | -0.788089687 | -3.177931679 | 0.001585164 |
| C5orf55 | -0.237907593 | 1.954142403 | -3.177553443 | 0.001587187 |
| IFIT3 | 0.460449073 | 5.466271406 | 3.177040691 | 0.001589934 |
| NRTN | 0.367549769 | -0.643539924 | 3.177019469 | 0.001590048 |
| TMEM185B | -0.165625964 | 5.791327689 | -3.176756136 | 0.00159146 |
| BTF3P6 | -0.327815135 | -0.434156934 | -3.176635353 | 0.001592109 |
| RP5-940J5.3 | -0.349469095 | 0.115946025 | -3.176605676 | 0.001592268 |
| DPH6 | -0.202948383 | 1.804804279 | -3.176547729 | 0.001592579 |
| PPDPF | 0.194927867 | 8.771635189 | 3.176417507 | 0.001593278 |
| RPL13P12 | -0.153044353 | 10.91068163 | -3.175976485 | 0.001595649 |
| ABLIM2 | -0.369679746 | -2.793272806 | -3.175947863 | 0.001595803 |
| CTD-3185P2.1 | -0.182214269 | 1.703076945 | -3.175038322 | 0.001600702 |
| PSMB3 | -0.127372526 | 7.681353238 | -3.174240792 | 0.00160501 |
| RP11-386G11.5 | 0.327341031 | 0.633260333 | 3.173179657 | 0.001610758 |
| POLR2J | 0.114638545 | 6.691044293 | 3.173171867 | 0.0016108 |
| SURF4 | 0.146106789 | 8.540671486 | 3.173144363 | 0.00161095 |
| COX6C | 0.142702903 | 6.506015331 | 3.172295063 | 0.001615564 |
| AL354718.1 | -0.357754743 | 1.299883581 | -3.171691401 | 0.001618852 |
| MBD3 | -0.138392795 | 5.164436459 | -3.171212732 | 0.001621463 |
| AC018463.4 | -0.220099537 | 3.658957431 | -3.171170087 | 0.001621696 |
| NGEF | 0.588437558 | -3.375285643 | 3.171069013 | 0.001622248 |
| URGCP | 0.156516738 | 3.797836176 | 3.16967216 | 0.001629893 |
| DENND5A | 0.167429297 | 7.129015811 | 3.169639603 | 0.001630072 |
| CARD17 | -0.465508599 | 0.297855714 | -3.16941933 | 0.001631281 |
| RP11-552F3.10 | -0.336866171 | -1.108177634 | -3.16940001 | 0.001631387 |
| RP11-127L20.3 | 0.538820014 | -0.343702993 | 3.168952553 | 0.001633845 |
| RNVU1-19 | -0.588925038 | -0.605279255 | -3.16853407 | 0.001636147 |
| AC091633.2 | 0.34032465 | 0.070666385 | 3.167924412 | 0.001639506 |
| SLC1A3 | -0.524512605 | 1.990861044 | -3.167793279 | 0.00164023 |
| RP11-578F21.3 | -0.441258938 | -0.496380866 | -3.167749943 | 0.001640469 |
| MPZL3 | 0.327164744 | 3.383364415 | 3.167595265 | 0.001641323 |
| GBP5 | 0.371210561 | 5.120665719 | 3.167367875 | 0.001642579 |
| GTF2F1 | 0.114960527 | 7.367356576 | 3.167051844 | 0.001644325 |
| HABP4 | 0.394650498 | 1.828432815 | 3.166625379 | 0.001646685 |
| CALN1 | 0.864667588 | 0.357453267 | 3.166417672 | 0.001647836 |
| SNORA16 | -0.287483177 | 2.394304346 | -3.166139123 | 0.00164938 |
| MCM4 | 0.316300894 | 6.496021579 | 3.166101114 | 0.001649591 |
| RP11-423O2.2 | -0.450294376 | -0.696268214 | -3.165272994 | 0.00165419 |
| PODXL2 | 0.509455872 | 3.266394651 | 3.165163427 | 0.001654799 |
| RP11-129J12.2 | -0.963949549 | 0.544034031 | -3.164977336 | 0.001655835 |
| NUP210L | -0.368111128 | -2.838750162 | -3.164717617 | 0.001657281 |
| LBH | 0.337575748 | 3.228532652 | 3.164471642 | 0.001658652 |
| MIR10A | -0.691797783 | -1.751106114 | -3.163963903 | 0.001661484 |
| CDYL2 | 0.31763638 | 4.308399229 | 3.163741458 | 0.001662727 |
| BTRC | -0.152671182 | 3.846902739 | -3.163479499 | 0.001664191 |
| MSRA | 0.196993186 | 4.898297131 | 3.16282315 | 0.001667865 |
| EXOSC1 | -0.089745206 | 4.492947554 | -3.162804072 | 0.001667972 |
| YBX1P2 | 0.140243589 | 8.327929697 | 3.162403494 | 0.001670218 |
| FUT4 | 0.311707266 | 6.718309485 | 3.162249028 | 0.001671085 |
| ERCC3 | 0.088014149 | 5.285537579 | 3.162154198 | 0.001671617 |
| TLR8-AS1 | -0.428270051 | -1.198518687 | -3.162007431 | 0.001672441 |
| HNRNPA3 | -0.12681189 | 7.493619905 | -3.161925865 | 0.0016729 |
| ESAM | 0.701903678 | 2.103991282 | 3.161860357 | 0.001673268 |
| FARSB | -0.158158958 | 5.486570334 | -3.160885643 | 0.001678754 |
| STON1 | -0.356968169 | 0.610339686 | -3.160786869 | 0.001679311 |
| CDK15 | -0.675293992 | -1.622540251 | -3.159884333 | 0.001684407 |
| CLCN2 | 0.242850235 | 1.922604455 | 3.159758111 | 0.001685121 |
| VAMP1 | 0.231833475 | 5.912083626 | 3.159006168 | 0.001689379 |
| SEPT2P1 | -0.17767872 | 3.782405374 | -3.158263243 | 0.001693596 |
| HCN3 | -0.233558263 | 1.996642625 | -3.15806293 | 0.001694735 |
| HMMR | 0.44593319 | 3.182771488 | 3.15775591 | 0.001696482 |
| KB-1460A1.1 | -0.488675223 | 0.540804991 | -3.157622433 | 0.001697242 |
| SUN3 | -0.350551073 | -1.545170482 | -3.157541562 | 0.001697702 |
| KRTAP5-8 | -0.389508092 | -0.753688445 | -3.15753594 | 0.001697734 |
| CTA-384D8.36 | 0.203775406 | 3.674095621 | 3.157105108 | 0.00170019 |
| GS1-124K5.6 | -0.296225635 | 0.749078679 | -3.157024564 | 0.001700649 |
| NLRP12 | -0.641911111 | 3.394996884 | -3.157023497 | 0.001700655 |
| AL591025.1 | 0.285044386 | 2.598553358 | 3.156290265 | 0.001704843 |
| TBL2 | -0.206203074 | 4.153662875 | -3.15623014 | 0.001705187 |
| MCU | 0.271971415 | 4.827040025 | 3.155980685 | 0.001706614 |
| AC092159.2 | 0.381979464 | -1.646503271 | 3.155705924 | 0.001708187 |
| ADAL | -0.1980147 | 2.659619662 | -3.15553576 | 0.001709162 |
| ZNF235 | -0.172550903 | 2.019205183 | -3.153980952 | 0.001718095 |
| CNTN1 | -0.849793761 | -3.187787679 | -3.153591731 | 0.001720337 |
| CTA-276O3.4 | 0.247407359 | 3.102828088 | 3.153174984 | 0.001722742 |
| RAD51-AS1 | -0.275940295 | 4.929620942 | -3.153091104 | 0.001723226 |
| ARHGEF35 | 0.482917716 | -0.850217849 | 3.153078684 | 0.001723298 |
| TTLL7 | -0.614313651 | -0.495203878 | -3.152986355 | 0.001723831 |
| RPS27P29 | 0.16702751 | 4.338376248 | 3.152810803 | 0.001724845 |
| ANKRD37 | 0.377735319 | 1.369242563 | 3.151634829 | 0.001731654 |
| TMEM243 | 0.270109449 | 3.451216757 | 3.151602809 | 0.00173184 |
| TIMM10B | 0.124933695 | 5.624533379 | 3.151550289 | 0.001732145 |
| ABHD1 | -0.347181533 | 0.09494471 | -3.151220139 | 0.001734061 |
| RP11-345M22.2 | -0.406524403 | -1.549023615 | -3.15110414 | 0.001734735 |
| DGCR6 | 0.31567641 | 2.464381459 | 3.150996005 | 0.001735364 |
| RP11-1079K10.3 | -0.298690727 | 0.990462964 | -3.150944392 | 0.001735664 |
| IRF2BP1 | -0.150131969 | 5.849727441 | -3.150555916 | 0.001737923 |
| BICD1 | -0.238273697 | 3.037615421 | -3.149298162 | 0.001745258 |
| UCHL1 | 0.664356976 | -0.864584208 | 3.148934287 | 0.001747385 |
| CD69 | 0.514923628 | 8.023661257 | 3.14883137 | 0.001747987 |
| ETV4 | 0.57060297 | -2.461556885 | 3.148747639 | 0.001748477 |
| XCL2 | 0.455820921 | 1.639343975 | 3.148338828 | 0.001750871 |
| EMB | -0.289799274 | 8.637126761 | -3.14825677 | 0.001751352 |
| RPAIN | 0.154983932 | 4.751396516 | 3.148250273 | 0.00175139 |
| RP11-649E7.5 | 0.285052627 | -0.035011497 | 3.148071635 | 0.001752438 |
| RP11-154H12.3 | 0.515330313 | 0.337742975 | 3.148011494 | 0.00175279 |
| APOL4 | -0.817632975 | 0.647723519 | -3.147730709 | 0.001754438 |
| AC013402.5 | -0.654863594 | -0.862809796 | -3.146960482 | 0.001758966 |
| AMOT | -0.394418034 | 1.391739488 | -3.14687095 | 0.001759493 |
| POLDIP2 | 0.094611244 | 7.111056128 | 3.145667427 | 0.00176659 |
| ANP32C | -0.204212127 | 5.021051992 | -3.14533592 | 0.00176855 |
| AIFM3 | -0.446678792 | 2.511428492 | -3.14526618 | 0.001768963 |
| RP11-344P13.3 | -0.368616252 | -0.351745797 | -3.145130407 | 0.001769766 |
| AC068282.3 | 0.280050909 | 1.756623935 | 3.144631172 | 0.001772723 |
| CTD-2256P15.4 | -0.433804915 | -2.240588566 | -3.144490865 | 0.001773555 |
| TPTE2P1 | -0.536100644 | -1.831610586 | -3.144143187 | 0.001775618 |
| C12orf66 | -0.179843442 | 3.396405983 | -3.144114165 | 0.00177579 |
| PCDH18 | 0.543683179 | -3.740080976 | 3.143719234 | 0.001778136 |
| RPN1 | -0.136073977 | 8.001696114 | -3.143174602 | 0.001781376 |
| RP11-696N14.1 | -0.430395725 | 0.156187016 | -3.142816642 | 0.001783509 |
| RP11-100N21.1 | 0.192376417 | 3.03914365 | 3.142518366 | 0.001785288 |
| PSMC1P1 | 0.090761755 | 7.759402238 | 3.142145697 | 0.001787512 |
| RP11-631M6.3 | 0.417183844 | 2.399661888 | 3.14137098 | 0.001792145 |
| KLHL23 | -0.407254367 | 2.646744739 | -3.141367587 | 0.001792166 |
| RP11-274B21.2 | 0.204841574 | 7.32995917 | 3.14097545 | 0.001794515 |
| PAIP2B | -0.424375767 | 1.274067021 | -3.140673962 | 0.001796323 |
| PLEKHO2 | 0.26153203 | 6.530575056 | 3.140609876 | 0.001796708 |
| RP11-334L9.1 | 0.346943978 | 3.045616994 | 3.140530077 | 0.001797187 |
| RP1-313I6.12 | -0.226165815 | 1.963089773 | -3.14045697 | 0.001797625 |
| TP53I3 | -0.319467978 | 4.82294745 | -3.140004149 | 0.001800346 |
| CDK20 | -0.508062183 | 0.689540511 | -3.139952819 | 0.001800655 |
| RP1-34B20.4 | -0.502259136 | 2.076525919 | -3.139813801 | 0.001801491 |
| ZXDC | 0.128839689 | 5.631106374 | 3.139490946 | 0.001803434 |
| CTD-2619J13.8 | -0.422067238 | -1.692289578 | -3.139444488 | 0.001803714 |
| FSBP | 0.393888748 | -1.202015129 | 3.138992835 | 0.001806436 |
| KRT23 | -0.833791819 | 0.913959038 | -3.138847903 | 0.001807311 |
| FH | -0.158949292 | 6.450645427 | -3.138646718 | 0.001808525 |
| RP11-463O12.3 | 0.369925684 | 0.524008153 | 3.137740625 | 0.001814003 |
| TRAV13-2 | -0.429465077 | -0.072808879 | -3.13743482 | 0.001815856 |
| HKR1 | 0.173387632 | 4.404236189 | 3.137079046 | 0.001818013 |
| RSL24D1P1 | 0.231607138 | 5.54376962 | 3.136836948 | 0.001819482 |
| YME1L1 | 0.059608261 | 7.030901214 | 3.136562416 | 0.00182115 |
| RP11-82L18.2 | -0.380629449 | -1.065635987 | -3.136560887 | 0.001821159 |
| SNORA19 | 0.336580103 | 1.205801991 | 3.135892776 | 0.001825223 |
| RP11-785H5.1 | -0.188903774 | 5.48896666 | -3.135818143 | 0.001825678 |
| TNRC6A | 0.104173475 | 5.053948414 | 3.135648513 | 0.001826711 |
| PIR | 0.509873285 | 0.430126976 | 3.135540632 | 0.001827369 |
| PCGF2 | 0.541738606 | -1.110129207 | 3.134781257 | 0.001832003 |
| S100A16 | 1.049139725 | 1.162464616 | 3.134523241 | 0.00183358 |
| RP11-849F2.9 | -0.336778768 | 0.611931235 | -3.134458354 | 0.001833977 |
| NUTF2 | -0.143543225 | 6.039582914 | -3.13372816 | 0.001838448 |
| PDIA2 | 0.417184297 | -1.988762455 | 3.133503848 | 0.001839824 |
| RPSAP53 | 0.347660459 | 0.998987182 | 3.133262076 | 0.001841307 |
| E2F3 | 0.121207747 | 6.045100684 | 3.133259264 | 0.001841324 |
| GIPR | 0.537949004 | 0.246586738 | 3.133227507 | 0.001841519 |
| ILF2 | 0.143903882 | 8.985720609 | 3.133056093 | 0.001842572 |
| SBNO2 | 0.17944812 | 6.377548092 | 3.133031382 | 0.001842724 |
| LINC00243 | -0.480347927 | -0.34808556 | -3.132572695 | 0.001845543 |
| PMS2 | -0.12177697 | 4.149830092 | -3.132294199 | 0.001847257 |
| RP11-3P17.4 | 0.454946384 | -0.933426524 | 3.132218445 | 0.001847724 |
| U91328.20 | 0.278925166 | 2.894184072 | 3.132060964 | 0.001848694 |
| XXyac-YRM2039.2 | 0.179062199 | 4.863918354 | 3.131965538 | 0.001849282 |
| MAN2A1 | -0.155960699 | 4.877457843 | -3.131445033 | 0.001852492 |
| UROS | 0.162906067 | 5.140658949 | 3.13123823 | 0.001853769 |
| ZC3H12C | 0.483044135 | 2.966079403 | 3.131021537 | 0.001855108 |
| ALKBH3 | -0.130770325 | 5.113337581 | -3.130997772 | 0.001855255 |
| RP11-1094M14.5 | -0.475833642 | 1.651896577 | -3.130783249 | 0.001856582 |
| SDHAF2 | 0.138813744 | 3.442938719 | 3.130425694 | 0.001858795 |
| RP11-622K12.1 | -0.182510534 | 6.629177136 | -3.130033967 | 0.001861222 |
| RP11-126O1.5 | 0.336812439 | -0.04850834 | 3.129808919 | 0.001862618 |
| CD300LF | -0.342661371 | 6.526829017 | -3.129648679 | 0.001863612 |
| RP11-349A22.5 | -0.134016926 | 6.265311658 | -3.129139402 | 0.001866776 |
| HCG11 | -0.438189865 | 3.243496064 | -3.129010729 | 0.001867576 |
| CTD-2144E22.10 | -0.309882094 | -0.864657198 | -3.128801415 | 0.001868878 |
| MEF2A | -0.189115467 | 6.044692903 | -3.127788187 | 0.001875193 |
| RNU6-100P | 0.354686079 | 0.900838061 | 3.126966858 | 0.001880327 |
| SEMA4C | 0.360026211 | 3.560935363 | 3.126795907 | 0.001881397 |
| YTHDC2 | 0.12299925 | 4.550431351 | 3.126098697 | 0.001885768 |
| STRN3 | -0.12616322 | 4.668274195 | -3.125150534 | 0.001891726 |
| TMEM258 | 0.144798429 | 4.988355402 | 3.124341036 | 0.001896827 |
| TMPRSS4 | 0.499068849 | -4.880352737 | 3.124281938 | 0.0018972 |
| CD163L1 | -0.575566397 | -1.271732033 | -3.124194288 | 0.001897753 |
| YAP1 | 0.558263922 | -3.966339523 | 3.124186966 | 0.001897799 |
| AP001469.9 | -0.303666224 | -0.223950287 | -3.123881239 | 0.00189973 |
| RP3-330M21.5 | 0.323153417 | -0.371150416 | 3.123515441 | 0.001902042 |
| AK1 | 0.319440795 | 1.13346731 | 3.123431924 | 0.001902571 |
| ADAMTS5 | -0.749672319 | -3.337538058 | -3.123157875 | 0.001904305 |
| SLC15A3 | 0.589684858 | 4.080617679 | 3.123119161 | 0.00190455 |
| DDX11-AS1 | -0.367752138 | 0.042600031 | -3.122891733 | 0.001905991 |
| NAALAD2 | 0.60171186 | -3.581530967 | 3.122555073 | 0.001908126 |
| FBXO38 | -0.146545933 | 4.811437878 | -3.122021883 | 0.001911511 |
| L2HGDH | 0.20238357 | 2.389600272 | 3.121656998 | 0.001913831 |
| CCDC181 | 0.356380497 | -2.961872661 | 3.121569064 | 0.00191439 |
| HSD17B14 | 0.490084263 | -0.285719612 | 3.121120177 | 0.001917248 |
| DDX54 | 0.113089295 | 6.405687832 | 3.121073745 | 0.001917544 |
| FAM21FP | -0.204538122 | 2.074530905 | -3.120174292 | 0.001923284 |
| RNU4-2 | -0.455404427 | 0.250010483 | -3.119830146 | 0.001925484 |
| OTUB2 | -0.416169829 | -0.318847239 | -3.119768057 | 0.001925881 |
| RP11-173P15.3 | -0.399009024 | -1.491505035 | -3.119691902 | 0.001926368 |
| SLC23A2 | 0.153322887 | 5.202451799 | 3.119535422 | 0.00192737 |
| DEFB1 | -0.973268786 | 1.658911172 | -3.119485183 | 0.001927692 |
| TRMT1 | -0.152591676 | 6.266488785 | -3.119405381 | 0.001928203 |
| RP11-46J23.1 | 0.397153508 | 0.584004608 | 3.119177708 | 0.001929662 |
| C11orf1 | 0.219737388 | 4.077855509 | 3.119105888 | 0.001930122 |
| RP11-466H18.1 | -0.130182988 | 11.34360432 | -3.119079069 | 0.001930294 |
| CTD-3074O7.5 | 0.219254252 | 2.165630857 | 3.119068609 | 0.001930361 |
| TUBGCP6 | -0.116570778 | 6.133101521 | -3.118924575 | 0.001931285 |
| DYNLRB1 | -0.137704392 | 6.537784186 | -3.118784653 | 0.001932182 |
| CYSTM1 | 0.463061518 | 5.188278324 | 3.11848672 | 0.001934095 |
| RP11-488L18.3 | -0.390848277 | -1.527490541 | -3.118086493 | 0.001936668 |
| CLYBL | -0.258180098 | 1.45890157 | -3.117834525 | 0.001938289 |
| TGIF1 | 0.270574613 | 4.551129875 | 3.117648591 | 0.001939486 |
| RASGRP2 | -0.16633472 | 7.431779588 | -3.116655887 | 0.001945888 |
| TLX2 | 0.443015401 | -2.446445681 | 3.116534027 | 0.001946675 |
| Z95704.1 | 0.454229865 | -0.344923975 | 3.116504261 | 0.001946868 |
| LINC00925 | -0.530720364 | -0.202934311 | -3.116383853 | 0.001947646 |
| RPGR | 0.198513437 | 3.953131805 | 3.115358983 | 0.001954282 |
| TBC1D3G | -0.45211216 | -3.302839119 | -3.11502064 | 0.001956477 |
| FAM98C | 0.153541562 | 3.483383085 | 3.114224479 | 0.001961652 |
| PRR11 | 0.244226942 | 4.893274593 | 3.113897971 | 0.001963777 |
| CAMTA2 | -0.162075038 | 5.656440194 | -3.113847259 | 0.001964108 |
| PPIL2 | -0.093542629 | 5.179035718 | -3.113619437 | 0.001965592 |
| ATP5A1 | -0.157631728 | 7.590054533 | -3.11352244 | 0.001966225 |
| FECH | 0.266924943 | 5.473354455 | 3.113371125 | 0.001967212 |
| MCM7 | 0.23101851 | 7.601394219 | 3.112808532 | 0.001970886 |
| SHANK2 | 0.6886548 | -5.099714691 | 3.112591742 | 0.001972303 |
| CCDC115 | -0.104988034 | 5.673135914 | -3.112274499 | 0.001974379 |
| GNG11 | 0.806020521 | 0.958937466 | 3.110550878 | 0.001985692 |
| KIAA1598 | -0.754264906 | 2.637238122 | -3.109786995 | 0.001990725 |
| RP11-366M4.17 | 0.329648758 | 1.436571472 | 3.109551469 | 0.001992279 |
| CRADD | 0.226703268 | 2.545754033 | 3.109344968 | 0.001993642 |
| CTD-2116N20.1 | -0.361522169 | 0.031861701 | -3.109258054 | 0.001994216 |
| DHPS | -0.118733292 | 6.115399601 | -3.109147098 | 0.00199495 |
| AC010883.5 | 0.30193994 | 3.108024077 | 3.108822208 | 0.001997098 |
| SMC1A | -0.209163984 | 6.470522832 | -3.108415542 | 0.00199979 |
| AP000936.1 | 0.172166978 | 3.692952306 | 3.108236356 | 0.002000977 |
| RP11-13A1.1 | -0.503994073 | -1.791293104 | -3.108152499 | 0.002001533 |
| RP11-73O6.3 | -0.45966584 | -0.829066267 | -3.108152302 | 0.002001534 |
| MADD | -0.134700447 | 6.569906496 | -3.10763407 | 0.002004972 |
| MRPL52 | -0.106639887 | 4.986840049 | -3.107549554 | 0.002005534 |
| KCNAB2 | -0.157841388 | 7.603162355 | -3.107354519 | 0.002006829 |
| RBM19 | 0.117907672 | 5.368623121 | 3.106969832 | 0.002009387 |
| RP11-169K16.7 | -0.162638316 | 7.824460642 | -3.106934283 | 0.002009624 |
| HCP5 | -0.377674606 | 4.817976554 | -3.106921397 | 0.002009709 |
| SMIM12 | 0.112218055 | 4.319871582 | 3.106431491 | 0.002012972 |
| SNAPC4 | -0.099240099 | 4.803240967 | -3.106170774 | 0.00201471 |
| PSMD6-AS2 | 0.296069809 | 3.821637606 | 3.105939731 | 0.002016251 |
| RP11-752G15.9 | -0.264297895 | 1.373230872 | -3.10590238 | 0.002016501 |
| TMEM132C | 0.440117001 | -4.060498613 | 3.105458714 | 0.002019464 |
| SENP3 | -0.104075899 | 4.537218231 | -3.105323887 | 0.002020365 |
| MMADHC | 0.126948462 | 6.998603689 | 3.105042762 | 0.002022246 |
| SLC40A1 | -0.550520403 | 5.823530689 | -3.104734454 | 0.00202431 |
| MARC1 | -0.664725842 | 4.125048368 | -3.103998848 | 0.002029243 |
| MT-TH | 0.335542067 | 2.088967224 | 3.103665149 | 0.002031485 |
| GUSBP4 | -0.240654942 | 2.246803063 | -3.103425392 | 0.002033097 |
| GPS2 | 0.205133013 | 2.479171308 | 3.102394951 | 0.002040037 |
| PRPSAP2 | -0.110103125 | 4.572559723 | -3.101630633 | 0.0020452 |
| RP11-382D12.2 | 0.417717151 | -0.321397797 | 3.101298779 | 0.002047445 |
| RP11-15H20.5 | -0.294886589 | 2.765152932 | -3.101081278 | 0.002048918 |
| KCNQ1OT1 | -0.363977495 | 4.205583451 | -3.100702526 | 0.002051485 |
| AC008753.6 | -0.326947359 | 0.81878719 | -3.100046193 | 0.00205594 |
| MRPL14 | -0.17131237 | 5.53212571 | -3.098751783 | 0.002064753 |
| RP11-335G20.7 | 0.371762099 | 2.755224529 | 3.098603227 | 0.002065766 |
| NIPSNAP1 | -0.196770159 | 5.559156219 | -3.098431174 | 0.002066941 |
| RN7SKP70 | 0.355675459 | -0.075019057 | 3.098372565 | 0.002067341 |
| RP11-498E2.8 | -0.241493262 | 1.955700243 | -3.098143119 | 0.002068909 |
| ZNF48 | -0.187703184 | 3.330350468 | -3.097572692 | 0.002072811 |
| RNU5E-4P | -0.447226459 | 1.142483941 | -3.097164311 | 0.002075608 |
| AC144521.1 | -0.336271073 | 2.661440264 | -3.096900154 | 0.00207742 |
| RP11-84C13.1 | -0.259038793 | 3.893254674 | -3.096471094 | 0.002080365 |
| CTC-559E9.5 | -0.2689753 | 3.709724723 | -3.096277245 | 0.002081697 |
| CD47 | -0.185910115 | 7.00894435 | -3.096008942 | 0.002083542 |
| FCRLB | -0.437716655 | 0.503841603 | -3.095948181 | 0.00208396 |
| ZNF815P | -0.193361912 | 2.697640858 | -3.095891524 | 0.00208435 |
| RP5-981O7.2 | 0.271449864 | 1.500066101 | 3.095010835 | 0.002090418 |
| LINC00843 | -0.203778679 | 3.464121264 | -3.094943175 | 0.002090885 |
| RTN4RL2 | 0.376083335 | 1.369496881 | 3.094739209 | 0.002092293 |
| SSU72 | -0.123957894 | 5.959785631 | -3.093701259 | 0.002099472 |
| FAM118A | -0.282358209 | 4.672597823 | -3.093586463 | 0.002100267 |
| TJP2 | 0.337219319 | 4.06884997 | 3.093503835 | 0.00210084 |
| GEMIN8 | -0.136495092 | 4.507992223 | -3.093156927 | 0.002103245 |
| SRC | 0.379604136 | 3.813135291 | 3.093010835 | 0.002104259 |
| ALDOC | 0.346627811 | 3.537360088 | 3.092911948 | 0.002104946 |
| CTD-2272G21.2 | 0.23900498 | 0.538387748 | 3.092600859 | 0.002107107 |
| TMEM256 | -0.224519988 | 3.186892447 | -3.092501446 | 0.002107798 |
| RP11-153M7.3 | -0.79855094 | -0.384151273 | -3.092481496 | 0.002107937 |
| IDSP1 | 0.297385253 | 0.980840629 | 3.092464166 | 0.002108057 |
| RALGAPA2 | -0.178996705 | 5.623694087 | -3.092301833 | 0.002109186 |
| SLC44A1 | -0.365981173 | 5.546628646 | -3.092141149 | 0.002110304 |
| CR1L | 0.510686097 | -0.488943458 | 3.092016762 | 0.00211117 |
| DSEL | 0.502714996 | -1.063366701 | 3.091907452 | 0.002111932 |
| RANP1 | -0.179662826 | 7.129904287 | -3.091222084 | 0.00211671 |
| MXRA5 | 0.640189718 | -5.044038296 | 3.09108805 | 0.002117646 |
| EMP2 | 0.657903134 | -2.196281041 | 3.090791074 | 0.00211972 |
| MS4A6A | -0.727438195 | 5.123829727 | -3.090781501 | 0.002119787 |
| LINC00920 | -0.433100819 | 0.939094434 | -3.090742954 | 0.002120057 |
| DDX10 | -0.179765486 | 3.787807587 | -3.090566478 | 0.00212129 |
| AC020907.2 | 0.409245489 | -0.913233337 | 3.090401879 | 0.002122442 |
| DYNC2LI1 | -0.215296426 | 2.875855391 | -3.090095093 | 0.002124589 |
| ZNF259 | 0.117332136 | 6.522494211 | 3.089215248 | 0.002130759 |
| KIF19 | 0.45544237 | -2.37850197 | 3.089067099 | 0.0021318 |
| RP11-17M16.1 | -0.362364972 | 0.045048769 | -3.088576038 | 0.002135252 |
| TREM1 | 0.603655294 | 5.326232337 | 3.088342339 | 0.002136897 |
| MIR3153 | -0.371928906 | 1.381765623 | -3.08817613 | 0.002138068 |
| RP13-297E16.5 | -0.41923857 | -0.451197829 | -3.087571702 | 0.002142329 |
| TANGO6 | -0.259807274 | 2.787119633 | -3.087512467 | 0.002142747 |
| RP4-800G7.1 | 0.24403216 | 1.832512185 | 3.0874721 | 0.002143032 |
| RARA | 0.242702111 | 6.441055698 | 3.087368152 | 0.002143766 |
| DSCC1 | 0.295192754 | 3.440985038 | 3.087264853 | 0.002144496 |
| TRAV8-3 | -0.419407755 | 0.921288485 | -3.087093208 | 0.002145709 |
| ABCB1 | 0.579979098 | 2.735312726 | 3.086861885 | 0.002147344 |
| LY86 | -0.746492748 | 5.698737352 | -3.086479188 | 0.002150052 |
| RANBP3 | 0.089230008 | 5.142979092 | 3.086377364 | 0.002150773 |
| F7 | -0.475424804 | -3.135566914 | -3.086240968 | 0.00215174 |
| CTD-2517O10.6 | -0.406644497 | -1.579999493 | -3.086191899 | 0.002152087 |
| RALY | 0.088181972 | 8.359925104 | 3.0859654 | 0.002153693 |
| INCENP | 0.229597667 | 5.020756034 | 3.085915864 | 0.002154044 |
| RAMP1 | 0.845879501 | 2.316485561 | 3.085835155 | 0.002154617 |
| TMSB4Y | 0.764557439 | 1.018999502 | 3.085600916 | 0.002156279 |
| AC004463.6 | -0.325900378 | 1.255811185 | -3.085439305 | 0.002157427 |
| TNFAIP8 | -0.248986377 | 5.084079441 | -3.085428656 | 0.002157502 |
| ATF5 | 0.208001533 | 5.025005619 | 3.084634175 | 0.002163153 |
| RGS5 | -0.335369494 | -0.792737026 | -3.084391042 | 0.002164884 |
| FLJ00104 | -0.206104964 | 1.176373719 | -3.084147642 | 0.002166619 |
| CALD1 | 0.685213392 | -1.883875951 | 3.083666608 | 0.002170052 |
| SHROOM1 | -0.55717777 | -0.510258218 | -3.083484207 | 0.002171355 |
| XPA | 0.237449688 | 5.291365264 | 3.082338008 | 0.002179559 |
| PDK2 | -0.181854811 | 4.055880187 | -3.082286351 | 0.002179929 |
| RP11-1002K11.1 | -0.43147311 | -2.4348461 | -3.081845873 | 0.00218309 |
| ALDH1A2 | 0.552712077 | -2.442059578 | 3.081463589 | 0.002185837 |
| PIP5K1A | -0.151141661 | 5.936701774 | -3.08125079 | 0.002187367 |
| C10orf35 | 0.465999468 | -0.024354261 | 3.081160873 | 0.002188014 |
| CPNE4 | 0.530430576 | -3.495108266 | 3.080611909 | 0.002191967 |
| AC005262.3 | -0.432214356 | 0.266588883 | -3.080442197 | 0.002193191 |
| KARS | -0.114708234 | 7.631880434 | -3.08017022 | 0.002195153 |
| ZNF541 | 0.455317538 | -2.457969946 | 3.080158319 | 0.002195239 |
| NFASC | 0.581007004 | -4.507739682 | 3.079500615 | 0.00219999 |
| CAPN10-AS1 | -0.294382243 | 0.363490323 | -3.079439868 | 0.002200429 |
| AC097523.3 | 0.326132155 | -0.268295868 | 3.078789881 | 0.002205135 |
| TBC1D14 | -0.149487458 | 6.368008528 | -3.078765995 | 0.002205309 |
| ARL15 | -0.208404803 | 3.376792486 | -3.078014402 | 0.002210762 |
| ZNF534 | 0.411568184 | -2.269080226 | 3.077618981 | 0.002213636 |
| AC006946.16 | -0.479341965 | -2.916733394 | -3.077601578 | 0.002213763 |
| RP11-1348G14.4 | -0.309765375 | 1.188234622 | -3.07721798 | 0.002216555 |
| IGHJ4 | 0.485653436 | 2.778530879 | 3.076959379 | 0.002218438 |
| PRG3 | 0.928705177 | 1.871851793 | 3.076906509 | 0.002218824 |
| C19orf48 | 0.199339544 | 5.812454643 | 3.07655729 | 0.00222137 |
| EPB42 | 0.758930317 | 1.05547589 | 3.076383743 | 0.002222637 |
| ARMC2 | -0.181686081 | 3.079641148 | -3.076296372 | 0.002223275 |
| RP11-893F2.9 | -0.394428401 | -0.046503533 | -3.076159537 | 0.002224274 |
| Z85986.1 | -0.358094906 | 1.264362647 | -3.076158001 | 0.002224285 |
| DIRAS1 | 0.474795273 | 1.2248944 | 3.075400937 | 0.002229822 |
| FCHO1 | -0.194940196 | 4.724907209 | -3.075383995 | 0.002229946 |
| GNAQ | -0.122264283 | 7.392728502 | -3.075174027 | 0.002231484 |
| DDX5 | -0.118490496 | 10.03264359 | -3.074692393 | 0.002235015 |
| RP11-983P16.2 | 0.323845516 | 0.722400692 | 3.074403087 | 0.002237139 |
| SERINC2 | 0.589925401 | 2.353675567 | 3.073984548 | 0.002240215 |
| ARID5B | 0.338206408 | 4.30131167 | 3.073690939 | 0.002242375 |
| SLC39A14 | -0.320203138 | 2.7031505 | -3.073557301 | 0.002243358 |
| GS1-259H13.7 | 0.314505142 | -0.04665049 | 3.07286363 | 0.002248471 |
| PLXNA3 | -0.264049092 | 4.258787224 | -3.072375911 | 0.002252072 |
| ACACB | -0.270905198 | 2.911964992 | -3.072374819 | 0.00225208 |
| FAM122B | -0.110600186 | 6.100554091 | -3.072248412 | 0.002253014 |
| CDH5 | 0.624870366 | -2.164992237 | 3.071962849 | 0.002255126 |
| ZBTB8A | 0.531322696 | 1.348949575 | 3.071620955 | 0.002257657 |
| VNN3P | 0.715757037 | 1.910189405 | 3.070843194 | 0.002263424 |
| RANGRF | -0.128285263 | 5.591995067 | -3.070684476 | 0.002264602 |
| PRCP | -0.23136622 | 6.004680428 | -3.070329094 | 0.002267243 |
| CCDC50 | 0.5903388 | 3.124790904 | 3.069562993 | 0.002272945 |
| RHOB | -0.386102248 | 8.872710068 | -3.069493732 | 0.002273461 |
| FCGR1A | -0.476871829 | 4.277285996 | -3.069050841 | 0.002276764 |
| SIK2 | -0.129066174 | 4.261947794 | -3.068482895 | 0.002281007 |
| PRKAR2A | 0.14396147 | 5.75115381 | 3.067969918 | 0.002284845 |
| SNORD19 | 0.288444292 | 1.558642516 | 3.067933707 | 0.002285116 |
| HIST1H4J | -0.405864481 | -0.367212063 | -3.067202473 | 0.002290598 |
| NT5C | -0.174331226 | 6.571212935 | -3.066894826 | 0.002292908 |
| RP13-104F24.3 | 0.13531608 | 3.256608953 | 3.066784423 | 0.002293738 |
| IMMP1L | 0.183077188 | 2.33587961 | 3.066204253 | 0.002298101 |
| AL592188.3 | -0.344946997 | 2.862716121 | -3.066111311 | 0.002298801 |
| HMGB1P4 | 0.336282511 | 0.558398173 | 3.065601893 | 0.002302639 |
| SNORA80B | -0.408985082 | 0.165077913 | -3.065257361 | 0.002305239 |
| PDHX | -0.126606303 | 4.328129287 | -3.065095453 | 0.002306462 |
| GHRLOS | -0.244777741 | 3.050835454 | -3.064674973 | 0.002309639 |
| MC1R | 0.284480955 | 1.010167216 | 3.063848025 | 0.002315901 |
| SPRY4 | 0.710624295 | -1.848694054 | 3.063761854 | 0.002316554 |
| TOMM34 | 0.140135657 | 6.06728098 | 3.063758947 | 0.002316576 |
| TLE1P1 | 0.424906778 | 3.113499029 | 3.062913505 | 0.002322996 |
| LARP1B | 0.156221104 | 4.030694691 | 3.062587641 | 0.002325474 |
| TMEM88 | 0.289212648 | 3.223031997 | 3.062581079 | 0.002325524 |
| DMD | 0.611327176 | -2.415426317 | 3.062509085 | 0.002326072 |
| SLFN14 | 0.575834068 | 0.062233231 | 3.062183991 | 0.002328548 |
| BTBD10 | -0.140412306 | 4.65411277 | -3.06200624 | 0.002329903 |
| SLC22A17 | 0.493742583 | -0.008774379 | 3.061855445 | 0.002331052 |
| IL18R1 | 0.492957759 | 3.032432696 | 3.061610975 | 0.002332918 |
| SKA3 | 0.443565526 | 3.623302507 | 3.0612567 | 0.002335623 |
| PCBP3 | 0.612980121 | 1.082822092 | 3.061095885 | 0.002336852 |
| BPGM | 0.248606315 | 4.991622291 | 3.060536659 | 0.00234113 |
| NLRP3 | 0.350747702 | 6.0417053 | 3.060482682 | 0.002341544 |
| CCDC168 | -0.42827961 | -2.326242899 | -3.060326519 | 0.00234274 |
| NKTR | 0.149382422 | 6.852644519 | 3.060233014 | 0.002343457 |
| OSTCP1 | -0.347313876 | -0.430449857 | -3.060189145 | 0.002343793 |
| ANGEL2 | -0.097013138 | 4.919602499 | -3.060061059 | 0.002344775 |
| AC118754.4 | -0.464289599 | 0.293517095 | -3.059792981 | 0.002346831 |
| RP11-665C16.6 | -0.411498755 | 0.818799316 | -3.059395217 | 0.002349886 |
| TSNARE1 | -0.180809328 | 3.880125937 | -3.059172263 | 0.002351599 |
| SLC1A5 | -0.236384817 | 6.532633056 | -3.058746412 | 0.002354875 |
| ITGA3 | -0.426276489 | 0.008899264 | -3.058640754 | 0.002355689 |
| CTD-2054N24.2 | -0.267018227 | 2.63260443 | -3.058636019 | 0.002355725 |
| HCFC2 | 0.132456734 | 3.772719843 | 3.058346392 | 0.002357957 |
| TCEAL2 | 0.407445792 | -1.946524266 | 3.05831272 | 0.002358216 |
| SCCPDH | -0.240316115 | 7.163900073 | -3.058300022 | 0.002358314 |
| SF3B5 | -0.123948616 | 8.155217949 | -3.058039187 | 0.002360326 |
| BTLA | -0.477668495 | 1.100100104 | -3.05764939 | 0.002363335 |
| ABI3 | -0.477756664 | 3.394149078 | -3.056975741 | 0.002368543 |
| RP11-359J14.2 | -0.334955627 | 1.435142746 | -3.056936743 | 0.002368845 |
| ZNF747 | -0.145133819 | 3.220746949 | -3.05688914 | 0.002369213 |
| RN7SL1 | -0.266930789 | 5.166311008 | -3.056140969 | 0.002375012 |
| AP000769.1 | -0.336584729 | 0.785719609 | -3.056045481 | 0.002375753 |
| SNORA11 | 0.237403211 | 1.245005334 | 3.055499894 | 0.00237999 |
| SDCCAG3P1 | -0.28891858 | 0.381874187 | -3.055195323 | 0.002382359 |
| VAC14 | -0.128956831 | 4.446533179 | -3.055076745 | 0.002383282 |
| GRPR | -0.323068962 | -0.755122447 | -3.055029977 | 0.002383646 |
| NIPA2 | -0.11057422 | 6.247605698 | -3.054922094 | 0.002384486 |
| SAT1 | 0.293865628 | 9.867465155 | 3.054519936 | 0.00238762 |
| LINC00657 | -0.150561624 | 8.243811552 | -3.053910363 | 0.002392377 |
| AP001007.1 | -0.614241546 | 2.029746184 | -3.053876256 | 0.002392643 |
| CASP3 | -0.250745306 | 6.469017046 | -3.053076474 | 0.002398898 |
| AQP10 | 0.516434202 | -1.467741251 | 3.052862691 | 0.002400573 |
| CYP4F12 | 0.524509122 | -2.052550567 | 3.052831372 | 0.002400818 |
| KIAA1522 | -0.315873481 | 4.189556567 | -3.052517845 | 0.002403276 |
| ARSA | -0.224219037 | 6.031463586 | -3.052216539 | 0.00240564 |
| MIR3176 | -0.35646559 | 2.289175852 | -3.052036496 | 0.002407054 |
| CLIP1 | 0.156292728 | 5.344647132 | 3.05172343 | 0.002409515 |
| TLE1 | 0.441737524 | 3.050799221 | 3.051343914 | 0.0024125 |
| MAP3K10 | -0.176472032 | 3.453979889 | -3.050105351 | 0.002422267 |
| RP1-89D4.1 | -0.188356039 | 6.515560915 | -3.050032576 | 0.002422842 |
| TAL1 | 0.728271311 | 3.028370607 | 3.049889284 | 0.002423975 |
| LINC00900 | -0.74960152 | -1.473955422 | -3.049862045 | 0.00242419 |
| AC006547.8 | 0.322533899 | 0.037484474 | 3.049815836 | 0.002424555 |
| MAP7D2 | 0.4807699 | -1.218197936 | 3.049435953 | 0.002427561 |
| DNM1P47 | -0.345101248 | -0.009867474 | -3.04908276 | 0.002430358 |
| DGKD | 0.163873249 | 6.17153412 | 3.048919551 | 0.002431652 |
| AC006160.5 | -0.291910442 | 0.004023662 | -3.048522787 | 0.0024348 |
| MYCBPAP | -0.429900044 | 1.451668844 | -3.048365639 | 0.002436048 |
| MS4A2 | -0.719841552 | 1.25310992 | -3.046898169 | 0.002447727 |
| CTSZ | -0.280684324 | 9.066644146 | -3.046823619 | 0.002448322 |
| C21orf91-OT1 | -0.385126715 | -0.780695676 | -3.046653814 | 0.002449677 |
| RP11-584P21.4 | -0.198671849 | 3.162130454 | -3.046557878 | 0.002450443 |
| HIST2H2BF | -0.390609464 | 2.424093758 | -3.046170974 | 0.002453534 |
| ASAH2 | 0.37687595 | -4.05305734 | 3.04594244 | 0.002455362 |
| FAM53B | 0.160200019 | 5.880480318 | 3.045737959 | 0.002456998 |
| RNVU1-18 | -0.395641192 | -0.448291012 | -3.044972094 | 0.002463136 |
| IFNLR1 | 0.472046756 | -0.822364891 | 3.044964026 | 0.0024632 |
| RBM5 | -0.119074872 | 6.281644971 | -3.044901669 | 0.002463701 |
| ZNF223 | -0.314700037 | 0.03922884 | -3.044545944 | 0.002466557 |
| PPP1R18 | 0.216989236 | 7.700692498 | 3.044276223 | 0.002468724 |
| GCSHP5 | -0.264272361 | 5.138890592 | -3.043877046 | 0.002471935 |
| ALAS1 | 0.239967937 | 6.815743606 | 3.0438062 | 0.002472506 |
| RP11-70C1.1 | -0.438705453 | 1.001113798 | -3.04361605 | 0.002474037 |
| RHOBTB2 | -0.251057066 | 3.657122797 | -3.043559494 | 0.002474492 |
| CNGA4 | -0.331454598 | 0.015914821 | -3.043324139 | 0.002476389 |
| ANKRD65 | 0.664248762 | -1.31899998 | 3.042908916 | 0.002479739 |
| CHAC1 | 0.431388066 | -0.107538795 | 3.041431707 | 0.00249169 |
| RP11-274K13.5 | -0.614152509 | -1.403551555 | -3.041427005 | 0.002491728 |
| MFF | 0.082912388 | 5.592138214 | 3.041282831 | 0.002492897 |
| RP11-345P4.10 | -0.328982271 | 0.758120765 | -3.041245953 | 0.002493197 |
| KCTD9P1 | 0.219358852 | 2.414244134 | 3.040816439 | 0.002496683 |
| RP11-33A14.1 | -0.491361972 | -1.956963591 | -3.040680771 | 0.002497786 |
| CLK1 | 0.245406203 | 7.761997102 | 3.040029088 | 0.002503087 |
| RP11-391M1.4 | -0.21368176 | 2.386456632 | -3.039810362 | 0.002504868 |
| PRKD1 | 0.462236086 | -3.603714248 | 3.039723937 | 0.002505572 |
| CTD-3126B10.1 | 0.38736316 | -1.114991532 | 3.039610615 | 0.002506496 |
| RP11-661A12.7 | 0.384328795 | 1.467756441 | 3.038556062 | 0.002515107 |
| RP11-2B6.3 | -0.267778942 | 0.811587432 | -3.038103159 | 0.002518813 |
| IMPDH2 | -0.265726274 | 8.570032023 | -3.038049515 | 0.002519252 |
| BOK | -0.433979118 | -1.461975657 | -3.037504551 | 0.002523719 |
| RPL36 | 0.155502106 | 9.526210776 | 3.037310134 | 0.002525315 |
| UNCX | -0.684563638 | -1.913394609 | -3.036766737 | 0.002529779 |
| DHX38 | 0.085976167 | 6.32874325 | 3.036710256 | 0.002530243 |
| CCND2-AS1 | -0.33407102 | -0.354383601 | -3.036451058 | 0.002532375 |
| RP11-697E2.7 | 0.169863285 | 3.417217474 | 3.035983817 | 0.002536223 |
| IL21R | 0.411270145 | 1.714735469 | 3.035737471 | 0.002538254 |
| CTC-499B15.6 | -0.348955245 | -0.513967748 | -3.035387685 | 0.00254114 |
| CREBZF | -0.149784465 | 6.149047117 | -3.0351394 | 0.002543191 |
| AC073342.12 | -0.356433876 | -0.462007858 | -3.034428252 | 0.002549073 |
| HSPD1P5 | -0.290545724 | 0.354576588 | -3.033848191 | 0.002553879 |
| RP11-571M6.17 | -0.251981193 | 1.002767052 | -3.033840283 | 0.002553945 |
| GPR89A | 0.113186264 | 2.576266099 | 3.033740625 | 0.002554771 |
| AC091729.9 | -0.263275785 | 2.363150639 | -3.033652096 | 0.002555506 |
| FOXP1 | 0.108517349 | 5.85743264 | 3.033594082 | 0.002555987 |
| SLC10A5 | -0.414273275 | -0.092555171 | -3.03352474 | 0.002556563 |
| CTB-35F21.1 | -0.403997377 | -0.837517549 | -3.033364761 | 0.002557891 |
| SDHAP3 | 0.302743344 | 3.656645434 | 3.033201269 | 0.00255925 |
| CHCHD2P9 | 0.201644033 | 4.365872219 | 3.032937815 | 0.00256144 |
| RP4-758J24.5 | 0.209070263 | 3.68995241 | 3.032552092 | 0.002564649 |
| AC027763.2 | -0.151125491 | 3.619653836 | -3.03241154 | 0.00256582 |
| CTD-3105H18.13 | -0.350726869 | -1.504770401 | -3.032237273 | 0.002567272 |
| STAG3L5P | -0.209141641 | 4.266789315 | -3.03188417 | 0.002570216 |
| FAM213A | 0.621849193 | 2.478335564 | 3.031586214 | 0.002572703 |
| MSL3P1 | -0.505207396 | 2.378284927 | -3.03140256 | 0.002574236 |
| ZNF784 | 0.146761045 | 4.347472975 | 3.030199319 | 0.002584307 |
| ARTN | -0.672383537 | 2.11386349 | -3.029515363 | 0.002590047 |
| SETD6 | -0.165662354 | 3.812343976 | -3.029339093 | 0.002591528 |
| POLR2G | -0.081380061 | 6.844877687 | -3.028873706 | 0.002595443 |
| CMTM8 | 0.405144977 | 0.748727242 | 3.028713122 | 0.002596795 |
| ANGPT1 | -0.736720684 | 4.273009086 | -3.028377429 | 0.002599623 |
| RP11-427H3.3 | -0.18399367 | 5.338643002 | -3.028289265 | 0.002600367 |
| ANKRD44 | -0.201049768 | 4.723520748 | -3.028151255 | 0.002601531 |
| RP11-785H5.2 | 0.296670467 | 1.260337123 | 3.028151101 | 0.002601532 |
| TBC1D3F | -0.242389108 | 2.219251386 | -3.027937623 | 0.002603333 |
| RP11-589C21.5 | 0.398279261 | -0.688047082 | 3.027587052 | 0.002606294 |
| AKR1C7P | 0.339355373 | 0.691878129 | 3.027091722 | 0.002610482 |
| FDPSP1 | -0.234680221 | 0.872835233 | -3.026963885 | 0.002611564 |
| BTG1 | 0.282152158 | 9.082417893 | 3.026407419 | 0.002616279 |
| ZNF808 | -0.144394994 | 4.721513014 | -3.025500046 | 0.002623983 |
| CTC-332L22.1 | 0.32710051 | 0.526248531 | 3.025348352 | 0.002625273 |
| TIMM9 | -0.12424467 | 5.906454742 | -3.025002791 | 0.002628214 |
| GALNS | -0.18942144 | 4.547682076 | -3.024659707 | 0.002631137 |
| RP13-870H17.3 | 0.486263252 | -0.033998783 | 3.024318425 | 0.002634047 |
| PLXND1 | -0.272016844 | 7.021234899 | -3.024167408 | 0.002635336 |
| ZNF561 | -0.145333617 | 4.31816851 | -3.023624616 | 0.002639973 |
| SNRNP35 | 0.100388133 | 4.218043704 | 3.023537384 | 0.002640719 |
| TCERG1 | 0.12465616 | 6.190081673 | 3.023208083 | 0.002643537 |
| TMEM191C | -0.315409006 | -0.314321995 | -3.022953318 | 0.002645718 |
| PITPNM2 | 0.273481743 | 3.543354236 | 3.022798449 | 0.002647045 |
| LCN2 | 0.917173662 | 5.102090144 | 3.022694852 | 0.002647934 |
| ENTPD5 | -0.158039842 | 3.365180506 | -3.022477864 | 0.002649794 |
| LRRC26 | -0.487282319 | -0.794630658 | -3.022473611 | 0.002649831 |
| GUSBP9 | -0.307979063 | 2.809449703 | -3.022275391 | 0.002651532 |
| MED22 | -0.087585326 | 4.661168717 | -3.022242496 | 0.002651814 |
| KB-1471A8.1 | -0.424431426 | -1.713534626 | -3.022187348 | 0.002652288 |
| RGS9 | 0.484570053 | -0.209119018 | 3.022155726 | 0.002652559 |
| RP3-418A9.3 | -0.267641819 | 1.873254592 | -3.022039634 | 0.002653556 |
| GS1-421I3.4 | -0.405193782 | -0.44550678 | -3.021725247 | 0.002656258 |
| GPR52 | 0.375197673 | -0.775798232 | 3.021662236 | 0.0026568 |
| RPL27A | 0.164536369 | 8.132675214 | 3.021269903 | 0.002660176 |
| THOC7 | -0.118889268 | 7.203434639 | -3.020264345 | 0.002668847 |
| LINC00607 | -0.492335077 | -3.707803111 | -3.020018531 | 0.00267097 |
| RP11-600F24.1 | 0.272433012 | 1.381416478 | 3.019738817 | 0.002673388 |
| SAMM50 | -0.121158859 | 4.829611624 | -3.019316298 | 0.002677045 |
| PRDM5 | 0.697293157 | -2.407425918 | 3.018916936 | 0.002680505 |
| SHISA7 | 0.592491983 | -3.174405252 | 3.018913994 | 0.002680531 |
| RP11-488C13.6 | 0.295106554 | 0.986453911 | 3.018910064 | 0.002680565 |
| DERL3 | 0.303916179 | 3.395709889 | 3.018688049 | 0.002682491 |
| DYTN | -0.581062374 | 1.454455592 | -3.018317127 | 0.00268571 |
| RP11-405O10.2 | 0.349915668 | 0.578182703 | 3.017836472 | 0.002689888 |
| AC073310.4 | 0.268509057 | 0.383759096 | 3.017805937 | 0.002690154 |
| IL10RB-AS1 | -0.416393813 | 1.376901835 | -3.017277424 | 0.002694755 |
| NPTN | -0.182069654 | 6.111350711 | -3.017181126 | 0.002695594 |
| CTSB | -0.314679429 | 7.680672668 | -3.016542352 | 0.002701166 |
| CTB-11I22.2 | 0.45849397 | -0.453408402 | 3.016508831 | 0.002701459 |
| GPCPD1 | 0.209510748 | 6.526829116 | 3.016222517 | 0.00270396 |
| LINC00883 | -0.456131808 | 0.473237987 | -3.016024798 | 0.002705688 |
| RP11-540B6.3 | -0.409169959 | 1.916823686 | -3.015661067 | 0.002708871 |
| DPY19L1P2 | -0.307310281 | 0.872472822 | -3.015440489 | 0.002710802 |
| TCF7L2 | 0.297452818 | 4.527991807 | 3.015319999 | 0.002711858 |
| ANKH | 0.495111141 | 2.196452142 | 3.015151747 | 0.002713333 |
| JAK1 | -0.140779156 | 7.829681466 | -3.014591942 | 0.002718245 |
| SMS | 0.117428859 | 6.155818353 | 3.014435145 | 0.002719622 |
| SNORD3C | -0.484963005 | -0.081376807 | -3.014215775 | 0.00272155 |
| PGBD4P1 | -0.815462627 | 1.232172998 | -3.013963666 | 0.002723767 |
| RP11-516A11.1 | 0.168728831 | 4.55326914 | 3.013937915 | 0.002723994 |
| FTCDNL1 | -0.348515062 | 2.63652882 | -3.013590425 | 0.002727053 |
| RP11-797H7.5 | 0.440147389 | 0.343815161 | 3.013267544 | 0.002729898 |
| C3AR1 | -0.520384353 | 5.98472051 | -3.01291391 | 0.002733018 |
| EBAG9 | -0.112781484 | 4.887757266 | -3.012878589 | 0.00273333 |
| RP11-405F3.4 | -0.49952565 | 0.545362185 | -3.012741591 | 0.002734539 |
| AP1B1 | -0.150595084 | 6.755758582 | -3.011522616 | 0.002745322 |
| XXbac-B444P24.10 | -0.469254586 | -1.012815616 | -3.011512677 | 0.00274541 |
| ABCA7 | -0.240644395 | 6.134054225 | -3.01151239 | 0.002745413 |
| RP11-1114A5.4 | -0.285579183 | 0.836284661 | -3.011254734 | 0.002747697 |
| RPL7P46 | -0.27371516 | 0.687944936 | -3.011163735 | 0.002748504 |
| TEX21P | -0.277661154 | 0.596063471 | -3.010645982 | 0.002753101 |
| LRRC37BP1 | -0.158876591 | 3.576615655 | -3.010087951 | 0.002758063 |
| TEKT2 | 0.440881298 | 1.156035741 | 3.009966414 | 0.002759145 |
| CDNF | 0.269581457 | 1.294943818 | 3.009940456 | 0.002759376 |
| SLC39A7 | 0.099445875 | 6.662877903 | 3.009722218 | 0.00276132 |
| TBP | 0.084684789 | 5.7420035 | 3.00907212 | 0.002767118 |
| RPL15P3 | -0.149935653 | 10.27237504 | -3.00862439 | 0.002771117 |
| C3orf80 | -0.780142381 | 2.588759453 | -3.008474446 | 0.002772458 |
| WDR45 | 0.128753821 | 4.532543742 | 3.00840329 | 0.002773094 |
| CYB5R2 | 0.734909036 | -2.968378392 | 3.008304704 | 0.002773976 |
| MATK | 0.288713806 | 5.046753878 | 3.008295025 | 0.002774062 |
| RP11-211N8.2 | -0.397700732 | -1.564742279 | -3.008010087 | 0.002776613 |
| RP11-1191J2.2 | 0.42168935 | -1.24242835 | 3.007953361 | 0.002777121 |
| ST8SIA4 | -0.245696695 | 4.6665352 | -3.007812174 | 0.002778386 |
| IGHJ1 | 0.644408138 | 1.361828527 | 3.007433004 | 0.002781785 |
| AAED1 | 0.276789969 | 5.257696523 | 3.007205359 | 0.002783827 |
| DUTP6 | -0.296174291 | 0.936191347 | -3.007158622 | 0.002784247 |
| CDC26 | -0.171201972 | 4.302689809 | -3.006691295 | 0.002788445 |
| LY6G6F | 0.519322887 | -1.414031928 | 3.006409044 | 0.002790984 |
| RP11-727F15.13 | 0.28257941 | 1.013490435 | 3.006188826 | 0.002792965 |
| PPP1R14BP3 | -0.216519379 | 7.891891738 | -3.005815808 | 0.002796326 |
| CTC-444N24.9 | 0.171716403 | 2.920122789 | 3.0054391 | 0.002799723 |
| PTGS2 | 0.570553952 | 5.454118056 | 3.005370539 | 0.002800341 |
| PEG10 | 0.473048697 | -1.56794519 | 3.004302858 | 0.002809992 |
| RPS15 | 0.153111741 | 9.143646008 | 3.00425649 | 0.002810412 |
| ERN1 | 0.204978461 | 4.518516324 | 3.003965692 | 0.002813046 |
| SRPK1 | 0.192251631 | 6.11645738 | 3.003638136 | 0.002816016 |
| RP4-706A16.3 | -0.15003255 | 9.499396899 | -3.003310849 | 0.002818986 |
| LRRC49 | -0.564431451 | -2.504653701 | -3.003192846 | 0.002820058 |
| GPR146 | 0.408732996 | 2.40425045 | 3.002180873 | 0.002829263 |
| ARID4B-IT1 | 0.299156205 | 1.173176771 | 3.002173583 | 0.002829329 |
| ZFYVE9 | 0.60396916 | 1.511975167 | 3.001108979 | 0.002839044 |
| CTD-2555A7.2 | -0.462740438 | -1.477483533 | -3.001068628 | 0.002839412 |
| RP11-247L20.4 | -0.255747086 | 2.311759923 | -3.000938692 | 0.0028406 |
| RP11-290F24.6 | -0.324586046 | 0.493413303 | -3.000884768 | 0.002841093 |
| CEACAM8 | 0.884881822 | 5.543067925 | 3.000852463 | 0.002841389 |
| IGHJ5 | 0.494986101 | 2.849621008 | 2.999044661 | 0.002857967 |
| HDGFP1 | 0.41187479 | 0.513407857 | 2.998699491 | 0.002861142 |
| HSD17B13 | 0.302635437 | 1.514956539 | 2.998436387 | 0.002863565 |
| CCNT2-AS1 | -0.178908237 | 2.051568381 | -2.9982291 | 0.002865475 |
| RPL4P3 | 0.220791812 | 2.850485443 | 2.998082083 | 0.00286683 |
| RNU1-16P | 0.316591252 | 0.490346152 | 2.997853267 | 0.00286894 |
| CNTLN | 0.233020111 | 3.221267372 | 2.996625978 | 0.002880285 |
| RP11-925D8.5 | 0.5374268 | -0.925226442 | 2.99640442 | 0.002882338 |
| RANBP6 | 0.209207379 | 5.996294795 | 2.996335133 | 0.00288298 |
| PAXIP1-AS1 | 0.222703829 | 4.585253492 | 2.996103899 | 0.002885124 |
| CCDC87 | -0.387924453 | -2.122613317 | -2.995804338 | 0.002887903 |
| TMEM208 | -0.114583887 | 4.958755399 | -2.995678749 | 0.002889069 |
| IFT57 | 0.187536419 | 5.524469451 | 2.99542792 | 0.002891399 |
| CTB-41I6.1 | -0.266370406 | 3.522640429 | -2.995082891 | 0.002894607 |
| AC004158.2 | 0.264371706 | 0.08433981 | 2.993731832 | 0.002907201 |
| RP11-38L15.3 | -0.422907194 | -0.431483992 | -2.992486562 | 0.002918852 |
| CASP3P1 | -0.356025823 | -0.087723805 | -2.992327798 | 0.002920341 |
| FER1L4 | 0.444967819 | -2.155643625 | 2.991297412 | 0.002930019 |
| SOCS3 | 0.492171862 | 7.174359037 | 2.990646559 | 0.002936147 |
| RP11-690I21.2 | -0.301158413 | -1.011692619 | -2.989149676 | 0.002950286 |
| RPL39P3 | -0.141893257 | 10.93214752 | -2.989126863 | 0.002950502 |
| RP11-15I11.3 | -0.428469077 | -0.541747451 | -2.988504316 | 0.002956401 |
| C8orf58 | 0.156358937 | 3.101841845 | 2.988383668 | 0.002957546 |
| ARHGEF9 | -0.138192018 | 3.752933689 | -2.98837565 | 0.002957622 |
| RAP1GAP | 0.574617272 | -0.124444945 | 2.988297135 | 0.002958367 |
| MAP2K2 | 0.095798036 | 6.225955047 | 2.988225122 | 0.00295905 |
| PWP2 | -0.147840913 | 4.175235457 | -2.988160566 | 0.002959663 |
| SPIRE1 | 0.505337936 | 1.88320067 | 2.987767092 | 0.002963401 |
| AP001619.2 | -0.336654662 | 0.184272391 | -2.987262385 | 0.002968201 |
| SCGB2B2 | 0.325184797 | -0.078800401 | 2.986605842 | 0.002974457 |
| ZNF474 | -0.4347531 | -1.221245331 | -2.986524673 | 0.002975231 |
| CTD-2037K23.2 | -0.173552381 | 4.919706722 | -2.986122899 | 0.002979066 |
| ITGA2B | 0.574632793 | 4.533242179 | 2.986112034 | 0.00297917 |
| TBC1D30 | -0.351263358 | -0.169764651 | -2.985492494 | 0.002985093 |
| NEGR1 | -0.745988069 | 2.331060227 | -2.985385619 | 0.002986116 |
| TANC2 | 0.336122123 | 3.707542732 | 2.984888345 | 0.002990879 |
| RUNDC3A | 0.513265031 | -0.746084138 | 2.983984506 | 0.002999554 |
| ZNF333 | 0.104210361 | 4.005610149 | 2.983810407 | 0.003001228 |
| AL354822.1 | -0.67396777 | 1.946668424 | -2.983568884 | 0.003003551 |
| NLK | -0.210724976 | 5.082327444 | -2.983253253 | 0.003006589 |
| NPM1P39 | -0.200828223 | 8.308589685 | -2.983209876 | 0.003007007 |
| RP11-466P24.4 | -0.207052448 | 2.340233217 | -2.983013771 | 0.003008897 |
| UBL5P2 | -0.227292918 | 1.962658686 | -2.982845264 | 0.003010521 |
| SPP1 | 0.73754573 | 1.638772602 | 2.982503436 | 0.003013819 |
| RP11-287D1.3 | -0.308778117 | -1.689169136 | -2.982257309 | 0.003016196 |
| RP11-848G14.2 | -0.414060576 | -0.128091452 | -2.981883765 | 0.003019806 |
| TELO2 | -0.156238887 | 5.725696738 | -2.981213112 | 0.003026298 |
| RP11-629O1.2 | 0.237097189 | 3.473701643 | 2.981141058 | 0.003026996 |
| RP11-151N17.1 | 0.328094457 | 0.638958677 | 2.980923322 | 0.003029107 |
| AC007879.5 | 0.302329479 | 1.014907196 | 2.980567107 | 0.003032563 |
| CTA-363E6.6 | 0.525740522 | -0.723237837 | 2.980100685 | 0.003037094 |
| RP1-90L6.2 | -0.370343546 | -0.103495591 | -2.9798963 | 0.003039082 |
| PHF19 | 0.140990025 | 5.530005308 | 2.979890115 | 0.003039142 |
| RP11-327F22.1 | -0.306970069 | 1.517018948 | -2.97922553 | 0.003045613 |
| RP11-167N5.5 | -0.291249331 | -0.430641759 | -2.979089032 | 0.003046943 |
| RP11-395I14.2 | 0.272171889 | 1.017635942 | 2.977948309 | 0.003058084 |
| RP11-66B24.2 | -0.406829275 | -1.876212803 | -2.977898016 | 0.003058576 |
| MIR1282 | 0.458628563 | 1.918325678 | 2.977790186 | 0.003059631 |
| PGAM1 | 0.22331275 | 6.350657469 | 2.977633706 | 0.003061163 |
| RP11-539L10.3 | -0.273508721 | 2.275848332 | -2.977071523 | 0.003066672 |
| PPHLN1 | -0.076712625 | 5.27797249 | -2.97690433 | 0.003068312 |
| PAX8-AS1 | -0.946655223 | 1.295162712 | -2.975372613 | 0.003083375 |
| DEFA8P | 0.74836848 | 0.873371012 | 2.975008618 | 0.003086965 |
| AKR1E2 | 0.456029401 | 1.361672923 | 2.974985093 | 0.003087197 |
| POLN | -0.295362533 | -0.733991149 | -2.974761759 | 0.003089402 |
| RP5-894A10.2 | -0.284332646 | 3.311022235 | -2.974123466 | 0.00309571 |
| SSR1 | -0.122320592 | 6.647048971 | -2.97407991 | 0.003096141 |
| SBF2-AS1 | 0.274444609 | 1.761191403 | 2.973630881 | 0.003100586 |
| RPL5P5 | 0.194962229 | 3.413524479 | 2.973450688 | 0.003102372 |
| ATP13A2 | -0.185738872 | 5.51273243 | -2.97338673 | 0.003103006 |
| RP11-447H19.3 | -0.286141882 | -0.432293106 | -2.973018876 | 0.003106655 |
| RPS10P28 | 0.28417524 | 1.190034009 | 2.972793763 | 0.00310889 |
| MSN | -0.121388314 | 10.37848849 | -2.972562432 | 0.003111188 |
| MFN1 | -0.09723539 | 4.6232136 | -2.972544922 | 0.003111362 |
| EHD1 | 0.226674754 | 6.358813597 | 2.972487588 | 0.003111932 |
| SLA | 0.342615182 | 5.813046494 | 2.972194842 | 0.003114843 |
| KCTD18 | 0.100185671 | 4.998199793 | 2.972040299 | 0.003116381 |
| NETO1 | -0.760403705 | -2.460539242 | -2.971983616 | 0.003116945 |
| ACSF2 | -0.222853846 | 3.972074248 | -2.971862738 | 0.003118148 |
| RAB33B | 0.192538851 | 4.643387201 | 2.970575901 | 0.003130987 |
| VPS26A | 0.098619724 | 6.429987096 | 2.969847289 | 0.003138277 |
| GPR19 | 0.291687438 | -0.342322754 | 2.969725574 | 0.003139497 |
| INIP | -0.182236615 | 4.448989819 | -2.969388228 | 0.003142879 |
| TSPAN18 | 0.421811182 | 1.041105702 | 2.969125134 | 0.003145519 |
| KCNA6 | -0.418661727 | -2.778818384 | -2.968769864 | 0.003149087 |
| TNFSF8 | 0.446607483 | 4.70661944 | 2.968680986 | 0.00314998 |
| HYI | 0.228259705 | 4.847596235 | 2.968561908 | 0.003151177 |
| FAM73B | 0.106940625 | 4.707444116 | 2.967952881 | 0.003157306 |
| IRF6 | 0.501185762 | -0.97843263 | 2.967543357 | 0.003161433 |
| RNU6ATAC36P | -0.412813582 | -0.168282244 | -2.967319676 | 0.00316369 |
| RP11-479O9.2 | -0.310196805 | -0.038471738 | -2.967298189 | 0.003163907 |
| PMS2P4 | 0.172111884 | 2.40212453 | 2.966903963 | 0.003167887 |
| TXNRD1 | 0.130293561 | 6.374120291 | 2.966752665 | 0.003169416 |
| DYNLT1 | 0.20876088 | 5.683620624 | 2.966617149 | 0.003170786 |
| MED18 | -0.175565906 | 3.922009852 | -2.966284259 | 0.003174154 |
| HMGB3P24 | 0.330194585 | -0.431962471 | 2.965366448 | 0.003183456 |
| PPFIBP1 | -0.359441737 | 3.367800264 | -2.965349702 | 0.003183626 |
| EXO5 | 0.128604467 | 3.908064026 | 2.96469995 | 0.003190227 |
| MAP4K5 | 0.153061122 | 5.490748184 | 2.964497657 | 0.003192284 |
| RPL3P6 | 0.274340796 | 1.718124808 | 2.964322811 | 0.003194064 |
| CPSF7 | -0.083523075 | 7.045082717 | -2.963905024 | 0.003198319 |
| RP11-16E18.1 | -0.316797309 | -1.191661433 | -2.963882855 | 0.003198545 |
| AC005229.7 | -0.347014806 | 0.604345059 | -2.963364435 | 0.003203833 |
| AC005618.6 | -0.443264959 | -1.32551663 | -2.962895641 | 0.003208622 |
| RP11-710M11.1 | 0.188659585 | 1.635551846 | 2.962759888 | 0.00321001 |
| SLC39A5 | -0.297102204 | -1.862247193 | -2.961993213 | 0.003217858 |
| PLCD1 | 0.267492566 | 3.448444241 | 2.961464121 | 0.003223285 |
| XRCC6 | 0.106858226 | 8.579635152 | 2.960107541 | 0.003237238 |
| RP11-747H7.3 | -0.503193936 | -0.597836184 | -2.959337795 | 0.003245179 |
| RP3-522J7.6 | -0.375065292 | 0.107023707 | -2.959115797 | 0.003247473 |
| RPS7P11 | -0.135316721 | 9.699441951 | -2.958988129 | 0.003248792 |
| LITAF | 0.261869766 | 7.571554375 | 2.958856607 | 0.003250152 |
| RPSAP54 | 0.224029924 | 5.206304749 | 2.958508641 | 0.003253753 |
| VMAC | -0.145770187 | 4.438579088 | -2.957881212 | 0.003260255 |
| FGF7 | 0.5848098 | -2.704780722 | 2.957610065 | 0.003263069 |
| TNFSF12 | -0.427377739 | 2.710734649 | -2.957402779 | 0.003265221 |
| RCC2P6 | 0.192047319 | 3.068517992 | 2.957047455 | 0.003268913 |
| OXCT1 | 0.223571191 | 4.906835554 | 2.956998208 | 0.003269426 |
| IPO4 | 0.261563742 | -1.049543516 | 2.95679783 | 0.00327151 |
| RAB5B | -0.190973483 | 4.700990961 | -2.956740796 | 0.003272103 |
| RP11-321E2.4 | -0.446458262 | -1.356603771 | -2.956365877 | 0.003276007 |
| IDH3A | -0.150775119 | 5.049727214 | -2.955927244 | 0.00328058 |
| LINC00629 | -0.391151331 | -1.32441153 | -2.955696613 | 0.003282986 |
| ARHGEF18 | -0.151223859 | 5.08904006 | -2.955618879 | 0.003283798 |
| LINC00341 | -0.253442216 | 5.539691274 | -2.955214552 | 0.003288022 |
| NOG | -0.618310485 | 2.690484644 | -2.954980948 | 0.003290465 |
| MROH8 | -0.257319087 | 0.847055571 | -2.954754791 | 0.003292831 |
| FZD8 | 0.50424545 | -0.272431587 | 2.95469982 | 0.003293406 |
| TAS2R30 | 0.363587113 | -0.719561104 | 2.95439745 | 0.003296573 |
| KLHL7 | -0.13029 | 3.295737853 | -2.954225421 | 0.003298376 |
| FAM174B | -0.374269782 | 1.163443506 | -2.954086603 | 0.003299832 |
| SEC1P | -0.36846927 | -0.922344714 | -2.953519788 | 0.003305781 |
| AC068522.4 | 0.283754046 | 1.49552262 | 2.95350593 | 0.003305926 |
| UNC13A | -0.474028971 | -4.009234231 | -2.95265626 | 0.003314863 |
| VWDE | 0.760272488 | -1.039123401 | 2.952118673 | 0.003320529 |
| CYP2B7P | -0.641438135 | -0.475798951 | -2.952084156 | 0.003320893 |
| TADA3 | -0.132213713 | 6.447803508 | -2.951902821 | 0.003322806 |
| RP11-345J4.8 | 0.194511316 | 3.423439646 | 2.951874942 | 0.0033231 |
| BMS1P1 | -0.469386816 | -0.483841989 | -2.951475525 | 0.003327319 |
| CASD1 | -0.145869808 | 4.691616616 | -2.951424693 | 0.003327856 |
| PXK | -0.190427011 | 6.401303224 | -2.951336102 | 0.003328792 |
| CHCHD2P6 | 0.119926535 | 5.268954917 | 2.951316922 | 0.003328995 |
| GNAS | 0.126573332 | 8.686508037 | 2.950883276 | 0.003333582 |
| HORMAD1 | -0.514078732 | 0.87946901 | -2.949857567 | 0.003344456 |
| RP4-561L24.3 | -0.318955874 | -0.296471969 | -2.949844414 | 0.003344596 |
| CPEB2-AS1 | -0.318701036 | -2.181092118 | -2.94954646 | 0.00334776 |
| RP11-73G16.2 | -0.404009289 | -1.684609784 | -2.949241849 | 0.003350999 |
| ADM5 | 0.291974462 | 2.699788175 | 2.949236416 | 0.003351057 |
| TMEM133 | 0.44089298 | -1.059450894 | 2.949044281 | 0.003353101 |
| M1AP | 0.437690274 | 2.279469672 | 2.94887923 | 0.003354858 |
| SNORD9 | 0.237217785 | 1.136032307 | 2.948833274 | 0.003355347 |
| CD37 | -0.173762457 | 8.346911788 | -2.948825811 | 0.003355426 |
| GAS2 | -0.530617015 | -1.847313116 | -2.948442498 | 0.00335951 |
| RP11-867G23.1 | -0.331572964 | -0.539839286 | -2.948394045 | 0.003360027 |
| IFNG | 0.535900216 | 1.598282765 | 2.948248345 | 0.003361581 |
| TMEM40 | 0.635495027 | -1.65914822 | 2.948065433 | 0.003363532 |
| WDFY3-AS2 | -0.473977836 | -2.441773212 | -2.946714911 | 0.003377973 |
| FAM19A5 | -0.567185766 | -2.406154679 | -2.946633701 | 0.003378844 |
| HOXB3 | -0.911829599 | 3.317963296 | -2.946331387 | 0.003382085 |
| WDR86-AS1 | -0.549570127 | 1.093736136 | -2.94619244 | 0.003383575 |
| TMEM65 | -0.224159046 | 5.177549466 | -2.945891614 | 0.003386805 |
| TYW1 | -0.108133146 | 4.68317789 | -2.945507378 | 0.003390933 |
| IFNGR1 | 0.267062456 | 7.406930139 | 2.94540593 | 0.003392024 |
| LAG3 | 0.46652153 | 2.162387791 | 2.945198492 | 0.003394256 |
| RP11-452H21.4 | 0.292922503 | 2.198658242 | 2.945053589 | 0.003395816 |
| CASC1 | -0.338117165 | -1.349254357 | -2.944673468 | 0.00339991 |
| DNAAF3 | -0.562828109 | -0.752089192 | -2.944575924 | 0.003400962 |
| SNORA50 | 0.267826778 | 1.734661643 | 2.944237258 | 0.003404614 |
| ZNF775 | -0.20236152 | 3.298555463 | -2.943996366 | 0.003407215 |
| RP11-923I11.7 | -0.407368948 | -0.46696335 | -2.943472316 | 0.003412878 |
| ALG5 | 0.10376534 | 5.371062136 | 2.943321701 | 0.003414507 |
| HMGN1P30 | 0.198092648 | 3.349491359 | 2.942734435 | 0.003420867 |
| SEMA4G | -0.373934617 | -1.388461945 | -2.94195246 | 0.003429352 |
| RILPL1 | 0.384595043 | 1.073012399 | 2.941893153 | 0.003429996 |
| TMED5 | 0.126622053 | 6.009285011 | 2.941690596 | 0.003432197 |
| SEC13 | 0.079408834 | 6.055824879 | 2.941361945 | 0.003435772 |
| HOXA1 | 0.52282288 | 1.895063648 | 2.940693725 | 0.00344305 |
| RP11-468H14.2 | 0.481513517 | -1.291940785 | 2.940522222 | 0.00344492 |
| BECN1 | 0.088450455 | 6.205269139 | 2.940510621 | 0.003445047 |
| RP11-798G7.7 | -0.320495584 | 0.212173277 | -2.940391481 | 0.003446346 |
| PCDHB15 | 0.547813346 | -1.547561639 | 2.940308637 | 0.00344725 |
| RP11-519C12.1 | 0.308971885 | 0.551556032 | 2.940274513 | 0.003447623 |
| HDAC11 | -0.203329552 | 2.06681366 | -2.939750808 | 0.003453344 |
| TERC | -0.37868052 | -0.056170627 | -2.93962299 | 0.003454741 |
| CLPP | 0.096147403 | 5.951915399 | 2.939560343 | 0.003455427 |
| RP11-2E11.5 | -0.449322905 | -2.224662885 | -2.939417016 | 0.003456995 |
| LINC01088 | -0.37905122 | -0.980820732 | -2.939187964 | 0.003459502 |
| CSGALNACT1 | 0.438744274 | 3.791095976 | 2.938811289 | 0.003463629 |
| RNF121 | -0.102719852 | 4.127998047 | -2.93862545 | 0.003465666 |
| BCL11A | -0.331841651 | 4.913875299 | -2.938020945 | 0.003472302 |
| AC004791.2 | -1.04126816 | 2.029493182 | -2.937991626 | 0.003472624 |
| SNN | 0.283346921 | 6.164743625 | 2.936698513 | 0.003486859 |
| DDX18 | -0.093292526 | 5.621354467 | -2.935650015 | 0.003498441 |
| HAUS1P2 | -0.240769185 | 0.831648357 | -2.935518134 | 0.0034999 |
| CTC-203F4.2 | -0.315634896 | 1.589686068 | -2.934956012 | 0.003506126 |
| RP11-112H10.6 | 0.165049533 | 3.212922359 | 2.934608788 | 0.003509976 |
| CYP2F1 | 0.427117426 | -2.77812208 | 2.93447558 | 0.003511455 |
| RAB37 | -0.300129332 | 7.162032227 | -2.934301861 | 0.003513383 |
| GRAPL | -0.417340815 | 0.259158489 | -2.933950915 | 0.003517283 |
| AC016739.2 | -0.140614558 | 10.59157268 | -2.93321514 | 0.003525471 |
| GOSR1 | 0.10413343 | 4.214460377 | 2.93290022 | 0.003528981 |
| RP3-337H4.9 | 0.208576657 | 2.078556354 | 2.932707657 | 0.003531129 |
| CASC10 | -0.866887882 | 0.628319417 | -2.932638341 | 0.003531902 |
| ADAMTS13 | -0.278700613 | 0.873420524 | -2.932335688 | 0.003535281 |
| PMS2P3 | 0.173110278 | 4.31294674 | 2.931743483 | 0.003541901 |
| PGAM4 | 0.30266689 | 1.709340188 | 2.931235587 | 0.003547587 |
| ARHGAP20 | 0.465493116 | -3.028647222 | 2.930998371 | 0.003550246 |
| ENO2 | 0.323739121 | 3.206541829 | 2.930984607 | 0.0035504 |
| LACTB | 0.217896152 | 5.423459224 | 2.929249998 | 0.003569898 |
| CTD-2651B20.1 | -0.312095389 | 0.195363985 | -2.92903378 | 0.003572335 |
| NDUFS7 | 0.116611817 | 4.332309018 | 2.928786068 | 0.003575129 |
| POLR2J3 | 0.2047183 | 1.971308108 | 2.928629225 | 0.003576899 |
| NEK10 | -0.607291863 | -1.871251468 | -2.928389388 | 0.003579607 |
| NEFL | -0.494671734 | -1.789212883 | -2.928261182 | 0.003581056 |
| MIR573 | 0.23127276 | 2.706509403 | 2.928036698 | 0.003583594 |
| RP11-3D4.3 | -0.314621498 | 1.645000722 | -2.92761036 | 0.003588418 |
| ZMAT5 | -0.13284069 | 4.095200772 | -2.927593934 | 0.003588604 |
| MXD4 | 0.162839465 | 6.116394308 | 2.927233347 | 0.003592688 |
| SLC22A4 | 0.333707679 | 3.711211163 | 2.926856913 | 0.003596957 |
| BTBD1 | 0.08536834 | 6.580234141 | 2.92623582 | 0.003604011 |
| RP11-38J22.3 | -0.480246212 | -0.663397146 | -2.925942788 | 0.003607343 |
| SLC26A7 | -0.426436241 | -3.097158225 | -2.925527165 | 0.003612074 |
| ARPC3P1 | -0.155309235 | 4.733797338 | -2.925441487 | 0.00361305 |
| P2RX4 | 0.188587978 | 4.869386266 | 2.925408622 | 0.003613424 |
| LINC01091 | 0.495084107 | -3.608570129 | 2.925062402 | 0.003617371 |
| KIF1B | 0.249864942 | 4.366546624 | 2.924423365 | 0.003624666 |
| HIST1H3E | -0.485736155 | -0.378147965 | -2.924011167 | 0.003629379 |
| VIMP | 0.129945273 | 5.10012645 | 2.923393595 | 0.00363645 |
| EBNA1BP2 | -0.154901168 | 5.544136241 | -2.922997613 | 0.00364099 |
| TM6SF1 | -0.418368814 | 3.482934809 | -2.922985625 | 0.003641128 |
| CTDNEP1 | -0.077693558 | 7.244168975 | -2.922966717 | 0.003641345 |
| MST4 | -0.177452217 | 7.64174855 | -2.922814256 | 0.003643095 |
| RP4-633O19__A.1 | -0.292407793 | 0.453828114 | -2.922012655 | 0.003652307 |
| ETV7 | 0.516235565 | -0.281273842 | 2.921818929 | 0.003654536 |
| MRRFP1 | -0.264861108 | -0.270483991 | -2.921725974 | 0.003655607 |
| AUH | 0.151954527 | 4.692856786 | 2.921328329 | 0.003660188 |
| NDUFS1 | -0.118287228 | 5.122675866 | -2.921246188 | 0.003661135 |
| ZSWIM4 | 0.2092915 | 3.720387841 | 2.920142069 | 0.003673887 |
| DPRXP2 | -0.442459861 | 0.201248221 | -2.919542642 | 0.003680827 |
| RP4-545C24.1 | -0.463051252 | 0.049455445 | -2.919220029 | 0.003684567 |
| KLC3 | 0.425914329 | -3.1062072 | 2.919146134 | 0.003685424 |
| PURB | 0.140985657 | 5.82210965 | 2.91907356 | 0.003686266 |
| GNA14 | -0.508511411 | -0.436188035 | -2.918987364 | 0.003687266 |
| XPO6 | 0.158135881 | 6.863374464 | 2.918566444 | 0.003692155 |
| CDH11 | 0.640192916 | -4.698218903 | 2.918461156 | 0.003693378 |
| TXNP5 | -0.185988733 | 6.096978309 | -2.918280774 | 0.003695475 |
| C11orf65 | -0.283577571 | -1.169482156 | -2.918036035 | 0.003698323 |
| TYROBP | -0.299931952 | 9.485979209 | -2.917890274 | 0.003700019 |
| ENTPD4 | 0.154079504 | 5.917378807 | 2.917814816 | 0.003700898 |
| ACRC | -0.213829144 | 1.936855594 | -2.917489265 | 0.003704691 |
| CUTA | -0.124123972 | 7.211445711 | -2.917399864 | 0.003705733 |
| RP11-466P24.2 | -0.193014396 | 8.783015922 | -2.917296794 | 0.003706935 |
| CLEC12B | -0.413753241 | 2.8508981 | -2.916749028 | 0.003713328 |
| AL590431.1 | 0.305624234 | 1.973937061 | 2.916391103 | 0.003717511 |
| TRIP13 | 0.301869287 | 3.30013907 | 2.916202297 | 0.003719719 |
| RP11-775J23.2 | 0.384345069 | -0.658182155 | 2.916074219 | 0.003721218 |
| LINC00683 | -0.425075634 | -2.995662036 | -2.915805344 | 0.003724366 |
| CHI3L1 | 0.807809121 | 3.919463054 | 2.91526649 | 0.003730682 |
| AC073043.1 | -0.308336079 | -2.599456834 | -2.914875994 | 0.003735266 |
| SNORA33 | -0.206154199 | 3.233258283 | -2.914729266 | 0.003736989 |
| C17orf105 | -0.336399745 | -1.802393689 | -2.913807825 | 0.003747829 |
| CYBB | -0.634329711 | 7.680180827 | -2.913646154 | 0.003749734 |
| CASC3 | 0.080292201 | 6.909285723 | 2.913366984 | 0.003753025 |
| ZNF844 | 0.28992081 | 3.757422443 | 2.913302409 | 0.003753787 |
| PRELID1P1 | 0.180053499 | 3.714706592 | 2.91289321 | 0.003758617 |
| EIF6 | -0.116379312 | 6.632393656 | -2.912365208 | 0.003764858 |
| LRRC8E | 0.450390138 | -3.368871533 | 2.912267132 | 0.003766018 |
| RP11-815J4.7 | -0.145831776 | 5.616580081 | -2.911400715 | 0.003776282 |
| NBPF8 | 0.145273402 | 3.368910804 | 2.911005559 | 0.003780972 |
| TRIM7 | -0.551266472 | -0.901240337 | -2.91094199 | 0.003781727 |
| RUFY2 | 0.113970615 | 3.547979254 | 2.91088909 | 0.003782355 |
| TNPO1P1 | 0.17983492 | 1.435828943 | 2.909181973 | 0.003802683 |
| LRRC2 | 0.50016421 | -2.290739724 | 2.908901842 | 0.003806029 |
| ANKRA2 | 0.111753124 | 5.248500828 | 2.908743077 | 0.003807926 |
| CEP152 | 0.191650215 | 4.061097555 | 2.908279875 | 0.003813465 |
| RP11-480I12.7 | -0.345887571 | -1.783719089 | -2.908071917 | 0.003815955 |
| PDCD10 | 0.112641089 | 5.866400214 | 2.90767889 | 0.003820664 |
| ETFA | -0.112053412 | 5.641225969 | -2.907647925 | 0.003821035 |
| P2RX5-TAX1BP3 | -0.381938283 | -1.13335764 | -2.90731372 | 0.003825044 |
| P4HA1 | -0.165648545 | 6.17554531 | -2.906280714 | 0.003837459 |
| ZZEF1 | 0.096585121 | 5.937725395 | 2.905147929 | 0.003851116 |
| LYRM9 | 0.203243115 | 2.261155263 | 2.905018151 | 0.003852684 |
| AL662800.1 | -0.268873988 | 2.165554154 | -2.90445776 | 0.003859458 |
| PLEK2 | 0.544996952 | -0.288175489 | 2.904083896 | 0.003863984 |
| KLHL17 | -0.225800573 | 3.352641148 | -2.903580625 | 0.003870084 |
| SELT | 0.119126758 | 6.527157889 | 2.903377935 | 0.003872544 |
| RP11-51O6.1 | -0.134780918 | 10.98812343 | -2.903300291 | 0.003873486 |
| PARVA | 0.449260991 | -3.912543507 | 2.902555885 | 0.003882532 |
| RP13-452N2.1 | -0.420315214 | -2.004379977 | -2.902517119 | 0.003883004 |
| RP11-134G8.8 | -0.450648468 | -0.122600495 | -2.902220827 | 0.00388661 |
| KCNMB4 | -0.537446003 | 0.701651138 | -2.90217651 | 0.00388715 |
| IRAK1BP1 | -0.286338128 | 1.599308678 | -2.901966111 | 0.003889713 |
| UBQLNL | -0.328592543 | 0.647240798 | -2.901770257 | 0.0038921 |
| RRAGB | -0.154144495 | 5.045634124 | -2.901726416 | 0.003892635 |
| SLC3A2 | 0.167930386 | 6.676227673 | 2.901537916 | 0.003894934 |
| USP44 | -0.661024523 | -1.189075489 | -2.900985719 | 0.003901676 |
| HDAC5 | -0.116880897 | 5.919975951 | -2.900241336 | 0.003910782 |
| RAP1GAP2 | 0.197150314 | 5.827323758 | 2.899879539 | 0.003915215 |
| RP11-613M5.2 | 0.170376754 | 3.548609342 | 2.897120203 | 0.003949174 |
| SHISA2 | 0.549819397 | -1.710280872 | 2.897116295 | 0.003949222 |
| RP5-837J1.4 | 0.310018818 | 0.150992164 | 2.896863794 | 0.003952343 |
| NFU1 | -0.106912029 | 5.776765837 | -2.896652302 | 0.003954959 |
| UBQLN4P1 | -0.164965923 | 4.801085735 | -2.89659857 | 0.003955623 |
| CACNA2D2 | 0.460885955 | 0.700114622 | 2.896468921 | 0.003957228 |
| STK4-DT | -0.303536126 | -1.317962855 | -2.89595 | 0.003963656 |
| SNX3 | -0.099639357 | 9.421316803 | -2.895865066 | 0.003964709 |
| ZNF311 | -0.413000458 | 0.209793023 | -2.895496993 | 0.003969275 |
| ROR2 | 0.534935604 | -2.902988583 | 2.895374421 | 0.003970797 |
| DLD | 0.124635772 | 6.075463508 | 2.894893966 | 0.003976767 |
| TESPA1 | -0.378043759 | 5.708213671 | -2.89449483 | 0.003981733 |
| YRDC | 0.210479185 | 6.04458351 | 2.894409472 | 0.003982795 |
| NUP37 | -0.157932499 | 4.431939726 | -2.894105517 | 0.003986581 |
| GPR18 | 0.399288821 | 2.194880684 | 2.893648559 | 0.00399228 |
| TRBV7-6 | -0.378237772 | -0.126312347 | -2.893202188 | 0.003997853 |
| RP11-396C23.2 | 0.312924352 | -0.720576712 | 2.892871286 | 0.004001989 |
| UTRN | -0.120973458 | 6.047749572 | -2.892763318 | 0.00400334 |
| ZNF621 | -0.127111751 | 4.702662671 | -2.892761057 | 0.004003368 |
| RPL21P28 | 0.1318883 | 4.702194823 | 2.892482151 | 0.004006858 |
| SNORA71A | 0.278472759 | 2.140702618 | 2.892162662 | 0.00401086 |
| GPT2 | -0.415070453 | 3.534663423 | -2.892084051 | 0.004011845 |
| RRP1 | -0.173074013 | 4.358910015 | -2.891954292 | 0.004013472 |
| HERC1 | 0.131617129 | 6.102232953 | 2.891868963 | 0.004014542 |
| PFN1 | -0.119721254 | 10.68040511 | -2.891732298 | 0.004016256 |
| FAM132B | 0.684809653 | -0.106507604 | 2.891729828 | 0.004016287 |
| MPPE1 | -0.144539194 | 4.463186712 | -2.891553774 | 0.004018497 |
| NCALD | -0.409381074 | 1.21979976 | -2.891379787 | 0.004020681 |
| MARC2 | -0.711313189 | 1.529455309 | -2.890985288 | 0.004025639 |
| AC004074.3 | 0.175576507 | 4.181736118 | 2.89051946 | 0.004031499 |
| FAM159A | 0.409480789 | 1.451415838 | 2.890290051 | 0.004034388 |
| THEMIS2 | 0.293990739 | 7.013141283 | 2.890025583 | 0.004037721 |
| NHLRC3 | -0.198215594 | 4.637202717 | -2.889838766 | 0.004040077 |
| SLC25A28 | 0.142346649 | 7.192487404 | 2.889815566 | 0.00404037 |
| SLC12A7 | -0.273789846 | 5.663624235 | -2.889365431 | 0.004046052 |
| P2RY4 | -0.321172226 | -0.297469266 | -2.888418314 | 0.004058031 |
| SMARCE1P1 | 0.274748664 | -0.56774838 | 2.888370005 | 0.004058643 |
| AC006539.1 | 0.318322829 | -0.141864052 | 2.887779529 | 0.004066129 |
| SNX12 | 0.097413092 | 6.134426409 | 2.887680361 | 0.004067387 |
| RP11-797H7.3 | -0.407341794 | -1.101554896 | -2.887656 | 0.004067697 |
| FYN | -0.273356758 | 5.539607163 | -2.886703679 | 0.0040798 |
| HDX | -0.599092848 | -1.527868507 | -2.886471067 | 0.004082762 |
| CTA-221G9.10 | -0.4640579 | 2.113644713 | -2.886141563 | 0.00408696 |
| MYO5B | 0.414760803 | -3.593610711 | 2.885821375 | 0.004091043 |
| FBXW11 | -0.110223287 | 5.450612293 | -2.885817374 | 0.004091095 |
| TIMM8BP2 | -0.276667863 | 1.338186578 | -2.885794329 | 0.004091389 |
| MIR23A | 0.431629725 | 1.784054839 | 2.885712046 | 0.004092439 |
| ARHGAP15 | -0.126127894 | 5.676835794 | -2.885475527 | 0.004095458 |
| LCA5 | 0.490209397 | 0.491662817 | 2.885109492 | 0.004100136 |
| CDK6 | -0.337609041 | 7.955476808 | -2.885000676 | 0.004101527 |
| NTAN1P2 | -0.289839347 | 0.31265134 | -2.884714629 | 0.004105187 |
| SRCAP | -0.096439126 | 6.744887509 | -2.884569277 | 0.004107047 |
| AATF | -0.09207426 | 6.31078537 | -2.884549408 | 0.004107302 |
| C6orf52 | -0.292261311 | -0.353507746 | -2.884485956 | 0.004108114 |
| C8G | 0.217259086 | 1.130349987 | 2.884213284 | 0.004111608 |
| ST7-OT4 | 0.383981249 | -1.414277631 | 2.884127793 | 0.004112704 |
| ZNF624 | 0.159911682 | 2.727591716 | 2.883994538 | 0.004114412 |
| C1orf27 | 0.109928453 | 5.306106494 | 2.883968577 | 0.004114745 |
| SF3B4 | 0.077504887 | 7.332952644 | 2.883843804 | 0.004116346 |
| RPLP1 | 0.135998315 | 10.60303775 | 2.883720111 | 0.004117933 |
| ARHGAP23 | 0.309023825 | 3.727024599 | 2.882860168 | 0.004128985 |
| CDCA7 | 0.404266214 | 5.435619552 | 2.882512771 | 0.004133457 |
| TMEM246 | 0.62249075 | -2.74896656 | 2.882273385 | 0.004136541 |
| FAM95B1 | 0.409018463 | -3.932357806 | 2.882128038 | 0.004138414 |
| DDB1 | -0.133489462 | 6.455513807 | -2.881812221 | 0.004142488 |
| AF131215.9 | -0.475461564 | -0.348969822 | -2.881580879 | 0.004145475 |
| C1orf112 | -0.180151502 | 2.971629887 | -2.881255401 | 0.00414968 |
| RP11-82H13.2 | -0.146658813 | 3.483937137 | -2.88113595 | 0.004151224 |
| STOML1 | -0.154851263 | 1.756359808 | -2.880737367 | 0.00415638 |
| MAPK13 | 0.309712274 | 3.86917877 | 2.879712126 | 0.004169671 |
| DLEU7 | -0.533536197 | -0.132225934 | -2.879426821 | 0.004173376 |
| LINC01094 | -0.415648664 | 0.388140429 | -2.879290968 | 0.004175141 |
| CTD-2192J16.26 | 0.284239896 | 0.523718195 | 2.879186958 | 0.004176493 |
| SEC14L4 | 0.670286984 | -1.439604689 | 2.879182708 | 0.004176549 |
| ZNF22 | -0.195507261 | 6.841091963 | -2.878320989 | 0.004187766 |
| RP5-1159O4.1 | 0.265548208 | 2.711080316 | 2.878186886 | 0.004189514 |
| MAN1A2 | -0.092670902 | 5.683664945 | -2.877760248 | 0.00419508 |
| AP000357.4 | -0.261346532 | 3.196576359 | -2.877660821 | 0.004196378 |
| MORN1 | -0.220767559 | 1.246602541 | -2.877631237 | 0.004196764 |
| RP11-715J22.1 | 0.182337365 | 1.799068509 | 2.876926215 | 0.00420598 |
| TMEM18 | 0.118122676 | 4.714700106 | 2.87628911 | 0.004214324 |
| EMCN | 0.448370701 | -3.490955268 | 2.876011605 | 0.004217963 |
| RP11-255C15.4 | 0.270979391 | 0.862510948 | 2.875925395 | 0.004219094 |
| HIST1H3I | -0.433085224 | -0.229617167 | -2.875852878 | 0.004220046 |
| TRGV3 | -0.41331844 | 1.832672987 | -2.875409232 | 0.004225872 |
| CTD-2287O16.1 | -0.134771315 | 9.801639863 | -2.875227583 | 0.00422826 |
| EEF1DP2 | 0.131772841 | 3.616674175 | 2.874914404 | 0.00423238 |
| RP11-473O4.5 | -0.521157754 | -0.602028153 | -2.874802016 | 0.004233859 |
| NRP1 | 0.706525124 | -0.278936023 | 2.873684395 | 0.004248594 |
| ZNF37BP | -0.169231207 | 4.542161793 | -2.87363174 | 0.00424929 |
| PTK6 | -0.523226123 | 1.156440897 | -2.873606659 | 0.004249621 |
| IAH1 | 0.104496019 | 4.344264898 | 2.873166234 | 0.004255442 |
| C2orf88 | 0.551615389 | 1.305652676 | 2.873099368 | 0.004256326 |
| GPR32 | -0.571920376 | -1.860580711 | -2.873023501 | 0.00425733 |
| GUCY1B3 | -0.506194168 | 3.648016001 | -2.873023384 | 0.004257331 |
| ALMS1P | -0.321479478 | -0.367881247 | -2.872776968 | 0.004260593 |
| RP11-713P17.3 | -0.428128469 | -2.205199084 | -2.872502627 | 0.004264226 |
| KIF6 | -0.558119181 | -4.621175872 | -2.872267519 | 0.004267342 |
| TTPAL | 0.166986915 | 3.624290157 | 2.871829952 | 0.004273147 |
| FAM166A | 0.310397928 | -1.025957615 | 2.871727996 | 0.004274501 |
| NBPF20 | 0.134225314 | 2.83385838 | 2.871232461 | 0.004281086 |
| CCDC127 | -0.215693299 | 1.799384415 | -2.871061694 | 0.004283357 |
| KLF12 | 0.434118579 | 2.860828845 | 2.871024769 | 0.004283848 |
| HIPK2 | 0.228926415 | 5.558269679 | 2.870379268 | 0.004292444 |
| CTD-2643K12.1 | 0.153678883 | 4.609141164 | 2.870157992 | 0.004295395 |
| PROB1 | -0.309621409 | 1.543495684 | -2.869977139 | 0.004297808 |
| RP11-430C7.5 | -0.332053617 | 2.780483248 | -2.869881209 | 0.004299088 |
| GDF9 | -0.2693849 | 0.285717688 | -2.869771726 | 0.004300549 |
| PCTP | -0.185587288 | 4.509206184 | -2.869359716 | 0.004306054 |
| CCNB1 | 0.338154797 | 4.564424961 | 2.869005871 | 0.004310786 |
| BMS1P5 | -0.369264774 | 1.41145356 | -2.868132879 | 0.004322483 |
| RUSC1 | -0.132431248 | 4.103277006 | -2.867579131 | 0.004329916 |
| CERS1 | -0.483220362 | -1.802263724 | -2.867399742 | 0.004332327 |
| CTD-2284J15.1 | -0.296026359 | -1.220102278 | -2.866655801 | 0.004342338 |
| VANGL1 | -0.373001286 | 3.47841292 | -2.865941612 | 0.004351968 |
| ATP6V0E2 | 0.288457682 | 3.541235259 | 2.864920036 | 0.004365776 |
| CTD-2186M15.3 | 0.214884694 | 4.969686362 | 2.864380669 | 0.004373083 |
| KCNK1 | 0.501462459 | -2.738751411 | 2.863638014 | 0.004383162 |
| ZNF215 | 0.608848284 | -1.511209579 | 2.863473427 | 0.004385398 |
| CCDC43 | 0.088128055 | 5.437952474 | 2.863269251 | 0.004388174 |
| VSIG10 | -0.3260913 | 2.407962724 | -2.862688809 | 0.004396074 |
| FAM105A | 0.499886329 | 5.071821911 | 2.862242192 | 0.004402162 |
| RPS3AP3 | 0.295705303 | -0.131679822 | 2.862215372 | 0.004402528 |
| PRSS12 | -0.662334325 | -2.399222569 | -2.862022284 | 0.004405162 |
| RP11-416A14.1 | 0.419815194 | 2.882142552 | 2.861188481 | 0.004416555 |
| RP11-981G7.2 | -0.257831313 | 1.665736625 | -2.861120494 | 0.004417485 |
| HMGA2 | 1.024217892 | -3.028685017 | 2.8609924 | 0.004419238 |
| AC008592.8 | -0.377495068 | -1.038912226 | -2.860914161 | 0.004420309 |
| MLLT4 | 0.501482449 | 1.669847093 | 2.860812117 | 0.004421706 |
| RP11-114M5.1 | 0.210416668 | 0.645626148 | 2.860722141 | 0.004422938 |
| TYMP | -0.464417889 | 6.376993743 | -2.860419446 | 0.004427086 |
| CTC-345K18.2 | -0.461381265 | 0.884229595 | -2.860312674 | 0.00442855 |
| SAV1 | 0.154024435 | 4.448797222 | 2.86027041 | 0.00442913 |
| RBMS1 | -0.143006471 | 5.302361598 | -2.860011052 | 0.004432688 |
| WDR7 | -0.157021707 | 3.77116719 | -2.859998066 | 0.004432866 |
| SMARCE1P5 | 0.114488835 | 4.9633817 | 2.859417007 | 0.004440848 |
| NHEJ1 | -0.256630525 | -1.62772899 | -2.858883711 | 0.004448185 |
| SLC6A9 | 0.532848845 | 0.337007031 | 2.858008066 | 0.004460256 |
| C17orf103 | -0.235714213 | 4.968115329 | -2.857894792 | 0.00446182 |
| AL590714.1 | 0.436310362 | 1.019725942 | 2.857666229 | 0.004464977 |
| RP11-6I2.3 | -0.371110661 | -0.563585226 | -2.857289371 | 0.004470186 |
| PROS1 | 0.540338064 | 0.601256006 | 2.857042528 | 0.004473601 |
| NAIF1 | 0.122785307 | 3.910284007 | 2.856951806 | 0.004474857 |
| AC007620.3 | -0.273356788 | -0.576257482 | -2.85597952 | 0.004488335 |
| RP11-356I18.1 | -0.265377247 | 0.158951127 | -2.855851697 | 0.00449011 |
| RP11-206L10.9 | -0.269163861 | 0.587986338 | -2.85580303 | 0.004490786 |
| HARBI1 | 0.175935405 | 2.847165351 | 2.855772105 | 0.004491215 |
| TAGAP | 0.276828862 | 6.782628518 | 2.855107532 | 0.004500455 |
| QDPR | -0.145960009 | 5.033384257 | -2.855043913 | 0.00450134 |
| FAM58A | -0.107381121 | 4.434576666 | -2.854774867 | 0.004505086 |
| ABCA6 | -0.536720746 | -1.781536122 | -2.854066514 | 0.004514962 |
| RP11-364L4.1 | 0.201441211 | 3.171274432 | 2.854010398 | 0.004515745 |
| ADSSL1 | 0.32131337 | 0.851879252 | 2.853863659 | 0.004517794 |
| SPTBN1 | 0.245200431 | 6.619146386 | 2.853689999 | 0.00452022 |
| RP11-342M1.3 | -0.253910281 | -0.082832794 | -2.853670982 | 0.004520485 |
| UNG | -0.280058535 | 5.825502617 | -2.853630184 | 0.004521055 |
| TAF1 | 0.076682858 | 5.231454295 | 2.852996272 | 0.004529921 |
| MCHR1 | -0.378382145 | -2.744575058 | -2.852868273 | 0.004531713 |
| COPG1 | -0.10065988 | 6.651152002 | -2.852668134 | 0.004534517 |
| RN7SKP180 | -0.338109778 | -0.426550556 | -2.85248459 | 0.004537089 |
| MIR3613 | -0.280930974 | 1.33692183 | -2.8522574 | 0.004540275 |
| ZNF497 | -0.293927897 | 0.837836458 | -2.852093129 | 0.00454258 |
| ARL4C | 0.408715504 | 5.711891929 | 2.851990141 | 0.004544025 |
| MTO1 | -0.137128007 | 3.782809563 | -2.851262955 | 0.004554244 |
| RP11-343N15.5 | 0.257557392 | 5.807340892 | 2.850501982 | 0.00456496 |
| NKAIN2 | 0.71564435 | -2.246879437 | 2.850439846 | 0.004565836 |
| C6orf1 | 0.148320795 | 4.857847315 | 2.850342972 | 0.004567202 |
| ALG10B | 0.179253625 | 2.701808698 | 2.849963796 | 0.004572552 |
| LRRC59 | 0.130829353 | 7.05202011 | 2.849475898 | 0.004579445 |
| SLC12A5 | 0.499757806 | -1.397744032 | 2.849317629 | 0.004581683 |
| FAM102A | 0.246579786 | 3.97539718 | 2.848996383 | 0.004586229 |
| PNMT | 0.559944665 | 0.546986124 | 2.848923696 | 0.004587258 |
| CYB5R1 | -0.150303196 | 4.690300499 | -2.84890587 | 0.00458751 |
| LINC00537 | -0.376818447 | 0.625435534 | -2.847326623 | 0.004609922 |
| FAT1 | 0.929960071 | -3.277694837 | 2.847299427 | 0.004610309 |
| RP5-888M10.2 | -0.417410623 | -0.202145977 | -2.84673348 | 0.004618365 |
| CEACAM19 | -0.486977285 | 1.095670948 | -2.846254553 | 0.004625192 |
| MEFV | -0.652974062 | 3.688409724 | -2.84609349 | 0.004627491 |
| FXYD6 | 0.505581727 | 2.265178201 | 2.845776247 | 0.00463202 |
| CTD-2308N23.2 | 0.366941504 | -1.535009168 | 2.845741031 | 0.004632523 |
| FAM212B-AS1 | 0.359914942 | -0.925952972 | 2.845671568 | 0.004633516 |
| AC022182.2 | 0.173928657 | 3.527099649 | 2.845579729 | 0.004634828 |
| SLA2 | 0.378798699 | 5.113736528 | 2.845356457 | 0.00463802 |
| THAP6 | -0.124479248 | 2.669527029 | -2.84518315 | 0.0046405 |
| RP1-140C12.2 | -0.338894859 | -2.272212878 | -2.8450156 | 0.004642898 |
| RNU6-60P | 0.328180246 | 0.263460781 | 2.844883098 | 0.004644795 |
| SOWAHD | -0.461180418 | 3.382381731 | -2.843799162 | 0.004660341 |
| RP11-390F4.8 | 0.286826717 | 3.563639313 | 2.843283349 | 0.004667755 |
| RP11-694I15.7 | 0.257010476 | 2.750702693 | 2.842404777 | 0.004680409 |
| TRIM16L | -0.23685215 | 1.569272008 | -2.842290201 | 0.004682061 |
| DPY19L1P1 | 0.390539822 | 2.8214003 | 2.841699131 | 0.004690595 |
| SNX10 | -0.299895449 | 6.321787832 | -2.841573498 | 0.00469241 |
| RP11-809O17.1 | -0.223450279 | 4.829901941 | -2.841327011 | 0.004695974 |
| C11orf24 | -0.185910324 | 4.336177306 | -2.840565847 | 0.004706995 |
| SNRPGP15 | 0.199690038 | 2.512972745 | 2.840354496 | 0.004710059 |
| MED29 | 0.0720281 | 5.971967811 | 2.839873718 | 0.004717037 |
| CD55 | 0.22182224 | 6.936405546 | 2.839713113 | 0.00471937 |
| RP11-317N8.5 | -0.325879683 | -1.6829348 | -2.839537195 | 0.004721927 |
| CDHR4 | 0.440755491 | -0.518028665 | 2.839370095 | 0.004724356 |
| FAM8A1 | 0.193569637 | 6.116473869 | 2.839031315 | 0.004729285 |
| STX3 | 0.260986722 | 5.240401198 | 2.838911788 | 0.004731026 |
| ASPRV1 | 0.272360908 | 1.379862124 | 2.838473804 | 0.004737407 |
| C14orf28 | 0.16242449 | 2.469609289 | 2.837802377 | 0.004747206 |
| WIPF2 | 0.117372386 | 5.109796304 | 2.837347814 | 0.00475385 |
| AP000344.4 | -0.502840782 | 0.587641365 | -2.837269488 | 0.004754995 |
| LA16c-306A4.2 | -0.319275671 | -1.786792749 | -2.836820877 | 0.004761562 |
| PLEKHB1 | -0.318782601 | 1.181260829 | -2.836808297 | 0.004761746 |
| ASB2 | 0.38208345 | 0.306079218 | 2.836611604 | 0.004764628 |
| CTD-2369P2.5 | 0.358803348 | -0.727595442 | 2.836604607 | 0.004764731 |
| CTB-52I2.4 | 0.095519061 | 5.899307584 | 2.836431495 | 0.004767269 |
| ARHGAP25 | -0.186532137 | 6.545793077 | -2.836299087 | 0.00476921 |
| CABIN1 | -0.123206644 | 6.137753779 | -2.836054405 | 0.004772801 |
| MFSD9 | 0.149961087 | 3.939685614 | 2.836029431 | 0.004773167 |
| TRIM32 | -0.38762145 | 2.0108477 | -2.835842767 | 0.004775908 |
| RP11-745A24.2 | 0.23055469 | 0.850308197 | 2.835734 | 0.004777506 |
| RP11-36C20.1 | -0.144321866 | 9.597775953 | -2.835554963 | 0.004780137 |
| HGFAC | 0.380095593 | -2.099300999 | 2.835526454 | 0.004780556 |
| SACM1L | -0.107634874 | 5.3901272 | -2.835345338 | 0.004783219 |
| RRAS | -0.359093497 | 4.932681532 | -2.834969587 | 0.004788748 |
| STK32C | -0.220833174 | 3.775480932 | -2.834937151 | 0.004789226 |
| LCT | -0.646901665 | -1.327873555 | -2.834483892 | 0.004795904 |
| APOOP5 | -0.283269502 | -0.198827659 | -2.834469718 | 0.004796113 |
| CORO2B | -0.451285941 | -1.105639527 | -2.834279014 | 0.004798926 |
| HCCS | 0.114758286 | 4.949062913 | 2.834201573 | 0.004800068 |
| TPRA1 | -0.134531233 | 4.784034349 | -2.834174065 | 0.004800474 |
| PLEKHB2 | 0.146359859 | 6.481419836 | 2.833596051 | 0.00480901 |
| PPP2R1A | -0.090250896 | 6.602789348 | -2.833426859 | 0.004811511 |
| CSRP2 | 0.681230856 | -1.966454597 | 2.833369239 | 0.004812363 |
| FASTKD3 | -0.119254921 | 3.505031571 | -2.832792514 | 0.004820899 |
| LIPT1 | 0.15379979 | 3.565764471 | 2.832320555 | 0.004827895 |
| PRKRIRP3 | 0.320555717 | -1.021628579 | 2.832066353 | 0.004831667 |
| ABR | -0.205337426 | 5.689626604 | -2.832055458 | 0.004831828 |
| C1orf54 | 0.324362503 | 3.243313821 | 2.831964023 | 0.004833186 |
| CTD-2538G9.5 | 0.322654381 | 0.95558282 | 2.831913655 | 0.004833933 |
| RP11-403A21.2 | -0.339427221 | -0.938573012 | -2.831818912 | 0.004835341 |
| LYPD5 | -0.418110121 | -2.00278439 | -2.831504858 | 0.004840007 |
| HR | 0.455949757 | -2.7950027 | 2.831288402 | 0.004843226 |
| RP11-296A18.5 | 0.208022054 | 3.725485659 | 2.830949528 | 0.004848269 |
| UPF3AP3 | -0.276022976 | 0.903913243 | -2.830654931 | 0.004852657 |
| ZNF200 | -0.235709212 | 1.87605245 | -2.830392012 | 0.004856577 |
| XK | 0.617227233 | 2.216628493 | 2.830158469 | 0.00486006 |
| SLC8A1 | -0.608935326 | 1.870822728 | -2.829927663 | 0.004863506 |
| MYO1A | -0.341933199 | -1.145636053 | -2.828992155 | 0.004877492 |
| ZNF345 | -0.168763058 | 2.627285089 | -2.828429133 | 0.004885928 |
| CTC-471F3.4 | -0.166207813 | 7.218040944 | -2.828285104 | 0.004888088 |
| CTB-179K24.3 | -0.306507884 | -0.489708632 | -2.828259282 | 0.004888475 |
| PANK2 | -0.107972258 | 4.749516628 | -2.827647081 | 0.004897667 |
| MTA2 | 0.094171567 | 7.564268234 | 2.827165281 | 0.004904911 |
| ZNF548 | 0.191381417 | 4.827985831 | 2.826991722 | 0.004907523 |
| SPIN1 | -0.157634123 | 6.680218661 | -2.826875785 | 0.004909269 |
| ZNF276 | 0.128104629 | 5.673404105 | 2.826362246 | 0.004917008 |
| HNRNPA3P1 | 0.239895175 | 0.34212909 | 2.82631239 | 0.00491776 |
| FAM46B | 0.371700979 | -1.301434625 | 2.826124242 | 0.004920598 |
| TRH | 0.952753347 | 3.073099346 | 2.826101526 | 0.004920941 |
| CTD-2020K17.3 | 0.319441385 | 0.650547785 | 2.826095286 | 0.004921035 |
| ETV6 | -0.194795647 | 7.348076108 | -2.826052721 | 0.004921678 |
| HNRNPA3P12 | 0.218961234 | 1.200617235 | 2.824982315 | 0.004937858 |
| OSBPL2 | 0.130947522 | 5.690398382 | 2.824776725 | 0.004940971 |
| MIPOL1 | 0.547009775 | -4.795070995 | 2.824748856 | 0.004941394 |
| S100A14 | 0.430639347 | -1.961803299 | 2.824607271 | 0.004943539 |
| RP11-57H14.2 | -0.283868899 | 2.285890181 | -2.82453092 | 0.004944696 |
| TPD52L1 | 0.462798639 | -3.718018055 | 2.824295643 | 0.004948263 |
| UFM1 | 0.118038516 | 7.126978859 | 2.82427115 | 0.004948635 |
| CDK9 | 0.139264581 | 7.411173435 | 2.824113572 | 0.004951026 |
| RP11-388P9.2 | -0.81600365 | -0.32641846 | -2.823811459 | 0.004955612 |
| RP11-744N12.3 | 0.37668832 | -2.187741962 | 2.823780317 | 0.004956085 |
| RP11-439C15.4 | 0.44432563 | 0.35455951 | 2.82326371 | 0.004963938 |
| PCDH1 | 0.478320628 | -1.698375229 | 2.82292014 | 0.004969166 |
| RP11-739L10.1 | -0.296199831 | -0.226957304 | -2.822896827 | 0.004969521 |
| PDIA3 | 0.125940781 | 8.32098049 | 2.822808213 | 0.004970871 |
| ADCY9 | -0.447603716 | 3.139847817 | -2.822476415 | 0.004975926 |
| RP11-564A8.4 | 0.235678045 | 1.206393907 | 2.821889602 | 0.004984879 |
| NUMBL | -0.1721475 | 3.273267559 | -2.820884411 | 0.005000249 |
| PMAIP1 | 0.342398806 | 7.529373659 | 2.820545487 | 0.005005441 |
| NCBP1 | -0.107149621 | 5.334112768 | -2.820389091 | 0.005007839 |
| AC074367.1 | -0.319802638 | -0.932891677 | -2.819825951 | 0.00501648 |
| PPP3R1 | 0.105384972 | 7.298549568 | 2.81948262 | 0.005021756 |
| MKLN1-AS | 0.201611125 | 1.259041937 | 2.819290161 | 0.005024715 |
| FER1L6-AS1 | -0.411263024 | -2.753465493 | -2.819165369 | 0.005026634 |
| ATP10A | 0.524684002 | 3.008326825 | 2.819036435 | 0.005028618 |
| TMPO | -0.182254072 | 6.783906865 | -2.818876926 | 0.005031074 |
| MYO1G | -0.192235932 | 7.322414451 | -2.818794727 | 0.00503234 |
| UNC80 | -0.527165364 | -3.019131208 | -2.818679285 | 0.005034118 |
| LILRB1 | -0.511104032 | 3.843033978 | -2.817875442 | 0.005046516 |
| TMEM185AP1 | -0.316439075 | -0.037674098 | -2.817557413 | 0.005051429 |
| HOXA-AS2 | 0.410823995 | -1.719190055 | 2.817316248 | 0.005055157 |
| ARHGAP39 | 0.49724122 | -2.355351135 | 2.816587773 | 0.005066434 |
| SLC14A1 | -0.499274393 | 0.959534026 | -2.816497377 | 0.005067835 |
| CAMSAP2 | -0.440140623 | 0.36947786 | -2.816135317 | 0.005073449 |
| ZNF710 | -0.153317081 | 5.024360659 | -2.816000264 | 0.005075545 |
| OAZ3 | -0.331761931 | -1.567407221 | -2.815721294 | 0.005079877 |
| C4orf47 | 0.31466534 | -0.49902251 | 2.815668669 | 0.005080694 |
| SCOC | -0.154258044 | 3.520390337 | -2.815458576 | 0.005083959 |
| WDR89 | -0.168194395 | 3.838992367 | -2.815344477 | 0.005085733 |
| MINOS1P2 | -0.272686336 | 2.834463202 | -2.815276703 | 0.005086787 |
| MIR4263 | -0.339381026 | 0.180299704 | -2.81517207 | 0.005088414 |
| LMNA | 0.432783318 | 6.54728605 | 2.815049679 | 0.005090319 |
| GPD2 | -0.174665981 | 5.136335208 | -2.814957299 | 0.005091756 |
| SGCB | -0.475156981 | 0.886819019 | -2.814744764 | 0.005095066 |
| IGHJ3 | 0.55850503 | 3.328056556 | 2.814527173 | 0.005098456 |
| ZNF663P | -0.462942013 | -2.532864927 | -2.814353228 | 0.005101167 |
| RN7SKP78 | 0.320992862 | 0.80242339 | 2.814117484 | 0.005104844 |
| NAGPA | -0.15785904 | 4.252782418 | -2.813793661 | 0.005109899 |
| FGD4 | 0.435665123 | 3.336485807 | 2.813263254 | 0.005118188 |
| RP11-552C15.1 | -0.340298532 | -0.530541353 | -2.812649907 | 0.005127788 |
| PCNXL2 | 0.3393706 | 2.41245752 | 2.812564427 | 0.005129128 |
| DDX51 | 0.133615278 | 4.58662554 | 2.812203742 | 0.005134782 |
| SYT1 | 0.654389152 | -2.855022721 | 2.812029246 | 0.00513752 |
| ENTPD7 | 0.282938332 | 4.222055692 | 2.811895295 | 0.005139622 |
| TRMT2B | -0.085867877 | 5.032396542 | -2.811802898 | 0.005141073 |
| RUVBL2 | -0.172887627 | 6.003248602 | -2.811473932 | 0.005146241 |
| RP1-29C18.8 | -0.328733245 | 1.162284972 | -2.811264618 | 0.005149532 |
| KDM4D | -0.314744707 | -0.360801878 | -2.810658013 | 0.005159079 |
| RP11-46D6.1 | 0.292824013 | 1.012426061 | 2.810313644 | 0.005164507 |
| CEP55 | 0.402597138 | 3.409625968 | 2.80951538 | 0.005177107 |
| RP11-302I18.1 | 0.266370579 | 1.396550693 | 2.809373131 | 0.005179355 |
| PRR3 | -0.147465057 | 4.123749004 | -2.808936782 | 0.005186258 |
| RP5-857K21.7 | -0.26359233 | 7.129592637 | -2.808507003 | 0.005193064 |
| AVL9 | -0.111443545 | 4.769995198 | -2.808483584 | 0.005193435 |
| NACC2 | -0.361236602 | 3.834665124 | -2.808130735 | 0.00519903 |
| TRIM36 | -0.527624592 | -0.676146851 | -2.80793751 | 0.005202096 |
| TEX2 | -0.197829287 | 5.426284027 | -2.807829901 | 0.005203804 |
| MRPS21P1 | -0.305998229 | -0.02045138 | -2.807757428 | 0.005204955 |
| AC078883.3 | 0.293074518 | 1.777087912 | 2.807559714 | 0.005208095 |
| NACAP1 | 0.190431159 | 3.698170839 | 2.806434163 | 0.005226006 |
| CTD-2336O2.1 | 0.17120698 | 4.922070224 | 2.806170455 | 0.00523021 |
| MTND6P21 | -0.333100583 | -0.494185848 | -2.805812745 | 0.005235918 |
| RP11-46H11.12 | -0.282302703 | 0.652391573 | -2.805550158 | 0.005240112 |
| RPS12P26 | -0.220533952 | 1.201936233 | -2.805377415 | 0.005242873 |
| PIGS | -0.097627198 | 6.102205502 | -2.805335612 | 0.005243541 |
| CDKN2B | -0.607672528 | -0.225056409 | -2.804984904 | 0.00524915 |
| RNASEH2A | -0.229288618 | 5.185729547 | -2.80390791 | 0.005266409 |
| NDE1 | 0.106945556 | 5.148406526 | 2.803724111 | 0.00526936 |
| RP3-347M6.2 | 0.273788141 | -0.603763784 | 2.803723477 | 0.00526937 |
| MAP3K6 | -0.298449431 | 3.532996447 | -2.80358213 | 0.00527164 |
| SLC35E4 | 0.380104331 | 0.776547286 | 2.803296634 | 0.005276228 |
| TRMT112 | -0.103708945 | 6.838719194 | -2.803156414 | 0.005278483 |
| CENPW | 0.229468068 | 4.898827663 | 2.803118634 | 0.005279091 |
| RP11-501J20.5 | 0.527004497 | -0.051956373 | 2.802776613 | 0.005284594 |
| RP11-967K21.1 | -0.273929542 | 2.039053714 | -2.80253023 | 0.005288562 |
| RPS6KB1 | 0.081629496 | 4.854438797 | 2.802469114 | 0.005289547 |
| RP11-638F5.1 | -0.55137611 | -2.042331575 | -2.801926077 | 0.005298304 |
| TVP23B | 0.131008884 | 3.539388825 | 2.801768766 | 0.005300843 |
| PAG1 | 0.289089179 | 5.2049493 | 2.801764389 | 0.005300913 |
| PRR24 | 0.121410581 | 5.376100647 | 2.801639989 | 0.005302922 |
| FXYD2 | 0.375376404 | -2.126184472 | 2.80143362 | 0.005306256 |
| INPP5K | 0.12675835 | 5.111938893 | 2.801403504 | 0.005306743 |
| RP11-6J24.6 | -0.175918579 | 2.015415279 | -2.801372373 | 0.005307246 |
| LY6G5C | -0.169189615 | 2.542525725 | -2.800999791 | 0.005313271 |
| MYL6B | 0.199852329 | 4.684332486 | 2.800684768 | 0.005318369 |
| SDK2 | 0.650348891 | 0.938914989 | 2.800204997 | 0.005326143 |
| SCARNA2 | -0.24844276 | 1.584361993 | -2.800014509 | 0.005329233 |
| DCN | 0.693839288 | -3.359819079 | 2.80000891 | 0.005329324 |
| AC068580.6 | 0.392858299 | -1.363542004 | 2.799854558 | 0.005331828 |
| RP11-525A16.4 | -0.365166894 | -0.617646377 | -2.799476561 | 0.005337966 |
| RP11-114F3.2 | -0.192880846 | 4.829404312 | -2.799453393 | 0.005338343 |
| CA1 | 0.911595215 | 3.480681985 | 2.799197035 | 0.005342509 |
| RPL14P1 | -0.099168422 | 10.99202298 | -2.799077376 | 0.005344455 |
| MGRN1 | 0.10380546 | 6.234514442 | 2.798929154 | 0.005346867 |
| AC021016.7 | -0.16453247 | 5.860190212 | -2.798908766 | 0.005347198 |
| MAP7 | -0.440534633 | 5.0076261 | -2.798782909 | 0.005349247 |
| DCAF8 | -0.114091773 | 5.735916774 | -2.798699553 | 0.005350604 |
| RYR3 | 0.713048583 | -2.046355 | 2.79868784 | 0.005350794 |
| THA1P | -0.335494759 | -1.114589318 | -2.798617463 | 0.00535194 |
| PROM2 | -0.366000548 | -1.47410226 | -2.798360382 | 0.005356129 |
| WNT7A | 0.464027845 | -1.984232134 | 2.798249539 | 0.005357935 |
| RP11-452L6.5 | -0.250658871 | 4.548377332 | -2.797794958 | 0.00536535 |
| PSMB9 | -0.209831869 | 5.74062393 | -2.797735741 | 0.005366317 |
| RP11-350G8.3 | 0.245577751 | 0.518153061 | 2.797459358 | 0.005370831 |
| TMEM256-PLSCR3 | -0.27612181 | -0.210401431 | -2.797445423 | 0.005371058 |
| CTD-2325A15.5 | -0.338586959 | -1.048470735 | -2.797319486 | 0.005373116 |
| RP1-232L22__B.1 | -0.224527523 | 4.861971625 | -2.79730225 | 0.005373398 |
| LL22NC03-86G7.1 | -0.286917482 | 1.558457086 | -2.797170866 | 0.005375546 |
| NSUN7 | 0.565468079 | -0.257492616 | 2.797126436 | 0.005376272 |
| HIST1H4F | -0.402841063 | -0.632573901 | -2.797087489 | 0.005376909 |
| CKAP4 | 0.533617888 | 5.979806439 | 2.796990048 | 0.005378503 |
| EEF1DP1 | 0.112497443 | 6.451433737 | 2.79667867 | 0.005383599 |
| COL12A1 | 0.589716849 | -4.008946525 | 2.796402218 | 0.005388127 |
| CTD-2319I12.1 | -0.404371736 | -1.203967215 | -2.795488156 | 0.005403123 |
| ZDHHC24 | -0.13942068 | 5.135662358 | -2.795076208 | 0.005409893 |
| HCCAT3 | -0.316387419 | -0.586154152 | -2.794889029 | 0.005412972 |
| CYP2D6 | 0.296691621 | 0.20448572 | 2.793496186 | 0.005435933 |
| SLC23A1 | -0.527291921 | 0.278469455 | -2.79339254 | 0.005437645 |
| HNRNPA1P39 | 0.203789387 | 4.150276609 | 2.79310124 | 0.00544246 |
| SGK1 | 0.499820086 | 4.489925292 | 2.792933299 | 0.005445238 |
| RAB42 | 0.372591658 | 0.533684272 | 2.792635144 | 0.005450172 |
| NECAB2 | 0.48435291 | -1.799223605 | 2.792552017 | 0.005451548 |
| ARHGAP35 | -0.116247007 | 5.188550579 | -2.792449808 | 0.005453241 |
| RP13-585F24.1 | 0.210186534 | 2.423471843 | 2.792374251 | 0.005454493 |
| ZFAT | -0.140235554 | 3.08437758 | -2.792052298 | 0.005459829 |
| CCDC73 | -0.35691059 | -0.25504041 | -2.791995641 | 0.005460769 |
| CCDC58P1 | -0.280524434 | 0.85730388 | -2.791624403 | 0.005466929 |
| RP11-715F3.2 | -0.265897062 | 0.296030594 | -2.79148501 | 0.005469243 |
| SLC35E1P1 | 0.303360078 | 1.377014959 | 2.791216963 | 0.005473697 |
| EP400NL | -0.131249311 | 3.313198521 | -2.790819057 | 0.005480314 |
| BDH1 | -0.236174723 | 3.442842516 | -2.790746601 | 0.00548152 |
| RP11-705O1.8 | -0.331260801 | -0.080774002 | -2.790155025 | 0.005491373 |
| TRIM37 | 0.099004399 | 4.731395948 | 2.789989353 | 0.005494135 |
| CHRNB4 | -0.434369 | -3.63990744 | -2.789338828 | 0.005504993 |
| FRS3 | -0.134348361 | 3.706582505 | -2.789085892 | 0.00550922 |
| USP34 | -0.067694035 | 6.66306147 | -2.78836423 | 0.005521297 |
| RP11-66N11.8 | 0.209638031 | 1.343731811 | 2.787831361 | 0.00553023 |
| EDF1 | -0.099191276 | 8.760533216 | -2.787644183 | 0.005533371 |
| RP11-467L19.8 | -0.418737469 | -0.753545613 | -2.787249037 | 0.005540007 |
| IDH2 | 0.159473144 | 8.137467561 | 2.787007149 | 0.005544073 |
| OSR2 | 0.500969487 | -1.694951996 | 2.786582847 | 0.005551211 |
| IMPDH1P4 | -0.249574768 | 1.435879502 | -2.786458248 | 0.005553309 |
| HPDL | -0.381081441 | 2.351271115 | -2.786377376 | 0.005554671 |
| INTS10 | -0.11128674 | 5.840805189 | -2.786307366 | 0.00555585 |
| AC008746.3 | -0.303961906 | 0.038087127 | -2.785735889 | 0.005565485 |
| ELP2 | -0.130042145 | 4.900679212 | -2.785674602 | 0.005566519 |
| PRKDC | -0.190665211 | 6.656688467 | -2.785285063 | 0.005573096 |
| CTD-2350C19.2 | 0.299846475 | 0.062204049 | 2.785158511 | 0.005575235 |
| RP3-486I3.7 | -0.332891032 | -0.811653791 | -2.784731596 | 0.005582454 |
| TOP3A | -0.110569062 | 4.950264976 | -2.784710648 | 0.005582808 |
| RP9P | 0.263012771 | 3.34101514 | 2.784506385 | 0.005586265 |
| IPO8P1 | -0.156600856 | 2.387258202 | -2.784455448 | 0.005587128 |
| P2RY14 | -0.556492232 | 2.085762906 | -2.784346422 | 0.005588974 |
| RP11-69M1.3 | -0.310169079 | -0.484568715 | -2.784230819 | 0.005590932 |
| CLIP2 | -0.334932103 | 5.821357483 | -2.784022771 | 0.005594458 |
| RP11-982M15.2 | 0.293104715 | -0.062784419 | 2.783984805 | 0.005595102 |
| SNORA7B | 0.268947349 | 0.744130619 | 2.783787428 | 0.005598449 |
| ZCCHC9 | -0.110879151 | 3.923326905 | -2.782964291 | 0.005612428 |
| LYRM4 | 0.159448393 | 3.364861315 | 2.782946613 | 0.005612728 |
| GYG1 | 0.218245614 | 6.546975859 | 2.782747259 | 0.005616119 |
| RPL18A | 0.161138554 | 7.132086829 | 2.782560514 | 0.005619296 |
| SKP1 | 0.143799266 | 5.162258616 | 2.782451783 | 0.005621147 |
| TGFA | -0.442511316 | 1.920811578 | -2.781728619 | 0.005633472 |
| NXNL2 | -0.400731529 | -1.764378219 | -2.781728308 | 0.005633477 |
| AP002954.4 | -0.499716762 | -0.09420298 | -2.781281209 | 0.005641109 |
| FAIM3 | -0.373289531 | 5.038706552 | -2.781176869 | 0.005642891 |
| DCT | -0.351160174 | -3.68504254 | -2.781136935 | 0.005643574 |
| TUBE1 | -0.120540296 | 3.021587829 | -2.781060148 | 0.005644886 |
| HLA-L | 0.343171124 | 3.547074784 | 2.780646745 | 0.005651955 |
| ZNF626 | -0.519025931 | -0.208834285 | -2.780524468 | 0.005654047 |
| S100A3 | 0.354712981 | -1.312786786 | 2.780471769 | 0.00565495 |
| PLA2G4A | -0.315938826 | 4.235742214 | -2.780313156 | 0.005657665 |
| MYO6 | 0.400590377 | -0.88660122 | 2.779932617 | 0.005664186 |
| RP11-95O2.5 | -0.334441836 | -0.352907603 | -2.779896039 | 0.005664813 |
| GPS1 | -0.09418464 | 5.935370216 | -2.779873233 | 0.005665204 |
| RSBN1L-AS1 | 0.161340992 | 2.972876599 | 2.779444224 | 0.005672564 |
| RP11-497G19.2 | -0.882550783 | 2.314909844 | -2.778795857 | 0.005683704 |
| HNMT | -0.715096883 | 1.821685479 | -2.778603248 | 0.005687017 |
| RP5-942I16.1 | -0.365895224 | -0.372062819 | -2.778531433 | 0.005688253 |
| GS1-309P15.4 | 0.267024263 | 0.300579063 | 2.777623544 | 0.005703896 |
| ECEL1 | 0.436216352 | -2.762239715 | 2.777094712 | 0.005713026 |
| TRDC | -0.443578166 | 2.610845411 | -2.776580879 | 0.00572191 |
| CNOT11 | -0.069484021 | 6.604173792 | -2.776458578 | 0.005724027 |
| POLR2L | -0.140008419 | 7.022374958 | -2.776199219 | 0.005728517 |
| MST1R | -0.466663585 | -1.252708568 | -2.775906196 | 0.005733594 |
| KIAA0226L | 0.444379949 | 1.023476732 | 2.775791021 | 0.005735591 |
| RP11-407G23.4 | 0.265820357 | 1.030325664 | 2.775329165 | 0.005743604 |
| DHX37 | 0.10428052 | 4.942120291 | 2.77529338 | 0.005744225 |
| IGSF10 | -0.894459918 | 2.524045189 | -2.775221653 | 0.005745471 |
| MRPL45 | -0.145735877 | 6.686166469 | -2.775202996 | 0.005745795 |
| PPIAP29 | -0.154608536 | 6.969575474 | -2.774944725 | 0.005750281 |
| ANXA2P3 | -0.43051274 | 2.298622749 | -2.774541761 | 0.005757288 |
| LINC01133 | 0.562477513 | -0.105213908 | 2.7744379 | 0.005759096 |
| RP11-517H2.6 | -0.312482558 | -1.913950688 | -2.774311536 | 0.005761295 |
| RP3-341D10.1 | 0.296803155 | -0.412352431 | 2.773911083 | 0.005768271 |
| LSMD1 | 0.14107097 | 6.159873212 | 2.773839654 | 0.005769516 |
| SPRYD4 | -0.148750467 | 4.541109288 | -2.77373081 | 0.005771413 |
| TPI1P1 | -0.16442887 | 8.734215999 | -2.773515616 | 0.005775167 |
| CDC14A | 0.13688575 | 4.852534492 | 2.773294628 | 0.005779023 |
| CTD-2331H12.7 | 0.297443668 | 0.296466607 | 2.772922417 | 0.005785524 |
| RP11-30K9.6 | -0.297555478 | -0.096670899 | -2.772552226 | 0.005791997 |
| RBMS1P1 | -0.152439789 | 6.036703471 | -2.772448082 | 0.005793819 |
| RP11-385D13.3 | 0.32837706 | 1.243266719 | 2.772293459 | 0.005796525 |
| MMP2 | -0.822065077 | 3.357850521 | -2.772062301 | 0.005800573 |
| SYCP3 | -0.292025893 | -0.749107945 | -2.771290767 | 0.005814102 |
| TP73-AS1 | -0.187373324 | 4.237757001 | -2.771078615 | 0.005817827 |
| PRR14 | 0.119542404 | 6.541382533 | 2.770549766 | 0.005827122 |
| NDUFB1 | -0.122388389 | 5.427655543 | -2.76998317 | 0.005837096 |
| TKTL2 | 0.383242443 | -2.453033806 | 2.769149041 | 0.005851807 |
| ANKRD34B | -0.659661766 | -1.285068865 | -2.768165657 | 0.005869193 |
| PEPD | -0.164434697 | 5.039893532 | -2.767677742 | 0.005877836 |
| LCP1 | -0.170760729 | 10.6166458 | -2.766952266 | 0.00589071 |
| RP11-24F11.2 | -0.454878555 | 1.551496215 | -2.766789196 | 0.005893607 |
| TRAV12-1 | -0.406930602 | 0.085652055 | -2.76669618 | 0.00589526 |
| F2RL3 | 0.525836033 | -0.892157372 | 2.766513278 | 0.005898512 |
| ZNF428 | 0.149755196 | 6.118940345 | 2.765095435 | 0.005923776 |
| SIGLEC7 | -0.53165728 | 3.18785126 | -2.764715398 | 0.005930564 |
| RP11-215P8.1 | 0.295767366 | -0.300161411 | 2.764670062 | 0.005931374 |
| MSH6 | 0.177835481 | 5.287186231 | 2.76406023 | 0.005942283 |
| PLEKHM1P | 0.145736373 | 5.517839632 | 2.762390734 | 0.00597224 |
| VWCE | 0.407779824 | 1.436427752 | 2.76168967 | 0.00598486 |
| TMX2 | -0.103944176 | 6.525855159 | -2.761652248 | 0.005985535 |
| AC005351.1 | -0.252822145 | 0.948307855 | -2.760933762 | 0.005998495 |
| KIF5A | 0.476849824 | -2.051761755 | 2.760696538 | 0.00600278 |
| LPAR1 | -0.499975652 | 0.876539517 | -2.759970741 | 0.006015906 |
| UAP1L1 | -0.2326159 | 4.233175583 | -2.759910279 | 0.006017001 |
| SLC37A2 | -0.565301972 | 1.708182912 | -2.759033723 | 0.006032891 |
| RP6-42F4.1 | 0.165438513 | 3.163980643 | 2.75827726 | 0.006046635 |
| MRPL17 | -0.152998791 | 4.808000558 | -2.757538594 | 0.006060082 |
| CAHM | -0.209657919 | 3.022135395 | -2.757440637 | 0.006061868 |
| TMED2 | 0.102457725 | 8.048271311 | 2.757075787 | 0.006068522 |
| GPRC5D | 0.353282214 | -0.807444567 | 2.757042191 | 0.006069135 |
| PPIAP31 | -0.157740478 | 5.202645982 | -2.75678114 | 0.0060739 |
| WAC-AS1 | -0.127091802 | 6.237425812 | -2.755487337 | 0.006097568 |
| P4HA2 | 0.354158365 | -0.725761335 | 2.755469472 | 0.006097895 |
| CTD-2574D22.4 | -0.170037009 | 2.2872798 | -2.755253357 | 0.006101857 |
| PRKD3 | 0.344103347 | 4.418632135 | 2.755056939 | 0.00610546 |
| COMMD4 | 0.123762729 | 4.027059517 | 2.754942026 | 0.006107568 |
| WDR74 | 0.151721666 | 5.428283603 | 2.754345015 | 0.006118534 |
| SNORD15B | -0.200948535 | 1.949148272 | -2.753742379 | 0.006129621 |
| OLR1 | 0.685695402 | 2.861059135 | 2.753667409 | 0.006131001 |
| SCD5 | -0.426626247 | 2.031553889 | -2.753647137 | 0.006131375 |
| RP3-395C13.1 | -0.310926844 | 0.828199253 | -2.753262323 | 0.006138466 |
| PFKM | 0.286119269 | 2.05116548 | 2.753223783 | 0.006139176 |
| RP11-17A1.3 | -0.329474922 | -0.346089568 | -2.752982911 | 0.006143619 |
| KPTN | -0.163944343 | 4.38199869 | -2.752411047 | 0.006154178 |
| TECRP2 | -0.266007537 | -0.02474362 | -2.752383136 | 0.006154694 |
| MAG | 0.364918162 | -2.829427136 | 2.751930642 | 0.006163061 |
| TRGC1 | -0.432200537 | 5.991021278 | -2.751465942 | 0.006171665 |
| RP3-393E18.2 | -0.325890293 | -1.503245798 | -2.751058289 | 0.006179222 |
| PRKAA1 | 0.124908569 | 6.470271384 | 2.750755922 | 0.006184832 |
| ZNF646 | -0.13342386 | 4.730739197 | -2.750685955 | 0.006186131 |
| IGFBP5 | 0.753582805 | -2.29967811 | 2.750641515 | 0.006186956 |
| GFRA3 | 0.427196975 | -2.268327976 | 2.749956914 | 0.006199679 |
| GUCA1B | 0.242586615 | 1.914961112 | 2.749914921 | 0.006200461 |
| HEG1 | 0.335837376 | 2.121042467 | 2.749882571 | 0.006201063 |
| XX-FW83128A1.2 | 0.311258391 | -0.182922239 | 2.749659051 | 0.006205223 |
| AC090587.4 | 0.168264946 | 3.763509176 | 2.749399554 | 0.006210056 |
| STUB1 | -0.082972883 | 6.97886689 | -2.749373643 | 0.006210538 |
| SRSF8 | 0.301065596 | 3.707541403 | 2.749332586 | 0.006211303 |
| SLC6A4 | 0.340965383 | -3.537788383 | 2.749321937 | 0.006211502 |
| ANXA2P1 | -0.434090875 | 1.464034947 | -2.749152234 | 0.006214665 |
| FAM102B | 0.189172021 | 4.834498225 | 2.748814846 | 0.006220958 |
| NKD2 | 0.757654731 | -0.515858058 | 2.748771286 | 0.006221771 |
| ELL3 | -0.280528079 | -2.123480374 | -2.7484388 | 0.006227978 |
| RP11-141C7.4 | -0.330329643 | -0.348759735 | -2.74821285 | 0.0062322 |
| LL22NC03-80A10.6 | -0.170494162 | 3.108751517 | -2.747844753 | 0.006239084 |
| CYP51A1 | 0.206303777 | 1.39930442 | 2.747433249 | 0.006246787 |
| C19orf57 | 0.343460403 | 1.053536015 | 2.74737025 | 0.006247967 |
| KRT8P15 | 0.342105544 | -1.51826965 | 2.746688829 | 0.006260744 |
| DNAJC8 | 0.080729207 | 7.336510231 | 2.746150714 | 0.006270851 |
| SNORD3A | -0.454074965 | 3.153806747 | -2.746112427 | 0.00627157 |
| LINC00856 | 0.323006858 | -2.794977086 | 2.7460454 | 0.00627283 |
| CFAP141 | 0.272335244 | -0.01444735 | 2.745605031 | 0.006281114 |
| TPMT | -0.145667527 | 5.210005321 | -2.745596339 | 0.006281278 |
| GAPDHP71 | 0.314354111 | 0.096274228 | 2.745323175 | 0.006286422 |
| LRRC34 | -0.322817408 | 1.61971001 | -2.744973118 | 0.006293019 |
| RP11-142C4.5 | -0.426626859 | -0.3612036 | -2.744651056 | 0.006299094 |
| RUFY3 | -0.244782735 | 4.141494399 | -2.744090498 | 0.006309681 |
| RP3-323P24.3 | -0.243441866 | 0.680264888 | -2.743828406 | 0.006314636 |
| CEACAM6 | 0.828241062 | 4.812969407 | 2.743306853 | 0.006324508 |
| CCDC13 | -0.295543178 | 0.041750029 | -2.743140627 | 0.006327657 |
| AC009299.5 | 0.360290607 | 4.535507156 | 2.743076923 | 0.006328864 |
| RP11-461A8.4 | -0.280903067 | 0.071654521 | -2.742989999 | 0.006330512 |
| BLNK | -0.557594217 | 1.837615306 | -2.742910455 | 0.00633202 |
| RP11-400L8.2 | -0.279838672 | -0.01643765 | -2.742900748 | 0.006332204 |
| DDX3YP1 | 0.530158799 | -1.058659078 | 2.742868627 | 0.006332813 |
| RP11-151F5.2 | 0.209882414 | 1.682804018 | 2.742732983 | 0.006335386 |
| EEFSEC | -0.118386198 | 5.293488948 | -2.742282309 | 0.00634394 |
| RP11-172F4.2 | -0.147242647 | 6.06769092 | -2.741303387 | 0.006362557 |
| LINC00263 | -0.293958685 | 1.884874238 | -2.740991564 | 0.006368498 |
| MRTO4 | -0.18000338 | 5.134408348 | -2.740958158 | 0.006369135 |
| RP11-95P2.1 | -0.205169743 | 2.411527972 | -2.74062622 | 0.006375465 |
| SNX6 | -0.090020674 | 6.105449116 | -2.740271731 | 0.006382231 |
| JAKMIP3 | 0.321742278 | -1.717044982 | 2.740232424 | 0.006382981 |
| C16orf71 | -0.292226599 | -2.361151788 | -2.740142133 | 0.006384706 |
| MGEA5 | 0.14698205 | 7.486428566 | 2.739915224 | 0.006389042 |
| AP001462.6 | -0.182156587 | 1.710513287 | -2.739674536 | 0.006393644 |
| AC138035.2 | 0.253491872 | 1.33824332 | 2.739621524 | 0.006394658 |
| KB-1572G7.3 | -0.303005872 | -0.617879716 | -2.739613479 | 0.006394812 |
| DGCR8 | -0.10646555 | 5.179649138 | -2.739535638 | 0.006396302 |
| DAPP1 | -0.273053428 | 5.224002871 | -2.739379346 | 0.006399293 |
| TRMT61B | -0.112491567 | 4.760193922 | -2.739058097 | 0.006405445 |
| CTB-50L17.9 | 0.250466244 | 0.841092197 | 2.738607224 | 0.006414089 |
| EBI3 | 0.457992369 | 0.556996055 | 2.738513635 | 0.006415884 |
| PANK4 | -0.161231526 | 3.572837279 | -2.738048457 | 0.006424815 |
| RPL12P4 | -0.149811547 | 10.61418705 | -2.737635858 | 0.006432746 |
| AC005943.4 | -0.290107448 | -0.404201613 | -2.736778942 | 0.006449246 |
| UBE2Q1 | 0.066830177 | 6.909061406 | 2.736765757 | 0.0064495 |
| RP11-254B13.1 | 0.17856318 | 5.100292078 | 2.736546596 | 0.006453727 |
| AC108479.2 | -0.106377001 | 4.474603029 | -2.736277829 | 0.006458913 |
| LRPPRC | -0.145136795 | 6.302529188 | -2.736226329 | 0.006459907 |
| CTB-31O20.2 | 0.213376066 | 5.190572585 | 2.736196181 | 0.006460489 |
| UBTF | -0.126748481 | 6.852050436 | -2.735593095 | 0.006472143 |
| RP11-245C23.3 | -0.310888068 | 1.145182015 | -2.73555836 | 0.006472815 |
| RHOT1P2 | -0.224757769 | 1.273063633 | -2.734774045 | 0.006488 |
| WDR66 | -0.342213168 | -1.373985648 | -2.734367793 | 0.006495878 |
| CTD-2033A16.2 | -0.116655548 | 7.210630453 | -2.734176618 | 0.006499589 |
| LSM4 | 0.112765289 | 6.469144402 | 2.734078712 | 0.00650149 |
| RP11-77H9.5 | 0.20018501 | 2.039495285 | 2.734035813 | 0.006502323 |
| CAPS | -0.165677624 | 3.656314996 | -2.733997624 | 0.006503064 |
| LYRM5 | -0.11791801 | 5.653455396 | -2.733330732 | 0.006516029 |
| DPYSL4 | 0.446467603 | -1.864046213 | 2.733038871 | 0.00652171 |
| GBAS | 0.110663177 | 5.752954718 | 2.732723875 | 0.006527846 |
| SLC8A1-AS1 | -0.421399236 | -3.435061618 | -2.732258175 | 0.006536928 |
| GLOD4 | -0.08956605 | 5.563713724 | -2.732160803 | 0.006538828 |
| RP5-887A10.1 | -0.397101161 | -0.654541897 | -2.73016596 | 0.00657787 |
| CTDSPL | 0.392963617 | 3.010733848 | 2.729988854 | 0.006581347 |
| RNF32 | -0.403945126 | -1.058484048 | -2.729547721 | 0.006590013 |
| B4GALT7 | 0.106480669 | 4.073310326 | 2.729331915 | 0.006594256 |
| CYP4F3 | 0.682995534 | 3.368920049 | 2.728628063 | 0.006608112 |
| ZNF789 | -0.136127113 | 3.312392271 | -2.728386194 | 0.00661288 |
| PTDSS1 | -0.140356717 | 7.079988257 | -2.727535736 | 0.006629669 |
| GPATCH11P1 | -0.264886127 | 0.864666328 | -2.727130923 | 0.006637674 |
| WNK2 | 0.469866104 | -4.445660714 | 2.727082564 | 0.00663863 |
| MFAP2 | 0.569080562 | -1.457266451 | 2.726888196 | 0.006642478 |
| RP3-395M20.8 | 0.317708898 | 2.038485588 | 2.726876762 | 0.006642704 |
| REEP3 | -0.274177897 | 4.474547067 | -2.72683795 | 0.006643472 |
| C16orf46 | -0.249698043 | 1.227273 | -2.725561256 | 0.006668795 |
| SEPHS2 | -0.129410551 | 7.078843115 | -2.725438662 | 0.006671231 |
| CABP5 | 0.388404888 | -2.246915654 | 2.725295822 | 0.006674071 |
| NELL2 | 0.472390449 | 1.45623626 | 2.724715092 | 0.006685626 |
| SMG9 | -0.124909048 | 4.612486697 | -2.724648704 | 0.006686948 |
| CCL18 | 0.541308839 | -0.987184846 | 2.722885372 | 0.006722152 |
| U82695.10 | 0.367795248 | 2.128198516 | 2.722798296 | 0.006723895 |
| AC092171.2 | -0.347012223 | -0.956509138 | -2.722778276 | 0.006724296 |
| ST14 | 0.538201025 | 3.547593608 | 2.722666757 | 0.006726529 |
| LPAL2 | -0.459215339 | 0.373464994 | -2.722415296 | 0.006731565 |
| RAMP3 | 0.399159175 | -1.85438567 | 2.722333143 | 0.006733212 |
| ARSE | -0.506233908 | -1.918067371 | -2.721933306 | 0.006741229 |
| FAM175B | 0.098902716 | 5.648639643 | 2.721868206 | 0.006742536 |
| HNRNPDP1 | 0.143884258 | 2.393234532 | 2.721499152 | 0.006749945 |
| RP11-572M11.4 | -0.273131842 | 0.18021482 | -2.721480961 | 0.00675031 |
| UBE2D4 | -0.136879839 | 3.566476503 | -2.720981549 | 0.006760349 |
| ZCWPW1 | -0.176645801 | 3.232858697 | -2.720519594 | 0.006769647 |
| ITGB3 | 0.721413747 | 0.957131849 | 2.720342518 | 0.006773214 |
| C15orf41 | -0.235863629 | 1.055331556 | -2.720258151 | 0.006774914 |
| ZNF396 | -0.285453695 | -0.515470773 | -2.720214491 | 0.006775794 |
| RP11-195B17.1 | -0.222255662 | 1.992453283 | -2.719532771 | 0.006789547 |
| HELB | 0.16335801 | 3.991967739 | 2.719324945 | 0.006793745 |
| PANK3 | 0.10137163 | 5.456208882 | 2.718309009 | 0.006814299 |
| RP11-56F10.3 | -0.437676727 | 0.518540248 | -2.717571631 | 0.006829252 |
| LGALS2 | -0.817301101 | 3.977233821 | -2.717480026 | 0.006831112 |
| ST13 | -0.131987322 | 7.345054365 | -2.717300075 | 0.006834766 |
| AC046143.3 | -0.309553782 | -0.6709339 | -2.71697361 | 0.006841401 |
| CIDEB | -0.390707471 | 5.629650993 | -2.716922932 | 0.006842431 |
| RPL3P3 | 0.252467749 | 1.662435006 | 2.716865724 | 0.006843595 |
| RP5-1028K7.2 | -0.390538042 | -0.363394194 | -2.716406811 | 0.006852934 |
| RP11-286H14.4 | 0.105510906 | 4.737375148 | 2.716319909 | 0.006854704 |
| PAQR7 | -0.186991433 | 5.113489128 | -2.716311476 | 0.006854875 |
| AL512428.1 | -0.353045519 | 1.280540776 | -2.716184643 | 0.006857459 |
| PRDX2 | 0.424454445 | 6.433773687 | 2.716175447 | 0.006857646 |
| DPY19L2P4 | -0.355038189 | -3.116597409 | -2.716036375 | 0.006860481 |
| CTB-119C2.1 | 0.257490594 | 1.499304196 | 2.715884155 | 0.006863584 |
| RP11-676M6.1 | 0.159843051 | 8.482922461 | 2.715882491 | 0.006863618 |
| HAR1B | 0.351427084 | -0.927423295 | 2.71551771 | 0.00687106 |
| RP11-45H22.1 | -0.275659348 | -0.810861034 | -2.715305103 | 0.006875401 |
| C1orf172 | 0.330199368 | -1.863100693 | 2.714810243 | 0.006885514 |
| FIBP | -0.101987504 | 6.131095191 | -2.714781303 | 0.006886105 |
| FRMPD3 | -0.391366411 | -2.608289263 | -2.714490028 | 0.006892065 |
| AHNAK2 | 0.608457273 | -3.82515065 | 2.713907092 | 0.006904005 |
| DLG4 | -0.217804921 | 4.973344373 | -2.713906987 | 0.006904007 |
| RPL24P8 | -0.159430603 | 6.700369471 | -2.713890583 | 0.006904344 |
| ANKLE1 | -0.265657645 | 3.978617132 | -2.71376281 | 0.006906964 |
| GNG10 | 0.286126218 | -0.746951172 | 2.713390288 | 0.006914607 |
| AC007255.8 | -0.394235243 | -1.461267543 | -2.712994749 | 0.006922731 |
| NUP214 | -0.082107315 | 5.830280116 | -2.712970955 | 0.006923219 |
| CRIP1P4 | -0.396067933 | -0.733437601 | -2.712637885 | 0.006930067 |
| USP3-AS1 | -0.255340806 | -0.206214903 | -2.712128364 | 0.006940555 |
| RP11-603J24.5 | -0.302768393 | 0.004451499 | -2.711592147 | 0.006951607 |
| RP11-343B5.1 | 0.12748 | 3.901708079 | 2.709588077 | 0.006993056 |
| ARPC1A | 0.113914586 | 6.663455286 | 2.709015299 | 0.007004943 |
| HIST2H2BA | -0.381834685 | -0.708953889 | -2.70881563 | 0.007009091 |
| BASP1 | 0.552569066 | 6.542694765 | 2.708329342 | 0.007019202 |
| ZNF23 | -0.244364175 | -0.536080186 | -2.708167065 | 0.00702258 |
| SLC7A3 | 0.327132989 | -2.922334771 | 2.707903026 | 0.007028078 |
| CERS5 | 0.089297774 | 5.001709619 | 2.707741062 | 0.007031453 |
| ANO5 | -0.67634903 | -2.302331046 | -2.707612542 | 0.007034131 |
| FXYD1 | 0.37259918 | -2.145214394 | 2.707445351 | 0.007037618 |
| CD63 | 0.160102546 | 9.021017681 | 2.707331687 | 0.007039989 |
| RPL3P12 | 0.286705974 | 0.601286245 | 2.707164356 | 0.00704348 |
| KLHL33 | -0.544074697 | -0.713914497 | -2.707159516 | 0.007043581 |
| PKP4 | -0.19692483 | 4.868024612 | -2.707153758 | 0.007043702 |
| FCRL1 | -0.549711547 | 2.046575635 | -2.706937846 | 0.00704821 |
| AC097495.2 | -0.268273392 | 1.035677205 | -2.706824663 | 0.007050574 |
| ARMCX6 | 0.108162307 | 4.770812337 | 2.706251725 | 0.007062552 |
| MIR4518 | -0.339951629 | -0.22974361 | -2.706041116 | 0.00706696 |
| GPI | 0.214579679 | 7.013725631 | 2.705839926 | 0.007071173 |
| PIGQ | 0.159646611 | 4.60984387 | 2.705523422 | 0.007077805 |
| RP11-109G23.3 | 0.311383235 | -0.528964844 | 2.705173294 | 0.007085149 |
| SDHAP1 | -0.139176623 | 4.12348569 | -2.70501943 | 0.007088378 |
| PKD1 | -0.112843987 | 5.328680865 | -2.704992333 | 0.007088947 |
| RP5-973M2.2 | -0.350361969 | -0.540504676 | -2.704943119 | 0.00708998 |
| TMEM147 | -0.122314095 | 6.047365818 | -2.704561692 | 0.007097993 |
| EFNB3 | -0.496876315 | -2.840325517 | -2.704560061 | 0.007098027 |
| RP11-446N19.1 | -0.291776775 | 2.025866319 | -2.704136897 | 0.007106926 |
| ENTPD2 | 0.461497722 | -1.882164205 | 2.704008341 | 0.007109631 |
| ST6GALNAC3 | 0.528491572 | -0.873786111 | 2.703902363 | 0.007111862 |
| FFAR3 | 0.467703155 | -0.479442744 | 2.703496419 | 0.007120414 |
| RAPGEF5 | 0.460993536 | -3.033759234 | 2.703077561 | 0.007129247 |
| RP11-469E19.1 | 0.28782021 | 0.453537125 | 2.702884307 | 0.007133326 |
| SCGB1B2P | -0.31709661 | 0.010247214 | -2.702562423 | 0.007140125 |
| THOC6 | -0.118719847 | 6.01001118 | -2.702462372 | 0.007142239 |
| PRKCQ-AS1 | -0.253216104 | 3.953181343 | -2.702251339 | 0.007146701 |
| BZW2 | -0.185444926 | 6.471740194 | -2.701734659 | 0.007157635 |
| LINC00654 | -0.466480912 | 1.631097677 | -2.701557512 | 0.007161387 |
| KIF26B | -0.716606567 | 0.504647698 | -2.700827556 | 0.007176867 |
| AK3P3 | -0.233830765 | 0.899410893 | -2.700089014 | 0.00719256 |
| ATG10 | -0.149240484 | 2.617967096 | -2.700022516 | 0.007193975 |
| TRAF2 | 0.104602977 | 4.487530598 | 2.699795376 | 0.007198809 |
| FBLN7 | 0.367338878 | -1.863479985 | 2.699752792 | 0.007199715 |
| SNAP29 | -0.093509845 | 6.165229771 | -2.699482183 | 0.007205478 |
| CACNA1I | 0.451927513 | -0.330322026 | 2.698977244 | 0.007216242 |
| WDR65 | -0.338612059 | -3.613128927 | -2.698738912 | 0.007221328 |
| RP11-290D2.3 | -0.286754904 | 0.211808354 | -2.698659991 | 0.007223013 |
| ZNF584 | -0.189305484 | 2.612357601 | -2.698456661 | 0.007227356 |
| RP11-252K23.2 | -0.27314449 | 0.013055529 | -2.6983371 | 0.00722991 |
| FKBP9L | -0.357436635 | -1.935065765 | -2.698248004 | 0.007231814 |
| MX1 | 0.42786712 | 4.239654116 | 2.698066728 | 0.00723569 |
| RGS12 | 0.244344327 | 2.734217889 | 2.698043555 | 0.007236185 |
| HOXB-AS3 | -0.931888314 | 1.601113284 | -2.697949879 | 0.007238189 |
| CYP4F29P | 0.549206315 | 0.213139521 | 2.697761346 | 0.007242223 |
| RPS15AP24 | -0.134533081 | 7.622004419 | -2.697534229 | 0.007247085 |
| CASC14 | -0.3874063 | -2.304178954 | -2.696163994 | 0.007276482 |
| PCDHGA1 | -0.40158065 | -3.105584339 | -2.696143031 | 0.007276933 |
| ALPK1 | -0.168084613 | 4.201755237 | -2.696139324 | 0.007277012 |
| NUTF2P2 | -0.211193766 | 4.904373842 | -2.695610151 | 0.007288394 |
| ARHGAP24 | 0.508860703 | 1.777428969 | 2.695066606 | 0.007300103 |
| COX6B1 | -0.107095874 | 8.624454569 | -2.694993883 | 0.00730167 |
| MAGI1 | 0.504326951 | -4.561896033 | 2.694973405 | 0.007302112 |
| PHKA1 | 0.610914694 | -0.319095219 | 2.694860263 | 0.007304552 |
| ARHGAP5 | 0.429968403 | 3.799916882 | 2.694583784 | 0.007310517 |
| IRF7 | -0.237138167 | 6.675533357 | -2.694539677 | 0.007311469 |
| LINC00115 | 0.202938025 | 2.769188217 | 2.693245105 | 0.007339462 |
| MDN1 | -0.148124038 | 5.670177225 | -2.693202369 | 0.007340387 |
| UNC13B | -0.601485175 | 1.60556681 | -2.692532833 | 0.007354904 |
| ATP7A | -0.158509558 | 3.459699925 | -2.691083952 | 0.007386408 |
| SFT2D1 | 0.129100077 | 5.242776848 | 2.690537301 | 0.007398325 |
| ADAM32 | 0.421232843 | -2.414069293 | 2.690399815 | 0.007401325 |
| GFOD2 | -0.155399201 | 3.693382779 | -2.689719638 | 0.007416183 |
| SLC9A3R2 | -0.492359259 | 3.239385832 | -2.689703777 | 0.00741653 |
| C5orf22 | -0.122010995 | 4.820934452 | -2.689645962 | 0.007417794 |
| HMGN2P3 | 0.209645657 | 6.362605477 | 2.68948743 | 0.007421262 |
| HLA-K | 0.28094555 | 4.215189652 | 2.689286777 | 0.007425653 |
| NPRL2 | -0.102884344 | 4.794079878 | -2.689028175 | 0.007431315 |
| RP1-65P5.3 | -0.274049088 | 0.503955691 | -2.689010026 | 0.007431713 |
| SLC7A8 | 0.433330983 | 1.091512081 | 2.688919163 | 0.007433703 |
| MICU1 | -0.14482144 | 5.749943242 | -2.688879881 | 0.007434564 |
| CDC42BPA | 0.793595009 | 0.280645378 | 2.688758454 | 0.007437225 |
| FAM98A | -0.139186896 | 4.719755988 | -2.688751457 | 0.007437379 |
| RP11-175K6.1 | 0.40874267 | -2.467060322 | 2.688234812 | 0.007448712 |
| AP000350.5 | -0.684852723 | 0.317902476 | -2.687950454 | 0.007454956 |
| SNORD99 | -0.231790238 | 1.847997205 | -2.686990553 | 0.007476069 |
| RP5-890O3.9 | -0.188863445 | 3.090291858 | -2.68693729 | 0.007477242 |
| AC004453.8 | -0.124520656 | 10.08560875 | -2.686780522 | 0.007480695 |
| USP18 | 0.362023225 | 2.521924407 | 2.686683373 | 0.007482836 |
| COL3A1 | 0.586225878 | -3.726307291 | 2.686500609 | 0.007486866 |
| ANKRD39 | 0.150685693 | 1.722728347 | 2.686467969 | 0.007487585 |
| MIR221 | 0.300492262 | 3.325386111 | 2.686465277 | 0.007487645 |
| RP11-477E3.2 | 0.268263316 | 1.17477141 | 2.68622146 | 0.007493023 |
| NAV2-AS2 | -0.363135702 | -1.616250108 | -2.684886785 | 0.007522529 |
| AC147651.3 | 0.417496744 | -1.712532479 | 2.68469079 | 0.007526871 |
| EPHA2 | 0.486434105 | -1.767547244 | 2.684183717 | 0.007538114 |
| HGF | -0.600228049 | 4.435545717 | -2.683874664 | 0.007544973 |
| CTD-2002H8.2 | 0.217803093 | 2.511854711 | 2.683536367 | 0.007552489 |
| PPT1 | -0.202821642 | 8.476946863 | -2.683447654 | 0.007554461 |
| HBM | 0.755132737 | 1.674742797 | 2.68321149 | 0.007559712 |
| EBPL | -0.225214503 | 5.734370663 | -2.683186467 | 0.007560269 |
| TNPO2 | 0.117790903 | 5.679449786 | 2.683128554 | 0.007561557 |
| TMEM117 | 0.398775368 | -0.054915959 | 2.683076904 | 0.007562707 |
| GALE | 0.192352617 | 3.369193664 | 2.683022645 | 0.007563914 |
| PRR5 | 0.229198167 | 2.841622748 | 2.682943915 | 0.007565667 |
| RP11-218E20.3 | -0.443106145 | -3.188333533 | -2.682763955 | 0.007569673 |
| RP11-857B24.3 | 0.214945883 | 0.756199184 | 2.682675867 | 0.007571635 |
| SULT1A1 | -0.240929198 | 4.346005745 | -2.682653803 | 0.007572127 |
| NR1D1 | 0.211930948 | 3.585049379 | 2.682535779 | 0.007574757 |
| IBA57-AS1 | -0.277856203 | 0.585380608 | -2.682383044 | 0.007578161 |
| NPM1P6 | -0.176307134 | 6.900363843 | -2.682287165 | 0.007580299 |
| RPS2P7 | 0.172774803 | 4.940783672 | 2.682059434 | 0.007585378 |
| SNX19P2 | -0.256555952 | -1.402715299 | -2.682004626 | 0.007586601 |
| CTD-2152M20.2 | -0.208506092 | 1.725664449 | -2.681968215 | 0.007587414 |
| RNU6-1219P | -0.323824016 | -0.096392608 | -2.681671371 | 0.007594041 |
| AP000345.2 | -0.411704618 | -1.028006521 | -2.681589717 | 0.007595865 |
| PRAF2 | 0.136855017 | 4.650478341 | 2.681565772 | 0.0075964 |
| RP11-439E19.3 | -0.439216756 | 2.620450159 | -2.681415388 | 0.007599761 |
| LINC00933 | -0.274922334 | -0.547899327 | -2.679577531 | 0.007640938 |
| RP11-91P24.6 | -0.250462118 | 1.638805515 | -2.679016919 | 0.007653538 |
| RP11-166D19.1 | -0.567913843 | -3.958730295 | -2.678727812 | 0.007660043 |
| OLIG1 | -0.487789261 | 3.808477434 | -2.678552859 | 0.007663982 |
| CREG2 | 0.516585105 | -3.320304322 | 2.678261623 | 0.007670543 |
| ARPC3 | -0.120199816 | 7.891835436 | -2.677338012 | 0.007691385 |
| HNRNPKP1 | 0.174121212 | 2.386569866 | 2.676212403 | 0.007716853 |
| CACNA1A | 0.341560158 | -0.761703191 | 2.676108507 | 0.007719208 |
| TRMT10C | -0.147143962 | 5.627895621 | -2.676030128 | 0.007720984 |
| FABP5 | 0.26667286 | 2.995684131 | 2.676017255 | 0.007721276 |
| ERVFRD-1 | -0.624948605 | 0.503215723 | -2.675918859 | 0.007723507 |
| TMEM97 | 0.245291674 | 4.417937623 | 2.675742329 | 0.007727511 |
| VIM | -0.199139685 | 11.27129871 | -2.675677411 | 0.007728984 |
| C14orf39 | 0.367834274 | -2.578365911 | 2.675587067 | 0.007731035 |
| RP11-600F24.2 | 0.214391374 | 1.440823275 | 2.674138075 | 0.007763986 |
| PTCD3 | -0.093373186 | 4.887896866 | -2.67382839 | 0.007771045 |
| C1QTNF3 | -0.216338892 | 2.460587747 | -2.673113787 | 0.007787355 |
| FBXL6 | -0.102968802 | 4.810035355 | -2.67294955 | 0.007791108 |
| PSME3 | -0.090033059 | 6.832593662 | -2.672102604 | 0.007810487 |
| RP1-229K20.5 | 0.459982986 | 1.818468029 | 2.671964987 | 0.00781364 |
| CTA-384D8.34 | -0.416905048 | 2.029094773 | -2.671334833 | 0.007828092 |
| TRIM8 | 0.1617141 | 7.482765916 | 2.671280487 | 0.00782934 |
| RP11-39K24.4 | 0.34955403 | 1.548639633 | 2.670577301 | 0.007845497 |
| GOT1 | -0.152220672 | 4.571684842 | -2.669721812 | 0.007865195 |
| RP11-867G23.3 | 0.364573852 | -2.211211786 | 2.669347946 | 0.007873817 |
| GPR137B | 0.310027124 | 3.34553763 | 2.669338363 | 0.007874039 |
| AP000442.1 | -0.23236209 | 0.198068933 | -2.669328195 | 0.007874273 |
| RP11-757O6.1 | -0.342027023 | -0.752060635 | -2.668435435 | 0.007894898 |
| NIFKP6 | -0.284097595 | 0.900431661 | -2.667964938 | 0.007905787 |
| CTB-13F3.1 | 0.273787553 | 1.155504386 | 2.667486303 | 0.007916878 |
| DCUN1D4 | -0.108323851 | 3.897305238 | -2.667261809 | 0.007922085 |
| EXOSC8 | 0.107112904 | 5.138504562 | 2.667047753 | 0.007927053 |
| RP11-10G12.1 | 0.19669253 | 1.610564416 | 2.666883992 | 0.007930855 |
| GSR | -0.1764342 | 6.972432318 | -2.666593109 | 0.007937613 |
| HOMER1 | -0.378611423 | -0.263779772 | -2.666425895 | 0.0079415 |
| HIST1H1B | -0.513565276 | 1.316301372 | -2.665664068 | 0.007959232 |
| PRKAG2-AS1 | -0.24409697 | 3.573488675 | -2.665452111 | 0.007964171 |
| RP11-933H2.4 | -0.394567267 | -0.778382962 | -2.665448444 | 0.007964257 |
| MALT1 | 0.200489828 | 4.862362956 | 2.664924636 | 0.007976476 |
| CYP51A1P1 | 0.197760261 | 2.331515767 | 2.664833413 | 0.007978606 |
| KIF3B | 0.125375339 | 4.94831357 | 2.664489291 | 0.007986645 |
| HTR2A | 0.390200385 | -3.728118535 | 2.664407076 | 0.007988566 |
| BAK1P1 | 0.111223052 | 4.135983918 | 2.664215423 | 0.007993048 |
| SLFN13 | 0.30050205 | 4.218904791 | 2.663714456 | 0.008004772 |
| TRBV5-5 | -0.340353157 | -0.446880602 | -2.663667786 | 0.008005865 |
| RP11-235E17.3 | -0.273622911 | -0.322148424 | -2.663468371 | 0.008010536 |
| SYNJ2BP | -0.15421483 | 4.386991673 | -2.662525537 | 0.008032658 |
| ZNF835 | -0.505973213 | -1.360018926 | -2.661873156 | 0.008047996 |
| AKT3-IT1 | 0.379521014 | -0.976398795 | 2.660933438 | 0.008070137 |
| CD38 | -0.308522872 | 6.108055057 | -2.660864358 | 0.008071767 |
| SLC22A18 | -0.241268921 | 3.219184501 | -2.660689467 | 0.008075894 |
| SLC35B2 | -0.114490702 | 6.560829592 | -2.660171637 | 0.008088126 |
| FAM86DP | -0.221816436 | 2.697868317 | -2.65990224 | 0.008094496 |
| PHB1P11 | -0.199943003 | 2.482350239 | -2.659607866 | 0.008101462 |
| CAP2P1 | 0.301011515 | 0.200530635 | 2.659465617 | 0.008104829 |
| MAN2A2 | 0.132820526 | 7.235128821 | 2.659284291 | 0.008109125 |
| CACNG8 | -0.603425854 | -3.341328278 | -2.659149298 | 0.008112323 |
| MIR600HG | -0.264788392 | 2.139941019 | -2.659143678 | 0.008112457 |
| GRHPR | -0.132387266 | 5.49173481 | -2.659092141 | 0.008113678 |
| PCNPP5 | 0.104502459 | 6.497696254 | 2.658545821 | 0.008126638 |
| AL358813.1 | -0.332001191 | -0.595780463 | -2.658526894 | 0.008127087 |
| PGK1P2 | 0.145177195 | 4.894251092 | 2.65838858 | 0.008130371 |
| GABPB2 | -0.148702785 | 3.765315219 | -2.658142554 | 0.008136215 |
| REV3L | -0.100233147 | 4.967652198 | -2.658125874 | 0.008136612 |
| MRPL28 | 0.088424069 | 6.302808701 | 2.657999814 | 0.008139608 |
| C6orf25 | 0.620148138 | 3.819270961 | 2.657585857 | 0.008149454 |
| RYR1 | -0.483465927 | -0.350650778 | -2.657573098 | 0.008149758 |
| RP11-336K24.12 | -0.233069939 | 1.510422398 | -2.657148736 | 0.008159863 |
| AP4E1 | -0.092465466 | 4.079430299 | -2.65707343 | 0.008161657 |
| RP11-448G15.3 | 0.255025072 | 3.308567807 | 2.657042423 | 0.008162396 |
| RP11-379B8.1 | -0.413326889 | -0.854727653 | -2.656368339 | 0.008178475 |
| PAICS | -0.216515886 | 6.767789052 | -2.65565219 | 0.008195589 |
| ZNF385C | -0.419403374 | 1.763232178 | -2.655565488 | 0.008197663 |
| ORAI2 | 0.171648273 | 5.681268279 | 2.655249713 | 0.008205221 |
| RP11-663P9.2 | -0.18676998 | 3.027777286 | -2.654238693 | 0.008229461 |
| GYPA | 0.834116412 | 1.0172711 | 2.654078991 | 0.008233296 |
| STK40 | -0.127361428 | 6.37535722 | -2.653995142 | 0.00823531 |
| LSM5 | 0.134281376 | 3.998078536 | 2.653790357 | 0.008240231 |
| INVS | -0.122983346 | 4.257515836 | -2.653605737 | 0.008244669 |
| FAM185BP | -0.168311617 | 3.112077731 | -2.653466281 | 0.008248024 |
| GEMIN8P4 | 0.253538487 | 1.324967552 | 2.652849357 | 0.008262877 |
| RP5-855D21.3 | -0.186750116 | 2.224536167 | -2.652624639 | 0.008268293 |
| BIRC6 | -0.082100709 | 6.285042649 | -2.65194828 | 0.008284614 |
| OTUD1 | 0.327598666 | 6.394951615 | 2.651829624 | 0.00828748 |
| KRTCAP2 | -0.240905794 | 2.858721753 | -2.651661041 | 0.008291554 |
| CTD-2165H16.3 | -0.44805527 | 0.16543523 | -2.651361416 | 0.008298799 |
| PSPC1P1 | 0.240273631 | 1.612315268 | 2.651284485 | 0.00830066 |
| RP11-529J17.3 | -0.401021844 | -0.972005117 | -2.651249694 | 0.008301502 |
| INTS6-AS1 | -0.179033069 | 1.127152507 | -2.651081867 | 0.008305563 |
| FAM188B | 0.352923089 | -2.243989555 | 2.650887624 | 0.008310267 |
| BTF3L4 | 0.097597163 | 5.479188602 | 2.650805551 | 0.008312255 |
| CD247 | 0.392800149 | 3.567057465 | 2.650674189 | 0.008315437 |
| AC020910.2 | 0.20473679 | 2.309108374 | 2.650141135 | 0.008328364 |
| VWA3B | -0.412684885 | -4.439230169 | -2.649580286 | 0.008341984 |
| SYNE2 | 0.391335544 | 4.134342831 | 2.64944869 | 0.008345182 |
| LINC00641 | 0.19394625 | 4.87417265 | 2.649231211 | 0.008350471 |
| AF011889.2 | -0.334419532 | 2.178292922 | -2.64921572 | 0.008350848 |
| C2orf49 | 0.107508021 | 4.871335202 | 2.649055662 | 0.008354742 |
| TRIP10 | 0.302461756 | 2.820025763 | 2.648763976 | 0.008361843 |
| BCAP31 | -0.091543933 | 7.750976018 | -2.648618459 | 0.008365388 |
| LARP1P1 | -0.370382239 | -0.697902653 | -2.648281341 | 0.008373605 |
| OPHN1 | -0.360049131 | 1.059074749 | -2.647975496 | 0.008381066 |
| CCDC175 | 0.501414808 | -1.208453114 | 2.647616969 | 0.00838982 |
| AC145212.3 | -0.366051289 | -0.158711582 | -2.647214608 | 0.008399653 |
| NDUFAB1 | -0.121301607 | 5.931998261 | -2.646745752 | 0.008411125 |
| RP11-225B17.2 | 0.330848109 | 4.506169535 | 2.646541112 | 0.008416137 |
| FAM43A | -0.316183969 | 3.785201805 | -2.646485279 | 0.008417505 |
| AAK1 | 0.176922816 | 4.014024634 | 2.646324058 | 0.008421455 |
| TMBIM4 | -0.132071567 | 3.378676709 | -2.646293149 | 0.008422213 |
| COQ10A | -0.151612109 | 3.398290135 | -2.645728382 | 0.008436067 |
| HIST1H2BL | -0.381860084 | 1.124525595 | -2.644871504 | 0.008457125 |
| CTD-2012K14.2 | -0.313290707 | -0.108234167 | -2.644228114 | 0.008472968 |
| RP11-439C15.5 | 0.318030792 | -0.544488176 | 2.644127046 | 0.008475459 |
| ME3 | 0.349854827 | 3.78776378 | 2.643973357 | 0.008479248 |
| AMDHD1 | -0.404174634 | 0.160583441 | -2.643918459 | 0.008480602 |
| ZEB1-AS1 | -0.37577559 | 2.185748693 | -2.643693451 | 0.008486153 |
| FNDC3B | -0.205584389 | 5.84929591 | -2.643158334 | 0.008499369 |
| WRB | -0.14976002 | 3.291172427 | -2.641926763 | 0.008529854 |
| LPIN3 | 0.489871703 | -2.336868971 | 2.641484325 | 0.008540829 |
| RP11-439E19.1 | 0.346795838 | -0.711662078 | 2.641243896 | 0.008546799 |
| DUS4L | 0.123744791 | 2.754668053 | 2.641024821 | 0.008552242 |
| LDHAP7 | 0.170236071 | 4.640337119 | 2.640873286 | 0.008556008 |
| JAKMIP2 | -0.492353818 | 0.807549722 | -2.640717243 | 0.008559889 |
| SERBP1P1 | -0.120717867 | 6.045390359 | -2.64034607 | 0.008569125 |
| ATG5 | 0.081388491 | 4.944661367 | 2.639718356 | 0.008584764 |
| RP11-159J3.1 | 0.28491624 | 0.718507721 | 2.639566895 | 0.008588542 |
| PITRM1-AS1 | -0.279728274 | -0.142083609 | -2.638666584 | 0.008611027 |
| TNFSF9 | 0.486266298 | 3.478955235 | 2.638486593 | 0.008615529 |
| RPS2 | 0.133566988 | 10.51175328 | 2.638405034 | 0.008617569 |
| LSM12P1 | -0.131626223 | 7.063570364 | -2.637867637 | 0.008631025 |
| FAM124B | 0.374673894 | 3.772022827 | 2.637555944 | 0.008638838 |
| RP11-626A1.1 | -0.268399539 | -0.357111954 | -2.637435208 | 0.008641867 |
| RP6-145B8.3 | -0.19220502 | 4.065397968 | -2.636617542 | 0.008662399 |
| FAM160A2 | -0.131779201 | 6.144275638 | -2.636092266 | 0.008675613 |
| NOL10 | 0.071750459 | 5.111082496 | 2.635706251 | 0.008685335 |
| RP5-968P14.2 | 0.212504025 | 1.490385943 | 2.635634536 | 0.008687142 |
| MGLL | 0.389326703 | 4.634785964 | 2.635109285 | 0.008700388 |
| VPS28 | -0.079420528 | 6.619702476 | -2.634923579 | 0.008705076 |
| CD300C | -0.47970752 | 5.062844083 | -2.634738819 | 0.008709742 |
| DSC2 | -0.774603769 | 2.516894083 | -2.634514064 | 0.008715422 |
| WDSUB1 | -0.108630117 | 5.187122991 | -2.634296616 | 0.008720919 |
| NFIA | 0.566800989 | 0.418665768 | 2.633584621 | 0.008738943 |
| RN7SL425P | 0.311029592 | 0.871884976 | 2.633257739 | 0.008747228 |
| RP11-50E11.3 | 0.194711492 | 1.72656316 | 2.633185216 | 0.008749068 |
| NFATC3 | -0.082025385 | 6.017087605 | -2.6328725 | 0.008757003 |
| FUT6 | -0.481755925 | -2.603338357 | -2.632630186 | 0.008763155 |
| TUBA1B | 0.179353705 | 9.787677073 | 2.632296586 | 0.008771633 |
| COX7C | -0.128303413 | 7.152144884 | -2.63224525 | 0.008772938 |
| FAM167B | 0.305470247 | -0.505971941 | 2.632094499 | 0.008776771 |
| AC099850.1 | -0.214929064 | 0.755042987 | -2.631761443 | 0.008785247 |
| AC017028.8 | 0.292174896 | 0.348472285 | 2.631528905 | 0.008791168 |
| RP11-505K9.4 | 0.267972672 | -0.397772548 | 2.631527008 | 0.008791216 |
| ZNHIT2 | -0.155341735 | 4.014821035 | -2.631325807 | 0.008796343 |
| RP11-4C20.4 | -0.406734213 | 0.756624933 | -2.631079136 | 0.008802632 |
| RHD | 0.610517447 | -1.121255374 | 2.630896157 | 0.008807299 |
| RP11-166B2.1 | -0.236664498 | 2.167308746 | -2.630403366 | 0.008819881 |
| WDR96 | -0.389134397 | -2.59112808 | -2.630280618 | 0.008823017 |
| UQCRH | 0.103483505 | 7.557474628 | 2.629858785 | 0.008833803 |
| RP11-14N7.2 | 0.262132794 | 3.083198082 | 2.629383774 | 0.008845963 |
| RP1-153P14.8 | -0.336894972 | -1.137222309 | -2.629199109 | 0.008850695 |
| PTCH1 | -0.449941109 | 0.067894339 | -2.627931582 | 0.008883232 |
| RP13-766D20.2 | -0.28202721 | -0.473262333 | -2.627868444 | 0.008884855 |
| S100A11 | -0.408151612 | 8.703301128 | -2.627352186 | 0.00889814 |
| RP11-310E22.4 | -0.417636207 | -0.560891498 | -2.626829944 | 0.008911597 |
| SOD1 | 0.143825073 | 7.752863188 | 2.626748377 | 0.008913701 |
| HOXB9 | -0.928048493 | -0.787537231 | -2.626615067 | 0.00891714 |
| CBFA2T3 | -0.235031414 | 5.810348576 | -2.626545598 | 0.008918932 |
| SRI | 0.114753227 | 5.844498356 | 2.626494004 | 0.008920264 |
| ZNF33A | -0.137959475 | 5.647902035 | -2.626155849 | 0.008928994 |
| USP32P2 | -0.5054833 | -2.885960115 | -2.626152852 | 0.008929072 |
| PITPNC1 | 0.225875066 | 3.830519046 | 2.625581925 | 0.00894383 |
| ZNF793 | -0.407546594 | 0.357955402 | -2.625236631 | 0.008952767 |
| GPX7 | 0.198277163 | 6.180229993 | 2.625018101 | 0.008958426 |
| GSC | -0.414629258 | -1.344352427 | -2.624875876 | 0.008962112 |
| C6orf141 | 0.479052513 | -2.812015687 | 2.624783686 | 0.008964501 |
| SLC2A12 | -0.437940408 | -3.405623416 | -2.624701196 | 0.00896664 |
| EMBP1 | -0.218640474 | 3.886736207 | -2.624260095 | 0.008978083 |
| WDR82 | 0.080442609 | 7.125117791 | 2.624217531 | 0.008979188 |
| AF131215.2 | -0.467197239 | 0.764090427 | -2.623913116 | 0.008987094 |
| OLA1P1 | -0.115541477 | 5.996323701 | -2.623816085 | 0.008989615 |
| SATB2-AS1 | -0.366696667 | -3.307337628 | -2.623741323 | 0.008991559 |
| TAF9B | 0.137367076 | 4.856540303 | 2.623715721 | 0.008992224 |
| AC026471.6 | -0.307059118 | 0.779562965 | -2.623530236 | 0.008997047 |
| LDOC1L | -0.184902436 | 4.750881788 | -2.623437364 | 0.008999462 |
| LINC01001 | 0.297339719 | 2.198445928 | 2.623298756 | 0.009003069 |
| RP11-651L5.2 | 0.419375809 | 0.315154486 | 2.623112935 | 0.009007905 |
| RP1-168P16.1 | 0.313949081 | -0.392219889 | 2.622800617 | 0.00901604 |
| MIR4258 | 0.258437047 | 0.322812663 | 2.622340339 | 0.00902804 |
| AL353354.1 | 0.249949944 | 1.125702617 | 2.621666773 | 0.009045627 |
| SLC43A2 | 0.33083286 | 4.468749636 | 2.62164607 | 0.009046168 |
| RRM1 | 0.179163176 | 5.939629631 | 2.621643815 | 0.009046227 |
| ERCC6 | -0.120602606 | 2.686358639 | -2.621506639 | 0.009049812 |
| WDR19 | -0.172028071 | 3.281557809 | -2.621445825 | 0.009051402 |
| TGOLN2 | -0.095483787 | 8.784784477 | -2.621372601 | 0.009053317 |
| PDCD4 | 0.126223983 | 7.751312609 | 2.62136462 | 0.009053526 |
| CHD8 | 0.069559433 | 6.421989491 | 2.620989318 | 0.009063346 |
| SCML2 | 0.274125503 | 4.147536794 | 2.620549712 | 0.009074861 |
| RP11-341N2.1 | -0.306066828 | -0.769355212 | -2.620427058 | 0.009078076 |
| RPSAP8 | 0.283214161 | 0.813786402 | 2.620268724 | 0.009082228 |
| PI4KAP2 | 0.137174563 | 3.792013304 | 2.620111484 | 0.009086352 |
| AGPAT4 | -0.275890142 | 3.554461347 | -2.61966392 | 0.009098102 |
| RP11-245G13.2 | -0.451019472 | -1.334221215 | -2.619182631 | 0.009110753 |
| RP11-20D14.6 | 0.678559117 | 0.038837637 | 2.618820967 | 0.00912027 |
| PSMD2 | 0.073225425 | 7.010775574 | 2.618479093 | 0.009129274 |
| MYL12B | -0.091479498 | 8.968857384 | -2.61826868 | 0.009134819 |
| LINC00664 | 0.486177996 | -0.094089165 | 2.617787905 | 0.009147502 |
| RPS28 | 0.333348877 | 5.027094187 | 2.617510818 | 0.009154819 |
| RP11-632K20.7 | 0.187488876 | 4.797131226 | 2.617395428 | 0.009157867 |
| CTD-2215E18.1 | -0.313522045 | -0.493416493 | -2.617193765 | 0.009163197 |
| RGPD2 | 0.47621922 | -0.208356195 | 2.616696579 | 0.00917635 |
| RPS2P48 | 0.262036254 | 0.621884569 | 2.616029816 | 0.009194014 |
| ZNF318 | 0.169556759 | 5.283714834 | 2.615813025 | 0.009199764 |
| PRAG1 | -0.374474978 | 2.520795691 | -2.615780027 | 0.00920064 |
| CAPG | -0.292997099 | 7.795723491 | -2.61572115 | 0.009202202 |
| RING1 | -0.095072869 | 7.128521584 | -2.615341026 | 0.009212295 |
| TFE3 | 0.125374644 | 6.68567335 | 2.615194365 | 0.009216191 |
| CACNB2 | 0.598844521 | -3.211405755 | 2.614445696 | 0.009236105 |
| AC104135.2 | -0.448447379 | -0.776911758 | -2.613891726 | 0.009250865 |
| IZUMO1 | 0.326315517 | -3.214827515 | 2.613636675 | 0.009257668 |
| HNRNPA1P30 | 0.255027688 | -0.029874092 | 2.613447003 | 0.00926273 |
| IFNL3P1 | -0.3616561 | -0.773569696 | -2.613369897 | 0.009264788 |
| SLC16A3 | -0.282955515 | 5.513341391 | -2.613311959 | 0.009266335 |
| RFWD2 | -0.084251463 | 5.827530095 | -2.612823234 | 0.009279394 |
| ZNF287 | -0.321254313 | 0.294918106 | -2.612442832 | 0.00928957 |
| RP11-507E23.1 | -0.131802056 | 5.241476528 | -2.611825763 | 0.009306097 |
| RHBDF1 | 0.517886554 | 1.712235417 | 2.611579628 | 0.009312697 |
| HAT1 | 0.106469074 | 5.128040097 | 2.611413338 | 0.009317159 |
| UBA6 | 0.089171779 | 5.037895783 | 2.610831565 | 0.009332782 |
| AC187652.1 | -0.400105353 | -0.533882163 | -2.610247173 | 0.009348499 |
| POTEG | -0.341372801 | -3.376737637 | -2.610193874 | 0.009349933 |
| YWHAQ | 0.115486029 | 8.952837352 | 2.609860542 | 0.00935891 |
| PTGER1 | -0.496716398 | -0.06264272 | -2.609608686 | 0.009365697 |
| CXCL9 | 0.46605994 | -1.088523536 | 2.608753598 | 0.009388775 |
| FAM20A | 0.672029325 | -1.026486705 | 2.608740078 | 0.009389141 |
| MIR103A2 | 0.322063865 | 0.997680896 | 2.608721151 | 0.009389652 |
| PHLDA2 | 0.470682929 | 2.569672573 | 2.608236643 | 0.009402752 |
| KRTAP16-1 | 0.305218599 | -1.498003502 | 2.607765389 | 0.009415509 |
| TCN2 | -0.38111762 | 4.304817903 | -2.607700463 | 0.009417268 |
| COPS5 | -0.073413216 | 4.665867046 | -2.607334765 | 0.00942718 |
| BEND5 | 0.292783703 | 3.518516949 | 2.60643332 | 0.009451654 |
| RNASEH2B-AS1 | -0.329646116 | -0.045046908 | -2.606430146 | 0.00945174 |
| UPF3B | -0.114315113 | 5.608858846 | -2.606272175 | 0.009456035 |
| DPM1 | 0.076887342 | 6.920825773 | 2.605120041 | 0.00948741 |
| NPTXR | -0.343938744 | 0.392214279 | -2.604929044 | 0.00949262 |
| KLKB1 | -0.355090508 | -1.338717772 | -2.604789282 | 0.009496434 |
| PORCN | 0.153123455 | 3.501115466 | 2.604448169 | 0.009505749 |
| CTD-2616J11.3 | 0.289753719 | 0.81687707 | 2.603993107 | 0.009518189 |
| PLCL1 | 0.46494766 | 1.235592972 | 2.603755885 | 0.009524679 |
| KLHL13 | -0.517995368 | 1.059742437 | -2.603188526 | 0.009540218 |
| EEF1A1P22 | 0.236077424 | 3.365881964 | 2.603131394 | 0.009541784 |
| RP11-578C11.2 | -0.265976786 | 1.278729861 | -2.603086869 | 0.009543005 |
| PTRF | 0.691958169 | 3.249836607 | 2.602828067 | 0.009550102 |
| DDX10P1 | -0.331121929 | 0.532425792 | -2.602638664 | 0.009555299 |
| MORC1 | -0.46009784 | -2.202180979 | -2.602493857 | 0.009559275 |
| GLRX | -0.22069725 | 5.181433566 | -2.602226467 | 0.009566619 |
| CHCHD2 | 0.120516218 | 8.33366246 | 2.602192103 | 0.009567563 |
| RP11-655M14.13 | -0.451923095 | 0.350913773 | -2.601119399 | 0.00959708 |
| SCARNA8 | 0.28283949 | 0.40127934 | 2.600809989 | 0.009605609 |
| MAP1LC3A | 0.461255873 | 2.99806024 | 2.600550903 | 0.009612756 |
| ADAMTS4 | 0.463600177 | -2.789012404 | 2.600102234 | 0.009625144 |
| C11orf89 | -0.381950014 | -1.005413809 | -2.599710983 | 0.009635958 |
| ZBED3-AS1 | -0.332395499 | -0.238118955 | -2.599460583 | 0.009642885 |
| APOO | -0.136655132 | 3.779797176 | -2.59901589 | 0.009655198 |
| LARP6 | -0.518126673 | -1.998395208 | -2.598448492 | 0.009670928 |
| IDH3B | 0.082402576 | 7.433376969 | 2.598262992 | 0.009676076 |
| ALPL | 0.701198445 | 2.827464479 | 2.597714495 | 0.009691311 |
| MRPS18AP1 | -0.252403658 | 0.284953535 | -2.597507091 | 0.009697078 |
| RP11-227J5.3 | -0.160175733 | 1.306757657 | -2.597196506 | 0.009705719 |
| PIP4K2C | 0.192455909 | 5.085285225 | 2.597178177 | 0.009706229 |
| WDR44 | -0.09603552 | 4.912923064 | -2.597073626 | 0.00970914 |
| TJAP1 | -0.082367432 | 4.9169214 | -2.596931557 | 0.009713096 |
| BLZF2P | -0.325560167 | -0.521908547 | -2.596577022 | 0.009722975 |
| SLC25A4 | 0.297825938 | 0.348759206 | 2.596486589 | 0.009725496 |
| RPL6P27 | -0.101051183 | 10.42811491 | -2.596151011 | 0.009734858 |
| RPPH1 | -0.284205908 | 2.889063248 | -2.596041311 | 0.00973792 |
| KDM7A | 0.283896828 | 4.911133824 | 2.595509546 | 0.009752774 |
| RP4-545K15.3 | 0.240863905 | -0.050639281 | 2.594670884 | 0.009776243 |
| CCND2 | 0.307543962 | 6.845430024 | 2.593861466 | 0.009798942 |
| IQCJ-SCHIP1 | 0.399800609 | -0.91632568 | 2.59367279 | 0.00980424 |
| RP11-11N7.4 | 0.303162351 | 1.218181527 | 2.593443516 | 0.009810681 |
| RP11-108B14.4 | -0.195167215 | 1.856454022 | -2.593306006 | 0.009814546 |
| CTD-2521M24.5 | -0.351731322 | 0.454580376 | -2.593301924 | 0.009814661 |
| RP11-680G24.4 | -0.142578696 | 2.875228543 | -2.59296058 | 0.009824261 |
| UBE2S | 0.160278999 | 6.429023157 | 2.592878406 | 0.009826573 |
| ANXA8 | -0.560433868 | -3.625070716 | -2.59284651 | 0.009827471 |
| EIF4EBP3 | -0.188252165 | 3.334875336 | -2.592351366 | 0.009841416 |
| CTA-204B4.2 | -0.203602361 | 2.149562033 | -2.591845547 | 0.009855679 |
| EXOSC6 | 0.134265272 | 6.169845981 | 2.591429795 | 0.009867417 |
| PRR4 | -0.195476117 | 1.508033333 | -2.591162723 | 0.009874964 |
| LINC00494 | -0.444564145 | -1.928918039 | -2.591132636 | 0.009875814 |
| BMS1P6 | -0.433183316 | 0.526788153 | -2.591018681 | 0.009879036 |
| CTD-2619J13.9 | -0.265972327 | 2.556013395 | -2.590850462 | 0.009883794 |
| UNC45B | -0.302279962 | -3.494688209 | -2.590771086 | 0.00988604 |
| RP11-134P9.3 | 0.326334709 | 0.955978384 | 2.590713236 | 0.009887677 |
| UBE2G2 | -0.119215757 | 6.844588524 | -2.590650679 | 0.009889447 |
| FGFBP3 | 0.182473107 | 2.965212371 | 2.5902207 | 0.009901624 |
| SLC39A3 | -0.194634633 | 4.975480772 | -2.589769524 | 0.009914415 |
| RP11-384C4.6 | 0.296574576 | -0.184695175 | 2.589606631 | 0.009919037 |
| RAB11FIP5 | 0.483387708 | 1.204652216 | 2.589559558 | 0.009920373 |
| MROH1 | 0.13489733 | 3.39126062 | 2.589396539 | 0.009925001 |
| WASH2P | 0.085065785 | 5.317125047 | 2.589291895 | 0.009927973 |
| GTPBP2 | 0.096340706 | 6.243577455 | 2.588963889 | 0.009937293 |
| ACVRL1 | -0.568688772 | -0.503224871 | -2.588749311 | 0.009943394 |
| ALKBH4 | 0.127484872 | 3.788952145 | 2.588159535 | 0.009960181 |
| HIST1H2BM | -0.403816156 | -0.253174393 | -2.587997669 | 0.009964793 |
| KCNK5 | 0.4424493 | 4.339387309 | 2.587917402 | 0.00996708 |
| HNRNPA3P6 | -0.124887642 | 9.205475906 | -2.587527483 | 0.009978199 |
| C15orf57 | 0.109925383 | 3.461312254 | 2.587285065 | 0.009985118 |
| MAPT | -0.43793512 | -3.491288226 | -2.587057026 | 0.00999163 |
| ARHGDIB | -0.120477351 | 11.26160364 | -2.58645345 | 0.010008885 |
| FDPS | -0.090373148 | 6.334274881 | -2.586368291 | 0.010011321 |
| TMPRSS13 | 0.327928909 | 0.019928101 | 2.586281115 | 0.010013816 |
| AC002117.1 | -0.239088283 | 4.292322843 | -2.586239665 | 0.010015002 |
| RP5-902P8.10 | -0.22153681 | 2.575391402 | -2.585971151 | 0.010022691 |
| LBX2 | -0.383455703 | 1.12704233 | -2.585859669 | 0.010025885 |
| ELOVL5 | 0.153330693 | 7.288644103 | 2.585815652 | 0.010027146 |
| FCRL5 | -0.565953446 | -0.907641634 | -2.585570115 | 0.010034185 |
| SH2D4B | 0.348909147 | -2.482348181 | 2.584990189 | 0.010050825 |
| API5P1 | -0.142921361 | 5.480335999 | -2.584732666 | 0.010058223 |
| EIF2AK2 | 0.138725941 | 6.239293758 | 2.584714228 | 0.010058753 |
| RBMS2 | 0.250225123 | 4.026250125 | 2.584515915 | 0.010064453 |
| CAPN15 | -0.096227035 | 5.925288675 | -2.584456737 | 0.010066155 |
| SNORD13 | -0.410345777 | 1.240478125 | -2.584171722 | 0.010074353 |
| ZDHHC12 | 0.129250576 | 5.544896024 | 2.58414824 | 0.010075029 |
| RP11-380M21.4 | -0.304657512 | -0.047490906 | -2.584014958 | 0.010078865 |
| CD200R1 | -0.465364245 | 1.634235121 | -2.583710189 | 0.010087642 |
| WIPF3 | 0.515001869 | -2.687250044 | 2.582153794 | 0.010132572 |
| CRIPAK | -0.182446754 | 3.7863024 | -2.582145706 | 0.010132806 |
| RP11-712P20.2 | 0.350604015 | -0.839533358 | 2.581993425 | 0.010137211 |
| CTD-2650P22.1 | -0.274002842 | -0.397514613 | -2.581753927 | 0.010144144 |
| PTK2B | -0.138130563 | 7.337798855 | -2.581417894 | 0.010153877 |
| RP11-407P2.1 | 0.221688831 | 1.344906506 | 2.581376571 | 0.010155075 |
| TTC29 | -0.506249357 | -3.00479599 | -2.580322579 | 0.010185663 |
| RP5-827C21.2 | -0.347481036 | -0.750460343 | -2.58017719 | 0.010189889 |
| PPAP2C | 0.335789901 | -2.315532821 | 2.580167535 | 0.01019017 |
| CHPF | 0.305486408 | 2.047879947 | 2.580109986 | 0.010191843 |
| SARDH | -0.395202966 | 0.96154548 | -2.579170012 | 0.010219206 |
| POU2F2 | -0.332199312 | 4.954985776 | -2.579086999 | 0.010221626 |
| IRX3 | -0.838839896 | 0.334086997 | -2.579002719 | 0.010224083 |
| TTTY15 | 1.06859729 | 0.227138772 | 2.578808222 | 0.010229756 |
| TMEM198 | 0.335383119 | -1.362433639 | 2.578434505 | 0.010240663 |
| RP11-548H3.1 | -0.140720086 | 3.21893029 | -2.57835777 | 0.010242904 |
| TNFRSF18 | 0.496234802 | 2.587549083 | 2.578067777 | 0.010251377 |
| MEGF9 | -0.351350516 | 6.400746457 | -2.57724568 | 0.01027543 |
| MED12L | 0.545030299 | 2.413071566 | 2.576993798 | 0.010282809 |
| PDZK1IP1 | 0.509510608 | 0.383265432 | 2.576949793 | 0.010284099 |
| COMMD9 | 0.16312682 | 3.745198073 | 2.576599237 | 0.010294379 |
| PEX12 | 0.216416446 | 2.212584258 | 2.576577949 | 0.010295003 |
| LINGO3 | 0.223954177 | 0.885719211 | 2.576451551 | 0.010298712 |
| ATL1 | -0.288421575 | 1.90381216 | -2.575890864 | 0.010315179 |
| CAPN14 | -0.378042592 | -0.196549189 | -2.57499775 | 0.010341457 |
| RP11-999E24.3 | -0.381490156 | -1.232470494 | -2.574847653 | 0.01034588 |
| MYO7A | -0.525126468 | 0.488465018 | -2.573493251 | 0.01038586 |
| GLUD2 | -0.115635349 | 6.089473329 | -2.573287391 | 0.010391949 |
| RAB17 | 0.518936711 | -1.973041694 | 2.573139021 | 0.010396339 |
| SLC8B1 | -0.149122051 | 5.150422223 | -2.573070024 | 0.010398381 |
| NR2C2AP | -0.168191903 | 4.028737734 | -2.572648722 | 0.010410859 |
| RP4-673D20.4 | -0.444638856 | 0.04132573 | -2.572323276 | 0.010420507 |
| RP11-110G21.1 | 0.159994731 | 2.717600526 | 2.572237609 | 0.010423048 |
| CTD-2537I9.13 | -0.209080145 | 2.468702075 | -2.572184659 | 0.010424619 |
| RP1-66C13.3 | -0.230373418 | 4.654299957 | -2.571957373 | 0.010431364 |
| RNA5SP307 | -0.292428934 | -0.025046274 | -2.571474963 | 0.010445694 |
| RP11-767C1.2 | -0.250004327 | -0.263180984 | -2.571293776 | 0.01045108 |
| APEX2 | -0.120876958 | 5.181998728 | -2.570802206 | 0.010465706 |
| CLCC1 | -0.152359545 | 4.454875353 | -2.570600294 | 0.010471719 |
| EIF2B5 | -0.069791374 | 5.383765653 | -2.570592804 | 0.010471942 |
| ANXA3 | 0.594513919 | 4.508675995 | 2.570483099 | 0.010475211 |
| TATDN1 | -0.133345124 | 3.558358856 | -2.5704159 | 0.010477213 |
| IGF1 | 0.459551316 | -2.496957586 | 2.570339009 | 0.010479505 |
| POMT2 | -0.152804725 | 2.799791928 | -2.569882167 | 0.01049313 |
| TFPI | 0.526906981 | 3.41488394 | 2.569815927 | 0.010495107 |
| C4BPB | 0.384132619 | -2.353857328 | 2.569641981 | 0.0105003 |
| RPL27 | 0.103865976 | 10.29427655 | 2.569277975 | 0.010511174 |
| CUEDC1 | 0.355868866 | 2.571951315 | 2.568868955 | 0.010523405 |
| LRRC24 | -0.201628715 | 4.071466947 | -2.568469177 | 0.010535373 |
| RP11-127B20.3 | 0.221457986 | 2.250030246 | 2.567877492 | 0.010553106 |
| ABCC6 | -0.45471631 | 0.338066463 | -2.567859063 | 0.010553659 |
| NSUN2 | -0.084459014 | 6.305567852 | -2.567003956 | 0.010579337 |
| UPK3B | 0.30850397 | -0.687548969 | 2.566666868 | 0.010589474 |
| MT-CO3 | 0.076524104 | 13.32147013 | 2.566605046 | 0.010591334 |
| HSP90AB2P | 0.214190023 | 2.998426302 | 2.566585773 | 0.010591914 |
| ANKRD18A | 0.611012739 | 0.936283015 | 2.56646149 | 0.010595655 |
| RP11-138I1.4 | 0.377312598 | 1.914679125 | 2.566082051 | 0.010607082 |
| RP11-39K24.7 | 0.287968017 | 0.841794448 | 2.566029777 | 0.010608658 |
| TARBP2 | -0.112822118 | 4.424988927 | -2.56572337 | 0.010617895 |
| TAC3 | 0.36254116 | -1.617779923 | 2.564713645 | 0.010648386 |
| CEP170P1 | 0.219007894 | 0.829495176 | 2.564305522 | 0.010660732 |
| RP11-467J12.1 | -0.205674714 | 3.116322116 | -2.564284762 | 0.01066136 |
| C5AR1 | 0.618686921 | 6.312127025 | 2.563658435 | 0.010680334 |
| AL020996.1 | 0.22224918 | 0.265870323 | 2.563068555 | 0.01069823 |
| ZNF513 | 0.107896341 | 5.626671922 | 2.562680673 | 0.010710013 |
| TAS2R18 | 0.293347838 | 0.76894963 | 2.562665576 | 0.010710472 |
| RP11-255H23.4 | 0.337080478 | -1.702334895 | 2.56247303 | 0.010716325 |
| RPL32P3 | -0.149727154 | 4.462969282 | -2.562398008 | 0.010718607 |
| PRDM11 | -0.220862185 | 2.229400986 | -2.561970328 | 0.010731621 |
| SDR42E1 | 0.383640365 | -0.874810171 | 2.561826751 | 0.010735993 |
| SMAD6 | -0.397055913 | 0.396640312 | -2.561592168 | 0.01074314 |
| ACER2 | -0.199810037 | 1.372575957 | -2.560936569 | 0.010763137 |
| HERPUD1 | 0.222217977 | 6.382146847 | 2.560831341 | 0.01076635 |
| MRPL50 | -0.166864839 | 4.286086623 | -2.560758088 | 0.010768587 |
| BCDIN3D | -0.230895676 | 2.224675368 | -2.560633569 | 0.01077239 |
| PRRG1 | -0.640495094 | -2.321331454 | -2.560618462 | 0.010772852 |
| CALR | -0.179135402 | 10.65278885 | -2.560272706 | 0.010783419 |
| XPOTP1 | 0.123644656 | 4.718985872 | 2.559817539 | 0.010797345 |
| CTB-167B5.2 | -0.595070026 | 0.191929251 | -2.559686808 | 0.010801348 |
| ZSCAN1 | 0.402280796 | -2.777061357 | 2.559436062 | 0.010809029 |
| DPH7 | -0.095113533 | 4.619620781 | -2.559429007 | 0.010809245 |
| ICAM4 | -0.290031704 | 4.530446332 | -2.559056086 | 0.010820678 |
| RP11-498E2.7 | -0.15622933 | 3.037930604 | -2.558933531 | 0.010824438 |
| TOMM40L | 0.112429624 | 5.063029084 | 2.558879897 | 0.010826083 |
| TCTN2 | -0.204788766 | 2.723748882 | -2.558727076 | 0.010830774 |
| WDFY3 | -0.431973314 | 3.887409545 | -2.558423294 | 0.010840103 |
| RP11-120B7.1 | 0.158005473 | 2.522755222 | 2.558367123 | 0.010841828 |
| HSPA1L | -0.187287537 | 2.905816439 | -2.558208996 | 0.010846688 |
| TUBAL3 | 0.442927023 | -1.627570599 | 2.557794733 | 0.010859428 |
| CHCHD3P3 | -0.152127905 | 4.788438066 | -2.557716054 | 0.010861849 |
| ZNF185 | -0.441059394 | 5.779955505 | -2.557649527 | 0.010863897 |
| PPT2 | 0.272942483 | 3.568929874 | 2.557528001 | 0.010867638 |
| POLR1C | 0.154671828 | 4.484875501 | 2.557399052 | 0.010871609 |
| ZSWIM5 | 0.341127451 | -0.979118873 | 2.557326627 | 0.01087384 |
| RPL23A | 0.143910254 | 7.212820418 | 2.557076456 | 0.010881549 |
| RP11-4K3__A.5 | -0.129854635 | 4.526019883 | -2.557032337 | 0.010882909 |
| RYR2 | 0.589070802 | -5.231968204 | 2.556531153 | 0.01089837 |
| VLDLR | 0.528932401 | 1.403079597 | 2.556142152 | 0.010910384 |
| HOXB4 | -0.655044657 | 3.017866143 | -2.555947406 | 0.010916403 |
| RP11-779O18.2 | -0.255094413 | 0.241347131 | -2.555771603 | 0.010921839 |
| CSN1S1 | -0.575260396 | -0.598249801 | -2.555537939 | 0.010929068 |
| SCRN1 | 0.57045691 | 4.173770681 | 2.555529173 | 0.010929339 |
| NOL5BP | -0.339595258 | -0.016810397 | -2.555278499 | 0.010937099 |
| C16orf54 | -0.179583338 | 7.170013339 | -2.55504341 | 0.010944381 |
| RSL24D1P3 | 0.238043604 | 0.102392424 | 2.554836433 | 0.010950795 |
| RELN | 0.596626957 | -5.479536107 | 2.554585741 | 0.01095857 |
| RP11-71G12.1 | -0.335033735 | -1.690247188 | -2.554261507 | 0.010968631 |
| NNMT | 0.535557355 | -0.742513901 | 2.554240806 | 0.010969274 |
| TERF1P4 | 0.101015286 | 3.4064869 | 2.554115814 | 0.010973155 |
| CTGLF11P | -0.258860024 | 0.907168865 | -2.554087363 | 0.010974039 |
| ESRRB | 0.356341135 | -2.352807665 | 2.553780556 | 0.010983572 |
| ZNF337 | -0.136623464 | 5.415702102 | -2.553691308 | 0.010986346 |
| FTH1P15 | 0.304637904 | 0.529020881 | 2.553012561 | 0.011007467 |
| OCRL | -0.123571036 | 4.931723571 | -2.552837723 | 0.011012913 |
| RPS7P4 | 0.190436359 | 3.354092204 | 2.552750091 | 0.011015644 |
| RP6-206I17.3 | -0.369676979 | -0.570744131 | -2.552636446 | 0.011019186 |
| RP11-339B21.8 | 0.334044125 | -0.757142757 | 2.552406366 | 0.01102636 |
| SEH1L | 0.125295073 | 4.532640565 | 2.552040518 | 0.011037777 |
| IQCD | -0.335328699 | -0.530530889 | -2.551693132 | 0.011048627 |
| RGS6 | 0.470428611 | -2.633526955 | 2.551490342 | 0.011054965 |
| SLC4A1 | 0.860258645 | 2.66916172 | 2.551466256 | 0.011055718 |
| FUCA2 | -0.162300018 | 6.080964616 | -2.551118209 | 0.011066605 |
| LINC00240 | -0.329544013 | -3.040852116 | -2.550874587 | 0.011074231 |
| RP11-314A15.2 | 0.173940425 | 3.045696305 | 2.550847135 | 0.011075091 |
| GNAZ | 0.440051417 | -0.060448803 | 2.550695683 | 0.011079834 |
| AC007228.9 | -0.31873998 | 1.562400053 | -2.550579103 | 0.011083487 |
| SWT1 | -0.126837343 | 3.117626925 | -2.54938092 | 0.011121089 |
| RP5-828H9.1 | -0.429060464 | -2.197724478 | -2.54932679 | 0.01112279 |
| FAM106A | -0.455448272 | -0.906619374 | -2.549318116 | 0.011123063 |
| TARBP1 | -0.22857999 | 6.181010848 | -2.549172844 | 0.01112763 |
| EXOC3L4 | 0.482464741 | -1.757936546 | 2.548895829 | 0.011136344 |
| CTB-47B8.1 | 0.15870685 | 2.946570405 | 2.548289184 | 0.011155449 |
| CTC-444N24.11 | -0.158421794 | 4.584144542 | -2.548090438 | 0.011161715 |
| BBS1 | -0.246528517 | -0.477152879 | -2.547875632 | 0.01116849 |
| DAB2IP | 0.369257156 | -1.089152096 | 2.547757187 | 0.011172227 |
| PRRG4 | -0.276016645 | 4.178311802 | -2.547618701 | 0.011176598 |
| CADM1 | 0.572838164 | -1.095643669 | 2.547352495 | 0.011185005 |
| HEMGN | 0.62836317 | 4.386861323 | 2.547041192 | 0.011194843 |
| ZNF346-IT1 | 0.22675332 | 1.724562146 | 2.546975505 | 0.01119692 |
| RNLS | -0.235819168 | 1.130813666 | -2.546912641 | 0.011198908 |
| TCEB1P19 | 0.172484375 | 3.747663233 | 2.545418661 | 0.011246246 |
| ATF3 | 0.453750634 | 5.907292802 | 2.54530162 | 0.011249962 |
| SOX7 | 0.290137672 | -1.647901258 | 2.545300817 | 0.011249987 |
| RP11-983P16.4 | 0.262673221 | -0.269418719 | 2.544142177 | 0.011286833 |
| LAGE3 | -0.142918607 | 4.860663983 | -2.543962927 | 0.011292543 |
| CA3 | 0.365236715 | 0.292480972 | 2.543846662 | 0.011296248 |
| RP11-561I11.4 | -0.375260327 | -1.491591861 | -2.543695959 | 0.011301051 |
| EIF3IP1 | 0.19906038 | 0.992334886 | 2.543627399 | 0.011303238 |
| AC141586.5 | -0.198064995 | 2.070381499 | -2.543554595 | 0.011305559 |
| SENP3-EIF4A1 | 0.277935799 | -0.334176503 | 2.543403473 | 0.01131038 |
| CTA-211A9.5 | -0.132439924 | 3.05135128 | -2.543056936 | 0.011321442 |
| SLC2A13 | -0.3104192 | 1.572090824 | -2.542701571 | 0.011332795 |
| KBTBD12 | 0.571950073 | -3.397635439 | 2.542658398 | 0.011334175 |
| RASAL3 | -0.152638843 | 6.48174087 | -2.54246876 | 0.011340238 |
| GLTP | 0.140893863 | 6.448797388 | 2.541683479 | 0.011365377 |
| TRAV6 | -0.33356484 | -0.590586598 | -2.541173616 | 0.011381726 |
| CCNB1IP1 | 0.19537034 | 6.048341524 | 2.540874938 | 0.011391313 |
| MALSU1 | 0.103023427 | 3.670870572 | 2.540489657 | 0.01140369 |
| CTD-2035E11.3 | -0.259366395 | 1.706606228 | -2.540424375 | 0.011405789 |
| ARHGAP9 | -0.164741777 | 7.124444967 | -2.540030255 | 0.011418464 |
| SNRPA | 0.101289524 | 7.195107915 | 2.539892735 | 0.01142289 |
| CTD-2547L16.3 | -0.182238271 | 1.973659767 | -2.5392358 | 0.011444053 |
| RP11-111M22.3 | -0.167956053 | 3.701614545 | -2.538901388 | 0.01145484 |
| GMDS | -0.164437094 | 4.863994696 | -2.538856571 | 0.011456286 |
| EPS8 | 0.509273986 | 2.468672938 | 2.53865967 | 0.011462642 |
| H2AFY | -0.173949937 | 8.408383427 | -2.538618709 | 0.011463965 |
| PPP1R14A | 0.496063654 | 0.610229076 | 2.538560216 | 0.011465854 |
| AL356776.1 | 0.214657403 | 2.071000757 | 2.538174844 | 0.011478306 |
| ANKRD35 | -0.420020941 | 0.252744525 | -2.538107349 | 0.011480488 |
| FLJ00273 | 0.272673571 | 0.920474069 | 2.538007841 | 0.011483706 |
| VCP | -0.084508355 | 7.739779828 | -2.537685402 | 0.011494138 |
| SFTPD | -0.36207151 | -0.886321722 | -2.537617381 | 0.01149634 |
| RPL15P18 | 0.180962091 | 3.423605747 | 2.537343205 | 0.011505219 |
| CLK4 | 0.124309435 | 4.480039383 | 2.536922583 | 0.011518853 |
| EID1 | -0.143459802 | 6.374635859 | -2.536076351 | 0.011546325 |
| DDX18P5 | -0.160405 | 1.720291951 | -2.535790504 | 0.011555617 |
| AC068657.2 | -0.182124517 | 2.181070005 | -2.535314174 | 0.011571118 |
| RP1-90G24.10 | -0.454526636 | -1.038390424 | -2.535012827 | 0.011580933 |
| FHP1 | -0.183146365 | 2.304993061 | -2.534800223 | 0.011587863 |
| CYB561 | -0.228004975 | 4.063158944 | -2.534571434 | 0.011595324 |
| ERAP2 | -0.327244506 | 5.728786434 | -2.534312718 | 0.011603767 |
| RP11-148K1.12 | 0.225414909 | 0.560668047 | 2.533947358 | 0.011615698 |
| NOP2 | 0.132033141 | 5.648097433 | 2.53383639 | 0.011619324 |
| INO80C | 0.145490464 | 3.307185456 | 2.533197022 | 0.011640236 |
| KIAA1324L | 0.579995963 | 0.268895795 | 2.532812167 | 0.01165284 |
| BUB3 | 0.092077385 | 6.773475553 | 2.532741233 | 0.011655164 |
| EEF1GP2 | 0.286896175 | 0.136581914 | 2.532654126 | 0.011658019 |
| FGD1 | 0.327892147 | -3.248071715 | 2.53208906 | 0.011676554 |
| NKAPP1 | 0.178568188 | 1.119619975 | 2.531747679 | 0.011687765 |
| RP11-96D1.9 | 0.247561727 | 1.484514898 | 2.531372337 | 0.011700101 |
| BOLA2B | 0.295879813 | 1.010573931 | 2.530865191 | 0.011716788 |
| NOC2L | -0.104111559 | 6.013410054 | -2.530815211 | 0.011718434 |
| TUBA1A | -0.195457843 | 9.02396543 | -2.530523962 | 0.011728028 |
| RP11-632K20.8 | -0.34611952 | 0.613751317 | -2.530218683 | 0.011738092 |
| SLAIN2 | -0.090586623 | 5.766368679 | -2.530044847 | 0.011743826 |
| RP11-848P1.5 | 0.270060697 | 0.93586468 | 2.529916688 | 0.011748055 |
| PARD6A | -0.157229957 | 3.059739407 | -2.529236091 | 0.011770536 |
| RNU6-2 | 0.237078831 | 1.487052532 | 2.528752999 | 0.011786517 |
| RP11-641A6.9 | -0.218307118 | 1.242480224 | -2.527669945 | 0.011822414 |
| SYNPO | -0.328553806 | -0.075660954 | -2.526968524 | 0.011845713 |
| ZBTB8OSP2 | -0.200437411 | 1.967201766 | -2.526472826 | 0.011862204 |
| RP11-290F20.1 | 0.473619994 | -0.138244428 | 2.526426512 | 0.011863746 |
| OXCT1-AS1 | 0.295459251 | -0.906938698 | 2.526384996 | 0.011865128 |
| GS1-393G12.12 | -0.178843953 | 3.131896118 | -2.526048124 | 0.011876349 |
| ITPKC | -0.127513055 | 4.049645451 | -2.526013341 | 0.011877509 |
| MT-TY | 0.262432751 | 0.994012755 | 2.525788673 | 0.011884998 |
| MYCL | -0.637740089 | 0.82323133 | -2.525751179 | 0.011886248 |
| STK38L | -0.113538758 | 5.402017664 | -2.525376423 | 0.011898752 |
| RP3-462E2.5 | -0.172851119 | 3.837718758 | -2.524611726 | 0.011924302 |
| MGAT3 | 0.45890803 | 1.478533974 | 2.522817585 | 0.011984438 |
| PIGL | 0.143528476 | 3.335290675 | 2.521892055 | 0.012015566 |
| AC009950.2 | -0.309276637 | -2.15050505 | -2.52179573 | 0.01201881 |
| CYB5D1 | -0.129768208 | 4.079141571 | -2.521743237 | 0.012020578 |
| ATP5O | 0.10057896 | 5.983920224 | 2.52145336 | 0.012030345 |
| CYP2U1 | -0.313789399 | 1.480717922 | -2.521041929 | 0.012044221 |
| XKR8 | 0.197728977 | 4.831925948 | 2.52045788 | 0.012063942 |
| USMG5P1 | -0.162739348 | 4.642694494 | -2.52029333 | 0.012069504 |
| AC006033.22 | -0.313330735 | 1.004333613 | -2.52011616 | 0.012075495 |
| RP1-232L24.3 | -0.466001056 | -0.102342649 | -2.520027991 | 0.012078477 |
| SLC26A11 | 0.387128281 | 2.439274311 | 2.520013171 | 0.012078978 |
| CHRFAM7A | 0.337790047 | 1.62033025 | 2.519997158 | 0.01207952 |
| ILDR1 | 0.316910938 | -1.793059416 | 2.519931094 | 0.012081755 |
| KTN1-AS1 | -0.231870812 | 1.084628988 | -2.519235808 | 0.012105301 |
| METTL9 | -0.165319034 | 6.708742866 | -2.519069115 | 0.012110952 |
| CTBS | 0.150939189 | 5.590181129 | 2.519019503 | 0.012112635 |
| SKA2P1 | -0.23093756 | 3.630480732 | -2.518818246 | 0.012119462 |
| DCAF13P1 | -0.187877099 | 2.065559184 | -2.517412744 | 0.012167233 |
| TXNRD3 | -0.292884976 | 2.02840072 | -2.516939621 | 0.012183352 |
| MAATS1 | -0.398029743 | -3.824742959 | -2.51690959 | 0.012184376 |
| SMARCA4 | -0.112538566 | 6.312751695 | -2.516901559 | 0.01218465 |
| PAK1 | -0.258868185 | 6.105994442 | -2.516890409 | 0.01218503 |
| HLA-DRB9 | -0.445671356 | -0.46387454 | -2.515913559 | 0.012218374 |
| FBXO15 | -0.296579566 | -1.471077693 | -2.515015769 | 0.012249091 |
| CTA-384D8.35 | -0.362726402 | 0.80190806 | -2.514852527 | 0.012254684 |
| CTD-2012K14.7 | -0.261183757 | 0.705928483 | -2.514546053 | 0.012265189 |
| GHc-362H12.3 | -0.344200812 | -1.468448749 | -2.514179019 | 0.012277782 |
| SPATA21 | 0.330711892 | -2.06835169 | 2.513734271 | 0.012293055 |
| SERPINB1 | -0.165541544 | 9.69356078 | -2.513491927 | 0.012301385 |
| RP11-556E13.1 | -0.49111537 | 2.871512173 | -2.513471851 | 0.012302076 |
| TRAPPC5 | -0.233447783 | 0.526194067 | -2.51332958 | 0.012306968 |
| HSPA8P15 | -0.23968108 | 0.305937504 | -2.512643689 | 0.01233058 |
| COX20 | -0.123087233 | 5.431711176 | -2.512196644 | 0.012345992 |
| RP11-152C15.1 | 0.225553097 | 1.070607199 | 2.5117546 | 0.012361247 |
| LINC00862 | 0.317596191 | -1.51912173 | 2.511557748 | 0.012368047 |
| AC004854.4 | -0.265450646 | -0.209955527 | -2.511543922 | 0.012368524 |
| PI15 | -0.710182334 | -3.011418578 | -2.511229731 | 0.012379383 |
| PTX3 | 0.420404427 | 5.160825517 | 2.511079287 | 0.012384586 |
| ALDH1A3 | 0.488278183 | -2.92559197 | 2.511010782 | 0.012386956 |
| RP1-92O14.3 | -0.234890494 | 1.285221637 | -2.51060633 | 0.012400955 |
| RP11-112J1.1 | -0.159172197 | 6.762585407 | -2.510484325 | 0.01240518 |
| CD3G | -0.401567328 | 1.735815969 | -2.510174677 | 0.012415911 |
| MAK | 0.337301203 | 0.231238405 | 2.509756357 | 0.01243042 |
| OSBPL7 | -0.104075093 | 4.601159849 | -2.509723325 | 0.012431566 |
| RP11-73B2.6 | -0.401033538 | -0.343011487 | -2.509665693 | 0.012433567 |
| RP11-119F19.2 | -0.152874734 | 3.107686597 | -2.509476006 | 0.012440152 |
| SS18L2 | 0.114616008 | 5.18427296 | 2.50906233 | 0.012454525 |
| LINC01106 | -0.403985663 | -1.500589919 | -2.50863765 | 0.012469296 |
| GPR65 | 0.382596526 | 4.922781563 | 2.507981016 | 0.012492165 |
| EFTUD2 | -0.09939867 | 6.072972711 | -2.507603753 | 0.012505321 |
| ADPRHL2 | -0.09975004 | 5.744596368 | -2.507548086 | 0.012507263 |
| CROCCP2 | -0.123744044 | 5.285535233 | -2.50695449 | 0.012527991 |
| EIF3E | -0.152635292 | 8.719509346 | -2.50687092 | 0.012530911 |
| BMP8A | 0.325705139 | -1.486632744 | 2.506640767 | 0.012538958 |
| CTB-83J4.1 | -0.37836177 | -0.120901899 | -2.506515776 | 0.01254333 |
| TSPAN32 | -0.2581178 | 6.280698663 | -2.506198416 | 0.012554436 |
| TRMT6 | 0.146753209 | 5.131955525 | 2.506148733 | 0.012556175 |
| IGLV3-12 | -0.359329539 | -0.431514107 | -2.505362957 | 0.012583716 |
| DHDDS | -0.074887443 | 4.512041793 | -2.505264083 | 0.012587185 |
| CTD-2206G10.2 | 0.188256508 | 2.332660492 | 2.504432473 | 0.012616397 |
| FMNL3 | 0.236289136 | 4.03251577 | 2.503908793 | 0.012634823 |
| MMP8 | 0.77202065 | 6.085048338 | 2.503888914 | 0.012635523 |
| RCHY1 | 0.098217899 | 4.736300684 | 2.503811057 | 0.012638265 |
| GNL3 | -0.128232249 | 6.794923305 | -2.503636636 | 0.012644409 |
| FKBP4P1 | -0.321449213 | 0.887067296 | -2.503619114 | 0.012645026 |
| NT5C3AP1 | 0.150937629 | 3.571217083 | 2.502903592 | 0.01267026 |
| RP11-308D16.4 | -0.235916269 | 0.947543794 | -2.502299554 | 0.012691596 |
| GADD45G | 0.35345749 | 3.519723931 | 2.502054561 | 0.012700259 |
| BCAP29 | 0.112275928 | 4.80932803 | 2.501897522 | 0.012705815 |
| RP11-294L13.1 | -0.230438989 | -0.802294398 | -2.501826904 | 0.012708314 |
| RP11-452K12.7 | -0.342845483 | -0.339394713 | -2.501644713 | 0.012714763 |
| COX7CP1 | -0.247544558 | 4.213970987 | -2.501338747 | 0.012725601 |
| RP11-47K11.2 | 0.144863924 | 2.892050166 | 2.501108338 | 0.012733767 |
| AHSP | 0.799987313 | 3.60222573 | 2.500973366 | 0.012738553 |
| PDE8B | 0.404628914 | -0.882321874 | 2.500873996 | 0.012742078 |
| AKR1C2 | 0.381966556 | -1.180526141 | 2.500151908 | 0.012767716 |
| CD40LG | 0.38040834 | 1.879535752 | 2.500099848 | 0.012769567 |
| AC097533.1 | -0.20700121 | 2.264399285 | -2.50000058 | 0.012773095 |
| ATP11B | 0.129103089 | 6.50213375 | 2.499995816 | 0.012773265 |
| S1PR5 | 0.423700642 | 2.572138434 | 2.499845548 | 0.012778608 |
| RNU5A-1 | -0.381387888 | 1.883406873 | -2.499331526 | 0.0127969 |
| CAMK4 | 0.389692731 | 1.233492019 | 2.499152481 | 0.012803278 |
| RP11-273B20.1 | -0.303078536 | -1.23259556 | -2.498972341 | 0.012809697 |
| SEPT1 | 0.278643713 | 1.826398079 | 2.497735335 | 0.012853853 |
| GRAP | -0.208298382 | 4.387152791 | -2.497727614 | 0.012854129 |
| HSH2D | -0.315359791 | 7.95156377 | -2.497700371 | 0.012855103 |
| LCMT1 | -0.078701797 | 5.02591969 | -2.497413199 | 0.012865374 |
| LINC00941 | 0.311537648 | -2.646382826 | 2.497362465 | 0.012867189 |
| SH3RF2 | 0.345395531 | -3.933019578 | 2.496840407 | 0.012885882 |
| RAD51 | 0.303009341 | 3.727810718 | 2.496808708 | 0.012887018 |
| FREM1 | 0.588923613 | -3.523625713 | 2.496338814 | 0.012903865 |
| CTD-3193K9.4 | -0.335065018 | -0.560069335 | -2.495903587 | 0.012919487 |
| DTNA | -0.704482318 | -1.783607036 | -2.495869562 | 0.012920709 |
| WDR59 | -0.106497834 | 4.674696581 | -2.495322908 | 0.012940356 |
| CDH3 | 0.397945814 | -2.937579928 | 2.495287502 | 0.012941629 |
| USP32P3 | -0.228464419 | 2.668607783 | -2.494548336 | 0.012968239 |
| SLC17A9 | -0.266806341 | 6.420959449 | -2.494505856 | 0.01296977 |
| THEM4 | -0.228524756 | 4.146496225 | -2.49424306 | 0.012979243 |
| TIMP4 | 0.512219388 | -0.834250744 | 2.493830299 | 0.012994135 |
| RP11-16L9.3 | -0.406119003 | -1.472314252 | -2.49366994 | 0.012999924 |
| ABCD1 | -0.183066364 | 4.851597584 | -2.493648979 | 0.013000681 |
| GLI3 | 0.489009761 | -3.035499801 | 2.493343704 | 0.01301171 |
| ETHE1 | -0.133570729 | 5.982145071 | -2.493270199 | 0.013014366 |
| EVA1C | 0.333303586 | 0.687875255 | 2.493264069 | 0.013014588 |
| TP63 | -0.543757165 | -1.506078721 | -2.493064167 | 0.013021816 |
| PSMB7 | 0.087004323 | 7.528925127 | 2.492879827 | 0.013028484 |
| MX2 | -0.288768045 | 4.725429412 | -2.492471976 | 0.013043248 |
| NRN1 | -0.41732261 | -0.747567338 | -2.49232384 | 0.013048614 |
| KLHL32 | -0.287648226 | -2.553676725 | -2.492110064 | 0.013056361 |
| RP1-257A7.4 | -0.175999544 | 1.700875606 | -2.491910801 | 0.013063586 |
| SLC22A1 | 0.392035909 | -1.36720389 | 2.490852698 | 0.013102011 |
| SLC1A7 | -0.388999208 | -2.339500933 | -2.490481832 | 0.013115503 |
| AARSD1 | 0.184921244 | 1.391648393 | 2.489775236 | 0.013141243 |
| ERO1L | 0.163599863 | 6.04435502 | 2.48966089 | 0.013145412 |
| UFL1 | 0.075922405 | 5.69989647 | 2.489654326 | 0.013145651 |
| STX12 | -0.140114297 | 5.42775259 | -2.489353291 | 0.013156634 |
| SH3BP5-AS1 | 0.20629971 | 3.453935232 | 2.488595388 | 0.013184321 |
| AP000240.9 | -0.263617941 | 2.134501651 | -2.488503765 | 0.013187672 |
| BCL2L14 | 0.462099887 | -2.906412709 | 2.487988801 | 0.013206518 |
| FBXO24 | -0.227290702 | 0.119813095 | -2.487226437 | 0.013234461 |
| NSFL1C | -0.077068215 | 5.971274566 | -2.487161674 | 0.013236838 |
| ZNF319 | 0.121516511 | 5.035598371 | 2.486592703 | 0.01325773 |
| CNOT3 | -0.091098508 | 5.564378373 | -2.486478418 | 0.01326193 |
| AMOTL1 | 0.471091754 | 2.651070313 | 2.48629675 | 0.013268609 |
| CXorf40A | 0.128968725 | 3.328464133 | 2.486052378 | 0.013277598 |
| UBE2L4 | -0.148510822 | 2.267711579 | -2.485901755 | 0.013283141 |
| EFCAB10 | 0.310449495 | -1.814997146 | 2.485815644 | 0.013286311 |
| ZBTB16 | 0.364785188 | 3.617890954 | 2.48532826 | 0.013304265 |
| RP1-28O10.1 | 0.367496792 | 0.355949121 | 2.484884705 | 0.013320623 |
| AIG1 | 0.320030969 | 3.317056453 | 2.484555129 | 0.01333279 |
| LPP-AS2 | -0.199840772 | 2.017136538 | -2.484457645 | 0.01333639 |
| GLDN | 0.55487889 | -2.078524917 | 2.484296332 | 0.01334235 |
| RP1-146I3.1 | 0.201883404 | 1.104581749 | 2.484220725 | 0.013345144 |
| PARD6G | 0.440449842 | -0.807286931 | 2.484075452 | 0.013350515 |
| TTC7A | -0.149269067 | 6.553452259 | -2.483934567 | 0.013355725 |
| SLC36A1 | -0.234210046 | 4.738079963 | -2.483191769 | 0.013383223 |
| RP11-498C9.2 | 0.376469766 | 0.593929407 | 2.483058471 | 0.013388163 |
| WWP1P1 | 0.139594249 | 2.428070904 | 2.482598331 | 0.013405228 |
| RP11-486O13.3 | -0.28574227 | 1.474994425 | -2.482409199 | 0.013412248 |
| CTD-2540B15.11 | -0.355037577 | -0.927692932 | -2.482157061 | 0.013421612 |
| TSSC1 | -0.108618543 | 3.886810972 | -2.481763745 | 0.01343623 |
| NSRP1P1 | 0.197748107 | 1.06425753 | 2.481654459 | 0.013440294 |
| UROD | 0.160437231 | 6.604104623 | 2.480855444 | 0.013470042 |
| RP11-85A1.3 | -0.256357217 | 0.664930514 | -2.480599981 | 0.013479565 |
| VDAC1 | -0.129777686 | 6.779729809 | -2.480273172 | 0.013491757 |
| PDZD3 | 0.293137581 | -1.102576034 | 2.480270984 | 0.013491838 |
| HEYL | -0.364226537 | 1.065673726 | -2.480127287 | 0.013497202 |
| FAAH | -0.281393411 | 4.810716595 | -2.479333208 | 0.013526877 |
| NCBP2-AS2 | 0.104282101 | 6.557128419 | 2.479281949 | 0.013528794 |
| OXTR | -0.295563364 | -0.714866872 | -2.47866985 | 0.013551711 |
| PKMP1 | 0.20004488 | 5.024472847 | 2.478624726 | 0.013553401 |
| TCHH | -0.370527444 | -2.895211218 | -2.478464991 | 0.013559388 |
| YBX1P10 | 0.132647243 | 8.159378497 | 2.477974337 | 0.013577792 |
| MAP2K1P1 | 0.200546791 | 4.050054944 | 2.477827879 | 0.01358329 |
| SRGAP2C | 0.230420963 | 4.37599741 | 2.477272041 | 0.013604172 |
| STARD4-AS1 | -0.346821408 | -1.853587664 | -2.477231235 | 0.013605707 |
| DPY19L1 | -0.21320876 | 4.288718255 | -2.477110376 | 0.013610252 |
| HNRNPA1P51 | 0.254660058 | 0.072102544 | 2.476162696 | 0.013645936 |
| AC144530.1 | -0.175137061 | 8.344635731 | -2.475707421 | 0.013663108 |
| TACC3 | -0.11889349 | 6.7059892 | -2.475640177 | 0.013665646 |
| ADPGK | -0.082856694 | 6.984033539 | -2.475325298 | 0.013677536 |
| ESRRA | -0.125180516 | 5.541346253 | -2.475241144 | 0.013680716 |
| PCDHB10 | 0.398580965 | -2.764555034 | 2.474976102 | 0.013690733 |
| HSPD1P11 | 0.419367736 | -0.022600938 | 2.474333952 | 0.01371503 |
| RN7SL844P | 0.191430782 | 2.339709185 | 2.474257327 | 0.013717932 |
| ZNF391 | -0.428680571 | 0.753585103 | -2.474146155 | 0.013722143 |
| AC016732.2 | 0.120693479 | 2.465865909 | 2.473663389 | 0.013740443 |
| POC1A | 0.218124389 | 4.106456683 | 2.473583879 | 0.013743459 |
| GALR2 | 0.37605476 | -0.54512638 | 2.473289292 | 0.013754639 |
| FHDC1 | 0.415180336 | 1.189249316 | 2.47239538 | 0.013788613 |
| RP11-134O21.1 | 0.466146141 | 0.124438292 | 2.472394533 | 0.013788645 |
| GRK4 | -0.17221216 | 2.523019178 | -2.472379727 | 0.013789208 |
| EXT1 | 0.362112465 | 1.911264455 | 2.471593785 | 0.013819141 |
| RP5-884G6.2 | -0.212885281 | 1.416379727 | -2.471447618 | 0.013824714 |
| TNFRSF14 | 0.236904081 | 5.945596571 | 2.47139903 | 0.013826567 |
| AHI1 | 0.192593234 | 3.118846618 | 2.471158589 | 0.01383574 |
| TBCEL | -0.140066859 | 3.514535081 | -2.470854118 | 0.013847364 |
| GNAL | 0.279845136 | -2.241204274 | 2.470808623 | 0.013849101 |
| GAN | 0.119718409 | 2.834871594 | 2.470683339 | 0.013853887 |
| MT2A | 0.30839106 | 5.821714051 | 2.47014579 | 0.013874439 |
| TMEM132A | 0.365346725 | -4.156590877 | 2.469670124 | 0.013892647 |
| ZNF543 | 0.181303547 | 3.029184942 | 2.469549599 | 0.013897264 |
| TRPM6 | 0.570227601 | -0.891928451 | 2.469310209 | 0.013906438 |
| ZDHHC21 | -0.149503589 | 4.127852714 | -2.469295256 | 0.013907011 |
| SLITRK4 | -0.78474701 | 1.884708073 | -2.469076403 | 0.013915404 |
| ALKBH6 | 0.131732258 | 2.188665588 | 2.46895011 | 0.013920248 |
| JAM3 | 0.48001799 | 1.447173686 | 2.468759712 | 0.013927555 |
| JMJD8 | 0.086931237 | 6.25453751 | 2.468114691 | 0.013952335 |
| LINC00936 | 0.322505372 | 4.460913551 | 2.467540491 | 0.013974427 |
| PCDHGA11 | 0.410291311 | -0.607104287 | 2.46740422 | 0.013979674 |
| SEC31B | -0.160066019 | 5.115416049 | -2.467248317 | 0.01398568 |
| EVI5 | -0.250266999 | 2.980078658 | -2.467247306 | 0.013985719 |
| FBXL20 | 0.12165353 | 5.113557303 | 2.467026292 | 0.013994236 |
| LGSN | -0.591962699 | -2.920387884 | -2.466931634 | 0.013997886 |
| CTC-232P5.3 | -0.326133923 | -0.163840042 | -2.466669912 | 0.014007981 |
| ANKRD55 | -0.41671993 | 0.38294411 | -2.466152764 | 0.014027946 |
| MLK4 | 0.363681016 | 1.180237524 | 2.465961345 | 0.014035343 |
| ADA | -0.309952404 | 6.374266956 | -2.464931959 | 0.014075178 |
| CD36 | -0.537205152 | 5.530945197 | -2.464921633 | 0.014075578 |
| EXOSC10 | 0.069575474 | 6.242627305 | 2.464189174 | 0.014103984 |
| HIST1H3H | -0.330757885 | 3.315103605 | -2.463993086 | 0.014111598 |
| DYRK3 | 0.34082474 | 2.429988244 | 2.463786714 | 0.014119614 |
| FAM35DP | -0.241504525 | 0.205146665 | -2.463782144 | 0.014119792 |
| AC105020.1 | -0.360478619 | -0.875697203 | -2.46364539 | 0.014125106 |
| CPAMD8 | -0.500847251 | -1.223944808 | -2.463239306 | 0.014140898 |
| RP11-373D23.3 | -0.314592219 | -0.125043946 | -2.463063421 | 0.014147742 |
| TRAM2-AS1 | 0.153431757 | 3.76103349 | 2.463050921 | 0.014148229 |
| CTC-471J1.10 | -0.204079869 | 0.670073975 | -2.462820195 | 0.014157212 |
| BEGAIN | 0.450502038 | 0.516362223 | 2.46279943 | 0.014158021 |
| RHEBP1 | 0.186228983 | 1.851188191 | 2.462558833 | 0.014167395 |
| RP11-710C12.1 | -0.434937114 | -3.210923728 | -2.462505975 | 0.014169455 |
| RP11-311P8.2 | 0.181322296 | 0.262377525 | 2.462370357 | 0.014174742 |
| PGD | -0.164045866 | 9.418756662 | -2.46109015 | 0.014224734 |
| TFR2 | 0.422034081 | 3.712098337 | 2.460719453 | 0.014239239 |
| ZNF517 | -0.102675183 | 2.13749472 | -2.460607668 | 0.014243615 |
| ADAM22 | -0.346774634 | 0.432426473 | -2.46030558 | 0.014255448 |
| SCNN1D | 0.205502239 | 2.932945189 | 2.460230843 | 0.014258377 |
| EPX | 0.779789868 | 4.270781333 | 2.460075519 | 0.014264466 |
| RP11-925D8.6 | 0.399555273 | -1.133398633 | 2.45937745 | 0.014291859 |
| FBXW5 | -0.091381665 | 7.598885015 | -2.458702181 | 0.014318401 |
| TNRC18P3 | -0.261865353 | -1.109320933 | -2.458660809 | 0.014320029 |
| AC068580.5 | 0.369547399 | 2.532673693 | 2.458540234 | 0.014324773 |
| RPSAP4 | 0.172395123 | 4.670402726 | 2.458074949 | 0.014343095 |
| CLCN7 | -0.123574764 | 6.719131205 | -2.45798241 | 0.014346741 |
| AFAP1-AS1 | 0.536548677 | -2.683135828 | 2.457964469 | 0.014347448 |
| CXCL12 | 0.844210179 | -0.828603661 | 2.457714429 | 0.014357305 |
| RP11-435F17.1 | 0.197905267 | 1.03931186 | 2.456672317 | 0.014398451 |
| CTD-2047H16.2 | -0.300140904 | 0.820033033 | -2.456584974 | 0.014401904 |
| INTU | -0.593735403 | -0.602431816 | -2.456394698 | 0.01440943 |
| CHCHD6 | 0.176082173 | 2.727103322 | 2.456160281 | 0.014418706 |
| CDK5RAP2 | 0.103491567 | 5.352884054 | 2.455972853 | 0.014426127 |
| CTD-3099C6.9 | 0.196840761 | 3.2835972 | 2.455481383 | 0.014445601 |
| GOLGA6L4 | 0.292393209 | 0.117102395 | 2.455139738 | 0.014459152 |
| ZSWIM8-AS1 | 0.236876948 | -0.563934792 | 2.455041091 | 0.014463067 |
| GAS1 | 0.423760543 | -1.10633575 | 2.455017312 | 0.014464011 |
| TTC7B | 0.243978783 | 4.51245066 | 2.454843673 | 0.014470904 |
| ZNF233 | -0.331642918 | -1.090008823 | -2.454783973 | 0.014473275 |
| KIAA1715 | 0.174176476 | 4.903842295 | 2.454751409 | 0.014474568 |
| RP11-1055B8.8 | -0.421893586 | -0.547525572 | -2.454364815 | 0.01448993 |
| CTD-2015B23.2 | -0.215529806 | 3.112537737 | -2.454223931 | 0.014495532 |
| RP11-436I9.3 | -0.297232601 | -1.199010184 | -2.454020013 | 0.014503644 |
| CRB3 | 0.286068944 | -1.582100679 | 2.454000752 | 0.01450441 |
| PRDX5 | -0.14878744 | 9.019635341 | -2.453503841 | 0.014524195 |
| ITSN1 | -0.30342482 | 2.444353437 | -2.453324061 | 0.014531358 |
| RHAG | 0.721784058 | 2.546697132 | 2.452876544 | 0.014549204 |
| UMODL1 | -0.649949384 | 0.609167849 | -2.452757026 | 0.014553974 |
| RP6-99M1.2 | 0.251775393 | 5.311084593 | 2.452727724 | 0.014555143 |
| MRVI1 | -0.372112312 | 1.775754812 | -2.452297509 | 0.014572324 |
| IQCA1 | 0.345115722 | -3.977559133 | 2.452275048 | 0.014573221 |
| KIAA1024 | -0.303547305 | -0.037780022 | -2.452220485 | 0.014575401 |
| CPHL1P | -0.330110153 | -2.495677223 | -2.452025412 | 0.014583199 |
| SDHCP4 | -0.210504984 | 0.952008673 | -2.452005515 | 0.014583994 |
| RP6-186E3.1 | -0.266239012 | -0.116288974 | -2.451872146 | 0.014589328 |
| DUSP8P5 | 0.152745495 | 4.21920658 | 2.451134144 | 0.014618872 |
| CTD-2228K2.5 | -0.165391032 | 3.763530769 | -2.450636715 | 0.014638815 |
| BTN2A2 | -0.189554185 | 3.15115173 | -2.449749284 | 0.014674454 |
| SEPW1P | -0.174383233 | 3.078930873 | -2.449739095 | 0.014674864 |
| LA16c-358B7.3 | 0.353377762 | -2.414338288 | 2.449566789 | 0.014681793 |
| RNU6-611P | 0.243943173 | 0.899990777 | 2.449232424 | 0.014695246 |
| NDUFA12 | -0.098842583 | 6.390719106 | -2.448833166 | 0.014711326 |
| DRAP1 | 0.098910138 | 7.617038186 | 2.448678912 | 0.014717542 |
| ADARB1 | -0.191561941 | 3.903034599 | -2.448671688 | 0.014717833 |
| WDR4 | -0.144793933 | 4.033962936 | -2.448589892 | 0.014721131 |
| RP11-204C16.4 | 0.147519592 | 4.35477436 | 2.448338379 | 0.014731274 |
| SIGLEC11 | -0.474235362 | -1.09156349 | -2.447336006 | 0.014771759 |
| ZC3H3 | 0.139335873 | 5.828844299 | 2.446870145 | 0.014790609 |
| SGSH | 0.387116252 | 3.543147274 | 2.446802861 | 0.014793333 |
| DDX11L10 | -0.472291385 | 0.026890997 | -2.446197242 | 0.014817873 |
| MAML3 | -0.149052898 | 5.29635068 | -2.445960827 | 0.014827462 |
| CLIC4 | 0.215129408 | 6.597748545 | 2.445734495 | 0.014836648 |
| TRAPPC2P1 | 0.134995713 | 3.429612855 | 2.445264681 | 0.014855731 |
| ZNF43 | 0.218771191 | 3.755941333 | 2.444997887 | 0.014866578 |
| CNOT10 | -0.075231227 | 5.173459393 | -2.444914278 | 0.014869978 |
| SYS1 | 0.104290439 | 4.638295614 | 2.44432547 | 0.014893946 |
| HERC2P4 | -0.495856821 | -1.638742721 | -2.444278264 | 0.014895869 |
| HBG2 | 0.732076902 | 2.71462111 | 2.443686396 | 0.014919998 |
| ATP9B | -0.085608469 | 3.630891096 | -2.443561129 | 0.014925109 |
| GIGYF1 | 0.109403107 | 6.538940295 | 2.443306289 | 0.014935513 |
| MED1 | 0.085264038 | 6.29917479 | 2.442958949 | 0.014949702 |
| CTC-301O7.4 | -0.204871278 | 1.773478913 | -2.442646475 | 0.014962477 |
| DRC1 | 0.326599369 | -1.779381997 | 2.442495314 | 0.014968661 |
| OR2L13 | -0.43201447 | 0.15984079 | -2.442209937 | 0.014980341 |
| TPRKB | 0.09125794 | 5.920006122 | 2.442105725 | 0.014984608 |
| ORC4 | -0.084198725 | 3.843282765 | -2.44138345 | 0.015014214 |
| RP11-863K10.7 | -0.189272129 | 3.209928073 | -2.441093646 | 0.015026107 |
| MIR4712 | -0.266973045 | 4.205038109 | -2.440383774 | 0.015055275 |
| CCNL1 | 0.226363093 | 8.591418821 | 2.440240289 | 0.015061177 |
| MSX2P1 | -0.352995291 | 0.594344968 | -2.439980195 | 0.01507188 |
| NMNAT2 | -0.413344419 | -3.875123431 | -2.43989856 | 0.015075241 |
| SPIN4 | 0.211011055 | 4.739881054 | 2.439758338 | 0.015081015 |
| RP11-175P13.2 | -0.276664852 | -0.185930402 | -2.439727036 | 0.015082304 |
| IFITM1 | 0.368793714 | 6.257429677 | 2.439174063 | 0.015105096 |
| TIMM22 | -0.095679771 | 4.429504345 | -2.43912915 | 0.015106948 |
| ITGA2 | -0.524547539 | -0.697372112 | -2.438899387 | 0.015116428 |
| RP11-429E11.3 | -0.515910265 | -0.223655388 | -2.438723804 | 0.015123676 |
| RP11-571M6.8 | 0.201766598 | 3.165528117 | 2.438267714 | 0.015142518 |
| RP11-456J20.1 | 0.306359469 | -1.422750107 | 2.437863267 | 0.015159244 |
| EIF3LP1 | 0.164216358 | 3.984987091 | 2.437705226 | 0.015165784 |
| STAMBPL1 | -0.184457595 | 2.179996864 | -2.437619348 | 0.015169339 |
| NUDT1 | 0.146884243 | 5.704762581 | 2.437248721 | 0.01518469 |
| ERCC1 | -0.102834506 | 5.884676487 | -2.437198619 | 0.015186766 |
| SH3D21 | 0.296027383 | 3.201190804 | 2.437119316 | 0.015190053 |
| ASNA1 | -0.09259616 | 7.019822499 | -2.436794054 | 0.01520354 |
| RP11-817O13.6 | 0.166397143 | 2.827016694 | 2.436238646 | 0.015226594 |
| C11orf95 | -0.234842586 | 4.109477421 | -2.435728429 | 0.0152478 |
| KIR2DS4 | 0.380713578 | -1.86357095 | 2.435478081 | 0.015258215 |
| MRFAP1 | -0.104954027 | 8.092862185 | -2.435047155 | 0.015276157 |
| ZFPM1 | 0.259714511 | 3.868846837 | 2.434760644 | 0.015288096 |
| LA16c-425C2.1 | 0.276323374 | 0.107632313 | 2.43458599 | 0.015295378 |
| RP4-604A21.1 | 0.189289348 | 1.749775356 | 2.434493993 | 0.015299215 |
| STXBP2 | 0.165522023 | 6.520868063 | 2.434447953 | 0.015301135 |
| SRPR | 0.07926606 | 7.887316776 | 2.434423879 | 0.01530214 |
| TCEA3 | -0.363961096 | 0.451574246 | -2.433405996 | 0.015344657 |
| CETP | 0.513553681 | 1.018458496 | 2.433141489 | 0.015355723 |
| OSMR | 0.525456448 | -3.17247142 | 2.432903979 | 0.015365665 |
| RP11-841C19.3 | -0.154822265 | 3.622498166 | -2.431634372 | 0.015418907 |
| FAXDC2 | 0.375781002 | 2.00067108 | 2.431308878 | 0.015432584 |
| KRT8P50 | -0.257910105 | -1.113694389 | -2.430559188 | 0.015464124 |
| C16orf45 | 0.407952532 | 0.22090564 | 2.42950372 | 0.015508625 |
| NEURL2 | -0.198197979 | 3.019663577 | -2.428909545 | 0.015533726 |
| RP11-503P10.1 | -0.222647927 | 0.22191154 | -2.428895149 | 0.015534335 |
| KMT2D | 0.103384292 | 7.031304018 | 2.428763491 | 0.015539902 |
| AC010525.6 | 0.213932644 | 1.933441678 | 2.428217157 | 0.015563022 |
| SPATC1L | -0.401486876 | 3.843032532 | -2.428122996 | 0.01556701 |
| RP2 | 0.143280075 | 5.943668704 | 2.428105807 | 0.015567738 |
| RP11-807E13.3 | -0.257501425 | 0.224456194 | -2.428105431 | 0.015567754 |
| TLE4 | 0.189156595 | 5.585122763 | 2.428031 | 0.015570907 |
| OR2L1P | 0.40087073 | 0.68636863 | 2.427332453 | 0.015600527 |
| AP001171.1 | 0.595246488 | 1.945282756 | 2.426394478 | 0.015640376 |
| PSMF1 | -0.059913294 | 6.946628373 | -2.426235166 | 0.015647153 |
| TM2D1 | -0.088934854 | 4.448428713 | -2.425511771 | 0.015677959 |
| SCGB3A2 | -0.43129085 | -1.88622771 | -2.425510155 | 0.015678028 |
| GS1-257G1.1 | 0.216748972 | 4.540141859 | 2.425479541 | 0.015679333 |
| RCC1 | -0.159978059 | 6.210435376 | -2.425413874 | 0.015682133 |
| TNIP3 | 0.345521474 | -1.703176612 | 2.425230557 | 0.01568995 |
| RP11-12A2.1 | -0.385223763 | 3.068663623 | -2.425173314 | 0.015692391 |
| RP11-522I20.3 | -0.174434923 | 1.855493898 | -2.424907819 | 0.01570372 |
| KCNA3 | 0.366825475 | 3.962951482 | 2.424880356 | 0.015704892 |
| MYOF | -0.557831816 | 2.993551589 | -2.424858196 | 0.015705838 |
| SORBS3 | 0.280386019 | 3.975752227 | 2.424532245 | 0.015719759 |
| CNTNAP2 | -0.526060193 | -2.557638016 | -2.423540582 | 0.015762176 |
| LEPR | -0.285617108 | 2.848542538 | -2.422961346 | 0.015786999 |
| HM13-IT1 | 0.191244682 | 2.156410119 | 2.422641588 | 0.015800716 |
| MORC3 | 0.0784327 | 5.96576455 | 2.422494335 | 0.015807037 |
| SCYL2 | -0.073138002 | 6.032931574 | -2.421850668 | 0.015834692 |
| PEG3 | 0.575441963 | -3.847580021 | 2.421811266 | 0.015836387 |
| SPTA1 | 0.534748919 | 2.848030993 | 2.421330024 | 0.015857093 |
| PBX2P1 | 0.110967463 | 5.466641202 | 2.421329459 | 0.015857118 |
| MICAL3 | 0.19070114 | 2.912611695 | 2.421168389 | 0.015864053 |
| PSMD14 | 0.112820537 | 4.565048761 | 2.420138937 | 0.015908445 |
| WNT5B | -0.42243941 | -0.421414829 | -2.419885343 | 0.015919398 |
| CNIH3 | -0.242410328 | -0.295363959 | -2.419857217 | 0.015920613 |
| RP11-101E13.5 | 0.177800171 | 2.687486812 | 2.41982132 | 0.015922164 |
| KDM4C | -0.075217973 | 4.907067765 | -2.419763226 | 0.015924674 |
| PRG4 | 0.337535491 | -2.548172339 | 2.41966135 | 0.015929077 |
| PHKA2-AS1 | 0.288716253 | -0.046957633 | 2.419451362 | 0.015938156 |
| AP2A2 | -0.118145456 | 5.214463758 | -2.419277673 | 0.015945669 |
| ITGB1 | -0.132327507 | 7.555304816 | -2.41912107 | 0.015952445 |
| MT1F | -0.365525717 | 2.063114684 | -2.419060946 | 0.015955048 |
| ST3GAL4 | -0.228880703 | 5.240129329 | -2.418884779 | 0.015962675 |
| ENOPH1 | -0.081101859 | 6.250560391 | -2.418760132 | 0.015968074 |
| KCTD7 | 0.290908102 | 2.138001881 | 2.418428616 | 0.01598244 |
| TMEM204 | -0.380073173 | 1.621081083 | -2.417601053 | 0.016018352 |
| HILPDA | 0.260598128 | 3.715841935 | 2.417383904 | 0.016027787 |
| MMP27 | 0.352521595 | -2.158738006 | 2.417263322 | 0.016033029 |
| KCTD12 | -0.510419273 | 5.385426248 | -2.417238926 | 0.016034089 |
| URM1 | 0.070389306 | 5.950573323 | 2.417036186 | 0.016042906 |
| B4GALT2 | 0.237206712 | 3.721223742 | 2.416727543 | 0.016056335 |
| N4BP2 | 0.140191393 | 5.302338565 | 2.416668532 | 0.016058904 |
| INF2 | -0.162021974 | 5.961291619 | -2.416553704 | 0.016063904 |
| LZTFL1 | -0.141365326 | 3.683831069 | -2.416371643 | 0.016071834 |
| GS1-251I9.4 | -0.106737018 | 4.50670228 | -2.416151548 | 0.016081425 |
| RP11-115L11.1 | 0.250637204 | 0.820999682 | 2.416067025 | 0.01608511 |
| TMPO-AS1 | 0.190457646 | 3.251533597 | 2.415854839 | 0.016094363 |
| PRPF18 | 0.079400103 | 4.98853753 | 2.414614067 | 0.016148566 |
| RTN2 | -0.223122122 | 2.869869627 | -2.414370713 | 0.016159215 |
| MITF | -0.323924966 | 2.798505441 | -2.414221492 | 0.016165749 |
| HSP90B3P | 0.192352967 | 2.139855163 | 2.41386087 | 0.016181547 |
| RTKN2 | 0.287847856 | 0.403847722 | 2.413831038 | 0.016182855 |
| PPP2R2D | -0.082923915 | 4.830432409 | -2.41308517 | 0.016215576 |
| CD101 | -0.458012337 | 3.011144221 | -2.413075975 | 0.01621598 |
| RP11-613F22.5 | 0.147105301 | 2.417696216 | 2.412889884 | 0.016224153 |
| MTERF | 0.135830768 | 3.497687524 | 2.412329563 | 0.016248785 |
| RP11-196G11.4 | 0.17802368 | 5.083346916 | 2.411674937 | 0.016277603 |
| TMEM91 | -0.164221664 | 4.512694015 | -2.411269656 | 0.016295468 |
| SMCHD1 | 0.125436266 | 6.904189502 | 2.411083349 | 0.016303686 |
| MMP25 | 0.558446424 | 4.619425623 | 2.410785689 | 0.016316823 |
| ARHGEF16 | 0.333252408 | -3.154721694 | 2.410548513 | 0.016327297 |
| NFE2L2 | 0.140991788 | 5.445534322 | 2.409965075 | 0.016353089 |
| EFCAB2 | -0.191185594 | 3.206461835 | -2.409915364 | 0.016355289 |
| LMBR1 | 0.111668059 | 4.405472996 | 2.409857293 | 0.016357858 |
| KCNA2 | -0.293396977 | -3.087365652 | -2.409754676 | 0.016362399 |
| PNPLA7 | -0.225105754 | 2.725735995 | -2.409382104 | 0.016378897 |
| RP11-79H23.3 | 0.345101776 | -2.394722187 | 2.409241054 | 0.016385146 |
| SPATA33 | -0.149244412 | 1.901834274 | -2.409049518 | 0.016393636 |
| RP11-3D4.2 | -0.267945913 | 1.966403314 | -2.408670259 | 0.016410457 |
| CCDC88A | -0.138202147 | 6.367319233 | -2.40851055 | 0.016417546 |
| SF3B14 | 0.073428446 | 7.919771465 | 2.408038809 | 0.016438499 |
| LAMA5 | 0.368650726 | 1.22568701 | 2.407845676 | 0.016447084 |
| GUSBP2 | -0.187047336 | 2.50282493 | -2.407780597 | 0.016449978 |
| RP1-68D18.4 | 0.367626898 | 0.315597483 | 2.407756506 | 0.016451049 |
| PLEKHA3P1 | 0.147172953 | 3.377186334 | 2.407026437 | 0.016483544 |
| FGD6 | 0.325355167 | 1.636804714 | 2.406969756 | 0.016486069 |
| NAPA-AS1 | -0.185667754 | 2.301380139 | -2.40673317 | 0.016496613 |
| RP5-1073O3.7 | 0.221165007 | 0.212229469 | 2.406254794 | 0.016517951 |
| RP11-543B16.1 | 0.197105497 | 3.280706298 | 2.406143544 | 0.016522917 |
| LRRN1 | -0.477964933 | 0.956505271 | -2.406113401 | 0.016524263 |
| MEGF6 | -0.245287776 | 5.554865478 | -2.405532328 | 0.016550223 |
| RP11-378A13.2 | -0.235903748 | 1.06883789 | -2.405307773 | 0.016560265 |
| KCNH4 | 0.345028741 | -0.302276546 | 2.405296631 | 0.016560763 |
| CDC42 | 0.073306198 | 9.163892105 | 2.405289096 | 0.0165611 |
| AC092535.3 | -0.380238587 | -2.508866633 | -2.404185265 | 0.016610543 |
| RP11-395P17.11 | 0.173831719 | 2.627440946 | 2.40401695 | 0.016618094 |
| CERKL | -0.323282233 | -1.963985522 | -2.403233402 | 0.016653283 |
| GATA1 | 0.558954678 | 3.49577055 | 2.402981106 | 0.016664628 |
| PTPRB | 0.56883338 | -3.25543147 | 2.402723423 | 0.016676222 |
| LCORL | 0.108065331 | 3.292403126 | 2.402284074 | 0.016696006 |
| ZDHHC11B | 0.500557244 | 0.380358707 | 2.402086592 | 0.016704905 |
| RP11-809C9.2 | 0.3742121 | -0.298364349 | 2.40122545 | 0.016743761 |
| POLR2J2 | -0.247301388 | -1.648063255 | -2.401137438 | 0.016747737 |
| SLC5A5 | 0.379534425 | -0.521400327 | 2.400878494 | 0.016759439 |
| NSG1 | -0.361539336 | -0.656087008 | -2.400850345 | 0.016760711 |
| SNRPD3 | -0.099153317 | 7.246562045 | -2.400846408 | 0.016760889 |
| COMMD6 | -0.123640537 | 6.003677803 | -2.400510536 | 0.01677608 |
| IGHG1 | 0.68264944 | 6.324287675 | 2.400239779 | 0.016788334 |
| TAFAZZIN | 0.104771817 | 5.531364899 | 2.400162395 | 0.016791838 |
| AC004017.1 | -0.35356223 | -0.129605094 | -2.39981926 | 0.016807382 |
| CTD-3193K9.3 | 0.272565599 | -0.30564138 | 2.399473737 | 0.016823047 |
| ODF3L1 | 0.421135501 | -0.441899568 | 2.399037371 | 0.016842849 |
| RPL7P49 | 0.293697955 | 0.100642935 | 2.399026106 | 0.01684336 |
| RP11-815N9.2 | -0.140575146 | 3.808938418 | -2.398473794 | 0.016868454 |
| DAG1 | 0.188091822 | 3.747123191 | 2.39794494 | 0.016892513 |
| RP11-355K23B.1 | -0.19862798 | 0.423317919 | -2.397925904 | 0.016893379 |
| RP11-58E21.3 | -0.25633792 | 0.1319978 | -2.397915766 | 0.016893841 |
| SLC44A3 | -0.417415375 | -1.793044347 | -2.397528504 | 0.016911479 |
| CYYR1 | 0.729943833 | -0.561655884 | 2.397096076 | 0.016931193 |
| TIMM10 | -0.134708427 | 5.776153534 | -2.397086175 | 0.016931644 |
| MYLIP | 0.237208407 | 7.019589458 | 2.396884642 | 0.016940839 |
| RPL5P18 | 0.126914438 | 3.835420343 | 2.396673432 | 0.01695048 |
| RP11-290F5.1 | 0.287059795 | 0.079248227 | 2.396046472 | 0.016979127 |
| DDX60L | -0.20064607 | 5.153264098 | -2.395917474 | 0.016985027 |
| GPRIN1 | 0.323104036 | -0.26981398 | 2.395775073 | 0.016991541 |
| ANAPC2 | 0.081125475 | 5.570838269 | 2.395487386 | 0.017004709 |
| CTD-2033D15.1 | 0.379032816 | -0.736277186 | 2.395474121 | 0.017005317 |
| COLQ | -0.200825584 | 2.130609845 | -2.395463689 | 0.017005794 |
| SMARCC1 | -0.1169787 | 7.206846292 | -2.39535388 | 0.017010823 |
| TUBB | 0.170912369 | 9.956008508 | 2.395297984 | 0.017013383 |
| TMEM87A | 0.10815326 | 5.675506173 | 2.395066576 | 0.017023986 |
| PTENP1 | -0.107499563 | 5.434484096 | -2.395060823 | 0.01702425 |
| HSF2BP | 0.287440354 | -0.125495262 | 2.395039643 | 0.017025221 |
| HNRNPCP4 | 0.092571859 | 4.802484687 | 2.395011348 | 0.017026518 |
| SNORD12C | -0.260226189 | 1.20139906 | -2.394758154 | 0.017038127 |
| STAP2 | 0.340071101 | -0.52346498 | 2.394250728 | 0.017061415 |
| AKR1C1 | 0.365848243 | 0.123052931 | 2.394160765 | 0.017065547 |
| PAK1IP1 | -0.124776073 | 5.36537625 | -2.393635727 | 0.017089677 |
| LINC01002 | 0.30405803 | 1.707721864 | 2.393128422 | 0.017113021 |
| RBBP4P5 | -0.238794613 | -0.010710452 | -2.392717806 | 0.017131937 |
| USP11 | -0.177021103 | 5.626031446 | -2.392497455 | 0.017142095 |
| ITGA11 | -0.454642834 | -2.210064159 | -2.392477605 | 0.01714301 |
| AP000347.4 | -0.227849516 | 1.888050247 | -2.392364657 | 0.01714822 |
| SDHB | 0.085486671 | 6.580251641 | 2.392332914 | 0.017149684 |
| CTA-29F11.1 | 0.201648344 | 5.53215374 | 2.391954148 | 0.017167164 |
| ATAD3A | 0.12454134 | 5.320487406 | 2.391656099 | 0.01718093 |
| RP11-618I10.1 | -0.531244508 | -1.266153013 | -2.391535728 | 0.017186493 |
| PIM1 | 0.27387291 | 8.191126276 | 2.390735072 | 0.017223532 |
| AADAT | 0.571158378 | -0.010447098 | 2.390693776 | 0.017225445 |
| ATXN7L3B | -0.133300882 | 7.450344654 | -2.390509891 | 0.017233962 |
| TCEB2 | 0.073532955 | 7.461614489 | 2.390445717 | 0.017236936 |
| CTD-2319I12.2 | -0.244530094 | -0.053098159 | -2.38994042 | 0.017260363 |
| AP000350.6 | 0.243876996 | -0.880407282 | 2.389882015 | 0.017263073 |
| LDHAP3 | 0.198713948 | 1.286237755 | 2.389771591 | 0.017268197 |
| RP11-496I9.1 | 0.310334166 | 0.696474187 | 2.389591374 | 0.017276563 |
| TNFAIP6 | 0.482953666 | 2.377723916 | 2.389136244 | 0.017297706 |
| RNF180 | 0.358586378 | -3.802608097 | 2.389016033 | 0.017303295 |
| SLAIN1 | 0.410417909 | 2.568588725 | 2.388626331 | 0.017321422 |
| MSMP | 0.319748744 | -0.470219921 | 2.388581025 | 0.01732353 |
| CLTC | -0.095836869 | 7.057027586 | -2.388365551 | 0.017333561 |
| RP11-302F12.1 | 0.233601083 | 1.498928944 | 2.388213869 | 0.017340626 |
| VAV3 | 0.146617977 | 5.591610943 | 2.388011191 | 0.017350069 |
| GPR124 | -0.493510526 | 3.734062708 | -2.387989429 | 0.017351083 |
| DDO | -0.483093681 | 2.464874284 | -2.387229105 | 0.017386551 |
| ALDH8A1 | -0.284796671 | 0.208562731 | -2.386890651 | 0.01740236 |
| MLNR | -0.376808478 | 0.92271908 | -2.386714233 | 0.017410606 |
| RP11-23F23.3 | 0.278216302 | 0.097244093 | 2.386643773 | 0.0174139 |
| EXOC3 | -0.065550417 | 5.576350191 | -2.386524885 | 0.017419459 |
| APOL6 | 0.158835093 | 5.410549721 | 2.386503051 | 0.01742048 |
| CTB-131B5.5 | 0.367698726 | -1.350279451 | 2.386470883 | 0.017421985 |
| HIST1H2BG | -0.452665999 | 4.741656092 | -2.386294653 | 0.01743023 |
| EPHA1-AS1 | -0.335842521 | 1.652039365 | -2.38600645 | 0.01744372 |
| CTC-260E6.6 | -0.284629523 | 0.108829788 | -2.385794919 | 0.017453628 |
| RP11-65L3.2 | -0.324363954 | 2.841882457 | -2.385195137 | 0.017481747 |
| MTSS1 | 0.385053686 | 4.481993492 | 2.384119378 | 0.017532281 |
| RAB11FIP3 | 0.146848713 | 4.588562245 | 2.383604743 | 0.017556501 |
| FAM157A | 0.294954879 | 2.59081845 | 2.383250428 | 0.017573194 |
| CYP4F11 | -0.455450652 | -2.842372269 | -2.383141499 | 0.017578328 |
| LRRC37A6P | -0.234910541 | -1.52539454 | -2.38312754 | 0.017578986 |
| PLAGL1 | -0.183181131 | 5.570478884 | -2.382834334 | 0.017592814 |
| RDH11 | -0.109960699 | 5.175055256 | -2.382658663 | 0.017601104 |
| RP11-897M7.1 | -0.368519083 | -0.902794014 | -2.382652229 | 0.017601407 |
| ALDH1A1 | 0.60649211 | 3.246903737 | 2.382625028 | 0.017602691 |
| RP11-67L2.2 | 0.202886951 | 3.633897674 | 2.382250428 | 0.01762038 |
| CTD-2555O16.2 | -0.202076234 | 1.955270965 | -2.38215305 | 0.017624981 |
| TRIB2 | 0.348213686 | 3.754038167 | 2.382086053 | 0.017628147 |
| STRCP1 | 0.32305589 | -2.676677802 | 2.381938211 | 0.017635136 |
| RENBP | -0.177641965 | 5.44154506 | -2.381798936 | 0.017641721 |
| FSTL1 | 0.508388332 | 0.422123284 | 2.381770784 | 0.017643053 |
| TBC1D29 | -0.432244455 | -0.649868247 | -2.381680134 | 0.017647341 |
| FAM72D | 0.328629801 | 1.20756439 | 2.381532605 | 0.017654321 |
| S100A2 | 0.34244901 | -0.334090477 | 2.381479574 | 0.017656831 |
| RP11-121A14.3 | -0.277724469 | -0.183932603 | -2.381213757 | 0.017669415 |
| EPS8L2 | 0.20992632 | 1.67213616 | 2.381137369 | 0.017673033 |
| ST8SIA6 | -0.484803564 | 4.108200456 | -2.381025291 | 0.017678343 |
| RP11-266K4.9 | -0.374937212 | 1.238650978 | -2.38098842 | 0.01768009 |
| PDGFB | 0.397514059 | -0.942048933 | 2.380558456 | 0.017700473 |
| RP11-355O1.11 | 0.216108947 | 3.032392258 | 2.380042048 | 0.017724982 |
| RP4-622L5.7 | -0.178312976 | 2.53638555 | -2.379835611 | 0.017734788 |
| C2orf50 | -0.255630372 | -2.180505833 | -2.379649378 | 0.017743638 |
| POC1B | -0.096677556 | 4.026476188 | -2.379595363 | 0.017746206 |
| CCNYL1 | 0.129078004 | 4.927938029 | 2.379143375 | 0.017767704 |
| AC099535.4 | 0.189094089 | 2.156016465 | 2.379042929 | 0.017772485 |
| PGPEP1 | 0.117323406 | 5.537879872 | 2.37902229 | 0.017773467 |
| CTH | 0.287317688 | 1.75687924 | 2.378980048 | 0.017775478 |
| TOR3A | 0.095110621 | 5.793329785 | 2.378937933 | 0.017777483 |
| CCPG1 | -0.204967889 | 3.498622635 | -2.378696684 | 0.017788973 |
| BCAM | 0.419008831 | 1.069562677 | 2.378147366 | 0.017815159 |
| RNA5SP383 | 0.183932735 | 2.483638783 | 2.377959039 | 0.017824145 |
| RPL37P2 | -0.105157062 | 7.21123421 | -2.377851618 | 0.017829272 |
| SLC4A3 | -0.489614952 | -0.383313573 | -2.37762475 | 0.017840104 |
| UNGP3 | -0.404915086 | -0.350448098 | -2.377077563 | 0.017866255 |
| SLC44A4 | 0.450809415 | -1.537302693 | 2.376901101 | 0.017874695 |
| GPRASP2 | -0.248139446 | 0.767802667 | -2.376671339 | 0.01788569 |
| GBF1 | -0.06699846 | 6.014608884 | -2.376282153 | 0.017904328 |
| SLC37A3 | 0.312509527 | 1.741431272 | 2.376170213 | 0.017909692 |
| EGR2 | 0.455668369 | 2.641008615 | 2.376143436 | 0.017910975 |
| RP11-452K12.6 | -0.315384442 | 0.005597563 | -2.376032022 | 0.017916316 |
| CYC1 | 0.105310992 | 7.505922459 | 2.375298551 | 0.017951508 |
| RP11-111E2.1 | -0.272029549 | -0.267525206 | -2.375163524 | 0.017957993 |
| RPL5P11 | 0.176440436 | 1.920227957 | 2.374516708 | 0.017989088 |
| RP11-561P12.5 | -0.4716301 | -0.542832419 | -2.374456715 | 0.017991975 |
| ZBED1 | -0.128209044 | 5.97024643 | -2.374170322 | 0.01800576 |
| TNFSF15 | -0.436751957 | -1.844799089 | -2.374076277 | 0.018010289 |
| UFC1 | -0.099465489 | 7.983356148 | -2.373547943 | 0.018035749 |
| NDUFAF2 | -0.140082029 | 4.773230649 | -2.37299488 | 0.018062435 |
| RP1-90J20.12 | -0.243184269 | 0.714837224 | -2.372633897 | 0.018079872 |
| YBX1P1 | 0.108730088 | 9.640075673 | 2.372231354 | 0.018099334 |
| CNN2P9 | 0.183753305 | 2.8826666 | 2.371882593 | 0.01811621 |
| RP11-325F22.2 | -0.308657768 | 5.167343511 | -2.37179389 | 0.018120504 |
| LINC01160 | 0.245952768 | 0.960132927 | 2.371708001 | 0.018124663 |
| Z83844.1 | 0.298431377 | -1.333033556 | 2.371706297 | 0.018124746 |
| PLEKHA5 | 0.259246426 | 3.178253268 | 2.371685392 | 0.018125758 |
| VSIG1 | -0.371735088 | -0.586968917 | -2.371441598 | 0.018137569 |
| ZNF300 | 0.56316413 | 0.005098603 | 2.371340071 | 0.01814249 |
| RHPN1-AS1 | -0.347491324 | 1.533218363 | -2.371115382 | 0.018153383 |
| DCK | -0.120774911 | 6.458013026 | -2.370450251 | 0.018185665 |
| ZNF578 | 0.384179047 | -1.44966994 | 2.370308688 | 0.018192542 |
| CITF22-92A6.1 | 0.219935144 | 2.869348857 | 2.369198147 | 0.018246572 |
| EEF1A1P25 | 0.175726433 | 4.270898035 | 2.369073244 | 0.018252657 |
| SAMD13 | -0.280039838 | 0.694202131 | -2.369054316 | 0.01825358 |
| ATP8A2 | -0.422436627 | -2.195656543 | -2.368896337 | 0.01826128 |
| FAM174A | -0.128996736 | 3.999928372 | -2.368660655 | 0.018272772 |
| TUSC1 | 0.602437895 | 0.94404453 | 2.368363306 | 0.018287281 |
| LEPREL4 | -0.282066428 | 1.495390154 | -2.368320362 | 0.018289377 |
| RP4-803J11.2 | -0.259464369 | 0.379248272 | -2.368178226 | 0.018296317 |
| RAP1A | -0.112266779 | 6.298035793 | -2.368116435 | 0.018299334 |
| HOXA11-AS | -0.710547037 | -0.852195346 | -2.368115952 | 0.018299358 |
| EDIL3 | 0.525084331 | -2.881375123 | 2.367818309 | 0.018313899 |
| VPS54 | -0.091085781 | 5.328918626 | -2.367529318 | 0.018328028 |
| ARHGAP42 | 0.397056321 | -1.499317554 | 2.367314813 | 0.018338521 |
| SNORD103A | 0.213666079 | 1.330831456 | 2.366730183 | 0.018367147 |
| AC013470.6 | -0.088474926 | 4.291124626 | -2.366591866 | 0.018373925 |
| BCL11B | 0.373354217 | 2.89099998 | 2.365312654 | 0.018436718 |
| POMT1 | -0.123075283 | 4.436561794 | -2.365270241 | 0.018438803 |
| STEAP3 | -0.326245589 | 4.260219362 | -2.36512055 | 0.018446164 |
| DACT1 | 0.292588653 | 1.586431919 | 2.36511506 | 0.018446434 |
| UCHL5 | -0.090233495 | 4.854110908 | -2.365081383 | 0.018448091 |
| RP11-397A16.2 | 0.350199893 | -3.00243525 | 2.364854534 | 0.018459252 |
| EEF1GP1 | 0.268273414 | 2.89221772 | 2.364653049 | 0.01846917 |
| AC009065.1 | 0.306998618 | -0.9180368 | 2.364528335 | 0.018475311 |
| SNRPD2 | 0.112402704 | 8.093950179 | 2.364105051 | 0.018496169 |
| ZNF180 | 0.113979272 | 3.359475879 | 2.364010772 | 0.018500817 |
| RPL7AP30 | -0.12573666 | 9.093705997 | -2.36391296 | 0.018505641 |
| MBLAC1 | 0.135953555 | 1.558953349 | 2.363653523 | 0.018518441 |
| MRVI1-AS1 | -0.32916529 | -0.892782926 | -2.36307296 | 0.018547112 |
| DGCR11 | 0.161963629 | 2.460592879 | 2.36287421 | 0.018556936 |
| LINC00937 | 0.471029966 | -0.121271457 | 2.362763749 | 0.018562398 |
| GNA15 | 0.26255884 | 8.191046316 | 2.362616575 | 0.018569678 |
| PFAS | -0.165268824 | 4.900826232 | -2.362389196 | 0.01858093 |
| FHIT | 0.206817736 | 2.315111868 | 2.361816928 | 0.018609275 |
| FN1 | 0.744736649 | 0.356888435 | 2.361126981 | 0.018643499 |
| RP11-245J9.4 | -0.245940152 | 0.800898831 | -2.361049165 | 0.018647363 |
| CHCHD7 | -0.166128113 | 4.845340557 | -2.36065435 | 0.018666976 |
| KCNK6 | -0.283129877 | 3.825794301 | -2.360231354 | 0.018688009 |
| RAB3B | 0.393482776 | -4.671576436 | 2.360100818 | 0.018694504 |
| CEP97 | 0.107535798 | 3.351355066 | 2.360078553 | 0.018695612 |
| MIR25 | 0.231798398 | 1.710651795 | 2.359990832 | 0.018699978 |
| BAALC | 0.671999996 | 4.197807737 | 2.359698961 | 0.018714511 |
| SLC25A30-AS1 | -0.250812045 | -0.354895244 | -2.359234565 | 0.018737655 |
| ZDHHC20-IT1 | 0.297242148 | 1.076281522 | 2.358564444 | 0.018771096 |
| ALAS2 | 0.765276003 | 3.368342994 | 2.358430088 | 0.018777808 |
| RP11-404O13.4 | -0.448839704 | -0.942091143 | -2.358029758 | 0.018797817 |
| TMEM175 | -0.10553332 | 5.195745246 | -2.35770416 | 0.018814104 |
| GTDC1 | 0.12949901 | 3.695280995 | 2.357418982 | 0.01882838 |
| LFNG | -0.30150738 | 4.887662502 | -2.356765334 | 0.018861137 |
| MTUS2 | -0.525207637 | -2.103915273 | -2.356534312 | 0.018872727 |
| LINC01107 | -0.392704134 | -2.162669959 | -2.356163742 | 0.01889133 |
| RP11-658F2.8 | 0.165530939 | 4.771544834 | 2.355768901 | 0.018911169 |
| RP11-861E21.2 | -0.411004036 | -0.94195721 | -2.355275435 | 0.01893599 |
| FAM156B | -0.169730196 | 1.410047581 | -2.35499872 | 0.01894992 |
| ERCC6L2-AS1 | -0.136902854 | 1.997126587 | -2.354990651 | 0.018950327 |
| ARHGAP22 | -0.400750692 | 2.773829843 | -2.354629837 | 0.018968505 |
| RP11-91P24.7 | -0.24496487 | 0.376989162 | -2.354409355 | 0.018979621 |
| IMPDH1P11 | -0.214051781 | 1.340841006 | -2.354318682 | 0.018984194 |
| AMN1 | -0.130744204 | 3.003815964 | -2.353965475 | 0.019002017 |
| RP11-365D23.4 | 0.135260272 | 5.013889977 | 2.353620394 | 0.019019444 |
| C15orf26 | 0.413226748 | -0.066984232 | 2.353577119 | 0.019021631 |
| C1orf226 | 0.466885377 | 0.033949927 | 2.353085299 | 0.019046495 |
| BEND7 | 0.594956024 | -2.656062331 | 2.35301501 | 0.019050051 |
| HPRT1 | 0.117100563 | 6.980880576 | 2.352810035 | 0.019060424 |
| PTPN2P2 | -0.211413639 | 0.115859775 | -2.351616867 | 0.019120902 |
| SNORD14A | -0.134644625 | 4.046944017 | -2.351233827 | 0.019140353 |
| RP6-109B7.2 | -0.462987083 | -1.051334144 | -2.351224211 | 0.019140842 |
| TRBJ2-7 | 0.317887177 | 0.472988428 | 2.35069815 | 0.019167585 |
| TOB2P1 | -0.32512497 | -0.246067744 | -2.350599797 | 0.019172588 |
| RNASET2 | -0.164987521 | 5.972670757 | -2.349979799 | 0.019204155 |
| PRSS23 | 0.354118096 | -0.318607702 | 2.34983313 | 0.01921163 |
| DEGS2 | -0.328165328 | 1.903815197 | -2.349762536 | 0.019215228 |
| LRRC28 | -0.184635859 | 3.23397707 | -2.349721852 | 0.019217302 |
| PODNL1 | -0.339319778 | -0.86535736 | -2.349481804 | 0.019229544 |
| ARIH2 | 0.070283059 | 6.255811632 | 2.349083011 | 0.019249896 |
| RP11-57K17.1 | -0.263918771 | -1.126043236 | -2.349033487 | 0.019252425 |
| RN7SL600P | -0.307790968 | 1.644204212 | -2.349007804 | 0.019253736 |
| ASGR1 | -0.321800932 | 1.860297832 | -2.348251264 | 0.019292405 |
| RP11-326C3.12 | 0.352534819 | 0.50810724 | 2.347975858 | 0.019306498 |
| AC091492.2 | 0.155924962 | 2.724880498 | 2.347660209 | 0.019322662 |
| AC093106.7 | -0.163910523 | 8.674278386 | -2.347602491 | 0.019325619 |
| INTS4L2 | -0.265145762 | -0.109172129 | -2.347507141 | 0.019330505 |
| TMEM63C | 0.451821197 | -0.546982077 | 2.346676428 | 0.019373116 |
| SPRY2 | 0.478629605 | 4.832948382 | 2.346390125 | 0.019387821 |
| ATP6V0D2 | 0.36307996 | -2.622155162 | 2.345995321 | 0.019408115 |
| RP11-326E7.1 | -0.260635996 | -0.330997098 | -2.345962272 | 0.019409815 |
| TSPO2 | 0.492397569 | -0.610045529 | 2.345852693 | 0.019415451 |
| RP4-775C13.1 | -0.102146505 | 5.390747731 | -2.345104746 | 0.019453961 |
| C21orf62 | -0.445278569 | -1.912453655 | -2.344974652 | 0.019460666 |
| RPL3P9 | 0.222037859 | 1.617816222 | 2.344886194 | 0.019465227 |
| STAR | -0.458032657 | 3.307773882 | -2.343813998 | 0.019520577 |
| RP11-335F8.2 | 0.186584705 | 1.72671293 | 2.343691196 | 0.019526925 |
| CNOT4 | 0.089072463 | 4.569180768 | 2.34305228 | 0.019559982 |
| ZNF419 | 0.163424208 | 2.364417073 | 2.343023231 | 0.019561487 |
| RP11-448G4.2 | 0.208304122 | 1.904573979 | 2.342397441 | 0.019593915 |
| CXorf23 | -0.117114952 | 3.555287268 | -2.342276793 | 0.019600172 |
| THRAP3P1 | 0.196832645 | 1.708276132 | 2.341566367 | 0.019637054 |
| TRBV5-4 | -0.325937566 | 0.370031923 | -2.341273134 | 0.019652294 |
| NYAP1 | 0.258802438 | -1.59054793 | 2.341135632 | 0.019659445 |
| NDUFV1 | 0.082450203 | 6.765224563 | 2.341066391 | 0.019663046 |
| RP11-632C17__A.1 | -0.109740122 | 10.78554742 | -2.340939651 | 0.01966964 |
| AGAP9 | -0.222600217 | 1.65364755 | -2.340142821 | 0.019711139 |
| HBG1 | 0.689570843 | 1.571842869 | 2.339794969 | 0.01972928 |
| LINC00890 | -0.547321125 | -1.595351122 | -2.339552119 | 0.019741953 |
| CLEC4C | -0.51895367 | -0.335875737 | -2.339362041 | 0.019751878 |
| MAP1S | 0.108700115 | 5.476758853 | 2.339145538 | 0.019763187 |
| CNNM1 | -0.538694044 | -0.907837596 | -2.338932556 | 0.019774318 |
| RNGTT | -0.102052588 | 4.497364237 | -2.338435855 | 0.019800298 |
| FGF14-AS2 | -0.343178423 | 0.097215945 | -2.337742867 | 0.019836595 |
| DIP2B | 0.08222748 | 5.513257353 | 2.337501061 | 0.019849274 |
| MESP2 | 0.29158841 | -1.794397607 | 2.337404926 | 0.019854317 |
| PAX8 | -0.583468464 | -0.436967296 | -2.337113796 | 0.019869595 |
| BRD2 | 0.101228529 | 8.554473714 | 2.336637198 | 0.019894628 |
| OR7E7P | -0.239380975 | 1.952918989 | -2.336410468 | 0.019906547 |
| LRRC4 | 0.421052129 | 3.440709756 | 2.33573367 | 0.019942162 |
| MRPL13 | 0.115283888 | 3.707104971 | 2.335568567 | 0.019950858 |
| AZI2 | -0.145572264 | 4.938219825 | -2.33537854 | 0.019960872 |
| CTD-2659N19.10 | 0.255723615 | -0.54314341 | 2.335198461 | 0.019970365 |
| TMEM185A | 0.11301564 | 2.292311063 | 2.334909999 | 0.019985581 |
| LINC01087 | -0.360831888 | -3.252467393 | -2.334829865 | 0.019989809 |
| HIST1H3G | 0.366301866 | -0.597017727 | 2.334811197 | 0.019990795 |
| C11orf30 | -0.093632066 | 4.008461497 | -2.334636472 | 0.020000018 |
| C16orf59 | 0.283935022 | 2.405410687 | 2.334614321 | 0.020001187 |
| CTB-131B5.2 | 0.303301053 | -1.916138258 | 2.334186765 | 0.020023774 |
| RP11-129K12.1 | -0.229720056 | 0.457168899 | -2.334068062 | 0.020030048 |
| RPL17 | 0.14079536 | 5.843626826 | 2.333397252 | 0.020065539 |
| PACSIN1 | 0.515847115 | 0.122625697 | 2.333308085 | 0.020070261 |
| RPS12 | -0.143849459 | 10.37966488 | -2.333237881 | 0.02007398 |
| RP11-361M10.3 | 0.24938466 | 0.383871413 | 2.333151387 | 0.020078562 |
| MT-TP | 0.246571908 | 4.557998536 | 2.332212754 | 0.020128343 |
| UBR3 | 0.085764068 | 4.551853827 | 2.331512058 | 0.020165576 |
| DGCR6L | -0.094286707 | 6.259939251 | -2.331419578 | 0.020170495 |
| HSPE1P4 | -0.210434021 | 1.066809119 | -2.331108772 | 0.020187033 |
| LIMK1 | -0.14778077 | 4.606833728 | -2.330253979 | 0.020232579 |
| PDPR | -0.118120017 | 5.889099824 | -2.330209447 | 0.020234954 |
| ZNF157 | -0.256231492 | -1.486858008 | -2.329922339 | 0.020250273 |
| Z83851.3 | -0.217395314 | -0.216618344 | -2.32873035 | 0.020313984 |
| SRSF12 | 0.381770544 | 0.270481976 | 2.328621614 | 0.020319805 |
| RP11-81H14.2 | -0.329671741 | 2.582270276 | -2.328160796 | 0.020344488 |
| RXRA | -0.255084662 | 5.126315367 | -2.327604687 | 0.020374311 |
| IER2 | -0.195374703 | 9.246458271 | -2.327340247 | 0.020388505 |
| RP11-977G19.11 | -0.180159027 | 1.629397196 | -2.327141599 | 0.020399174 |
| AHRR | 0.365144428 | -0.940435346 | 2.326603453 | 0.020428101 |
| HADH | -0.145417471 | 5.626359422 | -2.325221396 | 0.020502554 |
| MYBPC3 | 0.241144763 | 1.548244381 | 2.325172092 | 0.020505215 |
| RPL13A | 0.116531365 | 10.0655766 | 2.324046743 | 0.020566021 |
| RP11-346C16.4 | 0.249450996 | 2.562389049 | 2.323263971 | 0.02060841 |
| AC005519.4 | -0.190181732 | 2.202838831 | -2.322981922 | 0.020623702 |
| PCDHGA10 | 0.393585064 | -0.885517721 | 2.322207639 | 0.020665733 |
| RP11-20I23.8 | -0.362095743 | -1.003679174 | -2.321720922 | 0.020692193 |
| GSK3A | 0.072764876 | 5.904512574 | 2.321158476 | 0.020722806 |
| C3 | 0.385349492 | 0.380519412 | 2.321104362 | 0.020725753 |
| GREB1 | -0.40359678 | -3.138625232 | -2.320919562 | 0.020735822 |
| IGHEP2 | 0.242546928 | 1.699118479 | 2.320834617 | 0.020740451 |
| ALPK3 | 0.395078462 | -0.225317649 | 2.320364379 | 0.020766095 |
| PIGC | 0.075566278 | 4.690739435 | 2.319880475 | 0.020792513 |
| IGKV1-39 | 0.37610703 | 1.464615908 | 2.319775941 | 0.020798224 |
| FUK | -0.094675584 | 3.317662511 | -2.31957997 | 0.020808934 |
| RP11-85G20.1 | 0.250361291 | 1.109005747 | 2.319404848 | 0.020818508 |
| AP000704.5 | -0.278325915 | 0.135306893 | -2.319355028 | 0.020821233 |
| CRIPT | -0.08666316 | 4.29364288 | -2.318771272 | 0.02085318 |
| SERBP1P6 | -0.157533311 | 6.42019453 | -2.318543593 | 0.020865651 |
| LDHAP2 | 0.172836454 | 3.305941965 | 2.318010952 | 0.020894854 |
| RPLP0P2 | 0.252067253 | -1.666739849 | 2.317178623 | 0.020940558 |
| MYO15A | -0.28024431 | -1.612266226 | -2.316228748 | 0.020992824 |
| RP11-134K13.2 | 0.109133608 | 3.766135759 | 2.315409021 | 0.021038021 |
| PIWIL2 | -0.271065954 | -1.066074841 | -2.315395438 | 0.02103877 |
| RN7SKP23 | 0.249121599 | 0.778198691 | 2.315384171 | 0.021039392 |
| R3HDM2 | -0.085102076 | 5.467915963 | -2.315283949 | 0.021044924 |
| CORO1A | -0.171591709 | 9.089917446 | -2.315241853 | 0.021047248 |
| TPK1 | -0.230191866 | 1.964987639 | -2.314846527 | 0.021069084 |
| FCGR2C | -0.467941407 | 3.7300889 | -2.314602105 | 0.021082594 |
| CACNA1C-AS1 | -0.340860899 | 1.862906411 | -2.314554192 | 0.021085243 |
| SPINT1 | -0.360534652 | 3.606781655 | -2.314109956 | 0.02110982 |
| STARD8 | 0.349994397 | 3.4666317 | 2.313203529 | 0.021160045 |
| AC022819.3 | -0.337609136 | -2.623629856 | -2.313019899 | 0.021170233 |
| LMO7 | 0.152355883 | 1.487032412 | 2.312888619 | 0.021177519 |
| CERCAM | -0.33791901 | 3.17940328 | -2.31266348 | 0.021190019 |
| UPF3AP1 | 0.108874664 | 5.056919329 | 2.312242151 | 0.021213429 |
| LTV1 | 0.108658541 | 6.745841693 | 2.31221721 | 0.021214815 |
| ATRAID | -0.075071185 | 7.080862547 | -2.312082483 | 0.021222306 |
| ELK4 | 0.105033348 | 5.45178317 | 2.311950173 | 0.021229665 |
| ALKBH7 | -0.108151767 | 6.432258739 | -2.311946075 | 0.021229893 |
| GARIN1A | 0.262657901 | 0.695492323 | 2.311817609 | 0.021237041 |
| RP11-673E1.3 | 0.220347062 | 1.917456476 | 2.311772148 | 0.02123957 |
| L1CAM | 0.272612054 | -1.374503541 | 2.311321487 | 0.021264662 |
| PKIB | -0.45899128 | 0.784334344 | -2.311157181 | 0.021273817 |
| ZFX | 0.091924102 | 4.992031168 | 2.310998548 | 0.021282659 |
| AC093642.4 | -0.335801592 | -0.519843971 | -2.309990585 | 0.021338916 |
| LINC00865 | -0.603354743 | 0.584985803 | -2.309775853 | 0.021350917 |
| RPS15AP10 | 0.23410087 | 1.552459929 | 2.309596558 | 0.021360943 |
| SOX6 | 0.474993911 | -1.91973425 | 2.308926484 | 0.021398447 |
| CRELD2 | 0.132994169 | 4.39753961 | 2.308749477 | 0.021408364 |
| BMPR1APS1 | -0.391521539 | -0.87442627 | -2.308687028 | 0.021411863 |
| CTD-2020K17.4 | 0.26197608 | 0.245243972 | 2.308277902 | 0.021434803 |
| RPL30 | 0.097147938 | 10.15517647 | 2.308062091 | 0.021446912 |
| PLEKHA6 | -0.429087473 | 0.367407729 | -2.307618715 | 0.021471809 |
| CES3 | 0.344897924 | -0.625985631 | 2.307248793 | 0.0214926 |
| S100A10 | -0.408052573 | 8.418382724 | -2.307151514 | 0.021498071 |
| SPATA2L | 0.156974767 | 4.468145796 | 2.305912186 | 0.02156787 |
| CELF1 | 0.079875227 | 6.473533174 | 2.305798248 | 0.021574297 |
| PIWIL4 | -0.272285986 | 5.113769235 | -2.305134627 | 0.021611764 |
| GZMM | 0.322064272 | 3.395789751 | 2.304926322 | 0.021623536 |
| PTPN21 | 0.291143493 | -4.346800803 | 2.304727507 | 0.021634777 |
| KIF5C | 0.307374982 | -0.18717616 | 2.304595111 | 0.021642266 |
| DUOX2 | 0.403122177 | -3.551202573 | 2.304592359 | 0.021642421 |
| IGKV1-33 | 0.369846657 | 0.961381121 | 2.304200971 | 0.021664572 |
| DNAJC5 | -0.149417557 | 6.606449733 | -2.304111391 | 0.021669645 |
| DYNLL2 | 0.077126016 | 6.560930199 | 2.303469574 | 0.021706019 |
| RPL39L | 0.371225105 | 1.898306429 | 2.302945918 | 0.021735737 |
| RP13-977J11.5 | -0.238014173 | 1.921543996 | -2.302918956 | 0.021737268 |
| SEL1L | -0.118144773 | 5.952967774 | -2.302878233 | 0.02173958 |
| RP11-685N10.1 | -0.229220992 | 2.3443767 | -2.302549499 | 0.021758257 |
| RP11-457K10.2 | -0.176848581 | 1.473391007 | -2.302488539 | 0.021761722 |
| GCNT4 | 0.344776266 | 0.107323058 | 2.302289875 | 0.021773017 |
| MKRN3 | -0.657494025 | -0.679882804 | -2.302202442 | 0.02177799 |
| LINC00504 | -0.3406059 | -3.357127558 | -2.301997211 | 0.021789666 |
| TPM1 | 0.268250993 | 3.37157551 | 2.301299161 | 0.021829421 |
| ISOC1 | -0.142145295 | 5.493246569 | -2.301147827 | 0.021838048 |
| OSBPL9 | 0.089213442 | 5.673199338 | 2.301126069 | 0.021839289 |
| CD97 | -0.171881362 | 8.559965487 | -2.300780235 | 0.021859016 |
| ATP6V1E1P1 | -0.155526725 | 3.071746655 | -2.300228602 | 0.021890515 |
| XXcos-LUCA11.4 | -0.244406903 | 1.057861135 | -2.300183851 | 0.021893072 |
| RP5-1158E12.3 | 0.365973414 | -1.135220824 | 2.299900686 | 0.021909258 |
| CALU | -0.120327089 | 6.490210549 | -2.299734469 | 0.021918764 |
| MS4A4A | -0.509028789 | 2.517301037 | -2.299650759 | 0.021923552 |
| GLG1 | -0.12191473 | 6.044553243 | -2.299309813 | 0.021943066 |
| NRG4 | -0.589147836 | 1.718638766 | -2.299146795 | 0.021952401 |
| RP11-806H10.4 | 0.286313272 | -0.370628242 | 2.299111136 | 0.021954444 |
| OR52N2 | -0.314949072 | -1.242860757 | -2.298389243 | 0.02199583 |
| RP11-253I19.3 | 0.232110277 | 1.391583517 | 2.298258151 | 0.022003352 |
| BCCIP | -0.082786603 | 5.690475407 | -2.297529203 | 0.022045224 |
| RP11-834C11.7 | -0.369598556 | 0.129940407 | -2.297274532 | 0.022059869 |
| MYO18B | 0.730548516 | -3.226686183 | 2.297216718 | 0.022063195 |
| AC093106.5 | 0.202440225 | 1.314203894 | 2.29709135 | 0.022070408 |
| RP1-234P15.4 | -0.169454343 | 2.118333953 | -2.296756351 | 0.022089693 |
| AP001205.1 | 0.23057684 | 3.477103506 | 2.296749658 | 0.022090079 |
| PHOSPHO1 | 0.566023108 | 1.761710721 | 2.296705064 | 0.022092647 |
| RP11-667M19.9 | -0.240336993 | 0.40174627 | -2.296537361 | 0.022102308 |
| NDC1 | -0.134735404 | 4.662921182 | -2.296332828 | 0.022114096 |
| BCL2L15 | 0.340145169 | 1.746529537 | 2.29545862 | 0.02216454 |
| TAF6 | -0.088812844 | 5.481530963 | -2.295274788 | 0.02217516 |
| ECM2 | 0.34457064 | -2.792788449 | 2.294946849 | 0.022194117 |
| GCC2 | 0.093649458 | 5.16458532 | 2.294834191 | 0.022200633 |
| RP11-553L6.2 | -0.296547494 | -2.17305656 | -2.294595178 | 0.022214461 |
| RNU1-27P | -0.293689568 | -0.24542348 | -2.294369501 | 0.022227526 |
| DOK6 | 0.430643744 | -4.643373893 | 2.294345697 | 0.022228904 |
| SART3 | 0.103333837 | 4.854300171 | 2.293883771 | 0.022255666 |
| C11orf84 | 0.171781428 | 4.885660774 | 2.293786243 | 0.022261321 |
| ANG | -0.343639159 | 0.330504033 | -2.293356961 | 0.022286223 |
| RP11-455F5.5 | 0.203571518 | 3.388154155 | 2.293006124 | 0.022306592 |
| PRKAG1 | -0.070018727 | 5.468026798 | -2.292525946 | 0.022334498 |
| FGD5-AS1 | -0.063861299 | 6.926600763 | -2.292144936 | 0.022356662 |
| KCNH2 | 0.597398087 | 1.918904136 | 2.29213112 | 0.022357466 |
| FAM134B | 0.417850861 | 1.708293838 | 2.290718913 | 0.022439786 |
| SYNE1 | 0.25041737 | 6.242488612 | 2.290704397 | 0.022440633 |
| RP11-234A1.1 | -0.118098239 | 10.89162974 | -2.290701612 | 0.022440796 |
| RASSF1 | -0.128505231 | 7.247864257 | -2.290323686 | 0.022462872 |
| PARD3 | 0.490725002 | -0.25639087 | 2.289800293 | 0.022493476 |
| MT1L | 0.343270177 | -0.725534393 | 2.289612473 | 0.022504467 |
| SMG7-AS1 | -0.217663776 | -0.742878331 | -2.289244642 | 0.022526006 |
| RP11-386M24.4 | 0.116071767 | 5.416994995 | 2.289159526 | 0.022530993 |
| ARID1B | -0.120498068 | 6.285737218 | -2.289157179 | 0.02253113 |
| SNURF | 0.310220208 | -0.657507706 | 2.289049765 | 0.022537425 |
| ZNF664 | -0.141684385 | 6.041106941 | -2.289011798 | 0.02253965 |
| RBM23 | 0.093835991 | 5.782691805 | 2.288956756 | 0.022542876 |
| RTN1 | -0.528646491 | -0.038988133 | -2.288779434 | 0.022553273 |
| CROCC | -0.177471728 | 3.804423092 | -2.287978918 | 0.02260026 |
| VCAN | -0.721884997 | 6.031085169 | -2.287966065 | 0.022601015 |
| INE1 | -0.167273506 | 3.331188119 | -2.287947413 | 0.022602111 |
| SOX12 | -0.203106094 | 4.81154356 | -2.287842591 | 0.02260827 |
| IGLL1 | -0.65496438 | 5.555967622 | -2.287511872 | 0.022627713 |
| CAPN3 | -0.257921817 | 1.487268896 | -2.287496417 | 0.022628622 |
| RPP14 | 0.075037255 | 3.751742728 | 2.287413724 | 0.022633486 |
| CTB-114C7.3 | 0.293446233 | 0.197656303 | 2.28724801 | 0.022643236 |
| RAB24 | -0.167988974 | 4.106896849 | -2.286483018 | 0.022688292 |
| CTD-2325M2.1 | -0.236304121 | 0.393178715 | -2.286144722 | 0.022708242 |
| NXPH3 | 0.409736026 | -3.030559219 | 2.28612533 | 0.022709386 |
| DUSP5P1 | -0.398826482 | -1.711015647 | -2.285551908 | 0.022743238 |
| RP11-686D22.10 | -0.276000217 | 3.22849297 | -2.284808376 | 0.022787198 |
| HMG20B | 0.080999788 | 6.579180553 | 2.284697948 | 0.022793733 |
| ZDHHC20 | 0.133860279 | 6.677906829 | 2.284444335 | 0.022808748 |
| AC092594.1 | -0.247438015 | -0.583200954 | -2.284388616 | 0.022812048 |
| RPSAP47 | 0.346690175 | 6.488357485 | 2.284279608 | 0.022818506 |
| CTD-3220F14.2 | -0.225066828 | 1.552535139 | -2.284134627 | 0.022827096 |
| C17orf75 | 0.096439966 | 3.630674998 | 2.283759993 | 0.022849308 |
| YBEY | -0.142993516 | 4.152619416 | -2.283658392 | 0.022855335 |
| RP11-292F22.7 | 0.410269369 | -0.694061839 | 2.283480177 | 0.02286591 |
| RP11-840I19.3 | 0.30796763 | -1.231887949 | 2.283351847 | 0.022873527 |
| KIAA0226 | -0.09933694 | 5.05177277 | -2.283193186 | 0.022882949 |
| KBTBD11 | -0.3341927 | 5.092596463 | -2.282989814 | 0.02289503 |
| COX7A2L | 0.102198759 | 6.358717802 | 2.282747556 | 0.022909428 |
| FBXL17 | -0.132076873 | 4.078683761 | -2.282636433 | 0.022916035 |
| RP5-1068B5.3 | -0.213137892 | 0.557014104 | -2.281711737 | 0.022971079 |
| CLPB | -0.135353198 | 4.802111499 | -2.281283287 | 0.022996622 |
| ALPK2 | 0.350554851 | -3.965691029 | 2.281219516 | 0.023000426 |
| CCDC78 | 0.308128031 | 1.52838199 | 2.28083898 | 0.023023137 |
| TPRG1 | -0.375820724 | -1.918000071 | -2.280111688 | 0.023066596 |
| AF127936.7 | -0.284121608 | 1.661551151 | -2.280057577 | 0.023069833 |
| UBR7 | 0.090143772 | 5.809499519 | 2.279780759 | 0.023086395 |
| RP11-399E6.1 | 0.29530649 | -1.486755712 | 2.27954921 | 0.023100257 |
| PSMB4 | 0.070962301 | 8.412546925 | 2.279465055 | 0.023105296 |
| RPL7P18 | -0.307099187 | -0.097699662 | -2.279034107 | 0.023131119 |
| MYLK4 | -0.300706139 | 1.702670606 | -2.278502397 | 0.023163015 |
| GPR35 | 0.267025069 | 3.46616486 | 2.27825381 | 0.02317794 |
| RP5-855D21.2 | 0.199338047 | 1.73626138 | 2.278120964 | 0.023185919 |
| UBE2J1 | 0.127111955 | 8.607414971 | 2.277856223 | 0.023201828 |
| RP11-305B6.1 | -0.094761725 | 4.284270065 | -2.277761729 | 0.023207509 |
| DPF3 | 0.331708094 | -1.252300487 | 2.276700832 | 0.023271369 |
| EHD2 | -0.574628722 | 2.53829142 | -2.276641475 | 0.023274947 |
| TMEM140 | 0.255281481 | 4.248528605 | 2.276255547 | 0.023298219 |
| TUBG1 | 0.182617908 | 5.280933285 | 2.275980684 | 0.023314806 |
| SOCS2 | 0.478932679 | 4.208265788 | 2.275754595 | 0.023328457 |
| DHX58 | -0.130650876 | 4.675473748 | -2.27482424 | 0.023384706 |
| CTC-523E23.1 | -0.371938174 | 0.027440438 | -2.274271643 | 0.023418171 |
| NSUN6 | -0.119884022 | 4.768123399 | -2.273998839 | 0.023434707 |
| BLVRA | 0.320039039 | 5.260427149 | 2.273968952 | 0.02343652 |
| RP11-799O21.2 | -0.383889109 | -1.087024537 | -2.273685279 | 0.023453727 |
| AP003068.23 | -0.242979413 | 3.884933989 | -2.273176127 | 0.023484639 |
| RDH5 | -0.22632705 | 0.838966237 | -2.272967743 | 0.023497301 |
| EDEM1 | 0.121144737 | 6.889729799 | 2.272615299 | 0.02351873 |
| ENTPD1-AS1 | -0.17083599 | 1.862946421 | -2.272408901 | 0.023531287 |
| AC005532.5 | -0.263483658 | -0.535855654 | -2.272378158 | 0.023533157 |
| SUDS3 | -0.064349212 | 5.428391661 | -2.271844955 | 0.023565626 |
| FAM76A | 0.100564886 | 3.938074522 | 2.271708515 | 0.023573941 |
| SNX4 | 0.070596982 | 5.812668232 | 2.271404403 | 0.023592482 |
| ZDHHC14 | -0.206919089 | 2.411453218 | -2.270694742 | 0.0236358 |
| AB019441.29 | -0.128435266 | 9.106573528 | -2.270259379 | 0.023662408 |
| GNB1 | -0.068050101 | 8.709442318 | -2.270129597 | 0.023670345 |
| GLUL | 0.172999717 | 8.919179585 | 2.270030308 | 0.023676419 |
| RP11-23P13.6 | 0.375293661 | 0.336073907 | 2.269635588 | 0.023700578 |
| SEPT7P7 | 0.099759895 | 3.269643977 | 2.269326462 | 0.023719514 |
| DHRS12 | -0.150174739 | 3.400197178 | -2.269300534 | 0.023721103 |
| KMT2A | 0.110260959 | 6.544518957 | 2.268760005 | 0.023754246 |
| HNRNPA1P33 | 0.182584006 | 1.878426773 | 2.267654756 | 0.023822143 |
| FAM106CP | -0.410664307 | -0.654481438 | -2.267290772 | 0.023844539 |
| RP11-84G21.1 | -0.21547611 | 1.260078366 | -2.266831249 | 0.023872841 |
| BIK | -0.391752096 | 3.050974774 | -2.266289035 | 0.023906273 |
| PIP5K1B | 0.261953464 | 3.832213117 | 2.265845187 | 0.02393367 |
| FOXN2 | 0.097689739 | 6.606594339 | 2.265844679 | 0.023933702 |
| GUSBP11 | 0.230525291 | -2.240516418 | 2.265789217 | 0.023937127 |
| FERMT3 | -0.108606598 | 8.848516942 | -2.26536376 | 0.023963418 |
| NOMO1 | -0.109315822 | 5.479696757 | -2.265285451 | 0.02396826 |
| GNL2 | 0.065612822 | 5.693566617 | 2.264480644 | 0.02401807 |
| KANSL1 | 0.077582398 | 5.670425497 | 2.264087695 | 0.024042423 |
| RP11-326L17.1 | -0.293035753 | 0.756886131 | -2.263963924 | 0.024050098 |
| RPS7P1 | -0.092643346 | 10.44946587 | -2.263804641 | 0.024059978 |
| ACSL1 | 0.289993982 | 7.033799062 | 2.263676964 | 0.024067901 |
| MDFIC | -0.288258122 | 5.023743938 | -2.262929007 | 0.024114357 |
| HIST1H3A | -0.272504112 | 3.225969631 | -2.262339729 | 0.024151013 |
| DDX50 | -0.075204079 | 6.355895291 | -2.261899596 | 0.024178422 |
| RP11-459A10.1 | 0.233564921 | 2.598329514 | 2.26138411 | 0.024210559 |
| RP11-177G23.2 | -0.276203456 | 0.171551715 | -2.261272007 | 0.024217553 |
| PRORSD1P | 0.184148427 | 3.552347408 | 2.260163584 | 0.024286798 |
| EIF3M | -0.094409979 | 6.972465891 | -2.259693118 | 0.024316241 |
| CLSTN1 | -0.1393725 | 6.980028055 | -2.259595222 | 0.024322371 |
| TCF25 | 0.076158608 | 6.528767694 | 2.259553115 | 0.024325009 |
| RP5-1142A6.9 | -0.244287541 | -1.109371524 | -2.259476077 | 0.024329834 |
| RP11-353K11.1 | -0.188928388 | 1.335953302 | -2.259405552 | 0.024334253 |
| MAB21L1 | 0.360732448 | -3.08361995 | 2.258861543 | 0.024368359 |
| SLC22A5 | -0.235212708 | 1.584920668 | -2.258387836 | 0.024398091 |
| LYN | 0.177701811 | 8.935876887 | 2.258316215 | 0.024402589 |
| RP11-667K14.4 | 0.306299649 | 0.480854111 | 2.258084003 | 0.024417178 |
| CD177 | 0.708204715 | 2.774374633 | 2.257388121 | 0.024460942 |
| BRWD3 | 0.082229026 | 4.996501152 | 2.257291858 | 0.024467002 |
| AC007556.3 | -0.367486616 | 0.783208957 | -2.257232032 | 0.024470768 |
| MAST4 | 0.265769905 | 3.302186267 | 2.257099828 | 0.024479093 |
| PLD6 | -0.188825656 | 2.691296037 | -2.256673073 | 0.024505983 |
| RAB44 | -0.271396831 | 6.477129081 | -2.25640911 | 0.024522628 |
| RP13-228J13.8 | 0.138844331 | 2.244356104 | 2.256246361 | 0.024532896 |
| AC074019.2 | 0.165316834 | 1.288896291 | 2.255368312 | 0.024588355 |
| ANXA7 | 0.081071939 | 7.678700031 | 2.255081908 | 0.024606469 |
| MLXIPL | 0.528865595 | -2.053310872 | 2.254511418 | 0.024642583 |
| AL161626.1 | 0.315592419 | 3.567202016 | 2.254494443 | 0.024643659 |
| EFEMP1 | 0.42414973 | -3.201950728 | 2.254214073 | 0.024661425 |
| PIGK | -0.146081251 | 5.623202427 | -2.254176244 | 0.024663823 |
| ASTE1 | 0.097726008 | 3.490075413 | 2.253405923 | 0.024712698 |
| RP11-317N8.4 | 0.109635135 | 3.587097354 | 2.253224636 | 0.024724213 |
| KCTD17 | -0.239657163 | 3.327253216 | -2.253113345 | 0.024731283 |
| GS1-184P14.2 | 0.266051103 | 3.117608063 | 2.252176624 | 0.024790868 |
| RP11-7F18.2 | -0.219999652 | 2.296945725 | -2.25207801 | 0.024797148 |
| PSIP1 | 0.119814725 | 6.278393607 | 2.252045336 | 0.024799229 |
| ZNF562 | 0.111735004 | 5.085244853 | 2.251896675 | 0.0248087 |
| LINC00854 | 0.242307036 | -1.218225995 | 2.2518905 | 0.024809093 |
| HUS1 | 0.090907354 | 4.366053533 | 2.251612698 | 0.0248268 |
| KLK14 | -0.253545017 | -0.21926942 | -2.25048211 | 0.024898973 |
| HIST1H2AL | -0.344928336 | 1.958551085 | -2.250412485 | 0.024903424 |
| SCDP1 | 0.306615197 | 0.663858794 | 2.250324479 | 0.024909051 |
| ATP6V0B | 0.116545516 | 7.338191585 | 2.250011637 | 0.024929061 |
| AIRE | 0.34785721 | -3.532421276 | 2.249692105 | 0.024949513 |
| AC004510.3 | -0.29085625 | -0.930216569 | -2.249516514 | 0.024960759 |
| IL17RB | -0.348106579 | -2.205003945 | -2.249491944 | 0.024962332 |
| CTD-2013N24.2 | -0.208094812 | -0.51367463 | -2.24939942 | 0.02496826 |
| TAF6L | -0.078570122 | 4.1893582 | -2.248001695 | 0.025057955 |
| RP11-383F6.1 | -0.242027992 | 2.29232233 | -2.247916909 | 0.025063405 |
| CCDC58 | -0.108627569 | 3.75651101 | -2.24774076 | 0.025074731 |
| BTBD19 | 0.276365119 | 2.980052186 | 2.247613942 | 0.025082888 |
| CCDC141 | 0.313230912 | -1.605038554 | 2.24758605 | 0.025084682 |
| IL23A | 0.252643149 | 2.036181835 | 2.247108055 | 0.025115448 |
| API5P2 | -0.222911754 | -0.75467337 | -2.246926975 | 0.025127112 |
| RP11-399O19.9 | -0.232951392 | 0.573101419 | -2.246849193 | 0.025132124 |
| GLTPP1 | 0.142762885 | 3.796595408 | 2.246794008 | 0.02513568 |
| SSH2 | -0.154309304 | 5.95265114 | -2.24664995 | 0.025144966 |
| LINC00989 | 0.392098165 | -1.825355989 | 2.24655534 | 0.025151065 |
| EEF1B2P1 | 0.202778569 | 1.318391398 | 2.246283125 | 0.025168623 |
| CLEC2D | -0.222864381 | 4.628254071 | -2.246228663 | 0.025172137 |
| AC108059.1 | -0.202808239 | 0.686161257 | -2.246106923 | 0.025179994 |
| SQRDL | -0.175421615 | 5.306813174 | -2.245315628 | 0.025231112 |
| TM7SF2 | -0.14776383 | 3.475321113 | -2.245030352 | 0.025249563 |
| CTB-187L3.1 | 0.155639883 | 1.555699346 | 2.244397421 | 0.025290542 |
| FARP1 | 0.423823536 | -1.64789366 | 2.244388189 | 0.02529114 |
| DLEU2L | 0.119708697 | 2.399869695 | 2.244265255 | 0.025299106 |
| RP11-382J24.2 | 0.145544667 | 1.24848276 | 2.243997652 | 0.025316454 |
| TGIF2 | -0.163986793 | 5.711412097 | -2.243162082 | 0.025370689 |
| ZFP30 | -0.224030171 | 1.489615828 | -2.243100814 | 0.02537467 |
| ARHGEF3 | -0.189120646 | 5.155425476 | -2.242325917 | 0.025425064 |
| CTD-3092A11.2 | 0.183110193 | 4.799571658 | 2.241418239 | 0.025484204 |
| RP11-402J6.3 | 0.179436772 | 1.02617658 | 2.241381282 | 0.025486614 |
| ZNHIT6 | -0.093174012 | 4.747961059 | -2.241356505 | 0.02548823 |
| C15orf40 | -0.082837827 | 2.823777583 | -2.240928555 | 0.025516158 |
| ABCA13 | 0.554304248 | 2.164072816 | 2.240908971 | 0.025517437 |
| FOSB | -0.302008988 | 8.52461371 | -2.240874596 | 0.025519682 |
| AC159540.1 | 0.262221324 | 2.549580235 | 2.240639769 | 0.02553502 |
| CCDC116 | -0.216401934 | -0.885401119 | -2.240600135 | 0.025537609 |
| AC093159.1 | 0.272089282 | -0.635302743 | 2.240426713 | 0.025548943 |
| AKR1B1 | 0.201327856 | 4.810751964 | 2.239972677 | 0.025578635 |
| SSBP3 | -0.144584164 | 5.720057677 | -2.239512527 | 0.025608758 |
| C1RL-AS1 | 0.172111653 | 3.775717254 | 2.23873975 | 0.025659417 |
| CD226 | 0.331828999 | 2.181753278 | 2.238244807 | 0.025691907 |
| NADK2 | -0.10709273 | 4.837729562 | -2.238192469 | 0.025695345 |
| CDYL | 0.088934058 | 4.950634798 | 2.237996277 | 0.025708236 |
| DTYMK | 0.138985772 | 4.786937019 | 2.237771851 | 0.025722988 |
| FADS3 | 0.212738781 | 3.355372096 | 2.237703506 | 0.025727482 |
| COX7A2 | 0.104613092 | 7.071887258 | 2.237608338 | 0.025733741 |
| PNRC1 | 0.162533946 | 8.835405943 | 2.237526773 | 0.025739107 |
| SEC24C | 0.062755461 | 7.08703105 | 2.237464302 | 0.025743217 |
| CTD-3138B18.6 | -0.276124133 | 1.519629961 | -2.237448296 | 0.02574427 |
| CLEC1B | 0.486197245 | -0.250575164 | 2.237299683 | 0.02575405 |
| ZNF555 | 0.259102154 | 1.867304394 | 2.237197353 | 0.025760786 |
| SSSCA1 | 0.085627567 | 4.802279319 | 2.237183432 | 0.025761702 |
| RAD17 | -0.066312044 | 5.388819271 | -2.237087247 | 0.025768035 |
| PRTG | 0.42842761 | -5.86625987 | 2.236922257 | 0.025778902 |
| TMEM8B | -0.252964141 | 1.749851735 | -2.236618149 | 0.025798942 |
| MUC12 | 0.419610712 | -3.425772091 | 2.236429785 | 0.025811361 |
| MT-CYB | -0.0563845 | 12.79881102 | -2.235706099 | 0.025859124 |
| RP11-262H14.7 | -0.340503504 | -2.309527368 | -2.235692375 | 0.025860031 |
| SLC25A27 | 0.426638587 | -0.650295176 | 2.235561383 | 0.025868684 |
| MRPS18A | -0.107061292 | 5.705299746 | -2.235361663 | 0.025881884 |
| CNNM4 | -0.104974787 | 4.671064979 | -2.235346503 | 0.025882886 |
| SCAI | -0.153620832 | 3.760985008 | -2.234546578 | 0.025935812 |
| KCTD3 | -0.153851071 | 5.167795506 | -2.234347489 | 0.025948999 |
| VIPR1 | -0.382222483 | 1.622241525 | -2.233667955 | 0.025994053 |
| AC005102.1 | 0.225268174 | 1.25492339 | 2.233576809 | 0.026000101 |
| TRPM2 | -0.269964932 | 4.726337331 | -2.233332347 | 0.026016329 |
| ZACN | 0.260634394 | -2.209408165 | 2.233233516 | 0.026022892 |
| RIC3 | 0.327581503 | -0.432071937 | 2.233117362 | 0.026030608 |
| RPS6KA6 | 0.29133827 | -3.080906538 | 2.233059898 | 0.026034425 |
| DAPK2 | 0.355937616 | 2.092513732 | 2.232486623 | 0.026072538 |
| DLGAP1 | -0.327953312 | -3.39936246 | -2.232189932 | 0.026092282 |
| ZNF768 | -0.122595894 | 6.534013741 | -2.231436203 | 0.026142499 |
| HOXB7 | -0.553361206 | 0.604519678 | -2.23130802 | 0.026151047 |
| NANS | 0.1147775 | 5.210653147 | 2.23118607 | 0.026159182 |
| TMCC2 | 0.377114729 | 2.623786937 | 2.230605655 | 0.02619793 |
| PRKG1 | 0.433595066 | -4.140482641 | 2.230206057 | 0.026224636 |
| MAP3K15 | 0.37160724 | -0.055295773 | 2.23014623 | 0.026228637 |
| RP11-867G23.8 | -0.222933114 | 0.161708468 | -2.230073566 | 0.026233496 |
| RPL32 | 0.113665351 | 9.66548843 | 2.229885651 | 0.026246067 |
| U47924.30 | 0.138083627 | 3.554750903 | 2.229715015 | 0.026257486 |
| AC015971.2 | 0.228227207 | -0.785161505 | 2.229698444 | 0.026258595 |
| PCNA | 0.221910753 | 8.487873006 | 2.22873578 | 0.026323101 |
| DDX10P2 | -0.211425852 | -0.743135585 | -2.228269801 | 0.026354375 |
| XXbac-B476C20.9 | -0.222274888 | -0.352699508 | -2.228203125 | 0.026358852 |
| PRADC1 | -0.150826862 | 4.666853384 | -2.22814035 | 0.026363068 |
| RP11-192H23.4 | -0.137588054 | 1.694001283 | -2.227945524 | 0.026376157 |
| RP11-383G6.4 | 0.176056409 | -0.27060743 | 2.22649437 | 0.026473826 |
| RNU2-63P | -0.33291182 | 0.584263377 | -2.226491947 | 0.026473989 |
| RP11-401F2.3 | -0.225877086 | 0.46082542 | -2.225757302 | 0.026523553 |
| RP5-966M1.6 | -0.17319821 | 0.990036379 | -2.225748205 | 0.026524167 |
| RP11-632K20.6 | -0.326793411 | -0.710335972 | -2.225429133 | 0.02654572 |
| DDX60 | -0.195653688 | 3.891207689 | -2.22513347 | 0.026565704 |
| AP001059.5 | -0.309621364 | -0.668562997 | -2.224863458 | 0.026583966 |
| PLIN3 | -0.093576944 | 7.602790862 | -2.224477685 | 0.026610077 |
| RP11-95M15.2 | 0.17443915 | 0.80112611 | 2.224426631 | 0.026613534 |
| RP11-1055B8.6 | -0.633566592 | 3.36302731 | -2.22439378 | 0.026615759 |
| RP11-700P18.1 | 0.092451054 | 4.683957513 | 2.223913702 | 0.026648289 |
| RP11-624C23.1 | 0.26032428 | -1.366841233 | 2.223726129 | 0.026661009 |
| BRE | -0.149312141 | 5.437075127 | -2.223666324 | 0.026665065 |
| KCNK13 | -0.425504711 | -1.535205462 | -2.223179828 | 0.026698084 |
| AC026150.6 | -0.285998696 | -0.431506677 | -2.223150426 | 0.026700081 |
| SP4 | 0.112173129 | 4.093531787 | 2.222610372 | 0.026736778 |
| AC016735.1 | -0.809060077 | 3.696113222 | -2.222520453 | 0.026742892 |
| SLC16A1-AS1 | 0.238236566 | 0.949663853 | 2.222460054 | 0.026747 |
| INHA | 0.316252846 | -1.655881351 | 2.222400206 | 0.026751071 |
| SIGLEC9 | -0.452971361 | 3.783230484 | -2.221717273 | 0.026797561 |
| TAS2R43 | -0.292505154 | -1.239864731 | -2.221504363 | 0.026812069 |
| MLXIP | -0.09824972 | 6.535506642 | -2.2214351 | 0.02681679 |
| PPP6R3 | 0.056748723 | 6.528240939 | 2.22137903 | 0.026820613 |
| TRBJ2-6 | 0.286210754 | 0.558861976 | 2.220849804 | 0.026856715 |
| THNSL2 | -0.509898328 | -0.959687591 | -2.219821905 | 0.026926954 |
| MTMR8 | 0.227164528 | 0.691893333 | 2.219766264 | 0.026930761 |
| LRRC23 | -0.141437537 | 3.577689075 | -2.219699561 | 0.026935325 |
| ZNF660 | -0.215379889 | 2.730120891 | -2.219590169 | 0.026942811 |
| EEF1A1P16 | 0.145850538 | 5.157466706 | 2.219360991 | 0.026958502 |
| CGREF1 | 0.44614396 | 0.417943667 | 2.219208401 | 0.026968953 |
| AC073063.10 | -0.108073156 | 5.267444484 | -2.21902311 | 0.026981648 |
| PRRG2 | -0.220736592 | 0.072857441 | -2.218509077 | 0.027016895 |
| ZNF516 | -0.177021283 | 5.605757004 | -2.21814658 | 0.027041776 |
| RP11-730K11.1 | -0.255829088 | -2.029812944 | -2.217937425 | 0.02705614 |
| DLK1 | 0.759070099 | -0.503698633 | 2.217358137 | 0.02709596 |
| SUMF2 | -0.119133217 | 6.830348222 | -2.216997046 | 0.027120806 |
| RP11-96H19.1 | -0.178451062 | 2.749893199 | -2.216417166 | 0.027160749 |
| VNN1 | -0.489523775 | 4.751021614 | -2.215906858 | 0.027195941 |
| ZNF207 | 0.07459906 | 6.897304915 | 2.215258716 | 0.027240696 |
| LAD1 | 0.336231178 | -3.661506598 | 2.215173536 | 0.027246583 |
| TNPO1 | 0.094079808 | 5.728258596 | 2.214481386 | 0.027294456 |
| RPS25 | 0.103731526 | 10.98117257 | 2.214314869 | 0.027305984 |
| NFXL1 | -0.104855995 | 3.802800073 | -2.214170538 | 0.02731598 |
| PKDCC | 0.378822556 | -2.574874184 | 2.214141958 | 0.027317959 |
| SUCLA2P3 | -0.218086693 | -0.012880592 | -2.21381214 | 0.027340814 |
| ASTN2 | -0.298323661 | -2.467455134 | -2.213710009 | 0.027347895 |
| RP11-467P9.1 | -0.238447066 | -0.170309697 | -2.213008641 | 0.027396562 |
| RP11-750B16.1 | -0.208610949 | 5.180311004 | -2.212777133 | 0.027412642 |
| UBE2SP1 | 0.137165494 | 5.338557964 | 2.212208828 | 0.027452152 |
| RP11-126K1.6 | -0.172723616 | 3.949912113 | -2.211789189 | 0.027481357 |
| CTU2 | 0.11050432 | 3.526435324 | 2.211604808 | 0.027494198 |
| GRIN2D | -0.406963482 | -0.82449748 | -2.211244528 | 0.027519304 |
| POPDC2 | 0.148426737 | 1.924144083 | 2.211118546 | 0.027528087 |
| PCDHB14 | 0.435306333 | -1.011417198 | 2.211098989 | 0.027529451 |
| GNB2L1 | -0.108797935 | 10.17377745 | -2.209810369 | 0.027619439 |
| RP11-863K10.4 | 0.107414009 | 3.85608005 | 2.209745159 | 0.027623999 |
| PCDHGA12 | 0.364531253 | -1.823981043 | 2.209073023 | 0.027671044 |
| MRPL9 | 0.088712501 | 7.085035388 | 2.208400123 | 0.027718211 |
| WIZ | -0.067382688 | 4.902809267 | -2.208372125 | 0.027720175 |
| SAR1B | 0.113474262 | 3.815831536 | 2.208344787 | 0.027722093 |
| RP3-460G2.2 | -0.502770854 | 1.294749376 | -2.208276719 | 0.027726869 |
| LINC00689 | -0.429662921 | -4.804813879 | -2.208102441 | 0.0277391 |
| GPX1P2 | -0.286895559 | 1.23593906 | -2.207839612 | 0.027757554 |
| GTPBP3 | -0.083730023 | 3.763577789 | -2.207830071 | 0.027758224 |
| HBA1 | 0.665452051 | 6.037467928 | 2.207698985 | 0.027767432 |
| ILDR2 | 0.432623349 | 1.09875351 | 2.207621223 | 0.027772896 |
| RP11-678G14.4 | -0.375547966 | -1.957698006 | -2.207289311 | 0.027796227 |
| RPS7P10 | -0.107289539 | 7.897505049 | -2.20646777 | 0.027854048 |
| RPS4XP7 | 0.203993598 | 2.676388421 | 2.206171906 | 0.027874897 |
| SLC25A30 | 0.124472101 | 4.137380996 | 2.206157077 | 0.027875942 |
| ARL9 | 0.330330902 | -0.119263769 | 2.205606566 | 0.027914773 |
| C3orf62 | 0.131820835 | 4.580076625 | 2.205514778 | 0.027921251 |
| CHCHD2P2 | 0.182318329 | 5.336344274 | 2.205374258 | 0.027931172 |
| RP11-128A6.3 | -0.214962088 | 0.327690582 | -2.205370022 | 0.027931472 |
| TNFRSF25 | 0.285084471 | 3.826154991 | 2.205140148 | 0.027947708 |
| RP11-498C9.3 | 0.283484287 | 0.533658577 | 2.204939359 | 0.027961897 |
| PRSS27 | 0.24602326 | -0.361531719 | 2.204764317 | 0.027974271 |
| C19orf68 | -0.09352445 | 4.040116593 | -2.204651148 | 0.027982274 |
| ARID3A | -0.158054208 | 5.081519329 | -2.204325937 | 0.028005283 |
| BLZF1 | -0.090779825 | 4.925731731 | -2.204179897 | 0.028015621 |
| AC021860.1 | -0.385412499 | 1.366195161 | -2.204063881 | 0.028023835 |
| ADAMTSL2 | -0.394577551 | -3.052177291 | -2.203652523 | 0.028052979 |
| ATXN10 | -0.100239638 | 5.954000389 | -2.203589431 | 0.028057451 |
| AP1S3 | 0.26937165 | 1.783213263 | 2.203545907 | 0.028060537 |
| ZNF718 | -0.320613877 | 3.055525925 | -2.203258031 | 0.028080953 |
| ARL5B-AS1 | -0.170780173 | 1.696318377 | -2.202800939 | 0.028113395 |
| HEY2 | 0.271843834 | -2.666955163 | 2.202344911 | 0.028145795 |
| IPP | 0.145733458 | 1.709358307 | 2.202322182 | 0.028147411 |
| OLFML2B | -0.432498013 | 1.368276293 | -2.201886279 | 0.028178412 |
| PPATP1 | -0.193978869 | 0.990165789 | -2.201799748 | 0.02818457 |
| NIFKP3 | -0.388763842 | 0.913550317 | -2.201258844 | 0.028223087 |
| RP11-53I6.3 | 0.19312342 | 1.116875159 | 2.200539842 | 0.028274357 |
| DICER1-AS1 | -0.169941862 | 2.327392526 | -2.200261436 | 0.028294231 |
| ZNF717 | -0.266463317 | 0.540834298 | -2.200109882 | 0.028305055 |
| C17orf99 | 0.382807175 | -1.140379452 | 2.200051031 | 0.028309259 |
| RP11-362K14.6 | 0.184860124 | 1.607951389 | 2.199861866 | 0.028322775 |
| IGHJ3P | 0.345088492 | 1.627975979 | 2.199820558 | 0.028325728 |
| ATP1A3 | 0.27907341 | 1.403919619 | 2.199364046 | 0.028358373 |
| KIAA1551 | -0.17605993 | 6.685699548 | -2.199333072 | 0.028360589 |
| DNM1L | 0.070680291 | 6.262283042 | 2.199197072 | 0.028370322 |
| GDI1 | -0.084955382 | 6.499952205 | -2.198974983 | 0.028386221 |
| AOC1 | -0.490580776 | 0.764031178 | -2.198790217 | 0.028399454 |
| RIMKLA | -0.38281067 | -2.516542527 | -2.198365908 | 0.028429864 |
| CENPQ | -0.193272821 | 3.49259303 | -2.198364685 | 0.028429952 |
| GDE1 | 0.079793379 | 6.653327917 | 2.197837511 | 0.028467773 |
| MCL1 | 0.154116813 | 10.52110938 | 2.196964224 | 0.028530522 |
| ACTBP2 | -0.158369306 | 5.697109476 | -2.196639193 | 0.028553907 |
| EIF4BP3 | 0.123158233 | 6.364760671 | 2.196313923 | 0.028577326 |
| CCDC26 | -0.396438022 | 0.677350296 | -2.195899973 | 0.028607153 |
| RILPL2 | 0.118405055 | 8.534636326 | 2.195740926 | 0.028618621 |
| HMBS | 0.250392626 | 4.291840442 | 2.195580819 | 0.028630169 |
| GS1-124K5.2 | -0.143312238 | 2.463903279 | -2.195467468 | 0.028638346 |
| SH2D4A | -0.411236023 | -0.708721018 | -2.195246473 | 0.028654296 |
| CTC-429L19.3 | -0.204227279 | 0.539362921 | -2.194608233 | 0.028700404 |
| DIO3OS | 0.469245457 | -3.901145756 | 2.194487235 | 0.028709152 |
| CACNA1C | -0.43915006 | -2.21868555 | -2.194048401 | 0.0287409 |
| LINC01155 | -0.187451657 | 2.345781329 | -2.193587822 | 0.028774253 |
| DPY19L2 | -0.454355343 | 2.725447949 | -2.193507256 | 0.028780091 |
| LY6E | 0.229412926 | 7.430676754 | 2.19348163 | 0.028781948 |
| SPOCD1 | 0.393954723 | -4.074005983 | 2.193237752 | 0.028799626 |
| GRK6P1 | 0.194750466 | 1.953825623 | 2.193181234 | 0.028803724 |
| KLRG1 | -0.258588636 | 2.749305082 | -2.193166854 | 0.028804767 |
| ZBED3 | 0.252083653 | 4.565494804 | 2.193011773 | 0.028816015 |
| NAT14 | 0.168493612 | 4.05144557 | 2.192831078 | 0.028829126 |
| DAAM2 | 0.60434049 | -1.696111531 | 2.192651802 | 0.028842139 |
| RP11-707G14.8 | -0.166757215 | 2.030059079 | -2.192152526 | 0.028878406 |
| RP11-160H22.5 | 0.191433669 | -0.254019453 | 2.191730623 | 0.028909083 |
| IGKV3D-11 | 0.411103805 | 1.343304162 | 2.191594425 | 0.028918992 |
| AC096582.7 | -0.227979892 | 0.634768185 | -2.191507664 | 0.028925306 |
| RP11-580I16.2 | -0.168549953 | 1.970390598 | -2.191441249 | 0.02893014 |
| AL122127.25 | -0.536199555 | 4.115663941 | -2.190798517 | 0.028976958 |
| FFAR2 | 0.485204209 | 2.982352252 | 2.190686103 | 0.028985153 |
| MT-TC | 0.261619534 | 0.664619387 | 2.190252928 | 0.029016751 |
| AC026310.1 | 0.407715457 | -1.055673204 | 2.190157602 | 0.029023709 |
| GOLGA7B | -0.307566562 | 0.016615608 | -2.189950994 | 0.029038794 |
| DNAL1 | -0.127979649 | 2.38230516 | -2.189913625 | 0.029041523 |
| DDX3X | 0.103431628 | 9.140571356 | 2.189515981 | 0.029070576 |
| SSPO | 0.249393756 | 1.044021721 | 2.188336338 | 0.029156914 |
| MRPS23 | -0.093273035 | 5.590454456 | -2.188236031 | 0.029164266 |
| GALNT6 | -0.162895383 | 4.17589682 | -2.188122171 | 0.029172613 |
| SNORA68 | -0.237559226 | 0.792015656 | -2.18809467 | 0.029174629 |
| ZFP69B | 0.153200171 | 2.378722906 | 2.187864146 | 0.029191536 |
| SORL1 | -0.210400909 | 7.471895927 | -2.187855105 | 0.029192199 |
| BMI1 | 0.178844718 | 5.787764945 | 2.187425099 | 0.029223759 |
| FGFR2 | 0.404807949 | -3.196697662 | 2.187185379 | 0.029241366 |
| PHAX | -0.119297118 | 4.456529192 | -2.186669101 | 0.029279317 |
| FAM196A | 0.310891042 | -3.809441957 | 2.1866631 | 0.029279758 |
| GAPDHP1 | 0.125519681 | 8.576350101 | 2.186320888 | 0.029304937 |
| EIF3CL | 0.100963999 | 4.18045521 | 2.185888323 | 0.029336791 |
| ASMTL-AS1 | -0.154227044 | 5.05646712 | -2.185684113 | 0.029351839 |
| COMMD3 | 0.178841048 | 4.554472588 | 2.185140871 | 0.029391903 |
| MCRS1 | -0.060786789 | 6.069306295 | -2.185122184 | 0.029393282 |
| NLRP7 | -0.28100461 | -3.153074343 | -2.184932937 | 0.029407251 |
| CPSF1P1 | -0.404404183 | -0.279688572 | -2.184793392 | 0.029417554 |
| RP1-191J18.66 | -0.230989586 | 0.494941144 | -2.184713252 | 0.029423473 |
| UBAP1L | -0.140936836 | 2.153274957 | -2.184659347 | 0.029427455 |
| KDM8 | -0.123936287 | 1.991195795 | -2.184326676 | 0.029452038 |
| ADRBK2 | -0.171348635 | 5.241085766 | -2.184296148 | 0.029454295 |
| FAM110C | 0.279610406 | -2.829993966 | 2.183988774 | 0.029477026 |
| GAPDHP65 | 0.158046948 | 7.299061688 | 2.183873719 | 0.029485539 |
| TPTE2P4 | -0.242286358 | -1.037934956 | -2.183524643 | 0.029511379 |
| RP11-667K14.3 | 0.32618788 | -0.189642213 | 2.183468751 | 0.029515518 |
| RP11-1220K2.2 | -0.409127449 | -0.478754487 | -2.182534756 | 0.029584761 |
| AC004067.5 | -0.300793222 | 0.267597273 | -2.181988605 | 0.029625315 |
| CTB-181H17.1 | -0.27731168 | -0.421408417 | -2.181528311 | 0.029659531 |
| C7orf61 | 0.202874979 | 1.012922655 | 2.181493599 | 0.029662113 |
| WDR1 | 0.077351344 | 7.698885192 | 2.181403776 | 0.029668795 |
| PRDX2P1 | 0.316653547 | 1.397767899 | 2.180827361 | 0.029711702 |
| NOVA1 | 0.413115066 | -5.083585474 | 2.180724128 | 0.029719393 |
| RP11-22N19.2 | 0.265387977 | 3.455354673 | 2.180712418 | 0.029720265 |
| C12orf49 | -0.115492442 | 4.866832254 | -2.180516859 | 0.029734838 |
| SLC39A4 | -0.204201672 | 2.943211888 | -2.179966482 | 0.029775885 |
| GTPBP8 | 0.084378647 | 3.839905752 | 2.179892088 | 0.029781437 |
| MYBBP1A | -0.121703522 | 5.336877793 | -2.179872258 | 0.029782917 |
| TRGJP1 | -0.391338074 | 4.995217145 | -2.179740261 | 0.029792771 |
| IL9R | 0.376960328 | 0.187532401 | 2.179280676 | 0.029827101 |
| NCAPD2 | -0.152371562 | 6.215503516 | -2.179230922 | 0.029830819 |
| ERCC8 | -0.091705351 | 2.790189839 | -2.178915336 | 0.029854415 |
| MED25 | 0.087324491 | 5.264120795 | 2.178407647 | 0.029892408 |
| POLR2A | 0.105655032 | 7.879817288 | 2.178353956 | 0.029896429 |
| RP11-761N21.2 | -0.104607734 | 9.352582953 | -2.178179128 | 0.029909523 |
| BMS1P9 | -0.251753139 | -0.301266738 | -2.178096928 | 0.029915682 |
| BCORL1 | -0.149803456 | 4.184064513 | -2.177520498 | 0.029958898 |
| ENDOD1 | 0.260320751 | 4.24122209 | 2.177371899 | 0.029970048 |
| NUDT22 | -0.07773529 | 4.746779178 | -2.177159 | 0.029986029 |
| NDUFC2 | -0.117869418 | 3.990583748 | -2.176433436 | 0.030040545 |
| TMEM170A | 0.076641057 | 5.047337431 | 2.17576244 | 0.030091038 |
| RP11-417L19.4 | -0.214182002 | 0.148203376 | -2.175252528 | 0.030129458 |
| MYO1E | -0.381639604 | 1.089911219 | -2.175119126 | 0.030139517 |
| RP11-3D23.1 | -0.115769035 | 3.915088797 | -2.174951489 | 0.030152161 |
| TXNDC16 | 0.112344903 | 3.816392183 | 2.17488051 | 0.030157515 |
| ORAOV1 | -0.08848085 | 3.57332406 | -2.174663497 | 0.030173893 |
| SRSF10P1 | -0.27057409 | -0.823419917 | -2.173949439 | 0.030227834 |
| COX5BP6 | -0.252168253 | 1.441334488 | -2.173632997 | 0.030251765 |
| CKS1BP7 | 0.234183614 | 0.253995893 | 2.173431015 | 0.030267049 |
| AC010894.3 | -0.204604113 | 0.205752555 | -2.173195822 | 0.030284854 |
| GPBAR1 | -0.412102275 | 2.479136259 | -2.17278884 | 0.030315685 |
| IGKV3D-20 | 0.41607855 | 1.622167014 | 2.172763123 | 0.030317634 |
| IGLL3P | -0.340461568 | -0.167027512 | -2.171890065 | 0.030383869 |
| IGHA2 | 0.542816809 | 5.753947118 | 2.171800183 | 0.030390695 |
| SIRPG | -0.299104033 | 1.586555532 | -2.171759068 | 0.030393818 |
| INPP5A | 0.107942486 | 4.946386198 | 2.171445247 | 0.030417663 |
| AC009963.6 | -0.193710763 | 0.110618175 | -2.171399717 | 0.030421124 |
| CABLES1 | 0.45341738 | 2.165992499 | 2.171192408 | 0.030436886 |
| RP11-274E7.2 | -0.142077715 | 3.507162102 | -2.170593885 | 0.030482434 |
| NLRC5 | 0.169642861 | 5.600881024 | 2.170581325 | 0.03048339 |
| PM20D1 | 0.293795781 | -2.098058303 | 2.17055815 | 0.030485155 |
| CMAHP | -0.204331159 | 5.478267929 | -2.170200128 | 0.030512431 |
| PARK2 | 0.318669624 | -2.0042712 | 2.170016035 | 0.030526464 |
| RP11-534C12.1 | 0.198762763 | 0.946550148 | 2.169969239 | 0.030530032 |
| RP11-798M19.6 | -0.214983848 | 1.702268132 | -2.169730518 | 0.030548239 |
| TCEANC | 0.11608541 | 2.147725657 | 2.169586895 | 0.030559198 |
| HS3ST3B1 | 0.242516499 | 2.487796627 | 2.169176494 | 0.030590531 |
| SRA1 | 0.136470751 | 5.489869284 | 2.169161924 | 0.030591644 |
| CTD-3093M3.1 | -0.16327927 | 1.853264363 | -2.168673643 | 0.03062896 |
| AP001816.1 | -0.139416343 | 3.855332506 | -2.168404713 | 0.03064953 |
| DTX2P1 | -0.149465971 | 1.773377331 | -2.168347774 | 0.030653886 |
| STX4 | -0.07879956 | 5.943425372 | -2.168005092 | 0.030680117 |
| MT-RNR2 | 0.08036791 | 13.22706945 | 2.16789087 | 0.030688865 |
| KHNYN | 0.084846753 | 6.37588876 | 2.167395938 | 0.030726793 |
| RP3-340N1.2 | 0.413688957 | -1.637119158 | 2.167315946 | 0.030732927 |
| CFL1P5 | -0.199557311 | 2.162642658 | -2.167298716 | 0.030734248 |
| NYNRIN | -0.370295155 | 3.429069464 | -2.166963291 | 0.030759982 |
| RP11-342F17.2 | 0.241851938 | 0.392742484 | 2.166851306 | 0.030768577 |
| KLF16 | -0.085089829 | 6.525347541 | -2.166447451 | 0.030799592 |
| MPEG1 | -0.591822693 | 6.891289002 | -2.166364894 | 0.030805936 |
| EVI5L | -0.098082211 | 3.736181531 | -2.16622304 | 0.030816838 |
| RP11-383J24.6 | -0.177649293 | 4.583467997 | -2.165998725 | 0.030834085 |
| ARF3 | -0.108052884 | 7.555113015 | -2.165881126 | 0.03084313 |
| AC017074.2 | -0.240438427 | 0.555036409 | -2.165419349 | 0.030878669 |
| MYH15 | 0.268061502 | -2.210549302 | 2.164680301 | 0.030935622 |
| TMEM253 | 0.267467163 | -0.375207992 | 2.164533617 | 0.030946936 |
| PRKCE | 0.202376932 | 3.394658186 | 2.164264583 | 0.030967698 |
| RP11-517P14.7 | -0.266013231 | -1.125014346 | -2.164015419 | 0.030986936 |
| RP1-86C11.7 | -0.315397612 | 1.707638889 | -2.163999216 | 0.030988188 |
| CNTD1 | 0.263993356 | -2.507190851 | 2.163954513 | 0.030991641 |
| KAT8 | 0.074027702 | 5.986270144 | 2.163506357 | 0.031026274 |
| CHML | -0.126398951 | 5.481689537 | -2.16350241 | 0.03102658 |
| SEC62 | 0.10048403 | 6.187784742 | 2.163400757 | 0.03103444 |
| CC2D2A | -0.428429171 | -1.802468052 | -2.162985164 | 0.031066594 |
| AES | 0.111640569 | 7.819364518 | 2.162695877 | 0.031088993 |
| TMEM181 | -0.104574695 | 5.072595287 | -2.162365957 | 0.031114555 |
| BANP | -0.094265307 | 3.416438671 | -2.162096202 | 0.031135469 |
| RFPL1S | -0.495986844 | -1.769422917 | -2.161825601 | 0.031156461 |
| GOLGA8I | -0.316567786 | 0.515504071 | -2.16157558 | 0.031175867 |
| MMP21 | -0.252562542 | -1.116156819 | -2.161373039 | 0.031191596 |
| TSSC2 | -0.306742526 | -2.137839494 | -2.161185222 | 0.031206187 |
| COX6CP1 | 0.157253609 | 3.183008527 | 2.161087727 | 0.031213763 |
| MT2P1 | 0.300355921 | 1.614512635 | 2.161057046 | 0.031216148 |
| FNBP4 | 0.089599716 | 6.819387764 | 2.160118032 | 0.031289206 |
| ACTBP9 | -0.24444283 | -0.983078652 | -2.160093932 | 0.031291083 |
| RPL21P123 | -0.218722563 | 1.275381755 | -2.159580744 | 0.031331075 |
| SLC5A11 | -0.289224105 | -2.861341383 | -2.159555262 | 0.031333062 |
| RP11-158G18.1 | 0.26673955 | -0.137582743 | 2.158688612 | 0.031400702 |
| OLFM4 | 0.682989057 | 3.238874627 | 2.158688317 | 0.031400725 |
| RNU6-1017P | -0.31428811 | -0.614789942 | -2.158650252 | 0.031403699 |
| FAM50B | -0.347317215 | 2.658162623 | -2.157907544 | 0.03146177 |
| SUMO2 | -0.070126064 | 7.336056133 | -2.157900827 | 0.031462296 |
| TMEM52B | 0.398679194 | 1.133379995 | 2.157742114 | 0.031474718 |
| GS1-421I3.2 | 0.38219174 | -0.67484582 | 2.157670381 | 0.031480334 |
| ALOX15B | -0.473923186 | -1.495261276 | -2.157624552 | 0.031483922 |
| CTD-2161E19.1 | -0.130080822 | 8.122607146 | -2.157012317 | 0.03153189 |
| CTB-158E9.2 | -0.163595154 | 1.142244752 | -2.15677681 | 0.031550359 |
| WDR37 | 0.098835356 | 5.381731412 | 2.156603163 | 0.031563982 |
| TSHZ1 | -0.150260524 | 4.334109096 | -2.156582432 | 0.031565609 |
| PICK1 | -0.110484767 | 4.529366534 | -2.156350268 | 0.031583832 |
| UTP18 | -0.0776576 | 5.817401671 | -2.156050994 | 0.031607337 |
| MICAL1 | 0.181241908 | 7.053630239 | 2.155775339 | 0.031628999 |
| AC096772.6 | -0.117795156 | 4.104307093 | -2.155722104 | 0.031633184 |
| LINC00475 | -0.322692382 | -2.774720763 | -2.155116349 | 0.031680838 |
| SUMO4 | 0.237350701 | 0.187638715 | 2.155061018 | 0.031685194 |
| P2RX5 | -0.257243379 | 3.114426724 | -2.154807938 | 0.031705124 |
| FAM171A1 | 0.591855395 | 0.522270395 | 2.154751994 | 0.031709531 |
| LGALS3 | 0.397438877 | 5.9151649 | 2.154559192 | 0.031724724 |
| RHEBL1 | 0.202584388 | 2.171356523 | 2.153986262 | 0.031769906 |
| ARFGAP3 | 0.073811092 | 6.313034372 | 2.153930394 | 0.031774315 |
| SFRP1 | 0.427490134 | -3.045209214 | 2.153828131 | 0.031782386 |
| IGHV3-66 | 0.402681534 | 1.093036245 | 2.15365727 | 0.031795876 |
| PCDHB13 | 0.424437474 | -1.602343578 | 2.15365118 | 0.031796357 |
| HRH2 | -0.30220591 | 4.510346521 | -2.153482462 | 0.031809682 |
| TMED1 | -0.09167473 | 5.270391696 | -2.153228451 | 0.031829753 |
| SPCS1 | -0.080309226 | 7.166979001 | -2.153168852 | 0.031834464 |
| IGHA1 | 0.550454476 | 8.068804574 | 2.153149251 | 0.031836014 |
| PRKACA | -0.138309471 | 6.098721106 | -2.152874896 | 0.031857708 |
| WASIR2 | 0.383651822 | -0.361084718 | 2.152800503 | 0.031863593 |
| CLDN19 | -0.379966075 | -3.249986689 | -2.152215311 | 0.031909916 |
| PGBD1 | 0.184414266 | 3.483989341 | 2.150893034 | 0.032014801 |
| RPS15AP1 | -0.10857618 | 9.067482124 | -2.150636 | 0.032035223 |
| WBP2NL | -0.322821439 | -1.109428063 | -2.150543525 | 0.032042574 |
| POU2F3 | 0.331367096 | -3.87462347 | 2.150486396 | 0.032047115 |
| RP11-474G23.2 | -0.199098081 | 0.757467573 | -2.150481938 | 0.03204747 |
| CTD-2368P22.1 | -0.18810296 | 2.025349591 | -2.15004077 | 0.03208256 |
| MTOR | -0.068051434 | 5.437664689 | -2.149975594 | 0.032087747 |
| MYLPF | 0.181003056 | 1.887143477 | 2.149665113 | 0.032112466 |
| TECPR1 | -0.104183005 | 4.291137189 | -2.149356773 | 0.032137031 |
| CTC-510F12.2 | -0.273283645 | -0.55532524 | -2.149182469 | 0.032150924 |
| CIBAR1-DT | -0.342259346 | -2.273158544 | -2.14916191 | 0.032152564 |
| RP11-525K10.3 | -0.371807372 | -2.50547211 | -2.148839014 | 0.032178316 |
| KB-1683C8.1 | 0.188695084 | 1.420836857 | 2.148723137 | 0.032187562 |
| AL627309.1 | 0.409690854 | 5.165264052 | 2.148200313 | 0.032229307 |
| SEPHS1P6 | -0.181782745 | 0.401357369 | -2.148000121 | 0.032245304 |
| CLN6 | 0.1267304 | 5.340223455 | 2.147575227 | 0.032279279 |
| SMIM17 | -0.253848272 | 0.313665508 | -2.147507004 | 0.032284737 |
| TFDP2 | 0.198047516 | 5.481608385 | 2.14704812 | 0.03232147 |
| ADORA1 | -0.329395066 | -3.458286299 | -2.146956784 | 0.032328785 |
| RPL14 | 0.108499585 | 9.481869568 | 2.146792625 | 0.032341937 |
| AL592183.1 | -0.212072033 | 5.368189583 | -2.14638061 | 0.032374966 |
| RP11-167P11.2 | 0.206757445 | 0.276786798 | 2.146357335 | 0.032376833 |
| HES4 | 0.391206442 | 2.418171782 | 2.145697172 | 0.032429819 |
| RNF220 | -0.132324113 | 6.450951373 | -2.145376668 | 0.032455569 |
| AC093388.3 | -0.219777531 | 1.151711895 | -2.14451301 | 0.032525047 |
| AC026150.8 | -0.241280101 | 2.155428455 | -2.143839536 | 0.032579315 |
| ZNF431 | 0.107300398 | 4.971888265 | 2.143782918 | 0.03258388 |
| MMP9 | 0.57037186 | 6.176398601 | 2.143707003 | 0.032590003 |
| RP11-434B12.1 | -0.189554901 | 1.965709884 | -2.143011041 | 0.03264618 |
| CLEC4A | -0.387987243 | 3.971815145 | -2.142581928 | 0.032680858 |
| FGFR4 | 0.237998012 | -0.845651566 | 2.142522874 | 0.032685633 |
| ZFP36 | 0.213549242 | 10.29937398 | 2.142055105 | 0.032723477 |
| ACER1 | -0.253636851 | -1.49374609 | -2.141896773 | 0.032736295 |
| RN7SL334P | -0.34598937 | 0.951483498 | -2.141866579 | 0.032738739 |
| CRAMP1L | 0.113375513 | 4.308527062 | 2.141732967 | 0.03274956 |
| CTD-2012K14.4 | -0.249721812 | -0.324990464 | -2.141661106 | 0.032755381 |
| ABCA17P | -0.318581396 | -3.63614284 | -2.141000275 | 0.032808953 |
| KLHL3 | 0.218204622 | 1.451286132 | 2.140685603 | 0.032834489 |
| RNMTL1 | 0.092415358 | 4.697312155 | 2.140482432 | 0.032850985 |
| WWC3 | -0.211280944 | 4.62315779 | -2.140200538 | 0.032873885 |
| B4GALT3 | 0.078210494 | 5.852255839 | 2.13993305 | 0.032895628 |
| AQP9 | 0.548473346 | 5.13457847 | 2.13988754 | 0.032899329 |
| P2RY6 | -0.333696973 | -0.293151393 | -2.139869515 | 0.032900794 |
| DSTN | 0.151588382 | 6.360045688 | 2.139680261 | 0.032916187 |
| RP11-527L4.5 | -0.329179486 | -0.766646572 | -2.139460074 | 0.032934104 |
| IGHJ6 | 0.358444028 | 1.774926574 | 2.139195915 | 0.03295561 |
| PGGT1B | 0.105747963 | 5.040836027 | 2.138529569 | 0.033009912 |
| EEF1A1P7 | 0.149754902 | 4.056840375 | 2.138504922 | 0.033011922 |
| ZNF441 | -0.164847778 | 2.397105094 | -2.138005135 | 0.033052704 |
| TMEM241 | -0.099508096 | 2.611677604 | -2.137926824 | 0.033059098 |
| ZBTB25 | 0.104382249 | 3.228125623 | 2.137745601 | 0.033073898 |
| OVOL1 | 0.372209859 | -2.30222821 | 2.136897526 | 0.033143237 |
| LYVE1 | 0.433607967 | -0.997438364 | 2.136882564 | 0.033144461 |
| CHST10 | 0.175869633 | 3.767993072 | 2.136757375 | 0.033154707 |
| RP11-183C12.1 | 0.175723888 | 1.395333102 | 2.136732371 | 0.033156754 |
| RP11-497H16.7 | -0.32880107 | 1.345265386 | -2.136656452 | 0.03316297 |
| UNKL | 0.115018236 | 3.68163618 | 2.136552997 | 0.033171441 |
| RP11-34A14.3 | 0.238641126 | -0.285589826 | 2.136335992 | 0.033189216 |
| ZNF852 | -0.140858922 | 3.563987402 | -2.135582245 | 0.03325102 |
| CIZ1 | 0.088137994 | 7.276532105 | 2.135070309 | 0.033293054 |
| INPP5B | -0.107097834 | 5.0154158 | -2.134737091 | 0.033320437 |
| A2ML1 | 0.325878209 | -4.641430299 | 2.134467298 | 0.033342623 |
| ORM1 | 0.566999484 | 2.618591428 | 2.134435964 | 0.033345201 |
| DDAH1 | 0.435241942 | -0.132732435 | 2.134041404 | 0.033377671 |
| KIAA1468 | -0.080633555 | 5.139363961 | -2.133965779 | 0.033383898 |
| LINC01024 | 0.242702786 | 0.488521201 | 2.133745154 | 0.033402069 |
| ZFYVE26 | 0.066254078 | 4.35375735 | 2.133499959 | 0.033422274 |
| EPHA1 | -0.250611516 | 1.52506893 | -2.133415479 | 0.033429238 |
| SMPDL3B | 0.335927068 | 2.562486625 | 2.13331718 | 0.033437342 |
| AC005262.2 | -0.28355152 | 0.248338272 | -2.133306117 | 0.033438254 |
| MADCAM1 | -0.220368201 | -0.252404252 | -2.133146274 | 0.033451437 |
| LMO2 | -0.165256163 | 7.539663221 | -2.132926637 | 0.033469558 |
| ANKRD18DP | 0.374491638 | -1.254640867 | 2.132823722 | 0.033478052 |
| MID1IP1 | -0.157693444 | 7.2093812 | -2.132569417 | 0.033499049 |
| SETP14 | -0.115012865 | 8.99345556 | -2.132548622 | 0.033500766 |
| NBPF18P | -0.308973374 | -2.262888489 | -2.132417899 | 0.033511564 |
| MOCS3 | -0.186666032 | 3.002395978 | -2.1323389 | 0.033518091 |
| RP11-352D3.2 | -0.305881255 | -0.826443258 | -2.132081053 | 0.033539402 |
| RP11-26F2.1 | -0.44789251 | 2.502672545 | -2.131970074 | 0.033548578 |
| AF131215.5 | -0.301787525 | -1.810675274 | -2.131953911 | 0.033549914 |
| RP11-932O9.9 | 0.242574863 | 3.337725202 | 2.131026005 | 0.033626722 |
| IGKV1D-8 | 0.345765992 | 0.175254703 | 2.130968217 | 0.033631511 |
| IFIT1B | 0.535048095 | -0.27437722 | 2.130707384 | 0.033653131 |
| RP11-255E6.6 | -0.254141536 | -0.529666284 | -2.130319103 | 0.033685338 |
| RPL12P8 | -0.132652336 | 6.132046289 | -2.130096857 | 0.033703784 |
| IMPACT | -0.141891198 | 5.192444729 | -2.130049479 | 0.033707718 |
| PCBD1 | -0.164447745 | 5.79956626 | -2.129916101 | 0.033718793 |
| DNAJB2 | 0.11770803 | 4.748222244 | 2.129584191 | 0.033746369 |
| STAG1 | 0.06188954 | 5.284311514 | 2.129535361 | 0.033750427 |
| AFG3L1P | -0.089483357 | 3.566254259 | -2.129217584 | 0.033776849 |
| SMIM1 | 0.301995012 | 2.533291469 | 2.129103661 | 0.033786326 |
| MTUS1 | 0.369719261 | -1.863246054 | 2.128914397 | 0.033802075 |
| THOC3 | -0.134721554 | 3.858682275 | -2.128833065 | 0.033808844 |
| MYEOV2 | 0.083430076 | 4.592046226 | 2.12872652 | 0.033817714 |
| PABPC4L | 0.485608473 | -2.594066458 | 2.12845 | 0.033840744 |
| SH3BP4 | 0.588602184 | 0.928344661 | 2.128448826 | 0.033840842 |
| ATG9B | 0.218081727 | -0.341816157 | 2.127713634 | 0.033902138 |
| DDX18P1 | -0.204574372 | -1.277476961 | -2.127223084 | 0.03394309 |
| CASC7 | 0.122288042 | 5.047733581 | 2.126769638 | 0.033980982 |
| MAGIX | 0.141714548 | 0.386904188 | 2.126728268 | 0.033984441 |
| CLOCK | -0.123027593 | 3.201165409 | -2.126239167 | 0.034025357 |
| SVILP1 | 0.236518671 | 1.10574284 | 2.126167978 | 0.034031316 |
| ALDH3A1 | 0.300259192 | -2.028167713 | 2.125088069 | 0.03412182 |
| GRID1 | -0.370985961 | -3.511847847 | -2.125017392 | 0.03412775 |
| MEG3 | 0.740605762 | -1.017154218 | 2.12485085 | 0.034141728 |
| NUDT8 | 0.217090977 | 3.26677104 | 2.122979707 | 0.03429911 |
| LANCL3 | -0.379250895 | -0.257036795 | -2.122646691 | 0.034327185 |
| XRCC2 | 0.210887022 | 3.028795808 | 2.122356098 | 0.0343517 |
| MURC | 0.24450736 | -0.708117481 | 2.122093447 | 0.03437387 |
| SDC3 | 0.444797737 | 0.545614785 | 2.121560707 | 0.034418877 |
| RP11-77H9.2 | -0.125289224 | 2.299915151 | -2.12133168 | 0.03443824 |
| VPS13B | -0.062287744 | 5.461168805 | -2.121317772 | 0.034439417 |
| TSPYL5 | 0.442528952 | 0.566564968 | 2.121219121 | 0.034447761 |
| XXbac-BPG181M17.6 | 0.274045806 | -0.692144344 | 2.120683495 | 0.034493094 |
| BSN | 0.275850521 | -2.162596732 | 2.120255991 | 0.034529313 |
| ANXA8L2 | -0.463937386 | -1.693621973 | -2.120015912 | 0.034549668 |
| CA5BP1 | 0.107169482 | 5.270324873 | 2.120004396 | 0.034550644 |
| VWA5A | -0.280598594 | 3.065402296 | -2.119908622 | 0.034558767 |
| TACR2 | 0.298641569 | -1.713824514 | 2.11984877 | 0.034563844 |
| RP11-1007O24.4 | -0.213127225 | 0.089225235 | -2.119154343 | 0.034622798 |
| RP11-760N9.1 | -0.357180917 | -1.95158878 | -2.119086561 | 0.034628557 |
| KLF3P1 | 0.219256678 | -0.379197722 | 2.1187095 | 0.034660608 |
| RP11-613F7.1 | 0.227355825 | 4.822306111 | 2.118371626 | 0.03468935 |
| RP11-554D14.2 | -0.342088008 | -0.895700851 | -2.118132763 | 0.034709682 |
| RP11-307O1.1 | 0.233158362 | 2.046061974 | 2.118055458 | 0.034716264 |
| ZNF19 | -0.190691596 | -0.504451893 | -2.11800743 | 0.034720354 |
| TNS3 | -0.257649375 | 4.822375672 | -2.117942098 | 0.034725918 |
| IGFBP6 | 0.310609383 | 1.528711038 | 2.117851571 | 0.03473363 |
| RP11-447H19.1 | 0.286897647 | -0.694146573 | 2.117351518 | 0.034776252 |
| RP1-7G5.5 | 0.21935743 | 0.905863068 | 2.117258045 | 0.034784224 |
| WDR35 | -0.262584986 | 3.304345466 | -2.117255439 | 0.034784446 |
| SULT4A1 | 0.447098683 | -2.535376318 | 2.11675134 | 0.034827467 |
| RPSA | 0.143075549 | 8.289755122 | 2.116449302 | 0.034853266 |
| ARHGAP18 | -0.159520943 | 4.564157034 | -2.116042617 | 0.034888028 |
| CPSF6 | 0.101648393 | 6.971176815 | 2.11589058 | 0.034901032 |
| RP11-439E19.7 | -0.290535011 | -0.742026972 | -2.115480651 | 0.034936113 |
| ATXN3 | 0.07398814 | 4.240928621 | 2.115369493 | 0.034945631 |
| AC051649.6 | 0.29876621 | -0.379854924 | 2.114982671 | 0.03497877 |
| TRIP6 | 0.241670848 | 4.428202485 | 2.114544988 | 0.035016299 |
| RP11-63E5.6 | 0.174626672 | 1.544025816 | 2.114493649 | 0.035020703 |
| TMEM59 | -0.065069952 | 7.059154568 | -2.114402043 | 0.035028563 |
| RP11-339B21.10 | -0.277933711 | -0.603748191 | -2.113824314 | 0.035078168 |
| COL4A5 | -0.885808801 | 0.364559803 | -2.113224755 | 0.035129711 |
| NDST3 | -0.56799145 | -1.916437973 | -2.112966661 | 0.035151918 |
| RP1-153G14.4 | 0.270579032 | -1.73351173 | 2.112829079 | 0.035163762 |
| CD19 | -0.342540335 | 1.857727362 | -2.112656595 | 0.035178614 |
| NDUFA4L2 | 0.273912033 | -1.423918805 | 2.112242766 | 0.03521427 |
| USP31 | 0.155433895 | 2.464692249 | 2.112180504 | 0.035219637 |
| TRIM61 | 0.275367854 | -2.037466034 | 2.11142432 | 0.03528488 |
| PF4V1 | 0.373497371 | -0.34037024 | 2.111116233 | 0.035311491 |
| ACTR1B | 0.100154587 | 6.89293696 | 2.110936493 | 0.035327024 |
| MYO1C | 0.281479915 | 2.56488726 | 2.110452658 | 0.035368866 |
| COL4A3BP | 0.095454599 | 5.281241718 | 2.110081693 | 0.035400975 |
| DDX11L5 | -0.31493341 | -1.06101268 | -2.110049285 | 0.035403781 |
| RP11-47G11.2 | -0.322847663 | -0.579410145 | -2.109662246 | 0.035437311 |
| SLCO2B1 | 0.497016552 | -2.720434207 | 2.109588622 | 0.035443692 |
| AD000090.2 | -0.160313917 | 3.990983024 | -2.108637454 | 0.035526221 |
| ARHGAP26-IT1 | -0.305538966 | -0.127026643 | -2.108589771 | 0.035530363 |
| VTI1BP1 | -0.165108751 | 0.76181859 | -2.108539652 | 0.035534717 |
| IGHV3-43 | 0.382087103 | 0.955596915 | 2.108359977 | 0.035550328 |
| RP11-3P17.5 | 0.318553529 | 1.043115831 | 2.108111853 | 0.035571896 |
| EML5 | 0.257212303 | -1.117738704 | 2.107333783 | 0.035639603 |
| TRBV7-8 | -0.281629634 | 0.849329549 | -2.107248415 | 0.035647038 |
| PA2G4P4 | -0.10579571 | 6.151768593 | -2.107219175 | 0.035649585 |
| MCTP1 | -0.206725799 | 4.49296062 | -2.106968568 | 0.035671422 |
| KLHL18 | 0.101590731 | 4.722366334 | 2.106888989 | 0.035678358 |
| RP11-861E21.1 | -0.300959951 | -0.573737343 | -2.106813237 | 0.035684962 |
| UTP11L | 0.075054523 | 5.247053003 | 2.106711834 | 0.035693804 |
| PLCB4 | 0.535468436 | -0.529986522 | 2.106490569 | 0.035713103 |
| GATAD2B | 0.08498355 | 6.101175846 | 2.106274596 | 0.035731949 |
| TM9SF1 | -0.100983124 | 1.641635274 | -2.106017086 | 0.035754432 |
| FBXL19-AS1 | -0.182872991 | 2.55359681 | -2.105708685 | 0.035781373 |
| PTPRVP | 0.240415328 | 0.653376184 | 2.105671239 | 0.035784645 |
| SLC39A8 | 0.176212806 | 5.695302136 | 2.105234224 | 0.035822855 |
| PELP1 | -0.088880612 | 5.255465714 | -2.104943696 | 0.035848276 |
| SAFB2 | -0.066495203 | 6.269135213 | -2.104863078 | 0.035855333 |
| FAM194A | 0.238365044 | -1.413831555 | 2.10469533 | 0.03587002 |
| RARB | -0.330140988 | -3.363558544 | -2.104452573 | 0.035891284 |
| TPRKBP2 | 0.24450535 | 0.738247752 | 2.103973052 | 0.035933318 |
| HMGN1 | 0.118830933 | 8.043953904 | 2.10390183 | 0.035939565 |
| TACSTD2 | 0.565455563 | 2.073754755 | 2.103394047 | 0.03598413 |
| BRD7P5 | 0.13297518 | 1.444901737 | 2.103338595 | 0.035988999 |
| ENDOV | -0.12347124 | 2.651600192 | -2.10278326 | 0.036037797 |
| MS4A6E | -0.337243584 | -1.399393007 | -2.102611164 | 0.036052931 |
| TGFBR2 | 0.165721914 | 6.505455374 | 2.102079902 | 0.036099683 |
| AK3 | 0.111560971 | 6.764331525 | 2.102072592 | 0.036100326 |
| THUMPD1 | 0.09132754 | 5.974596114 | 2.101772959 | 0.036126718 |
| CLCN1 | -0.36915041 | -0.065829123 | -2.1016262 | 0.03613965 |
| SERHL | -0.267879637 | -0.751285355 | -2.101407597 | 0.036158921 |
| CDK3 | -0.166500217 | 0.855883137 | -2.100808336 | 0.036211794 |
| TRBV6-1 | -0.282497084 | -0.233038516 | -2.100658414 | 0.036225032 |
| ZNF93 | -0.157318894 | 3.162532975 | -2.100594059 | 0.036230715 |
| RPL29 | 0.097843923 | 10.01616484 | 2.10040121 | 0.036247752 |
| NT5C3A | 0.109882804 | 5.713688287 | 2.100337243 | 0.036253404 |
| RP11-801F7.1 | -0.242427597 | -0.008563405 | -2.100061764 | 0.036277756 |
| CTD-2616J11.10 | 0.225310623 | 0.469679271 | 2.099602274 | 0.036318404 |
| ARHGAP23P1 | 0.28900489 | -1.903600239 | 2.098641932 | 0.036403486 |
| ACADVL | -0.094382499 | 8.331658071 | -2.098317669 | 0.036432253 |
| LANCL1 | 0.104549657 | 5.665436278 | 2.098307203 | 0.036433182 |
| PPM1E | 0.322932453 | -2.74726432 | 2.09815217 | 0.036446942 |
| LAP3P2 | 0.139899059 | 3.801350449 | 2.098052316 | 0.036455808 |
| BTBD7P1 | -0.465294809 | -0.367837458 | -2.097528034 | 0.036502386 |
| H2BFS | -0.247267681 | 0.999529938 | -2.097205169 | 0.036531095 |
| C1orf173 | -0.325268211 | -4.040137432 | -2.097031959 | 0.036546505 |
| CTD-2630F21.1 | -0.213441278 | 1.0079651 | -2.096576379 | 0.036587062 |
| BAZ1B | -0.093229587 | 6.592674291 | -2.096412679 | 0.036601645 |
| TMED4 | 0.071875069 | 6.535153709 | 2.096064125 | 0.036632711 |
| AMPH | -0.453980944 | -2.631597243 | -2.095980402 | 0.036640177 |
| RP11-549B18.1 | -0.315499442 | 3.101957091 | -2.095639204 | 0.036670614 |
| KIF27 | -0.136994599 | 2.92150298 | -2.095523149 | 0.036680972 |
| SLC26A8 | -0.355321991 | 1.876485395 | -2.095468064 | 0.03668589 |
| RP11-539G18.2 | -0.234257154 | -1.807469112 | -2.095105457 | 0.036718272 |
| TMEM217 | 0.274029666 | 2.463538329 | 2.09502047 | 0.036725866 |
| ADRM1 | 0.092020503 | 7.338001503 | 2.095019402 | 0.036725961 |
| C12orf55 | -0.259457344 | -2.991742819 | -2.094941376 | 0.036732934 |
| ZFP37 | -0.29493583 | -2.431370071 | -2.094568734 | 0.03676625 |
| SIT1 | -0.302265452 | 2.123097157 | -2.094558254 | 0.036767187 |
| DEF8 | -0.165865812 | 4.597388656 | -2.09439868 | 0.036781462 |
| IFITM4P | 0.265718075 | -0.259268626 | 2.094385734 | 0.03678262 |
| SPNS3 | -0.322685666 | 6.200896703 | -2.093921587 | 0.036824169 |
| GALNT14 | 0.404531046 | 2.355570613 | 2.093717207 | 0.036842477 |
| ANKS6 | 0.21553332 | 2.413168034 | 2.093662712 | 0.03684736 |
| AXIN2 | 0.320089327 | -0.87942653 | 2.093570891 | 0.036855588 |
| KCTD20 | 0.077563771 | 7.102319836 | 2.093261954 | 0.036883285 |
| RP5-1074L1.1 | -0.145856707 | 0.627209962 | -2.092893376 | 0.036916353 |
| PTPN3 | 0.352332014 | -4.67648243 | 2.092615508 | 0.036941299 |
| MDM4 | 0.115082814 | 6.452210193 | 2.092215299 | 0.036977254 |
| AC016894.1 | 0.235769598 | -0.314577535 | 2.091957738 | 0.037000409 |
| AC138649.1 | -0.358231081 | -0.963721573 | -2.09174384 | 0.037019648 |
| FHL1 | 0.341353936 | 4.429122988 | 2.091461112 | 0.037045091 |
| ADAM28 | -0.27858532 | 4.624723296 | -2.091147362 | 0.037073344 |
| HNRNPA3P10 | 0.10174908 | 5.293509773 | 2.090935193 | 0.037092459 |
| LOX | 0.553900477 | -0.784099412 | 2.090213686 | 0.037157528 |
| CLNK | -0.461626365 | -0.816920108 | -2.089962785 | 0.037180178 |
| TBC1D10C | -0.157027504 | 6.119840042 | -2.089861626 | 0.037189313 |
| ADD2 | 0.500582377 | 2.161063213 | 2.089845203 | 0.037190797 |
| ARHGAP33 | 0.141769666 | 4.928354714 | 2.089394445 | 0.037231529 |
| PTMAP3 | 0.184742984 | 1.253370258 | 2.089036383 | 0.037263911 |
| PVT1 | 0.185314147 | 3.761221354 | 2.08884199 | 0.037281502 |
| RP4-758J18.10 | -0.397591415 | 0.255396176 | -2.088743769 | 0.037290393 |
| RNF144B | 0.307881524 | 4.527559643 | 2.088738912 | 0.037290832 |
| RP11-324I22.4 | -0.161538775 | 2.397355018 | -2.088692509 | 0.037295033 |
| MAFA | 0.33496801 | -1.762307009 | 2.088585748 | 0.0373047 |
| AC006024.4 | 0.265324839 | -1.726993168 | 2.08834268 | 0.037326717 |
| RP1-96H9.5 | -0.246989204 | -0.169423031 | -2.088262146 | 0.037334014 |
| AC010240.2 | 0.258787953 | 1.275540872 | 2.088111704 | 0.037347649 |
| CLIC6 | 0.32387957 | -2.6018239 | 2.088053896 | 0.03735289 |
| GSS | -0.081557635 | 6.356583592 | -2.087920078 | 0.037365023 |
| PTPRU | 0.362625238 | -1.166920656 | 2.087734333 | 0.03738187 |
| GYLTL1B | -0.32554957 | -0.132391008 | -2.087674642 | 0.037387285 |
| PHRF1 | -0.09881346 | 5.687104424 | -2.087661787 | 0.037388451 |
| RPS20 | 0.087732873 | 10.54097474 | 2.087315171 | 0.037419911 |
| ZWILCH | 0.087452023 | 4.324814747 | 2.08615686 | 0.037525207 |
| RP11-424G14.1 | 0.640487078 | 0.618036164 | 2.086028693 | 0.037536874 |
| H3F3AP4 | -0.090864554 | 9.488647017 | -2.085821698 | 0.037555722 |
| ZNF619 | 0.089654896 | 3.45554289 | 2.085709938 | 0.037565902 |
| IGLV3-25 | 0.432665703 | 2.355277208 | 2.085579792 | 0.03757776 |
| AC012354.8 | -0.113979308 | 4.552278256 | -2.085504204 | 0.037584648 |
| VAV3-AS1 | -0.249640871 | 0.077613752 | -2.084809048 | 0.037648048 |
| CTC-325J23.3 | -0.208575371 | -0.195360745 | -2.084716787 | 0.037656469 |
| CDC42-IT1 | 0.227107785 | 1.467169786 | 2.084468532 | 0.037679137 |
| NHP2 | -0.110083595 | 6.643334409 | -2.084379892 | 0.037687233 |
| RPS3AP6 | -0.091584594 | 11.28582337 | -2.084340385 | 0.037690842 |
| RP11-543P15.1 | -0.093761605 | 11.02248212 | -2.084250231 | 0.037699079 |
| AC023490.1 | -0.280795875 | 1.372729871 | -2.084080909 | 0.037714554 |
| DEFA4 | 0.664146406 | 6.491263619 | 2.083319037 | 0.037784248 |
| SCAND2P | -0.095730892 | 2.931035146 | -2.082562922 | 0.037853524 |
| LOXL2 | 0.185187799 | 0.174809623 | 2.082361162 | 0.037872028 |
| TMSB4XP2 | 0.227470786 | 2.551959353 | 2.082240269 | 0.037883119 |
| ANKRD20A4 | 0.405265441 | -2.055614757 | 2.082235851 | 0.037883524 |
| ZNF85 | -0.150187383 | 3.308665868 | -2.08218854 | 0.037887866 |
| NSMCE4A | 0.07648346 | 5.292207901 | 2.08196386 | 0.037908488 |
| COL28A1 | -0.330626549 | -2.524197295 | -2.081721139 | 0.037930777 |
| KIAA1191 | -0.078406264 | 6.04533734 | -2.081647988 | 0.037937497 |
| CTD-2589M5.5 | -0.293798109 | -0.811106563 | -2.081519721 | 0.037949282 |
| TMEM14C | -0.145511772 | 6.841540835 | -2.08136224 | 0.037963755 |
| CHIT1 | 0.508078881 | 0.252195266 | 2.08129468 | 0.037969966 |
| FBLN1 | 0.487755337 | -2.830448473 | 2.081137428 | 0.037984425 |
| RP5-882O7.1 | 0.176589968 | 1.283561071 | 2.081036814 | 0.037993678 |
| RPL37P23 | -0.11429282 | 8.288723981 | -2.080500096 | 0.038043075 |
| RP1-228P16.1 | 0.193938506 | 1.03556556 | 2.080384429 | 0.038053727 |
| AMIGO1 | -0.261741368 | 1.145558355 | -2.080009784 | 0.038088248 |
| RP11-175B9.2 | 0.177625021 | -0.385155325 | 2.07935836 | 0.038148335 |
| AKR7A2 | -0.14215533 | 6.24525034 | -2.079158156 | 0.038166818 |
| YARS | 0.084558655 | 6.026201286 | 2.079120242 | 0.038170319 |
| CPA3 | -0.592095314 | 6.189298385 | -2.078796151 | 0.038200258 |
| METTL24 | -0.27880495 | -2.20952725 | -2.078768448 | 0.038202818 |
| DDX55 | -0.074633256 | 4.690732383 | -2.078548812 | 0.03822312 |
| AC022400.1 | -0.199000722 | 1.351269802 | -2.078481397 | 0.038229354 |
| CTB-50E14.5 | -0.125050306 | 6.322570365 | -2.078386381 | 0.038238141 |
| CYP46A1 | 0.432544146 | -2.452843873 | 2.078124153 | 0.0382624 |
| MROH7 | -0.310467016 | -0.311198217 | -2.07706087 | 0.038360901 |
| RLN3 | -0.349755745 | -1.0140698 | -2.076940766 | 0.038372041 |
| RP11-138I1.2 | -0.232440094 | -1.760859416 | -2.076614287 | 0.038402337 |
| TRAV2 | -0.26727025 | -0.319797987 | -2.07660565 | 0.038403139 |
| LMF1 | -0.102340611 | 2.684291629 | -2.07650368 | 0.038412605 |
| CEACAM3 | 0.48660663 | 2.295712047 | 2.076162618 | 0.038444283 |
| LINC01146 | -0.30577547 | -1.44427013 | -2.075621126 | 0.038494622 |
| SSR4P1 | -0.220469801 | 0.230811817 | -2.075149614 | 0.038538502 |
| 2-Mar | 0.130357569 | 5.933849381 | 2.074676597 | 0.038582564 |
| TGM5 | 0.478213786 | 0.014008014 | 2.074362242 | 0.038611871 |
| RP11-556K13.1 | 0.121961555 | 7.134936728 | 2.074205577 | 0.038626484 |
| CPE | 0.379234876 | -1.961435729 | 2.073864454 | 0.038658317 |
| OXA1L | 0.084692767 | 8.06669923 | 2.073747152 | 0.038669269 |
| CD177P1 | 0.508986364 | -0.219480263 | 2.073580095 | 0.038684871 |
| RBM25 | 0.080708082 | 7.098986294 | 2.073544422 | 0.038688203 |
| AP001065.15 | -0.350046242 | -1.664470453 | -2.073433011 | 0.038698612 |
| IGKV1D-39 | 0.399552865 | 2.103146115 | 2.073353251 | 0.038706065 |
| ZNHIT3 | 0.083826432 | 3.567793344 | 2.073075222 | 0.038732055 |
| AGA | -0.098688518 | 5.236789193 | -2.073060768 | 0.038733406 |
| AKT2 | 0.062217379 | 5.779068458 | 2.072304195 | 0.038804207 |
| SLC6A6 | 0.152902059 | 6.608784464 | 2.07221312 | 0.038812737 |
| SLC9B2 | 0.160146293 | 3.906821564 | 2.071556324 | 0.038874302 |
| CD84 | -0.178696692 | 6.131890631 | -2.071337771 | 0.038894806 |
| DSC3 | -0.299974908 | -4.784474003 | -2.07123126 | 0.038904802 |
| GPR155 | -0.265448909 | 1.437512457 | -2.071167771 | 0.038910762 |
| TXNP6 | -0.119848263 | 5.511137661 | -2.070739334 | 0.038950998 |
| TMEM116 | -0.14370141 | 1.622251087 | -2.070201373 | 0.03900157 |
| CH25H | 0.467795464 | -0.095218617 | 2.070133691 | 0.039007937 |
| RADIL | 0.399155405 | -1.431453981 | 2.070065937 | 0.039014311 |
| KIFAP3 | -0.121793943 | 3.82444684 | -2.070030406 | 0.039017654 |
| RP11-706O15.3 | -0.490396333 | 0.896753474 | -2.070011609 | 0.039019423 |
| CSF2RA | -0.245924404 | 5.157593332 | -2.069704883 | 0.039048294 |
| GYPB | 0.550313571 | 0.639266866 | 2.069050959 | 0.039109905 |
| ADAMTS18 | -0.450748401 | -4.068701273 | -2.068446213 | 0.039166958 |
| RP11-15B17.1 | 0.239435675 | -0.748167912 | 2.068302955 | 0.039180483 |
| RP11-773H22.2 | -0.219251435 | 0.864415324 | -2.068268267 | 0.039183759 |
| MME | 0.560427273 | 0.93295144 | 2.068038528 | 0.039205459 |
| INTS1 | -0.080558466 | 6.347322978 | -2.067922782 | 0.039216396 |
| PUSL1 | -0.093858939 | 3.650521597 | -2.067788516 | 0.039229086 |
| ZNF496 | 0.168827683 | 5.177093742 | 2.067720075 | 0.039235556 |
| RP11-465L10.10 | 0.280960079 | -2.110013897 | 2.067478291 | 0.03925842 |
| RP11-16E12.1 | 0.178410789 | 2.118919664 | 2.067063796 | 0.039297642 |
| RN7SL192P | 0.197463912 | 0.598179134 | 2.066739614 | 0.039328342 |
| RBM20 | 0.328195681 | -3.070016905 | 2.06650956 | 0.03935014 |
| MYH9 | -0.092521675 | 9.912406787 | -2.065989963 | 0.039399411 |
| RP13-996F3.3 | 0.247345993 | -1.552674334 | 2.065958129 | 0.039402432 |
| C14orf93 | -0.078078329 | 4.412859932 | -2.065934173 | 0.039404705 |
| RAD54B | 0.182271493 | 0.321690359 | 2.06585785 | 0.039411947 |
| ATP6V1E1 | -0.080622547 | 6.450065019 | -2.065238776 | 0.039470736 |
| ARF1P2 | 0.256084987 | -0.186163811 | 2.06472561 | 0.039519524 |
| RPUSD1 | -0.068320448 | 5.727083792 | -2.063812144 | 0.039606497 |
| GABRR2 | -0.243022605 | 1.708789759 | -2.063211057 | 0.039663816 |
| SNX25P1 | 0.281855513 | 0.551419838 | 2.063120256 | 0.039672481 |
| C9orf129 | -0.15224483 | 1.361421052 | -2.063077826 | 0.03967653 |
| GPD1 | 0.200678459 | -1.655811864 | 2.06291825 | 0.039691763 |
| AC074138.3 | -0.154848611 | 2.871773707 | -2.062250799 | 0.039755532 |
| AC087380.14 | -0.252656299 | 0.407745237 | -2.061656973 | 0.03981234 |
| FILIP1L | 0.367188548 | -0.143038297 | 2.061357544 | 0.039841011 |
| RP11-535M15.2 | 0.331387516 | 0.010815806 | 2.060413076 | 0.039931561 |
| TMEM184A | 0.35333739 | -2.385032947 | 2.060293339 | 0.039943053 |
| RP11-460N20.5 | -0.193741633 | 1.53532275 | -2.060279234 | 0.039944407 |
| RP11-175B9.3 | -0.127041533 | 8.887327049 | -2.060065151 | 0.039964962 |
| RP3-395M20.7 | 0.229718792 | 0.512001412 | 2.059527286 | 0.040016644 |
| DAP3 | -0.064519763 | 6.458487894 | -2.059377889 | 0.040031009 |
| PCDHGB7 | 0.403997189 | -0.303283353 | 2.059339095 | 0.04003474 |
| TRBV4-2 | -0.304965071 | 0.467818774 | -2.058875575 | 0.040079341 |
| TMED10 | -0.110019174 | 7.330773822 | -2.058362983 | 0.040128714 |
| B3GNTL1 | -0.130756133 | 4.879330305 | -2.05788023 | 0.040175259 |
| CUL1 | -0.126867451 | 6.372961651 | -2.057855227 | 0.040177671 |
| RP11-700P18.2 | -0.118519479 | 2.416263754 | -2.057770405 | 0.040185855 |
| CIB3 | -0.27692752 | -0.883156251 | -2.057677974 | 0.040194774 |
| SNHG7 | 0.165345829 | 5.408906957 | 2.057602855 | 0.040202024 |
| CMTM2 | 0.388027608 | 2.909292366 | 2.057286408 | 0.040232577 |
| ALDH7A1 | -0.368004184 | 0.60142064 | -2.057153914 | 0.040245375 |
| P4HB | 0.160697484 | 9.246153146 | 2.056957641 | 0.04026434 |
| RPS27A | 0.130169805 | 7.909066712 | 2.056943435 | 0.040265713 |
| HNRNPUL2-BSCL2 | -0.182179148 | 0.509248964 | -2.056808707 | 0.040278737 |
| RPS18P12 | -0.140661997 | 8.920855047 | -2.056530677 | 0.040305623 |
| UBA5 | -0.073851553 | 4.776661461 | -2.056196488 | 0.040337961 |
| SFRP4 | 0.360059931 | -2.084307284 | 2.055991572 | 0.0403578 |
| HLA-J | 0.298846923 | 4.471307969 | 2.055645606 | 0.040391314 |
| RP11-523H20.3 | -0.271168093 | -0.815258331 | -2.055470581 | 0.040408278 |
| SUGT1P2 | 0.127685891 | 2.074565845 | 2.054508244 | 0.040501659 |
| RP11-543B16.2 | 0.153799847 | 3.8415567 | 2.054285728 | 0.040523277 |
| C12orf5 | 0.191036043 | 4.525284959 | 2.05421679 | 0.040529977 |
| ZNF608 | -0.429021702 | 2.10107887 | -2.054125219 | 0.040538877 |
| COMMD7 | -0.103448747 | 7.115960023 | -2.053953088 | 0.040555613 |
| ENO3 | 0.118321717 | 3.163552759 | 2.053947343 | 0.040556171 |
| TP53I13 | -0.109231043 | 5.537051899 | -2.053530368 | 0.040596736 |
| RNASE1 | 0.484170763 | 1.957871416 | 2.053442965 | 0.040605243 |
| SNHG3 | 0.156197534 | 6.311148151 | 2.053071823 | 0.040641385 |
| RP11-497H17.1 | 0.204848371 | 2.150501695 | 2.052771952 | 0.040670606 |
| NLGN1 | 0.442652651 | -4.639080735 | 2.052136015 | 0.040732634 |
| RAD21-AS1 | -0.217793022 | -0.523479832 | -2.051983957 | 0.040747478 |
| RP11-137H2.6 | 0.40215478 | 0.239895767 | 2.051651475 | 0.04077995 |
| LDB2 | 0.287124844 | -3.07915877 | 2.05157224 | 0.040787691 |
| CISD3 | -0.116049739 | 4.208636153 | -2.051232096 | 0.04082094 |
| LINC01036 | 0.287815434 | -1.056920647 | 2.050890378 | 0.040854365 |
| AC103563.8 | -0.253996678 | -1.086693001 | -2.050616474 | 0.040881174 |
| SSBP4 | -0.097592643 | 5.998197823 | -2.050427495 | 0.04089968 |
| TRBV30 | -0.289868828 | -0.230720234 | -2.050405715 | 0.040901813 |
| AC000095.9 | 0.243985444 | -0.819015793 | 2.0495036 | 0.040990253 |
| SLIRP | 0.080148822 | 4.657427323 | 2.049488919 | 0.040991694 |
| LPHN2 | 0.502970752 | -2.755078388 | 2.04919474 | 0.04102057 |
| GLIS2 | 0.319701074 | -1.528777263 | 2.04901947 | 0.041037783 |
| RP11-334A14.2 | 0.30088083 | 2.153908596 | 2.048906211 | 0.041048909 |
| RP11-299H22.5 | -0.292110862 | -2.438275327 | -2.048893772 | 0.041050131 |
| SEC16A | 0.069341134 | 6.215326853 | 2.048726133 | 0.041066604 |
| DNM3 | 0.290012642 | 0.177812305 | 2.048422976 | 0.041096408 |
| CFLAR | -0.095751446 | 7.03059341 | -2.04833888 | 0.041104679 |
| EFNA1 | 0.391091802 | 1.090236431 | 2.047919156 | 0.04114598 |
| RP11-10H3.1 | -0.529993414 | -1.28046406 | -2.047667705 | 0.04117074 |
| L3MBTL3 | 0.154869752 | 4.337668699 | 2.047516597 | 0.041185625 |
| RNU6-8 | 0.215655144 | 1.10964276 | 2.04741515 | 0.041195621 |
| GRIK4 | 0.288584219 | -4.127387725 | 2.047405617 | 0.041196561 |
| TNFSF14 | -0.351635327 | 1.744057224 | -2.047361724 | 0.041200887 |
| AC010522.1 | -0.138992436 | 3.753655159 | -2.046407135 | 0.041295057 |
| IFT43 | -0.116790914 | 2.056238859 | -2.046375616 | 0.04129817 |
| DNAJA2 | 0.066748452 | 6.135679461 | 2.046348394 | 0.041300858 |
| RP11-690G19.3 | 0.113071372 | 5.304916894 | 2.045730534 | 0.041361915 |
| PTBP1 | 0.097008032 | 7.758974452 | 2.045651077 | 0.041369773 |
| ZNF277 | -0.118603167 | 4.847493274 | -2.045422247 | 0.041392409 |
| PTPN12 | 0.115586822 | 6.933415783 | 2.04541552 | 0.041393075 |
| RP11-677M14.2 | -0.266399921 | -0.462291171 | -2.045369777 | 0.041397601 |
| RP11-307E17.8 | -0.365888883 | -0.233580336 | -2.045047779 | 0.041429474 |
| FNDC3A | -0.083785097 | 5.834379669 | -2.044632623 | 0.0414706 |
| RPL7AP28 | -0.432194243 | -1.340325313 | -2.044376794 | 0.04149596 |
| BDH2 | 0.115586131 | 3.019263289 | 2.044041193 | 0.041529248 |
| RP11-64B16.2 | -0.082501277 | 4.772312782 | -2.043965657 | 0.041536743 |
| SLC31A1 | 0.170744981 | 4.466755571 | 2.043632384 | 0.041569828 |
| ARHGAP10 | 0.303629158 | 1.957368803 | 2.043492576 | 0.041583713 |
| HIST1H2AE | -0.347163577 | 4.335723191 | -2.043056234 | 0.041627075 |
| TNF | 0.386776662 | 4.426438561 | 2.042820119 | 0.041650556 |
| RNFT2 | 0.327958249 | -0.677989607 | 2.04278057 | 0.04165449 |
| FKBP11 | 0.122661051 | 4.222737481 | 2.042740313 | 0.041658494 |
| RP11-89K10.1 | 0.243135084 | -0.83196689 | 2.042484567 | 0.041683944 |
| RPTOR | -0.097684204 | 4.458973177 | -2.042431253 | 0.041689251 |
| U73166.2 | 0.221924524 | 1.186748286 | 2.041704215 | 0.041761678 |
| AL391994.1 | 0.255789692 | -0.481561374 | 2.041495595 | 0.041782481 |
| KIF21A | 0.324263359 | 0.082293502 | 2.041235185 | 0.04180846 |
| AL049758.2 | -0.21592946 | -0.149635943 | -2.040945256 | 0.0418374 |
| RASAL2-AS1 | -0.290562907 | -1.924714551 | -2.040886301 | 0.041843287 |
| PAF1 | -0.070987853 | 6.79438546 | -2.040851711 | 0.041846741 |
| RP11-219G10.1 | 0.262790156 | 3.044121187 | 2.040693489 | 0.041862544 |
| MDS2 | -0.292831097 | -1.75486224 | -2.040620479 | 0.041869838 |
| HNRNPA3P11 | 0.206041442 | 0.669672183 | 2.039191758 | 0.042012793 |
| GAK | -0.058873347 | 5.786002243 | -2.038891574 | 0.042042881 |
| TMEM134 | -0.092004176 | 4.410660583 | -2.03844674 | 0.042087502 |
| UBXN1 | 0.077686939 | 7.626433061 | 2.038081314 | 0.042124187 |
| FBLN2 | 0.444804643 | -0.004312506 | 2.037741066 | 0.042158369 |
| RABL3 | 0.084114019 | 4.28238459 | 2.037040151 | 0.042228859 |
| SLC14A2 | -0.262535495 | -1.89200038 | -2.036918221 | 0.042241131 |
| C2CD2 | -0.167226987 | 3.528742786 | -2.036426949 | 0.04229061 |
| RP11-476D10.1 | -0.318337029 | -1.46306944 | -2.036107877 | 0.042322771 |
| HLA-V | 0.43033846 | -2.970915791 | 2.036083537 | 0.042325226 |
| C1orf53 | -0.295207555 | 0.01799418 | -2.036039715 | 0.042329644 |
| CHRNB1 | -0.158640215 | 2.982890721 | -2.035817352 | 0.042352073 |
| RPL18P13 | 0.188872394 | 1.68931823 | 2.035701319 | 0.042363781 |
| ZDHHC9 | -0.142730878 | 4.319453804 | -2.035654179 | 0.042368538 |
| BEST1 | 0.254832792 | 2.270878305 | 2.035570071 | 0.042377028 |
| GLT1D1 | 0.504150852 | 3.547521611 | 2.035506284 | 0.042383467 |
| PCDHB11 | 0.34424472 | -3.179910336 | 2.035116547 | 0.042422827 |
| RP11-1006G14.4 | -0.124655841 | 2.287440802 | -2.034800158 | 0.042454803 |
| RP11-334C17.5 | -0.32681239 | 1.773775161 | -2.034309561 | 0.042504425 |
| MED8 | -0.095074278 | 5.318025977 | -2.033189049 | 0.042617945 |
| SERINC4 | 0.186321072 | -1.638875653 | 2.032294695 | 0.042708738 |
| RP11-65N13.6 | 0.198598045 | 1.075602149 | 2.031848447 | 0.042754101 |
| EFCAB5 | -0.19253678 | -0.038037606 | -2.03146331 | 0.042793285 |
| AC015987.2 | 0.278850391 | -1.324663188 | 2.031149233 | 0.042825262 |
| LRRC37A7P | -0.306290392 | -2.657385563 | -2.031085296 | 0.042831774 |
| ATP5L | 0.079390666 | 7.74310031 | 2.030990083 | 0.042841473 |
| NOP58 | 0.093374671 | 7.042160542 | 2.030699548 | 0.042871081 |
| ATAD3C | 0.216305665 | 0.517300074 | 2.030617652 | 0.04287943 |
| RP11-347C12.1 | -0.209198463 | -1.524862195 | -2.030596194 | 0.042881618 |
| SKP2 | -0.102712253 | 4.623238471 | -2.030372492 | 0.042904431 |
| RP11-259P15.4 | -0.169469537 | 2.012383597 | -2.029508388 | 0.04299265 |
| HMGN1P4 | 0.111009316 | 4.431222019 | 2.029428814 | 0.043000782 |
| CDCP1 | 0.406266274 | 1.741006804 | 2.029343464 | 0.043009505 |
| RP11-379F4.9 | -0.18851428 | 1.342080202 | -2.029295882 | 0.043014369 |
| POLRMTP1 | -0.083162546 | 3.587968101 | -2.028479695 | 0.043097873 |
| TC2N | 0.337650177 | 3.411408895 | 2.027763173 | 0.043171293 |
| NAALADL2 | -0.416303044 | -1.390870174 | -2.02761324 | 0.04318667 |
| HDAC2 | -0.098654189 | 5.488073391 | -2.027157861 | 0.043233401 |
| RP11-160H22.3 | -0.227646413 | 0.784511199 | -2.027150902 | 0.043234116 |
| DDX11 | 0.150564177 | 4.43965851 | 2.027106311 | 0.043238694 |
| TRBV20-1 | -0.295359447 | 3.787979294 | -2.027053158 | 0.043244152 |
| ULBP1 | 0.328897823 | -1.216163064 | 2.026007989 | 0.043351592 |
| TUT1 | -0.106595204 | 2.683575763 | -2.025778308 | 0.043375233 |
| NACC1 | 0.072417935 | 6.012680029 | 2.024969511 | 0.043458568 |
| LINC00999 | -0.128465845 | 2.655810605 | -2.024836133 | 0.043472324 |
| RP4-584D14.5 | 0.224389502 | -0.520349384 | 2.024793676 | 0.043476703 |
| TRGV4 | -0.327659668 | 1.132559522 | -2.024039104 | 0.043554601 |
| SELENBP1 | 0.592142249 | 1.042160898 | 2.02392019 | 0.043566888 |
| MBD6 | 0.09914278 | 6.833844331 | 2.023754701 | 0.043583992 |
| HCG4 | -0.344185038 | -1.418567928 | -2.023634303 | 0.04359644 |
| PSMA6P2 | -0.141414439 | 1.964054551 | -2.023504498 | 0.043609863 |
| BATF | -0.172361276 | 5.177708128 | -2.023326802 | 0.043628244 |
| GOLGA6L3 | 0.280304682 | 0.427705645 | 2.023225329 | 0.043638744 |
| AC008088.4 | -0.245195235 | -0.754947967 | -2.022720444 | 0.043691017 |
| RP11-497G19.1 | -0.710902156 | 3.655367596 | -2.022160078 | 0.043749096 |
| HN1 | -0.095087995 | 6.250140673 | -2.021858024 | 0.04378043 |
| EPB41 | -0.117650585 | 6.782610847 | -2.021696696 | 0.043797173 |
| STARD3 | 0.068275345 | 5.497523039 | 2.021679727 | 0.043798935 |
| TFAM | 0.069623453 | 5.433621424 | 2.021602569 | 0.043806945 |
| ANKRD10-IT1 | -0.138606239 | 5.280222139 | -2.021535942 | 0.043813862 |
| POMP | 0.088477144 | 7.109574253 | 2.020547335 | 0.043916615 |
| SATB1 | -0.132473473 | 6.503174199 | -2.020216668 | 0.04395103 |
| INPP5E | -0.087126348 | 4.879642656 | -2.019314589 | 0.044045029 |
| EFHD2 | 0.139398926 | 9.037048058 | 2.018896554 | 0.044088648 |
| CTD-2301A4.3 | 0.224820213 | 1.039414211 | 2.018860022 | 0.044092461 |
| TP53BP1 | -0.12531433 | 5.254423319 | -2.018758597 | 0.04410305 |
| MS4A1 | -0.466157306 | 2.455820169 | -2.018152155 | 0.04416641 |
| ZZZ3 | -0.066811831 | 4.888011779 | -2.018143521 | 0.044167313 |
| EPCAM | 0.506658855 | 0.595304268 | 2.017954128 | 0.044187116 |
| MAK16 | 0.09432371 | 4.282511455 | 2.017818774 | 0.044201274 |
| GAREML | 0.241288765 | 0.120034426 | 2.017652333 | 0.044218689 |
| PLLP | 0.256750204 | -1.643056114 | 2.017220313 | 0.044263917 |
| SAMD3 | -0.29233276 | 1.444820655 | -2.017179089 | 0.044268235 |
| ALDH9A1 | -0.095581737 | 6.021867237 | -2.01687517 | 0.044300079 |
| TUFMP1 | 0.145334577 | 1.321095047 | 2.016852011 | 0.044302506 |
| RPS27AP1 | 0.160695849 | 2.507795944 | 2.016718824 | 0.044316468 |
| TFCP2L1 | 0.408662134 | -1.097325671 | 2.01667947 | 0.044320594 |
| RN7SL130P | 0.210461019 | -0.125985519 | 2.015963717 | 0.044395695 |
| RP4-639F20.1 | 0.290225334 | -1.287219036 | 2.01569319 | 0.044424108 |
| RP11-455O6.2 | -0.396284396 | -0.72298166 | -2.015610958 | 0.044432748 |
| AC108004.3 | -0.335021701 | 3.96592337 | -2.015582428 | 0.044435746 |
| LMBRD1 | -0.093428098 | 6.315569042 | -2.015043246 | 0.044492434 |
| RP11-327P2.5 | -0.151822648 | 2.481701637 | -2.014986365 | 0.044498418 |
| FAM19A2 | -0.22557666 | 1.687909288 | -2.014507128 | 0.044548861 |
| PHACTR3 | -0.615091062 | -1.558831265 | -2.01436531 | 0.044563797 |
| SYT15 | -0.333641362 | -2.440239707 | -2.014070521 | 0.044594859 |
| STAT3 | 0.089249209 | 7.29095066 | 2.013756853 | 0.044627929 |
| ARRDC3 | 0.241699446 | 7.395061411 | 2.01369519 | 0.044634433 |
| RP11-296I10.6 | -0.111636408 | 2.776901069 | -2.012348852 | 0.044776634 |
| RP11-366M4.1 | 0.188747953 | 0.685038063 | 2.012206012 | 0.044791744 |
| LRRC3DN | -0.183724675 | 1.80531906 | -2.012154758 | 0.044797166 |
| EVI2B | -0.211452078 | 9.392074777 | -2.012096686 | 0.044803311 |
| RP11-192C21.1 | 0.204708771 | 0.213733932 | 2.012032982 | 0.044810052 |
| RP11-384C4.2 | 0.20780616 | 0.684549622 | 2.011670294 | 0.044848449 |
| RPS5 | 0.1349091 | 9.003463304 | 2.011528409 | 0.044863478 |
| RP11-318K12.3 | 0.248122976 | -0.003806591 | 2.011473785 | 0.044869265 |
| KALRN | 0.346364637 | -1.481396865 | 2.010954763 | 0.044924282 |
| MGMT | 0.247856069 | 3.6805846 | 2.010507745 | 0.044971713 |
| BORA | -0.1042131 | 4.355917659 | -2.010204423 | 0.045003922 |
| RP11-256L6.3 | -0.351822031 | 3.072737663 | -2.010156926 | 0.045008967 |
| RP11-321A17.3 | 0.118255699 | 4.481532316 | 2.01007792 | 0.04501736 |
| RPS8 | 0.098721369 | 10.33349936 | 2.009927691 | 0.045033323 |
| hsa-mir-1199 | 0.22647902 | 1.722954277 | 2.009900547 | 0.045036208 |
| HSPB11 | 0.063754705 | 4.847448252 | 2.009887045 | 0.045037643 |
| MDH2 | -0.084170894 | 7.389716224 | -2.009748304 | 0.045052391 |
| AC006547.14 | -0.417677263 | 0.951766685 | -2.009031012 | 0.045128704 |
| SCNN1B | 0.246510867 | -3.608610132 | 2.008959302 | 0.045136339 |
| NFRKB | 0.090518942 | 5.497170706 | 2.00888139 | 0.045144636 |
| SLC2A8 | 0.261340991 | 1.446372618 | 2.008877605 | 0.045145039 |
| EEF1A1P4 | 0.15450732 | 4.663406567 | 2.00854265 | 0.045180723 |
| RPL5P22 | 0.165814897 | 3.7991816 | 2.008305837 | 0.045205966 |
| RARRES2 | 0.398371124 | -1.375988176 | 2.008237134 | 0.045213292 |
| NETO2 | -0.312388058 | 2.902133657 | -2.008060736 | 0.045232105 |
| GNAI2 | -0.086125039 | 8.602296743 | -2.007715199 | 0.045268977 |
| RP11-864G5.1 | 0.17041799 | 3.998496466 | 2.006903735 | 0.045355668 |
| MLLT10 | -0.097207522 | 6.154082688 | -2.006884575 | 0.045357716 |
| XXbac-BPG181B23.7 | -0.227660842 | 3.811821575 | -2.006666687 | 0.045381018 |
| SOGA2 | -0.373048575 | -0.10678425 | -2.00653361 | 0.045395255 |
| RBPMS-AS1 | -0.385014164 | -0.563324246 | -2.006511636 | 0.045397607 |
| CTD-2537I9.5 | -0.193498858 | 0.017544456 | -2.006261364 | 0.045424393 |
| PPIAL4G | -0.115094227 | 5.99999714 | -2.006065335 | 0.045445383 |
| PRR16 | -0.464613851 | -0.415793391 | -2.005998441 | 0.045452548 |
| LEF1 | 0.316979564 | 3.05512632 | 2.005629723 | 0.045492056 |
| TLCD2 | -0.245165113 | -0.959052401 | -2.005245119 | 0.045533298 |
| ZBTB14 | -0.07895375 | 4.865826437 | -2.005219617 | 0.045536034 |
| SGOL2 | -0.171854789 | 3.826956973 | -2.004756382 | 0.045585752 |
| RPSAP58 | 0.140636377 | 8.970429491 | 2.004733632 | 0.045588195 |
| RP11-169E6.1 | -0.205076014 | 0.527789483 | -2.003890832 | 0.045678772 |
| UBR2 | -0.070410238 | 5.789798511 | -2.003860654 | 0.045682018 |
| PPAT | -0.118875264 | 3.820863856 | -2.003654039 | 0.045704248 |
| SLC35F3 | -0.333884404 | -2.017972705 | -2.002854051 | 0.045790406 |
| ABHD4 | -0.168864147 | 5.438860578 | -2.002770544 | 0.045799407 |
| CTD-2323K18.1 | 0.150688403 | 1.065286239 | 2.001628966 | 0.045922612 |
| RP11-778D9.4 | -0.132248869 | 9.148809117 | -2.001625365 | 0.045923001 |
| RAB43P1 | 0.227534682 | 3.555588934 | 2.001470336 | 0.045939754 |
| EBF4 | 0.337864877 | -0.172129914 | 2.001369192 | 0.045950687 |
| CCDC112 | -0.203725217 | 4.160929525 | -2.000732605 | 0.046019548 |
| NPAS2 | 0.270131579 | -3.378669684 | 2.000710915 | 0.046021896 |
| NBEAL2 | -0.133493468 | 7.655264391 | -2.000709099 | 0.046022092 |
| HOXA11 | -0.687012612 | -0.603200164 | -2.00063806 | 0.046029782 |
| RP11-307C18.1 | 0.262218064 | -0.020852337 | 2.000372838 | 0.046058503 |
| GSTM5 | 0.486019881 | -0.811878117 | 2.000305857 | 0.046065759 |
| TNPO3 | -0.078363887 | 6.40253597 | -1.999660443 | 0.046135722 |
| RP11-104L21.2 | 0.287691 | -0.421551933 | 1.997411718 | 0.046380189 |
| RRP7A | 0.08894125 | 5.451262811 | 1.996953451 | 0.046430143 |
| JTB | -0.077806786 | 7.874860579 | -1.996692697 | 0.046458587 |
| ZMYND8 | -0.136786158 | 5.36927926 | -1.996358107 | 0.046495107 |
| ZC3HAV1L | -0.233871119 | 3.264043091 | -1.995761014 | 0.046560339 |
| PRRT4 | 0.428252502 | 3.293303215 | 1.99557456 | 0.046580725 |
| TAS1R3 | 0.215397266 | 0.157008117 | 1.994915198 | 0.046652876 |
| ABCB6 | 0.232134575 | -0.48873538 | 1.994683915 | 0.046678206 |
| DLL3 | -0.333958945 | -2.403649647 | -1.994650903 | 0.046681823 |
| RP11-367G6.3 | 0.256627331 | 5.4951507 | 1.994633878 | 0.046683688 |
| RPS16 | 0.107417308 | 9.252038476 | 1.994430962 | 0.046705923 |
| FAM86HP | -0.204205356 | 2.278736067 | -1.993946663 | 0.046759029 |
| RP11-434C1.3 | -0.240699725 | -1.181948667 | -1.993655993 | 0.046790927 |
| ARHGEF10L | -0.566088484 | 0.929444122 | -1.993269588 | 0.046833359 |
| CYBA | -0.120892072 | 8.680666785 | -1.993097318 | 0.046852287 |
| RP11-390E23.6 | 0.143843478 | 2.90870578 | 1.993055882 | 0.046856841 |
| MTRNR2L1 | 0.203453135 | 3.47818098 | 1.993001729 | 0.046862792 |
| MRPS35 | -0.073329304 | 6.763547851 | -1.992950965 | 0.046868372 |
| AKR1C3 | 0.277100356 | 3.94551007 | 1.992378663 | 0.046931318 |
| EEF1A1P8 | 0.190882341 | 1.214485921 | 1.992353193 | 0.046934121 |
| NKAP | 0.06573122 | 4.243120854 | 1.992077215 | 0.046964502 |
| PPM1M | -0.17506086 | 5.807326059 | -1.991759273 | 0.046999523 |
| CTC-308K20.2 | -0.247360848 | 0.205341572 | -1.991607874 | 0.047016207 |
| CYP2C8 | -0.354019551 | -0.167400477 | -1.991604412 | 0.047016588 |
| APITD1 | 0.162131018 | 1.299165646 | 1.991524012 | 0.047025451 |
| RP1-97D16.1 | -0.232666163 | -1.178268727 | -1.991470734 | 0.047031324 |
| KISS1R | -0.277025555 | -1.214269767 | -1.991441075 | 0.047034594 |
| MAPRE3 | -0.244091551 | 1.73547993 | -1.991377567 | 0.047041596 |
| HIST1H3C | -0.340189965 | 0.756732533 | -1.991327255 | 0.047047144 |
| AL592188.7 | 0.234303556 | 3.281338072 | 1.99122885 | 0.047057997 |
| GALNT18 | 0.353528394 | -2.507690253 | 1.990965298 | 0.047087074 |
| MOK | 0.273599562 | 1.731058185 | 1.990893064 | 0.047095046 |
| RP11-146D12.2 | 0.263294515 | -2.115001472 | 1.990809906 | 0.047104225 |
| PGRMC2 | 0.083716879 | 5.161947602 | 1.990230688 | 0.047168202 |
| RP11-474C8.4 | -0.220389521 | -0.686155083 | -1.990088354 | 0.047183935 |
| HBBP1 | 0.421413957 | -0.222761092 | 1.989962807 | 0.047197816 |
| CCDC85B | -0.104495997 | 7.409040525 | -1.989935084 | 0.047200881 |
| TREML2 | 0.2307802 | 4.79850338 | 1.989923309 | 0.047202183 |
| MLTK | 0.262277184 | 4.224949322 | 1.989772397 | 0.047218874 |
| GPM6A | -0.290201629 | -3.718794001 | -1.989653122 | 0.04723207 |
| KIAA1432 | 0.087560427 | 5.443156693 | 1.989545237 | 0.047244008 |
| CTD-2532N20.1 | -0.215135551 | 0.584993457 | -1.989221667 | 0.047279828 |
| AJ006998.2 | 0.560558213 | 1.015330991 | 1.988842141 | 0.047321872 |
| AC012442.5 | -0.191077695 | -0.02471375 | -1.988579435 | 0.047350992 |
| HMGB1P5 | 0.106898782 | 9.096548315 | 1.987896095 | 0.047426811 |
| UNC5CL | 0.155528206 | 2.183433654 | 1.987800414 | 0.047437436 |
| RP11-545E17.3 | -0.167378852 | 2.176916132 | -1.987646337 | 0.047454548 |
| FAM65A | 0.119635005 | 5.639342757 | 1.987528654 | 0.047467623 |
| SAPCD2 | 0.228655165 | 3.066097683 | 1.987307076 | 0.047492247 |
| TMSB4XP8 | -0.154448882 | 10.17023454 | -1.987288494 | 0.047494313 |
| IQSEC3 | 0.457669258 | 1.537154532 | 1.987253132 | 0.047498244 |
| C15orf62 | 0.22630555 | 0.235565512 | 1.98699364 | 0.047527099 |
| ZBTB26 | 0.130287444 | 4.39069039 | 1.98667468 | 0.047562587 |
| SALL4 | 0.449324027 | -0.454355151 | 1.986543797 | 0.047577155 |
| HIST1H3J | -0.256590986 | -0.927658031 | -1.986509024 | 0.047581027 |
| ZBTB7B | -0.186264886 | 6.271802312 | -1.986432308 | 0.047589568 |
| KIF9 | 0.14606182 | 2.796941728 | 1.986297852 | 0.047604542 |
| GOLGA8R | -0.192980283 | 4.195285348 | -1.986244789 | 0.047610452 |
| CERS3 | -0.230419006 | -3.158499049 | -1.985948841 | 0.047643428 |
| LPAR6 | -0.421943714 | 4.19638461 | -1.985661343 | 0.04767548 |
| RP11-685M7.3 | -0.213782301 | -0.442920526 | -1.9855191 | 0.047691345 |
| GABPB1-AS1 | -0.170571473 | 5.318306814 | -1.98532183 | 0.047713355 |
| CDH23 | -0.252568839 | 1.956365731 | -1.985027782 | 0.047746179 |
| ANKHD1-EIF4EBP3 | 0.172479809 | 0.833372585 | 1.984297787 | 0.047827747 |
| ST7L | -0.091922719 | 3.42920139 | -1.983377377 | 0.047930761 |
| PMEL | -0.197498798 | -0.636057226 | -1.983361829 | 0.047932502 |
| HPS6 | 0.112346487 | 4.979817787 | 1.983304796 | 0.047938892 |
| EVC | 0.491879117 | -1.208980746 | 1.983201126 | 0.047950508 |
| UQCRC1 | -0.076514852 | 7.136487531 | -1.982823625 | 0.047992827 |
| NEURL1B | -0.371234697 | 2.474870964 | -1.982371157 | 0.048043592 |
| C9orf72 | 0.203768124 | 5.552583627 | 1.982125028 | 0.048071225 |
| KLRD1 | -0.308862259 | 1.312193588 | -1.982081792 | 0.048076081 |
| CLEC4D | 0.487654858 | 3.460919574 | 1.98175496 | 0.048112799 |
| MRGBP | 0.073402402 | 5.598066644 | 1.981078125 | 0.048188913 |
| ZFY | 0.826600456 | 0.837085665 | 1.981040523 | 0.048193144 |
| OTUD7B | 0.264152111 | 0.461356885 | 1.980873538 | 0.04821194 |
| ITK | 0.296061458 | 2.808622629 | 1.980518407 | 0.048251933 |
| KRT81 | 0.30195146 | -2.222522751 | 1.980296326 | 0.048276957 |
| RORB | 0.421441731 | -3.848078543 | 1.979740744 | 0.048339608 |
| RP11-546B8.5 | 0.116570667 | 3.817748523 | 1.979640065 | 0.048350968 |
| TNFRSF11A | 0.362371657 | 0.41712732 | 1.979544604 | 0.048361742 |
| HIST1H2AG | -0.23017938 | 3.258902588 | -1.97929523 | 0.048389896 |
| AVEN | -0.142734593 | 4.519618955 | -1.979159795 | 0.048405192 |
| ZNF436 | 0.101143035 | 3.697415943 | 1.979135801 | 0.048407902 |
| RP11-44N11.2 | -0.241903375 | -0.951088874 | -1.97895778 | 0.048428016 |
| POLR1B | -0.105390745 | 4.146085044 | -1.978280915 | 0.048504553 |
| MROH6 | -0.161551709 | 5.379464728 | -1.977988828 | 0.048537613 |
| AQP3 | -0.266434166 | 3.992130146 | -1.977707311 | 0.048569494 |
| CXCL2 | 0.400035529 | 5.577981698 | 1.977135378 | 0.048634319 |
| RP11-815I9.4 | 0.1954012 | 1.651876604 | 1.976498585 | 0.04870658 |
| RP3-467L1.4 | -0.339359406 | -1.180958762 | -1.976300031 | 0.04872913 |
| KRT18P38 | -0.322916451 | -1.472925028 | -1.975917615 | 0.048772586 |
| RP11-119F7.5 | -0.269237211 | 1.046291359 | -1.975855614 | 0.048779635 |
| RBM12 | -0.077547734 | 6.014010177 | -1.975782556 | 0.048787942 |
| TSNAX | -0.094132653 | 5.010950876 | -1.975596417 | 0.048809111 |
| CTC-278L1.1 | -0.219294079 | -0.172369313 | -1.975402737 | 0.048831146 |
| RP11-28F1.2 | 0.232989523 | 1.06006181 | 1.975261363 | 0.048847236 |
| TSC22D1 | 0.224693517 | 6.197732026 | 1.974985585 | 0.048878635 |
| SLC10A7 | 0.078579267 | 3.669309552 | 1.97413268 | 0.048975851 |
| RP11-573D15.9 | 0.210302537 | -0.345625812 | 1.974102471 | 0.048979297 |
| CD4 | -0.304870608 | 6.433450707 | -1.973918911 | 0.049000242 |
| SAMD11 | -0.436439574 | 0.503178951 | -1.973896308 | 0.049002822 |
| AMMECR1L | -0.070790063 | 5.282514236 | -1.97349598 | 0.049048529 |
| HNRNPCL1 | 0.170081111 | 0.624749653 | 1.973113222 | 0.049092264 |
| CC2D1B | -0.06597465 | 4.886504468 | -1.972772312 | 0.049131245 |
| CDKN1A | 0.299973724 | 6.768610892 | 1.972653956 | 0.049144784 |
| RN7SL617P | -0.221454574 | -0.034117731 | -1.972588951 | 0.049152222 |
| ACAP2-IT1 | -0.163727809 | 3.816120576 | -1.972534496 | 0.049158453 |
| RPL26P37 | -0.211337706 | 1.192080617 | -1.971681765 | 0.049256117 |
| CAB39L | -0.126487607 | 2.077370068 | -1.971304158 | 0.049299417 |
| GDF7 | 0.30248635 | -2.468808619 | 1.971202063 | 0.049311129 |
| AC018816.3 | 0.170932425 | 0.192313964 | 1.970865344 | 0.049349775 |
| CTD-2013N17.1 | 0.213408312 | 1.464180965 | 1.970733795 | 0.04936488 |
| CTD-3193O13.9 | 0.272082657 | -2.212143325 | 1.970454343 | 0.049396981 |
| MARK3 | -0.058976204 | 5.985782787 | -1.970152378 | 0.049431688 |
| RBFA | -0.118686743 | 4.304314742 | -1.969981658 | 0.049451319 |
| RP11-530N7.2 | -0.260609677 | -0.748757879 | -1.969543513 | 0.049501732 |
| MPZL2 | -0.457263735 | 0.259240521 | -1.969429686 | 0.049514835 |
| TOX | 0.277869975 | 4.46525726 | 1.969111834 | 0.049551442 |
| CPT1B | -0.192937896 | 1.078941201 | -1.968522564 | 0.049619368 |
| ERP44 | -0.083303118 | 6.730021991 | -1.96836655 | 0.049637365 |
| RP11-1379J22.5 | -0.194985967 | 0.147121053 | -1.967663559 | 0.049718527 |
| KLRF1 | -0.333998055 | 3.076287861 | -1.966901882 | 0.04980659 |
| ZNF705EP | 0.196427116 | -1.317602822 | 1.966882285 | 0.049808858 |
| COL24A1 | -0.315690392 | 3.855648214 | -1.966837443 | 0.049814047 |
| SMURF2P1 | -0.138912848 | 2.510368711 | -1.966348086 | 0.049870701 |
| RP11-104L21.3 | 0.311628224 | 0.137774452 | 1.966253162 | 0.049881697 |
| AC004466.1 | -0.163599899 | 1.503343545 | -1.965811842 | 0.049932846 |
| ISOC2 | -0.171676248 | 3.855152432 | -1.965782157 | 0.049936289 |
| RP11-174G6.5 | 0.203861401 | 1.535500525 | 1.965680786 | 0.049948044 |
| RNF111 | -0.083329855 | 4.888623324 | -1.965283631 | 0.049994124 |
| RP11-422P24.9 | -0.134492191 | 6.023515658 | -1.965271965 | 0.049995479 |
| RP11-533E19.7 | -0.162836765 | 1.938165783 | -1.965163695 | 0.050008047 |
| SLC18A2 | 0.522344451 | 0.828864416 | 1.965084244 | 0.050017272 |
| RP3-325F22.5 | -0.332994597 | -0.051176069 | -1.965058078 | 0.05002031 |
| FHAD1 | -0.34927588 | -2.887974378 | -1.964575565 | 0.050076367 |
| CTC-537E7.3 | -0.243307179 | -0.345580602 | -1.964352445 | 0.050102306 |
| CINP | 0.071340882 | 4.851450858 | 1.963917675 | 0.050152883 |
| MAML1 | -0.079388508 | 5.072625327 | -1.963558533 | 0.050194694 |
| TOLLIP-DT | 0.182903295 | 1.611867173 | 1.963423802 | 0.050210387 |
| IL7R | -0.319313405 | 4.320424612 | -1.963398947 | 0.050213283 |
| FGF16 | 0.31408572 | -1.41947476 | 1.963319674 | 0.050222519 |
| POSTN | -0.365093438 | -3.735222815 | -1.962902986 | 0.050271089 |
| AVPR2 | -0.269907232 | -2.018718054 | -1.962674001 | 0.050297798 |
| TRAV16 | -0.255127623 | -0.351903437 | -1.96252623 | 0.050315039 |
| RP11-284N8.3 | 0.305802493 | 3.975730028 | 1.962506746 | 0.050317313 |
| RP11-497H16.6 | -0.364384582 | 1.179408927 | -1.962395701 | 0.050330274 |
| CD80 | 0.305078053 | -0.449749079 | 1.962074843 | 0.050367738 |
| DACH1 | 0.393663312 | 3.575491972 | 1.961818428 | 0.050397694 |
| GPR183 | 0.35160544 | 6.20243905 | 1.961783508 | 0.050401775 |
| FAM225A | -0.336464119 | -2.395844643 | -1.96052538 | 0.050548988 |
| TRPC1 | 0.30312536 | -0.634499566 | 1.960245275 | 0.050581811 |
| TBX18 | -0.435317 | -4.593371791 | -1.959872906 | 0.050625475 |
| RP11-148O21.3 | -0.369949046 | -0.448856814 | -1.95957389 | 0.05066056 |
| TACC2 | 0.529030697 | -4.657180525 | 1.959555805 | 0.050662683 |
| TTC36 | -0.211431539 | -0.150304989 | -1.958849235 | 0.050745673 |
| PDZD7 | -0.32921217 | 0.725743993 | -1.958133653 | 0.050829838 |
| CCR8 | -0.254488649 | -1.718920872 | -1.956851563 | 0.050980927 |
| IFRD2 | -0.120003396 | 5.114852573 | -1.956697605 | 0.050999096 |
| RBM22 | 0.080023334 | 7.09700268 | 1.956686023 | 0.051000463 |
| IQCB1 | -0.072140468 | 5.674537536 | -1.956518131 | 0.051020283 |
| AIM1L | 0.282518152 | -2.11371683 | 1.956398865 | 0.051034366 |
| PAIP2 | 0.076979668 | 7.729997039 | 1.95639071 | 0.051035329 |
| RP11-112J3.16 | -0.260321764 | 1.268611026 | -1.956235948 | 0.051053609 |
| RBFOX2 | 0.387615621 | 0.434773922 | 1.956096315 | 0.051070107 |
| AC008265.2 | -0.224927073 | -0.509524348 | -1.956053583 | 0.051075157 |
| GLB1L | 0.201387496 | 2.656275121 | 1.955851428 | 0.051099052 |
| SLC35F2 | 0.264605129 | 4.332894975 | 1.955736979 | 0.051112583 |
| ANGPT2 | -0.520533934 | -2.08407978 | -1.955171833 | 0.051179448 |
| PDE9A | 0.388553165 | 0.382973831 | 1.954583864 | 0.051249091 |
| OR2A1-AS1 | -0.304348823 | -0.482466213 | -1.954071013 | 0.051309902 |
| AMT | -0.131387473 | 3.915398339 | -1.953908867 | 0.051329141 |
| HOXB6 | -0.734214853 | 1.041196962 | -1.95363927 | 0.051361142 |
| EXOC6B | -0.192402744 | 1.987847542 | -1.953228338 | 0.051409952 |
| ABCB4 | 0.281929702 | -1.43021629 | 1.953118707 | 0.051422981 |
| PDPK1 | -0.073491008 | 5.047841093 | -1.953107737 | 0.051424285 |
| MZB1 | -0.335567367 | 4.688126225 | -1.953063383 | 0.051429557 |
| ECHDC3 | 0.271307285 | 2.626409134 | 1.952755126 | 0.051466209 |
| FAM124A | -0.425753416 | 0.084169748 | -1.952670885 | 0.051476229 |
| SERP2 | 0.233663127 | -1.247804358 | 1.952392016 | 0.051509411 |
| IGKV1D-13 | 0.322692789 | 0.048488752 | 1.95221521 | 0.051530458 |
| ZNF549 | 0.116610887 | 2.568720149 | 1.95203761 | 0.051551606 |
| GAB1 | 0.141022137 | 3.583459239 | 1.951695985 | 0.051592308 |
| UNC119B | -0.136397424 | 6.03383824 | -1.951685462 | 0.051593562 |
| MGAM | 0.421444927 | 4.279218193 | 1.951567576 | 0.051607614 |
| EPS15L1 | -0.082736343 | 5.278794151 | -1.951133885 | 0.051659336 |
| ZNF652 | 0.084795603 | 4.822529744 | 1.950930317 | 0.051683629 |
| UPF1 | 0.068189324 | 5.943538625 | 1.950790554 | 0.051700313 |
| SHANK3 | 0.447283763 | 4.233407827 | 1.95073447 | 0.051707009 |
| RN7SK | -0.162303845 | 6.165224253 | -1.950733804 | 0.051707089 |
| MIR548AR | 0.17865541 | 2.698498959 | 1.950684608 | 0.051712963 |
| GS1-166A23.2 | -0.193861437 | 0.805208607 | -1.950084559 | 0.051784659 |
| PROKR2 | 0.313625363 | -2.066192048 | 1.949997945 | 0.051795015 |
| SULF2 | -0.422728623 | 4.768626747 | -1.949951208 | 0.051800604 |
| LTB4R | -0.129279419 | 6.217139159 | -1.949923397 | 0.05180393 |
| CYTH3 | 0.148904502 | 4.218589032 | 1.949849133 | 0.051812812 |
| RPS3AP44 | 0.180119407 | 0.229437305 | 1.949516745 | 0.051852581 |
| RPL15P20 | 0.182692954 | 2.675147652 | 1.948855287 | 0.051931798 |
| TUFM | -0.090387829 | 8.591159321 | -1.948482367 | 0.051976505 |
| FAM114A1 | -0.25381346 | 1.181704427 | -1.948278625 | 0.052000943 |
| RP1-102H19.8 | -0.201565018 | -0.376056978 | -1.947688423 | 0.052071792 |
| LDHAP5 | 0.146583378 | 2.279417102 | 1.947344255 | 0.052113144 |
| RPS3AP26 | -0.102013925 | 10.16657818 | -1.947063715 | 0.052146871 |
| HEXIM1 | 0.129640648 | 6.126432103 | 1.94681119 | 0.052177246 |
| SP140 | -0.261485145 | 2.905689967 | -1.946718437 | 0.052188406 |
| HNRNPA3P5 | -0.092183066 | 7.328276495 | -1.946686987 | 0.052192191 |
| RXFP1 | -0.61010469 | 0.130552091 | -1.946667283 | 0.052194562 |
| AL450992.2 | -0.26108771 | 0.802715606 | -1.946464264 | 0.052219 |
| IGKV1-32 | 0.343454213 | -0.239238313 | 1.946162112 | 0.052255388 |
| RP11-403I13.9 | -0.361718064 | -0.38842962 | -1.94607546 | 0.052265827 |
| AC141928.1 | -0.43550624 | -3.602559282 | -1.945270568 | 0.05236288 |
| DDX11L16 | -0.287887818 | -1.277386277 | -1.945208216 | 0.052370405 |
| ATN1 | 0.201816014 | 6.337080777 | 1.944505534 | 0.052455268 |
| MAEA | -0.082530759 | 4.873640014 | -1.943777437 | 0.052543321 |
| HERC2 | -0.117068218 | 5.60719357 | -1.943693723 | 0.052553453 |
| ABHD17B | 0.151296051 | 5.38952414 | 1.943542429 | 0.052571769 |
| RAB1C | -0.113717735 | 5.725805526 | -1.943401898 | 0.052588786 |
| RP11-370B11.1 | 0.177622191 | 3.34533354 | 1.943399405 | 0.052589088 |
| MPND | -0.133006414 | 5.410722004 | -1.943284784 | 0.052602971 |
| GPR63 | -0.367887281 | -2.272802107 | -1.942861614 | 0.052654254 |
| COPS8P2 | 0.078678635 | 4.099389044 | 1.942569522 | 0.052689676 |
| GRIN3B | 0.280877979 | -0.7138973 | 1.942433983 | 0.052706119 |
| RP11-362K14.7 | 0.174174541 | 1.50767042 | 1.941634022 | 0.052803258 |
| TCF7 | 0.294892972 | 3.228624076 | 1.941601408 | 0.052807222 |
| PRUNE2 | 0.359077826 | 0.414388415 | 1.940981834 | 0.052882564 |
| MTRNR2L8 | 0.190435087 | 4.188565094 | 1.940679757 | 0.052919331 |
| SREK1IP1 | 0.075463384 | 4.470146148 | 1.940596813 | 0.05292943 |
| UBL3 | 0.11440871 | 5.787654926 | 1.940407742 | 0.052952457 |
| SPRED1 | 0.418267047 | 0.28920003 | 1.9401839 | 0.052979729 |
| RP11-670E13.2 | 0.215912757 | 0.87742219 | 1.940101492 | 0.052989772 |
| RP11-490G8.1 | 0.098576612 | 4.265128382 | 1.939237447 | 0.053095173 |
| USP20 | -0.122590417 | 5.951673014 | -1.939128196 | 0.053108512 |
| PDP2 | 0.149691328 | 2.110748299 | 1.938931664 | 0.053132516 |
| RP11-347H15.2 | 0.198039993 | 0.208734431 | 1.93832591 | 0.053206557 |
| IDUA | -0.120390918 | 5.438063457 | -1.938312812 | 0.053208159 |
| MARS2 | -0.134153733 | 3.974382028 | -1.93809155 | 0.053235226 |
| VDAC1P2 | -0.111814195 | 4.706916015 | -1.93770521 | 0.053282515 |
| TFEB | -0.224633478 | 3.879474969 | -1.937339973 | 0.053327253 |
| TRIM10 | 0.509704553 | 0.483344483 | 1.937322106 | 0.053329442 |
| GTF2IRD2P1 | 0.184411466 | 0.148341683 | 1.937294516 | 0.053332823 |
| RP11-499E18.1 | -0.203358683 | -0.594400971 | -1.936567899 | 0.053421928 |
| DRAM1 | -0.158192628 | 5.563632885 | -1.936309309 | 0.053453669 |
| CPNE2 | -0.171014376 | 5.367039099 | -1.935975056 | 0.053494721 |
| RP11-755F10.3 | -0.212639786 | -0.395773887 | -1.935484846 | 0.053554975 |
| C12orf73 | -0.117075164 | 2.420306863 | -1.93531591 | 0.053575752 |
| STYK1 | -0.328976513 | 1.015363874 | -1.935087936 | 0.053603802 |
| KLC4 | -0.07413651 | 3.918282154 | -1.934785661 | 0.053641013 |
| BCL9L | 0.139584952 | 4.824342047 | 1.934498985 | 0.053676323 |
| CAMK2D | 0.333502274 | 2.36576629 | 1.934249617 | 0.053707054 |
| CPQ | 0.171039647 | 5.235899445 | 1.933909986 | 0.053748932 |
| CEP350 | 0.0592289 | 5.874600007 | 1.933755418 | 0.053768 |
| RP13-270P17.1 | 0.199063599 | 0.420835362 | 1.933694337 | 0.053775537 |
| EMG1 | -0.085363395 | 4.801403435 | -1.933457919 | 0.053804716 |
| GOLGA8N | -0.199886902 | 4.406035834 | -1.9331135 | 0.05384725 |
| AC012442.6 | -0.169352576 | 0.862873729 | -1.933013988 | 0.053859544 |
| PLEKHM1 | 0.084279032 | 5.334303007 | 1.932799194 | 0.053886089 |
| RP11-104N10.1 | -0.200130263 | -0.223226127 | -1.932646431 | 0.053904974 |
| LILRB5 | 0.378548854 | -0.488560463 | 1.932533985 | 0.053918879 |
| PEBP1 | 0.135541569 | 8.58708942 | 1.932533933 | 0.053918886 |
| PTP4A2P2 | 0.15175031 | 1.265811361 | 1.932521885 | 0.053920376 |
| KB-226F1.2 | -0.306623729 | -0.50956832 | -1.931865697 | 0.05400158 |
| RP11-360F5.3 | 0.218443784 | 1.428115705 | 1.931821425 | 0.054007063 |
| CREG1 | -0.112805273 | 8.528736147 | -1.931174548 | 0.054087223 |
| NAP1L4 | -0.073725691 | 6.634478264 | -1.93116074 | 0.054088935 |
| DHRS11 | 0.198063227 | 3.078788314 | 1.931000725 | 0.054108779 |
| SLC27A5 | -0.175256422 | 0.745870664 | -1.930931288 | 0.054117393 |
| TM2D2 | -0.085056214 | 4.209096074 | -1.930901473 | 0.054121091 |
| NPHP4 | -0.104497238 | 2.797378433 | -1.930724296 | 0.054143075 |
| AC226118.1 | -0.292464905 | -2.304367377 | -1.930647034 | 0.054152665 |
| PTDSS2 | -0.080315427 | 4.041281502 | -1.930078733 | 0.054223241 |
| MORF4L2 | 0.072796514 | 7.323774825 | 1.929747263 | 0.054264441 |
| PTPRH | -0.420385773 | -1.001422839 | -1.929581995 | 0.054284993 |
| CFI | 0.482802779 | -1.52284724 | 1.929572311 | 0.054286197 |
| LINC00853 | 0.240277844 | 0.063017267 | 1.928964466 | 0.054361843 |
| RFPL2 | -0.285441597 | -1.681926186 | -1.928393434 | 0.054432988 |
| HERC2P9 | -0.114465015 | 4.330201367 | -1.927469034 | 0.054548324 |
| MAPK8IP3 | 0.106201327 | 6.498149253 | 1.927406557 | 0.054556126 |
| RP11-508N22.12 | -0.269333877 | -1.62374899 | -1.927315711 | 0.054567473 |
| ERP29P1 | -0.108937551 | 4.747865161 | -1.927262942 | 0.054574065 |
| AMD1 | 0.138735545 | 8.073569755 | 1.927159246 | 0.054587021 |
| TNRC18P2 | -0.201409467 | -1.533274238 | -1.927082837 | 0.054596569 |
| KRT5 | 0.496027887 | -3.089368929 | 1.926705075 | 0.054643796 |
| PDCL3 | -0.08732609 | 5.180967572 | -1.926442727 | 0.054676614 |
| FIP1L1 | 0.065188468 | 5.782904899 | 1.926197727 | 0.054707277 |
| RP11-430C7.2 | 0.153190566 | 1.906835825 | 1.925653934 | 0.054775386 |
| CLEC1A | -0.337999199 | -1.284056247 | -1.925626169 | 0.054778866 |
| PFDN5 | 0.089567928 | 8.521344306 | 1.925306701 | 0.054818914 |
| RP3-466P17.1 | 0.211255053 | 0.673497547 | 1.92512759 | 0.054841378 |
| GNB4 | 0.099863179 | 6.213730331 | 1.924907757 | 0.054868959 |
| KIAA0355 | 0.178513163 | 3.003423489 | 1.924900717 | 0.054869843 |
| CREB5 | 0.311514341 | 3.215340032 | 1.924895232 | 0.054870531 |
| RP11-511B23.2 | -0.27067211 | -1.614914264 | -1.924798899 | 0.054882622 |
| PIGG | -0.069513045 | 4.500327822 | -1.924704251 | 0.054894503 |
| PSME2 | -0.087343072 | 6.243993423 | -1.924643409 | 0.054902142 |
| AC005083.1 | -0.283237015 | 0.016128164 | -1.923536044 | 0.055041326 |
| CMTM4 | 0.228765143 | 3.022302497 | 1.923433341 | 0.055054249 |
| RP11-443B7.1 | -0.241648315 | 3.156831238 | -1.92334256 | 0.055065675 |
| HINT3 | -0.06266527 | 5.650273452 | -1.923229058 | 0.055079963 |
| ALDH3B1 | -0.208619816 | 5.64776595 | -1.923166958 | 0.055087781 |
| ZHX3 | 0.131477545 | 3.11332133 | 1.922992069 | 0.055109806 |
| GRIPAP1 | -0.058870836 | 6.466307147 | -1.922866602 | 0.055125611 |
| SMCO2 | 0.273379549 | -0.717588674 | 1.922501524 | 0.055171621 |
| USP4 | -0.062188741 | 6.377065429 | -1.922377242 | 0.055187291 |
| DLG5 | 0.197446259 | 2.964017754 | 1.922326655 | 0.05519367 |
| RP11-328L11.1 | 0.221522309 | 0.133341638 | 1.92207908 | 0.0552249 |
| FAM32B | -0.111803141 | 3.366365243 | -1.921804674 | 0.055259532 |
| AATK | 0.288324982 | 3.097933943 | 1.921371343 | 0.055314258 |
| OR7E38P | 0.111646699 | 4.200221111 | 1.921039213 | 0.055356235 |
| ARSG | 0.267020365 | 1.289255627 | 1.92084603 | 0.055380662 |
| UQCRQ | -0.090259821 | 6.25226362 | -1.920507015 | 0.055423552 |
| HLA-W | -0.24931856 | 0.661351475 | -1.920445026 | 0.055431397 |
| HMHA1 | -0.126453392 | 8.112521026 | -1.920436594 | 0.055432464 |
| PSMB6 | -0.064460471 | 7.194111248 | -1.920084963 | 0.055476985 |
| SNORD3B-1 | -0.331352303 | 0.655652149 | -1.91974795 | 0.055519683 |
| DUSP10 | 0.215164502 | 6.860615903 | 1.919693846 | 0.05552654 |
| GPR133 | -0.432215186 | -1.520925396 | -1.919589419 | 0.055539778 |
| KRT1 | 0.450192739 | -0.38921859 | 1.919437458 | 0.055559046 |
| APBA1 | -0.42399033 | -0.965283469 | -1.919360528 | 0.055568802 |
| RP11-529K1.4 | -0.198263608 | -0.128189873 | -1.918987137 | 0.055616176 |
| MPP1 | 0.113868085 | 7.105781162 | 1.91893217 | 0.055623153 |
| CTD-2192J16.15 | -0.092573538 | 10.0654358 | -1.918553766 | 0.055671202 |
| ST13P6 | -0.102743848 | 6.743949834 | -1.918251769 | 0.055709575 |
| RP11-417N10.3 | -0.205884493 | -0.610329265 | -1.918129363 | 0.055725134 |
| FUZ | -0.09532404 | 4.32675843 | -1.918042041 | 0.055736236 |
| ROBO1 | 0.52428376 | -2.767101497 | 1.917803714 | 0.055766546 |
| CTB-180A7.6 | 0.197726897 | 1.183197028 | 1.917551708 | 0.055798611 |
| UBE2Q2P2 | 0.228697429 | -1.768181669 | 1.916918588 | 0.055879236 |
| NUP133 | -0.062859916 | 5.494133291 | -1.916713668 | 0.055905352 |
| NCOA1 | -0.078749577 | 6.289394687 | -1.916082565 | 0.055985849 |
| AC068831.15 | 0.222758945 | -0.384773157 | 1.915910221 | 0.056007848 |
| SNHG10 | -0.140979481 | 4.052044327 | -1.915835216 | 0.056017424 |
| ENO1-IT1 | 0.182163769 | 2.158081706 | 1.915532249 | 0.05605612 |
| IFI44L | 0.404453541 | 3.042813521 | 1.914979557 | 0.056126768 |
| ZNF550 | -0.143551828 | 4.058232384 | -1.914790285 | 0.056150979 |
| RP11-304L19.5 | -0.155214969 | 4.790878922 | -1.914772416 | 0.056153266 |
| PPIAL4C | -0.179683216 | 0.101708248 | -1.914530657 | 0.056184204 |
| RPL7P6 | 0.161771582 | 2.464331229 | 1.913971496 | 0.056255817 |
| PSMC5 | -0.060439325 | 6.852818511 | -1.913776389 | 0.056280822 |
| RP11-265N6.3 | -0.130957297 | 2.146962858 | -1.913770909 | 0.056281525 |
| PCLO | 0.467170337 | -4.570100729 | 1.913622864 | 0.056300505 |
| OFD1P17 | 0.125944703 | 1.858837799 | 1.913563692 | 0.056308093 |
| HYOU1 | -0.127748473 | 6.640219836 | -1.913346192 | 0.05633599 |
| HBA2 | 0.611376139 | 6.596125052 | 1.912740621 | 0.056413725 |
| JPH4 | -0.32103356 | 0.708380571 | -1.912374927 | 0.056460711 |
| ALDH4A1 | -0.191063592 | 2.979266794 | -1.912005177 | 0.056508251 |
| KRT18P29 | -0.360911792 | -0.643354102 | -1.911918071 | 0.056519456 |
| CTD-3065J16.6 | -0.29990639 | 1.542421627 | -1.911588493 | 0.056561866 |
| SYNCRIP | -0.07342355 | 7.18590641 | -1.91154451 | 0.056567528 |
| SEC11A | -0.091531497 | 6.350997462 | -1.911463875 | 0.056577909 |
| CPOX | -0.158178044 | 4.697496203 | -1.911035998 | 0.056633021 |
| DNAJC9-AS1 | 0.182410738 | -1.500337618 | 1.910917522 | 0.056648289 |
| EMC4 | 0.051960538 | 5.615324063 | 1.910743617 | 0.056670707 |
| RFXANK | -0.057167102 | 6.22350624 | -1.910656681 | 0.056681916 |
| PDE5A | 0.33221955 | -1.534313918 | 1.910302714 | 0.056727575 |
| RP3-322G13.5 | 0.157083831 | 1.345325448 | 1.910252598 | 0.056734042 |
| RP11-611L7.1 | -0.081143196 | 6.843229442 | -1.910231228 | 0.0567368 |
| SHKBP1 | -0.087549556 | 7.49399985 | -1.910221241 | 0.056738089 |
| MAFB | 0.500292573 | 5.23989374 | 1.909759971 | 0.056797645 |
| LAMTOR4 | -0.078180071 | 6.661093398 | -1.9097018 | 0.056805159 |
| PAOX | -0.204775789 | 1.890275191 | -1.909558462 | 0.056823678 |
| AC006942.4 | -0.213336622 | 0.589227962 | -1.909180083 | 0.05687259 |
| RP11-434C1.1 | -0.288854339 | 0.633121303 | -1.909067572 | 0.05688714 |
| RP11-415F23.2 | 0.191970931 | 2.368512287 | 1.908998118 | 0.056896124 |
| ITGB3BP | 0.084911006 | 3.622443693 | 1.90876903 | 0.056925764 |
| TSPAN33 | 0.23885479 | 5.019436647 | 1.908729549 | 0.056930874 |
| BSCL2 | -0.108526293 | 2.729876808 | -1.908692183 | 0.05693571 |
| CTB-50L17.14 | 0.267991849 | -1.431929811 | 1.90868682 | 0.056936404 |
| PHB2 | 0.095358348 | 8.249006198 | 1.908571512 | 0.05695133 |
| IQANK1 | -0.314623533 | -2.765181474 | -1.908017443 | 0.057023098 |
| ZNF217 | 0.110104811 | 6.327146612 | 1.90788316 | 0.057040503 |
| RPL30P4 | 0.166665694 | 1.189059556 | 1.907816117 | 0.057049194 |
| ZNF611 | -0.083985787 | 3.407542134 | -1.907423707 | 0.057100088 |
| IGHV3OR16-13 | 0.295869618 | 0.244560127 | 1.906933291 | 0.057163745 |
| TTC24 | -0.333807705 | 3.334432351 | -1.906016268 | 0.057282937 |
| LL0XNC01-7P3.1 | 0.143821009 | 2.816208898 | 1.905216953 | 0.057386998 |
| AL928768.3 | -0.285263467 | -0.48465101 | -1.905017256 | 0.057413021 |
| CENPK | 0.14771326 | 3.990294505 | 1.904717629 | 0.057452085 |
| DNAH3 | -0.419118763 | -5.079547666 | -1.904619319 | 0.057464906 |
| RECK | -0.217263791 | 2.873640811 | -1.904333906 | 0.057502144 |
| LRRC16A | 0.283325761 | 1.507823722 | 1.903980513 | 0.057548279 |
| RP11-930P14.2 | 0.186390557 | -0.541102587 | 1.903939899 | 0.057553583 |
| RP11-542M13.3 | -0.300998818 | 0.575907692 | -1.903864347 | 0.057563451 |
| RP11-234N17.1 | 0.117928868 | 3.334201353 | 1.903859019 | 0.057564147 |
| FKBPL | -0.136414313 | 3.17427892 | -1.903659656 | 0.057590193 |
| PRPF38A | -0.055460247 | 6.321870607 | -1.903643031 | 0.057592366 |
| AP001189.4 | 0.376033882 | -0.472786507 | 1.903375909 | 0.057627281 |
| KLRB1 | 0.305002611 | 3.679141 | 1.902936845 | 0.057684709 |
| OR52K3P | -0.410972032 | 2.703670464 | -1.902741869 | 0.057710226 |
| COL5A1 | 0.473342018 | 0.323622775 | 1.902627044 | 0.057725258 |
| ABCB9 | 0.154869808 | 0.625860542 | 1.902622553 | 0.057725846 |
| CDADC1 | 0.115353702 | 4.660475141 | 1.902493909 | 0.057742691 |
| RP11-309L24.9 | 0.213507643 | 0.239900709 | 1.901806092 | 0.057832826 |
| CXCL11 | 0.304489178 | -1.424344326 | 1.901805071 | 0.05783296 |
| AC068587.2 | -0.223388796 | 0.176651038 | -1.901755922 | 0.057839405 |
| TRAV8-6 | -0.277017786 | 0.098630412 | -1.901428889 | 0.057882307 |
| RP11-158M2.3 | -0.221310259 | -1.099092011 | -1.901423516 | 0.057883012 |
| CCL3 | 0.318779787 | 5.88453112 | 1.901325143 | 0.057895922 |
| SPECC1L | -0.072631303 | 4.584981947 | -1.9011423 | 0.057919924 |
| RP11-288C17.1 | -0.162044132 | 1.17794038 | -1.89999775 | 0.058070361 |
| RP11-320A16.1 | -0.12483879 | 4.478854294 | -1.899962007 | 0.058075064 |
| RP5-1142A6.2 | 0.189947708 | -2.457816378 | 1.899880594 | 0.058085778 |
| TSPAN3 | -0.122234657 | 6.294554453 | -1.899642549 | 0.058117114 |
| CERK | -0.111783447 | 6.452711096 | -1.89931241 | 0.058160596 |
| ACTRT3 | -0.226382349 | 0.110559989 | -1.899306427 | 0.058161384 |
| CAPN7 | 0.06587095 | 5.231426686 | 1.899255608 | 0.05816808 |
| CBX2 | -0.337446556 | 3.780947722 | -1.898680447 | 0.058243907 |
| CIAPIN1P | 0.089306033 | 3.281515474 | 1.898555091 | 0.058260444 |
| OLFML3 | 0.316052792 | -0.252156037 | 1.898239432 | 0.058302104 |
| C2orf27A | -0.366563342 | -0.43247308 | -1.89822294 | 0.058304281 |
| ECT2 | 0.161800412 | 3.609042768 | 1.898200834 | 0.0583072 |
| CHAC2 | -0.193105064 | 2.265666942 | -1.897951439 | 0.058340134 |
| NDUFB2 | -0.109444573 | 4.305373858 | -1.897365815 | 0.058417531 |
| RP11-84A19.4 | 0.19261545 | 2.858509182 | 1.897219748 | 0.058436849 |
| EFTUD1P1 | -0.171586846 | -0.327550527 | -1.896881861 | 0.058481556 |
| AC005037.3 | 0.187874857 | 0.705700547 | 1.896336815 | 0.058553733 |
| MCTP2 | 0.241210312 | 4.69521775 | 1.896126948 | 0.058581544 |
| RP11-387D10.2 | -0.191025221 | 0.423666036 | -1.896023284 | 0.058595286 |
| SLC35D2 | -0.131813306 | 5.837332239 | -1.896008562 | 0.058597237 |
| DNAJC6 | 0.360204335 | 1.808458038 | 1.895999168 | 0.058598483 |
| VGLL4 | 0.098406236 | 5.378245994 | 1.89526666 | 0.058695663 |
| ITLN1 | 0.326213732 | -0.882921473 | 1.895053102 | 0.05872402 |
| KLRC3 | 0.295110001 | -0.332805149 | 1.895049264 | 0.05872453 |
| GPR157 | -0.184433904 | 1.541087421 | -1.894981266 | 0.058733562 |
| THRA | 0.092547081 | 5.079384889 | 1.894942401 | 0.058738725 |
| AC092580.4 | -0.259974447 | 0.698933132 | -1.89472796 | 0.058767217 |
| RP11-222K16.2 | -0.278242519 | 0.630473988 | -1.894642432 | 0.058778584 |
| SLC2A5 | 0.297950845 | 4.860953095 | 1.894545376 | 0.058791485 |
| IGHV1-8 | 0.365380221 | 1.077212057 | 1.894531186 | 0.058793372 |
| LLGL1 | 0.078343753 | 4.047055905 | 1.894397515 | 0.058811145 |
| SIDT2 | -0.141717958 | 4.708329852 | -1.894258115 | 0.058829684 |
| DOCK9-AS2 | 0.262919168 | -0.932332335 | 1.89422524 | 0.058834057 |
| MCM3 | -0.16785811 | 7.069360098 | -1.893749413 | 0.05889738 |
| SFI1 | 0.078179165 | 4.39875687 | 1.893135653 | 0.058979142 |
| ZNF45 | -0.082061011 | 4.295027215 | -1.893134305 | 0.058979322 |
| RPLP0 | 0.12443446 | 9.181032695 | 1.892987175 | 0.058998936 |
| CNFN | -0.25255125 | 0.477140106 | -1.892655473 | 0.059043176 |
| KRT16 | 0.371176727 | -2.807573887 | 1.892535701 | 0.059059157 |
| PTMAP2 | -0.102708273 | 10.91767877 | -1.892363051 | 0.0590822 |
| SUCNR1 | -0.357694865 | 4.710758736 | -1.892013016 | 0.05912894 |
| AC097658.1 | 0.223853518 | -1.927860296 | 1.891921758 | 0.059141131 |
| TMC6 | 0.108385664 | 6.0858945 | 1.891804364 | 0.059156817 |
| DNAJC30 | 0.078918038 | 4.074178154 | 1.891576111 | 0.059187324 |
| MIR3682 | 0.170966456 | 2.19530712 | 1.891573227 | 0.05918771 |
| AP000487.5 | 0.24647255 | -2.59259081 | 1.891522466 | 0.059194496 |
| IPO5 | 0.0962547 | 6.426662573 | 1.891494816 | 0.059198193 |
| PPP1R3F | -0.117041233 | 2.070664961 | -1.890900673 | 0.059277677 |
| CIPC | -0.07323924 | 3.89176687 | -1.890831117 | 0.059286988 |
| SIL1 | -0.102695536 | 5.134105522 | -1.890460051 | 0.059336681 |
| FN3KRP | 0.075707076 | 5.5765878 | 1.89035408 | 0.059350879 |
| RP11-393M11.2 | -0.190872711 | 0.673353338 | -1.88973713 | 0.059433594 |
| ITM2A | 0.366833805 | 7.036700275 | 1.889663423 | 0.059443482 |
| AVPR1A | -0.394914114 | -1.413469202 | -1.889519707 | 0.059462766 |
| FAM208B | 0.080918129 | 5.157136759 | 1.889202438 | 0.059505357 |
| RABL5 | 0.176770838 | 2.442949188 | 1.88917496 | 0.059509047 |
| TMEM176B | -0.522708147 | 3.397132919 | -1.888807143 | 0.059558458 |
| PDCD2L | -0.128622258 | 2.881590967 | -1.888632695 | 0.059581905 |
| NAT6 | -0.121858551 | 5.805239472 | -1.888562781 | 0.059591304 |
| RP11-452G18.2 | 0.189356553 | 2.786324049 | 1.888390339 | 0.059614491 |
| DYNLT3 | 0.1401154 | 5.094372605 | 1.887809172 | 0.059692694 |
| ARL6IP1P3 | 0.197240595 | -0.19417745 | 1.887739138 | 0.059702124 |
| DOCK7 | -0.162764165 | 3.701149903 | -1.887736742 | 0.059702446 |
| C10orf10 | 0.408957571 | 2.860516833 | 1.88752493 | 0.059730973 |
| MRPL48 | -0.085548208 | 4.052190917 | -1.88743554 | 0.059743015 |
| HNRNPA3P4 | 0.222450799 | 0.927138873 | 1.886785768 | 0.059830612 |
| CA6 | -0.277401021 | -2.208335739 | -1.886784617 | 0.059830767 |
| STOML2 | 0.089674173 | 7.249593881 | 1.886687414 | 0.05984388 |
| CARD10 | 0.350942865 | -1.741251816 | 1.886593414 | 0.059856564 |
| RAD51B | -0.095442412 | 1.824583405 | -1.886489712 | 0.059870559 |
| HNRNPA3P3 | 0.123472461 | 4.47244307 | 1.886488275 | 0.059870753 |
| FPR3 | -0.356297832 | -0.597649171 | -1.886236749 | 0.059904709 |
| GRINA | 0.147783366 | 8.178765099 | 1.885936405 | 0.059945277 |
| LY96 | -0.297800117 | 4.158572943 | -1.885899985 | 0.059950197 |
| NUP62 | -0.106940463 | 5.438811974 | -1.885624056 | 0.05998749 |
| RP11-274B21.9 | -0.239737684 | -0.069787383 | -1.885557061 | 0.059996547 |
| AL356585.3 | 0.209312755 | -0.13020818 | 1.885317832 | 0.060028899 |
| SFXN3 | -0.175414056 | 6.032798862 | -1.884433984 | 0.060148552 |
| LGR6 | 0.297815618 | 0.214798335 | 1.884394315 | 0.060153927 |
| TTC34 | 0.174316416 | 0.446027522 | 1.883831824 | 0.060230185 |
| RP11-1082L8.3 | -0.264540035 | 0.706196988 | -1.883781174 | 0.060237055 |
| CUL9 | -0.058859367 | 4.923386305 | -1.883709179 | 0.060246823 |
| PHF8 | 0.05292819 | 5.024738748 | 1.883670183 | 0.060252113 |
| ASPHD2 | -0.216880521 | 2.454917677 | -1.883644311 | 0.060255624 |
| U52111.14 | -0.261333781 | -1.351295749 | -1.883570568 | 0.060265631 |
| APOD | 0.337384341 | -1.961511309 | 1.883438003 | 0.060283623 |
| DENND6B | -0.180761631 | 4.295730569 | -1.883206287 | 0.060315084 |
| RTCB | -0.058372606 | 6.469605474 | -1.882160475 | 0.060457246 |
| POLA2 | 0.120214694 | 4.3326784 | 1.881636954 | 0.060528515 |
| TBL1XR1 | 0.064941832 | 6.065846789 | 1.881161834 | 0.060593255 |
| LINC00954 | -0.259451392 | 1.665593757 | -1.880698627 | 0.060656428 |
| TOMM22 | -0.07080511 | 7.515840352 | -1.880675987 | 0.060659517 |
| ARHGAP5-AS1 | -0.300766104 | 1.065121941 | -1.8804199 | 0.060694468 |
| Z98881.1 | -0.208630794 | 1.022345072 | -1.880223487 | 0.060721285 |
| FCER1G | -0.280352925 | 9.095292659 | -1.87993422 | 0.060760798 |
| RP11-48B14.1 | 0.137065784 | 0.974686167 | 1.879561266 | 0.060811775 |
| GLCCI1 | 0.169475329 | 3.655469557 | 1.87944501 | 0.060827672 |
| GTF2IP1 | -0.069988232 | 4.911787519 | -1.879278257 | 0.060850481 |
| RP11-254F19.2 | -0.251640582 | -0.579725772 | -1.878192571 | 0.060999157 |
| PON2 | -0.281832303 | 4.051436164 | -1.878173188 | 0.061001814 |
| RP11-99A1.2 | -0.264493097 | -1.264948618 | -1.877699091 | 0.061066835 |
| OXCT2 | 0.237424857 | -1.265270308 | 1.877156241 | 0.061141356 |
| BNIP3L | 0.112291293 | 7.373991867 | 1.877099499 | 0.061149149 |
| EIF4A1P9 | -0.20413674 | -0.298740926 | -1.875773162 | 0.061331562 |
| AC016757.3 | -0.274646122 | 0.937252393 | -1.8753602 | 0.061388449 |
| TRNAU1AP | -0.057803337 | 4.281092405 | -1.874948724 | 0.061445175 |
| IFI44 | 0.229982904 | 5.898732244 | 1.874056165 | 0.061568373 |
| AP000692.10 | 0.186603209 | -0.259634975 | 1.874047906 | 0.061569514 |
| PLD3 | -0.18072404 | 6.781974294 | -1.873954203 | 0.06158246 |
| RP11-291H24.1 | 0.203272441 | 1.001607629 | 1.873885281 | 0.061591983 |
| RP11-110G21.2 | 0.210589325 | -0.101476055 | 1.873763538 | 0.061608808 |
| PNLDC1 | -0.282397892 | -0.977345979 | -1.873731862 | 0.061613187 |
| NPRL3 | 0.099408941 | 4.785603808 | 1.873502407 | 0.06164491 |
| TCEA2 | 0.107408499 | 3.839662182 | 1.872919399 | 0.061725576 |
| TAS2R12 | -0.209550966 | -0.284224109 | -1.872667 | 0.061760525 |
| RP11-498P14.5 | -0.213685053 | 0.129964847 | -1.87255085 | 0.061776613 |
| CTD-2033D24.2 | -0.155548488 | 1.150213728 | -1.871529382 | 0.061918253 |
| HSPB1P2 | -0.244249158 | 1.01830915 | -1.871520519 | 0.061919483 |
| ECSIT | 0.083554149 | 5.389718973 | 1.871346648 | 0.06194362 |
| SMYD2 | -0.114680886 | 4.211819352 | -1.871298745 | 0.061950271 |
| AC018735.1 | -0.199981459 | -0.799009396 | -1.871284481 | 0.061952251 |
| ITGA6 | -0.241884571 | 5.676590307 | -1.871048159 | 0.061985074 |
| DDX27 | 0.0615769 | 6.296753566 | 1.871041148 | 0.061986048 |
| AC012074.1 | 0.198861016 | 0.219125053 | 1.870924665 | 0.062002231 |
| RALGPS2 | -0.190914673 | 4.532544732 | -1.870909489 | 0.06200434 |
| RP11-575G13.2 | 0.240323829 | -1.34815205 | 1.870626194 | 0.062043716 |
| ADAM17 | -0.102065652 | 6.219506371 | -1.870444694 | 0.062068954 |
| LRP3 | 0.309670421 | 4.578932753 | 1.870287547 | 0.062090812 |
| IL10 | 0.399493177 | 0.889497774 | 1.869929817 | 0.062140594 |
| DNASE1L3 | 0.349923482 | -1.747344058 | 1.869925507 | 0.062141194 |
| MAN2C1 | 0.080745824 | 6.283835097 | 1.869879907 | 0.062147542 |
| RAB34 | 0.287754111 | 5.774041796 | 1.869710844 | 0.062171083 |
| LMBR1L | 0.123890818 | 5.337043315 | 1.869709495 | 0.062171271 |
| RP4-753P9.3 | 0.17293812 | 3.195254712 | 1.86969736 | 0.062172961 |
| RPL21P10 | 0.16551078 | 0.839853958 | 1.868430971 | 0.062349537 |
| C19orf18 | -0.211419749 | 1.612233745 | -1.86771528 | 0.062449511 |
| RPS26P47 | 0.229609824 | 2.839797555 | 1.867420866 | 0.062490676 |
| INADL | 0.247066612 | 1.128595701 | 1.867399497 | 0.062493665 |
| RP11-16E12.2 | -0.251660883 | -0.879613694 | -1.866735695 | 0.062586564 |
| ZNF486 | -0.333141661 | 2.55442929 | -1.866371207 | 0.062637623 |
| TRBV7-3 | -0.220362824 | 2.409859298 | -1.866338779 | 0.062642168 |
| IGHV1-2 | 0.369606186 | 2.403616181 | 1.866153051 | 0.0626682 |
| RP5-994D16.3 | -0.183067241 | 0.439008502 | -1.865971914 | 0.062693598 |
| B2M | 0.067367052 | 11.90888591 | 1.865662768 | 0.062736963 |
| RP11-77E14.2 | 0.204721366 | -0.507438801 | 1.864818886 | 0.062855466 |
| FAM90A1 | -0.240229405 | -0.223376327 | -1.864751969 | 0.062864871 |
| RPL13AP6 | 0.162191964 | 2.343685625 | 1.864480107 | 0.062903091 |
| TXN | -0.090803609 | 8.249912742 | -1.864232688 | 0.062937892 |
| CD99 | -0.201086673 | 9.328205387 | -1.863458681 | 0.063046865 |
| SAT2 | -0.119002335 | 6.060296561 | -1.862593156 | 0.063168907 |
| MEPCE | -0.088057002 | 6.144046473 | -1.86245925 | 0.063187805 |
| CTD-2589H19.6 | 0.185670366 | 0.805688577 | 1.862446121 | 0.063189659 |
| RP11-361F15.2 | -0.166032679 | 4.550071391 | -1.862187802 | 0.06322613 |
| RP11-48G14.1 | -0.2282098 | -0.90069273 | -1.861939454 | 0.063261211 |
| AKAP9 | -0.071672148 | 6.063083319 | -1.861337466 | 0.063346312 |
| LINC00852 | 0.123942445 | 3.902793002 | 1.86119015 | 0.063367152 |
| EHD3 | -0.238492195 | 3.531038747 | -1.861184946 | 0.063367888 |
| NKX2-3 | -0.746977652 | 0.784009867 | -1.861129843 | 0.063375685 |
| IL1B | -0.293848087 | 5.748597607 | -1.861111544 | 0.063378274 |
| KCNE1L | -0.57987136 | 0.778769868 | -1.86097913 | 0.063397013 |
| GPR113 | 0.143298167 | 0.52492554 | 1.86075424 | 0.063428851 |
| UBXN11 | -0.158470417 | 6.074085564 | -1.860729722 | 0.063432323 |
| CDC123 | 0.0658886 | 7.238998174 | 1.860424372 | 0.063475574 |
| AKT3 | 0.333607094 | 2.795337129 | 1.860293194 | 0.063494162 |
| RPS9 | 0.085182846 | 9.576953215 | 1.859874528 | 0.063553518 |
| ARRDC4 | 0.186359953 | 5.602423875 | 1.859758174 | 0.063570022 |
| CTC-461F20.1 | 0.222948301 | -1.365910968 | 1.859405576 | 0.063620057 |
| RP11-402J7.2 | -0.355468843 | 2.048798608 | -1.859178757 | 0.063652261 |
| TP53TG3D | -0.325051835 | -2.392497283 | -1.85906141 | 0.063668928 |
| MAX | 0.088000014 | 7.121293982 | 1.858926534 | 0.063688088 |
| LILRP2 | -0.245779802 | -2.097019855 | -1.858862761 | 0.06369715 |
| PTMAP8 | 0.13959739 | 5.029982349 | 1.858637915 | 0.063729105 |
| MTND6P4 | -0.19692414 | 2.208717002 | -1.858615682 | 0.063732266 |
| HIATL2 | 0.080785643 | 4.616291923 | 1.858574908 | 0.063738063 |
| DOLK | 0.134991687 | 3.481186829 | 1.857727266 | 0.063858666 |
| C19orf83 | 0.160127499 | 2.862169862 | 1.857656488 | 0.063868745 |
| MTND6P5 | -0.2383988 | 1.12723794 | -1.857465746 | 0.063895914 |
| DOCK9 | 0.28179199 | 1.634216619 | 1.857250894 | 0.063926528 |
| TAF1B | 0.089591171 | 3.702414466 | 1.857153042 | 0.063940475 |
| RP11-76H14.2 | -0.116817633 | 3.108327308 | -1.8567149 | 0.064002955 |
| ASPSCR1 | -0.083616723 | 3.670856048 | -1.856515437 | 0.064031415 |
| HERPUD2 | -0.074101029 | 7.067872711 | -1.8558717 | 0.064123339 |
| RP6-109B7.5 | -0.254032423 | 0.535653782 | -1.855385681 | 0.064192813 |
| ZCCHC14 | 0.150589756 | 3.465559054 | 1.855370561 | 0.064194976 |
| DLEU2 | 0.120916393 | 3.865929302 | 1.855206243 | 0.064218479 |
| MST1L | -0.247762819 | -0.179938747 | -1.85506805 | 0.064238251 |
| CLINT1 | -0.068959783 | 7.239606209 | -1.854607582 | 0.064304169 |
| CITF22-1A6.3 | -0.167240906 | 0.791834148 | -1.85448081 | 0.064322327 |
| AC073046.25 | 0.120508187 | 4.344728111 | 1.853544184 | 0.064456613 |
| NBPF9 | 0.128746909 | 3.328856859 | 1.853394679 | 0.06447807 |
| SLC34A1 | 0.290999819 | -3.075250552 | 1.853157333 | 0.064512145 |
| RN7SKP80 | -0.170617913 | 1.527793269 | -1.852826899 | 0.064559609 |
| TBCC | 0.117499571 | 6.511209042 | 1.852429049 | 0.064616796 |
| WDR24 | -0.075786716 | 4.307054422 | -1.852258685 | 0.064641296 |
| IGKV1D-33 | 0.340914937 | 0.994700449 | 1.852232034 | 0.06464513 |
| UQCR11 | -0.093751621 | 3.636461627 | -1.852125751 | 0.06466042 |
| CCDC135 | -0.225118955 | 0.014977107 | -1.852125296 | 0.064660485 |
| ST13P13 | 0.103833403 | 3.314033235 | 1.852075121 | 0.064667704 |
| SERPINH1 | -0.244516677 | 3.36656958 | -1.852072914 | 0.064668022 |
| RP11-394D2.1 | -0.226846413 | 0.003687807 | -1.851777065 | 0.064710602 |
| DCTN2 | 0.057586432 | 6.544336999 | 1.851600141 | 0.064736077 |
| hsa-mir-335 | -0.203620918 | -1.276023594 | -1.851147923 | 0.064801229 |
| RP11-73M18.6 | 0.135380308 | 3.958408493 | 1.850988666 | 0.064824186 |
| RPL31P2 | 0.226430687 | 2.103913917 | 1.850792546 | 0.064852466 |
| AQP11 | -0.213863955 | -1.130855312 | -1.85040021 | 0.064909072 |
| ABHD17AP1 | 0.116467212 | 2.282463675 | 1.850131956 | 0.064947798 |
| TPP2 | -0.081161328 | 6.724300902 | -1.850131083 | 0.064947924 |
| PCCB | 0.12076387 | 4.532025303 | 1.849797159 | 0.064996158 |
| RGMB | 0.293299918 | -0.411075275 | 1.849787691 | 0.064997526 |
| DEFA3 | 0.614528752 | 7.21207406 | 1.849243917 | 0.065076137 |
| TRBV3-1 | -0.266595339 | 1.276493987 | -1.848927402 | 0.06512193 |
| HOXA13 | -0.4817954 | -3.190957425 | -1.84857564 | 0.065172854 |
| RNF2 | -0.077238016 | 4.967979995 | -1.848405286 | 0.065197528 |
| RP11-1000B6.3 | -0.174431645 | 3.340352335 | -1.848387132 | 0.065200158 |
| INCA1 | -0.210752169 | -1.296271443 | -1.848233481 | 0.06522242 |
| CDHR1 | 0.28062592 | -1.057223031 | 1.848020786 | 0.065253246 |
| AC024940.1 | -0.211097995 | -0.15771473 | -1.846968789 | 0.065405894 |
| RN7SL473P | -0.253656799 | 1.432277647 | -1.846366333 | 0.065493446 |
| AC021021.1 | -0.165955327 | 1.539569303 | -1.846273697 | 0.065506916 |
| HDDC2 | 0.112797517 | 3.999675987 | 1.846234383 | 0.065512634 |
| RPL30P14 | -0.204220033 | 0.285088818 | -1.846008832 | 0.065545445 |
| NDUFAF6 | 0.096485423 | 4.008960453 | 1.845803976 | 0.065575257 |
| VIP | 0.276308989 | -2.142399939 | 1.845736448 | 0.065585087 |
| ZNRF2P1 | 0.197946483 | 4.088637657 | 1.845131131 | 0.065673253 |
| ZFP28 | -0.152438087 | 2.102523556 | -1.844369484 | 0.065784329 |
| RP5-943J3.2 | -0.14866865 | 3.280201192 | -1.84413776 | 0.065818154 |
| FIZ1 | 0.079239941 | 2.80772339 | 1.843980189 | 0.065841162 |
| RP11-61A14.2 | -0.171046407 | 1.909850003 | -1.8436217 | 0.065893534 |
| SMO | 0.330312349 | 1.007086194 | 1.842926316 | 0.065995221 |
| HEPACAM2 | 0.315542281 | -1.776141774 | 1.842646562 | 0.066036167 |
| C7orf57 | 0.251813678 | -1.999607634 | 1.842215605 | 0.066099284 |
| PALM2AKAP2 | 0.303285955 | -1.774220174 | 1.842212801 | 0.066099695 |
| PLXDC1 | 0.246864832 | -0.120807871 | 1.841869918 | 0.066149949 |
| LYPD6B | -0.359358092 | -2.357356732 | -1.841709751 | 0.066173434 |
| PLEKHG3 | 0.280343805 | 3.691216633 | 1.841536478 | 0.066198849 |
| LSM3P3 | 0.100672971 | 2.718321765 | 1.841330441 | 0.06622908 |
| ACTN1 | -0.169240169 | 6.716765283 | -1.841079961 | 0.066265847 |
| ARPC4 | -0.08114448 | 6.626282816 | -1.841029058 | 0.066273321 |
| NIPBL | 0.042181968 | 6.483415232 | 1.841027676 | 0.066273524 |
| PCDHGC5 | 0.284647902 | -3.033112749 | 1.840504219 | 0.066350422 |
| KIF2A | -0.092537252 | 6.294970583 | -1.840431896 | 0.066361053 |
| ATP2C2 | 0.481452983 | -0.84174273 | 1.840189007 | 0.066396764 |
| AIF1L | -0.46586208 | 2.145447678 | -1.840020945 | 0.066421483 |
| RP11-791G16.2 | 0.139967995 | 5.220996353 | 1.839912083 | 0.066437499 |
| IGHGP | 0.48762666 | 3.909398629 | 1.83923772 | 0.066536783 |
| LHX6 | 0.477036902 | -1.202028597 | 1.83890009 | 0.066586537 |
| NOMO2 | -0.094080407 | 5.204024682 | -1.8388778 | 0.066589823 |
| BHLHE23 | -0.34603715 | -0.633715934 | -1.838767472 | 0.066606088 |
| MTND5P11 | 0.171761937 | 3.731669406 | 1.838480578 | 0.0666484 |
| TRGJP | 0.289399471 | 1.045025073 | 1.838026241 | 0.066715452 |
| EEF1A2 | 0.425676997 | 0.469640043 | 1.837953749 | 0.066726156 |
| RP11-571M6.1 | 0.177639366 | 1.099567122 | 1.837904631 | 0.066733409 |
| AURKAPS1 | 0.199962649 | -0.412588381 | 1.837315484 | 0.066820459 |
| RP11-474N24.6 | -0.220613829 | 1.133948388 | -1.837309797 | 0.0668213 |
| PTPLAD1 | -0.122156066 | 5.562729157 | -1.837257154 | 0.066829083 |
| LA16c-60G3.8 | -0.186814095 | -0.965866151 | -1.836860873 | 0.066887695 |
| CAPN13 | 0.274897157 | -3.421417492 | 1.836629177 | 0.066921983 |
| AL603926.1 | -0.219164558 | -0.572992155 | -1.83648233 | 0.066943723 |
| RP11-127I20.5 | 0.23610541 | -0.151002903 | 1.836190603 | 0.066986927 |
| RPL32P1 | 0.298833769 | 2.89153513 | 1.836001799 | 0.067014902 |
| MSTO1 | -0.082161402 | 2.9245326 | -1.835970036 | 0.067019609 |
| RP11-617D20.1 | -0.264841353 | 2.557276866 | -1.835945669 | 0.06702322 |
| TBX21 | 0.278621511 | 2.885307809 | 1.835679803 | 0.067062632 |
| RP3-400B16.4 | -0.206085989 | -0.557525873 | -1.835632501 | 0.067069647 |
| AC091878.1 | 0.270348564 | -1.935491282 | 1.83522889 | 0.067129521 |
| HS6ST1 | -0.140865854 | 5.067867223 | -1.835222288 | 0.0671305 |
| STARD9 | 0.266745845 | 3.043709009 | 1.835203987 | 0.067133216 |
| DNAH8 | -0.396297013 | -3.741056744 | -1.835067804 | 0.067153429 |
| SETD9 | 0.124339733 | 3.58566016 | 1.834548742 | 0.067230517 |
| TTTY10 | 0.394047708 | -0.868805035 | 1.834193892 | 0.06728326 |
| PCBP1-AS1 | 0.091235572 | 3.62513557 | 1.83382465 | 0.067338177 |
| RP11-621K7.1 | 0.12316951 | 5.365604176 | 1.83351919 | 0.067383636 |
| PIP5K1P1 | -0.194059991 | -0.45699598 | -1.833408872 | 0.06740006 |
| C1QBP | -0.116472832 | 7.914138939 | -1.833148402 | 0.067438852 |
| COL5A2 | -0.221417054 | -0.945595664 | -1.832848149 | 0.067483591 |
| RP11-320M2.1 | 0.146609301 | 1.873190518 | 1.832706104 | 0.067504765 |
| VN1R81P | -0.200821556 | 2.346870202 | -1.832443189 | 0.067543971 |
| CCDC147 | 0.193030585 | 0.01187542 | 1.832238536 | 0.067574502 |
| DND1 | -0.12844386 | 1.406410071 | -1.831809527 | 0.067638541 |
| RPF2 | -0.089223392 | 4.222730648 | -1.831426351 | 0.06769578 |
| RN7SKP296 | 0.247358403 | 2.844212049 | 1.831235398 | 0.067724319 |
| FAM115A | -0.148047212 | 2.447974111 | -1.831186285 | 0.067731661 |
| RPS26P31 | 0.236682435 | 2.591731183 | 1.83099626 | 0.067760075 |
| IKBKG | 0.092509496 | 2.543214405 | 1.830995696 | 0.067760159 |
| RP11-631N16.2 | -0.153221026 | 1.929783362 | -1.830479025 | 0.067837464 |
| ATP6V0C | 0.190680288 | 2.687002904 | 1.830315217 | 0.067861988 |
| AEBP1 | -0.312169744 | 3.575111782 | -1.830233861 | 0.067874171 |
| CD1B | -0.298527149 | -1.783072022 | -1.829722687 | 0.067950758 |
| C7orf43 | -0.092140693 | 5.205211432 | -1.828992418 | 0.068060297 |
| RAB6B | 0.370345413 | 0.905176654 | 1.82846505 | 0.068139491 |
| RHOC | 0.209407376 | 4.128041844 | 1.828227278 | 0.068175221 |
| AACS | -0.128635489 | 2.622841483 | -1.827877056 | 0.068227878 |
| MDGA1 | -0.470509388 | 0.50690534 | -1.827597603 | 0.068269919 |
| MINK1 | 0.112712957 | 5.602340734 | 1.826932358 | 0.068370085 |
| AP003041.2 | 0.224171801 | 1.451225267 | 1.826931887 | 0.068370155 |
| ALAD | -0.107975932 | 6.245447742 | -1.826786647 | 0.06839204 |
| AC109333.10 | -0.231399816 | 0.318148961 | -1.826686029 | 0.068407205 |
| MAGEE1 | 0.258186984 | 0.455572767 | 1.826572281 | 0.068424351 |
| C10orf54 | -0.182783728 | 8.550386513 | -1.826511287 | 0.068433547 |
| RP1-151F17.2 | 0.210074763 | 1.693470129 | 1.825783414 | 0.068543365 |
| ACOT9 | 0.116360625 | 4.874860227 | 1.825524438 | 0.068582473 |
| STK10 | -0.091475571 | 6.979496554 | -1.824559574 | 0.06872834 |
| AKT1 | -0.060817424 | 6.524674882 | -1.824475012 | 0.068741136 |
| RP11-169K16.9 | -0.093682745 | 3.765251063 | -1.824449684 | 0.068744969 |
| FAM195A | 0.102207476 | 4.761231091 | 1.824365517 | 0.068757708 |
| KHDRBS3 | 0.362863335 | -0.618715819 | 1.824244665 | 0.068776002 |
| SCN11A | -0.271638256 | -2.0542562 | -1.823880382 | 0.068831172 |
| RP11-646I6.5 | -0.151343919 | 1.672126475 | -1.823186105 | 0.068936419 |
| PIPSL | -0.059856523 | 4.394867453 | -1.823099952 | 0.068949489 |
| CTC-428H11.2 | 0.13206094 | 3.48382264 | 1.822668936 | 0.069014905 |
| RP11-377K22.2 | 0.28200235 | 0.271263219 | 1.82255481 | 0.069032234 |
| CHN1 | 0.212460883 | -0.580811837 | 1.822544867 | 0.069033744 |
| KLHL22 | -0.086945865 | 3.727252345 | -1.821890691 | 0.069133149 |
| CASC15 | -0.374526682 | 0.24766054 | -1.82167684 | 0.069165671 |
| LARS2 | -0.087545255 | 4.215217511 | -1.821504305 | 0.069191918 |
| LINC01123 | -0.249173468 | -2.961184888 | -1.820757045 | 0.069305692 |
| LURAP1L | 0.266649292 | -2.478255475 | 1.820212491 | 0.0693887 |
| FGFRL1 | -0.197510389 | 5.08204173 | -1.820191327 | 0.069391927 |
| NFATC2 | 0.180220427 | 4.611464226 | 1.819871516 | 0.069440717 |
| CLEC6A | -0.310066644 | -1.210606475 | -1.819843895 | 0.069444932 |
| UBFD1 | -0.089801802 | 4.700392117 | -1.819418232 | 0.069509918 |
| AF131215.3 | -0.283491069 | -1.222192267 | -1.818750788 | 0.069611916 |
| LDHAL6A | -0.294952434 | -2.841691239 | -1.818467401 | 0.06965526 |
| PTP4A2P1 | 0.09477601 | 6.807159743 | 1.818278875 | 0.069684108 |
| PPP1R17 | -0.306852718 | -2.400160481 | -1.818131849 | 0.069706612 |
| NNAT | 0.319211039 | -1.8279436 | 1.817438988 | 0.069812744 |
| ENO1 | -0.100274207 | 10.23250851 | -1.817273256 | 0.069838151 |
| AC098823.3 | -0.219162228 | -1.21865334 | -1.816877433 | 0.06989886 |
| RP11-439L8.3 | -0.320130816 | -1.248450437 | -1.816640732 | 0.069935186 |
| RP11-39K24.13 | 0.229180648 | 1.675772455 | 1.816568867 | 0.069946217 |
| RP13-516M14.1 | 0.14578403 | 4.874007783 | 1.816213559 | 0.07000078 |
| GPR150 | 0.244299679 | -0.696586885 | 1.815884589 | 0.07005133 |
| RP4-612B15.2 | 0.119809108 | 1.903591252 | 1.815881243 | 0.070051845 |
| IRX1 | -0.465915709 | -1.68060193 | -1.815772377 | 0.07006858 |
| RPS5P3 | 0.206655885 | 1.068775395 | 1.815028367 | 0.070183039 |
| RFX3 | -0.095020281 | 3.151344364 | -1.814979243 | 0.070190602 |
| IGHD7-27 | 0.295540835 | -0.472808802 | 1.81477479 | 0.070222085 |
| USP30-AS1 | -0.21268297 | 3.090124729 | -1.81469231 | 0.070234789 |
| NPIPB4 | 0.11299542 | 2.792309155 | 1.814487667 | 0.070266317 |
| RP11-755B10.4 | -0.108807937 | 2.254611854 | -1.814456625 | 0.070271101 |
| GYS2 | -0.216790263 | -2.070309265 | -1.814282475 | 0.070297942 |
| NHLRC2 | 0.080984132 | 4.37372138 | 1.814218937 | 0.070307737 |
| GS1-44D20.1 | -0.173294077 | 6.7082115 | -1.814120167 | 0.070322966 |
| PPARG | 0.455738267 | -0.292688947 | 1.813593034 | 0.070404287 |
| TLR8 | -0.449929032 | 3.783841871 | -1.813591415 | 0.070404537 |
| RP4-613B23.1 | -0.191009818 | 0.712340663 | -1.813306986 | 0.070448448 |
| TTC22 | -0.24914156 | -0.19043661 | -1.812979109 | 0.070499095 |
| RP11-283G6.3 | 0.303783344 | -0.928702646 | 1.812923718 | 0.070507654 |
| SLX4 | 0.084136756 | 3.592540079 | 1.812234325 | 0.070614253 |
| FAM60A | 0.115302968 | 5.977701846 | 1.812029634 | 0.070645929 |
| F8A1 | 0.169123804 | 5.541218906 | 1.811872772 | 0.070670211 |
| FITM1 | -0.144721447 | 0.892080001 | -1.811855701 | 0.070672854 |
| BLOC1S6 | 0.055527026 | 5.832704024 | 1.811613163 | 0.070710414 |
| RP11-420K14.6 | 0.181467829 | -0.723690594 | 1.810372077 | 0.070902868 |
| MMP19 | -0.421241781 | 2.026311117 | -1.80943272 | 0.07104882 |
| RPS4XP6 | 0.131100535 | 4.008276059 | 1.808776282 | 0.07115096 |
| STOML3 | -0.332302498 | -2.299827136 | -1.807668057 | 0.071323671 |
| GOLGA6L18 | 0.226792011 | -2.792254574 | 1.807346333 | 0.071373875 |
| RP1-95L4.4 | -0.091575065 | 3.704983573 | -1.80691701 | 0.071440914 |
| 9-Mar | 0.158483899 | 3.131266033 | 1.806776939 | 0.071462797 |
| KDELC2 | 0.147678636 | 4.658173297 | 1.806561228 | 0.071496509 |
| RPL18 | 0.095664241 | 9.213773093 | 1.806156967 | 0.071559722 |
| LILRA4 | -0.348324546 | -0.024569509 | -1.805955937 | 0.071591174 |
| FAM210B | -0.131195388 | 5.969057706 | -1.805859986 | 0.07160619 |
| EPB41L3 | -0.449673073 | 0.950650017 | -1.80578218 | 0.071618368 |
| SESN3 | 0.235809316 | 5.08555643 | 1.805737629 | 0.071625342 |
| RNVU1-20 | -0.227340541 | 2.18803707 | -1.80567623 | 0.071634954 |
| PLS3 | 0.434252529 | -1.321573887 | 1.805268899 | 0.071698749 |
| SMARCB1 | 0.074792448 | 7.10285312 | 1.805073584 | 0.071729355 |
| METTL2A | -0.079122264 | 3.662703019 | -1.804873643 | 0.071760697 |
| SDC1 | 0.31302016 | -0.214213117 | 1.80468221 | 0.071790716 |
| RP11-212I21.2 | -0.296573034 | -1.000640716 | -1.80426929 | 0.071855502 |
| DBIL5P | -0.180086368 | -0.108385369 | -1.804255648 | 0.071857644 |
| RP1-202O8.3 | -0.172689312 | 0.058509909 | -1.804249745 | 0.07185857 |
| THAP5P1 | -0.154831904 | 0.969039197 | -1.80394642 | 0.071906193 |
| IL3RA | -0.2243803 | 6.692122264 | -1.803725648 | 0.071940872 |
| LAPTM4A | 0.101592862 | 8.382939073 | 1.803537769 | 0.071970394 |
| DTD1 | 0.161604951 | 5.076992913 | 1.803392318 | 0.071993257 |
| C9orf91 | 0.168074093 | 4.236554616 | 1.803288737 | 0.072009542 |
| TMEM154 | -0.209157464 | 4.47871226 | -1.803270273 | 0.072012445 |
| RP11-574O7.1 | -0.284578738 | -1.734515729 | -1.803027077 | 0.072050693 |
| NAPSB | -0.46035334 | 4.240915891 | -1.802911631 | 0.072068855 |
| IGHJ1P | 0.349521774 | 0.531050745 | 1.802850278 | 0.072078509 |
| CD44 | -0.135206722 | 8.944675609 | -1.802444805 | 0.072142335 |
| WASIR1 | 0.337579316 | -1.010308758 | 1.802287774 | 0.072167067 |
| IGHV3-53 | 0.363850546 | 2.105602331 | 1.801994678 | 0.072213246 |
| MAN2B1 | -0.113396197 | 7.484836814 | -1.801716623 | 0.072257078 |
| TRBV4-1 | -0.269898925 | 0.519054513 | -1.801258097 | 0.072329406 |
| RP4-583P15.10 | 0.197482667 | 1.558542492 | 1.800560487 | 0.072439562 |
| RP4-816N1.6 | -0.131158665 | 1.209806846 | -1.800507046 | 0.072448006 |
| RPS20P35 | 0.129029928 | 2.31250733 | 1.800352025 | 0.072472506 |
| CTD-2589O24.1 | 0.186684938 | -0.173207284 | 1.799911995 | 0.072542085 |
| FOLR3 | 0.443021433 | 3.316870605 | 1.799868061 | 0.072549035 |
| RP11-624L12.1 | 0.126263141 | 2.127997492 | 1.799798557 | 0.072560031 |
| ATP13A3-DT | -0.215326719 | 1.301487876 | -1.799644408 | 0.072584424 |
| ANXA2 | -0.257651009 | 5.757897488 | -1.79941122 | 0.072621337 |
| SCAF11 | -0.043391929 | 6.670439192 | -1.799284183 | 0.072641453 |
| CFH | -0.396889897 | 1.549266677 | -1.799261358 | 0.072645067 |
| HES7 | -0.239106282 | -1.642289443 | -1.799172286 | 0.072659175 |
| RP11-192N10.2 | 0.224250258 | 1.486013622 | 1.79893311 | 0.072697068 |
| RPL7 | 0.116605482 | 7.449674476 | 1.798268469 | 0.072802453 |
| RPL15P2 | 0.137414244 | 4.029060041 | 1.797956812 | 0.072851912 |
| COL4A3 | -0.301476905 | -2.751283016 | -1.79773345 | 0.072887376 |
| HTT | -0.065343515 | 5.92963557 | -1.797698893 | 0.072892864 |
| RP11-160A9.3 | 0.240094708 | -0.463366187 | 1.797632744 | 0.07290337 |
| C21orf119 | 0.134888387 | 3.846711459 | 1.797265761 | 0.072961678 |
| SERPINI1 | -0.206598914 | 2.250789737 | -1.797166363 | 0.072977478 |
| RP11-118B22.4 | -0.24952001 | 0.333513537 | -1.797129787 | 0.072983292 |
| VDAC1P3 | -0.198981626 | 0.09248793 | -1.796821855 | 0.07303226 |
| SFRP5 | 0.336907474 | -0.926019876 | 1.79630845 | 0.073113961 |
| AC062029.1 | 0.138131137 | 1.492530549 | 1.796307867 | 0.073114054 |
| H2AFX | 0.147809704 | 7.043016198 | 1.796109953 | 0.07314557 |
| LGALS12 | 0.325929978 | 4.157144561 | 1.796016023 | 0.073160531 |
| SLC35G1 | 0.328801089 | 0.093080495 | 1.795971505 | 0.073167623 |
| CTD-3088G3.8 | -0.305080479 | 2.213423506 | -1.795510934 | 0.073241025 |
| RP11-26H16.1 | 0.172745305 | -0.053456429 | 1.794661333 | 0.073376586 |
| TGFB1 | 0.101013268 | 9.258444408 | 1.794462952 | 0.073408269 |
| GBP4 | 0.268647333 | 5.301593886 | 1.794320168 | 0.073431079 |
| NPM1 | -0.120683146 | 8.624567046 | -1.794208508 | 0.073448922 |
| GABARAP | 0.116445308 | 4.953174978 | 1.793748052 | 0.073522537 |
| IFNG-AS1 | -0.259705993 | -1.359092844 | -1.793549089 | 0.073554365 |
| PRPF19 | -0.092290089 | 7.69668311 | -1.793504561 | 0.073561489 |
| AL162151.3 | -0.170364098 | 7.33461175 | -1.792756791 | 0.07368122 |
| RP11-472I20.1 | 0.132980802 | 3.067405924 | 1.792702863 | 0.073689861 |
| PRSS50 | -0.249570977 | -2.692392843 | -1.792341439 | 0.073747793 |
| EPB41L1 | 0.273292038 | -1.834865043 | 1.792143243 | 0.073779578 |
| ZC2HC1A | -0.331829781 | 1.103319509 | -1.791303894 | 0.073914309 |
| MMP17 | -0.327185756 | 2.650297565 | -1.790675555 | 0.074015302 |
| ZNF84 | -0.19074565 | -1.458443324 | -1.790268765 | 0.074080745 |
| EEF1B2P2 | 0.130524348 | 3.587821589 | 1.789987472 | 0.074126026 |
| AC007036.4 | 0.226873729 | 0.697897898 | 1.789851049 | 0.074147995 |
| RPS2P55 | 0.100022894 | 5.750476011 | 1.789591244 | 0.074189848 |
| RP6-109B7.3 | 0.15707158 | 3.335138163 | 1.789331984 | 0.074231632 |
| AC079630.4 | -0.277047185 | 0.031044881 | -1.788802616 | 0.074317009 |
| GPR12 | -0.48871384 | -1.905982279 | -1.788552275 | 0.074357412 |
| ARHGEF5 | 0.298914989 | 0.195529517 | 1.788498255 | 0.074366132 |
| TBL3 | -0.065206756 | 4.239971013 | -1.787938004 | 0.074456626 |
| ETV2 | 0.208562601 | -0.339227522 | 1.787877314 | 0.074466435 |
| CYP2S1 | -0.433277287 | 2.571240239 | -1.787660732 | 0.074501446 |
| PFDN1 | 0.066474048 | 6.567647142 | 1.787632929 | 0.074505941 |
| CBWD6 | -0.115152093 | 1.072384496 | -1.78762457 | 0.074507293 |
| SEPT6 | -0.157276336 | 8.102289132 | -1.787553376 | 0.074518806 |
| RNA5-8S5 | 0.255310732 | 0.150185751 | 1.787320539 | 0.074556467 |
| HCN2 | -0.224626029 | 0.663877379 | -1.787251722 | 0.074567601 |
| RWDD3 | 0.093011403 | 2.795850514 | 1.78710754 | 0.074590933 |
| IGHG4 | 0.486491308 | 4.186564591 | 1.786541355 | 0.074682612 |
| IGF1R | 0.268219096 | 3.990695515 | 1.785668489 | 0.074824132 |
| DGKH | 0.238903036 | 1.785451111 | 1.785541696 | 0.074844708 |
| PHLDB3 | 0.148195266 | 2.750404853 | 1.785497035 | 0.074851956 |
| BOC | 0.292283937 | -4.654392323 | 1.785474138 | 0.074855673 |
| KIF13B | 0.101271947 | 4.305630698 | 1.785451598 | 0.074859331 |
| C19orf24 | -0.068320362 | 4.839430865 | -1.785238125 | 0.074893989 |
| RP11-757G1.6 | -0.322054794 | 1.431439264 | -1.785157951 | 0.074907009 |
| RP11-550F7.1 | -0.117862446 | 5.249027697 | -1.784896587 | 0.074949465 |
| OLFM2 | -0.241520895 | -1.17725974 | -1.784875164 | 0.074952946 |
| MRPL39 | 0.08012146 | 5.556074016 | 1.784615203 | 0.074995196 |
| LINC01013 | -0.241335182 | -3.775302301 | -1.784308524 | 0.075045064 |
| C1orf95 | 0.255671943 | -4.610366578 | 1.784089615 | 0.075080677 |
| EEF1B2P3 | -0.095824466 | 10.10280973 | -1.784048898 | 0.075087303 |
| TRBJ2-2P | 0.266199726 | 1.127852404 | 1.783931557 | 0.075106399 |
| GTSF1 | -0.567386007 | 2.603761713 | -1.783886136 | 0.075113792 |
| SETP20 | 0.149156595 | 1.315530871 | 1.783491095 | 0.075178116 |
| HTATIP2 | -0.173617547 | 5.53881099 | -1.782588702 | 0.075325222 |
| HTRA3 | 0.392679492 | 3.831114478 | 1.782219701 | 0.075385444 |
| HOTTIP | -0.346981639 | -4.107984115 | -1.782090986 | 0.075406459 |
| ZNF853 | 0.227726213 | 1.848730258 | 1.781873405 | 0.075441995 |
| RPS26P11 | 0.220546706 | 1.380303129 | 1.781870664 | 0.075442443 |
| DNTTIP1 | -0.084995895 | 6.422063593 | -1.781732118 | 0.075465078 |
| PGBD5 | -0.316569652 | -2.513308019 | -1.781257356 | 0.075542685 |
| RP11-318C24.2 | -0.195981051 | 0.990250051 | -1.78114486 | 0.075561084 |
| SLC31A2 | -0.183101425 | 5.278340856 | -1.781001532 | 0.07558453 |
| ACOT11 | -0.208945961 | 2.355236322 | -1.780754077 | 0.075625025 |
| AP004290.1 | -0.205602125 | -1.054155801 | -1.77992606 | 0.075760654 |
| RP11-754N21.1 | -0.319541705 | -1.746814419 | -1.779766359 | 0.075786836 |
| LA16c-60G3.7 | -0.154330317 | 0.060347684 | -1.779373435 | 0.075851284 |
| SGPL1 | -0.116061251 | 4.749080104 | -1.778553085 | 0.075985986 |
| CKM | -0.356811279 | 1.03958668 | -1.778349679 | 0.076019415 |
| HNRNPA1P54 | 0.190637361 | -2.47355125 | 1.778334086 | 0.076021978 |
| SNX22 | 0.245310901 | 0.541079365 | 1.777753671 | 0.076117437 |
| SCN9A | -0.541118337 | 0.194811652 | -1.777675213 | 0.076130349 |
| HCAR2 | 0.307615067 | 4.215267733 | 1.777445144 | 0.07616822 |
| PRRC2A | -0.074119848 | 8.019542448 | -1.777386995 | 0.076177794 |
| YPEL1 | -0.149368395 | 3.946375681 | -1.77715434 | 0.07621611 |
| NAPB | 0.088264695 | 4.498431453 | 1.777045175 | 0.076234094 |
| PPAPDC3 | -0.398596879 | -0.616879166 | -1.776798759 | 0.076274702 |
| RP11-141M1.3 | -0.267686199 | -1.394478862 | -1.776581325 | 0.076310549 |
| TRIM74 | 0.220717596 | 1.655944646 | 1.776504906 | 0.076323151 |
| KB-208E9.1 | -0.211288049 | 0.930616858 | -1.776031265 | 0.076401294 |
| SIRPA | -0.209123422 | 6.811619267 | -1.775699569 | 0.076456058 |
| CD2 | -0.262586879 | 4.526590385 | -1.775655652 | 0.076463311 |
| PHF20 | -0.071472648 | 5.458198606 | -1.775535388 | 0.076483176 |
| RP11-616M22.5 | -0.493016929 | 1.537395323 | -1.775342272 | 0.076515084 |
| INSRR | 0.323723248 | -2.911401185 | 1.775282011 | 0.076525043 |
| SHROOM3 | 0.333828964 | -5.594722338 | 1.775062555 | 0.07656132 |
| LINC00173 | 0.256307416 | 3.310760681 | 1.774942466 | 0.076581177 |
| NDUFV2P1 | -0.071207702 | 6.375269049 | -1.774931483 | 0.076582993 |
| TCTEX1D4 | 0.206612297 | 0.248486083 | 1.774905183 | 0.076587343 |
| MT-ND1 | 0.058204727 | 13.08720205 | 1.774458495 | 0.076661246 |
| IGLV2-23 | 0.366633148 | 2.870442244 | 1.773366374 | 0.076842181 |
| VGLL3 | 0.377397114 | -4.114160067 | 1.773211949 | 0.076867793 |
| LGALS9B | 0.195249855 | -0.05895404 | 1.773042675 | 0.076895876 |
| SNX18P3 | 0.391436649 | 1.219985256 | 1.772250487 | 0.077027413 |
| SHC1P2 | 0.156548912 | 0.093723932 | 1.7717421 | 0.077111924 |
| PHF2P2 | 0.220498625 | -2.338189612 | 1.7710997 | 0.077218821 |
| SSRP1 | 0.081872089 | 7.028625384 | 1.770903072 | 0.077251564 |
| LINC00338 | -0.13202416 | 3.231599727 | -1.770851525 | 0.07726015 |
| CBWD7 | -0.135709995 | 0.330080686 | -1.770768322 | 0.07727401 |
| HIST4H4 | 0.18569425 | 1.426073233 | 1.77050577 | 0.07731776 |
| CTD-2547L24.3 | -0.230410165 | 0.002974816 | -1.770389244 | 0.077337183 |
| HIGD1AP1 | 0.176012733 | 3.247871606 | 1.770309628 | 0.077350457 |
| IGHG3 | 0.444711741 | 5.02072808 | 1.770260845 | 0.07735859 |
| CCR5 | -0.35710836 | 2.513181309 | -1.770174894 | 0.077372923 |
| CTD-2562J15.6 | -0.237467578 | -0.333035734 | -1.770104069 | 0.077384735 |
| RP11-314A20.1 | -0.122557883 | 7.342769679 | -1.769961424 | 0.07740853 |
| LINC02910 | -0.251230211 | 3.232168106 | -1.769777319 | 0.077439249 |
| AC007246.3 | 0.188843347 | 0.601923939 | 1.769715799 | 0.077449517 |
| RP11-529F4.1 | -0.195583309 | -0.336178342 | -1.769318235 | 0.077515895 |
| TRGJ2 | -0.241486643 | 0.351043255 | -1.76929448 | 0.077519862 |
| RP11-475N22.4 | 0.305047545 | 2.547467384 | 1.768728404 | 0.077614459 |
| HIST1H4I | -0.198813609 | 1.16825527 | -1.768394019 | 0.077670383 |
| FANCD2P2 | 0.214946251 | -0.94174824 | 1.768191387 | 0.077704288 |
| RP5-884M6.1 | 0.369765494 | -3.012389805 | 1.768110823 | 0.077717771 |
| PITRM1 | -0.07108409 | 6.113005872 | -1.767828011 | 0.077765119 |
| TSSK6 | -0.124420847 | 2.158365263 | -1.767713806 | 0.077784245 |
| DUT | 0.09698505 | 6.206789819 | 1.767612677 | 0.077801185 |
| MIR663A | 0.256797514 | -2.559721306 | 1.767607155 | 0.07780211 |
| PPP2R2B | -0.255832992 | -0.621009746 | -1.767199027 | 0.077870506 |
| HIST1H1D | -0.348328674 | 2.816961022 | -1.767152495 | 0.077878307 |
| PSMD10P2 | -0.187521856 | -0.426799341 | -1.766919659 | 0.077917352 |
| TGM3 | 0.444609992 | -0.097168586 | 1.766664618 | 0.077960138 |
| CAPNS1 | -0.07747736 | 8.887823554 | -1.766408717 | 0.078003089 |
| RP11-278C7.1 | -0.090094522 | 5.495770899 | -1.766209382 | 0.078036558 |
| AC002398.5 | 0.35379551 | -1.844170585 | 1.765657538 | 0.078129278 |
| PPP1R12B | -0.085530696 | 4.255484143 | -1.765573749 | 0.078143364 |
| RP11-464D20.2 | 0.090266629 | 5.786401022 | 1.765391706 | 0.078173975 |
| LRRC48 | -0.224128168 | -2.429220246 | -1.765013089 | 0.078237671 |
| RPS15AP5 | 0.114238472 | 3.250799584 | 1.764905769 | 0.078255733 |
| DPP4 | -0.280594542 | 1.850248084 | -1.764655907 | 0.078297799 |
| RAD9A | 0.070308036 | 4.786106018 | 1.764464932 | 0.078329964 |
| MVB12B | 0.169956164 | 3.973637087 | 1.764164926 | 0.078380514 |
| RPS10P3 | 0.12071418 | 4.264624964 | 1.764023899 | 0.078404286 |
| STK4 | 0.074040105 | 6.779886879 | 1.763601175 | 0.078475576 |
| UBE2Q2P3 | 0.218677732 | -1.544164523 | 1.763556162 | 0.07848317 |
| LINC00398 | -0.238931824 | -0.672479822 | -1.762816486 | 0.07860805 |
| PDLIM5 | -0.162497238 | 4.020356291 | -1.762478103 | 0.078665233 |
| RP4-794H19.1 | -0.238108571 | -0.528185956 | -1.761924253 | 0.078758901 |
| DUSP14 | 0.15824364 | 4.212972665 | 1.761767351 | 0.078785454 |
| RP11-267J23.4 | 0.106330314 | 5.494151886 | 1.761624345 | 0.078809661 |
| AC018462.2 | -0.231167575 | -1.667608534 | -1.761143649 | 0.078891073 |
| HRH4 | -0.278686846 | 0.075108825 | -1.76103866 | 0.078908864 |
| DCAF6 | -0.080231061 | 5.441007251 | -1.760687338 | 0.07896842 |
| ST13P18 | -0.083531998 | 5.356265393 | -1.760473518 | 0.079004685 |
| RP1-273G13.2 | -0.116944088 | 4.04790232 | -1.76042296 | 0.079013262 |
| RP11-58H15.1 | 0.202660368 | -0.566165779 | 1.760272316 | 0.079038822 |
| STMN3 | -0.167502892 | 6.406328275 | -1.760248905 | 0.079042794 |
| UCKL1 | -0.058167955 | 5.891595224 | -1.760177186 | 0.079054966 |
| SLC22A13 | 0.183686882 | -1.636195012 | 1.760078075 | 0.079071789 |
| AC107983.4 | 0.168389055 | 3.063939123 | 1.760071008 | 0.079072989 |
| RP11-119H12.4 | 0.163307557 | -0.443316769 | 1.759579394 | 0.079156479 |
| SERGEF | -0.090942073 | 2.969490447 | -1.759406808 | 0.079185806 |
| RPL10AP6 | -0.108567425 | 9.080805178 | -1.75927431 | 0.079208327 |
| ADCK2 | 0.101276352 | 4.734828543 | 1.758998878 | 0.079255159 |
| RPL34P34 | 0.100939727 | 3.199011497 | 1.758949433 | 0.079263569 |
| FKBP9 | -0.298422212 | 2.07963666 | -1.758815788 | 0.079286303 |
| MAP2K1 | 0.13330086 | 5.921469383 | 1.758369107 | 0.079362326 |
| NPIPB8 | 0.157985695 | 1.109246108 | 1.757781947 | 0.079462348 |
| RCC2 | 0.101069008 | 8.118046901 | 1.757702903 | 0.079475821 |
| RP11-296K13.4 | -0.186714975 | -0.000710509 | -1.757455881 | 0.079517938 |
| SLITRK5 | 0.470378114 | -0.607481029 | 1.757317481 | 0.079541542 |
| HDAC8 | -0.059852097 | 3.955181135 | -1.7568614 | 0.079619369 |
| CTA-384D8.31 | 0.313813441 | -0.009187071 | 1.756679486 | 0.079650429 |
| EIF3EP1 | -0.108067281 | 4.705276035 | -1.756674579 | 0.079651267 |
| GS1-57L11.1 | 0.406513026 | 0.51774595 | 1.75653288 | 0.079675468 |
| AC005932.1 | -0.319131465 | 2.353536616 | -1.756432641 | 0.079692591 |
| F2RL1 | 0.368644814 | 2.550609171 | 1.756221549 | 0.079728661 |
| PSMD10P1 | 0.247355925 | -0.401522724 | 1.755989233 | 0.079768372 |
| DNAJC12 | -0.371459079 | 0.880709904 | -1.755436871 | 0.079862856 |
| TPTEP1 | 0.470142805 | 0.098392857 | 1.755360867 | 0.079875864 |
| RP11-712L6.5 | -0.202633237 | 0.5627207 | -1.75533101 | 0.079880974 |
| ACTA2-AS1 | -0.214051812 | -0.027704565 | -1.755294139 | 0.079887286 |
| RP11-1023L17.2 | -0.269808625 | -2.513789014 | -1.754825323 | 0.07996757 |
| CEP170 | 0.082592905 | 5.429302499 | 1.754675887 | 0.079993175 |
| SH3TC1 | 0.18621815 | 4.545854493 | 1.754302198 | 0.080057233 |
| AC083884.8 | -0.228637287 | -2.44487866 | -1.753525781 | 0.08019046 |
| AQP4-AS1 | -0.253058864 | -3.156912111 | -1.753269465 | 0.080234482 |
| TBXA2R | -0.344858495 | 1.451995741 | -1.753081365 | 0.0802668 |
| AC018804.7 | 0.087381745 | 3.242125044 | 1.752853205 | 0.080306016 |
| RNF175 | -0.352850639 | -0.571103661 | -1.752477542 | 0.080370618 |
| PSMD3 | 0.050848176 | 6.648969156 | 1.752354282 | 0.080391823 |
| WFDC3 | -0.169289665 | -1.392243108 | -1.752341875 | 0.080393958 |
| RPL29P12 | 0.143832709 | 1.903261609 | 1.752172297 | 0.080423141 |
| AMPD2 | 0.098003442 | 6.722553051 | 1.7516754 | 0.080508701 |
| USP32P1 | -0.497610624 | -1.178931501 | -1.751380979 | 0.080559433 |
| RAB40B | -0.165062137 | 1.286214333 | -1.750781837 | 0.08066275 |
| RPL9P18 | 0.154646332 | 1.809975355 | 1.750261743 | 0.080752524 |
| C16orf93 | -0.152051961 | 4.609445478 | -1.750162429 | 0.080769676 |
| RP11-830F9.6 | -0.238292273 | -0.372739075 | -1.750153707 | 0.080771183 |
| PHF11 | -0.081883747 | 5.613621158 | -1.750115944 | 0.080777705 |
| AL163953.3 | -0.198538677 | 0.416955047 | -1.749936897 | 0.080808638 |
| RPL21P3 | 0.185842486 | 2.920666185 | 1.749813108 | 0.080830029 |
| HNRNPA1 | 0.121898858 | 8.729536196 | 1.748871337 | 0.080992924 |
| RPS7 | 0.099868017 | 6.694740643 | 1.748836393 | 0.080998973 |
| SHPK | -0.123075403 | 0.818824087 | -1.748749959 | 0.081013937 |
| LRRC1 | -0.126394185 | 1.978215994 | -1.74863691 | 0.081033513 |
| ZNF764 | 0.081144539 | 3.902494099 | 1.748544935 | 0.081049443 |
| AF064858.11 | -0.273582847 | 1.442443935 | -1.748386207 | 0.081076939 |
| LRP1B | -0.383339554 | -5.494330771 | -1.748209234 | 0.081107605 |
| NUFIP2 | 0.077715383 | 7.149828321 | 1.748140849 | 0.081119457 |
| CDK4 | -0.128194631 | 6.251405716 | -1.74777349 | 0.081183151 |
| HPD | 0.353603297 | -0.594165751 | 1.747368136 | 0.08125348 |
| MYO5A | 0.103535846 | 5.573456665 | 1.747351041 | 0.081256448 |
| RBMXP4 | 0.091180398 | 2.880367579 | 1.747281412 | 0.081268534 |
| GOLGA8EP | -0.291311229 | -2.102715546 | -1.747121311 | 0.081296329 |
| AC008753.3 | 0.144893963 | 1.484376933 | 1.747119053 | 0.081296721 |
| hsa-mir-8072 | 0.1441408 | 1.797616171 | 1.746997274 | 0.081317869 |
| TFF3 | 0.387036011 | -0.349350069 | 1.746868285 | 0.081340273 |
| PRPS2 | -0.09457653 | 6.169990123 | -1.746457324 | 0.081411688 |
| EIF3F | 0.071524633 | 6.370908087 | 1.74627383 | 0.081443591 |
| KIF26A | 0.371451956 | 0.287339677 | 1.746150965 | 0.081464958 |
| AC090602.2 | 0.122185081 | 1.692441025 | 1.745162048 | 0.081637108 |
| AGPAT9 | 0.280496174 | 4.107243061 | 1.745029014 | 0.081660289 |
| RBMS3 | 0.333254876 | -3.253049368 | 1.744835903 | 0.081693947 |
| KDM1A | -0.07483356 | 6.510907178 | -1.74475339 | 0.081708333 |
| FOXP3 | 0.221210542 | 0.572862074 | 1.744705932 | 0.081716608 |
| IDS | -0.10606783 | 6.559472216 | -1.744660338 | 0.081724558 |
| B3GAT3 | -0.062956953 | 5.949592825 | -1.744398584 | 0.081770213 |
| NPIPA5 | 0.184445722 | 3.850433849 | 1.743905311 | 0.081856306 |
| S100B | -0.36591069 | 3.102607787 | -1.743706616 | 0.081891006 |
| FAM195B | 0.090197379 | 2.986562289 | 1.743702094 | 0.081891796 |
| AC002075.4 | 0.122027137 | 2.233060121 | 1.743376091 | 0.081948755 |
| CCDC155 | 0.327230732 | -3.465854121 | 1.742852072 | 0.082040379 |
| ATE1 | -0.054944041 | 4.87647221 | -1.742727664 | 0.082062144 |
| CSE1L | -0.091372469 | 6.257150631 | -1.742609606 | 0.082082802 |
| COBL | -0.485418811 | -4.273550492 | -1.742447702 | 0.08211114 |
| FGF18 | 0.242984837 | -1.381327833 | 1.742397869 | 0.082119864 |
| PDE1C | 0.343749038 | -4.678000938 | 1.741974251 | 0.082194052 |
| CAPZA1 | -0.092272351 | 7.715166596 | -1.741904136 | 0.082206337 |
| RPL11 | 0.061402945 | 11.57449298 | 1.741757687 | 0.082232 |
| FAM127A | -0.310972438 | 5.003815687 | -1.74150792 | 0.082275784 |
| LSP1 | 0.245380084 | 7.834264056 | 1.741501145 | 0.082276972 |
| BNC2 | 0.298992168 | -2.895592299 | 1.740285132 | 0.082490411 |
| PRC1-AS1 | -0.153414124 | 1.003416381 | -1.740121776 | 0.082519118 |
| RP5-968J1.1 | -0.221765782 | 0.181980268 | -1.740064871 | 0.08252912 |
| RP11-620J15.3 | -0.194671977 | 6.644427917 | -1.73993566 | 0.082551835 |
| SERPINB10 | 0.418862948 | 4.405751794 | 1.739656834 | 0.082600869 |
| RP11-1036E20.7 | -0.297708545 | -1.628805852 | -1.739610042 | 0.0826091 |
| MARK4 | 0.078400563 | 4.881534859 | 1.739558363 | 0.082618191 |
| RP11-438L7.3 | -0.075783405 | 5.035349474 | -1.739270704 | 0.082668812 |
| RP4-647C14.3 | 0.18682878 | 2.898277264 | 1.73919896 | 0.082681441 |
| ZNF487 | 0.120603755 | 2.632658065 | 1.73913281 | 0.082693086 |
| RP11-188D8.1 | -0.190966946 | 0.731224254 | -1.738928146 | 0.082729126 |
| PTGES3P2 | 0.216779049 | 1.78368379 | 1.738808442 | 0.082750211 |
| MOV10 | 0.108631013 | 5.347321637 | 1.738550605 | 0.082795641 |
| RP11-288E14.2 | 0.062586441 | 4.93328069 | 1.738137642 | 0.082868446 |
| MAGOHB | -0.087334453 | 4.078265895 | -1.738078545 | 0.08287887 |
| AC010468.1 | -0.110425819 | 6.710117907 | -1.737840441 | 0.082920875 |
| TRAV3 | -0.244018765 | -0.358889002 | -1.737713757 | 0.082943232 |
| GOT2P2 | -0.182402614 | -1.021855061 | -1.737290033 | 0.083018043 |
| RPS26P6 | 0.207525869 | 2.785669731 | 1.737261209 | 0.083023134 |
| SCAMP2 | -0.071170262 | 6.714222142 | -1.737179315 | 0.0830376 |
| RP11-445H22.3 | -0.226232701 | 0.027764883 | -1.736865006 | 0.08309314 |
| HERC2P10 | 0.213412379 | 2.894344623 | 1.736559204 | 0.083147205 |
| IGHV5-51 | 0.364720194 | 2.938660752 | 1.736123315 | 0.083224319 |
| FAM21D | 0.114940226 | 3.307017145 | 1.736037372 | 0.083239531 |
| ZMYND10 | 0.172834402 | 0.677066789 | 1.735922288 | 0.083259903 |
| GCNT1P1 | -0.203879693 | -1.298083637 | -1.735709808 | 0.083297528 |
| ODF2L | 0.096800603 | 4.458843569 | 1.735399048 | 0.083352579 |
| WDR86 | -0.39498708 | -0.077953943 | -1.735206835 | 0.083386645 |
| PTPRM | 0.387716588 | 1.451460506 | 1.735140385 | 0.083398425 |
| RN7SL288P | 0.226445762 | 0.921315668 | 1.734931139 | 0.083435527 |
| RP4-764D2.1 | -0.142101046 | 0.281007052 | -1.73439399 | 0.083530831 |
| RP11-973H7.1 | -0.269418337 | -0.637650068 | -1.734056765 | 0.083590708 |
| GYPE | 0.374238734 | 0.776271087 | 1.734045422 | 0.083592723 |
| WNK4 | 0.233998477 | -2.817100307 | 1.733955693 | 0.083608662 |
| GNGT2 | -0.254065438 | 1.963302501 | -1.73381615 | 0.083633453 |
| TICAM2 | -0.245849961 | -1.420459134 | -1.733414307 | 0.08370488 |
| CD14 | -0.480666174 | 5.939072202 | -1.73324385 | 0.083735193 |
| DNAH2 | 0.458462018 | -4.464572834 | 1.733130118 | 0.083755424 |
| TAGLN2 | -0.104113096 | 10.38002664 | -1.733068818 | 0.083766329 |
| RP11-1348G14.1 | 0.128345042 | 2.555196921 | 1.732994587 | 0.083779537 |
| AL359195.1 | -0.38860623 | -0.912556554 | -1.732516967 | 0.083864558 |
| BRAT1 | 0.070669432 | 5.23634224 | 1.731987174 | 0.083958949 |
| AC144652.1 | 0.138564502 | 2.962537649 | 1.731835862 | 0.083985924 |
| RP11-667K14.8 | -0.180868404 | -0.193076945 | -1.731584561 | 0.084030739 |
| CALM3 | 0.08445917 | 8.866833468 | 1.731228727 | 0.084094229 |
| RP11-555H23.1 | -0.186865895 | -0.92489246 | -1.730300445 | 0.084260041 |
| NDUFC1 | -0.067725819 | 5.734446644 | -1.730277754 | 0.084264098 |
| RAPGEF4 | 0.277642714 | -1.881694363 | 1.730132121 | 0.084290136 |
| CCDC28A | 0.068663069 | 6.031533729 | 1.730128744 | 0.08429074 |
| C1orf200 | -0.210116664 | 1.04549475 | -1.729973193 | 0.084318559 |
| PTGER3 | -0.426225586 | -1.829712119 | -1.729127074 | 0.084470012 |
| KDM3B | -0.055654673 | 6.723157083 | -1.728644662 | 0.084556461 |
| TDRD9 | -0.403289186 | 1.584022884 | -1.728529634 | 0.084577085 |
| RP11-75C10.7 | -0.228494659 | 0.065869745 | -1.728525314 | 0.08457786 |
| ZBTB37 | -0.086588491 | 4.598059781 | -1.728445234 | 0.08459222 |
| MPPED2 | 0.406770676 | -3.168177665 | 1.728344114 | 0.084610356 |
| RNA5SP464 | 0.207110868 | 1.503633796 | 1.728281646 | 0.084621562 |
| IL2RB | 0.260046761 | 4.079519218 | 1.727697137 | 0.084726469 |
| MIA3 | -0.056041107 | 5.636546804 | -1.727530563 | 0.084756384 |
| LAMC2 | 0.347967923 | -4.601377054 | 1.726817865 | 0.084884478 |
| SLC44A2 | 0.193531676 | 6.251613592 | 1.726660686 | 0.084912749 |
| TNFAIP8L3 | 0.329216335 | -1.216868194 | 1.72653188 | 0.084935922 |
| KB-318B8.7 | 0.165802698 | 2.982742329 | 1.726421749 | 0.08495574 |
| RP11-425D10.10 | -0.186743212 | 2.526110348 | -1.726407967 | 0.08495822 |
| KCNJ2 | -0.382478143 | 0.3814179 | -1.726102955 | 0.085013127 |
| CTB-131K11.1 | -0.104832086 | 2.532997892 | -1.725813871 | 0.085065193 |
| CDCA7L | -0.164303198 | 6.745762383 | -1.725485007 | 0.085124455 |
| DPH1 | 0.102899464 | 4.379726033 | 1.725368739 | 0.085145414 |
| GFRA2 | -0.292624508 | -1.132795645 | -1.725102005 | 0.085193515 |
| RP11-782C8.3 | -0.366463743 | 0.717092105 | -1.724571262 | 0.085289289 |
| IGLV3-21 | 0.374262644 | 3.112061971 | 1.724368101 | 0.085325974 |
| RP1-228H13.1 | 0.159626922 | 0.05461614 | 1.723367507 | 0.085506835 |
| KIAA2026 | -0.053174731 | 5.553763551 | -1.723327774 | 0.085514024 |
| SNED1 | 0.285644356 | 1.125162184 | 1.723273569 | 0.085523831 |
| ARHGAP29 | 0.387107312 | -2.811296942 | 1.723126952 | 0.085550363 |
| TMEM109 | -0.095937033 | 7.096853917 | -1.723078987 | 0.085559044 |
| MACF1 | -0.102907571 | 5.630157612 | -1.722765061 | 0.08561588 |
| CTC-534B23.1 | -0.131362018 | 2.014579091 | -1.722624487 | 0.08564134 |
| BRD7P2 | -0.049561344 | 6.353935698 | -1.722397171 | 0.085682525 |
| NUP85 | 0.060770583 | 5.19139905 | 1.722151198 | 0.085727107 |
| RPL24 | 0.096047609 | 8.623304209 | 1.722092379 | 0.085737771 |
| C9orf96 | -0.165606133 | -0.573002915 | -1.72203649 | 0.085747904 |
| ZBTB46 | -0.26609453 | 2.840993628 | -1.72178597 | 0.085793339 |
| MPG | -0.101913016 | 7.251130513 | -1.721642607 | 0.085819348 |
| IGHV3OR16-9 | 0.236296487 | -0.269844201 | 1.721491249 | 0.085846815 |
| TRBV27 | -0.405248635 | 4.716618048 | -1.721366496 | 0.085869459 |
| PEX1 | -0.064387668 | 4.019824415 | -1.721102076 | 0.085917471 |
| VWA8 | -0.11193133 | 4.209154821 | -1.721000382 | 0.085935941 |
| RP11-445N18.3 | 0.175341362 | 0.383598954 | 1.720808073 | 0.085970879 |
| NOL6 | -0.075625741 | 5.399410023 | -1.720780941 | 0.085975809 |
| SRFBP1 | 0.070416082 | 4.486027999 | 1.72045451 | 0.086035143 |
| IL4R | 0.172625367 | 5.965565033 | 1.720053985 | 0.086107989 |
| GP1BA | 0.372435952 | 1.943423561 | 1.719972939 | 0.086122736 |
| CYP4F10P | 0.222579786 | -1.419716208 | 1.71989282 | 0.086137316 |
| FRY-AS1 | 0.197318331 | 0.570480876 | 1.719606533 | 0.08618943 |
| TRA2A | 0.089158566 | 7.597246849 | 1.719454387 | 0.086217136 |
| KB-1507C5.2 | -0.132015196 | 1.460418811 | -1.719161684 | 0.086270459 |
| CTD-2006K23.2 | -0.245347088 | 0.714906212 | -1.719036027 | 0.086293358 |
| PLEKHA8 | -0.262999689 | 1.506777918 | -1.718664713 | 0.086361054 |
| ZNF574 | 0.101073852 | 4.82914821 | 1.718573015 | 0.086377779 |
| RPL19 | 0.071229334 | 10.67448094 | 1.717826316 | 0.086514065 |
| C4A | 0.1665816 | -0.186403281 | 1.71778536 | 0.086521546 |
| RP11-261C10.7 | 0.223545882 | 1.166913502 | 1.717513008 | 0.086571302 |
| SCUBE1 | -0.424579578 | 1.354769085 | -1.717492028 | 0.086575136 |
| HSD17B8 | -0.116471707 | 4.42794123 | -1.717099622 | 0.086646868 |
| TMEM136 | 0.380394896 | -1.550110434 | 1.716573476 | 0.086743123 |
| CLN3 | 0.135456971 | 2.717200903 | 1.716509875 | 0.086754765 |
| TNR | -0.394015904 | -3.592475479 | -1.716241215 | 0.086803953 |
| C21orf58 | 0.149375752 | 3.761260476 | 1.716156179 | 0.086819527 |
| TEAD1 | 0.311542745 | -4.702897294 | 1.716123721 | 0.086825472 |
| RP11-467L13.5 | 0.267695646 | 4.000503844 | 1.716105273 | 0.086828851 |
| MIIP | -0.086759497 | 5.816374465 | -1.716016134 | 0.08684518 |
| AC068831.10 | -0.173120745 | -1.61878396 | -1.715936485 | 0.086859773 |
| RBMXP2 | 0.066736906 | 4.767688803 | 1.715625471 | 0.086916774 |
| RP11-3P17.3 | -0.090321624 | 10.70285372 | -1.7155918 | 0.086922947 |
| DNAJC14 | 0.060587102 | 5.338946566 | 1.715385248 | 0.086960822 |
| RPSAP15 | 0.108403468 | 7.90342197 | 1.715170458 | 0.087000221 |
| WDR36 | -0.081162191 | 4.679266539 | -1.715043728 | 0.087023474 |
| ABCA2 | -0.150387647 | 5.874711297 | -1.714674704 | 0.087091214 |
| PLCD4 | 0.221737339 | -1.444970461 | 1.714304199 | 0.087159268 |
| RP11-148O21.6 | -0.330872086 | -0.191251507 | -1.714115155 | 0.087194008 |
| XPO7 | -0.079176413 | 6.2539094 | -1.713911588 | 0.08723143 |
| UBE2T | 0.220959074 | 4.147603516 | 1.713831061 | 0.087246236 |
| ZNF600 | -0.118115417 | 3.839066459 | -1.713766517 | 0.087258106 |
| DPP10 | 0.562091855 | -2.872608892 | 1.713220163 | 0.08735863 |
| WBSCR16 | 0.084863184 | 3.712358796 | 1.713212781 | 0.087359989 |
| IPO9-AS1 | -0.151829904 | 0.642673922 | -1.713049284 | 0.08739009 |
| CDC34 | 0.072752328 | 6.116361621 | 1.713004883 | 0.087398266 |
| SPAG17 | -0.383373155 | -2.911010235 | -1.712891352 | 0.087419174 |
| PVRL1 | 0.231697439 | 4.241076959 | 1.712435613 | 0.087503145 |
| RP11-421L21.2 | 0.178084547 | 0.135678383 | 1.712401097 | 0.087509508 |
| FOXD4L1 | -0.230019992 | -1.74383495 | -1.712222677 | 0.087542402 |
| UTY | 0.772348261 | 0.524306972 | 1.71215746 | 0.087554428 |
| PPIE | 0.065957051 | 4.124670131 | 1.711833774 | 0.087614136 |
| NDUFA6-AS1 | 0.100653624 | 1.643348918 | 1.711008307 | 0.087766553 |
| CTD-2035E11.4 | -0.225501694 | 0.307351685 | -1.710832064 | 0.087799123 |
| ZNF629 | 0.117870196 | 4.730672038 | 1.710715137 | 0.087820736 |
| REG4 | -0.288024208 | -2.792040503 | -1.710688844 | 0.087825597 |
| NUPR1 | 0.254291702 | -1.262108864 | 1.710469808 | 0.087866099 |
| CMBL | -0.275931119 | 3.746561094 | -1.71045179 | 0.087869431 |
| ZNF230 | 0.124292733 | 2.502220391 | 1.7103392 | 0.087890256 |
| NPTX1 | -0.384706329 | -2.307575883 | -1.709571879 | 0.088032291 |
| TAF7 | -0.099305542 | 7.951175557 | -1.709561959 | 0.088034129 |
| ARPC3P2 | -0.168515959 | 1.254090059 | -1.709541461 | 0.088037926 |
| YWHAQP6 | 0.17282584 | 1.116762072 | 1.709500199 | 0.088045569 |
| CCDC6 | 0.098760473 | 6.064427861 | 1.709494823 | 0.088046565 |
| ABHD2 | -0.105444493 | 6.993657174 | -1.709436183 | 0.088057428 |
| CD81 | 0.141801155 | 5.57996336 | 1.709326589 | 0.088077735 |
| SSC5D | -0.285063914 | -0.477093352 | -1.709237147 | 0.08809431 |
| SERPINB9 | 0.257983056 | 4.880051051 | 1.709211786 | 0.08809901 |
| KCP | 0.232901016 | -1.991220593 | 1.709176587 | 0.088105534 |
| NR3C2 | -0.240992183 | 0.417451472 | -1.709093799 | 0.08812088 |
| CLTCL1 | -0.374823718 | 2.289574609 | -1.708597178 | 0.088212981 |
| NCF1B | 0.332999406 | 5.551392004 | 1.708416664 | 0.088246477 |
| C11orf63 | -0.246810512 | 1.950201801 | -1.708198604 | 0.088286955 |
| IGLV3-10 | 0.350405578 | 1.612772189 | 1.707406748 | 0.088434069 |
| CCT2 | -0.095334298 | 7.307756191 | -1.70708893 | 0.08849317 |
| RP11-568K15.1 | -0.368548271 | 2.732514887 | -1.706940913 | 0.088520706 |
| RPS19 | 0.099935493 | 9.551463532 | 1.706549126 | 0.088593625 |
| RP11-517P14.2 | 0.121206379 | 2.823814916 | 1.706413604 | 0.08861886 |
| GOLGA8O | -0.18342712 | 3.919351611 | -1.706385778 | 0.088624042 |
| CELSR2 | 0.217645131 | 0.48267329 | 1.706183951 | 0.088661635 |
| CISH | -0.184847061 | 4.04511712 | -1.705580912 | 0.088774036 |
| COPZ2 | -0.263920328 | 0.49384314 | -1.705477232 | 0.088793373 |
| RPL38 | 0.085775306 | 9.034345452 | 1.705300379 | 0.088826364 |
| LINC01021 | -0.349663894 | -2.542073084 | -1.705293662 | 0.088827618 |
| RPL7AP34 | 0.153546016 | 1.245800587 | 1.705231307 | 0.088839252 |
| PPIEL | -0.247468189 | 1.885866677 | -1.70337897 | 0.089185438 |
| LRTOMT | -0.136257655 | -0.088681817 | -1.703363877 | 0.089188263 |
| AC022532.1 | 0.155002884 | 0.595410862 | 1.702823075 | 0.089289543 |
| NSD1 | -0.061718417 | 6.431508396 | -1.702719823 | 0.08930889 |
| IGLV4-69 | 0.33100848 | 1.600033258 | 1.702663556 | 0.089319435 |
| PTPN2 | -0.067672077 | 5.441850773 | -1.701967246 | 0.089450009 |
| RP11-475J5.6 | 0.26692404 | 1.820574931 | 1.701752383 | 0.089490332 |
| NIM1K | -0.171764151 | -0.832418771 | -1.701733154 | 0.089493941 |
| ULK4P3 | 0.234102753 | 1.064271011 | 1.701612108 | 0.089516665 |
| CTD-2643I7.1 | 0.303529417 | -1.547133744 | 1.700866667 | 0.089656708 |
| NAGLU | -0.06250917 | 5.022118619 | -1.700773801 | 0.089674166 |
| TCEB1P2 | 0.075901869 | 5.205557289 | 1.700713333 | 0.089685536 |
| VPS39 | 0.046948535 | 6.159640101 | 1.700693549 | 0.089689256 |
| AAGAB | 0.053692262 | 5.847592801 | 1.700207645 | 0.089780662 |
| IFNGR2 | 0.14963107 | 7.200358764 | 1.700000005 | 0.089819746 |
| MIR3654 | -0.180048393 | -1.923393789 | -1.699674351 | 0.08988107 |
| TEF | -0.148708208 | 1.767951485 | -1.699410223 | 0.089930834 |
| HNRNPC | 0.056613367 | 8.574790011 | 1.699040916 | 0.090000451 |
| RP1-167A14.2 | 0.16551973 | 1.547125792 | 1.698940416 | 0.090019403 |
| FAM21A | -0.072922214 | 4.602306378 | -1.698847958 | 0.090036842 |
| MT-TS1 | 0.176378848 | 3.579506389 | 1.698737766 | 0.090057629 |
| GYG1P1 | 0.169769459 | 1.501193745 | 1.698596357 | 0.090084311 |
| LTK | -0.35901956 | 3.96453132 | -1.698497148 | 0.090103034 |
| IGHG2 | 0.448268974 | 5.473607149 | 1.697809171 | 0.090232958 |
| RP11-102N12.3 | -0.261784629 | -0.303334807 | -1.697729367 | 0.090248039 |
| CNBP | -0.0533716 | 9.86474452 | -1.697548473 | 0.09028223 |
| SDHDP2 | -0.206966724 | -0.173589219 | -1.697515319 | 0.090288498 |
| SNCAIP | -0.501960221 | -2.857684474 | -1.697276479 | 0.090333661 |
| PEBP1P2 | 0.098558804 | 4.729089595 | 1.697225945 | 0.090343219 |
| TCP11 | 0.273713406 | -1.24014625 | 1.696861086 | 0.090412251 |
| RP11-1191J2.5 | -0.196952646 | 3.060480278 | -1.696765722 | 0.090430302 |
| CASK | -0.162397014 | 2.313923646 | -1.696680506 | 0.090446433 |
| NFATC2IP | -0.057315513 | 6.192917402 | -1.696381985 | 0.090502964 |
| CTC-428G20.3 | -0.104014066 | 4.107248577 | -1.696123993 | 0.090551842 |
| GCNT2 | 0.214795697 | 1.393316709 | 1.696098862 | 0.090556605 |
| MRPL49 | 0.066864443 | 5.815500996 | 1.69608964 | 0.090558352 |
| SCIN | 0.321647389 | -3.22687838 | 1.696000821 | 0.090575186 |
| HS2ST1 | -0.082723277 | 4.120963373 | -1.695893505 | 0.090595528 |
| LRFN2 | -0.201958706 | -3.154920097 | -1.695455325 | 0.090678625 |
| ZNF521 | 0.581383259 | 3.089987321 | 1.695308385 | 0.090706505 |
| ACTR3C | -0.159805025 | 0.167920123 | -1.69529128 | 0.090709751 |
| ZNF142 | 0.055604987 | 4.987424703 | 1.695246901 | 0.090718173 |
| ALOX5 | -0.287579766 | 6.728914281 | -1.695148725 | 0.090736806 |
| OSCAR | -0.304216831 | 6.068763803 | -1.695124839 | 0.09074134 |
| RP11-327F22.6 | -0.277792861 | 1.326175894 | -1.694711789 | 0.090819772 |
| ACTBP7 | -0.192965894 | 1.005754118 | -1.694563568 | 0.09084793 |
| AP1M2 | 0.220281092 | -2.265326976 | 1.694537937 | 0.0908528 |
| F2R | 0.240350392 | 4.363009091 | 1.694288009 | 0.090900298 |
| RP11-2F9.3 | 0.140287761 | 1.328976588 | 1.694089561 | 0.090938026 |
| PRF1 | -0.261611985 | 4.782070119 | -1.694022542 | 0.090950771 |
| ZNF582-AS1 | -0.171283896 | 1.025344084 | -1.693765649 | 0.090999635 |
| AC016734.2 | -0.089990451 | 5.933857643 | -1.693609428 | 0.091029361 |
| HSPB1P1 | -0.219081841 | 1.768710753 | -1.693466046 | 0.09105665 |
| DDX3Y | 0.850268754 | 3.138922502 | 1.693442082 | 0.091061212 |
| RP11-288H12.4 | 0.285224811 | 2.01164682 | 1.693374212 | 0.091074132 |
| TICRR | 0.270205032 | 1.996042653 | 1.693359897 | 0.091076857 |
| RP11-324J13.1 | 0.180133364 | -0.140842205 | 1.693071233 | 0.091131828 |
| PRODH | -0.468932552 | 0.369029971 | -1.692754219 | 0.091192228 |
| NABP2 | 0.063722162 | 5.522989493 | 1.692632315 | 0.091215463 |
| RP11-359G22.2 | -0.252284655 | -2.563398478 | -1.692498582 | 0.091240957 |
| RP11-449P15.2 | -0.196892316 | 1.479617881 | -1.692267464 | 0.091285031 |
| RP11-640M9.1 | 0.129060915 | 1.594657981 | 1.692078337 | 0.09132111 |
| RP11-996F15.2 | 0.227920496 | 2.611723199 | 1.691826051 | 0.091369255 |
| CDKN2B-AS1 | -0.248438703 | -0.561034744 | -1.69176404 | 0.091381093 |
| RAB2A | 0.076977469 | 6.221782275 | 1.691421068 | 0.091446584 |
| SPTBN5 | 0.218507323 | -0.449866809 | 1.691109807 | 0.091506053 |
| PBDC1 | 0.067467627 | 5.861206409 | 1.69097574 | 0.091531677 |
| IL20RB | 0.156011612 | 0.96584817 | 1.690672183 | 0.091589717 |
| CS | 0.052582597 | 7.445785446 | 1.690374947 | 0.091646577 |
| GAL3ST4 | 0.221721939 | 1.829860763 | 1.69002415 | 0.09171372 |
| RPS3AP5 | -0.098144495 | 7.121647057 | -1.689674871 | 0.091780611 |
| EDA | 0.269311348 | -1.314755515 | 1.689639451 | 0.091787397 |
| LDOC1 | 0.34332281 | 1.262584328 | 1.689506443 | 0.091812882 |
| RP11-1007O24.3 | 0.113574786 | 2.408141668 | 1.689260126 | 0.091860092 |
| UBE2SP2 | 0.110705574 | 4.157606115 | 1.689134092 | 0.091884255 |
| RP11-301O19.1 | -0.129416225 | 1.480098585 | -1.688940531 | 0.091921376 |
| TRIM28 | -0.09285696 | 8.332397524 | -1.6888583 | 0.091937149 |
| TMEM67 | -0.156353925 | 1.502430468 | -1.688487586 | 0.092008287 |
| MPRIPP1 | 0.110162845 | 4.625693112 | 1.688379248 | 0.092029084 |
| CDV3 | 0.05395137 | 8.998267069 | 1.688065651 | 0.092089307 |
| SUMO1P4 | 0.12038335 | 5.337909174 | 1.68806313 | 0.092089791 |
| KCNQ5 | 0.261502036 | 3.496561642 | 1.687845867 | 0.092131533 |
| CD22 | -0.24544398 | 2.32351517 | -1.686492276 | 0.092391935 |
| RAB27A | -0.178028439 | 6.787944493 | -1.686490501 | 0.092392277 |
| PRKAR2B | 0.256460597 | 5.21142223 | 1.6864734 | 0.092395571 |
| PRSS1 | 0.320589874 | -2.063433667 | 1.685437814 | 0.092595202 |
| GON4L | -0.050108672 | 5.168491978 | -1.68539069 | 0.092604294 |
| ABLIM3 | 0.331786993 | -1.152081916 | 1.68527369 | 0.092626872 |
| CLEC7A | 0.363028681 | 5.040559306 | 1.68462924 | 0.092751313 |
| RP11-252E2.1 | -0.211688989 | -1.349721898 | -1.684446028 | 0.092786715 |
| GAPDHP69 | 0.184094149 | -0.920836658 | 1.684406184 | 0.092794416 |
| C9orf24 | 0.189927235 | -1.374285736 | 1.684356203 | 0.092804076 |
| NRBF2 | 0.116739475 | 6.163351327 | 1.683635007 | 0.09294356 |
| AC011242.6 | -0.263156524 | -0.912925866 | -1.683577374 | 0.092954714 |
| RP11-380L11.4 | -0.200752996 | -0.413267493 | -1.682803445 | 0.093104599 |
| RP11-30K9.1 | 0.149721912 | 1.59519869 | 1.682467779 | 0.093169667 |
| SBF1 | -0.065547524 | 6.259596939 | -1.682442293 | 0.093174609 |
| ALS2CL | -0.246148751 | 2.289978757 | -1.682323635 | 0.09319762 |
| IGKV1-6 | 0.321068976 | 1.683939444 | 1.681936431 | 0.093272743 |
| RP11-638I2.4 | -0.153781273 | 1.116642185 | -1.681654344 | 0.093327502 |
| RP11-290D2.6 | 0.224073945 | 5.251814768 | 1.681464321 | 0.093364404 |
| SKAP1 | 0.268474394 | 3.330174275 | 1.681032508 | 0.093448305 |
| OR2W3 | 0.265768673 | 3.724167712 | 1.680917949 | 0.093470573 |
| CTD-3065B20.3 | -0.198641754 | -0.412161335 | -1.680653603 | 0.093521975 |
| HNRNPA1L3 | 0.087593748 | 10.34653511 | 1.680604008 | 0.093531622 |
| RP11-299J3.8 | -0.11846262 | 2.792733843 | -1.680172255 | 0.093615632 |
| LINC00984 | 0.119493328 | 6.382378419 | 1.679821396 | 0.093683946 |
| RP11-347C12.2 | 0.104607415 | 2.510058626 | 1.679655144 | 0.09371633 |
| NRM | 0.092052255 | 6.131826375 | 1.679509411 | 0.093744725 |
| SERAC1 | -0.109935174 | 2.103760798 | -1.679425338 | 0.093761109 |
| RP5-1042I8.7 | -0.176054541 | 2.789495474 | -1.678985376 | 0.093846886 |
| CTB-92J24.2 | 0.199122016 | 0.472387732 | 1.678785499 | 0.093885876 |
| UBE2R2 | -0.082367199 | 7.162322078 | -1.678747383 | 0.093893313 |
| RORC | -0.230554106 | -1.3460836 | -1.678723696 | 0.093897934 |
| RPF2P1 | -0.12093259 | 1.816606892 | -1.678615493 | 0.093919049 |
| CPED1 | -0.403495205 | 0.141257899 | -1.678588908 | 0.093924237 |
| CMPK1 | 0.079513392 | 7.876619526 | 1.678517785 | 0.093938119 |
| RP11-797A18.3 | -0.243657723 | 3.446960117 | -1.67845669 | 0.093950044 |
| MTND4P12 | 0.198064213 | 5.482359664 | 1.678398635 | 0.093961378 |
| RARS2 | -0.041026314 | 5.896253061 | -1.678287278 | 0.09398312 |
| RP5-1125A11.1 | -0.107664241 | 1.766691139 | -1.678242383 | 0.093991887 |
| HCFC1R1 | -0.069636788 | 5.512807384 | -1.678162768 | 0.094007435 |
| RP11-266K22.2 | 0.208356301 | -0.949682889 | 1.677814908 | 0.094075393 |
| RP11-349N19.2 | 0.080764839 | 4.291925495 | 1.677807674 | 0.094076807 |
| RPL24P2 | 0.128220239 | 3.133068866 | 1.677802711 | 0.094077776 |
| PILRA | -0.239081874 | 6.339049191 | -1.677700313 | 0.094097789 |
| STXBP5-AS1 | 0.181590313 | 0.976527011 | 1.67755667 | 0.094125868 |
| PTPLA | -0.230171137 | 4.652057823 | -1.677496887 | 0.094137556 |
| CTC-273B12.6 | 0.174044884 | 0.897669284 | 1.677260111 | 0.09418386 |
| LINC00189 | -0.344681004 | -1.079392575 | -1.677052724 | 0.094224432 |
| PMEPA1 | 0.286057979 | -0.087948164 | 1.676863439 | 0.094261475 |
| RPL35A | 0.0886551 | 9.694836116 | 1.676724685 | 0.094288636 |
| RP11-330A16.1 | -0.296735512 | 0.249678202 | -1.676541858 | 0.094324435 |
| C19orf60 | -0.067234375 | 5.345180322 | -1.67646019 | 0.094340429 |
| TNFRSF17 | -0.279462215 | -0.240257365 | -1.676358447 | 0.094360358 |
| AC008592.3 | -0.109254477 | 1.547236885 | -1.675763424 | 0.094476978 |
| FAM193A | 0.046802156 | 6.035950084 | 1.674647951 | 0.094695913 |
| OAT | 0.167792246 | 6.23302039 | 1.674468603 | 0.094731152 |
| SNX29 | -0.077909852 | 4.770331681 | -1.674409857 | 0.094742697 |
| EEF1A1P14 | 0.14177333 | 2.783654525 | 1.674407002 | 0.094743258 |
| SPTLC1P1 | -0.093395161 | 4.731854355 | -1.673690942 | 0.094884071 |
| CTC-250I14.1 | 0.127415201 | 1.692355602 | 1.673553202 | 0.094911177 |
| RP3-370M22.8 | 0.200385743 | 0.558661723 | 1.673488208 | 0.09492397 |
| DCUN1D2 | 0.083136784 | 4.656777518 | 1.673409577 | 0.094939448 |
| ZNF518B | 0.136315328 | 3.404016782 | 1.673210759 | 0.094978594 |
| SLC18B1 | 0.238550569 | 2.488787582 | 1.673112083 | 0.094998027 |
| RP11-320P7.1 | 0.143194084 | 1.572728285 | 1.673077808 | 0.095004778 |
| SDS | 0.333623506 | -0.76000726 | 1.672670393 | 0.095085053 |
| GIGYF2 | 0.055989383 | 5.027233925 | 1.672650092 | 0.095089055 |
| HLTF | -0.131990609 | 4.600490237 | -1.672343043 | 0.095149593 |
| NUMB | -0.096259835 | 6.04698849 | -1.672019695 | 0.095213377 |
| LINC00355 | 0.353822722 | -2.185973189 | 1.671821511 | 0.095252489 |
| AMPD1 | 0.208435216 | -2.473748578 | 1.671800901 | 0.095256557 |
| NOTCH3 | 0.368617628 | 0.099227075 | 1.671366261 | 0.095342381 |
| ANXA8L1 | -0.369850961 | -1.598346807 | -1.670723452 | 0.095469423 |
| RPL21P75 | 0.068200442 | 6.330138413 | 1.670557369 | 0.09550227 |
| CETN2 | -0.082297279 | 5.757323096 | -1.670462422 | 0.095521051 |
| RPL41 | -0.077389274 | 7.450243408 | -1.670259995 | 0.095561104 |
| CTC-429P9.5 | -0.106887326 | 4.106410383 | -1.670089264 | 0.095594896 |
| C4orf36 | -0.135588219 | 1.782737805 | -1.669856492 | 0.095640982 |
| FYCO1 | 0.066569921 | 4.584754312 | 1.669583053 | 0.095695143 |
| TSPO | -0.157434043 | 8.266732871 | -1.669131485 | 0.095784641 |
| EXTL3 | -0.117399095 | 3.948154421 | -1.668992817 | 0.095812137 |
| SLC30A7 | 0.05403816 | 4.652655246 | 1.668908429 | 0.095828873 |
| RP11-420O16.2 | 0.166877308 | -0.775390681 | 1.668654595 | 0.09587923 |
| SRM | -0.113384165 | 7.172668722 | -1.668255903 | 0.095958366 |
| RP11-266A24.1 | -0.270511754 | 0.184318769 | -1.668145032 | 0.095980382 |
| C20orf194 | -0.087167284 | 4.537221266 | -1.667061998 | 0.096195658 |
| AC002454.1 | -0.301651462 | 4.55702515 | -1.66650189 | 0.096307143 |
| ITGB2 | -0.178513082 | 9.154173413 | -1.666343851 | 0.096338619 |
| RP11-152F13.7 | 0.240108453 | -0.852765446 | 1.665677687 | 0.096471383 |
| LRRFIP1P1 | 0.114831708 | 3.709419939 | 1.665417119 | 0.096523354 |
| GALNT11 | -0.107360372 | 4.120995875 | -1.664796717 | 0.096647184 |
| HIVEP3 | 0.165527678 | 3.847845744 | 1.664758493 | 0.096654817 |
| CUL3 | 0.053432504 | 5.530565646 | 1.664413052 | 0.096723826 |
| PCOLCE-AS1 | -0.206598372 | -1.959483678 | -1.663935032 | 0.096819385 |
| TM9SF3 | -0.058866547 | 6.984299904 | -1.663782351 | 0.096849922 |
| PDE3B | 0.17326723 | 4.881767837 | 1.66309882 | 0.09698673 |
| RP11-349K21.1 | -0.174237118 | -0.082497728 | -1.662442467 | 0.097118244 |
| TSKS | -0.379141844 | -3.14E-05 | -1.662344753 | 0.097137835 |
| ZNF677 | 0.237541186 | 1.227768654 | 1.66228112 | 0.097150595 |
| RPS4XP17 | 0.153225569 | 2.902680933 | 1.661144554 | 0.097378729 |
| ABCA8 | 0.317372172 | -5.436911694 | 1.660972743 | 0.097413253 |
| JAKMIP1 | -0.248997699 | 0.142389709 | -1.660860302 | 0.097435852 |
| CCDC144A | -0.460562797 | -0.422332812 | -1.660420285 | 0.09752433 |
| AC008155.1 | 0.183081867 | 0.204324868 | 1.660372625 | 0.097533918 |
| RPL38P4 | 0.17496567 | 1.967990653 | 1.659471693 | 0.097715291 |
| PNPLA1 | 0.309160799 | 1.561840771 | 1.659148634 | 0.097780394 |
| KIF17 | -0.414217848 | -0.1629925 | -1.659000508 | 0.097810257 |
| HEXA | 0.089023596 | 5.540322695 | 1.658918791 | 0.097826734 |
| ADAT3 | 0.107301027 | 2.402180586 | 1.658838727 | 0.09784288 |
| CCL8 | 0.218541621 | -2.115602045 | 1.658786504 | 0.097853413 |
| AC195454.1 | -0.220488535 | -0.678517743 | -1.658714035 | 0.09786803 |
| PTP4A1P1 | 0.175387148 | -0.13405953 | 1.658438916 | 0.09792354 |
| AMY1B | -0.20145962 | -1.265447051 | -1.658331003 | 0.09794532 |
| CTD-2116N17.1 | 0.181477762 | -1.278787913 | 1.658279073 | 0.097955803 |
| CNOT7P1 | -0.078141161 | 3.156059833 | -1.658000841 | 0.098011981 |
| RP11-293D9.2 | -0.154180682 | 0.592430625 | -1.65770647 | 0.098071446 |
| SNX18P9 | 0.233378362 | -0.988993059 | 1.657516789 | 0.098109778 |
| RP11-478B9.3 | 0.176547239 | -0.046553628 | 1.657012441 | 0.098211759 |
| AC093698.2 | -0.101033435 | 6.31201765 | -1.656297451 | 0.098356478 |
| RP11-333E13.4 | 0.14942219 | 0.572023503 | 1.655932023 | 0.098430509 |
| RPL36AP45 | 0.162764668 | 1.023673097 | 1.655924755 | 0.098431982 |
| PPOX | -0.10359082 | 4.243862117 | -1.654913933 | 0.098636997 |
| HBD | 0.461049897 | 4.817379732 | 1.654666904 | 0.098687152 |
| KB-1507C5.4 | -0.211141709 | 1.782930756 | -1.653881348 | 0.098846779 |
| SPTBN2 | 0.371748846 | -0.370395701 | 1.653593933 | 0.098905235 |
| RP11-384P7.7 | 0.39762062 | -0.935756596 | 1.653310797 | 0.098962847 |
| TSPYL4 | 0.098263639 | 5.579470221 | 1.653308721 | 0.098963269 |
| TCP11L2 | 0.140758856 | 3.875307499 | 1.653261791 | 0.098972821 |
| RP11-244F12.3 | 0.197472965 | -2.823373804 | 1.653247318 | 0.098975767 |
| PCYT1B | 0.345538486 | -0.381237416 | 1.653135054 | 0.09899862 |
| ARHGAP32 | 0.299921735 | 2.644336043 | 1.653018232 | 0.099022406 |
| IGHV3-65 | -0.186589018 | -0.900044359 | -1.652964504 | 0.099033347 |
| TMED8 | -0.084006961 | 6.055449622 | -1.652519234 | 0.099124055 |
| HNRNPA1P7 | 0.095188907 | 9.782596514 | 1.652366588 | 0.099155168 |
| RP1-308E4.1 | -0.134464152 | 1.072602905 | -1.652089207 | 0.099211722 |
| FUNDC1 | -0.072926675 | 4.86459432 | -1.651597721 | 0.099311995 |
| CTB-55B8.1 | 0.191779265 | -0.228605623 | 1.651217973 | 0.099389526 |
| RP11-551L14.1 | 0.221585438 | -1.913842739 | 1.650275735 | 0.099582107 |
| AMH | 0.2366907 | 0.866174741 | 1.65004518 | 0.099629275 |
| RP11-326C3.11 | 0.23932819 | 2.021518525 | 1.650036681 | 0.099631014 |
| AC016708.2 | 0.107750205 | 4.881796406 | 1.649871916 | 0.099664733 |
| RP11-152P23.2 | 0.168343888 | 0.497532977 | 1.649739557 | 0.099691828 |
| RP5-1136G13.2 | 0.1761396 | 1.731082034 | 1.649421127 | 0.099757035 |
| MBD2 | 0.094998591 | 6.915231664 | 1.649256008 | 0.099790862 |
| SNHG6 | 0.111061549 | 8.13783261 | 1.649129611 | 0.099816762 |
| GH1 | -0.226630383 | -0.794378607 | -1.648984787 | 0.099846444 |
| KAT2B | 0.110076685 | 5.396844183 | 1.648511332 | 0.099943531 |
| RP11-783K16.14 | 0.139948337 | -0.161106795 | 1.648502428 | 0.099945357 |
| COL2A1 | 0.377473965 | -2.355099326 | 1.647924411 | 0.100063989 |
| HBB | 0.521910825 | 8.118827902 | 1.64787345 | 0.100074454 |
| RP11-3L10.3 | 0.154484627 | 1.788131175 | 1.647675451 | 0.10011512 |
| VSIG10L | 0.197069065 | 1.073929526 | 1.647596444 | 0.100131351 |
| MT-RNR1 | 0.069219594 | 12.11552448 | 1.647473946 | 0.10015652 |
| ZNF527 | -0.101400928 | 1.936403296 | -1.646341808 | 0.100389378 |
| SNRPE | 0.083381326 | 5.087485487 | 1.64629535 | 0.100398943 |
| RPS10P2 | 0.177910926 | -0.16667656 | 1.646224276 | 0.100413577 |
| PPP2R4 | -0.063777066 | 5.720941593 | -1.646199615 | 0.100418655 |
| ALDH16A1 | -0.071450172 | 4.956761745 | -1.646185347 | 0.100421593 |
| RP11-325L7.1 | -0.169255458 | 0.169262745 | -1.646134277 | 0.10043211 |
| RP11-680A11.5 | -0.206488641 | -0.235830795 | -1.644638756 | 0.100740477 |
| KIAA1109 | 0.059843149 | 6.307097093 | 1.644456223 | 0.100778166 |
| RP11-34P13.7 | -0.219244604 | -1.813174489 | -1.644417084 | 0.100786248 |
| VMA21 | 0.062664193 | 5.814533505 | 1.644212016 | 0.100828606 |
| RPL7AP6 | -0.072213821 | 10.66872632 | -1.644179083 | 0.10083541 |
| IGHE | 0.272144798 | -1.025438021 | 1.644129768 | 0.100845599 |
| MMP14 | 0.388753865 | 2.92955226 | 1.643959996 | 0.100880682 |
| HLA-DQB1 | 0.432198596 | 4.764916955 | 1.643910288 | 0.100890956 |
| SPON1 | 0.4560785 | 0.408809244 | 1.643812283 | 0.100911215 |
| RP11-782C8.1 | -0.300694239 | -0.346652381 | -1.643729056 | 0.100928421 |
| VPS11 | 0.036905868 | 5.921754225 | 1.643589529 | 0.100957272 |
| LY9 | 0.253581002 | 1.842292047 | 1.643449597 | 0.100986214 |
| C12orf57 | 0.118780658 | 6.312178986 | 1.643414805 | 0.10099341 |
| PRR7-AS1 | -0.210815113 | -1.182919843 | -1.643056724 | 0.101067505 |
| SOBP | 0.284810774 | -2.053979071 | 1.643018702 | 0.101075375 |
| ADRBK1 | 0.080820535 | 8.830612344 | 1.64261323 | 0.101159334 |
| VAMP3 | 0.074712886 | 7.414295185 | 1.642098416 | 0.101266013 |
| CMC1 | -0.094646417 | 2.599595202 | -1.641197226 | 0.101452974 |
| HCG4P7 | -0.231269817 | 1.887433359 | -1.641149713 | 0.101462838 |
| RPS26P8 | 0.198655832 | 1.574126286 | 1.640989686 | 0.101496069 |
| IGF2BP2 | -0.197185843 | 6.437420227 | -1.64022489 | 0.101655003 |
| QPCT | 0.381754225 | 4.410896827 | 1.640120844 | 0.101676641 |
| RYKP1 | 0.169254272 | -1.062356502 | 1.640100824 | 0.101680804 |
| RP11-327F22.2 | -0.155646949 | 3.076045613 | -1.640050418 | 0.101691289 |
| RP11-804H8.6 | 0.165976858 | 2.924655117 | 1.639672573 | 0.101769905 |
| RPS6KA2 | 0.256650457 | 2.58687692 | 1.639564735 | 0.101792352 |
| FBXL15 | 0.052298833 | 4.502004608 | 1.639141964 | 0.101880389 |
| ZAN | -0.349185631 | -4.179090364 | -1.638887784 | 0.101933349 |
| RHOBTB3 | 0.257174547 | 3.347981185 | 1.638852564 | 0.101940689 |
| NPM1P9 | -0.149959725 | 0.811748297 | -1.638659301 | 0.101980973 |
| ARFGAP1 | -0.052173722 | 5.598207866 | -1.638641865 | 0.101984608 |
| SEPT5 | 0.241867427 | 4.224644824 | 1.638632284 | 0.101986605 |
| RP11-603J24.14 | 0.147465238 | 0.237518453 | 1.638389699 | 0.10203719 |
| FLNA | -0.089770733 | 9.992670424 | -1.638217424 | 0.102073126 |
| HNRNPCP3 | 0.093533452 | 2.968973779 | 1.638145424 | 0.102088148 |
| RP11-439L18.2 | 0.213956575 | 1.94373881 | 1.637972401 | 0.102124255 |
| RP11-796G6.1 | -0.094420766 | 5.026922337 | -1.63785502 | 0.102148756 |
| USP43 | 0.188264219 | 1.735721601 | 1.637454098 | 0.102232475 |
| FAM189B | -0.081245757 | 4.390401883 | -1.637330477 | 0.1022583 |
| GABRA4 | -0.399206846 | -4.856874603 | -1.637296557 | 0.102265387 |
| ARF4 | -0.06018315 | 7.380611964 | -1.637056458 | 0.102315563 |
| BHLHA15 | -0.229672382 | -0.291361797 | -1.636931534 | 0.102341678 |
| RBPMS2 | 0.395069453 | 1.01521823 | 1.636829954 | 0.102362916 |
| CCDC74B | 0.266685608 | -3.06004234 | 1.636822653 | 0.102364443 |
| RIN2 | -0.281586332 | 2.720697836 | -1.635578066 | 0.102624951 |
| SLIT2 | -0.243979502 | -4.405451625 | -1.634555974 | 0.102839284 |
| HCG15 | -0.215853627 | -1.03436642 | -1.634202483 | 0.102913494 |
| RP11-203M5.8 | 0.220784813 | -0.655709594 | 1.633345992 | 0.103093479 |
| ZNF358 | -0.179277354 | 4.609262757 | -1.632925449 | 0.103181944 |
| AC138623.1 | -0.145078593 | 1.136321886 | -1.63170908 | 0.103438161 |
| ALDH7A1P1 | -0.260270592 | 1.41506638 | -1.631707532 | 0.103438488 |
| STOX2 | 0.42005743 | -0.755734263 | 1.631486195 | 0.103485165 |
| RP6-65G23.1 | -0.214825831 | -0.837195688 | -1.63107399 | 0.103572139 |
| FAM85B | -0.215996581 | -1.068032657 | -1.630968208 | 0.103594468 |
| RP11-196O16.1 | -0.202926353 | 0.910837559 | -1.630950158 | 0.103598278 |
| RP11-147L13.2 | -0.181525123 | 0.569395059 | -1.630909952 | 0.103606766 |
| MRPL47 | 0.052626706 | 6.309312326 | 1.630875548 | 0.10361403 |
| ATP5LP3 | 0.116242776 | 1.60666661 | 1.630606787 | 0.103670787 |
| NOMO3 | -0.102010433 | 3.818351945 | -1.630589168 | 0.103674509 |
| KIAA1211 | 0.352532005 | 2.583307462 | 1.630154132 | 0.103766435 |
| GUSBP3 | -0.185260106 | 0.385066651 | -1.630021386 | 0.103794499 |
| OSGEP | -0.063238365 | 5.792055898 | -1.629049022 | 0.104000246 |
| GNAI3 | 0.058800559 | 7.237416349 | 1.628951203 | 0.104020962 |
| TNPO1P2 | 0.142696003 | -0.532258016 | 1.628626763 | 0.104089695 |
| ANKRD40 | -0.05086738 | 5.322900177 | -1.628601068 | 0.10409514 |
| OLAH | -0.363834849 | -1.588872689 | -1.628473514 | 0.104122174 |
| RAB39A | -0.316574547 | 0.849867694 | -1.628340673 | 0.104150334 |
| RP11-603J24.7 | -0.21554142 | 6.2462952 | -1.62818265 | 0.10418384 |
| FASTKD2 | -0.055427134 | 4.415415887 | -1.628051441 | 0.104211667 |
| SPNS2 | -0.281122086 | 5.486728069 | -1.627366594 | 0.104357008 |
| HHIPL1 | -0.356273531 | -3.664411518 | -1.627135866 | 0.10440601 |
| WWP1 | 0.084102173 | 4.940803128 | 1.627056819 | 0.104422802 |
| PRSS8 | -0.20017697 | -2.775896838 | -1.626245197 | 0.104595343 |
| GULP1 | 0.286716585 | -4.905281045 | 1.625613121 | 0.104729871 |
| IGKV3-15 | 0.334303412 | 2.593017772 | 1.625608175 | 0.104730925 |
| TWSG1 | 0.114837966 | 5.122667949 | 1.625262682 | 0.104804517 |
| AP002884.2 | 0.171479668 | -0.867294013 | 1.625180845 | 0.104821955 |
| LINC00669 | 0.301191047 | -0.308910068 | 1.624750338 | 0.104913725 |
| PES1 | -0.068258631 | 5.867864591 | -1.624367747 | 0.104995336 |
| C1orf21 | 0.258470905 | 2.602614816 | 1.624298955 | 0.105010015 |
| UBIAD1 | -0.060737163 | 4.701200104 | -1.624067421 | 0.105059433 |
| MROH7-TTC4 | -0.234141278 | -3.829037688 | -1.624040461 | 0.105065189 |
| SMC2 | 0.134889113 | 5.105031204 | 1.622501172 | 0.105394218 |
| ACKR4 | 0.186103412 | -1.487599667 | 1.621770563 | 0.105550675 |
| TRIM9 | 0.25095679 | -0.582934771 | 1.62102547 | 0.105710425 |
| RP11-10C24.1 | -0.144034786 | 1.687626213 | -1.620176536 | 0.105892673 |
| PIK3R3 | -0.246010305 | 0.225693596 | -1.62014945 | 0.105898492 |
| RP11-483F11.7 | -0.205385299 | 0.278663995 | -1.619888718 | 0.105954518 |
| IER3IP1 | -0.069221076 | 5.934372485 | -1.619826696 | 0.105967849 |
| IL12RB2 | 0.378238224 | 1.219624025 | 1.61963092 | 0.106009937 |
| RP11-529H2.2 | -0.175327469 | 0.684102789 | -1.619321976 | 0.106076381 |
| LPO | -0.262222647 | -2.927707124 | -1.619040239 | 0.106137003 |
| CTD-3184A7.4 | -0.116247763 | 5.563682069 | -1.618919963 | 0.106162891 |
| RPL21P7 | 0.1625821 | 0.344275249 | 1.618745329 | 0.106200488 |
| RP11-13K12.1 | -0.38553888 | -2.225273823 | -1.618581386 | 0.106235793 |
| PAXBP1 | 0.068111943 | 6.011793236 | 1.618425044 | 0.106269471 |
| ATP5G2 | -0.073991905 | 7.932494731 | -1.618348937 | 0.106285867 |
| RP11-701P16.2 | -0.295743535 | -0.014820898 | -1.617257847 | 0.106521161 |
| SPRYD7 | 0.071494328 | 3.539486761 | 1.61724764 | 0.106523364 |
| RP11-304F15.7 | -0.135703169 | 2.022840283 | -1.617040424 | 0.106568097 |
| RPS3AP49 | 0.144903651 | 1.119486865 | 1.616935016 | 0.106590858 |
| CLCN5 | -0.16792867 | 2.248948568 | -1.616884059 | 0.106601863 |
| MTRNR2L9 | 0.164991684 | 0.865171934 | 1.616812349 | 0.106617351 |
| GGT1 | 0.225065014 | 3.921902016 | 1.616807961 | 0.106618299 |
| FAM13A | 0.14754293 | 2.78945364 | 1.616277876 | 0.106732844 |
| C8orf56 | -0.298868994 | -0.38707583 | -1.616047866 | 0.106782577 |
| HAAO | -0.156759408 | 3.854900879 | -1.615863518 | 0.10682245 |
| CTD-2240E14.4 | -0.220843841 | -0.816932377 | -1.615834302 | 0.10682877 |
| RPS3 | 0.082166058 | 10.39956538 | 1.615336985 | 0.1069364 |
| CTC-484M2.1 | 0.137502988 | 1.036138058 | 1.615211569 | 0.106963556 |
| AP3B2 | 0.299092401 | -2.359556026 | 1.615211389 | 0.106963595 |
| PDIA3P1 | 0.071532835 | 9.559670741 | 1.615122051 | 0.106982943 |
| CDC37 | 0.050054897 | 8.454044765 | 1.61511862 | 0.106983686 |
| AC005944.2 | 0.22639825 | 0.089813051 | 1.614994691 | 0.10701053 |
| LINC00422 | -0.211429885 | 3.865718435 | -1.614773313 | 0.107058495 |
| EFHB | -0.229901901 | -0.801937049 | -1.614421192 | 0.107134823 |
| CACNA1E | 0.362485636 | -3.544159918 | 1.614337142 | 0.107153048 |
| CTD-2154I11.2 | -0.261422551 | 0.564850223 | -1.614268855 | 0.107167858 |
| STAG3L3 | 0.128902238 | 4.256078413 | 1.61406751 | 0.107211532 |
| RP11-166P13.3 | 0.121901787 | 2.233222609 | 1.613931258 | 0.107241096 |
| BAX | -0.06582842 | 6.674075951 | -1.6136775 | 0.107296172 |
| NRXN2 | -0.34052909 | 2.901266378 | -1.613364413 | 0.107364156 |
| ZNF66 | -0.127103733 | 2.778178263 | -1.613220016 | 0.107395522 |
| SNX21 | -0.213799994 | 2.058879118 | -1.613068699 | 0.107428399 |
| EMCN-IT1 | 0.272445006 | -0.888109127 | 1.613055834 | 0.107431195 |
| RP1-59D14.5 | 0.177108971 | 0.219792037 | 1.612996485 | 0.107444093 |
| AC098824.6 | -0.131158841 | 1.904703704 | -1.612551533 | 0.107540826 |
| COMMD5 | 0.068564145 | 4.484498886 | 1.612517052 | 0.107548325 |
| RP4-796I17.5 | 0.115950739 | 1.881588906 | 1.612486958 | 0.107554871 |
| RP11-804M7.1 | -0.164213887 | -0.729346475 | -1.612457819 | 0.107561208 |
| SNORD12 | -0.177753521 | 1.479537748 | -1.612435112 | 0.107566148 |
| AGER | 0.102167999 | 5.583810616 | 1.611985593 | 0.107663963 |
| CTD-2611O12.6 | 0.148569797 | 1.624931366 | 1.61165884 | 0.107735108 |
| RP11-114H7.1 | 0.128801282 | 1.86109422 | 1.611477798 | 0.107774544 |
| PHF21A | -0.060186466 | 5.84784577 | -1.611451518 | 0.107780269 |
| ZNF34 | 0.076343169 | 4.126123427 | 1.611397114 | 0.107792122 |
| RP11-397E7.2 | -0.134384227 | 1.571027292 | -1.611207631 | 0.107833414 |
| IGKV1D-16 | 0.267431256 | 0.668953416 | 1.611176076 | 0.107840291 |
| ART4 | 0.339824883 | -2.817307291 | 1.610955596 | 0.107888356 |
| PCP4L1 | 0.277068817 | -1.343220199 | 1.61085462 | 0.107910374 |
| ERI1 | 0.091758491 | 4.310002416 | 1.610808243 | 0.107920488 |
| LA16c-60H5.7 | 0.1287211 | 1.026032379 | 1.609656323 | 0.108171943 |
| FLJ20306 | -0.168838284 | -1.451482585 | -1.609458278 | 0.108215221 |
| CDKN1C | -0.221110587 | 4.557691481 | -1.608991619 | 0.108317254 |
| PSMC4 | 0.044094134 | 6.574954785 | 1.608913142 | 0.108334419 |
| TTC28-AS1 | 0.071856405 | 2.704578372 | 1.608793603 | 0.108360572 |
| ME1 | -0.343437554 | 1.408434813 | -1.608758206 | 0.108368317 |
| FLVCR2 | -0.253067649 | 2.594409109 | -1.608499081 | 0.108425027 |
| PTMS | 0.216648544 | 6.484271069 | 1.608459178 | 0.108433762 |
| ZNF841 | 0.152599477 | 3.88769739 | 1.608143465 | 0.108502893 |
| INPP4B | 0.262962666 | 2.618690327 | 1.608051537 | 0.108523029 |
| TIGD6 | 0.115773176 | 2.257593754 | 1.607508346 | 0.10864207 |
| MRPS17 | 0.095434315 | 2.568041259 | 1.607091908 | 0.108733404 |
| NDUFA9P1 | 0.156744907 | -0.181752549 | 1.606780498 | 0.108801742 |
| CDA | 0.351631125 | 6.097633533 | 1.606434074 | 0.108877804 |
| ABO | -0.421035249 | 0.413088198 | -1.605922723 | 0.108990155 |
| hsa-mir-4537 | 0.260008505 | 0.564107956 | 1.605801986 | 0.109016696 |
| AF131215.4 | -0.212386254 | -1.92474996 | -1.605652456 | 0.109049574 |
| RP11-720N19.1 | -0.177390349 | -0.005996242 | -1.605636174 | 0.109053154 |
| CSPG4P11 | 0.223311432 | -2.092634353 | 1.604608352 | 0.109279363 |
| CUX1 | -0.070059093 | 6.492612903 | -1.603942587 | 0.109426087 |
| RPL21 | 0.105349288 | 5.928818882 | 1.603588771 | 0.109504126 |
| CCDC25 | 0.062976482 | 4.924485276 | 1.603548593 | 0.109512991 |
| KIR2DL3 | 0.251821296 | -1.461664702 | 1.60351228 | 0.109521003 |
| CDC42EP2 | -0.150730341 | 2.703358541 | -1.603488762 | 0.109526193 |
| FAM20C | 0.309861749 | 3.324367566 | 1.60332643 | 0.109562018 |
| RP11-178L8.7 | -0.16111615 | 0.211291678 | -1.603229608 | 0.10958339 |
| RP11-486O13.2 | 0.232868985 | -1.416394123 | 1.603084827 | 0.109615354 |
| DYNC1H1 | -0.064255541 | 7.243030357 | -1.60272177 | 0.109695542 |
| CRB1 | -0.199888342 | -3.684465503 | -1.602698021 | 0.109700789 |
| RP11-686O6.2 | -0.17275658 | 1.617942395 | -1.602598662 | 0.109722743 |
| C8orf59 | 0.056936354 | 5.986138508 | 1.60259046 | 0.109724556 |
| ACVR1C | 0.205144502 | -1.769388747 | 1.602527796 | 0.109738404 |
| RPL7P50 | 0.181600894 | -0.220272842 | 1.601840189 | 0.109890449 |
| RP11-356J5.12 | -0.156989069 | 0.769565723 | -1.601836666 | 0.109891228 |
| HCFC1 | 0.066665925 | 6.968180669 | 1.601312549 | 0.110007235 |
| RARRES3 | -0.143987954 | 5.494556777 | -1.601147256 | 0.11004384 |
| B3GNT3 | -0.271954718 | -1.920416322 | -1.601041858 | 0.110067187 |
| RFC2 | 0.080744689 | 5.759243505 | 1.600869267 | 0.110105425 |
| ITPKB | -0.088389541 | 5.854616445 | -1.600784371 | 0.110124238 |
| EGFL8 | 0.189674568 | 1.028762692 | 1.600773029 | 0.110126752 |
| HACE1 | -0.089034314 | 3.425706012 | -1.600645511 | 0.110155015 |
| OR8R1P | -0.247054732 | -0.112397023 | -1.600435295 | 0.110201621 |
| MIR4639 | 0.175817471 | 1.654327247 | 1.600434908 | 0.110201706 |
| EIF4E3 | -0.215970023 | 5.243527009 | -1.600370861 | 0.110215909 |
| TTC40 | 0.308832116 | -4.67596228 | 1.599912621 | 0.110317566 |
| AMY2B | -0.133023216 | 3.024452178 | -1.599878729 | 0.110325088 |
| CLGN | 0.3519932 | 0.586523381 | 1.599183326 | 0.110479509 |
| NDUFS5 | -0.081478552 | 8.343834664 | -1.59907562 | 0.110503442 |
| HCG9 | -0.196946603 | -0.956128941 | -1.598627045 | 0.11060316 |
| CACNA2D4 | -0.255313548 | 3.913921203 | -1.598597646 | 0.110609698 |
| RP1-90G24.6 | -0.274765049 | 0.208023577 | -1.598461612 | 0.110639954 |
| KRT8P45 | -0.211885684 | -1.313841967 | -1.598270952 | 0.11068237 |
| AP005482.1 | 0.222850058 | -0.952831013 | 1.597932495 | 0.110757699 |
| RP13-20L14.6 | -0.126668936 | 2.244268578 | -1.597909869 | 0.110762737 |
| MAST3 | -0.14742177 | 5.162526455 | -1.597787063 | 0.11079008 |
| NFAM1 | -0.303911487 | 5.563432777 | -1.597444925 | 0.110866287 |
| DKFZP761J1410 | -0.153704194 | 5.292125964 | -1.596890637 | 0.110989836 |
| IL17RA | -0.206190664 | 6.830267992 | -1.596871664 | 0.110994067 |
| AC011899.9 | -0.25560268 | 2.711733487 | -1.596810432 | 0.111007723 |
| PGK1 | 0.074194713 | 8.299114592 | 1.596551585 | 0.111065464 |
| RGL3 | -0.286983703 | -2.449884835 | -1.596323418 | 0.111116381 |
| RP11-313J2.1 | 0.348951759 | -2.464049729 | 1.59627684 | 0.111126777 |
| RP11-864N7.2 | -0.070562153 | 10.64200265 | -1.596156681 | 0.111153601 |
| NCF1C | 0.308699092 | 5.558529141 | 1.596107505 | 0.11116458 |
| DDI2 | -0.091701874 | 4.432717776 | -1.595991839 | 0.111190408 |
| TMEM259 | 0.072259057 | 8.312511283 | 1.595828824 | 0.111226816 |
| SLC7A7 | -0.352789289 | 4.180670046 | -1.595255582 | 0.111354921 |
| USP19 | -0.04499309 | 6.099691213 | -1.595094838 | 0.111390864 |
| RP11-78H18.2 | 0.187204497 | -0.311834507 | 1.595079041 | 0.111394397 |
| ELN | 0.458334233 | -0.619597653 | 1.594973999 | 0.11141789 |
| ZNF385D | 0.402665381 | -1.760950408 | 1.594838246 | 0.111448258 |
| TUBB1 | 0.401784447 | 2.518136001 | 1.594795208 | 0.111457887 |
| DEFA1 | 0.535641304 | 6.126986076 | 1.59459496 | 0.111502698 |
| RPL37 | 0.088322197 | 8.965633455 | 1.594580265 | 0.111505987 |
| CTC-575D19.1 | -0.097033554 | 9.074541568 | -1.594255903 | 0.111578603 |
| GLI2 | -0.470561098 | -3.635747086 | -1.593888506 | 0.111660899 |
| STXBP5L | 0.373966549 | -4.97847583 | 1.593636552 | 0.111717364 |
| RPS24P7 | 0.156068522 | 1.792628581 | 1.59321497 | 0.111811894 |
| PCSK9 | 0.276775003 | -3.835158618 | 1.593070324 | 0.111844342 |
| TSR1 | -0.073459342 | 5.507763195 | -1.592600759 | 0.11194973 |
| MAP4K1 | -0.103860474 | 6.003799558 | -1.591410417 | 0.112217239 |
| FDPSP3 | 0.191202119 | 0.580008673 | 1.59135371 | 0.112229996 |
| AC005943.5 | -0.156106862 | 0.799605042 | -1.591171355 | 0.112271025 |
| RP11-53B2.6 | -0.153600722 | 1.617023997 | -1.590975352 | 0.112315139 |
| MFAP3L | 0.29688279 | 0.309327471 | 1.590461268 | 0.112430907 |
| RFNG | -0.055750136 | 5.443223906 | -1.590368872 | 0.112451724 |
| RPS15AP40 | 0.141246077 | 0.543398088 | 1.589860023 | 0.112566422 |
| SLC25A6 | 0.072234295 | 10.42090683 | 1.589711472 | 0.112599924 |
| ODF3B | -0.238092436 | 5.807046733 | -1.589696021 | 0.11260341 |
| CMTM6 | 0.082410502 | 8.076179919 | 1.589405544 | 0.112668944 |
| CCDC14 | -0.091632326 | 5.637479913 | -1.588832195 | 0.112798385 |
| ST13P4 | -0.078685245 | 6.268314262 | -1.588741178 | 0.112818945 |
| RP11-758P17.2 | -0.160490136 | 0.762537957 | -1.588594534 | 0.112852075 |
| RP11-307I14.2 | 0.163234025 | 0.786177676 | 1.588322323 | 0.112913595 |
| AC156455.1 | -0.296214585 | 0.098029296 | -1.588056703 | 0.112973651 |
| IQCE | -0.10618049 | 3.587418663 | -1.588023396 | 0.112981183 |
| GPN1 | -0.045369676 | 5.072187464 | -1.587942772 | 0.112999418 |
| FAS | 0.130970944 | 5.014623965 | 1.587859543 | 0.113018244 |
| GPSM2 | -0.153330017 | 3.941144965 | -1.58784769 | 0.113020925 |
| OSBPL8 | -0.08370095 | 6.929870036 | -1.587529293 | 0.113092971 |
| TCP11L1 | 0.071409467 | 4.230699867 | 1.586849265 | 0.113246966 |
| GOLGA8S | -0.236960512 | -1.890537827 | -1.586697403 | 0.113281379 |
| GNB3 | 0.264490417 | 0.297024794 | 1.586600112 | 0.11330343 |
| RP11-408A13.4 | -0.147991571 | 2.4028384 | -1.586308842 | 0.113369466 |
| TUBB8P7 | -0.214652795 | -2.422344803 | -1.586277755 | 0.113376516 |
| PDE1A | 0.247691902 | -3.523599103 | 1.58590495 | 0.113461086 |
| HES2 | 0.274828165 | -3.565048367 | 1.585521817 | 0.113548051 |
| RPH3A | 0.443664478 | -1.381010321 | 1.585492603 | 0.113554684 |
| PPIAP6 | -0.104645338 | 5.599936682 | -1.585127512 | 0.113637606 |
| CEP57 | 0.062878394 | 5.870749694 | 1.585125841 | 0.113637986 |
| IL18BP | 0.10446921 | 4.207310008 | 1.585060891 | 0.113652743 |
| TMUB2 | -0.050925594 | 6.45566703 | -1.584857357 | 0.113698997 |
| MECOM | 0.539100602 | -2.81496946 | 1.58475777 | 0.113721634 |
| PVRL3 | 0.228330511 | -2.147975271 | 1.584420513 | 0.113798322 |
| SEMA6D | 0.249425765 | -4.621616108 | 1.584145601 | 0.113860864 |
| RP11-458F8.2 | -0.153219915 | 2.819826069 | -1.583953796 | 0.113904515 |
| KRT18P10 | -0.30325006 | -0.559578248 | -1.583919336 | 0.113912359 |
| RP11-15F12.1 | -0.247421951 | -1.432972034 | -1.583812834 | 0.113936604 |
| HSPD1P6 | -0.158154877 | 0.190906316 | -1.583699235 | 0.113962469 |
| RP11-110H1.4 | -0.155120788 | 0.14260083 | -1.583486153 | 0.114010997 |
| RP3-467K16.4 | 0.161812771 | -1.510943943 | 1.58342339 | 0.114025294 |
| SURF1 | -0.059270148 | 5.876012366 | -1.583137419 | 0.114090455 |
| CEACAM21 | 0.186243734 | 4.485831648 | 1.583010029 | 0.114119491 |
| RPS26P3 | 0.182897874 | 1.998996896 | 1.582235482 | 0.11429616 |
| TSTD1 | -0.107013528 | 5.310137607 | -1.582183159 | 0.114308103 |
| KLC1 | 0.065709754 | 5.031947359 | 1.582164128 | 0.114312447 |
| SYCP2 | 0.260346949 | 2.024463575 | 1.581933061 | 0.114365199 |
| KCNAB3 | 0.125019694 | 2.400333652 | 1.581640144 | 0.1144321 |
| CFL1 | 0.062838406 | 9.427718327 | 1.58149314 | 0.114465687 |
| POLR2F | 0.068522092 | 3.751788367 | 1.581412732 | 0.114484061 |
| RHOU | 0.269601673 | 4.228042007 | 1.581214316 | 0.114529412 |
| CATSPER1 | -0.218377167 | 2.484160303 | -1.581189215 | 0.11453515 |
| RP11-874J12.4 | 0.202538439 | -1.30142668 | 1.580971602 | 0.114584907 |
| AC093391.2 | -0.16290249 | 0.382421319 | -1.580887571 | 0.114604126 |
| PXDN | 0.658767306 | -0.109727112 | 1.580150768 | 0.114772744 |
| TMEM72 | 0.257931164 | -3.075104395 | 1.579939219 | 0.114821194 |
| RPL31P49 | -0.10416927 | 2.14245299 | -1.579745861 | 0.114865491 |
| C21orf67 | -0.169657314 | 0.37646754 | -1.579476415 | 0.114927243 |
| SETP2 | 0.149804753 | 0.616205211 | 1.579285102 | 0.114971103 |
| ADM2 | 0.232246163 | 0.112277761 | 1.579258059 | 0.114977304 |
| LRG1 | 0.257227433 | 4.837770713 | 1.57906085 | 0.115022533 |
| FAM21B | -0.093698903 | 4.461869808 | -1.579048137 | 0.115025449 |
| RP11-730G20.2 | 0.057147278 | 8.031230141 | 1.578964831 | 0.115044559 |
| RP11-43F13.3 | -0.410182307 | -1.820355427 | -1.578905905 | 0.115058078 |
| KLHDC4 | 0.065058686 | 3.332083964 | 1.578216364 | 0.115216368 |
| FAM50A | -0.077040643 | 6.218266161 | -1.578202617 | 0.115219525 |
| AC079305.10 | 0.179927804 | 3.184276692 | 1.57808898 | 0.115245628 |
| RP11-1017G21.4 | 0.20201112 | -1.615652118 | 1.577938879 | 0.115280115 |
| IGLV6-57 | 0.325661928 | 2.256800413 | 1.577879847 | 0.11529368 |
| CSMD1 | -0.429171532 | -5.157904987 | -1.577467244 | 0.115388529 |
| TRAPPC9 | 0.084653109 | 4.944981851 | 1.577146341 | 0.11546234 |
| DUS2 | -0.079595525 | 3.69358916 | -1.577070251 | 0.115479847 |
| LILRB2 | -0.296217971 | 6.149965063 | -1.576946956 | 0.11550822 |
| ATP8B2 | -0.157684692 | 5.294837892 | -1.576915405 | 0.115515481 |
| RND2 | 0.202158406 | -2.315710997 | 1.576907135 | 0.115517385 |
| HLA-DOB | 0.17539248 | 2.442482406 | 1.576768074 | 0.115549394 |
| RP11-36B15.1 | 0.16365708 | 1.500419219 | 1.576740882 | 0.115555654 |
| IGKV3D-15 | 0.280054085 | 1.245203246 | 1.576590102 | 0.11559037 |
| CD70 | 0.33900864 | 1.50607965 | 1.576581066 | 0.115592451 |
| IGKV1D-17 | 0.210721302 | -0.707465887 | 1.576108091 | 0.115701405 |
| RPS15P5 | 0.143167211 | 1.907853398 | 1.575532426 | 0.115834124 |
| RP11-94L15.2 | -0.2121395 | 2.912334357 | -1.575495605 | 0.115842617 |
| TRBJ2-3 | 0.233186683 | 1.713785057 | 1.574538925 | 0.116063458 |
| RP11-312J18.5 | -0.065316794 | 6.830574923 | -1.573992341 | 0.11618978 |
| LOXL1-AS1 | 0.248832959 | 1.422033783 | 1.573894371 | 0.116212433 |
| VN1R1 | -0.183953321 | 0.536875419 | -1.57380084 | 0.116234064 |
| RPSAP14 | 0.13435992 | 3.370710053 | 1.573730147 | 0.116250415 |
| CPA5 | -0.206873473 | -2.973984453 | -1.573103937 | 0.116395333 |
| RNF8 | -0.075443317 | 3.434708913 | -1.573059972 | 0.116405512 |
| ATG4A | -0.059484638 | 4.422832713 | -1.572587127 | 0.11651504 |
| RPS4XP20 | 0.183493905 | 0.118501165 | 1.572447496 | 0.1165474 |
| IFIH1 | 0.103953306 | 5.104889122 | 1.572246163 | 0.11659407 |
| RP1-8B1.4 | 0.191228078 | 0.677498591 | 1.571664529 | 0.116728981 |
| GRAMD1A | -0.07524116 | 6.384816388 | -1.571662991 | 0.116729338 |
| DCLRE1C | -0.061969009 | 4.916906259 | -1.571626687 | 0.116737763 |
| RP11-296P7.4 | 0.148974208 | -0.258557505 | 1.571508135 | 0.116765278 |
| PCED1B | 0.199692737 | 2.810820319 | 1.571498739 | 0.116767459 |
| CAPN2 | -0.171562145 | 6.137308078 | -1.571257986 | 0.116823352 |
| CEL | -0.210915415 | 2.122636738 | -1.571139807 | 0.116850797 |
| RPL28 | 0.078617199 | 9.61536908 | 1.571011105 | 0.116880691 |
| RBBP4P4 | -0.129834967 | 0.967928562 | -1.570765462 | 0.116937763 |
| TRIM38 | -0.084958645 | 6.162949667 | -1.570621503 | 0.116971221 |
| RP11-545N8.3 | -0.216693061 | -1.252406828 | -1.57057354 | 0.11698237 |
| ZNF184 | 0.110073476 | 4.502525739 | 1.5705264 | 0.116993328 |
| DHFRP1 | 0.14307248 | 5.453901143 | 1.569720556 | 0.117180781 |
| RPL9P8 | 0.188906052 | 4.239493814 | 1.569604947 | 0.117207694 |
| ZMYND19 | -0.075983005 | 4.990600327 | -1.569544502 | 0.117221766 |
| FBXL5 | -0.10443948 | 7.593245213 | -1.569436207 | 0.117246983 |
| SNHG14 | 0.285719514 | 2.964536994 | 1.56942079 | 0.117250573 |
| NPR1 | 0.211567528 | -3.746562586 | 1.56921396 | 0.117298746 |
| RP11-254F7.2 | 0.265418329 | 3.289328352 | 1.568967197 | 0.11735624 |
| RN7SKP173 | -0.12738857 | 2.373347675 | -1.568805691 | 0.117393882 |
| AURKA | 0.218163291 | 3.296514382 | 1.568526693 | 0.11745893 |
| SEPP1 | -0.44836113 | 3.242437255 | -1.568353506 | 0.117499323 |
| LIN52 | 0.073356559 | 3.73002301 | 1.568294197 | 0.117513158 |
| RP11-326C3.2 | 0.258175532 | 4.020901495 | 1.568099832 | 0.117558507 |
| NDUFB4 | 0.042209455 | 6.930137937 | 1.568024567 | 0.117576071 |
| AC007383.3 | 0.135245547 | 5.547873053 | 1.567759031 | 0.117638056 |
| PRMT1 | -0.089571156 | 6.515643956 | -1.567720532 | 0.117647045 |
| CDR2L | 0.234063209 | -0.698116256 | 1.567672364 | 0.117658292 |
| CHDH | 0.250798172 | -2.4395234 | 1.567284734 | 0.117748836 |
| SNRPEP6 | -0.151597284 | 0.261965218 | -1.566806358 | 0.117860651 |
| NBPF14 | -0.118468421 | 5.560250367 | -1.566455162 | 0.117942793 |
| DEFA1B | 0.537848884 | 6.994583773 | 1.566425168 | 0.11794981 |
| PCNT | -0.073444413 | 5.489299821 | -1.566059657 | 0.118035352 |
| RNF208 | -0.162214486 | 2.911332009 | -1.565632465 | 0.118135391 |
| CSTF1 | -0.081786032 | 5.121692949 | -1.565266676 | 0.118221104 |
| MPDU1 | -0.068405244 | 6.294082385 | -1.565128324 | 0.118253536 |
| CPVL | -0.318444515 | 5.804609593 | -1.564774321 | 0.118336551 |
| PRMT3 | 0.058192953 | 3.883516723 | 1.564705025 | 0.118352807 |
| RP11-297L17.6 | -0.071946399 | 7.788905981 | -1.564593182 | 0.118379047 |
| IGKV1-13 | 0.20792286 | -0.710571579 | 1.564470592 | 0.118407814 |
| EGR4 | 0.257704242 | -1.437409229 | 1.564252642 | 0.118458971 |
| NES | 0.258791447 | -2.86210792 | 1.564213829 | 0.118468083 |
| FPR1 | 0.388160792 | 5.788235969 | 1.56416482 | 0.11847959 |
| CDH26 | -0.180620698 | 1.130135284 | -1.564096194 | 0.118495704 |
| IFT88 | -0.067555552 | 3.184198292 | -1.564081232 | 0.118499217 |
| ANKRD18B | 0.410927146 | -0.341370316 | 1.564034317 | 0.118510234 |
| ARHGAP26 | -0.143928583 | 4.85132011 | -1.563952697 | 0.118529403 |
| SUMO2P17 | -0.087138202 | 2.285626568 | -1.563591201 | 0.118614332 |
| RNF166 | 0.095527978 | 6.816191382 | 1.562959211 | 0.118762925 |
| SMC4 | -0.121959795 | 6.758257843 | -1.562151969 | 0.118952936 |
| RP11-423O2.5 | -0.209412967 | 0.576400041 | -1.561684451 | 0.119063091 |
| LA16c-431H6.6 | 0.182958288 | -1.843118223 | 1.561157991 | 0.119187229 |
| RP11-281O15.7 | -0.061164413 | 6.693127723 | -1.560790987 | 0.119273828 |
| PTPN22 | -0.119181348 | 6.130739387 | -1.560634556 | 0.119310755 |
| PAGE2B | 0.185658021 | -0.629667366 | 1.560459165 | 0.119352169 |
| FAM89A | 0.229351021 | 2.297272718 | 1.560312852 | 0.119386725 |
| RASSF8-AS1 | 0.281119228 | -0.645459663 | 1.560167728 | 0.119421008 |
| RP11-33B1.1 | -0.068005859 | 3.658767101 | -1.560156496 | 0.119423661 |
| DPEP1 | -0.358330659 | -0.990755401 | -1.560130193 | 0.119429876 |
| NELFCD | 0.05568954 | 5.934439398 | 1.559876714 | 0.119489778 |
| PRPH | -0.259543531 | -3.123740622 | -1.559675725 | 0.119537292 |
| CSDE1 | 0.04340108 | 9.520300099 | 1.558975376 | 0.119702973 |
| RP11-745L13.2 | -0.245779785 | -1.5438265 | -1.558807868 | 0.119742627 |
| NANOGP5 | -0.193441393 | -0.852278601 | -1.558489762 | 0.11981796 |
| C4BPA | -0.28634059 | -1.816017191 | -1.558210704 | 0.119884077 |
| SMARCE1P2 | 0.150969297 | 0.08552202 | 1.558010005 | 0.119931645 |
| RPL13AP3 | 0.167530005 | -0.36784884 | 1.557971293 | 0.119940822 |
| RP11-313P13.4 | -0.225768977 | 0.333233464 | -1.557820154 | 0.119976657 |
| PCDHGA2 | -0.250577431 | -3.279349824 | -1.557627359 | 0.12002238 |
| NMNAT3 | -0.184426144 | 3.374532695 | -1.557591574 | 0.120030868 |
| RP11-244H3.1 | 0.115147161 | 3.271934701 | 1.556943739 | 0.120184616 |
| RP11-388M20.1 | 0.162559381 | 1.386308477 | 1.556843185 | 0.120208494 |
| MFSD4 | 0.185146917 | -1.178723886 | 1.55655648 | 0.120276596 |
| RNASE2 | -0.308161309 | 9.921990391 | -1.55644093 | 0.120304052 |
| FAM179B | 0.067797704 | 3.721203697 | 1.556269468 | 0.120344802 |
| C14orf37 | -0.297670403 | -1.732560089 | -1.556093687 | 0.12038659 |
| ADSL | -0.06373257 | 5.528515907 | -1.556044761 | 0.120398223 |
| AC040977.1 | -0.145983509 | 1.522801794 | -1.555854278 | 0.120443522 |
| WASF1 | -0.245568398 | 4.794029521 | -1.555449249 | 0.120539888 |
| CBS | 0.267821311 | -1.77478849 | 1.55473706 | 0.12070948 |
| ZNF672 | -0.087060505 | 5.343533826 | -1.554601513 | 0.120741779 |
| CTD-2303H24.2 | -0.342969955 | -0.557023647 | -1.554468386 | 0.120773507 |
| XRCC6P4 | 0.135445797 | -0.058196206 | 1.554197942 | 0.120837984 |
| GNPTG | -0.053787913 | 5.998692723 | -1.554183398 | 0.120841452 |
| CCSER1 | 0.291053513 | 0.199437235 | 1.553855373 | 0.120919694 |
| DENND3 | 0.088947224 | 6.578678125 | 1.553280558 | 0.121056897 |
| HLA-DQB2 | 0.282582815 | 2.3280016 | 1.553128935 | 0.121093109 |
| CTA-293F17.1 | -0.261615885 | 0.377798145 | -1.552869628 | 0.121155058 |
| ACSS3 | 0.270762827 | -3.360559667 | 1.55272968 | 0.121188502 |
| OTOA | 0.285192619 | -2.744499107 | 1.552586345 | 0.121222763 |
| SLC8A3 | 0.392116205 | -0.419313985 | 1.552373557 | 0.121273639 |
| LDLRAD2 | -0.344349595 | 2.739729567 | -1.55231117 | 0.121288559 |
| HAR1A | 0.210314526 | -0.554858147 | 1.552295275 | 0.12129236 |
| GPATCH11 | -0.080348803 | 5.615851101 | -1.552221484 | 0.121310009 |
| SNORD17 | 0.115572253 | 3.097289316 | 1.552202121 | 0.121314641 |
| PSMA8 | -0.259862822 | -1.838348699 | -1.552102825 | 0.121338394 |
| RPS4XP8 | 0.141700249 | 1.985400785 | 1.551863194 | 0.121395732 |
| GAS2L2 | 0.208556425 | -2.424997484 | 1.551621019 | 0.121453701 |
| IGKV1D-27 | 0.206127136 | -0.179604382 | 1.551605903 | 0.12145732 |
| ID1 | 0.248701653 | 4.05456641 | 1.551364256 | 0.121515185 |
| RP11-475C16.1 | -0.05555088 | 11.54999653 | -1.551315691 | 0.121526817 |
| RP5-943J3.1 | -0.220705398 | -0.338021441 | -1.550855289 | 0.121637134 |
| NFU1P1 | -0.171302229 | -0.525379663 | -1.550843966 | 0.121639849 |
| ATP5A1P3 | -0.086847757 | 5.921411856 | -1.550662588 | 0.121683331 |
| ATP6AP2 | 0.080557227 | 8.194015495 | 1.549994427 | 0.121843616 |
| UBE3A | -0.046595386 | 6.414818103 | -1.549971101 | 0.121849215 |
| ACVR2B | 0.211618268 | 0.855302434 | 1.549945857 | 0.121855274 |
| TMEM110-MUSTN1 | 0.137771606 | 0.334851235 | 1.549685215 | 0.12191785 |
| RP11-203J24.9 | 0.17144294 | 0.181720586 | 1.549683423 | 0.12191828 |
| UNC5C | -0.419149225 | -3.760362526 | -1.549545296 | 0.121951452 |
| MAL2 | 0.29431049 | -3.224080433 | 1.549429819 | 0.12197919 |
| TEX9 | 0.209277175 | 0.68696176 | 1.548957476 | 0.122092699 |
| HSP90B1 | -0.086880033 | 9.323147542 | -1.548340137 | 0.122241178 |
| RP11-344N10.5 | -0.157084903 | 1.099992338 | -1.548307544 | 0.122249021 |
| OSGIN1 | 0.167077808 | 0.678822703 | 1.548277263 | 0.122256308 |
| EAF1-AS1 | -0.166594811 | -1.181621473 | -1.548081671 | 0.122303385 |
| ZAP70 | 0.232783378 | 4.556762249 | 1.547721936 | 0.122390006 |
| ENPP3 | -0.291335944 | -0.325002797 | -1.547721917 | 0.122390011 |
| ZNF334 | -0.359326866 | 0.797151833 | -1.547463383 | 0.122452293 |
| PRPF4 | -0.058718414 | 5.827630735 | -1.54743058 | 0.122460197 |
| SIX5 | -0.230237862 | 0.586401885 | -1.54720656 | 0.122514188 |
| RP4-610C12.3 | -0.308017471 | 0.559703886 | -1.547152179 | 0.122527297 |
| ZDHHC4 | 0.071869872 | 5.170242883 | 1.545970151 | 0.122812508 |
| PPP1R36 | -0.17896933 | -1.773830118 | -1.545708061 | 0.122875819 |
| GSTO1 | 0.095702824 | 6.853318066 | 1.54552799 | 0.122919331 |
| RP11-298J20.4 | 0.138314555 | 0.856932033 | 1.545109955 | 0.123020392 |
| NDUFB11 | 0.054950515 | 7.317407322 | 1.545082171 | 0.123027111 |
| ABCC4 | -0.187290656 | 4.02428493 | -1.544666229 | 0.123127735 |
| RP1-102E24.1 | 0.148928071 | 3.085390146 | 1.544235008 | 0.123232123 |
| JUP | 0.234683522 | 6.385003659 | 1.5440409 | 0.123279135 |
| KDSR | -0.084393735 | 4.031909298 | -1.543730505 | 0.123354339 |
| RP11-706O15.5 | -0.334193803 | -0.168516505 | -1.543679969 | 0.123366587 |
| SLC36A4 | 0.140202401 | 4.523793038 | 1.543461726 | 0.12341949 |
| UBA52P6 | 0.167504859 | 0.797025202 | 1.543077029 | 0.123512785 |
| NAT8L | -0.272786003 | -0.471658905 | -1.542599087 | 0.123628771 |
| CR2 | -0.216670309 | -1.042483709 | -1.542533332 | 0.123644735 |
| PCOLCE | 0.133811886 | 1.704078937 | 1.542530715 | 0.12364537 |
| IGLJ2 | 0.187977856 | -0.839915553 | 1.542505565 | 0.123651477 |
| ZNF182 | -0.064087248 | 4.292079457 | -1.542288527 | 0.123704182 |
| STX17 | -0.064907727 | 4.733382346 | -1.542151161 | 0.12373755 |
| PKP2 | 0.400264474 | -0.24144685 | 1.541384651 | 0.123923871 |
| DBF4B | -0.100767648 | 3.29729196 | -1.541188124 | 0.123971677 |
| ZNF208 | -0.268081526 | -2.480372528 | -1.541120259 | 0.123988189 |
| TBC1D27 | -0.258509985 | -1.926252339 | -1.540773373 | 0.124072616 |
| RP11-206L10.10 | -0.157301825 | -0.053354089 | -1.540689666 | 0.124092996 |
| EPHB6 | -0.12770058 | 5.14201282 | -1.539998255 | 0.12426143 |
| AC004980.7 | -0.085472822 | 2.699648773 | -1.539893264 | 0.124287022 |
| SCHIP1 | -0.350242272 | 0.41978823 | -1.539635136 | 0.12434996 |
| CTC1 | -0.078167817 | 5.438295231 | -1.539610249 | 0.12435603 |
| ATP9A | 0.299088457 | 1.959696463 | 1.539509451 | 0.124380615 |
| NKIRAS2 | -0.059699808 | 5.977896575 | -1.539043215 | 0.12449438 |
| CNN2 | 0.120499129 | 8.183605953 | 1.538961262 | 0.124514386 |
| KCNQ3 | 0.285245843 | -4.280241309 | 1.538846544 | 0.124542395 |
| LRRN2 | -0.380076974 | -1.261781211 | -1.538824212 | 0.124547847 |
| TMSB4XP6 | -0.112521827 | 9.572824172 | -1.538572923 | 0.124609219 |
| SPINT2 | 0.205799601 | 5.251177497 | 1.538495963 | 0.124628019 |
| IGKV1-12 | 0.281811478 | 1.65423171 | 1.538398367 | 0.124651863 |
| DHX57 | -0.068468226 | 3.857740257 | -1.538308825 | 0.124673744 |
| GPR68 | 0.211605151 | 0.400449618 | 1.537885461 | 0.124777235 |
| PDLIM1P4 | -0.189423854 | 1.771637608 | -1.537545942 | 0.124860279 |
| RAB15 | 0.209103668 | 2.229536 | 1.537190097 | 0.124947363 |
| PP13439 | 0.188536553 | 2.220166459 | 1.537161958 | 0.124954251 |
| ARHGEF25 | 0.269810335 | -1.183927818 | 1.537117505 | 0.124965134 |
| TTLL7-IT1 | 0.221729033 | -2.493152028 | 1.536977283 | 0.124999467 |
| PARP11 | -0.069784505 | 3.916933289 | -1.536455556 | 0.125127273 |
| CENPB | -0.062080244 | 6.891670468 | -1.536274386 | 0.125171678 |
| CAMP | 0.439487932 | 4.888060833 | 1.53623232 | 0.12518199 |
| GPATCH2 | 0.07243018 | 3.191915138 | 1.536221859 | 0.125184555 |
| IQCB2P | -0.149262608 | -0.467961317 | -1.536213438 | 0.125186619 |
| RP11-33B1.3 | -0.15407436 | 0.092147249 | -1.535406559 | 0.125384554 |
| RP1-292B18.1 | -0.085636397 | 7.755237558 | -1.534814702 | 0.125529897 |
| MYBL1 | -0.197474372 | 1.868255247 | -1.534584882 | 0.12558637 |
| MTFR1 | -0.090935003 | 3.269700815 | -1.534545028 | 0.125596165 |
| GNPNAT1 | -0.082776805 | 4.275128754 | -1.534380957 | 0.125636497 |
| CAMKMT | -0.083903185 | 2.276164451 | -1.533938806 | 0.125745235 |
| AC051649.12 | 0.210370878 | -0.331585927 | 1.533876081 | 0.125760666 |
| TMSB10P2 | -0.22053543 | 4.459665801 | -1.53322386 | 0.125921216 |
| AC098614.2 | -0.148212056 | 6.775335952 | -1.53312629 | 0.125945248 |
| APPL1 | -0.067998367 | 6.361859658 | -1.533016721 | 0.125972239 |
| GMFBP1 | 0.143467157 | -0.035321042 | 1.5328639 | 0.126009892 |
| DPF1 | 0.299423106 | -1.383522074 | 1.532690853 | 0.12605254 |
| RP11-561C5.4 | 0.179502244 | -0.646502652 | 1.532540129 | 0.126089695 |
| AC005702.1 | -0.168368872 | 1.388716885 | -1.532319465 | 0.126144106 |
| RP4-791C19.1 | 0.212535143 | -2.176720601 | 1.532178965 | 0.12617876 |
| RP11-453F18__B.1 | -0.200143652 | 2.785772395 | -1.532119247 | 0.126193492 |
| XRCC1 | -0.050871546 | 5.445980137 | -1.531455511 | 0.126357317 |
| LAMP1 | -0.059395921 | 7.911212461 | -1.531348546 | 0.126383734 |
| INSR | -0.14921068 | 4.347986979 | -1.531224023 | 0.126414492 |
| OGFOD2 | -0.101793086 | 1.009890381 | -1.530932772 | 0.126486458 |
| AC009495.2 | 0.213609481 | 2.574356212 | 1.530592753 | 0.126570514 |
| RP11-732M18.3 | 0.150673417 | 2.344149153 | 1.530298402 | 0.126643315 |
| ECI1 | 0.064600232 | 4.391905646 | 1.529673395 | 0.126798006 |
| SLC22A31 | 0.330124284 | 0.063513592 | 1.529633459 | 0.126807895 |
| IGFBP2 | -0.377864712 | 3.449620327 | -1.529370668 | 0.126872984 |
| CLDN4 | 0.226812321 | -2.95587606 | 1.529354059 | 0.126877099 |
| GSN-AS1 | -0.226647064 | -2.683046899 | -1.529144857 | 0.126928935 |
| AIFM2 | 0.160752139 | 1.669480499 | 1.528881363 | 0.126994247 |
| TIMM23B | 0.09044832 | 3.647287303 | 1.52875365 | 0.127025913 |
| RP11-252A24.2 | -0.073357627 | 3.660551535 | -1.528612007 | 0.12706104 |
| TOP1 | 0.081573993 | 7.207550512 | 1.528226893 | 0.127156584 |
| AC118138.2 | 0.131238995 | 2.476771204 | 1.528051737 | 0.127200058 |
| ELP5 | 0.059733354 | 5.79816952 | 1.527743975 | 0.127276472 |
| OR2T8 | 0.233813732 | 1.167510363 | 1.527506296 | 0.127335511 |
| NPM1P12 | -0.108142418 | 4.422241014 | -1.527383689 | 0.127365974 |
| RP11-573G6.8 | 0.265285901 | -0.292967993 | 1.527351719 | 0.127373918 |
| MESTP1 | 0.279203353 | 2.749828316 | 1.526392996 | 0.127612333 |
| RP11-69E11.4 | -0.204025151 | 2.42057132 | -1.525978335 | 0.127715558 |
| MECP2 | 0.058305622 | 5.248477857 | 1.525526695 | 0.127828064 |
| CTC-250P20.2 | 0.118102518 | 0.883833998 | 1.525424617 | 0.127853502 |
| RP11-228B15.4 | -0.147790738 | 3.655135109 | -1.524693916 | 0.128035715 |
| IGHV3OR16-8 | 0.22311666 | -0.177295063 | 1.524605349 | 0.128057814 |
| ESM1 | 0.239620161 | -2.207626429 | 1.524600988 | 0.128058902 |
| COL4A4 | -0.227479607 | -2.689252097 | -1.524464313 | 0.128093012 |
| DGUOK | -0.056937626 | 6.448973378 | -1.524316968 | 0.128129792 |
| IGKV1D-12 | 0.255311822 | 0.566941989 | 1.524029919 | 0.128201469 |
| CTB-102L5.7 | -0.158992455 | 0.292776427 | -1.524026218 | 0.128202393 |
| RP4-756H11.3 | 0.125444202 | 2.539889021 | 1.522617834 | 0.128554525 |
| PFN1P2 | -0.182384375 | -0.17160856 | -1.522319741 | 0.128629153 |
| RP11-454F8.2 | -0.152326319 | -0.151094192 | -1.52228898 | 0.128636856 |
| XXbac-BPGBPG55C20.2 | 0.098379406 | 3.066218619 | 1.52209818 | 0.128684642 |
| EMR3 | -0.318808481 | 2.487819819 | -1.522046955 | 0.128697474 |
| EXOSC4 | -0.092862985 | 5.112122173 | -1.521963837 | 0.128718298 |
| TMEM125 | -0.212558115 | -2.540016644 | -1.521850397 | 0.128746721 |
| GP5 | 0.237823988 | -0.821955566 | 1.52148816 | 0.128837517 |
| MICU3 | 0.290115568 | 0.434612733 | 1.521470439 | 0.128841961 |
| ZDHHC20P4 | 0.116413848 | 3.512042055 | 1.520545473 | 0.129074039 |
| NT5C3B | 0.149812784 | 4.011544542 | 1.520348533 | 0.129123495 |
| RP11-206L10.1 | 0.115062407 | 3.58709919 | 1.519911972 | 0.129233176 |
| DSCR9 | -0.189586874 | -1.038637253 | -1.519816409 | 0.129257195 |
| RBM26-AS1 | -0.124720401 | 0.553234074 | -1.519469745 | 0.129344355 |
| EEF1GP5 | 0.169592834 | 0.203592097 | 1.519094173 | 0.129438835 |
| TIGD1 | -0.117502472 | 4.875966554 | -1.519031657 | 0.129454567 |
| AC093375.1 | -0.237713313 | -2.580432825 | -1.518729173 | 0.129530707 |
| CDC25B | 0.100168395 | 5.587248732 | 1.518602939 | 0.129562492 |
| RP11-293M10.6 | 0.151081883 | 1.871128151 | 1.518556336 | 0.129574228 |
| ZMIZ1-AS1 | 0.210750031 | -0.430677524 | 1.518390166 | 0.129616081 |
| RASIP1 | 0.202115432 | -1.369250863 | 1.518175186 | 0.129670244 |
| CCDC176 | 0.134318671 | 1.10745395 | 1.518133733 | 0.12968069 |
| ARFGEF1 | -0.063602706 | 6.037317875 | -1.517622685 | 0.129809524 |
| RP11-242C19.2 | -0.188024543 | 1.874857564 | -1.517383414 | 0.129869878 |
| ARHGAP6 | 0.357831577 | -0.730636768 | 1.516943559 | 0.129980885 |
| FAM21C | -0.058555808 | 5.723612127 | -1.516853969 | 0.130003504 |
| IL27 | -0.243901867 | -1.179699687 | -1.516613685 | 0.130064184 |
| MT-ND2 | -0.046156021 | 12.84026907 | -1.516568737 | 0.130075537 |
| ULK2 | -0.173828718 | 3.031180649 | -1.516528011 | 0.130085825 |
| RP11-84A1.1 | 0.192282277 | 0.425828378 | 1.516444805 | 0.130106845 |
| CD300LB | -0.316138639 | 4.031039085 | -1.516292876 | 0.130145234 |
| PDE6G | -0.158223449 | 5.160549799 | -1.515813851 | 0.130266328 |
| CACYBP | -0.065892548 | 5.693730841 | -1.515747233 | 0.130283176 |
| GATA5 | 0.235634609 | -2.710063563 | 1.515381425 | 0.130375719 |
| FOXH1 | 0.169496875 | -1.657942268 | 1.515122982 | 0.130441131 |
| RP11-13P5.1 | 0.155527955 | -0.659171141 | 1.514614216 | 0.130569975 |
| FBXO6 | 0.119648796 | 4.754840747 | 1.514445805 | 0.130612646 |
| RPS18P9 | -0.082084986 | 6.532447089 | -1.514396097 | 0.130625243 |
| RP11-214N9.1 | -0.141235298 | 1.133927088 | -1.51437803 | 0.130629822 |
| CLIC3 | -0.209055114 | 2.38623224 | -1.514361431 | 0.130634029 |
| ACN9 | -0.092683402 | 4.332526394 | -1.514021563 | 0.130720188 |
| PRDM15 | -0.062040884 | 3.078385519 | -1.513923757 | 0.13074499 |
| HIST2H2AB | -0.220149139 | 1.268495577 | -1.513833568 | 0.130767865 |
| DCLRE1B | 0.088441005 | 4.335642659 | 1.513712934 | 0.130798465 |
| CREB3L1 | 0.249110515 | -1.778315234 | 1.5136175 | 0.130822678 |
| ATP5G1 | -0.081949007 | 5.386887791 | -1.513490434 | 0.130854921 |
| SESTD1 | 0.22829703 | 3.848762798 | 1.513378417 | 0.13088335 |
| ADAR | -0.061372576 | 8.017311157 | -1.513265728 | 0.130911956 |
| NFKBIZ | 0.176836617 | 7.445830636 | 1.511573043 | 0.131342212 |
| RPS4XP14 | -0.140773183 | 7.562297114 | -1.511240003 | 0.131426995 |
| CAMK1 | -0.326145385 | 3.197021792 | -1.511135672 | 0.131453564 |
| IGKV3-20 | 0.33019485 | 4.191267567 | 1.511123368 | 0.131456697 |
| PLA1A | 0.185083716 | -1.521228094 | 1.510971522 | 0.131495374 |
| VCL | -0.120273886 | 7.26917133 | -1.510702775 | 0.131563849 |
| SNORA14B | 0.147598731 | 0.836980793 | 1.510672261 | 0.131571626 |
| TWIST1 | 0.340746786 | -0.984905 | 1.510525942 | 0.13160892 |
| C5orf56 | -0.156315385 | 3.92216252 | -1.510524471 | 0.131609295 |
| RASL10B | 0.215185641 | -1.331563442 | 1.51031242 | 0.131663358 |
| PPRC1 | 0.083687031 | 5.574208253 | 1.510056955 | 0.131728512 |
| CTC-523E23.4 | -0.248076968 | 0.480753878 | -1.510053393 | 0.131729421 |
| CHMP1B | -0.076424263 | 7.574860794 | -1.509200179 | 0.13194721 |
| OR52K2 | -0.224817338 | -0.962298103 | -1.508918446 | 0.132019186 |
| RP11-230B22.1 | 0.208676445 | 0.318980707 | 1.508748322 | 0.132062663 |
| RPS26P15 | 0.185436616 | 2.559050013 | 1.508739086 | 0.132065024 |
| C19orf53 | 0.039363064 | 7.093306896 | 1.507624432 | 0.132350167 |
| SKIDA1 | -0.405081471 | 0.510607076 | -1.507385485 | 0.132411355 |
| RP11-385F5.4 | -0.134383313 | 1.208771159 | -1.507300957 | 0.132433006 |
| BTF3P7 | -0.070670946 | 5.113100616 | -1.507116679 | 0.132480215 |
| RP5-1033H22.2 | 0.254141884 | -0.062085075 | 1.50699743 | 0.132510773 |
| RP11-611D20.2 | 0.158906787 | 2.26892045 | 1.506991901 | 0.132512189 |
| RP11-783K16.13 | 0.118673143 | 2.508940951 | 1.506881965 | 0.132540365 |
| RP11-387D10.3 | -0.236188253 | -1.284721419 | -1.506856093 | 0.132546997 |
| MT-TQ | -0.169641157 | 2.93521502 | -1.50662734 | 0.132605641 |
| LINC00092 | 0.20213255 | -1.594786048 | 1.506303819 | 0.132688616 |
| UBE2O | -0.063353033 | 4.910351086 | -1.506184557 | 0.132719214 |
| CCDC11 | 0.128866246 | 1.365997501 | 1.506079045 | 0.132746288 |
| FBF1 | -0.102748997 | 1.639003975 | -1.50577007 | 0.132825597 |
| GSTM3 | 0.243295532 | 1.225446174 | 1.505670778 | 0.132851091 |
| CCSER2 | 0.086149865 | 4.737884251 | 1.505363375 | 0.132930044 |
| ATP5J | -0.069171097 | 7.303484423 | -1.505340441 | 0.132935936 |
| NUDT16L1 | -0.063919475 | 5.522593264 | -1.504743777 | 0.133089292 |
| CCNI2 | 0.183688568 | -1.922154641 | 1.50462502 | 0.133119832 |
| RP11-505P4.7 | -0.178741828 | -0.893477375 | -1.504597714 | 0.133126855 |
| RP11-432J24.2 | 0.188781725 | -0.217415145 | 1.504270312 | 0.133211081 |
| FAM9C | 0.206177614 | -3.567257325 | 1.504246409 | 0.133217232 |
| MIR451B | 0.214410865 | -1.74886045 | 1.503540571 | 0.133398961 |
| PKM | 0.111781269 | 8.894926651 | 1.502588803 | 0.133644314 |
| AC010524.2 | -0.226850562 | -3.023142605 | -1.502143803 | 0.133759148 |
| C12orf65 | 0.10417707 | 2.311489123 | 1.501974138 | 0.133802952 |
| SRP68 | 0.046699432 | 5.519316273 | 1.501614639 | 0.133895802 |
| DCP2 | -0.071420237 | 5.295925037 | -1.501320083 | 0.133971916 |
| DDAH2 | 0.128870661 | 7.275113233 | 1.500449051 | 0.134197191 |
| ASNSD1 | 0.048978244 | 6.349418987 | 1.499790748 | 0.134367642 |
| CSTA | -0.260808512 | 7.187991598 | -1.499576717 | 0.134423096 |
| ARHGEF11 | -0.241712484 | 3.897796961 | -1.4992291 | 0.134513199 |
| RP1-144F13.4 | -0.12373658 | 0.746113513 | -1.499150609 | 0.134533551 |
| RNF25 | 0.068683011 | 4.433478474 | 1.49887558 | 0.134604881 |
| SUSD3 | 0.238525898 | 4.686590939 | 1.49845588 | 0.134713788 |
| FAM193B | -0.071509195 | 5.98224924 | -1.498269547 | 0.134762161 |
| BMP8B | -0.265455952 | 0.649050826 | -1.497971433 | 0.134839582 |
| NCAM2 | 0.288800161 | -2.00390152 | 1.497727635 | 0.134902922 |
| RP11-1094M14.1 | 0.135365035 | 3.155290817 | 1.4977187 | 0.134905244 |
| ROBO2 | 0.361479269 | -6.163593738 | 1.497297622 | 0.135014698 |
| RP11-1246C19.1 | -0.159592829 | 1.818296698 | -1.497089422 | 0.135068842 |
| RP11-214C8.2 | 0.188989485 | -1.688502719 | 1.495945859 | 0.135366538 |
| NUTM2A | 0.176154602 | -0.688152196 | 1.495940649 | 0.135367895 |
| ADAT2 | 0.099270579 | 4.027063921 | 1.495879406 | 0.135383853 |
| MACROD2 | -0.298548999 | -1.515184673 | -1.495533543 | 0.135473997 |
| RPS3AP47 | 0.095485422 | 4.129097645 | 1.495435121 | 0.135499659 |
| CTA-373H7.7 | 0.17309413 | -2.194941604 | 1.495378974 | 0.135514299 |
| OBSCN | -0.171712713 | 4.488454254 | -1.494988271 | 0.13561621 |
| PCSK6 | 0.314772524 | -0.657931744 | 1.494938447 | 0.135629211 |
| FEM1A | 0.070081831 | 5.692369766 | 1.494702424 | 0.135690809 |
| FCN2 | 0.205376077 | -0.153288393 | 1.494419726 | 0.135764616 |
| IMPDH1P10 | -0.121107468 | 2.137935657 | -1.494325377 | 0.135789256 |
| MFAP1 | 0.10625847 | 5.252434731 | 1.49418446 | 0.135826064 |
| KIAA1161 | 0.182756614 | -0.360574864 | 1.493697044 | 0.135953438 |
| RAB11FIP1 | 0.129318595 | 6.414844568 | 1.493610353 | 0.135976103 |
| LCN10 | -0.314471467 | -2.512881291 | -1.493532419 | 0.13599648 |
| FAM115B | -0.162131906 | 1.796362678 | -1.49340695 | 0.136029291 |
| NPR3 | 0.413593889 | 3.732458641 | 1.492653677 | 0.136226407 |
| KRBA1 | 0.105707314 | 2.969780591 | 1.492642663 | 0.136229291 |
| TSPAN13 | 0.227881501 | 4.297608765 | 1.491608949 | 0.136500157 |
| POLDIP3 | -0.042366289 | 7.257587276 | -1.491520882 | 0.136523253 |
| ZNF708 | 0.082595077 | 4.668032643 | 1.491292777 | 0.136583087 |
| PITPNB | -0.05752986 | 4.061936904 | -1.491075796 | 0.136640023 |
| AC079781.8 | -0.182547131 | -0.08615747 | -1.490939629 | 0.136675763 |
| DHX34 | 0.096229826 | 4.844796059 | 1.490737196 | 0.136728908 |
| RP11-341G5.2 | 0.160386042 | -0.786489779 | 1.49065387 | 0.136750789 |
| RGAG1 | -0.222583301 | -3.301803429 | -1.490488357 | 0.136794259 |
| KCNJ11 | 0.225645207 | -1.789471094 | 1.490418174 | 0.136812695 |
| COL6A4P2 | 0.24907783 | 0.220320815 | 1.49026864 | 0.136851981 |
| RP11-53I6.1 | 0.1342155 | 0.819373355 | 1.490170945 | 0.136877653 |
| CD8BP | -0.215779092 | -0.556713619 | -1.490038686 | 0.136912414 |
| MORF4L1P1 | -0.05460987 | 9.817977458 | -1.489875231 | 0.136955383 |
| FUBP3 | -0.04850126 | 5.649404739 | -1.489571141 | 0.137035349 |
| MYADM | 0.119314811 | 8.984818225 | 1.489550572 | 0.137040759 |
| RP11-436I9.5 | -0.211989719 | -0.670588679 | -1.488940992 | 0.137201174 |
| AC016712.2 | 0.115465423 | 1.469253207 | 1.488750185 | 0.137251416 |
| RP11-395P17.3 | 0.099014783 | 3.959460284 | 1.488579494 | 0.137296373 |
| RP11-456K23.1 | 0.214331505 | -1.07212993 | 1.48850892 | 0.137314965 |
| GZMB | 0.247628249 | 4.195445027 | 1.488236335 | 0.13738679 |
| CECR2 | -0.301500603 | -3.281070818 | -1.48820496 | 0.137395059 |
| RP11-255H23.2 | -0.252006183 | -0.343233642 | -1.487397502 | 0.137608003 |
| RP11-273G15.2 | 0.242511428 | -0.92999298 | 1.487032038 | 0.137704468 |
| LIPT2 | 0.095311842 | 2.686863867 | 1.486934658 | 0.13773018 |
| DZIP1 | 0.351691391 | -3.196475602 | 1.486547765 | 0.137832372 |
| ARHGEF34P | 0.249335844 | -0.548447101 | 1.486526426 | 0.137838011 |
| C6orf48 | 0.103305167 | 8.795503031 | 1.486471248 | 0.13785259 |
| ITGA8 | 0.249055239 | -3.932650312 | 1.486428276 | 0.137863946 |
| RP11-421E14.2 | 0.161612073 | -0.395287065 | 1.485937316 | 0.137993734 |
| AC097639.8 | 0.142412565 | 0.281833449 | 1.485911039 | 0.138000683 |
| ACP6 | -0.182277351 | 2.276646549 | -1.485638985 | 0.138072645 |
| DUOXA1 | 0.238554696 | -2.785178228 | 1.485566789 | 0.138091747 |
| BAK1 | 0.061266911 | 6.119923321 | 1.485421809 | 0.138130112 |
| ATP6V1C2 | 0.225237371 | -1.592871958 | 1.485369879 | 0.138143856 |
| SH3PXD2B | -0.280453487 | 1.333759582 | -1.485082697 | 0.138219882 |
| CTAGE12P | -0.200667726 | -2.232694234 | -1.484856339 | 0.138279828 |
| ADAMDEC1 | -0.301003941 | 0.68448596 | -1.484491549 | 0.138376478 |
| DLAT | -0.066616075 | 5.161737057 | -1.484164284 | 0.13846323 |
| RPS9P1 | 0.135540925 | 1.12701884 | 1.484109763 | 0.138477687 |
| FAM58DP | -0.107879933 | 1.386195215 | -1.483420655 | 0.13866051 |
| IGLV3-1 | 0.311448862 | 3.244048347 | 1.482612099 | 0.13887526 |
| RP11-660L16.2 | 0.128105821 | 4.498401293 | 1.482608791 | 0.138876139 |
| BDKRB2 | 0.255729931 | -4.392322897 | 1.482443994 | 0.13891994 |
| CLC | -0.395187886 | 5.879894792 | -1.481778771 | 0.139096858 |
| NMT2 | 0.189041024 | 2.256626613 | 1.481737888 | 0.139107737 |
| PDIA6 | -0.09037262 | 8.067448427 | -1.481427208 | 0.139190428 |
| ZEB2 | -0.093023883 | 6.826608434 | -1.481401597 | 0.139197246 |
| NFE4 | -0.316773996 | 0.703622006 | -1.481398558 | 0.139198055 |
| RP11-66N24.4 | 0.119279455 | 1.495810028 | 1.481296979 | 0.139225101 |
| NRXN3 | -0.319265584 | -4.737615524 | -1.481275029 | 0.139230946 |
| RPS3AP30 | 0.16252188 | -0.299409201 | 1.481256512 | 0.139235877 |
| RPA3-AS1 | -0.073303275 | 3.459743996 | -1.481182557 | 0.139255571 |
| RP5-1053E7.3 | 0.117433802 | 2.357729445 | 1.480936168 | 0.139321201 |
| CTB-63M22.1 | -0.11231425 | 8.483329279 | -1.480926816 | 0.139323692 |
| ZNF174 | -0.074218054 | 3.782633125 | -1.480567515 | 0.139419443 |
| IGHV1-24 | 0.273522566 | 1.0616945 | 1.480511194 | 0.139434456 |
| MIR631 | 0.139927392 | 1.25332431 | 1.480385676 | 0.13946792 |
| MEIS3P2 | 0.226906229 | -1.731249571 | 1.480204161 | 0.139516324 |
| SNHG15 | 0.114588037 | 5.751401401 | 1.479335287 | 0.139748205 |
| B3GALT1 | -0.279974679 | -2.858923204 | -1.479175865 | 0.139790782 |
| SOCS1 | 0.205517552 | 3.402386181 | 1.47901506 | 0.13983374 |
| C12orf68 | 0.172172965 | -2.147031328 | 1.478897122 | 0.139865252 |
| MAP3K3 | 0.09459154 | 6.216892971 | 1.47885491 | 0.139876533 |
| AC073283.7 | -0.162816303 | 0.866189192 | -1.478597403 | 0.13994536 |
| GMIP | -0.085591525 | 7.437202649 | -1.478570702 | 0.139952499 |
| GLB1L2 | 0.257361915 | -0.963737878 | 1.478251615 | 0.140037826 |
| AC000068.10 | -0.160456825 | -0.456831414 | -1.478043459 | 0.14009351 |
| DDX19A | -0.043026877 | 4.592757941 | -1.477397215 | 0.140266499 |
| ZNF503-AS1 | 0.250438427 | -2.998696555 | 1.477343214 | 0.140280961 |
| FAM101B | 0.13688076 | 9.02735626 | 1.476950061 | 0.14038629 |
| AHCY | -0.093384592 | 6.987098907 | -1.476933265 | 0.140390791 |
| SLC26A10 | 0.202651359 | -3.614265094 | 1.476921575 | 0.140393924 |
| SRPX | 0.241128047 | -1.551130065 | 1.476476288 | 0.140513299 |
| ADAM23 | 0.322365159 | -1.797774338 | 1.476430373 | 0.140525613 |
| THRB | 0.306090969 | -2.16494002 | 1.476376156 | 0.140540154 |
| TTN-AS1 | -0.179516174 | 3.554230817 | -1.476063696 | 0.140623979 |
| BCL2L2 | 0.077196924 | 5.055272372 | 1.475682866 | 0.140726198 |
| MEF2C-AS1 | -0.201700766 | 0.291614202 | -1.475597094 | 0.140749228 |
| IGKV1-5 | 0.327137586 | 3.860325629 | 1.47555279 | 0.140761125 |
| HRASLS5 | 0.271323658 | -1.09831165 | 1.475493195 | 0.140777129 |
| PROX2 | -0.197722643 | -1.202229619 | -1.475135428 | 0.140873237 |
| TIMP1 | 0.147864793 | 8.21070307 | 1.475017657 | 0.140904885 |
| SLC4A8 | -0.150550265 | 1.289992641 | -1.474671276 | 0.140997999 |
| PRLR | -0.491045998 | -1.021667483 | -1.474494525 | 0.141045531 |
| MINOS1P3 | -0.264936106 | 0.201814511 | -1.474489345 | 0.141046924 |
| GLYCTK-AS1 | -0.241547844 | -1.468664526 | -1.473719368 | 0.141254134 |
| IGLL5 | 0.248611086 | -0.215958804 | 1.47350196 | 0.141312683 |
| RP11-458F8.1 | -0.141571404 | 3.185127534 | -1.473490429 | 0.141315789 |
| PGLYRP1 | 0.421574606 | 5.799394626 | 1.472678557 | 0.1415346 |
| ELMO1 | -0.079116553 | 6.105670939 | -1.472411096 | 0.141606742 |
| ISL2 | -0.253409909 | -1.137212736 | -1.472390032 | 0.141612425 |
| CREB3 | -0.048235395 | 5.608187386 | -1.471884243 | 0.141748932 |
| CCZ1B | 0.080131089 | 3.583003581 | 1.471481709 | 0.141857645 |
| SRP54 | -0.062046424 | 5.880318448 | -1.471270846 | 0.141914618 |
| SLC43A1 | -0.121032331 | 5.089941581 | -1.470981982 | 0.141992695 |
| TRPC5OS | -0.187300402 | -2.501233596 | -1.470880158 | 0.142020225 |
| HAS3 | 0.136958296 | 0.075483787 | 1.470724153 | 0.142062412 |
| CTD-3074O7.12 | -0.088106103 | 4.754865457 | -1.470544327 | 0.142111052 |
| ERVK13-1 | -0.066352569 | 3.696872714 | -1.470511284 | 0.142119991 |
| TMEM52 | 0.179306481 | -0.940463564 | 1.470318841 | 0.142172061 |
| FAM156A | -0.167484574 | -1.035596579 | -1.470289162 | 0.142180093 |
| RP11-392O18.2 | -0.168130446 | 4.061978001 | -1.470121965 | 0.142225346 |
| ZNF268 | -0.088129528 | 3.118623078 | -1.469866852 | 0.142294415 |
| SNORD101 | -0.126206093 | 1.656272949 | -1.469331507 | 0.142439438 |
| HSPBP1 | -0.07276947 | 5.537491429 | -1.469150753 | 0.14248843 |
| CTD-2576F9.1 | 0.100025696 | 6.224055412 | 1.468489597 | 0.142667739 |
| IFT52 | 0.067245575 | 5.217871954 | 1.468416042 | 0.142687699 |
| CTD-2616J11.14 | 0.165398684 | 0.187670704 | 1.468318988 | 0.142714038 |
| AR | -0.445067992 | -0.965083425 | -1.468272986 | 0.142726523 |
| LINC00562 | 0.13242943 | -0.084985194 | 1.46823925 | 0.14273568 |
| AGBL3 | -0.147725626 | 0.326038917 | -1.46810377 | 0.142772458 |
| NPIPA7 | 0.098554605 | 1.125086265 | 1.468041403 | 0.142789391 |
| IGKV2D-30 | 0.236877747 | 0.855169792 | 1.466416504 | 0.143231102 |
| ARPP21 | -0.462288376 | -2.865918466 | -1.466360793 | 0.143246265 |
| NPM1P46 | 0.086689353 | 3.183287926 | 1.465689063 | 0.143429189 |
| RP11-109N23.6 | -0.148917524 | 1.254841687 | -1.46553916 | 0.143470035 |
| RP1-172N19.4 | -0.151104062 | -0.689248188 | -1.465499857 | 0.143480746 |
| RNF112 | 0.193465403 | -1.349926859 | 1.465368526 | 0.14351654 |
| CLEC9A | 0.363041172 | 0.401145653 | 1.464948029 | 0.143631195 |
| PPIAP2 | 0.148985542 | 0.040054345 | 1.464936386 | 0.14363437 |
| INO80B | 0.051635509 | 5.582967341 | 1.464733753 | 0.143689647 |
| THG1L | -0.086625658 | 4.636387717 | -1.46462537 | 0.143719219 |
| RAPGEF3 | -0.183727736 | 1.725700988 | -1.464612926 | 0.143722615 |
| RNF216-IT1 | 0.162786776 | 0.400732293 | 1.464461296 | 0.143763997 |
| ITM2B | -0.076058946 | 10.01814285 | -1.464383948 | 0.143785109 |
| RNF185 | -0.072956131 | 5.131221015 | -1.463953716 | 0.143902587 |
| PHGDH | 0.237184811 | 3.356823716 | 1.463926505 | 0.14391002 |
| SUMO1P1 | -0.275024225 | -0.191038454 | -1.463059003 | 0.144147132 |
| RPL19P21 | 0.136461389 | 1.370093012 | 1.462476626 | 0.14430648 |
| SLC5A10 | 0.349567532 | -0.583374284 | 1.462433887 | 0.14431818 |
| LMAN1 | -0.077083983 | 6.376864123 | -1.462247298 | 0.144369265 |
| ORM2 | 0.329713783 | 0.270414271 | 1.462207845 | 0.144380069 |
| RP11-148O21.2 | -0.283236869 | -0.41406645 | -1.462175909 | 0.144388815 |
| MT-TL1 | 0.113356245 | 6.080317484 | 1.462145228 | 0.144397217 |
| RP11-429J17.2 | 0.137161203 | 0.496519158 | 1.461491139 | 0.144576435 |
| ZNF778 | 0.071285798 | 3.255736244 | 1.461284342 | 0.144633132 |
| KIF13A | 0.142418184 | 4.667381997 | 1.461132901 | 0.144674663 |
| LAMC1 | 0.38888541 | 2.284300739 | 1.460880695 | 0.144743848 |
| USP22 | 0.081718991 | 7.522852739 | 1.460636118 | 0.144810965 |
| MNAT1 | 0.076254034 | 3.418504811 | 1.460291614 | 0.144905544 |
| ANKRD13D | 0.092973975 | 6.984543902 | 1.460284455 | 0.14490751 |
| IGHV1-18 | 0.29947139 | 2.428764882 | 1.459902862 | 0.145012328 |
| LLGL2 | 0.106794245 | 3.044058304 | 1.459710687 | 0.145065138 |
| BBS4 | -0.088331832 | 3.139780642 | -1.459662126 | 0.145078485 |
| FUBP1 | 0.065417373 | 7.542346247 | 1.459519438 | 0.145117708 |
| FAHD2A | 0.062565032 | 3.997152433 | 1.459493213 | 0.145124917 |
| GPR56 | -0.239833903 | 4.458888595 | -1.459044692 | 0.145248268 |
| IGLC3 | 0.346362606 | 5.71683826 | 1.458934454 | 0.145278597 |
| QKI | 0.069905831 | 6.362771656 | 1.458921294 | 0.145282218 |
| SHD | 0.41018435 | -0.023096703 | 1.458704623 | 0.145341846 |
| MTCYBP3 | 0.209263186 | 0.347411742 | 1.458649178 | 0.145357107 |
| SDF2L1 | -0.107991998 | 5.562907683 | -1.458334762 | 0.145443674 |
| CTD-3035D6.1 | -0.09494196 | 7.928283811 | -1.458190359 | 0.145483446 |
| OR2T33 | 0.212410729 | -0.273132408 | 1.457630516 | 0.145637716 |
| OTOF | 0.333924032 | -1.655939129 | 1.457497587 | 0.145674364 |
| MIR4458HG | -0.305196738 | -1.129280266 | -1.457301838 | 0.145728345 |
| RP11-756H6.1 | 0.166546392 | -0.163553208 | 1.456956974 | 0.145823483 |
| RPL7P32 | 0.092357242 | 3.725272711 | 1.456640639 | 0.145910793 |
| GOLGA1 | -0.057335237 | 4.590432281 | -1.456571387 | 0.145929912 |
| RPL13AP20 | 0.078537059 | 4.799500548 | 1.456245093 | 0.146020023 |
| PDZK1 | -0.15537591 | -1.481523765 | -1.455863988 | 0.146125324 |
| AC004381.6 | 0.140467913 | 4.510889212 | 1.455651257 | 0.146184127 |
| RP11-16P6.1 | 0.131148851 | 2.078738166 | 1.455442528 | 0.146241842 |
| XKR9 | -0.192779139 | -2.386384439 | -1.455296671 | 0.146282183 |
| KCTD13 | -0.04983146 | 4.238808363 | -1.454830489 | 0.146411177 |
| TRPT1 | -0.062416465 | 4.395463156 | -1.454415517 | 0.146526073 |
| SERPINB6 | 0.179812672 | 6.588641279 | 1.454250768 | 0.146571708 |
| PDE3A | 0.315451617 | -0.932316493 | 1.453988153 | 0.146644474 |
| AL122127.4 | 0.234104691 | 0.60192053 | 1.453484398 | 0.146784133 |
| NRGN | 0.290725047 | 6.471449152 | 1.453249404 | 0.146849316 |
| ROCK1 | -0.042881215 | 6.664713567 | -1.453079285 | 0.146896518 |
| ZNF439 | -0.147025302 | 3.807134258 | -1.453024199 | 0.146911805 |
| PNPT1 | -0.065304569 | 4.614079312 | -1.452983084 | 0.146923216 |
| RP11-114H24.4 | -0.142058727 | -0.377591033 | -1.452762084 | 0.146984561 |
| IGKV2-30 | 0.272471879 | 1.954062312 | 1.452706872 | 0.14699989 |
| MAST2 | 0.0755543 | 4.285299757 | 1.452689785 | 0.147004635 |
| MIF | 0.117699593 | 8.518383156 | 1.452585853 | 0.147033494 |
| RP11-355O1.7 | 0.135278652 | 1.628905341 | 1.45147792 | 0.147341412 |
| RP11-475I24.3 | -0.199981446 | -0.792282092 | -1.451450507 | 0.147349037 |
| RP11-42I10.1 | -0.165332751 | 0.312804514 | -1.450770923 | 0.147538159 |
| NBEAP1 | 0.198769926 | -1.139761575 | 1.450740155 | 0.147546726 |
| CAST | -0.148009141 | 6.349461918 | -1.450337772 | 0.147658799 |
| RPL31P28 | -0.148937945 | -0.038912717 | -1.450279834 | 0.147674941 |
| TPT1P1 | -0.156899048 | 0.96492863 | -1.450266938 | 0.147678534 |
| AC027319.1 | -0.161025993 | 2.882931878 | -1.449875804 | 0.147787548 |
| CD93 | -0.28671019 | 5.897639566 | -1.449419826 | 0.147914711 |
| VWA9 | -0.05669891 | 5.345971999 | -1.449405562 | 0.147918691 |
| RP11-368I23.2 | -0.232377801 | -1.707414107 | -1.449329689 | 0.147939859 |
| CEBPE | -0.299841504 | 4.25202534 | -1.449285607 | 0.147952158 |
| RPS18 | 0.087654641 | 10.30356573 | 1.448938164 | 0.148049129 |
| RP11-480I12.2 | 0.117433952 | 2.729262141 | 1.448501871 | 0.148170966 |
| FAM135A | -0.106010934 | 3.120740753 | -1.448320908 | 0.148221524 |
| ATP5C1P1 | -0.133040628 | 0.640872483 | -1.448154957 | 0.148267899 |
| CHSY1 | 0.090407776 | 6.871028849 | 1.447881152 | 0.148344438 |
| FCHSD2 | -0.067173795 | 6.630582143 | -1.447532913 | 0.148441828 |
| BCR | 0.114165323 | 6.158400185 | 1.447441349 | 0.148467444 |
| DZIP1L | 0.227152787 | 0.618242514 | 1.447402068 | 0.148478434 |
| BAG6 | -0.040430138 | 7.946570124 | -1.446870817 | 0.148627128 |
| WIPI1 | 0.174245471 | 4.17303427 | 1.446786817 | 0.14865065 |
| RAB3GAP1 | 0.043522933 | 5.280269604 | 1.446770239 | 0.148655293 |
| AP000330.8 | -0.219981066 | -2.787385194 | -1.446499432 | 0.148731145 |
| RP11-524O24.2 | -0.159700986 | -0.336294399 | -1.446404565 | 0.148757724 |
| HSPA4L | 0.34371069 | -0.545191567 | 1.446336773 | 0.14877672 |
| ADAM33 | 0.208978533 | -3.875560939 | 1.446242792 | 0.148803057 |
| RP11-423F24.3 | 0.086408148 | 3.537986319 | 1.446209554 | 0.148812372 |
| ZFAND4 | 0.085281545 | 3.48093355 | 1.445772202 | 0.148934987 |
| RP11-1134I14.2 | -0.404551563 | -0.741584866 | -1.445327669 | 0.149059695 |
| SNORD109A | -0.211117324 | -0.134232411 | -1.444426853 | 0.149312652 |
| PRRC2B | -0.057049058 | 7.06394108 | -1.444248573 | 0.149362754 |
| MBOAT4 | -0.218295254 | -1.601354589 | -1.444104379 | 0.149403286 |
| HSP90AA4P | 0.140782796 | 0.435330339 | 1.443913688 | 0.1494569 |
| SLC2A6 | -0.218763109 | 3.49936169 | -1.443795873 | 0.149490032 |
| PROCR | -0.182693616 | 1.991007831 | -1.44323146 | 0.149648836 |
| TES | 0.136218984 | 6.303168705 | 1.443202483 | 0.149656993 |
| EEF1A1P12 | 0.089051007 | 6.304246338 | 1.442841707 | 0.149758573 |
| RP11-347C12.3 | 0.188266643 | -2.425395489 | 1.442658924 | 0.149810058 |
| AP000640.10 | -0.167208221 | 0.116954971 | -1.442516485 | 0.149850188 |
| RP11-592N21.1 | -0.095508474 | 8.058816401 | -1.442478762 | 0.149860817 |
| SMIM4 | -0.093033889 | 3.060279338 | -1.442401493 | 0.149882592 |
| CD2BP2 | -0.046722975 | 6.113562211 | -1.442272524 | 0.14991894 |
| NUDT16P1 | -0.347277968 | 1.454735034 | -1.442263026 | 0.149921617 |
| RPL5P4 | -0.078477555 | 7.609302939 | -1.442180785 | 0.149944799 |
| PSRC1 | -0.204680927 | 2.725958386 | -1.442126741 | 0.149960035 |
| BDP1 | 0.042739783 | 5.458296845 | 1.441376225 | 0.150171737 |
| AC021016.6 | 0.130501709 | 2.330933506 | 1.441219666 | 0.150215928 |
| NADK | -0.115405746 | 6.377561333 | -1.441131865 | 0.150240715 |
| NLRX1 | -0.07461103 | 4.644916433 | -1.440892292 | 0.150308364 |
| ANP32AP1 | -0.075299948 | 3.379321413 | -1.440809472 | 0.150331756 |
| GPC5 | 0.249386049 | -2.12992018 | 1.44076504 | 0.150344307 |
| RP11-316M1.12 | -0.177492355 | -0.098066639 | -1.440544131 | 0.150406718 |
| AC115617.2 | -0.090006813 | 3.371077696 | -1.440416758 | 0.150442712 |
| ACOT4 | -0.192147655 | 0.94918626 | -1.439920582 | 0.15058299 |
| CYB5R4 | -0.068573973 | 5.272519328 | -1.439811735 | 0.150613777 |
| RP11-829H16.2 | 0.099004782 | 4.881879148 | 1.439644152 | 0.150661186 |
| LA16c-390E6.3 | -0.154457115 | -0.029754225 | -1.439509795 | 0.150699203 |
| MAPK10 | -0.267576748 | -3.564183585 | -1.43950277 | 0.150701191 |
| SMIM3 | 0.276955962 | -1.47531774 | 1.439345612 | 0.15074567 |
| RP11-693N9.2 | -0.178652193 | 0.111685029 | -1.439302401 | 0.150757901 |
| CCDC15 | -0.138023398 | 2.210349968 | -1.439282807 | 0.150763448 |
| AL390877.1 | 0.062837077 | 5.378223235 | 1.439214628 | 0.150782749 |
| CLIP3 | -0.282138675 | 2.205072612 | -1.439182726 | 0.150791781 |
| YWHAZP4 | 0.107665709 | 3.550997746 | 1.438080124 | 0.151104195 |
| SMAD9 | 0.254329539 | -2.874997071 | 1.438008368 | 0.151124544 |
| ADAP2 | -0.254885813 | 3.377431901 | -1.437988532 | 0.151130169 |
| GDA | -0.241752891 | -5.31514177 | -1.437501149 | 0.151268442 |
| RP11-533E19.5 | -0.129510648 | 0.675385458 | -1.437154213 | 0.151366928 |
| DCLRE1CP1 | -0.202264915 | -0.37275288 | -1.436807927 | 0.151465278 |
| WNT10A | 0.182707867 | -1.145480248 | 1.436526277 | 0.151545306 |
| HPX | -0.16793355 | -1.814024687 | -1.436437574 | 0.151570517 |
| GOLGA8H | 0.131465511 | 3.472034314 | 1.436167992 | 0.151647157 |
| TMIGD2 | 0.299999527 | 3.541309947 | 1.436037352 | 0.151684307 |
| PCOLCE2 | 0.397606267 | 0.5009091 | 1.435798999 | 0.151752106 |
| HCAR3 | 0.255361588 | 4.542889458 | 1.43570729 | 0.151778199 |
| RP11-68I3.11 | -0.14960391 | 2.849920555 | -1.434939812 | 0.151996692 |
| TSTA3 | 0.072965929 | 5.533535478 | 1.434885496 | 0.152012164 |
| XXyac-YM21GA2.7 | -0.250017833 | -2.395540997 | -1.434541631 | 0.152110144 |
| CBFB | -0.062438003 | 6.551303451 | -1.434513633 | 0.152118124 |
| NBPF15 | 0.112213251 | 2.668630396 | 1.433987868 | 0.152268034 |
| RPL12P1 | 0.090969979 | 4.221028603 | 1.433469355 | 0.152415987 |
| AC026150.5 | -0.206218837 | -3.327670615 | -1.433413677 | 0.152431881 |
| IGKV1OR2-108 | 0.20599224 | -0.610320068 | 1.433134191 | 0.152511681 |
| GOLM2P1 | 0.094874256 | 1.52003284 | 1.432992291 | 0.152552209 |
| CSF3R | -0.141783783 | 8.604974971 | -1.432540281 | 0.152681364 |
| FSIP1 | -0.129646862 | -0.140792282 | -1.432015981 | 0.152831278 |
| AC007969.5 | -0.067860632 | 9.363376886 | -1.43178918 | 0.152896163 |
| ALKBH5 | 0.053375186 | 7.319607758 | 1.431724665 | 0.152914624 |
| RALGPS1 | -0.118643968 | 1.369873264 | -1.431663924 | 0.152932006 |
| AC027601.1 | 0.247630347 | -1.170218421 | 1.431660337 | 0.152933032 |
| SNX30 | 0.10996045 | 4.831014491 | 1.430558704 | 0.153248552 |
| RP11-634H22.1 | -0.13979225 | 1.31175826 | -1.430397089 | 0.153294882 |
| AVP | 0.255561029 | -0.202239538 | 1.4302984 | 0.153323178 |
| RP1-100J12.1 | -0.140546969 | 0.013601097 | -1.429924253 | 0.15343049 |
| RP11-762I7.5 | -0.149448755 | -1.451410464 | -1.429532298 | 0.153542972 |
| NCLN | -0.045761782 | 6.571382094 | -1.429193555 | 0.153640233 |
| RP11-2C24.6 | 0.142735198 | -0.686416098 | 1.429024663 | 0.153688744 |
| BCKDHA | 0.098986275 | 1.341205392 | 1.429015083 | 0.153691496 |
| JAM2 | -0.20671364 | -2.186925693 | -1.428975748 | 0.153702796 |
| FCHO2 | 0.173719949 | 3.798872059 | 1.42870193 | 0.153781475 |
| NPC2 | 0.103497805 | 7.11917319 | 1.428375535 | 0.153875301 |
| THRAP3 | -0.077666149 | 7.578604373 | -1.428268872 | 0.153905972 |
| LINC00051 | -0.23701939 | -1.741915933 | -1.428071124 | 0.153962847 |
| PRRC1 | 0.065349967 | 5.229001317 | 1.428030651 | 0.15397449 |
| AC004987.9 | -0.094554931 | 6.262233088 | -1.428006941 | 0.153981311 |
| RP11-169K16.6 | 0.10799235 | 2.253636476 | 1.427815934 | 0.154036267 |
| TTC39C | 0.113987889 | 4.481408504 | 1.427699565 | 0.154069756 |
| TBC1D2B | -0.068913197 | 5.632364072 | -1.42767297 | 0.15407741 |
| KIAA1328 | 0.079105302 | 2.191490842 | 1.427428487 | 0.154147789 |
| MALAT1 | -0.104397439 | 10.34425723 | -1.427226536 | 0.154205943 |
| RP11-615I2.3 | 0.150857869 | 0.495465056 | 1.426789927 | 0.154331726 |
| PHYHIP | 0.215851695 | -3.074995618 | 1.426581674 | 0.154391749 |
| RP11-324H6.5 | -0.107650252 | 0.889678081 | -1.426412608 | 0.15444049 |
| MIR503HG | 0.331706421 | 0.926922535 | 1.426171765 | 0.154509946 |
| CD209 | -0.314124421 | -1.249929447 | -1.426110273 | 0.154527683 |
| XPOT | -0.061955779 | 6.263240495 | -1.425321278 | 0.154755403 |
| YWHAZP3 | -0.068108816 | 7.790495101 | -1.425125854 | 0.154811846 |
| DOK2 | -0.163921219 | 6.536253752 | -1.424866639 | 0.154886737 |
| RFTN1 | 0.113009837 | 5.429944718 | 1.424854571 | 0.154890224 |
| SCO2 | -0.139032559 | 6.143808775 | -1.424675732 | 0.154941911 |
| GABARAPL2 | -0.062839138 | 6.166261485 | -1.424270751 | 0.155059003 |
| RP11-681N23.1 | 0.129032642 | 1.031614634 | 1.423947974 | 0.155152376 |
| AMDP1 | 0.136384385 | 0.537596153 | 1.423617163 | 0.155248118 |
| ZNF503 | 0.282617939 | 3.302329159 | 1.423021595 | 0.155420597 |
| RN7SL21P | -0.152461186 | 0.059130254 | -1.422869765 | 0.155464591 |
| AC097523.1 | 0.088615042 | 3.907247343 | 1.422827964 | 0.155476705 |
| MSR1 | 0.407081448 | 0.835345856 | 1.422752992 | 0.155498434 |
| XG | -0.233838649 | -1.552661763 | -1.422735957 | 0.155503371 |
| TMED7 | 0.070902814 | 5.997528951 | 1.42165132 | 0.15581799 |
| MFAP4 | 0.339227583 | 2.712090575 | 1.421601231 | 0.155832531 |
| EIF4HP1 | 0.062420289 | 8.134074977 | 1.421330759 | 0.155911067 |
| TFB2M | 0.091203061 | 5.284484771 | 1.421165747 | 0.155958996 |
| RP11-613C6.4 | 0.116808373 | 1.087197922 | 1.421050358 | 0.155992518 |
| RRBP1 | -0.079084094 | 6.544290801 | -1.420723537 | 0.156087495 |
| RP11-698N11.4 | 0.128162683 | 2.997020615 | 1.4205132 | 0.156148643 |
| RP11-511B7.1 | -0.142073604 | 0.085979294 | -1.420350313 | 0.15619601 |
| RPS20P10 | -0.14207968 | 5.494397206 | -1.420128517 | 0.156260525 |
| NEFH | -0.365238944 | -0.156551297 | -1.419344695 | 0.15648868 |
| RHOA | 0.048084076 | 10.57559007 | 1.419130774 | 0.156550992 |
| ATP5JP1 | -0.149688766 | 0.009979031 | -1.418853229 | 0.156631865 |
| STX1B | 0.186201745 | -0.028251046 | 1.418744369 | 0.156663594 |
| NUDT10 | 0.37176204 | -0.657337782 | 1.418692179 | 0.156678808 |
| TEX30 | 0.121859247 | 4.743331112 | 1.418522748 | 0.156728205 |
| PPP2R5E | -0.026893723 | 5.175066915 | -1.418392661 | 0.156766139 |
| ZNF418 | 0.253263825 | 1.697137278 | 1.418330382 | 0.156784303 |
| NRCAM | 0.259986294 | -2.74116826 | 1.417762837 | 0.1569499 |
| RP11-138C9.1 | -0.184099666 | -0.746078956 | -1.417480336 | 0.157032377 |
| TLR2 | -0.181358413 | 6.470235082 | -1.417443306 | 0.15704319 |
| HAVCR1 | 0.211361303 | -0.440423673 | 1.417439563 | 0.157044283 |
| TNFRSF13B | -0.238428081 | -1.286840659 | -1.417392261 | 0.157058097 |
| PCK2 | -0.096749464 | 4.203202055 | -1.417330787 | 0.157076051 |
| TCF20 | 0.053334279 | 5.711780158 | 1.416776812 | 0.157237916 |
| ZYX | 0.078074503 | 9.652478541 | 1.416744061 | 0.15724749 |
| VPS25 | -0.056625272 | 5.994676078 | -1.416088449 | 0.157439224 |
| UROC1 | -0.204260712 | -2.698929597 | -1.415480074 | 0.157617303 |
| SETMAR | -0.091415822 | 3.986873869 | -1.414265922 | 0.157973158 |
| CLEC17A | -0.234786592 | 0.496266128 | -1.413672599 | 0.158147277 |
| FOXRED2 | -0.159812597 | 3.73259303 | -1.413561481 | 0.158179902 |
| TSTD3 | -0.155904243 | 2.358687625 | -1.413350655 | 0.158241816 |
| XDH | 0.227418068 | -4.301476818 | 1.412976335 | 0.158351791 |
| MAP7D3 | -0.078044894 | 4.368231295 | -1.412426952 | 0.158513303 |
| LPCAT1 | -0.124200375 | 5.694764688 | -1.412227874 | 0.158571861 |
| DYRK4 | -0.08275176 | 2.912941673 | -1.4118588 | 0.158680465 |
| REEP1 | 0.194286001 | -3.385263987 | 1.411212695 | 0.158870725 |
| AP001056.1 | -0.178226312 | 1.120489068 | -1.411146818 | 0.158890133 |
| GBP1P1 | 0.224426993 | 0.893193747 | 1.410816288 | 0.158987541 |
| TMEM51-AS1 | -0.250664721 | -2.037935161 | -1.410814008 | 0.158988213 |
| FMO2 | 0.225485914 | -4.328313759 | 1.410779628 | 0.158998348 |
| PTP4A1P2 | 0.164308605 | -0.416775207 | 1.410725509 | 0.159014302 |
| TNKS2-DT | -0.155973054 | 0.048464439 | -1.410246606 | 0.159155534 |
| OR7D2 | 0.224390007 | -2.264606025 | 1.409625399 | 0.159338874 |
| OTX1 | -0.216721943 | -3.341922767 | -1.409466924 | 0.159385671 |
| RP11-359M6.3 | -0.224077657 | -0.306591557 | -1.409371177 | 0.15941395 |
| PPP1R3D | 0.101778636 | 4.237104987 | 1.409301582 | 0.159434508 |
| SNRPEP2 | -0.101253839 | 3.785743337 | -1.409170846 | 0.159473131 |
| IGHV4-39 | 0.268317303 | 2.78014035 | 1.408762767 | 0.159593734 |
| RP11-229C3.2 | 0.166316098 | -0.90110535 | 1.40852752 | 0.159663289 |
| CD27 | -0.207498721 | 3.344882008 | -1.408499953 | 0.159671442 |
| RPA3 | 0.085170954 | 3.349695171 | 1.408416788 | 0.159696038 |
| ERMARD | -0.064547101 | 4.544030085 | -1.407518771 | 0.159961809 |
| ISYNA1 | 0.150841271 | 5.58716468 | 1.407332283 | 0.160017043 |
| NBPF13P | -0.226947315 | 0.059235601 | -1.406095691 | 0.160383662 |
| RP11-161M6.3 | -0.20311702 | -1.245965521 | -1.406094417 | 0.16038404 |
| NOC4L | -0.04935307 | 5.5401529 | -1.405219912 | 0.160643694 |
| MRAS | -0.287930214 | 1.126101344 | -1.40472234 | 0.160791572 |
| IGLV1-44 | 0.303980706 | 2.798688995 | 1.404522494 | 0.160850996 |
| MEST | 0.268668738 | 5.233631016 | 1.404508628 | 0.160855119 |
| IGLC7 | 0.301890748 | 2.163253159 | 1.404457001 | 0.160870473 |
| SIGLEC8 | -0.248910916 | -1.488215823 | -1.404452093 | 0.160871933 |
| PLEKHG4 | -0.176751779 | 2.948301737 | -1.404418309 | 0.160881981 |
| MYH14 | 0.242735769 | -5.564106695 | 1.403850151 | 0.161051037 |
| IGKV4-1 | 0.33187722 | 4.781167066 | 1.403511404 | 0.161151895 |
| IL11RA | -0.103441399 | 4.422032568 | -1.403029039 | 0.161295596 |
| PNMA3 | 0.185886409 | 1.396412196 | 1.402858926 | 0.161346298 |
| SMAD4 | -0.052372284 | 4.711414969 | -1.402706106 | 0.161391855 |
| S100A8 | 0.308665242 | 10.31427153 | 1.402381967 | 0.161488518 |
| EP400 | -0.048505207 | 5.907547889 | -1.402006837 | 0.161600441 |
| RN7SL5P | -0.120537911 | 2.906349173 | -1.401997104 | 0.161603346 |
| RP11-366L20.2 | -0.319070954 | -0.598938241 | -1.401981971 | 0.161607862 |
| CTD-2102P23.1 | 0.094439248 | 4.613251567 | 1.401957301 | 0.161615225 |
| ZBTB8OS | -0.061491176 | 4.277458769 | -1.401228214 | 0.161832938 |
| PTPN14 | -0.387747523 | 1.394423377 | -1.401103535 | 0.16187019 |
| OR2T32P | 0.19684675 | 0.004809899 | 1.400908492 | 0.16192848 |
| RP11-12M9.3 | -0.066822541 | 6.734055646 | -1.400769654 | 0.161969982 |
| ARC | 0.273839491 | -0.176082138 | 1.400568184 | 0.162030221 |
| HMGN1P36 | 0.103755234 | 4.145228489 | 1.400336819 | 0.162099419 |
| CCDC109B | 0.199362414 | 4.15346148 | 1.40013635 | 0.162159395 |
| RP11-93K22.13 | -0.154732707 | 0.616954028 | -1.399854612 | 0.162243713 |
| GCN1L1 | -0.064044327 | 6.098603245 | -1.399787378 | 0.162263839 |
| MRPS12 | -0.058384701 | 5.013407851 | -1.399663681 | 0.162300873 |
| LINC00511 | 0.190929957 | -3.199207636 | 1.399559514 | 0.162332065 |
| C9orf3 | -0.095118042 | 1.230017199 | -1.399551805 | 0.162334373 |
| RP11-70P17.1 | 0.174552468 | -0.087196134 | 1.399419619 | 0.162373962 |
| AC084018.1 | -0.098588441 | 4.615889311 | -1.398975004 | 0.162507173 |
| CTA-407F11.6 | 0.154827129 | 1.297704659 | 1.398928207 | 0.162521199 |
| RC3H1 | 0.045673115 | 5.982825982 | 1.398905126 | 0.162528117 |
| CTD-2587M2.1 | -0.142130819 | 0.065467946 | -1.398841866 | 0.162547079 |
| RP11-152H18.3 | 0.161407643 | -0.190251247 | 1.398821825 | 0.162553087 |
| PEX10 | 0.056508119 | 4.458223907 | 1.398527889 | 0.162641217 |
| COX5A | -0.058870905 | 7.669826745 | -1.398488532 | 0.16265302 |
| MIR3609 | -0.165069429 | 0.135581885 | -1.398429181 | 0.162670821 |
| IGLV1-36 | 0.221841661 | -0.109368659 | 1.398302475 | 0.162708828 |
| TMEM66 | 0.068496812 | 9.249188328 | 1.397919705 | 0.162823684 |
| RP11-645C24.5 | -0.142426911 | 0.93654612 | -1.397838653 | 0.162848013 |
| KRT2 | -0.181321507 | -2.061409528 | -1.397476355 | 0.162956795 |
| CTGLF12P | -0.186219817 | 1.407826354 | -1.397308969 | 0.163007072 |
| IFI6 | 0.172245923 | 7.030832845 | 1.397103866 | 0.163068694 |
| ADAM12 | -0.17367641 | -2.1339198 | -1.396082931 | 0.163375691 |
| RP13-93L13.2 | 0.090821824 | 1.51665536 | 1.395700698 | 0.163490742 |
| RP11-813P10.2 | 0.110541473 | 1.651086598 | 1.395367157 | 0.163591186 |
| HIGD1AP14 | 0.102286285 | 3.994265565 | 1.395309367 | 0.163608594 |
| ATP2A3 | -0.100889093 | 8.085126162 | -1.395176079 | 0.16364875 |
| C5orf30 | 0.127555714 | 4.332060164 | 1.395106283 | 0.16366978 |
| KIAA0513 | -0.183685817 | 4.423642689 | -1.394979675 | 0.163707933 |
| MCUR1 | -0.061387329 | 4.441626432 | -1.394705494 | 0.163790581 |
| SRP72 | 0.054716808 | 7.602684325 | 1.394488514 | 0.163856009 |
| RP11-216N14.5 | -0.195901971 | -1.381813144 | -1.394212772 | 0.163939185 |
| HMGN2P5 | 0.10353344 | 9.152510613 | 1.394133829 | 0.163963003 |
| C19orf66 | 0.081635771 | 5.794001306 | 1.394069391 | 0.163982447 |
| RP1-99E18.2 | 0.147864334 | 0.028877553 | 1.394014333 | 0.163999062 |
| RP11-638I2.2 | 0.130485776 | 1.57449605 | 1.39393847 | 0.164021957 |
| NFIX | -0.187365181 | 5.288868907 | -1.393803784 | 0.164062612 |
| RP11-549J18.1 | 0.139315387 | 0.409197507 | 1.393676348 | 0.164101084 |
| GGCT | -0.077185129 | 6.123331407 | -1.393496405 | 0.16415542 |
| AL592284.1 | 0.162934314 | 1.585288255 | 1.393479125 | 0.164160639 |
| RP11-50B3.4 | -0.112275839 | 1.83347784 | -1.393435838 | 0.164173713 |
| ENKUR | -0.2126731 | -2.08073424 | -1.392909822 | 0.164332641 |
| TRBV29-1 | 0.21956951 | 2.347830833 | 1.392623345 | 0.164419245 |
| DUSP7 | 0.127617005 | 4.369458721 | 1.392058688 | 0.164590047 |
| FAM127B | -0.217539579 | 4.650360716 | -1.392001792 | 0.164607264 |
| TMC4 | -0.192166303 | 0.804176516 | -1.391862609 | 0.164649389 |
| C12orf10 | 0.055406747 | 5.912213059 | 1.391773316 | 0.164676419 |
| RNF113A | -0.055441626 | 6.620579949 | -1.391680661 | 0.164704469 |
| RP11-191G24.1 | 0.183183367 | 1.25841361 | 1.391597289 | 0.164729713 |
| NFKBIL1 | -0.060344689 | 5.566035589 | -1.391502665 | 0.164758367 |
| CTD-2270N23.1 | -0.076066434 | 7.951579747 | -1.391451986 | 0.164773715 |
| NOP16 | -0.073003088 | 4.573626378 | -1.390989145 | 0.164913936 |
| ADAMTS6 | -0.20042003 | -0.096162975 | -1.390819522 | 0.164965347 |
| RP3-475N16.1 | -0.074853873 | 4.584624495 | -1.390481809 | 0.165067741 |
| XKR5 | 0.226443604 | -0.990389005 | 1.390266526 | 0.165133039 |
| PRIM1 | 0.100197499 | 4.849598314 | 1.39025476 | 0.165136609 |
| CTD-2575K13.6 | 0.181507629 | 0.877647298 | 1.390128208 | 0.165175003 |
| CHST11 | -0.074579728 | 6.957824496 | -1.390016758 | 0.165208822 |
| RP11-802E16.3 | -0.122833734 | 5.788845038 | -1.389541926 | 0.165352963 |
| NAT1 | -0.088721225 | 2.820603413 | -1.389367563 | 0.165405918 |
| VSTM1 | 0.355101762 | 5.625662341 | 1.389095781 | 0.165488483 |
| RP11-540A21.2 | 0.149858035 | -0.907507159 | 1.388958416 | 0.165530226 |
| MRPS7 | -0.046873641 | 5.193394984 | -1.388955601 | 0.165531081 |
| RP11-404K5.1 | -0.205183395 | -0.812971234 | -1.388921855 | 0.165541338 |
| KRTAP5-AS1 | 0.176538639 | -1.956780924 | 1.388556772 | 0.165652324 |
| GAS6 | 0.285395861 | 1.41991251 | 1.388471763 | 0.165678174 |
| PRELID1P4 | -0.159572428 | -0.314313204 | -1.387551048 | 0.165958356 |
| ZNF443 | -0.097599329 | 2.068837601 | -1.386819105 | 0.166181348 |
| YJU2B | 0.050188048 | 5.747567055 | 1.386311339 | 0.166336175 |
| RP3-508I15.9 | 0.182427799 | 0.397247952 | 1.386209729 | 0.166367171 |
| SEZ6L2 | 0.281556728 | -1.356138476 | 1.386039704 | 0.166419046 |
| MEF2C | -0.182396116 | 5.521513118 | -1.385997835 | 0.166431823 |
| DNAJC1 | 0.108780068 | 6.600569481 | 1.385309688 | 0.166641916 |
| RPL3P7 | 0.085663654 | 6.3179261 | 1.38448604 | 0.166893641 |
| RP11-718G2.5 | -0.233366239 | -0.912847147 | -1.384484124 | 0.166894227 |
| TUBBP2 | 0.16119166 | 0.439726608 | 1.384123974 | 0.167004387 |
| EIF3K | 0.049987284 | 7.81436741 | 1.383783441 | 0.167108598 |
| NCEH1 | -0.146580392 | 3.881471771 | -1.383440399 | 0.167213626 |
| RBMX | -0.063745085 | 7.733411226 | -1.383428 | 0.167217423 |
| POR | 0.090878395 | 4.832689871 | 1.383131586 | 0.167308216 |
| ERH | 0.056339228 | 7.569474442 | 1.382844779 | 0.167396102 |
| DNTT | -0.539136911 | 2.205879611 | -1.382331425 | 0.167553496 |
| AC093668.2 | 0.152229621 | 1.480733463 | 1.382124632 | 0.16761693 |
| NLGN2 | 0.170959082 | 2.040279441 | 1.381751883 | 0.167731317 |
| KLHDC9 | 0.186307303 | -0.413092356 | 1.38163139 | 0.167768306 |
| MCF2L | 0.200469617 | -0.063261992 | 1.381111752 | 0.167927894 |
| RP11-713C19.2 | 0.128592968 | 0.92043616 | 1.380588733 | 0.168088636 |
| hsa-mir-7162 | -0.2676929 | -2.642876001 | -1.380452637 | 0.168130482 |
| RRH | -0.141492671 | -0.90910674 | -1.380310281 | 0.168174261 |
| PSME1 | -0.055699443 | 8.953143725 | -1.38012996 | 0.168229728 |
| BCRP3 | 0.141597707 | 1.388101201 | 1.379543151 | 0.168410327 |
| RP13-487P22.1 | 0.166929008 | -1.005261715 | 1.37944756 | 0.16843976 |
| ZNF593 | 0.075595117 | 4.60344954 | 1.37933564 | 0.168474226 |
| LZTS1 | -0.222343416 | -2.560776949 | -1.378806143 | 0.168637358 |
| ACER3 | -0.079699883 | 4.803193414 | -1.378097005 | 0.168856022 |
| PICALM | -0.072305679 | 7.607035324 | -1.377950474 | 0.168901231 |
| DHRSX-IT1 | -0.138383402 | 0.859941165 | -1.377792556 | 0.168949964 |
| DAB1 | -0.176618011 | -3.376085276 | -1.377321264 | 0.169095466 |
| DEF6 | -0.063336306 | 7.639609393 | -1.377109323 | 0.16916093 |
| CHST3 | 0.252755369 | -2.945421919 | 1.376245918 | 0.169427812 |
| PEX11B | -0.06805566 | 5.47572887 | -1.376185046 | 0.16944664 |
| PSD2 | 0.24665407 | -0.941199109 | 1.376177991 | 0.169448822 |
| WDR54 | 0.084074458 | 3.653011733 | 1.376155454 | 0.169455793 |
| AC074289.1 | 0.208588998 | 3.610429388 | 1.375735158 | 0.169585839 |
| ZNF813 | -0.084972419 | 2.609897616 | -1.375611455 | 0.169624129 |
| DLGAP1-AS1 | -0.138536251 | 2.867437439 | -1.375605554 | 0.169625956 |
| SLC7A1 | 0.106915751 | 5.519077684 | 1.375222178 | 0.169744665 |
| RP11-180M15.4 | -0.167111206 | -0.136692717 | -1.375209897 | 0.169748469 |
| TFAP2A | -0.210516904 | -3.826565318 | -1.375177677 | 0.169758448 |
| FAM153C | 0.223672857 | -1.956549266 | 1.374764658 | 0.169886414 |
| RNVU1-6 | -0.185870637 | 0.768124375 | -1.374467293 | 0.169978591 |
| CST7 | 0.235813034 | 8.695184836 | 1.37405813 | 0.170105485 |
| MCFD2 | -0.044587152 | 6.336408103 | -1.373657987 | 0.17022965 |
| RP11-69L16.5 | -0.089806257 | 7.983278213 | -1.373574791 | 0.170255475 |
| ERP27 | 0.111676929 | 3.223691161 | 1.37346229 | 0.170290401 |
| KLRC4 | -0.220263776 | 0.170956986 | -1.373202211 | 0.170371162 |
| TLR3 | 0.209600407 | -0.60948754 | 1.373020867 | 0.170427491 |
| GRN | -0.157929837 | 9.661877109 | -1.372888617 | 0.170468579 |
| RP11-297A16.2 | -0.136942204 | -0.567136723 | -1.372731476 | 0.170517411 |
| GLTSCR2 | 0.093973046 | 8.498860153 | 1.372086851 | 0.170717837 |
| SLC6A13 | -0.203479689 | -2.651312682 | -1.372028963 | 0.170735844 |
| CTD-2521M24.9 | -0.123411503 | 4.417641185 | -1.371679077 | 0.170844714 |
| EHBP1L1 | -0.086057081 | 7.580132461 | -1.371460627 | 0.170912712 |
| GALM | 0.205650306 | 2.716548663 | 1.37145542 | 0.170914333 |
| FBXO25 | 0.113114068 | 4.210896522 | 1.370913399 | 0.171083141 |
| CASP10 | -0.072127043 | 4.854651752 | -1.370694021 | 0.1711515 |
| ZNF565 | 0.077201353 | 1.983916044 | 1.370666054 | 0.171160216 |
| FAM45A | -0.11698672 | 3.723653211 | -1.370435899 | 0.171231958 |
| EFR3A | -0.084047481 | 5.537610798 | -1.370299127 | 0.171274602 |
| FAM110A | 0.10439875 | 4.833968692 | 1.370282139 | 0.171279899 |
| NUTM2B | 0.146057685 | -1.223989879 | 1.370251475 | 0.171289461 |
| AC005776.1 | -0.141257655 | 1.7013381 | -1.369654707 | 0.171475633 |
| RPL3P10 | 0.138179932 | 0.57598868 | 1.369603143 | 0.171491726 |
| CTD-2114J12.1 | -0.195031351 | -1.298020872 | -1.369521275 | 0.17151728 |
| RP11-732A19.9 | -0.151049671 | -0.30957834 | -1.369512046 | 0.171520161 |
| RP11-299L17.1 | -0.110101287 | 1.10416492 | -1.369419681 | 0.171548995 |
| RP11-325K4.2 | -0.156881858 | 1.016455027 | -1.368617916 | 0.171799438 |
| NOTCH2 | -0.099930711 | 6.660402283 | -1.368205476 | 0.171928377 |
| NEXN | -0.213830696 | 2.201481349 | -1.368162558 | 0.171941798 |
| IGKV2D-24 | -0.200567251 | -0.043598362 | -1.367910561 | 0.172020619 |
| FRYL | -0.042371606 | 5.614066127 | -1.367377117 | 0.172187561 |
| PDAP1 | 0.05071845 | 6.779746702 | 1.366838023 | 0.172356395 |
| WAPAL | -0.036543868 | 6.540354443 | -1.366644174 | 0.172417135 |
| NBPF24 | 0.070999249 | 1.31619781 | 1.366082451 | 0.172593235 |
| DUOX1 | -0.273670304 | -3.939083167 | -1.365978914 | 0.172625709 |
| SPESP1 | -0.302520522 | -1.276215886 | -1.36570538 | 0.172711522 |
| SLMAP | 0.038090077 | 4.946691283 | 1.365601099 | 0.172744246 |
| TECR | -0.056341461 | 5.044137726 | -1.365457206 | 0.172789408 |
| BCL2L1 | 0.096015735 | 6.365814404 | 1.365123173 | 0.17289428 |
| DPRXP4 | -0.125444701 | 2.459190529 | -1.365046596 | 0.172918329 |
| RPS8P3 | 0.164721647 | 0.364684947 | 1.365014407 | 0.172928438 |
| RP11-23P13.7 | 0.203253059 | 2.028737719 | 1.36482919 | 0.172986618 |
| FAM229B | 0.153818801 | 1.290557552 | 1.364794864 | 0.172997402 |
| PRKCQ | 0.151742456 | 4.847865938 | 1.364676082 | 0.173034723 |
| ATP6V1G2 | -0.134893377 | 0.97480816 | -1.36422953 | 0.173175081 |
| TSEN15 | 0.046379497 | 6.015938041 | 1.364214916 | 0.173179676 |
| SEC24D | -0.09181928 | 4.392999588 | -1.364143146 | 0.173202243 |
| HBE1 | 0.288141721 | -0.428548892 | 1.36408533 | 0.173220424 |
| RPS15AP38 | -0.070807096 | 7.951042593 | -1.364084354 | 0.173220731 |
| MFGE8 | -0.171907185 | 4.201817081 | -1.36398113 | 0.173253195 |
| C8orf48 | -0.224234718 | -1.908397567 | -1.363915923 | 0.173273704 |
| GXYLT2 | 0.3232238 | -0.388704091 | 1.363694439 | 0.173343382 |
| PARP1P1 | -0.104390585 | 2.268158697 | -1.363198822 | 0.173499376 |
| NUBP1 | 0.057707895 | 5.678252206 | 1.362955677 | 0.173575944 |
| USP9Y | 0.609903881 | 0.470470049 | 1.362906072 | 0.173591568 |
| SEMA3D | -0.249714083 | -3.081574077 | -1.362801439 | 0.173624527 |
| RASGEF1A | 0.147211085 | 2.148109904 | 1.362639964 | 0.173675401 |
| ZDHHC5 | 0.049852263 | 6.399941405 | 1.3626097 | 0.173684937 |
| SEPT10 | 0.230208383 | -1.016604958 | 1.362534123 | 0.173708754 |
| CCNDBP1 | -0.050231939 | 6.193886841 | -1.362246784 | 0.173799323 |
| XBP1 | -0.09446104 | 8.777433851 | -1.362158308 | 0.173827218 |
| PRSS53 | -0.090074665 | 1.160851783 | -1.361736744 | 0.173960175 |
| IGKV3-7 | 0.191035138 | -0.045601249 | 1.361040285 | 0.174179998 |
| RP11-132A1.4 | -0.185712028 | -1.412491214 | -1.360936894 | 0.174212649 |
| RP11-65L3.4 | -0.172009634 | 3.341614367 | -1.360875209 | 0.174232131 |
| RP11-713M15.2 | 0.133796498 | 2.282647958 | 1.360269653 | 0.174423475 |
| RP11-328C8.2 | 0.088793685 | 1.431123473 | 1.360085861 | 0.17448158 |
| KLK1 | -0.262403553 | -0.484325819 | -1.359932125 | 0.174530195 |
| MOCS2 | -0.10716276 | 3.23441695 | -1.359931924 | 0.174530258 |
| ZNF783 | -0.070559187 | 4.076738741 | -1.359815052 | 0.174567222 |
| MTND5P10 | 0.188316673 | -0.829316621 | 1.359401102 | 0.174698194 |
| ACE | 0.239047836 | -0.292583853 | 1.359262499 | 0.174742063 |
| CENPBD2P | -0.055779711 | 4.481887825 | -1.359217851 | 0.174756196 |
| SH3BP1 | -0.078803431 | 6.075734276 | -1.359163073 | 0.174773538 |
| RP1-224A6.3 | 0.133095222 | 0.658216841 | 1.359093407 | 0.174795594 |
| EIF1AY | 0.590925734 | 1.812824563 | 1.359004806 | 0.174823648 |
| HNRNPH1 | -0.071496974 | 9.022338368 | -1.358967161 | 0.174835569 |
| DVL1 | 0.04568911 | 5.481583743 | 1.358957559 | 0.174838609 |
| ZCCHC24 | 0.159987712 | 2.623567711 | 1.358922079 | 0.174849845 |
| TAOK2 | -0.047221668 | 4.953655205 | -1.358919422 | 0.174850687 |
| NBPF16 | 0.096108115 | 2.485902123 | 1.358785865 | 0.174892987 |
| RETN | 0.339890149 | 5.14426002 | 1.358707791 | 0.174917718 |
| WDR25 | -0.066420899 | 2.451282247 | -1.358403365 | 0.175014174 |
| CTD-2350C19.1 | 0.158801239 | 1.99595202 | 1.357716742 | 0.175231873 |
| B4GALT5 | 0.135702222 | 7.02295606 | 1.357625736 | 0.175260743 |
| ACYP2 | -0.071736055 | 2.697162745 | -1.357061743 | 0.175439735 |
| RP11-1023L17.1 | -0.081989933 | 2.786414399 | -1.356927922 | 0.175482226 |
| WDR73 | -0.04651303 | 4.164094283 | -1.356540756 | 0.175605201 |
| CCDC57 | 0.097054495 | 4.410406329 | 1.35642604 | 0.17564165 |
| EEF1A1P38 | 0.125172917 | 3.687629725 | 1.356352729 | 0.175664947 |
| RP11-242F4.2 | 0.123952877 | 1.834878829 | 1.35626839 | 0.175691751 |
| SEC14L5 | 0.259641022 | -3.105916473 | 1.356110356 | 0.175741984 |
| KIAA1045 | 0.199254703 | -3.881993656 | 1.35592734 | 0.175800171 |
| UBE2D3P2 | 0.048403672 | 6.878197312 | 1.355762303 | 0.175852655 |
| THSD1 | 0.153342249 | 0.255257021 | 1.355609685 | 0.1759012 |
| MED19 | -0.070636806 | 3.879701691 | -1.355546687 | 0.175921241 |
| FGFBP2 | 0.230971799 | 4.058156805 | 1.355349751 | 0.175983902 |
| IFIT1P1 | 0.160541886 | -0.796575873 | 1.355338571 | 0.17598746 |
| DHRS1 | -0.07202316 | 3.773351335 | -1.355160265 | 0.176044209 |
| PINX1 | 0.071106965 | 3.065088496 | 1.35507137 | 0.176072507 |
| RP11-570P14.1 | -0.099413275 | 6.165620124 | -1.354811401 | 0.176155281 |
| PPM1H | -0.184689495 | 3.523831323 | -1.35474468 | 0.17617653 |
| PLP2 | 0.09284005 | 9.613012784 | 1.354187795 | 0.176353956 |
| VPREB1 | -0.316584981 | 0.790073457 | -1.354093659 | 0.176383961 |
| PRDM8 | 0.154588913 | 4.833989679 | 1.353623223 | 0.176533968 |
| RP11-226P1.3 | 0.156521326 | -1.285825369 | 1.35347512 | 0.176581213 |
| ATP5B | -0.053515609 | 9.977260828 | -1.353228478 | 0.176659912 |
| IGHV1-46 | 0.263043115 | 1.743675393 | 1.353173535 | 0.176677447 |
| RP11-54O7.3 | -0.303446353 | -0.98582515 | -1.352974389 | 0.176741016 |
| SNRK-AS1 | -0.139869326 | 1.330123132 | -1.352920077 | 0.176758355 |
| CEP120 | 0.057053201 | 5.213286026 | 1.352225929 | 0.176980081 |
| AL592494.5 | 0.212365552 | -2.661460562 | 1.351879664 | 0.177090763 |
| RP11-206L10.5 | -0.176795155 | -0.509501439 | -1.351863723 | 0.177095859 |
| NDUFAF7 | -0.043580324 | 4.375618752 | -1.351582899 | 0.177185663 |
| NUTM2D | 0.124059992 | 0.629840912 | 1.351564988 | 0.177191392 |
| THAP1 | -0.054569187 | 5.174236801 | -1.351485851 | 0.177216706 |
| CASKIN1 | 0.181002802 | -1.713677162 | 1.351369365 | 0.177253972 |
| IGHV1OR16-3 | 0.169732307 | -0.941509459 | 1.351346725 | 0.177261215 |
| LYPD2 | -0.246524625 | -0.778982106 | -1.351300244 | 0.177276087 |
| KRT19 | 0.271668714 | -3.028331322 | 1.35121702 | 0.177302718 |
| CLEC2L | 0.270935375 | -2.222775553 | 1.351109432 | 0.177337149 |
| JUN | -0.175166361 | 9.992454505 | -1.351040326 | 0.177359268 |
| RP5-1007M22.2 | -0.101862672 | 3.233892069 | -1.350739378 | 0.177455615 |
| SLC12A8 | -0.17429748 | -1.893758487 | -1.350683728 | 0.177473436 |
| RP11-97N19.2 | -0.237274375 | -0.772672887 | -1.350512839 | 0.177528167 |
| IGHV1OR15-1 | -0.194844644 | 1.326649604 | -1.350434696 | 0.177553198 |
| RIN3 | 0.080113545 | 6.673046233 | 1.350384194 | 0.177569377 |
| PDCD7 | -0.061211794 | 5.44631238 | -1.350180349 | 0.177634691 |
| MT-TE | 0.16400832 | 1.670719347 | 1.349953724 | 0.177707325 |
| RPS12P27 | -0.131452664 | 1.491688557 | -1.349880189 | 0.177730898 |
| SEPN1 | 0.089997406 | 6.618707953 | 1.349613416 | 0.177816436 |
| RP1-37N7.3 | -0.156426609 | -1.110861605 | -1.349529704 | 0.177843284 |
| STRBP | 0.094832148 | 4.829101824 | 1.349013474 | 0.178008914 |
| MDH1B | -0.179405171 | -1.612663281 | -1.348985336 | 0.178017945 |
| RP5-1029F21.3 | -0.189614027 | -0.415935523 | -1.348314854 | 0.178233247 |
| CEACAM1 | 0.308865887 | 3.809919031 | 1.348269573 | 0.178247794 |
| ZCCHC6 | -0.07860411 | 6.002114415 | -1.348251887 | 0.178253476 |
| PDF | -0.1051611 | 1.797303608 | -1.347849192 | 0.178382892 |
| HNRNPA1P15 | 0.148574256 | -0.301694032 | 1.347800123 | 0.178398666 |
| ZC3H10 | -0.082909162 | 2.939025717 | -1.347372277 | 0.178536251 |
| DPCD | 0.10676861 | 2.655391216 | 1.347275176 | 0.178567487 |
| LCP2 | -0.078309733 | 7.046740864 | -1.347026167 | 0.178647609 |
| RAET1E | -0.255233812 | -2.025357966 | -1.347000677 | 0.178655812 |
| NEAT1 | -0.116232896 | 9.008898351 | -1.346775972 | 0.178728139 |
| SNCA | 0.209686955 | 3.991112736 | 1.346774009 | 0.178728771 |
| NOTCH1 | 0.091076808 | 6.356539932 | 1.346584196 | 0.178789885 |
| LIPC | -0.24409139 | -0.093520139 | -1.346459724 | 0.178829969 |
| RP11-198M15.1 | -0.095886129 | 2.572851414 | -1.346395699 | 0.17885059 |
| MYRF | 0.274029763 | 0.634773778 | 1.346288308 | 0.178885182 |
| PEX19 | -0.04142747 | 5.196873456 | -1.346267724 | 0.178891813 |
| TLDC2 | -0.18791119 | -2.127966606 | -1.346085306 | 0.178950586 |
| PSD3 | 0.241536721 | 0.911852386 | 1.344988673 | 0.179304208 |
| MARVELD2 | 0.214643183 | -0.81972929 | 1.344279566 | 0.179533145 |
| RP11-1029M24.1 | 0.145884277 | 1.221114528 | 1.344182152 | 0.179564612 |
| ST13P15 | -0.067518328 | 5.78151877 | -1.343888988 | 0.179659337 |
| CD34 | 0.462643301 | 6.213038856 | 1.343676284 | 0.179728088 |
| IGLV3-19 | 0.279360313 | 2.800057159 | 1.343604054 | 0.179751439 |
| RPL5P9 | -0.0957313 | 7.837238173 | -1.343529946 | 0.179775399 |
| SMC5 | 0.053547835 | 6.676300559 | 1.342417029 | 0.180135509 |
| RNF149 | 0.116772122 | 6.520424261 | 1.34213437 | 0.180227055 |
| FADS1 | -0.128031443 | 4.953332101 | -1.341964644 | 0.180282042 |
| FBN2 | 0.348302136 | 1.988234615 | 1.341449218 | 0.180449103 |
| IGHV1OR15-3 | 0.175687794 | 0.48889973 | 1.341028113 | 0.180585678 |
| TSC22D3 | 0.131123047 | 9.222844533 | 1.340764724 | 0.180671141 |
| NAP1L2 | 0.253468767 | 0.148000256 | 1.340725318 | 0.18068393 |
| LINC00665 | -0.198184822 | 2.178809979 | -1.339944996 | 0.180937315 |
| BEST3 | -0.189806647 | -4.288665859 | -1.339857452 | 0.180965758 |
| AXDND1 | 0.162678729 | -3.231603861 | 1.339760302 | 0.180997327 |
| TSPAN4 | 0.157105054 | 3.831430962 | 1.339552402 | 0.181064897 |
| GNB5 | 0.123509166 | 3.377062036 | 1.339379705 | 0.18112104 |
| BRCA1 | -0.141864545 | 4.097359578 | -1.338969667 | 0.181254394 |
| AC010536.1 | -0.114904337 | 0.055613778 | -1.338905493 | 0.181275271 |
| TRIO | 0.128619116 | 5.347267392 | 1.338845548 | 0.181294774 |
| C10orf111 | -0.148782425 | -0.683304931 | -1.338714971 | 0.181337263 |
| SCNM1 | 0.04983108 | 5.744657934 | 1.338285465 | 0.181477074 |
| AC096677.1 | 0.233720008 | 0.654560028 | 1.33827362 | 0.181480932 |
| IER5L | 0.157144507 | 3.880875312 | 1.338174983 | 0.181513051 |
| RP1-159A19.3 | 0.069757662 | 5.204886751 | 1.338045418 | 0.181555249 |
| LA16c-349E10.1 | -0.283039452 | 1.478937783 | -1.337893003 | 0.181604897 |
| LA16c-385E7.1 | -0.15198093 | 0.188441202 | -1.33748641 | 0.181737393 |
| RPL41P1 | -0.06369794 | 6.364969703 | -1.337427099 | 0.181756727 |
| PTMAP1 | -0.147407064 | 0.457468896 | -1.337418275 | 0.181759603 |
| VEGFC | 0.252128442 | -1.215184672 | 1.337285045 | 0.181803038 |
| H2AFZP3 | 0.110958024 | 5.533434702 | 1.336902658 | 0.181927745 |
| SCN5A | 0.285899775 | -3.517803799 | 1.33657998 | 0.182033029 |
| TSPAN12 | 0.215520309 | -2.764537343 | 1.336209398 | 0.182153999 |
| LCE5A | 0.219258267 | -1.509413072 | 1.335601348 | 0.182352616 |
| NDUFB2-AS1 | -0.102074516 | 1.468138422 | -1.335415033 | 0.182413507 |
| ZNF655 | 0.060067832 | 5.981892623 | 1.334980103 | 0.182555709 |
| ZNRD1 | 0.060849859 | 4.017016243 | 1.334763168 | 0.182626667 |
| PRIM2 | 0.07118261 | 3.03470031 | 1.334754845 | 0.18262939 |
| PDE6H | -0.203711936 | 0.460827809 | -1.334674968 | 0.182655523 |
| FAM157B | 0.201232081 | 1.591742434 | 1.334034754 | 0.182865078 |
| RP11-108L7.15 | 0.149411578 | 0.003835587 | 1.333844561 | 0.182927367 |
| PRPF38B | -0.050445473 | 6.73132768 | -1.333789381 | 0.182945442 |
| HDAC3 | 0.054395725 | 6.014424809 | 1.333716019 | 0.182969474 |
| IGLV1-41 | 0.196388055 | 0.466851918 | 1.33354958 | 0.183024005 |
| COPB1 | 0.034792452 | 6.643181242 | 1.333448227 | 0.183057218 |
| RP5-1107A17.4 | 0.185684747 | -1.449817643 | 1.332946904 | 0.183221565 |
| TMPRSS3 | -0.193920868 | -2.61412672 | -1.332846321 | 0.183254552 |
| CCDC88C | -0.05056501 | 6.110556384 | -1.332335706 | 0.18342208 |
| RP11-102M11.2 | -0.154040116 | -2.200011772 | -1.331799523 | 0.183598119 |
| KLRC4-KLRK1 | 0.210633327 | -1.014032739 | 1.33163054 | 0.183653625 |
| IGKV1OR10-1 | 0.2189612 | -0.050958141 | 1.331181583 | 0.183801156 |
| FAM69B | 0.283292841 | 5.044659343 | 1.330922077 | 0.183886472 |
| ARHGAP31 | 0.213966073 | 2.920361264 | 1.330619129 | 0.183986107 |
| SOCS7 | 0.072164311 | 4.014768081 | 1.329706491 | 0.184286503 |
| OBSL1 | -0.274965776 | 3.401410346 | -1.329656168 | 0.184303077 |
| RP11-695J4.2 | -0.27311188 | 0.856862534 | -1.329601823 | 0.184320978 |
| RFPL4AL1 | -0.223369845 | -1.775197605 | -1.329245962 | 0.184438225 |
| RP11-425I13.1 | 0.073624411 | 2.292594628 | 1.329036814 | 0.184507159 |
| ELOVL4 | 0.172335619 | -2.323280857 | 1.328748821 | 0.184602112 |
| IL1RAP | 0.134772997 | 5.359964051 | 1.328398876 | 0.18471754 |
| AC005562.1 | -0.134125265 | -1.030734019 | -1.328166725 | 0.184794144 |
| MIR1254-1 | -0.10079678 | 2.840955721 | -1.32807392 | 0.184824774 |
| MLLT11 | -0.149125165 | 3.260867431 | -1.328022483 | 0.184841752 |
| RN7SL2 | -0.13717026 | 4.759248934 | -1.327901688 | 0.184881628 |
| DPY19L2P3 | -0.144291183 | 0.383628707 | -1.327877442 | 0.184889633 |
| RP11-559M23.1 | -0.141447654 | -1.01937484 | -1.327785212 | 0.184920084 |
| PAK6-AS1 | -0.183174595 | -1.612969306 | -1.327772878 | 0.184924157 |
| CTA-292E10.6 | 0.087240519 | 2.898105838 | 1.327503798 | 0.185013022 |
| SEMA3A | 0.25602891 | -1.907587721 | 1.327302554 | 0.185079505 |
| C22orf29 | -0.07145934 | 4.228860042 | -1.326885395 | 0.185217373 |
| PPFIBP2 | 0.203476049 | 1.726088379 | 1.326788953 | 0.185249257 |
| FAM221A | 0.159134722 | 1.186236758 | 1.326530792 | 0.185334627 |
| STXBP1 | -0.217706247 | 1.089354058 | -1.326335204 | 0.185399324 |
| RP11-61L19.1 | 0.155141348 | -1.314412456 | 1.326255086 | 0.18542583 |
| CADM4 | -0.161328581 | 1.17831621 | -1.326156167 | 0.185458561 |
| AC010084.1 | -0.188206606 | -1.455552232 | -1.32555013 | 0.185659182 |
| C9orf172 | 0.135082327 | 2.053697806 | 1.325411062 | 0.185705241 |
| STOX1 | -0.201957186 | -0.082846321 | -1.323781043 | 0.186245736 |
| PTPN11 | 0.047548579 | 6.797125932 | 1.323775859 | 0.186247457 |
| FCGR3B | -0.370100226 | 4.718232204 | -1.323461419 | 0.186351856 |
| MSMO1 | 0.095521973 | 3.729219055 | 1.323445769 | 0.186357053 |
| ACAT1 | 0.107594822 | 4.524605193 | 1.323262702 | 0.186417856 |
| APOBEC3A | 0.296972788 | 5.211874366 | 1.322940096 | 0.18652504 |
| ASGR2 | -0.325218616 | 2.569864761 | -1.322908265 | 0.186535618 |
| EFCAB7 | -0.060662564 | 2.552763855 | -1.322903685 | 0.18653714 |
| UBR5 | 0.044951946 | 6.424747433 | 1.322662647 | 0.186617257 |
| CD72 | 0.130708124 | 2.788506353 | 1.322520115 | 0.186664644 |
| SNX29P2 | -0.114060899 | 0.007092874 | -1.321902159 | 0.186870198 |
| RP5-1085F17.3 | -0.096511077 | 3.342477339 | -1.321718828 | 0.186931212 |
| NXN | 0.242550844 | -1.480564028 | 1.321712248 | 0.186933403 |
| AC006129.1 | 0.160958517 | 0.332780984 | 1.321706762 | 0.186935229 |
| RP11-137N23.1 | -0.088572069 | 4.196883426 | -1.320723882 | 0.187262599 |
| MTRNR2L12 | 0.114115691 | 5.237753849 | 1.320482201 | 0.187343161 |
| RNF7P1 | 0.114497262 | 2.321854081 | 1.320444268 | 0.187355808 |
| TMSB10P1 | -0.14908707 | 2.533379979 | -1.320247031 | 0.187421577 |
| RP11-91G21.1 | -0.100571617 | 1.49639379 | -1.320240633 | 0.187423711 |
| PALB2 | -0.05746447 | 3.992377913 | -1.320156572 | 0.187451747 |
| SMPD1 | 0.066946508 | 5.376207453 | 1.320090964 | 0.187473631 |
| RP11-11N9.4 | -0.29273566 | 2.241867471 | -1.319920282 | 0.187530571 |
| EML6 | -0.132643425 | -1.426111911 | -1.319573576 | 0.187646272 |
| SOX4 | -0.183183646 | 7.718535873 | -1.319469917 | 0.187680876 |
| FCHSD1 | 0.108705869 | 5.163738745 | 1.319213966 | 0.187766337 |
| AC007381.3 | -0.187468822 | -1.732326025 | -1.318414767 | 0.188033371 |
| SNRNP48 | 0.043957955 | 4.884036828 | 1.318318276 | 0.18806563 |
| AMY1A | -0.182274557 | -1.85397606 | -1.317491255 | 0.188342292 |
| CRTAM | 0.165842466 | 1.775935568 | 1.317243761 | 0.188425144 |
| RASA4B | -0.170105381 | -3.058390888 | -1.317072801 | 0.188482391 |
| RP11-393I2.4 | 0.122168967 | -0.806617364 | 1.316942967 | 0.188525875 |
| RPL5P34 | -0.072442441 | 9.323311824 | -1.316885026 | 0.188545283 |
| RP11-563H6.1 | 0.137543738 | 0.49780532 | 1.316789206 | 0.188577383 |
| BMS1P8 | -0.13168122 | -0.956184066 | -1.316464691 | 0.188686125 |
| RP11-761E20.1 | -0.214224571 | -1.39852576 | -1.316337614 | 0.18872872 |
| HSP90AB1 | -0.07831736 | 10.83675085 | -1.316197197 | 0.188775794 |
| REEP4 | 0.059150803 | 6.336731036 | 1.315840434 | 0.188895438 |
| TSPAN5 | -0.187232702 | 1.542233357 | -1.315739165 | 0.188929409 |
| AC018804.6 | 0.194918585 | 0.991045004 | 1.315032624 | 0.189166551 |
| AC007283.4 | 0.104740249 | 2.612111981 | 1.314887445 | 0.189215306 |
| GARIN5A | -0.152974401 | -0.28399888 | -1.314183422 | 0.189451866 |
| RP5-894A10.6 | -0.138950024 | 3.898944151 | -1.313939331 | 0.189533935 |
| LRRC37A3 | 0.08736012 | 1.74096281 | 1.313650409 | 0.18963111 |
| IGLJ3 | 0.173634378 | -0.638867339 | 1.313501134 | 0.189681332 |
| RP11-180N14.1 | 0.24478434 | -1.301296716 | 1.313432167 | 0.189704538 |
| IGLV5-37 | 0.202469201 | -0.592817538 | 1.312964944 | 0.189861806 |
| KCNJ12 | -0.293389888 | -1.736348418 | -1.312889939 | 0.189887062 |
| AC005062.2 | -0.196474535 | -1.923685603 | -1.312850901 | 0.189900208 |
| ABCB10 | 0.090268963 | 5.487332613 | 1.312798187 | 0.18991796 |
| RP11-161H23.5 | 0.19967105 | -0.942352301 | 1.312231308 | 0.190108944 |
| RP11-148O21.4 | -0.264801685 | -0.013498518 | -1.312058598 | 0.190167159 |
| WDR75 | 0.047708801 | 5.46204197 | 1.311544964 | 0.190340366 |
| BTF3P13 | -0.072395316 | 5.733538588 | -1.311104509 | 0.190488988 |
| ACTBP1 | -0.156153144 | -0.64240526 | -1.31102869 | 0.19051458 |
| PACS1 | 0.055719942 | 6.353304075 | 1.31056085 | 0.190672553 |
| HOXA3 | -0.414670107 | 2.459474724 | -1.309872348 | 0.190905211 |
| RPS12P23 | 0.106835434 | 2.678203307 | 1.309823909 | 0.190921587 |
| ROBO3 | 0.187424621 | 2.937912881 | 1.309806574 | 0.190927448 |
| FAM35CP | 0.099632761 | 0.572585508 | 1.309713418 | 0.190958946 |
| TRBV11-2 | -0.179119143 | 0.370989114 | -1.309704292 | 0.190962032 |
| TMEM25 | -0.153014077 | 2.019733348 | -1.309557931 | 0.191011528 |
| FSCN2 | -0.151475426 | -0.628650516 | -1.309192842 | 0.191135035 |
| RPN2 | -0.063671376 | 9.041479651 | -1.309174459 | 0.191141255 |
| LRRC37A2 | 0.088812313 | 3.668518166 | 1.309078909 | 0.191173589 |
| OR6K3 | -0.177232949 | -1.739487074 | -1.308740878 | 0.191288012 |
| RP11-212P7.2 | -0.091542779 | 2.579313765 | -1.308610497 | 0.191332159 |
| RP11-640M9.2 | 0.112820784 | 3.578011224 | 1.308094741 | 0.191506868 |
| AC006369.2 | -0.184581867 | 0.321953065 | -1.308038776 | 0.191525832 |
| GLIPR2 | -0.131075802 | 7.345614135 | -1.307805623 | 0.191604856 |
| MPL | 0.30519675 | 3.171838399 | 1.307719239 | 0.19163414 |
| IGSF9B | -0.205905781 | -1.009171452 | -1.307179072 | 0.191817334 |
| SGCE | 0.191495527 | -1.90710626 | 1.30712832 | 0.191834553 |
| RP11-554A11.9 | 0.192421145 | 0.424573517 | 1.307013794 | 0.191873413 |
| KRT18P17 | -0.232171965 | -0.713984855 | -1.30683318 | 0.191934709 |
| ESR1 | 0.242152696 | -2.345580839 | 1.306424392 | 0.192073496 |
| C7orf31 | -0.087047003 | 3.514717537 | -1.306128671 | 0.192173941 |
| MEMO1 | 0.059654821 | 2.490433848 | 1.306106942 | 0.192181323 |
| WNT9A | 0.23491983 | -2.283715379 | 1.30571995 | 0.192312832 |
| SCXB | -0.20166388 | -0.334237479 | -1.305212862 | 0.192485253 |
| RP11-553P9.1 | 0.114384562 | 4.346655814 | 1.304945907 | 0.192576069 |
| GSTA4 | 0.179926723 | 1.183869901 | 1.304905898 | 0.192589682 |
| AHDC1 | -0.111504588 | 4.763305133 | -1.304341716 | 0.192781727 |
| VWA3A | 0.143907998 | -2.910662333 | 1.304216803 | 0.192824266 |
| AGAP2-AS1 | 0.175710794 | -0.80600674 | 1.302911735 | 0.193269118 |
| RP11-742N3.1 | -0.060948442 | 10.7018015 | -1.302304632 | 0.193476316 |
| AC018766.4 | -0.071444736 | 5.348974153 | -1.302237662 | 0.193499182 |
| PPP1R9A | 0.339919157 | -2.100484313 | 1.301830706 | 0.193638176 |
| RP11-284F21.7 | -0.208657372 | -0.532158015 | -1.30128986 | 0.193823012 |
| FTLP3 | -0.086091795 | 9.531215549 | -1.301256672 | 0.193834359 |
| RP11-152N13.16 | 0.090538936 | 2.10717282 | 1.301207322 | 0.193851232 |
| IGLV2-8 | 0.279808682 | 2.946583365 | 1.301201177 | 0.193853333 |
| RGS11 | -0.216963743 | -2.744080702 | -1.300909832 | 0.193952967 |
| RPL3P2 | 0.073670077 | 6.213404001 | 1.300828352 | 0.193980838 |
| AC005540.3 | -0.13355261 | -0.310385276 | -1.300051153 | 0.194246836 |
| PABPC1 | -0.03809808 | 11.52786478 | -1.300028562 | 0.194254572 |
| PPL | -0.313252539 | -2.408289202 | -1.299893426 | 0.194300851 |
| RPL26P30 | -0.068263116 | 3.704769678 | -1.299780779 | 0.194339435 |
| KCNK9 | 0.169998748 | -2.98609772 | 1.299725032 | 0.194358532 |
| NDN | -0.386472281 | 1.059057696 | -1.299151286 | 0.194555153 |
| GFM1 | 0.047983411 | 4.470998959 | 1.299121111 | 0.194565498 |
| RP11-315I20.1 | 0.130304811 | -1.309320428 | 1.299076583 | 0.194580764 |
| PLCB1 | -0.14767036 | 3.229156672 | -1.298798867 | 0.194675999 |
| EMID1 | 0.235842134 | 4.076535459 | 1.298797039 | 0.194676626 |
| LINC01118 | 0.15667978 | -1.77494561 | 1.29871263 | 0.194705578 |
| ATP1A4 | -0.242908341 | -3.66226305 | -1.298709278 | 0.194706728 |
| PYURF | -0.048650767 | 7.469985482 | -1.298220663 | 0.194874387 |
| C10orf91 | 0.136877296 | 2.123647985 | 1.298072894 | 0.194925112 |
| RN7SL119P | 0.124931232 | 0.941132958 | 1.297644247 | 0.195072309 |
| CHADL | 0.114945914 | 0.585526207 | 1.297496749 | 0.195122979 |
| RP11-104D3.2 | 0.139795555 | -0.87918387 | 1.297363587 | 0.195168732 |
| FLT4 | 0.261841733 | -0.343325696 | 1.297144714 | 0.195243952 |
| LETM1 | -0.054067187 | 4.946872525 | -1.296589368 | 0.195434903 |
| LINC01057 | -0.214392199 | -2.101070677 | -1.295544044 | 0.195794702 |
| NEBL | 0.323284247 | -2.976680258 | 1.29550511 | 0.195808112 |
| RP11-458F8.4 | 0.113774514 | 4.537675869 | 1.295307162 | 0.195876304 |
| CDC42BPB | -0.175166737 | 3.897097435 | -1.294626042 | 0.196111078 |
| MSTN | -0.176715749 | -1.670306666 | -1.294359348 | 0.19620306 |
| hsa-mir-4539 | 0.191312572 | -0.256923976 | 1.294320674 | 0.196216402 |
| DPY19L2P2 | 0.150685996 | 2.618969955 | 1.294150753 | 0.196275027 |
| TMSB4XP4 | 0.10536983 | 3.525879612 | 1.2934525 | 0.196516069 |
| PALD1 | 0.240876829 | 2.276600365 | 1.293359049 | 0.196548346 |
| RP11-489E7.4 | 0.149110483 | 4.231324245 | 1.293246686 | 0.196587159 |
| RP11-578F21.10 | -0.247573527 | -1.842173103 | -1.293017932 | 0.196666195 |
| SUV39H2 | 0.095161179 | 3.087222691 | 1.293010313 | 0.196668828 |
| CXXC5 | 0.148403491 | 6.333472636 | 1.292788618 | 0.196745448 |
| RP11-247A12.2 | -0.145548651 | -1.471829447 | -1.292784977 | 0.196746707 |
| SYVN1 | 0.04607139 | 6.044074628 | 1.292742876 | 0.19676126 |
| STARD13 | -0.205504034 | -0.342646289 | -1.292502584 | 0.196844337 |
| TRIB1 | 0.171870856 | 7.962660769 | 1.29166748 | 0.197133261 |
| AC005071.1 | 0.126420338 | 1.93660591 | 1.291081138 | 0.197336305 |
| TRBV19 | -0.19427007 | 1.576357976 | -1.290957808 | 0.197379033 |
| RP11-629O4.1 | -0.138683228 | -0.406745585 | -1.290419764 | 0.197565518 |
| RP11-369K16.1 | -0.221880055 | -0.448982949 | -1.290135552 | 0.197664077 |
| H19 | 0.297295973 | 1.611148566 | 1.289837057 | 0.197767628 |
| COG3 | 0.040898025 | 5.527801717 | 1.289014549 | 0.198053171 |
| TTLL10 | -0.27763164 | -1.163042627 | -1.288953204 | 0.19807448 |
| RP11-27K13.3 | -0.122194873 | -1.273093625 | -1.28873655 | 0.19814975 |
| CTD-2008P7.8 | -0.205057288 | -2.207153677 | -1.288706689 | 0.198160126 |
| SACS | 0.084405275 | 4.665818499 | 1.28866481 | 0.198174678 |
| PODXL | -0.185000002 | 0.639566293 | -1.288360688 | 0.198280382 |
| RP11-486L19.2 | -0.163013611 | -2.686957109 | -1.2883275 | 0.19829192 |
| NUP107 | 0.049880512 | 5.29849727 | 1.288086449 | 0.198375734 |
| NPIPB7 | -0.138718714 | -0.103672916 | -1.287307544 | 0.198646742 |
| FIS1 | 0.054618673 | 7.031896351 | 1.287285051 | 0.198654572 |
| AC019181.2 | -0.19840414 | -0.820203095 | -1.286535966 | 0.198915468 |
| KCNK17 | 0.338549245 | 3.220760001 | 1.286535459 | 0.198915645 |
| RRM2B | -0.053233217 | 4.890002964 | -1.286046498 | 0.199086079 |
| AC092299.8 | 0.212392822 | 0.4895291 | 1.285443578 | 0.199296383 |
| AC007292.7 | 0.145317667 | -0.009777166 | 1.285304647 | 0.199344866 |
| MAL | 0.205041113 | 2.775486382 | 1.28523139 | 0.199370434 |
| ATP1A1 | -0.054113099 | 7.523979531 | -1.284829327 | 0.199510806 |
| C6orf89 | 0.057215232 | 5.86710415 | 1.283928945 | 0.199825417 |
| EIF4A1P7 | -0.111440328 | 2.071117311 | -1.283882372 | 0.199841701 |
| AC017076.5 | 0.17865216 | 1.651428258 | 1.283859705 | 0.199849626 |
| FGF17 | -0.235154437 | 0.530216002 | -1.283606769 | 0.199938081 |
| FGF2 | 0.200016139 | -3.448814255 | 1.283571861 | 0.19995029 |
| STK24-AS1 | 0.12406901 | -0.114780557 | 1.283418063 | 0.200004092 |
| NAALADL1 | -0.199886443 | 2.849942058 | -1.283357891 | 0.200025144 |
| RASSF8 | 0.223514383 | 0.727035097 | 1.28334438 | 0.200029871 |
| AC017035.2 | -0.153517834 | -0.99698997 | -1.283188185 | 0.200084526 |
| TAF5 | 0.075526842 | 3.4556647 | 1.283133286 | 0.200103739 |
| MIR24-2 | 0.150603461 | 5.708749103 | 1.283035727 | 0.200137885 |
| UTP20 | -0.06917965 | 3.640031518 | -1.282821484 | 0.200212885 |
| XXYLT1 | -0.076623641 | 4.019506539 | -1.282698892 | 0.20025581 |
| TTC38 | 0.067565554 | 5.421870748 | 1.282681225 | 0.200261997 |
| RPL34P31 | 0.097801327 | 2.448588593 | 1.28265198 | 0.200272238 |
| TTC4 | -0.088398726 | 0.797945777 | -1.282518069 | 0.200319137 |
| C6orf183 | -0.170825346 | -0.656163644 | -1.282408276 | 0.200357595 |
| FHL3 | 0.10177249 | 6.552104755 | 1.281995725 | 0.200502151 |
| UST | -0.213292688 | 1.175657535 | -1.281690322 | 0.200609213 |
| FBXO2 | 0.157901585 | -0.770248804 | 1.281664253 | 0.200618353 |
| CDK2AP1 | 0.084132951 | 5.467317622 | 1.281650699 | 0.200623106 |
| PODN | 0.181246016 | -2.500440205 | 1.280263247 | 0.201110039 |
| UBAP2L | -0.044310912 | 7.205616426 | -1.279918634 | 0.201231117 |
| ATP5LP4 | 0.092558556 | 1.556451588 | 1.279905634 | 0.201235685 |
| TG | -0.152594075 | -1.675317448 | -1.279797106 | 0.201273827 |
| GCLC | 0.069560828 | 4.795237178 | 1.279393616 | 0.201415681 |
| MPP3 | -0.227003363 | 0.001536251 | -1.279333346 | 0.201436876 |
| RP11-122K13.7 | -0.141351596 | 0.507177194 | -1.279056054 | 0.201534413 |
| MIR320B2 | 0.135030873 | 0.482508302 | 1.278918771 | 0.201582715 |
| NPIPP1 | 0.087350946 | 2.922240635 | 1.27877653 | 0.201632769 |
| TMC8 | 0.106179279 | 7.50400917 | 1.278729562 | 0.201649299 |
| SMAP1 | 0.047779244 | 5.47259857 | 1.27867341 | 0.201669063 |
| TAF8 | 0.04135352 | 4.46135422 | 1.278652816 | 0.201676312 |
| IGLV1-47 | 0.263995965 | 2.583841301 | 1.278469505 | 0.201740843 |
| PLA2G4B | -0.120041789 | -0.242514224 | -1.278122775 | 0.201862944 |
| TTC3P1 | -0.078018889 | 4.801484137 | -1.27811472 | 0.201865782 |
| PCSK4 | -0.142804056 | 2.226048654 | -1.277861492 | 0.201954991 |
| LRRC37A9P | 0.188968727 | -2.269671799 | 1.27775301 | 0.201993217 |
| CLDN12 | 0.141830035 | 1.764025659 | 1.277681985 | 0.202018247 |
| SRGN | 0.12379213 | 12.04752622 | 1.277617441 | 0.202040995 |
| ZNF587 | -0.051390717 | 4.781860638 | -1.27758347 | 0.202052968 |
| CDKL1 | -0.092201206 | 1.385156103 | -1.27724461 | 0.202172432 |
| RP11-500M8.6 | 0.092988829 | 1.510391464 | 1.277163355 | 0.202201086 |
| KIFC2 | -0.091234809 | 5.111401654 | -1.277045921 | 0.202242503 |
| PGP | 0.063501485 | 5.222585456 | 1.276240742 | 0.202526646 |
| GPR75-ASB3 | 0.086335627 | 0.355975778 | 1.276150874 | 0.202558378 |
| GSTO2 | 0.135439704 | -1.125756736 | 1.275601848 | 0.202752315 |
| MPV17L2 | 0.081629393 | 3.866262161 | 1.27546249 | 0.202801563 |
| SPCS3 | 0.040212432 | 6.980058428 | 1.275457193 | 0.202803436 |
| RPL7P19 | 0.093479637 | 2.62734229 | 1.2753299 | 0.202848428 |
| MYO3B | 0.203526346 | -4.119450209 | 1.275064537 | 0.202942245 |
| IGKV1-8 | 0.207295379 | 0.448538491 | 1.274695806 | 0.203072661 |
| APOA1BP | -0.064541453 | 6.185596103 | -1.274488133 | 0.203146139 |
| RNF32-DT | 0.198028958 | -1.566043846 | 1.2744722 | 0.203151777 |
| RP11-265N6.1 | -0.125531413 | 0.11037773 | -1.274447587 | 0.203160487 |
| VASH1 | -0.127183397 | 3.643428144 | -1.274296915 | 0.203213813 |
| GIPC1 | -0.098658696 | 3.222512652 | -1.274281731 | 0.203219187 |
| ZNF219 | 0.131159331 | 4.487771215 | 1.274073525 | 0.203292893 |
| NRG2 | -0.171392716 | -3.193826401 | -1.273682178 | 0.203431484 |
| CLEC5A | 0.300728196 | 4.081081136 | 1.273656602 | 0.203440543 |
| ZNF544 | -0.135811834 | 3.173635855 | -1.273303702 | 0.203565582 |
| CTC-308K20.1 | -0.130916813 | -1.440897649 | -1.273294944 | 0.203568686 |
| RP11-759L5.2 | -0.140513564 | -0.538594768 | -1.273190674 | 0.203605642 |
| RBBP5 | -0.061903489 | 4.669364274 | -1.272559055 | 0.203829608 |
| ZSCAN12P1 | 0.169238989 | 0.10138142 | 1.271905498 | 0.204061543 |
| SLC2A11 | -0.090224748 | 1.971062438 | -1.2716095 | 0.20416665 |
| ITGB2-AS1 | -0.189988485 | 5.282400404 | -1.27148478 | 0.204210949 |
| HDC | 0.354705409 | 3.615924342 | 1.271396111 | 0.204242448 |
| MEX3B | -0.196666707 | 4.515441363 | -1.271185773 | 0.204317182 |
| KCTD15 | -0.182035438 | 3.788977699 | -1.270963393 | 0.204396216 |
| IGKV3-11 | 0.286813994 | 3.866069728 | 1.270884716 | 0.204424183 |
| NPIPB1P | -0.129750709 | 0.044164338 | -1.270771599 | 0.204464398 |
| RPL4 | 0.052923086 | 10.11023951 | 1.270602152 | 0.20452465 |
| SYT2 | -0.296216174 | -2.881502708 | -1.270149514 | 0.204685661 |
| GNG7 | -0.136691936 | 4.311854175 | -1.269978282 | 0.204746595 |
| WBSCR27 | -0.191802815 | -1.237538553 | -1.269939017 | 0.20476057 |
| IGHV1-58 | -0.17393391 | -0.116656509 | -1.269700925 | 0.204845323 |
| SOX13 | -0.200904443 | 0.238276894 | -1.269429915 | 0.204941825 |
| SMG5 | -0.038480031 | 6.823471203 | -1.269419381 | 0.204945577 |
| PLEKHA1 | 0.150946956 | 1.402989126 | 1.269201796 | 0.205023081 |
| RNF217 | 0.297912765 | 2.906522051 | 1.269183866 | 0.205029468 |
| MLST8 | 0.044397973 | 5.126787511 | 1.268862949 | 0.20514382 |
| RDH13 | 0.096513436 | 1.900168109 | 1.268694977 | 0.205203692 |
| HCG4B | 0.137014062 | 0.032902844 | 1.268334094 | 0.205332367 |
| ZNF571 | 0.099356467 | 1.752239647 | 1.268201431 | 0.205379684 |
| SPG7 | -0.049620527 | 5.268944185 | -1.267971226 | 0.20546181 |
| ABCC2 | 0.106502055 | 1.392518171 | 1.267863987 | 0.205500076 |
| CCR6 | 0.176295873 | -2.601572263 | 1.266764308 | 0.205892772 |
| MTATP6P1 | -0.076374438 | 11.9090585 | -1.266737046 | 0.205902514 |
| RP11-489M13.1 | -0.139120689 | -0.015178015 | -1.266570808 | 0.205961927 |
| CMAS | -0.065410903 | 6.036015631 | -1.266527364 | 0.205977456 |
| TTC25 | -0.138336978 | 0.546111329 | -1.266505188 | 0.205985383 |
| NLRC3 | -0.13855246 | 4.819919167 | -1.266328636 | 0.206048502 |
| GUF1 | -0.041067606 | 5.338845921 | -1.266284866 | 0.206064152 |
| CHGB | 0.234860284 | -1.304893353 | 1.266171701 | 0.206104619 |
| HSPBAP1 | -0.079104194 | 4.25667241 | -1.266008013 | 0.206163162 |
| CDHR2 | 0.155786391 | -0.483356938 | 1.265873053 | 0.20621144 |
| MED14-AS1 | 0.095952758 | 2.175440607 | 1.265810447 | 0.206233839 |
| PLCXD1 | -0.085139202 | 5.496490197 | -1.26572574 | 0.206264147 |
| KIF28P | -0.221900739 | -1.535549052 | -1.265491384 | 0.206348016 |
| TMEM9 | 0.087453897 | 4.581064624 | 1.265431284 | 0.206369528 |
| ANXA2R | -0.113190204 | 4.807610086 | -1.265244548 | 0.206436378 |
| CREB3L2 | -0.071310609 | 6.148844138 | -1.265175815 | 0.206460989 |
| SH3GL1P2 | -0.206628759 | -0.492365084 | -1.265033485 | 0.206511957 |
| OR6N1 | -0.167775886 | -1.849955381 | -1.264255038 | 0.20679088 |
| KDELR3 | -0.1834087 | -0.623725465 | -1.264201041 | 0.206810238 |
| HAUS1P1 | -0.10798277 | 1.196524288 | -1.264147088 | 0.206829582 |
| CXCR2P1 | -0.291284419 | 1.495170041 | -1.263708901 | 0.206986729 |
| RPS26 | -0.073979929 | 5.689854176 | -1.263135218 | 0.207192602 |
| VSTM4 | 0.2899036 | -2.248021977 | 1.263111627 | 0.207201071 |
| RPSAP55 | 0.153835194 | 0.153909317 | 1.262828145 | 0.20730286 |
| LRRC37A | 0.096318445 | 1.792242689 | 1.262783026 | 0.207319064 |
| RP11-326C3.13 | -0.16894469 | 0.148802194 | -1.262778132 | 0.207320821 |
| DFNB59 | -0.13017975 | 1.377355657 | -1.261930825 | 0.207625296 |
| CHMP4C | 0.230155129 | -0.887278122 | 1.261833595 | 0.207660256 |
| IL23R | -0.154925519 | -2.748805482 | -1.261787136 | 0.207676963 |
| KCNA5 | -0.231041757 | -2.747252882 | -1.261712393 | 0.207703842 |
| TPSAB1 | 0.338855706 | 5.51377885 | 1.261701842 | 0.207707636 |
| NCR2 | -0.299958972 | -1.655527622 | -1.261636801 | 0.207731028 |
| MTND2P9 | -0.17927878 | -0.824994879 | -1.261552268 | 0.207761434 |
| E2F3-IT1 | -0.15504852 | 1.017574715 | -1.261268937 | 0.207863369 |
| PRR12 | -0.053601416 | 5.511542667 | -1.261038843 | 0.207946177 |
| RP11-487I5.4 | 0.157851437 | 0.310014704 | 1.26086524 | 0.208008671 |
| POLR1D | -0.046041984 | 6.909660471 | -1.260809436 | 0.208028762 |
| RNU6-1099P | 0.135254863 | 0.194329979 | 1.260781493 | 0.208038823 |
| RP1-40G4P.1 | -0.092357142 | 5.18249705 | -1.260764801 | 0.208044833 |
| PCNX | -0.054807675 | 6.121967351 | -1.260745879 | 0.208051646 |
| RP11-253E3.1 | 0.105991016 | 4.326254799 | 1.26065753 | 0.20808346 |
| MTURN | 0.148305046 | 6.033031179 | 1.260468077 | 0.208151693 |
| NCR1 | -0.186988736 | 1.397807903 | -1.260148934 | 0.208266671 |
| PTGR1 | -0.201882649 | 1.934771111 | -1.259827839 | 0.208382399 |
| LATS1 | -0.037577541 | 5.372419142 | -1.259585167 | 0.208469893 |
| SERF1B | 0.123570736 | 0.380286235 | 1.259579204 | 0.208472044 |
| RPL7L1P8 | -0.147956924 | 2.689202671 | -1.259285513 | 0.208577969 |
| MTND4P9 | -0.179658237 | -0.636388121 | -1.258908904 | 0.208713858 |
| LPHN3 | -0.385590811 | -2.442095193 | -1.25889019 | 0.208720612 |
| PNMA1 | 0.101893374 | 6.144265804 | 1.258622133 | 0.208817374 |
| RP11-558F24.4 | -0.208448476 | 0.144134875 | -1.258532842 | 0.208849613 |
| RCN2 | 0.05464431 | 5.171361746 | 1.258457655 | 0.208876763 |
| IGHV3-19 | 0.151793986 | -1.04346024 | 1.258070315 | 0.209016669 |
| RP11-313P13.3 | 0.130316639 | 0.396465687 | 1.257908071 | 0.209075292 |
| WT1 | -0.360535245 | 2.392030465 | -1.257706182 | 0.209148256 |
| RP11-263K19.6 | -0.116592343 | 2.214817753 | -1.257197316 | 0.209332246 |
| SOCS2-AS1 | 0.274007251 | 1.923168079 | 1.256714593 | 0.209506892 |
| LINC00671 | 0.282676394 | 0.979854267 | 1.256090006 | 0.20973302 |
| RP11-661A12.5 | -0.235599625 | 3.261545891 | -1.255859517 | 0.209816513 |
| KALP | 0.348229838 | -0.326705919 | 1.255858616 | 0.209816839 |
| DCAF11 | -0.036837067 | 6.195740134 | -1.255709774 | 0.209870769 |
| ATP2B2 | 0.25345389 | -3.705653391 | 1.255644608 | 0.209894383 |
| RP11-648M2.2 | 0.12263608 | 0.134134322 | 1.255103766 | 0.210090445 |
| ZNF581 | -0.059994541 | 5.791720112 | -1.254996532 | 0.210129334 |
| GPM6B | 0.307652574 | -1.398604355 | 1.254895005 | 0.210166159 |
| EGLN3 | 0.214060697 | -1.779119818 | 1.25439629 | 0.210347114 |
| AC004540.5 | -0.224965946 | 1.441476285 | -1.254344678 | 0.210365847 |
| UBE2D3P1 | 0.042691073 | 7.284150121 | 1.25395209 | 0.210508384 |
| ZNF707 | -0.061990184 | 2.946432406 | -1.253941675 | 0.210512167 |
| RP11-677M14.7 | -0.139187946 | -1.465461959 | -1.253380031 | 0.210716207 |
| RPL37AP8 | 0.091873498 | 3.383840771 | 1.25329753 | 0.210746191 |
| C17orf67 | -0.095032093 | 2.920622674 | -1.253016939 | 0.210848191 |
| RPS27 | 0.078545031 | 7.809756247 | 1.253007534 | 0.210851611 |
| EXOC3L2 | 0.271738844 | 0.267407513 | 1.252927049 | 0.210880876 |
| RP11-44F14.8 | -0.222558945 | -0.973915417 | -1.252746882 | 0.210946396 |
| C22orf43 | -0.144732271 | 1.173779268 | -1.252650667 | 0.210981392 |
| C1orf85 | -0.079439867 | 5.098954857 | -1.252563925 | 0.211012946 |
| TCF15 | -0.210859734 | -0.709286936 | -1.252220095 | 0.211138055 |
| CAPN11 | -0.229757634 | 2.110997896 | -1.252052906 | 0.21119891 |
| PHLDB1 | 0.205781764 | 0.410784317 | 1.252047084 | 0.211201029 |
| LINC00421 | -0.191192511 | -2.041398296 | -1.251829646 | 0.211280193 |
| PF4 | 0.362533314 | 3.231940687 | 1.251643757 | 0.211347887 |
| AL122127.2 | 0.206606823 | 0.524651306 | 1.251368004 | 0.211448336 |
| ATP5I | -0.03906453 | 7.121939986 | -1.251327487 | 0.211463099 |
| MIR4664 | -0.168858688 | -0.057989168 | -1.250971997 | 0.211592651 |
| NUS1 | 0.04747181 | 5.182320658 | 1.250954212 | 0.211599134 |
| TMEM51 | -0.220393708 | 2.55482226 | -1.250914881 | 0.211613471 |
| CAPRIN2 | -0.11235038 | 3.496312344 | -1.250162993 | 0.211887694 |
| IGKV1OR22-5 | 0.19670906 | -0.053968789 | 1.250064056 | 0.211923797 |
| CTD-3065J16.9 | -0.099256169 | 0.920071185 | -1.250050166 | 0.211928866 |
| AJUBA | -0.190567059 | -0.756142527 | -1.249958061 | 0.21196248 |
| MUC19 | -0.282143313 | -0.759966452 | -1.249807745 | 0.212017347 |
| BRWD1 | 0.046620618 | 5.574130302 | 1.249684662 | 0.212062281 |
| RP1-159M24.1 | -0.063023483 | 5.736151204 | -1.249513228 | 0.212124879 |
| PRSS3P2 | 0.259259299 | -1.634176766 | 1.249372808 | 0.212176162 |
| RP11-434O22.1 | -0.08631365 | 5.114697028 | -1.249114436 | 0.212270546 |
| SUMO2P20 | -0.097181566 | 1.571431473 | -1.248850165 | 0.212367116 |
| AC064852.5 | 0.099181099 | 1.791416861 | 1.248723398 | 0.212413451 |
| NPC1L1 | -0.162319012 | -2.893167391 | -1.248592383 | 0.212461346 |
| IGHV4-4 | 0.227615041 | 1.674371196 | 1.248460552 | 0.212509547 |
| FXYD5 | 0.076790769 | 8.976792488 | 1.248447613 | 0.212514278 |
| RP11-162O12.2 | -0.12184903 | 0.769169316 | -1.248444406 | 0.212515451 |
| RP11-53O19.1 | -0.152570106 | 0.173563601 | -1.24782455 | 0.212742199 |
| SZT2 | -0.035909053 | 5.228055547 | -1.247802375 | 0.212750314 |
| CTD-2005H7.2 | -0.162631604 | -1.101667057 | -1.247788417 | 0.212755422 |
| ZNF792 | -0.110902377 | 4.211086654 | -1.247563717 | 0.212837666 |
| AC012146.7 | -0.094909647 | 2.549295849 | -1.247368855 | 0.212909008 |
| RP11-566K19.6 | -0.286040576 | 2.734004966 | -1.247319584 | 0.21292705 |
| MICU2 | 0.032715885 | 5.861857119 | 1.24716317 | 0.212984331 |
| IGLV1-40 | 0.2831414 | 3.593104767 | 1.247147559 | 0.212990048 |
| MFSD7 | -0.175172818 | 2.77859963 | -1.247080467 | 0.213014622 |
| ABHD12B | 0.295811941 | -1.454561793 | 1.246806888 | 0.213114848 |
| KLRC1 | -0.20556059 | -0.388283988 | -1.246776912 | 0.213125832 |
| AGPAT2 | -0.06276956 | 7.235394619 | -1.246693882 | 0.213156258 |
| AC073869.1 | -0.082567543 | 1.576098306 | -1.246640569 | 0.213175796 |
| RP11-197M22.2 | 0.119165545 | 1.305540785 | 1.246530523 | 0.21321613 |
| GTF2F2 | -0.046165848 | 5.096309059 | -1.24649198 | 0.213230258 |
| RP11-51L5.3 | -0.135037378 | -0.118853319 | -1.245919815 | 0.213440063 |
| RIMKLBP1 | 0.135482463 | 0.60687857 | 1.245694952 | 0.213522559 |
| RP11-365H23.1 | -0.048808821 | 5.068499152 | -1.245528635 | 0.21358359 |
| RP11-766H1.1 | -0.061339456 | 3.916658716 | -1.245351977 | 0.213648431 |
| KCNMB1 | -0.217249237 | 1.265148293 | -1.245098966 | 0.21374132 |
| SNAPC2 | 0.045916291 | 5.045276946 | 1.245081542 | 0.213747718 |
| IFT74 | -0.060665412 | 4.166794725 | -1.244770558 | 0.213861934 |
| OXNAD1 | -0.069144017 | 3.649207777 | -1.244432445 | 0.213986163 |
| LL0XNC01-131B10.2 | 0.187259163 | 2.325089557 | 1.243571117 | 0.214302869 |
| LINC01004 | -0.083347484 | 2.441223995 | -1.243362173 | 0.214379747 |
| MRC1L1 | -0.338939245 | -0.946530766 | -1.243206425 | 0.214437066 |
| EEF1A1P6 | -0.062436998 | 10.03915329 | -1.243019355 | 0.214505926 |
| ARF5 | 0.046677599 | 7.887487466 | 1.242148687 | 0.214826631 |
| RP11-182L21.6 | -0.0964517 | 4.129568454 | -1.242120844 | 0.214836892 |
| ERV3-1 | -0.105050617 | 5.106368921 | -1.241716555 | 0.214985932 |
| IGLC6 | 0.192400449 | 0.344843896 | 1.241697792 | 0.21499285 |
| NUP93 | 0.042993684 | 5.727512114 | 1.241356217 | 0.215118832 |
| RP11-329A14.1 | -0.087005494 | 4.885161869 | -1.241231272 | 0.215164928 |
| ZRANB2 | -0.051831733 | 7.257876799 | -1.241224956 | 0.215167259 |
| C22orf26 | -0.128437069 | 0.529315005 | -1.240974309 | 0.215259753 |
| WI2-89927D4.1 | -0.094361816 | 2.148214255 | -1.240955117 | 0.215266836 |
| DPM3 | -0.06040156 | 5.351598231 | -1.240751084 | 0.215342152 |
| RP11-493K19.3 | 0.165271943 | -2.387832619 | 1.240730448 | 0.21534977 |
| SNORA53 | -0.106737753 | 1.377511551 | -1.23980535 | 0.215691503 |
| CRTAP | -0.074554549 | 6.815271915 | -1.239755905 | 0.215709779 |
| SLC16A11 | 0.143790496 | -1.756028881 | 1.239690692 | 0.215733885 |
| TEC | -0.122618855 | 5.69750479 | -1.23937611 | 0.215850198 |
| AC004951.6 | 0.090960597 | 1.470559636 | 1.239017109 | 0.215982989 |
| IGKV1-27 | 0.222783478 | 1.529448862 | 1.238992989 | 0.215991913 |
| NPM3 | -0.089598036 | 6.125080359 | -1.238990375 | 0.21599288 |
| SCLT1 | -0.067097497 | 3.844202835 | -1.238966316 | 0.216001782 |
| GCSAML | -0.302971877 | 3.887649958 | -1.238655141 | 0.216116938 |
| NONO | 0.04650652 | 8.442408866 | 1.238642777 | 0.216121515 |
| NTRK3 | -0.241689919 | -4.463556753 | -1.238425609 | 0.216201909 |
| ADAM11 | 0.15141359 | 1.114594953 | 1.238412978 | 0.216206586 |
| ZMIZ1 | 0.08497354 | 7.162450753 | 1.238147466 | 0.216304908 |
| FBXL13 | -0.162042658 | 0.693911319 | -1.237975954 | 0.216368438 |
| ASLP1 | -0.10824592 | 1.960777919 | -1.237845377 | 0.216416814 |
| CCT8 | -0.062727272 | 7.841007342 | -1.237694071 | 0.21647288 |
| RPS6 | 0.054228395 | 11.72145333 | 1.237554001 | 0.216524792 |
| RBKS | 0.142923412 | 3.349441679 | 1.237097248 | 0.216694132 |
| LURAP1 | -0.265348015 | 0.46921104 | -1.236883211 | 0.216773519 |
| RN7SL49P | -0.106538147 | 1.138928306 | -1.236658616 | 0.216856845 |
| AC005546.2 | 0.11238838 | 0.87933991 | 1.236302917 | 0.216988857 |
| MYCNOS | 0.222501788 | -1.27586907 | 1.236165162 | 0.217039999 |
| SNX7 | 0.210716544 | -1.879733806 | 1.235633318 | 0.217237527 |
| AP5B1 | 0.132288149 | 5.382127615 | 1.23497983 | 0.217480412 |
| SLC35A3 | 0.032524057 | 4.767106669 | 1.234499495 | 0.217659065 |
| TASP1 | 0.072117918 | 3.452196862 | 1.234370525 | 0.217707052 |
| EEF1A1P1 | 0.15165379 | 1.048743148 | 1.234199077 | 0.217770855 |
| HHLA2 | 0.2121436 | -0.40183919 | 1.234165623 | 0.217783306 |
| RP11-231L11.3 | 0.150866006 | 2.479673271 | 1.233547498 | 0.218013458 |
| ALDOA | 0.047646746 | 9.452436433 | 1.233446561 | 0.218051058 |
| RNA5SP195 | 0.142792413 | 0.651827052 | 1.232042497 | 0.218574563 |
| RPS4Y1 | 0.677811961 | 4.013105195 | 1.231957614 | 0.218606241 |
| CTD-2330K9.3 | -0.144248478 | -0.90001435 | -1.231672449 | 0.218712686 |
| IL9RP3 | -0.134136343 | 1.21557612 | -1.231494138 | 0.218779264 |
| ADPGK-AS1 | 0.128381194 | 1.569578997 | 1.231422723 | 0.218805934 |
| HERC6 | -0.086501927 | 3.637629063 | -1.231394445 | 0.218816495 |
| RP13-254B10.1 | -0.138048719 | 0.304318768 | -1.230644503 | 0.219096705 |
| CPNE8 | -0.284604855 | 3.35283635 | -1.230498686 | 0.219151218 |
| CHODL | -0.157186906 | -2.173243283 | -1.229956704 | 0.219353923 |
| PTMAP5 | 0.065377077 | 9.309936621 | 1.229560801 | 0.219502078 |
| NPM1P33 | -0.127461377 | -0.558757995 | -1.229492916 | 0.219527489 |
| RP11-552J9.9 | 0.131676707 | 0.859677836 | 1.229298634 | 0.219600226 |
| MYCBP2-AS1 | 0.136002428 | -1.191394982 | 1.229131562 | 0.21966279 |
| RPL22L1 | -0.080315849 | 6.030282316 | -1.229007799 | 0.219709144 |
| SLC39A13 | 0.054134628 | 4.577233287 | 1.228756789 | 0.219803179 |
| MMP28 | 0.258625069 | 1.065169332 | 1.228735607 | 0.219811115 |
| SH2D1B | -0.20847774 | 0.987283644 | -1.227681663 | 0.220206276 |
| SLC22A18AS | -0.178770957 | -0.585785701 | -1.227620762 | 0.220229125 |
| AC018766.5 | -0.061047169 | 5.636074064 | -1.2267712 | 0.220548051 |
| CTB-83J4.2 | -0.195347776 | 2.967981176 | -1.226728861 | 0.220563953 |
| RP11-12J10.3 | 0.120157981 | -0.158117636 | 1.226712547 | 0.220570081 |
| AC003002.6 | -0.130304878 | -0.888242868 | -1.226443509 | 0.220671155 |
| IGKV2D-40 | 0.19628221 | -0.182054261 | 1.225818179 | 0.220906211 |
| AC007381.2 | -0.172790479 | -1.190441944 | -1.225222551 | 0.221130269 |
| CNN2P1 | 0.142559663 | -0.124714793 | 1.225208423 | 0.221135585 |
| EPB41L4A | 0.184113058 | -1.278534173 | 1.225140016 | 0.221161329 |
| PPFIA4 | 0.217493131 | -0.007524378 | 1.224616729 | 0.22135833 |
| LBR | 0.078045299 | 7.108861063 | 1.224575308 | 0.221373929 |
| RP11-383G6.3 | 0.121606708 | 1.368455448 | 1.224362455 | 0.221454101 |
| EGFEM1P | 0.291675577 | -2.477612137 | 1.224315343 | 0.221471849 |
| TAF1C | -0.048441214 | 5.698788784 | -1.224022802 | 0.221582076 |
| ZCCHC18 | 0.171283856 | 0.783979929 | 1.223936668 | 0.221614539 |
| RP11-435F13.2 | 0.096471154 | 3.825360707 | 1.223913317 | 0.22162334 |
| RPL24P7 | 0.111467888 | 1.771018832 | 1.22304188 | 0.22195197 |
| RP11-367J11.2 | 0.165130456 | -3.037125849 | 1.223018777 | 0.221960687 |
| RP11-1024P17.1 | 0.130595863 | 2.128629571 | 1.222774322 | 0.22205294 |
| IGKV2-29 | 0.214301332 | -0.029931367 | 1.222596313 | 0.222120134 |
| PDGFRB | 0.195324941 | 0.742853688 | 1.222498751 | 0.222156968 |
| RRP36 | -0.042077499 | 6.059182039 | -1.2222762 | 0.222241007 |
| NUS1P1 | 0.052396642 | 5.760957805 | 1.222224726 | 0.222260447 |
| MRPS10 | 0.037841409 | 5.995868862 | 1.222068326 | 0.222319524 |
| LYRM2 | -0.053224012 | 3.913958289 | -1.221186626 | 0.222652776 |
| TRAK1 | -0.037721127 | 5.668811313 | -1.220971348 | 0.222734199 |
| GPR174 | -0.186506462 | 4.380055695 | -1.220731174 | 0.222825062 |
| CHST2 | 0.167209773 | 4.805437019 | 1.220514804 | 0.222906943 |
| RP11-72M17.1 | 0.166941921 | -0.847012982 | 1.22016372 | 0.223039849 |
| EFEMP2 | 0.149608209 | 1.859776528 | 1.21970593 | 0.223213235 |
| HMG20A | -0.046211172 | 5.000954842 | -1.219537952 | 0.223276881 |
| NPIPB9 | 0.127150295 | 0.279009976 | 1.219274011 | 0.223376912 |
| GLB1L3 | 0.17644115 | -4.0098357 | 1.219060512 | 0.22345785 |
| AC060834.2 | -0.208144939 | -1.636976582 | -1.218269439 | 0.22375793 |
| USF1 | 0.054857208 | 7.447044334 | 1.218072349 | 0.223832738 |
| TSPAN14 | 0.110980088 | 5.594336549 | 1.217905553 | 0.223896061 |
| EOLA1-DT | -0.115555241 | 2.538843598 | -1.217879133 | 0.223906092 |
| KRT8P10 | -0.193873505 | -1.173658859 | -1.217110717 | 0.224197994 |
| ULK3 | -0.063907261 | 6.136036577 | -1.216868319 | 0.22429013 |
| EIF4A1 | -0.099768097 | 5.786583272 | -1.216499069 | 0.224430537 |
| DYSF | 0.229354971 | 5.644229355 | 1.215532731 | 0.224798284 |
| ITM2C | 0.167598242 | 8.407005112 | 1.215424309 | 0.224839572 |
| KLHL14 | -0.217877535 | -1.911867424 | -1.21516419 | 0.224938648 |
| CDC14B | 0.202905883 | -0.135036968 | 1.215136975 | 0.224949016 |
| ERMAP | 0.069346797 | 5.049571096 | 1.215091789 | 0.224966231 |
| DNAH1 | 0.050371414 | 5.016634055 | 1.214951672 | 0.225019618 |
| RP11-285A1.1 | -0.146193786 | -1.346028093 | -1.214701029 | 0.22511514 |
| AC009093.1 | -0.134619445 | 1.106180676 | -1.214635049 | 0.22514029 |
| THAP3 | 0.056900137 | 3.249419126 | 1.214589397 | 0.225157693 |
| RPL7AP31 | 0.07243868 | 4.858767944 | 1.214418029 | 0.225223028 |
| TMEM132E | -0.168345791 | -3.636092294 | -1.214363903 | 0.225243667 |
| NRL | -0.070123405 | 1.241923162 | -1.214181999 | 0.225313038 |
| CTD-2542L18.1 | 0.163763128 | -0.365682451 | 1.214140152 | 0.225328999 |
| PPIAP9 | -0.116446769 | 2.253571826 | -1.214115277 | 0.225338487 |
| GPR153 | 0.188389731 | 1.649233314 | 1.214067705 | 0.225356633 |
| ADAM15 | 0.088836066 | 6.301779778 | 1.21400841 | 0.225379253 |
| SPATS2L | 0.191601482 | 1.919025184 | 1.213219433 | 0.22568038 |
| GOLGA8B | 0.160596926 | 4.243713607 | 1.213167197 | 0.225700327 |
| F8A3 | 0.130801165 | -0.237211776 | 1.213049448 | 0.225745295 |
| RP11-66N24.3 | 0.085210213 | 5.081262866 | 1.212882786 | 0.225808955 |
| AKAP17A | 0.054236484 | 7.518539007 | 1.212806169 | 0.225838224 |
| BAIAP2L2 | 0.114932833 | 0.904230336 | 1.212602691 | 0.225915971 |
| CTC-550B14.7 | -0.181065253 | 1.702883301 | -1.212466479 | 0.225968027 |
| NID2 | 0.211788121 | -1.399902405 | 1.212172328 | 0.226080471 |
| PLTP | -0.147474478 | 5.002453836 | -1.212072235 | 0.226118742 |
| RP11-170M17.2 | -0.055460278 | 6.985728586 | -1.211658259 | 0.226277079 |
| SPOCK3 | 0.311838488 | -1.694111719 | 1.211458603 | 0.226353471 |
| GLP1R | -0.24965243 | -2.66436399 | -1.211367581 | 0.226388304 |
| CXCL10 | -0.246603074 | 0.708660917 | -1.211310304 | 0.226410225 |
| KRT18P11 | -0.18558091 | -1.487375097 | -1.210952857 | 0.226547062 |
| RP3-455J7.4 | 0.162987694 | 0.955993332 | 1.210927476 | 0.22655678 |
| NCKIPSD | -0.065601911 | 3.940828979 | -1.210603222 | 0.226680966 |
| RP4-635E18.8 | 0.094294926 | 5.487581629 | 1.210435137 | 0.226745359 |
| PIGW | 0.069396091 | 3.651898071 | 1.21030932 | 0.226793568 |
| TCF7L1 | -0.166468404 | -1.562920037 | -1.210015201 | 0.226906294 |
| SC22CB-1E7.1 | -0.072877474 | 6.799312897 | -1.209804587 | 0.22698704 |
| UNC93B1 | -0.113048301 | 6.538064101 | -1.209738808 | 0.227012263 |
| GLRB | 0.207215411 | -2.470532734 | 1.209721663 | 0.227018837 |
| ACADS | 0.056782258 | 5.118174143 | 1.209180246 | 0.227226522 |
| UBBP1 | 0.102697499 | 1.352021531 | 1.208867769 | 0.227346449 |
| MEI4 | 0.210534929 | -2.395670855 | 1.20880356 | 0.227371098 |
| IDO1 | -0.190799613 | -2.818002674 | -1.208294315 | 0.227566655 |
| RP11-106M3.3 | 0.153406859 | -2.394281878 | 1.207404931 | 0.227908479 |
| HS3ST3A1 | 0.179705134 | -1.645276224 | 1.20704703 | 0.228046137 |
| RP11-531H8.2 | -0.198576461 | -1.040365622 | -1.206845106 | 0.228123829 |
| PRCD | -0.22375263 | -2.010365503 | -1.20632339 | 0.228324651 |
| FAM13A-AS1 | 0.099651929 | 3.964312796 | 1.205854637 | 0.228505193 |
| SERF1A | 0.130854585 | -0.77202006 | 1.205837291 | 0.228511876 |
| RP11-630I5.1 | 0.112555031 | 0.796900938 | 1.205481615 | 0.228648937 |
| UNC5B | -0.185872106 | 0.407723822 | -1.205449524 | 0.228661306 |
| FUT1 | 0.222903675 | -1.057998251 | 1.205204831 | 0.228755638 |
| LRRC58 | -0.056517731 | 5.546819299 | -1.205152602 | 0.228775776 |
| FAM81B | 0.231726943 | 0.389392268 | 1.205091214 | 0.228799448 |
| GPR110 | -0.244141733 | -5.865102512 | -1.205060468 | 0.228811304 |
| RPL5P3 | -0.076759089 | 6.219814768 | -1.204726327 | 0.228940185 |
| EFCAB8 | -0.145167088 | -1.323480403 | -1.204686022 | 0.228955735 |
| VSTM2L | 0.227733292 | -1.440747386 | 1.204677802 | 0.228958907 |
| LCN6 | 0.288526181 | -1.230336422 | 1.203926915 | 0.229248737 |
| CRB2 | -0.160004605 | -3.477259389 | -1.203866583 | 0.229272036 |
| NOXA1 | -0.129425751 | 4.776390764 | -1.203399194 | 0.229452586 |
| LIPN | 0.235300003 | 2.490679517 | 1.203128513 | 0.229557195 |
| RP5-1165K10.2 | -0.116028176 | -0.557550443 | -1.202608828 | 0.229758131 |
| COL13A1 | 0.163319457 | -3.999563447 | 1.202235143 | 0.229902694 |
| SCAMP5 | -0.156004443 | 2.056228734 | -1.20210588 | 0.229952716 |
| CLECL1 | -0.278484698 | 2.171018325 | -1.202028121 | 0.22998281 |
| SMAD5 | 0.078254412 | 4.052841388 | 1.202005179 | 0.22999169 |
| PLEKHH1 | 0.209442382 | -2.965917929 | 1.201771568 | 0.230082122 |
| H2AFY2 | -0.300769938 | 2.808862112 | -1.201720112 | 0.230102044 |
| SLC22A20 | -0.179199218 | 1.63908548 | -1.201510147 | 0.23018335 |
| SPATA6L | -0.131923731 | -1.835475633 | -1.201433261 | 0.230213128 |
| ZNF121 | 0.083773841 | 5.784195256 | 1.201432938 | 0.230213252 |
| KB-1562D12.2 | -0.143081806 | -0.273901652 | -1.201200872 | 0.230303148 |
| RP11-387H17.4 | -0.144027397 | -0.926581402 | -1.201175215 | 0.230313089 |
| TRPC4AP | 0.041290307 | 7.680727963 | 1.201143027 | 0.23032556 |
| GRK1 | 0.177439857 | -3.575707214 | 1.200907305 | 0.230416904 |
| TRIAP1 | -0.0547739 | 6.180576793 | -1.200854259 | 0.230437463 |
| TMEM44 | -0.11302919 | 3.79528269 | -1.200688026 | 0.230501899 |
| RP11-144N1.1 | 0.114767605 | 0.325470683 | 1.200675075 | 0.23050692 |
| CTAGE1 | 0.071451078 | 1.124373659 | 1.199613699 | 0.230918645 |
| HSPA2 | 0.156311386 | 0.841806714 | 1.199233832 | 0.231066129 |
| EEF1E1 | 0.067359496 | 3.804523243 | 1.199196538 | 0.231080612 |
| LRRC66 | -0.15065521 | -3.019775512 | -1.19902933 | 0.231145555 |
| PCDHB12 | 0.174746144 | -3.141478128 | 1.198948225 | 0.231177061 |
| MOB3A | -0.08603181 | 6.991742171 | -1.198741165 | 0.231257508 |
| HYKK | 0.135545317 | -1.517112982 | 1.198459371 | 0.231367023 |
| RP11-498C9.15 | 0.075406585 | 2.790506193 | 1.197669126 | 0.231674337 |
| MGC10955 | -0.141389173 | -0.75638728 | -1.196974112 | 0.231944858 |
| FRG1B | -0.096370949 | 4.926096934 | -1.196858828 | 0.231989751 |
| RAB38 | -0.175376207 | 2.740650745 | -1.19613514 | 0.23227171 |
| AC009005.2 | 0.130349762 | 3.702328938 | 1.196091961 | 0.232288541 |
| C5AR2 | 0.216166069 | 3.237115735 | 1.196086069 | 0.232290838 |
| RP11-390K5.1 | 0.106886038 | 2.580770567 | 1.195512313 | 0.232514568 |
| GEMIN4 | -0.067813174 | 4.602849062 | -1.194842481 | 0.232775956 |
| ATP8A1 | 0.110582814 | 5.368145316 | 1.194496963 | 0.232910869 |
| CTC-782O7.1 | 0.160446695 | -2.753725962 | 1.194424341 | 0.232939232 |
| RP11-761I4.3 | -0.134733297 | 0.721933052 | -1.194226047 | 0.233016691 |
| STK19P | 0.181231453 | -0.846286378 | 1.194116948 | 0.233059316 |
| KIAA1467 | -0.085053536 | 2.68606929 | -1.194017168 | 0.233098304 |
| USE1 | -0.043537766 | 5.049600256 | -1.194014818 | 0.233099223 |
| RP11-10A14.4 | 0.135620983 | -1.325091371 | 1.193117225 | 0.233450164 |
| TSIX | -0.33217577 | -3.536650657 | -1.193088131 | 0.233461545 |
| MNX1 | 0.248610381 | -3.536649052 | 1.193059464 | 0.23347276 |
| LRP11 | -0.197216418 | 2.308370536 | -1.192777388 | 0.233583131 |
| ZNF316 | -0.062446024 | 6.178643633 | -1.192709669 | 0.233609634 |
| RP4-575N6.2 | -0.115771554 | 5.289038798 | -1.192623021 | 0.233643548 |
| UBL5 | 0.037584452 | 6.771621979 | 1.192109402 | 0.23384465 |
| CDH4 | 0.341917925 | -1.705959378 | 1.191982547 | 0.233894338 |
| RP11-211A18.1 | -0.097261253 | 0.844220271 | -1.191665075 | 0.234018721 |
| PNN | 0.072736198 | 8.162706205 | 1.191074266 | 0.234250321 |
| NSA2 | -0.055235804 | 8.006158521 | -1.19105414 | 0.234258213 |
| RNF216 | 0.043196781 | 5.58596095 | 1.190567954 | 0.234448927 |
| NCR3 | -0.142527696 | 2.260355986 | -1.190471816 | 0.234486651 |
| PLB1 | -0.138747194 | 3.950176513 | -1.190295512 | 0.234555844 |
| GZMH | -0.206067596 | 4.188911105 | -1.190080626 | 0.234640199 |
| IFT122 | 0.071121112 | 4.362431618 | 1.189887998 | 0.234715835 |
| OR2L2 | 0.188653123 | -0.373822195 | 1.189553048 | 0.234847394 |
| AC009362.2 | 0.095616914 | 1.472090026 | 1.189207301 | 0.23498325 |
| HNRNPA1P67 | 0.116917536 | -0.40346778 | 1.189118048 | 0.235018329 |
| RP11-498E2.9 | -0.151275882 | 1.062083384 | -1.189017616 | 0.235057807 |
| TXNL4A | 0.033288528 | 5.720425795 | 1.188382113 | 0.235307719 |
| IGFBP7-AS1 | -0.162508923 | -0.77523426 | -1.188086701 | 0.235423954 |
| SLC4A4 | 0.197547004 | -0.734699309 | 1.187998578 | 0.235458636 |
| AP000350.4 | -0.164750929 | 1.851030367 | -1.187355432 | 0.235711861 |
| GPX8 | 0.148960522 | -3.606363086 | 1.187281751 | 0.235740883 |
| CTD-2373J19.1 | -0.075429775 | 2.05063241 | -1.18705567 | 0.235829951 |
| RP3-406A7.7 | -0.221246231 | 1.718884928 | -1.186831455 | 0.235918308 |
| RAB39B | 0.12895511 | 2.100374058 | 1.186810397 | 0.235926607 |
| C6orf106 | 0.041763087 | 7.150993134 | 1.186676254 | 0.235979482 |
| NUP210 | -0.095004637 | 7.396097697 | -1.18665131 | 0.235989315 |
| CHRM3 | 0.257230472 | -3.394488774 | 1.186392361 | 0.23609141 |
| OXSM | -0.050974695 | 3.613002135 | -1.186348487 | 0.236108712 |
| DNHD1 | 0.075242113 | 3.878961282 | 1.186038377 | 0.236231026 |
| LIN7A | 0.306668999 | 3.189385754 | 1.186021696 | 0.236237606 |
| NLGN4Y | -0.213338465 | -4.563250546 | -1.185936226 | 0.236271326 |
| TRAPPC2L | -0.045112695 | 4.006449418 | -1.185788516 | 0.236329609 |
| FAM120AOS | 0.034603851 | 5.686671999 | 1.185786461 | 0.23633042 |
| RNU6-828P | -0.12811383 | 0.044494918 | -1.18550326 | 0.236442193 |
| TRPV3 | 0.187043381 | -2.327315406 | 1.185501747 | 0.23644279 |
| C11orf94 | -0.118267832 | -0.210385993 | -1.185400452 | 0.236482778 |
| MRPL32 | -0.043740482 | 4.989868673 | -1.185348831 | 0.236503159 |
| SLC50A1 | -0.05595077 | 6.193560115 | -1.185203512 | 0.236560538 |
| RTFDC1 | 0.030533362 | 7.188613252 | 1.18462569 | 0.236788789 |
| MAP3K14-AS1 | 0.127722718 | 1.220275958 | 1.184202623 | 0.236956008 |
| AC104297.1 | 0.095087886 | 1.807950946 | 1.184111605 | 0.236991994 |
| RP3-467N11.1 | -0.094343082 | 2.338477338 | -1.183836881 | 0.237100636 |
| RELL2 | -0.12382827 | 3.78360194 | -1.183702542 | 0.237153775 |
| ZDHHC8P1 | 0.183938873 | -2.691491194 | 1.18348502 | 0.237239835 |
| SETSIP | -0.064834506 | 4.254137067 | -1.18297804 | 0.237440501 |
| RP11-372E1.1 | 0.053007387 | 5.69642669 | 1.182962361 | 0.237446709 |
| CRYGD | 0.29765403 | 0.694126128 | 1.182855479 | 0.237489029 |
| KIAA0125 | -0.284356781 | 5.753301529 | -1.182823079 | 0.237501859 |
| COL9A3 | -0.192768615 | 1.246292963 | -1.182531717 | 0.237617258 |
| NOL11 | 0.051428355 | 5.527046436 | 1.182146637 | 0.237769836 |
| CRISPLD1 | 0.233790526 | 2.084109361 | 1.181602896 | 0.237985398 |
| HPSE | -0.20329485 | 3.491297 | -1.181523471 | 0.238016897 |
| GNA11 | 0.152627045 | 1.999450557 | 1.181333145 | 0.23809239 |
| RP11-262H14.3 | -0.240508614 | -1.651689527 | -1.181296049 | 0.238107106 |
| TDG | -0.046786334 | 4.867854193 | -1.180658747 | 0.238360027 |
| SLC27A6 | -0.282118372 | -0.544061581 | -1.180570298 | 0.238395144 |
| GEMIN2 | -0.061906991 | 2.965603867 | -1.180419955 | 0.238454844 |
| SUMO2P6 | -0.107116841 | 2.041795988 | -1.180399061 | 0.238463142 |
| NPAT | -0.060305735 | 4.075708387 | -1.18035813 | 0.238479397 |
| CDK14 | 0.14977032 | 3.458770438 | 1.180193414 | 0.23854482 |
| AP000253.1 | -0.137235019 | -1.428032484 | -1.179964955 | 0.238635583 |
| EIF2AK1 | 0.053340556 | 7.343763839 | 1.179880317 | 0.238669215 |
| RCAN1 | -0.119186907 | 3.240984119 | -1.179628989 | 0.238769101 |
| PAICSP4 | -0.089346539 | 3.771632082 | -1.179420473 | 0.238851995 |
| RPL5P23 | -0.061661011 | 7.107320081 | -1.178987653 | 0.239024125 |
| NAV1 | 0.194352848 | 2.806136957 | 1.17888607 | 0.239064536 |
| CTA-246H3.11 | -0.070288914 | 3.392418836 | -1.178648744 | 0.239158968 |
| IGHV3-20 | 0.222004019 | 0.854974809 | 1.17835714 | 0.239275032 |
| RP11-1055B8.3 | -0.209524915 | -0.871196719 | -1.177954651 | 0.239435297 |
| RPS11P7 | 0.12705032 | 1.211146753 | 1.177495562 | 0.239618191 |
| MRPL23 | 0.046076475 | 5.874950889 | 1.177435763 | 0.239642022 |
| GK | -0.104438616 | 3.504665503 | -1.17735682 | 0.239673484 |
| RP11-963H1.1 | -0.081762841 | 3.178548516 | -1.177150518 | 0.239755717 |
| ENPP2 | -0.212147005 | 1.798951198 | -1.17604487 | 0.240196776 |
| XRCC6BP1 | -0.082359863 | 4.924601425 | -1.175933294 | 0.240241317 |
| RP11-771F20.1 | 0.105858275 | 1.333646919 | 1.175673297 | 0.240345131 |
| AL133245.2 | 0.128707916 | 0.24857136 | 1.175527425 | 0.24040339 |
| RSPO2 | 0.224088822 | -2.281367417 | 1.174919339 | 0.240646356 |
| IGHV3-21 | 0.254635208 | 3.365958211 | 1.174900694 | 0.240653808 |
| RP11-571I18.4 | 0.100563402 | 2.441645318 | 1.174822048 | 0.240685246 |
| ABTB2 | 0.161685237 | 2.96349838 | 1.1747409 | 0.240717686 |
| CHD3 | -0.072042211 | 7.333814743 | -1.174671407 | 0.240745469 |
| TNIP1 | 0.064006865 | 7.178223942 | 1.174134141 | 0.240960346 |
| RPL9P32 | 0.108802323 | 1.328759963 | 1.173922772 | 0.241044919 |
| ILF2P1 | 0.1304107 | -0.763221641 | 1.173835954 | 0.241079662 |
| BCL2L2-PABPN1 | 0.115032151 | 0.786481139 | 1.173202153 | 0.24133341 |
| PLEKHO1 | 0.094448434 | 6.201241028 | 1.172331029 | 0.24168248 |
| STXBP6 | -0.232146313 | -2.427881783 | -1.172278529 | 0.241703529 |
| RPS4Y2 | 0.169668474 | -1.367144457 | 1.172014538 | 0.24180939 |
| RP11-420A23.1 | -0.136845514 | 1.448254487 | -1.172014242 | 0.241809509 |
| RPS21P4 | 0.076704044 | 4.177549733 | 1.171996627 | 0.241816574 |
| KIAA1958 | 0.147256469 | 3.18220147 | 1.171956156 | 0.241832806 |
| RP11-678G14.3 | 0.335850423 | 2.448560069 | 1.171890828 | 0.24185901 |
| ANO9 | 0.16720645 | 2.261149499 | 1.171859088 | 0.241871742 |
| CTD-2036P10.3 | 0.119188893 | -0.492248679 | 1.1717214 | 0.241926978 |
| NKIRAS1 | -0.078777148 | 1.831568795 | -1.17137684 | 0.242065245 |
| RP4-725G10.4 | -0.115456927 | 1.512485128 | -1.171247878 | 0.242117009 |
| HIST1H3B | 0.200110969 | 0.512055909 | 1.171242948 | 0.242118988 |
| LINGO2 | -0.176280998 | -2.818011648 | -1.171007523 | 0.242213507 |
| PTPRD | 0.319092097 | -2.112782547 | 1.170756956 | 0.242314135 |
| RSPO1 | 0.161826252 | -1.93332669 | 1.170475309 | 0.242427279 |
| THSD7A | -0.385731103 | -2.082590292 | -1.170348376 | 0.242478283 |
| FADS2 | -0.14536263 | 5.258490038 | -1.17020906 | 0.242534271 |
| PTGS1 | 0.133258687 | 5.581269907 | 1.169680027 | 0.242746962 |
| ZNF282 | -0.057739187 | 5.366141574 | -1.169648941 | 0.242759464 |
| GCLM | 0.101187957 | 3.591491075 | 1.168979498 | 0.243028804 |
| PCED1B-AS1 | 0.160359717 | 3.457962757 | 1.168751051 | 0.243120764 |
| RP11-452C13.1 | -0.18592205 | 1.929192417 | -1.168668173 | 0.243154133 |
| GPSM1 | -0.154002422 | 5.010697091 | -1.168637263 | 0.243166579 |
| LAX1 | 0.139908686 | 3.360696186 | 1.168512315 | 0.243216893 |
| CR848007.2 | 0.172485894 | -1.724868755 | 1.168202092 | 0.243341846 |
| RP11-344P13.6 | -0.168970368 | 0.778341579 | -1.168088055 | 0.24338779 |
| RP5-1068E13.7 | 0.098935565 | 3.698162276 | 1.167991944 | 0.243426516 |
| PLEKHN1 | -0.172627121 | 0.074769551 | -1.167973722 | 0.243433858 |
| INTS4L1 | -0.124680761 | 0.590968911 | -1.167628718 | 0.243572911 |
| KRT17 | 0.31224892 | -0.785840974 | 1.167520658 | 0.243616476 |
| RP11-159H10.3 | -0.218412241 | -0.8571753 | -1.167387019 | 0.24367036 |
| RP11-689P11.2 | 0.122585851 | 0.288709931 | 1.16738439 | 0.24367142 |
| RDH16 | -0.132727031 | -0.847905713 | -1.16687638 | 0.243876332 |
| BMP4 | -0.1943767 | -2.541339068 | -1.16680728 | 0.243904213 |
| LYRM1 | -0.072892649 | 5.288137498 | -1.166702207 | 0.243946614 |
| RP11-686G8.2 | 0.117421275 | 0.385684432 | 1.16664544 | 0.243969524 |
| HNRNPKP4 | -0.042440096 | 8.67764325 | -1.166501018 | 0.244027816 |
| FAM153A | 0.197242739 | -0.974532491 | 1.166468617 | 0.244040895 |
| DNAJC17 | -0.048962989 | 3.987451666 | -1.16645079 | 0.244048091 |
| HPR | 0.229215051 | -0.660249182 | 1.16610612 | 0.244187256 |
| WI2-89031B12.1 | 0.112858549 | -0.092967972 | 1.165992287 | 0.24423323 |
| ZNF280B | -0.102196718 | 1.452297971 | -1.165689384 | 0.244355593 |
| CTD-2061E19.1 | 0.124590097 | 0.078272782 | 1.165403057 | 0.2444713 |
| RP11-299G20.2 | -0.121227344 | -0.369097332 | -1.164922869 | 0.244665433 |
| RP11-661A12.9 | 0.13124465 | -0.289338406 | 1.164552027 | 0.244815434 |
| AP3B1 | -0.037456677 | 5.749515456 | -1.164381597 | 0.244884393 |
| ITPKB-IT1 | 0.137497354 | 0.670647602 | 1.164305704 | 0.244915105 |
| YTHDF3-AS1 | 0.083913233 | 2.27350587 | 1.16393324 | 0.24506587 |
| GNAI1 | 0.256853493 | 3.643001696 | 1.163714648 | 0.245154382 |
| ZFP36L2 | -0.083566198 | 9.797060574 | -1.163569682 | 0.245213093 |
| KRT72 | -0.221634539 | 0.73188889 | -1.16287417 | 0.245494915 |
| CTD-3185P2.2 | -0.111193097 | -0.121157804 | -1.162744347 | 0.245547545 |
| RP1-179N16.6 | 0.105496166 | 2.135541176 | 1.162101873 | 0.245808118 |
| EPB41L2 | 0.191877887 | 4.000220564 | 1.161634206 | 0.245997915 |
| RP11-577H5.1 | -0.104926931 | 1.294558249 | -1.161295717 | 0.246135351 |
| RP11-286N22.8 | -0.106200304 | -0.189178519 | -1.161281463 | 0.24614114 |
| PATL2 | 0.095708596 | 3.430320725 | 1.161247314 | 0.246155009 |
| RP13-228J13.6 | 0.168808568 | -0.336542914 | 1.161051394 | 0.246234587 |
| NT5E | -0.269478085 | 1.5046684 | -1.161006791 | 0.246252706 |
| PCDHGA8 | 0.181277434 | -2.519730419 | 1.160992474 | 0.246258523 |
| RP4-584D14.7 | 0.16486864 | -0.777081387 | 1.160964818 | 0.246269758 |
| AC006129.4 | 0.138751216 | 1.387132445 | 1.160925211 | 0.246285849 |
| RP11-472I20.2 | -0.135818013 | -0.283325149 | -1.160688051 | 0.246382217 |
| FSD1 | 0.161215893 | -1.303116623 | 1.160259247 | 0.246556523 |
| RPL36AP26 | 0.122701185 | 2.687695808 | 1.160233689 | 0.246566916 |
| PANX1 | -0.065718966 | 5.011581242 | -1.159894081 | 0.24670503 |
| FASN | -0.081450075 | 6.310041143 | -1.15939448 | 0.246908311 |
| NPC1 | 0.075006242 | 3.690483947 | 1.159101973 | 0.247027383 |
| FAM201A | 0.248469868 | 0.518142385 | 1.158976313 | 0.247078548 |
| RP1-170O19.21 | -0.225221414 | -0.334062829 | -1.158800695 | 0.247150068 |
| TARS | -0.059496189 | 6.484404022 | -1.158661952 | 0.24720658 |
| ABCB8 | -0.062658154 | 3.410194982 | -1.158208641 | 0.247391285 |
| RPS3AP34 | -0.129476597 | 0.291731697 | -1.157990118 | 0.247480358 |
| RP11-351I24.3 | 0.096195685 | 3.8380678 | 1.157502752 | 0.247679097 |
| RP11-277P12.20 | 0.174411341 | -1.250829895 | 1.156112422 | 0.248246665 |
| CPSF3L | -0.028089333 | 5.615626352 | -1.156062798 | 0.248266939 |
| AC021188.4 | 0.146824112 | 2.535666224 | 1.155921883 | 0.248324518 |
| RP11-462G12.1 | -0.160525347 | -0.752086464 | -1.155407082 | 0.248534951 |
| AC137932.1 | -0.150124887 | -2.345931312 | -1.155280879 | 0.248586557 |
| LCK | -0.160244934 | 3.886189674 | -1.155077574 | 0.248669707 |
| PPP2R5B | -0.082227603 | 4.297047739 | -1.154852972 | 0.248761591 |
| REN | 0.255624232 | -1.696998833 | 1.154166403 | 0.24904261 |
| RP11-443B7.2 | 0.158292137 | 0.274640267 | 1.154149394 | 0.249049575 |
| IGHV3-48 | 0.229069442 | 2.575289213 | 1.153645913 | 0.2492558 |
| TRBV7-4 | -0.214480525 | 0.422303348 | -1.152910942 | 0.249557058 |
| BTNL10 | 0.154214199 | -1.83163295 | 1.152548086 | 0.249705883 |
| AASDHPPT | 0.044817589 | 4.415416132 | 1.152325616 | 0.24979716 |
| NDUFA2 | -0.053504949 | 5.82804481 | -1.152221455 | 0.249839904 |
| CTB-25B13.13 | -0.120265781 | 1.646333007 | -1.15211887 | 0.249882007 |
| RPL34P27 | 0.083842389 | 3.048521295 | 1.15192211 | 0.249962774 |
| SUSD4 | -0.166008061 | -1.547207228 | -1.151818556 | 0.250005288 |
| GJC2 | -0.14987772 | 0.53987637 | -1.151626494 | 0.250084154 |
| FLRT3 | -0.218827117 | -3.719921816 | -1.151603153 | 0.25009374 |
| RP11-1223D19.1 | 0.15080019 | -2.525881071 | 1.151601765 | 0.25009431 |
| TRRAP | 0.044082847 | 5.821167482 | 1.151013223 | 0.250336096 |
| RFESD | -0.150528823 | 0.276509612 | -1.150973752 | 0.250352317 |
| GOLGA6L20 | 0.14752821 | -2.071179384 | 1.150633508 | 0.250492178 |
| MESTP4 | 0.202913146 | 0.495410769 | 1.150275682 | 0.250639325 |
| RP1-223E5.4 | 0.084355294 | 1.720817864 | 1.150249485 | 0.250650101 |
| PPM1J | 0.193570807 | -0.705019687 | 1.14989843 | 0.250794526 |
| ANKRD20A5P | -0.190988161 | -0.232046473 | -1.149781284 | 0.250842734 |
| KDM6B | 0.076635879 | 6.579245483 | 1.149304846 | 0.251038863 |
| SCG5 | 0.133088664 | -0.432356783 | 1.149184719 | 0.251088331 |
| IGKV1-9 | 0.234887146 | 2.144851111 | 1.149125829 | 0.251112584 |
| RP11-213G2.1 | -0.168392726 | -0.088320322 | -1.148911394 | 0.251200911 |
| RP4-789D17.5 | -0.119792249 | 0.487106585 | -1.148625873 | 0.251318552 |
| SALL2 | 0.201075848 | -0.589864026 | 1.14835626 | 0.251429674 |
| ZNF532 | 0.243026773 | 1.662998496 | 1.148343046 | 0.251435121 |
| RSKR | -0.075173597 | 2.211305835 | -1.147752713 | 0.251678553 |
| IGHV3-9 | 0.248512499 | 2.106225977 | 1.14652104 | 0.252186981 |
| ALOX5AP | 0.185714536 | 7.950758336 | 1.146504592 | 0.252193776 |
| CD52 | -0.199761453 | 8.416838027 | -1.146435914 | 0.252222147 |
| DLX2 | 0.188022331 | -1.671013898 | 1.146278051 | 0.25228737 |
| JADE3 | 0.082974343 | 3.439077241 | 1.146064345 | 0.252375685 |
| FAM3B | 0.199986725 | -3.208105951 | 1.145736531 | 0.252511197 |
| KCNE1 | -0.221953019 | 1.405041578 | -1.145490656 | 0.25261287 |
| RP11-368I7.4 | -0.113969498 | 0.300298305 | -1.145283134 | 0.252698706 |
| AC092610.12 | 0.117715781 | 0.723672909 | 1.14491164 | 0.252852415 |
| OVCH1-AS1 | 0.285712692 | -1.270008759 | 1.144744181 | 0.252921724 |
| SNX32 | -0.119444009 | 1.50742132 | -1.144740096 | 0.252923415 |
| UXT | 0.042174543 | 7.589483817 | 1.144424857 | 0.253053925 |
| SOAT2 | -0.142739472 | -1.430043242 | -1.144296251 | 0.253107182 |
| LRP4 | 0.243779546 | -0.921465153 | 1.144281097 | 0.253113458 |
| BTNL3 | -0.26134638 | -1.294423759 | -1.143939672 | 0.253254886 |
| RP11-573G6.9 | -0.154382445 | -0.362594011 | -1.143530971 | 0.253424253 |
| PRDX2P3 | -0.132829823 | 0.121795734 | -1.143228952 | 0.253549462 |
| PMS2P2 | 0.132333776 | 0.941481468 | 1.142986415 | 0.253650043 |
| EIF3FP1 | 0.124535206 | -0.018657135 | 1.142271288 | 0.25394677 |
| RP11-396K3.1 | -0.055441452 | 5.285869107 | -1.142013701 | 0.25405371 |
| TEAD4 | 0.228474795 | -0.508562769 | 1.141722239 | 0.254174751 |
| CNPPD1 | 0.03774468 | 7.064049869 | 1.141674848 | 0.254194436 |
| AC015849.16 | 0.165469714 | -1.727406488 | 1.141138733 | 0.254417196 |
| FAM228A | -0.126929579 | -1.418108235 | -1.141116962 | 0.254426245 |
| CCDC162P | -0.200882457 | 0.411991981 | -1.140701851 | 0.254598825 |
| RP11-98I9.4 | 0.076000136 | 3.265225998 | 1.140677874 | 0.254608796 |
| RP11-137J7.3 | 0.095386109 | 1.246420714 | 1.140666247 | 0.254613631 |
| LRP10 | 0.078404606 | 6.690956808 | 1.140229439 | 0.254795327 |
| NPM1P50 | 0.13654911 | -0.037630204 | 1.139823718 | 0.254964173 |
| RP11-9M16.2 | 0.10541138 | 1.586273206 | 1.139741311 | 0.254998477 |
| SLC30A3 | 0.173910934 | -3.805133058 | 1.139484829 | 0.255105265 |
| ASS1P1 | -0.163347621 | 3.232375188 | -1.139310207 | 0.255177989 |
| SHARPIN | -0.032988697 | 6.317251511 | -1.139132728 | 0.255251917 |
| MT-ND5 | -0.031839048 | 12.71145363 | -1.139057523 | 0.255283248 |
| RPS23P2 | -0.127123572 | -0.19999785 | -1.139039369 | 0.255290811 |
| ZNF721 | -0.127244867 | 3.859881387 | -1.138879039 | 0.255357615 |
| ANKRD11 | 0.038120622 | 6.289414732 | 1.138397394 | 0.255558375 |
| DNMT3B | -0.180468433 | 4.688419339 | -1.138291675 | 0.255602455 |
| AC005795.1 | -0.052679838 | 7.768476579 | -1.137895837 | 0.255767551 |
| NPM1P21 | 0.066711663 | 3.065702252 | 1.137708827 | 0.255845575 |
| RNA5SP82 | 0.088660685 | 2.81346625 | 1.13734437 | 0.255997681 |
| RP11-293M10.2 | -0.149390889 | -0.582124255 | -1.137327649 | 0.256004661 |
| ENAH | -0.281599617 | -0.756550031 | -1.137310015 | 0.256012022 |
| RP11-25K21.6 | -0.178619564 | 0.342149205 | -1.137117204 | 0.25609252 |
| RP1-266L20.9 | 0.076350516 | 2.131631304 | 1.136344151 | 0.256415444 |
| TEX15 | 0.259100462 | -4.151750145 | 1.136107268 | 0.256514453 |
| CAPZA2 | -0.060598397 | 5.848822917 | -1.135939328 | 0.256584663 |
| C19orf70 | -0.041794254 | 4.966719675 | -1.135678762 | 0.256693622 |
| AC010525.7 | 0.110600865 | 0.421065315 | 1.135183457 | 0.256900829 |
| CD24P4 | 0.274624408 | 6.008459485 | 1.134996912 | 0.256978899 |
| LINC00264 | 0.142386094 | 2.699307464 | 1.134567611 | 0.257158626 |
| RP11-254G11.1 | 0.100173883 | 0.283743332 | 1.134474487 | 0.257197624 |
| ZNF618 | 0.099249965 | 4.078761731 | 1.134377907 | 0.257238074 |
| GNAQP1 | 0.054710601 | 5.098304696 | 1.13398036 | 0.257404621 |
| GSG1L | 0.211368313 | -3.779864916 | 1.133843803 | 0.257461847 |
| RP11-1094M14.9 | -0.135750641 | -0.936519544 | -1.133497135 | 0.257607163 |
| NT5M | 0.120266061 | 4.590230722 | 1.133457342 | 0.257623847 |
| DDX11L1 | -0.158368553 | -1.576954497 | -1.133242719 | 0.257713845 |
| RP11-348F1.2 | 0.142181001 | -1.486028516 | 1.132920787 | 0.257848882 |
| RFX2 | 0.101040098 | 4.180362715 | 1.132651293 | 0.257961961 |
| AC004878.3 | -0.128267674 | 0.592355627 | -1.132518298 | 0.258017779 |
| IGKV1-16 | 0.204759709 | 1.594765257 | 1.132356995 | 0.258085488 |
| MUC20 | -0.145889239 | -0.121446711 | -1.131638513 | 0.258387231 |
| RPS11 | 0.038207243 | 11.7838317 | 1.131624816 | 0.258392986 |
| KATNAL1 | 0.069789173 | 3.867872746 | 1.131262877 | 0.258545086 |
| IGKV3D-7 | 0.13811615 | -0.99336369 | 1.131062655 | 0.258629253 |
| ZNF569 | 0.056198817 | 2.8511784 | 1.131030145 | 0.258642921 |
| MFSD10 | -0.07425052 | 7.900285576 | -1.13099174 | 0.258659068 |
| SPAG16 | 0.17687887 | 0.032690363 | 1.130856095 | 0.258716104 |
| GSTCD | -0.062969352 | 2.985208056 | -1.130225714 | 0.258981284 |
| CCDC138 | -0.066796282 | 2.379738425 | -1.130067476 | 0.259047879 |
| ADC | -0.158086141 | -1.420771 | -1.129748075 | 0.259182336 |
| LRRC7 | 0.217145479 | -4.072490852 | 1.129638457 | 0.259228493 |
| CASS4 | 0.127252821 | 3.901058526 | 1.129482117 | 0.259294332 |
| GFI1 | 0.145690033 | 6.548847863 | 1.129411541 | 0.259324058 |
| PRDM10-DT | 0.124930311 | -1.135552773 | 1.128933931 | 0.259525283 |
| SETP8 | 0.079686727 | 2.343007983 | 1.128933223 | 0.259525581 |
| MIR4737 | -0.119485647 | 0.695732623 | -1.128921952 | 0.259530331 |
| SPIB | -0.200540124 | 0.064442958 | -1.128744636 | 0.259605066 |
| RN7SL4P | -0.11836202 | 2.162285043 | -1.128254499 | 0.259811725 |
| PPIAP19 | -0.093546262 | 3.613013682 | -1.127456876 | 0.260148276 |
| SEPT4 | 0.177097261 | -1.373839906 | 1.12715991 | 0.260273656 |
| PFN4 | -0.127028643 | -0.204044078 | -1.127049306 | 0.260320364 |
| PRTN3 | 0.301083474 | 8.655070336 | 1.126235169 | 0.260664353 |
| LSM1 | 0.03509384 | 6.213365667 | 1.125825777 | 0.260837448 |
| PPM1N | 0.120062472 | 1.213152587 | 1.125424392 | 0.261007235 |
| CTD-2538C1.2 | -0.091034583 | 3.522921092 | -1.125420551 | 0.26100886 |
| MYL6 | 0.050676417 | 8.343611992 | 1.125272218 | 0.261071625 |
| RP11-499P20.2 | 0.087705215 | 3.004880615 | 1.124837503 | 0.261255629 |
| DYNLT3P2 | 0.114468061 | 0.648422323 | 1.124717754 | 0.261306332 |
| B3GNT4 | 0.136308517 | -3.021533237 | 1.124484358 | 0.261405173 |
| RP11-15E18.5 | 0.111724246 | -0.003662415 | 1.124207227 | 0.261522569 |
| MAU2 | 0.030488539 | 5.442611979 | 1.123719579 | 0.261729231 |
| EML3 | 0.042009447 | 6.228364519 | 1.123626836 | 0.261768548 |
| DYNC1LI1 | 0.034928537 | 5.325082252 | 1.123287785 | 0.261912318 |
| CNTROB | -0.063454476 | 6.047736855 | -1.122719049 | 0.262153605 |
| RP11-15N24.4 | 0.110213544 | 0.70329802 | 1.122496398 | 0.262248107 |
| AFAP1L1 | 0.241100326 | -0.819583308 | 1.122243932 | 0.262355292 |
| CD300LD | 0.172154941 | -1.424699669 | 1.122198143 | 0.262374735 |
| HOXA10-AS | -0.299426288 | 2.975732204 | -1.121934722 | 0.262486609 |
| AL807752.1 | -0.103532631 | 2.469257567 | -1.121727945 | 0.26257445 |
| RHOXF1 | -0.160809256 | -0.349366235 | -1.121531776 | 0.262657803 |
| AGRP | -0.193871537 | -1.027547633 | -1.121443684 | 0.26269524 |
| LINC01108 | -0.272510937 | -2.29525657 | -1.121170475 | 0.26281137 |
| PARP12 | 0.086311225 | 4.937976697 | 1.120658224 | 0.263029203 |
| CCDC152 | -0.203458462 | -0.245206917 | -1.120492157 | 0.263099849 |
| KIAA1257 | 0.201184628 | -0.934635282 | 1.120477566 | 0.263106057 |
| TMEM115 | -0.050772606 | 5.546811838 | -1.119730588 | 0.263423994 |
| SLC5A2 | -0.117561651 | -1.864178443 | -1.119716929 | 0.263429811 |
| CCL25 | 0.183710493 | -0.629478141 | 1.119549079 | 0.263501291 |
| RP13-297E16.4 | -0.173494172 | 2.963025427 | -1.119188482 | 0.263654899 |
| LRRC43 | 0.149641216 | 0.084552463 | 1.119098183 | 0.263693375 |
| MRC1 | -0.287372365 | -1.659228298 | -1.119092433 | 0.263695825 |
| RBP5 | -0.145216658 | -0.840767012 | -1.119080024 | 0.263701112 |
| ARHGEF37 | 0.162804427 | -0.51374766 | 1.119016728 | 0.263728085 |
| ICAM3 | 0.103765728 | 8.492877243 | 1.118976178 | 0.263745366 |
| LINC00969 | -0.070391302 | 2.781294293 | -1.118875045 | 0.263788468 |
| OSTCP8 | 0.134219412 | -0.56405466 | 1.118756789 | 0.263838874 |
| CAPN12 | 0.133953963 | 0.951975375 | 1.118442991 | 0.263972662 |
| AC083873.4 | -0.049075513 | 5.618074507 | -1.118090653 | 0.264122937 |
| NPIPA1 | 0.063009152 | 3.201685486 | 1.118081795 | 0.264126716 |
| RN7SL608P | -0.109301543 | 1.855962226 | -1.117478754 | 0.264384057 |
| AL122127.3 | 0.185579648 | 0.681116578 | 1.117323281 | 0.264450432 |
| AGXT | -0.295052518 | -3.227138731 | -1.117292825 | 0.264463436 |
| INMT | 0.158004276 | -2.639604365 | 1.117090322 | 0.264549908 |
| CTC-246B18.10 | -0.110995933 | 2.27374196 | -1.116561101 | 0.264775989 |
| SHOC2 | 0.040192635 | 6.800045085 | 1.116196167 | 0.264931965 |
| DHFR | 0.101947948 | 5.326505099 | 1.115896434 | 0.26506012 |
| SLC6A19 | 0.242853056 | -3.300095809 | 1.11560154 | 0.265186249 |
| ZNRF2P2 | -0.122906238 | 3.285755049 | -1.115552084 | 0.265207406 |
| C19orf40 | 0.081178094 | 2.500241246 | 1.115486889 | 0.265235298 |
| CTC-512J14.5 | 0.038157656 | 4.571361075 | 1.11499907 | 0.26544406 |
| KRTAP5-9 | -0.140366473 | 0.009809942 | -1.114743361 | 0.265553536 |
| CTD-2278I10.4 | -0.112120937 | 1.24058818 | -1.114516268 | 0.265650787 |
| TATDN3 | 0.052581743 | 4.061746083 | 1.114325388 | 0.265732549 |
| RP11-54D18.4 | 0.074829783 | 5.628157893 | 1.114064351 | 0.26584439 |
| ATG3 | -0.06496217 | 6.361635028 | -1.113986693 | 0.265877669 |
| SUPT7L | -0.032574196 | 6.043385272 | -1.113911675 | 0.265909819 |
| EFHD1 | 0.123345313 | -2.423272167 | 1.113581864 | 0.266051197 |
| KRT18P31 | 0.11371859 | 0.687091829 | 1.113475132 | 0.26609696 |
| ZBTB45 | -0.057313239 | 3.846170563 | -1.113283749 | 0.266179033 |
| IGSF11 | 0.157768018 | -3.554267856 | 1.11317356 | 0.266226294 |
| RP11-804F13.2 | -0.120719535 | 0.429070687 | -1.112933537 | 0.266329263 |
| TERT | 0.2026594 | -1.10159266 | 1.112649984 | 0.266450941 |
| ZBTB9 | -0.077281271 | 3.645353173 | -1.11228964 | 0.266605627 |
| RP11-627K11.1 | -0.064765143 | 6.515777757 | -1.111817768 | 0.266808282 |
| RP11-649A18.4 | 0.103992037 | 2.240922531 | 1.111036574 | 0.267144017 |
| PCDHGB8P | 0.15301517 | -1.925588468 | 1.110615658 | 0.267325035 |
| RP11-8H2.1 | -0.108167101 | -0.299137039 | -1.110522434 | 0.267365138 |
| NPM1P19 | -0.099835758 | 2.080878476 | -1.110504017 | 0.267373061 |
| RP11-558O12.1 | 0.098186107 | 2.187711675 | 1.10980109 | 0.267675586 |
| RP11-410E4.1 | -0.112328651 | -0.76014911 | -1.109663192 | 0.267734963 |
| SAPCD1-AS1 | 0.137215518 | 1.557419915 | 1.10960227 | 0.267761197 |
| HAGH | -0.066643719 | 4.168791243 | -1.109262961 | 0.267907346 |
| NDUFA3 | -0.038914802 | 5.393487758 | -1.108384412 | 0.268286013 |
| MT-TI | -0.135662733 | 5.870449209 | -1.108139486 | 0.268391645 |
| DNAH10OS | -0.138620704 | -0.095633615 | -1.108117686 | 0.268401048 |
| GPC6 | -0.331286429 | -2.461342121 | -1.10803047 | 0.268438671 |
| TTC8 | 0.120096962 | 3.372579804 | 1.107839073 | 0.268521247 |
| CTD-2552B11.2 | 0.117846144 | -0.634834996 | 1.107670145 | 0.268594144 |
| GGACT | -0.087152535 | 2.229806377 | -1.10717846 | 0.268806396 |
| FAM151B | -0.141099634 | 2.214129425 | -1.107123681 | 0.26883005 |
| RP11-423H2.3 | -0.121927743 | 0.882017157 | -1.107122988 | 0.268830349 |
| OSBPL3 | -0.096233367 | 4.704870706 | -1.106844336 | 0.268950697 |
| CCDC88B | -0.088539466 | 6.837580769 | -1.106329492 | 0.269173152 |
| CTD-2090I13.2 | -0.11640273 | -0.414583628 | -1.106125114 | 0.269261496 |
| RP3-508I15.20 | -0.1018641 | 1.424727321 | -1.106019435 | 0.269307183 |
| IGHV3-73 | 0.188521775 | 1.17516032 | 1.10509583 | 0.269706712 |
| ZNF883 | 0.212409173 | -0.7724752 | 1.104990839 | 0.269752154 |
| CLCN6 | 0.043249269 | 4.830822259 | 1.104842608 | 0.269816321 |
| IGHJ2P | 0.205705773 | 0.786046379 | 1.10484039 | 0.269817281 |
| PC | 0.148308067 | 0.994566019 | 1.104661861 | 0.269894577 |
| TMEM27 | -0.116919463 | -0.304671137 | -1.104600659 | 0.269921078 |
| IGLV2-18 | 0.192415945 | 1.1141859 | 1.104575286 | 0.269932066 |
| LAMA3 | 0.269900167 | -1.543011469 | 1.104369364 | 0.270021249 |
| NAT9 | -0.052457621 | 4.801824925 | -1.10411967 | 0.270129417 |
| DDX59 | 0.036725696 | 5.061414401 | 1.103872657 | 0.270236453 |
| SNHG1 | -0.077668828 | 7.145952292 | -1.103442366 | 0.270422977 |
| RUNX2 | -0.094382765 | 5.89900004 | -1.103227093 | 0.270516328 |
| RPS3AP25 | 0.093360716 | 2.299586276 | 1.102117765 | 0.270997725 |
| RAI1 | -0.064974392 | 4.886391125 | -1.102103908 | 0.271003742 |
| RP11-566F5.1 | 0.082964074 | 1.901044026 | 1.101719918 | 0.271170516 |
| CCDC171 | 0.119336064 | 1.942736911 | 1.101536193 | 0.271250336 |
| RN7SL441P | 0.121491949 | 0.026692042 | 1.10113116 | 0.271426362 |
| SCN2A | -0.290880314 | -3.603519412 | -1.1011162 | 0.271432865 |
| POLK | -0.039359355 | 4.710439093 | -1.100798254 | 0.271571099 |
| OLIG2 | -0.240185442 | -2.534115365 | -1.100405066 | 0.271742115 |
| TLE2 | 0.154372583 | -0.092148974 | 1.100210409 | 0.271826807 |
| EEF1A1P24 | -0.132624046 | -0.509783946 | -1.100141287 | 0.271856885 |
| IGHV3-23 | 0.256255351 | 5.128923481 | 1.099821738 | 0.271995966 |
| RP11-195F19.9 | 0.166138251 | 1.908650909 | 1.099386744 | 0.272185372 |
| FBXO4 | 0.044069356 | 4.115902713 | 1.099052713 | 0.272330878 |
| KRT18P5 | 0.111864402 | -0.232032315 | 1.098744188 | 0.272465321 |
| RP13-150K15.1 | -0.113642977 | -0.6029744 | -1.098614744 | 0.272521741 |
| HSPB1 | -0.115876948 | 6.906232771 | -1.098266372 | 0.272673624 |
| ZNF214 | -0.156077219 | -3.199770662 | -1.098152432 | 0.272723312 |
| RP11-726G1.1 | 0.20456954 | -0.380379207 | 1.098097779 | 0.272747148 |
| RP11-344E13.3 | 0.195624311 | -3.39286465 | 1.097625585 | 0.272953146 |
| POLR2I | 0.043144822 | 5.234323558 | 1.097364172 | 0.273067234 |
| RP11-574F21.2 | -0.112600965 | 0.810097565 | -1.097298328 | 0.273095976 |
| KLRG2 | -0.189839824 | -1.165419634 | -1.097120279 | 0.273173707 |
| GJA3 | -0.261611901 | -2.974133462 | -1.096710364 | 0.273352721 |
| MAML2 | 0.131603645 | 3.393195194 | 1.096564113 | 0.273416609 |
| MT-ND4 | -0.039368315 | 13.82512232 | -1.096509983 | 0.273440258 |
| WDHD1 | 0.102150255 | 3.527117298 | 1.096502571 | 0.273443496 |
| LRP5 | 0.145984445 | 3.934823918 | 1.09630897 | 0.273528091 |
| GRAP2 | 0.174272102 | 2.599917412 | 1.09575258 | 0.273771307 |
| MIR186 | -0.101697429 | 1.872080342 | -1.095637362 | 0.273821692 |
| LRRC14 | -0.04089104 | 4.56303863 | -1.095545875 | 0.273861703 |
| AC093609.1 | 0.132682241 | 0.380519651 | 1.095488267 | 0.273886899 |
| AC008440.5 | 0.145752726 | -0.381791875 | 1.094741633 | 0.274213605 |
| TBX3 | 0.21465508 | -4.023241494 | 1.094109729 | 0.274490317 |
| CCT8P1 | -0.054688422 | 6.702186603 | -1.093243464 | 0.274869967 |
| COPRS | 0.054854471 | 4.662458009 | 1.093105154 | 0.274930616 |
| USP6 | -0.199130267 | -1.530916733 | -1.09309105 | 0.274936801 |
| GRB2 | -0.050329155 | 7.490816766 | -1.092494373 | 0.275198554 |
| PAFAH1B3 | 0.073765612 | 5.250730554 | 1.092079572 | 0.275380621 |
| CHCHD3 | 0.054154441 | 7.872730238 | 1.091192919 | 0.275770073 |
| IGLV8-61 | 0.225950808 | 1.282600753 | 1.09115876 | 0.275785084 |
| EMR4P | -0.207216238 | 2.322561085 | -1.091036085 | 0.275839 |
| RP11-333I13.1 | 0.115727624 | 0.286548679 | 1.091024765 | 0.275843975 |
| BAIAP2 | -0.113729813 | 2.428344866 | -1.090846936 | 0.275922144 |
| METTL1 | 0.079949572 | 3.588878657 | 1.090753513 | 0.275963217 |
| ACKR3 | 0.184192658 | 2.817158634 | 1.090398816 | 0.276119193 |
| MTA3 | 0.121622899 | 2.239226075 | 1.090218032 | 0.276198715 |
| RP11-51L5.5 | 0.109027363 | 1.009253036 | 1.090163074 | 0.276222893 |
| CTNNBL1 | 0.051169583 | 6.046238646 | 1.089895439 | 0.276340655 |
| ANXA11 | 0.042330741 | 6.954397645 | 1.089677388 | 0.276436625 |
| FLNB | 0.133585524 | 5.768743158 | 1.089403681 | 0.276557122 |
| UQCRBP1 | -0.130680751 | 7.059082411 | -1.089108904 | 0.276686936 |
| IMPAD1 | 0.04373789 | 5.93228043 | 1.089066928 | 0.276705425 |
| SPON2 | -0.123559673 | 3.676478236 | -1.088948548 | 0.276757571 |
| RANGAP1 | -0.051112484 | 5.891850462 | -1.088681413 | 0.276875268 |
| TMSB4X | 0.060597021 | 10.55553213 | 1.088629726 | 0.276898045 |
| ST5 | 0.164593239 | -1.523904883 | 1.088629445 | 0.276898169 |
| CTD-3018O17.3 | -0.151098897 | 1.18325708 | -1.08852077 | 0.276946062 |
| SMARCD3 | -0.180848164 | 3.538781657 | -1.088339886 | 0.277025792 |
| IGHV2-5 | 0.230458905 | 2.338804055 | 1.088338355 | 0.277026467 |
| XPR1 | -0.042028829 | 4.508303879 | -1.087820577 | 0.277254778 |
| AP000476.1 | -0.186574073 | 0.460567551 | -1.087590144 | 0.277356427 |
| SLC44A5 | 0.274807549 | -3.046484343 | 1.087454537 | 0.277416258 |
| AP001063.1 | -0.148700888 | -0.135920082 | -1.087446344 | 0.277419873 |
| RP11-213H15.1 | 0.150870637 | -0.884447999 | 1.08697489 | 0.277627954 |
| RP5-1198O20.4 | -0.166338966 | -1.200175791 | -1.086949361 | 0.277639225 |
| RNF144A-AS1 | -0.210128953 | -1.415365715 | -1.086651012 | 0.277770963 |
| RP11-404O13.5 | -0.218925172 | -1.821403647 | -1.086501376 | 0.277837052 |
| HNRNPA1P28 | 0.112976385 | -0.173166149 | 1.086139425 | 0.277996957 |
| CNGA1 | 0.167970675 | -2.757806937 | 1.085792778 | 0.27815016 |
| LIMCH1 | 0.216802533 | -3.17251916 | 1.08565493 | 0.278211099 |
| TUBGCP3 | 0.029345069 | 5.686992716 | 1.084828395 | 0.27857668 |
| RPL29P3 | 0.114582805 | 0.600385029 | 1.08437667 | 0.278776619 |
| CD163 | -0.293621123 | 4.175406274 | -1.084076752 | 0.27890942 |
| CLEC18A | -0.137951662 | -2.349788214 | -1.084058427 | 0.278917535 |
| RP11-89C3.4 | -0.185001373 | -1.128177853 | -1.084000793 | 0.278943061 |
| LTB | -0.148733477 | 6.084650253 | -1.083826634 | 0.279020203 |
| RP4-794H19.4 | -0.141432751 | 0.04859083 | -1.083806876 | 0.279028956 |
| GRAMD2 | -0.173498153 | -3.378812243 | -1.083754726 | 0.279052059 |
| TF | 0.16963835 | -0.848881707 | 1.083692366 | 0.279079686 |
| LINC00487 | 0.171059907 | -1.599709967 | 1.083184472 | 0.27930477 |
| FAM47E-STBD1 | -0.126000354 | 0.45007475 | -1.083074194 | 0.279353658 |
| FAM129C | -0.160320666 | 1.799186295 | -1.082917459 | 0.279423151 |
| FAM63B | 0.047013075 | 3.730048954 | 1.082707808 | 0.279516125 |
| LYNX1 | -0.183561066 | -0.567174341 | -1.082473422 | 0.279620094 |
| PHF7 | 0.060817421 | 4.67413138 | 1.082131781 | 0.279771685 |
| RNU6-878P | 0.125090721 | 1.680746073 | 1.082083966 | 0.279792906 |
| KCNK12 | 0.175187303 | -3.084428104 | 1.081453262 | 0.28007292 |
| NDFIP1 | -0.104056248 | 6.296347064 | -1.081088606 | 0.280234905 |
| FAM210A | 0.049991147 | 3.773607429 | 1.0804715 | 0.280509175 |
| GUCY1B2 | -0.190227477 | -2.82324193 | -1.080158513 | 0.280648351 |
| PRR15 | -0.234078062 | -0.464306989 | -1.079889889 | 0.280767838 |
| CHRNA5 | -0.135047565 | -0.312715596 | -1.079823464 | 0.280797389 |
| ANKRD42 | 0.064873074 | 3.098515862 | 1.07960294 | 0.280895513 |
| TTTY14 | 0.329859816 | -0.53346675 | 1.079561087 | 0.280914139 |
| GPR89C | -0.100220808 | -0.686734592 | -1.079535014 | 0.280925742 |
| RP3-340B19.2 | -0.051883364 | 7.029029538 | -1.07920557 | 0.281072386 |
| TONSL | -0.094936385 | 4.210896326 | -1.079199606 | 0.281075041 |
| NME1-NME2 | -0.10886862 | 1.092359894 | -1.079089261 | 0.28112417 |
| RP11-635N19.2 | 0.123223759 | 0.179400003 | 1.078918779 | 0.281200086 |
| RPL3 | 0.061720418 | 10.41010288 | 1.078622358 | 0.281332115 |
| CTD-3064H18.1 | -0.185737787 | 0.18953825 | -1.077662678 | 0.281759857 |
| CYP20A1 | 0.039358776 | 4.228149106 | 1.077634763 | 0.281772306 |
| RP11-220I1.1 | -0.046469939 | 6.127219479 | -1.077402725 | 0.281875798 |
| LRRFIP1 | -0.044714823 | 7.514116803 | -1.077050538 | 0.282032927 |
| TPM2 | 0.135762073 | 4.831116169 | 1.076994675 | 0.282057856 |
| ITFG3 | -0.055008394 | 4.548853145 | -1.076641139 | 0.282215656 |
| HEBP1 | -0.061804063 | 5.467370109 | -1.07650666 | 0.282275697 |
| IGLV5-45 | 0.182056534 | 0.333628132 | 1.076498287 | 0.282279436 |
| LILRP1 | -0.204747408 | 1.445499884 | -1.076214162 | 0.282406318 |
| GAS5 | -0.078218639 | 6.931131098 | -1.076022624 | 0.282491875 |
| AMHR2 | 0.173189368 | -2.025014436 | 1.075836807 | 0.282574893 |
| ZNF888 | 0.080946546 | 3.616247875 | 1.07566308 | 0.282652525 |
| BMP1 | -0.086823313 | 4.166731689 | -1.075401461 | 0.282769461 |
| FCF1P1 | -0.103687104 | 0.799781297 | -1.074988112 | 0.282954281 |
| CLIP4 | -0.135364204 | 3.028021973 | -1.074972438 | 0.282961291 |
| ZP3 | -0.098614675 | 2.786257209 | -1.074964792 | 0.282964711 |
| QSOX2 | 0.063211967 | 5.358345401 | 1.074556061 | 0.283147552 |
| ZNF670 | 0.093699127 | 0.279886896 | 1.07445195 | 0.283194137 |
| GHR | 0.155487439 | -3.757570379 | 1.074440222 | 0.283199386 |
| LNX2 | 0.06197824 | 4.006005651 | 1.074410682 | 0.283212604 |
| NEIL2 | -0.075779982 | 3.238861583 | -1.074328743 | 0.283249274 |
| RNU6-1002P | 0.140907758 | 0.924468131 | 1.074036033 | 0.283380296 |
| NIPSNAP3B | -0.09241474 | 3.185553253 | -1.073751073 | 0.283507888 |
| RP11-1060J15.4 | -0.183652834 | -2.229250554 | -1.073663639 | 0.283547045 |
| ATP6AP1 | -0.043010264 | 7.355436269 | -1.073593027 | 0.283578671 |
| MAGED1 | -0.11003554 | 5.50104988 | -1.073135701 | 0.283783557 |
| RBM47 | 0.214247932 | 3.674266581 | 1.072873713 | 0.283900976 |
| CTA-445C9.15 | -0.082591415 | 5.04836182 | -1.072156456 | 0.284222606 |
| E2F4 | 0.03733188 | 6.834498497 | 1.071777609 | 0.284392588 |
| APOLD1 | -0.109395568 | 1.28381106 | -1.071750026 | 0.284404967 |
| BYSL | -0.077520797 | 4.28223683 | -1.071545003 | 0.284496988 |
| TAP2 | 0.06571759 | 5.435999517 | 1.07143675 | 0.284545584 |
| NDUFA1 | -0.045363707 | 7.75756842 | -1.071360754 | 0.284579702 |
| ACVR1B | 0.106743291 | 4.454485653 | 1.071317384 | 0.284599175 |
| RP11-692N5.2 | -0.257841533 | -0.506173675 | -1.071173485 | 0.28466379 |
| PLA2R1 | -0.212003995 | -3.01215108 | -1.070957543 | 0.284760772 |
| SHROOM4 | -0.281838656 | -0.143562425 | -1.070889561 | 0.284791309 |
| TYW3 | 0.042506592 | 5.011073855 | 1.07044083 | 0.284992927 |
| RP11-392P7.1 | 0.090188659 | 2.542870892 | 1.068120748 | 0.286036901 |
| CPD | -0.087875523 | 6.485972341 | -1.067744116 | 0.286206619 |
| AC105399.2 | 0.067979357 | 4.210808354 | 1.067614372 | 0.2862651 |
| SLC12A2 | -0.081251131 | 3.633809936 | -1.067389551 | 0.286366456 |
| CACHD1 | -0.217642509 | 0.342877971 | -1.067142367 | 0.286477922 |
| CASP7 | 0.057294654 | 4.968322458 | 1.066859734 | 0.286605409 |
| ZNF826P | 0.195652927 | -1.725144463 | 1.066742656 | 0.286658231 |
| TTC19 | 0.046743144 | 5.595754157 | 1.066500528 | 0.286767492 |
| SH3RF3 | 0.166034125 | 2.265032526 | 1.066414199 | 0.286806455 |
| SAMD10 | -0.085369582 | 4.364165755 | -1.066413876 | 0.2868066 |
| FCGRT | 0.160501992 | 6.254357158 | 1.066413709 | 0.286806676 |
| AF001548.5 | 0.086787737 | 4.441816527 | 1.066182657 | 0.286910974 |
| RP11-496I2.5 | 0.105634096 | -0.060946454 | 1.066129794 | 0.286934841 |
| RP11-603J24.17 | -0.14253705 | 0.237247146 | -1.065728276 | 0.287116161 |
| PLEKHA2 | 0.046279005 | 6.91167971 | 1.065423284 | 0.287253942 |
| LTBP3 | 0.120164087 | 4.705615786 | 1.065296648 | 0.287311164 |
| TPT1P12 | -0.120060754 | 0.152098121 | -1.064940917 | 0.287471946 |
| KIAA1147 | -0.097362362 | 5.985446052 | -1.064624223 | 0.287615135 |
| YPEL3 | -0.075146876 | 6.713962881 | -1.064503763 | 0.287669612 |
| HIST2H2AA4 | -0.165564681 | 2.583067609 | -1.064335496 | 0.287745721 |
| IP6K1 | 0.056744735 | 5.474250423 | 1.063945716 | 0.287922075 |
| MEX3A | -0.219122421 | 0.491822338 | -1.063530235 | 0.288110139 |
| HEBP2 | -0.062570283 | 5.358309556 | -1.062714398 | 0.288479661 |
| RP13-395E19.3 | -0.129016883 | -0.816780737 | -1.062699966 | 0.2884862 |
| RPL13AP5 | -0.038486797 | 12.17779651 | -1.06207537 | 0.288769323 |
| ZNF80 | 0.149585221 | -1.872745201 | 1.061955086 | 0.288823869 |
| RN7SL558P | 0.123808509 | 0.57865847 | 1.061937946 | 0.288831642 |
| RP11-295P22.2 | 0.144065602 | -0.606157192 | 1.061654148 | 0.288960365 |
| MTL5 | -0.088224424 | 4.159621375 | -1.061654032 | 0.288960418 |
| TRIM47 | -0.1552087 | 2.230342709 | -1.061450882 | 0.289052586 |
| IGSF3 | 0.199085598 | -1.455918577 | 1.061220117 | 0.289157306 |
| DPPA4 | 0.363618789 | 0.448188988 | 1.061212071 | 0.289160958 |
| YIPF2 | -0.044516437 | 6.37796979 | -1.061174979 | 0.289177792 |
| KLHL35 | -0.13533987 | -0.169268007 | -1.060479665 | 0.289493494 |
| RPL36AP15 | -0.103467701 | 1.281452453 | -1.059885312 | 0.289763539 |
| RP13-578N3.3 | 0.127068468 | -0.952142649 | 1.059824384 | 0.289791232 |
| RP11-473M20.7 | -0.090886016 | 3.725580167 | -1.058792608 | 0.290260455 |
| FABP5P7 | -0.11445191 | 6.80221475 | -1.058405363 | 0.290436696 |
| NPIPB15 | 0.171167216 | 2.443782983 | 1.058309473 | 0.290480348 |
| IGLV3-9 | 0.180558541 | 0.888789854 | 1.058118619 | 0.290567243 |
| LINC01003 | -0.064295631 | 4.04493732 | -1.057895178 | 0.290668998 |
| LRRK1 | -0.092640615 | 4.85674023 | -1.057855867 | 0.290686903 |
| PIK3R4 | 0.045428715 | 4.721576021 | 1.057631336 | 0.290789183 |
| IGHV3-7 | 0.229602156 | 3.545769443 | 1.057605077 | 0.290801146 |
| TTC9 | 0.164910337 | 2.872027629 | 1.057176397 | 0.290996494 |
| RP11-809N8.5 | -0.124306282 | 1.338885775 | -1.057103251 | 0.291029836 |
| FAM127C | -0.236607906 | 0.202375892 | -1.056880935 | 0.291131187 |
| RP1-130G2.1 | 0.077608781 | 1.604749142 | 1.056861844 | 0.291139891 |
| C7orf60 | 0.05381788 | 4.37639805 | 1.056833939 | 0.291152615 |
| C4orf32 | -0.130644004 | 2.826947695 | -1.05681611 | 0.291160744 |
| RN7SKP26 | 0.123331935 | 1.156736545 | 1.056226743 | 0.291429563 |
| ATP13A4 | -0.145033472 | -1.194663 | -1.056062989 | 0.291504284 |
| EEF1A1P5 | 0.03925314 | 12.46364409 | 1.056052365 | 0.291509132 |
| PCDHGB2 | 0.241273334 | 0.478986425 | 1.055224535 | 0.29188707 |
| AC104135.4 | -0.143272906 | -1.343769016 | -1.055161118 | 0.291916036 |
| VWF | 0.230560584 | 2.065698307 | 1.054705645 | 0.292124133 |
| SETP21 | -0.102308433 | -0.178190716 | -1.05466499 | 0.292142712 |
| ATP6V0A1 | 0.087488552 | 4.622829839 | 1.054501218 | 0.292217564 |
| C5orf66 | 0.144451089 | -1.475673467 | 1.054419562 | 0.29225489 |
| TNFRSF10D | 0.090972727 | 5.087618223 | 1.054047337 | 0.292425077 |
| IL12A | -0.134496291 | 3.199361303 | -1.0536894 | 0.292588796 |
| VWA1 | -0.210357304 | 0.676310105 | -1.053511948 | 0.292669984 |
| COTL1 | 0.126585084 | 8.145151006 | 1.053418815 | 0.2927126 |
| BHLHE41 | 0.208696804 | 0.781796088 | 1.052972123 | 0.292917059 |
| LIG3 | -0.05412752 | 4.640754169 | -1.052863403 | 0.292966837 |
| TRBVB | -0.300747159 | 3.62113315 | -1.052630255 | 0.293073603 |
| HLCS | -0.068531927 | 3.393364457 | -1.052499279 | 0.293133593 |
| KIR2DL2 | 0.231143133 | -2.617264529 | 1.052444779 | 0.293158558 |
| NLE1 | -0.066872393 | 3.618591744 | -1.052442588 | 0.293159561 |
| RASGRP3 | -0.176160182 | 3.418349183 | -1.052306439 | 0.293221933 |
| CTB-31N19.3 | -0.134681839 | 4.07306483 | -1.052068453 | 0.293330979 |
| AC096559.1 | -0.148575707 | -3.032184028 | -1.051978416 | 0.293372242 |
| RNF207 | -0.137901353 | -0.280798062 | -1.051746224 | 0.293478669 |
| RP11-397E7.4 | 0.069065626 | 2.236522912 | 1.051676424 | 0.293510668 |
| FIBCD1 | -0.17914632 | -2.927500064 | -1.051600203 | 0.293545613 |
| SLC25A6P2 | 0.123035833 | 0.332216156 | 1.051451434 | 0.293613827 |
| ST13P19 | -0.064940085 | 6.398445679 | -1.051133561 | 0.293759614 |
| ADO | -0.044486209 | 5.135626886 | -1.050990282 | 0.293825343 |
| WBP2 | 0.047194164 | 6.420966533 | 1.050848274 | 0.293890499 |
| CHRNA7 | 0.149463032 | 0.336692733 | 1.050728487 | 0.293945466 |
| GTF2IRD1 | 0.188094475 | -0.442943454 | 1.05050777 | 0.294046767 |
| ZNF799 | -0.079102758 | 1.710592296 | -1.050387204 | 0.294102111 |
| AL353671.3 | 0.100650924 | -0.070522085 | 1.050368313 | 0.294110784 |
| RNU4-82P | -0.114860356 | 0.247707024 | -1.049992725 | 0.294283243 |
| RP13-582O9.5 | -0.067302621 | 3.542110726 | -1.049694592 | 0.294420185 |
| CA2 | 0.205572887 | 5.719909249 | 1.049549824 | 0.294486698 |
| FAM122A | -0.044904487 | 4.817957989 | -1.049472904 | 0.294522042 |
| WDR83OS | 0.030963031 | 6.304782153 | 1.049356074 | 0.29457573 |
| CISD2 | 0.04501539 | 5.188985421 | 1.048689969 | 0.294881958 |
| ARHGEF26 | 0.183492405 | -2.424840613 | 1.048661032 | 0.294895266 |
| TMTC1 | -0.27821956 | -2.522293523 | -1.04806346 | 0.295170178 |
| LAMC3 | -0.180583247 | -1.569105017 | -1.047792982 | 0.295294667 |
| RP11-309G3.3 | -0.11864908 | 1.203589454 | -1.047602203 | 0.295382495 |
| OIP5 | 0.127998286 | 2.922335184 | 1.047534992 | 0.295413442 |
| BCAT1 | -0.187610205 | 4.679273325 | -1.047507141 | 0.295426266 |
| NDUFA10 | 0.034364024 | 5.517066433 | 1.0473032 | 0.295520183 |
| TMEM126A | -0.048088892 | 5.046260061 | -1.047234032 | 0.295552039 |
| RHPN1 | -0.149115952 | 5.535578005 | -1.046715329 | 0.295791015 |
| RFPL4A | -0.255307939 | -0.961846161 | -1.046647849 | 0.295822113 |
| COLGALT2 | 0.18344636 | -0.015884051 | 1.046023554 | 0.296109929 |
| CD151 | -0.09793885 | 5.532721367 | -1.045977916 | 0.296130977 |
| ACPL2 | 0.099631987 | 1.876633165 | 1.045885323 | 0.296173683 |
| RP11-587D21.1 | -0.056578427 | 5.592706654 | -1.045699308 | 0.296259489 |
| SLC25A38P1 | 0.062781879 | 1.826907169 | 1.045424019 | 0.296386507 |
| GDAP2 | -0.041882208 | 3.805554932 | -1.045278875 | 0.296453491 |
| SLC9A1 | 0.057363602 | 4.896823303 | 1.045088588 | 0.296541323 |
| RN7SL23P | -0.108952616 | -0.060928548 | -1.0448682 | 0.296643072 |
| SPARC | 0.200806752 | 5.557110714 | 1.044838282 | 0.296656887 |
| CEP135 | -0.036611186 | 4.420604053 | -1.044177948 | 0.296961899 |
| FAM217B | 0.058037257 | 4.679794854 | 1.044051115 | 0.297020508 |
| ESPNP | -0.183860116 | -2.023220855 | -1.044024784 | 0.297032677 |
| ZDHHC11 | 0.126120725 | -0.04457788 | 1.043835411 | 0.297120202 |
| RP11-617F23.1 | -0.080527952 | 4.336424661 | -1.043156541 | 0.297434108 |
| KCNK10 | 0.223606053 | -3.300004174 | 1.04302815 | 0.2974935 |
| SLC28A3 | -0.24408519 | 1.103765782 | -1.042759483 | 0.297617809 |
| KLHL4 | -0.189202558 | -3.826874934 | -1.042669673 | 0.29765937 |
| ESYT3 | -0.162095531 | -3.498390704 | -1.04224142 | 0.297857607 |
| CD86 | -0.214155591 | 4.692765495 | -1.041904594 | 0.298013585 |
| KNSTRN | 0.07357361 | 3.618432742 | 1.041833543 | 0.298046494 |
| SRP19 | 0.05523221 | 3.32542811 | 1.041635756 | 0.298138118 |
| C4orf33 | 0.055398137 | 3.522822717 | 1.041268307 | 0.298308387 |
| PIAS3 | 0.046975408 | 4.768618118 | 1.040812372 | 0.298519749 |
| CTB-114C7.4 | -0.146738297 | -1.511043406 | -1.040510501 | 0.298659746 |
| RP11-274B21.4 | 0.06185892 | 6.92436367 | 1.040406991 | 0.29870776 |
| VKORC1 | -0.053473173 | 3.934942633 | -1.040278575 | 0.298767334 |
| TENC1 | 0.133966418 | 0.339655468 | 1.039980924 | 0.29890545 |
| AP001092.4 | -0.181342262 | 0.052247546 | -1.039860365 | 0.298961404 |
| COL8A2 | -0.240024951 | 0.507562943 | -1.039620481 | 0.29907276 |
| ANKRD22 | -0.202532533 | 2.696542701 | -1.039553501 | 0.299103857 |
| ZMYM6 | -0.041609943 | 3.809681926 | -1.039290432 | 0.299226016 |
| CD1D | -0.257246535 | 4.394889037 | -1.039216503 | 0.299260352 |
| RNU1-59P | -0.148943382 | 0.529120659 | -1.039210097 | 0.299263327 |
| AMY2A | -0.119669465 | -1.080810485 | -1.039166012 | 0.299283803 |
| SLC25A5 | -0.048548424 | 9.634496123 | -1.038859687 | 0.299426109 |
| VPS13A | 0.07742724 | 5.089731383 | 1.038390986 | 0.299643935 |
| TRGJ1 | 0.144457919 | 0.345724057 | 1.038298822 | 0.299686781 |
| DBNDD2 | -0.114563943 | -0.744235891 | -1.03815417 | 0.299754035 |
| C3orf14 | -0.197321458 | -2.310555557 | -1.037954318 | 0.29984697 |
| RPL17P50 | 0.058028862 | 4.618950922 | 1.03738633 | 0.300111201 |
| FAM134A | 0.035632944 | 6.723745816 | 1.037243998 | 0.300177439 |
| PRSS57 | -0.196023156 | 8.795012024 | -1.036902925 | 0.300336207 |
| AC097500.2 | 0.10836533 | 0.516426793 | 1.036822619 | 0.300373597 |
| PKMP5 | 0.116112872 | -0.637827025 | 1.036695484 | 0.300432797 |
| PLEK | 0.074955428 | 9.55599282 | 1.036656678 | 0.300450868 |
| LEPROTL1 | 0.052963881 | 5.772801881 | 1.036303808 | 0.300615228 |
| RP11-553A21.3 | 0.149697516 | -1.391495845 | 1.036244466 | 0.300642875 |
| FBXW11P1 | -0.081665746 | 0.459198749 | -1.036122148 | 0.300699865 |
| VAMP7 | 0.029390792 | 7.178904392 | 1.036022123 | 0.300746475 |
| CTBP1-AS2 | 0.046903284 | 3.694643433 | 1.035980197 | 0.300766013 |
| LILRA5 | 0.241470634 | 5.376742475 | 1.035096905 | 0.301177834 |
| PDXDC1 | 0.030152755 | 5.698155703 | 1.034538948 | 0.301438167 |
| SLC38A1 | 0.137326959 | 6.467206823 | 1.034116772 | 0.301635247 |
| RP11-96C23.9 | 0.114715548 | 0.142343647 | 1.034008099 | 0.301685992 |
| RPS4X | 0.052343882 | 11.32775693 | 1.033906193 | 0.301733582 |
| IGHD | 0.187034662 | 4.719969549 | 1.033229408 | 0.302049767 |
| MIR4426 | -0.065575876 | 5.295201153 | -1.032949156 | 0.302180761 |
| PIK3R2 | 0.104237244 | -2.484020486 | 1.032088393 | 0.302583334 |
| RLTPR | -0.115473137 | 4.767406424 | -1.032058769 | 0.302597195 |
| AC009133.14 | 0.112013697 | -0.132252523 | 1.032053472 | 0.302599674 |
| METTL17 | -0.034849591 | 5.727337982 | -1.031565219 | 0.302828193 |
| RP11-49C24.1 | 0.075229682 | 2.222370463 | 1.031529768 | 0.30284479 |
| PLA2G2D | 0.183309907 | -1.830518336 | 1.031368364 | 0.302920361 |
| HNRNPA0 | 0.052949858 | 8.756237248 | 1.031353279 | 0.302927425 |
| CTD-2047H16.4 | 0.128338822 | -1.33729389 | 1.031269227 | 0.302966784 |
| PELI3 | -0.125520578 | 2.141046101 | -1.030809547 | 0.303182101 |
| RPL34P33 | 0.077161554 | 2.196098322 | 1.030795147 | 0.303188848 |
| THBS3 | -0.069567057 | 5.089744077 | -1.030756637 | 0.303206891 |
| TP73 | 0.191368558 | 0.044741423 | 1.030686314 | 0.303239842 |
| RP11-563N6.6 | -0.098478705 | 1.488738448 | -1.030643807 | 0.30325976 |
| EFCC1 | 0.246042794 | 1.780494654 | 1.030629331 | 0.303266544 |
| AIDA | -0.052093553 | 3.21683768 | -1.030587301 | 0.30328624 |
| RP11-388M20.9 | -0.186704449 | -0.882112536 | -1.03051267 | 0.303321215 |
| LILRA2 | -0.145776986 | 6.825017501 | -1.030359496 | 0.303393007 |
| PGGT1BP2 | 0.078155192 | 1.299499157 | 1.03007749 | 0.303525213 |
| AC108488.4 | -0.14993457 | 1.570077951 | -1.029382267 | 0.3038513 |
| ZNF354C | 0.138811301 | 1.767370778 | 1.028609308 | 0.304214122 |
| RP11-229M1.2 | 0.081384596 | 1.981912475 | 1.028159904 | 0.304425202 |
| TSHR | -0.139978606 | -3.152785296 | -1.028143362 | 0.304432974 |
| RP11-495K9.6 | 0.160224698 | -1.968992729 | 1.027827885 | 0.304581211 |
| FAM198A | -0.150919986 | -2.647204562 | -1.027780654 | 0.304603408 |
| AC022498.1 | 0.132001936 | -0.495893851 | 1.027443467 | 0.304761906 |
| SRPK2 | -0.055227311 | 4.746910832 | -1.027235983 | 0.304859464 |
| DENND1A | 0.064983833 | 5.066465847 | 1.027234195 | 0.304860305 |
| FXYD3 | 0.169908195 | -2.599141429 | 1.027167147 | 0.304891835 |
| EIF2AK4 | -0.045737758 | 5.680144875 | -1.027058384 | 0.304942987 |
| RP5-1043L13.1 | -0.127488701 | 1.135001696 | -1.027047006 | 0.304948338 |
| RP11-455G16.1 | -0.128266893 | -1.979040992 | -1.026987628 | 0.304976267 |
| TMEM130 | 0.152534873 | -2.656411045 | 1.026725475 | 0.305099591 |
| ZNF160 | 0.051222567 | 4.528471762 | 1.026658414 | 0.305131143 |
| GNG5 | -0.043397516 | 8.349175542 | -1.0266496 | 0.30513529 |
| PKIA | 0.178516327 | 1.383793598 | 1.026624891 | 0.305146917 |
| PML | 0.047099818 | 5.550056121 | 1.026441866 | 0.305233045 |
| CCNI | 0.033453151 | 9.915636502 | 1.026289331 | 0.305304838 |
| ZNF519 | -0.063690761 | 1.661743945 | -1.026248307 | 0.305324148 |
| PKD1P1 | 0.095754574 | -0.853082429 | 1.026212648 | 0.305340934 |
| PPAPDC1B | 0.055945762 | 4.963916346 | 1.025980425 | 0.305450263 |
| RP3-322G13.7 | 0.124755472 | -0.759021544 | 1.02558912 | 0.305634547 |
| SDR39U1 | -0.042658769 | 5.985556083 | -1.024894241 | 0.305961979 |
| UBE2HP1 | 0.108654715 | 1.551857863 | 1.02486252 | 0.305976931 |
| TMEM14D | 0.072217723 | 3.93800272 | 1.024811844 | 0.30600082 |
| MTDH | -0.039575745 | 7.818448309 | -1.024553636 | 0.306122559 |
| NPTX2 | -0.341591655 | 0.249455873 | -1.023586359 | 0.306578895 |
| PSTPIP1 | -0.115938673 | 5.736053071 | -1.023354186 | 0.306688495 |
| OGFRL1 | 0.107864365 | 6.197305052 | 1.023335474 | 0.306697329 |
| GOLGA6L9 | 0.135327507 | -1.80325799 | 1.023272247 | 0.306727181 |
| ZNF148 | -0.031665312 | 4.897577979 | -1.022636246 | 0.307027572 |
| MTMR9LP | -0.179454591 | 0.301378072 | -1.022385476 | 0.307146068 |
| RPL35AP21 | 0.102335704 | 0.529407892 | 1.021484157 | 0.307572216 |
| WDR63 | -0.138185626 | -2.947326828 | -1.020985138 | 0.307808323 |
| DCLK2 | 0.221827353 | -0.706548688 | 1.020894227 | 0.30785135 |
| PIPOX | -0.120856345 | 0.661496773 | -1.020889277 | 0.307853693 |
| GSTT1 | 0.255560153 | -0.387280738 | 1.020703086 | 0.307941827 |
| CTNND1 | -0.082933604 | 5.144030363 | -1.02069729 | 0.307944571 |
| ZNF502 | 0.179361033 | 1.213687167 | 1.020675019 | 0.307955115 |
| KAL1 | -0.178478288 | -4.280994602 | -1.020534154 | 0.308021807 |
| DMGDH | -0.141288134 | -2.179237989 | -1.020200513 | 0.308179806 |
| TRPS1 | 0.174090349 | 3.499174916 | 1.020175183 | 0.308191804 |
| COX16 | -0.040297613 | 4.365018262 | -1.020129933 | 0.308213237 |
| RP11-889L3.4 | -0.125162098 | 0.488123574 | -1.019985554 | 0.308281631 |
| RP11-3J10.7 | -0.068648678 | 3.501499227 | -1.019381163 | 0.308568047 |
| TRBV6-5 | -0.166167035 | 1.994083513 | -1.019253862 | 0.308628397 |
| IGKV2-28 | 0.170584444 | 0.671040336 | 1.018347551 | 0.309058278 |
| GSTM2 | 0.106889184 | 4.082533662 | 1.018098914 | 0.30917628 |
| FAM49B | 0.054032698 | 6.656831857 | 1.01741843 | 0.309499389 |
| INSC | -0.138624334 | -2.374425588 | -1.01715511 | 0.30962448 |
| NACA | 0.054378657 | 8.163633591 | 1.016869382 | 0.309760253 |
| COL26A1 | -0.154859253 | -2.965736395 | -1.016496763 | 0.309937374 |
| UPF3AP2 | 0.078814677 | 3.552369912 | 1.016340076 | 0.310011874 |
| PATZ1 | -0.055025588 | 5.652598731 | -1.01611083 | 0.310120895 |
| HLA-DQA2 | -0.249864489 | 2.565238497 | -1.015312522 | 0.310500739 |
| SPATA31C2 | -0.149504974 | -3.265060471 | -1.015278309 | 0.310517025 |
| XXbac-BPG299F13.17 | -0.075652061 | 4.187848744 | -1.015168258 | 0.310569415 |
| NAA40 | -0.051516703 | 4.993011437 | -1.015062552 | 0.310619742 |
| MT-TV | 0.096539951 | 5.370005132 | 1.014885186 | 0.310704198 |
| HOXB5 | -0.365937839 | 1.969405892 | -1.014680685 | 0.310801594 |
| SLC9A7 | 0.117686056 | 4.722669842 | 1.014479036 | 0.310897651 |
| RP11-39K24.12 | 0.133407518 | 1.407824175 | 1.014182506 | 0.311038941 |
| RC3H1-IT1 | 0.122352145 | 0.088897099 | 1.014063626 | 0.311095597 |
| ZNF473 | -0.062819598 | 2.893491431 | -1.014033775 | 0.311109824 |
| SMCR2 | -0.146109581 | 0.547542806 | -1.013739524 | 0.311250092 |
| CD82 | 0.088637051 | 7.528487506 | 1.013256927 | 0.311480233 |
| ANKRD28 | -0.097354304 | 7.029732437 | -1.012153029 | 0.312007085 |
| RAB6C | -0.068896534 | 1.099706161 | -1.012145412 | 0.312010722 |
| ICA1 | 0.162466769 | 4.075498958 | 1.01205959 | 0.312051707 |
| NOP14 | -0.046703929 | 6.526128558 | -1.012006546 | 0.31207704 |
| IGLC2 | 0.244636364 | 5.471643175 | 1.012001201 | 0.312079593 |
| ASL | 0.068059556 | 4.229175838 | 1.011454906 | 0.312340579 |
| RP11-263K19.4 | -0.118982043 | -1.257881363 | -1.010983182 | 0.312566054 |
| MTHFD1 | -0.075027183 | 5.794294023 | -1.010756069 | 0.312674649 |
| STRA13 | -0.059439412 | 5.776238638 | -1.010738607 | 0.312683 |
| CASKIN2 | -0.144363828 | 1.139230013 | -1.0103298 | 0.312878538 |
| OCLN | -0.209329517 | 0.854984333 | -1.010095661 | 0.312990566 |
| SIRPB3P | -0.16814691 | -0.561091805 | -1.010065864 | 0.313004825 |
| AC079807.4 | 0.135254486 | -1.73697089 | 1.010042527 | 0.313015993 |
| CEBPZ-AS1 | -0.039116697 | 5.538535818 | -1.009622783 | 0.313216904 |
| LYPLA2 | 0.029617334 | 5.964827439 | 1.009279791 | 0.313381141 |
| NEURL1 | -0.175322299 | 0.769287029 | -1.008886089 | 0.313569731 |
| TIRAP | -0.067720044 | 3.7198313 | -1.008790055 | 0.313615744 |
| S100A12 | 0.263639005 | 8.051730767 | 1.008780005 | 0.31362056 |
| RP4-610C12.4 | -0.192193651 | -1.805470728 | -1.008485777 | 0.313761563 |
| ABCC3 | -0.215617484 | 0.598344659 | -1.008063423 | 0.313964043 |
| MICALL2 | -0.142222817 | 3.939433025 | -1.008049109 | 0.313970906 |
| RP11-873E20.1 | 0.10317781 | 1.330750738 | 1.007605311 | 0.314183762 |
| HPGD | 0.233614451 | 2.082711937 | 1.007266991 | 0.314346091 |
| TRABD2A | 0.142223767 | 1.400648338 | 1.007250704 | 0.314353907 |
| HNRNPD | -0.037585297 | 8.15577951 | -1.007234645 | 0.314361614 |
| CES1P2 | -0.156393159 | -2.053705993 | -1.007045209 | 0.314452535 |
| RWDD4P2 | -0.043010558 | 5.495720959 | -1.007025934 | 0.314461787 |
| RP11-685N3.1 | 0.135083941 | -0.712284557 | 1.006909977 | 0.314517451 |
| RSAD2 | 0.137383639 | 3.306583293 | 1.006888416 | 0.314527801 |
| GPR107 | 0.030559142 | 5.570940271 | 1.00682262 | 0.31455939 |
| CRIP1 | -0.098801327 | 3.55209448 | -1.006533204 | 0.314698361 |
| ZFAND2B | 0.04013661 | 5.521863297 | 1.00640775 | 0.314758614 |
| AC006486.1 | -0.099516452 | -0.3297154 | -1.006359046 | 0.314782007 |
| RP11-382A20.4 | 0.124030946 | -1.536254819 | 1.006065563 | 0.314922997 |
| BCL2L10 | -0.194032116 | -0.011066547 | -1.00571277 | 0.315092535 |
| RPGRIP1 | -0.210485539 | -0.590728013 | -1.00487289 | 0.315496389 |
| ZC3H18 | -0.037515087 | 5.463767996 | -1.004705164 | 0.315577081 |
| RP11-848P1.2 | -0.09249168 | 2.58967463 | -1.004577198 | 0.315638653 |
| PPBP | 0.280785656 | 4.420419182 | 1.004293164 | 0.315775348 |
| MAPK15 | -0.212356752 | -1.075062641 | -1.003853234 | 0.315987146 |
| ADARB2 | 0.229408967 | -0.719379784 | 1.003133981 | 0.316333623 |
| RP11-803P9.1 | 0.104668256 | 0.100234619 | 1.002162487 | 0.316802005 |
| NUP205 | -0.048649828 | 5.499392517 | -1.00203617 | 0.316862939 |
| RP11-212I21.4 | 0.120750377 | 2.712533618 | 1.001824074 | 0.31696527 |
| C1orf106 | 0.214823439 | -0.623531132 | 1.000937155 | 0.31739342 |
| RP11-6B6.3 | -0.077770299 | 2.891203981 | -1.000925594 | 0.317399004 |
| CFL1P2 | 0.096377933 | 1.316226172 | 1.000420766 | 0.317642876 |
| RP11-378J18.8 | -0.078750138 | 1.134410581 | -1.000265411 | 0.31771795 |
| ANK3 | 0.142331371 | -0.2819788 | 1.000188264 | 0.317755235 |
| CCDC144B | -0.195688636 | 1.648424282 | -0.999799489 | 0.317943172 |
| SENCR | -0.102568303 | 3.370936869 | -0.999704881 | 0.317988917 |
| IFITM3 | 0.173545118 | 7.418742657 | 0.999661227 | 0.318010027 |
| RP11-404F10.2 | -0.131715662 | -1.393855493 | -0.998838102 | 0.318408229 |
| ST13P3 | 0.045982557 | 4.221494515 | 0.998833364 | 0.318410522 |
| RP11-797A18.4 | 0.114396356 | -0.241803903 | 0.998466231 | 0.318588236 |
| RP11-443N24.1 | -0.056410265 | 4.143707851 | -0.998379517 | 0.31863022 |
| C9orf66 | -0.079628629 | 1.936393072 | -0.997989162 | 0.318819262 |
| PCDHGB6 | 0.16195946 | -1.179240178 | 0.997920617 | 0.318852465 |
| RYK | 0.054089493 | 4.678156794 | 0.996359098 | 0.319609472 |
| OR7E140P | -0.163769451 | -1.232204365 | -0.996316985 | 0.319629904 |
| COX17P1 | -0.065437405 | 3.043108603 | -0.996044572 | 0.319762093 |
| HSD3B7 | -0.130861892 | 3.145223452 | -0.996002902 | 0.319782317 |
| RP11-872J21.3 | -0.0810889 | 1.319798783 | -0.995992577 | 0.319787328 |
| MAGOH2 | -0.056078513 | 3.322941369 | -0.995943345 | 0.319811223 |
| CNR1 | -0.175034111 | -3.265059577 | -0.995261893 | 0.320142089 |
| HOXA7 | -0.325821025 | 3.536243555 | -0.995080096 | 0.320230395 |
| ANTXR1 | 0.173317232 | -2.506920005 | 0.994760754 | 0.320385551 |
| CCDC163P | -0.105454345 | 2.134680074 | -0.993925003 | 0.320791844 |
| RPS3AP43 | -0.110146106 | -0.674041528 | -0.993886817 | 0.320810416 |
| CCNH | 0.043660004 | 5.381001841 | 0.993186191 | 0.321151291 |
| MTND5P2 | 0.136899937 | 0.295285832 | 0.992963201 | 0.321259833 |
| FAM66B | -0.120012798 | -0.994879692 | -0.99281984 | 0.321329627 |
| RRN3P1 | 0.065206072 | 3.78483774 | 0.992414621 | 0.321526958 |
| RP11-20O24.1 | 0.098770954 | 0.696154927 | 0.99173824 | 0.321856516 |
| CYP4F26P | 0.210423848 | -0.45495351 | 0.990725154 | 0.322350541 |
| RP11-396B14.2 | 0.111666165 | 0.277129764 | 0.990680472 | 0.322372342 |
| AC009302.2 | 0.0506114 | 5.242196582 | 0.990659636 | 0.322382508 |
| PJA2 | 0.038731397 | 7.669779224 | 0.990587908 | 0.322417507 |
| ONECUT2 | -0.260347663 | -5.208462194 | -0.990556765 | 0.322432704 |
| KCNMB3 | 0.086252588 | 2.040562627 | 0.990465267 | 0.322477354 |
| RP11-367E12.4 | 0.0928419 | 0.354377598 | 0.990432396 | 0.322493396 |
| UBXN4 | 0.028112185 | 7.325274053 | 0.990240331 | 0.322587138 |
| H3F3A | 0.059182207 | 6.957655203 | 0.989799556 | 0.322802338 |
| SLFN5 | -0.140734456 | 3.237384808 | -0.989581807 | 0.322908684 |
| BACE2 | 0.185237299 | 0.455625506 | 0.989418781 | 0.322988319 |
| FRMD8P1 | 0.075472259 | 0.701856954 | 0.989377184 | 0.323008641 |
| ZNF385A | 0.127469268 | 6.262589942 | 0.989316471 | 0.323038303 |
| KITLG | -0.207538264 | -1.106018942 | -0.989203798 | 0.323093354 |
| PCAT6 | -0.11763549 | 2.706773091 | -0.98909537 | 0.323146338 |
| CARD11 | 0.12942107 | 4.450251132 | 0.988601156 | 0.323387908 |
| PRKAG2 | 0.062666801 | 4.764643097 | 0.988394683 | 0.323488866 |
| FAUP1 | -0.03180178 | 9.297817601 | -0.988077681 | 0.32364391 |
| AC005307.3 | 0.141377947 | -3.264093989 | 0.987881175 | 0.323740043 |
| RP11-777B9.5 | 0.133395698 | 0.316796276 | 0.98776109 | 0.3237988 |
| LINC00299 | 0.130058435 | -0.73482409 | 0.987580015 | 0.323887412 |
| MFAP3 | 0.057196831 | 3.969846215 | 0.986864048 | 0.324237937 |
| C6orf163 | -0.08239561 | 1.398886321 | -0.986699013 | 0.324318771 |
| DOHH | 0.04615561 | 4.314931691 | 0.986635473 | 0.324349896 |
| REEP2 | 0.132129675 | -1.397336309 | 0.986592649 | 0.324370874 |
| CTC-250P20.1 | -0.090392321 | 1.221251822 | -0.986381992 | 0.324474083 |
| NKG7 | 0.10888486 | 8.655343982 | 0.985643666 | 0.324835986 |
| GK3P | -0.09520041 | 1.455626027 | -0.985635013 | 0.324840229 |
| RP11-332L8.1 | 0.092982147 | 0.293303369 | 0.985528992 | 0.324892219 |
| PHACTR2 | -0.066567041 | 4.640016548 | -0.985262425 | 0.325022961 |
| HSP90AA1 | -0.051877635 | 9.796671289 | -0.98512802 | 0.325088895 |
| RP11-89K11.1 | -0.107485385 | 1.785509848 | -0.985076957 | 0.325113947 |
| REXO2 | -0.068147945 | 4.532145658 | -0.985067998 | 0.325118343 |
| BUD13 | 0.046304886 | 5.749759723 | 0.985054174 | 0.325125125 |
| GBP7 | 0.166711832 | -1.452023671 | 0.984941474 | 0.325180422 |
| SLC11A1 | 0.209659363 | 4.809011187 | 0.984407486 | 0.325442511 |
| HCG27 | -0.106815074 | 2.896437744 | -0.984317871 | 0.325486509 |
| AURKAIP1 | 0.035970429 | 6.705178424 | 0.983917851 | 0.325682952 |
| YWHAZ | 0.03708375 | 8.725794229 | 0.983694396 | 0.325792721 |
| AC090804.1 | -0.106506459 | 0.021325186 | -0.983264944 | 0.32600375 |
| RP1-199J3.5 | -0.102379605 | -0.148328209 | -0.983184693 | 0.326043194 |
| RP11-436H11.1 | 0.066209218 | 3.252890797 | 0.982866995 | 0.326199378 |
| TTLL3 | -0.054079115 | 4.361259038 | -0.982655311 | 0.326303471 |
| ZNF263 | 0.031661857 | 4.693346219 | 0.982548866 | 0.326355822 |
| CTC-308K20.3 | -0.072346135 | 5.285837207 | -0.982506369 | 0.326376725 |
| RPS27AP2 | 0.089232947 | 0.781402462 | 0.982370637 | 0.326443491 |
| EEF1A1P17 | 0.100809881 | 1.549968135 | 0.982289084 | 0.32648361 |
| RPS4XP2 | 0.096895222 | 1.772930987 | 0.982248508 | 0.326503572 |
| RPL22 | -0.066871502 | 7.808677638 | -0.981664096 | 0.326791178 |
| WNK1 | 0.04900958 | 7.142627683 | 0.981469695 | 0.326886885 |
| CDH6 | 0.205129761 | -5.389352264 | 0.98127238 | 0.326984045 |
| ZFP82 | -0.085163456 | 1.380864825 | -0.981071024 | 0.327083214 |
| UQCRB | 0.050667025 | 6.118884709 | 0.980874352 | 0.327180095 |
| AC092106.1 | -0.088347924 | 0.137853525 | -0.980127456 | 0.327548189 |
| CRYBB1 | -0.133424646 | 1.192488862 | -0.980076912 | 0.327573108 |
| MYL9 | 0.207086805 | 2.736454488 | 0.980032569 | 0.327594971 |
| LINC00887 | -0.192891536 | -2.988757937 | -0.979742853 | 0.327737838 |
| ZBED4 | 0.045039194 | 6.261226628 | 0.979663888 | 0.327776785 |
| RP1-68D18.2 | 0.115622689 | 2.994191992 | 0.97944497 | 0.327884775 |
| LAPTM4B | 0.181069297 | 4.823186671 | 0.979052787 | 0.328078292 |
| AC068491.3 | 0.076784996 | 1.001479679 | 0.978962341 | 0.328122932 |
| RP3-465N24.5 | 0.203479613 | 2.181499608 | 0.978345042 | 0.328427707 |
| PCGF7P | 0.064898863 | 2.048648391 | 0.977775525 | 0.328709055 |
| hsa-mir-6723 | -0.084738783 | 8.256803668 | -0.977652474 | 0.328769864 |
| CASP9 | 0.043854087 | 4.286715515 | 0.97753483 | 0.328828007 |
| IGLV2-11 | 0.201518425 | 2.958329216 | 0.977211607 | 0.32898779 |
| CTF1 | 0.126873785 | 1.606973612 | 0.977074598 | 0.329055535 |
| RASSF2 | -0.08062048 | 7.914375605 | -0.976886357 | 0.329148626 |
| RP5-837J1.2 | 0.0492887 | 4.011130165 | 0.976766994 | 0.329207664 |
| JUNB | 0.085438379 | 10.74897356 | 0.976499143 | 0.32934017 |
| CD96 | -0.207175516 | 5.033077577 | -0.976432686 | 0.329373052 |
| ZNF554 | 0.056673697 | 2.252179944 | 0.976116509 | 0.329529519 |
| RNU1-133P | -0.180537017 | -0.270240317 | -0.975986502 | 0.32959387 |
| NUDCD2 | -0.049670705 | 3.584128864 | -0.975888988 | 0.329642143 |
| SNORA45 | 0.084721307 | 2.220621835 | 0.975394096 | 0.329887203 |
| IGKV3OR2-268 | 0.122735459 | -1.003624497 | 0.975384405 | 0.329892003 |
| AC011515.2 | 0.18470572 | -0.259908449 | 0.97516102 | 0.330002659 |
| PIEZO2 | -0.319445226 | -0.055662819 | -0.97514435 | 0.330010917 |
| AC022182.1 | 0.111551681 | -0.877462828 | 0.974994435 | 0.330085194 |
| CCDC8 | -0.243657964 | -1.555876454 | -0.974891776 | 0.330136063 |
| MMEL1 | -0.182702469 | 0.378201793 | -0.974724034 | 0.330219193 |
| ADORA3 | -0.129157818 | 1.08659057 | -0.974348176 | 0.330405511 |
| RP4-575N6.5 | 0.128778324 | -0.243391051 | 0.973635016 | 0.330759221 |
| CTD-2611O12.8 | -0.098975074 | 0.078077099 | -0.973539806 | 0.330806461 |
| FPR2 | 0.232790036 | 2.574236997 | 0.973474305 | 0.330838964 |
| PPP2R3B | -0.066993473 | 3.147400853 | -0.973233747 | 0.330958349 |
| APMAP | -0.077500697 | 8.404281347 | -0.97309764 | 0.331025909 |
| EFHC2 | 0.149813364 | 2.776598827 | 0.972732861 | 0.331207021 |
| ABHD17AP2 | 0.076587439 | 0.927809445 | 0.971954282 | 0.331593798 |
| TRBV24-1 | -0.131975779 | -0.170259074 | -0.97192052 | 0.331610576 |
| EBF3 | 0.228223095 | -2.555974462 | 0.971374454 | 0.33188203 |
| TEX261 | -0.026177909 | 6.157420797 | -0.971320254 | 0.331908981 |
| AC005481.5 | -0.140752991 | -2.824028633 | -0.971294036 | 0.331922019 |
| FUT8 | -0.070764718 | 3.358728978 | -0.971106525 | 0.332015273 |
| MIR3936 | -0.115281393 | 0.6099712 | -0.971102525 | 0.332017262 |
| AC010327.2 | 0.122793945 | -2.085880759 | 0.970858417 | 0.332138689 |
| KLHDC8B | -0.109328315 | 3.831274594 | -0.97061898 | 0.33225782 |
| RP11-536K7.3 | 0.125298269 | -0.214572306 | 0.970578818 | 0.332277806 |
| AC011893.3 | -0.147810532 | -1.514831794 | -0.970411622 | 0.332361014 |
| FAM168A | -0.041427917 | 5.384419354 | -0.969330122 | 0.332899565 |
| GPRC5B | -0.14255484 | -2.437685691 | -0.968996639 | 0.333065742 |
| ATP5LP2 | -0.041062425 | 5.36037173 | -0.968370937 | 0.33337768 |
| RP11-465N4.2 | 0.078174056 | 0.797275148 | 0.968295845 | 0.333415129 |
| NPM1P8 | 0.088120814 | 1.324997851 | 0.968275864 | 0.333425094 |
| FAM66C | -0.105804476 | -0.393800282 | -0.968170156 | 0.333477818 |
| AP001429.1 | 0.105613421 | 0.913859058 | 0.967739065 | 0.333692886 |
| RN7SL280P | -0.110491072 | -0.009899037 | -0.967342403 | 0.333890857 |
| RP11-330L19.1 | 0.117029541 | 1.819401941 | 0.966583738 | 0.334269712 |
| TBCD | -0.043916548 | 4.646697103 | -0.966427457 | 0.334347789 |
| AC138783.12 | -0.100216043 | 0.357194375 | -0.966297657 | 0.334412645 |
| RPS4XP1 | 0.073440544 | 2.682423784 | 0.966158094 | 0.334482389 |
| SLC41A3 | 0.049593774 | 4.269405042 | 0.966149178 | 0.334486845 |
| RP11-284B18.3 | 0.068174104 | 1.007041087 | 0.965939993 | 0.334591399 |
| RP11-509J21.1 | -0.083578558 | 2.758806301 | -0.965521676 | 0.334800543 |
| DST | -0.183319209 | 3.905511771 | -0.965206613 | 0.33495812 |
| RNU7-57P | -0.126399827 | 0.454862882 | -0.965036002 | 0.33504347 |
| STXBP5 | 0.084126544 | 5.887361004 | 0.964678994 | 0.335222112 |
| CERS4 | -0.147964362 | 3.335333753 | -0.964285173 | 0.335419247 |
| RNVU1-14 | -0.136756165 | 3.148774722 | -0.963919258 | 0.33560248 |
| DIAPH2 | -0.070208341 | 5.145361448 | -0.963893498 | 0.335615382 |
| RP11-203B9.4 | 0.115583413 | -2.948376434 | 0.963863765 | 0.335630274 |
| UBE2Q2P6 | 0.103439134 | 5.433631316 | 0.96355042 | 0.335787242 |
| LHFPL2 | 0.131902779 | 3.882959022 | 0.963179895 | 0.335972915 |
| PUF60 | 0.031589417 | 6.299844726 | 0.962863604 | 0.336131464 |
| ST8SIA1 | 0.165604123 | -2.130062577 | 0.96255144 | 0.336287991 |
| NPM1P25 | -0.080958801 | 3.114859123 | -0.962548734 | 0.336289349 |
| BAI1 | -0.14398588 | 1.962809925 | -0.962314871 | 0.336406645 |
| EHD4 | 0.111492172 | 4.583467099 | 0.962091435 | 0.336518736 |
| AIM1 | -0.095294686 | 5.589761803 | -0.96141513 | 0.336858164 |
| ZNF667 | 0.241687827 | 0.73569074 | 0.961314743 | 0.336908566 |
| PNMAL2 | -0.181273917 | -3.005368486 | -0.961124031 | 0.337004332 |
| RP11-248J23.6 | -0.12411671 | -1.402247998 | -0.960816387 | 0.33715885 |
| AMY1C | -0.130239304 | -1.774401536 | -0.960571295 | 0.337281984 |
| DHX40P1 | -0.104318604 | 0.079752814 | -0.960325704 | 0.337405398 |
| SLC10A4 | 0.159960728 | -0.89412608 | 0.959728512 | 0.337705619 |
| ZNF580 | 0.04230882 | 5.663653425 | 0.959437975 | 0.33785174 |
| RN7SKP269 | 0.098372893 | 0.35357549 | 0.959205884 | 0.337968495 |
| RP11-1079K10.4 | -0.129507444 | -1.662310341 | -0.958863908 | 0.338140577 |
| ZNF135 | -0.200485804 | 0.786819086 | -0.958169657 | 0.338490098 |
| AC104651.2 | 0.044265437 | 2.237204998 | 0.958149706 | 0.338500145 |
| MTRNR2L2 | 0.091790971 | 3.745132914 | 0.957900847 | 0.338625492 |
| LDHC | -0.169509207 | -1.881813728 | -0.957799335 | 0.338676631 |
| C8orf31 | -0.211035192 | -1.079094081 | -0.957253119 | 0.338951884 |
| HLA-F-AS1 | -0.112883142 | 0.351490502 | -0.956837701 | 0.339161321 |
| C11orf58 | 0.025044711 | 6.167188642 | 0.956201751 | 0.339482103 |
| MCAT | -0.049682221 | 4.375171947 | -0.955890623 | 0.339639111 |
| TOP3B | 0.028666967 | 4.313020038 | 0.95571336 | 0.339728586 |
| CTD-2506J14.1 | -0.126110432 | -0.916824436 | -0.955710161 | 0.339730201 |
| FANCE | -0.076653604 | 3.857737018 | -0.95553549 | 0.339818383 |
| RP4-607I7.1 | -0.128135691 | -0.427559474 | -0.955176045 | 0.339999894 |
| SHH | 0.18150263 | -2.833947712 | 0.954785661 | 0.340197098 |
| RP11-142C4.6 | -0.165586641 | -0.126044918 | -0.954271183 | 0.340457102 |
| AC007285.6 | -0.128824613 | -1.298181643 | -0.954126285 | 0.340530353 |
| MTMR7 | -0.184620165 | -3.498320874 | -0.953766129 | 0.340712468 |
| C4B | 0.100661252 | -0.762783168 | 0.953331821 | 0.340932161 |
| RP11-1149O23.3 | -0.106374432 | 1.993986593 | -0.953184845 | 0.341006529 |
| RP11-169D4.2 | -0.146461734 | 0.996963783 | -0.952803556 | 0.341199504 |
| DXO | 0.043602679 | 5.351640198 | 0.952729825 | 0.341236829 |
| AP000695.6 | 0.144875198 | -0.907137281 | 0.952554717 | 0.341325483 |
| AC096664.2 | -0.08553072 | 0.075752826 | -0.951605966 | 0.341806078 |
| RP11-284F21.9 | 0.164730598 | -1.911351076 | 0.95122683 | 0.341998253 |
| RP11-649A18.12 | -0.064375637 | 2.072744285 | -0.95104222 | 0.342091852 |
| RP4-800G7.2 | -0.102240792 | 2.317246608 | -0.950770599 | 0.342229597 |
| LRRK2 | -0.217985958 | 3.772234909 | -0.950700387 | 0.342265209 |
| POU5F2 | -0.131997585 | -2.534560568 | -0.950194267 | 0.342521985 |
| BMX | -0.216514689 | 1.958344229 | -0.950124903 | 0.342557186 |
| SLC24A3 | -0.231490766 | 2.284316096 | -0.950039489 | 0.342600535 |
| NPM1P24 | -0.059787541 | 4.820555386 | -0.949958446 | 0.342641669 |
| ECSCR | 0.126346583 | -0.280316889 | 0.949789888 | 0.342727233 |
| MAP3K5 | -0.056199077 | 5.528978441 | -0.949642564 | 0.342802028 |
| SYNGAP1 | -0.075827849 | 5.498180975 | -0.949383393 | 0.342933633 |
| KRT6A | 0.226162707 | -3.409206574 | 0.949308748 | 0.342971544 |
| POLD1 | 0.064622157 | 5.544774673 | 0.949189377 | 0.343032175 |
| FOXD2-AS1 | 0.120344833 | 2.717587807 | 0.948549559 | 0.343357269 |
| MAST1 | -0.138910543 | -0.927201378 | -0.948351045 | 0.343458175 |
| RP11-685B14.1 | 0.105472367 | 0.800486212 | 0.948310931 | 0.343478567 |
| KLC2 | 0.075927508 | 3.029372793 | 0.9482167 | 0.343526474 |
| UBE2QL1 | -0.142730968 | -3.232002582 | -0.948197867 | 0.343536049 |
| FLT3LG | -0.098349647 | -0.590787858 | -0.948092387 | 0.34358968 |
| KSR1 | 0.073135971 | 4.60749308 | 0.947722922 | 0.343777578 |
| TBC1D20 | 0.031998136 | 5.576862997 | 0.947721639 | 0.343778231 |
| AC012065.7 | 0.133694077 | -1.526311711 | 0.947672244 | 0.343803356 |
| CTTNBP2 | 0.1509777 | -4.340105775 | 0.947433587 | 0.343924771 |
| CTA-242H14.1 | 0.093071925 | 0.433607161 | 0.9470463 | 0.344121857 |
| KREMEN2 | 0.129041725 | -1.915297611 | 0.946751173 | 0.344272092 |
| WRAP53 | 0.04701139 | 3.491243048 | 0.94639937 | 0.344451234 |
| AC005082.12 | -0.201114474 | -1.576352586 | -0.945420573 | 0.344949961 |
| SYTL2 | 0.109283672 | 1.608457774 | 0.945338957 | 0.344991568 |
| RP11-177H13.2 | 0.151867124 | -3.352719573 | 0.945013813 | 0.345157354 |
| ZC4H2 | -0.070527402 | 2.619668033 | -0.944891568 | 0.345219697 |
| CEACAM4 | -0.140170002 | 5.445036115 | -0.944868701 | 0.34523136 |
| GBP1 | 0.17049843 | 3.012696847 | 0.944380606 | 0.345480362 |
| PAM16 | 0.063605408 | 3.620222443 | 0.94398809 | 0.345680687 |
| SLC29A1 | 0.091667308 | 5.887192919 | 0.943914893 | 0.345718053 |
| RIN1 | -0.123130813 | 2.823024619 | -0.943810941 | 0.345771122 |
| MAPK11 | 0.127011276 | 3.476823622 | 0.943799479 | 0.345776974 |
| SEMA4A | -0.124835206 | 5.544737689 | -0.943635473 | 0.345860713 |
| POLA1 | 0.059704623 | 4.549832204 | 0.943151089 | 0.346108109 |
| RP11-705C15.3 | -0.081155021 | 4.006553337 | -0.942864114 | 0.346254734 |
| PRSS3P1 | 0.182876211 | -1.468318809 | 0.942850858 | 0.346261508 |
| MIR647 | -0.073699304 | 2.834464198 | -0.942732574 | 0.346321954 |
| PFKL | -0.03665281 | 6.768921491 | -0.942729667 | 0.34632344 |
| ITGB7 | -0.117238392 | 5.891115814 | -0.942668926 | 0.346354483 |
| GPA33 | -0.169924187 | 2.996451788 | -0.942646304 | 0.346366045 |
| MLH1 | -0.035305885 | 5.576077646 | -0.94189656 | 0.346749378 |
| TP53TG1 | -0.049829423 | 4.445257812 | -0.941638262 | 0.346881504 |
| COQ6 | 0.035760946 | 3.609194403 | 0.941170182 | 0.347121022 |
| LOXL3 | -0.105647127 | 4.042657004 | -0.940948161 | 0.347234667 |
| ACAD11 | 0.05875822 | 1.591072235 | 0.94078942 | 0.347315937 |
| TRPM4 | 0.238970362 | 0.299831745 | 0.940677324 | 0.347373333 |
| TYW1B | 0.059633943 | 2.713986505 | 0.940627875 | 0.347398654 |
| RASA4DP | -0.09033525 | 2.59118089 | -0.940620092 | 0.347402639 |
| IGHV2-70 | 0.18135574 | 1.162057619 | 0.940441349 | 0.347494178 |
| PLXNB1 | -0.146120068 | 2.564939744 | -0.940381249 | 0.34752496 |
| CD58 | -0.066654789 | 6.320160129 | -0.940105747 | 0.347666089 |
| RP11-39K24.14 | 0.123142213 | 1.393428875 | 0.939274514 | 0.348092119 |
| RP11-154J22.1 | -0.092772817 | -0.048800541 | -0.93926333 | 0.348097853 |
| GOLGA6L19 | 0.126087445 | -1.879718141 | 0.93891208 | 0.348277981 |
| RP11-559N14.5 | 0.146961321 | -1.877631529 | 0.938747495 | 0.348362403 |
| CCL24 | 0.208802302 | 0.024306678 | 0.938526604 | 0.348475729 |
| HNRNPA1P26 | 0.082616393 | 2.486205247 | 0.937735807 | 0.348881628 |
| HIVEP2 | -0.10279074 | 4.403829908 | -0.937499798 | 0.349002825 |
| GALNT4 | 0.078876688 | -0.435953844 | 0.937184445 | 0.349164808 |
| RP11-589F5.3 | -0.143407544 | -1.996819222 | -0.937164438 | 0.349175087 |
| KCND3 | 0.13281227 | -4.400171993 | 0.93698793 | 0.349265774 |
| KRT73 | 0.172013918 | 0.342425889 | 0.936876377 | 0.349323097 |
| S100A9 | 0.188106076 | 11.52934168 | 0.935707664 | 0.349924008 |
| C1QTNF6 | 0.082694869 | 2.020788002 | 0.935692877 | 0.349931615 |
| C9orf117 | -0.143970946 | -3.158814605 | -0.935689923 | 0.349933134 |
| MSNP1 | -0.048205538 | 7.264858282 | -0.935662842 | 0.349947067 |
| RP11-541F9.2 | -0.122759504 | -2.117608864 | -0.935438377 | 0.350062559 |
| AL590762.6 | 0.059143379 | 1.987173156 | 0.934887433 | 0.350346135 |
| ITGA1 | -0.16531237 | -0.487865224 | -0.93484992 | 0.350365448 |
| LSM7 | -0.04814615 | 6.56379013 | -0.934645754 | 0.350470575 |
| RP4-737E23.2 | 0.184786534 | -0.450786834 | 0.93424546 | 0.350676748 |
| NRG1 | -0.238377231 | -2.829893697 | -0.934192527 | 0.350704017 |
| RP11-783K16.5 | -0.115233964 | 0.494271183 | -0.933965378 | 0.350821051 |
| RASGEF1C | 0.139867033 | -2.689685411 | 0.933322043 | 0.351152651 |
| SPCS2 | 0.041409719 | 3.861100863 | 0.933281001 | 0.351173813 |
| BSG | -0.054059012 | 8.637773737 | -0.932949417 | 0.351344809 |
| SCARF1 | -0.106387648 | 4.643405622 | -0.932658627 | 0.351494811 |
| MIR3191 | -0.136624478 | 0.43040648 | -0.932638556 | 0.351505166 |
| C10orf105 | -0.150395772 | 0.50310053 | -0.932411201 | 0.351622476 |
| ZNF711 | 0.260813459 | 2.517635124 | 0.931423308 | 0.352132497 |
| GABRE | -0.274272549 | -3.510774889 | -0.931294863 | 0.352198843 |
| ASCC3 | -0.034199753 | 4.899912487 | -0.931197006 | 0.352249396 |
| CTD-2105E13.13 | -0.10747299 | 1.401035659 | -0.931184968 | 0.352255615 |
| NANOS1 | -0.128143959 | -1.593009299 | -0.931131615 | 0.352283179 |
| SPINK8 | -0.146736285 | -0.197999551 | -0.931021328 | 0.352340161 |
| LINC01119 | 0.110422005 | -1.17242055 | 0.930801167 | 0.352453931 |
| MKL1 | 0.038729665 | 6.717171692 | 0.930792105 | 0.352458614 |
| FAH | -0.074758253 | 4.17173645 | -0.930691459 | 0.352510632 |
| YIPF5 | -0.048464032 | 4.57549184 | -0.930534659 | 0.352591681 |
| ZFR | 0.044269481 | 6.948383115 | 0.930519832 | 0.352599346 |
| FZD9 | 0.136905426 | -1.621400863 | 0.92997927 | 0.352878856 |
| GSTM1 | -0.307339793 | 1.71021612 | -0.929567198 | 0.353092022 |
| RP11-318K15.2 | 0.142008634 | 1.86284061 | 0.92912244 | 0.353322188 |
| AP001628.6 | 0.098861064 | 0.8093791 | 0.929066086 | 0.353351359 |
| RP11-109N23.4 | -0.08533916 | 0.092655983 | -0.928989659 | 0.353390922 |
| COL6A6 | 0.210218538 | -3.223310202 | 0.92869207 | 0.353544999 |
| PCDHGA9 | 0.139161122 | -2.043299201 | 0.928269353 | 0.353763934 |
| CACNB3 | -0.110446343 | 2.20904514 | -0.928168379 | 0.353816244 |
| RP11-479G22.8 | 0.104963831 | 1.535243807 | 0.928001616 | 0.353902646 |
| CTSV | 0.175866138 | -1.335888654 | 0.927764477 | 0.354025534 |
| PLK2 | 0.117749256 | 3.763807772 | 0.92774891 | 0.354033602 |
| FABP5P1 | 0.136355262 | 0.382913169 | 0.927632973 | 0.354093693 |
| ZNF365 | 0.138326981 | -2.688191143 | 0.927222708 | 0.354306387 |
| HSD17B3 | -0.137480306 | -1.064885636 | -0.927122069 | 0.354358573 |
| ESRP1 | 0.123448239 | -4.164916947 | 0.926742005 | 0.354555701 |
| CTSH | 0.176528605 | 4.793190753 | 0.926697806 | 0.35457863 |
| RP11-91K8.2 | -0.063518065 | 1.740004194 | -0.926652055 | 0.354602366 |
| RWDD4P1 | -0.048379236 | 2.537462403 | -0.926502273 | 0.354680079 |
| RP11-98J23.1 | -0.137006583 | -2.887520685 | -0.926333847 | 0.354767478 |
| ABTB1 | -0.07310079 | 5.911145592 | -0.925788171 | 0.355050731 |
| RNF165 | -0.162498513 | -1.754851552 | -0.925672387 | 0.355110852 |
| RP11-383H13.1 | -0.192084817 | -1.347045392 | -0.925427023 | 0.355238278 |
| CTD-2366F13.2 | -0.107407859 | -0.143750681 | -0.925258261 | 0.355325938 |
| KIAA0922 | 0.047947093 | 5.636679886 | 0.92519495 | 0.355358827 |
| GAPDHP21 | 0.113417602 | -0.795468819 | 0.925007015 | 0.355456469 |
| C4orf50 | -0.121765877 | -2.806504834 | -0.924979261 | 0.35547089 |
| RP11-313P13.5 | -0.097352841 | 1.396689978 | -0.924854718 | 0.355535607 |
| AUP1 | -0.028609434 | 8.083092293 | -0.92418833 | 0.355882015 |
| TMEM178A | -0.169769846 | -0.000203643 | -0.923828766 | 0.356069015 |
| CTC-425F1.4 | -0.131996349 | 0.632274845 | -0.923711922 | 0.356129796 |
| WTIP | 0.09279982 | -2.268172797 | 0.923556319 | 0.356210749 |
| EIF4ENIF1 | 0.025190622 | 4.914062259 | 0.923240439 | 0.356375123 |
| AKR1C6P | 0.184092036 | -1.231202979 | 0.923159627 | 0.356417183 |
| ATP5D | 0.031931842 | 6.610709038 | 0.922786419 | 0.356611464 |
| RP3-414A15.10 | -0.120411139 | -0.641440782 | -0.922426839 | 0.356798715 |
| XIST | -0.559081196 | 2.021239638 | -0.92167925 | 0.35718822 |
| TAX1BP1 | 0.037620954 | 6.484635279 | 0.921372305 | 0.357348221 |
| AVPR1B | 0.158840391 | -2.23752201 | 0.920916496 | 0.357585903 |
| PIFO | -0.129027946 | -1.843818664 | -0.920797404 | 0.35764802 |
| CPEB3 | -0.064140347 | 2.603119507 | -0.920674964 | 0.357711891 |
| RP6-65G23.3 | 0.113088139 | -1.005367281 | 0.920474983 | 0.357816226 |
| EPB41L5 | -0.125584945 | 0.559618367 | -0.91997212 | 0.358078667 |
| ZNF596 | -0.054429397 | 1.41264359 | -0.919521904 | 0.358313735 |
| MIR4442 | -0.112636073 | 0.722511759 | -0.919221337 | 0.358470722 |
| FAR2P2 | -0.14423666 | -3.754228782 | -0.918682728 | 0.358752147 |
| MCF2 | 0.112902145 | -0.697078135 | 0.917759494 | 0.359234866 |
| RP11-229P13.19 | -0.153600143 | 1.129491425 | -0.917684664 | 0.359274009 |
| RP11-844P9.2 | 0.206706936 | -2.745300232 | 0.917639536 | 0.359297616 |
| RP11-540B6.6 | -0.062738835 | 5.002702606 | -0.916983505 | 0.359640912 |
| SLC16A6P1 | 0.095692748 | -0.976930068 | 0.916965532 | 0.35965032 |
| THAP9 | 0.039836009 | 2.606500837 | 0.916563477 | 0.359860818 |
| LGALS9C | -0.093054485 | 0.091580359 | -0.915902557 | 0.360207015 |
| RP11-673E1.1 | 0.109228433 | -1.784910892 | 0.915890915 | 0.360213115 |
| AP000356.1 | -0.16129422 | 0.706393748 | -0.914968052 | 0.360696876 |
| INPP5J | -0.182121936 | -2.381094186 | -0.914734182 | 0.360819535 |
| MAEL | 0.167836459 | -1.639311465 | 0.914591075 | 0.360894603 |
| RP11-1148O4.1 | 0.04124651 | 3.784146154 | 0.914519343 | 0.360932235 |
| IQCH | -0.106399308 | -0.827509342 | -0.914474849 | 0.360955579 |
| RP11-385F5.5 | -0.069231854 | 2.917416245 | -0.914304308 | 0.361045061 |
| LRCH2 | 0.131853938 | -3.969402811 | 0.914135832 | 0.361133473 |
| ACAT2 | 0.043911627 | 4.712118994 | 0.913761073 | 0.361330186 |
| RP4-612B18.1 | 0.067547026 | 2.622729689 | 0.913513896 | 0.361459968 |
| TMEM161BP1 | -0.078415553 | 0.117348085 | -0.913174271 | 0.361638338 |
| RP11-504P24.5 | 0.107007938 | 1.340505276 | 0.912836226 | 0.361815933 |
| SDPR | 0.194042302 | 3.722064078 | 0.912705262 | 0.361884752 |
| TBKBP1 | -0.075064857 | 5.305684472 | -0.912645027 | 0.361916406 |
| ELFN2 | 0.161790352 | -2.815537677 | 0.912462511 | 0.362012332 |
| TPSG1 | 0.155489825 | -1.549295092 | 0.912178256 | 0.362161762 |
| RPL12P2 | 0.095134554 | 1.512728387 | 0.912094397 | 0.362205853 |
| BHLHB9 | 0.091709386 | 0.605987522 | 0.911842324 | 0.362338407 |
| LRMP | 0.056866491 | 6.132065271 | 0.911537345 | 0.362498823 |
| TIPARP-AS1 | 0.06371443 | 2.26893698 | 0.91081106 | 0.362881021 |
| NLRP11 | -0.152791838 | -3.403994573 | -0.910795905 | 0.362889 |
| RP11-212P7.1 | 0.053801293 | 6.761926187 | 0.910777782 | 0.36289854 |
| PCDHGC3 | 0.182829302 | -0.298535415 | 0.910671338 | 0.362954578 |
| YY2 | -0.069221842 | 0.525725673 | -0.910588016 | 0.362998446 |
| RP11-812E19.9 | 0.103502088 | -1.091726016 | 0.91056435 | 0.363010907 |
| RPLP0P6 | 0.046393346 | 11.3672378 | 0.910344371 | 0.363126745 |
| HNRNPA1P27 | 0.093655616 | -0.902081809 | 0.909684747 | 0.363474231 |
| RPL23AP69 | 0.094937717 | -0.186631287 | 0.909367516 | 0.36364142 |
| RP11-77P6.2 | -0.078992183 | 0.179995335 | -0.908922424 | 0.363876078 |
| KIF7 | 0.157717115 | 1.162249921 | 0.908875815 | 0.363900656 |
| RP11-44F14.2 | -0.143707303 | -1.126722794 | -0.908873359 | 0.363901951 |
| ANAPC1P1 | -0.082626391 | -0.003614096 | -0.908473108 | 0.364113059 |
| RP11-265B8.5 | -0.097181444 | 0.081406598 | -0.908204663 | 0.36425469 |
| SOX5 | 0.160508639 | -3.736712309 | 0.907713602 | 0.364513861 |
| PADI3 | -0.169324154 | -2.499331878 | -0.907453506 | 0.364651182 |
| NCL | -0.051373791 | 9.114513801 | -0.907386699 | 0.364686458 |
| ZNF20 | -0.084850319 | -1.114100227 | -0.90694148 | 0.364921605 |
| PKP1 | 0.161196684 | -4.875425037 | 0.906914247 | 0.364935992 |
| IL17RE | -0.173351327 | 1.989380078 | -0.906888381 | 0.364949657 |
| CKLF | 0.05616786 | 4.50532457 | 0.906439519 | 0.365186836 |
| PSCA | 0.140819849 | -0.836606032 | 0.90633567 | 0.365241723 |
| TMEM120B | -0.056693757 | 3.405430962 | -0.906301537 | 0.365259765 |
| CCL23 | -0.211111702 | 2.787010676 | -0.90618852 | 0.365319506 |
| MIR3648 | 0.104319277 | 2.858112164 | 0.905904418 | 0.36546971 |
| UBB | 0.035148772 | 10.89438083 | 0.905400381 | 0.365736287 |
| RP11-434D12.1 | 0.075785284 | 1.858804738 | 0.905231819 | 0.365825464 |
| NFYAP1 | 0.162514178 | -1.928134286 | 0.905062304 | 0.365915159 |
| PAQR4 | -0.067914164 | 5.388594983 | -0.905026113 | 0.365934311 |
| RP11-503E24.2 | 0.061234707 | 3.820107335 | 0.904479503 | 0.36622364 |
| ADAMTS1 | -0.219957941 | -0.343353231 | -0.90447528 | 0.366225876 |
| RP11-702H23.2 | -0.104316382 | -0.410800765 | -0.904253929 | 0.366343082 |
| CSNK1G3 | 0.046275944 | 4.919773972 | 0.903575215 | 0.366702609 |
| SPDL1 | 0.052677006 | 3.571372854 | 0.903504447 | 0.366740109 |
| KRT18 | -0.241950726 | 2.696316266 | -0.90300028 | 0.367007334 |
| TMEM176A | -0.237884278 | 1.721017236 | -0.90250355 | 0.367270738 |
| IMMP2L | -0.080204036 | 2.44092565 | -0.901948526 | 0.367565193 |
| AC092641.2 | -0.058736472 | 2.050439325 | -0.901574604 | 0.367763652 |
| RP11-572O17.1 | -0.073905276 | 3.334409211 | -0.901421649 | 0.367844852 |
| KDM5D | 0.484415376 | 1.414482704 | 0.901396616 | 0.367858142 |
| HSPA5 | 0.063210153 | 10.03245729 | 0.900885922 | 0.368129344 |
| RP11-728F11.4 | -0.171848464 | -0.263729177 | -0.900792181 | 0.368179138 |
| SH3BP2 | 0.053509705 | 6.183189602 | 0.900663885 | 0.368247295 |
| HMGN3P1 | -0.09871136 | 0.050940679 | -0.900099566 | 0.368547179 |
| PPP1R12A | -0.032479604 | 6.088733662 | -0.8997399 | 0.368738389 |
| RP11-130F10.1 | -0.0903052 | 0.798206198 | -0.899721173 | 0.368748346 |
| RP13-88F20.1 | -0.049308505 | 2.633477582 | -0.899012209 | 0.369125441 |
| CHCHD2P8 | 0.08426644 | 0.859684768 | 0.898941103 | 0.369163275 |
| LDB1 | 0.056563126 | 7.943841287 | 0.898937348 | 0.369165273 |
| TREML4 | 0.252781724 | -1.267375721 | 0.898825289 | 0.369224903 |
| MFSD11 | 0.035764887 | 3.562885036 | 0.898800938 | 0.369237862 |
| COL23A1 | 0.211611617 | 2.938681184 | 0.89875483 | 0.369262399 |
| RAB26 | 0.113435108 | -1.860326998 | 0.898731815 | 0.369274647 |
| KIAA1586 | -0.048185585 | 4.373301824 | -0.898707163 | 0.369287768 |
| LDHAL6FP | -0.109428952 | -1.127955229 | -0.898634704 | 0.369326332 |
| DIP2C | -0.113162991 | -1.691493919 | -0.898491329 | 0.369402648 |
| AC004947.2 | -0.14573308 | -2.869997952 | -0.898382443 | 0.369460612 |
| CHTOP | -0.026518376 | 6.336657038 | -0.898151097 | 0.369583786 |
| HS6ST1P1 | -0.080303979 | 2.895374015 | -0.897425482 | 0.369970285 |
| CLDN9 | 0.142692734 | 1.466401888 | 0.897271328 | 0.370052428 |
| LGALS3BP | -0.19448654 | 5.583068281 | -0.89704656 | 0.370172218 |
| ANKRD20A7P | 0.177289558 | -2.017748241 | 0.896656569 | 0.370380122 |
| CTC-250I14.6 | 0.129786864 | 1.314259084 | 0.896464074 | 0.370482768 |
| HMGB3P4 | 0.088862227 | 1.235223505 | 0.89621716 | 0.370614458 |
| CMTM3 | 0.055167223 | 6.718714255 | 0.89556234 | 0.370963842 |
| BMS1 | -0.037089235 | 5.29223622 | -0.895541847 | 0.37097478 |
| HACL1 | -0.033253141 | 4.621213257 | -0.895460706 | 0.371018089 |
| FUOM | -0.067513366 | 4.588940282 | -0.895253526 | 0.371128684 |
| C11orf35 | 0.070037153 | 2.952762744 | 0.894982574 | 0.371273352 |
| OFD1 | -0.046351152 | 5.648282175 | -0.894754891 | 0.371394946 |
| KDM5B | -0.066478644 | 6.253419999 | -0.894570438 | 0.371493471 |
| RPL4P5 | 0.041878603 | 9.120920259 | 0.894560186 | 0.371498947 |
| MSX2 | -0.160563023 | -2.405680982 | -0.894533612 | 0.371513143 |
| ISPD | 0.1043833 | 1.830867695 | 0.89406164 | 0.371765327 |
| MOCOS | -0.164614345 | -2.306483501 | -0.894047812 | 0.371772718 |
| RP11-16F15.1 | -0.04004509 | 5.71706852 | -0.893859928 | 0.371873138 |
| CCDC124 | 0.031852334 | 6.588799711 | 0.893851694 | 0.37187754 |
| FCGBP | -0.153697381 | -0.660912182 | -0.893760869 | 0.371926091 |
| FAM24B | -0.129209995 | 0.146814459 | -0.893745513 | 0.3719343 |
| PAK6 | 0.127509264 | -0.767552421 | 0.89350153 | 0.372064743 |
| RP11-96D1.10 | -0.07578859 | 1.368440132 | -0.892567538 | 0.372564358 |
| CR1 | -0.177681977 | 3.90458313 | -0.892053605 | 0.37283945 |
| SHMT2 | 0.048986768 | 6.183043199 | 0.891349229 | 0.373216687 |
| RPL37A | 0.049447029 | 9.25625665 | 0.890298949 | 0.373779615 |
| RP11-618N24.1 | 0.080507597 | 0.173536446 | 0.890065128 | 0.373905011 |
| AC073072.7 | 0.118077903 | 0.058467178 | 0.890038234 | 0.373919435 |
| LYPD6 | -0.156560514 | -3.611044961 | -0.889928711 | 0.373978181 |
| REEP6 | -0.100184049 | 2.775417642 | -0.889187572 | 0.374375865 |
| PTPRK | 0.133425453 | -2.034822652 | 0.888989242 | 0.37448233 |
| TPM3P9 | -0.042330832 | 4.267508537 | -0.888838991 | 0.374562999 |
| PRPF38AP2 | -0.073533777 | 1.070145787 | -0.888822205 | 0.374572011 |
| RNA28S5 | 0.056195225 | 10.19730092 | 0.888724864 | 0.37462428 |
| ZNF204P | -0.146656329 | -0.348701676 | -0.88867166 | 0.37465285 |
| RP11-353N4.5 | -0.196237573 | -0.053602324 | -0.888657106 | 0.374660666 |
| TRIM45 | 0.098986844 | 1.955636383 | 0.888517861 | 0.374735447 |
| TNFAIP8L1 | -0.055204368 | 3.879046885 | -0.88841375 | 0.374791365 |
| RDM1 | 0.118993951 | -0.874571843 | 0.888321089 | 0.374841139 |
| MEIS1 | 0.254468814 | 3.522054231 | 0.888267019 | 0.374870184 |
| RP5-1180E21.4 | -0.094901732 | 2.417064711 | -0.888043814 | 0.374990102 |
| AC012074.2 | 0.099729209 | -0.024882492 | 0.887806226 | 0.375117773 |
| GRK6 | 0.049593941 | 7.171786609 | 0.887376276 | 0.375348882 |
| RPS19P1 | 0.04765276 | 4.747808526 | 0.886848831 | 0.375632516 |
| KATNAL2 | 0.104959636 | 0.580068978 | 0.886626024 | 0.375752371 |
| TRIM25 | -0.045442135 | 6.860894601 | -0.886466585 | 0.375838153 |
| RP11-388C12.8 | -0.095345488 | -0.95677563 | -0.886268467 | 0.375944762 |
| RP11-594N15.3 | 0.123106989 | -1.851598225 | 0.886183947 | 0.375990248 |
| TBC1D4 | -0.118025882 | 2.84425536 | -0.886014605 | 0.376081394 |
| RP11-290H9.4 | 0.099271184 | -0.624691742 | 0.886010567 | 0.376083568 |
| RP11-76E16.2 | -0.106732214 | 3.047441887 | -0.885877311 | 0.376155301 |
| RP11-599J14.2 | 0.093831821 | 3.9320519 | 0.885565064 | 0.376323419 |
| CLXN | 0.147104694 | -3.928385734 | 0.885179072 | 0.376531308 |
| SCO1 | 0.032020489 | 5.347808311 | 0.885105837 | 0.376570759 |
| RP11-510H23.1 | -0.146821523 | -1.200958997 | -0.884788451 | 0.376741762 |
| PKDREJ | -0.117215876 | -3.686869102 | -0.884625966 | 0.376829325 |
| ZNF154 | -0.168208643 | 1.763769571 | -0.884614505 | 0.376835502 |
| RP11-87G24.3 | 0.128880357 | -1.742141455 | 0.884408542 | 0.376946515 |
| IGBP1P4 | -0.083781539 | -0.351642459 | -0.884290402 | 0.3770102 |
| TRBV26OR9-2 | 0.155679141 | -0.533594875 | 0.884275878 | 0.37701803 |
| TM4SF19-AS1 | 0.097628496 | 0.86951323 | 0.883805812 | 0.377271498 |
| ARL5A | -0.024633102 | 5.459913783 | -0.883688442 | 0.377334802 |
| LEPREL2 | 0.158809269 | 1.206665373 | 0.883651886 | 0.37735452 |
| RP11-436I9.6 | -0.119802188 | 0.708416186 | -0.883519932 | 0.377425701 |
| IGLV10-54 | 0.159930545 | 0.020204024 | 0.883348496 | 0.377518192 |
| RP11-278A23.2 | 0.131026291 | 1.372219722 | 0.883335624 | 0.377525137 |
| PQLC2 | 0.06416355 | 4.440789499 | 0.883110487 | 0.377646623 |
| ATP6V0D1 | -0.063899869 | 5.704281579 | -0.883064924 | 0.377671212 |
| AC092653.5 | -0.13064169 | -0.484931141 | -0.882730272 | 0.377851844 |
| WDR60 | -0.053620168 | 4.019261394 | -0.882676968 | 0.377880621 |
| CYP4F2 | 0.192620796 | 0.452965501 | 0.882613288 | 0.377915 |
| GALNTL6 | 0.168388493 | -3.758061417 | 0.882592703 | 0.377926114 |
| ARMCX1 | -0.09598968 | 3.950640233 | -0.881954149 | 0.378270972 |
| BIVM | -0.116394793 | 3.20629642 | -0.881749656 | 0.378381451 |
| CTB-12A17.3 | -0.115816581 | 0.495067115 | -0.881733022 | 0.378390439 |
| SMDT1 | 0.046741249 | 6.217662388 | 0.88100508 | 0.378783885 |
| TRPV4 | -0.176896825 | -1.845131539 | -0.880662548 | 0.378969108 |
| TMPRSS9 | 0.139405042 | 0.77268868 | 0.880502424 | 0.379055714 |
| MTND5P14 | 0.110915068 | 1.285218816 | 0.880456318 | 0.379080654 |
| RPL8P2 | 0.083366809 | 1.023422784 | 0.880018551 | 0.379317499 |
| RP11-170L3.7 | 0.144069832 | 0.160619499 | 0.879960322 | 0.37934901 |
| NMUR1 | 0.15757024 | 1.654397702 | 0.879544411 | 0.379574126 |
| RP11-261C10.3 | 0.082710949 | 1.03450806 | 0.879464843 | 0.379617203 |
| DEGS1 | 0.051192067 | 6.546179538 | 0.878376763 | 0.38020657 |
| C7orf63 | -0.154614397 | -2.574662476 | -0.877815493 | 0.380510806 |
| RP11-347C18.5 | -0.059496225 | 3.523515103 | -0.877577438 | 0.380639889 |
| NPIPA3 | 0.059559784 | 2.521800193 | 0.877480371 | 0.380692531 |
| PLD4 | -0.162642174 | 5.728435111 | -0.877048474 | 0.380926812 |
| DAAM1 | -0.071175523 | 3.248482432 | -0.876720128 | 0.381104982 |
| RP11-146N23.1 | 0.067773209 | 6.201687859 | 0.876508435 | 0.381219879 |
| MRRF | -0.034151582 | 5.080613617 | -0.876372151 | 0.38129386 |
| MGST3 | -0.053098481 | 4.482015379 | -0.876226364 | 0.381373008 |
| TBC1D9 | -0.17482195 | 2.755543579 | -0.875845584 | 0.381579783 |
| GPR173 | 0.23619322 | -1.163583052 | 0.87547207 | 0.38178268 |
| AP000620.1 | -0.101720568 | 0.82641501 | -0.875437146 | 0.381801655 |
| RP11-849F2.5 | -0.143401715 | -1.497570287 | -0.874714159 | 0.38219459 |
| PROC | -0.147379825 | -0.677403218 | -0.874650766 | 0.382229055 |
| C14orf182 | 0.090078163 | 0.061688017 | 0.874569814 | 0.382273069 |
| ZNF24 | 0.033476711 | 5.846156605 | 0.874549726 | 0.382283992 |
| IGKV2D-28 | 0.152840602 | 1.021298613 | 0.874470899 | 0.382326855 |
| ATP5S | 0.038279278 | 4.132798423 | 0.874364368 | 0.382384787 |
| TMEM150C | 0.173400593 | -0.950540905 | 0.873852371 | 0.382663287 |
| ZNF703 | 0.187919841 | 2.471972297 | 0.873594889 | 0.382803391 |
| OAF | -0.082979212 | 5.251632914 | -0.872874761 | 0.383195403 |
| SYT3 | -0.146567658 | -3.120716129 | -0.872852629 | 0.383207455 |
| AL049840.1 | 0.076603092 | 2.27404103 | 0.87256767 | 0.383362647 |
| RN7SL3 | -0.099561325 | 0.734140522 | -0.872550819 | 0.383371826 |
| SPATA20 | -0.076888993 | 5.283349449 | -0.87241444 | 0.383446114 |
| B4GALNT3 | 0.155722471 | -0.584286256 | 0.872361337 | 0.383475043 |
| UBE2D1 | 0.084833904 | 5.540759778 | 0.871770626 | 0.383796933 |
| TPSB2 | 0.238819865 | 4.246033548 | 0.871409062 | 0.383994039 |
| RAB31 | 0.16564064 | 6.168849378 | 0.870858017 | 0.384294558 |
| AC007238.1 | -0.032328906 | 6.756592704 | -0.87067619 | 0.384393751 |
| RP11-108K14.4 | -0.096672873 | 0.557923881 | -0.870416726 | 0.384535326 |
| ZNF765 | 0.041020559 | 2.988066473 | 0.870294169 | 0.384602209 |
| RP1-273G13.1 | 0.084154388 | 0.982625227 | 0.87018564 | 0.384661443 |
| C16orf80 | -0.051407136 | 6.283346045 | -0.870161012 | 0.384674886 |
| SFTPB | -0.14008549 | -2.95870132 | -0.870108703 | 0.384703438 |
| RP1-74M1.3 | -0.156678021 | -3.374886478 | -0.869266753 | 0.385163185 |
| GOLGA8Q | -0.08164002 | 3.720489724 | -0.869156495 | 0.385223416 |
| RPS23 | -0.060734967 | 8.849233745 | -0.869150946 | 0.385226448 |
| ST13P5 | -0.038039016 | 5.426722577 | -0.868682706 | 0.385482302 |
| TXNDC12 | -0.032211095 | 6.477643339 | -0.86862436 | 0.38551419 |
| RPS20P14 | -0.047580071 | 5.740419587 | -0.868557631 | 0.385550662 |
| ADAMTS7P4 | -0.114453576 | -2.749457723 | -0.868344751 | 0.385667031 |
| GLIS3 | 0.207659704 | -2.098535339 | 0.868240919 | 0.385723797 |
| USP13 | 0.063257946 | 3.030953721 | 0.868050828 | 0.385827736 |
| SRSF2 | -0.035123211 | 9.184591289 | -0.867964731 | 0.385874818 |
| MIF4GD | -0.039439001 | 6.168250431 | -0.86789453 | 0.385913209 |
| LIPH | 0.136685445 | -2.800658143 | 0.867417291 | 0.386174267 |
| CBWD1 | 0.054223883 | 3.200222196 | 0.867156843 | 0.386316781 |
| HFM1 | 0.16385497 | -3.045699311 | 0.866764906 | 0.386531306 |
| TVP23A | 0.115075885 | -3.321369753 | 0.866361991 | 0.386751916 |
| ACP5 | 0.116456816 | 3.644451693 | 0.866187034 | 0.386847735 |
| CTAGE7P | 0.082492577 | -0.575221817 | 0.866032007 | 0.386932651 |
| BMS1P16 | 0.096609929 | -0.165310603 | 0.865710826 | 0.387108614 |
| PNPLA3 | -0.122367493 | -2.818417236 | -0.86561844 | 0.387159238 |
| ZFAS1 | -0.062746157 | 9.072880619 | -0.865434328 | 0.387260136 |
| CORO1C | 0.053381831 | 6.810419291 | 0.865371232 | 0.387294718 |
| TNFRSF10B | 0.065868629 | 5.795217117 | 0.865328815 | 0.387317967 |
| FAHD2CP | 0.066710953 | 3.068377931 | 0.865192458 | 0.387392712 |
| IL12B | -0.145854727 | -2.742536977 | -0.865083289 | 0.387452559 |
| LRRC56 | -0.074812187 | 2.780825846 | -0.864910325 | 0.387547392 |
| AC027307.3 | 0.075492522 | 1.06865569 | 0.86489466 | 0.387555981 |
| QARS1 | -0.046326224 | 8.119473452 | -0.864750336 | 0.387635123 |
| HEATR6 | 0.039735351 | 4.62190858 | 0.864512715 | 0.387765446 |
| RP11-528A10.1 | -0.076919816 | 2.078806743 | -0.864451336 | 0.387799114 |
| RP11-511B23.1 | 0.112463896 | -1.303373245 | 0.864421362 | 0.387815556 |
| RP11-796E2.4 | -0.093468539 | -0.56182963 | -0.86417049 | 0.387953187 |
| SETD5-AS1 | 0.045222826 | 3.853756895 | 0.864155822 | 0.387961235 |
| ANKMY2 | -0.04180449 | 5.588846245 | -0.864104283 | 0.387989514 |
| FAM129B | 0.121240187 | 4.827236168 | 0.864100409 | 0.38799164 |
| ZNF662 | 0.161087946 | 1.874262732 | 0.864033653 | 0.38802827 |
| SIGLEC5 | -0.137813286 | 2.571886025 | -0.863753322 | 0.388182116 |
| SEPT7P6 | 0.052039102 | 5.24815964 | 0.863412104 | 0.388369427 |
| FAM157C | 0.130283344 | 3.243191933 | 0.862961935 | 0.388616631 |
| RP11-5N19.3 | 0.112797498 | -0.7408397 | 0.862548607 | 0.388843689 |
| RPL12P33 | 0.081157635 | 0.518567758 | 0.862385198 | 0.388933479 |
| FIGNL1 | 0.057211307 | 3.969028435 | 0.862359857 | 0.388947404 |
| MUC4 | -0.224820886 | -4.459446069 | -0.862209052 | 0.389030281 |
| CTC-248O19.1 | -0.104454004 | -0.364738699 | -0.862109557 | 0.389084966 |
| RBP4 | 0.148665439 | -1.239588784 | 0.862098995 | 0.389090771 |
| SLC25A23 | 0.115605925 | 2.133467755 | 0.861867032 | 0.389218283 |
| GNAO1 | 0.119160525 | -2.058871037 | 0.861717039 | 0.389300749 |
| TMEM255B | 0.153395746 | 0.182676649 | 0.861667893 | 0.389327772 |
| SUN1 | -0.061461919 | 4.36001854 | -0.861380389 | 0.389485877 |
| RP11-566K19.8 | -0.087830182 | -0.106147243 | -0.861356688 | 0.389498913 |
| IRX5 | -0.254490994 | 0.099222422 | -0.861153514 | 0.389610669 |
| C19orf38 | 0.127522959 | 6.474087971 | 0.861068167 | 0.38965762 |
| RP11-535M15.1 | -0.145370327 | -0.40873137 | -0.860501731 | 0.389969316 |
| COMMD2 | -0.026137953 | 5.023247795 | -0.860346914 | 0.390054534 |
| CTD-2622I13.3 | -0.093820671 | 1.475528596 | -0.860250905 | 0.390107388 |
| PPP2R5A | -0.043832961 | 6.659595157 | -0.860122358 | 0.39017816 |
| MALRD1 | -0.21488924 | -1.788716663 | -0.86006694 | 0.390208674 |
| CD248 | 0.164150755 | 0.847360298 | 0.85975947 | 0.390377994 |
| ASAP2 | -0.15043676 | 2.582360456 | -0.859170724 | 0.390702334 |
| GCSAML-AS1 | -0.180861732 | -2.080447269 | -0.858957042 | 0.390820092 |
| SLITRK6 | 0.152606892 | -3.601550186 | 0.85883685 | 0.390886339 |
| COX6A1P2 | -0.043957421 | 7.709916501 | -0.858685138 | 0.390969967 |
| SERPINE1 | 0.167790879 | 2.97102345 | 0.858421603 | 0.391115263 |
| PCDHGA5 | -0.151644148 | -1.963828887 | -0.858398554 | 0.391127972 |
| C10orf128 | -0.164012282 | 2.734674629 | -0.858012318 | 0.39134098 |
| U2SURP | 0.024562009 | 6.521538741 | 0.85758476 | 0.39157686 |
| RIBC2 | -0.134900215 | 1.385335264 | -0.857511871 | 0.39161708 |
| SNHG8 | -0.060799048 | 7.910689909 | -0.857497242 | 0.391625153 |
| CTC-232P5.1 | 0.083778659 | 1.402564615 | 0.857436134 | 0.391658875 |
| SLFN12L | -0.097676441 | 2.515929812 | -0.85737599 | 0.391692067 |
| SEPW1 | -0.042701006 | 4.698395908 | -0.857362059 | 0.391699756 |
| CGN | -0.13039875 | -2.842527997 | -0.857270693 | 0.391750183 |
| IL15 | 0.158534932 | 0.14776521 | 0.857207249 | 0.3917852 |
| AC018738.2 | 0.191574496 | 5.524437191 | 0.857025751 | 0.39188539 |
| RP11-25I15.1 | 0.063522122 | 1.862009837 | 0.857012162 | 0.391892892 |
| CTAGE5 | -0.0523504 | 0.863277781 | -0.855995105 | 0.392454616 |
| LPCAT3 | -0.048225986 | 5.775511169 | -0.855561789 | 0.392694086 |
| HIST1H2BC | 0.118178282 | 5.94811983 | 0.855467007 | 0.392746478 |
| ATOX1 | -0.038364232 | 4.230692861 | -0.855215973 | 0.392885263 |
| CTD-3195I5.5 | 0.059996069 | 3.604614005 | 0.855128088 | 0.392933858 |
| LRRC8C | -0.07008587 | 4.636599582 | -0.854968271 | 0.393022235 |
| RPL13AP7 | 0.047549196 | 5.37504996 | 0.854831198 | 0.393098045 |
| PTPRS | 0.171321322 | 1.325227206 | 0.854754961 | 0.393140213 |
| ATP1B1P1 | 0.08235686 | -0.297556083 | 0.854237936 | 0.393426258 |
| RP11-113C12.2 | 0.085913434 | 0.679709036 | 0.853189183 | 0.394006872 |
| RP11-576C2.1 | -0.162573569 | -0.077512282 | -0.853105949 | 0.394052975 |
| RP11-173E2.1 | -0.051875515 | 3.076945542 | -0.852920302 | 0.394155815 |
| CTD-2184C24.2 | 0.083702951 | -0.305922513 | 0.852768293 | 0.394240033 |
| IGHV3-33 | 0.169934073 | 2.770518931 | 0.852282142 | 0.39450945 |
| RBM8B | -0.032545808 | 7.057382206 | -0.85188747 | 0.394728254 |
| EMC6 | -0.030820875 | 6.350462912 | -0.85135048 | 0.395026075 |
| RPL9P7 | 0.06493607 | 4.604515331 | 0.851305963 | 0.395050771 |
| TNNC1 | 0.094766941 | -0.917624485 | 0.850997566 | 0.395221881 |
| TPD52 | 0.116228767 | 2.439510724 | 0.850941229 | 0.395253143 |
| SLC4A11 | -0.150113267 | -0.296107887 | -0.850696439 | 0.395388999 |
| IKBKGP1 | 0.074180712 | 2.37704681 | 0.850546535 | 0.395472208 |
| GTF3C5 | -0.048389498 | 6.226741709 | -0.85052306 | 0.395485241 |
| RPS15AP11 | -0.038433398 | 6.025986971 | -0.850337342 | 0.395588347 |
| RP11-34F13.2 | 0.166705496 | -0.716772824 | 0.849454762 | 0.396078555 |
| RP11-54O7.16 | -0.158252554 | 0.549133342 | -0.849413914 | 0.396101252 |
| PDE6B | -0.076581352 | 2.047528043 | -0.84938558 | 0.396116996 |
| SFXN2 | -0.067910586 | 2.325924136 | -0.848815127 | 0.396434056 |
| RP4-798A10.7 | -0.099620958 | 0.120152322 | -0.848773153 | 0.396457391 |
| MMP7 | -0.165293663 | -0.464449076 | -0.848626204 | 0.396539094 |
| HP | 0.203660112 | 3.810131364 | 0.848603029 | 0.39655198 |
| SMOC1 | 0.128322095 | -3.152909631 | 0.848602138 | 0.396552475 |
| RP5-934G17.6 | -0.079258646 | -0.621728409 | -0.848595445 | 0.396556197 |
| TTC12 | -0.123288056 | 1.42806236 | -0.848439941 | 0.396642669 |
| ANKRD6 | -0.075540477 | 2.621484024 | -0.848393558 | 0.396668464 |
| RP11-388M20.6 | -0.109709746 | -1.042539977 | -0.848267152 | 0.396738767 |
| ERG | -0.146046795 | 5.012812692 | -0.847837762 | 0.396977635 |
| AQPEP | -0.116129128 | -2.232692498 | -0.847466419 | 0.397184283 |
| RP11-14K2.1 | 0.075216485 | 0.711461402 | 0.84745294 | 0.397191785 |
| CLEC11A | -0.17963771 | 8.542186627 | -0.847062252 | 0.397409271 |
| FAM218A | -0.107401784 | -2.333333563 | -0.846641444 | 0.397643604 |
| PEA15 | -0.06583129 | 6.765289778 | -0.846611804 | 0.397660112 |
| RP3-395M20.9 | 0.100209222 | 0.559797093 | 0.846108755 | 0.397940359 |
| ZNF7 | -0.034706872 | 3.258989272 | -0.846028543 | 0.397985056 |
| NXF1 | 0.041003654 | 7.820626685 | 0.845471767 | 0.398295394 |
| HEPH | 0.1328205 | -2.875582322 | 0.845385254 | 0.398343628 |
| TCTEX1D2 | -0.086673809 | 0.203354229 | -0.845355212 | 0.398360379 |
| RPL7AP66 | 0.039605606 | 5.149640608 | 0.844535742 | 0.398817449 |
| HLA-DMA | 0.108157621 | 7.607818992 | 0.844476639 | 0.398850427 |
| RP11-114H24.2 | -0.132476288 | -2.115768753 | -0.844316857 | 0.398939589 |
| AC000120.7 | 0.073456756 | 1.88072638 | 0.843384512 | 0.399460098 |
| RPL5P1 | -0.045240859 | 7.218207403 | -0.843206687 | 0.399559421 |
| APOBEC3H | -0.104533842 | 2.915000135 | -0.843031867 | 0.39965708 |
| NEIL3 | 0.140789228 | 2.387573603 | 0.842948249 | 0.399703796 |
| RP11-73M18.10 | 0.086863572 | 0.136870785 | 0.842555457 | 0.399923287 |
| RP11-303G3.6 | -0.103851348 | 0.44585421 | -0.842552746 | 0.399924802 |
| AC013733.5 | 0.086967995 | -0.204664741 | 0.842490284 | 0.399959713 |
| AC007690.1 | 0.083826302 | 0.688937348 | 0.842458678 | 0.399977378 |
| CT45A5 | 0.196827665 | -1.957131559 | 0.841875205 | 0.400303582 |
| ZNF75A | 0.044450622 | 3.878304873 | 0.841802082 | 0.400344475 |
| LENG8 | -0.053161199 | 8.637730809 | -0.84174052 | 0.400378904 |
| PMS2P10 | -0.089139678 | -0.565718701 | -0.841326001 | 0.400610774 |
| CTD-2666L21.3 | 0.079730745 | 0.447875414 | 0.841273584 | 0.400640101 |
| MIR5010 | -0.101451709 | 0.467118388 | -0.841263476 | 0.400645756 |
| PSMD10 | 0.030878752 | 5.514947463 | 0.841185133 | 0.400689591 |
| RP11-23J18.1 | 0.12376522 | 1.497084226 | 0.840752629 | 0.400931636 |
| IGHV3-62 | 0.101138865 | -0.712152088 | 0.84059151 | 0.401021827 |
| SIMC1 | -0.105516031 | 3.011318953 | -0.840524323 | 0.40105944 |
| RBMX2P5 | -0.048437237 | 2.805995174 | -0.840490821 | 0.401078197 |
| CNTNAP1 | -0.11591924 | 0.457308436 | -0.840256397 | 0.401209455 |
| LZTS2 | 0.066648827 | 5.132628989 | 0.839821861 | 0.401452828 |
| GAREM | -0.133605508 | -3.339824505 | -0.839756313 | 0.401489547 |
| PLA2G1B | 0.121859419 | -0.06396106 | 0.839560292 | 0.401599369 |
| AC012066.1 | -0.0657619 | 3.778555922 | -0.839496192 | 0.401635286 |
| TOM1L1 | -0.217444946 | -0.943827807 | -0.839179764 | 0.401812614 |
| GCA | -0.098173685 | 6.58566914 | -0.83895506 | 0.401938567 |
| PDXK | 0.070911809 | 4.59733541 | 0.838703119 | 0.402079817 |
| SH2D6 | 0.089767925 | -2.44149101 | 0.838432659 | 0.402231482 |
| RP11-752G15.6 | 0.053177423 | 2.24043899 | 0.838200097 | 0.402361923 |
| PNMAL1 | 0.219892169 | -2.286970422 | 0.838091203 | 0.402423009 |
| TENM4 | -0.204707469 | -4.297755569 | -0.837275407 | 0.402880821 |
| HSPE1P3 | 0.059910646 | 2.028797039 | 0.836740979 | 0.403180903 |
| TIAM1 | 0.136383817 | 1.597660524 | 0.836586441 | 0.403267702 |
| ZNF257 | -0.150704228 | 0.716590986 | -0.836274602 | 0.403442885 |
| SAFB | -0.028048644 | 6.50758727 | -0.836182237 | 0.403494781 |
| NPLOC4 | 0.025324914 | 6.459437374 | 0.835458368 | 0.40390164 |
| HOXA6 | -0.264327243 | 3.031976798 | -0.835267281 | 0.404009083 |
| RP11-206L10.3 | 0.091919614 | -0.069772424 | 0.835214805 | 0.404038592 |
| PDIA3P2 | 0.082457674 | 1.09280326 | 0.835064489 | 0.404123127 |
| MTND4P14 | 0.103297285 | 1.358872842 | 0.83489842 | 0.404216533 |
| MIS12 | -0.038218402 | 5.133027742 | -0.834861383 | 0.404237366 |
| TRIM15 | -0.172517326 | -1.73083606 | -0.834775171 | 0.404285863 |
| POU2AF1 | -0.152921172 | 0.570699864 | -0.834771 | 0.40428821 |
| TRBV28 | -0.166215994 | 3.494246307 | -0.834710913 | 0.404322013 |
| ST3GAL2 | 0.052173307 | 5.194816325 | 0.834663503 | 0.404348685 |
| POLR1E | -0.041721104 | 5.395817605 | -0.834544696 | 0.40441553 |
| L3MBTL4 | 0.209206296 | -0.179837506 | 0.834465377 | 0.404460161 |
| ACTN4P1 | -0.044026755 | 3.714266894 | -0.83444878 | 0.4044695 |
| DKC1 | 0.041202136 | 6.660575099 | 0.834157691 | 0.404633317 |
| CYCSP55 | 0.082051354 | 1.195043179 | 0.834109154 | 0.404660636 |
| SCN4B | 0.145368905 | -1.50940699 | 0.834096232 | 0.40466791 |
| PRPF8 | -0.041306494 | 8.058185056 | -0.833701483 | 0.404890139 |
| RP11-544M22.13 | -0.102260511 | -0.774211046 | -0.833239681 | 0.405150209 |
| AMER1 | 0.048810801 | 3.077617569 | 0.832745711 | 0.405428506 |
| PIWIL3 | -0.128436061 | -3.259106246 | -0.832632419 | 0.40549235 |
| PRSS30P | -0.127547231 | -0.423141057 | -0.832378079 | 0.405635701 |
| TIE1 | -0.165724311 | 2.906797966 | -0.832368783 | 0.405640941 |
| RP11-1260E13.4 | -0.14953623 | -2.081640497 | -0.832325175 | 0.405665522 |
| SAP30 | -0.090302872 | 6.160209457 | -0.832315998 | 0.405670695 |
| DDX50P2 | -0.068517533 | -0.073440767 | -0.831919497 | 0.405894242 |
| CENPI | 0.08254224 | 1.777291358 | 0.831526065 | 0.406116132 |
| VARS2 | 0.040014882 | 4.135584928 | 0.831352711 | 0.406213924 |
| RP11-298C3.2 | -0.058114991 | 6.495394951 | -0.831185112 | 0.406308483 |
| ZNRF2 | 0.035309627 | 5.379901833 | 0.830900848 | 0.406468895 |
| HSDL2-AS1 | -0.107491317 | -0.405189454 | -0.830572588 | 0.40665418 |
| CTD-2291D10.4 | 0.128924291 | 1.069551716 | 0.830532681 | 0.406676709 |
| AC009133.15 | -0.09317942 | 0.939491868 | -0.830274974 | 0.406822211 |
| TRBV5-6 | -0.116363024 | 0.122392267 | -0.830103503 | 0.406919042 |
| SLC45A1 | 0.100296502 | -2.114877354 | 0.830029486 | 0.406960844 |
| KYNU | 0.162035913 | 3.88870945 | 0.829684513 | 0.407155706 |
| DNAJC5B | -0.15898257 | -0.935176078 | -0.829563503 | 0.407224073 |
| MAPK8IP2 | 0.078395406 | -1.389602709 | 0.82914787 | 0.407458945 |
| ANKRD54 | 0.034427737 | 4.397069765 | 0.829023916 | 0.407529007 |
| DSE | -0.091846 | 3.914566936 | -0.828889577 | 0.407604947 |
| RP11-720N19.2 | 0.104194573 | -0.317718055 | 0.82860957 | 0.407763256 |
| RP11-93B14.4 | -0.100859091 | -1.257116319 | -0.828174691 | 0.408009201 |
| WDR5 | -0.031005637 | 5.842995654 | -0.828153836 | 0.408020998 |
| VAMP5 | 0.093878464 | 5.116495698 | 0.828127269 | 0.408036026 |
| STAG2 | 0.031717423 | 6.935933229 | 0.827904891 | 0.408161831 |
| EIF4BP6 | 0.046119302 | 8.374668497 | 0.827844939 | 0.408195751 |
| FAM110B | -0.180088251 | 0.734661552 | -0.827818362 | 0.408210789 |
| F5 | 0.167794123 | 3.844658186 | 0.826870324 | 0.408747415 |
| CTC-338M12.4 | 0.05941157 | 2.600874537 | 0.826450249 | 0.408985328 |
| RP11-704M14.1 | -0.247236049 | 1.240332169 | -0.826391483 | 0.409018617 |
| ASRGL1 | -0.11700297 | 3.571533118 | -0.826143059 | 0.409159361 |
| NDRG2 | 0.118487356 | 2.416643962 | 0.826123974 | 0.409170174 |
| CTC-463N11.1 | -0.058273593 | 1.202604577 | -0.825961142 | 0.409262443 |
| RSL24D1P8 | 0.076998699 | 0.685975147 | 0.825920069 | 0.409285718 |
| ZNHIT1 | 0.03100791 | 5.181461864 | 0.825848726 | 0.40932615 |
| LL22NC03-N14H11.1 | -0.154072383 | -1.377302763 | -0.825846876 | 0.409327198 |
| LEPREL1 | 0.145671522 | -2.187541968 | 0.825627992 | 0.40945126 |
| TNRC18P1 | 0.128480008 | -1.758213894 | 0.825552647 | 0.40949397 |
| ARL6IP1P2 | 0.080531716 | 0.32390355 | 0.825527706 | 0.409508109 |
| IGHV3-11 | 0.160730942 | 2.24217022 | 0.825047318 | 0.40978049 |
| R3HDM1 | -0.032863369 | 5.727654769 | -0.824944965 | 0.409838538 |
| AP003733.1 | -0.095968529 | 2.067211585 | -0.824260437 | 0.410226886 |
| SS18 | -0.028827561 | 6.081638154 | -0.824211905 | 0.410254428 |
| RPS18P6 | 0.091680271 | 0.677891322 | 0.824201615 | 0.410260268 |
| HAUS4 | 0.047208959 | 5.115050614 | 0.823623973 | 0.410588164 |
| MFAP5 | 0.144293879 | -3.202726941 | 0.823120923 | 0.410873846 |
| RAB3C | -0.150965981 | -2.332097369 | -0.823012961 | 0.410935172 |
| ARSB | -0.061773723 | 4.655705453 | -0.822924235 | 0.410985576 |
| STX5 | 0.024267465 | 6.615461733 | 0.822713563 | 0.411105272 |
| AC093415.2 | 0.119994398 | -0.754365812 | 0.822313992 | 0.411332348 |
| RP11-367B6.2 | 0.10565324 | 0.029407153 | 0.822236207 | 0.411376562 |
| PRKY | 0.265165069 | 2.224999304 | 0.822077336 | 0.411466875 |
| CAAP1 | 0.025591079 | 4.998180539 | 0.821852929 | 0.411594463 |
| RP11-342I1.2 | 0.104986649 | 0.398962658 | 0.821806394 | 0.411620924 |
| C12orf45 | -0.038225926 | 4.282429532 | -0.82153689 | 0.411774189 |
| RP11-126L15.4 | -0.093673554 | 0.263065077 | -0.8214544 | 0.411821107 |
| PPP2R2C | -0.132412304 | -5.020568782 | -0.820897669 | 0.412137846 |
| ZNF675 | 0.04846553 | 3.540436071 | 0.820826516 | 0.412178337 |
| SLC12A4 | -0.059816672 | 4.137099668 | -0.820296637 | 0.412479952 |
| CBWD2 | -0.039560038 | 3.808651152 | -0.82012267 | 0.412579005 |
| C10ORF68 | 0.080883791 | 0.900119796 | 0.820111384 | 0.412585431 |
| IGLV7-46 | 0.154973861 | 1.432673766 | 0.820103626 | 0.412589849 |
| GPR126 | 0.231620912 | -1.129981213 | 0.819840587 | 0.412739646 |
| ANTXRL | 0.127895114 | -2.334015226 | 0.819728398 | 0.412803546 |
| AC002116.7 | 0.062423959 | 1.411222053 | 0.81961372 | 0.41286887 |
| RP11-231C14.4 | 0.060124837 | 3.110560587 | 0.819613637 | 0.412868918 |
| TLN2 | -0.105686501 | 0.601819614 | -0.81945679 | 0.412958272 |
| RPS2P35 | 0.096265253 | -0.521079572 | 0.81939725 | 0.412992195 |
| ZFYVE27 | 0.031694244 | 5.610643794 | 0.818969505 | 0.413235949 |
| AC010980.2 | -0.139407903 | -1.089995833 | -0.818888302 | 0.413282232 |
| RNF40 | -0.025235247 | 6.721749073 | -0.818364646 | 0.413580777 |
| GCAT | 0.100378632 | 3.521942724 | 0.81807667 | 0.413745012 |
| TTC28 | -0.109301751 | 3.011914929 | -0.818027111 | 0.41377328 |
| RASSF6 | -0.147819842 | -2.246199814 | -0.817885095 | 0.41385429 |
| APBA3 | -0.032736603 | 3.643991792 | -0.817679686 | 0.413971478 |
| AC008964.1 | -0.112176396 | -0.606696657 | -0.817603289 | 0.414015069 |
| IGSF9 | 0.132326661 | -1.923374011 | 0.817583408 | 0.414026413 |
| PFDN2 | -0.037557291 | 7.083512422 | -0.817549994 | 0.414045479 |
| KCNN3 | 0.109770945 | -3.171328151 | 0.817328491 | 0.414171884 |
| EID2 | 0.032376594 | 4.894531205 | 0.816940155 | 0.414393552 |
| PLEKHG6 | 0.133109894 | -2.707797803 | 0.816697664 | 0.414532005 |
| RP11-345J4.6 | -0.085588704 | 1.62396405 | -0.816385473 | 0.414710295 |
| GATM | -0.173661446 | 1.060540745 | -0.816116351 | 0.414864024 |
| ZFY-AS1 | 0.135643824 | -0.899487613 | 0.815852775 | 0.415014619 |
| GATA2 | -0.163213446 | 6.306847438 | -0.815759774 | 0.415067762 |
| WI2-1896O14.1 | 0.118606441 | -0.814396787 | 0.815351573 | 0.41530107 |
| AC131097.3 | -0.085958982 | 2.997483106 | -0.814631245 | 0.415712964 |
| DYNLT3P1 | 0.084420668 | 0.125540259 | 0.81430133 | 0.415901694 |
| AHCYP1 | -0.093279043 | -0.965564644 | -0.81369306 | 0.416249794 |
| STARD7-AS1 | -0.034144918 | 3.644133517 | -0.813314476 | 0.416466535 |
| AC005077.14 | -0.172388946 | -1.672915775 | -0.813129154 | 0.416572658 |
| CRISP2 | 0.162303374 | -0.955013742 | 0.812395857 | 0.41699273 |
| TMEM86A | -0.092424672 | 0.687662019 | -0.811499691 | 0.417506441 |
| ISM1 | -0.112721207 | -1.342532064 | -0.81078619 | 0.417915711 |
| RP11-539L10.2 | 0.105961743 | 1.522604777 | 0.810148218 | 0.418281857 |
| PCDHGC4 | 0.123595116 | -3.106090338 | 0.810115088 | 0.418300876 |
| GPBP1 | -0.022680829 | 6.646931866 | -0.809386753 | 0.418719126 |
| NUDT21 | 0.026827492 | 6.9366859 | 0.808146929 | 0.419431669 |
| SH3BGRL3 | -0.042544798 | 10.42791992 | -0.807975591 | 0.419530196 |
| CTD-2015H6.3 | -0.109851917 | 1.604460058 | -0.807823778 | 0.419617505 |
| IGHV4-59 | 0.14078607 | 2.554293042 | 0.807410767 | 0.419855089 |
| ITGA7 | -0.135911567 | 2.222862188 | -0.807354608 | 0.419887401 |
| CYTL1 | -0.204091608 | 5.234883404 | -0.807333155 | 0.419899744 |
| MT1P1 | -0.112464166 | 0.409957462 | -0.807325486 | 0.419904157 |
| RIMBP2 | -0.134124251 | -5.15103992 | -0.806881537 | 0.420159644 |
| C11orf83 | -0.037994732 | 3.831924836 | -0.80685369 | 0.420175672 |
| RP11-887P2.6 | 0.099660757 | -0.700453425 | 0.806576028 | 0.420335513 |
| RBMS2P1 | 0.073655419 | 4.395205049 | 0.806361782 | 0.420458872 |
| AP000695.4 | 0.13076342 | -0.730345485 | 0.80627054 | 0.420511414 |
| PROK2 | 0.176316773 | 5.841759047 | 0.805959724 | 0.420690427 |
| CEP128 | 0.049788586 | 3.154784848 | 0.805939932 | 0.420701828 |
| PRKG2 | -0.146535031 | -0.561541733 | -0.805743627 | 0.420814914 |
| KIAA1377 | -0.125859385 | -0.787356289 | -0.805239948 | 0.421105151 |
| SRPK3 | 0.15485061 | -1.700109546 | 0.804513903 | 0.421523732 |
| RP11-509J21.3 | -0.082984807 | 2.243939 | -0.804093642 | 0.421766133 |
| TPI1P2 | 0.061459792 | 1.571541163 | 0.803672056 | 0.42200938 |
| NLRP1 | -0.086216013 | 5.534822767 | -0.803625783 | 0.422036084 |
| CDK18 | -0.116261489 | 2.114722509 | -0.803578176 | 0.422063559 |
| PSMA2P3 | -0.079899347 | -0.350842031 | -0.803453505 | 0.422135513 |
| XXYLT1-AS2 | 0.184603105 | 0.882570212 | 0.803375491 | 0.422180543 |
| SIGLEC10 | -0.108750874 | 5.191840951 | -0.803225549 | 0.422267097 |
| RP1-224A6.9 | -0.080812456 | 0.358828547 | -0.803087786 | 0.42234663 |
| KLK10 | 0.10506651 | -4.178326258 | 0.802706751 | 0.422566656 |
| ARHGAP11B | 0.055590191 | 3.612424821 | 0.802677284 | 0.422583673 |
| TSHZ2 | 0.143466402 | -1.618942594 | 0.802562687 | 0.422649861 |
| GABBR1 | -0.147419856 | 4.014885582 | -0.802525113 | 0.422671564 |
| CTDP1 | -0.043130682 | 5.660736409 | -0.802207808 | 0.422854867 |
| MID2 | 0.134862639 | 0.215762695 | 0.801418367 | 0.423311119 |
| ZNF506 | -0.056599438 | 3.66913846 | -0.801113833 | 0.423487199 |
| RP11-169K16.8 | -0.04031614 | 7.447405267 | -0.800088072 | 0.424080607 |
| COL14A1 | 0.164933357 | -0.889993294 | 0.799899401 | 0.424189807 |
| HSF5 | 0.102345738 | 0.70043608 | 0.799650058 | 0.424334148 |
| SBK1 | 0.107673966 | 1.37873062 | 0.799600389 | 0.424362905 |
| C17orf104 | 0.144957258 | -2.270505914 | 0.799544054 | 0.424395522 |
| FAM27E3 | 0.098461291 | -1.353789909 | 0.799442732 | 0.424454189 |
| AL591479.1 | -0.09771045 | 0.846045536 | -0.798999991 | 0.4247106 |
| EGLN1 | -0.100245988 | 4.977532128 | -0.798815825 | 0.424817285 |
| RP11-547D23.1 | -0.083152997 | -0.440824526 | -0.798619976 | 0.424930756 |
| DDOST | -0.038069784 | 9.000511964 | -0.79861465 | 0.424933842 |
| HK1 | -0.06094762 | 6.716541109 | -0.798473262 | 0.42501577 |
| RP11-428L21.2 | -0.090166207 | 0.947577156 | -0.798005312 | 0.425286994 |
| STK32B | 0.176958673 | 2.406878975 | 0.797920936 | 0.425335909 |
| MSLN | 0.280725685 | -0.062082783 | 0.797745589 | 0.425437573 |
| ZEB2-AS1 | 0.109507695 | 2.077430995 | 0.797618602 | 0.425511207 |
| RP11-727A23.5 | -0.049331836 | 2.075259899 | -0.797548946 | 0.425551601 |
| RP13-638C3.4 | -0.087538167 | 0.702794898 | -0.796593442 | 0.426105927 |
| RP11-68L18.1 | 0.112879199 | -0.942650109 | 0.796554648 | 0.426128441 |
| CWC27 | -0.029224733 | 5.276749688 | -0.796390198 | 0.426223891 |
| AC115618.1 | -0.074911324 | -0.122771704 | -0.796078171 | 0.426405031 |
| CELSR1 | -0.128591123 | 0.926112457 | -0.795922635 | 0.42649534 |
| CSNK1A1L | 0.063772448 | 1.427163908 | 0.795897471 | 0.426509953 |
| ADAMTS3 | 0.212507711 | -1.14702377 | 0.79581751 | 0.426556386 |
| SNRNP200 | 0.031469961 | 7.554755638 | 0.795392759 | 0.426803089 |
| RP11-459O1.2 | -0.157272997 | -0.51935702 | -0.795227849 | 0.426898894 |
| PLEKHG5 | 0.134419314 | -0.301551448 | 0.795184856 | 0.426923873 |
| ANKRD13B | -0.085152195 | 3.01551813 | -0.795183226 | 0.426924821 |
| AP001437.1 | 0.067665109 | 2.533289368 | 0.794784279 | 0.427156651 |
| RP13-314C10.5 | 0.092222467 | -0.309652068 | 0.794488253 | 0.427328721 |
| LPP | 0.045068376 | 4.876589205 | 0.794417722 | 0.427369724 |
| RP11-137H2.4 | 0.130579751 | 1.47286679 | 0.793986732 | 0.42762033 |
| CYP2R1 | -0.040807883 | 3.912538052 | -0.793811294 | 0.427722366 |
| RPL12 | 0.052066496 | 9.240965845 | 0.793734478 | 0.427767047 |
| SERPINA1 | -0.188817887 | 7.042393699 | -0.793691522 | 0.427792034 |
| PGM5 | -0.176708387 | -2.36723263 | -0.793147467 | 0.428108581 |
| ZBTB47 | -0.070549794 | 3.315027663 | -0.793001427 | 0.428193574 |
| LINC01125 | -0.059124698 | 1.751135388 | -0.792878763 | 0.428264971 |
| PECR | 0.144103539 | 1.696786647 | 0.792431462 | 0.428525382 |
| NAP1L1 | -0.0499574 | 8.592311696 | -0.792412959 | 0.428536156 |
| AGAP7 | -0.115936762 | 2.055556281 | -0.79232708 | 0.428586164 |
| ACTG1P1 | -0.065847661 | 1.177901629 | -0.792080886 | 0.428729545 |
| RP11-460N11.2 | 0.121745002 | -2.797029416 | 0.791876375 | 0.428848671 |
| HTR7 | -0.180685723 | 1.241054551 | -0.791874125 | 0.428849982 |
| MTATP6P2 | -0.102560315 | 0.519738765 | -0.791865483 | 0.428855016 |
| NIPAL1 | -0.095334482 | -2.689439718 | -0.791705707 | 0.428948099 |
| FKBP1B | 0.109283622 | 0.376888262 | 0.791606621 | 0.429005831 |
| RCAN3 | -0.070185213 | 4.546291067 | -0.791413262 | 0.429118503 |
| RP11-153M7.1 | -0.057379593 | 2.403414237 | -0.791363163 | 0.429147699 |
| FRMD8 | 0.030308382 | 5.778809631 | 0.791119991 | 0.429289428 |
| ADAM19 | 0.114830253 | 3.26186902 | 0.791041116 | 0.429335405 |
| SGOL1-AS1 | 0.090707947 | 0.032191544 | 0.789876947 | 0.430014342 |
| EIF4G2 | 0.031099504 | 9.386813755 | 0.78973951 | 0.430094537 |
| VENTX | 0.185629085 | 2.739476196 | 0.789188504 | 0.430416133 |
| RP11-705C15.2 | 0.063366514 | 4.146975809 | 0.789078138 | 0.430480566 |
| RNVU1-15 | -0.14491932 | 0.844226515 | -0.788673062 | 0.4307171 |
| NRROS | -0.059683005 | 6.659710002 | -0.78861286 | 0.43075226 |
| CACNA2D1 | 0.128744766 | -4.42322745 | 0.788117766 | 0.431041474 |
| GDAP1 | 0.057003688 | 2.907814088 | 0.787506773 | 0.431398548 |
| EPM2AIP1 | -0.035744201 | 5.552604326 | -0.787293261 | 0.431523368 |
| CITED2 | 0.075375196 | 9.210105015 | 0.78724202 | 0.431553327 |
| RUNDC3B | -0.123284619 | -3.164952422 | -0.786550742 | 0.431957612 |
| ZNF788 | 0.09405022 | -0.920779594 | 0.786387222 | 0.432053277 |
| SPRY3 | 0.058570389 | 1.121739464 | 0.786007612 | 0.432275409 |
| SERPINF1 | 0.161444636 | 2.965100491 | 0.78592182 | 0.432325621 |
| RP3-469D22.1 | -0.056455664 | 3.818539851 | -0.785811224 | 0.432390354 |
| CFB | -0.154981136 | -3.13762274 | -0.785125341 | 0.432791935 |
| FANCM | 0.029874075 | 3.018774487 | 0.784991339 | 0.432870418 |
| CMIP | 0.040875824 | 6.370747944 | 0.784733137 | 0.433021666 |
| IGHV1-69 | 0.162616797 | 2.723194295 | 0.784526536 | 0.433142709 |
| DOCK1 | 0.259228536 | 1.954319848 | 0.784448763 | 0.43318828 |
| PA2G4 | -0.036520306 | 7.026503964 | -0.784182848 | 0.433344113 |
| SCGB3A1 | 0.122227929 | 0.948701272 | 0.784134291 | 0.433372572 |
| CLSTN2 | 0.257326436 | -2.349511333 | 0.783446907 | 0.433775563 |
| SULT1C2 | -0.117583916 | -4.898053183 | -0.782935711 | 0.4340754 |
| CCDC102A | -0.101321549 | 3.717690151 | -0.782772843 | 0.434170955 |
| RP11-770J1.5 | 0.099341958 | -1.925015216 | 0.782506492 | 0.434327248 |
| ZG16B | 0.129552571 | 2.509247018 | 0.78234323 | 0.434423066 |
| BICC1 | 0.177231962 | -1.954497828 | 0.782294766 | 0.434451512 |
| C2 | -0.172534574 | 0.745730407 | -0.782291996 | 0.434453138 |
| CTC-297N7.8 | -0.043396972 | 3.966461515 | -0.782247 | 0.434479548 |
| DEAF1 | 0.044742946 | 5.351581335 | 0.781985481 | 0.43463307 |
| SEMA4D | -0.042068087 | 5.831824597 | -0.781874737 | 0.434698091 |
| ARL4D | 0.080606548 | 1.894546563 | 0.781787827 | 0.434749121 |
| FAM92A1P1 | -0.166347241 | 2.064518547 | -0.781566863 | 0.434878879 |
| RASD1 | 0.150386554 | 3.112823592 | 0.781513162 | 0.434910418 |
| TRAF7 | -0.025074231 | 6.675839565 | -0.781492366 | 0.434922632 |
| LINC00426 | -0.110043358 | 2.468238354 | -0.781473273 | 0.434933846 |
| GPR137C | 0.088099401 | -1.199034791 | 0.781383843 | 0.434986373 |
| CTA-984G1.5 | -0.137400563 | 2.085563884 | -0.781369234 | 0.434994954 |
| DMAP1 | -0.027311276 | 5.465080431 | -0.780293356 | 0.435627169 |
| MIR4489 | 0.087622837 | 0.220001348 | 0.780216841 | 0.435672151 |
| RP11-49C9.2 | 0.052665949 | 1.872524237 | 0.780136749 | 0.435719239 |
| CMSS1 | 0.049979825 | 3.954041238 | 0.779168547 | 0.436288705 |
| CTC-359D24.3 | 0.101855695 | 0.480272539 | 0.778989336 | 0.436394158 |
| RP11-109L13.1 | 0.075680571 | 2.049255872 | 0.778580687 | 0.436634675 |
| RP11-85G21.3 | 0.168529685 | -0.066229282 | 0.77838251 | 0.436751343 |
| GPRC5C | 0.113816753 | 0.139391158 | 0.778171325 | 0.436875689 |
| AL139099.1 | 0.069901061 | 0.496259429 | 0.778090328 | 0.436923385 |
| AC005884.1 | 0.040655102 | 5.308421544 | 0.778073745 | 0.43693315 |
| RP11-320L11.2 | -0.048774999 | 2.477624158 | -0.777962003 | 0.436998957 |
| BNIP3P1 | -0.077826426 | 2.774813313 | -0.777250008 | 0.437418398 |
| IL32 | -0.115761607 | 4.000117707 | -0.777185839 | 0.437456212 |
| BSPRY | 0.097993157 | 5.125436657 | 0.776836292 | 0.437662228 |
| C20orf201 | -0.085540275 | 1.684670929 | -0.776489974 | 0.437866396 |
| RP11-407N17.5 | 0.061030054 | 2.211900745 | 0.77643496 | 0.437898834 |
| PDCD5 | -0.032835802 | 5.837347066 | -0.775957454 | 0.438180445 |
| GABRD | -0.146600036 | -1.470536468 | -0.775827148 | 0.438257311 |
| RP4-612B15.3 | -0.042840147 | 2.543380929 | -0.775795032 | 0.438276257 |
| ZNF823 | -0.065994551 | 2.57280677 | -0.775574532 | 0.43840635 |
| C1orf132 | 0.074783552 | 2.122344489 | 0.775507041 | 0.438446173 |
| ACSL6 | 0.136212635 | -0.66970055 | 0.775377008 | 0.438522905 |
| LHPP | 0.050953745 | 3.98074765 | 0.775363847 | 0.438530672 |
| QPRT | -0.102402042 | 4.065967333 | -0.775282702 | 0.43857856 |
| ERVMER34-1 | -0.101049179 | -3.613993419 | -0.774917002 | 0.438794416 |
| RP11-4F5.2 | -0.096036778 | 5.045084557 | -0.77468888 | 0.438929097 |
| CHD5 | -0.143965792 | -0.798179036 | -0.774441674 | 0.439075072 |
| RP1-80N2.2 | -0.116086573 | 0.725436364 | -0.774113077 | 0.439269152 |
| SPATA5 | -0.041774762 | 2.789522788 | -0.773934893 | 0.439374414 |
| GOLGA8UP | -0.091301554 | -0.064597763 | -0.773859404 | 0.439419014 |
| AKAP6 | -0.130491344 | -2.366930957 | -0.773851578 | 0.439423637 |
| RPL10AP2 | 0.039967827 | 5.421716385 | 0.773815389 | 0.439445019 |
| RP11-124N2.1 | -0.085401262 | -0.300777106 | -0.773531142 | 0.439612983 |
| TSPEAR-AS1 | -0.151044618 | -1.505999245 | -0.773455677 | 0.439657582 |
| ZBED6 | 0.044303482 | 4.497123081 | 0.773138007 | 0.43984535 |
| ABHD8 | -0.046586813 | 4.827270059 | -0.772990372 | 0.43993263 |
| SMYD3 | -0.091287572 | 4.239808672 | -0.772861826 | 0.440008633 |
| MYL6P2 | 0.05879125 | 2.601424029 | 0.772289981 | 0.440346828 |
| NCAM1 | -0.183679362 | 1.093722358 | -0.772176712 | 0.440413834 |
| TEX29 | 0.079688847 | 0.211766695 | 0.772114334 | 0.440450737 |
| FGGY | -0.057260251 | 1.908587576 | -0.772046313 | 0.440490981 |
| PUM1 | -0.022817608 | 6.607791401 | -0.771898254 | 0.440578586 |
| AC061992.2 | 0.107753221 | -0.303158469 | 0.771888365 | 0.440584437 |
| ZNF114 | -0.116153175 | 0.355783956 | -0.771854699 | 0.440604358 |
| RP11-168O16.1 | -0.099271257 | -1.961775675 | -0.771707421 | 0.440691514 |
| TNRC6C | -0.053464706 | 3.175429589 | -0.771321712 | 0.440919813 |
| PTK7 | -0.126948823 | 3.779610489 | -0.771304889 | 0.440929772 |
| RP11-102M11.1 | -0.037096203 | 5.796447938 | -0.771180866 | 0.441003196 |
| DDX11L2 | -0.112562975 | 2.665433837 | -0.771094122 | 0.441054555 |
| RP11-849H4.2 | -0.043079355 | 1.507278226 | -0.770908036 | 0.441164742 |
| RPL13P2 | 0.063300352 | 2.180058206 | 0.770574749 | 0.44136213 |
| RP11-598D12.3 | 0.09955775 | 2.124990507 | 0.770544315 | 0.441380158 |
| DNAH14 | -0.172258219 | -0.059794565 | -0.77031856 | 0.441513893 |
| GSAP | -0.077013695 | 3.991191076 | -0.770180333 | 0.441595788 |
| RP5-892K4.1 | -0.106221528 | -2.742270403 | -0.770072285 | 0.44165981 |
| GJC1 | -0.124261481 | -3.480363101 | -0.770043072 | 0.44167712 |
| PMPCB | 0.030335194 | 6.147065498 | 0.76991061 | 0.441755617 |
| VPS51 | 0.036541255 | 6.865585777 | 0.76984586 | 0.441793991 |
| SARM1 | -0.069819167 | 3.175696349 | -0.769822345 | 0.441807927 |
| PLSCR1 | 0.0630836 | 5.92421637 | 0.769515949 | 0.44198954 |
| SPINK2 | -0.210442647 | 6.108715126 | -0.769064526 | 0.442257194 |
| MIR181A1HG | 0.150691421 | 3.320119501 | 0.768327375 | 0.442694459 |
| RP11-254B13.4 | 0.065840781 | 1.075610508 | 0.766589245 | 0.443726469 |
| LNX1 | -0.132491964 | -2.124823327 | -0.766509558 | 0.443773816 |
| COMMD1 | -0.025982922 | 4.331272181 | -0.765998518 | 0.444077526 |
| PRKCSH | 0.029505617 | 8.357180638 | 0.765618819 | 0.444303257 |
| THAP2 | 0.060205315 | 2.891020083 | 0.765474822 | 0.44438888 |
| CTD-2031P19.4 | -0.034222908 | 5.831642366 | -0.765413658 | 0.444425252 |
| ALKBH8 | -0.036186221 | 3.55407215 | -0.765037885 | 0.444648748 |
| STK33 | 0.159857535 | -1.295411574 | 0.764698097 | 0.444850898 |
| PKN1 | 0.027129578 | 8.092121612 | 0.764668058 | 0.444868771 |
| RP11-490O6.2 | 0.078148303 | -1.118199675 | 0.764068203 | 0.445225778 |
| TRIM17 | 0.119361148 | -1.808320845 | 0.763879686 | 0.445338009 |
| ERCC5 | 0.02820424 | 4.668586837 | 0.763756589 | 0.445411302 |
| QTRT1 | 0.04608891 | 4.821489513 | 0.762571878 | 0.446117038 |
| RP11-217B7.3 | -0.093320566 | -0.697485197 | -0.762299359 | 0.44627947 |
| NBPF11 | 0.045019428 | 1.027689593 | 0.762161704 | 0.446361529 |
| SUGT1P | 0.102036907 | 2.991704634 | 0.762145375 | 0.446371264 |
| CD8B | 0.111139228 | 2.953319084 | 0.761368456 | 0.446834575 |
| IGHV4-28 | -0.099679904 | 0.161177583 | -0.761338083 | 0.446852694 |
| HLA-DPB1 | 0.129976383 | 8.218719897 | 0.761007123 | 0.447050147 |
| HIST1H1E | -0.117544068 | 4.379424725 | -0.760870827 | 0.447131477 |
| UPP2 | -0.123475261 | -2.050398324 | -0.760379113 | 0.447424961 |
| TPPP | -0.149694719 | 1.249153314 | -0.76017886 | 0.447544515 |
| ITPRIPL1 | 0.078917822 | 3.059806978 | 0.759715671 | 0.447821116 |
| ATP8B1 | -0.148231461 | -2.858988529 | -0.75963484 | 0.447869395 |
| RP11-589N15.2 | 0.102431796 | 1.554527657 | 0.759596715 | 0.447892168 |
| RP11-122G18.7 | 0.056845394 | 0.665265848 | 0.759375497 | 0.448024318 |
| CTSD | 0.063971739 | 9.500652144 | 0.758968991 | 0.448267213 |
| RPL7P48 | 0.084229037 | -0.686185841 | 0.758898524 | 0.448309326 |
| IGLV2-34 | 0.119614994 | 0.154359399 | 0.758350631 | 0.448636838 |
| CLIC2 | -0.132329078 | 3.061208582 | -0.758051997 | 0.448815409 |
| SLC2A3P2 | 0.087526868 | -0.288381829 | 0.757670372 | 0.449043664 |
| RP11-611O2.1 | -0.092967977 | 0.472787909 | -0.757229161 | 0.449307641 |
| RP11-106M3.1 | 0.071211418 | 2.547493996 | 0.757101879 | 0.44938381 |
| CTA-292E10.8 | -0.065247478 | 1.20923408 | -0.757097414 | 0.449386482 |
| GLDC | 0.111653099 | -2.776954813 | 0.756751978 | 0.449593239 |
| CTA-313A17.3 | -0.078078171 | 0.394806122 | -0.75662807 | 0.449667415 |
| AC011484.1 | -0.166073344 | 0.185959434 | -0.756301913 | 0.449862701 |
| CPS1 | 0.141779445 | -3.614175986 | 0.756122224 | 0.449970309 |
| RPL10A | 0.040809203 | 10.68913381 | 0.755554453 | 0.450310422 |
| EFNB1 | 0.082621134 | 3.722498332 | 0.755510974 | 0.450336473 |
| RP11-199F11.2 | -0.053307269 | 3.249567458 | -0.755187316 | 0.450530426 |
| AC090616.2 | 0.081129014 | -0.36187441 | 0.754794314 | 0.450765997 |
| HK3 | -0.171781044 | 6.373443197 | -0.754534788 | 0.450921599 |
| KCNQ1 | 0.115105032 | 4.130751895 | 0.753896239 | 0.451304579 |
| MAPK3 | 0.036348081 | 6.239377693 | 0.753810504 | 0.451356014 |
| RAVER1 | 0.027237878 | 5.25133332 | 0.753799574 | 0.451362571 |
| GPR132 | 0.078195808 | 4.195346 | 0.753789732 | 0.451368476 |
| TRAF3IP2-AS1 | 0.04414913 | 2.580146577 | 0.753544168 | 0.451515817 |
| SMPD2 | 0.03069622 | 5.044450243 | 0.753470964 | 0.451559746 |
| C19orf59 | 0.154392659 | 5.200966483 | 0.753156042 | 0.451748754 |
| PALLD | 0.12729258 | 1.186077388 | 0.75274162 | 0.451997548 |
| TRAP1 | 0.048518369 | 5.357877061 | 0.75252284 | 0.452128921 |
| IFITM2 | -0.073624375 | 9.265422778 | -0.752480123 | 0.452154575 |
| MESTP3 | 0.127658926 | 0.439771204 | 0.752190575 | 0.452328482 |
| RPL4P4 | 0.026438671 | 10.86062019 | 0.752135705 | 0.452361442 |
| ZNF124 | -0.051181942 | 2.451985191 | -0.752017117 | 0.452432682 |
| MAOB | 0.128251956 | -2.275915857 | 0.75187509 | 0.452518011 |
| MT1G | -0.15385114 | -0.352062006 | -0.751808636 | 0.452557939 |
| TMEM191A | -0.063060483 | 1.02929004 | -0.751628716 | 0.452666052 |
| C19orf71 | 0.075614134 | 3.219677271 | 0.751617414 | 0.452672843 |
| AGTPBP1 | 0.062935012 | 6.902611901 | 0.751271353 | 0.452880833 |
| SPDYE4 | -0.09783999 | -0.507320272 | -0.751252083 | 0.452892417 |
| SNORA71D | 0.082036331 | 0.096335345 | 0.751044934 | 0.453016945 |
| ABCD3 | -0.044438652 | 4.278066925 | -0.750981297 | 0.453055204 |
| UBE2Q1-AS1 | 0.100834368 | -0.64238373 | 0.750899664 | 0.453104285 |
| ADAMTS15 | 0.147025501 | -3.505364242 | 0.750884151 | 0.453113613 |
| ZFAND5 | -0.046561791 | 7.684085097 | -0.750121368 | 0.453572385 |
| FAM35A | 0.027658016 | 5.314161706 | 0.749824852 | 0.453750794 |
| CTD-2260A17.1 | 0.09542806 | 1.402790268 | 0.749813342 | 0.45375772 |
| CHL1 | 0.170091368 | -4.14505906 | 0.748976989 | 0.454261159 |
| RP3-452M16.1 | -0.069253328 | 3.123890994 | -0.748522554 | 0.454534837 |
| CSRP1 | 0.042221259 | 5.260083531 | 0.748335114 | 0.454647747 |
| AZU1 | 0.176993415 | 9.31321904 | 0.748223055 | 0.454715256 |
| CTSF | -0.103663466 | 4.090256497 | -0.748022738 | 0.454835951 |
| ZRSR2 | 0.037002256 | 5.816431807 | 0.747833307 | 0.454950104 |
| SIGMAR1 | -0.052117076 | 6.288139998 | -0.747795513 | 0.454972881 |
| MPZ | 0.056950435 | 3.838015133 | 0.747014965 | 0.455443429 |
| MPO | 0.189357069 | 10.28673285 | 0.746939859 | 0.45548872 |
| EXD3 | 0.052342908 | 2.78137235 | 0.746747184 | 0.455604922 |
| TDRD10 | 0.115580264 | -2.209636053 | 0.746477974 | 0.45576731 |
| TMED10P2 | 0.108551254 | 0.054740522 | 0.746343464 | 0.455848458 |
| ROGDI | -0.095763804 | 5.443435254 | -0.746100208 | 0.455995233 |
| C1orf228 | -0.094605898 | 7.166729042 | -0.745939105 | 0.456092454 |
| TNFRSF1B | 0.113541485 | 7.549507828 | 0.745743755 | 0.456210357 |
| H3F3AP6 | 0.040153269 | 4.892025545 | 0.745705377 | 0.456233522 |
| CHKB | -0.050249869 | 1.567947135 | -0.745664806 | 0.456258012 |
| AC069213.1 | -0.1039399 | -0.200319908 | -0.745547357 | 0.45632891 |
| L3MBTL1 | -0.04684625 | 2.831760625 | -0.745004404 | 0.456656747 |
| FAM92A1 | -0.177164696 | 0.477833624 | -0.744272643 | 0.457098796 |
| RPL6 | 0.036869098 | 8.662054626 | 0.744211792 | 0.457135567 |
| RP3-423B22.5 | 0.054255534 | 5.312874958 | 0.743723575 | 0.457430641 |
| RP11-392P7.6 | 0.147436813 | -2.601086603 | 0.743599181 | 0.457505841 |
| USP21 | -0.024520903 | 5.30427067 | -0.743529229 | 0.457548132 |
| DNASE1L1 | -0.037957658 | 5.682615429 | -0.742783062 | 0.457999382 |
| GPR27 | -0.149444795 | 5.072833273 | -0.742693331 | 0.458053664 |
| CRIM1 | 0.125733117 | 2.858661787 | 0.742593783 | 0.45811389 |
| RP11-713H12.1 | 0.09597517 | -0.706364754 | 0.742570473 | 0.458127993 |
| CBX3 | -0.026370697 | 7.085629835 | -0.742512433 | 0.458163109 |
| PROSER2-AS1 | -0.211225944 | -1.228131381 | -0.74223087 | 0.458333485 |
| AC005682.7 | 0.067355464 | 0.481373587 | 0.741851017 | 0.458563394 |
| LILRB3 | 0.149290429 | 2.870046101 | 0.741275872 | 0.458911629 |
| TLR1 | 0.081058074 | 4.704398753 | 0.741211786 | 0.458950441 |
| BTF3P10 | -0.041056624 | 4.808227771 | -0.741083203 | 0.459028319 |
| CITED4 | -0.071173719 | 7.148488391 | -0.740882783 | 0.45914972 |
| AC019129.1 | -0.056022964 | 1.036586444 | -0.740270524 | 0.459520698 |
| RP11-459I19.1 | -0.142526588 | -0.340974627 | -0.73963297 | 0.459907181 |
| TCTEX1D1 | 0.14197134 | 3.69640875 | 0.739616443 | 0.459917203 |
| RP11-497H16.2 | 0.154880378 | 1.876783775 | 0.739331307 | 0.460090113 |
| ZNF227 | -0.037771568 | 3.572002189 | -0.739183291 | 0.460179886 |
| RP11-61F12.1 | -0.115817625 | -1.575597166 | -0.738991313 | 0.460296336 |
| CRIP2 | -0.13421548 | 2.955935774 | -0.738942813 | 0.460325758 |
| GCH1 | 0.084305925 | 5.593316851 | 0.738872405 | 0.460368473 |
| CD79A | 0.095095973 | 5.166902883 | 0.738821242 | 0.460399513 |
| SAMD12 | -0.127537179 | -1.465590223 | -0.738206758 | 0.460772408 |
| RP11-2J18.1 | -0.090864524 | 0.843094576 | -0.737720939 | 0.461067344 |
| FAM184B | -0.095956786 | -3.523928675 | -0.737709888 | 0.461074054 |
| RANBP17 | 0.171676033 | -0.887523584 | 0.737389808 | 0.461268431 |
| PTCHD2 | -0.100086484 | -3.210952834 | -0.73701037 | 0.461498913 |
| FTLP2 | 0.070275192 | 2.782403141 | 0.736911294 | 0.461559106 |
| RPL34 | 0.049102144 | 8.000470169 | 0.736658357 | 0.461712795 |
| MAB21L3 | 0.11367362 | -2.032477332 | 0.736497896 | 0.461810308 |
| DNALI1 | 0.108850871 | -2.789009774 | 0.736355552 | 0.461896822 |
| ACTR5 | 0.041685452 | 4.892993497 | 0.73564092 | 0.462331297 |
| DRP2 | 0.082744825 | -2.067889812 | 0.735322178 | 0.462525157 |
| CTAGE13P | -0.07040604 | -0.506750907 | -0.735297649 | 0.462540077 |
| CCR1 | 0.151532322 | 4.631467348 | 0.7349872 | 0.46272894 |
| MATN4 | 0.143823628 | -1.322713861 | 0.734957961 | 0.46274673 |
| TBC1D1 | 0.041239711 | 6.502248662 | 0.734405813 | 0.463082745 |
| MTMR4 | -0.031660516 | 5.497953126 | -0.73417012 | 0.463226219 |
| CHST1 | 0.134625178 | -2.188341915 | 0.734109738 | 0.46326298 |
| DUSP6 | -0.089749708 | 7.733336975 | -0.734083753 | 0.463278801 |
| RP11-277L2.3 | 0.073098915 | 0.667154997 | 0.733449694 | 0.463664923 |
| IGHV3-74 | 0.128959691 | 2.447757889 | 0.733449336 | 0.463665141 |
| SLC2A10 | 0.14604459 | -0.653144397 | 0.733434204 | 0.463674358 |
| EPHX3 | 0.102648872 | -1.415289862 | 0.733283882 | 0.463765927 |
| WASF4P | 0.040164409 | 4.72625422 | 0.732995541 | 0.463941599 |
| ENTPD1 | 0.124058462 | 3.870453419 | 0.732857164 | 0.464025918 |
| AF064858.8 | -0.124271784 | 2.728206281 | -0.732343099 | 0.464339236 |
| RPL13P5 | -0.045213882 | 2.391678429 | -0.73228221 | 0.464376355 |
| DENND1C | -0.038062283 | 6.267133875 | -0.732160618 | 0.464450485 |
| SYDE1 | -0.145994637 | -1.889265107 | -0.731654877 | 0.464758886 |
| RP11-85K15.2 | 0.040879148 | 2.405316041 | 0.731538416 | 0.46482992 |
| snoU2_19 | 0.071228721 | 1.64516797 | 0.731484802 | 0.464862624 |
| HNRNPA1P10 | 0.04248495 | 9.943306413 | 0.731437883 | 0.464891244 |
| ZHX2 | 0.066468782 | 4.517065608 | 0.73112773 | 0.465080462 |
| RPL36AP13 | 0.074421595 | 0.739847079 | 0.731113421 | 0.465089192 |
| EHF | -0.100159975 | -3.918777837 | -0.731065406 | 0.465118489 |
| APTX | 0.020032912 | 4.614260095 | 0.730246109 | 0.465618555 |
| IGLV2-14 | 0.155080183 | 4.091522791 | 0.730227772 | 0.465629751 |
| CALCRL | 0.169452917 | 1.863187389 | 0.730156074 | 0.465673527 |
| IPCEF1 | 0.112357495 | 2.941007178 | 0.72986605 | 0.46585063 |
| PDPK2 | 0.060257441 | 0.06125063 | 0.729506584 | 0.466070189 |
| PLBD1 | 0.205025047 | 5.974245309 | 0.729348644 | 0.466166675 |
| DZANK1 | -0.052050517 | 1.578636086 | -0.729096573 | 0.466320691 |
| RP11-95P9.1 | 0.078650855 | -0.102995005 | 0.729076494 | 0.46633296 |
| TERF1P2 | -0.062856342 | -0.352671111 | -0.728939003 | 0.46641698 |
| AC079250.1 | 0.037195533 | 7.367335667 | 0.728812951 | 0.466494017 |
| IGFBP7 | -0.085945931 | 7.821169597 | -0.72867337 | 0.466579331 |
| NME2P1 | 0.04049876 | 3.6903374 | 0.728182261 | 0.466879572 |
| CNIH4 | 0.060716247 | 4.373168113 | 0.728177124 | 0.466882713 |
| ITSN2 | 0.024094891 | 6.241691721 | 0.728107027 | 0.466925576 |
| AP5Z1 | 0.028488056 | 5.29506552 | 0.727753609 | 0.467141718 |
| ROMO1 | -0.03392147 | 6.373369734 | -0.727460631 | 0.467320937 |
| TRAM1 | 0.03266754 | 8.329231206 | 0.727069771 | 0.467560093 |
| DBNDD1 | 0.109693939 | -0.340666525 | 0.726983321 | 0.467612999 |
| CAMKV | -0.162275736 | -0.517268594 | -0.726542598 | 0.467882764 |
| ULBP3 | -0.112200196 | 0.080947245 | -0.726429305 | 0.467952124 |
| SSTR2 | 0.134082262 | -2.253595701 | 0.726317904 | 0.468020331 |
| TPI1 | 0.036301001 | 8.494888885 | 0.725937348 | 0.468253375 |
| CRYL1 | -0.045349235 | 5.43061725 | -0.725485957 | 0.468529881 |
| DPP7 | 0.035826745 | 8.283196247 | 0.725110664 | 0.46875984 |
| IGHV1OR15-2 | 0.110532143 | 0.029386852 | 0.724838938 | 0.468926378 |
| TMEM104 | 0.063291229 | 4.931344611 | 0.724828255 | 0.468932927 |
| AP000695.1 | 0.102475753 | -2.050110981 | 0.724739263 | 0.468987477 |
| TLR6 | -0.079370589 | 3.740758888 | -0.724586513 | 0.469081117 |
| NPIPB5 | -0.039810341 | 5.027796388 | -0.724571647 | 0.469090231 |
| LDHAP4 | 0.060493455 | 5.565610045 | 0.724372128 | 0.469212559 |
| DRG2 | -0.024157382 | 4.358655097 | -0.724342239 | 0.469230886 |
| GTF2IRD2 | 0.049196902 | 1.570026939 | 0.724254574 | 0.469284641 |
| RP11-49O14.2 | -0.090211779 | 0.378265852 | -0.72278842 | 0.470184181 |
| COMTD1 | -0.05147377 | 4.049835487 | -0.7218724 | 0.470746678 |
| APOPT1 | -0.02682974 | 2.952663477 | -0.721651592 | 0.470882324 |
| TUBB4A | -0.137533387 | -0.262130797 | -0.721630153 | 0.470895496 |
| SP100 | -0.029507518 | 6.003001598 | -0.721398022 | 0.471038123 |
| AC073109.2 | 0.085565676 | -0.69324637 | 0.720900616 | 0.471343824 |
| LRRC25 | 0.128513694 | 6.030184717 | 0.720769074 | 0.471424687 |
| WWP2 | 0.029677285 | 5.540779423 | 0.720480568 | 0.471602068 |
| FAM66D | 0.093239199 | -0.164004519 | 0.720409336 | 0.471645868 |
| TTBK2 | -0.029217665 | 4.797634321 | -0.720200643 | 0.471774207 |
| PRKCDBP | 0.16655256 | -0.12331956 | 0.720003042 | 0.471895742 |
| TAF1D | 0.03892196 | 5.883596426 | 0.719955253 | 0.471925138 |
| ZNF415 | -0.124642568 | 0.566343951 | -0.718919927 | 0.472562225 |
| TSPAN7 | 0.197023974 | 0.275070508 | 0.71827978 | 0.472956377 |
| NOS2 | -0.10412841 | -3.208393075 | -0.718183204 | 0.473015856 |
| HCAR1 | -0.137256455 | -2.154072491 | -0.718146258 | 0.473038612 |
| TMSB15B | 0.088243922 | 0.126913627 | 0.717945242 | 0.473162431 |
| HPS1 | 0.02189723 | 6.603647324 | 0.717851679 | 0.473220069 |
| TET1 | -0.085563062 | 1.506762546 | -0.717752515 | 0.473281161 |
| LDLRAD4 | 0.080546862 | 3.54142449 | 0.717595551 | 0.473377871 |
| MTND2P28 | -0.067564768 | 10.50512276 | -0.7174314 | 0.473479022 |
| POLR2B | 0.024245695 | 6.59758627 | 0.717343458 | 0.473533216 |
| SPSB3 | 0.029282093 | 6.619487229 | 0.716787816 | 0.473875714 |
| AC015969.3 | 0.099622507 | -1.939330927 | 0.716748211 | 0.473900132 |
| SNRPD1 | -0.031906582 | 6.408846734 | -0.716070202 | 0.474318253 |
| RP4-673D20.3 | 0.100664518 | 0.566346066 | 0.715964667 | 0.474383354 |
| AOX1 | 0.137322875 | -0.514022507 | 0.71577073 | 0.474502999 |
| AL122127.1 | 0.125984243 | 1.646809175 | 0.715388446 | 0.47473889 |
| RNU6-1237P | 0.080871743 | 0.056305446 | 0.715023189 | 0.474964335 |
| LEF1-AS1 | 0.101861112 | -2.63827955 | 0.715015186 | 0.474969275 |
| U47924.31 | -0.068381984 | 0.161663463 | -0.714931415 | 0.475020989 |
| RP11-567M16.1 | 0.097346626 | -3.222937315 | 0.714860006 | 0.475065074 |
| TRBV25-1 | -0.094127688 | -0.143091042 | -0.714676096 | 0.475178623 |
| RP11-485G7.5 | -0.081223221 | -0.558633219 | -0.714176452 | 0.475487186 |
| JMJD7 | -0.061638027 | 0.383554984 | -0.714008761 | 0.475590771 |
| KIF1A | -0.172031265 | -4.969763838 | -0.713951664 | 0.475626044 |
| AGBL4 | -0.106627089 | -3.293648588 | -0.712541336 | 0.47649775 |
| MTATP8P2 | 0.09676293 | 1.919890942 | 0.712402304 | 0.476583731 |
| CGNL1 | -0.142113979 | -2.961099489 | -0.71217339 | 0.476725317 |
| GBP2 | 0.083291263 | 6.12997174 | 0.711968092 | 0.476852315 |
| PDIA5 | 0.057370728 | 4.315292351 | 0.711660583 | 0.477042577 |
| LARGE | 0.122397109 | -1.968248274 | 0.711618382 | 0.477068691 |
| IGKV6D-21 | -0.102378326 | -0.587764844 | -0.71100329 | 0.477449397 |
| IGLV2-5 | 0.086071898 | -0.411285145 | 0.710763864 | 0.477597632 |
| GOLGA8T | -0.07335359 | 2.245170509 | -0.710414192 | 0.47781417 |
| TOMM7 | 0.037326047 | 7.339134433 | 0.709966993 | 0.478091181 |
| IGLV7-43 | 0.126735143 | 1.01756186 | 0.709847214 | 0.478165391 |
| COX8A | 0.035740492 | 7.599451866 | 0.709053764 | 0.47865714 |
| NPW | 0.111729069 | 3.974351499 | 0.708742669 | 0.47885002 |
| FAM26E | -0.107312882 | -4.606879057 | -0.708487539 | 0.479008233 |
| ITGAE | 0.077158018 | 3.900656367 | 0.708189765 | 0.479192927 |
| FBRSL1 | -0.030920772 | 6.262393879 | -0.707783734 | 0.479444831 |
| COA7 | -0.0461168 | 4.494945536 | -0.707657912 | 0.479522906 |
| RBMXP1 | 0.074204019 | -0.837917858 | 0.707642313 | 0.479532586 |
| RP11-683L23.1 | -0.110189015 | -2.631277407 | -0.707605184 | 0.479555627 |
| FKBP1C | -0.041836971 | 5.322614221 | -0.707539862 | 0.479596165 |
| FAM35BP | -0.078859479 | -1.20696657 | -0.707504787 | 0.479617933 |
| EPHB4 | 0.079137726 | 2.925946893 | 0.707431532 | 0.479663397 |
| EEF1B2 | 0.044930434 | 9.380626294 | 0.707391144 | 0.479688464 |
| RP11-78A19.3 | 0.086200582 | 0.063815329 | 0.707345868 | 0.479716566 |
| GRAMD1C | -0.104020951 | 0.391734551 | -0.707232989 | 0.479786631 |
| MS4A3 | -0.172900968 | 6.894284547 | -0.707193632 | 0.479811062 |
| RASGRF2 | -0.097549238 | -0.054275153 | -0.707011913 | 0.479923872 |
| AC019185.4 | -0.104240896 | -0.346726279 | -0.706988798 | 0.479938222 |
| NASP | 0.044517139 | 6.32579991 | 0.706876273 | 0.480008086 |
| NID1 | 0.169541932 | 3.829091324 | 0.706006401 | 0.480548349 |
| AC004540.4 | -0.184143725 | 1.476247051 | -0.705124956 | 0.481096139 |
| ZPR1P1 | -0.071185319 | -2.165686303 | -0.705116734 | 0.48110125 |
| RPL7A | 0.039929834 | 9.393328853 | 0.704828106 | 0.481280698 |
| RPL13AP25 | -0.043069081 | 6.790374082 | -0.70481202 | 0.481290701 |
| BFAR | -0.024869307 | 5.466951162 | -0.704635415 | 0.48140052 |
| C11orf57 | 0.026840363 | 5.18361215 | 0.704446516 | 0.481518 |
| C1QTNF4 | -0.162904246 | 5.815648521 | -0.704120073 | 0.481721058 |
| ZDHHC7 | -0.041590986 | 7.243280672 | -0.703724305 | 0.4819673 |
| NAB1 | -0.02849707 | 5.312428801 | -0.703290671 | 0.482237182 |
| TRPM5 | 0.08756518 | -3.005698486 | 0.70299408 | 0.482421818 |
| PCBP2P1 | -0.067773538 | -0.395457956 | -0.702140263 | 0.482953561 |
| NDUFS6 | -0.024067945 | 6.673437333 | -0.701643408 | 0.483263141 |
| RNU6-5P | -0.071113637 | 0.737323808 | -0.700321147 | 0.48408754 |
| MAN1C1 | -0.099634321 | 0.837824453 | -0.700304198 | 0.484098113 |
| PTPRJ | 0.102669508 | 5.324016529 | 0.70023731 | 0.484139836 |
| IGHV3-15 | 0.14444758 | 2.877158022 | 0.699410221 | 0.484655926 |
| ZNF678 | -0.037563201 | 3.330116724 | -0.699401864 | 0.484661142 |
| GTF2IRD1P1 | -0.073766074 | -1.839413361 | -0.699185545 | 0.484796172 |
| KCNC3 | -0.161566155 | 0.576859899 | -0.698978753 | 0.484925274 |
| C1QC | 0.18344864 | 2.512253314 | 0.698587489 | 0.485169594 |
| TKT | -0.052234916 | 8.627795272 | -0.698356806 | 0.485313673 |
| TM4SF1 | 0.192122985 | 1.279037796 | 0.698226774 | 0.485394898 |
| PHF20L1 | 0.029035932 | 5.822864159 | 0.69818839 | 0.485418876 |
| PCCA | -0.084346713 | 3.355617156 | -0.698040651 | 0.485511173 |
| NTN4 | 0.080703482 | -3.048952963 | 0.698033842 | 0.485515427 |
| ESF1 | -0.025686601 | 4.84956773 | -0.697730127 | 0.485705198 |
| PPP1R32 | -0.049535471 | 2.805786551 | -0.697663735 | 0.485746687 |
| TARM1 | -0.16817451 | 1.198310985 | -0.697574425 | 0.485802501 |
| CITED1 | 0.073254733 | -1.083245851 | 0.697560467 | 0.485811224 |
| GOLGA2 | -0.025393866 | 5.589878255 | -0.696817602 | 0.486275615 |
| TPM3 | 0.027702228 | 9.140718201 | 0.696440828 | 0.486511241 |
| SOX8 | -0.109462083 | -1.904534784 | -0.696253211 | 0.486628596 |
| TMBIM1 | -0.039721645 | 6.909848767 | -0.696118268 | 0.486713012 |
| ROBO4 | 0.178868316 | 0.844795739 | 0.69599024 | 0.48679311 |
| PTCRA | -0.128584624 | 0.192144056 | -0.69561087 | 0.487030497 |
| RP11-513I15.6 | -0.061712782 | 0.031274488 | -0.695337852 | 0.487201374 |
| FLNC | -0.162275311 | -2.702912714 | -0.694707499 | 0.487596025 |
| KIAA0368 | -0.029632758 | 6.613479724 | -0.694251835 | 0.487881414 |
| SYP | -0.07422414 | -0.105086788 | -0.694124858 | 0.487960958 |
| MIR616 | 0.076852679 | 3.732678123 | 0.693795253 | 0.488167469 |
| LIFR | -0.180478158 | -3.24496669 | -0.693410529 | 0.488408575 |
| LRRC29 | -0.041194453 | 1.75113113 | -0.69337784 | 0.488429063 |
| LMTK3 | -0.09293578 | 0.350581268 | -0.69332332 | 0.488463237 |
| CDKN2A | 0.123462447 | 2.20596117 | 0.693172813 | 0.488557583 |
| RPL29P11 | 0.037057186 | 5.067260967 | 0.692464006 | 0.489002032 |
| RPL36AP33 | 0.084617591 | -0.355788897 | 0.691546521 | 0.489577655 |
| CYP2F2P | 0.098580634 | -1.056204371 | 0.69136058 | 0.489694357 |
| AP001877.1 | 0.065848926 | 0.846112631 | 0.691312175 | 0.48972474 |
| C17orf72 | 0.091230526 | -1.403719803 | 0.691148908 | 0.489827227 |
| RP11-428P16.2 | 0.119812611 | -1.791110946 | 0.691130721 | 0.489838644 |
| TJP3 | -0.09405013 | -1.47040607 | -0.691101636 | 0.489856903 |
| CDC42P1 | 0.074259043 | -0.02361763 | 0.690812898 | 0.490038186 |
| LST1 | -0.074958838 | 7.785745519 | -0.69061713 | 0.490161119 |
| TRA2B | -0.04501361 | 7.719062072 | -0.690409735 | 0.490291372 |
| CTB-43P18.1 | -0.082756229 | -0.18446872 | -0.690244395 | 0.490395225 |
| PLXNA2 | 0.100220691 | 0.631523079 | 0.690040606 | 0.490523246 |
| TP53RK | -0.040484062 | 4.49663278 | -0.689890938 | 0.490617279 |
| TNFAIP2 | -0.093941909 | 7.672445405 | -0.689775735 | 0.490689665 |
| NXF3 | 0.170126039 | 1.72630185 | 0.689735619 | 0.490714872 |
| RP11-571F15.2 | 0.091229212 | 0.276341361 | 0.689635316 | 0.490777903 |
| LAMB2 | -0.206506644 | 2.952050746 | -0.689486433 | 0.490871469 |
| KIAA0556 | -0.031680142 | 4.107371792 | -0.689290659 | 0.490994518 |
| RNF157-AS1 | -0.074017265 | -2.266157555 | -0.688950218 | 0.491208534 |
| SLC12A3 | 0.128364347 | -3.719433198 | 0.688908131 | 0.491234996 |
| RPS3A | 0.046834374 | 9.324406376 | 0.688789735 | 0.491309439 |
| IGLV3-27 | 0.131247855 | 0.481105841 | 0.688646103 | 0.491399757 |
| CDC42EP1 | 0.157797592 | 1.451662016 | 0.6884691 | 0.491511073 |
| UBLCP1 | -0.029284971 | 6.227554811 | -0.688346329 | 0.49158829 |
| CTD-2517M22.14 | -0.080566951 | 0.126567048 | -0.688178575 | 0.49169381 |
| FAM213B | 0.046035464 | 4.721198185 | 0.68812774 | 0.491725788 |
| MAN1A1 | -0.085497118 | 6.900662806 | -0.687719 | 0.491982953 |
| USP27X | 0.054696274 | 2.845682858 | 0.687702528 | 0.491993318 |
| ADIPOR2 | 0.033705115 | 6.228675079 | 0.687441253 | 0.492157742 |
| SNORA2A | 0.074032423 | -0.05789309 | 0.687123681 | 0.492357635 |
| RPL23AP2 | 0.032042581 | 5.183392043 | 0.686982021 | 0.492446816 |
| PDP1 | 0.047866598 | 4.815530229 | 0.686974478 | 0.492451564 |
| RP11-365F18.1 | 0.059566627 | 1.16070783 | 0.686815101 | 0.49255191 |
| PLCL2 | 0.063288053 | 5.305518516 | 0.686417794 | 0.492802105 |
| NDUFAF4P1 | 0.069723859 | -0.294785638 | 0.685921175 | 0.493114937 |
| SWAP70 | -0.044675076 | 5.459937919 | -0.685774965 | 0.493207059 |
| CLIC5 | -0.090705941 | -2.36959578 | -0.685738118 | 0.493230276 |
| SVOPL | -0.148438059 | -0.317548946 | -0.685689281 | 0.493261049 |
| MUC8 | -0.104627782 | 1.454786566 | -0.685629386 | 0.493298791 |
| RP11-848G14.5 | -0.120400487 | 3.259003213 | -0.684743586 | 0.493857154 |
| RPEP4 | 0.073393975 | -0.51909016 | 0.684661455 | 0.493908942 |
| RNU6-1053P | -0.065946148 | 2.021511011 | -0.684584951 | 0.493957184 |
| BTNL9 | -0.118040153 | -2.031171807 | -0.684426595 | 0.494057051 |
| RPL22P11 | 0.058547059 | 1.725655138 | 0.684133848 | 0.494241698 |
| RP11-84H6.1 | 0.090705527 | 0.046594758 | 0.684132939 | 0.494242272 |
| CTC-281F24.1 | -0.087679654 | -0.883759434 | -0.684116883 | 0.4942524 |
| AC005775.2 | 0.06745649 | -0.632797848 | 0.684045771 | 0.494297259 |
| HAGHL | 0.078768199 | 2.779401453 | 0.684028048 | 0.494308439 |
| RP6-99M1.3 | 0.079999419 | 4.427231498 | 0.683984884 | 0.494335669 |
| GOLGA8J | -0.079266497 | 1.180893512 | -0.683513306 | 0.494633221 |
| ZNF606 | -0.048675875 | 2.754101048 | -0.683100936 | 0.494893492 |
| FCGR2A | -0.116451088 | 5.963759297 | -0.682696222 | 0.495149003 |
| RP11-350G8.7 | 0.054739881 | 0.58959883 | 0.682088752 | 0.495532654 |
| SRIP3 | 0.072085352 | 0.725191861 | 0.681992938 | 0.49559318 |
| TPT1 | 0.023077845 | 11.9984119 | 0.681983398 | 0.495599207 |
| HMGN1P38 | 0.041072851 | 7.230063385 | 0.68163128 | 0.495821678 |
| LDHA | 0.035626257 | 8.77481622 | 0.681130203 | 0.496138353 |
| LGR4 | 0.132522447 | 0.706853529 | 0.68085135 | 0.496314634 |
| VIPR2 | -0.104214917 | -2.906470167 | -0.680562777 | 0.496497093 |
| ENOSF1 | 0.058518859 | 4.050651246 | 0.679878096 | 0.496930147 |
| SLC22A15 | -0.131878471 | 4.221105662 | -0.679671704 | 0.497060728 |
| FAM131A | -0.04046359 | 3.063054317 | -0.679482337 | 0.497180553 |
| RP11-25K19.1 | -0.098871003 | 3.751338442 | -0.679159782 | 0.497384692 |
| ASPHD1 | -0.096358919 | -1.585854361 | -0.679110663 | 0.497415782 |
| DOK7 | -0.108017003 | -2.228439623 | -0.678840223 | 0.497586978 |
| RPS17P2 | -0.03066776 | 6.157223943 | -0.678768662 | 0.497632283 |
| GHRL | 0.071550424 | 3.302480792 | 0.678609116 | 0.4977333 |
| PARP8 | 0.071321043 | 6.073952773 | 0.678603212 | 0.497737038 |
| NPL | -0.116423594 | 3.714226986 | -0.678510481 | 0.497795756 |
| RP3-402G11.28 | -0.061388498 | 1.716794298 | -0.678508669 | 0.497796903 |
| C5orf34 | 0.055256567 | 2.022769389 | 0.678331729 | 0.497908954 |
| MOB2 | 0.022641516 | 4.299191344 | 0.677900903 | 0.498181837 |
| SIRT2 | -0.026432315 | 5.184686627 | -0.677575894 | 0.49838775 |
| ZNF528 | 0.061919311 | 2.941638483 | 0.677417078 | 0.498488386 |
| RP11-849F2.10 | -0.076559082 | 0.229368868 | -0.676996527 | 0.498754926 |
| RPL27AP5 | 0.066280673 | 0.439991366 | 0.676484421 | 0.499079595 |
| ATP5E | 0.031011094 | 6.282098315 | 0.676087989 | 0.499331006 |
| RP11-258C19.4 | -0.071714888 | -0.11486374 | -0.675881317 | 0.4994621 |
| RP11-500C11.3 | 0.051365016 | 3.178738968 | 0.675866243 | 0.499471663 |
| OSM | 0.098219062 | 5.783958312 | 0.675743772 | 0.499549357 |
| CCDC3 | -0.159089528 | 0.303935126 | -0.675556904 | 0.499667917 |
| CTC-435M10.6 | -0.071460049 | -0.185247159 | -0.675518801 | 0.499692093 |
| NDNL2 | -0.037510362 | 6.315383977 | -0.675161673 | 0.499918723 |
| SIGLEC18P | 0.099072597 | -1.004547039 | 0.673880381 | 0.500732267 |
| PPA2 | 0.02976311 | 5.114539461 | 0.673828821 | 0.50076502 |
| BDP1P | -0.065482968 | -0.279504549 | -0.673797037 | 0.50078521 |
| C19orf54 | -0.035329099 | 4.73509483 | -0.673546005 | 0.500944692 |
| SGPP2 | -0.123806718 | 0.038152511 | -0.67337043 | 0.501056252 |
| HIST1H2BH | 0.106399426 | 1.825050277 | 0.673139748 | 0.501202847 |
| KCNH3 | -0.125241766 | 1.213406101 | -0.673078167 | 0.501241984 |
| B3GAT1 | -0.109102131 | -1.273824775 | -0.672922974 | 0.501340623 |
| AC002059.10 | 0.08145413 | 0.613634482 | 0.672861016 | 0.501380006 |
| FBXO44 | 0.066979017 | 3.607833953 | 0.672339351 | 0.501711662 |
| RP11-1105G2.3 | 0.060358638 | 2.011685924 | 0.671962109 | 0.501951572 |
| NPM1P32 | 0.070511351 | 3.331875136 | 0.671701575 | 0.502117295 |
| VPS9D1-AS1 | 0.076602082 | 3.589642377 | 0.671254105 | 0.502401995 |
| TMEM110 | -0.039559542 | 1.424564568 | -0.670655355 | 0.50278308 |
| B4GALT6 | -0.088177159 | 4.481430548 | -0.670527217 | 0.502864656 |
| HLA-DPA1 | 0.120476701 | 7.467184679 | 0.669891459 | 0.503269498 |
| RP11-615J4.4 | 0.086125972 | 0.137735261 | 0.669220753 | 0.503696782 |
| STAT5A | -0.038796906 | 7.436826632 | -0.669211493 | 0.503702682 |
| RNA5SP492 | 0.083593412 | -0.467835567 | 0.668645343 | 0.504063508 |
| KHDRBS1 | 0.024492223 | 8.741502007 | 0.668243216 | 0.50431988 |
| RP11-439L8.4 | -0.138788125 | -1.420215507 | -0.668234857 | 0.504325211 |
| ASAH1 | -0.046642972 | 7.677809739 | -0.668211866 | 0.50433987 |
| RP11-351D16.3 | 0.063072971 | 2.171652183 | 0.668168297 | 0.504367652 |
| DNAJC4 | 0.029617075 | 6.357750585 | 0.668073217 | 0.504428283 |
| ILF3-AS1 | -0.041726319 | 5.24776148 | -0.667924358 | 0.504523215 |
| LINC00086 | 0.091287741 | -2.467561581 | 0.66777496 | 0.504618499 |
| CKS2 | -0.057794542 | 6.633092142 | -0.667396078 | 0.504860191 |
| DCAF4 | 0.046545445 | 3.081744012 | 0.667393216 | 0.504862016 |
| MTHFD1P1 | 0.052512099 | 1.882166871 | 0.667287047 | 0.504929753 |
| RP11-75C9.1 | 0.08329969 | -3.083864257 | 0.666789011 | 0.50524757 |
| KIAA2022 | 0.120078613 | -3.863512847 | 0.66626968 | 0.505579088 |
| METRN | 0.071726804 | 1.921613801 | 0.666005404 | 0.505747835 |
| YWHAH | 0.041080618 | 8.436120773 | 0.665898688 | 0.505815984 |
| CHPF2 | -0.033237069 | 6.639194419 | -0.665631154 | 0.505986853 |
| PARP3 | 0.052645505 | 4.419367899 | 0.665435648 | 0.506111739 |
| TULP4 | 0.059494542 | 3.058434452 | 0.665358351 | 0.506161119 |
| RNF141 | 0.043913514 | 4.969715843 | 0.664890833 | 0.506459841 |
| IGHVIII-38-1 | 0.119429832 | -0.228548419 | 0.664594698 | 0.506649106 |
| C21orf90 | -0.119020244 | -2.743780181 | -0.664076884 | 0.50698014 |
| RP5-1160K1.3 | -0.047526485 | 1.786731543 | -0.663810065 | 0.507150759 |
| AD000092.3 | 0.036005429 | 5.394256015 | 0.66375968 | 0.507182982 |
| SLC25A5P2 | 0.061647891 | 0.703064528 | 0.663640627 | 0.507259123 |
| ATP1B3P1 | -0.051611554 | 2.023969042 | -0.66291825 | 0.507721256 |
| TIGIT | -0.09862115 | 0.790366554 | -0.66286715 | 0.507753955 |
| RP11-251G23.2 | -0.093449133 | -0.522444609 | -0.662863362 | 0.507756379 |
| MIR4312 | -0.0724666 | 2.022687674 | -0.662840041 | 0.507771302 |
| RP11-161M6.2 | -0.108197884 | -0.352337949 | -0.662534287 | 0.507966982 |
| LOXL4 | -0.153792728 | -0.297902284 | -0.661384523 | 0.508703176 |
| ADORA2B | -0.087649035 | 2.112256796 | -0.66137464 | 0.508709506 |
| STK31 | -0.092814329 | -2.550246934 | -0.661329095 | 0.508738681 |
| ANXA5 | 0.129578068 | 7.010629688 | 0.661327565 | 0.508739661 |
| UTS2 | -0.134013759 | -0.573504319 | -0.661231508 | 0.508801193 |
| SUGT1P3 | 0.052341885 | 1.023720609 | 0.661108313 | 0.508880116 |
| RP11-57A19.2 | -0.071612778 | -2.74035377 | -0.660971416 | 0.508967825 |
| TPGS1 | 0.025689629 | 3.598601581 | 0.660839609 | 0.50905228 |
| MED24 | 0.02417553 | 5.802265381 | 0.660809711 | 0.509071438 |
| TMEM120A | 0.052022114 | 5.270227283 | 0.660516711 | 0.509259206 |
| OXSR1 | -0.025030949 | 6.225645087 | -0.660325449 | 0.509381796 |
| MAZ | 0.030167563 | 8.696016313 | 0.660183225 | 0.509472965 |
| PAM | 0.069154729 | 3.921214315 | 0.659769343 | 0.509738321 |
| GSTZ1 | -0.04590989 | 2.323722941 | -0.659421635 | 0.509961307 |
| RPL36P2 | 0.067115473 | -0.086930606 | 0.659361469 | 0.509999897 |
| RFTN2 | 0.113069827 | -4.040708337 | 0.659312521 | 0.510031293 |
| TSN | 0.02246413 | 6.68281249 | 0.659044639 | 0.510203133 |
| SNORD116-20 | 0.114870666 | 0.721638797 | 0.658782881 | 0.510371075 |
| RPS6KA5 | 0.038186942 | 3.67054804 | 0.657891155 | 0.510943415 |
| SIRPAP1 | -0.087760298 | 3.168814929 | -0.657803549 | 0.510999662 |
| RP11-131L23.1 | 0.067054594 | -1.015641811 | 0.657658396 | 0.511092864 |
| POLD3 | -0.043111366 | 4.679961868 | -0.657478719 | 0.511208244 |
| PRKAR1B | 0.060623823 | 3.094223514 | 0.657014358 | 0.511506502 |
| ZBTB40-IT1 | 0.052718239 | 2.135485304 | 0.656501588 | 0.511835957 |
| ASAP3 | 0.093995468 | -1.397493512 | 0.656246124 | 0.512000135 |
| ABI3BP | 0.103361262 | -3.910753033 | 0.655398301 | 0.512545198 |
| SCUBE3 | -0.13092912 | -3.590350871 | -0.655128375 | 0.512718796 |
| RHBDF2 | 0.060343787 | 5.007996068 | 0.654708443 | 0.51298893 |
| SUMO1P3 | 0.022636341 | 6.64385546 | 0.654577953 | 0.513072886 |
| RP11-220D10.1 | 0.036367367 | 3.874228416 | 0.654517665 | 0.513111678 |
| RP11-1084J3.4 | 0.070607501 | -1.211390752 | 0.6543548 | 0.513216478 |
| UTF1 | 0.118759824 | 0.716070076 | 0.654281045 | 0.513263942 |
| TMEM135 | -0.02783893 | 3.266699847 | -0.653903985 | 0.513506628 |
| RPL5P6 | 0.063880807 | -0.0719559 | 0.65361365 | 0.513693537 |
| COLEC12 | 0.134755312 | -1.205385662 | 0.65355713 | 0.513729926 |
| NF1 | -0.03338871 | 5.347043577 | -0.652814597 | 0.514208124 |
| EGFL7 | 0.142549553 | 6.550880794 | 0.652557004 | 0.514374071 |
| RP6-201G10.2 | 0.088435607 | -0.55082752 | 0.65244694 | 0.514444984 |
| MTMR12 | 0.028643998 | 5.585592696 | 0.652251885 | 0.51457067 |
| RP11-367G18.2 | -0.04404523 | 5.214115363 | -0.652028396 | 0.514714698 |
| CAMLG | -0.042169488 | 6.231859184 | -0.652014059 | 0.514723938 |
| CACTIN | 0.01778194 | 4.788690674 | 0.651929042 | 0.514778733 |
| RP11-872D17.4 | 0.08735104 | -0.264229482 | 0.651705852 | 0.514922598 |
| SRGAP2 | -0.05636319 | 4.817118833 | -0.651491096 | 0.515061046 |
| EEF1A1P29 | 0.052728987 | 5.33388307 | 0.650565308 | 0.515658103 |
| ARVCF | 0.070122504 | 2.562577461 | 0.649688508 | 0.516223898 |
| RP11-269F19.2 | -0.06207414 | 0.601335531 | -0.649362679 | 0.516434236 |
| ATOH8 | 0.107655821 | -2.117979953 | 0.649252181 | 0.516505578 |
| CCNB3 | -0.077320883 | -1.606393301 | -0.649204535 | 0.516536342 |
| RHOQ | 0.041907476 | 5.814255121 | 0.649176975 | 0.516554137 |
| IGLV9-49 | 0.106580371 | 0.26846519 | 0.648901426 | 0.516732073 |
| ZCCHC10 | 0.028698069 | 4.859045985 | 0.64877306 | 0.516814977 |
| IGHMBP2 | 0.027806401 | 4.240329726 | 0.648757323 | 0.516825141 |
| IFRD1 | 0.054079952 | 5.919885176 | 0.648446768 | 0.517025739 |
| ULK4P1 | 0.078583012 | -1.217917883 | 0.648145653 | 0.517220279 |
| MYBPH | -0.144028741 | -0.390419661 | -0.646635141 | 0.51819674 |
| ABCA5 | 0.078659835 | 2.723005787 | 0.646295962 | 0.518416131 |
| RNF212 | -0.153101115 | -1.692606455 | -0.646075867 | 0.518558521 |
| SCAF1 | 0.025044288 | 6.286048137 | 0.645476297 | 0.518946515 |
| ZNF251 | 0.042725996 | 4.383170142 | 0.645026888 | 0.519237435 |
| RP11-778J16.2 | 0.031368023 | 3.503708474 | 0.644965344 | 0.519277282 |
| DDX50P1 | 0.041050771 | 1.386120387 | 0.644710511 | 0.519442289 |
| NT5DC3 | 0.079145886 | 3.777412018 | 0.64466716 | 0.519470362 |
| HDAC7 | -0.031754298 | 5.904849778 | -0.644609087 | 0.51950797 |
| SLC25A14 | 0.02805615 | 3.676620525 | 0.644252588 | 0.519738868 |
| SERTAD1 | 0.059638974 | 4.658349506 | 0.644203589 | 0.519770608 |
| C16orf95 | -0.065793263 | -0.761293939 | -0.643524212 | 0.520210789 |
| RP11-353N4.2 | 0.048600491 | 1.360526916 | 0.643501277 | 0.520225653 |
| RP11-159H22.2 | -0.080055089 | -1.994552248 | -0.643321439 | 0.520342207 |
| RP11-115H15.2 | 0.085264291 | -0.690734476 | 0.642870116 | 0.520634772 |
| AC114776.1 | -0.067366812 | 0.16065938 | -0.642853613 | 0.520645472 |
| RP11-351I24.1 | -0.092222916 | -0.231786879 | -0.642577654 | 0.520824404 |
| WDR82P1 | 0.028945406 | 4.738797822 | 0.64238372 | 0.520950169 |
| ARMCX4 | 0.0439809 | 2.022284385 | 0.642244291 | 0.521040598 |
| RP1L1 | 0.098564587 | -2.699702109 | 0.641822035 | 0.521314508 |
| ZNF880 | 0.057245947 | 3.368149191 | 0.641774958 | 0.521345051 |
| CAMK2B | 0.112406724 | -3.228165245 | 0.641773866 | 0.52134576 |
| LILRA3 | 0.159520152 | 4.389782173 | 0.641757832 | 0.521356162 |
| DPP10-AS1 | 0.124545622 | -1.531671676 | 0.641725656 | 0.521377038 |
| NCOA3 | -0.027008542 | 6.223746798 | -0.641674291 | 0.521410365 |
| GLO1 | -0.031669058 | 7.419271299 | -0.641632387 | 0.521437554 |
| ATAT1 | -0.042765068 | 4.262219006 | -0.641373965 | 0.521605245 |
| TRAJ3 | 0.085735259 | -0.280223417 | 0.641361384 | 0.52161341 |
| ASS1P2 | -0.064464909 | -0.372195945 | -0.641334335 | 0.521630964 |
| NHP2L1 | 0.020000473 | 7.306634379 | 0.640900094 | 0.521912814 |
| RP1-283E3.8 | 0.04704221 | 2.943778374 | 0.640478636 | 0.522186443 |
| PAX5 | 0.142582856 | 1.27501479 | 0.639778652 | 0.522641066 |
| RP11-503N18.1 | 0.099355187 | -2.502856397 | 0.639738196 | 0.522667348 |
| GTF2IRD2B | -0.040988164 | 2.010880354 | -0.639711785 | 0.522684506 |
| COL4A1 | 0.125474507 | -2.588207993 | 0.639444439 | 0.522858202 |
| MIER2 | 0.040259204 | 4.048232079 | 0.639434931 | 0.52286438 |
| ENOX1 | 0.087323325 | -2.495232839 | 0.639343908 | 0.522923525 |
| IREB2 | 0.021728159 | 5.688025098 | 0.639155423 | 0.523046011 |
| C2orf66 | 0.100647227 | -1.34464692 | 0.638834051 | 0.523254886 |
| WDYHV1 | -0.030223625 | 3.779782611 | -0.638765737 | 0.523299292 |
| USP45 | -0.070665105 | 2.134964895 | -0.638748517 | 0.523310486 |
| AC139099.3 | -0.070834319 | -1.526575566 | -0.638678829 | 0.523355788 |
| RP11-343H5.4 | 0.091205829 | 3.396759677 | 0.638657897 | 0.523369395 |
| C2CD4B | 0.151233449 | -0.844442058 | 0.638639493 | 0.523381359 |
| ST6GALNAC2 | -0.099567411 | 1.150972047 | -0.638228692 | 0.523648455 |
| NTN1 | 0.144026262 | 0.979876604 | 0.637775095 | 0.523943457 |
| RPL35P1 | -0.033709008 | 8.188769356 | -0.63761733 | 0.524046081 |
| RP11-369G6.2 | -0.072708829 | -1.069046747 | -0.637559117 | 0.524083951 |
| CTC-205M6.5 | -0.062934672 | 2.838848461 | -0.637014245 | 0.524438476 |
| HSP90AB3P | -0.044483227 | 7.921927304 | -0.636912229 | 0.524504867 |
| RPS24P16 | -0.059393705 | 1.655131335 | -0.636853242 | 0.524543257 |
| RPS13 | 0.031472288 | 9.364835875 | 0.636552379 | 0.52473909 |
| FDPSP8 | 0.063438542 | -0.546350831 | 0.636468259 | 0.52479385 |
| MIEF2 | 0.032194346 | 2.512422924 | 0.636261845 | 0.524928234 |
| RP11-350G24.2 | 0.040327498 | 4.191886997 | 0.636131244 | 0.52501327 |
| PDCD4-AS1 | -0.037452221 | 5.008290141 | -0.636101172 | 0.525032851 |
| SRL | -0.095470618 | -3.830379967 | -0.635830892 | 0.525208859 |
| TCIRG1 | -0.05488835 | 7.856748935 | -0.635286199 | 0.525563658 |
| CHP1 | -0.032056632 | 7.297985217 | -0.634775317 | 0.525896546 |
| CTD-2554C21.2 | -0.104991244 | -1.538106506 | -0.634505783 | 0.526072217 |
| FTCD | -0.100276757 | -1.481318359 | -0.634214563 | 0.526262054 |
| CTC-338M12.6 | -0.06457051 | -1.124822904 | -0.634070327 | 0.526356091 |
| ADCK3 | 0.031611639 | 5.686580372 | 0.633736562 | 0.526573726 |
| PSMA2P2 | -0.063037521 | 0.6165714 | -0.633426385 | 0.526776022 |
| RP11-354E11.2 | -0.16149482 | 3.22828469 | -0.633142406 | 0.526961266 |
| FBXO39 | -0.09820946 | -1.361545982 | -0.633029112 | 0.527035179 |
| EID2B | -0.037967887 | 2.747253247 | -0.632983658 | 0.527064835 |
| IGHV3-64 | 0.102384444 | 0.094733606 | 0.632873661 | 0.527136604 |
| TRPC2 | 0.097265408 | 2.309835901 | 0.632566173 | 0.527337255 |
| SP140L | -0.034416213 | 4.669517821 | -0.632523737 | 0.52736495 |
| NECAB3 | 0.047991584 | 2.801312236 | 0.632404161 | 0.527442991 |
| NQO2 | 0.052050369 | 5.449994139 | 0.632028636 | 0.527688119 |
| ALS2CR12 | -0.057610037 | 1.28629829 | -0.63170875 | 0.527896973 |
| RP11-673C5.1 | 0.034395387 | 7.449161235 | 0.631583325 | 0.527978875 |
| PADI6 | 0.104101801 | -2.080050308 | 0.631478841 | 0.528047107 |
| RP11-34P13.13 | 0.090189842 | 0.485076067 | 0.631318508 | 0.52815182 |
| Z95704.5 | -0.08155887 | -1.309963363 | -0.630900211 | 0.528425058 |
| VSIG4 | -0.171193772 | 2.897377616 | -0.630651316 | 0.528587675 |
| RP11-1033A18.1 | 0.035222003 | 7.035072166 | 0.630482828 | 0.528697771 |
| PLD2 | -0.095995811 | 2.760041342 | -0.63019982 | 0.528882726 |
| RN7SL535P | 0.080694726 | 0.91981687 | 0.629775846 | 0.529159869 |
| RP11-195M16.3 | -0.084458824 | -1.534784194 | -0.629362958 | 0.529429836 |
| TNFRSF10C | -0.110644703 | 4.035360455 | -0.629357025 | 0.529433716 |
| CORO2A | 0.087900269 | 4.23822962 | 0.628937005 | 0.529708419 |
| AMDHD2 | 0.038887176 | 3.97849633 | 0.62886787 | 0.529753642 |
| MICAL2 | 0.074615648 | 4.318209968 | 0.627567559 | 0.530604576 |
| TVP23CP2 | -0.064828528 | -0.196635201 | -0.627305586 | 0.530776097 |
| SIGLEC1 | 0.166863352 | 0.786881923 | 0.627226499 | 0.530827884 |
| CTD-2288F12.1 | -0.06299548 | 0.067280337 | -0.626694231 | 0.531176479 |
| RP11-277B15.3 | -0.07083787 | 0.550974531 | -0.626446541 | 0.531338737 |
| SCML4 | -0.087376105 | 0.987921009 | -0.625921729 | 0.531682617 |
| GRAMD3 | 0.075214253 | 1.502296276 | 0.625341703 | 0.532062808 |
| AOX3P | -0.143431554 | -0.706750074 | -0.62520181 | 0.532154524 |
| CSF2RB | 0.080429367 | 6.54369946 | 0.624526757 | 0.532597214 |
| RP11-800A3.2 | 0.092217239 | -0.304434376 | 0.624327216 | 0.532728106 |
| FOXP1-IT1 | 0.070500312 | 0.798236548 | 0.624111355 | 0.532869722 |
| CDH2 | -0.182064024 | -1.341871596 | -0.624092155 | 0.532882319 |
| HNRNPA1P21 | 0.056117005 | 0.611167385 | 0.624059409 | 0.532903804 |
| TXLNG | 0.025233625 | 5.208367472 | 0.623902999 | 0.533006432 |
| NR5A1 | -0.121489661 | -1.4391373 | -0.62389219 | 0.533013525 |
| C9orf153 | 0.066094402 | -1.069235369 | 0.623720643 | 0.533126097 |
| RP11-214O1.3 | 0.085025018 | -1.163347828 | 0.622659976 | 0.533822396 |
| AC107982.4 | -0.094623428 | -1.412036491 | -0.622562973 | 0.533886099 |
| C9orf142 | -0.031415931 | 6.162862645 | -0.622551493 | 0.533893638 |
| SLC45A4 | 0.063027496 | 3.27652377 | 0.622383996 | 0.534003645 |
| GOLGA8K | 0.061040342 | 2.37787937 | 0.62231466 | 0.534049186 |
| MIR5581 | 0.062629383 | 0.597435527 | 0.622021256 | 0.53424192 |
| FAM129A | -0.062093847 | 6.537419317 | -0.621784373 | 0.534397552 |
| TRAM2 | -0.032976888 | 5.007690268 | -0.621662514 | 0.534477623 |
| GAMT | 0.064812381 | 4.68231518 | 0.621481238 | 0.534596746 |
| RNF145 | 0.047506923 | 5.565751497 | 0.621412209 | 0.53464211 |
| CTC-510F12.4 | 0.096853458 | 4.504712017 | 0.621378494 | 0.534664268 |
| CTA-134P22.2 | -0.110565492 | -2.05744697 | -0.621068998 | 0.534867693 |
| IGHV3-30 | 0.119982858 | 3.08725421 | 0.620894864 | 0.534982164 |
| PNP | 0.052644343 | 7.024846924 | 0.620683291 | 0.535121264 |
| EIF4BP7 | 0.036197835 | 7.473100808 | 0.620674574 | 0.535126995 |
| IL31RA | 0.162994876 | -1.221364151 | 0.620510055 | 0.535235172 |
| SLC9C1 | -0.096798125 | -3.066100736 | -0.620350997 | 0.535339769 |
| RP11-425L10.1 | -0.026063705 | 10.12429238 | -0.62011612 | 0.535494243 |
| CYTH4 | 0.065487242 | 6.468271725 | 0.620084778 | 0.535514858 |
| HNRNPKP2 | 0.017867185 | 6.292279576 | 0.619818598 | 0.535689951 |
| TEN1-CDK3 | -0.055232745 | -0.186469219 | -0.619469491 | 0.535919636 |
| HLA-DPB2 | -0.119288149 | -0.509068284 | -0.619450959 | 0.53593183 |
| RP11-159H3.1 | 0.041691747 | 4.136349791 | 0.619301416 | 0.536030234 |
| ATP5G2P1 | -0.055507742 | 0.725605979 | -0.61925027 | 0.536063891 |
| PNMA5 | -0.131501726 | -2.17975847 | -0.619166402 | 0.536119085 |
| HSPG2 | -0.09049774 | 4.95978466 | -0.619036691 | 0.536204454 |
| CTD-2010I16.1 | 0.067595701 | 0.444997306 | 0.618969612 | 0.536248604 |
| ACTA1 | 0.064602382 | -0.418148539 | 0.618919207 | 0.536281781 |
| NPM1P18 | -0.045540656 | 2.45121369 | -0.618726682 | 0.536408512 |
| GAS6-AS1 | -0.081195274 | 4.386021798 | -0.618364983 | 0.536646644 |
| TMEM209 | -0.029982636 | 4.393485916 | -0.617761735 | 0.537043924 |
| AIMP1 | 0.022771677 | 6.021857653 | 0.617650888 | 0.53711694 |
| TXLNG2P | 0.317230352 | 1.16252827 | 0.616691173 | 0.537749326 |
| TCEAL4 | -0.06217029 | 6.226699902 | -0.616483746 | 0.537886055 |
| SNORD20 | -0.055561662 | 3.28071952 | -0.616238026 | 0.538048049 |
| RP11-326G21.1 | -0.073339049 | 2.773757552 | -0.615724515 | 0.538386665 |
| KM-PA-2 | 0.055847594 | 0.819654308 | 0.6156456 | 0.538438713 |
| TEAD2 | -0.092810571 | -2.059006058 | -0.615490227 | 0.538541194 |
| CTA-126B4.7 | 0.094969455 | 0.078016234 | 0.615443415 | 0.538572072 |
| SNRPGP2 | -0.040594788 | 4.497594937 | -0.615353373 | 0.538631469 |
| KCNG2 | 0.113363972 | 0.845142842 | 0.615267332 | 0.538688229 |
| CTD-2576D5.4 | -0.078426792 | -0.613728966 | -0.615096223 | 0.538801116 |
| CRISPLD2 | -0.119111445 | 3.041893735 | -0.615019137 | 0.538851977 |
| RP4-595K12.1 | 0.062461636 | 0.501310941 | 0.614629955 | 0.539108793 |
| AKAP13 | -0.03091672 | 7.024018268 | -0.614514022 | 0.539185307 |
| AF064858.6 | 0.089942919 | -2.976245127 | 0.614232885 | 0.539370877 |
| HNRNPK | -0.015534121 | 9.368899178 | -0.614055987 | 0.539487658 |
| NDUFA5P11 | -0.031129984 | 4.639460106 | -0.61402428 | 0.539508591 |
| CBX7 | -0.054446262 | 3.376590961 | -0.613597348 | 0.539790493 |
| AC104820.2 | 0.082478881 | -1.168821117 | 0.613591633 | 0.539794267 |
| PALM | -0.126796506 | 2.749285403 | -0.613237349 | 0.540028257 |
| BCAS1 | -0.085684878 | -4.318243573 | -0.613174123 | 0.54007002 |
| RP11-552M11.4 | 0.055337837 | 1.138437521 | 0.612953907 | 0.540215495 |
| PEMT | -0.032422463 | 4.820912425 | -0.612921912 | 0.540236633 |
| ZRANB3 | 0.041089369 | 2.218310854 | 0.612592513 | 0.540454274 |
| ABC7-42389800N19.1 | 0.038557542 | 6.034445681 | 0.612236246 | 0.540689718 |
| CCER2 | 0.056998916 | 2.0607822 | 0.61219506 | 0.54071694 |
| ZSCAN9 | 0.053115145 | 4.049694013 | 0.612043797 | 0.540816922 |
| CTD-2267D19.3 | 0.06333636 | 3.114742156 | 0.611730912 | 0.541023763 |
| RP11-752G15.4 | -0.046109521 | 2.149130767 | -0.611722612 | 0.541029251 |
| MCM8 | 0.045645903 | 4.519585783 | 0.611698496 | 0.541045195 |
| RP11-384B12.2 | 0.056262026 | -0.221354415 | 0.611624463 | 0.541094143 |
| SIKE1 | 0.017236666 | 5.491846266 | 0.611598747 | 0.541111146 |
| ERICH1-AS1 | -0.144631058 | -2.276775354 | -0.611470417 | 0.541196 |
| TSPEAR | 0.101970114 | -3.764405796 | 0.611446032 | 0.541212125 |
| PEX5 | -0.025625605 | 4.532081 | -0.610999869 | 0.541507193 |
| HTRA2 | 0.029337434 | 5.407758734 | 0.610910727 | 0.541566157 |
| GLDCP1 | -0.071191931 | -2.795642785 | -0.610641793 | 0.541744064 |
| RP11-475J5.5 | -0.078433518 | -0.433629239 | -0.610599817 | 0.541771834 |
| PMS2P1 | -0.033520362 | 5.450876026 | -0.609651637 | 0.54239933 |
| TMSB4XP1 | -0.054127846 | 6.317487235 | -0.609570052 | 0.542453339 |
| FBXW9 | 0.035310666 | 3.908524309 | 0.609418491 | 0.54255368 |
| TMEM255A | -0.109612979 | -0.838053027 | -0.609323002 | 0.542616902 |
| RDH14 | 0.026082373 | 4.637329164 | 0.609103847 | 0.542762017 |
| LYZ | -0.104002723 | 12.02285178 | -0.608761725 | 0.542988595 |
| CDKL5 | -0.085300604 | 1.337338041 | -0.60875856 | 0.542990692 |
| RPL7P10 | 0.069906465 | -0.388879095 | 0.608742368 | 0.543001416 |
| VEPH1 | 0.120436789 | -2.11275871 | 0.608622257 | 0.543080975 |
| RPL31P11 | 0.062183194 | -0.420543099 | 0.608518647 | 0.543149608 |
| ADCY10 | 0.060609247 | -2.257257302 | 0.608466456 | 0.543184182 |
| ZNF449 | 0.035029382 | 2.558573226 | 0.608264121 | 0.543318229 |
| TMEM121 | 0.099760938 | 1.274600876 | 0.608042058 | 0.543465365 |
| AC093484.4 | -0.062984814 | 0.169073716 | -0.608038651 | 0.543467623 |
| AKTIP | 0.054558526 | 2.659569982 | 0.607722835 | 0.543676913 |
| RPS3P6 | -0.03820084 | 6.205192149 | -0.60736293 | 0.54391547 |
| RP11-2B6.2 | 0.055567999 | 3.370303866 | 0.606126385 | 0.54473549 |
| LINC00656 | -0.082516217 | -1.689435189 | -0.606010351 | 0.54481247 |
| HSPA6 | -0.113997357 | 3.407475075 | -0.605860427 | 0.544911942 |
| PAN3 | -0.034827021 | 6.97946759 | -0.605838129 | 0.544926736 |
| RPL10 | 0.035406182 | 8.921674221 | 0.605017317 | 0.545471497 |
| STAG3L1 | 0.05021499 | 4.677891604 | 0.604679767 | 0.545695602 |
| MIR5192 | 0.074164429 | 0.787992769 | 0.604655241 | 0.545711888 |
| PDXP | 0.044793965 | 1.534101033 | 0.604588764 | 0.545756029 |
| MTHFS | 0.038480735 | 2.883349346 | 0.604561319 | 0.545774253 |
| WHSC1L1 | -0.016672221 | 6.299549109 | -0.604388365 | 0.545889106 |
| CILP | -0.075612159 | -1.211893727 | -0.604377597 | 0.545896258 |
| LRRC61 | -0.065493505 | 3.73475686 | -0.604373072 | 0.545899262 |
| RNU6-312P | 0.061532854 | 0.613297219 | 0.603965131 | 0.546170213 |
| ACPP | 0.095331795 | 4.265465599 | 0.603622908 | 0.546397566 |
| RNU6-1223P | -0.065824011 | -0.040632272 | -0.60304109 | 0.5467842 |
| FUS | -0.02973482 | 8.303363898 | -0.603020958 | 0.54679758 |
| UBP1 | 0.019871787 | 6.017510879 | 0.602024391 | 0.54746015 |
| KCNMA1 | 0.11915933 | -2.811526293 | 0.601937705 | 0.547517803 |
| EIF3B | 0.027189634 | 6.710896341 | 0.601858388 | 0.547570556 |
| C17orf64 | -0.083129054 | -0.486262948 | -0.601604206 | 0.54773963 |
| ANGPTL6 | 0.084152967 | 3.477954558 | 0.601524706 | 0.547792516 |
| SNORD12B | -0.055579282 | 3.575401123 | -0.601070846 | 0.548094489 |
| RBPMS | 0.190983932 | 0.929765262 | 0.600665203 | 0.548364451 |
| GLMN | 0.025614658 | 4.168170025 | 0.600396222 | 0.548543498 |
| TMEM8A | -0.025839432 | 6.04704656 | -0.600180381 | 0.548687194 |
| RPL6P25 | 0.080734661 | -0.26344837 | 0.599788938 | 0.548947843 |
| CBLN2 | 0.118344714 | -3.699757087 | 0.599771503 | 0.548959454 |
| AQP7P1 | 0.090338958 | -2.047839271 | 0.599561293 | 0.549099453 |
| CTC-268N12.3 | -0.069420149 | 0.156360915 | -0.599467576 | 0.549161874 |
| RPL7L1P3 | 0.068179911 | -0.620871064 | 0.599324038 | 0.549257485 |
| IGHV4-61 | 0.098861404 | 1.471179743 | 0.599153317 | 0.549371213 |
| MAOA | -0.120808852 | -0.221430189 | -0.598886869 | 0.549548736 |
| LILRA6 | -0.13524504 | 3.78319549 | -0.598690535 | 0.549679562 |
| LINC00618 | -0.056463355 | 0.199035015 | -0.598119944 | 0.550059861 |
| XXbac-B562F10.11 | -0.04310324 | 1.095489526 | -0.597837189 | 0.550248366 |
| RHEBP2 | -0.046145062 | 2.402154976 | -0.59720942 | 0.550666995 |
| LINC00441 | 0.056076025 | 0.716752166 | 0.596867067 | 0.55089536 |
| AC023490.2 | -0.077595269 | -1.037779464 | -0.59650889 | 0.551134331 |
| APOL3 | -0.070972822 | 4.361332834 | -0.595864626 | 0.551564302 |
| AC006026.9 | -0.039029849 | 1.742569014 | -0.595268756 | 0.551962124 |
| CPNE7 | 0.132985136 | 1.850483622 | 0.595250646 | 0.551974217 |
| LINC00987 | -0.082702259 | 0.285625769 | -0.593994726 | 0.552813181 |
| CTA-351J1.1 | 0.058259902 | 0.174054162 | 0.593746488 | 0.552979081 |
| TAS2R64P | -0.074392136 | -1.634486317 | -0.593036419 | 0.55345376 |
| EFCAB4A | 0.088386113 | 3.99820939 | 0.592991012 | 0.553484121 |
| FIGN | 0.159212358 | -4.567021745 | 0.592939504 | 0.553518563 |
| PGAP3 | 0.027418537 | 4.321938405 | 0.592894576 | 0.553548606 |
| ELAVL4 | -0.118994004 | -4.007285448 | -0.592869191 | 0.553565581 |
| ZNF423 | -0.111927455 | -3.405647765 | -0.592722687 | 0.553663552 |
| RP11-416L21.2 | 0.075627387 | 0.436104529 | 0.592635889 | 0.553721601 |
| RP11-513M16.7 | 0.06068216 | 2.809250332 | 0.592489046 | 0.553819814 |
| SLC17A7 | 0.085932809 | -3.191881463 | 0.592479028 | 0.553826515 |
| RP11-717A5.2 | 0.082469877 | 3.32733721 | 0.592158649 | 0.554040824 |
| ZSCAN18 | -0.07624376 | 3.837270808 | -0.59160755 | 0.554409562 |
| ANKK1 | 0.072857491 | -2.103990752 | 0.59159484 | 0.554418067 |
| RP11-936I5.1 | -0.067359297 | -1.487766446 | -0.591513319 | 0.554472623 |
| GOLGA2P7 | 0.08647614 | -1.905644704 | 0.591198464 | 0.554683357 |
| LINC00278 | 0.159603212 | 0.632482332 | 0.590721971 | 0.555002351 |
| RP11-512F24.1 | -0.032513963 | 7.068345798 | -0.590720686 | 0.555003211 |
| ATP6V0A2 | 0.035145013 | 4.940635597 | 0.590699708 | 0.555017257 |
| GALNT5 | -0.129240561 | -2.75808413 | -0.59049832 | 0.555152108 |
| PADI4 | -0.11008606 | 6.566115279 | -0.590120303 | 0.555405273 |
| C11orf31 | -0.022583503 | 6.011568119 | -0.589958094 | 0.555513925 |
| NFIC | 0.029859785 | 5.400796065 | 0.589875121 | 0.555569506 |
| SNTB2 | 0.074567786 | 1.543650161 | 0.589545202 | 0.555790538 |
| RPL5P29 | -0.043825628 | 7.208563547 | -0.588792131 | 0.556295224 |
| RP11-960L18.1 | -0.069694853 | 2.693419988 | -0.588512442 | 0.55648272 |
| RP11-332O19.2 | -0.069390401 | 0.067984671 | -0.58845737 | 0.556519643 |
| AC111200.7 | 0.029158452 | 2.651874532 | 0.588357405 | 0.556586667 |
| DNAJC8P1 | 0.038690457 | 2.09009805 | 0.588225385 | 0.556675188 |
| PRICKLE2 | 0.121902299 | -3.281330893 | 0.588214466 | 0.55668251 |
| CEP112 | 0.102283136 | -1.124835101 | 0.588179012 | 0.556706284 |
| CTSW | -0.084896226 | 7.907454214 | -0.588090447 | 0.556765674 |
| DLX1 | -0.103921767 | -2.478595869 | -0.587897897 | 0.556894805 |
| REPS1 | -0.020383119 | 5.097387111 | -0.587871808 | 0.556912302 |
| CAMK1D | -0.067058202 | 4.961552283 | -0.587645729 | 0.55706394 |
| ALOX15 | 0.1231617 | -0.383672562 | 0.587587742 | 0.557102836 |
| CYTH2 | -0.025604805 | 4.522926843 | -0.587550572 | 0.55712777 |
| GPR83 | 0.06147443 | -1.713214377 | 0.587378612 | 0.557243128 |
| RP11-39K24.2 | -0.062071318 | -0.185957074 | -0.587294733 | 0.557299403 |
| SPEG | -0.086911074 | -1.92917043 | -0.587130168 | 0.557409816 |
| CD81-AS1 | -0.072636263 | -1.152964417 | -0.586913525 | 0.557555187 |
| NCOA6 | -0.01735538 | 5.744997654 | -0.586811232 | 0.557623835 |
| KLF2 | 0.067804359 | 9.031632818 | 0.586284122 | 0.557977634 |
| RPL5 | 0.02971107 | 10.55611386 | 0.585920166 | 0.558221987 |
| ZNF442 | -0.070196333 | 0.417051255 | -0.584508909 | 0.559169972 |
| RP1-203P18.1 | 0.027434535 | 4.305727139 | 0.584497702 | 0.559177503 |
| FRK | 0.112887003 | -5.166947746 | 0.584299348 | 0.559310807 |
| RP5-1109J22.1 | -0.073135041 | 0.431848269 | -0.583987912 | 0.55952014 |
| CTD-3214H19.12 | 0.059391106 | 0.590369991 | 0.583851361 | 0.559611935 |
| C20orf166-AS1 | -0.197617509 | 0.128541074 | -0.583653296 | 0.559745095 |
| NUDT17 | -0.032716043 | 4.355707015 | -0.583514708 | 0.559838278 |
| DAPK3 | -0.018530038 | 5.04014691 | -0.582820965 | 0.560304845 |
| DNM1P46 | 0.066586037 | 0.728878257 | 0.582366356 | 0.560610688 |
| EIF1AX | 0.026449435 | 6.057391197 | 0.58220162 | 0.560721536 |
| CNRIP1 | 0.125615901 | 2.337359193 | 0.582180023 | 0.560736069 |
| PEX26 | 0.020196229 | 5.210711763 | 0.581641185 | 0.561098723 |
| RAB13 | 0.078026554 | 5.32675052 | 0.581519825 | 0.561180417 |
| RP11-96K19.4 | 0.051369633 | -0.449209375 | 0.580895449 | 0.561600813 |
| RPS4XP11 | -0.053749651 | 8.374322592 | -0.580668649 | 0.561753556 |
| MPP7 | 0.070844361 | 4.289473872 | 0.58054442 | 0.561837229 |
| SUB1P3 | 0.034731968 | 4.140220312 | 0.579898488 | 0.562272388 |
| RP11-736N17.4 | 0.084136238 | -2.138780385 | 0.579855864 | 0.562301109 |
| PPIB | 0.026790195 | 9.298684395 | 0.579829377 | 0.562318957 |
| STK17B | 0.057766118 | 7.9522098 | 0.579806335 | 0.562334484 |
| NPDC1 | -0.112064043 | 4.981133399 | -0.579494742 | 0.562544471 |
| GAR1 | -0.028564417 | 5.779460496 | -0.579420054 | 0.56259481 |
| C1orf141 | -0.095462989 | -3.363898521 | -0.579367649 | 0.562630132 |
| STRC | -0.081716788 | -3.102510952 | -0.578801522 | 0.563011778 |
| ERBB3 | 0.073300861 | -3.505004144 | 0.578747056 | 0.563048502 |
| RP11-182N22.7 | 0.060768789 | -0.720204391 | 0.578562814 | 0.563172737 |
| RP11-397G5.2 | -0.043299406 | 0.95393755 | -0.578511475 | 0.563207358 |
| IL22RA2 | 0.106894959 | -2.430928502 | 0.57825992 | 0.563377008 |
| RP11-118D22.3 | -0.058087796 | -0.674781144 | -0.578122929 | 0.563469407 |
| RP11-692N5.1 | -0.057367154 | 0.069449984 | -0.577457297 | 0.56391847 |
| HLA-DMB | -0.094053249 | 4.887021418 | -0.577305534 | 0.56402088 |
| PLEKHA7 | 0.074335399 | 0.1291526 | 0.57694562 | 0.564263786 |
| SNRPFP1 | 0.036750013 | 6.375584014 | 0.576934527 | 0.564271274 |
| BAI2 | 0.073093245 | -2.173917158 | 0.576588789 | 0.564504661 |
| RP11-165M1.2 | -0.068080684 | -0.408417031 | -0.576261232 | 0.564725819 |
| OPLAH | 0.090215513 | 2.547874092 | 0.576175697 | 0.564783577 |
| VIT | -0.101845229 | -2.700243177 | -0.576021549 | 0.564887674 |
| MRPL16 | 0.027160097 | 6.48993308 | 0.575757573 | 0.565065959 |
| C10orf131 | -0.064525926 | -0.84608068 | -0.575661578 | 0.565130798 |
| FOXO6 | -0.083129365 | -0.550127039 | -0.575551986 | 0.565204827 |
| RP11-927P21.4 | 0.047401908 | 4.110011443 | 0.575476329 | 0.565255936 |
| AP2A1 | 0.022921457 | 6.022110108 | 0.575465463 | 0.565263276 |
| NUCKS1 | -0.033799719 | 7.34030656 | -0.575441369 | 0.565279553 |
| RP11-862L9.3 | 0.057669471 | -1.160261308 | 0.575010048 | 0.565570971 |
| RP11-77K12.3 | -0.055120952 | 1.677561683 | -0.574818241 | 0.565700587 |
| FBL | 0.035367871 | 8.582445745 | 0.574740277 | 0.565753276 |
| NUP35 | 0.029824661 | 2.637481818 | 0.574683785 | 0.565791456 |
| RP11-728B21.3 | -0.090798746 | -0.171569344 | -0.574507366 | 0.565910695 |
| RP11-195E2.4 | -0.105879646 | 1.404246956 | -0.574398036 | 0.565984596 |
| RP11-796G6.2 | -0.072352009 | -0.544184428 | -0.574265838 | 0.56607396 |
| RP11-377G16.2 | 0.073261936 | -0.622830693 | 0.574020717 | 0.566239677 |
| AC004014.4 | 0.058532562 | 0.549264972 | 0.573840483 | 0.566361541 |
| RP11-380G5.3 | -0.030855979 | 5.994047653 | -0.573609516 | 0.566517727 |
| ZEB2P1 | 0.066177797 | -1.003421636 | 0.573317219 | 0.566715415 |
| RP11-188P20.3 | -0.056949769 | 1.452239356 | -0.573159351 | 0.566822199 |
| BTNL8 | 0.118759752 | 0.313755594 | 0.573059949 | 0.566889441 |
| STX18-AS1 | -0.04793681 | 0.745352637 | -0.573008711 | 0.566924103 |
| TRAV8-2 | 0.078532791 | 0.160972162 | 0.573001004 | 0.566929317 |
| PNPT1P1 | -0.037854299 | 1.476136841 | -0.572766308 | 0.567088101 |
| CTB-174O21.2 | -0.071808883 | -0.978321395 | -0.572513085 | 0.567259444 |
| CTD-2331H12.5 | -0.054036347 | 1.321036687 | -0.571885101 | 0.567684474 |
| FOXJ1 | 0.083871848 | -0.968892077 | 0.57152715 | 0.56792681 |
| SERINC5 | 0.050950424 | 3.416757967 | 0.571192982 | 0.568153089 |
| CT45A1 | -0.150389262 | -1.797386945 | -0.571158391 | 0.568176515 |
| RP1-90J20.8 | 0.072292319 | -0.31630655 | 0.570906777 | 0.568346925 |
| RP11-290F20.3 | -0.126029469 | 4.316528934 | -0.570653626 | 0.568518401 |
| PLS1 | 0.095960642 | 1.360894896 | 0.570570538 | 0.568574688 |
| PCSK5 | -0.093529679 | -0.328602438 | -0.570524418 | 0.568605932 |
| AC002310.7 | -0.061479951 | -1.691494837 | -0.570476276 | 0.568638547 |
| CTD-2260A17.3 | 0.072177393 | -0.752053936 | 0.570417465 | 0.568678391 |
| DLG1 | -0.018997102 | 4.965857296 | -0.570234615 | 0.568802279 |
| CHI3L2 | -0.070960566 | 0.672844925 | -0.570198447 | 0.568826787 |
| TET2 | -0.029395884 | 5.991873306 | -0.570124961 | 0.568876581 |
| SPATC1 | -0.096959062 | 1.75920831 | -0.570040093 | 0.568934091 |
| RPL37P1 | 0.058618549 | -0.184782664 | 0.569306391 | 0.56943139 |
| AP000662.4 | -0.082899531 | -1.527395831 | -0.56879409 | 0.569778749 |
| UNC93B5 | -0.094699219 | -0.098815774 | -0.568595626 | 0.569913342 |
| MPHOSPH9 | -0.025370012 | 5.175612109 | -0.568474779 | 0.569995304 |
| SLX9 | -0.025712567 | 4.146048737 | -0.568406871 | 0.570041365 |
| RP11-777J24.1 | 0.086712002 | 0.726403307 | 0.568275517 | 0.570130464 |
| PDLIM1 | 0.088330247 | 7.539481758 | 0.568028395 | 0.570298107 |
| AC007318.5 | -0.025766247 | 7.514378023 | -0.567864977 | 0.57040898 |
| RPS2P5 | 0.025414358 | 11.03276769 | 0.567831789 | 0.570431498 |
| HIST1H2BE | -0.074016734 | 0.21739687 | -0.56748676 | 0.570665625 |
| HIPK4 | 0.070207145 | -2.109184756 | 0.567316888 | 0.570780913 |
| CBX5 | 0.034105882 | 5.830454263 | 0.566794028 | 0.571135833 |
| CAP1P2 | -0.044023163 | 3.224035985 | -0.566646087 | 0.571236276 |
| TTC26 | -0.047993611 | 1.95053831 | -0.566636672 | 0.571242668 |
| SPI1 | 0.042141253 | 9.446336666 | 0.56624773 | 0.571506776 |
| YLPM1 | 0.020173583 | 6.331061976 | 0.566047388 | 0.57164284 |
| CRNDE | -0.123980016 | 2.731694892 | -0.565601449 | 0.571945757 |
| SHISA4 | -0.086969443 | 0.822206425 | -0.565498176 | 0.572015919 |
| STAG3 | 0.087884882 | 0.263049576 | 0.565471694 | 0.572033912 |
| NOD2 | -0.089302024 | 3.911481421 | -0.565452428 | 0.572047001 |
| FAM83B | -0.099278112 | -2.868350968 | -0.56541426 | 0.572072934 |
| RAC1P2 | -0.030978949 | 8.428957489 | -0.564758351 | 0.572518666 |
| AC006129.2 | 0.081130503 | 4.445353535 | 0.56451936 | 0.572681117 |
| ADI1 | -0.025280736 | 5.593741649 | -0.564407489 | 0.572757167 |
| RHOT2 | 0.017852451 | 6.539010608 | 0.56434895 | 0.572796964 |
| ISG20L2 | 0.017610655 | 5.34093819 | 0.564294713 | 0.572833837 |
| KIF25-AS1 | 0.087188676 | -3.376619137 | 0.564220404 | 0.572884359 |
| TRO | 0.131156764 | 0.307548023 | 0.563516178 | 0.573363256 |
| PTOV1 | -0.023653432 | 5.786711592 | -0.563183124 | 0.573589809 |
| NDFIP2 | 0.092607748 | 1.421122985 | 0.562976227 | 0.573730568 |
| ZNF100 | 0.031688769 | 4.096394583 | 0.562788499 | 0.573858301 |
| GLRX3 | 0.017942759 | 5.625356031 | 0.562683223 | 0.573929937 |
| B9D2 | 0.028681444 | 4.71381618 | 0.562647502 | 0.573954245 |
| AC139452.2 | -0.055915284 | 0.25523573 | -0.562599018 | 0.573987239 |
| NOM1 | 0.027577903 | 4.281993792 | 0.561931779 | 0.574441394 |
| SASH3 | -0.039994487 | 8.235762126 | -0.561475681 | 0.574751933 |
| STMN1 | 0.057062923 | 6.803445316 | 0.560977106 | 0.575091485 |
| RP13-7D7.1 | -0.063931918 | -0.343124326 | -0.560840653 | 0.575184433 |
| FOXQ1 | 0.078035889 | -3.028506661 | 0.560684879 | 0.57529055 |
| GGT5 | -0.13101497 | 3.37637436 | -0.560338405 | 0.575526609 |
| RP11-471L13.3 | 0.044655097 | 3.899853789 | 0.560327597 | 0.575533973 |
| AP000525.9 | 0.140231651 | -2.89019288 | 0.560106082 | 0.575684921 |
| RP11-1000B6.5 | 0.059046113 | 1.171434729 | 0.560053989 | 0.575720421 |
| XCR1 | -0.085684403 | -3.577037908 | -0.560035483 | 0.575733033 |
| CD276 | 0.134786064 | 1.044262547 | 0.559279412 | 0.576248405 |
| AC007392.3 | 0.096356325 | -0.570849888 | 0.559267701 | 0.576256389 |
| OAS3 | -0.068156396 | 5.289827725 | -0.559171002 | 0.57632232 |
| STAG3L2 | 0.045038203 | 4.94140182 | 0.559107414 | 0.576365677 |
| NTSR1 | 0.115192358 | 1.115082579 | 0.558927461 | 0.576488385 |
| KCNH8 | 0.096825935 | -0.611062699 | 0.558661885 | 0.576669501 |
| GOLGA8M | -0.09468286 | 1.928295305 | -0.558595542 | 0.57671475 |
| SYNGR2 | -0.023042629 | 6.751160834 | -0.558515731 | 0.576769187 |
| CES4A | -0.100512084 | 1.268192725 | -0.558219348 | 0.57697136 |
| DDX43 | -0.128648246 | -1.029506254 | -0.557878504 | 0.577203904 |
| UHRF1 | 0.078410978 | 4.16413103 | 0.557743062 | 0.577296323 |
| RAB3GAP2 | -0.015944433 | 4.753858602 | -0.557658458 | 0.577354056 |
| SLCO3A1 | 0.040884628 | 4.800510506 | 0.55746858 | 0.577483637 |
| C19orf43 | 0.019074691 | 9.431822053 | 0.557362706 | 0.577555897 |
| CCDC126 | -0.044952047 | 3.32915289 | -0.557286465 | 0.577607934 |
| PIKFYVE | 0.018363911 | 6.387051636 | 0.557068332 | 0.57775683 |
| PKHD1L1 | -0.124114869 | -3.555539067 | -0.556941961 | 0.577843098 |
| SLAMF7 | -0.086714691 | 2.959316145 | -0.556790777 | 0.577946312 |
| CHST15 | -0.120877 | 4.221852229 | -0.556622456 | 0.578061237 |
| CCR3 | -0.107883404 | -0.013927094 | -0.555564148 | 0.578784066 |
| RP11-423H2.1 | -0.034770432 | 3.397364523 | -0.555518627 | 0.578815166 |
| NUDC | -0.023307602 | 6.870386162 | -0.555484792 | 0.578838283 |
| RWDD2A | 0.053345108 | 1.043039469 | 0.555313932 | 0.578955027 |
| MEIS2 | 0.122563734 | -2.937803034 | 0.555096222 | 0.579103796 |
| MAGI3 | 0.090707436 | 0.045210346 | 0.554701385 | 0.579373651 |
| ZNF467 | -0.068525692 | 5.895785962 | -0.554588598 | 0.579450747 |
| RP11-381K20.2 | 0.063588727 | 1.964603432 | 0.554346273 | 0.579616406 |
| SRRM2 | -0.021402619 | 9.554201673 | -0.554146439 | 0.579753033 |
| SCT | 0.084580454 | 0.000264177 | 0.554062274 | 0.579810582 |
| RNASE3 | 0.137540462 | 7.636470727 | 0.553755153 | 0.580020602 |
| CCHCR1 | -0.022580705 | 4.21409445 | -0.55374495 | 0.58002758 |
| IGHV1-3 | -0.090601378 | 1.138322958 | -0.55357357 | 0.580144791 |
| MTMR9 | -0.022107848 | 3.964121038 | -0.553529733 | 0.580174775 |
| SYCP2L | 0.12254364 | -1.378143131 | 0.553234627 | 0.580376637 |
| EMC10 | 0.022040632 | 7.119818856 | 0.553091001 | 0.580474894 |
| KIR3DL1 | 0.091788834 | -1.438165044 | 0.552806677 | 0.580669428 |
| TUB | -0.109053137 | -3.119078963 | -0.552805494 | 0.580670237 |
| BATF3 | -0.073697862 | 0.903264376 | -0.552467994 | 0.580901194 |
| FAM177B | 0.064593963 | -0.492053236 | 0.552396395 | 0.580950196 |
| ASMTL | -0.031188981 | 5.651941149 | -0.5522172 | 0.581072844 |
| ARL6IP6 | -0.022708656 | 5.391924036 | -0.552163929 | 0.581109308 |
| PARM1 | -0.085754388 | -0.246462931 | -0.551862327 | 0.58131577 |
| CABYR | 0.059235218 | -1.763809185 | 0.551599714 | 0.58149557 |
| RP11-1H8.3 | 0.079817177 | 2.383487079 | 0.551489193 | 0.581571247 |
| TCF3 | -0.033217474 | 6.396351847 | -0.551293021 | 0.581705583 |
| RP11-270C12.3 | -0.030650288 | 5.968344738 | -0.551002869 | 0.581904302 |
| RABGAP1L | -0.022902488 | 4.813454007 | -0.55086479 | 0.581998881 |
| PITPNM1 | -0.0405281 | 6.07596191 | -0.55032825 | 0.582366458 |
| MT1E | 0.105443207 | 1.504215223 | 0.54987108 | 0.582679745 |
| RP11-553N16.1 | 0.05574779 | 0.117121941 | 0.549753199 | 0.582760539 |
| LRRC37A16P | 0.071696225 | -2.434049398 | 0.549744984 | 0.58276617 |
| RASAL2 | 0.115596315 | 1.219362554 | 0.549715257 | 0.582786545 |
| RP11-353N14.5 | -0.099355402 | -3.320391086 | -0.549275178 | 0.583088221 |
| MIR155HG | 0.077845883 | 3.897130822 | 0.548695075 | 0.583485995 |
| FOXC1 | 0.18408097 | 2.524564552 | 0.547921468 | 0.584016652 |
| KLF4 | -0.102599234 | 6.279152576 | -0.547325917 | 0.584425324 |
| MIR5587 | -0.066456104 | 0.380512879 | -0.546657131 | 0.58488441 |
| RP11-313F23.4 | -0.080269951 | -0.96438988 | -0.546328249 | 0.585110232 |
| BANF1P2 | -0.054711979 | 0.956905823 | -0.546145925 | 0.585235439 |
| SLC30A4 | 0.073580329 | -0.689478515 | 0.546061694 | 0.585293287 |
| BAHD1 | 0.024252538 | 5.392352365 | 0.54597651 | 0.585351793 |
| FSCN1 | -0.076759787 | 6.374886618 | -0.545952819 | 0.585368065 |
| ATPIF1 | 0.028843114 | 7.147418465 | 0.545485521 | 0.585689063 |
| NFYC-AS1 | -0.035836074 | 2.107181645 | -0.545022674 | 0.586007085 |
| RP11-731I19.1 | 0.052984133 | 1.990413434 | 0.544937223 | 0.586065807 |
| CHCHD10 | -0.048106248 | 5.433620075 | -0.54467488 | 0.586246106 |
| DUSP15 | 0.092678181 | -1.519746819 | 0.544644859 | 0.586266741 |
| RP11-195E2.1 | -0.1053329 | 1.586074442 | -0.544541483 | 0.586337796 |
| RP11-103G8.2 | -0.050576205 | 2.824905247 | -0.544050413 | 0.586675386 |
| TRBV23-1 | -0.079662788 | 0.480584689 | -0.544035362 | 0.586685734 |
| SH3BGRL2 | 0.106513069 | 1.736871382 | 0.543680176 | 0.586929969 |
| CYP2E1 | -0.099022656 | -0.433027845 | -0.543660077 | 0.58694379 |
| MAGT1 | 0.021867253 | 6.145665813 | 0.543033445 | 0.587374797 |
| CNST | -0.042902546 | 5.995474828 | -0.542946003 | 0.587434953 |
| SYN2 | 0.067403892 | 0.027452883 | 0.542908577 | 0.5874607 |
| ATPAF1 | -0.023518757 | 4.671703952 | -0.542816227 | 0.587524237 |
| DNER | 0.073561217 | -3.454722505 | 0.542532976 | 0.587719132 |
| RP11-495K9.3 | 0.08796361 | -1.896840627 | 0.54247881 | 0.587756405 |
| PCDHGB4 | 0.096687453 | -1.311111177 | 0.542300835 | 0.587878883 |
| EIF3LP3 | 0.038966024 | 1.855572336 | 0.542132238 | 0.587994917 |
| TIAL1 | -0.017014644 | 6.959390153 | -0.542111017 | 0.588009523 |
| SCN8A | 0.097493917 | -0.806558947 | 0.542069601 | 0.588038029 |
| KIAA1549L | 0.105129203 | -5.445887216 | 0.5420333 | 0.588063015 |
| C6orf132 | 0.082720641 | -1.286554064 | 0.542025634 | 0.588068291 |
| GZMK | -0.088568534 | 3.460379986 | -0.541449667 | 0.588464796 |
| ZNF337-AS1 | -0.051914151 | 0.427916869 | -0.541108691 | 0.588699588 |
| EDAR | -0.088330478 | -1.714775955 | -0.540804111 | 0.588909354 |
| PTP4A3 | -0.083197055 | 5.418145201 | -0.540443952 | 0.589157443 |
| AC016700.6 | 0.039169035 | 4.490889538 | 0.539961821 | 0.589489626 |
| C12orf42 | 0.09205078 | -1.820431877 | 0.539775199 | 0.58961823 |
| CTAGE3P | -0.045690331 | -0.428038796 | -0.539313152 | 0.589936688 |
| RASL11A | -0.064371918 | 2.887661959 | -0.53909595 | 0.590086418 |
| PCBP4 | 0.062070668 | 2.342836322 | 0.539046606 | 0.590120437 |
| LCAT | -0.048757338 | 3.489435169 | -0.539013336 | 0.590143374 |
| MTND1P23 | 0.060402359 | 6.866164522 | 0.538882742 | 0.590233413 |
| FLRT1 | 0.091394062 | -3.011954202 | 0.538852986 | 0.590253929 |
| SRSF9 | -0.013957618 | 7.245781225 | -0.538556031 | 0.590458693 |
| HIST1H2AJ | -0.088560041 | 0.209844256 | -0.538525214 | 0.590479945 |
| PA2G4P1 | -0.033020139 | 4.656572274 | -0.53835211 | 0.590599326 |
| MIXL1 | 0.082783246 | -0.758723003 | 0.538269173 | 0.590656527 |
| RANBP10 | -0.022913896 | 5.338823684 | -0.538173922 | 0.590722224 |
| PIK3C2B | -0.058915848 | 3.859093914 | -0.538034197 | 0.590818602 |
| RP11-521L9.2 | -0.056056392 | -0.459500126 | -0.537991399 | 0.590848124 |
| SEZ6L | -0.129380075 | -0.024367915 | -0.537214422 | 0.591384204 |
| RPS24P17 | 0.046854234 | 1.975026279 | 0.537039857 | 0.591504677 |
| TSSK3 | -0.057652956 | -0.85910983 | -0.536734738 | 0.591715277 |
| RP11-181C3.1 | 0.048479912 | 0.148187815 | 0.536696146 | 0.591741916 |
| AC010677.5 | -0.047428047 | 1.032848583 | -0.536533055 | 0.591854502 |
| hsa-mir-4538 | 0.076796732 | -0.434401667 | 0.536453752 | 0.59190925 |
| RP11-390F4.3 | 0.092097878 | 0.10113966 | 0.53630479 | 0.592012095 |
| FTO-IT1 | -0.055363745 | 0.652651152 | -0.536184929 | 0.592094854 |
| ACOT8 | -0.016824558 | 4.267479303 | -0.535954976 | 0.592253642 |
| SYCE1 | 0.090726427 | -2.547620062 | 0.535686515 | 0.592439047 |
| DCBLD2 | -0.099818956 | 0.091942659 | -0.535647594 | 0.592465928 |
| RP11-567J20.3 | -0.077635498 | -0.809539589 | -0.535407784 | 0.592631571 |
| RP11-291B21.2 | -0.085937101 | 1.442954535 | -0.534908666 | 0.592976393 |
| C19orf10 | 0.027387953 | 7.28999351 | 0.534849815 | 0.593017057 |
| ERP29 | -0.024415866 | 8.441350421 | -0.534779547 | 0.593065612 |
| TUBG2 | -0.048431025 | 3.443412847 | -0.534169057 | 0.593487531 |
| ABHD17AP6 | 0.056124068 | 2.155012779 | 0.533925677 | 0.593655773 |
| CPA6 | -0.100262926 | -0.263813742 | -0.533703229 | 0.593809565 |
| LAIR2 | -0.062110345 | 2.541925812 | -0.533685037 | 0.593822143 |
| MATN2 | -0.094893851 | 0.127594138 | -0.533670627 | 0.593832106 |
| RP11-15H20.6 | 0.045030102 | 1.823787062 | 0.533656342 | 0.593841983 |
| DPM2 | 0.021586313 | 5.093016104 | 0.533620635 | 0.593866671 |
| RPL5P17 | 0.029912378 | 5.122109547 | 0.533591504 | 0.593886813 |
| LRGUK | 0.078056953 | -0.546619244 | 0.533564415 | 0.593905545 |
| TCN1 | 0.130969949 | 4.981476952 | 0.533504779 | 0.593946781 |
| PID1 | -0.134636061 | 0.479639034 | -0.533487775 | 0.593958538 |
| CNOT8 | 0.024487786 | 7.1148827 | 0.533040298 | 0.594267997 |
| ARL16 | -0.023649901 | 5.117833804 | -0.532939986 | 0.594337379 |
| LAS1L | 0.019899581 | 5.532662231 | 0.532627612 | 0.594553461 |
| RANP6 | 0.039715826 | 3.704297625 | 0.532310266 | 0.594773019 |
| LYSMD1 | -0.069387778 | 0.423112984 | -0.531975462 | 0.595004696 |
| SERPING1 | -0.097105198 | 5.336947832 | -0.531759703 | 0.595154018 |
| HLA-F | 0.06752057 | 5.853805296 | 0.531543296 | 0.595303806 |
| CTC-546K23.1 | 0.122645825 | -1.194064945 | 0.53139715 | 0.595404972 |
| MED15P6 | 0.049195174 | -0.707341328 | 0.531395809 | 0.5954059 |
| RPA4 | -0.051816863 | -0.109632761 | -0.531304768 | 0.595468925 |
| AC084125.4 | 0.053909259 | 0.436172669 | 0.53123203 | 0.595519282 |
| RP11-54A4.2 | -0.068289214 | 3.063464541 | -0.530536169 | 0.596001125 |
| BLK | -0.103587393 | 0.852818723 | -0.530492568 | 0.596031321 |
| RP11-420K14.2 | -0.150888962 | 0.978495226 | -0.530483042 | 0.596037919 |
| TRDJ1 | -0.074521695 | -0.073248663 | -0.530058767 | 0.5963318 |
| C9orf37 | -0.023344538 | 4.131000937 | -0.529384825 | 0.596798753 |
| SCARNA17 | 0.049922258 | 3.894472617 | 0.52852442 | 0.597395144 |
| MBNL1 | -0.024964062 | 8.189738616 | -0.528429454 | 0.597460986 |
| MPC1 | -0.01871712 | 6.374086609 | -0.528330661 | 0.597529485 |
| EIF3LP2 | -0.029772672 | 4.279228063 | -0.528196893 | 0.59762224 |
| SIGLEC17P | 0.097750561 | 3.384700441 | 0.528118683 | 0.597676475 |
| MPHOSPH10 | -0.019853752 | 5.319141686 | -0.528103365 | 0.597687097 |
| RPL17P43 | 0.063543453 | 1.812033964 | 0.527988349 | 0.597766859 |
| POLE4 | 0.037938063 | 4.839955058 | 0.527885684 | 0.59783806 |
| S1PR3 | 0.114209742 | 0.129908935 | 0.527634019 | 0.598012611 |
| ATP1B1 | -0.070599261 | 4.959566773 | -0.527606931 | 0.5980314 |
| CLEC14A | -0.141538739 | 0.056724473 | -0.527577827 | 0.598051589 |
| ADCY6 | -0.093717471 | 3.170234923 | -0.52756178 | 0.59806272 |
| CFL1P1 | -0.050994067 | -0.193904463 | -0.527256695 | 0.598274363 |
| AC083899.3 | 0.028654364 | 2.776746934 | 0.527094063 | 0.598387198 |
| CRHBP | 0.105294018 | 2.612298584 | 0.527017874 | 0.598440061 |
| CTC-548K16.6 | -0.056471179 | 0.59877754 | -0.526694328 | 0.598664576 |
| UGT8 | -0.085732243 | -2.144898085 | -0.526655315 | 0.59869165 |
| CCR12P | -0.066317308 | -1.414984551 | -0.526611424 | 0.59872211 |
| BMS1P10 | 0.078042843 | 0.353337105 | 0.52614823 | 0.599043611 |
| RAVER2 | 0.058772359 | 4.599517127 | 0.525985484 | 0.599156591 |
| RP11-887P2.3 | 0.043987237 | 5.9973139 | 0.525737694 | 0.599328627 |
| RP11-231I16.1 | -0.06811499 | -2.91138447 | -0.525435591 | 0.599538403 |
| KHDRBS2 | 0.099550906 | -0.188054793 | 0.525342364 | 0.599603145 |
| CTC-398G3.2 | 0.053267958 | 0.917097225 | 0.525326631 | 0.599614071 |
| CTC-451P13.1 | 0.030033374 | 5.69468784 | 0.525175422 | 0.599719087 |
| ALDOAP2 | -0.031285901 | 6.320948577 | -0.525101971 | 0.599770102 |
| GSTP1 | -0.031641601 | 9.410644907 | -0.524957786 | 0.599870252 |
| NUP188 | -0.023296163 | 5.709595525 | -0.524890953 | 0.599916675 |
| NEDD4 | -0.053106223 | 4.088386125 | -0.524560845 | 0.600146001 |
| TRNP1 | -0.10290728 | 0.075511406 | -0.524474933 | 0.60020569 |
| RNPC3 | -0.02801093 | 4.861287007 | -0.524176608 | 0.600412981 |
| RP11-800A3.7 | 0.062415034 | 0.284301156 | 0.524102653 | 0.600464373 |
| HIST1H4H | 0.050403904 | 3.622373809 | 0.523967242 | 0.600558476 |
| SORCS2 | -0.111305799 | -0.236279012 | -0.523930571 | 0.600583963 |
| RPL15 | -0.025872384 | 9.551644533 | -0.52391605 | 0.600594054 |
| UBE2E1 | -0.024569833 | 7.228466141 | -0.523596309 | 0.600816291 |
| HYLS1 | -0.044179855 | 2.044384367 | -0.52350789 | 0.600877754 |
| RPL7P26 | 0.046752492 | 0.848560144 | 0.523293054 | 0.601027104 |
| COLEC11 | -0.070440195 | -2.646325047 | -0.52328797 | 0.601030638 |
| BTF3L4P1 | -0.025100549 | 3.27479256 | -0.522897351 | 0.601302234 |
| NRADDP | 0.051190633 | 1.354371925 | 0.522830438 | 0.601348764 |
| PCDHGA6 | 0.085782296 | -0.179270544 | 0.522464621 | 0.601603173 |
| GMPSP1 | 0.026985511 | 5.955765794 | 0.522441016 | 0.601619591 |
| KATNB1 | -0.026447196 | 5.213642856 | -0.522278453 | 0.601732664 |
| ERCC6L2 | 0.015762267 | 3.899905949 | 0.522209395 | 0.601780701 |
| CYP19A1 | 0.101617395 | -3.672468027 | 0.521326132 | 0.602395254 |
| DBP | -0.042126332 | 2.715966496 | -0.521298649 | 0.602414381 |
| USP25 | 0.021642942 | 5.66288711 | 0.521232252 | 0.602460591 |
| EPHX2 | -0.081870981 | 0.297889811 | -0.520801309 | 0.602760549 |
| RP11-445P19.3 | 0.068869984 | -1.058836934 | 0.520404985 | 0.60303647 |
| TMEM141 | -0.026334006 | 3.619979034 | -0.520000412 | 0.603318192 |
| RP11-80H8.4 | -0.067876529 | 0.974549459 | -0.519941926 | 0.603358923 |
| AC074389.9 | -0.08914833 | -0.846529942 | -0.519503907 | 0.603664012 |
| RP4-773N10.4 | -0.027778391 | 2.951190604 | -0.519179645 | 0.603889911 |
| PHC1P1 | -0.044891279 | 5.193197196 | -0.518974361 | 0.604032944 |
| ZNF667-AS1 | -0.119380232 | 1.547704769 | -0.518948226 | 0.604051154 |
| TANK | 0.027731526 | 5.976306852 | 0.518789718 | 0.604161607 |
| AC074338.4 | 0.057788781 | 0.107584353 | 0.518632969 | 0.604270844 |
| BTF3 | 0.022009693 | 10.04191571 | 0.517903886 | 0.604779048 |
| HLA-DRB6 | -0.098611858 | 4.8201726 | -0.51788396 | 0.60479294 |
| TRAT1 | 0.088734567 | 3.499023527 | 0.517772607 | 0.604870576 |
| UNC50 | -0.019423026 | 5.773612867 | -0.517722011 | 0.604905853 |
| TMEM38A | -0.094079509 | 1.40728068 | -0.517534997 | 0.605036254 |
| TRIM24 | -0.031929493 | 5.423056706 | -0.517134202 | 0.605315762 |
| LINC01023 | -0.053846944 | 1.913681062 | -0.516743727 | 0.605588129 |
| ABHD14B | -0.028293229 | 6.304018604 | -0.516596803 | 0.605690626 |
| AL136376.1 | 0.069304038 | 0.235947106 | 0.516313532 | 0.605888265 |
| RP11-93O14.2 | 0.051159619 | 0.284492032 | 0.51629979 | 0.605897853 |
| TCFL5 | -0.027857186 | 4.217841152 | -0.516255273 | 0.605928916 |
| SRRM5 | 0.051880962 | 0.784927349 | 0.516193789 | 0.605971818 |
| SAMD4A | 0.070884629 | -0.250557374 | 0.516068516 | 0.606059236 |
| STAC | 0.122916857 | -1.903171296 | 0.515974287 | 0.606124995 |
| RP11-598F7.3 | -0.107054213 | 0.178903373 | -0.515864761 | 0.606201432 |
| DNAH10 | -0.069234483 | 0.276604799 | -0.515506255 | 0.606451662 |
| SLC9A9 | -0.076237351 | 2.549060206 | -0.515211279 | 0.606657584 |
| SV2C | -0.079277523 | -3.496698573 | -0.514987908 | 0.606813539 |
| AHI1-DT | -0.065607482 | -2.035434867 | -0.514951776 | 0.606838768 |
| TECTA | -0.053077825 | -1.061846037 | -0.51477229 | 0.606964098 |
| C19orf84 | 0.097002245 | 0.051317985 | 0.514168938 | 0.60738549 |
| USP2-AS1 | -0.072933744 | -2.53413474 | -0.513752186 | 0.607676633 |
| HEATR1 | -0.021066642 | 5.034010091 | -0.513611439 | 0.607774972 |
| CLCN4 | 0.10195459 | 0.061567544 | 0.513425999 | 0.60790455 |
| OGFOD3 | -0.022740656 | 3.768179714 | -0.513010755 | 0.608194751 |
| RP11-290L1.2 | 0.07483902 | 0.304529343 | 0.512073748 | 0.608849822 |
| RHOD | 0.065344524 | -1.999758886 | 0.511833632 | 0.609017739 |
| TWIST2 | -0.068311786 | -1.92907163 | -0.511766804 | 0.609064477 |
| MARCO | -0.13898564 | 2.319182587 | -0.511603916 | 0.609178404 |
| RIC8A | -0.012843921 | 5.846196502 | -0.511282672 | 0.609403116 |
| ANKRD18CP | 0.064444383 | -1.900639682 | 0.511258976 | 0.609419692 |
| DLG2 | -0.050714866 | -1.708371586 | -0.511138598 | 0.609503907 |
| PGM1 | 0.046137484 | 5.410012056 | 0.510981427 | 0.609613871 |
| SYNGR1 | 0.051154916 | 4.848123348 | 0.510970976 | 0.609621183 |
| NIPA1 | 0.05416054 | 3.56897058 | 0.510870817 | 0.609691264 |
| SMC1B | -0.11613177 | -1.083706704 | -0.51082711 | 0.609721847 |
| GLI1 | 0.058726939 | -1.810853755 | 0.510273969 | 0.610108947 |
| TPT1P6 | -0.050342835 | 4.186418278 | -0.510218322 | 0.610147896 |
| LSAMP | 0.132519064 | -4.345635392 | 0.509403483 | 0.610718355 |
| SEMA3G | -0.078126855 | -0.92186745 | -0.509338138 | 0.610764113 |
| RP11-350G8.9 | 0.053561192 | -0.249708175 | 0.50913044 | 0.610909562 |
| CTD-3232M19.2 | -0.060462183 | -0.187696476 | -0.509021375 | 0.610985946 |
| PYHIN1 | -0.081616607 | 3.043397101 | -0.508797069 | 0.611143052 |
| SKA2 | -0.036643619 | 5.044575334 | -0.508650547 | 0.611245688 |
| BTG2 | 0.053817168 | 8.890064515 | 0.508325864 | 0.611473148 |
| RP11-356I2.4 | -0.053611861 | 2.032690505 | -0.508284393 | 0.611502204 |
| HSP90AA6P | 0.052568964 | 2.288328464 | 0.508184822 | 0.611571968 |
| UBA52P5 | 0.05487319 | 0.543720382 | 0.507893614 | 0.611776024 |
| RP11-83J16.1 | 0.046559517 | 5.304841123 | 0.507215204 | 0.612251517 |
| NOL7 | -0.01243301 | 7.006501432 | -0.507199703 | 0.612262383 |
| CCDC92 | -0.038380227 | 4.036422047 | -0.50705376 | 0.612364696 |
| TREX2 | 0.045294339 | -0.088000254 | 0.506823393 | 0.612526209 |
| ABHD14A | -0.03631219 | 3.806135376 | -0.506234309 | 0.612939309 |
| EIF3D | 0.023470327 | 8.476590398 | 0.506169081 | 0.612985059 |
| ZFAND1 | 0.026185194 | 5.77469455 | 0.505634773 | 0.613359866 |
| RP11-268J15.5 | 0.077254037 | 3.7305678 | 0.505083641 | 0.613746581 |
| ATP6V1F | 0.024809708 | 8.298290752 | 0.504921514 | 0.613860362 |
| RP11-71H17.9 | 0.046602734 | 1.704575494 | 0.504916183 | 0.613864103 |
| GOLGA8A | 0.067516672 | 5.152948403 | 0.504453174 | 0.614189096 |
| MRPL4 | 0.021666008 | 5.433056417 | 0.504170972 | 0.614387214 |
| RP4-669L17.10 | 0.049456629 | -0.324966606 | 0.503941514 | 0.614548325 |
| C2CD2L | -0.026473206 | 4.203579074 | -0.503879609 | 0.614591794 |
| NCOA7 | -0.045511867 | 6.169350171 | -0.503678288 | 0.614733169 |
| PLCH2 | -0.06793414 | 0.134218073 | -0.50365458 | 0.614749818 |
| KIF24 | 0.060837123 | 1.996172871 | 0.503293966 | 0.615003093 |
| LINC01138 | -0.061544607 | -0.691833263 | -0.503210357 | 0.615061822 |
| MT-TA | -0.058229441 | 3.176949308 | -0.502990141 | 0.615216519 |
| MINPP1 | -0.05648696 | 4.231310871 | -0.502886704 | 0.615289186 |
| URB2 | 0.027683479 | 3.395556655 | 0.502590193 | 0.615497516 |
| NUBP2 | -0.016834493 | 4.512291115 | -0.502329699 | 0.615680566 |
| PTH1R | 0.102455413 | -1.851671167 | 0.502115185 | 0.615831323 |
| HEXB | -0.043558521 | 6.300897704 | -0.50209318 | 0.615846789 |
| IGBP1P1 | -0.02603479 | 2.805357372 | -0.501794613 | 0.616056647 |
| RNU1-134P | 0.058171022 | -0.159852028 | 0.501680076 | 0.616137162 |
| MATR3 | 0.02187173 | 7.518604341 | 0.501634951 | 0.616168883 |
| PRDX6 | -0.022858543 | 8.337404447 | -0.501603033 | 0.616191322 |
| RP11-477G18.2 | -0.049468581 | 0.315182475 | -0.501002326 | 0.616613686 |
| DGAT1 | -0.02427997 | 6.522382739 | -0.50020864 | 0.61717193 |
| OGDHL | -0.093459763 | -2.939568923 | -0.500122521 | 0.617232516 |
| RP11-182J1.12 | -0.058604107 | 0.380677041 | -0.499677533 | 0.617545612 |
| TMC5 | 0.073279018 | -3.510260458 | 0.499552657 | 0.617633488 |
| RPS24 | 0.025269459 | 9.826283756 | 0.499441954 | 0.617711395 |
| MIR635 | -0.056083953 | 0.407378991 | -0.49943763 | 0.617714438 |
| AC022201.5 | -0.053445822 | 0.077734961 | -0.499218913 | 0.617868372 |
| MCOLN1 | 0.03309113 | 4.646069353 | 0.498853957 | 0.618125268 |
| ALDH2 | -0.088047365 | 5.974151254 | -0.498836214 | 0.618137759 |
| LAT | 0.056936715 | -0.020077145 | 0.498820049 | 0.618149139 |
| RGAG4 | -0.075491417 | 2.096326797 | -0.498208739 | 0.618579559 |
| BLM | 0.034469141 | 4.221362256 | 0.498169329 | 0.618607311 |
| RPL35P5 | -0.02927131 | 7.996218111 | -0.497721904 | 0.61892243 |
| TXNIP | 0.043070142 | 10.61782682 | 0.497303363 | 0.619217268 |
| RPL17P26 | 0.047940442 | 0.654695784 | 0.497134194 | 0.619336456 |
| RP11-2E11.9 | -0.086916144 | 0.436856897 | -0.496789792 | 0.619579135 |
| SRSF3 | -0.020010728 | 8.285749498 | -0.496768056 | 0.619594453 |
| OXT | -0.05953981 | -0.626235571 | -0.496599436 | 0.619713285 |
| SLX1B | 0.056132384 | -1.327251914 | 0.4963885 | 0.619861954 |
| NEB | 0.054211723 | 0.369087002 | 0.496192332 | 0.620000228 |
| KB-1572G7.2 | 0.046715714 | -1.288621456 | 0.495780826 | 0.620290331 |
| CHMP2A | 0.020699707 | 7.440480812 | 0.495608891 | 0.620411559 |
| TMEM194A | -0.029259783 | 4.91363765 | -0.495526848 | 0.62046941 |
| RP11-446H13.2 | 0.054740241 | 0.063891101 | 0.495034319 | 0.620816755 |
| SNRPGP10 | -0.034068341 | 5.595392901 | -0.494684465 | 0.621063533 |
| RSRC1 | -0.020547506 | 4.219345266 | -0.494664987 | 0.621077274 |
| FADD | 0.032831933 | 5.172373396 | 0.494654493 | 0.621084676 |
| WWC1 | 0.088681815 | -2.272987933 | 0.494327188 | 0.621315592 |
| IGHV4-55 | 0.073892446 | -0.095441059 | 0.494002309 | 0.621544831 |
| ELK3 | -0.036900309 | 6.068839803 | -0.493775877 | 0.621704628 |
| AL133458.1 | -0.049045362 | 5.370344533 | -0.493051346 | 0.622216059 |
| IZUMO4 | -0.039228825 | 2.720098527 | -0.492516775 | 0.622593519 |
| AF196970.3 | -0.062986807 | 0.054075492 | -0.491830083 | 0.623078539 |
| ATP11C | -0.020304671 | 4.442290554 | -0.491803943 | 0.623097005 |
| NR5A2 | 0.114506486 | -3.050177158 | 0.490811221 | 0.623798476 |
| OSBPL1A | -0.061611287 | 3.960139725 | -0.490731738 | 0.623854654 |
| ERGIC3 | 0.019759627 | 6.779787851 | 0.490359609 | 0.624117704 |
| SUMO2P3 | -0.041912317 | 0.654323778 | -0.490252022 | 0.624193764 |
| SEMA5A | -0.116554222 | -3.226583484 | -0.490206036 | 0.624226275 |
| YWHAZP2 | 0.023871113 | 6.20270893 | 0.489618888 | 0.624641446 |
| RP11-384O8.1 | -0.091750536 | -0.58900837 | -0.489313778 | 0.624857236 |
| PARP14 | 0.033404288 | 6.030397686 | 0.489273452 | 0.624885759 |
| RP11-443B20.1 | -0.037291372 | 2.468600693 | -0.489195666 | 0.62494078 |
| GOLM1 | 0.089370682 | 2.955226862 | 0.488875116 | 0.625167538 |
| RP3-496C20.1 | -0.043130997 | 0.087834946 | -0.488356319 | 0.625534611 |
| SGSM1 | 0.068813906 | -2.320283551 | 0.488355796 | 0.625534981 |
| KIAA1549 | -0.082970475 | 2.087925776 | -0.488270285 | 0.625595493 |
| RP11-56M3.1 | 0.061613428 | -0.306983879 | 0.488074182 | 0.625734275 |
| RP3-400B16.1 | -0.059183357 | 0.136160373 | -0.487608671 | 0.626063771 |
| GRWD1 | -0.025946266 | 4.463773837 | -0.487534999 | 0.626115924 |
| SAP18 | -0.01640654 | 7.389936266 | -0.487369921 | 0.626232791 |
| VARS | -0.024047901 | 6.236792167 | -0.487222153 | 0.626337411 |
| UBA52P3 | 0.051948923 | -0.003355972 | 0.487194405 | 0.626357058 |
| CAMK2G | -0.023902155 | 5.914697533 | -0.486659081 | 0.626736138 |
| GCNT1P3 | -0.074522929 | 0.169220196 | -0.486539348 | 0.626820938 |
| PGLYRP2 | 0.072174363 | -1.663715639 | 0.485928185 | 0.627253868 |
| FAM136A | -0.020999042 | 6.527941957 | -0.485818886 | 0.627331306 |
| NPIPB11 | -0.035891069 | 2.947910241 | -0.485763401 | 0.627370618 |
| RFX8 | -0.092981447 | 4.025782657 | -0.485641007 | 0.627457341 |
| ARFGAP2 | 0.01573018 | 6.529691286 | 0.485488424 | 0.627565462 |
| RPL7P11 | -0.061021943 | 2.674863205 | -0.485055012 | 0.627872622 |
| PAPSS2 | 0.093349489 | 1.919593678 | 0.484640085 | 0.628166743 |
| GSPT2 | 0.051342992 | 3.877938777 | 0.484583911 | 0.628206566 |
| KIF1C | -0.049389021 | 4.074732377 | -0.484121945 | 0.628534108 |
| LGALS8 | 0.016036464 | 6.396547152 | 0.483744183 | 0.628802003 |
| RP4-684O24.5 | 0.069882627 | -2.108808704 | 0.483505894 | 0.628971014 |
| RP11-274B18.2 | -0.091641072 | 2.007385462 | -0.483475396 | 0.628992647 |
| C15orf27 | -0.082414325 | -2.840235752 | -0.483326891 | 0.629097988 |
| RPL13 | 0.025726111 | 10.12780275 | 0.483148071 | 0.629224843 |
| RP11-658F2.3 | 0.054879828 | -0.62245858 | 0.483090765 | 0.629265499 |
| IL12RB1 | -0.052937858 | 3.817375314 | -0.483056453 | 0.629289841 |
| RP11-317J10.2 | -0.069423589 | 0.677173613 | -0.482884191 | 0.629412061 |
| CLU | -0.103564378 | 5.086654624 | -0.482726635 | 0.629523855 |
| TMEM200A | -0.086174844 | -1.759082963 | -0.48238798 | 0.629764176 |
| RP13-735L24.1 | 0.066581438 | -2.391951787 | 0.482145649 | 0.629936167 |
| SECTM1 | -0.095570691 | 4.081045893 | -0.481809997 | 0.630174424 |
| KCNJ5-AS1 | -0.067191338 | 2.04167247 | -0.481737227 | 0.630226084 |
| GLRX2 | -0.023713608 | 3.895807093 | -0.481735882 | 0.630227039 |
| PLEKHG4B | 0.138346214 | -4.688201512 | 0.481379295 | 0.630480208 |
| RP1-212P9.3 | -0.054599891 | 0.409366474 | -0.481284016 | 0.630547861 |
| GNLY | 0.082921907 | 4.91813641 | 0.48119248 | 0.63061286 |
| CTCF | -0.016789729 | 6.705730035 | -0.48081261 | 0.630882631 |
| RAET1G | 0.073337974 | -1.900797993 | 0.480553653 | 0.631066563 |
| AC012309.5 | -0.08583055 | -0.488739551 | -0.480371074 | 0.631196258 |
| SV2A | -0.072195407 | 4.242916099 | -0.479776974 | 0.631618358 |
| CARD14 | 0.0685144 | -4.146456555 | 0.479374423 | 0.631904433 |
| EEF1A1P30 | -0.044726024 | -0.063417316 | -0.4789069 | 0.63223675 |
| STAT1 | -0.034676489 | 6.90147913 | -0.478170197 | 0.632760553 |
| GLA | -0.024252503 | 6.922616216 | -0.477949864 | 0.632917248 |
| XKR6 | 0.079348854 | -0.74946384 | 0.477672344 | 0.633114636 |
| APC | 0.019040133 | 4.717925625 | 0.477380889 | 0.633321964 |
| PLXNC1 | 0.066444411 | 5.183626137 | 0.477332231 | 0.63335658 |
| PPP1CC | 0.017516592 | 6.899767697 | 0.477117261 | 0.633509521 |
| DOCK10 | 0.05046549 | 4.851509913 | 0.476855206 | 0.633695984 |
| UNK | 0.021625268 | 5.432581141 | 0.476813237 | 0.633725848 |
| LMNB2 | -0.028682959 | 6.342052083 | -0.476178874 | 0.634177328 |
| SKIV2L | -0.013068857 | 5.692127743 | -0.475881874 | 0.634388751 |
| RNU6-195P | 0.040763294 | 1.5541073 | 0.475173573 | 0.634893084 |
| SMIM6 | 0.061935692 | 0.512703705 | 0.475110408 | 0.634938069 |
| MTPN | 0.021732453 | 8.384885866 | 0.474656024 | 0.635261704 |
| TENM1 | 0.084103816 | -0.619586377 | 0.474193934 | 0.6355909 |
| TBL1X | -0.05009034 | 4.722067799 | -0.47415358 | 0.635619652 |
| TRPV5 | -0.066451498 | -2.657794748 | -0.473525848 | 0.636066977 |
| RP11-366M4.11 | 0.070993637 | -1.439596283 | 0.472077268 | 0.637099751 |
| AC004057.1 | 0.048377526 | 7.350743124 | 0.471769031 | 0.637319602 |
| AL133493.2 | 0.104941418 | 0.72372855 | 0.471504433 | 0.637508352 |
| RP11-428G5.2 | -0.049234592 | 0.369905943 | -0.471157631 | 0.637755779 |
| PPP1R27 | -0.116648082 | 2.318500315 | -0.4707623 | 0.638037878 |
| BMP3 | -0.114211446 | -3.050000309 | -0.470683839 | 0.638093872 |
| ZFYVE28 | 0.054953322 | 1.395338846 | 0.47003345 | 0.638558106 |
| EIF3L | -0.024473682 | 9.213410506 | -0.469628619 | 0.638847138 |
| CTB-118N6.1 | 0.051706808 | 2.049207539 | 0.469502218 | 0.638937395 |
| ITGAX | 0.060574495 | 7.092406219 | 0.469501039 | 0.638938236 |
| C20orf195 | -0.059903664 | -0.207580861 | -0.469373892 | 0.63902903 |
| ZNF510 | -0.021005855 | 3.891067673 | -0.468877167 | 0.639383788 |
| CD74 | -0.053967848 | 11.08997757 | -0.46887336 | 0.639386508 |
| RPL26 | -0.028648146 | 7.47614289 | -0.468745603 | 0.639477764 |
| CTNNA3 | -0.100564544 | -4.471904126 | -0.468628339 | 0.63956153 |
| CTD-2349P21.5 | 0.060852782 | 1.694334296 | 0.467670296 | 0.640246072 |
| TMEM220 | -0.048771623 | 3.897323082 | -0.466696165 | 0.640942424 |
| RAB36 | 0.068652777 | -0.872274176 | 0.465889354 | 0.641519409 |
| CPXM1 | -0.088976525 | 7.239136467 | -0.465819532 | 0.641569351 |
| ITIH4 | 0.041858467 | -0.318704078 | 0.465651964 | 0.641689218 |
| TPSD1 | -0.133142434 | 3.581261436 | -0.465276252 | 0.641958009 |
| C1QA | -0.122594157 | 3.598352429 | -0.465223594 | 0.641995685 |
| RPS11P5 | -0.03702776 | 8.51029854 | -0.465138302 | 0.642056712 |
| TAPBP | -0.017873383 | 8.349502707 | -0.465015568 | 0.642144535 |
| HIST1H2BJ | -0.058919873 | 3.616325047 | -0.464949463 | 0.642191838 |
| RP11-274B21.8 | 0.05434363 | -0.11646854 | 0.464629763 | 0.642420629 |
| PRR19 | 0.047108791 | -0.848982351 | 0.464545769 | 0.642480744 |
| ARHGEF28 | -0.087528356 | -3.091579485 | -0.464314631 | 0.642646185 |
| TLE6 | -0.068450028 | -2.960744383 | -0.464014749 | 0.642860856 |
| NPM1P35 | -0.042323053 | 0.395352956 | -0.463734983 | 0.643061154 |
| SERF2 | 0.0179445 | 7.396713724 | 0.462703471 | 0.643799887 |
| SF3B1 | -0.013962611 | 8.68635796 | -0.462688569 | 0.643810562 |
| RP3-486I3.4 | -0.026280554 | 6.763097032 | -0.462276329 | 0.644105895 |
| RNU6-125P | -0.051402025 | 0.929649713 | -0.462177985 | 0.644176359 |
| CRBN | -0.018886405 | 5.548274426 | -0.462177693 | 0.644176568 |
| RP11-802O23.3 | 0.034502267 | 3.946515681 | 0.462140794 | 0.644203007 |
| PKP3 | 0.084002455 | -1.596163814 | 0.462085435 | 0.644242673 |
| SCAMP4 | -0.020430102 | 4.095249389 | -0.461973721 | 0.644322723 |
| PFKFB4 | 0.05440983 | 3.750802732 | 0.461785074 | 0.64445791 |
| HTR7P1 | -0.032961162 | 3.040389242 | -0.461693295 | 0.644523683 |
| C1orf115 | 0.077889843 | 0.064344911 | 0.461109691 | 0.644941993 |
| KIN | 0.018300829 | 3.496961724 | 0.460858536 | 0.645122048 |
| CHRM3-AS2 | -0.070700395 | 1.895097147 | -0.460622628 | 0.64529119 |
| AC034220.3 | 0.062768936 | 0.438667899 | 0.460565833 | 0.645331915 |
| RILP | -0.031435285 | 5.134217905 | -0.460487602 | 0.645388011 |
| TMED3 | 0.024457691 | 6.642644196 | 0.460447781 | 0.645416566 |
| EIF3I | 0.01684205 | 8.451093432 | 0.460051578 | 0.645700703 |
| AGAP3 | -0.054467883 | 3.333487202 | -0.45989003 | 0.645816572 |
| RP11-727A23.4 | -0.037715554 | 1.949865749 | -0.459637979 | 0.645997371 |
| AK4 | -0.08646517 | 1.608030976 | -0.458610444 | 0.64673465 |
| CTC-490G23.2 | -0.114474788 | 1.826887467 | -0.458189254 | 0.647036964 |
| RP11-93B14.6 | -0.049253515 | -1.819315743 | -0.457822578 | 0.647300197 |
| MIR3671 | -0.056454904 | 1.983261578 | -0.457698647 | 0.647389176 |
| PGGT1BP1 | 0.040992514 | 0.887349707 | 0.457591325 | 0.647466234 |
| CD9 | 0.075940455 | 5.630390976 | 0.457399015 | 0.647604324 |
| DOC2GP | 0.068482442 | 1.296662886 | 0.457384577 | 0.647614692 |
| PPA1 | 0.036552395 | 5.743057996 | 0.457216197 | 0.64773561 |
| NMRK1 | -0.032993149 | 3.853486086 | -0.456922655 | 0.647946431 |
| GTF3A | -0.026199946 | 8.154971291 | -0.456787951 | 0.648043185 |
| BLOC1S4 | 0.022300291 | 4.93029355 | 0.456778636 | 0.648049876 |
| FER1L6 | -0.079473003 | -3.201651209 | -0.456625485 | 0.648159887 |
| AC006449.1 | 0.059752868 | -0.40559608 | 0.456099915 | 0.648537473 |
| AL591704.5 | -0.060098259 | -1.052403786 | -0.455872629 | 0.648700791 |
| SULT1A2 | -0.049336774 | 1.387783286 | -0.455524125 | 0.648951243 |
| SNAI1 | -0.067643045 | 3.948052661 | -0.45502411 | 0.649310647 |
| ZNF90 | -0.089486441 | 0.471268993 | -0.454913855 | 0.649389908 |
| RP3-337O18.9 | -0.052944436 | -0.495035161 | -0.454867874 | 0.649422965 |
| KIAA1462 | -0.115090721 | -0.307051022 | -0.454457994 | 0.649717664 |
| RP11-34P13.15 | 0.075444243 | 0.828045771 | 0.454389214 | 0.649767122 |
| AP000254.8 | -0.037191308 | 2.727916612 | -0.454327443 | 0.64981154 |
| AP000322.53 | -0.07256589 | -1.223914194 | -0.453675138 | 0.650280682 |
| PABPN1 | -0.019942893 | 6.722280186 | -0.453242944 | 0.650591595 |
| NME8 | -0.063278787 | 2.702519911 | -0.453227306 | 0.650602846 |
| EXOSC2 | -0.018631634 | 5.060599125 | -0.452694836 | 0.650985982 |
| CAV2 | 0.115224093 | 0.022869955 | 0.452149565 | 0.651378425 |
| PPP1R14B | 0.026280448 | 6.148932347 | 0.452078781 | 0.651429377 |
| CTA-228A9.3 | -0.060568784 | 1.903327752 | -0.45180211 | 0.651628546 |
| ZNF346 | 0.017691089 | 3.996419639 | 0.45171027 | 0.651694665 |
| NT5DC4 | -0.064322809 | -2.034875146 | -0.451358501 | 0.651947943 |
| RP11-282K24.3 | 0.065898239 | -1.786904237 | 0.451348901 | 0.651954855 |
| RPL9 | 0.043351216 | 6.289819854 | 0.451269062 | 0.652012345 |
| IGHV2-26 | 0.075138224 | 0.488126225 | 0.451244741 | 0.652029859 |
| LRBA | 0.03260862 | 5.260179282 | 0.450888923 | 0.652286106 |
| PPFIA3 | 0.058058935 | 0.009813785 | 0.450844299 | 0.652318245 |
| AP000688.8 | -0.115257626 | -0.895995683 | -0.450575537 | 0.652511829 |
| PMFBP1 | 0.039239275 | 0.999488627 | 0.450551014 | 0.652529494 |
| SDCBPP2 | 0.045137637 | 2.61560072 | 0.450082023 | 0.652867358 |
| OAZ1 | -0.018870981 | 9.761994178 | -0.448967319 | 0.653670685 |
| RP11-165H4.2 | 0.046923668 | 0.084280531 | 0.448898502 | 0.653720292 |
| RP11-480A16.1 | -0.049549702 | 1.669005096 | -0.44888007 | 0.653733579 |
| PACSIN3 | -0.062718073 | 0.407703323 | -0.448764086 | 0.653817191 |
| SH3GLB1 | 0.012760505 | 6.979179239 | 0.448538115 | 0.653980104 |
| RP11-303E16.2 | -0.047293541 | 2.581664051 | -0.448203119 | 0.654221649 |
| EIF3H | -0.019378022 | 8.06705194 | -0.448143159 | 0.654264886 |
| TSPY26P | -0.040566968 | 2.435941454 | -0.448058913 | 0.654325638 |
| SPPL2B | 0.025055242 | 6.021067703 | 0.447817921 | 0.654499436 |
| AC005329.7 | 0.053895855 | 0.971508662 | 0.447782913 | 0.654524685 |
| MEIS3 | 0.070089865 | -3.406427142 | 0.447523673 | 0.654711667 |
| RP5-857K21.11 | -0.039596341 | 8.011451038 | -0.44739266 | 0.654806171 |
| RP11-723O4.3 | -0.047442136 | 0.039258757 | -0.44723678 | 0.654918619 |
| ZNF286B | 0.048977792 | -0.217349851 | 0.447004838 | 0.655085952 |
| RP11-442A17.2 | 0.037492733 | 1.657873636 | 0.446608061 | 0.655372244 |
| RP11-620J15.1 | -0.056882151 | -0.443570252 | -0.446598391 | 0.655379222 |
| ZNF286A | -0.048173261 | 0.573368027 | -0.446419902 | 0.655508027 |
| hsa-mir-6080 | 0.030063002 | 3.843601163 | 0.4463046 | 0.655591239 |
| NLRP2 | -0.101579155 | 0.859300169 | -0.446303658 | 0.655591919 |
| IGLV3-16 | 0.060574962 | -0.342836432 | 0.446130258 | 0.655717068 |
| TMEM161B | 0.017223793 | 3.381865609 | 0.446033172 | 0.655787142 |
| RP4-635A23.4 | -0.04691214 | 0.051008514 | -0.445986735 | 0.65582066 |
| POTEKP | -0.049092205 | -2.099839334 | -0.445981419 | 0.655824497 |
| NPM2 | -0.078299902 | 3.062750269 | -0.445875453 | 0.655900987 |
| CASQ1 | -0.068384104 | -2.262737819 | -0.445774548 | 0.655973826 |
| HNRNPA3P13 | 0.046665979 | -0.737575144 | 0.445603115 | 0.656097584 |
| NDRG1 | 0.035503739 | 6.470548781 | 0.445140519 | 0.656431581 |
| MOB1A | -0.016672239 | 8.044975455 | -0.445133104 | 0.656436936 |
| KTN1 | 0.018926996 | 6.583197558 | 0.444969464 | 0.656555102 |
| RP11-737O24.2 | 0.049239655 | -0.717374967 | 0.444824648 | 0.656659682 |
| RPL36AP21 | 0.038315758 | 1.606806011 | 0.444792111 | 0.65668318 |
| RASA4CP | -0.02922318 | 3.520376128 | -0.444721473 | 0.656734195 |
| FBXO16 | 0.048827331 | -3.606722732 | 0.444545508 | 0.656861285 |
| MSI2 | 0.059249344 | 6.236966594 | 0.444262458 | 0.657065737 |
| LRRC37A4P | -0.069125336 | 4.019222991 | -0.443677362 | 0.657488444 |
| CDHR5 | 0.063066614 | -2.669029189 | 0.443627551 | 0.657524435 |
| CLIC1 | -0.016141261 | 10.29510047 | -0.44344432 | 0.657656837 |
| HRH1 | -0.0735591 | -2.05384575 | -0.443308939 | 0.65775467 |
| CNTNAP3 | 0.112798464 | -1.139875903 | 0.442727447 | 0.658174951 |
| ATPAF2 | -0.016715123 | 4.32394525 | -0.442443672 | 0.658380093 |
| PPP1R16A | 0.018849871 | 3.918491545 | 0.442417591 | 0.658398948 |
| AC009404.2 | 0.042817651 | 0.502660899 | 0.442218868 | 0.658542622 |
| SNORD4A | 0.034573395 | 2.720466505 | 0.441972625 | 0.65872067 |
| SHE | 0.086046099 | -2.573114061 | 0.441575855 | 0.659007599 |
| TRHDE | 0.0844992 | -4.30897396 | 0.441320191 | 0.659192513 |
| AC116614.1 | 0.05438452 | -1.262925825 | 0.441316716 | 0.659195025 |
| ARHGEF17 | -0.057000538 | 4.070492897 | -0.440780277 | 0.659583083 |
| CTA-392E5.1 | -0.071951533 | -0.358371526 | -0.440714992 | 0.659630315 |
| IGLV1-51 | 0.062754368 | 1.9896391 | 0.440274473 | 0.659949062 |
| CTC-297N7.5 | -0.049956574 | 2.01298869 | -0.440261854 | 0.659958194 |
| RP1-317E23.3 | 0.039855909 | 0.55866816 | 0.440230775 | 0.659980684 |
| ZNF876P | 0.055685626 | 0.204377516 | 0.440219043 | 0.659989174 |
| PTPRF | 0.082343986 | -0.139044009 | 0.440197738 | 0.660004591 |
| PTCH2 | -0.032246621 | 0.935863702 | -0.440096267 | 0.660078024 |
| NME1 | 0.034655321 | 4.536823095 | 0.440085548 | 0.660085782 |
| RPL31P7 | -0.03870313 | 3.385564854 | -0.439440989 | 0.660552316 |
| RP11-67M1.1 | 0.058203759 | -2.311344933 | 0.439423054 | 0.6605653 |
| LIMS2 | -0.059786277 | -0.975058758 | -0.439416228 | 0.660570241 |
| ECHDC2 | -0.057781961 | 3.082636174 | -0.439398546 | 0.660583041 |
| ZCCHC4 | 0.015458559 | 2.861924704 | 0.438883632 | 0.66095584 |
| LINC00938 | 0.028529981 | 6.663935415 | 0.438798857 | 0.661017226 |
| ZNF469 | -0.056977916 | 2.216505952 | -0.438397127 | 0.661308148 |
| BRSK1 | -0.057772934 | 0.448214597 | -0.438360704 | 0.661334528 |
| CLCNKA | 0.063605643 | -2.192987721 | 0.438320994 | 0.661363288 |
| SDK1 | 0.103235709 | -2.130450862 | 0.437832689 | 0.661716987 |
| RP11-707O23.5 | 0.104077534 | -0.857553897 | 0.437591462 | 0.661891746 |
| FAM84A | 0.105732119 | -4.486555319 | 0.437490535 | 0.661964869 |
| PINLYP | -0.078386884 | 0.367459624 | -0.437420725 | 0.662015448 |
| SCUBE2 | -0.054925609 | -2.789493487 | -0.436966722 | 0.662344429 |
| FGF13 | 0.111162584 | -1.435487707 | 0.436817636 | 0.662452474 |
| RP11-187C18.4 | -0.062586682 | -0.218166594 | -0.436609247 | 0.662603509 |
| PI4KAP1 | 0.036031825 | 4.335838234 | 0.436535683 | 0.66265683 |
| DANCR | 0.031174355 | 6.168804489 | 0.436485525 | 0.662693186 |
| DKK1 | 0.070275204 | -2.749750093 | 0.43644192 | 0.662724793 |
| SNHG5 | 0.03450786 | 7.626765295 | 0.436304072 | 0.662824717 |
| IGKV1-17 | 0.086625188 | 1.702727227 | 0.43604059 | 0.663015727 |
| ROPN1L | 0.056808244 | 2.419946207 | 0.435997659 | 0.663046851 |
| ZNF713 | 0.030810081 | 1.63511954 | 0.43595749 | 0.663075974 |
| SETD5 | 0.014234718 | 6.270812305 | 0.435437209 | 0.663453228 |
| CTB-33G10.1 | 0.021646188 | 5.432797694 | 0.434717718 | 0.663975069 |
| RP11-129H15.4 | 0.032325114 | 0.650738304 | 0.434707675 | 0.663982354 |
| SUGP2 | 0.01742387 | 7.114644771 | 0.434320133 | 0.664263504 |
| ZNF831 | -0.054605195 | 1.081071708 | -0.434223333 | 0.664333737 |
| DNPH1 | 0.031746319 | 5.172889925 | 0.43418533 | 0.664361311 |
| HTATSF1 | -0.018036819 | 7.491990254 | -0.43394157 | 0.664538186 |
| RPL12P15 | 0.044907224 | -0.168098341 | 0.433906977 | 0.664563289 |
| HK2 | -0.038605572 | 6.055818986 | -0.433822591 | 0.664624526 |
| AC006126.4 | 0.084517619 | 0.206768739 | 0.433724637 | 0.664695611 |
| RPL4P6 | -0.035238846 | 1.990693995 | -0.43346506 | 0.664884002 |
| CCDC153 | -0.039096693 | 1.621208374 | -0.433407262 | 0.664925952 |
| TPTE2 | 0.051634753 | -1.633305904 | 0.433392209 | 0.664936878 |
| ZNF384 | -0.01276023 | 6.394144806 | -0.433151704 | 0.665111451 |
| ZNF577 | -0.03060248 | 3.229385546 | -0.432551873 | 0.665546925 |
| MANSC1 | 0.069130508 | 3.265699566 | 0.432433686 | 0.665632741 |
| PCDHGA4 | 0.073684771 | -2.464884319 | 0.432268019 | 0.66575304 |
| AC093642.3 | 0.058550357 | 3.086747099 | 0.431956215 | 0.665979481 |
| TNNI3 | 0.073926107 | -2.320485053 | 0.431719715 | 0.666151254 |
| LINC00958 | 0.111242804 | -1.349466242 | 0.431035237 | 0.666648498 |
| LTBP1 | 0.104871502 | 3.042442492 | 0.430078364 | 0.667343872 |
| RBMX2 | 0.01655746 | 4.957849279 | 0.429078515 | 0.668070783 |
| RP11-578F21.12 | 0.081294251 | 2.006044359 | 0.42899331 | 0.668132744 |
| IKZF2 | -0.074867784 | 3.002841213 | -0.427861749 | 0.66895582 |
| THSD4 | 0.098250898 | -5.956274896 | 0.427828184 | 0.66898024 |
| NBPF3 | -0.053650009 | 2.428086139 | -0.427818348 | 0.668987397 |
| ABHD17AP5 | -0.066203756 | 2.812936342 | -0.427510387 | 0.669211475 |
| RASA4 | 0.046450531 | -0.141555633 | 0.427327704 | 0.669344413 |
| RPL31P52 | -0.044739661 | -0.239449091 | -0.427061217 | 0.669538353 |
| C14orf166 | -0.015889611 | 7.830825115 | -0.426946822 | 0.669621612 |
| AC091814.2 | -0.136189985 | 1.045869217 | -0.426468216 | 0.669969998 |
| TBC1D8 | 0.062065797 | 3.786347919 | 0.426381849 | 0.670032874 |
| PMS2P5 | 0.031283376 | 3.386101626 | 0.426116875 | 0.67022579 |
| CLEC3B | 0.077265847 | -0.589854484 | 0.42596891 | 0.670333527 |
| EZH2P1 | -0.045994688 | 1.100219837 | -0.425849521 | 0.670420462 |
| THAP7-AS1 | -0.030134963 | 1.864862901 | -0.425682968 | 0.670541748 |
| IRGM | -0.063821371 | 2.787898883 | -0.425658229 | 0.670559763 |
| RP11-333A23.4 | 0.078181698 | -2.887329756 | 0.425516973 | 0.670662635 |
| VNN2 | -0.088604634 | 6.033664498 | -0.425497418 | 0.670676877 |
| RNU6-892P | -0.043817293 | 1.545263938 | -0.425421881 | 0.670731891 |
| AP000769.7 | -0.046267549 | -0.04248476 | -0.425419774 | 0.670733425 |
| CYCSP45 | -0.040347824 | 1.14088193 | -0.424976838 | 0.671056054 |
| CES1P1 | -0.09583764 | -2.201098791 | -0.424578572 | 0.671346197 |
| CARS | 0.016288166 | 4.458950106 | 0.424183961 | 0.671633726 |
| CEP78 | 0.023717039 | 3.563228188 | 0.423222511 | 0.672334479 |
| IGKV2OR22-4 | 0.07392129 | 0.120811016 | 0.422726251 | 0.672696289 |
| LINC00484 | -0.065357061 | -1.702480555 | -0.422723952 | 0.672697965 |
| RP11-152P17.2 | 0.076325095 | -2.421405948 | 0.422683674 | 0.672727334 |
| FGR | -0.071190606 | 8.084141453 | -0.422376695 | 0.672951187 |
| RP11-326A19.5 | 0.0191557 | 5.250720953 | 0.422148562 | 0.673117563 |
| AC096579.7 | 0.077731204 | 2.13360629 | 0.422110659 | 0.673145207 |
| BCORP1 | -0.117494939 | -2.364399436 | -0.422026192 | 0.673206814 |
| RRN3P3 | 0.02225257 | 3.663716789 | 0.421487305 | 0.673599905 |
| MUTYH | 0.016130466 | 4.460730071 | 0.421469969 | 0.673612552 |
| SIRT3 | -0.017235096 | 3.777423262 | -0.421450866 | 0.673626489 |
| POU5F1B | 0.053638326 | -2.718513168 | 0.421423175 | 0.67364669 |
| AC104841.2 | 0.056901979 | -0.317943962 | 0.421401296 | 0.673662653 |
| HIST1H2BN | 0.047656433 | 1.854318258 | 0.421231713 | 0.673786378 |
| RP11-45M22.4 | 0.043021718 | -1.54723051 | 0.420595722 | 0.674250467 |
| ATG4B | 0.011785174 | 5.540986812 | 0.420576567 | 0.674264446 |
| SNORA66 | -0.032854738 | 2.508196078 | -0.420541718 | 0.674289879 |
| NOV | 0.08724269 | 2.646359985 | 0.420470198 | 0.674342078 |
| HNRNPDL | 0.017881618 | 8.64828002 | 0.419692094 | 0.674910067 |
| CTC-548K16.5 | 0.061624639 | 0.229622679 | 0.419641824 | 0.674946769 |
| RP11-583F2.1 | -0.038433528 | 1.644638475 | -0.419440258 | 0.675093939 |
| RP11-764K9.4 | -0.040252459 | 2.119157291 | -0.419153575 | 0.675303277 |
| ADAMTSL5 | -0.057716019 | -1.347906286 | -0.418841052 | 0.675531511 |
| NPIPB6 | 0.050699176 | -0.150527864 | 0.418639375 | 0.675678811 |
| ZNF229 | -0.088641431 | -0.354886766 | -0.418431504 | 0.675830649 |
| RP11-815J21.1 | 0.050580698 | 0.48248756 | 0.418293264 | 0.675931633 |
| QSOX1 | -0.033286689 | 6.500697287 | -0.417952426 | 0.676180637 |
| EIF2D | -0.020006892 | 6.62607074 | -0.417914161 | 0.676208594 |
| C2orf82 | -0.05245672 | -0.10722815 | -0.41778567 | 0.676302476 |
| RP11-678G14.5 | 0.02298018 | 2.834154514 | 0.417783455 | 0.676304095 |
| RP11-121C2.2 | 0.02909274 | 1.918164618 | 0.417435056 | 0.676558678 |
| PDE8A | 0.029245881 | 3.667583099 | 0.417044513 | 0.676844101 |
| STC2 | -0.070452324 | -4.390618826 | -0.416671988 | 0.677116399 |
| EEF1A1P13 | -0.021987373 | 9.591105158 | -0.416668991 | 0.67711859 |
| CXCR6 | -0.060074116 | -1.56117467 | -0.416469147 | 0.677264684 |
| RPL18AP3 | -0.016732559 | 11.39295758 | -0.416435702 | 0.677289136 |
| ABCC6P1 | -0.068942794 | -2.326352138 | -0.415963041 | 0.677634723 |
| TULP3 | 0.023513319 | 3.388242028 | 0.415956858 | 0.677639244 |
| CTD-2184D3.1 | 0.037710878 | 2.298940133 | 0.415205612 | 0.678188662 |
| SNORD46 | -0.033027796 | 2.798395844 | -0.415073876 | 0.678285024 |
| SHOX2 | 0.072624094 | -2.351001531 | 0.414973051 | 0.678358779 |
| RBFOX3 | -0.106973692 | -4.318966965 | -0.414692646 | 0.678563915 |
| RP11-377D9.3 | 0.052627197 | -1.62313838 | 0.414437783 | 0.678750385 |
| P2RY13 | 0.084807502 | 5.135828819 | 0.414363417 | 0.678804799 |
| EPHB2 | -0.101870723 | -0.315258245 | -0.414346318 | 0.67881731 |
| RFX7 | 0.022317712 | 5.724357125 | 0.414211034 | 0.678916303 |
| CT45A6 | 0.077106439 | -2.277596776 | 0.413845574 | 0.679183752 |
| TSPAN17 | 0.032606024 | 4.529595904 | 0.413838554 | 0.67918889 |
| IGHV3-13 | 0.073389872 | 0.918585642 | 0.41369723 | 0.679292324 |
| NBEA | 0.067596349 | 0.269809796 | 0.413123906 | 0.679711998 |
| CSTF2T | 0.020926398 | 5.640864994 | 0.413055967 | 0.679761736 |
| EEF1A1P9 | 0.024401303 | 8.134811812 | 0.413013408 | 0.679792895 |
| CCDC183 | -0.033998527 | 0.483299676 | -0.412650406 | 0.680058676 |
| RP11-305O6.3 | 0.061514807 | 0.401587297 | 0.412597913 | 0.680097113 |
| PRL | -0.093807537 | -1.414223248 | -0.412523114 | 0.680151886 |
| ARMC5 | 0.031327772 | 5.123225325 | 0.412424131 | 0.680224369 |
| CCNG1 | -0.032103109 | 7.609524874 | -0.412416703 | 0.680229809 |
| VASP | -0.029949676 | 8.074219139 | -0.412209324 | 0.68038168 |
| LINC00923 | -0.079121439 | -1.857544661 | -0.411586823 | 0.680837636 |
| STON2 | -0.084494364 | 3.367467431 | -0.411536067 | 0.680874819 |
| RP1-249H1.4 | -0.11505472 | 3.097218496 | -0.411420906 | 0.680959184 |
| CBWD5 | 0.026734224 | 2.063141784 | 0.411121146 | 0.681178803 |
| RPS14P3 | 0.023970867 | 4.10212422 | 0.410832475 | 0.681390323 |
| HRK | 0.063152823 | -3.778902767 | 0.410764059 | 0.681440458 |
| RP11-567M16.3 | 0.056134913 | -0.061632909 | 0.410028787 | 0.681979348 |
| ZSWIM8 | -0.01535652 | 6.243861981 | -0.409907252 | 0.682068438 |
| RP11-326C3.15 | -0.066385503 | 0.888856653 | -0.409748274 | 0.682184982 |
| AJAP1 | 0.082100126 | -5.258909482 | 0.409432407 | 0.682416562 |
| UBE2L5P | -0.034017693 | 1.12777407 | -0.409328221 | 0.682492953 |
| HOTAIRM1 | 0.05763071 | 4.273696394 | 0.408795016 | 0.68288396 |
| MID1 | 0.051598413 | -2.074396143 | 0.408529309 | 0.683078838 |
| RP13-188A5.1 | 0.046200873 | 1.543997537 | 0.408140079 | 0.683364351 |
| WDR55 | -0.016952097 | 4.665180865 | -0.408113295 | 0.683384 |
| CXCR1 | -0.097813379 | 2.802894244 | -0.407769798 | 0.683636007 |
| SNCG | 0.060695152 | -1.58252079 | 0.407287749 | 0.683989722 |
| BTF3P4 | 0.026446321 | 2.623100933 | 0.407278954 | 0.683996177 |
| AP2S1 | 0.021711933 | 6.334514456 | 0.407222045 | 0.68403794 |
| RET | -0.072048105 | 0.968256938 | -0.407166931 | 0.684078386 |
| FAM184A | -0.06130216 | -2.035524401 | -0.406796383 | 0.684350346 |
| ZC3H12B | -0.078236737 | -1.469703623 | -0.406541467 | 0.684537462 |
| ATAD2B | -0.014426884 | 4.446136173 | -0.406424032 | 0.684623669 |
| NFATC1 | -0.031981701 | 4.512160128 | -0.406279243 | 0.684729963 |
| RP11-16E18.3 | 0.034373378 | 0.685121406 | 0.406210957 | 0.684780096 |
| RP11-359B12.2 | -0.028795616 | 1.854534486 | -0.406071332 | 0.684882607 |
| DRG1 | -0.012554873 | 6.430772186 | -0.405939677 | 0.684979272 |
| CLEC4G | 0.087417611 | 0.553659829 | 0.405456669 | 0.685333956 |
| RP11-531A24.5 | -0.037410483 | 0.887125022 | -0.405270286 | 0.685470839 |
| PPP1R14BP2 | -0.043962632 | 0.498921999 | -0.405113794 | 0.685585778 |
| RP11-458F8.3 | 0.038149713 | 0.94492249 | 0.404965698 | 0.685694557 |
| ATP1B3 | 0.020720946 | 7.14890275 | 0.404907118 | 0.685737587 |
| RP5-1132H15.3 | 0.029380685 | 1.494387163 | 0.404770394 | 0.685838022 |
| GNB2 | 0.021439334 | 8.111978959 | 0.404615247 | 0.685951996 |
| AC061992.1 | 0.046874961 | 0.372891063 | 0.404573557 | 0.685982624 |
| RP11-475J5.4 | 0.053460791 | 0.31345786 | 0.404477491 | 0.6860532 |
| ADRB1 | -0.10328501 | 1.348635027 | -0.40446623 | 0.686061473 |
| PES1P2 | 0.038476161 | 0.560546989 | 0.404247483 | 0.686222192 |
| RP11-395G23.3 | 0.059766322 | 1.884020578 | 0.40404681 | 0.686369643 |
| ICAM5 | 0.074514308 | 2.257326899 | 0.403787605 | 0.68656012 |
| NTN5 | -0.051734715 | 0.15077548 | -0.4035954 | 0.686701376 |
| SEMA6C | -0.038021899 | 1.282520232 | -0.403543269 | 0.68673969 |
| HYAL3 | -0.04979397 | 5.213569716 | -0.403413899 | 0.686834774 |
| FAM8A6P | 0.04103124 | -0.836673257 | 0.403172914 | 0.687011908 |
| SH2D1A | 0.077457491 | 4.358624572 | 0.403147423 | 0.687030645 |
| TMEM45A | -0.075638916 | -1.241271428 | -0.402432433 | 0.687556297 |
| ABCB11 | -0.058577016 | -3.527939536 | -0.402388496 | 0.687588604 |
| SIRPB1 | 0.079178297 | 4.978928573 | 0.401988762 | 0.687882554 |
| RPS4XP22 | -0.098549368 | 3.206355262 | -0.4019598 | 0.687903854 |
| AC013460.1 | -0.051043209 | -2.311505707 | -0.401881341 | 0.687961557 |
| AL049829.1 | 0.050839957 | -0.270533758 | 0.401448724 | 0.688279755 |
| PAK3 | -0.059305713 | -4.383376434 | -0.401271853 | 0.688409863 |
| SAGE1 | 0.117123982 | -2.292181966 | 0.400617691 | 0.688891152 |
| PA2G4P2 | 0.020035905 | 4.974009956 | 0.400329322 | 0.689103355 |
| C19orf77 | -0.079345746 | 7.10793344 | -0.400258051 | 0.689155805 |
| AK4P1 | -0.059666879 | 2.962184804 | -0.400193167 | 0.689203556 |
| XIRP2 | 0.107429284 | -3.323571883 | 0.400192931 | 0.68920373 |
| SRGAP1 | -0.077328352 | -0.828965224 | -0.399946031 | 0.689385446 |
| SNX13 | 0.020414429 | 4.322204341 | 0.399568971 | 0.689662994 |
| RPL12L3 | -0.025460095 | 5.454612409 | -0.398910185 | 0.690148017 |
| RAB3A | 0.040646812 | 1.873060594 | 0.398797725 | 0.690230826 |
| FAM214B | -0.030476285 | 5.114525492 | -0.398606882 | 0.690371362 |
| KLRC2 | -0.058423561 | -1.334842974 | -0.398402737 | 0.690521705 |
| ASS1 | -0.097766639 | -1.071724426 | -0.39820249 | 0.69066919 |
| TOMM70A | 0.014929279 | 5.868289312 | 0.398005794 | 0.69081407 |
| AC007204.2 | 0.050354079 | -0.884469584 | 0.397919665 | 0.690877514 |
| AC022154.7 | 0.041801734 | -1.009855018 | 0.397598667 | 0.691113984 |
| ACTBP11 | -0.045077274 | 1.61584505 | -0.397581726 | 0.691126465 |
| AL590762.11 | -0.030877936 | 0.630453789 | -0.397454312 | 0.691220336 |
| KIAA1211L | -0.074092812 | -0.385166941 | -0.396943215 | 0.691596931 |
| UBTD1 | 0.041221586 | 4.626809255 | 0.396825095 | 0.691683977 |
| AC005534.6 | -0.040045242 | -0.214375564 | -0.396811027 | 0.691694344 |
| RP11-359E3.4 | -0.070304395 | -4.252603921 | -0.396661379 | 0.691804631 |
| ZNF525 | 0.022097249 | 2.6907244 | 0.396570094 | 0.691871908 |
| GPAM | -0.023953792 | 2.457922764 | -0.396554251 | 0.691883585 |
| RP11-495P10.8 | -0.065111973 | -0.424868122 | -0.396534428 | 0.691898195 |
| LAMP5 | 0.129034368 | 0.501943491 | 0.39640339 | 0.691994777 |
| TRAF3IP1 | -0.017151483 | 3.51222715 | -0.395858228 | 0.692396643 |
| SMIM7 | -0.012391178 | 4.280409414 | -0.395571457 | 0.69260807 |
| ATP7B | -0.064113258 | 2.017238372 | -0.395227989 | 0.692861331 |
| GPT | 0.064698785 | -0.462528797 | 0.395151223 | 0.69291794 |
| HINT1 | 0.028525974 | 8.518050079 | 0.395143173 | 0.692923877 |
| NAP1L1P3 | -0.026763943 | 5.535725009 | -0.395115687 | 0.692944146 |
| PRPF38AP1 | -0.034870413 | -0.214281214 | -0.39488536 | 0.693114007 |
| GALNT10 | 0.023984675 | 5.087322133 | 0.394677689 | 0.693267174 |
| NALF2 | -0.067129611 | -3.524058725 | -0.394424324 | 0.693454058 |
| LRP1 | -0.100159063 | 4.93705382 | -0.394105218 | 0.69368946 |
| AGPAT3 | -0.031438596 | 5.205722261 | -0.393630539 | 0.694039683 |
| GCSH | -0.034814099 | 1.110589915 | -0.393326238 | 0.694264234 |
| PTPRN2 | 0.064955073 | 1.869014869 | 0.392232902 | 0.695071253 |
| UPF3A | -0.017088261 | 5.643137837 | -0.3919769 | 0.695260266 |
| IGKV6-21 | -0.059881287 | 0.275528718 | -0.391546283 | 0.695578242 |
| CTPS1 | 0.025960246 | 4.663960141 | 0.390874764 | 0.696074214 |
| NGFRAP1 | -0.082668008 | 5.259347579 | -0.390785747 | 0.69613997 |
| FAM109B | 0.046091666 | 3.122002346 | 0.390767094 | 0.696153748 |
| RP11-1070N10.3 | 0.043701623 | 0.416174629 | 0.39044025 | 0.696395207 |
| TUBBP5 | 0.067163702 | -1.722891808 | 0.390431649 | 0.696401562 |
| AK8 | -0.054197908 | -1.201754982 | -0.389703207 | 0.696939816 |
| RP11-542K23.7 | 0.062669346 | -1.330672645 | 0.389535465 | 0.697063783 |
| RP11-306G20.1 | -0.051737831 | -1.282388318 | -0.389258848 | 0.697268232 |
| RP11-902B17.1 | 0.056646094 | -0.502320938 | 0.38923647 | 0.697284773 |
| RP1-228H13.5 | -0.028610956 | 2.252728067 | -0.389223183 | 0.697294595 |
| RP11-809F4.2 | -0.048248638 | 1.610791224 | -0.388737254 | 0.697653805 |
| RTEL1P1 | 0.038687857 | -1.040344674 | 0.388647685 | 0.697720024 |
| SLC25A42 | -0.025192336 | 3.599036921 | -0.388259454 | 0.698007074 |
| ACAA2 | 0.02837296 | 5.112542146 | 0.388026169 | 0.69817958 |
| LMO4 | 0.045635144 | 4.776793122 | 0.387926408 | 0.698253354 |
| KIRREL2 | -0.055189681 | -2.948960212 | -0.387879569 | 0.698287994 |
| DAGLB | 0.021448177 | 5.239590952 | 0.387655166 | 0.698453956 |
| MIR941-1 | 0.047170877 | 0.667420491 | 0.387236055 | 0.698763959 |
| CD200 | -0.089163451 | 3.357404187 | -0.387193632 | 0.69879534 |
| TXNP4 | -0.028362331 | 1.627220667 | -0.387132449 | 0.6988406 |
| GOLGB1 | 0.01419073 | 6.27479807 | 0.386846697 | 0.699051999 |
| CORIN | -0.057234686 | -4.410270126 | -0.386728668 | 0.699139323 |
| MYO1B | -0.080765827 | 0.352111681 | -0.386527872 | 0.699287892 |
| RP11-1094M14.12 | 0.043293032 | -0.37048587 | 0.386187616 | 0.699539674 |
| SNORA28 | -0.037062482 | 2.102078396 | -0.386125738 | 0.699585466 |
| FAM60CP | -0.028514153 | 5.51469576 | -0.385786825 | 0.699836294 |
| MGAT5 | -0.020031456 | 5.479958932 | -0.385722392 | 0.699883984 |
| RP11-849H4.4 | -0.038888632 | 0.187514243 | -0.385337299 | 0.700169035 |
| TAP1 | 0.027401196 | 6.309923308 | 0.385202974 | 0.700268475 |
| HSPE1P11 | 0.038263691 | 0.897817259 | 0.38518639 | 0.700280752 |
| SNX5P1 | -0.019515159 | 2.955180442 | -0.38506718 | 0.700369007 |
| RMI2 | 0.04667149 | 3.222504156 | 0.385038306 | 0.700390384 |
| LIMS3 | -0.035623095 | 0.812906404 | -0.384389782 | 0.700870582 |
| ZBTB38 | 0.039784668 | 3.583875763 | 0.384335094 | 0.700911081 |
| RP1-182O16.1 | 0.033339548 | 1.055716873 | 0.383867092 | 0.701257694 |
| HOXA-AS3 | -0.079852037 | -2.193322722 | -0.383612946 | 0.701445947 |
| ANO10 | -0.025799628 | 4.378160336 | -0.382679634 | 0.702137433 |
| RP11-417J8.3 | 0.064552749 | -2.617088538 | 0.382252338 | 0.702454097 |
| RP5-827C21.1 | -0.020879914 | 6.42933958 | -0.382192926 | 0.702498131 |
| HRAS | -0.015333017 | 5.009229995 | -0.382065049 | 0.702592911 |
| FTL | 0.013869061 | 12.31775549 | 0.381938028 | 0.702687062 |
| CNR2 | -0.052176372 | 1.191478067 | -0.381668133 | 0.702887129 |
| SMARCA1 | -0.084332628 | -0.440602577 | -0.381570862 | 0.702959239 |
| ANPEP | 0.060752948 | 7.494112561 | 0.381532385 | 0.702987763 |
| PRAME | 0.133596762 | 0.701872452 | 0.381490814 | 0.703018583 |
| DCUN1D5 | 0.016952522 | 5.783509374 | 0.380913454 | 0.703446664 |
| FAM66A | -0.048115274 | -1.429134768 | -0.380868966 | 0.703479654 |
| DAP | -0.018667089 | 7.486230055 | -0.380831909 | 0.703507133 |
| VANGL2 | -0.08269815 | 0.150720089 | -0.38062456 | 0.703660899 |
| BEX4 | -0.047575504 | 7.476242843 | -0.379862226 | 0.704226336 |
| ZDHHC15 | 0.089174488 | -1.793122067 | 0.379526756 | 0.704475212 |
| RSPO4 | 0.057943238 | -2.000138985 | 0.379066625 | 0.704816622 |
| MUC16 | 0.057609203 | -3.911694207 | 0.37898253 | 0.704879026 |
| RP11-384C4.3 | 0.029618771 | 0.823064237 | 0.378931125 | 0.704917173 |
| NPAS1 | 0.054575379 | 0.308773195 | 0.377938345 | 0.705654041 |
| AC140076.1 | -0.030038669 | 4.184230989 | -0.377896329 | 0.705685233 |
| RP11-274B21.3 | 0.023275042 | 6.934550819 | 0.377871644 | 0.705703559 |
| FAM3C | -0.024809136 | 3.57335249 | -0.377489136 | 0.705987548 |
| DTX1 | 0.061275154 | 0.070136934 | 0.377207201 | 0.706196895 |
| MOXD1 | 0.047159204 | -3.023956235 | 0.377173704 | 0.706221769 |
| PDZRN4 | -0.066066948 | -3.511387351 | -0.37709991 | 0.706276567 |
| HIST1H3F | -0.056830344 | -0.569736087 | -0.37706315 | 0.706303866 |
| HLA-DRB1 | 0.065630996 | 8.995019496 | 0.376953674 | 0.706385166 |
| ANTXRLP1 | -0.051976544 | -0.999528836 | -0.376766512 | 0.706524166 |
| RPL31 | 0.024838387 | 8.273756263 | 0.376737239 | 0.706545906 |
| GGN | -0.048727122 | -1.614758892 | -0.37651988 | 0.706707347 |
| ABHD17AP4 | 0.033180142 | 1.587336551 | 0.376396318 | 0.706799126 |
| RP11-37J13.1 | -0.034912918 | -0.457565845 | -0.376359045 | 0.706826813 |
| BZRAP1-AS1 | 0.022539956 | 4.853484346 | 0.376230505 | 0.706922296 |
| CSNK1G1 | 0.013858041 | 4.70550515 | 0.376207027 | 0.706939737 |
| MST1 | 0.034770017 | 2.697937894 | 0.376080359 | 0.707033835 |
| MYO5C | 0.066277136 | 2.372665893 | 0.375777397 | 0.707258915 |
| AC007041.2 | 0.019700498 | 2.378754828 | 0.375241204 | 0.707657332 |
| RP11-301G19.1 | 0.100569629 | 0.955091113 | 0.374845645 | 0.707951303 |
| RP11-43D4.2 | -0.032357936 | 0.875556093 | -0.374341073 | 0.708326354 |
| RPS21 | -0.02518695 | 9.366435607 | -0.374131639 | 0.708482048 |
| TRAF5 | -0.037938153 | 4.330061768 | -0.374108014 | 0.708499612 |
| ASCC1 | -0.017673815 | 4.268861763 | -0.373639049 | 0.708848289 |
| HLA-AS1 | -0.03977366 | 1.268371365 | -0.37363303 | 0.708852765 |
| SLC25A5-AS1 | -0.022676105 | 1.371146927 | -0.373527036 | 0.708931581 |
| BLMH | -0.018204531 | 5.693680525 | -0.373040073 | 0.70929372 |
| SIRT7 | 0.018359984 | 4.825831352 | 0.372709299 | 0.709539744 |
| HIST1H2BI | -0.057004292 | -0.37866974 | -0.37248513 | 0.709706494 |
| TPM4 | -0.035286413 | 8.399792414 | -0.372305829 | 0.709839879 |
| ARL2BP | 0.014847093 | 7.145343736 | 0.37212569 | 0.709973896 |
| YBX3P1 | 0.034891699 | 5.859480165 | 0.371982901 | 0.710080133 |
| GPR88 | -0.063982957 | -1.586611674 | -0.371229 | 0.710641137 |
| MPV17L | 0.083876121 | 0.409434846 | 0.370699058 | 0.711035579 |
| ADAP1 | 0.039626678 | 3.410427927 | 0.370631454 | 0.711085904 |
| PI3 | 0.09864969 | 1.78528201 | 0.370587771 | 0.711118421 |
| CTD-2267D19.2 | -0.038406598 | 2.570902697 | -0.369987765 | 0.711565125 |
| SLC27A2 | 0.047361256 | 4.555377101 | 0.369859683 | 0.711660495 |
| MAGED4 | 0.079640599 | -2.919535262 | 0.369691051 | 0.711786066 |
| ALG1L13P | -0.066766631 | 1.627095645 | -0.369304485 | 0.712073948 |
| BID | 0.019475659 | 5.88740441 | 0.369269639 | 0.712099901 |
| SPATA1 | 0.030938528 | 0.668511823 | 0.368884026 | 0.712387118 |
| TAS2R60 | 0.047923786 | -0.985711952 | 0.368640359 | 0.712568631 |
| RP1-315G1.3 | -0.034062575 | 0.659391763 | -0.368586762 | 0.712608558 |
| TPT1P8 | -0.044629557 | 0.626484479 | -0.36854473 | 0.712639871 |
| LINC02913 | 0.062548358 | -1.072174837 | 0.368495325 | 0.712676677 |
| HAVCR2 | -0.052442371 | 4.436640147 | -0.367719555 | 0.713254704 |
| RAB25 | 0.040412584 | -1.636467726 | 0.366819626 | 0.713925448 |
| IDI2-AS1 | 0.038692443 | -0.618312717 | 0.366744629 | 0.713981356 |
| COL5A3 | -0.059157305 | -1.40166413 | -0.366500003 | 0.714163726 |
| KLF1 | -0.080501196 | 3.282754212 | -0.366238166 | 0.714358945 |
| CCDC183-AS1 | -0.023490305 | 2.385987618 | -0.365989108 | 0.714544655 |
| AC111155.1 | 0.021658813 | 2.519679313 | 0.365923483 | 0.714593591 |
| NPPC | 0.053541992 | -1.181233783 | 0.365798506 | 0.714686788 |
| ZFHX3 | -0.056731812 | 2.68350664 | -0.365682552 | 0.71477326 |
| NOX5 | -0.081708542 | -4.812896749 | -0.365103919 | 0.71520483 |
| FAM101A | 0.073522456 | -2.303757432 | 0.364809474 | 0.715424475 |
| TTYH1 | -0.058608927 | -2.706507706 | -0.364224033 | 0.715861263 |
| XYLB | -0.044619136 | 1.073990556 | -0.364141294 | 0.715923 |
| RP11-106M3.2 | -0.039875683 | 0.28289979 | -0.364050624 | 0.715990658 |
| COL6A5 | -0.091537267 | -3.094034239 | -0.363848677 | 0.716141359 |
| F8 | -0.029222975 | 1.807174547 | -0.363665627 | 0.716277967 |
| LDLRAD3 | 0.081399837 | 2.517075785 | 0.363342607 | 0.716519056 |
| ETFDH | -0.013825755 | 4.673547392 | -0.363278426 | 0.716566961 |
| LILRB4 | -0.102585786 | 3.230648112 | -0.363089104 | 0.716708279 |
| TRIM3 | 0.02499476 | 2.592199678 | 0.362916505 | 0.716837124 |
| ARRB2 | 0.031399341 | 7.915189609 | 0.362138122 | 0.717418282 |
| CSAD | 0.022998004 | 4.682950217 | 0.361934519 | 0.717570324 |
| RPL35P2 | -0.021570863 | 7.448115125 | -0.361898898 | 0.717596926 |
| NOL12 | 0.015020374 | 3.260603726 | 0.361521234 | 0.717878982 |
| AC124944.3 | 0.040757094 | 0.279365188 | 0.360997759 | 0.718270001 |
| CTD-2314B22.3 | 0.104521553 | -3.913950129 | 0.36088773 | 0.718352198 |
| SPAG7 | -0.014210291 | 6.21334546 | -0.360778986 | 0.718433439 |
| SDHD | -0.020355311 | 5.691973883 | -0.360775993 | 0.718435675 |
| HOXA10 | 0.098585235 | 4.419671474 | 0.360470087 | 0.718664229 |
| SLMO1 | 0.064706976 | 1.013814191 | 0.359934308 | 0.719064591 |
| SLC25A15 | 0.027022102 | 4.224160077 | 0.359253674 | 0.719573308 |
| PRRT2 | -0.053692521 | -1.256127606 | -0.359127138 | 0.719667897 |
| GLYATL2 | -0.064537454 | -1.156641776 | -0.359123345 | 0.719670732 |
| AC097724.3 | -0.031966651 | 0.972814471 | -0.359054904 | 0.719721896 |
| RP11-551G24.2 | -0.040700112 | -1.032852359 | -0.358879617 | 0.719852938 |
| DLC1 | 0.07355561 | 2.491190838 | 0.358213889 | 0.720350701 |
| KIAA1875 | -0.025914168 | 0.905723713 | -0.357149301 | 0.721146939 |
| CSPG4 | -0.108890886 | -1.430348568 | -0.356527614 | 0.721612057 |
| S100A5 | 0.040592523 | 0.097071157 | 0.355921634 | 0.722065524 |
| ATP5J2 | -0.019827597 | 5.882074672 | -0.355611907 | 0.722297337 |
| CLPX | 0.010124728 | 5.489895221 | 0.35544362 | 0.7224233 |
| TPM3P8 | -0.017423637 | 5.884635682 | -0.35438348 | 0.723216995 |
| C11orf80 | -0.032685835 | 1.839565098 | -0.354365853 | 0.723230194 |
| WARS2 | 0.02003521 | 3.415208113 | 0.354252477 | 0.723315093 |
| RP3-395M20.12 | -0.034486118 | 3.603483108 | -0.354203193 | 0.723352 |
| PSAP | -0.029087481 | 10.65356238 | -0.353917411 | 0.723566021 |
| VAV2 | 0.042969289 | 3.281220816 | 0.35372202 | 0.723712361 |
| PHYH | 0.022969249 | 4.098358693 | 0.353154442 | 0.724137512 |
| RP11-495P10.7 | -0.060077791 | -0.631165598 | -0.352887899 | 0.724337198 |
| RP1-136J15.5 | 0.029411964 | 0.372955597 | 0.352780444 | 0.724417706 |
| FOXRED1 | -0.014340706 | 4.267228373 | -0.35274048 | 0.724447649 |
| RP11-588H23.3 | -0.0675695 | -2.26999256 | -0.352530131 | 0.724605258 |
| SOAT1 | 0.026175247 | 4.951746499 | 0.352503397 | 0.72462529 |
| TESK1 | 0.015791028 | 5.349068777 | 0.352445154 | 0.724668932 |
| RP11-30K9.5 | -0.063950373 | -1.004285491 | -0.352315492 | 0.724766093 |
| CDHR3 | 0.048707418 | -2.784580522 | 0.352124657 | 0.724909102 |
| ZDHHC17 | 0.012551375 | 5.246867374 | 0.351940602 | 0.725047039 |
| MMAB | -0.019991564 | 3.307326481 | -0.351716871 | 0.725214723 |
| FAM228B | -0.021437658 | 4.167784103 | -0.351607479 | 0.725296715 |
| GPR21 | 0.055645827 | -0.027906253 | 0.35149346 | 0.72538218 |
| SOX15 | 0.064807083 | 0.312061414 | 0.351482471 | 0.725390417 |
| PTHLH | 0.049803461 | -2.974686257 | 0.351206646 | 0.72559718 |
| GPR98 | 0.051537933 | -3.447640116 | 0.350960087 | 0.725782021 |
| ZNF849P | -0.041621328 | -1.094775543 | -0.350814343 | 0.725891291 |
| RP11-331F4.1 | 0.046994651 | -1.321108557 | 0.350417369 | 0.726188944 |
| RPL23 | 0.019131436 | 8.433926118 | 0.350112582 | 0.726417505 |
| RP11-64K12.1 | -0.033764947 | 0.082503557 | -0.349899491 | 0.726577316 |
| TEK | -0.064672134 | -1.1853447 | -0.34980134 | 0.72665093 |
| RTEL1 | -0.02575564 | -0.156429503 | -0.349738609 | 0.72669798 |
| PIK3CB | -0.020877027 | 5.602263157 | -0.349392238 | 0.726957788 |
| RP11-830F9.7 | -0.047279793 | 0.390598866 | -0.349082266 | 0.72719032 |
| NOTCH2NL | -0.023564373 | 3.666103348 | -0.348906155 | 0.727322444 |
| AP006222.2 | 0.048854649 | -0.103161409 | 0.348673665 | 0.727496878 |
| PGDP1 | -0.029680409 | 4.847676553 | -0.348472476 | 0.727647839 |
| OR1Q1 | -0.052227018 | -1.872072613 | -0.348277745 | 0.727793964 |
| CYP4A22-AS1 | -0.046922569 | -0.033409018 | -0.348070978 | 0.727949133 |
| RN7SL431P | 0.034628711 | 3.328385736 | 0.347828475 | 0.728131133 |
| IGHV3-71 | 0.044024824 | -0.749825327 | 0.347772049 | 0.728173483 |
| NXT2 | 0.023069982 | 5.772845448 | 0.347759013 | 0.728183268 |
| MIEN1 | 0.012273752 | 5.237754228 | 0.34769819 | 0.728228919 |
| LIMD2 | -0.019813419 | 7.138755673 | -0.347416406 | 0.728440429 |
| MIR621 | -0.03402576 | 3.311323698 | -0.347169543 | 0.728625744 |
| RP11-420G6.4 | 0.045761311 | 2.363659543 | 0.346976659 | 0.728770549 |
| CLIC1P1 | 0.021080327 | 3.627187978 | 0.346925177 | 0.7288092 |
| PPP1R3G | 0.083566081 | 0.170587702 | 0.346922586 | 0.728811145 |
| SQLE | 0.030159562 | 5.153122537 | 0.346757815 | 0.728934855 |
| CTIF | -0.020530066 | 3.787131516 | -0.346408397 | 0.72919722 |
| RP11-373D23.2 | 0.044632474 | 3.823948327 | 0.346379166 | 0.72921917 |
| RP11-93K22.6 | -0.04906946 | -0.327984156 | -0.345935607 | 0.729552272 |
| SGSM2 | 0.014873669 | 5.451424599 | 0.345612246 | 0.729795141 |
| AC093323.3 | 0.020244611 | 5.193907465 | 0.345176053 | 0.730122797 |
| NAV3 | 0.096079224 | -1.448355238 | 0.344908758 | 0.730323607 |
| IGKV1OR2-118 | 0.043560345 | -0.607091142 | 0.34443726 | 0.730677872 |
| ANKFN1 | 0.068316713 | -4.00287819 | 0.344409039 | 0.730699078 |
| RPL12P41 | 0.036586799 | -0.115500489 | 0.344040241 | 0.730976222 |
| BAI3 | -0.075850238 | -3.665389887 | -0.344025186 | 0.730987536 |
| ADAMTS7 | 0.073532002 | -1.294827259 | 0.343973827 | 0.731026134 |
| RP11-686D22.6 | 0.021124843 | 2.307122916 | 0.343836009 | 0.731129713 |
| TRIM73 | 0.043762245 | -0.328254209 | 0.343768216 | 0.731180665 |
| HADHAP1 | 0.023592991 | 2.070574003 | 0.343582996 | 0.73131988 |
| MAP2 | -0.055605001 | -4.055103561 | -0.342875601 | 0.731851654 |
| ARID1A | 0.012228631 | 7.076281161 | 0.342750934 | 0.731945384 |
| NAA35 | -0.011556019 | 4.700092761 | -0.342639324 | 0.7320293 |
| HMGCR | -0.018467451 | 5.19850886 | -0.342443764 | 0.732176345 |
| NRXN1 | -0.07256012 | -5.228075325 | -0.342191409 | 0.732366108 |
| RP4-765C7.2 | 0.030439683 | 4.723311853 | 0.341972819 | 0.732530494 |
| NANOS3 | 0.041938425 | 0.817005852 | 0.341816543 | 0.732648026 |
| HAUS1 | -0.014273353 | 5.175892444 | -0.341055087 | 0.733220792 |
| SOS2 | 0.014975561 | 6.498631305 | 0.340952947 | 0.733297633 |
| CNTNAP3B | 0.071521666 | -5.28199323 | 0.340898115 | 0.733338884 |
| RP11-320M16.1 | -0.037045025 | 1.477149234 | -0.340500728 | 0.733637874 |
| NDUFAF3 | -0.015038141 | 5.933479536 | -0.340457023 | 0.733670759 |
| RMDN2 | -0.047488452 | 1.035961987 | -0.340197261 | 0.733866225 |
| DGKA | -0.021498867 | 5.158760951 | -0.340165205 | 0.733890348 |
| RP11-452L6.7 | 0.029375334 | 1.102254695 | 0.339711446 | 0.734231839 |
| RP11-723O4.9 | -0.045355775 | 1.78413746 | -0.339691653 | 0.734246736 |
| RP11-451B8.1 | 0.058732323 | -0.939024538 | 0.339049893 | 0.734729807 |
| PPT2-EGFL8 | 0.037140809 | -2.642878859 | 0.339009482 | 0.734760229 |
| KDELC1 | -0.069156901 | 2.614433082 | -0.339008402 | 0.734761042 |
| LHFP | -0.064996097 | 1.918308799 | -0.33898978 | 0.734775061 |
| RP4-758J18.13 | 0.02451969 | 2.168910421 | 0.338743183 | 0.734960713 |
| RP11-571F15.3 | 0.021245385 | 4.951562935 | 0.338549212 | 0.735106756 |
| AC016700.5 | -0.034854812 | -0.039348684 | -0.338504896 | 0.735140124 |
| CTD-2555O16.4 | 0.030108137 | 1.584694606 | 0.338338918 | 0.735265101 |
| FOLR2 | 0.066022337 | 1.885837529 | 0.338131769 | 0.735421087 |
| AL118506.1 | 0.038833749 | 0.262774187 | 0.338131142 | 0.73542156 |
| RP11-1081L13.4 | -0.045457134 | -0.146561632 | -0.337955953 | 0.735553488 |
| IL2RA | 0.068501945 | 3.075316038 | 0.337405207 | 0.735968288 |
| RP11-307C12.11 | -0.029804147 | 1.86743944 | -0.337301049 | 0.736046745 |
| TTLL10-AS1 | -0.077853071 | -1.757380549 | -0.337289839 | 0.736055188 |
| CTC-548K16.2 | 0.033399472 | 0.730638333 | 0.33719162 | 0.736129174 |
| FASLG | 0.053320493 | -0.029380214 | 0.337054772 | 0.736232262 |
| GSTM2P1 | -0.032376496 | 2.917630984 | -0.336910394 | 0.736341027 |
| NUGGC | -0.044405096 | -0.68437556 | -0.33684343 | 0.736391475 |
| AC011747.4 | -0.047145293 | -2.272241311 | -0.336118164 | 0.736937938 |
| NRBF2P3 | 0.034469623 | -0.057353269 | 0.335922025 | 0.737085744 |
| UBE2W | -0.016187915 | 4.613568383 | -0.335813966 | 0.73716718 |
| RP11-649A18.5 | -0.032517736 | 1.244785132 | -0.335409917 | 0.737471706 |
| IRF8 | 0.066745678 | 5.652242908 | 0.335263474 | 0.737582088 |
| RPL38P3 | -0.054305213 | 0.974123605 | -0.335179338 | 0.737645508 |
| MICC | 0.041551906 | -1.26132979 | 0.335156478 | 0.73766274 |
| VPS26B | 0.011425634 | 6.120099454 | 0.334861207 | 0.737885326 |
| TRERF1 | 0.025669116 | 5.228453383 | 0.334664168 | 0.738033874 |
| CTD-2027G2.1 | 0.04622773 | 0.542544289 | 0.334337489 | 0.738280179 |
| PSMG3 | -0.019044655 | 5.271145198 | -0.334317748 | 0.738295064 |
| CTD-2357A8.3 | -0.057615379 | -0.384035274 | -0.334293973 | 0.738312991 |
| AC010492.2 | -0.029991594 | 2.443580508 | -0.334088216 | 0.738468141 |
| SH2B2 | 0.03522935 | 3.521016572 | 0.333812729 | 0.738675887 |
| DCHS1 | 0.057960984 | 2.560195475 | 0.333686278 | 0.73877125 |
| RP11-350N15.6 | 0.042602364 | -0.925725869 | 0.333626061 | 0.738816665 |
| CYP7B1 | -0.084534782 | 1.632404884 | -0.33344005 | 0.738956956 |
| NINL | 0.050328402 | -0.845430714 | 0.33313722 | 0.739185373 |
| RP11-407N17.4 | -0.029102118 | 0.995780182 | -0.332927726 | 0.739343402 |
| AGK | -0.015515211 | 3.999401248 | -0.332805345 | 0.739435724 |
| SLC43A3 | -0.022433018 | 5.324541516 | -0.332700581 | 0.739514758 |
| RP11-125B21.2 | -0.05173963 | -2.716998766 | -0.332627487 | 0.739569902 |
| RP11-624D20.1 | 0.030261259 | -0.559791868 | 0.332003938 | 0.74004038 |
| MT-CO1 | -0.011522013 | 14.23047764 | -0.331846551 | 0.740159147 |
| CD48 | 0.054729286 | 6.39280777 | 0.331702114 | 0.740268147 |
| NPIPB3 | 0.020242388 | 4.652240634 | 0.331662998 | 0.740297667 |
| UGT3A2 | 0.085059112 | 0.547374727 | 0.331553966 | 0.740379953 |
| RP11-445P17.8 | 0.038636149 | 0.386786109 | 0.331496312 | 0.740423465 |
| JAK3 | -0.028516412 | 6.276710393 | -0.331064134 | 0.740749663 |
| CTD-2523D13.2 | -0.038677306 | -1.045322917 | -0.330981912 | 0.740811728 |
| RP11-62H7.2 | -0.044113559 | -1.009646931 | -0.330821459 | 0.740932849 |
| RP11-77P16.4 | -0.039728457 | 2.617694486 | -0.330536999 | 0.741147595 |
| DCTD | 0.014308189 | 6.373393546 | 0.330434713 | 0.741224819 |
| CYP2J2 | -0.045265796 | -2.127936826 | -0.330226749 | 0.741381834 |
| CACNB4 | -0.054695441 | 1.6515406 | -0.329883812 | 0.741640781 |
| MYH11 | 0.066530848 | -2.297815894 | 0.329575352 | 0.741873719 |
| RP4-799D16.1 | 0.040963682 | -1.430279566 | 0.32952854 | 0.741909071 |
| SPA17 | 0.043279273 | 0.638075927 | 0.329435068 | 0.741979664 |
| ELANE | 0.08676832 | 9.196689215 | 0.329403105 | 0.742003804 |
| OR1H1P | -0.050410964 | -1.476023898 | -0.329314825 | 0.742070477 |
| DDR1 | -0.057706678 | 0.121087434 | -0.328833727 | 0.742433864 |
| KIAA1683 | 0.032926004 | 3.602676642 | 0.328623014 | 0.74259304 |
| CTD-2554C21.3 | -0.057456579 | -2.179143034 | -0.328315391 | 0.742825442 |
| AC017104.6 | -0.036465969 | -0.086267464 | -0.328097754 | 0.742989876 |
| PDGFRA | 0.058042324 | -3.874759739 | 0.328031823 | 0.743039693 |
| GPR141 | -0.046103762 | 3.784790702 | -0.327640241 | 0.743335586 |
| DENND4C | 0.019099432 | 4.917281402 | 0.327581857 | 0.743379706 |
| F11R | 0.025151081 | 5.678640882 | 0.327479143 | 0.743457328 |
| DOK3 | 0.030004186 | 7.158960688 | 0.326650352 | 0.744083751 |
| PREX2 | 0.08826416 | -4.020178903 | 0.326505707 | 0.744193095 |
| EEF1A1P19 | 0.021612858 | 7.631199347 | 0.326273525 | 0.744368623 |
| ADPRM | -0.022148171 | 5.393360982 | -0.325905881 | 0.744646587 |
| RP11-477G18.1 | -0.034519919 | -0.277528291 | -0.325872992 | 0.744671455 |
| PPIA | 0.015158345 | 7.522689972 | 0.325863656 | 0.744678514 |
| RP11-90O23.1 | -0.024870815 | 3.904825826 | -0.325582168 | 0.744891363 |
| MT-TM | 0.041781211 | 4.565595078 | 0.325500639 | 0.744953016 |
| CCAR1 | 0.01051213 | 6.396419342 | 0.324918022 | 0.74539364 |
| RP11-553L6.5 | -0.030139428 | 4.097139504 | -0.324734908 | 0.745532144 |
| FAM26F | -0.042011448 | 5.131182891 | -0.324696243 | 0.745561391 |
| UNC93B2 | -0.046693374 | -0.847063342 | -0.324655732 | 0.745592034 |
| SCN4A | 0.051516556 | -1.876468436 | 0.324407363 | 0.745779913 |
| C3orf27 | -0.058136208 | -1.903275675 | -0.324395505 | 0.745788884 |
| CTD-2012K14.6 | -0.034365718 | 1.28800532 | -0.324178408 | 0.74595312 |
| RP11-461G12.2 | -0.040842649 | -0.648586234 | -0.32416668 | 0.745961993 |
| ENPP1 | 0.04306391 | -4.535733151 | 0.323755978 | 0.746272727 |
| TNNT1 | 0.087935688 | 1.954425502 | 0.323070322 | 0.746791581 |
| FOXO4 | -0.01589941 | 5.800798312 | -0.32300617 | 0.746840133 |
| PAQR5 | 0.065928435 | -0.813317132 | 0.322997238 | 0.746846892 |
| RP11-564D11.3 | 0.04049229 | -0.049012352 | 0.32272473 | 0.747053144 |
| ACOXL | 0.041846846 | -3.630554586 | 0.322675688 | 0.747090264 |
| RP11-307L3.2 | -0.017482883 | 5.425292806 | -0.322105619 | 0.747521793 |
| CA8 | -0.071087397 | -0.139372839 | -0.321894104 | 0.747681925 |
| RP11-369K17.1 | 0.057073275 | 0.312214149 | 0.321773444 | 0.747773278 |
| MTRNR2L10 | -0.041634439 | -0.589220716 | -0.321709836 | 0.747821438 |
| ACTBP8 | -0.056183808 | 0.217198551 | -0.321545097 | 0.747946173 |
| GGTLC2 | -0.044794492 | -1.215958725 | -0.321438955 | 0.748026543 |
| LL0XNC01-116E7.4 | -0.039131111 | -0.815104834 | -0.321319553 | 0.748116957 |
| WDR11-DT | -0.040814971 | -1.354977966 | -0.321273727 | 0.748151658 |
| SEPT7 | -0.009675556 | 6.688217866 | -0.320659495 | 0.748616832 |
| UBQLN2 | -0.014627359 | 6.327300088 | -0.320560269 | 0.748691987 |
| RIBC1 | -0.02933019 | 0.515278336 | -0.320178611 | 0.748981081 |
| FBP1 | 0.066841793 | 4.774923534 | 0.319788854 | 0.749276347 |
| GNG5P2 | -0.031810412 | 0.217490955 | -0.319786227 | 0.749278337 |
| LAMTOR5-AS1 | -0.025935602 | -0.01484053 | -0.319160612 | 0.749752358 |
| POLD2 | -0.022157907 | 6.164154653 | -0.318788779 | 0.750034136 |
| LMF2 | -0.010203788 | 6.798819811 | -0.318610153 | 0.750169512 |
| HIST1H1C | 0.039670557 | 7.522795134 | 0.31852287 | 0.750235665 |
| RASL10A | 0.046751595 | 0.976102123 | 0.318029802 | 0.750609399 |
| LPAR3 | 0.069721626 | -1.263835997 | 0.317554391 | 0.750969805 |
| ADRA2A | 0.067460347 | -0.142570875 | 0.317477236 | 0.751028301 |
| AGR2 | -0.089905975 | -1.482762229 | -0.31676023 | 0.751571976 |
| AC073869.20 | 0.021448715 | 1.357769267 | 0.315454765 | 0.752562172 |
| KBTBD11-OT1 | 0.038948542 | -1.00626459 | 0.315450479 | 0.752565423 |
| PLCH1 | 0.06627369 | -0.552535629 | 0.315223275 | 0.7527378 |
| CLUH | -0.017501715 | 5.662994187 | -0.315202154 | 0.752753824 |
| ST3GAL3 | -0.024312583 | 4.220857397 | -0.315077617 | 0.752848315 |
| CTD-2210P15.2 | 0.023628342 | 1.806706014 | 0.314835439 | 0.753032074 |
| PURG | -0.061507599 | -3.009977075 | -0.314815352 | 0.753047316 |
| DOK4 | 0.041251174 | 2.665282331 | 0.314579481 | 0.753226303 |
| ZSCAN16-AS1 | 0.016232652 | 3.155120186 | 0.314026528 | 0.753645957 |
| ZNF542 | 0.029197594 | 2.884550264 | 0.313934637 | 0.753715704 |
| SUPT16H | 0.016407736 | 6.788371054 | 0.313797321 | 0.753819932 |
| RNU6-890P | -0.035917089 | 0.395122059 | -0.313746262 | 0.753858689 |
| LPIN1 | -0.027853103 | 4.743921138 | -0.313673355 | 0.75391403 |
| TWISTNB | 0.029501464 | 5.933592368 | 0.313509044 | 0.754038759 |
| ZNF697 | 0.040203993 | 2.12581864 | 0.313473963 | 0.75406539 |
| RGS2 | 0.047104353 | 8.173882108 | 0.312679506 | 0.754668562 |
| ST3GAL6-AS1 | 0.053489474 | 0.653378701 | 0.31221803 | 0.755018995 |
| NIT2 | 0.020181601 | 4.661938399 | 0.311940444 | 0.755229811 |
| DCBLD1 | 0.052286162 | 0.950329017 | 0.311859108 | 0.755291586 |
| AC079922.2 | -0.018951969 | 5.420444739 | -0.311695585 | 0.755415787 |
| RP11-15B24.5 | -0.035254841 | -1.805261308 | -0.311153272 | 0.755827738 |
| DESI1 | 0.018662296 | 5.172034131 | 0.310953319 | 0.755979643 |
| HNRNPH1P3 | 0.028319238 | 4.713145031 | 0.310904756 | 0.756016538 |
| ITPK1 | 0.016099896 | 5.940346655 | 0.310736803 | 0.756144143 |
| HS3ST4 | 0.070485747 | -3.156431444 | 0.310642141 | 0.756216066 |
| SIAE | 0.047425359 | 3.43386505 | 0.310495457 | 0.756327519 |
| RPL37AP1 | -0.023696248 | 3.792865809 | -0.310140047 | 0.756597588 |
| ST6GAL2 | 0.056165915 | -4.144038132 | 0.309760135 | 0.756886309 |
| BTF3P12 | -0.02342055 | 1.667672175 | -0.309128971 | 0.757366047 |
| DYNAP | 0.050671159 | -2.5601368 | 0.309041208 | 0.757432762 |
| PARP15 | -0.028626293 | 4.614422914 | -0.308942375 | 0.757507894 |
| RP11-616K22.1 | -0.03126012 | 0.243947655 | -0.308830941 | 0.757592608 |
| CACNA1F | -0.036534882 | 0.683332586 | -0.308357124 | 0.757952844 |
| RPL5P30 | -0.021722082 | 6.398766435 | -0.307248653 | 0.758795805 |
| STAP1 | 0.045464071 | 4.711281621 | 0.307188698 | 0.758841408 |
| RP11-215A21.2 | 0.01689977 | 4.612021737 | 0.30673041 | 0.759190013 |
| RP11-161H23.10 | 0.028502181 | 0.123400454 | 0.306601523 | 0.759288062 |
| RNF125 | -0.027767175 | 5.639520451 | -0.306048874 | 0.759708527 |
| KCNIP1 | 0.059849558 | -2.429311506 | 0.305605988 | 0.760045534 |
| APOOL | 0.012867263 | 4.076765305 | 0.305504358 | 0.760122875 |
| UVSSA | 0.015953525 | 4.192549725 | 0.305377096 | 0.760219724 |
| GPR161 | -0.04675268 | -1.39060415 | -0.3051409 | 0.760399485 |
| RPS7P14 | -0.032404602 | -0.124737117 | -0.304915313 | 0.760571183 |
| C5orf27 | 0.038426749 | -0.969968846 | 0.304543441 | 0.760854249 |
| LOC124685 | 0.026683906 | 1.929068082 | 0.304392418 | 0.760969215 |
| GNPDA1 | -0.040195908 | 4.28101213 | -0.30412672 | 0.76117149 |
| RPL26P35 | -0.033998909 | 0.884613882 | -0.304036592 | 0.761240108 |
| TMEM170B | 0.038986662 | 5.774376186 | 0.303675694 | 0.761514893 |
| SLC39A9 | 0.010626262 | 5.5931091 | 0.303658809 | 0.76152775 |
| RP11-396M11.1 | -0.037444008 | -0.407297233 | -0.303618108 | 0.761558741 |
| CTAGE16P | 0.040410288 | -0.891833036 | 0.303590644 | 0.761579654 |
| SLC4A7 | -0.031527176 | 3.773203874 | -0.303305295 | 0.761796943 |
| ZNF830 | -0.017761579 | 5.667311457 | -0.303029231 | 0.76200718 |
| RP11-253E3.3 | -0.053047408 | -2.453867348 | -0.302837524 | 0.762153185 |
| RP6-159A1.2 | 0.013057677 | 5.007855804 | 0.302523581 | 0.762392304 |
| KRT17P1 | -0.06163559 | -0.764420535 | -0.302136092 | 0.762687472 |
| SMPD3 | -0.047642768 | 0.383104971 | -0.301821206 | 0.76292736 |
| STARD3NL | 0.012762708 | 5.59741777 | 0.301422907 | 0.763230828 |
| LINC00342 | -0.026810492 | 5.408472303 | -0.301232426 | 0.76337597 |
| BNIP3 | -0.030623909 | 3.816198781 | -0.301009248 | 0.763546037 |
| AC008268.2 | 0.064496909 | -2.268100366 | 0.300930891 | 0.763605749 |
| VTI1BP2 | -0.020412459 | 1.862693428 | -0.300200593 | 0.764162347 |
| GPR15 | 0.052760215 | -0.066462401 | 0.300196745 | 0.76416528 |
| RP11-819C21.1 | -0.018993902 | 1.855453275 | -0.29989142 | 0.76439802 |
| RP11-7D5.2 | -0.029589216 | -0.474280098 | -0.299795607 | 0.76447106 |
| SLC25A38 | -0.015755284 | 6.178509907 | -0.299637627 | 0.764591495 |
| HLA-DOA | 0.057001796 | 4.54861612 | 0.299613878 | 0.7646096 |
| CTD-2369P2.8 | 0.04118129 | -2.301127066 | 0.299302802 | 0.764846766 |
| SCPEP1 | 0.047736107 | 6.323029415 | 0.299224288 | 0.764906629 |
| RP11-326F20.5 | -0.030324841 | 0.83315054 | -0.299092345 | 0.765007232 |
| OR2A7 | 0.042887729 | -0.912366328 | 0.299069169 | 0.765024903 |
| C14orf1 | -0.015890048 | 5.449211921 | -0.298077839 | 0.765780899 |
| ZDHHC2 | 0.025696643 | 5.625154852 | 0.29804547 | 0.765805588 |
| RP11-252A24.3 | 0.028666364 | -1.057870573 | 0.297406667 | 0.766292866 |
| 8-Mar | 0.017716874 | 5.639543908 | 0.297314725 | 0.766363008 |
| UCP3 | -0.019695114 | 2.081684181 | -0.296885125 | 0.766690768 |
| CTNNBIP1 | -0.016644679 | 5.685427853 | -0.296645906 | 0.766873296 |
| MTA1 | -0.012742113 | 6.175021575 | -0.296632214 | 0.766883743 |
| ARIH2OS | 0.016333285 | 2.790403549 | 0.29632386 | 0.767119044 |
| TRPV6 | -0.073441652 | -3.109295265 | -0.296241246 | 0.767182088 |
| ADIRF-AS1 | -0.040217343 | 1.270252354 | -0.296046664 | 0.767330585 |
| GRID2IP | 0.040958952 | -1.412998035 | 0.296035866 | 0.767338826 |
| RUSC2 | -0.032929828 | 4.652860627 | -0.296026518 | 0.76734596 |
| NCF2 | -0.053078829 | 7.837370637 | -0.296021958 | 0.767349441 |
| RP11-723O4.6 | -0.058055157 | -0.45944095 | -0.29553197 | 0.76772342 |
| KIAA1671 | 0.043631888 | -1.232991675 | 0.295465354 | 0.767774268 |
| DBH-AS1 | -0.04469852 | 0.881974412 | -0.295409051 | 0.767817246 |
| SBF2 | 0.025307213 | 3.740771977 | 0.295380209 | 0.767839261 |
| ZYG11B | 0.010714613 | 4.693475178 | 0.295092099 | 0.768059194 |
| FAR1 | -0.021942857 | 6.42046132 | -0.294879676 | 0.768221363 |
| POLR2E | 0.00947429 | 6.465086328 | 0.294112399 | 0.768807203 |
| ZNF700 | 0.017957728 | 4.903784429 | 0.29390094 | 0.768968682 |
| TIAM2 | 0.029224066 | 0.406602437 | 0.293409402 | 0.769344079 |
| RP11-424N24.2 | 0.031896695 | -0.176892127 | 0.293356469 | 0.769384509 |
| CTD-2231H16.1 | 0.050316329 | -1.506684142 | 0.293286858 | 0.769437677 |
| C9orf43 | -0.042790843 | 3.104489386 | -0.292990096 | 0.769664355 |
| GINS1 | -0.036583978 | 2.723152346 | -0.292690508 | 0.769893211 |
| LINC00960 | -0.043415607 | -3.016432952 | -0.292645917 | 0.769927276 |
| LINC00877 | -0.04312566 | 2.256244622 | -0.292638937 | 0.769932609 |
| AGTRAP | -0.035868905 | 6.493716666 | -0.292548248 | 0.770001891 |
| CEBPD | 0.052101403 | 7.336298157 | 0.292497305 | 0.770040811 |
| KAZN | -0.050668603 | -1.024322442 | -0.292295951 | 0.770194646 |
| FAM60BP | 0.029305939 | 1.182069411 | 0.291854976 | 0.770531583 |
| CT45A4 | 0.053598286 | -2.598385246 | 0.291847675 | 0.770537162 |
| RP1-5O6.4 | -0.034305641 | 0.109125325 | -0.291747581 | 0.770613647 |
| RP11-670E13.3 | -0.032677057 | 0.508316266 | -0.291455056 | 0.77083719 |
| STIM1 | 0.010048925 | 6.591944243 | 0.291449142 | 0.770841709 |
| CRAT | 0.016393232 | 5.239082506 | 0.290961472 | 0.77121442 |
| LRPAP1 | -0.020878583 | 6.150313684 | -0.290605279 | 0.771486681 |
| RNF157 | -0.040761626 | 1.160921597 | -0.29059173 | 0.771497038 |
| RP5-1074L1.4 | 0.025208306 | 3.547397888 | 0.290340289 | 0.771689248 |
| TRHDE-AS1 | 0.053394419 | -4.635393108 | 0.289642804 | 0.772222502 |
| IGLV1-50 | 0.046937865 | 0.539042087 | 0.289347333 | 0.772448435 |
| CTB-152G17.6 | -0.030918326 | -0.021993514 | -0.289234498 | 0.772534719 |
| RP11-807H22.6 | 0.041098325 | -1.289591871 | 0.288923498 | 0.772772553 |
| FBXO42 | 0.009915073 | 4.559283302 | 0.288457142 | 0.773129235 |
| HDHD3 | -0.017192607 | 4.46675288 | -0.288176646 | 0.77334379 |
| RP11-40C6.2 | 0.016761917 | 10.12782789 | 0.288127724 | 0.773381212 |
| ABI2 | -0.02900759 | 3.995857003 | -0.287919513 | 0.773540489 |
| RNPEPL1 | -0.012611086 | 6.108331736 | -0.287740366 | 0.773677539 |
| S100A1 | -0.063244298 | -0.940367029 | -0.287368434 | 0.773962097 |
| RP11-262M14.2 | -0.016099048 | 3.107069019 | -0.287333055 | 0.773989166 |
| RP11-634B7.4 | 0.046771544 | -1.282692495 | 0.287253925 | 0.774049711 |
| AP001065.2 | 0.05084211 | -2.65652433 | 0.28713654 | 0.774139529 |
| SOX18 | -0.059644117 | 0.493545134 | -0.286987246 | 0.774253767 |
| ANKRD24 | 0.028216673 | 0.621912319 | 0.286098671 | 0.774933795 |
| FMN1 | -0.057519793 | 0.352732723 | -0.285812278 | 0.775153008 |
| CP | 0.067305357 | -0.367295954 | 0.285671814 | 0.77526053 |
| RP5-864K19.4 | -0.01680476 | 1.948318323 | -0.285658601 | 0.775270645 |
| RP3-365I19.2 | -0.020502769 | 2.73089786 | -0.285656342 | 0.775272374 |
| UBAP2 | 0.013832876 | 4.999179527 | 0.285305365 | 0.775541058 |
| RMND5B | -0.023418556 | 3.425386309 | -0.284903385 | 0.775848822 |
| GGT7 | -0.027406327 | 3.849719937 | -0.284736683 | 0.775976462 |
| NKD1 | 0.046777871 | -0.317081834 | 0.284251786 | 0.776347771 |
| MIRLET7D | 0.032830068 | 0.532692259 | 0.283954227 | 0.776575653 |
| CTXN1 | -0.04379594 | 0.140814308 | -0.283729445 | 0.776747811 |
| RP11-295G20.2 | 0.034081086 | 2.64271603 | 0.283179023 | 0.777169421 |
| PABPC3 | -0.01487731 | 6.542401907 | -0.283040581 | 0.777275475 |
| RP11-723O4.2 | 0.029624982 | 0.788389535 | 0.282660615 | 0.77756657 |
| ISG15 | -0.035826269 | 5.814368335 | -0.282508056 | 0.777683455 |
| PTH2R | 0.069598434 | 2.321810006 | 0.281945189 | 0.778114748 |
| PPP1R35 | -0.012629776 | 6.422670345 | -0.281931169 | 0.778125492 |
| PCGF3 | 0.016469378 | 6.049433629 | 0.281877394 | 0.7781667 |
| NOS3 | -0.038794648 | -0.997061941 | -0.281795705 | 0.778229301 |
| PPWD1 | -0.011476847 | 5.928036065 | -0.281749612 | 0.778264624 |
| DNAJC15 | -0.021686167 | 5.400820232 | -0.281128585 | 0.778740588 |
| TRAJ5 | -0.034073779 | -0.499118041 | -0.281046022 | 0.778803871 |
| AP4B1 | -0.011773398 | 5.03834778 | -0.280857145 | 0.778948649 |
| CCDC167 | 0.021262356 | 4.13388907 | 0.280590431 | 0.779153103 |
| AC005740.4 | -0.04515347 | -0.597303808 | -0.280516769 | 0.779209573 |
| RAB27B | 0.050623676 | 3.571208522 | 0.280218765 | 0.779438036 |
| BAIAP3 | -0.044718341 | 1.889496983 | -0.280126194 | 0.779509009 |
| AP000654.5 | 0.02302532 | 0.764472315 | 0.279971598 | 0.77962754 |
| LDHB | -0.020470742 | 9.011865617 | -0.279607076 | 0.779907044 |
| CES1 | -0.095449361 | 1.855853837 | -0.279580262 | 0.779927605 |
| RP11-118H4.1 | -0.033006692 | -0.2994564 | -0.279548113 | 0.779952258 |
| FCER2 | -0.056953277 | 3.083712176 | -0.279469244 | 0.780012737 |
| KB-1980E6.3 | 0.035522298 | -0.975998334 | 0.279236232 | 0.780191426 |
| CHIC1 | -0.034857305 | 2.149616642 | -0.27916725 | 0.780244327 |
| DIRAS3 | -0.064175342 | -0.734187689 | -0.278874606 | 0.780468766 |
| IGHV3-49 | 0.054424011 | 1.493154894 | 0.278760105 | 0.780556586 |
| LINC00035 | -0.037882868 | -0.299072695 | -0.278744013 | 0.780568928 |
| PCBP1 | 0.008168578 | 10.30386344 | 0.278254573 | 0.780944351 |
| RP11-10B2.1 | 0.045369108 | -0.089482232 | 0.278009879 | 0.781132062 |
| LCLAT1 | -0.0139501 | 2.88160928 | -0.277982043 | 0.781153416 |
| UNC93B7 | -0.050624934 | 1.131716971 | -0.277685214 | 0.78138114 |
| ZC3HC1 | 0.011934477 | 4.097276092 | 0.277620817 | 0.781430547 |
| RP11-632F7.1 | 0.032046116 | -0.286337986 | 0.27743825 | 0.781570622 |
| GS1-24F4.2 | -0.044767133 | 0.553370157 | -0.276937148 | 0.781955131 |
| ADK | -0.013143175 | 5.359553707 | -0.276803364 | 0.782057796 |
| GPC3 | -0.049247083 | -2.335841794 | -0.276257571 | 0.782476673 |
| SDF2 | 0.010223011 | 4.931483076 | 0.27605833 | 0.7826296 |
| AL590452.1 | -0.042319935 | 0.127874464 | -0.275944506 | 0.782716969 |
| GHITM | 0.00872037 | 8.689092454 | 0.275730572 | 0.782881187 |
| GS1-259H13.2 | -0.035292627 | -0.856097459 | -0.275538613 | 0.783028546 |
| RP11-54O7.1 | 0.060165 | 0.184894724 | 0.274940478 | 0.783487758 |
| CHD1L | 0.014760844 | 7.086791746 | 0.27468803 | 0.783681595 |
| PPP2R3C | -0.009301069 | 5.59036883 | -0.274410169 | 0.78389496 |
| RP11-876N24.4 | -0.036379358 | 3.112009551 | -0.27409472 | 0.784137209 |
| NCK2 | -0.015904147 | 6.361524137 | -0.274054357 | 0.784168207 |
| MAPK12 | 0.039345397 | 3.287961133 | 0.274038342 | 0.784180507 |
| CRYBB2P1 | 0.01158655 | 3.238132863 | 0.273726709 | 0.784419849 |
| OR2B6 | 0.038381836 | -0.796427981 | 0.273684728 | 0.784452093 |
| BET1L | 0.010132385 | 5.461700145 | 0.273431096 | 0.784646907 |
| ZNF205 | -0.066199407 | -0.500292064 | -0.273199069 | 0.784825137 |
| AC002456.2 | 0.034584716 | 0.192931096 | 0.273070704 | 0.784923745 |
| RP11-7F17.3 | -0.045122733 | -2.616446793 | -0.272859757 | 0.785085799 |
| XYLT1 | 0.02141269 | 5.537501251 | 0.272073379 | 0.785689992 |
| ADAMTSL4-AS1 | -0.033357625 | 1.676859893 | -0.27203291 | 0.785721089 |
| LYPLA1 | 0.010964561 | 6.458767888 | 0.271977791 | 0.785763443 |
| RP11-255J3.2 | -0.019383921 | 2.038885412 | -0.271444282 | 0.786173436 |
| PRELID2 | 0.030436495 | 2.691688492 | 0.271141275 | 0.786406318 |
| SLC7A10 | -0.043771459 | -2.715786791 | -0.271092859 | 0.786443531 |
| RSPH1 | 0.043595534 | 0.201947076 | 0.27085114 | 0.786629325 |
| RP11-747H12.4 | -0.023886058 | 0.969284075 | -0.270677419 | 0.786762861 |
| AC013717.3 | -0.029258584 | 0.796556235 | -0.27065164 | 0.786782677 |
| PTGIS | -0.048788021 | -3.954870991 | -0.270138345 | 0.787177277 |
| TMEM231 | 0.030586913 | 0.97088261 | 0.269875183 | 0.787379605 |
| MBL1P | 0.041198256 | -2.24306278 | 0.268906653 | 0.788124373 |
| AC006946.15 | 0.044173316 | -1.279542435 | 0.2688653 | 0.788156176 |
| SNORA71B | 0.025490355 | 1.96296748 | 0.268828694 | 0.788184329 |
| DCUN1D3 | -0.021349723 | 3.559591426 | -0.26830871 | 0.788584267 |
| STRADB | 0.016309773 | 4.747590045 | 0.267390087 | 0.789290951 |
| PTPN4 | 0.015711782 | 3.331591161 | 0.266954571 | 0.789626047 |
| C1QTNF9B-AS1 | 0.029468957 | -0.150447559 | 0.266897353 | 0.789670075 |
| C1QB | 0.068980448 | 2.56030804 | 0.26684516 | 0.789710237 |
| LSG1 | 0.011458071 | 5.717770989 | 0.266729299 | 0.789799393 |
| ARHGAP44 | 0.05419264 | -3.470937917 | 0.266531886 | 0.789951309 |
| IGHV3-38 | -0.03313238 | -0.638868098 | -0.266447994 | 0.790015869 |
| MAPK9 | 0.008604581 | 4.616988138 | 0.266227 | 0.790185946 |
| RP11-380M21.2 | -0.025185599 | 4.777967848 | -0.266091774 | 0.79029002 |
| EEF1A1P11 | 0.015596268 | 7.260561264 | 0.26583728 | 0.790485897 |
| HTRA1 | 0.045468374 | -1.546007965 | 0.265264709 | 0.790926639 |
| AC126365.1 | 0.028030788 | 0.33965169 | 0.26523144 | 0.790952249 |
| AP006621.8 | 0.030987846 | 0.023722358 | 0.265179847 | 0.790991967 |
| SEPT7P8 | -0.025094827 | 0.548583618 | -0.265064969 | 0.791080406 |
| CHRNE | 0.032537059 | 2.695259335 | 0.264994642 | 0.791134547 |
| GPC4 | 0.08146581 | -0.977821315 | 0.264927648 | 0.791186125 |
| LUM | 0.066579195 | -1.193917992 | 0.264756469 | 0.791317916 |
| COL9A2 | 0.036227612 | 4.803500022 | 0.264397616 | 0.791594218 |
| COL27A1 | 0.048597123 | -1.665263519 | 0.264350471 | 0.791630519 |
| MKRN5P | -0.0310641 | -1.272443966 | -0.264280521 | 0.791684381 |
| RPL39P38 | -0.025746453 | 1.51594679 | -0.264043374 | 0.791866994 |
| C16orf96 | 0.037238916 | -0.170063531 | 0.263663145 | 0.792159811 |
| RP11-231C14.3 | -0.021325592 | 1.226442567 | -0.26322841 | 0.792494638 |
| LINC01037 | 0.040597667 | -0.8495857 | 0.263108657 | 0.792586877 |
| RP11-34P13.14 | 0.037096428 | 1.955220491 | 0.263002881 | 0.792668353 |
| RP1-161N10.1 | 0.037140276 | -1.308456831 | 0.262960381 | 0.79270109 |
| DNAH7 | -0.037078405 | -2.898141217 | -0.262826724 | 0.792804046 |
| FOLH1 | -0.050797842 | -2.819511762 | -0.262736877 | 0.792873257 |
| RPRD2 | 0.008868562 | 5.701027301 | 0.262714452 | 0.792890532 |
| ST8SIA5 | 0.039949264 | -3.009582578 | 0.262444461 | 0.793098523 |
| C17orf50 | 0.039551317 | -0.811219117 | 0.26209018 | 0.793371471 |
| RBP7 | 0.059120882 | 1.975463457 | 0.261816905 | 0.793582026 |
| C1orf145 | -0.039708761 | -0.119884574 | -0.261772313 | 0.793616385 |
| ANKRD31 | -0.036201765 | -2.336970497 | -0.261755032 | 0.793629701 |
| NUDT14 | -0.026633601 | 3.174903784 | -0.261033702 | 0.79418556 |
| DLGAP2 | -0.071410687 | -3.226188567 | -0.26048397 | 0.794609255 |
| BEND2 | -0.04224817 | -3.558814533 | -0.260377306 | 0.794691472 |
| NCF1 | 0.052700751 | 5.947630536 | 0.259915753 | 0.795047262 |
| RP11-428J1.4 | -0.036997436 | -1.278021493 | -0.259888622 | 0.795068177 |
| LSM6 | -0.012940309 | 5.033452699 | -0.259715115 | 0.795201938 |
| PDGFC | -0.042075857 | 3.860758055 | -0.259177323 | 0.795616574 |
| COQ3 | -0.017826909 | 2.743619337 | -0.25917308 | 0.795619844 |
| CTD-2108O9.4 | -0.027766927 | 0.437870998 | -0.259169411 | 0.795622674 |
| RP11-13N13.2 | 0.022945511 | 1.524129865 | 0.258805356 | 0.795903392 |
| PTCHD3P1 | 0.012780629 | 4.164340171 | 0.258759939 | 0.795938414 |
| PTPN13 | 0.042213629 | -0.211920876 | 0.25874676 | 0.795948577 |
| EFR3B | -0.037775822 | -3.440718574 | -0.258326421 | 0.796272732 |
| MOCS1 | -0.052170384 | -2.173037714 | -0.258312016 | 0.796283842 |
| PRX | -0.038363498 | 2.170903056 | -0.258207924 | 0.796364121 |
| LIPE-AS1 | 0.015835337 | 1.042438125 | 0.258060497 | 0.796477826 |
| RP11-321E8.5 | 0.046129905 | -1.197355824 | 0.25775182 | 0.796715909 |
| TYRO3 | -0.04145744 | -0.528776063 | -0.257701167 | 0.796754979 |
| FAT3 | 0.066013847 | -5.010254343 | 0.257651191 | 0.796793528 |
| AP003774.1 | -0.026400013 | 1.319912076 | -0.257143063 | 0.797185501 |
| RP11-43A14.1 | -0.032091653 | 0.222128716 | -0.256968297 | 0.797320328 |
| CCP110 | 0.011899506 | 4.744341938 | 0.256954233 | 0.797331177 |
| RPL31P63 | -0.014507603 | 4.085022793 | -0.256876846 | 0.797390882 |
| PDZD2 | 0.060011308 | -1.118310672 | 0.256542716 | 0.797648676 |
| LINC01122 | -0.046939407 | -1.116364662 | -0.256297866 | 0.797837602 |
| PLA2G16 | -0.04808174 | 1.834249638 | -0.255460391 | 0.798483887 |
| RP11-440G5.2 | -0.031487459 | -0.060773301 | -0.255340535 | 0.798576392 |
| DOCK6 | 0.053301727 | 0.323787267 | 0.255275215 | 0.798626807 |
| GALC | 0.025674102 | 5.007421914 | 0.254992975 | 0.798844655 |
| POP7 | 0.014114435 | 5.5185868 | 0.254904237 | 0.79891315 |
| NPIPA2 | 0.014178793 | 3.689785661 | 0.254588802 | 0.799156644 |
| CTD-2162K18.4 | 0.03205631 | 0.042640741 | 0.25445217 | 0.79926212 |
| AC020983.5 | 0.031160912 | 3.877122691 | 0.254198011 | 0.799458334 |
| RNU6-807P | 0.022891242 | 1.139935701 | 0.254188794 | 0.79946545 |
| RP11-890B15.3 | -0.013956779 | 3.781017439 | -0.25414841 | 0.799496627 |
| AC005786.7 | 0.025423525 | 2.280083807 | 0.253997529 | 0.799613117 |
| RP11-65J3.1 | 0.040061558 | 1.840034122 | 0.253790562 | 0.799772916 |
| MTF2 | 0.01101762 | 5.434981522 | 0.253700484 | 0.799842467 |
| SIK3 | 0.009013378 | 5.771633872 | 0.253267701 | 0.800176653 |
| KCNS1 | 0.041314418 | -3.245608467 | 0.253190054 | 0.800236614 |
| POMC | 0.058421294 | 0.942401338 | 0.25262829 | 0.800670459 |
| RPL41P2 | 0.012993576 | 4.692474875 | 0.251995348 | 0.801159347 |
| ARL14EP | -0.013074643 | 4.577979266 | -0.251893936 | 0.801237686 |
| SLC9A3R1 | 0.016498153 | 8.303622969 | 0.251542553 | 0.801509137 |
| AC132186.1 | 0.032072517 | -0.855741032 | 0.251350336 | 0.801657639 |
| RP4-603I14.3 | 0.026255969 | 0.366595547 | 0.251269828 | 0.80171984 |
| RP11-259G18.2 | 0.03476503 | -2.012514661 | 0.250786075 | 0.802093615 |
| KLHL36 | -0.010084855 | 4.907932252 | -0.250286186 | 0.802479905 |
| IGLC5 | 0.031305315 | -0.845278341 | 0.24991426 | 0.802767343 |
| SNORD6 | -0.030183897 | 0.695829315 | -0.249878116 | 0.802795278 |
| HIST2H2AA3 | 0.038230204 | 2.191537806 | 0.249487747 | 0.803097001 |
| C20orf196 | 0.011198338 | 3.692039983 | 0.249431422 | 0.803140538 |
| RP11-350G8.5 | -0.02579826 | -0.915907259 | -0.248935922 | 0.803523566 |
| CTC-360J11.4 | 0.017409127 | 1.019655189 | 0.24889847 | 0.80355252 |
| NR3C1 | -0.010763387 | 6.151350585 | -0.248885574 | 0.803562489 |
| RIPK1 | -0.009670057 | 5.977013686 | -0.24869564 | 0.803709325 |
| RPL10P1 | 0.024897861 | 0.626144614 | 0.247640107 | 0.804525473 |
| FSTL4 | 0.034735659 | -3.8354248 | 0.247146065 | 0.804907545 |
| CDC42SE1 | 0.015317035 | 8.283789153 | 0.246902726 | 0.80509575 |
| RP11-66B24.7 | -0.039970912 | -2.290007825 | -0.246847451 | 0.805138503 |
| RAB3IP | 0.02690245 | 2.601920049 | 0.246469281 | 0.805431017 |
| C12orf23 | -0.022709786 | 4.506926332 | -0.246075014 | 0.805736012 |
| CCND3 | 0.016472174 | 7.427272898 | 0.245492772 | 0.806186473 |
| HLA-DRA | 0.03550005 | 10.95424 | 0.245243815 | 0.806379102 |
| NPM1P5 | 0.015497726 | 3.355007768 | 0.245205954 | 0.806408398 |
| RP11-490H24.5 | 0.020545072 | 5.765274279 | 0.245118504 | 0.806476065 |
| GPC1 | -0.042913567 | 0.246215314 | -0.24503709 | 0.806539063 |
| RP11-337A23.5 | -0.027617536 | -0.428955668 | -0.244737096 | 0.80677121 |
| SEPT9 | -0.014285036 | 6.703402756 | -0.244640671 | 0.806845831 |
| MRPL42 | 0.009193131 | 5.795415393 | 0.24453471 | 0.806927833 |
| NPM1P31 | 0.027216061 | 1.008075353 | 0.244272826 | 0.807130513 |
| RPS4XP13 | -0.018750994 | 5.705643072 | -0.244189126 | 0.807195293 |
| PCDHGB3 | 0.040377616 | 0.064529271 | 0.244125796 | 0.807244309 |
| RP11-1348G14.5 | 0.023539795 | 0.327464669 | 0.244112285 | 0.807254767 |
| TRIM72 | -0.045518331 | -0.845169742 | -0.244085241 | 0.807275698 |
| IGHV4-31 | -0.045649149 | 1.3118218 | -0.243470375 | 0.807751633 |
| ABHD17AP3 | -0.021070514 | 0.649619757 | -0.243446791 | 0.80776989 |
| AC096574.4 | 0.02087508 | -0.159396691 | 0.243223341 | 0.807942869 |
| SFXN5 | -0.015645118 | 3.70715559 | -0.242383772 | 0.808592889 |
| C3orf70 | 0.038523623 | -3.176325829 | 0.24233152 | 0.808633348 |
| MAPRE2 | -0.014194798 | 6.389337052 | -0.242284332 | 0.808669887 |
| FAM160B2 | 0.010693759 | 4.476857681 | 0.241717892 | 0.809108528 |
| RP4-549L20.3 | -0.024853212 | 0.456871728 | -0.241502142 | 0.809275616 |
| BTF3P5 | -0.011437936 | 5.144967817 | -0.2408944 | 0.809746332 |
| RP11-474N24.2 | -0.031985154 | -1.16356736 | -0.240489267 | 0.81006016 |
| SREBF2 | 0.01405221 | 6.959243695 | 0.240139764 | 0.810330919 |
| PGAP2 | -0.010995882 | 4.353864775 | -0.240048241 | 0.810401825 |
| LRIG2 | 0.008594691 | 3.567021983 | 0.239734834 | 0.810644645 |
| AC025627.9 | 0.056635263 | 0.206923533 | 0.239462614 | 0.810855569 |
| ARMC4 | -0.038248067 | -3.027603143 | -0.239224227 | 0.811040291 |
| CCDC104 | 0.016984235 | 4.505124545 | 0.238945449 | 0.811256323 |
| KIT | -0.046150492 | 5.915182716 | -0.238816316 | 0.811356396 |
| CYCS | 0.012025374 | 5.974231036 | 0.238567065 | 0.811549566 |
| HIST1H2BO | -0.032274699 | 1.612153433 | -0.238526555 | 0.811580962 |
| RP11-532F12.5 | -0.033087301 | -0.115135588 | -0.238403977 | 0.811675965 |
| AK5 | 0.057071382 | 0.998969331 | 0.237893546 | 0.8120716 |
| TMEM156 | -0.023077232 | 4.212258403 | -0.237772748 | 0.812165237 |
| KCTD5 | 0.01363686 | 5.222299158 | 0.236871143 | 0.812864209 |
| HERC3 | 0.009759117 | 4.977294636 | 0.236035379 | 0.81351227 |
| TTLL13 | 0.024379845 | -1.596892468 | 0.236031297 | 0.813515436 |
| RP4-631H13.6 | 0.011277983 | 5.836723257 | 0.235505043 | 0.813923566 |
| AC027612.6 | -0.026229976 | 2.012295079 | -0.235157531 | 0.814193102 |
| RP11-162G10.1 | 0.023590032 | -0.217953199 | 0.235134499 | 0.814210967 |
| CTD-2179L22.1 | -0.028453494 | -0.870537258 | -0.235114084 | 0.814226803 |
| CSNK1A1 | 0.008869902 | 6.313490977 | 0.234787687 | 0.814479984 |
| ST18 | -0.073320978 | -1.862531174 | -0.234465267 | 0.814730101 |
| SYBU | 0.037673545 | -3.24698272 | 0.234376181 | 0.814799213 |
| FUT11 | 0.01034971 | 4.501657215 | 0.234178726 | 0.8149524 |
| TTC39A | -0.039566875 | -2.676885782 | -0.233546301 | 0.815443089 |
| ETS1 | -0.028092917 | 6.429356545 | -0.233406755 | 0.815551371 |
| IGKV2D-29 | 0.041787113 | 0.739189028 | 0.232946136 | 0.815908816 |
| IMMTP1 | -0.023073092 | -1.11456515 | -0.231846266 | 0.816762482 |
| COL4A6 | -0.056649985 | -4.188516419 | -0.231348511 | 0.817148887 |
| TMEM86B | -0.010178532 | 4.492030355 | -0.230856989 | 0.817530498 |
| TCEA1P4 | 0.018582283 | 1.157923457 | 0.230745121 | 0.817617356 |
| RP13-216E22.4 | 0.021371299 | 2.754301849 | 0.23023801 | 0.818011125 |
| RIMS3 | 0.024355671 | 1.451323143 | 0.230103775 | 0.818115366 |
| CBLN3 | -0.023103534 | 0.829052513 | -0.229601565 | 0.818505387 |
| AC093677.1 | -0.02548167 | -0.653699456 | -0.229593106 | 0.818511956 |
| AFF1 | -0.013090591 | 6.948531832 | -0.229522185 | 0.818567037 |
| NDOR1 | -0.008836516 | 6.064382215 | -0.229155804 | 0.818851606 |
| OGFR | -0.013038579 | 6.26445217 | -0.228661262 | 0.819235755 |
| RIMS2 | -0.042539978 | -5.153156927 | -0.228589042 | 0.819291857 |
| RPL21P5 | 0.026016333 | -0.303541274 | 0.228425166 | 0.819419164 |
| EIF4A3 | -0.012572058 | 5.995105438 | -0.228246625 | 0.81955787 |
| CDH9 | -0.065456038 | -2.499799181 | -0.227638007 | 0.820030734 |
| KLHL29 | -0.036659375 | -3.724503286 | -0.227423984 | 0.820197035 |
| GFI1B | -0.04525475 | 4.486874397 | -0.227227292 | 0.820349877 |
| RPL9P28 | 0.025153018 | 0.121005968 | 0.227058757 | 0.820480844 |
| AC093642.5 | -0.012744706 | 3.159093462 | -0.227041304 | 0.820494406 |
| HESX1 | -0.024595874 | 3.13281578 | -0.226927317 | 0.820582988 |
| TRIM58 | 0.032635394 | 4.800214414 | 0.22679767 | 0.820683742 |
| FAM83A | -0.058511556 | -0.861570542 | -0.22642706 | 0.820971774 |
| CTC-429P9.3 | -0.01287059 | 4.365875962 | -0.225695886 | 0.821540103 |
| CSTB | -0.018211489 | 6.695069818 | -0.225376835 | 0.821788125 |
| ARL8B | 0.011181201 | 7.376544639 | 0.225063284 | 0.822031888 |
| RP11-375N15.2 | -0.019846553 | 1.518961006 | -0.224651571 | 0.822351993 |
| RP11-68I3.4 | -0.020627202 | 2.253956079 | -0.224506077 | 0.822465121 |
| RP11-150O12.6 | -0.033117815 | -1.160867279 | -0.224347174 | 0.822588678 |
| IMPDH1P6 | 0.020326517 | -0.155798225 | 0.224156082 | 0.822737272 |
| ZNF749 | 0.013968567 | 2.910799464 | 0.223834834 | 0.822987088 |
| CLEC4E | 0.056046173 | 3.172262102 | 0.22377174 | 0.823036155 |
| TNFRSF19 | -0.047514376 | -0.698510617 | -0.223706686 | 0.823086747 |
| JHDM1D-AS1 | -0.023911317 | 2.600085121 | -0.222772566 | 0.823813283 |
| SETP4 | 0.022411772 | 0.260916947 | 0.222513433 | 0.824014857 |
| CTD-2228K2.7 | -0.030407452 | 3.721873761 | -0.222322834 | 0.824163127 |
| CERS6-AS1 | 0.028687804 | -2.787946575 | 0.222158076 | 0.824291301 |
| FBXO46 | 0.008061453 | 4.751889747 | 0.222107078 | 0.824330975 |
| STEAP4 | 0.055560799 | 1.612617331 | 0.221684947 | 0.824659397 |
| AL441883.1 | 0.021615583 | 0.822173325 | 0.221526094 | 0.824782994 |
| SORT1 | -0.042193102 | 4.875287525 | -0.221398854 | 0.824881998 |
| FAM83H | 0.032601102 | 2.26605608 | 0.221374472 | 0.824900969 |
| EMC2 | 0.009899638 | 4.464206928 | 0.220988291 | 0.825201469 |
| CACNA1C-AS2 | -0.030908431 | 3.436677512 | -0.220748578 | 0.82538801 |
| KLHL12 | 0.00809035 | 5.543803045 | 0.220407064 | 0.825653789 |
| AC004980.10 | -0.021701146 | 0.230987558 | -0.220284812 | 0.825748935 |
| SCG2 | 0.049855377 | -0.525699313 | 0.22016826 | 0.825839647 |
| C11orf82 | 0.023455755 | 2.419854619 | 0.219980147 | 0.82598606 |
| RP11-401P9.4 | 0.036532149 | -0.44523702 | 0.219917202 | 0.826035052 |
| IKZF3 | 0.030586155 | 3.08054321 | 0.219864492 | 0.826076079 |
| CTD-3064M3.7 | -0.03163537 | -2.061076638 | -0.219840172 | 0.826095009 |
| NAGS | 0.029749571 | -1.439795142 | 0.219828785 | 0.826103872 |
| FAHD2B | 0.017936102 | 3.395794024 | 0.21982865 | 0.826103978 |
| CACNG6 | 0.033143702 | -1.946955961 | 0.219798036 | 0.826127807 |
| SLC29A4 | 0.037175998 | -1.079236085 | 0.219784153 | 0.826138612 |
| LINC00265 | 0.015456405 | 3.301706746 | 0.219661632 | 0.826233981 |
| UBE2MP1 | -0.007493633 | 6.628629782 | -0.219440634 | 0.826406009 |
| DDX26B | -0.009262723 | 4.861538492 | -0.218688657 | 0.826991419 |
| BMPR2 | -0.02851131 | 3.410884011 | -0.218633724 | 0.827034188 |
| RP1-140K8.2 | 0.026467343 | 0.156683182 | 0.218506527 | 0.827133221 |
| EIF4B | 0.010592893 | 9.174783108 | 0.218185012 | 0.827383559 |
| TDH | 0.026526088 | -1.332773775 | 0.21812708 | 0.827428668 |
| TTC39B | 0.02502401 | 1.714107486 | 0.217849814 | 0.827644569 |
| PARP16 | -0.011326312 | 4.378659681 | -0.217824005 | 0.827664666 |
| HOXA5 | 0.071012915 | 4.585629445 | 0.217748833 | 0.827723204 |
| RP11-22P6.3 | 0.023867587 | 0.643345972 | 0.217490217 | 0.827924599 |
| TPI1P3 | 0.025502919 | 0.700378337 | 0.217118126 | 0.828214382 |
| RP11-325K4.3 | -0.023416462 | 2.16245994 | -0.216915722 | 0.828372023 |
| ARAP3 | -0.039066789 | 3.333254516 | -0.216509923 | 0.828688098 |
| MYLK2 | 0.03175152 | -1.572079398 | 0.216470988 | 0.828718426 |
| ASIC1 | 0.039081778 | -1.153235725 | 0.216442143 | 0.828740895 |
| HOXA2 | -0.052228309 | 0.627870055 | -0.216385118 | 0.828785314 |
| KLHDC7B | -0.027204873 | 3.019089948 | -0.216215991 | 0.828917058 |
| PQBP1 | -0.007456415 | 6.869432909 | -0.216090214 | 0.829015037 |
| HNRNPCP5 | -0.021972906 | -0.734953383 | -0.21606496 | 0.82903471 |
| PHYHD1 | 0.030818689 | 0.716798985 | 0.215595312 | 0.829400587 |
| CAV1 | 0.050657726 | 0.659749883 | 0.215530093 | 0.829451398 |
| CTD-2620I22.2 | -0.020338452 | 0.053612421 | -0.215337528 | 0.829601428 |
| TMEM123 | 0.010766442 | 9.118139663 | 0.215162751 | 0.829737604 |
| HNRNPR | -0.008064832 | 7.305272879 | -0.214605045 | 0.83017217 |
| EFCAB4B | 0.009695266 | 3.699012434 | 0.213991051 | 0.830650657 |
| IGDCC4 | 0.035689733 | -3.045767392 | 0.213745164 | 0.830842295 |
| C1orf213 | -0.022332444 | 2.000672904 | -0.213726143 | 0.83085712 |
| DOCK5 | 0.020523458 | 5.876802253 | 0.213573307 | 0.830976242 |
| AC012671.2 | 0.023888102 | -0.016138694 | 0.213542966 | 0.83099989 |
| RP11-3N2.13 | -0.046559377 | -1.600910022 | -0.213501715 | 0.831032043 |
| METTL18 | -0.014333469 | 3.727754324 | -0.213468052 | 0.831058281 |
| SORCS3 | 0.031580191 | -3.809351635 | 0.213415607 | 0.831099159 |
| SLC1A4 | -0.021532724 | 4.965283154 | -0.213354465 | 0.831146816 |
| MTATP8P1 | 0.020454994 | 3.774173372 | 0.213177917 | 0.831284431 |
| OLA1 | -0.01057775 | 6.157088413 | -0.21286585 | 0.831527692 |
| TXNL4B | -0.007157532 | 4.339890576 | -0.212765544 | 0.831605885 |
| AC007879.2 | 0.025361758 | -0.253909826 | 0.212641344 | 0.831702707 |
| SUCLG2P2 | 0.028771285 | 3.842791871 | 0.21223258 | 0.832021385 |
| CRABP1 | -0.049403664 | -0.348986109 | -0.212078661 | 0.832141389 |
| HOXA9 | 0.070189995 | 5.339513657 | 0.212045362 | 0.832167352 |
| RN7SL146P | -0.021494603 | 0.007910288 | -0.211908021 | 0.832274435 |
| FNBP1L | 0.034423933 | 3.269049515 | 0.211742269 | 0.832403674 |
| RN7SL368P | -0.035117339 | 3.296993025 | -0.211695599 | 0.832440064 |
| RP1-151F17.1 | 0.02341804 | 1.547389341 | 0.211528158 | 0.832570626 |
| SRD5A3-AS1 | 0.019357868 | -1.124376001 | 0.211468486 | 0.832617156 |
| C15orf38 | -0.042269158 | 0.650384846 | -0.211165133 | 0.83285371 |
| RP11-251M1.1 | 0.04951426 | 1.461064518 | 0.210902723 | 0.833058349 |
| MESP1 | -0.022963145 | -0.247311863 | -0.210892286 | 0.833066489 |
| RP11-249L21.4 | -0.024273376 | 3.661818475 | -0.210854676 | 0.83309582 |
| FOXK2 | 0.007647744 | 5.523946261 | 0.210569959 | 0.83331787 |
| EXTL3-AS1 | -0.026837739 | -0.610290627 | -0.210355425 | 0.833485193 |
| TRIM71 | 0.052076519 | 1.9232377 | 0.210113219 | 0.833674108 |
| AGMAT | 0.022727994 | 1.64753681 | 0.209912725 | 0.833830495 |
| SNX24 | -0.024510339 | 1.595816938 | -0.209689492 | 0.834004628 |
| KANK2 | -0.024407176 | 3.896936778 | -0.209514257 | 0.834141325 |
| TMEM178B | -0.028867848 | -3.786976851 | -0.208924666 | 0.83460129 |
| SLC16A9 | -0.05104832 | 0.381999477 | -0.20890788 | 0.834614386 |
| PTPN7 | 0.013771466 | 6.793135465 | 0.208589513 | 0.834862782 |
| PTGER2 | -0.021910834 | 6.975610852 | -0.20853484 | 0.834905441 |
| CPNE3 | 0.024006865 | 6.164164801 | 0.208453945 | 0.83496856 |
| TEX10 | -0.01139183 | 4.486319461 | -0.208443462 | 0.834976739 |
| ADCY1 | -0.048836062 | -3.235154497 | -0.2084054 | 0.835006438 |
| OR52N4 | -0.027750403 | 0.302380019 | -0.208387354 | 0.835020519 |
| SFR1 | 0.008703946 | 3.634598188 | 0.20782847 | 0.83545663 |
| RP11-386I14.4 | -0.026809605 | 6.991755499 | -0.207658677 | 0.835589133 |
| SPIN2A | 0.02628388 | -1.901573731 | 0.207631755 | 0.835610143 |
| ANP32E | -0.012098705 | 7.642140513 | -0.207481708 | 0.835727241 |
| RPL10P3 | 0.030373683 | 3.354176783 | 0.206964763 | 0.8361307 |
| AC098831.4 | -0.029472041 | 0.380573124 | -0.206539365 | 0.836462742 |
| SGCA | -0.02600948 | -2.1141205 | -0.206316707 | 0.836636548 |
| ITGB8 | 0.051391088 | -0.764073274 | 0.206299059 | 0.836650325 |
| AGT | -0.048000139 | -2.053373436 | -0.205887696 | 0.836971455 |
| RPL23AP3 | -0.022194508 | 0.291240909 | -0.205623629 | 0.837177613 |
| ASPA | 0.031166348 | -3.922716127 | 0.205519669 | 0.837258778 |
| SDCBP | 0.016731017 | 7.437335769 | 0.205406473 | 0.837347156 |
| P4HA3 | -0.030112564 | -2.612481678 | -0.205402839 | 0.837349993 |
| RNY5P8 | -0.025124658 | -0.19461326 | -0.205311598 | 0.837421231 |
| GPC2 | 0.024735372 | 1.808212126 | 0.205135074 | 0.83755906 |
| WDPCP | -0.010341709 | 2.18922564 | -0.205031616 | 0.837639841 |
| MEX3D | -0.019910565 | 2.406457067 | -0.204800418 | 0.83782037 |
| ACSM1 | -0.030314187 | -0.183679547 | -0.20467206 | 0.8379206 |
| KIAA0319 | -0.031973652 | -2.849164177 | -0.204297643 | 0.838212985 |
| FOXP2 | -0.044029501 | -5.642602257 | -0.204154268 | 0.838324954 |
| UBL7-DT | 0.007461186 | 3.933019508 | 0.203968055 | 0.838470382 |
| PNOC | -0.03219706 | 0.716452714 | -0.203855794 | 0.838558058 |
| LRRD1 | -0.02761899 | -3.449114543 | -0.203823677 | 0.838583142 |
| RNU2-7P | 0.022943109 | 0.115980281 | 0.203820266 | 0.838585805 |
| TRBC2 | -0.030918719 | 7.376912755 | -0.203688831 | 0.83868846 |
| RP11-570G20.1 | -0.021476399 | -0.45903534 | -0.203631007 | 0.838733622 |
| NUP88 | 0.007790037 | 5.395643076 | 0.203168771 | 0.839094666 |
| EYA1 | -0.042420632 | -3.148203978 | -0.203086332 | 0.839159061 |
| SDCBPP3 | 0.018881921 | 4.7998327 | 0.202964709 | 0.839254066 |
| RPL5P12 | -0.010504263 | 5.820425279 | -0.202963105 | 0.839255319 |
| MRPL40 | -0.00723489 | 4.971884556 | -0.202818749 | 0.839368085 |
| LINC00982 | -0.068134081 | -1.19383493 | -0.202399027 | 0.839695975 |
| CAMKK1 | 0.017447482 | 3.187927728 | 0.202379794 | 0.839711 |
| TMX4 | 0.01135175 | 6.882388881 | 0.202349748 | 0.839734474 |
| C14orf80 | 0.014870097 | 2.719050046 | 0.202345353 | 0.839737907 |
| NTNG2 | -0.034288479 | 4.40444016 | -0.201520984 | 0.840381998 |
| HLA-U | -0.023180823 | 0.41140642 | -0.201478921 | 0.840414865 |
| SLC12A6 | 0.011410796 | 6.635833005 | 0.201470205 | 0.840421676 |
| TMUB1 | 0.008136496 | 6.383950307 | 0.201450412 | 0.840437142 |
| RP3-402G11.27 | -0.019609736 | 1.143441354 | -0.20105251 | 0.840748072 |
| CPM | -0.041736366 | 1.950376263 | -0.200900901 | 0.840866549 |
| PDLIM2 | -0.015055694 | 3.940786442 | -0.199821466 | 0.841710194 |
| IGF2R | 0.032522296 | 6.048610034 | 0.199445505 | 0.842004073 |
| SLC25A45 | -0.011742215 | 3.295737992 | -0.199373578 | 0.842060299 |
| AC142381.1 | 0.023053271 | -0.949918937 | 0.198812566 | 0.842498876 |
| KCNJ2-AS1 | -0.035700371 | -0.326382335 | -0.19873832 | 0.842556922 |
| LDHAP1 | 0.023325076 | -0.754765057 | 0.198581561 | 0.842679481 |
| IFI27L2 | -0.021733158 | 4.332432689 | -0.198503298 | 0.842740671 |
| LRRC37A17P | 0.024332595 | 0.958218583 | 0.198436893 | 0.84279259 |
| AC113189.5 | 0.01080218 | 2.964061923 | 0.198338753 | 0.842869323 |
| TUBA8 | -0.02275426 | -1.369852565 | -0.198180153 | 0.84299333 |
| TMEM55A | -0.019596153 | 5.069804494 | -0.197981161 | 0.843148926 |
| RP11-400K9.1 | -0.022862564 | 0.321734238 | -0.19779124 | 0.843297434 |
| RCN3 | -0.033278694 | 3.552847862 | -0.1975231 | 0.843507115 |
| WARS | -0.016905302 | 6.896773864 | -0.197359731 | 0.843634873 |
| AC018865.5 | -0.014633018 | 2.866626317 | -0.197322315 | 0.843664133 |
| GMPPB | 0.017735495 | -1.980282421 | 0.196787933 | 0.844082061 |
| RP11-1407O15.2 | 0.009760934 | 3.172112766 | 0.196726751 | 0.844129912 |
| MLLT6 | 0.011832785 | 6.551973937 | 0.196716366 | 0.844138035 |
| SHF | -0.027147654 | 1.718255276 | -0.196709592 | 0.844143333 |
| ARL2 | -0.014519299 | 5.129778072 | -0.196690987 | 0.844157884 |
| RP1-181J22.1 | 0.014014786 | 3.514665924 | 0.196517223 | 0.844293793 |
| MTX1P1 | 0.025832301 | 0.710611356 | 0.196468683 | 0.84433176 |
| MAGED4B | 0.041520256 | -3.058718788 | 0.196365838 | 0.844412202 |
| RHOQP3 | -0.015493124 | 3.640986459 | -0.1962401 | 0.844510554 |
| NUCB2 | -0.020429447 | 7.320856657 | -0.196060114 | 0.844651341 |
| OLFM1 | 0.041170418 | -3.033597345 | 0.196014477 | 0.844687041 |
| SMIM10 | 0.035832444 | 3.811079412 | 0.196011585 | 0.844689303 |
| PGM3 | -0.010556586 | 3.142782179 | -0.195781021 | 0.844869662 |
| SNORA5C | 0.020097735 | 0.792276097 | 0.195574458 | 0.845031255 |
| SLC48A1 | 0.012528334 | 4.435642166 | 0.194938002 | 0.845529188 |
| RP11-90H3.2 | 0.01690532 | 0.429254437 | 0.194664278 | 0.845743356 |
| ITGAM | -0.030450068 | 7.488490658 | -0.194314868 | 0.846016759 |
| OTUD7A | -0.019269439 | -2.674311215 | -0.194305085 | 0.846024413 |
| AC119673.1 | 0.026930597 | -1.390264498 | 0.193797309 | 0.846421767 |
| GPAA1 | 0.008756281 | 6.78944575 | 0.193774092 | 0.846439936 |
| PNKP | 0.005890831 | 5.669050072 | 0.193721777 | 0.846480877 |
| FAM49A | 0.024870507 | 4.714049576 | 0.193651053 | 0.846536225 |
| FCN1 | 0.046605794 | 8.918973987 | 0.193412551 | 0.846722881 |
| MYL6P3 | -0.01732057 | 1.51777823 | -0.193194175 | 0.846893793 |
| EVL | 0.01631437 | 5.394712759 | 0.193193914 | 0.846893997 |
| KRT18P15 | -0.025106085 | -1.545048219 | -0.19312765 | 0.84694586 |
| CAPN5 | -0.025131758 | 1.103407176 | -0.193114764 | 0.846955945 |
| CTD-2162K18.5 | 0.021730893 | 2.682758421 | 0.192873004 | 0.84714517 |
| CUX2 | 0.039886571 | -2.080646288 | 0.192480745 | 0.847452209 |
| FEZ1 | 0.041484289 | -0.563499888 | 0.192306158 | 0.847588874 |
| ZHX1 | -0.007254744 | 5.35685681 | -0.192123664 | 0.847731733 |
| C20orf203 | 0.036457391 | -0.242837728 | 0.190838073 | 0.848738255 |
| HLA-DRB5 | 0.038744541 | 8.00767178 | 0.190681939 | 0.848860514 |
| RP11-416N2.4 | 0.032820722 | 0.046372929 | 0.19057574 | 0.848943673 |
| NSUN5 | 0.009969629 | 4.996207787 | 0.190513778 | 0.848992193 |
| COX14 | -0.006699535 | 5.387518369 | -0.190151958 | 0.849275534 |
| SENP7 | -0.008136224 | 5.55306045 | -0.190150716 | 0.849276506 |
| ASB9 | 0.036288101 | 1.782937397 | 0.189714127 | 0.849618425 |
| NOTUM | 0.028769627 | -0.847478416 | 0.189653715 | 0.849665738 |
| MOSPD3 | -0.012446166 | 5.266318177 | -0.189508254 | 0.849779665 |
| PSTPIP2 | -0.015329762 | 6.32834214 | -0.18917348 | 0.850041875 |
| DYNC1I1 | 0.038018174 | -2.431977541 | 0.18917078 | 0.85004399 |
| CDSN | 0.029701934 | -2.29614851 | 0.189094853 | 0.850103462 |
| HK2P1 | -0.019087463 | 2.157228885 | -0.188834253 | 0.85030759 |
| RP11-222A11.1 | -0.041886948 | -1.60769358 | -0.188691126 | 0.850419706 |
| SLCO2A1 | 0.038731927 | -2.622347082 | 0.1885933 | 0.850496338 |
| TRAPPC3L | -0.024245584 | -2.740584642 | -0.188396342 | 0.850650629 |
| TUSC2 | 0.007729485 | 5.08231638 | 0.188174295 | 0.850824581 |
| COX5B | 0.00615053 | 8.052776078 | 0.187481562 | 0.851367316 |
| UBE2A | -0.00504994 | 6.78721406 | -0.187396193 | 0.851434205 |
| KDM5C | -0.007345601 | 6.623654322 | -0.187064448 | 0.851694147 |
| HTR1B | 0.033146748 | -2.226977409 | 0.186792128 | 0.851907537 |
| ABCB10P4 | -0.01998362 | -1.699914739 | -0.186787695 | 0.85191101 |
| DYM | -0.007997989 | 5.991818212 | -0.186583722 | 0.852070851 |
| PPAP2A | -0.024530209 | 2.648562867 | -0.186363807 | 0.852243192 |
| KXD1 | 0.004680017 | 6.293677667 | 0.186286291 | 0.852303941 |
| COL19A1 | -0.037640688 | -2.379458545 | -0.185339929 | 0.853045666 |
| AC079767.4 | 0.02781161 | 0.219367723 | 0.185332723 | 0.853051315 |
| RP11-290F5.2 | 0.022057405 | -0.008508982 | 0.18510265 | 0.853231658 |
| RAP1AP | -0.013226907 | 2.853640787 | -0.185086273 | 0.853244495 |
| RP11-105N14.1 | -0.026940274 | 1.58892403 | -0.185038677 | 0.853281804 |
| RP11-473P24.2 | -0.019173738 | -0.375881355 | -0.184791731 | 0.853475384 |
| RPL19P12 | 0.00878108 | 4.783894608 | 0.184452629 | 0.853741219 |
| SUGCT | 0.018892204 | -2.08731352 | 0.184320913 | 0.853844481 |
| IGSF22 | -0.024575595 | -2.754670529 | -0.184276691 | 0.85387915 |
| USP32 | -0.009795144 | 5.778592124 | -0.184031132 | 0.85407167 |
| RP11-379K17.4 | -0.020446066 | -3.286124732 | -0.183767078 | 0.8542787 |
| HLA-DQA1 | 0.042627634 | 3.700331796 | 0.183713965 | 0.854320344 |
| CNKSR1 | 0.018964564 | 0.710226718 | 0.183571785 | 0.854431824 |
| LTBP4 | -0.019733463 | 4.105520823 | -0.183483914 | 0.854500723 |
| MAP4K2 | -0.008551581 | 6.428023963 | -0.183246892 | 0.854686578 |
| IGHM | 0.031305983 | 8.539003118 | 0.183066539 | 0.854828001 |
| CTD-2213F21.1 | -0.019621342 | 0.145094922 | -0.182764375 | 0.855064953 |
| MTERFD3 | 0.017125202 | 2.998706953 | 0.182679041 | 0.855131874 |
| PDZD4 | 0.0291891 | 1.702653225 | 0.182551624 | 0.855231798 |
| MAP3K4 | 0.005500648 | 4.648435743 | 0.182439341 | 0.855319855 |
| BMPER | 0.025233796 | -3.516276854 | 0.181891644 | 0.855749409 |
| SNORD69 | 0.018176803 | 1.454106143 | 0.181773352 | 0.855842191 |
| GRIK5 | -0.035226396 | 4.027602988 | -0.181757095 | 0.855854942 |
| CAPS2 | 0.01785475 | 0.835767147 | 0.181728336 | 0.855877499 |
| ST3GAL5 | 0.020910991 | 3.521861698 | 0.181720682 | 0.855883502 |
| C16orf91 | -0.009591573 | 4.148145973 | -0.181717701 | 0.855885841 |
| RP1-225E12.2 | 0.028300994 | -2.978945669 | 0.181618202 | 0.855963884 |
| MRPS2 | 0.007853513 | 5.782817355 | 0.181161456 | 0.856322157 |
| BTF3P8 | 0.015649886 | 1.400736271 | 0.180820779 | 0.856589405 |
| RP11-616M22.3 | -0.027890276 | -0.943197284 | -0.180656537 | 0.856718252 |
| RCOR2 | -0.032899544 | -0.030718544 | -0.180404175 | 0.856916236 |
| ZSCAN2 | 0.014054547 | 3.600196914 | 0.180399156 | 0.856920174 |
| CACNA1D | 0.022055321 | -1.80664693 | 0.180343655 | 0.856963717 |
| ARHGAP12 | -0.014065353 | 4.316673262 | -0.179768966 | 0.857414614 |
| GAPDHP14 | 0.030694805 | -1.410147696 | 0.179472481 | 0.857647252 |
| RHPN2 | -0.02295836 | -3.124539738 | -0.179448322 | 0.857666209 |
| RP11-526K21.2 | 0.020660284 | 0.714881154 | 0.179294409 | 0.857786983 |
| RP11-338C15.3 | 0.024490773 | -0.136410687 | 0.179170071 | 0.857884552 |
| UBE2L2 | 0.017345035 | 1.107811327 | 0.179168955 | 0.857885428 |
| RP11-413E1.4 | -0.024130155 | 0.00786686 | -0.178969459 | 0.858041979 |
| IGHV4-34 | 0.030471583 | 2.175203451 | 0.178873287 | 0.85811745 |
| RP11-430L17.1 | 0.014058518 | 3.396370442 | 0.178723609 | 0.858234912 |
| TPM3P6 | -0.013032874 | 3.053293398 | -0.17858183 | 0.85834618 |
| RP11-325P15.2 | 0.011598818 | 1.778867466 | 0.178178845 | 0.858662453 |
| NCAPD2P1 | -0.014793903 | 1.076482138 | -0.17786018 | 0.858912567 |
| KIAA1324 | 0.028097337 | -0.341770115 | 0.177625739 | 0.859096585 |
| SLC5A6 | -0.011121228 | 4.752889147 | -0.177620554 | 0.859100654 |
| RP11-6L6.2 | 0.024172668 | -0.620081135 | 0.177523526 | 0.859176816 |
| CD40 | 0.023848929 | 2.915082515 | 0.177401495 | 0.859272605 |
| MRPS36 | -0.006498388 | 4.377811014 | -0.177279396 | 0.859368449 |
| KRT8P3 | 0.031079078 | 0.337656679 | 0.177183298 | 0.859443885 |
| TUBB4B | 0.012908864 | 8.776578305 | 0.177067163 | 0.859535052 |
| RN7SL127P | 0.021028575 | 0.707426957 | 0.176863693 | 0.859694781 |
| RP11-60I3.5 | 0.019284093 | 0.202352675 | 0.176790404 | 0.859752316 |
| RPL32P31 | -0.017509049 | -0.174761186 | -0.176644258 | 0.85986705 |
| PLA2G4C | 0.028781904 | -0.513707084 | 0.176636518 | 0.859873126 |
| RP11-380L11.3 | -0.018177805 | 1.01324208 | -0.176443766 | 0.860024453 |
| DAK | -0.007268286 | 4.4073223 | -0.176090547 | 0.860301774 |
| CCDC137 | -0.007548875 | 5.436628402 | -0.175491844 | 0.860771869 |
| ATP5G3 | 0.008526139 | 5.687957833 | 0.175469423 | 0.860789475 |
| CT45A3 | -0.033969888 | -2.244006729 | -0.175267359 | 0.860948145 |
| MYLK3 | 0.021984777 | -2.808247837 | 0.174832928 | 0.8612893 |
| AC074212.5 | 0.018891951 | -2.127594742 | 0.174692389 | 0.86139967 |
| SLC38A2 | 0.009096777 | 7.499221631 | 0.174661505 | 0.861423924 |
| SASH1 | -0.046988997 | -1.13123368 | -0.174569978 | 0.861495805 |
| CCDC64B | 0.025966171 | -3.587403921 | 0.174547577 | 0.861513398 |
| WFS1 | -0.030976866 | -0.005769343 | -0.174215146 | 0.861774484 |
| AK7 | 0.022802798 | -2.007823173 | 0.173887955 | 0.86203147 |
| SYPL1 | 0.009693672 | 7.115292229 | 0.172681978 | 0.862978805 |
| RP11-661A12.12 | 0.019508027 | -0.513424737 | 0.17241048 | 0.863192103 |
| CEP70 | -0.021080489 | 4.005778968 | -0.172153986 | 0.863393623 |
| ENTPD8 | -0.026943023 | -2.161490226 | -0.172145997 | 0.8633999 |
| AC010733.5 | 0.010344544 | 2.970199015 | 0.171587144 | 0.863839005 |
| TRIM6 | 0.027021536 | 1.272245037 | 0.171351663 | 0.864024042 |
| HDAC4 | -0.014206224 | 4.300166258 | -0.171100249 | 0.864221606 |
| CLEC18B | -0.019218809 | -1.348557937 | -0.17027718 | 0.864868444 |
| PFKFB1 | -0.015140668 | -0.920492197 | -0.170169013 | 0.864953458 |
| PARP9 | 0.013059341 | 5.292503453 | 0.170055575 | 0.865042616 |
| FGFR3 | 0.029861373 | 0.607496652 | 0.168715916 | 0.866095668 |
| ALDH1L2 | -0.030237843 | -0.480236639 | -0.168527042 | 0.866244153 |
| AC073343.13 | 0.016052851 | -0.202666941 | 0.168438859 | 0.866313481 |
| AC099522.2 | 0.007261778 | 5.498331272 | 0.168372013 | 0.866366035 |
| EI24 | 0.007672095 | 5.919207464 | 0.168026461 | 0.866637714 |
| RP11-1143G9.4 | 0.03454211 | 1.832457902 | 0.167792067 | 0.866822008 |
| SP1 | -0.005127351 | 7.137474356 | -0.167780723 | 0.866830927 |
| SLC16A2 | 0.041379883 | -1.779970973 | 0.167706807 | 0.866889045 |
| FSTL3 | 0.019967348 | 4.565155671 | 0.167669181 | 0.866918631 |
| POGLUT2P1 | -0.019617523 | -0.725947382 | -0.167529476 | 0.86702848 |
| DES | -0.043282487 | -0.278798327 | -0.167343849 | 0.867174442 |
| KCNQ2 | 0.03108302 | -2.978652104 | 0.167331642 | 0.867184041 |
| ATP5F1P1 | 0.023652947 | 1.817001473 | 0.166922517 | 0.86750576 |
| PRSS21 | 0.063370016 | 2.562758368 | 0.166905161 | 0.867519409 |
| RP11-326I11.5 | -0.014998288 | 0.913243213 | -0.166680169 | 0.867696343 |
| ZNF107 | -0.012022675 | 4.78291577 | -0.166627337 | 0.867737891 |
| TRIM21 | -0.009729168 | 6.291833172 | -0.166392073 | 0.867922912 |
| ZNF83 | -0.009786124 | 5.118374028 | -0.166318027 | 0.867981147 |
| PLA2G7 | 0.03856608 | 0.316828797 | 0.166287907 | 0.868004835 |
| RP11-478C6.4 | -0.008531807 | 7.399928173 | -0.166260368 | 0.868026493 |
| NARFL | -0.010449147 | 3.888368025 | -0.166255817 | 0.868030073 |
| CCDC69 | -0.012446312 | 7.194807007 | -0.166139461 | 0.868121584 |
| PABPC1P1 | -0.011530015 | 5.318910859 | -0.165710918 | 0.868458641 |
| RP6-206I17.1 | -0.013120676 | 2.35308984 | -0.165661292 | 0.868497675 |
| PPME1 | 0.008787866 | 3.976178018 | 0.165405608 | 0.868698787 |
| TREM2 | -0.031629743 | -0.596137562 | -0.165372078 | 0.868725161 |
| CTD-2341M24.1 | -0.018987189 | 2.029004214 | -0.165347738 | 0.868744307 |
| UGGT2 | 0.046489664 | 0.371691468 | 0.165313146 | 0.868771517 |
| WBP5 | -0.033801316 | 3.98540998 | -0.16502077 | 0.869001504 |
| NR6A1 | 0.024783899 | 2.626121971 | 0.164815301 | 0.869163136 |
| EEF1A1P10 | 0.015535319 | 0.389560369 | 0.164349511 | 0.869529569 |
| ATP5F1 | -0.005365923 | 6.643513123 | -0.164152832 | 0.869684304 |
| SERPINB8 | 0.021678417 | 5.336518557 | 0.163813874 | 0.869950986 |
| LSMEM1 | -0.01502871 | 1.525212194 | -0.163784162 | 0.869974363 |
| PBX3 | 0.026330004 | 5.133039311 | 0.163745214 | 0.870005007 |
| HOXA4 | -0.037879332 | 1.530235375 | -0.16334221 | 0.870322101 |
| ECM1 | -0.028387432 | 3.245677214 | -0.163017809 | 0.870577364 |
| PRPF39 | 0.005208918 | 5.712421548 | 0.162881162 | 0.870684892 |
| AC009245.3 | 0.008822086 | 5.977230599 | 0.162713773 | 0.870816614 |
| AC012358.4 | 0.018139142 | 0.116116761 | 0.162491694 | 0.870991378 |
| SLC7A2 | -0.042473855 | -2.166330359 | -0.162164754 | 0.871248674 |
| C3orf67 | 0.023980474 | -2.006706714 | 0.162001491 | 0.871377165 |
| ITPKA | -0.025365652 | 1.663465871 | -0.161745477 | 0.871578658 |
| CAMSAP3 | 0.025618243 | -1.787694021 | 0.161692442 | 0.8716204 |
| BACH1-IT2 | -0.016786233 | 1.22001065 | -0.161478416 | 0.871788855 |
| SELP | 0.029402336 | 2.504492588 | 0.161392906 | 0.871856159 |
| CLPTM1L | -0.005318917 | 5.711128798 | -0.161160274 | 0.872039269 |
| CCNL2 | -0.008356299 | 6.790065197 | -0.161127302 | 0.872065222 |
| REPIN1 | -0.008562712 | 6.886663541 | -0.160976874 | 0.87218363 |
| LA16c-380H5.5 | -0.02469526 | 1.676993119 | -0.160888992 | 0.872252808 |
| CARNS1 | 0.020218245 | 0.943020811 | 0.160716135 | 0.872388876 |
| RP4-751H13.7 | 0.018251019 | -2.469177834 | 0.160621963 | 0.872463008 |
| BTF3P2 | -0.012642271 | 1.640798797 | -0.160602235 | 0.872478538 |
| RP11-644F5.15 | -0.016015646 | -0.345086252 | -0.16029076 | 0.872723738 |
| RP11-248E9.7 | -0.015478695 | 0.062986969 | -0.160215116 | 0.872783288 |
| PER3 | 0.022225952 | 1.274484989 | 0.159498463 | 0.873347507 |
| IGHV4OR15-8 | -0.025675175 | 0.854897384 | -0.159413958 | 0.873414042 |
| HOXB2 | -0.039894725 | 3.985436884 | -0.159339643 | 0.873472554 |
| RP11-353N4.6 | -0.012052166 | 1.715168868 | -0.159241593 | 0.873549756 |
| SSR2 | 0.005016448 | 6.999012777 | 0.159120769 | 0.87364489 |
| ERHP1 | 0.009849274 | 4.587612622 | 0.1590859 | 0.873672346 |
| CACTIN-AS1 | -0.023510233 | -0.296618202 | -0.158882313 | 0.873832652 |
| RP11-245J9.6 | 0.015888254 | 1.721695274 | 0.158687828 | 0.873985795 |
| EMILIN1 | -0.037134939 | 3.717487314 | -0.158614349 | 0.874043656 |
| RP11-342F17.1 | -0.016749193 | -0.274393155 | -0.158453654 | 0.874170198 |
| STEAP1 | 0.024719076 | -2.016946576 | 0.158390737 | 0.874219743 |
| LCN8 | -0.032435142 | -2.628811155 | -0.158386698 | 0.874222924 |
| GPANK1 | 0.00728232 | 4.853688341 | 0.158327142 | 0.874269823 |
| GDPD5 | 0.015591338 | 2.637151575 | 0.15832092 | 0.874274723 |
| AP001055.6 | 0.016988888 | 2.582030983 | 0.157908656 | 0.874599387 |
| ADAT1 | -0.005490602 | 4.672737333 | -0.157414505 | 0.874988566 |
| PPIC | 0.027957648 | -1.436042333 | 0.157372395 | 0.875021732 |
| CTD-2060C23.1 | -0.015001707 | -0.057651285 | -0.157327113 | 0.875057397 |
| SYTL4 | 0.038250016 | 0.579448093 | 0.157272466 | 0.875100438 |
| IMPA1 | -0.006607159 | 5.136455569 | -0.157164499 | 0.875185476 |
| CTD-3064H18.4 | -0.028783726 | -2.130268736 | -0.156896323 | 0.875396704 |
| RP11-61L19.2 | 0.017507186 | 1.048898735 | 0.156869543 | 0.875417799 |
| FZD1 | -0.018585429 | 3.195612751 | -0.156506313 | 0.875703911 |
| ZNRF3 | -0.018731994 | 0.589736531 | -0.155909672 | 0.876173916 |
| BOP1 | 0.00905865 | 2.807176153 | 0.155627415 | 0.87639628 |
| RPL7P47 | 0.011984598 | 2.220210022 | 0.155475634 | 0.876515858 |
| GPAT2 | -0.04091329 | -1.870831408 | -0.155452816 | 0.876533835 |
| RPL23P2 | -0.00969755 | 3.873346649 | -0.155102223 | 0.876810055 |
| RPS14 | -0.01022499 | 8.882785297 | -0.155012928 | 0.876880411 |
| FABP5P2 | 0.019575687 | -0.48976611 | 0.15490553 | 0.87696503 |
| CECR7 | 0.031599539 | -1.848457427 | 0.154787348 | 0.877058148 |
| RP11-723D22.2 | 0.019269133 | 0.160810326 | 0.154628283 | 0.877183481 |
| RP11-343L5.2 | -0.0134914 | 1.840900876 | -0.154474672 | 0.87730452 |
| D2HGDH | 0.0099009 | 5.115053945 | 0.154374717 | 0.877383281 |
| RPL4P1 | 0.016243303 | -0.444390364 | 0.154323387 | 0.877423728 |
| RP11-124N14.3 | 0.025894705 | 2.275335888 | 0.154266138 | 0.877468839 |
| MYRIP | 0.027847039 | -2.723698202 | 0.154233792 | 0.877494328 |
| RP11-20I20.1 | -0.022864396 | -1.691749412 | -0.154199339 | 0.877521477 |
| RP11-791G15.2 | 0.016701879 | 0.559339926 | 0.153938185 | 0.877727269 |
| CWF19L2 | -0.005868743 | 4.444462049 | -0.153582061 | 0.878007914 |
| AGRN | 0.022054423 | 3.11337015 | 0.153177088 | 0.878327072 |
| FRMD4A | -0.034971233 | -0.160676186 | -0.152682337 | 0.87871701 |
| RNF139-DT | 0.011012416 | 1.821097981 | 0.152170172 | 0.879120706 |
| HMBOX1 | 0.006747868 | 5.007319232 | 0.152167744 | 0.87912262 |
| ZNF683 | 0.027532932 | 0.539005597 | 0.152123676 | 0.879157356 |
| H3F3BP1 | -0.008713739 | 4.395318325 | -0.151943489 | 0.87929939 |
| ARHGEF39 | -0.020175652 | 1.144372393 | -0.151776557 | 0.879430979 |
| INHBA-AS1 | 0.021784765 | -2.876254988 | 0.151519708 | 0.879633455 |
| AMPD3 | 0.01491042 | 5.209126982 | 0.151454727 | 0.879684681 |
| CYP27A1 | -0.040140489 | 2.445923857 | -0.151421926 | 0.879710539 |
| RP11-560J1.2 | -0.012262514 | 1.39463623 | -0.150998742 | 0.88004416 |
| FSIP2 | 0.03388749 | -4.716785923 | 0.150722653 | 0.880261828 |
| AC008132.12 | 0.011309093 | 1.818072041 | 0.15031161 | 0.880585911 |
| NANOGP4 | -0.020697714 | -0.635761374 | -0.150199705 | 0.880674146 |
| GBE1 | -0.009564505 | 5.242574074 | -0.150068476 | 0.880777618 |
| CTB-134H23.2 | -0.011050543 | 1.616604226 | -0.149953177 | 0.880868531 |
| CCDC114 | -0.015672333 | 0.281424635 | -0.149359536 | 0.881336643 |
| CECR6 | -0.029412662 | 1.540417564 | -0.1493522 | 0.881342427 |
| DDX41 | 0.005136249 | 6.419498518 | 0.149148229 | 0.881503277 |
| TMCC1 | 0.006655896 | 4.139959211 | 0.148915005 | 0.881687202 |
| RNF31 | 0.004752728 | 4.825832713 | 0.148700382 | 0.881856463 |
| JAZF1 | -0.021673616 | 4.390705905 | -0.147579231 | 0.882740739 |
| CACNB1 | -0.017215994 | 2.158498986 | -0.147395524 | 0.882885646 |
| TXN2 | -0.005531214 | 7.285309231 | -0.147218146 | 0.883025565 |
| WSCD1 | -0.031909823 | -4.053529942 | -0.147161578 | 0.883070188 |
| NAP1L4P3 | 0.009266762 | 5.103339815 | 0.147079255 | 0.883135128 |
| AC074183.4 | 0.01256072 | 4.052813643 | 0.147004934 | 0.883193756 |
| RP11-1151B14.4 | -0.019759795 | -2.286206866 | -0.146960183 | 0.883229058 |
| BTBD7 | 0.004455167 | 4.430753399 | 0.146739206 | 0.88340338 |
| PBLD | 0.010798085 | 1.931741534 | 0.146000907 | 0.883985845 |
| AC000089.3 | 0.007253621 | 4.845341964 | 0.145917546 | 0.884051615 |
| GSTM4 | 0.011345663 | 3.390221618 | 0.145690167 | 0.884231016 |
| SERHL2 | 0.021266317 | -1.454145593 | 0.145583717 | 0.884315006 |
| LRRCC1 | 0.011945862 | 3.948512456 | 0.145075502 | 0.884716011 |
| KIAA1430 | 0.011548552 | 5.016637938 | 0.14499869 | 0.884776622 |
| DIRC3 | -0.019739786 | -1.307431806 | -0.144856826 | 0.884888566 |
| DCAF5 | -0.004948961 | 5.96409578 | -0.144688979 | 0.885021016 |
| RP11-867G2.8 | -0.024599764 | 1.111794444 | -0.144685514 | 0.885023751 |
| RP1-286D6.5 | -0.017127712 | 0.30966784 | -0.144663663 | 0.885040993 |
| TSEN34 | -0.006974847 | 5.539122311 | -0.144232028 | 0.885381618 |
| KLRK1 | 0.019371087 | -1.435825155 | 0.143193386 | 0.886201347 |
| POTEF | 0.016487165 | -2.498305225 | 0.143117227 | 0.88626146 |
| CTB-36O1.7 | -0.021395847 | 1.115745092 | -0.142958079 | 0.886387077 |
| NRBF2P4 | 0.014157649 | 0.840006156 | 0.142926221 | 0.886412223 |
| RPL7AP11 | -0.006853558 | 6.690738152 | -0.14286978 | 0.886456773 |
| PDSS1 | -0.008568694 | 4.343867678 | -0.142054827 | 0.887100075 |
| EEF2 | -0.005975084 | 11.81695604 | -0.14161874 | 0.887444342 |
| CHD9 | -0.005994283 | 5.869473582 | -0.1415657 | 0.887486216 |
| LEPROT | 0.007665185 | 6.666311761 | 0.14156139 | 0.887489618 |
| PBX2 | -0.005829639 | 7.333055069 | -0.141128419 | 0.887831449 |
| KRT8 | -0.030256175 | 1.9140551 | -0.140690335 | 0.888177337 |
| PREX1 | 0.008920845 | 7.643891943 | 0.140502368 | 0.888325752 |
| SPSB4 | -0.028502821 | -2.127419615 | -0.139987638 | 0.888732195 |
| PENK | -0.029081599 | -2.882458512 | -0.139940719 | 0.888769245 |
| RPS15AP12 | 0.007093031 | 6.576287212 | 0.13966313 | 0.888988448 |
| C1QL1 | -0.024434024 | -0.985659957 | -0.139637874 | 0.889008393 |
| SMAD1 | 0.023021965 | 3.187092934 | 0.139520039 | 0.889101447 |
| OGN | -0.017733212 | -0.84058494 | -0.139381017 | 0.889211234 |
| AC109826.1 | -0.018339511 | 0.476773544 | -0.139035146 | 0.889484381 |
| AC098617.1 | -0.022240498 | -3.004005552 | -0.138740991 | 0.889716696 |
| IGHV3-72 | 0.025125311 | 1.246293252 | 0.138434399 | 0.889958844 |
| RNF215 | -0.007648688 | 3.48175724 | -0.138189146 | 0.890152553 |
| RP11-98D18.9 | -0.01677617 | 0.364563345 | -0.137973627 | 0.890322783 |
| NT5DC2 | 0.016065941 | 4.29290284 | 0.137843342 | 0.890425692 |
| PTGIR | -0.022875563 | 2.90728471 | -0.137727112 | 0.890517501 |
| PRDM16 | 0.052556657 | -0.493412492 | 0.136969887 | 0.891115664 |
| VEZT | 0.006952188 | 4.651546473 | 0.136913385 | 0.891160299 |
| MAF | 0.018649498 | 2.807693974 | 0.136594314 | 0.891412367 |
| RAN | -0.006418774 | 7.077702149 | -0.136562227 | 0.891437717 |
| ATF7IP | 0.006456996 | 5.72234945 | 0.136434516 | 0.891538613 |
| RP11-1149M10.2 | 0.017366492 | -1.225328632 | 0.13629421 | 0.89164946 |
| CTA-109P11.4 | 0.021129538 | -1.198702481 | 0.136117703 | 0.891788912 |
| AC137932.5 | -0.016954586 | -0.500806676 | -0.136063266 | 0.891831922 |
| TTYH3 | 0.010547069 | 6.859156663 | 0.135879497 | 0.891977116 |
| RP11-542K23.9 | -0.020909615 | -0.907284973 | -0.135848566 | 0.892001554 |
| RP11-433O3.1 | 0.023447801 | -0.568197136 | 0.135833193 | 0.892013701 |
| PILRB | -0.012543884 | 2.324827752 | -0.135777404 | 0.89205778 |
| C2ORF15 | -0.013921917 | 0.548629082 | -0.135486362 | 0.892287739 |
| RP11-290C10.1 | -0.015308251 | -0.161189393 | -0.135475233 | 0.892296533 |
| VAT1L | -0.02894894 | -2.798261545 | -0.135166408 | 0.892540554 |
| SNORD14E | 0.02067884 | 1.694362108 | 0.134797981 | 0.892831684 |
| DCST2 | -0.013572869 | 0.277408868 | -0.134754541 | 0.89286601 |
| CTD-2377D24.6 | -0.019927432 | -1.899403492 | -0.134626655 | 0.892967069 |
| PROM1 | 0.041683359 | 3.825113693 | 0.134438838 | 0.89311549 |
| MIR4461 | 0.021601041 | 0.872442143 | 0.134357681 | 0.893179625 |
| EIF2A | -0.006001742 | 7.581457523 | -0.133888874 | 0.893550117 |
| RP11-296I10.3 | 0.017196613 | -2.125625437 | 0.133879876 | 0.893557228 |
| PRELID1 | 0.006291601 | 7.315285749 | 0.133584709 | 0.893790507 |
| RP11-192H23.7 | 0.016754978 | -1.755195483 | 0.132520207 | 0.894631889 |
| HMGXB3 | 0.005996229 | 5.782965507 | 0.132000701 | 0.89504255 |
| KRT80 | -0.028057319 | -1.271717101 | -0.131928974 | 0.895099252 |
| MAS1 | 0.020030072 | -1.599770165 | 0.131680684 | 0.895295533 |
| PTAFR | 0.022584121 | 5.670888213 | 0.131367548 | 0.895543085 |
| ANKRD26P3 | 0.028575206 | -2.199576777 | 0.131361479 | 0.895547883 |
| IGHV3OR16-6 | -0.016569085 | -0.871289049 | -0.131268167 | 0.895621654 |
| CCDC42 | 0.018878442 | -0.599010443 | 0.131219591 | 0.895660058 |
| REPS2 | 0.010718573 | 3.167554894 | 0.131186518 | 0.895686205 |
| RP1-20N2.6 | 0.012675067 | -0.201445579 | 0.130935551 | 0.895884622 |
| B3GALNT2 | 0.009086384 | 3.196441082 | 0.130934331 | 0.895885586 |
| TMEM33 | 0.00677527 | 5.1205869 | 0.130169011 | 0.896490695 |
| SEMA3C | 0.031154325 | -0.055384452 | 0.12992004 | 0.89668756 |
| PDK4 | 0.033763001 | 2.103709358 | 0.129682887 | 0.896875086 |
| GPER1 | -0.029791113 | -0.379795491 | -0.129079467 | 0.897352259 |
| AC005034.3 | 0.010332624 | 1.209051876 | 0.129057165 | 0.897369896 |
| CXorf22 | -0.020267985 | -3.043148797 | -0.128812672 | 0.897563248 |
| PLA2G6 | -0.008698914 | 4.451240349 | -0.128748311 | 0.897614147 |
| IMP4 | 0.004505144 | 5.950578195 | 0.12806313 | 0.898156044 |
| RBMX2P2 | 0.012231971 | 1.145938071 | 0.12804094 | 0.898173595 |
| LDHBP2 | -0.012454041 | 0.481742786 | -0.128019232 | 0.898190764 |
| GPHN | -0.02008803 | 2.0959143 | -0.127908126 | 0.89827864 |
| MMAA | 0.007315815 | 1.733509204 | 0.127861773 | 0.898315303 |
| AOX2P | -0.036127705 | 0.778660195 | -0.127640008 | 0.898490707 |
| RP1-305B16.3 | 0.011018522 | 0.427101872 | 0.127595864 | 0.898525623 |
| FAM212B | -0.013496213 | 3.141734529 | -0.127510078 | 0.898593477 |
| LIPE | 0.014265605 | 1.204157874 | 0.127191585 | 0.898845402 |
| SGMS2 | -0.025561882 | 2.817702333 | -0.127165816 | 0.898865785 |
| CACNG4 | -0.031439864 | -2.870856432 | -0.127041638 | 0.898964011 |
| BBS5 | 0.011685523 | 0.304432744 | 0.126995766 | 0.899000297 |
| RP13-638C3.6 | -0.017486579 | -0.925643331 | -0.126907108 | 0.899070428 |
| FAM72C | 0.017686748 | 0.242139329 | 0.126304584 | 0.899547063 |
| DVL2 | 0.005505913 | 4.980206516 | 0.126211498 | 0.899620703 |
| SLC35E3 | -0.00560369 | 3.608596739 | -0.125755425 | 0.899981514 |
| PABPC1P10 | -0.0134456 | 0.193604555 | -0.125691032 | 0.900032458 |
| RP11-44F14.1 | -0.027362368 | 1.158122171 | -0.125666711 | 0.9000517 |
| AC098474.1 | -0.018754324 | 0.463289274 | -0.125392144 | 0.90026893 |
| RPL29P7 | 0.013320118 | -0.10292882 | 0.125296467 | 0.900344629 |
| SEL1L3 | 0.015886832 | 5.162758162 | 0.12509173 | 0.900506618 |
| AC147651.4 | -0.01044165 | 4.097788574 | -0.125086528 | 0.900510733 |
| PLK1S1 | 0.006799093 | 4.602580392 | 0.124924134 | 0.900639223 |
| AP000251.3 | -0.017913059 | -1.437571874 | -0.124671881 | 0.900838817 |
| KIAA1614 | 0.021056556 | -2.270165201 | 0.124646176 | 0.900859156 |
| MTND4P24 | -0.015546219 | 1.96034878 | -0.124557799 | 0.900929085 |
| IGLV4-60 | 0.021414727 | 0.248760907 | 0.124549963 | 0.900935286 |
| ENG | -0.016104224 | 6.186516439 | -0.124527166 | 0.900953324 |
| FAM153B | 0.023447171 | -2.532562495 | 0.124177303 | 0.901230166 |
| DNM1 | -0.020710413 | 3.704662272 | -0.12397513 | 0.901390149 |
| RP11-1D12.2 | 0.03312114 | 3.322473089 | 0.123887768 | 0.901459281 |
| FAM131B | -0.017755289 | 0.081229126 | -0.123843274 | 0.901494491 |
| MSANTD1 | 0.011457525 | -1.662291193 | 0.123657826 | 0.901641244 |
| PPIL3 | -0.005104793 | 5.341332272 | -0.123540414 | 0.90173416 |
| PSMG1 | 0.006889217 | 5.720661552 | 0.123463456 | 0.901795062 |
| ESPNL | -0.019496719 | -2.487675807 | -0.123357016 | 0.901879296 |
| EPS8L1 | 0.017888379 | 0.85286084 | 0.123206335 | 0.901998544 |
| KLHL7-AS1 | -0.014789419 | -0.328203568 | -0.123012378 | 0.902152043 |
| RP11-261P9.4 | -0.010931009 | 3.101945816 | -0.12264846 | 0.902440062 |
| GPSM3 | -0.00693239 | 8.886702149 | -0.122569309 | 0.902502706 |
| BST1 | -0.017594742 | 6.209428704 | -0.122291931 | 0.902722245 |
| AC008697.1 | -0.016556738 | -2.0642299 | -0.122230431 | 0.902770921 |
| APLP2 | -0.008854522 | 9.467971104 | -0.122073703 | 0.902894972 |
| HMGCS1 | 0.007975737 | 5.513178352 | 0.121692126 | 0.903197001 |
| FAM126A | -0.007564637 | 4.259009647 | -0.120439548 | 0.90418855 |
| XXbac-BPG157A10.21 | -0.010851082 | 0.950893497 | -0.120419789 | 0.904204193 |
| RP11-473M20.16 | 0.013363141 | 0.147345717 | 0.120211682 | 0.904368947 |
| ZC3HAV1 | 0.005647262 | 7.0073289 | 0.119467188 | 0.904958382 |
| BLVRB | 0.013672451 | 6.24073014 | 0.119380652 | 0.905026898 |
| DNAH11 | 0.015009116 | -3.281891413 | 0.119377856 | 0.905029111 |
| RP11-87G24.6 | 0.01523312 | -1.846044269 | 0.119323442 | 0.905072195 |
| RPS4XP3 | -0.00898438 | 4.047243045 | -0.119316169 | 0.905077953 |
| C20orf24 | -0.00590306 | 6.170002694 | -0.119280718 | 0.905106023 |
| TCAM1P | -0.023342571 | -2.854254203 | -0.119257614 | 0.905124316 |
| OR2A9P | 0.017204518 | -2.758614164 | 0.118836835 | 0.905457489 |
| C20orf27 | 0.011488676 | 6.177729667 | 0.118784589 | 0.905498858 |
| RP11-415J8.7 | -0.010892853 | 1.655240931 | -0.11864449 | 0.905609793 |
| SVIL | -0.009856629 | 5.253898078 | -0.118304085 | 0.905879344 |
| ATG2B | -0.004302424 | 4.795190266 | -0.118249658 | 0.905922443 |
| CTD-2267D19.6 | -0.015069659 | -0.028054255 | -0.118223796 | 0.905942923 |
| RIOK2 | -0.004674846 | 4.037414866 | -0.118223013 | 0.905943543 |
| KIAA1755 | 0.021095003 | -4.242552324 | 0.118081453 | 0.906055641 |
| HPGDS | -0.033885027 | 2.935076438 | -0.118068255 | 0.906066093 |
| RAC1 | 0.005699648 | 7.369650413 | 0.117717108 | 0.906344169 |
| RP11-568J23.1 | 0.014508954 | -0.273346495 | 0.117559406 | 0.906469059 |
| ASCC2 | 0.002661376 | 6.073734575 | 0.117044114 | 0.90687715 |
| CYP1B1 | 0.034660437 | 1.972182733 | 0.116910829 | 0.906982711 |
| PYCR1 | 0.015298529 | 3.857721394 | 0.116637231 | 0.907199404 |
| AC139426.2 | 0.017656829 | -1.165970397 | 0.116486833 | 0.907318524 |
| C19orf26 | -0.019293259 | -1.4849361 | -0.11634424 | 0.907431465 |
| ARIH2P1 | -0.008829225 | 0.729212415 | -0.115705261 | 0.907937588 |
| ZNF772 | 0.010588065 | 2.299444302 | 0.115578625 | 0.908037899 |
| AC013271.3 | -0.01587912 | -3.463689773 | -0.11534561 | 0.908222478 |
| AFF3 | -0.018039668 | 2.997316682 | -0.115162151 | 0.908367805 |
| MALL | 0.014607769 | -2.981140628 | 0.114633777 | 0.908786375 |
| SHANK1 | 0.026796718 | 1.629641414 | 0.114120902 | 0.909192691 |
| DCXR | -0.005215451 | 5.945516373 | -0.113918993 | 0.909352657 |
| AKNAD1 | -0.016137657 | -3.19233189 | -0.113810139 | 0.9094389 |
| PWAR6 | -0.023038267 | 1.606985352 | -0.113717005 | 0.909512689 |
| RAG2 | -0.020808126 | -3.240769643 | -0.113650876 | 0.909565082 |
| UBASH3A | 0.015793209 | 1.642705515 | 0.113244827 | 0.909886802 |
| MFSD6 | -0.007955599 | 5.264008838 | -0.11316069 | 0.909953466 |
| WRAP73 | 0.00465245 | 4.269437456 | 0.113104234 | 0.909998199 |
| CD300E | -0.035038944 | 4.842573131 | -0.11260236 | 0.910395869 |
| GGT3P | -0.016491391 | -2.534083613 | -0.111881368 | 0.9109672 |
| CTAGE11P | 0.009810739 | -0.270424807 | 0.111815947 | 0.911019044 |
| PLEKHA4 | 0.015015632 | 0.29019599 | 0.111248817 | 0.911468488 |
| SEPHS1 | 0.003898491 | 5.328692108 | 0.110814023 | 0.911813075 |
| RP11-119H12.1 | -0.012443569 | -0.348624384 | -0.110643852 | 0.911947946 |
| IGKV5-2 | 0.017277019 | -0.135632968 | 0.110291157 | 0.912227485 |
| KRT7 | -0.019039424 | -2.447588074 | -0.110270945 | 0.912243505 |
| MMP11 | 0.015255933 | -0.781689493 | 0.110140092 | 0.91234722 |
| ENHO | -0.024641187 | 0.017911231 | -0.110032789 | 0.91243227 |
| LAP3 | 0.006544944 | 6.423240086 | 0.109956939 | 0.91249239 |
| SGCD | 0.01983339 | -4.157386353 | 0.109937531 | 0.912507774 |
| ADRA2C | -0.026667379 | -0.748188603 | -0.109355697 | 0.912968966 |
| DOCK3 | 0.018384473 | -0.378879112 | 0.109057857 | 0.913205061 |
| RABGGTA | 0.003813415 | 5.400067579 | 0.108845847 | 0.913373124 |
| RP13-131K19.1 | -0.010636025 | 0.12231006 | -0.108618016 | 0.913553733 |
| AC004692.5 | -0.011752388 | -0.043163075 | -0.108549278 | 0.913608225 |
| EIF3G | -0.004222712 | 7.861510994 | -0.108449539 | 0.913687293 |
| RGMA | 0.022041078 | -1.586922325 | 0.108125897 | 0.913943868 |
| UBE2E2 | 0.013362407 | 4.528969763 | 0.10789962 | 0.914123259 |
| HOPX | 0.021805461 | 3.208003835 | 0.107662537 | 0.914311222 |
| HECTD4 | -0.003981234 | 5.734666668 | -0.107084313 | 0.914769666 |
| AC005076.5 | 0.007838538 | 1.820382973 | 0.107063984 | 0.914785784 |
| PIGZ | -0.010507433 | 2.775055607 | -0.106863571 | 0.914944689 |
| PGAM5 | 0.006193773 | 4.685601622 | 0.106158721 | 0.91550358 |
| SHMT1 | 0.008214772 | 3.407468011 | 0.106143588 | 0.915515579 |
| TTLL5 | -0.005505992 | 4.139396821 | -0.105640249 | 0.915914715 |
| SLCO4C1 | 0.016843391 | 3.428251211 | 0.105531968 | 0.916000582 |
| GAS7 | -0.011476617 | 6.610527283 | -0.10518497 | 0.916275757 |
| APIP | 0.004350734 | 3.683964902 | 0.104475661 | 0.916838285 |
| RP11-401P9.5 | -0.013375837 | -2.650798904 | -0.104317234 | 0.916963933 |
| KRT17P2 | 0.023954172 | -0.698333922 | 0.103740437 | 0.917421408 |
| FAM171A2 | -0.015133229 | 0.860270274 | -0.103596984 | 0.917535189 |
| MYRFL | 0.014712115 | -2.80522046 | 0.103542469 | 0.917578428 |
| AC026740.1 | -0.009436981 | 1.906052999 | -0.103211428 | 0.917841005 |
| CDKAL1 | -0.003773496 | 4.410233926 | -0.102972941 | 0.918030175 |
| SETD7 | -0.011395252 | 3.900490011 | -0.102405475 | 0.918480312 |
| RP11-486G15.2 | 0.014880452 | 1.934312866 | 0.10233653 | 0.918535003 |
| RP11-165J3.6 | 0.00720376 | 2.402319893 | 0.102290388 | 0.918571606 |
| DNAI2 | -0.012065504 | -1.800744575 | -0.102010255 | 0.918793831 |
| LA16c-3G11.7 | -0.011809876 | -1.229839339 | -0.101812693 | 0.918950558 |
| LINC00639 | 0.019929334 | -2.241120801 | 0.101165924 | 0.919463664 |
| CEP85 | -0.004038009 | 5.705718526 | -0.100074987 | 0.920329221 |
| ZNF671 | 0.005817724 | 4.351444406 | 0.099898725 | 0.920469077 |
| SRD5A1 | -0.008892423 | 3.669293186 | -0.099606419 | 0.920701016 |
| ALOX12B | -0.01361673 | -1.623911398 | -0.099354021 | 0.920901293 |
| ZNF266 | -0.005436265 | 5.69057622 | -0.099229081 | 0.921000434 |
| IL7 | -0.018150524 | -0.13640457 | -0.099093737 | 0.921107833 |
| EMP3 | -0.007173034 | 8.796473586 | -0.098911001 | 0.921252841 |
| SCARA3 | -0.026640924 | -1.145265079 | -0.098683745 | 0.92143318 |
| INPP1 | -0.006195267 | 4.396932741 | -0.098618763 | 0.921484747 |
| LARP7 | 0.002635591 | 6.034973561 | 0.09851235 | 0.921569194 |
| TMEM254-AS1 | -0.011673444 | 0.504122604 | -0.098404395 | 0.921654864 |
| TRBV2 | -0.014487308 | 1.273741104 | -0.097900514 | 0.922054745 |
| ACOX2 | -0.019207083 | -1.05848375 | -0.09785802 | 0.922088469 |
| ERI3 | -0.004330441 | 5.243104133 | -0.097732492 | 0.922188093 |
| RP11-22P6.2 | 0.009194254 | 0.651708736 | 0.097546382 | 0.922335798 |
| RN7SKP292 | -0.011203061 | 0.040664798 | -0.097346687 | 0.922494287 |
| RGP1 | -0.003675365 | 5.431934864 | -0.097223163 | 0.922592324 |
| SRCIN1 | -0.01398404 | -2.733931463 | -0.096959547 | 0.922801552 |
| PDCD1LG2 | 0.014076932 | -0.990140889 | 0.0965665 | 0.923113518 |
| RP5-821D11.7 | 0.011247774 | 1.060468949 | 0.096515764 | 0.923153788 |
| PRKCZ | -0.01189778 | 1.789459248 | -0.096417528 | 0.923231761 |
| ANKRD10 | -0.004656569 | 7.011099324 | -0.096305136 | 0.923320971 |
| NRBP2 | -0.011765491 | 3.443017394 | -0.096050529 | 0.923523066 |
| OAZ2 | 0.005094838 | 5.646854732 | 0.095910832 | 0.923633954 |
| LINC00346 | 0.01521419 | -1.264568594 | 0.095878969 | 0.923659246 |
| TRUB1 | -0.004676403 | 4.371996164 | -0.095608712 | 0.923873773 |
| MIS18A | -0.006286432 | 4.491506263 | -0.09559825 | 0.923882078 |
| RIMKLB | -0.010377977 | 4.740594723 | -0.095532251 | 0.923934467 |
| GTF2H2B | 0.009268109 | 2.251842408 | 0.095475578 | 0.923979455 |
| PPP1R13B | -0.010691702 | 0.601347796 | -0.094930857 | 0.924411871 |
| RP11-87C12.2 | -0.010238278 | -1.283981091 | -0.094611699 | 0.924665238 |
| CTC-329H14.1 | -0.010690977 | -0.049589191 | -0.094151108 | 0.925030897 |
| GCFC2 | -0.006009781 | 3.96504301 | -0.093594563 | 0.925472754 |
| KIF9-AS1 | -0.005308846 | 1.52965635 | -0.093367766 | 0.925652822 |
| RRP7B | 0.005936423 | 3.498697187 | 0.093214075 | 0.925774848 |
| NAP1L1P1 | 0.005076388 | 5.678322726 | 0.092422772 | 0.926403149 |
| IGHV6-1 | -0.017490254 | 1.377770683 | -0.092401902 | 0.926419721 |
| WFDC1 | -0.017889952 | 0.381142774 | -0.092195404 | 0.926583689 |
| MIR1285-1 | -0.008478595 | 0.981395142 | -0.092022865 | 0.926720696 |
| AC104843.3 | -0.007908575 | 0.895251628 | -0.091765144 | 0.926925345 |
| MRC2 | 0.018428228 | 3.73664489 | 0.090786825 | 0.927702249 |
| SNORA47 | -0.011018424 | 0.04170472 | -0.09029596 | 0.928092081 |
| ADTRP | 0.013951122 | -0.000647007 | 0.090013031 | 0.928316783 |
| RP13-39P12.2 | 0.006359197 | 2.647918832 | 0.089606786 | 0.928639434 |
| AC062017.1 | -0.006343947 | 1.773207197 | -0.089605768 | 0.928640242 |
| COCH | -0.015646332 | 3.122646711 | -0.089455002 | 0.928759988 |
| RP1-90J20.11 | 0.007676498 | 2.251131237 | 0.089310454 | 0.928874796 |
| SNRPB2 | -0.002258779 | 6.835932767 | -0.089132837 | 0.929015871 |
| AC006953.1 | -0.012443655 | 1.879972724 | -0.089115896 | 0.929029327 |
| CHSY3 | -0.013952583 | -2.46320658 | -0.089091505 | 0.9290487 |
| AFF2 | -0.017803474 | 3.362910552 | -0.088628432 | 0.929416516 |
| MTND1P11 | -0.011909922 | 1.416159411 | -0.088603244 | 0.929436523 |
| CD3E | 0.013144213 | 5.117871557 | 0.0882458 | 0.92972045 |
| ACTG1P9 | 0.009697399 | 1.202739587 | 0.087997297 | 0.929917847 |
| RBM15B | -0.003459725 | 6.388720443 | -0.087930637 | 0.929970799 |
| SNX11 | 0.004772705 | 4.994455867 | 0.08781408 | 0.930063387 |
| PCDHGB1 | 0.015645233 | -3.011961737 | 0.087363823 | 0.930421064 |
| HSPA1B | 0.012393151 | 5.549246113 | 0.087347037 | 0.930434399 |
| ACY3 | -0.021276209 | 3.801341989 | -0.086768157 | 0.930894273 |
| SNRPD2P1 | -0.009246721 | -0.193781263 | -0.086562988 | 0.931057269 |
| PCDH9 | -0.017518658 | -1.152073357 | -0.086075253 | 0.93144476 |
| KLF6 | -0.006955541 | 9.738981446 | -0.086016936 | 0.931491092 |
| EEPD1 | -0.006478125 | 4.202245417 | -0.085897444 | 0.931586028 |
| SEPT3 | 0.013395804 | -2.442279193 | 0.08554736 | 0.931864174 |
| TCEA1P2 | 0.003921101 | 8.098424926 | 0.08431734 | 0.932841505 |
| RAB4A | 0.003792318 | 5.642737297 | 0.084284284 | 0.932867772 |
| RAP1B | -0.004076342 | 6.29628784 | -0.083543242 | 0.933456629 |
| LRRC3 | -0.007090196 | 2.156471527 | -0.083326173 | 0.933629126 |
| SCARA5 | 0.014218603 | -3.637650097 | 0.083274443 | 0.933670235 |
| ZNF730 | -0.011978301 | 0.636549499 | -0.083262786 | 0.933679498 |
| SLC6A16 | 0.006915204 | 1.727565841 | 0.082928792 | 0.933944919 |
| ENSAP2 | 0.003772702 | 7.538650281 | 0.082834682 | 0.934019709 |
| SNRPGP9 | 0.008680433 | 0.116554909 | 0.082525961 | 0.934265055 |
| RP11-61A14.3 | -0.006799534 | 1.764307604 | -0.081902174 | 0.934760807 |
| SLC4A5 | 0.00863072 | 0.396146605 | 0.081880075 | 0.934778371 |
| PRMT5-AS1 | -0.010546017 | -2.503572355 | -0.081543098 | 0.935046193 |
| DBN1 | 0.010256412 | 4.554422847 | 0.080524013 | 0.935856185 |
| ITGAV | 0.009583676 | 3.625693402 | 0.079931486 | 0.93632717 |
| ZNF57 | 0.007553257 | 2.976697235 | 0.079759096 | 0.936464203 |
| ABCC13 | 0.017371482 | -3.922254669 | 0.079547789 | 0.936632173 |
| LPIN2 | 0.004310068 | 5.505954193 | 0.079474718 | 0.936690258 |
| ACRBP | 0.007183202 | 4.856094269 | 0.079440778 | 0.936717238 |
| RP5-1159O4.2 | 0.008002082 | 0.768835231 | 0.079183987 | 0.93692137 |
| ZBTB20-AS1 | -0.009142327 | -2.262850114 | -0.078797422 | 0.937228671 |
| YIF1B | 0.00366034 | 5.698981502 | 0.078764034 | 0.937255214 |
| CT45A2 | 0.013926522 | -2.349814309 | 0.078707542 | 0.937300123 |
| TBC1D32 | 0.005478218 | 1.874743148 | 0.078432896 | 0.937518461 |
| SEPT7P3 | -0.005652296 | 2.337374819 | -0.078201728 | 0.937702238 |
| PRKCA | -0.012141889 | 3.56602211 | -0.078023523 | 0.937843913 |
| PPP1R14C | -0.01473958 | -1.052687184 | -0.077122909 | 0.938559939 |
| NSRP1 | -0.002971749 | 5.268280087 | -0.076188811 | 0.939302639 |
| CTAGE10P | 0.006762762 | -0.778460402 | 0.075932864 | 0.939506151 |
| XRCC6P5 | 0.007867341 | -1.35755339 | 0.075865015 | 0.939560101 |
| GPLD1 | 0.008901829 | 0.069652246 | 0.075798695 | 0.939612834 |
| LOXHD1 | 0.020002541 | 0.544415676 | 0.075754287 | 0.939648146 |
| POP4 | 0.003120588 | 3.953426151 | 0.075674745 | 0.939711394 |
| AC019097.7 | 0.003458363 | 5.131132959 | 0.075658922 | 0.939723975 |
| RMRP | -0.006957592 | 2.649844797 | -0.075642251 | 0.939737232 |
| RP11-611O2.5 | -0.010187658 | -0.64233179 | -0.075632781 | 0.939744762 |
| FTLP5 | -0.009344752 | -0.514409059 | -0.07562247 | 0.939752961 |
| LPL | -0.01922624 | 0.817737785 | -0.075399688 | 0.93993011 |
| PITPNA | -0.003425453 | 6.398582038 | -0.075288971 | 0.94001815 |
| IGHV3-41 | -0.008766819 | -0.936399231 | -0.075260819 | 0.940040535 |
| MT1X | 0.010246529 | 1.642378705 | 0.075089739 | 0.940176576 |
| S100A6 | 0.007258548 | 9.458065429 | 0.074743496 | 0.94045191 |
| RP11-1299A16.3 | -0.011368233 | -2.835497552 | -0.074641521 | 0.940533002 |
| MTRNR2L6 | 0.008649595 | -1.193112563 | 0.074621919 | 0.94054859 |
| C1QTNF2 | 0.011325665 | -2.61617709 | 0.074616114 | 0.940553206 |
| PHC1 | 0.006056519 | 3.723677275 | 0.074580001 | 0.940581924 |
| RP3-525N10.2 | -0.018899371 | -1.406468346 | -0.074194132 | 0.94088878 |
| AC118278.1 | -0.010482566 | 1.052896317 | -0.074121864 | 0.940946251 |
| GPR144 | 0.011145359 | -2.787497249 | 0.074072038 | 0.940985875 |
| RPL5P24 | -0.007659028 | 3.185461675 | -0.073907571 | 0.94111667 |
| TUBB6 | 0.011333431 | 5.691622361 | 0.073649148 | 0.941322186 |
| MCOLN3 | -0.011740692 | -3.370224761 | -0.073640458 | 0.941329097 |
| P2RY2 | -0.011676153 | 4.372922289 | -0.072920936 | 0.941901332 |
| SIGLEC14 | 0.015684148 | 4.458051647 | 0.072912189 | 0.941908289 |
| IGJ | -0.014299442 | 6.324166986 | -0.072648217 | 0.942118234 |
| SLC26A6 | 0.005895127 | 4.336450333 | 0.072348993 | 0.94235622 |
| KCNIP3 | -0.013961418 | -2.763612482 | -0.07214637 | 0.942517378 |
| PPM1G | 0.002751249 | 7.355718703 | 0.071204345 | 0.943266658 |
| HIRIP3 | 0.005264333 | 4.69355503 | 0.071169225 | 0.943294593 |
| GSN | -0.006560518 | 6.758230153 | -0.07104497 | 0.943393428 |
| ARHGEF4 | -0.010739664 | -3.523600116 | -0.070465603 | 0.943854283 |
| SLC9B1 | -0.007212691 | 0.182283617 | -0.070465155 | 0.943854639 |
| DAGLA | -0.012347165 | 1.026741573 | -0.070464222 | 0.943855381 |
| KIAA0087 | -0.02056551 | -2.331176125 | -0.070336653 | 0.943956858 |
| PIGR | 0.011824857 | -3.049049837 | 0.069748263 | 0.944424913 |
| MTMR11 | 0.016977223 | 1.801541929 | 0.069474366 | 0.944642801 |
| CALML6 | 0.007573217 | -1.052872834 | 0.069175533 | 0.944880529 |
| RN7SL449P | 0.010738295 | 0.114766171 | 0.06885078 | 0.945138884 |
| RP11-17A4.1 | -0.004249422 | 3.818160327 | -0.068196782 | 0.945659184 |
| RPL10P15 | 0.005497058 | 1.460068204 | 0.068098562 | 0.945737328 |
| ANAPC10P1 | 0.005066303 | 1.678642253 | 0.067972826 | 0.945837362 |
| MYCN | 0.01957513 | 3.902565741 | 0.06787572 | 0.94591462 |
| RP3-467L1.6 | -0.00567918 | 2.767231169 | -0.067855231 | 0.945930921 |
| C1orf192 | -0.011123484 | -0.065931416 | -0.067842067 | 0.945941395 |
| RP11-472B18.1 | 0.004029047 | 1.212625256 | 0.067784086 | 0.945987524 |
| RP11-629G13.1 | 0.01146253 | -2.196223173 | 0.067615618 | 0.94612156 |
| PDLIM1P1 | 0.009590678 | -1.218266599 | 0.067612385 | 0.946124132 |
| DCTN5 | 0.002094614 | 5.905572604 | 0.067447197 | 0.94625556 |
| MPST | -0.00442721 | 4.899277682 | -0.066660347 | 0.946881615 |
| TRBV5-1 | -0.010101101 | 1.666311306 | -0.066546415 | 0.946972268 |
| ITGA9 | -0.012698541 | 4.657147965 | -0.06645878 | 0.947041997 |
| RP11-29B2.5 | 0.00698546 | 1.041358048 | 0.066147297 | 0.94728984 |
| CPT1C | 0.010336497 | -3.356506579 | 0.065709722 | 0.947638022 |
| CYP4F35P | -0.01105364 | -0.559544419 | -0.065691835 | 0.947652255 |
| MICE | 0.007694504 | 0.59596609 | 0.065422895 | 0.947866258 |
| MORN2 | -0.00459925 | 3.408392656 | -0.064915781 | 0.948269793 |
| SNPH | -0.008078649 | 0.796621092 | -0.064692038 | 0.94844784 |
| PLEC | -0.00619925 | 7.74191053 | -0.064251641 | 0.9487983 |
| YJEFN3 | 0.005068397 | 2.35140833 | 0.064211212 | 0.948830474 |
| ZNF99 | -0.009408606 | -3.353375718 | -0.063849397 | 0.949118408 |
| NINJ2 | 0.005358565 | 4.457537569 | 0.063847788 | 0.949119688 |
| PTGES3L | 0.008141547 | 0.485518028 | 0.063750925 | 0.949196774 |
| PLBD2 | 0.004585039 | 5.428081646 | 0.063705672 | 0.949232787 |
| LINC00926 | -0.009297234 | 4.550705213 | -0.06351446 | 0.949384959 |
| PAK4 | -0.003571031 | 3.216837807 | -0.063399129 | 0.949476743 |
| ADAMTS14 | -0.013257053 | 1.230696369 | -0.063272008 | 0.949577912 |
| CCDC74A | 0.014199043 | -1.534392171 | 0.063177413 | 0.949653195 |
| EFCAB13 | -0.006241616 | 1.596824092 | -0.06304784 | 0.949756316 |
| PYY2 | 0.008029022 | -0.160842286 | 0.062967981 | 0.949819872 |
| ANKRD52 | -0.002356517 | 5.559982717 | -0.062206178 | 0.950426176 |
| RP11-309L24.4 | 0.007366447 | -0.316490915 | 0.062152271 | 0.95046908 |
| SHROOM2 | -0.013020682 | -4.158794969 | -0.062142037 | 0.950477226 |
| AC104978.1 | -0.006668604 | 0.216641883 | -0.062133856 | 0.950483737 |
| CLPTM1 | -0.001847243 | 5.97293674 | -0.061716331 | 0.95081605 |
| CSPG4P13 | -0.013046257 | -3.582862019 | -0.060608554 | 0.951697784 |
| DMKN | 0.010419513 | -3.254733881 | 0.060605517 | 0.951700201 |
| CMYA5 | 0.00616273 | -1.77208085 | 0.060573699 | 0.951725528 |
| CCDC169 | -0.008423443 | 0.435310513 | -0.060295133 | 0.951947262 |
| DEPDC7 | 0.00976597 | 1.75125698 | 0.060287104 | 0.951953653 |
| LA16c-321D4.2 | -0.010308291 | 2.878068103 | -0.060220566 | 0.952006617 |
| TNFRSF4 | -0.011221243 | 3.270606667 | -0.060065552 | 0.952130008 |
| RP11-127B20.2 | 0.005242098 | 1.759278057 | 0.060000301 | 0.952181948 |
| RP11-284F21.10 | -0.011324423 | -1.151366824 | -0.059793374 | 0.952346664 |
| VSIG2 | -0.007911977 | -1.999630286 | -0.059783598 | 0.952354445 |
| ATAD1 | -0.002252189 | 5.250128023 | -0.059721868 | 0.952403584 |
| TGFBI | 0.015981865 | 2.84668938 | 0.0597159 | 0.952408334 |
| RP11-678G14.2 | 0.00963854 | 1.114179326 | 0.059659533 | 0.952453204 |
| AASS | -0.013902915 | -0.12105804 | -0.059534398 | 0.952552814 |
| OTUD6B | -0.002076781 | 4.75270064 | -0.058912413 | 0.953047941 |
| SET | -0.002721821 | 8.753238992 | -0.058613003 | 0.953286291 |
| CCDC28B | 0.004632415 | 3.183986943 | 0.058085293 | 0.953706392 |
| MAP3K7CL | -0.008267815 | 2.300532183 | -0.057801718 | 0.953932147 |
| RUNDC1 | 0.002732289 | 4.44725278 | 0.057798833 | 0.953934443 |
| MRPS11 | -0.001883468 | 4.376378427 | -0.057556498 | 0.954127369 |
| C19orf81 | -0.010253449 | 0.890792743 | -0.057314034 | 0.954320401 |
| KPNA4 | -0.001743917 | 6.207235881 | -0.056667282 | 0.954835311 |
| EGF | 0.011410516 | -1.027836348 | 0.056656207 | 0.954844127 |
| MED28 | -0.002248048 | 5.505978346 | -0.056250855 | 0.955166857 |
| PRPF31 | 0.00161287 | 6.632368293 | 0.056115867 | 0.955274332 |
| ENO1-AS1 | -0.005132539 | 0.574598828 | -0.056067825 | 0.955312583 |
| HOXB-AS1 | 0.010597151 | 2.358452911 | 0.056065289 | 0.955314601 |
| DPF2 | 0.001872552 | 5.335130285 | 0.055953993 | 0.955403214 |
| AC100830.3 | -0.006538752 | 0.585604138 | -0.055875485 | 0.955465722 |
| AC012360.4 | -0.006205444 | 0.139096278 | -0.055087553 | 0.956093084 |
| S100A13 | -0.004167862 | 2.225331763 | -0.054913836 | 0.956231404 |
| FERMT1 | 0.014273564 | -1.353220176 | 0.054812686 | 0.956311944 |
| CLDN10 | 0.015650204 | -0.53326007 | 0.054352623 | 0.95667827 |
| CTSG | 0.01439648 | 8.774620455 | 0.05412798 | 0.956857147 |
| AC011933.2 | -0.004669045 | 4.8482951 | -0.054013535 | 0.956948276 |
| SAYSD1 | 0.002109309 | 4.483438605 | 0.05398069 | 0.95697443 |
| CBWD3 | 0.002516875 | 2.421242896 | 0.053422137 | 0.957419201 |
| DIXDC1 | 0.007634099 | 1.10590636 | 0.053298826 | 0.957517394 |
| RPL29P33 | -0.004845318 | 0.995830435 | -0.053029893 | 0.957731549 |
| LPAR5 | 0.007331806 | 2.096188682 | 0.052779808 | 0.957930698 |
| ST13P10 | 0.005683617 | 0.29970086 | 0.052701963 | 0.957992689 |
| TMEM45B | 0.010139207 | -0.960041149 | 0.052153853 | 0.958429173 |
| RP11-206L10.2 | 0.003494189 | 2.626556364 | 0.05189231 | 0.958637456 |
| DCAF4L1 | -0.006024767 | -0.769377561 | -0.051877077 | 0.958649587 |
| KL | 0.009075745 | -1.530627235 | 0.051186752 | 0.95919935 |
| FBXO9 | -0.002922743 | 5.323272138 | -0.050820562 | 0.959490985 |
| KIAA0907 | -0.002982873 | 5.467508125 | -0.050748928 | 0.959548035 |
| SRRM2-AS1 | 0.004325918 | 0.309657292 | 0.050654561 | 0.959623191 |
| GKAP1 | -0.003890733 | 3.337184045 | -0.050587717 | 0.959676427 |
| TRIM41 | 0.00162442 | 4.533791584 | 0.05012565 | 0.960044431 |
| BVES | 0.011558039 | -3.177464406 | 0.050120463 | 0.960048562 |
| DPYSL3 | -0.012954772 | 2.354283517 | -0.049772816 | 0.960325444 |
| RP11-244J10.1 | 0.00418 | 4.969704954 | 0.049636939 | 0.960433664 |
| LINC00674 | -0.003691059 | 4.486132787 | -0.049550814 | 0.960502259 |
| PLA2G2C | -0.009383865 | -2.320093291 | -0.049375093 | 0.960642215 |
| MKL2 | -0.003069427 | 2.5317584 | -0.048926542 | 0.960999476 |
| AC011513.4 | -0.009803293 | 0.257935678 | -0.048364365 | 0.961447249 |
| FAM69A | 0.006662367 | 0.731847917 | 0.048357544 | 0.961452682 |
| GIPC3 | 0.008816265 | 2.604208853 | 0.048249099 | 0.96153906 |
| ANO7 | 0.011284103 | 1.130173891 | 0.048196 | 0.961581353 |
| NPM1P26 | -0.004601856 | 0.948768012 | -0.048164148 | 0.961606724 |
| FRMD4B | 0.004553974 | 3.998165391 | 0.047661885 | 0.96200679 |
| GAS2L1 | 0.006191991 | 3.362175909 | 0.047411862 | 0.962205943 |
| RP11-537H15.4 | -0.004780943 | 1.145360897 | -0.047315013 | 0.962283088 |
| RP4-605O3.4 | -0.003377885 | 1.421214349 | -0.047155734 | 0.962409962 |
| WLS | 0.011182976 | 0.089448964 | 0.047073526 | 0.962475445 |
| DPY19L2P1 | -0.008617589 | -2.306927103 | -0.046977784 | 0.96255171 |
| RPL13P6 | 0.00473954 | -0.104072596 | 0.046123905 | 0.96323189 |
| LINC00888 | -0.003038805 | 3.625017855 | -0.045963141 | 0.963359954 |
| FLYWCH1 | -0.002249712 | 4.755293582 | -0.04481754 | 0.964272562 |
| YBX3 | -0.004151979 | 6.73230681 | -0.044284869 | 0.964696915 |
| RP4-701O16.5 | -0.004193807 | 0.370377802 | -0.043981076 | 0.964938936 |
| NFYB | -0.001775212 | 4.664727106 | -0.043880613 | 0.965018972 |
| RP11-313F23.5 | 0.006878642 | -0.981955754 | 0.042788571 | 0.965888994 |
| EPHB1 | -0.006508656 | -0.990373754 | -0.042666304 | 0.965986405 |
| POU4F1 | -0.01321393 | -0.859983876 | -0.042532071 | 0.966093352 |
| RP11-1399P15.1 | 0.006096665 | -0.458974444 | 0.042431416 | 0.966173545 |
| CALM1 | -0.002355828 | 7.726296515 | -0.042405301 | 0.966194352 |
| HHEX | 0.003012064 | 6.432590373 | 0.042010037 | 0.966509271 |
| PPP1R12C | 0.001384274 | 5.858796159 | 0.041741502 | 0.966723225 |
| CCDC34 | -0.00430479 | 2.523553275 | -0.041673743 | 0.966777211 |
| RP11-54O7.17 | -0.006174553 | -1.800655103 | -0.041447492 | 0.966957478 |
| VASH2 | -0.007407664 | -1.463505735 | -0.041416765 | 0.966981959 |
| CA15P1 | -0.005336363 | 0.192027188 | -0.041411065 | 0.966986501 |
| PABPC1P4 | 0.002319423 | 6.866695458 | 0.041323012 | 0.967056658 |
| TRAFD1 | -0.002238501 | 5.795687539 | -0.04129623 | 0.967077997 |
| RP11-805F19.4 | 0.003912454 | -0.378076054 | 0.041071461 | 0.967257085 |
| ANO2 | 0.007413915 | -3.329843996 | 0.040975249 | 0.967333744 |
| AL589743.1 | 0.012175935 | -4.234431294 | 0.040937283 | 0.967363994 |
| CCNJL | 0.008314188 | 0.031142114 | 0.040866426 | 0.967420451 |
| FRMD3 | -0.006276231 | 2.76685865 | -0.040628159 | 0.967610298 |
| CD3D | 0.006727592 | 5.017685314 | 0.040507763 | 0.967706227 |
| AC073869.2 | 0.002839502 | 2.025859525 | 0.040506959 | 0.967706868 |
| ZNF625 | 0.005086887 | -0.641471452 | 0.040397129 | 0.96779438 |
| ABHD11 | 0.002801824 | 3.631377079 | 0.040055509 | 0.968066581 |
| UBE3AP2 | 0.003586401 | -0.131172042 | 0.040044977 | 0.968074973 |
| PCDHGA7 | -0.006457796 | -1.520812547 | -0.039790201 | 0.96827798 |
| TBL1Y | 0.007272813 | -2.133798581 | 0.039373013 | 0.968610402 |
| DDB2 | 0.002514389 | 6.557036588 | 0.039370652 | 0.968612284 |
| LCN12 | 0.003959811 | -0.523758461 | 0.039288695 | 0.968677589 |
| ASNS | 0.00351179 | 4.109813078 | 0.039075944 | 0.968847115 |
| RPL7P23 | 0.002006893 | 5.724136419 | 0.039044474 | 0.968872192 |
| TRMT61A | -0.001684194 | 4.393789078 | -0.038865925 | 0.969014466 |
| HSPA12B | -0.005439159 | -2.975435813 | -0.03875383 | 0.969103788 |
| SH3PXD2A | -0.004386822 | 3.848070392 | -0.038251362 | 0.96950418 |
| RP11-118F19.1 | 0.007141783 | -1.439216437 | 0.038116679 | 0.969611503 |
| EEF1DP3 | 0.005399457 | 0.027668186 | 0.03796945 | 0.969728825 |
| OR3A2 | -0.005317485 | -2.103277289 | -0.037797479 | 0.969865864 |
| AC097721.2 | -0.003117555 | 0.867549947 | -0.037301759 | 0.970260893 |
| EME2 | 0.001915592 | 4.20871045 | 0.037150081 | 0.970381763 |
| LETMD1 | 0.001617452 | 5.839179523 | 0.036904715 | 0.970577294 |
| RP11-181G12.2 | 0.003296463 | 0.830483398 | 0.036710258 | 0.970732257 |
| LRP2 | -0.007042666 | -4.80843189 | -0.036588396 | 0.97082937 |
| NBPF10 | -0.002139424 | 5.566522929 | -0.036531139 | 0.970874998 |
| SLC2A1-DT | 0.004630115 | -1.495247702 | 0.036126235 | 0.971197673 |
| TCTE3 | -0.003144464 | 1.034449114 | -0.035817 | 0.97144411 |
| PADI2 | -0.00726227 | 4.102682002 | -0.035641978 | 0.971583591 |
| CHMP3 | 0.001406423 | 5.452267935 | 0.035578642 | 0.971634066 |
| NEDD8 | 0.001316241 | 4.896930717 | 0.035559005 | 0.971649715 |
| RP11-120M18.2 | -0.00450294 | -0.792018076 | -0.035436391 | 0.971747431 |
| BMS1P15 | -0.003994495 | -0.859830177 | -0.035345624 | 0.971819767 |
| DGUOK-AS1 | -0.004501593 | 1.105962588 | -0.034636228 | 0.972385123 |
| SNX19P3 | -0.003907097 | -1.767555613 | -0.034149952 | 0.97277267 |
| LINC00944 | 0.005103441 | -0.838990746 | 0.034008099 | 0.972885725 |
| TAGLN | 0.003457753 | 3.416608384 | 0.03316689 | 0.973556161 |
| RP11-779O18.3 | -0.004074924 | 0.153534746 | -0.03284958 | 0.973809059 |
| RPL26L1 | -0.00134387 | 4.566915016 | -0.032243174 | 0.974292377 |
| RP11-424C20.2 | -0.004678455 | 5.31886146 | -0.032206321 | 0.97432175 |
| RNF144A | 0.00269228 | 5.268097522 | 0.032180994 | 0.974341936 |
| RAB1B | -0.001302724 | 8.702937401 | -0.032017695 | 0.974472091 |
| RP11-73M18.9 | -0.00284179 | 1.898588415 | -0.0318662 | 0.974592837 |
| EDARADD | -0.004247267 | -0.732601574 | -0.031788281 | 0.974654942 |
| PNPLA2 | 0.001479886 | 7.774155094 | 0.031109776 | 0.975195743 |
| CTD-2561B21.11 | -0.003670755 | 0.417930841 | -0.030994472 | 0.975287647 |
| ENTHD2 | -0.001216034 | 5.537752242 | -0.03093777 | 0.975332841 |
| PKD2L1 | -0.003936988 | -2.300922649 | -0.030883735 | 0.975375911 |
| ATHL1 | 0.002877273 | 6.193255371 | 0.030858082 | 0.975396358 |
| RUFY4 | 0.005924055 | -0.107789716 | 0.030715682 | 0.97550986 |
| TTF2 | -0.00162927 | 4.241646851 | -0.030415972 | 0.97574875 |
| RP11-296O14.3 | -0.001850942 | 2.002400623 | -0.030347144 | 0.975803611 |
| ZNF79 | 0.001423447 | 3.77222465 | 0.029923745 | 0.976141093 |
| ORMDL3 | 0.001783194 | 5.746103343 | 0.029820119 | 0.976223692 |
| MACC1 | -0.006369909 | 1.300106919 | -0.029349377 | 0.976598918 |
| AC072062.1 | 0.004168564 | -0.231115085 | 0.029209297 | 0.976710575 |
| ZNF620 | 0.00190187 | 2.31323239 | 0.029105483 | 0.976793326 |
| PIANP | 0.00342569 | -3.021677048 | 0.028514937 | 0.977264057 |
| ADNP | 0.001176437 | 6.429084125 | 0.028471561 | 0.977298633 |
| HHIP | 0.007831049 | -3.496090883 | 0.02846172 | 0.977306477 |
| HHIP-AS1 | -0.006039571 | 0.127363911 | -0.028276251 | 0.977454318 |
| CTD-2001C12.1 | -0.002880808 | -0.427317543 | -0.027344076 | 0.978197386 |
| KCNJ15 | -0.007183615 | 0.427275033 | -0.027042235 | 0.978437998 |
| CAPN6 | 0.004317359 | -3.560506367 | 0.026314159 | 0.97901839 |
| RP11-712B9.2 | 0.002386871 | 1.069213676 | 0.026287258 | 0.979039834 |
| CKAP5 | 0.001654859 | 5.614986469 | 0.02620683 | 0.979103949 |
| USP53 | -0.002597898 | 2.861847934 | -0.025840166 | 0.979396244 |
| ZNF571-AS1 | 0.004519178 | -0.321886723 | 0.024907667 | 0.980139618 |
| SDF4 | 0.000743934 | 6.989896115 | 0.024864592 | 0.980173957 |
| KNDC1 | -0.005237098 | -2.662783014 | -0.024785752 | 0.980236808 |
| PSMD13 | 0.000667346 | 6.414954184 | 0.024489195 | 0.980473224 |
| VDAC1P6 | -0.001719819 | 3.036452512 | -0.024302716 | 0.980621885 |
| PACS2 | -0.000952693 | 4.892404931 | -0.024297141 | 0.98062633 |
| SCFD1 | -0.000702266 | 5.224873725 | -0.024233222 | 0.980677287 |
| DPEP3 | 0.004258291 | 1.382599907 | 0.02418406 | 0.980716479 |
| RP11-497H16.9 | 0.003470264 | 0.950133938 | 0.023995914 | 0.980866471 |
| RNU6-213P | -0.002423196 | 0.075596413 | -0.023657893 | 0.981135947 |
| SORBS2 | -0.004196201 | -5.686405601 | -0.023570987 | 0.98120523 |
| SCN3A | -0.005885745 | -2.353741214 | -0.02326948 | 0.981445598 |
| EOMES | -0.003589679 | 1.17870414 | -0.023258483 | 0.981454366 |
| LPGAT1 | -0.001376546 | 6.126217651 | -0.023120322 | 0.981564512 |
| UBE2D2 | 0.00081785 | 6.874573113 | 0.02295357 | 0.981697452 |
| SUCLG2 | -0.003346782 | 4.980282193 | -0.022904868 | 0.981736278 |
| AE000662.92 | 0.003119474 | -1.126141289 | 0.022865323 | 0.981767805 |
| RP11-146I2.1 | -0.004223669 | -1.044466908 | -0.022606603 | 0.981974066 |
| SNORD100 | -0.002521857 | 0.50139846 | -0.022458745 | 0.982091944 |
| PSPHP1 | -0.004726173 | 0.604473837 | -0.02220921 | 0.982290884 |
| RP11-325P15.1 | -0.002564871 | 2.527931493 | -0.022004113 | 0.982454397 |
| SLC37A4 | -0.001496818 | 3.750865189 | -0.021659157 | 0.982729414 |
| ZNF526 | 0.001065962 | 3.470162735 | 0.021592425 | 0.982782617 |
| GVINP2 | -0.003386661 | -1.77084532 | -0.021044013 | 0.983219844 |
| CTB-79E8.3 | 0.001112723 | 8.628367016 | 0.020884173 | 0.98334728 |
| SCMH1 | -0.001407994 | 5.785251504 | -0.020820025 | 0.983398423 |
| NUCB1-AS1 | -0.00272749 | -0.442721872 | -0.020704646 | 0.983490411 |
| LRRC16B | -0.002126726 | -0.0596936 | -0.020628839 | 0.98355085 |
| FTLP17 | 0.002647964 | -0.153697581 | 0.020558946 | 0.983606573 |
| HSP90AB7P | -0.002316389 | -1.709744447 | -0.020459859 | 0.983685573 |
| CTD-2022H16.3 | -0.001271363 | 2.986289541 | -0.019859091 | 0.984164554 |
| TOP1MT | 0.001934753 | 5.954735452 | 0.019771877 | 0.984234089 |
| SLC29A2 | -0.002527081 | 2.332727089 | -0.019737918 | 0.984261163 |
| RP11-256K7.1 | -0.002034962 | 0.709336167 | -0.019626984 | 0.98434961 |
| PPIAP11 | 0.001385146 | 2.195351972 | 0.01943224 | 0.984504877 |
| RP11-597A11.3 | 0.001303777 | 2.274534964 | 0.018488117 | 0.985257626 |
| LDHD | 0.003764735 | 0.806263196 | 0.018305514 | 0.985403217 |
| RP11-126K1.2 | 0.00160465 | 2.014961792 | 0.018040193 | 0.98561476 |
| SNX18P7 | -0.003575324 | -0.26715031 | -0.017470441 | 0.986069033 |
| RP11-145M9.4 | 0.000849719 | 6.665192208 | 0.017455222 | 0.986081167 |
| BANK1 | 0.002404399 | 3.364363068 | 0.017088999 | 0.986373166 |
| CLASP2 | 0.000741085 | 4.540187048 | 0.016899012 | 0.986524647 |
| FDPSP5 | 0.001367872 | 0.792006714 | 0.01686862 | 0.986548879 |
| EVPL | 0.004397376 | 1.562515351 | 0.016783273 | 0.98661693 |
| AC137934.1 | 0.001838013 | -0.476732837 | 0.016644141 | 0.986727864 |
| IK | 0.000547811 | 6.525931975 | 0.016322559 | 0.986984272 |
| UPK3A | -0.003492355 | 0.074863311 | -0.016277899 | 0.987019881 |
| RP11-79L9.2 | -0.001753776 | 2.396209322 | -0.016198054 | 0.987083545 |
| PET117 | 0.000949506 | 2.633234137 | 0.015861509 | 0.987351886 |
| RHOQP2 | 0.001241093 | 4.588653682 | 0.01577719 | 0.987419117 |
| PRSS16 | -0.002047314 | -3.275127608 | -0.015406588 | 0.987714614 |
| CTC-425O23.2 | -0.001499096 | 2.193919893 | -0.015134733 | 0.987931378 |
| MFSD12 | -0.000584404 | 5.612331478 | -0.0146558 | 0.988313257 |
| PPP2R3A | 0.002893724 | -1.597287043 | 0.014248748 | 0.988637824 |
| SNX5 | -0.000556475 | 5.648332439 | -0.013857653 | 0.988949669 |
| HNRNPCP6 | -0.001178949 | 0.520387403 | -0.013435818 | 0.989286026 |
| LINC00478 | 0.002948455 | -2.107563687 | 0.013424882 | 0.989294746 |
| C12orf44 | -0.000660082 | 4.76084205 | -0.01316679 | 0.989500542 |
| KCNJ1 | -0.001845235 | -3.381710785 | -0.013142435 | 0.989519962 |
| RP11-420A6.2 | 0.001393793 | 0.468028024 | 0.013075981 | 0.989572951 |
| CCT6P3 | 0.000797795 | 2.410233083 | 0.012563289 | 0.98998176 |
| RPS2P46 | -0.000646304 | 10.45682558 | -0.012510051 | 0.990024211 |
| SEPT8 | 0.001306916 | 3.319427386 | 0.01234987 | 0.990151936 |
| RP13-270P17.2 | -0.001233956 | -0.716653005 | -0.011569907 | 0.990773867 |
| WDR17 | -0.002254539 | -1.05788515 | -0.011277187 | 0.991007279 |
| RP11-262D11.2 | 0.000594103 | 5.24158733 | 0.01091992 | 0.991292162 |
| C17orf89 | 0.000529482 | 5.061167497 | 0.010804796 | 0.991383961 |
| ESR2 | 0.001509462 | -3.518920893 | 0.01066697 | 0.991493863 |
| RP4-669P10.16 | 0.001126488 | -0.610750571 | 0.010309993 | 0.991778516 |
| SLC35F6 | 0.000827799 | 4.085231815 | 0.010306288 | 0.991781471 |
| FAM179A | -0.001205739 | 0.454116018 | -0.010209018 | 0.991859034 |
| TNK1 | -0.001271141 | -0.624830053 | -0.010178876 | 0.991883069 |
| MOB3B | 0.001284237 | 2.89648122 | 0.010129539 | 0.991922411 |
| EIF3FP3 | -0.000433352 | 9.053259622 | -0.009801435 | 0.992184042 |
| RPL7P24 | 0.0010518 | -0.650026623 | 0.009780201 | 0.992200974 |
| AC024082.3 | 0.000963158 | -0.163379755 | 0.009748274 | 0.992226433 |
| DSP | -0.001915162 | -2.663104401 | -0.009725603 | 0.99224451 |
| CCT6P2 | -0.000512976 | 3.590134863 | -0.009714027 | 0.992253741 |
| L1TD1 | 0.001391424 | -3.528885112 | 0.009627782 | 0.992322513 |
| KBTBD7 | 0.001000505 | 2.72172074 | 0.009528674 | 0.992401543 |
| CACNA1H | 0.001876993 | -1.010071989 | 0.009486088 | 0.992435501 |
| TREX1 | 0.00056368 | 5.235001768 | 0.009097688 | 0.992745214 |
| CATSPERG | -0.000646706 | 1.176477726 | -0.008559124 | 0.993174672 |
| RCAN2 | -0.001199824 | -2.173306681 | -0.008473467 | 0.993242976 |
| TPT1P5 | -0.000760615 | 5.39002962 | -0.008407771 | 0.993295362 |
| DNPEP | 0.000333527 | 5.30547787 | 0.008220677 | 0.993444554 |
| AC092295.7 | -0.000509235 | 3.437457109 | -0.008210548 | 0.993452631 |
| CTB-27N1.1 | 0.001037918 | -1.986048282 | 0.007906556 | 0.99369504 |
| TNK2 | 0.000380001 | 5.990528364 | 0.007858075 | 0.993733699 |
| CLSTN3 | -0.000606745 | 5.31188726 | -0.00774905 | 0.993820638 |
| UBE2NL | -0.000819667 | -0.550640998 | -0.007639415 | 0.993908063 |
| IL17RC | 0.000896444 | 2.985729299 | 0.007122043 | 0.994320626 |
| RP13-15M17.1 | -0.000745467 | 1.632951356 | -0.006832564 | 0.994551464 |
| ARHGAP4 | -0.000524519 | 8.266534723 | -0.006508 | 0.994810279 |
| DCLRE1A | -0.000418731 | 3.835081777 | -0.006473317 | 0.994837936 |
| IGHV3OR15-7 | -0.000733849 | -0.581105433 | -0.005867489 | 0.99532104 |
| CTC-523E23.11 | 0.000767695 | 2.259795635 | 0.005849525 | 0.995335365 |
| BLCAP | -0.000218764 | 5.891615778 | -0.0056436 | 0.995499576 |
| AC004951.5 | -0.00043583 | 1.210976726 | -0.005582698 | 0.995548141 |
| MST1P2 | 0.000513371 | 1.69396954 | 0.005257299 | 0.995807624 |
| AC083843.1 | 0.00059878 | 0.266200992 | 0.005195983 | 0.99585652 |
| MEIS3P1 | 0.00098963 | -0.905434855 | 0.004955934 | 0.996047943 |
| NPPA-AS1 | -0.000395185 | 2.330750827 | -0.004639331 | 0.996300412 |
| AC009961.5 | -0.000472981 | 1.397523172 | -0.004420808 | 0.99647467 |
| MMRN1 | -0.000961485 | 4.261919792 | -0.004107876 | 0.996724213 |
| CCDC62 | 0.000351714 | 1.744725545 | 0.003665783 | 0.997076754 |
| USB1 | 0.000187919 | 5.399838409 | 0.003607981 | 0.997122848 |
| CATSPERB | 0.000497462 | -2.872496664 | 0.003231601 | 0.997422988 |
| ERBB2IP | 0.000134331 | 6.53073472 | 0.003073443 | 0.997549109 |
| CYFIP2 | 0.00031218 | 6.132285774 | 0.00301738 | 0.997593816 |
| AC073343.1 | 0.000256412 | 1.480960124 | 0.002964195 | 0.997636227 |
| LINC00528 | 0.000264879 | 4.220887959 | 0.002844689 | 0.997731527 |
| ATF2 | 7.17E-05 | 5.72895142 | 0.002761906 | 0.997797541 |
| EPPK1 | 0.000493933 | -1.247181351 | 0.002733562 | 0.997820143 |
| CPNE5 | -0.000301246 | 0.938625429 | -0.00222414 | 0.998226377 |
| MPRIP | -0.000131698 | 6.226034241 | -0.002218132 | 0.998231168 |
| PKD1L3 | -0.000250281 | -2.503331936 | -0.001838156 | 0.998534177 |
| MMP16 | -0.000393281 | -4.584665969 | -0.00176123 | 0.998595521 |
| EEF1A1 | 6.73E-05 | 10.69638286 | 0.00163639 | 0.998695073 |
| WWC2 | -0.000268055 | 1.378433465 | -0.001412106 | 0.998873926 |
| CSTF2 | 6.75E-05 | 4.903792048 | 0.001340265 | 0.998931216 |
| FAM211A-AS1 | 0.000103168 | 9.634843398 | 0.00132899 | 0.998940207 |
| RP4-717I23.2 | -9.47E-05 | 1.878960095 | -0.001019857 | 0.999186723 |
| CHID1 | -5.89E-05 | 4.504451302 | -0.000939901 | 0.999250483 |
| CTB-171A8.1 | 0.000121616 | -1.179928028 | 0.000784143 | 0.999374691 |
| RP11-147I3.1 | -6.24E-05 | 2.376158306 | -0.000713676 | 0.999430885 |
| RP11-606P2.1 | -7.48E-05 | 1.459235661 | -0.000712909 | 0.999431496 |
| DIS3L2 | 2.00E-05 | 3.604814322 | 0.000560871 | 0.999552737 |
| CWC22 | 1.56E-05 | 5.524409135 | 0.0004031 | 0.999678551 |

TableS6

GSEA results (P<0.05)

| ID | Description | p.adjust |
| --- | --- | --- |
| hsa04061 | Viral protein interaction with cytokine and cytokine receptor | 1.65E-08 |
| hsa04060 | Cytokine-cytokine receptor interaction | 1.65E-08 |
| hsa05144 | Malaria | 4.44E-07 |
| hsa05200 | Pathways in cancer | 5.84E-06 |
| hsa04512 | ECM-receptor interaction | 2.17E-05 |
| hsa05206 | MicroRNAs in cancer | 2.88E-05 |
| hsa04110 | Cell cycle | 3.86E-05 |
| hsa04062 | Chemokine signaling pathway | 3.86E-05 |
| hsa04657 | IL-17 signaling pathway | 3.86E-05 |
| hsa05202 | Transcriptional misregulation in cancer | 4.22E-05 |
| hsa04151 | PI3K-Akt signaling pathway | 4.70E-05 |
| hsa05205 | Proteoglycans in cancer | 4.91E-05 |
| hsa04350 | TGF-beta signaling pathway | 4.91E-05 |
| hsa05215 | Prostate cancer | 7.26E-05 |
| hsa04015 | Rap1 signaling pathway | 0.000124071 |
| hsa04668 | TNF signaling pathway | 0.000143007 |
| hsa04115 | p53 signaling pathway | 0.00024179 |
| hsa04068 | FoxO signaling pathway | 0.000257539 |
| hsa05165 | Human papillomavirus infection | 0.000472889 |
| hsa05224 | Breast cancer | 0.000646858 |
| hsa05323 | Rheumatoid arthritis | 0.000648507 |
| hsa05226 | Gastric cancer | 0.00072343 |
| hsa04974 | Protein digestion and absorption | 0.001064152 |
| hsa05146 | Amoebiasis | 0.001077569 |
| hsa04064 | NF-kappa B signaling pathway | 0.001154108 |
| hsa04550 | Signaling pathways regulating pluripotency of stem cells | 0.001260355 |
| hsa04610 | Complement and coagulation cascades | 0.00128499 |
| hsa04010 | MAPK signaling pathway | 0.003141859 |
| hsa04390 | Hippo signaling pathway | 0.003149813 |
| hsa05171 | Coronavirus disease - COVID-19 | 0.003283279 |
| hsa04625 | C-type lectin receptor signaling pathway | 0.003661496 |
| hsa00640 | Propanoate metabolism | 0.004870604 |
| hsa05222 | Small cell lung cancer | 0.005526853 |
| hsa04218 | Cellular senescence | 0.006464262 |
| hsa04933 | AGE-RAGE signaling pathway in diabetic complications | 0.007203446 |
| hsa05143 | African trypanosomiasis | 0.007776445 |
| hsa05217 | Basal cell carcinoma | 0.008372367 |
| hsa04510 | Focal adhesion | 0.011921536 |
| hsa05332 | Graft-versus-host disease | 0.012026009 |
| hsa04710 | Circadian rhythm | 0.016065737 |
| hsa05219 | Bladder cancer | 0.016065737 |
| hsa05218 | Melanoma | 0.017267357 |
| hsa04934 | Cushing syndrome | 0.019567192 |
| hsa05167 | Kaposi sarcoma-associated herpesvirus infection | 0.020132532 |
| hsa04146 | Peroxisome | 0.020132532 |
| hsa04360 | Axon guidance | 0.020132532 |
| hsa05022 | Pathways of neurodegeneration - multiple diseases | 0.020132532 |
| hsa04940 | Type I diabetes mellitus | 0.020765676 |
| hsa05418 | Fluid shear stress and atherosclerosis | 0.021432609 |
| hsa05225 | Hepatocellular carcinoma | 0.023168306 |
| hsa04520 | Adherens junction | 0.027853791 |
| hsa04928 | Parathyroid hormone synthesis, secretion and action | 0.032109799 |
| hsa00970 | Aminoacyl-tRNA biosynthesis | 0.032109799 |
| hsa04914 | Progesterone-mediated oocyte maturation | 0.034856745 |
| hsa00280 | Valine, leucine and isoleucine degradation | 0.034856745 |
| hsa00020 | Citrate cycle (TCA cycle) | 0.035921605 |
| hsa04310 | Wnt signaling pathway | 0.037783306 |
| hsa05330 | Allograft rejection | 0.03781391 |
| hsa04020 | Calcium signaling pathway | 0.038658747 |
| hsa00071 | Fatty acid degradation | 0.038658747 |
| hsa00620 | Pyruvate metabolism | 0.039746183 |
| hsa05163 | Human cytomegalovirus infection | 0.040213424 |
| hsa04540 | Gap junction | 0.047776567 |
| hsa04114 | Oocyte meiosis | 0.047776567 |

TableS7

GSVA results (P<0.05)

| id | adj.P.Val |
| --- | --- |
| KEGG_CIRCADIAN_RHYTHM_MAMMAL | 9.79E-21 |
| KEGG_SELENOAMINO_ACID_METABOLISM | 4.60E-20 |
| KEGG_SULFUR_METABOLISM | 4.48E-19 |
| KEGG_GLYCOSAMINOGLYCAN_BIOSYNTHESIS_KERATAN_SULFATE | 3.55E-16 |
| KEGG_PROPANOATE_METABOLISM | 1.53E-15 |
| KEGG_MELANOMA | 1.31E-14 |
| KEGG_PROSTATE_CANCER | 1.95E-14 |
| KEGG_ALPHA_LINOLENIC_ACID_METABOLISM | 2.24E-14 |
| KEGG_PEROXISOME | 2.02E-13 |
| KEGG_FATTY_ACID_METABOLISM | 2.61E-13 |
| KEGG_GLYCOSPHINGOLIPID_BIOSYNTHESIS_LACTO_AND_NEOLACTO_SERIES | 2.61E-13 |
| KEGG_VALINE_LEUCINE_AND_ISOLEUCINE_DEGRADATION | 3.41E-13 |
| KEGG_SMALL_CELL_LUNG_CANCER | 1.36E-11 |
| KEGG_CHRONIC_MYELOID_LEUKEMIA | 1.97E-11 |
| KEGG_NON_HOMOLOGOUS_END_JOINING | 1.97E-11 |
| KEGG_P53_SIGNALING_PATHWAY | 4.41E-11 |
| KEGG_BLADDER_CANCER | 5.08E-11 |
| KEGG_ERBB_SIGNALING_PATHWAY | 1.97E-10 |
| KEGG_COLORECTAL_CANCER | 3.81E-10 |
| KEGG_RENAL_CELL_CARCINOMA | 4.94E-10 |
| KEGG_BETA_ALANINE_METABOLISM | 8.49E-10 |
| KEGG_PANCREATIC_CANCER | 1.10E-09 |
| KEGG_AMINOACYL_TRNA_BIOSYNTHESIS | 1.62E-09 |
| KEGG_MTOR_SIGNALING_PATHWAY | 6.93E-09 |
| KEGG_ETHER_LIPID_METABOLISM | 7.21E-09 |
| KEGG_TGF_BETA_SIGNALING_PATHWAY | 1.14E-08 |
| KEGG_PROGESTERONE_MEDIATED_OOCYTE_MATURATION | 1.87E-08 |
| KEGG_HISTIDINE_METABOLISM | 3.86E-08 |
| KEGG_PPAR_SIGNALING_PATHWAY | 5.99E-08 |
| KEGG_ALDOSTERONE_REGULATED_SODIUM_REABSORPTION | 7.94E-08 |
| KEGG_GLIOMA | 1.22E-07 |
| KEGG_CELL_CYCLE | 1.56E-07 |
| KEGG_ECM_RECEPTOR_INTERACTION | 1.59E-07 |
| KEGG_VEGF_SIGNALING_PATHWAY | 3.14E-07 |
| KEGG_ACUTE_MYELOID_LEUKEMIA | 6.74E-07 |
| KEGG_ADHERENS_JUNCTION | 6.92E-07 |
| KEGG_SPLICEOSOME | 1.47E-06 |
| KEGG_STARCH_AND_SUCROSE_METABOLISM | 1.77E-06 |
| KEGG_NON_SMALL_CELL_LUNG_CANCER | 2.18E-06 |
| KEGG_TASTE_TRANSDUCTION | 3.23E-06 |
| KEGG_TRYPTOPHAN_METABOLISM | 3.23E-06 |
| KEGG_GLYCINE_SERINE_AND_THREONINE_METABOLISM | 3.98E-06 |
| KEGG_BUTANOATE_METABOLISM | 7.38E-06 |
| KEGG_ENDOMETRIAL_CANCER | 7.38E-06 |
| KEGG_PROTEASOME | 1.13E-05 |
| KEGG_PENTOSE_AND_GLUCURONATE_INTERCONVERSIONS | 2.05E-05 |
| KEGG_PRIMARY_BILE_ACID_BIOSYNTHESIS | 3.30E-05 |
| KEGG_PANTOTHENATE_AND_COA_BIOSYNTHESIS | 3.99E-05 |
| KEGG_GALACTOSE_METABOLISM | 0.000204063 |
| KEGG_PYRUVATE_METABOLISM | 0.000208774 |
| KEGG_DNA_REPLICATION | 0.001313239 |
| KEGG_OTHER_GLYCAN_DEGRADATION | 0.002627768 |
| KEGG_RIBOSOME | 0.034127567 |
